# Cross-platform genomic identification and clinical validation of breast cancer diagnostic biomarkers

**DOI:** 10.18632/aging.202388

**Published:** 2021-01-20

**Authors:** Dongdong Liu, Beibei Li, Xiaoshun Shi, Jiexia Zhang, Allen Menglin Chen, Jiarui Xu, Wan Wang, Kailing Huang, Jinwei Gao, Zhouxia Zheng, Dan Liu, Huimin Wang, Wen Shi, Lin Chen, Jianhua Xu

**Affiliations:** 1 Department of Laboratory Science, The Second Affiliated Hospital of Guangzhou University of Chinese Medicine, Guangzhou 510120, P.R. China; 2 Department of Thoracic Surgery, Nanfang Hospital, Southern Medical University, Guangzhou 510515, P.R. China; 3 Department of Respiratory, The First Affiliated Hospital of Guangzhou Medical University, State Key Laboratory of Respiratory Disease, National Clinical Research Center for Respiratory Disease, Guangzhou Institute of Respiratory Health, Guangzhou 510120, P.R. China; 4 Guangzhou Mendel Genomics and Medical Technology Co., Ltd., Guangzhou 510530, P.R. China; 5 Department of Laboratory Science, Shenzhen Traditional Chinese Medicine Hospital, Shenzhen 518000, P.R. China; 6 Department of Laboratory Science, Shunde Hospital of Guangzhou University of Chinese Medicine, Foshan 528300, P.R. China

**Keywords:** breast cancer, biomarkers, non-coding RNA, exosome, hsa-miR-423-5p

## Abstract

Introduction: Circulating non-coding RNA is an ideal source to discover novel biomarkers for non-invasive screening. However, studies for the discovery of universal miRNAs in serum and exosomes for breast cancer early diagnosis are limited.

Methods: Based on bioinformatic analysis, *in vitro* and *in vivo* studies were performed to understand the role of identified hsa-miR-423-5p in cancer proliferation, migration, cancer stem cell properties. Next, global non-coding RNA expression profiles in blood serum and exosome were performed. hsa-miR-423-5p expression from a total of 356 peripheral blood samples was evaluated and the association of hsa-miR-423-5p expression with clinical characteristics, sensitivity and specificity for breast cancer diagnosis were assessed.

Results: The expression of serum and exosomal hsa-miR-423-5p is abnormally increased in breast cancer. Suppression of hsa-miR-423-5p inhibited cell proliferation and invasion in both T47D and MDA-MB-231 breast cancer cell lines, and tumor growth *in vivo*. Compared with 113 healthy women, quantification analysis of hsa-miR-423-5p in 224 breast cancer samples confirmed the abnormal expression. Serum hsa-miR-423-5p was significantly associated with the clinical stage (P=0.001) and Ki-67 level (P=0.004).

Conclusions: A translational bioinformatics analysis procedure and validation by *in vitro*, *in vivo*, and clinical samples reveal that hsa-miR-423-5p could be used as a non-invasive breast cancer biomarker.

## INTRODUCTION

Breast cancer is the one of the leading female malignant tumors worldwide [1]. Although surgery, radiotherapy, chemotherapy, and endocrine therapy have improved the long-term survival rate of breast cancer patients, the overall survival rate of advanced stage breast cancer is still not acceptable. Therefore, identifying effective genetic and molecular biomarkers for early diagnosis of breast cancer is of importance.

MicroRNAs are non-coding genes of about 22 nucleotides in length, accounting for 2% to 3% of total genes in the human body [2]. Increased evidence has shown that miRNA can be used as a potential early diagnostic and prognostic biomarker as well as therapeutic monitors of breast cancer [3]. However, for easy degeneration in serum, miRNA that is used for early diagnosis of breast cancer at a non-invasive level needs to be further investigated.

Exosomes are cell-secreted vesicles that are enveloped by a lipid bilayer membrane containing cell-specific proteins, lipids, and nucleic acids (including miRNA and lncRNA) [4]. The application of circulating exosomal miRNAs in cancer diagnosis and treatment is promising [5], however measuring exosomal miRNA cannot be replaced by measuring miRNA in plasma [6]. Therefore, identifying stable microRNAs that are consistently expressed in both plasma and exosomes is crucial for identifying reliable non-coding biomarkers.

In our previous study, in which we established a microRNA detection method for clinical testing, we observed that hsa-miR-423-5p was abnormally elevated in gastric cancer patients [7] and glioma [8]. Whether hsa-miR-423-5p is elevated in breast cancer patients and which miRNAs can be used as diagnostic markers for breast cancer diagnosis remains unknown. Therefore, we systematically explored potential miRNA biomarkers, and performed high-throughput non-coding RNA sequencing in both serum and exosomes, *in vitro* and *in vivo* studies, and clinical blood sample verification. Our study provided a pipeline for the discovery of miRNA cancer biomarkers at three dimensions: from bioinformatics, then bench, to bedside.

## RESULTS

### Abnormal expression of hsa-miR-423-5p and its roles in carcinogenesis

**Figure 1:**
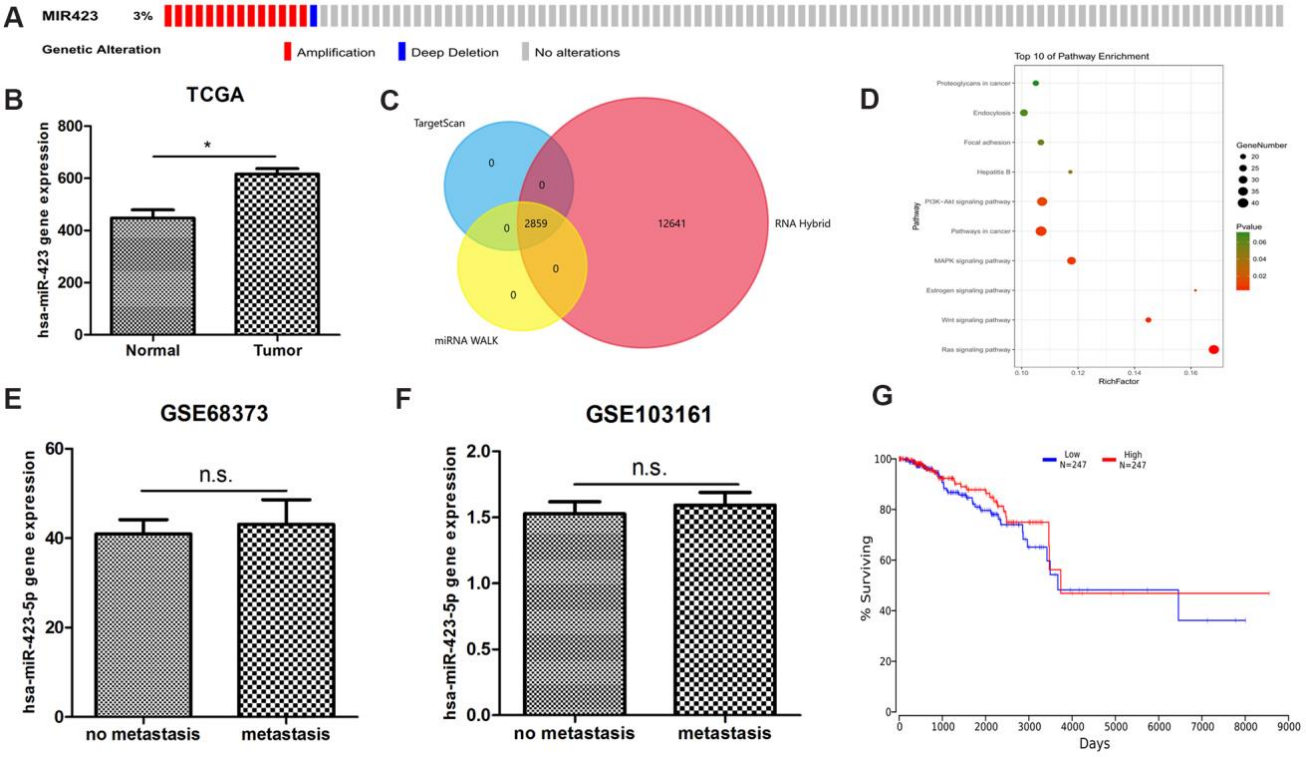
**The bioinformatics discovery of breast cancer non-coding RNA biomarkers.** (**A**) The abnormal amplification of hsa-miR-423-5p is shown from the cbioportal analysis. (**B**) hsa-miR-423-5p is significantly expressed in the TCGA RNA sequencing datasets. (**C**) A total of 2859 high confidential hsa-miR-423-5p target lncRNAs are found. (**D**) hsa-miR-423-5p target mRNAs enriched in a number of in cancer-relevant pathways indicate that hsa-miR-423-5p plays a pivotal role in the communication of cancer signaling. (**E**, **F**) Two independent microarray data show that hsa-miR-423-5p is not abnormally expressed in breast cancer metastatic tissue compared to the primary tumor, suggesting that hsa-miR-423-5p does not maintain its regulatory role in the advanced stage of breast cancer. (**G**) Online survival analysis shows that abnormal hsa-miR-423-5p expression is not associated with breast cancer survival.

We postulated that as an ideal marker, for easy detection, should be highly expressed in tumor tissues compared to adjacent cancer or breast tissue. We noticed that the expression of hsa-miR-423-5p in serum of breast cancer patients is at moderate or high levels, which is not detectable or detectable at low-levels in healthy human serum. By interrogating copy number alteration data of tumor samples recorded in cBioportal [9, 10], we found that 2.9% (14 of 482) of patients showed gene amplification [11] (Figure 1A). We next analyzed RNA sequencing data in TCGA breast cancer datasets, which showed that breast cancer patients are of increased hsa-miR-423 expression (Figure 1B). These results suggested that hsa-miR-423 had potential biological significance in breast cancer. We further explored the potential biological role of hsa-miR-423-5p by investigating its high confidential target genes. According to the Mendel score, 1404 highly confident miRNA target genes were selected out of a total of 60996 predicted genes (Supplementary Table 1). Similarly, the prediction results of high confidential miRNA target lncRNA (Mendel score ≥ 3, Figure 1C) are presented in Supplementary Table 2. Subsequently, pathway enrichment analysis of highly confidential miRNA target genes was performed. The data indicated that hsa-miR-423-5p targeting mRNAs were enriched in cancer-relevant pathways, including pathways in cancer, MAPK signaling pathway, Wnt signaling pathway, and the Ras signaling pathway (Figure 1D). However, hsa-miR-423-5p was not differentially expressed between breast cancer metastasis and non-metastasis samples (Figure 1E, 1F) and did not affect prognosis (Figure 1G) in clinical samples, which indicated that hsa-miR-423-5p mainly functions in cancer initiation and development rather than playing pivotal roles in advanced breast cancer.

### hsa-miR-423-5p is involved breast cancer cell growth

**Figure 2:**
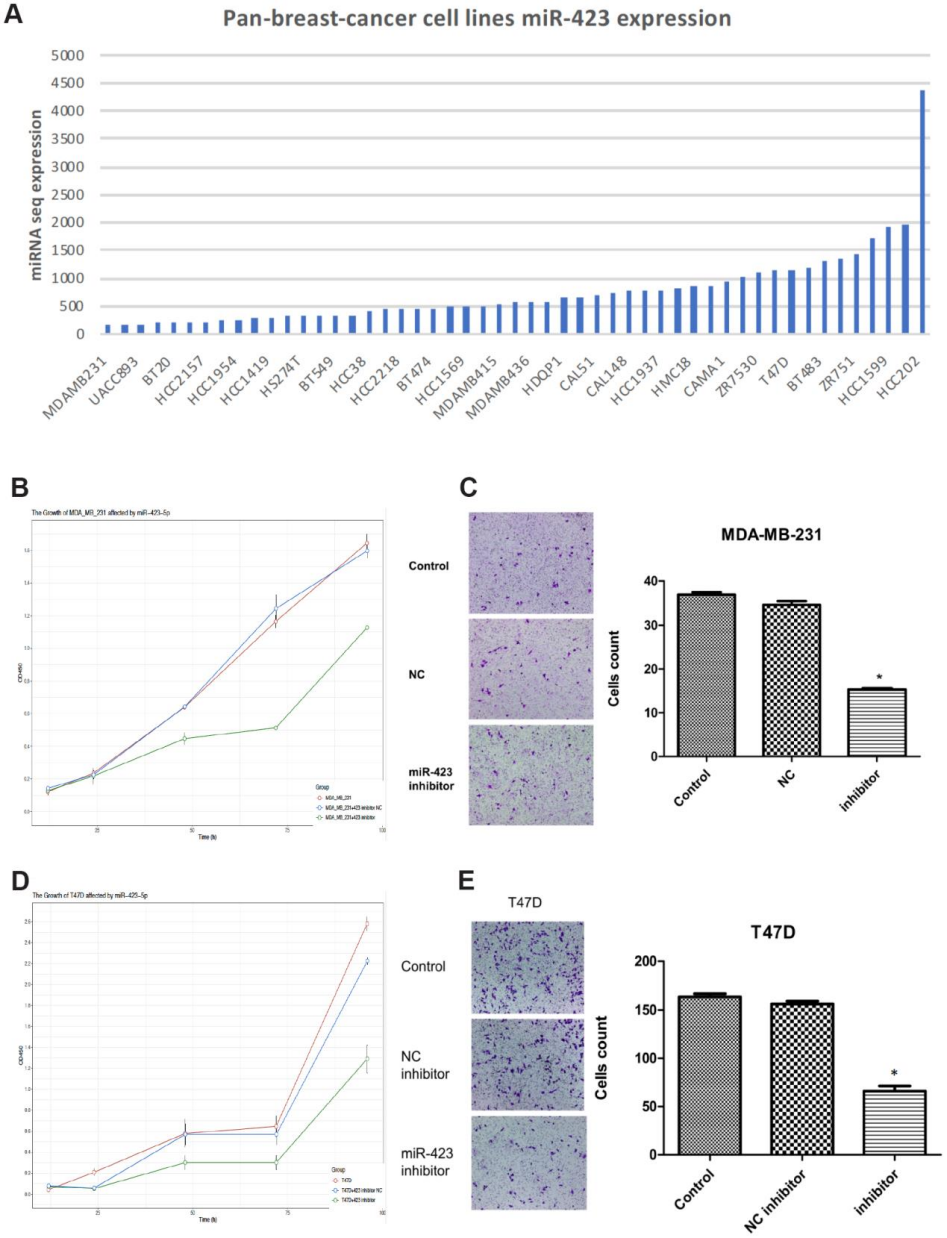
**Inhibition of hsa-miR-423-5p impairs proliferation and invasion of breast cancer cells.** (**A**) In board breast cancer cell lines, hsa-miR-423-5p expression was performed for the selection of cell model prior to *in vitro* studies. (**B**, **D**) Both MDA-MB-231 and T47D cells were treated with siRNA inhibitor or siNC for 96h, and cell proliferation was measured by CCK-8 assays. (**C**, **E**) For migration analysis, Transwell cell assay was performed in both MDA-MB-231 and T47D cells, and invasive cells were stained with crystal violet and counted under a microscope. *P < 0.05.

Based on the abnormal expression of hsa-miR-423-5p, we next explored its roles in breast cancer development. First, hsa-miR-423-5p expression in breast cancer cell lines (MDA-MB-231, UACC-893, BT-20, HCC2157, HCC1954, HCC1419, HS274T, BT-549, HCC38, HCC2218, BT-474, HCC1569, MDA-MB-415, MDA-MB-436, HDQP1, CAL51, CAL-148, HCC1937, HMC18, CAMA-1, ZR7530, T47D, BT-483, ZR751, HCC1599, and HCC202) were investigated by using Cancer Cell Line Encyclopedia (CCLE) data (Figure 2A) [12]. MDA-MB-231 cell and T47D cell representing lower and upper top 20% expression were selected out as the appropriate cell lines for *in vitro* studies. With hsa-miR-423-5p knockdown in transfected cell lines (Supplementary Figure 1), the cell viability assay showed that hsa-miR-423-5p inhibitors reduced the cell viability of both MDA-MB-231 and T47D cells when compared with the negative control (NC) group (P<0.05) (Figure 2B, 2D). This is further supported by the observation that the knockdown of hsa-miR-423-5p significantly inhibit breast cancer cell line *in vivo* (P<0.05, Supplementary Figure 2A, 2B). Real-time fluorescent quantitative PCR was used to detect the expression of stem cell-associated genes *c-Myc*, *KLF4*, and *SOX2*, resulting that the mRNA levels of stem cell-related genes were significantly reduced after miR-423-5p knockdown (Supplementary Figure 3A, 3C). Transwell assays indicated that knockdown of the hsa-miR-423-5p inhibitor significantly inhibited MDA-MB-231 and T47D invasion (P<0.05) (Figure 2C, 2E), potentiating cancer cell migration to normal cells. The above results suggest that miR-423-5p has a potential function for maintaining the stemness of breast cancer stem cells, cell growth, and initialization of cell migration.

### High expression of hsa-miR-423-5p in serum exosome

**Figure 3:**
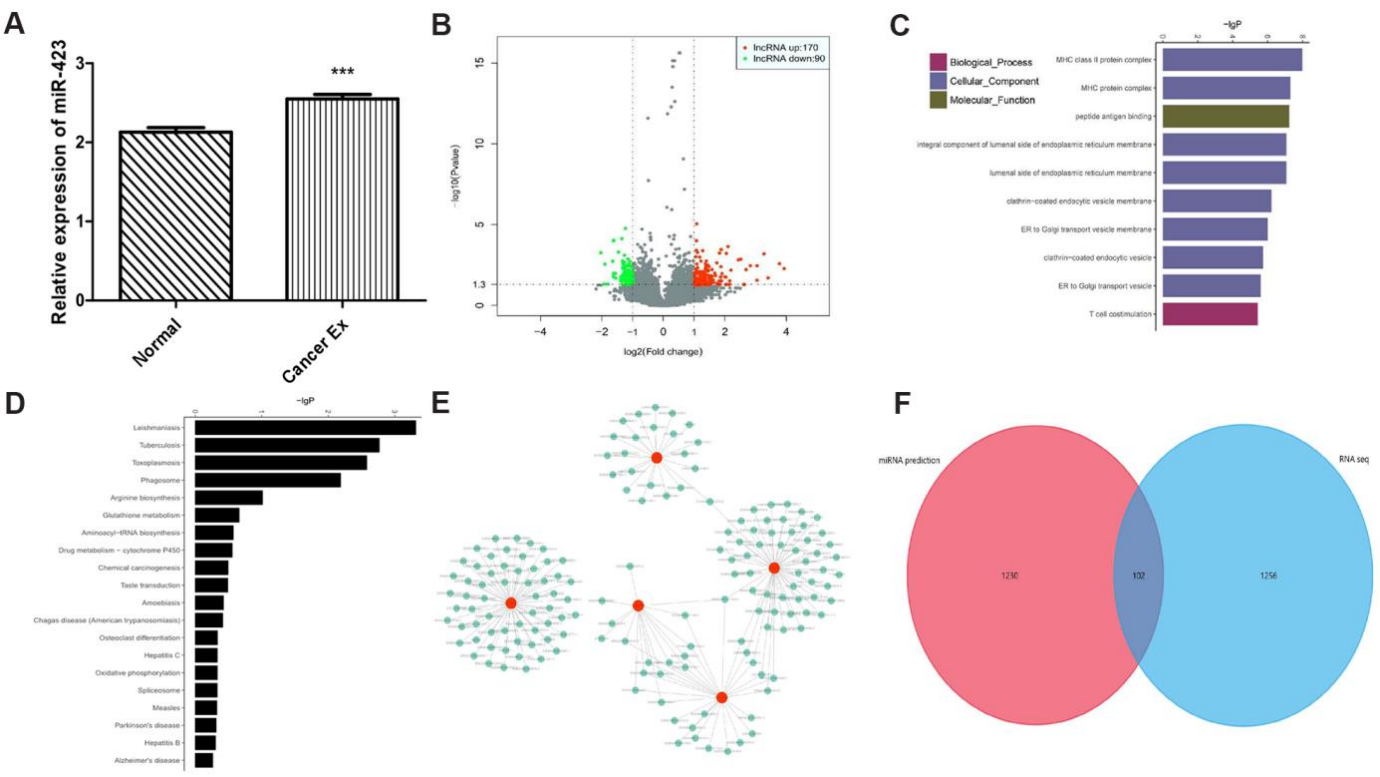
**The exosomal transcriptome landscape of breast cancer.** (**A**) The hsa-miR-423-5p expression level in breast cancer serum exosome is significantly elevated compared to those in serum from healthy female volunteers. (**B**) A total of 260 lncRNA and 110 mRNA transcripts are differentially expressed. (**C**) Top 10 GOs upregulated in breast cancer patients compared to controls. (**D**) Analysis of 20 enriched KEGG pathways between breast cancer patients and controls. (**E**) A total of 162 lncRNAs interact with 5 mRNAs (HLA-DPA1, HLA-DQA1, HLA-DRB1, HLA-DRB5, and KISS1R) between breast cancer patients and controls. (**F**) A total of 102 intersected genes of hsa-miR-423-5p predicted target mRNAs and differential expressed mRNAs in exosomes.

Given that *in vitro* and *in vivo* studies demonstrating a role of hsa-miR-423-5p in breast cancer growth, we proposed that breast cancer cells could release hsa-miR-423-5p to the blood via exosomes, facilitating proliferation process. After successful exosome extraction (Supplementary Figure 4), the hsa-miR-423-5p expression level in breast cancer serum exosomes was significantly higher compared to that of those in serum of healthy female volunteers (Figure 3A). Next, we hypothesized that the transcriptome status in exosomes could also be different between healthy women and breast cancer patients. Therefore, the expression landscape of mRNA and lncRNA were observed by exosomal RNA sequencing. In brief, in the breast cancer group, 260 lncRNA transcripts (170 upregulation and 90 downregulation) and 110 mRNA transcripts (79 upregulation and 31 downregulation) were differently expressed when compared to the control group (Figure 3B, Supplementary Table 3). To illustrate the potential biological impact of hsa-miR-423-5p alterations on breast cancer development, we conducted Gene ontology (GO) and Kyoto Encyclopedia of Genes and Genomes (KEGG) analysis. GO analysis showed that the top ten enriched biological process included the major histocompatibility complex (MHC) class II protein complex, MHC protein complex, peptide antigen binding, integral component of the luminal side of the endoplasmic reticulum, luminal side of the endoplasmic reticulum membrane, clathrin-coated endocytic vesicle membrane, endoplasmic reticulum (ER) to the Golgi transport vesicle, clathrin-coated endocytic vesicle, ER to Golgi transport vesicles, and T-cell co-stimulation (Figure 3C). Genes in these pathways were selected for QPCR analysis. Consistent with bioinformatic prediction of the trend of gene expression, the expression level of selected immune genes in *in vivo* tissues varied, suggesting that hsa-miR-423-5p affects immune regulation at multiple levels (Supplementary Figure 2C–2E). The KEGG pathway analysis maps the dysregulated protein-coding genes to enriched signal pathways, which include leishmaniasis, Tuberculosis, Toxoplasmosis, phagocytosis, and arginine biosynthesis (Figure 3D). Our data showed that a co-expression network between 1319 lncRNA and 143 mRNAs consisted of 1462 network nodes and 15255 connections (Supplementary Table 4). The co-expression network revealed that one mRNA may be associated with 1 to 56 lncRNAs, and that one lncRNA may be associated with 28 to 189 mRNAs, suggesting that for peptide binding, 162 lncRNAs interacted with 5 mRNAs (Figure 3E). The Venn diagram showed intersected genes of predicted hsa-miR-423-5p mRNA targets and differential expressed mRNAs in exosomes (Figure 3F), indicating that hsa-miR-423-5p preserved several pivotal microRNA signals during exosomal secretion.

### Serum microRNA sequencing and clinical validation

**Figure 4:**
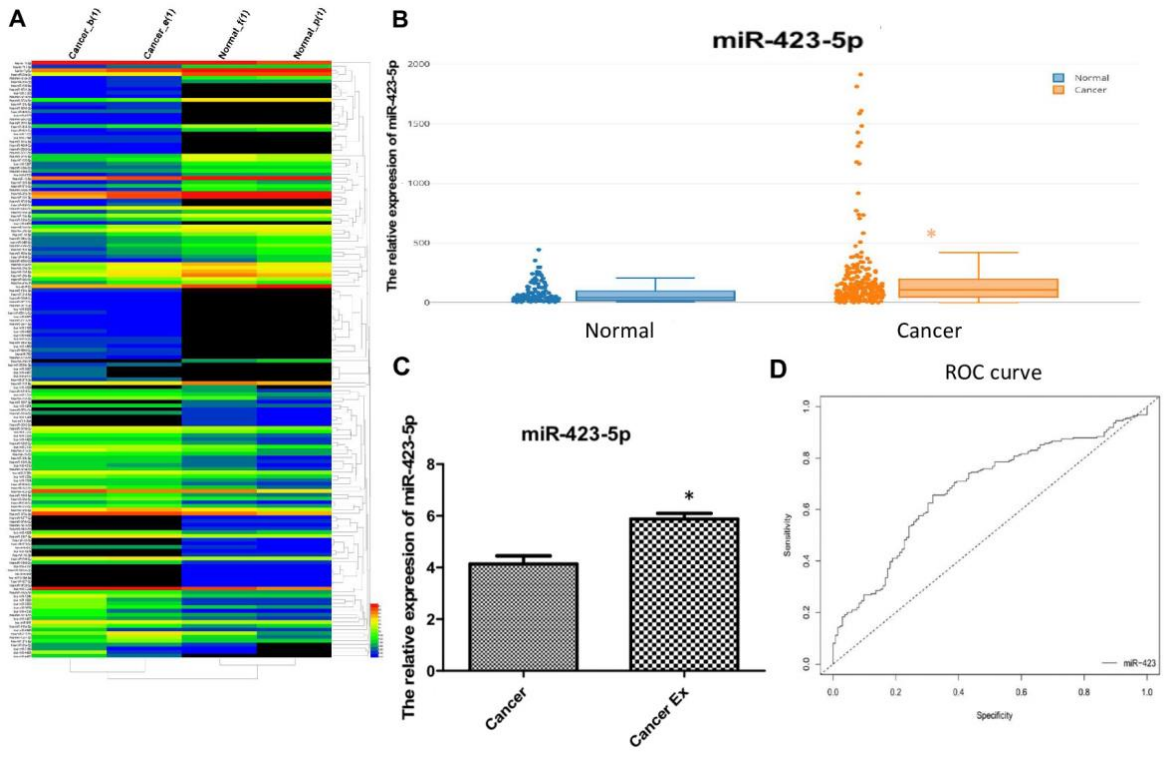
**Clinical validation of hsa-miR-423-5p as an early diagnostic biomarker.** (**A**) Heatmap for the differential expression of miRNA in plasma. Red labels indicate high expression and black labels indicate low expression. (**B**) A large cohort of breast cancer patients enrolled in serum hsa-miR-423-5p quantification, and the relative expression of hsa-miR-423-5p in breast cancer patients was significantly higher compared to that in controls (p<0.05). (**C**) A 1.5-fold increase in expression of hsa-miR-423-5p in the exosome of breast cancer patients was found. (**D**) The sensitivity and specificity analysis of hsa-miR-423-5p included analysis by the web tool easyROC (http://www.biosoft.hacettepe.edu.tr/easyROC).

To further study the expression level of hsa-miR-423-5p and the expression profile of miRNAs in plasma, pooled RNA sequencing in duplicate was performed in 5 pairs of patients and healthy volunteers (Figure 4A, Supplementary Table 5). Given that our goal was to identify diagnostic markers for breast cancer, up-regulated miRNAs were easier to detect compared to down-regulated miRNAs, we prioritized up-regulated hsa-miR-423-5p in the RNA-seq expression profile for further serological expression analysis. Our data showed that the relative expression of hsa-miR-423-5p in breast cancer patients was significantly higher compared to the control group (p<0.05) (Figure 4B). Additional comparison of hsa-miR-423-5p expression in 6 paired serum and exosome samples showed that there was a 1.5-fold enrichment of hsa-miR-423-5p expression in the exosome of breast cancer patients (Figure 4C). Next, the correlation among the expression level of hsa-miR-423-5p and clinical features including age, pathological stage, histological grade, and lymph node status was analyzed. The results showed that the expression of hsa-miR-423-5p positively correlated with the clinical stage (P<0.05) and proliferation marker protein Ki-67 (P<0.05) (Table 1). However, no significant correlations were observed between the expression of hsa-miR-423-5p and age or histological grade. Furthermore, receiver operating characteristic (ROC) analysis showed that at the cut-off point for the breast cancer diagnostic tool of hsa-miR-423-5p was 70.02 with a sensitivity of 66% and specificity of 68%. The area under curve (AUC) value was 0.68 (Figure 4D), suggesting that hsa-miR-423-5p can be used as one of the non-coding biomarkers for breast cancer.

**Table 1:** The association of clinicopathological features and serum hsa-miR-423-5p expression in breast cancer.

	**N(%)**	**has-miR-423-5p**
**Age**		
≤60	85 (75.4)	2.93±0.62
>60	28 (24.4)	3.12±0.80
**Stage**		
I	27 (24.1)	2.55±0.89
II	56 (50)	2.85±1.06
III	30 (26.9)	3.22±0.92^a^
**Grade**		
I	26 (23.2)	2.90±1.66
II	59 (52.2)	2.68±1.23
III	28 (24.6)	2.80±0.92
**Lymph Node**		
Negative	31 (37.4)	0.75±0.60
Positive	82 (72.6)	1.37±0.45
**Estrogen receptor**		
Negative	65 (57.1)	0.44±0.32
Positive	48 (42.9)	0.47±0.46
**Progesterone receptor**		
Negative	66 (58.6)	2.68±0.92
Positive	47 (41.4)	2.97±1.18
**HER2/neu**		
Negative	49 (43.8)	3.02±0.87
Positive	64 (56.2)	3.16±1.09
**Ki-67**		
≤14%	46 (40.6)	2.20±0.81
>14%	67 (59.4)	3.47±0.76^b^

Note: (a) P=0.001, (b) P=0.004, (c) No label indicates not significant.

## DISCUSSION

In several studies, it has been demonstrated that hsa-miR-423-5p was abnormally expressed in breast cancer tissues. Pollard et al. reported that expression levels of hsa-miR-423-5p were significantly higher in four ethnic groups, including British Caucasian, British Black, Nigerian and Indian [13]. In another study, it was found that hsa-miR-423-5p was expressed higher in surgically resected samples compared with biopsy samples obtained from neoadjuvant chemotherapy [14]. In addition, the expression of hsa-miR-423-5p in the serum of elderly breast cancer patients was higher compared to that of younger patients, but no significant differences were observed in the intra-study validation tests [15]. Increased expression of hsa-miR-423-5p has been reported to be associated with breast cancer metastasis [16]. Combined with the TCGA database, this study confirmed that hsa-miR-423-5p was abnormally expressed in breast cancer in either tissue or serum in over 1000 breast cancer patients. Here, we confirm for the first time that hsa-miR-423-5p expression levels in serum of breast cancer patients are higher compared to those in healthy female individuals. However, the expression was not associated with breast cancer metastasis and prognosis in clinical data. Together with our observation on *in vitro* invasion assay, stemness features analysis, and *in vivo* nude mice hsa-miR-423-5p inoculation, our study implies that hsa-miR-423-5p could be mainly involved in breast cancer initialization and development.

The difference in expression levels of miRNAs in plasma and exosomes remains controversial. Xie et al. showed that the expression of total miRNAs, based on qRT-PCR analysis, in plasma was higher compared to those in plasma-derived exosomes [6]. In contrast, in a previous study, it was indicated that the level of specific miRNAs in exosomes in lung cancer patients were significantly higher than that in plasma [17]. Therefore, for clinical diagnostic testing, it is not reasonable to detect the expression level of miRNAs in exosomes by detecting the expression of miRNAs in serum, and vice versa. In addition to the observation of abnormal mRNA and lncRNA expression exosomal RNA sequencing, hsa-miR-423-5p was expressed at a higher level in breast cancer patients in both exosome and serum when compared to healthy individuals, and the expression level of hsa-miR-423-5p in exosomes was 1.5 times higher in plasma compared to that in cancer patients. In addition, the expression of hsa-miR-423-5p positively correlated with the clinical stage (P=0.001), and multiple lines of evidence have shown that and abnormal hsa-miR-423-5p upregulation may be one of the signals sent out by breast cancer cells at an early stage.

The potential value of hsa-miR-423-5p for early screening of breast cancer and therapeutic monitoring was further studied. Firstly, our findings showed that hsa-miR-423-5p was associated with Ki-67 expression. Ki-67 expression reflects the proliferation activity of tumor cells and is often used as a reliable marker for the activity of tumor cell proliferation [18]. In several studies, it has been shown that breast cancer with positive expression of Ki-67 was invasive, with active cell proliferation, and an increased chance of metastasis and poor prognosis. The disease-free survival rate and overall survival rate of these patients were reduced [19]. Therefore, the expression of hsa-miR-423-5p in serum can be used to evaluate the malignancy of breast cancer at an early stage. With the cut-off value of hsa-miR-423-5p expression at 70.02 in plasma, our data indicated that hsa-miR-423-5p had an auxiliary diagnostic value for early breast cancer screening as well as recurrent monitoring.

In summary, we showed that the expression of hsa-miR-423-5p in both plasma and blood exosomes of breast cancer patients was abnormally high when compared to that of healthy controls. The hsa-miR-423-5p-regulated coding genes are widely distributed in tumor-associated signaling pathways in silica. Furthermore, we systematically validated the abnormality in expression by *in vitro* functional experiments, *in vivo* animal model, transcriptome analysis, and large clinical sample size. In this study, we provided a bioinformatics analysis pipeline and clinical validation protocol for the discovery of potential biomarkers for breast cancer early diagnosis. Further studies on the underlying mechanism of hsa-miR-423-5p in the development of breast cancer are needed.

## MATERIALS AND METHODS

### Patients and materials

This study was approved and reviewed by the Medical Ethics Committee of the Second Affiliated Hospital of Guangzhou University of Chinese Medicine (Guangzhou, China). A total of 224 cases of chemotherapy and radiotherapy naïve breast cancer patients with a pathological diagnosis and 113 healthy female adults of the same age range and demographic region were recruited for this study. From each patient, 5 ml venous blood was drawn at the fasting state prior to surgery. The healthy control group included females who did not have sign of malignant tumors or other benign mammalian diseases. Clinical information of each patient can be obtained by reviewing the patient’s electronic file.

### Bioinformatics analysis

Breast cancer TCGA RNA sequencing data were obtained from the Cancer Genome Atlas website (http://cancergenome.nih.gov). RNA sequencing data of both serum and exosomes were processed and assessed by a standard bioinformatics pipeline in RiboBio Co., Ltd. (Guangzhou, China). To measure gene expression levels, fragments per kilobase per million reads were used [20]. Differentially expressed genes were evaluated by | log2FoldChange | and q-value [21, 22]. Functional enrichment analysis of genes was assessed by the Kyoto encyclopedia of genes and genomes (KEGG) [23] and gene ontology (GO) [24]. Highly confidential miRNA target genes were predicted by integrating the following 12 prediction tools-miRWalk [25], miRDB [26], PITA [27], MicroT4 [28], miRMap [29], RNA22 [30], miRanda [31], miRNAMap [32], RNAhybrid [33], miRBridge [34], PICTAR2 [35], and Targetscan [36]. Similarly, the high confidential target lncRNAs were predicted by the following four tools, miRWalk, miRanda, RNAhybrid, and Targetscan. The total number of times that the target gene was predicted by different tools was summed. The criteria of highly confidential target genes were defined as the Mendel score, with genes with a score more than or equal to 7 for mRNA and 3 for lncRNA.

### Blood sample collection and RNA sequencing

Blood samples of controls and breast cancer patients were mixed respectively then stayed at 4° C for more than half an hour prior to centrifugation at 400 g, at 4° C for 10 min. Next, the supernatant was subjected to centrifugation at 1800 g for 10 min. Serums were collected and stored at -80° C. Exosomes were extracted by Ribo TM Exosome Isolation Reagent (Guangzhou RiboBio Co., Ltd.). Exosomes were evaluated by JEM-1200EX transmission electron microscope (JEOL Japan Electronics Co., Ltd and Western Blot). RNA was extracted and followed by library preparation and Illumina HiSeq 3000 system sequencing. Experiments were performed in duplicate.

### Cell culture

MDA-MB-231 and T47D cells were provided by Min Deng from the affiliated Cancer Hospital and Institute of Guangzhou Medical University (Guangzhou, China). Cryogenic vials were taken out from the liquid nitrogen, cells were rapidly thawed in a water bath at 37° C, and suspended in a centrifuge tube for centrifugation at 1000 rpm/min for 5 min. The supernatant was discarded, then 2 ml of medium was added to suspend the cells. The cell suspension was added to a tube containing 5 ml RPMI-1640 solution supplemented with 10% FBS and 2% penicillin-streptomycin and incubated at 37° C in a 5% CO_2_ incubator.

### Cancer cell function assays *in vitro*

The synthetic sequence of hsa-miR-423-5p inhibitor (*acuccccgucucucgcucugaaa*) was designed and synthesized by Guangzhou RiboBio Co., Ltd. The dry powder was centrifuged and dissolved in DEPC water at a final concentration of 20 μM. The transfection protocol was performed according to the manual of Lipofactamine™ 2000 (Invitrogen, Carlsbad, CA, USA). In brief, we changed non-antibiotic medium when the cell density under microscope was 40% to 60%. Dilute lipofectamine™ 2000 with Opti-MEM of miRNA inhibitor, miRNA inhibitor NC and medium alone were added. The cell medium was changed after 6h. The cell proliferation ability was measured by the cell counting kit-8 (CCK-8, Dojindo, China). The absorbance of CCK-8 reactions was measured at the time interval of 12h, 24h, 48h, 72h, and 96h post-transfection. These experiments were run in triplicate.

For Transwell migration assay, 3×10^4^ cells per millilitre were incubation for 22h in condition as mentioned. Cells remaining at the lower surface of the 8μm chamber were stained by crystal violet then the number of cells in 6 fields were captured by inverted microscope.

Additionally, we also tested sphere-forming capacity of MDA-MB-231 based on the stem cell spheres (the diameter is greater than 50μm is considered as sphere) and use the sphere formation efficiency (number of stem cell spheres/number of inoculated cells × 100%). The cells in this experiment are culture with DMEM/F12 + B27 (1:50)+ bFGF (20mg/ml) +1ml EGF(20ng/ml) + 1g insulin (per 500ml DMEM/F12). The expression of stem cell associated genes (*cMYC*, *KLF4* and *SOX2*) were evaluated.

### MicroRNA-Based *in vivo* assay

For observation of the impact of hsa-miR-423-5p on tumor growth, MDA-MB-231 cells transfected with saline, negative control of miRNA inhibitor, and hsa-miR-423-5p inhibitor were subcutaneously injected into the flanks of nude mice to generate a xenograph model, 5 mice per group. Tumor volume is evaluated according to the formula: Length×Width^2^×0.52. The differences among groups were compared by the Pairwise comparisons using t tests with pooled SD.

### Quantitative real-time PCR

For qRT-PCR, peripheral blood miRNAs were extracted, reverse transcribed and qRT-PCR was performed following the manufacturer's protocol (Beijing Tiangen Biotech Co. LTD, Beijing, China). The qPCR reactions were run on an ABI-7500 real-time PCR system using the following conditions: 94° C for 2 minutes, then for 20 seconds, followed by 5 cycles at 64° C for 30 seconds, then 72° C for 34 seconds, and finally 40 cycles at 94° C for 20 seconds, and 60° C for 34 seconds. The primers used were as follows: 5'- AGGGGCAGAGAGCGAGACTTT -3' (forward) and universal adaptor PCR primer (reverse). The relative expression of hsa-miR-423-5p was calculated as follows: Relative quantification=2^-ΔΔCT^, where ^Δ^CT_sample_=CT_sample_- CT_sample reference_, ^Δ^CT_control_=CT_control_-CT _control reference_, ^ΔΔ^CT=^Δ^CT _sample_-^Δ^CT _control_. Experiments were performed in triplicate.

Other primers used in these study are: *KLF4* (Forward: 5'-CAGAATTGGACCCGGTGTA-3', Reverse: 5'-GGCAAAACCTACACAAAGA-3'), *SOX2* (Forward: 5’- GACAGTTACGCGCACATGAA -3’, Reverse: 5’- TAGGTCTGCGAGCTGGTCAT -3’), *cMYC* (Forward: 5'-TTGAAGGCTGGATTTCCTTTGGGC-3', Reverse: 5’- TCGTCGCAGATGAAATAGGGCTGT-3'), *TGF-β1* (Forward: 5’-GCCCTGGACACCAACTATTG-3’, Reverse: 5’-TTTTAGCTGCACTTGCAGC C-3’), *TRB1* (Forward: 5'-GATCCCCGCGTGGACAGTTCTGCATTTTCAAGAGAAATGCAGAACTGTCCACGCTTTTTA-3′, Reverse: 5'-AGCTTAAAAAGCGTGGACAGTTCTGCATTTCTCTTGAAAATGCAGAACTGTCCACGCGGG-3′), *CBL* (Forward: 5'-TAGGCGAAACCTAACCAAACTG-3', Reverse: 5'-AGAGTCCACTTGGAAAGATTCCT-3').

### Statistics analysis

The association of hsa-miR-423-5p expression and clinicopathological findings were assessed by *χ*^2^ tests and Student's *t*-tests by using R (version 3.5). The survival curves were automatically calculated by OncoLnc (http://www.oncolnc.org/), setting the expression level of miR-423-5p in the lower 25% percentile versus the higher 25% percentile of invasive breast cancer. The statistically significant cut-off was set as *p*-values less than 0.05.

## Supplementary Material

Supplementary Figures

Supplementary Table 1

Supplementary Table 2

Supplementary Table 3

Supplementary Table 4

Supplementary Table 5
