# Supplementary material for: Cross-platform genomic identification and clinical validation of breast cancer diagnostic biomarkers
Source: Aging (Albany NY). 2021 Jan 20;13(3):4258–73. doi: 10.18632/aging.202388 (PMC7906147; doi:10.18632/aging.202388)
Supplement: Supplementary Figures [file aging-13-202388-s001.pdf]

SUPPLEMENTARY FIGURES

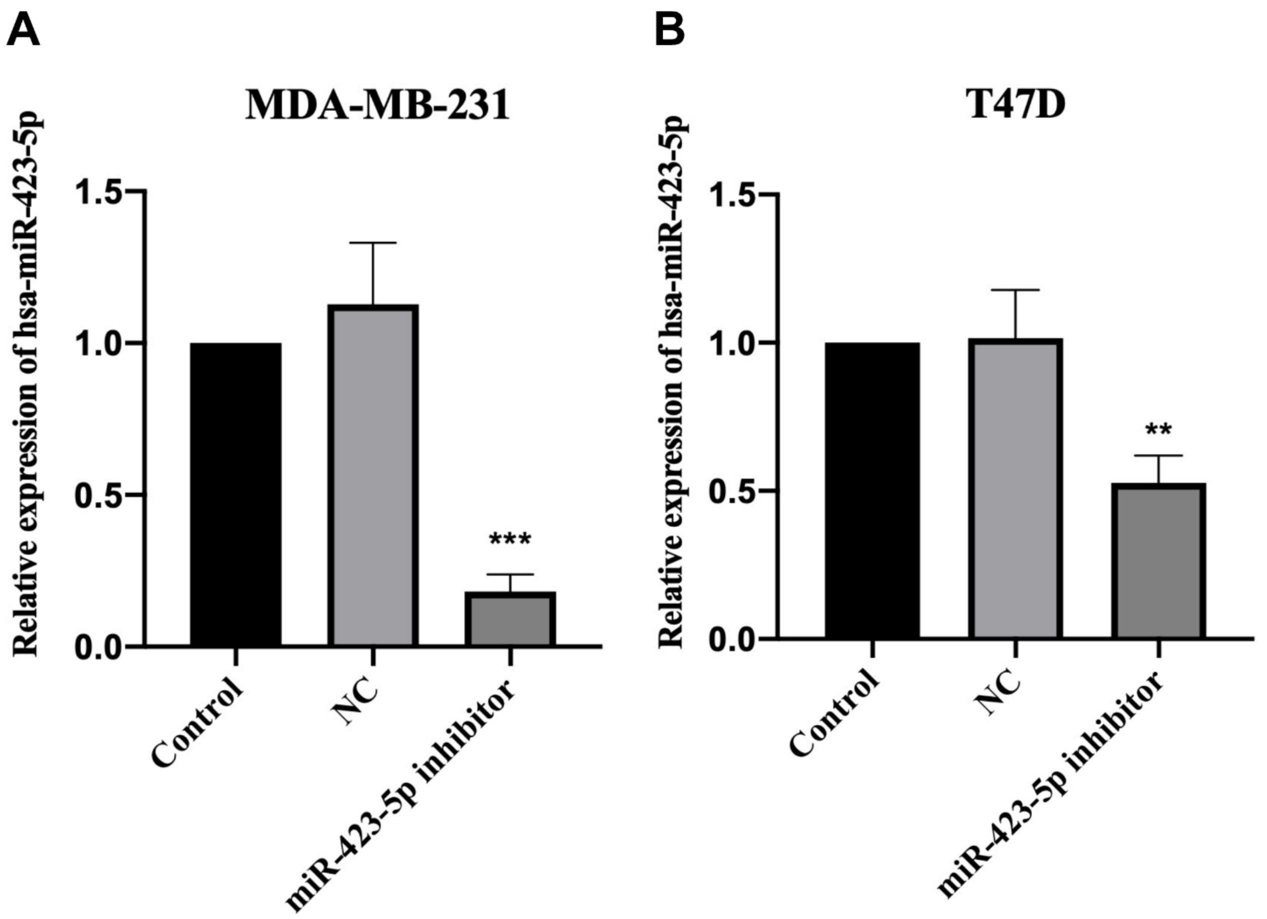

Supplementary Figure 1. The hsa-miR-423-5p inhibition in breast cancer cell lines. The expression level of hsa-miR-423-5p in control, negative control and hsa-miR-423-5p inhibitors of (**A**) MDA-MB-231 and (**B**) T47D.

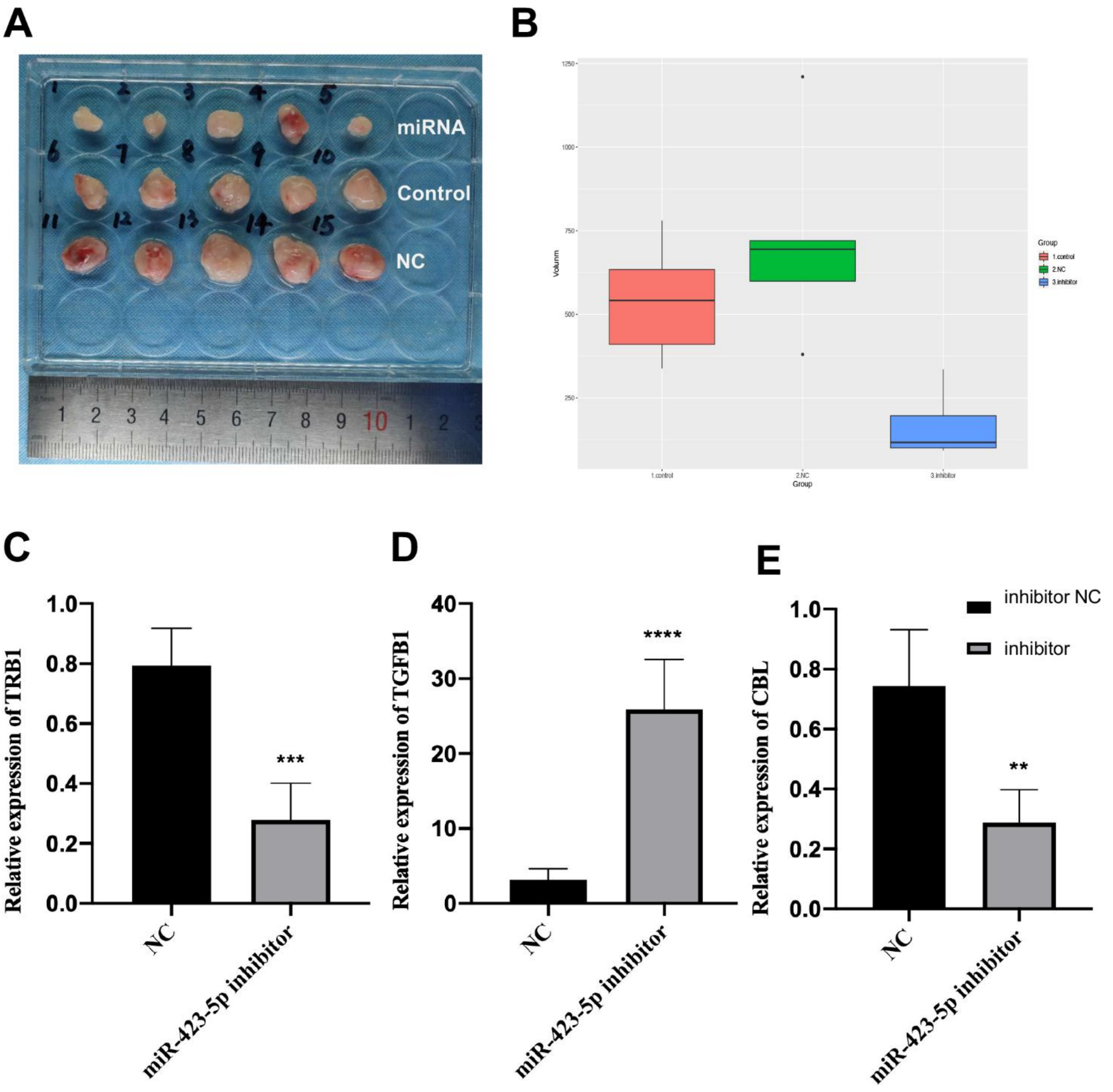

Supplementary Figure 2. The *in vivo* hsa-miR-423-5p inoculation and immune network analysis. (**A**) The resected tumors form control, negative control and hsa-miR-423-5p knockdowned group and (**B**) analysis of tumor size (mm^3). The relative expression of (**C**) *TRB1*, (**D**) *TGFB1*, and (**E**) *CBL*.

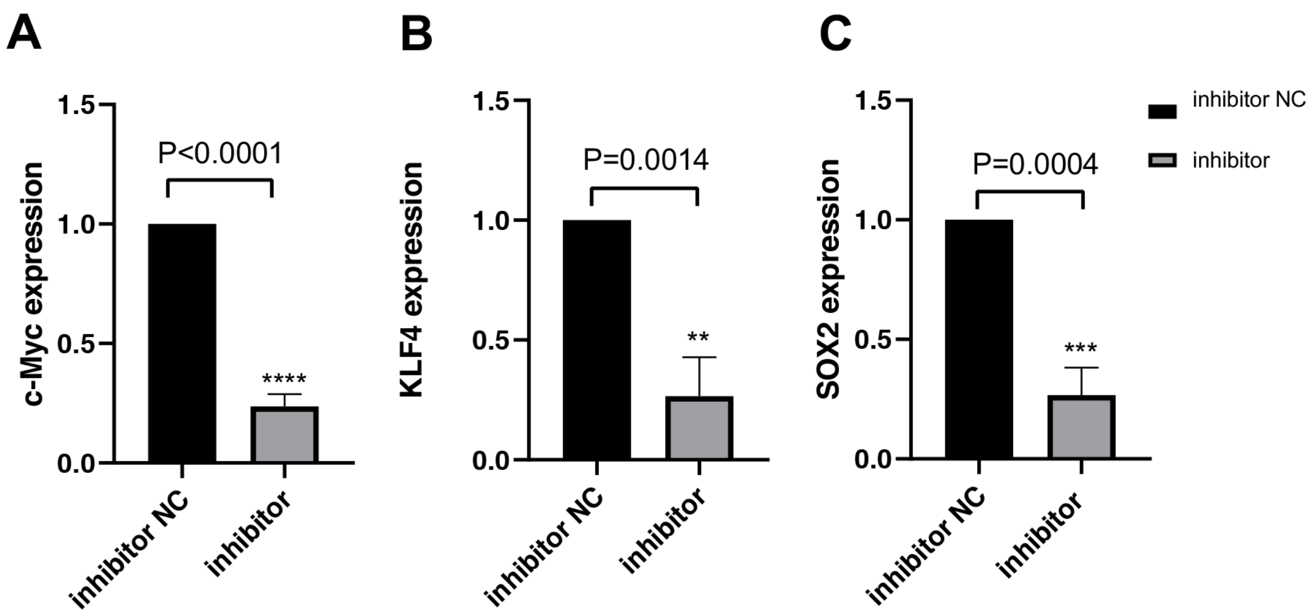

Supplementary Figure 3. The *in vitro* hsa-miR-423-5p breast cancer cell stemness analysis. The relative expression of (**A**) *c-MYC*, (**B**) *KLF4*, and (**C**) *SOX2* in negative control and hsa-miR-423-5p knockdowned group.

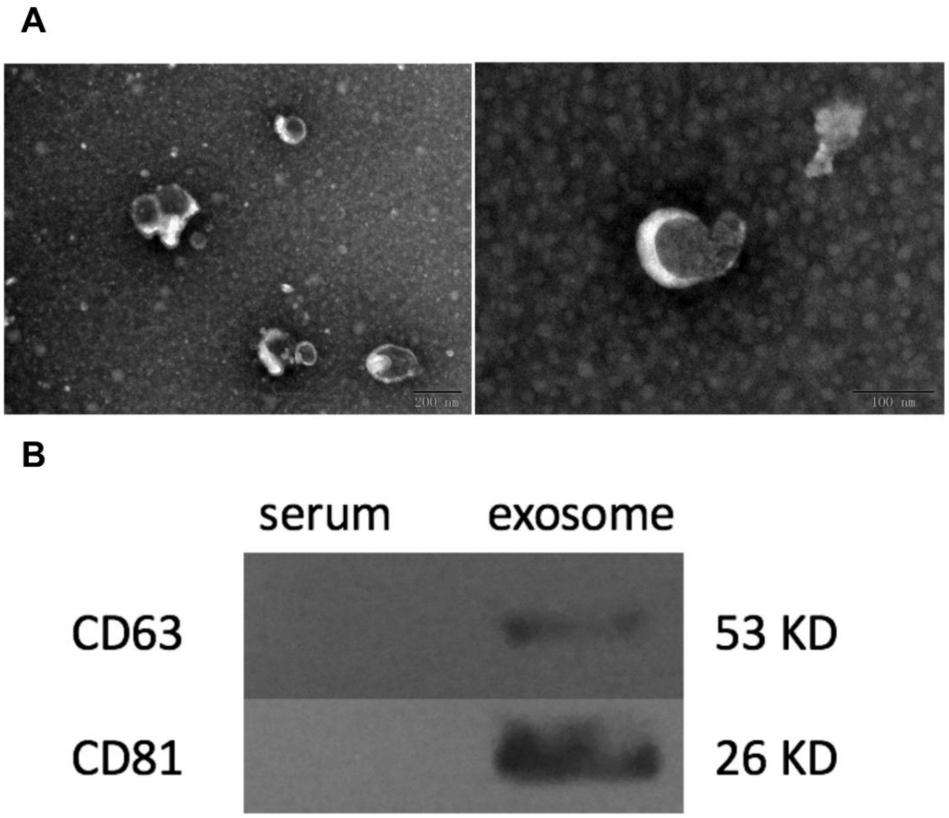

Supplementary Figure 4. Transmission Electron Microscopy (TEM) and Western Blot were used to verify the isolated exosomes from blood plasma. (**A**) Representative exosome image obtained from a JEM-1200EX transmission electron microscope. Scale bars represent 100 nm and 200 nm. (**B**) the expression of exosomal markers CD81 and CD63 were performed by Western Blot.
