# Supplementary material for: Cross-platform genomic identification and clinical validation of breast cancer diagnostic biomarkers
Source: Aging (Albany NY). 2021 Jan 20;13(3):4258–73. doi: 10.18632/aging.202388 (PMC7906147; doi:10.18632/aging.202388)
Supplement: Supplementary Table 4 [file aging-13-202388-s005.pdf]

**Supplementary Table 4. Co-expression network between breast cancer patients and controls.**

| gene1 | lncRNA | gene2 | COR | Z-score | P-value |
|---|---|---|---|---|---|
| APOBEC3B | ENST00000390540.2 | ENSG00000254140.1 | 0.804625965 | 1.79628287 | 0.072449541 |
| APOBEC3B | ENST00000413969.1 | ENSG00000224189.2 | -0.89532516 | -1.972908339 | 0.048506006 |
| APOBEC3B | ENST00000414377.1 | ENSG00000230470.1 | -0.873370622 | -1.932920095 | 0.053246042 |
| APOBEC3B | ENST00000415106.1 | ENSG00000226733.1 | -0.880158151 | -1.99411181 | 0.046139837 |
| APOBEC3B | ENST00000417782.1 | ENSG00000228587.1 | -0.842374873 | -1.866115715 | 0.062025178 |
| APOBEC3B | ENST00000418621.1 | ENSG00000224731.1 | -0.877901636 | -1.970671432 | 0.04876147 |
| APOBEC3B | ENST00000420830.1 | ENSG00000231512.1 | -0.881829911 | -1.96467023 | 0.049452418 |
| APOBEC3B | ENST00000421006.1 | ENSG00000234548.1 | -0.833449186 | -1.870564706 | 0.06140544 |
| APOBEC3B | ENST00000422763.1 | ENSG00000231131.2 | -0.800903293 | -1.774748564 | 0.075939413 |
| APOBEC3B | ENST00000426213.1 | ENSG00000223660.1 | 0.814382743 | 1.827169145 | 0.067674342 |
| APOBEC3B | ENST00000427524.1 | ENSG00000236065.2 | 0.918835638 | 2.046525193 | 0.040704726 |
| APOBEC3B | ENST00000428765.1 | ENSG00000230107.1 | 0.815462707 | 1.847096872 | 0.064733102 |
| APOBEC3B | ENST00000429080.1 | ENSG00000233047.1 | -0.819455149 | -1.826237546 | 0.067814488 |
| APOBEC3B | ENST00000433174.1 | ENSG00000162947.4 | -0.853885487 | -1.895830576 | 0.057982451 |
| APOBEC3B | ENST00000434292.1 | ENSG00000229796.1 | -0.837583282 | -1.8740706 | 0.060920695 |
| APOBEC3B | ENST00000435271.1 | ENSG00000231132.1 | 0.852731951 | 1.929445184 | 0.053675619 |
| APOBEC3B | ENST00000435832.1 | ENSG00000229201.1 | -0.958409914 | -2.142540199 | 0.032150037 |
| APOBEC3B | ENST00000437680.1 | ENSG00000237133.1 | 0.967515599 | 2.156698228 | 0.031029185 |
| APOBEC3B | ENST00000438850.1 | ENSG00000267338.1 | -0.83574481 | -1.847761029 | 0.064636921 |
| APOBEC3B | ENST00000438969.2 | ENSG00000228031.2 | -0.894224433 | -2.007066193 | 0.044742615 |
| APOBEC3B | ENST00000440038.2 | ENSG00000237094.7 | 0.810162722 | 1.814658973 | 0.069576334 |
| APOBEC3B | ENST00000448001.1 | ENSG00000229639.1 | -0.888010204 | -1.984441433 | 0.047206651 |
| APOBEC3B | ENST00000448431.1 | ENSG00000232548.1 | -0.876170926 | -1.98707467 | 0.046914123 |
| APOBEC3B | ENST00000448674.1 | ENSG00000235119.1 | 0.838173102 | 1.872199346 | 0.06117903 |
| APOBEC3B | ENST00000450206.1 | ENSG00000234311.1 | 0.912169762 | 2.03081178 | 0.042274092 |
| APOBEC3B | ENST00000450365.1 | ENSG00000224404.1 | -0.831977695 | -1.843549117 | 0.065248877 |
| APOBEC3B | ENST00000451556.2 | ENSG00000228386.2 | -0.846324169 | -1.896355374 | 0.057913069 |
| APOBEC3B | ENST00000451575.2 | ENSG00000224251.2 | -0.868290328 | -1.93310478 | 0.053223291 |
| APOBEC3B | ENST00000452553.1 | ENSG00000233973.1 | -0.811644576 | -1.826709701 | 0.067743429 |
| APOBEC3B | ENST00000453878.1 | ENSG00000224850.1 | -0.816437574 | -1.815653126 | 0.069423599 |
| APOBEC3B | ENST00000455416.1 | ENSG00000229337.1 | 0.893292535 | 1.999244282 | 0.045581929 |
| APOBEC3B | ENST00000457632.1 | ENSG00000234248.1 | -0.841906232 | -1.889361659 | 0.058843385 |
| APOBEC3B | ENST00000483283.1 | ENSG00000240571.1 | -0.807067624 | -1.786374117 | 0.074038704 |
| APOBEC3B | ENST00000502684.1 | ENSG00000251670.1 | 0.808205864 | 1.818382207 | 0.069005736 |
| APOBEC3B | ENST00000504017.1 | ENSG00000248388.1 | -0.803276738 | -1.825266972 | 0.067960752 |
| APOBEC3B | ENST00000505575.1 | ENSG00000248939.1 | 0.841007174 | 1.887612876 | 0.05907794 |
| APOBEC3B | ENST00000506059.1 | ENSG00000248311.1 | -0.82059974 | -1.832679426 | 0.066850259 |
| APOBEC3B | ENST00000508081.1 | ENSG00000248254.1 | 0.865683561 | 1.943515511 | 0.051953903 |
| APOBEC3B | ENST00000508191.1 | ENSG00000250910.3 | -0.8400599 | -1.875433392 | 0.060733125 |
| APOBEC3B | ENST00000508823.1 | ENSG00000250716.1 | -0.838404994 | -1.883722283 | 0.059602551 |
| APOBEC3B | ENST00000509629.1 | ENSG00000250164.1 | 0.81248272 | 1.819981327 | 0.068761849 |
| APOBEC3B | ENST00000513023.1 | ENSG00000248809.1 | -0.868519925 | -1.962514499 | 0.049702614 |
| APOBEC3B | ENST00000514802.1 | ENSG00000250190.1 | -0.906156995 | -2.020799591 | 0.043300515 |
| APOBEC3B | ENST00000518416.1 | ENSG00000253901.1 | -0.81276659 | -1.819165692 | 0.068886155 |
| APOBEC3B | ENST00000519005.1 | ENSG00000253507.1 | -0.835484177 | -1.885578532 | 0.059351773 |
| APOBEC3B | ENST00000519695.1 | ENSG00000253507.1 | -0.827415935 | -1.839242294 | 0.065879556 |
| APOBEC3B | ENST00000520192.1 | ENSG00000253807.1 | -0.834258858 | -1.848937931 | 0.064466776 |
| APOBEC3B | ENST00000521294.1 | ENSG00000253664.1 | 0.937601515 | 2.119178248 | 0.034075406 |
| APOBEC3B | ENST00000521378.1 | ENSG00000254222.1 | 0.92615122 | 2.065474925 | 0.038878086 |

| | | | | | |
|---|---|---|---|---|---|
| APOBEC3B | ENST00000522300.1 | ENSG00000249484.4 | -0.875475396 | -1.974676133 | 0.048304913 |
| APOBEC3B | ENST00000523806.1 | ENSG00000253616.1 | 0.955672223 | 2.124871918 | 0.033597302 |
| APOBEC3B | ENST00000523935.1 | ENSG00000253567.1 | -0.883176359 | -1.992356171 | 0.046331993 |
| APOBEC3B | ENST00000524133.1 | ENSG00000253174.2 | 0.82383552 | 1.853762056 | 0.063773209 |
| APOBEC3B | ENST00000524275.1 | ENSG00000253507.1 | -0.828423427 | -1.855051056 | 0.063588936 |
| APOBEC3B | ENST00000527100.1 | ENSG00000255015.1 | -0.898200786 | -2.030811934 | 0.042274076 |
| APOBEC3B | ENST00000527274.2 | ENSG00000255517.2 | 0.91103676 | 2.036200029 | 0.041730273 |
| APOBEC3B | ENST00000527727.1 | ENSG00000255227.1 | -0.92214621 | -2.068829139 | 0.038562123 |
| APOBEC3B | ENST00000529875.1 | ENSG00000254404.1 | 0.916924892 | 2.038477542 | 0.041502199 |
| APOBEC3B | ENST00000531157.1 | ENSG00000254754.1 | -0.857184873 | -1.906847202 | 0.056540377 |
| APOBEC3B | ENST00000535315.1 | ENSG00000250748.2 | -0.970602857 | -2.201964653 | 0.027667805 |
| APOBEC3B | ENST00000536529.1 | ENSG00000256422.1 | -0.905783077 | -2.031552107 | 0.042199019 |
| APOBEC3B | ENST00000538641.1 | ENSG00000256422.1 | -0.915010969 | -2.051282781 | 0.040239416 |
| APOBEC3B | ENST00000541391.1 | ENSG00000256268.1 | -0.971563132 | -2.156768613 | 0.031023698 |
| APOBEC3B | ENST00000549329.1 | ENSG00000224189.2 | -0.845589971 | -1.896984934 | 0.057829927 |
| APOBEC3B | ENST00000549616.1 | ENSG00000258168.1 | -0.938920353 | -2.091849298 | 0.036451999 |
| APOBEC3B | ENST00000550805.1 | ENSG00000244306.5 | 0.838225973 | 1.88216334 | 0.059813842 |
| APOBEC3B | ENST00000552156.1 | ENSG00000224189.2 | -0.804728071 | -1.800856415 | 0.071725514 |
| APOBEC3B | ENST00000552261.1 | ENSG00000257959.1 | -0.92379461 | -2.074631858 | 0.038020667 |
| APOBEC3B | ENST00000552541.1 | ENSG00000258294.1 | -0.9086027 | -2.01481127 | 0.043924428 |
| APOBEC3B | ENST00000552634.1 | ENSG00000257496.1 | 0.839590001 | 1.890498718 | 0.058691292 |
| APOBEC3B | ENST00000553477.1 | ENSG00000259123.1 | -0.957299476 | -2.1158283 | 0.034359413 |
| APOBEC3B | ENST00000555636.1 | ENSG00000259072.1 | -0.844074479 | -1.846439003 | 0.064828488 |
| APOBEC3B | ENST00000555913.1 | ENSG00000259077.1 | -0.884468964 | -1.992784367 | 0.046285064 |
| APOBEC3B | ENST00000556458.1 | ENSG00000258504.2 | 0.894465142 | 1.994683095 | 0.046077454 |
| APOBEC3B | ENST00000557368.1 | ENSG00000258444.1 | 0.910206588 | 2.024487916 | 0.042919974 |
| APOBEC3B | ENST00000557903.1 | ENSG00000259182.1 | -0.809785875 | -1.816959465 | 0.06922332 |
| APOBEC3B | ENST00000558221.1 | ENSG00000259704.1 | 0.877494505 | 1.9574824 | 0.050290779 |
| APOBEC3B | ENST00000563855.1 | ENSG00000260658.1 | -0.85111173 | -1.908838938 | 0.056282872 |
| APOBEC3B | ENST00000564809.1 | ENSG00000261471.1 | 0.815463657 | 1.851802229 | 0.064054228 |
| APOBEC3B | ENST00000565310.1 | ENSG00000261118.1 | 0.804067651 | 1.788536196 | 0.073689541 |
| APOBEC3B | ENST00000565735.1 | ENSG00000261213.1 | -0.870848614 | -1.935370175 | 0.052944887 |
| APOBEC3B | ENST00000568410.1 | ENSG00000260277.1 | -0.81770057 | -1.816463091 | 0.069299365 |
| APOBEC3B | ENST00000569313.1 | ENSG00000261604.1 | -0.880917709 | -1.965205273 | 0.049390484 |
| APOBEC3B | ENST00000569849.1 | ENSG00000260640.1 | -0.900540173 | -2.021158123 | 0.043263399 |
| APOBEC3B | ENST00000572417.1 | ENSG00000263171.1 | -0.936611965 | -2.099996115 | 0.035729183 |
| APOBEC3B | ENST00000573861.1 | ENSG00000263320.1 | -0.80781845 | -1.814691695 | 0.069571302 |
| APOBEC3B | ENST00000576021.1 | ENSG00000262413.1 | 0.841834769 | 1.868457346 | 0.06169835 |
| APOBEC3B | ENST00000578035.1 | ENSG00000266743.1 | 0.916968639 | 2.04520483 | 0.040834669 |
| APOBEC3B | ENST00000578349.1 | ENSG00000263688.1 | -0.865499471 | -1.918894187 | 0.054997726 |
| APOBEC3B | ENST00000578572.1 | ENSG00000196295.7 | -0.9204378 | -2.075584762 | 0.037932372 |
| APOBEC3B | ENST00000579775.1 | ENSG00000264108.1 | 0.940372168 | 2.096387688 | 0.036047814 |
| APOBEC3B | ENST00000581996.1 | ENSG00000265778.1 | -0.845937685 | -1.901070863 | 0.057292731 |
| APOBEC3B | ENST00000582348.1 | ENSG00000265148.1 | -0.856878337 | -1.896438864 | 0.057902037 |
| APOBEC3B | ENST00000586952.1 | ENSG00000226994.3 | -0.819166942 | -1.844746325 | 0.065074449 |
| APOBEC3B | ENST00000588177.1 | ENSG00000234899.5 | -0.860211432 | -1.948527705 | 0.051351853 |
| APOBEC3B | ENST00000589380.1 | ENSG00000267488.1 | 0.866810885 | 1.921308216 | 0.054692862 |
| APOBEC3B | ENST00000590357.1 | ENSG00000267175.1 | 0.825058173 | 1.861388853 | 0.062689284 |
| APOBEC3B | ENST00000591469.1 | ENSG00000267374.1 | -0.903036918 | -2.004530597 | 0.045013253 |
| APOBEC3B | ENST00000591621.1 | ENSG00000232116.2 | -0.95686605 | -2.167061001 | 0.030230203 |
| APOBEC3B | ENST00000592022.1 | ENSG00000267383.2 | -0.883139434 | -1.966153731 | 0.049280855 |

| | | | | | |
|---|---|---|---|---|---|
| APOBEC3B | ENST00000592431.1 | ENSG00000267475.1 | 0.877122519 | 1.938555137 | 0.052555531 |
| APOBEC3B | ENST00000592498.1 | ENSG00000267488.1 | 0.867693362 | 1.946149286 | 0.05163681 |
| APOBEC3B | ENST00000593175.1 | ENSG00000229036.3 | -0.935712008 | -2.084903879 | 0.037078027 |
| APOBEC3B | ENST00000593486.1 | ENSG00000250910.3 | -0.891049679 | -1.97349034 | 0.048439724 |
| APOBEC3B | ENST00000593599.1 | ENSG00000231898.4 | -0.862781482 | -1.95385553 | 0.050718305 |
| APOBEC3B | ENST00000594589.1 | ENSG00000269321.1 | 0.897633158 | 2.025716358 | 0.042793859 |
| APOBEC3B | ENST00000594762.1 | ENSG00000231898.4 | -0.805953673 | -1.812000556 | 0.069986111 |
| APOBEC3B | ENST00000594776.1 | ENSG00000269807.1 | 0.841585589 | 1.889864988 | 0.058776019 |
| APOBEC3B | ENST00000594850.1 | ENSG00000268093.1 | 0.878059539 | 1.965631247 | 0.049341222 |
| APOBEC3B | ENST00000596567.1 | ENSG00000226647.2 | -0.874707357 | -1.967518965 | 0.049123412 |
| APOBEC3B | ENST00000602790.1 | ENSG00000270000.1 | 0.836921333 | 1.87810921 | 0.060366227 |
| APOBEC3B | ENST00000602835.1 | ENSG00000270096.1 | -0.816051757 | -1.811289183 | 0.070096099 |
| APOBEC3B | ENST00000605692.1 | ENSG00000270810.1 | 0.832649347 | 1.886008641 | 0.05929379 |
| APOBEC3B | ENST00000605780.1 | ENSG00000270755.1 | 0.879183772 | 1.957576937 | 0.050279676 |
| APOBEC3B | ENST00000606869.1 | ENSG00000272349.1 | -0.855042946 | -1.909315984 | 0.056221342 |
| APOBEC3B | ENST00000606898.1 | ENSG00000272094.1 | 0.91990619 | 2.068927585 | 0.038552883 |
| APOBEC3B | ENST00000607715.1 | ENSG00000271788.1 | -0.837749648 | -1.90152257 | 0.057233599 |
| APOBEC3B | ENST00000607769.1 | ENSG00000272438.1 | 0.813297107 | 1.80664986 | 0.07081689 |
| APOBEC3B | ENST00000608943.1 | ENSG00000273368.1 | -0.892195278 | -2.006949468 | 0.044755044 |
| APOBEC3B | ENST00000609934.1 | ENSG00000273271.1 | -0.903249343 | -2.001253217 | 0.045365109 |
| APOBEC3B | ENST00000609952.1 | ENSG00000233766.3 | -0.928613801 | -2.080097142 | 0.037516624 |
| APOBEC3B | NR_027402.1 | FAM223B | 0.842834347 | 1.86553501 | 0.06210645 |
| APOBEC3B | NR_033914.1 | LINC00254 | -0.958454413 | -2.123770869 | 0.033689308 |
| APOBEC3B | NR_038194.1 | LINC00583 | -0.831148829 | -1.870238432 | 0.061450715 |
| APOBEC3B | NR_040049.1 | SDCBP2-AS1 | 0.822981327 | 1.855036081 | 0.063591074 |
| APOBEC3B | NR_046845.1 | DNM3-IT1 | -0.833948303 | -1.866215276 | 0.062011253 |
| APOBEC3B | NR_046871.1 | LINC00333 | -0.904172028 | -2.014930012 | 0.043911983 |
| APOBEC3B | NR_047698.1 | VWC2L-IT1 | -0.834223182 | -1.863160731 | 0.062439656 |
| APOBEC3B | NR_103776.1 | CHRM3-AS2 | -0.931494044 | -2.077560037 | 0.0377499 |
| APOBEC3B | NR_104620.1 | LINC01672 | -0.801561041 | -1.771516444 | 0.076474861 |
| APOBEC3B | NR_104998.1 | LOC102467225 | -0.832891883 | -1.8657349 | 0.062078464 |
| APOBEC3B | NR_109877.1 | LINC01470 | -0.850713131 | -1.930090251 | 0.053595656 |
| APOBEC3B | NR_110123.1 | GRM7-AS3 | -0.853176854 | -1.882137912 | 0.059817293 |
| APOBEC3B | NR_110824.1 | LINC01986 | -0.994984939 | -2.244395102 | 0.024806991 |
| APOBEC3B | NR_125849.1 | LOC101928140 | -0.840248996 | -1.909384174 | 0.056212551 |
| APOBEC3B | NR_131204.1 | XACT | 0.94320223 | 2.098479203 | 0.035862835 |
| APOBEC3B | NR_133930.1 | LOC105375556 | -0.907851691 | -2.007481643 | 0.044698403 |
| APOBEC3B | NR_134632.1 | LOC105373051 | 0.931685948 | 2.069211406 | 0.038526253 |
| APOBEC3B | NR_135040.1 | LOC101927038 | 0.81558874 | 1.820905832 | 0.068621172 |
| APOBEC3B | NR_135041.1 | LOC101927038 | 0.824993028 | 1.823562653 | 0.068218217 |
| APOBEC3B | NR_135549.1 | LOC101929411 | -0.952627944 | -2.140351789 | 0.032326348 |
| APOBEC3B | NR_135840.1 | LOC105376114 | -0.801544242 | -1.77701829 | 0.075565231 |
| APOBEC3B | NR_136215.1 | VCAN-AS1 | -0.895154989 | -2.030157948 | 0.042340487 |
| APOBEC3B | NR_136218.1 | MEF2C-AS1 | -0.908964861 | -2.013694466 | 0.044041621 |
| APOBEC3B | NR_136320.1 | LOC105373656 | 0.882346393 | 1.952544066 | 0.050873644 |
| ARG1 | ENST00000295549.4 | ENSG00000163364.5 | 0.904983851 | 2.017135814 | 0.043681342 |
| ARG1 | ENST00000412896.1 | ENSG00000197585.5 | -0.827078435 | -1.829732598 | 0.067289934 |
| ARG1 | ENST00000413969.1 | ENSG00000224189.2 | 0.824433159 | 1.823131633 | 0.068283457 |
| ARG1 | ENST00000413989.1 | ENSG00000242628.1 | 0.801631202 | 1.790011027 | 0.073452139 |
| ARG1 | ENST00000414992.1 | ENSG00000233613.1 | -0.826314535 | -1.853990908 | 0.063740461 |
| ARG1 | ENST00000416641.1 | ENSG00000226956.1 | -0.839588924 | -1.904778299 | 0.056808896 |

| | | | | | |
|---|---|---|---|---|---|
| ARG1 | ENST00000417315.1 | ENSG00000242486.1 | -0.891466845 | -1.996965251 | 0.045828958 |
| ARG1 | ENST00000417782.1 | ENSG00000228587.1 | 0.802487574 | 1.832889898 | 0.066818946 |
| ARG1 | ENST00000418621.1 | ENSG00000224731.1 | 0.955028369 | 2.138903421 | 0.032443491 |
| ARG1 | ENST00000420830.1 | ENSG00000231512.1 | 0.844932954 | 1.890169905 | 0.05873524 |
| ARG1 | ENST00000421597.1 | ENSG00000227851.1 | 0.950485901 | 2.133324337 | 0.032898129 |
| ARG1 | ENST00000426125.1 | ENSG00000223653.1 | 0.829760324 | 1.86283855 | 0.062484985 |
| ARG1 | ENST00000426444.1 | ENSG00000239395.1 | 0.889574994 | 1.987256423 | 0.046893988 |
| ARG1 | ENST00000428160.1 | ENSG00000236897.1 | 0.967430297 | 2.172095729 | 0.029848443 |
| ARG1 | ENST00000428853.2 | ENSG00000229206.2 | 0.911493796 | 2.039729773 | 0.041377248 |
| ARG1 | ENST00000430842.1 | ENSG00000230433.1 | -0.913155542 | -2.034515995 | 0.041899598 |
| ARG1 | ENST00000433174.1 | ENSG00000162947.4 | 0.848804527 | 1.880562619 | 0.060031441 |
| ARG1 | ENST00000434292.1 | ENSG00000229796.1 | 0.890062745 | 1.979360256 | 0.04777546 |
| ARG1 | ENST00000434493.1 | ENSG00000224605.1 | 0.817033184 | 1.83456553 | 0.06657009 |
| ARG1 | ENST00000435271.1 | ENSG00000231132.1 | -0.87903307 | -1.950843384 | 0.051075679 |
| ARG1 | ENST00000437680.1 | ENSG00000237133.1 | -0.819657294 | -1.835104181 | 0.066490255 |
| ARG1 | ENST00000438850.1 | ENSG00000267338.1 | 0.838345677 | 1.859446812 | 0.062963832 |
| ARG1 | ENST00000441029.2 | ENSG00000229188.2 | -0.918892614 | -2.072739445 | 0.038196535 |
| ARG1 | ENST00000442831.1 | ENSG00000229550.1 | -0.89232637 | -1.969990546 | 0.048839454 |
| ARG1 | ENST00000442852.1 | ENSG00000237923.1 | -0.850098564 | -1.895736683 | 0.057994872 |
| ARG1 | ENST00000443306.1 | ENSG00000233891.3 | 0.864812537 | 1.899985858 | 0.057434976 |
| ARG1 | ENST00000445233.1 | ENSG00000233928.1 | 0.860372444 | 1.904735435 | 0.05681447 |
| ARG1 | ENST00000447111.1 | ENSG00000231903.1 | 0.857021074 | 1.940769484 | 0.052286244 |
| ARG1 | ENST00000447183.2 | ENSG00000271593.1 | 0.887127988 | 1.992939217 | 0.046268103 |
| ARG1 | ENST00000447709.1 | ENSG00000237473.1 | 0.855613773 | 1.919728519 | 0.0548922 |
| ARG1 | ENST00000448001.1 | ENSG00000229639.1 | 0.870853144 | 1.923809919 | 0.054378414 |
| ARG1 | ENST00000450206.1 | ENSG00000234311.1 | -0.808113202 | -1.804436502 | 0.071162905 |
| ARG1 | ENST00000451556.2 | ENSG00000228386.2 | 0.839995677 | 1.880406487 | 0.0600527 |
| ARG1 | ENST00000453579.1 | ENSG00000232529.1 | 0.951422817 | 2.104972922 | 0.035293665 |
| ARG1 | ENST00000453584.1 | ENSG00000233613.1 | -0.897952261 | -2.01295005 | 0.044119884 |
| ARG1 | ENST00000454489.1 | ENSG00000231403.1 | 0.85191702 | 1.895046357 | 0.05808626 |
| ARG1 | ENST00000469846.2 | ENSG00000206573.4 | -0.892006002 | -1.984296986 | 0.047222742 |
| ARG1 | ENST00000469931.2 | ENSG00000272030.1 | 0.802171487 | 1.786365424 | 0.07404011 |
| ARG1 | ENST00000472596.1 | ENSG00000239774.1 | 0.803447622 | 1.795868276 | 0.072515469 |
| ARG1 | ENST00000476099.1 | ENSG00000244158.1 | -0.834699374 | -1.878511363 | 0.060311244 |
| ARG1 | ENST00000480904.2 | ENSG00000206573.4 | -0.972226998 | -2.187267912 | 0.028722972 |
| ARG1 | ENST00000483283.1 | ENSG00000240571.1 | 0.920186488 | 2.079737703 | 0.037549598 |
| ARG1 | ENST00000498199.1 | ENSG00000206573.4 | -0.902034823 | -2.03025841 | 0.042330279 |
| ARG1 | ENST00000500498.2 | ENSG00000245311.2 | -0.885259338 | -1.946832824 | 0.05155478 |
| ARG1 | ENST00000501133.2 | ENSG00000246560.2 | 0.899676301 | 2.012299486 | 0.044188377 |
| ARG1 | ENST00000503323.1 | ENSG00000249881.1 | -0.833273273 | -1.872271779 | 0.061169013 |
| ARG1 | ENST00000504755.1 | ENSG00000250252.1 | 0.894102122 | 2.022449493 | 0.043129936 |
| ARG1 | ENST00000505575.1 | ENSG00000248939.1 | -0.945606976 | -2.141308549 | 0.032249164 |
| ARG1 | ENST00000506852.1 | ENSG00000250945.1 | 0.807176264 | 1.798520334 | 0.072094589 |
| ARG1 | ENST00000507997.1 | ENSG00000250551.1 | -0.80531717 | -1.779426201 | 0.075169914 |
| ARG1 | ENST00000508191.1 | ENSG00000250910.3 | 0.884291991 | 1.98033787 | 0.047665576 |
| ARG1 | ENST00000508823.1 | ENSG00000250716.1 | 0.965038356 | 2.155270132 | 0.031140702 |
| ARG1 | ENST00000509629.1 | ENSG00000250164.1 | -0.853754553 | -1.911160323 | 0.055983981 |
| ARG1 | ENST00000515750.1 | ENSG00000249061.1 | -0.858097215 | -1.945242477 | 0.051745802 |
| ARG1 | ENST00000518620.1 | ENSG00000253892.1 | -0.847093979 | -1.902382749 | 0.057121135 |
| ARG1 | ENST00000519375.1 | ENSG00000253980.1 | 0.838472973 | 1.835912418 | 0.066370611 |
| ARG1 | ENST00000520192.1 | ENSG00000253807.1 | 0.882527334 | 1.972882101 | 0.048508996 |

| | | | | | |
|---|---|---|---|---|---|
| ARG1 | ENST00000521378.1 | ENSG00000254222.1 | -0.84809248 | -1.895063071 | 0.058084046 |
| ARG1 | ENST00000522190.1 | ENSG00000254165.1 | 0.938660496 | 2.092433562 | 0.036399749 |
| ARG1 | ENST00000522426.1 | ENSG00000253538.1 | 0.964182446 | 2.172160402 | 0.029843566 |
| ARG1 | ENST00000523935.1 | ENSG00000253567.1 | 0.907967314 | 2.040546939 | 0.041295881 |
| ARG1 | ENST00000524073.1 | ENSG00000253774.1 | 0.817287441 | 1.840862264 | 0.065641746 |
| ARG1 | ENST00000524133.1 | ENSG00000253174.2 | -0.811145917 | -1.808219322 | 0.070572373 |
| ARG1 | ENST00000529875.1 | ENSG00000254404.1 | -0.82772109 | -1.832898458 | 0.066817673 |
| ARG1 | ENST00000531627.1 | ENSG00000254584.1 | 0.801582486 | 1.793586192 | 0.072879242 |
| ARG1 | ENST00000531661.1 | ENSG00000254473.1 | 0.863764811 | 1.935076916 | 0.052980858 |
| ARG1 | ENST00000531977.1 | ENSG00000224023.6 | 0.811023942 | 1.82019001 | 0.068730074 |
| ARG1 | ENST00000536412.1 | ENSG00000256072.1 | -0.825370258 | -1.866212717 | 0.062011611 |
| ARG1 | ENST00000541391.1 | ENSG00000256268.1 | 0.880214809 | 1.971146809 | 0.048707086 |
| ARG1 | ENST00000545642.1 | ENSG00000256342.1 | 0.830667078 | 1.848766527 | 0.064491533 |
| ARG1 | ENST00000547175.1 | ENSG00000257395.1 | -0.904441694 | -2.00847827 | 0.044592493 |
| ARG1 | ENST00000548199.1 | ENSG00000257614.1 | 0.894288374 | 1.99282618 | 0.046280484 |
| ARG1 | ENST00000549329.1 | ENSG00000224189.2 | 0.816738635 | 1.81035592 | 0.07024061 |
| ARG1 | ENST00000549616.1 | ENSG00000258168.1 | 0.825629225 | 1.831767505 | 0.066986067 |
| ARG1 | ENST00000550805.1 | ENSG00000244306.5 | -0.896380131 | -2.000804133 | 0.045413502 |
| ARG1 | ENST00000551067.1 | ENSG00000257891.1 | -0.89653942 | -1.988391966 | 0.046768357 |
| ARG1 | ENST00000552261.1 | ENSG00000257959.1 | 0.882170236 | 1.978626841 | 0.047858035 |
| ARG1 | ENST00000552558.1 | ENSG00000257947.1 | 0.921469276 | 2.046404222 | 0.040716617 |
| ARG1 | ENST00000553477.1 | ENSG00000259123.1 | 0.931550962 | 2.077122704 | 0.037790236 |
| ARG1 | ENST00000553537.1 | ENSG00000258481.1 | -0.873538639 | -1.982750351 | 0.047395322 |
| ARG1 | ENST00000556458.1 | ENSG00000258504.2 | -0.878915315 | -1.956550896 | 0.050400293 |
| ARG1 | ENST00000557903.1 | ENSG00000259182.1 | 0.911035532 | 2.038421631 | 0.041507785 |
| ARG1 | ENST00000562582.1 | ENSG00000259779.1 | 0.944333945 | 2.113475908 | 0.034560055 |
| ARG1 | ENST00000562834.1 | ENSG00000261116.1 | 0.817195419 | 1.836284894 | 0.066315533 |
| ARG1 | ENST00000563342.1 | ENSG00000259914.1 | 0.959892081 | 2.126806273 | 0.033436183 |
| ARG1 | ENST00000563570.1 | ENSG00000259961.1 | -0.942744187 | -2.111302401 | 0.034746328 |
| ARG1 | ENST00000563601.1 | ENSG00000260589.1 | -0.84263945 | -1.884068177 | 0.059555754 |
| ARG1 | ENST00000565271.1 | ENSG00000261335.1 | -0.870151002 | -1.95507938 | 0.050573702 |
| ARG1 | ENST00000568410.1 | ENSG00000260277.1 | 0.86244579 | 1.938415681 | 0.052572529 |
| ARG1 | ENST00000568836.1 | ENSG00000259967.1 | 0.847234195 | 1.912871454 | 0.055764511 |
| ARG1 | ENST00000569778.1 | ENSG00000260823.1 | -0.849897236 | -1.893821382 | 0.058248723 |
| ARG1 | ENST00000569849.1 | ENSG00000260640.1 | 0.923521694 | 2.071215782 | 0.038338635 |
| ARG1 | ENST00000572417.1 | ENSG00000263171.1 | 0.826957244 | 1.853462346 | 0.063816118 |
| ARG1 | ENST00000573861.1 | ENSG00000263320.1 | 0.860986048 | 1.924045987 | 0.054348819 |
| ARG1 | ENST00000576021.1 | ENSG00000262413.1 | -0.849492611 | -1.92081188 | 0.054755428 |
| ARG1 | ENST00000577360.1 | ENSG00000264273.1 | 0.934345147 | 2.079303533 | 0.037589461 |
| ARG1 | ENST00000578035.1 | ENSG00000266743.1 | -0.82274406 | -1.849383773 | 0.064402417 |
| ARG1 | ENST00000578334.1 | ENSG00000265148.1 | 0.868678762 | 1.935087089 | 0.05297961 |
| ARG1 | ENST00000578349.1 | ENSG00000263688.1 | 0.934482478 | 2.088427555 | 0.036759284 |
| ARG1 | ENST00000578572.1 | ENSG00000196295.7 | 0.888835069 | 1.989940212 | 0.046597522 |
| ARG1 | ENST00000580975.1 | ENSG00000266237.1 | 0.822354525 | 1.843202763 | 0.065299412 |
| ARG1 | ENST00000581996.1 | ENSG00000265778.1 | 0.899263605 | 2.011791517 | 0.044241919 |
| ARG1 | ENST00000583826.1 | ENSG00000265148.1 | 0.810998459 | 1.808741274 | 0.070491208 |
| ARG1 | ENST00000583841.1 | ENSG00000265148.1 | 0.865806256 | 1.949840239 | 0.051195164 |
| ARG1 | ENST00000585684.1 | ENSG00000267057.1 | 0.835031878 | 1.854902839 | 0.063610103 |
| ARG1 | ENST00000586297.1 | ENSG00000267633.1 | 0.946576142 | 2.113902808 | 0.034523569 |
| ARG1 | ENST00000586399.1 | ENSG00000228430.4 | 0.860726523 | 1.891382051 | 0.058573363 |
| ARG1 | ENST00000588177.1 | ENSG00000234899.5 | 0.831054809 | 1.874539634 | 0.060856085 |

| | | | | | |
|---|---|---|---|---|---|
| ARG1 | ENST00000588835.1 | ENSG00000267476.1 | 0.929992281 | 2.087870049 | 0.036809559 |
| ARG1 | ENST00000589983.1 | ENSG00000267057.1 | 0.805859766 | 1.802503992 | 0.071466147 |
| ARG1 | ENST00000590357.1 | ENSG00000267175.1 | -0.809686195 | -1.8175843 | 0.069127693 |
| ARG1 | ENST00000591469.1 | ENSG00000267374.1 | 0.899517748 | 2.023045136 | 0.043068494 |
| ARG1 | ENST00000591621.1 | ENSG00000232116.2 | 0.857113289 | 1.938530491 | 0.052558535 |
| ARG1 | ENST00000592022.1 | ENSG00000267383.2 | 0.835174625 | 1.830517485 | 0.067172595 |
| ARG1 | ENST00000592431.1 | ENSG00000267475.1 | -0.90728041 | -2.068991523 | 0.038546882 |
| ARG1 | ENST00000593486.1 | ENSG00000250910.3 | 0.957608906 | 2.122918728 | 0.033760663 |
| ARG1 | ENST00000593599.1 | ENSG00000231898.4 | 0.95961007 | 2.142890596 | 0.032121884 |
| ARG1 | ENST00000595892.1 | ENSG00000269640.1 | 0.889978278 | 1.975548447 | 0.048205942 |
| ARG1 | ENST00000596135.1 | ENSG00000269843.1 | 0.822279786 | 1.863194138 | 0.062434957 |
| ARG1 | ENST00000598065.1 | ENSG00000231731.3 | -0.873827664 | -1.978614527 | 0.047859423 |
| ARG1 | ENST00000599050.1 | ENSG00000268366.1 | 0.984987596 | 2.214387912 | 0.026802106 |
| ARG1 | ENST00000600365.1 | ENSG00000231898.4 | 0.909300465 | 2.038358932 | 0.04151405 |
| ARG1 | ENST00000601511.1 | ENSG00000244513.2 | 0.912254937 | 2.064941233 | 0.038928561 |
| ARG1 | ENST00000602443.1 | ENSG00000270076.1 | 0.802058424 | 1.794978869 | 0.072657067 |
| ARG1 | ENST00000602485.1 | ENSG00000270163.1 | -0.935500238 | -2.134098586 | 0.032834712 |
| ARG1 | ENST00000605692.1 | ENSG00000270810.1 | -0.821000477 | -1.829509 | 0.067323392 |
| ARG1 | ENST00000606898.1 | ENSG00000272094.1 | -0.908031093 | -2.038588082 | 0.041491156 |
| ARG1 | ENST00000606938.1 | ENSG00000272198.1 | -0.822516817 | -1.817667575 | 0.069114956 |
| ARG1 | ENST00000607715.1 | ENSG00000271788.1 | 0.954284255 | 2.153948235 | 0.031244232 |
| ARG1 | ENST00000608133.1 | ENSG00000273193.1 | 0.829391319 | 1.856258495 | 0.063416722 |
| ARG1 | ENST00000608173.1 | ENSG00000197099.4 | 0.861864146 | 1.929406303 | 0.053680442 |
| ARG1 | ENST00000608289.1 | ENSG00000272958.1 | -0.872359748 | -1.913912475 | 0.05563134 |
| ARG1 | ENST00000608856.1 | ENSG00000272600.1 | -0.808292868 | -1.801095758 | 0.071687788 |
| ARG1 | ENST00000609349.1 | ENSG00000272861.1 | 0.928287734 | 2.077292374 | 0.037774582 |
| ARG1 | ENST00000609890.1 | ENSG00000231898.4 | 0.879190232 | 1.960079561 | 0.049986492 |
| ARG1 | NR_022011.1 | PWARSN | 0.9257019 | 2.067333983 | 0.038702694 |
| ARG1 | NR_027402.1 | FAM223B | -0.824393848 | -1.846263278 | 0.064853987 |
| ARG1 | NR_027440.1 | LOC100272217 | -0.929974448 | -2.07249927 | 0.038218904 |
| ARG1 | NR_033914.1 | LINC00254 | 0.871693156 | 1.961557761 | 0.049813994 |
| ARG1 | NR_038194.1 | LINC00583 | 0.980185898 | 2.168803173 | 0.03009763 |
| ARG1 | NR_040001.2 | LINC01116 | 0.86047596 | 1.918983945 | 0.054986366 |
| ARG1 | NR_046556.1 | RBMS3-AS1 | 0.815563965 | 1.829017412 | 0.067397 |
| ARG1 | NR_046578.1 | CACNA1C-AS4 | 0.904969418 | 2.029158044 | 0.042442195 |
| ARG1 | NR_046748.1 | ARHGAP31-AS1 | 0.883967774 | 1.975156065 | 0.04825044 |
| ARG1 | NR_046766.1 | ATP2B2-IT2 | 0.943030898 | 2.117386896 | 0.034227025 |
| ARG1 | NR_047040.1 | LINC00424 | 0.889660797 | 1.954256254 | 0.05067092 |
| ARG1 | NR_047698.1 | VWC2L-IT1 | 0.859092846 | 1.916229867 | 0.055335843 |
| ARG1 | NR_104618.1 | LINC01017 | 0.969093372 | 2.141995229 | 0.032193866 |
| ARG1 | NR_104620.1 | LINC01672 | 0.885938435 | 1.998021912 | 0.045714285 |
| ARG1 | NR_104998.1 | LOC102467225 | 0.866803196 | 1.950897046 | 0.051069294 |
| ARG1 | NR_109975.1 | ARNTL2-AS1 | -0.878018223 | -1.95077771 | 0.051083494 |
| ARG1 | NR_110053.1 | LOC101927464 | 0.830667078 | 1.850835514 | 0.06419322 |
| ARG1 | NR_110504.1 | LOC101929572 | -0.821605287 | -1.831040081 | 0.067094561 |
| ARG1 | NR_120330.1 | LOC101928227 | -0.951703713 | -2.141698916 | 0.032217718 |
| ARG1 | NR_120466.1 | LINC01489 | 0.852263068 | 1.901901122 | 0.057184083 |
| ARG1 | NR_120527.1 | LOC100506675 | 0.900875112 | 2.030985377 | 0.042256478 |
| ARG1 | NR_120566.1 | LOC101928896 | 0.872479992 | 1.954284335 | 0.0506676 |
| ARG1 | NR_125769.1 | LINC01269 | -0.883086636 | -1.967692736 | 0.049103402 |
| ARG1 | NR_126041.1 | LOC101930071 | -0.804083371 | -1.810533132 | 0.070213151 |

| | | | | | |
|---|---|---|---|---|---|
| ARG1 | NR_130916.1 | LOC105274304 | -0.877580954 | -1.94609935 | 0.051642807 |
| ARG1 | NR_131186.1 | LOC105377348 | 0.869017157 | 1.952976534 | 0.050822376 |
| ARG1 | NR_134610.1 | LOC105375014 | -0.84293787 | -1.889082154 | 0.058880821 |
| ARG1 | NR_134632.1 | LOC105373051 | -0.885998232 | -1.978884883 | 0.047828969 |
| ARG1 | NR_135076.1 | LOC102723838 | 0.871861399 | 1.920630507 | 0.054778306 |
| ARG1 | NR_135274.1 | LOC105370619 | 0.81008166 | 1.804806543 | 0.07110496 |
| ARG1 | NR_135549.1 | LOC101929411 | 0.947516015 | 2.122351151 | 0.033808261 |
| ARG1 | NR_135840.1 | LOC105376114 | 0.917799459 | 2.064597548 | 0.038961095 |
| ARG1 | NR_136218.1 | MEF2C-AS1 | 0.947647306 | 2.096644677 | 0.036025041 |
| ASPN | ENST00000295549.4 | ENSG00000163364.5 | 0.80974758 | 1.795145874 | 0.072630462 |
| ASPN | ENST00000412896.1 | ENSG00000197585.5 | -0.813415447 | -1.798606481 | 0.072080951 |
| ASPN | ENST00000413650.1 | ENSG00000230880.2 | 0.836079283 | 1.885760311 | 0.059327261 |
| ASPN | ENST00000413969.1 | ENSG00000224189.2 | 0.870867652 | 1.938376829 | 0.052577265 |
| ASPN | ENST00000413989.1 | ENSG00000242628.1 | 0.809598756 | 1.82666036 | 0.067750852 |
| ASPN | ENST00000414992.1 | ENSG00000233613.1 | -0.83983872 | -1.878911059 | 0.060256639 |
| ASPN | ENST00000416641.1 | ENSG00000226956.1 | -0.955302863 | -2.12983988 | 0.033184835 |
| ASPN | ENST00000416657.1 | ENSG00000235858.1 | 0.839899101 | 1.880755476 | 0.060005189 |
| ASPN | ENST00000417315.1 | ENSG00000242486.1 | -0.818465449 | -1.8249909 | 0.068002403 |
| ASPN | ENST00000417426.1 | ENSG00000233145.1 | 0.819721939 | 1.83370098 | 0.066698393 |
| ASPN | ENST00000417782.1 | ENSG00000228587.1 | 0.822058712 | 1.844456495 | 0.06511664 |
| ASPN | ENST00000418621.1 | ENSG00000224731.1 | 0.845616406 | 1.897558794 | 0.057754227 |
| ASPN | ENST00000420830.1 | ENSG00000231512.1 | 0.83154411 | 1.855120473 | 0.063579025 |
| ASPN | ENST00000421597.1 | ENSG00000227851.1 | 0.87913177 | 1.968649359 | 0.04899337 |
| ASPN | ENST00000422038.1 | ENSG00000227935.1 | 0.869103575 | 1.958435174 | 0.050178971 |
| ASPN | ENST00000425371.2 | ENSG00000235872.2 | 0.844342986 | 1.884508615 | 0.059496211 |
| ASPN | ENST00000426213.1 | ENSG00000223660.1 | -0.89168531 | -1.992362493 | 0.0463313 |
| ASPN | ENST00000426504.1 | ENSG00000234190.1 | -0.820782014 | -1.842473397 | 0.065405934 |
| ASPN | ENST00000428160.1 | ENSG00000236897.1 | 0.941396328 | 2.101772741 | 0.035573188 |
| ASPN | ENST00000428440.1 | ENSG00000232827.2 | -0.888874425 | -1.98232411 | 0.047442977 |
| ASPN | ENST00000430842.1 | ENSG00000230433.1 | -0.869079483 | -1.96950793 | 0.048894793 |
| ASPN | ENST00000432957.1 | ENSG00000231534.1 | -0.911777993 | -2.037077285 | 0.041642298 |
| ASPN | ENST00000433174.1 | ENSG00000162947.4 | 0.814822334 | 1.816136198 | 0.069349482 |
| ASPN | ENST00000434493.1 | ENSG00000224605.1 | 0.834661822 | 1.86532588 | 0.06213574 |
| ASPN | ENST00000435271.1 | ENSG00000231132.1 | -0.839800758 | -1.892144896 | 0.058471678 |
| ASPN | ENST00000437680.1 | ENSG00000237133.1 | -0.894842468 | -2.024232717 | 0.042946213 |
| ASPN | ENST00000438850.1 | ENSG00000267338.1 | 0.961755695 | 2.146564922 | 0.031827935 |
| ASPN | ENST00000442831.1 | ENSG00000229550.1 | -0.950028606 | -2.106960764 | 0.035120981 |
| ASPN | ENST00000443306.1 | ENSG00000233891.3 | 0.857903832 | 1.931266778 | 0.05345007 |
| ASPN | ENST00000447183.2 | ENSG00000271593.1 | 0.940521832 | 2.085124316 | 0.037058018 |
| ASPN | ENST00000447514.1 | ENSG00000236753.1 | 0.828757253 | 1.870217397 | 0.061453635 |
| ASPN | ENST00000447709.1 | ENSG00000237473.1 | 0.907031921 | 2.021114456 | 0.043267918 |
| ASPN | ENST00000448001.1 | ENSG00000229639.1 | 0.963546998 | 2.137824539 | 0.032530987 |
| ASPN | ENST00000448650.1 | ENSG00000223536.1 | -0.801462603 | -1.810403981 | 0.070233162 |
| ASPN | ENST00000450206.1 | ENSG00000234311.1 | -0.838702067 | -1.875548003 | 0.060717372 |
| ASPN | ENST00000450848.1 | ENSG00000225539.1 | 0.805041629 | 1.794601613 | 0.072717196 |
| ASPN | ENST00000452412.1 | ENSG00000233860.1 | 0.831144847 | 1.883859867 | 0.059583933 |
| ASPN | ENST00000453579.1 | ENSG00000232529.1 | 0.962562972 | 2.195521878 | 0.028126183 |
| ASPN | ENST00000453584.1 | ENSG00000233613.1 | -0.83719041 | -1.878912061 | 0.060256502 |
| ASPN | ENST00000453878.1 | ENSG00000224850.1 | 0.822544883 | 1.844554266 | 0.065102405 |
| ASPN | ENST00000454489.1 | ENSG00000231403.1 | 0.953507637 | 2.133226319 | 0.032906165 |
| ASPN | ENST00000454526.1 | ENSG00000234136.1 | 0.913871782 | 2.020031911 | 0.043380078 |

| | | | | | |
|---|---|---|---|---|---|
| ASPN | ENST00000454709.1 | ENSG00000237280.1 | -0.8509391 | -1.920446283 | 0.054801552 |
| ASPN | ENST00000454928.1 | ENSG00000186148.7 | 0.800316576 | 1.817579701 | 0.069128396 |
| ASPN | ENST00000457848.1 | ENSG00000226412.1 | 0.801102259 | 1.823761737 | 0.068188101 |
| ASPN | ENST00000469846.2 | ENSG00000206573.4 | -0.816085736 | -1.824918042 | 0.068013398 |
| ASPN | ENST00000469931.2 | ENSG00000272030.1 | 0.846272172 | 1.890948467 | 0.058631224 |
| ASPN | ENST00000476099.1 | ENSG00000244158.1 | -0.927397301 | -2.092672397 | 0.036378409 |
| ASPN | ENST00000480904.2 | ENSG00000206573.4 | -0.90822139 | -2.020430358 | 0.043338767 |
| ASPN | ENST00000482142.1 | ENSG00000243276.1 | 0.853773606 | 1.905802595 | 0.056675822 |
| ASPN | ENST00000485347.1 | ENSG00000239991.1 | -0.86982997 | -1.966059293 | 0.049291762 |
| ASPN | ENST00000498199.1 | ENSG00000206573.4 | -0.814146283 | -1.835588077 | 0.066418602 |
| ASPN | ENST00000501133.2 | ENSG00000246560.2 | 0.886418832 | 1.978385206 | 0.047885267 |
| ASPN | ENST00000501405.2 | ENSG00000247402.2 | -0.88025355 | -1.960133555 | 0.049980182 |
| ASPN | ENST00000502684.1 | ENSG00000251670.1 | -0.86547672 | -1.932932115 | 0.053244561 |
| ASPN | ENST00000504755.1 | ENSG00000250252.1 | 0.866138072 | 1.933636101 | 0.053157885 |
| ASPN | ENST00000505575.1 | ENSG00000248939.1 | -0.863931758 | -1.940042849 | 0.052374483 |
| ASPN | ENST00000506059.1 | ENSG00000248311.1 | 0.919557692 | 2.047868213 | 0.040572914 |
| ASPN | ENST00000508081.1 | ENSG00000248254.1 | -0.819632825 | -1.83274672 | 0.066840246 |
| ASPN | ENST00000508823.1 | ENSG00000250716.1 | 0.831805386 | 1.850799605 | 0.064198388 |
| ASPN | ENST00000508845.1 | ENSG00000271724.1 | 0.856959025 | 1.925659012 | 0.054146965 |
| ASPN | ENST00000509629.1 | ENSG00000250164.1 | -0.805253634 | -1.801984107 | 0.071547906 |
| ASPN | ENST00000514459.1 | ENSG00000248211.1 | -0.883553331 | -1.960723561 | 0.049911279 |
| ASPN | ENST00000514802.1 | ENSG00000250190.1 | 0.800182149 | 1.803838812 | 0.071256579 |
| ASPN | ENST00000515750.1 | ENSG00000249061.1 | -0.858474295 | -1.904606846 | 0.056831196 |
| ASPN | ENST00000517716.1 | ENSG00000253515.1 | -0.819895559 | -1.813036157 | 0.069826245 |
| ASPN | ENST00000518416.1 | ENSG00000253901.1 | 0.881393237 | 1.973365711 | 0.048453911 |
| ASPN | ENST00000518620.1 | ENSG00000253892.1 | -0.900441369 | -2.000660697 | 0.045428968 |
| ASPN | ENST00000519375.1 | ENSG00000253980.1 | 0.803478739 | 1.805410623 | 0.07101045 |
| ASPN | ENST00000519660.1 | ENSG00000253416.1 | 0.903943337 | 2.012809838 | 0.044134639 |
| ASPN | ENST00000521378.1 | ENSG00000254222.1 | -0.897254816 | -1.987536371 | 0.04686299 |
| ASPN | ENST00000522190.1 | ENSG00000254165.1 | 0.856911767 | 1.910074538 | 0.056123617 |
| ASPN | ENST00000522426.1 | ENSG00000253538.1 | 0.833563341 | 1.892682482 | 0.058400107 |
| ASPN | ENST00000523935.1 | ENSG00000253567.1 | 0.829617376 | 1.861693411 | 0.062646318 |
| ASPN | ENST00000524073.1 | ENSG00000253774.1 | 0.818432248 | 1.834567187 | 0.066569844 |
| ASPN | ENST00000524133.1 | ENSG00000253174.2 | -0.806054517 | -1.814981113 | 0.069526812 |
| ASPN | ENST00000527100.1 | ENSG00000255015.1 | 0.881177403 | 1.992434508 | 0.046323404 |
| ASPN | ENST00000528869.1 | ENSG00000255443.1 | 0.827954244 | 1.834945744 | 0.066513729 |
| ASPN | ENST00000529875.1 | ENSG00000254404.1 | -0.844501413 | -1.882861824 | 0.059719096 |
| ASPN | ENST00000531071.1 | ENSG00000255248.2 | -0.807392387 | -1.836417808 | 0.066295888 |
| ASPN | ENST00000531627.1 | ENSG00000254584.1 | 0.80138447 | 1.800801576 | 0.07173416 |
| ASPN | ENST00000531661.1 | ENSG00000254473.1 | 0.908236935 | 2.019271076 | 0.043459052 |
| ASPN | ENST00000533938.1 | ENSG00000255142.1 | -0.835097373 | -1.870798192 | 0.061373058 |
| ASPN | ENST00000541391.1 | ENSG00000256268.1 | 0.812742919 | 1.825949968 | 0.067857799 |
| ASPN | ENST00000544089.1 | ENSG00000256273.1 | 0.843756155 | 1.879154286 | 0.06022343 |
| ASPN | ENST00000547175.1 | ENSG00000257395.1 | -0.92997356 | -2.096826 | 0.036008981 |
| ASPN | ENST00000548199.1 | ENSG00000257614.1 | 0.808430857 | 1.795103294 | 0.072637244 |
| ASPN | ENST00000549140.1 | ENSG00000258332.1 | 0.816559849 | 1.850639121 | 0.064221488 |
| ASPN | ENST00000549329.1 | ENSG00000224189.2 | 0.828344198 | 1.860466888 | 0.062819499 |
| ASPN | ENST00000549756.1 | ENSG00000257769.1 | -0.888986034 | -1.976505732 | 0.048097527 |
| ASPN | ENST00000550805.1 | ENSG00000244306.5 | -0.828227708 | -1.830345196 | 0.067198337 |
| ASPN | ENST00000552261.1 | ENSG00000257959.1 | 0.856389919 | 1.928268295 | 0.053821763 |
| ASPN | ENST00000552558.1 | ENSG00000257947.1 | 0.896623616 | 2.016867146 | 0.043709379 |

| | | | | | |
|---|---|---|---|---|---|
| ASPN | ENST00000553477.1 | ENSG00000259123.1 | 0.918200519 | 2.042478908 | 0.04110405 |
| ASPN | ENST00000555966.1 | ENSG00000258843.1 | 0.825751686 | 1.882703732 | 0.059740529 |
| ASPN | ENST00000556458.1 | ENSG00000258504.2 | -0.875579045 | -1.969883391 | 0.048851736 |
| ASPN | ENST00000557903.1 | ENSG00000259182.1 | 0.968929701 | 2.177072069 | 0.029475191 |
| ASPN | ENST00000559569.1 | ENSG00000259760.1 | -0.817767888 | -1.848774194 | 0.064490425 |
| ASPN | ENST00000562582.1 | ENSG00000259779.1 | 0.918968678 | 2.061218858 | 0.039282165 |
| ASPN | ENST00000563342.1 | ENSG00000259914.1 | 0.889624664 | 2.01660044 | 0.043737227 |
| ASPN | ENST00000563570.1 | ENSG00000259961.1 | -0.929358261 | -2.081260245 | 0.037410092 |
| ASPN | ENST00000563601.1 | ENSG00000260589.1 | -0.917356771 | -2.049432332 | 0.040419858 |
| ASPN | ENST00000565271.1 | ENSG00000261335.1 | -0.841867532 | -1.91038985 | 0.056083037 |
| ASPN | ENST00000567089.1 | ENSG00000261822.1 | 0.843577949 | 1.880648264 | 0.060019782 |
| ASPN | ENST00000568410.1 | ENSG00000260277.1 | 0.805529491 | 1.803597105 | 0.07129449 |
| ASPN | ENST00000569849.1 | ENSG00000260640.1 | 0.868915117 | 1.950997527 | 0.05105734 |
| ASPN | ENST00000572417.1 | ENSG00000263171.1 | 0.862239082 | 1.931727936 | 0.053393095 |
| ASPN | ENST00000573861.1 | ENSG00000263320.1 | 0.814956434 | 1.811217833 | 0.070107139 |
| ASPN | ENST00000577360.1 | ENSG00000264273.1 | 0.813381505 | 1.810584334 | 0.070205219 |
| ASPN | ENST00000578035.1 | ENSG00000266743.1 | -0.915413399 | -2.025912784 | 0.042773723 |
| ASPN | ENST00000578349.1 | ENSG00000263688.1 | 0.955576429 | 2.122847563 | 0.033766628 |
| ASPN | ENST00000578572.1 | ENSG00000196295.7 | 0.916955442 | 2.048497177 | 0.040511308 |
| ASPN | ENST00000580975.1 | ENSG00000266237.1 | 0.878990068 | 1.966183854 | 0.049277377 |
| ASPN | ENST00000581996.1 | ENSG00000265778.1 | 0.839637551 | 1.897074228 | 0.057818142 |
| ASPN | ENST00000582348.1 | ENSG00000265148.1 | 0.865758734 | 1.906081506 | 0.056639632 |
| ASPN | ENST00000583841.1 | ENSG00000265148.1 | 0.864294448 | 1.935047275 | 0.052984495 |
| ASPN | ENST00000586010.1 | ENSG00000267606.1 | 0.815664779 | 1.789438458 | 0.07354423 |
| ASPN | ENST00000586297.1 | ENSG00000267633.1 | 0.896714546 | 2.019220225 | 0.043464335 |
| ASPN | ENST00000588177.1 | ENSG00000234899.5 | 0.851508556 | 1.880784368 | 0.060001257 |
| ASPN | ENST00000588835.1 | ENSG00000267476.1 | 0.891097478 | 2.02394816 | 0.042975486 |
| ASPN | ENST00000590357.1 | ENSG00000267175.1 | -0.801053171 | -1.794587595 | 0.072719431 |
| ASPN | ENST00000590364.1 | ENSG00000267613.1 | -0.870557516 | -1.965220769 | 0.049388691 |
| ASPN | ENST00000591103.1 | ENSG00000272895.1 | 0.865020351 | 1.911217343 | 0.055976656 |
| ASPN | ENST00000592431.1 | ENSG00000267475.1 | -0.902715233 | -2.0105528 | 0.044372714 |
| ASPN | ENST00000593486.1 | ENSG00000250910.3 | 0.94711737 | 2.111829045 | 0.034701115 |
| ASPN | ENST00000593599.1 | ENSG00000231898.4 | 0.954121619 | 2.105659432 | 0.035233946 |
| ASPN | ENST00000595892.1 | ENSG00000269640.1 | 0.873591298 | 1.944785693 | 0.051800777 |
| ASPN | ENST00000599050.1 | ENSG00000268366.1 | 0.919818049 | 2.067870179 | 0.038652232 |
| ASPN | ENST00000601511.1 | ENSG00000244513.2 | 0.816700487 | 1.797543468 | 0.072249384 |
| ASPN | ENST00000602485.1 | ENSG00000270163.1 | -0.929072929 | -2.083681857 | 0.037189116 |
| ASPN | ENST00000602790.1 | ENSG00000270000.1 | -0.84824433 | -1.923149951 | 0.054461221 |
| ASPN | ENST00000605692.1 | ENSG00000270810.1 | -0.810023515 | -1.797402982 | 0.072271668 |
| ASPN | ENST00000606898.1 | ENSG00000272094.1 | -0.93115722 | -2.049256865 | 0.040437004 |
| ASPN | ENST00000606938.1 | ENSG00000272198.1 | -0.831377029 | -1.863588134 | 0.062379565 |
| ASPN | ENST00000607222.1 | ENSG00000272106.1 | 0.817788242 | 1.828824175 | 0.067425952 |
| ASPN | ENST00000607715.1 | ENSG00000271788.1 | 0.931347383 | 2.084341891 | 0.037129079 |
| ASPN | ENST00000607876.1 | ENSG00000272848.1 | 0.828102926 | 1.846686197 | 0.064792633 |
| ASPN | ENST00000608173.1 | ENSG00000197099.4 | 0.845692962 | 1.895939937 | 0.057967987 |
| ASPN | ENST00000608289.1 | ENSG00000272958.1 | -0.900911929 | -2.009261998 | 0.044509356 |
| ASPN | ENST00000608476.1 | ENSG00000232675.3 | 0.861569704 | 1.914472194 | 0.055559849 |
| ASPN | ENST00000609610.1 | ENSG00000232675.3 | 0.886695587 | 1.97103067 | 0.048720368 |
| ASPN | ENST00000609890.1 | ENSG00000231898.4 | 0.978544581 | 2.1981677 | 0.027937158 |
| ASPN | ENST00000610185.1 | ENSG00000273355.1 | -0.851758769 | -1.913234678 | 0.055718016 |
| ASPN | NR_022011.1 | PWARSN | 0.991362733 | 2.213426279 | 0.026868271 |

| | | | | | |
|---|---|---|---|---|---|
| ASPN | NR_026822.1 | FAM138C | -0.836428824 | -1.873922789 | 0.060941068 |
| ASPN | NR_033914.1 | LINC00254 | 0.803610752 | 1.806224945 | 0.07088321 |
| ASPN | NR_038194.1 | LINC00583 | 0.939815806 | 2.113906494 | 0.034523254 |
| ASPN | NR_046578.1 | CACNA1C-AS4 | 0.881641913 | 1.976586634 | 0.048088374 |
| ASPN | NR_046766.1 | ATP2B2-IT2 | 0.844383069 | 1.895443087 | 0.058033725 |
| ASPN | NR_047040.1 | LINC00424 | 0.819255998 | 1.792772753 | 0.073009268 |
| ASPN | NR_104618.1 | LINC01017 | 0.881170125 | 1.965066431 | 0.049406549 |
| ASPN | NR_104620.1 | LINC01672 | 0.991574588 | 2.236723644 | 0.025304408 |
| ASPN | NR_104998.1 | LOC102467225 | 0.94119154 | 2.11766281 | 0.034203635 |
| ASPN | NR_109975.1 | ARNTL2-AS1 | -0.820692737 | -1.845993528 | 0.064893145 |
| ASPN | NR_110370.1 | STAM-AS1 | -0.806620401 | -1.795062938 | 0.072643673 |
| ASPN | NR_110504.1 | LOC101929572 | -0.802254994 | -1.78355126 | 0.074496612 |
| ASPN | NR_120330.1 | LOC101928227 | -0.909795767 | -2.033944651 | 0.041957176 |
| ASPN | NR_120527.1 | LOC100506675 | 0.887593383 | 1.986155069 | 0.047016108 |
| ASPN | NR_126041.1 | LOC101930071 | -0.881608584 | -1.969777538 | 0.048863872 |
| ASPN | NR_130916.1 | LOC105274304 | -0.829639163 | -1.853455334 | 0.063817122 |
| ASPN | NR_131186.1 | LOC105377348 | 0.858313544 | 1.913278875 | 0.055712361 |
| ASPN | NR_134632.1 | LOC105373051 | -0.817852741 | -1.840102166 | 0.065753239 |
| ASPN | NR_135274.1 | LOC105370619 | 0.834690011 | 1.859906046 | 0.06289882 |
| ASPN | NR_135549.1 | LOC101929411 | 0.895075865 | 2.009740372 | 0.044458675 |
| ASPN | NR_135840.1 | LOC105376114 | 0.969645103 | 2.161748395 | 0.030637575 |
| ASPN | NR_136218.1 | MEF2C-AS1 | 0.841729 | 1.868612053 | 0.061676808 |
| ATP5D | ENST00000318291.4 | ENSG00000177406.4 | 0.836178718 | 1.83412179 | 0.066635918 |
| ATP5D | ENST00000412085.1 | ENSG00000233825.1 | 0.801847095 | 1.792786169 | 0.073007122 |
| ATP5D | ENST00000412759.1 | ENSG00000236933.1 | 0.852505864 | 1.914628621 | 0.055539882 |
| ATP5D | ENST00000412772.1 | ENSG00000231507.1 | 0.858191724 | 1.9250968 | 0.054217249 |
| ATP5D | ENST00000414740.2 | ENSG00000229646.2 | 0.821531757 | 1.833342002 | 0.066751727 |
| ATP5D | ENST00000429608.1 | ENSG00000237480.1 | 0.833444243 | 1.823400755 | 0.068242716 |
| ATP5D | ENST00000430920.1 | ENSG00000234203.1 | 0.820441053 | 1.825202332 | 0.067970502 |
| ATP5D | ENST00000433876.2 | ENSG00000228423.2 | 0.801224831 | 1.774772392 | 0.075935477 |
| ATP5D | ENST00000434627.1 | ENSG00000230074.1 | 0.813932069 | 1.82572499 | 0.067891697 |
| ATP5D | ENST00000447206.1 | ENSG00000230839.1 | 0.805713215 | 1.787934359 | 0.073786599 |
| ATP5D | ENST00000450531.1 | ENSG00000229536.1 | 0.849558312 | 1.907208262 | 0.056493624 |
| ATP5D | ENST00000451034.1 | ENSG00000229805.1 | -0.86562222 | -1.922579497 | 0.054532881 |
| ATP5D | ENST00000453051.1 | ENSG00000229407.1 | 0.849589199 | 1.902402499 | 0.057118555 |
| ATP5D | ENST00000454530.1 | ENSG00000226649.1 | -0.822949192 | -1.828368327 | 0.067494292 |
| ATP5D | ENST00000457115.1 | ENSG00000227245.1 | 0.84132448 | 1.867898823 | 0.061776175 |
| ATP5D | ENST00000458194.1 | ENSG00000226193.1 | 0.852952197 | 1.906779996 | 0.056549083 |
| ATP5D | ENST00000459985.1 | ENSG00000273066.1 | 0.914539864 | 2.051843168 | 0.040184907 |
| ATP5D | ENST00000466431.2 | ENSG00000254485.1 | 0.801136901 | 1.805879207 | 0.07093721 |
| ATP5D | ENST00000468165.1 | ENSG00000239480.1 | 0.846514305 | 1.884367596 | 0.05951527 |
| ATP5D | ENST00000490013.1 | ENSG00000184115.12 | 0.863971379 | 1.905502784 | 0.056714746 |
| ATP5D | ENST00000498358.1 | ENSG00000184115.12 | 0.850079893 | 1.898725345 | 0.057600597 |
| ATP5D | ENST00000498693.1 | ENSG00000244198.1 | 0.810175353 | 1.777105165 | 0.075550939 |
| ATP5D | ENST00000504301.1 | ENSG00000250696.1 | -0.803292583 | -1.785054867 | 0.074252418 |
| ATP5D | ENST00000519852.1 | ENSG00000253716.1 | 0.83250856 | 1.849785051 | 0.064344536 |
| ATP5D | ENST00000521207.1 | ENSG00000253716.1 | 0.889370651 | 1.96605634 | 0.049292103 |
| ATP5D | ENST00000522600.1 | ENSG00000246582.2 | 0.964947004 | 2.143622898 | 0.032063115 |
| ATP5D | ENST00000524335.1 | ENSG00000253716.1 | 0.815690594 | 1.830659173 | 0.06715143 |
| ATP5D | ENST00000526611.1 | ENSG00000246982.2 | 0.875868342 | 1.978205437 | 0.047905536 |
| ATP5D | ENST00000527086.1 | ENSG00000255182.1 | 0.883975207 | 1.972308991 | 0.048574344 |

| | | | | | |
|---|---|---|---|---|---|
| ATP5D | ENST00000528000.1 | ENSG00000254804.1 | 0.856700355 | 1.924166282 | 0.054333744 |
| ATP5D | ENST00000528887.1 | ENSG00000254501.1 | 0.836213176 | 1.866199295 | 0.062013488 |
| ATP5D | ENST00000543072.1 | ENSG00000256092.2 | -0.900867021 | -2.015810449 | 0.0438198 |
| ATP5D | ENST00000543275.1 | ENSG00000256944.1 | 0.943174013 | 2.08588671 | 0.036988886 |
| ATP5D | ENST00000543494.1 | ENSG00000256514.1 | 0.961261475 | 2.163986723 | 0.030465368 |
| ATP5D | ENST00000548731.1 | ENSG00000257809.1 | 0.853778155 | 1.939827057 | 0.052400711 |
| ATP5D | ENST00000549878.1 | ENSG00000257284.1 | 0.837051945 | 1.8553655 | 0.063544051 |
| ATP5D | ENST00000551135.1 | ENSG00000258294.1 | -0.82928936 | -1.848845688 | 0.064480098 |
| ATP5D | ENST00000554049.1 | ENSG00000258763.1 | 0.822175818 | 1.84468421 | 0.065083489 |
| ATP5D | ENST00000558575.1 | ENSG00000259687.1 | 0.942147827 | 2.096488293 | 0.036038897 |
| ATP5D | ENST00000563018.1 | ENSG00000260193.1 | 0.829604952 | 1.869933906 | 0.061492997 |
| ATP5D | ENST00000563611.1 | ENSG00000261583.1 | 0.804198365 | 1.78749482 | 0.073857549 |
| ATP5D | ENST00000563806.1 | ENSG00000238045.5 | 0.81211531 | 1.817765304 | 0.069100012 |
| ATP5D | ENST00000565359.1 | ENSG00000260601.1 | 0.85898545 | 1.944059799 | 0.05188824 |
| ATP5D | ENST00000565667.1 | ENSG00000261253.1 | 0.846283035 | 1.898163737 | 0.057674516 |
| ATP5D | ENST00000565798.2 | ENSG00000259786.2 | -0.893619471 | -2.01212879 | 0.044206362 |
| ATP5D | ENST00000565823.1 | ENSG00000260686.1 | -0.906229747 | -2.035858704 | 0.041764546 |
| ATP5D | ENST00000565829.1 | ENSG00000260148.1 | 0.904483791 | 2.016470056 | 0.043750846 |
| ATP5D | ENST00000568659.1 | ENSG00000260004.1 | -0.897962271 | -2.018499248 | 0.043539292 |
| ATP5D | ENST00000570843.1 | ENSG00000261889.1 | 0.865649577 | 1.917517442 | 0.055172227 |
| ATP5D | ENST00000570929.1 | ENSG00000262223.2 | 0.868920839 | 1.958657603 | 0.050152899 |
| ATP5D | ENST00000570974.1 | ENSG00000263300.1 | 0.866726605 | 1.933849689 | 0.053131611 |
| ATP5D | ENST00000576086.1 | ENSG00000262823.1 | 0.944395654 | 2.122221372 | 0.033819153 |
| ATP5D | ENST00000577064.1 | ENSG00000262823.1 | 0.902869035 | 2.03092001 | 0.04226311 |
| ATP5D | ENST00000577176.1 | ENSG00000262823.1 | 0.893050774 | 2.014368967 | 0.04397081 |
| ATP5D | ENST00000578936.1 | ENSG00000265547.1 | 0.840941492 | 1.894704464 | 0.058131566 |
| ATP5D | ENST00000583916.1 | ENSG00000264196.1 | 0.868857559 | 1.919497687 | 0.054921379 |
| ATP5D | ENST00000588908.1 | ENSG00000267751.1 | 0.801380109 | 1.801566023 | 0.071613711 |
| ATP5D | ENST00000589673.1 | ENSG00000267755.1 | 0.924761218 | 2.074068073 | 0.038072989 |
| ATP5D | ENST00000590328.1 | ENSG00000256995.2 | -0.911989924 | -2.040212162 | 0.041329199 |
| ATP5D | ENST00000591174.1 | ENSG00000267289.1 | 0.899970271 | 2.011571664 | 0.044265109 |
| ATP5D | ENST00000592413.1 | ENSG00000266933.1 | 0.824716037 | 1.882100727 | 0.059822341 |
| ATP5D | ENST00000592518.1 | ENSG00000267786.1 | 0.805955761 | 1.802592655 | 0.071452211 |
| ATP5D | ENST00000593642.1 | ENSG00000267858.1 | 0.856921739 | 1.924503488 | 0.054291504 |
| ATP5D | ENST00000594590.2 | ENSG00000268199.2 | 0.893215108 | 2.005927093 | 0.044864027 |
| ATP5D | ENST00000595955.1 | ENSG00000268401.1 | 0.844457774 | 1.871644348 | 0.061255824 |
| ATP5D | ENST00000596887.1 | ENSG00000237031.3 | -0.819488752 | -1.815361746 | 0.069468336 |
| ATP5D | ENST00000597309.1 | ENSG00000232098.2 | -0.834409928 | -1.904516244 | 0.056842983 |
| ATP5D | ENST00000598092.1 | ENSG00000228065.6 | -0.834837292 | -1.880782632 | 0.060001494 |
| ATP5D | ENST00000600007.1 | ENSG00000268655.1 | 0.865245925 | 1.941702385 | 0.05217314 |
| ATP5D | ENST00000600071.1 | ENSG00000269199.1 | 0.801139594 | 1.784493717 | 0.074343475 |
| ATP5D | ENST00000600534.1 | ENSG00000267858.1 | 0.891727098 | 1.971373906 | 0.048681123 |
| ATP5D | ENST00000600716.1 | ENSG00000269487.1 | 0.831542091 | 1.847371152 | 0.064693367 |
| ATP5D | ENST00000600726.1 | ENSG00000267858.1 | 0.876398925 | 1.975279314 | 0.048236459 |
| ATP5D | ENST00000602532.1 | ENSG00000270091.1 | 0.871163981 | 1.939124473 | 0.052486183 |
| ATP5D | ENST00000606068.1 | ENSG00000272342.1 | 0.835636778 | 1.887514631 | 0.05909114 |
| ATP5D | ENST00000607014.1 | ENSG00000272345.1 | -0.836045036 | -1.885466942 | 0.059366823 |
| ATP5D | ENST00000607119.1 | ENSG00000272541.1 | -0.874440212 | -1.940728023 | 0.052291275 |
| ATP5D | ENST00000608940.1 | ENSG00000272763.1 | 0.840258007 | 1.89798831 | 0.057697622 |
| ATP5D | ENST00000608952.1 | ENSG00000272689.1 | -0.850579385 | -1.8830336 | 0.059695815 |
| ATP5D | ENST00000609113.1 | ENSG00000272827.1 | 0.955427153 | 2.17424379 | 0.029686831 |

| | | | | | |
|---|---|---|---|---|---|
| ATP5D | ENST00000609146.1 | ENSG00000272851.1 | -0.818561242 | -1.828696536 | 0.067445082 |
| ATP5D | ENST00000609976.1 | ENSG00000272582.1 | 0.915769741 | 2.056124332 | 0.039770533 |
| ATP5D | ENST00000610145.1 | ENSG00000273175.1 | 0.801583311 | 1.79193368 | 0.07314359 |
| ATP5D | ENST00000610270.1 | ENSG00000272576.1 | -0.911845823 | -2.023484383 | 0.043023232 |
| ATP5D | NR_003604.2 | ZFAS1 | 0.851168565 | 1.899172123 | 0.057541849 |
| ATP5D | NR_003606.2 | ZFAS1 | 0.83089952 | 1.841716665 | 0.065516605 |
| ATP5D | NR_027052.1 | THAP7-AS1 | 0.837528643 | 1.90209276 | 0.057159029 |
| ATP5D | NR_027271.1 | CIRBP-AS1 | 0.829321101 | 1.859126015 | 0.063009279 |
| ATP5D | NR_027334.2 | MZF1-AS1 | 0.880549736 | 1.955446028 | 0.050530448 |
| ATP5D | NR_036480.1 | VPS9D1-AS1 | 0.807580119 | 1.801375252 | 0.071643754 |
| ATP5D | NR_036658.1 | ZFAS1 | 0.826033135 | 1.841430407 | 0.06555851 |
| ATP5D | NR_038421.1 | LINC01220 | 0.860434467 | 1.925220642 | 0.054201761 |
| ATP5D | NR_040096.1 | LOC643339 | 0.811005934 | 1.859721048 | 0.062925002 |
| ATP5D | NR_046571.1 | POTEH-AS1 | 0.899037284 | 2.016540137 | 0.043743526 |
| ATP5D | NR_073552.1 | LOC101059948 | 0.865245925 | 1.928856225 | 0.053748714 |
| ATP5D | NR_104158.1 | NRG1-IT1 | 0.908303487 | 2.039284131 | 0.041421679 |
| ATP5D | NR_108036.1 | CFAP58-AS1 | 0.803431823 | 1.78375731 | 0.074463109 |
| ATP5D | NR_110919.1 | LOC101928530 | 0.83852812 | 1.87075919 | 0.061378467 |
| ATP5D | NR_111951.1 | LINC00869 | 0.860565777 | 1.931886892 | 0.053373468 |
| ATP5D | NR_111952.1 | LINC00869 | 0.881378057 | 1.966642036 | 0.049224494 |
| ATP5D | NR_111953.1 | LINC00869 | 0.856271425 | 1.886849384 | 0.059180587 |
| ATP5D | NR_117097.1 | LINC01353 | 0.858191724 | 1.918031859 | 0.055106972 |
| ATP5D | NR_117098.1 | LINC01353 | 0.894501149 | 2.040469813 | 0.041303555 |
| ATP5D | NR_121188.1 | PGM5P3-AS1 | -0.821571984 | -1.830781686 | 0.067133135 |
| ATP5D | NR_130143.1 | LOC104968399 | 0.801916132 | 1.779232912 | 0.075201585 |
| ATP5D | NR_135041.1 | LOC101927038 | 0.810848122 | 1.82046134 | 0.068688779 |
| ATP5D | NR_135644.1 | LOC105371506 | -0.88254228 | -1.950058485 | 0.051169149 |
| ATP5J2 | ENST00000411824.1 | ENSG00000232803.1 | 0.825080871 | 1.846244243 | 0.06485675 |
| ATP5J2 | ENST00000412812.1 | ENSG00000225342.1 | -0.82086832 | -1.829134363 | 0.067379482 |
| ATP5J2 | ENST00000414992.1 | ENSG00000233613.1 | 0.804048789 | 1.813421917 | 0.069766772 |
| ATP5J2 | ENST00000416861.1 | ENSG00000227308.2 | 0.843890328 | 1.874791051 | 0.060821475 |
| ATP5J2 | ENST00000422118.1 | ENSG00000231189.1 | 0.837360349 | 1.862013239 | 0.062601224 |
| ATP5J2 | ENST00000422204.1 | ENSG00000238160.1 | 0.894556613 | 1.993712832 | 0.046183446 |
| ATP5J2 | ENST00000423925.1 | ENSG00000223536.1 | 0.816568784 | 1.805743692 | 0.070958384 |
| ATP5J2 | ENST00000425364.1 | ENSG00000231046.1 | 0.889247724 | 2.00116568 | 0.045374538 |
| ATP5J2 | ENST00000426929.1 | ENSG00000230184.1 | 0.849052401 | 1.89228945 | 0.058452426 |
| ATP5J2 | ENST00000427132.1 | ENSG00000232121.1 | 0.827159061 | 1.839600308 | 0.065826939 |
| ATP5J2 | ENST00000429878.1 | ENSG00000224184.1 | 0.823070866 | 1.817710814 | 0.069108344 |
| ATP5J2 | ENST00000430545.1 | ENSG00000237153.1 | -0.84099233 | -1.895095865 | 0.058079702 |
| ATP5J2 | ENST00000431290.1 | ENSG00000183822.2 | -0.827496892 | -1.859457142 | 0.062962369 |
| ATP5J2 | ENST00000432244.1 | ENSG00000234265.1 | -0.909304185 | -2.053048502 | 0.040067874 |
| ATP5J2 | ENST00000432559.2 | ENSG00000228229.2 | -0.871659687 | -1.952781575 | 0.050845483 |
| ATP5J2 | ENST00000433035.1 | ENSG00000230483.1 | 0.864372684 | 1.91115441 | 0.055984741 |
| ATP5J2 | ENST00000435357.1 | ENSG00000225444.1 | -0.882790901 | -1.994507023 | 0.046096673 |
| ATP5J2 | ENST00000435984.1 | ENSG00000204792.2 | -0.850051264 | -1.926442254 | 0.054049176 |
| ATP5J2 | ENST00000437334.1 | ENSG00000226134.1 | 0.933952928 | 2.064984498 | 0.038924467 |
| ATP5J2 | ENST00000439443.1 | ENSG00000236911.2 | 0.914024047 | 2.017070071 | 0.043688201 |
| ATP5J2 | ENST00000440518.1 | ENSG00000226571.1 | 0.828637316 | 1.858896962 | 0.063041745 |
| ATP5J2 | ENST00000441532.1 | ENSG00000234206.1 | 0.827855417 | 1.863754862 | 0.062356137 |
| ATP5J2 | ENST00000444731.1 | ENSG00000227131.1 | 0.875486388 | 1.971552552 | 0.048660708 |
| ATP5J2 | ENST00000445260.2 | ENSG00000231429.2 | -0.924078159 | -2.065190489 | 0.03890498 |

| | | | | | |
|---|---|---|---|---|---|
| ATP5J2 | ENST00000445631.1 | ENSG00000231052.1 | 0.903846243 | 2.041052582 | 0.041245601 |
| ATP5J2 | ENST00000448650.1 | ENSG00000223536.1 | 0.835517895 | 1.857922599 | 0.063180007 |
| ATP5J2 | ENST00000448942.1 | ENSG00000237499.2 | -0.858677868 | -1.942663452 | 0.052056835 |
| ATP5J2 | ENST00000449586.1 | ENSG00000235257.4 | 0.866282444 | 1.93480624 | 0.053014077 |
| ATP5J2 | ENST00000451648.1 | ENSG00000232803.1 | 0.864050145 | 1.910843816 | 0.056024655 |
| ATP5J2 | ENST00000452002.1 | ENSG00000236501.1 | 0.802134737 | 1.791149322 | 0.073269336 |
| ATP5J2 | ENST00000454182.1 | ENSG00000230379.1 | -0.940533746 | -2.085573569 | 0.037017268 |
| ATP5J2 | ENST00000454387.1 | ENSG00000223726.1 | 0.888122815 | 2.019457778 | 0.043439661 |
| ATP5J2 | ENST00000454709.1 | ENSG00000237280.1 | 0.814044973 | 1.805914738 | 0.070931659 |
| ATP5J2 | ENST00000454957.1 | ENSG00000224899.1 | -0.859166033 | -1.939630308 | 0.052424635 |
| ATP5J2 | ENST00000457602.1 | ENSG00000237576.1 | 0.889785221 | 2.001886661 | 0.045296923 |
| ATP5J2 | ENST00000458082.1 | ENSG00000231210.2 | 0.886229797 | 1.989297178 | 0.046668411 |
| ATP5J2 | ENST00000458107.3 | ENSG00000248478.2 | -0.94743458 | -2.111891467 | 0.034695759 |
| ATP5J2 | ENST00000460993.1 | ENSG00000241231.1 | -0.819868341 | -1.837593762 | 0.066122289 |
| ATP5J2 | ENST00000477643.1 | ENSG00000241224.2 | 0.81084502 | 1.790669627 | 0.073346326 |
| ATP5J2 | ENST00000479233.1 | ENSG00000243150.1 | -0.939201005 | -2.108554352 | 0.034983067 |
| ATP5J2 | ENST00000480919.1 | ENSG00000242474.1 | 0.806100635 | 1.823386221 | 0.068244916 |
| ATP5J2 | ENST00000485347.1 | ENSG00000239991.1 | 0.820411423 | 1.848880657 | 0.064475047 |
| ATP5J2 | ENST00000487368.1 | ENSG00000273328.1 | 0.95085266 | 2.132851983 | 0.03293687 |
| ATP5J2 | ENST00000505844.1 | ENSG00000248455.1 | -0.898124684 | -2.010253295 | 0.044404387 |
| ATP5J2 | ENST00000507525.1 | ENSG00000250431.1 | -0.852708762 | -1.888153766 | 0.059005311 |
| ATP5J2 | ENST00000507558.1 | ENSG00000248445.1 | -0.803642179 | -1.794683495 | 0.072704142 |
| ATP5J2 | ENST00000507808.1 | ENSG00000250333.1 | 0.973723666 | 2.148554153 | 0.031669759 |
| ATP5J2 | ENST00000509983.1 | ENSG00000248173.1 | 0.835075942 | 1.865326233 | 0.06213569 |
| ATP5J2 | ENST00000510602.1 | ENSG00000249122.1 | 0.89124785 | 2.017028665 | 0.043692522 |
| ATP5J2 | ENST00000511234.1 | ENSG00000250865.1 | 0.991755829 | 2.195045771 | 0.028160315 |
| ATP5J2 | ENST00000513836.1 | ENSG00000251266.1 | -0.84513633 | -1.875185474 | 0.060767212 |
| ATP5J2 | ENST00000514459.1 | ENSG00000248211.1 | 0.812809898 | 1.829188226 | 0.067371416 |
| ATP5J2 | ENST00000518894.1 | ENSG00000204758.3 | -0.815522628 | -1.810807445 | 0.070170664 |
| ATP5J2 | ENST00000519038.2 | ENSG00000254054.2 | 0.806338142 | 1.805741059 | 0.070958796 |
| ATP5J2 | ENST00000520411.1 | ENSG00000253355.1 | 0.89034546 | 1.968461723 | 0.049014936 |
| ATP5J2 | ENST00000521030.1 | ENSG00000253802.1 | 0.878495819 | 1.955626469 | 0.050509172 |
| ATP5J2 | ENST00000521359.1 | ENSG00000253140.1 | -0.823418652 | -1.847576604 | 0.064663617 |
| ATP5J2 | ENST00000523703.1 | ENSG00000214803.3 | 0.987775765 | 2.220292454 | 0.026398922 |
| ATP5J2 | ENST00000523859.1 | ENSG00000251136.4 | 0.913605992 | 2.048138721 | 0.040546409 |
| ATP5J2 | ENST00000524286.1 | ENSG00000253658.1 | -0.810247758 | -1.794650951 | 0.07270933 |
| ATP5J2 | ENST00000524309.1 | ENSG00000240915.2 | 0.835313617 | 1.86820661 | 0.061733278 |
| ATP5J2 | ENST00000525855.1 | ENSG00000254746.1 | 0.808263732 | 1.826132115 | 0.067830364 |
| ATP5J2 | ENST00000528869.1 | ENSG00000255443.1 | -0.84534961 | -1.874337598 | 0.060883908 |
| ATP5J2 | ENST00000531071.1 | ENSG00000255248.2 | 0.868195314 | 1.928102985 | 0.053842318 |
| ATP5J2 | ENST00000531136.1 | ENSG00000255558.1 | 0.805679301 | 1.794716539 | 0.072698874 |
| ATP5J2 | ENST00000532454.1 | ENSG00000255120.1 | 0.872072711 | 1.957643385 | 0.050271873 |
| ATP5J2 | ENST00000533938.1 | ENSG00000255142.1 | 0.807369747 | 1.786106528 | 0.074082011 |
| ATP5J2 | ENST00000538294.1 | ENSG00000250748.2 | 0.830312157 | 1.864846014 | 0.062202991 |
| ATP5J2 | ENST00000546789.1 | ENSG00000257740.1 | -0.803579633 | -1.816057547 | 0.069361545 |
| ATP5J2 | ENST00000548748.1 | ENSG00000258252.1 | 0.816593225 | 1.837024709 | 0.066206248 |
| ATP5J2 | ENST00000552378.1 | ENSG00000257294.1 | 0.868545726 | 1.946249065 | 0.051624829 |
| ATP5J2 | ENST00000553668.1 | ENSG00000258733.1 | -0.90407459 | -2.007366114 | 0.044710694 |
| ATP5J2 | ENST00000557855.1 | ENSG00000259176.1 | -0.804804138 | -1.790540003 | 0.073367142 |
| ATP5J2 | ENST00000561847.1 | ENSG00000260293.1 | 0.892946551 | 1.989748533 | 0.046618643 |
| ATP5J2 | ENST00000565623.1 | ENSG00000261118.1 | 0.843818901 | 1.893716842 | 0.058262605 |

| | | | | | |
|---|---|---|---|---|---|
| ATP5J2 | ENST00000566639.1 | ENSG00000261061.1 | 0.902596441 | 2.031249839 | 0.042229657 |
| ATP5J2 | ENST00000569215.1 | ENSG00000260756.1 | -0.899655006 | -2.012135317 | 0.044205675 |
| ATP5J2 | ENST00000572471.1 | ENSG00000262721.1 | 0.814590067 | 1.824332998 | 0.068101745 |
| ATP5J2 | ENST00000572608.1 | ENSG00000263305.1 | -0.927945506 | -2.05079116 | 0.040287289 |
| ATP5J2 | ENST00000573414.1 | ENSG00000263072.1 | -0.914624418 | -2.038984289 | 0.041451596 |
| ATP5J2 | ENST00000586338.1 | ENSG00000219410.4 | 0.938989858 | 2.086669343 | 0.036918034 |
| ATP5J2 | ENST00000587049.1 | ENSG00000235535.3 | 0.844278197 | 1.898563897 | 0.057621839 |
| ATP5J2 | ENST00000587696.1 | ENSG00000225313.2 | 0.814584625 | 1.840818437 | 0.06564817 |
| ATP5J2 | ENST00000587850.1 | ENSG00000267683.1 | 0.96232979 | 2.160888338 | 0.030703966 |
| ATP5J2 | ENST00000589281.1 | ENSG00000267707.1 | 0.884541507 | 1.988291879 | 0.046779418 |
| ATP5J2 | ENST00000590989.1 | ENSG00000267011.1 | 0.908049464 | 2.042008842 | 0.041150654 |
| ATP5J2 | ENST00000591414.1 | ENSG00000267011.1 | 0.914811053 | 2.041217375 | 0.041229225 |
| ATP5J2 | ENST00000592368.1 | ENSG00000267231.1 | 0.867378013 | 1.930623131 | 0.053529675 |
| ATP5J2 | ENST00000593967.1 | ENSG00000232732.5 | 0.888961521 | 1.989339036 | 0.046663794 |
| ATP5J2 | ENST00000597420.1 | ENSG00000269564.1 | 0.910188137 | 2.022525281 | 0.043122115 |
| ATP5J2 | ENST00000599387.1 | ENSG00000227733.4 | -0.881520746 | -1.949442473 | 0.051242606 |
| ATP5J2 | ENST00000599572.1 | ENSG00000233783.3 | 0.859991639 | 1.897875957 | 0.057712424 |
| ATP5J2 | ENST00000600848.1 | ENSG00000228065.6 | -0.940267349 | -2.105701536 | 0.035230286 |
| ATP5J2 | ENST00000602418.1 | ENSG00000232295.3 | 0.839064318 | 1.865778719 | 0.062072331 |
| ATP5J2 | ENST00000603948.1 | ENSG00000222041.6 | 0.823801786 | 1.804383663 | 0.071171182 |
| ATP5J2 | ENST00000606921.1 | ENSG00000272402.1 | 0.940112967 | 2.082141412 | 0.037329555 |
| ATP5J2 | ENST00000606942.1 | ENSG00000271835.1 | -0.836933594 | -1.884948435 | 0.0594368 |
| ATP5J2 | ENST00000607740.1 | ENSG00000271916.1 | -0.891949277 | -1.971692856 | 0.04864468 |
| ATP5J2 | ENST00000609182.1 | ENSG00000273248.1 | 0.81150174 | 1.796904321 | 0.07235081 |
| ATP5J2 | ENST00000609789.1 | ENSG00000272707.1 | 0.890505474 | 2.012996052 | 0.044115045 |
| ATP5J2 | ENST00000610008.1 | ENSG00000272711.1 | 0.928562605 | 2.076005207 | 0.037893469 |
| ATP5J2 | NR_031762.2 | HCP5B | 0.840717318 | 1.924704247 | 0.054266369 |
| ATP5J2 | NR_034131.1 | LINC00272 | 0.833074696 | 1.871493375 | 0.061276727 |
| ATP5J2 | NR_040047.1 | SDCBP2-AS1 | 0.86468607 | 1.937968995 | 0.052627005 |
| ATP5J2 | NR_108085.1 | OVOL1-AS1 | 0.895061472 | 2.025635729 | 0.042802127 |
| ATP5J2 | NR_109870.1 | LINC01723 | -0.892274565 | -2.004112796 | 0.045057979 |
| ATP5J2 | NR_109985.1 | LOC101927830 | 0.827157177 | 1.844394558 | 0.06512566 |
| ATP5J2 | NR_110504.1 | LOC101929572 | 0.811250677 | 1.795171225 | 0.072626423 |
| ATP5J2 | NR_110930.1 | LOC101927814 | 0.938141986 | 2.105672736 | 0.03523279 |
| ATP5J2 | NR_125407.1 | LOC102724604 | -0.814568116 | -1.82245239 | 0.068386371 |
| ATP5J2 | NR_125420.1 | LOC101927588 | 0.99077454 | 2.206396205 | 0.02735627 |
| ATP5J2 | NR_125925.1 | LOC101929448 | -0.836603504 | -1.8976196 | 0.057746211 |
| ATP5J2 | NR_126041.1 | LOC101930071 | 0.812772058 | 1.825796392 | 0.067880937 |
| ATP5J2 | NR_126412.1 | SCEL-AS1 | 0.956960708 | 2.115396944 | 0.03439613 |
| ATP5J2 | NR_126413.1 | SCEL-AS1 | 0.939648661 | 2.122762225 | 0.033773782 |
| ATP5J2 | NR_133658.1 | HTR3E-AS1 | 0.873312835 | 1.942045697 | 0.052131568 |
| ATP5J2 | NR_133941.1 | LOC105377247 | -0.84513633 | -1.889382781 | 0.058840556 |
| ATP5J2 | NR_133942.1 | LOC105377247 | -0.964341895 | -2.154161749 | 0.031227489 |
| ATP5J2 | NR_134273.1 | LOC101929544 | -0.885893302 | -1.963474313 | 0.049591086 |
| ATP5J2 | NR_135239.1 | LINC01867 | -0.868907435 | -1.939046349 | 0.052495695 |
| ATP5J2 | NR_135816.1 | LOC100996664 | 0.957389006 | 2.160311063 | 0.030748597 |
| AURKB | ENST00000400768.2 | ENSG00000215692.2 | 0.86190505 | 1.90106668 | 0.057293279 |
| AURKB | ENST00000411489.1 | ENSG00000227112.1 | -0.924453575 | -2.041868984 | 0.041164529 |
| AURKB | ENST00000411694.1 | ENSG00000225331.1 | 0.850763993 | 1.898138943 | 0.057677781 |
| AURKB | ENST00000420766.1 | ENSG00000228679.1 | 0.89445972 | 2.002368705 | 0.045245092 |
| AURKB | ENST00000432314.1 | ENSG00000231532.1 | 0.82373393 | 1.84475505 | 0.065073179 |

| | | | | | |
|---|---|---|---|---|---|
| AURKB | ENST00000434790.1 | ENSG00000240040.1 | -0.810304881 | -1.800098416 | 0.0718451 |
| AURKB | ENST00000437095.1 | ENSG00000237087.1 | 0.875418321 | 1.947442154 | 0.051481748 |
| AURKB | ENST00000452176.1 | ENSG00000223659.1 | -0.825134709 | -1.834018345 | 0.066651271 |
| AURKB | ENST00000454957.1 | ENSG00000224899.1 | -0.812704229 | -1.800850312 | 0.071726476 |
| AURKB | ENST00000455788.1 | ENSG00000236263.1 | 0.892236398 | 1.992868369 | 0.046275863 |
| AURKB | ENST00000457371.1 | ENSG00000237401.2 | 0.885063721 | 1.986659076 | 0.04696019 |
| AURKB | ENST00000494509.1 | ENSG00000240095.1 | 0.887902352 | 1.988609911 | 0.046744277 |
| AURKB | ENST00000500267.2 | ENSG00000246323.2 | 0.833099529 | 1.866448116 | 0.061978697 |
| AURKB | ENST00000500496.2 | ENSG00000245479.2 | 0.859902447 | 1.938926896 | 0.05251024 |
| AURKB | ENST00000503571.1 | ENSG00000249592.1 | 0.905101584 | 2.043464174 | 0.04100651 |
| AURKB | ENST00000513836.1 | ENSG00000251266.1 | -0.839854895 | -1.850670105 | 0.064217027 |
| AURKB | ENST00000519506.1 | ENSG00000253103.1 | 0.962113587 | 2.174010429 | 0.029704352 |
| AURKB | ENST00000527789.1 | ENSG00000255173.1 | 0.912811183 | 2.058674719 | 0.03952541 |
| AURKB | ENST00000532454.1 | ENSG00000255120.1 | 0.821122509 | 1.8488833 | 0.064474666 |
| AURKB | ENST00000535720.1 | ENSG00000256364.1 | 0.846827335 | 1.895718365 | 0.057997296 |
| AURKB | ENST00000548722.2 | ENSG00000257194.2 | -0.85656837 | -1.91405231 | 0.055613472 |
| AURKB | ENST00000554451.1 | ENSG00000258683.1 | 0.908034152 | 2.022865531 | 0.043087013 |
| AURKB | ENST00000558475.1 | ENSG00000259604.1 | 0.890970025 | 1.956293037 | 0.050430644 |
| AURKB | ENST00000558568.1 | ENSG00000272639.1 | 0.885173373 | 1.962954058 | 0.049651513 |
| AURKB | ENST00000559673.1 | ENSG00000259604.1 | 0.896644251 | 2.009391721 | 0.044495608 |
| AURKB | ENST00000559960.1 | ENSG00000259354.1 | 0.875789783 | 1.94626347 | 0.051623099 |
| AURKB | ENST00000562970.1 | ENSG00000260145.1 | 0.801935495 | 1.79502684 | 0.072649424 |
| AURKB | ENST00000567127.1 | ENSG00000260264.1 | -0.802352938 | -1.789349419 | 0.07355856 |
| AURKB | ENST00000567395.1 | ENSG00000261090.1 | 0.845133038 | 1.879754075 | 0.060141602 |
| AURKB | ENST00000569981.1 | ENSG00000238045.5 | 0.820178546 | 1.830713417 | 0.067143329 |
| AURKB | ENST00000574365.1 | ENSG00000262837.1 | 0.846068228 | 1.893574995 | 0.058281445 |
| AURKB | ENST00000577807.1 | ENSG00000263427.1 | 0.921121168 | 2.031162942 | 0.042238468 |
| AURKB | ENST00000580311.1 | ENSG00000266803.1 | -0.815566143 | -1.835821557 | 0.066384052 |
| AURKB | ENST00000581798.1 | ENSG00000266288.1 | -0.824094384 | -1.848110829 | 0.064586312 |
| AURKB | ENST00000582386.1 | ENSG00000265174.1 | 0.800248843 | 1.808603041 | 0.070512696 |
| AURKB | ENST00000584758.1 | ENSG00000265356.1 | 0.818435896 | 1.823249118 | 0.068265669 |
| AURKB | ENST00000586694.1 | ENSG00000267141.1 | 0.838611773 | 1.881895938 | 0.059850146 |
| AURKB | ENST00000587696.1 | ENSG00000225313.2 | 0.869866273 | 1.950682335 | 0.051094846 |
| AURKB | ENST00000588380.1 | ENSG00000266990.1 | 0.808400386 | 1.803774188 | 0.071266714 |
| AURKB | ENST00000599467.1 | ENSG00000244513.2 | 0.924074795 | 2.056189471 | 0.039764257 |
| AURKB | ENST00000602418.1 | ENSG00000232295.3 | 0.804972323 | 1.808704989 | 0.070496848 |
| AURKB | ENST00000602594.1 | ENSG00000269930.1 | -0.864414491 | -1.936195245 | 0.052843793 |
| AURKB | ENST00000602809.1 | ENSG00000270105.1 | -0.847111508 | -1.90989722 | 0.056146448 |
| AURKB | ENST00000606470.1 | ENSG00000271913.1 | 0.845321198 | 1.868084078 | 0.061750353 |
| AURKB | ENST00000608159.1 | ENSG00000273093.1 | -0.932961518 | -2.090999362 | 0.036528121 |
| AURKB | ENST00000608258.1 | ENSG00000229042.2 | -0.828793733 | -1.863929457 | 0.062331611 |
| AURKB | ENST00000608275.1 | ENSG00000273343.1 | 0.862570079 | 1.955582121 | 0.050514401 |
| AURKB | ENST00000608856.1 | ENSG00000272600.1 | -0.837415203 | -1.896443268 | 0.057901455 |
| AURKB | NR_028324.1 | LINC01002 | 0.850414871 | 1.900520944 | 0.057364789 |
| AURKB | NR_047498.1 | LINC00853 | 0.832856862 | 1.849877945 | 0.064331143 |
| AURKB | NR_108085.1 | OVOL1-AS1 | 0.832687977 | 1.868477623 | 0.061695526 |
| AURKB | NR_120318.1 | RORA-AS2 | 0.89897634 | 2.033255031 | 0.042026764 |
| AURKB | NR_120371.1 | LINC01585 | 0.857734522 | 1.924158585 | 0.054334709 |
| AURKB | NR_125957.1 | LOC101928626 | -0.825134709 | -1.830364981 | 0.06719538 |
| AURKB | NR_133941.1 | LOC105377247 | -0.839854895 | -1.887230181 | 0.059129373 |
| AURKB | NR_135626.1 | LOC100505585 | 0.897044595 | 1.984870022 | 0.047158934 |

| | | | | | |
|---|---|---|---|---|---|
| AURKB | NR_138419.1 | ARHGEF9-IT1 | 0.818949863 | 1.849594149 | 0.064372067 |
| BAD | ENST00000411694.1 | ENSG00000225331.1 | 0.856799674 | 1.930848177 | 0.053501831 |
| BAD | ENST00000415205.1 | ENSG00000182057.4 | 0.922042238 | 2.058532259 | 0.039539068 |
| BAD | ENST00000417260.1 | ENSG00000231734.4 | -0.876730681 | -1.961297194 | 0.049844364 |
| BAD | ENST00000418387.1 | ENSG00000235056.1 | -0.915414038 | -2.051133334 | 0.040253964 |
| BAD | ENST00000426030.2 | ENSG00000228686.2 | -0.929577748 | -2.084252408 | 0.037137214 |
| BAD | ENST00000426302.1 | ENSG00000230454.1 | 0.874230653 | 1.950029726 | 0.051172576 |
| BAD | ENST00000430920.1 | ENSG00000234203.1 | 0.957273271 | 2.145736583 | 0.031894001 |
| BAD | ENST00000431730.1 | ENSG00000237401.2 | 0.904831409 | 2.02560318 | 0.042805465 |
| BAD | ENST00000436515.1 | ENSG00000224521.1 | -0.854597888 | -1.893161076 | 0.058336452 |
| BAD | ENST00000441592.2 | ENSG00000224078.8 | 0.836248248 | 1.876583006 | 0.060575268 |
| BAD | ENST00000442829.1 | ENSG00000225284.1 | 0.98802349 | 2.200981949 | 0.027737302 |
| BAD | ENST00000446562.1 | ENSG00000233896.1 | 0.913479013 | 2.054153943 | 0.039960794 |
| BAD | ENST00000446816.1 | ENSG00000204685.5 | 0.832355591 | 1.866037427 | 0.062036129 |
| BAD | ENST00000448674.1 | ENSG00000235119.1 | 0.850013854 | 1.885823367 | 0.059318761 |
| BAD | ENST00000448858.1 | ENSG00000237734.1 | -0.860776309 | -1.925910114 | 0.054115598 |
| BAD | ENST00000451034.1 | ENSG00000229805.1 | -0.882766752 | -1.927724387 | 0.053889417 |
| BAD | ENST00000451090.1 | ENSG00000235215.2 | -0.88548479 | -1.964748049 | 0.049443406 |
| BAD | ENST00000451507.1 | ENSG00000229539.1 | 0.85879579 | 1.919470006 | 0.054924879 |
| BAD | ENST00000452176.1 | ENSG00000223659.1 | -0.8730132 | -1.961848 | 0.049780183 |
| BAD | ENST00000455373.1 | ENSG00000226097.1 | -0.828359665 | -1.84806514 | 0.06459292 |
| BAD | ENST00000457371.1 | ENSG00000237401.2 | 0.845979857 | 1.882340568 | 0.05978979 |
| BAD | ENST00000463255.1 | ENSG00000243305.1 | -0.884418139 | -1.970880402 | 0.048737557 |
| BAD | ENST00000466431.2 | ENSG00000254485.1 | 0.822991378 | 1.829008444 | 0.067398343 |
| BAD | ENST00000468165.1 | ENSG00000239480.1 | 0.813106009 | 1.8219405 | 0.068464014 |
| BAD | ENST00000488310.1 | ENSG00000240449.1 | 0.860401433 | 1.941071063 | 0.052249658 |
| BAD | ENST00000489077.1 | ENSG00000244198.1 | 0.8318316 | 1.867805818 | 0.061789142 |
| BAD | ENST00000489090.1 | ENSG00000240045.1 | -0.899577825 | -2.005992906 | 0.044857005 |
| BAD | ENST00000489690.1 | ENSG00000243944.1 | -0.923047315 | -2.061534807 | 0.039252046 |
| BAD | ENST00000503723.1 | ENSG00000250472.1 | -0.904102823 | -2.018599427 | 0.043528871 |
| BAD | ENST00000506394.1 | ENSG00000251665.1 | 0.865546738 | 1.951671193 | 0.050977255 |
| BAD | ENST00000512036.1 | ENSG00000250993.1 | -0.827606604 | -1.851430505 | 0.064107644 |
| BAD | ENST00000515128.1 | ENSG00000248215.1 | -0.856505801 | -1.904009921 | 0.056908892 |
| BAD | ENST00000517846.1 | ENSG00000254485.1 | 0.898262781 | 2.005585401 | 0.044900501 |
| BAD | ENST00000521653.1 | ENSG00000253301.1 | 0.921670465 | 2.070211282 | 0.038432563 |
| BAD | ENST00000522390.1 | ENSG00000254262.1 | -0.82716154 | -1.848871573 | 0.064476359 |
| BAD | ENST00000527086.1 | ENSG00000255182.1 | 0.877183266 | 1.958799822 | 0.050136234 |
| BAD | ENST00000527757.1 | ENSG00000255109.1 | -0.892872256 | -1.99612541 | 0.045920274 |
| BAD | ENST00000529247.1 | ENSG00000254741.1 | 0.971293159 | 2.178130623 | 0.029396313 |
| BAD | ENST00000534178.1 | ENSG00000255120.1 | 0.896672583 | 2.016542401 | 0.043743289 |
| BAD | ENST00000537032.1 | ENSG00000255933.1 | 0.820523785 | 1.827545632 | 0.067617772 |
| BAD | ENST00000537850.1 | ENSG00000251002.3 | 0.827347165 | 1.8824946 | 0.059768892 |
| BAD | ENST00000543275.1 | ENSG00000256944.1 | 0.847078693 | 1.872335603 | 0.061160188 |
| BAD | ENST00000543403.1 | ENSG00000256684.1 | -0.949756547 | -2.138253853 | 0.032496146 |
| BAD | ENST00000543494.1 | ENSG00000256514.1 | 0.809831654 | 1.814300025 | 0.069631548 |
| BAD | ENST00000549806.1 | ENSG00000257252.1 | 0.950550377 | 2.108469808 | 0.034990372 |
| BAD | ENST00000550263.1 | ENSG00000257605.1 | 0.804707376 | 1.779374821 | 0.075178332 |
| BAD | ENST00000550279.1 | ENSG00000258338.1 | -0.939450318 | -2.105455301 | 0.035251694 |
| BAD | ENST00000552525.1 | ENSG00000257286.1 | 0.848041251 | 1.893423036 | 0.058301634 |
| BAD | ENST00000554431.1 | ENSG00000258616.1 | -0.947637171 | -2.105316907 | 0.035263731 |
| BAD | ENST00000557602.1 | ENSG00000258616.1 | -0.948990156 | -2.110659202 | 0.034801615 |

| | | | | | |
|---|---|---|---|---|---|
| BAD | ENST00000558221.1 | ENSG00000259704.1 | 0.820005336 | 1.834389259 | 0.066596233 |
| BAD | ENST00000558237.1 | ENSG00000259684.1 | -0.88245891 | -1.959759785 | 0.050023874 |
| BAD | ENST00000558575.1 | ENSG00000259687.1 | 0.800635545 | 1.806160148 | 0.070893328 |
| BAD | ENST00000562191.1 | ENSG00000261292.1 | -0.947315346 | -2.097253336 | 0.035971155 |
| BAD | ENST00000562995.1 | ENSG00000261253.1 | 0.894927732 | 1.976196921 | 0.048132478 |
| BAD | ENST00000563610.1 | ENSG00000260051.1 | 0.934493403 | 2.075854978 | 0.037907366 |
| BAD | ENST00000563611.1 | ENSG00000261583.1 | 0.877739118 | 1.960834101 | 0.049898379 |
| BAD | ENST00000563806.1 | ENSG00000238045.5 | 0.83094626 | 1.86529421 | 0.062140176 |
| BAD | ENST00000564809.1 | ENSG00000261471.1 | 0.945208271 | 2.116235348 | 0.034324796 |
| BAD | ENST00000565735.1 | ENSG00000261213.1 | -0.802407253 | -1.794588906 | 0.072719222 |
| BAD | ENST00000569313.1 | ENSG00000261604.1 | -0.808488888 | -1.7900402 | 0.073447449 |
| BAD | ENST00000570493.2 | ENSG00000261898.2 | 0.819159624 | 1.840834311 | 0.065645843 |
| BAD | ENST00000570974.1 | ENSG00000263300.1 | 0.840454429 | 1.881635127 | 0.059885573 |
| BAD | ENST00000571775.1 | ENSG00000262456.1 | 0.860788748 | 1.914227178 | 0.055591135 |
| BAD | ENST00000576271.1 | ENSG00000263342.1 | 0.828299905 | 1.870481881 | 0.061416931 |
| BAD | ENST00000577853.1 | ENSG00000264207.1 | 0.933583322 | 2.089832387 | 0.036632859 |
| BAD | ENST00000578800.1 | ENSG00000264235.1 | 0.813975919 | 1.807766299 | 0.070642881 |
| BAD | ENST00000579775.1 | ENSG00000264108.1 | 0.802100716 | 1.795477366 | 0.072577676 |
| BAD | ENST00000580311.1 | ENSG00000266803.1 | -0.921272531 | -2.078723256 | 0.037642794 |
| BAD | ENST00000580622.1 | ENSG00000264634.1 | 0.838295805 | 1.861164108 | 0.062721005 |
| BAD | ENST00000582044.1 | ENSG00000263715.2 | 0.8939509 | 1.987234769 | 0.046896387 |
| BAD | ENST00000588380.1 | ENSG00000266990.1 | 0.895926822 | 2.014016131 | 0.04400784 |
| BAD | ENST00000588402.1 | ENSG00000267006.1 | -0.968967087 | -2.181760062 | 0.029127246 |
| BAD | ENST00000590328.1 | ENSG00000256995.2 | -0.805795853 | -1.798346756 | 0.072122075 |
| BAD | ENST00000591174.1 | ENSG00000267289.1 | 0.864243986 | 1.942541155 | 0.052071623 |
| BAD | ENST00000592518.1 | ENSG00000267786.1 | 0.850857371 | 1.916847945 | 0.055257252 |
| BAD | ENST00000592525.1 | ENSG00000267214.1 | 0.886921082 | 1.988644445 | 0.046740462 |
| BAD | ENST00000592816.1 | ENSG00000236172.2 | 0.803203213 | 1.825512261 | 0.067923763 |
| BAD | ENST00000593139.1 | ENSG00000267042.1 | 0.866701981 | 1.937332541 | 0.052704707 |
| BAD | ENST00000596091.1 | ENSG00000227733.4 | -0.835649214 | -1.836649497 | 0.066261655 |
| BAD | ENST00000596567.1 | ENSG00000226647.2 | -0.839850235 | -1.879319005 | 0.060200948 |
| BAD | ENST00000596887.1 | ENSG00000237031.3 | -0.948733732 | -2.114070706 | 0.034509229 |
| BAD | ENST00000597256.1 | ENSG00000267986.1 | 0.824784943 | 1.861511111 | 0.062672033 |
| BAD | ENST00000598092.1 | ENSG00000228065.6 | -0.860130478 | -1.9419909 | 0.052138202 |
| BAD | ENST00000600242.1 | ENSG00000269583.1 | 0.929592611 | 2.072003505 | 0.038265114 |
| BAD | ENST00000600716.1 | ENSG00000269487.1 | 0.859682023 | 1.919187015 | 0.05496067 |
| BAD | ENST00000600726.1 | ENSG00000267858.1 | 0.873621231 | 1.938236134 | 0.05259442 |
| BAD | ENST00000604142.1 | ENSG00000271308.1 | 0.830981007 | 1.83719024 | 0.066181816 |
| BAD | ENST00000604183.1 | ENSG00000271185.1 | 0.95279589 | 2.142532682 | 0.032150642 |
| BAD | ENST00000606909.1 | ENSG00000271821.1 | 0.817092265 | 1.84847695 | 0.064533376 |
| BAD | ENST00000607476.1 | ENSG00000272540.1 | 0.843121261 | 1.863736768 | 0.062358679 |
| BAD | ENST00000607943.1 | ENSG00000273188.1 | 0.900712777 | 2.016790128 | 0.04371742 |
| BAD | ENST00000608259.1 | ENSG00000272627.1 | -0.846734288 | -1.89643445 | 0.05790262 |
| BAD | ENST00000608367.1 | ENSG00000273361.1 | 0.972999669 | 2.182982733 | 0.029037082 |
| BAD | ENST00000608489.1 | ENSG00000272716.1 | 0.853842657 | 1.906390884 | 0.056599511 |
| BAD | ENST00000609113.1 | ENSG00000272827.1 | 0.868131735 | 1.960661765 | 0.049918492 |
| BAD | ENST00000609813.1 | ENSG00000272719.1 | 0.807820443 | 1.796500996 | 0.072414874 |
| BAD | NR_026802.1 | FAM74A4 | 0.83209853 | 1.868750591 | 0.061657522 |
| BAD | NR_026951.1 | LINC00324 | 0.90125524 | 2.017943283 | 0.043597169 |
| BAD | NR_027052.1 | THAP7-AS1 | 0.899383141 | 2.010747779 | 0.044352105 |
| BAD | NR_036480.1 | VPS9D1-AS1 | 0.863186457 | 1.93526315 | 0.052958012 |

| | | | | | |
|---|---|---|---|---|---|
| BAD | NR_037169.1 | LOC100507547 | 0.801852647 | 1.804674155 | 0.071125686 |
| BAD | NR_038421.1 | LINC01220 | 0.902502367 | 2.008664015 | 0.044572777 |
| BAD | NR_038923.1 | SSSCA1-AS1 | 0.933139959 | 2.081931978 | 0.037348683 |
| BAD | NR_040049.1 | SDCBP2-AS1 | 0.835931736 | 1.847570375 | 0.064664519 |
| BAD | NR_047498.1 | LINC00853 | 0.805680219 | 1.810294116 | 0.070250189 |
| BAD | NR_073155.1 | C1orf145 | -0.838855694 | -1.879993631 | 0.060108946 |
| BAD | NR_103830.1 | LINC00587 | -0.829806326 | -1.87524578 | 0.060758919 |
| BAD | NR_108106.1 | LINC01135 | 0.897206844 | 2.009887764 | 0.044443069 |
| BAD | NR_109831.1 | RASSF1-AS1 | 0.921024598 | 2.071122403 | 0.038347359 |
| BAD | NR_110245.1 | LOC101929282 | -0.866941947 | -1.955012112 | 0.050581641 |
| BAD | NR_110556.1 | LOC102724890 | -0.814837645 | -1.80740768 | 0.070698737 |
| BAD | NR_110630.1 | LOC101927478 | 0.905654223 | 2.020415977 | 0.043340258 |
| BAD | NR_110998.1 | FAM74A4 | 0.83209853 | 1.873102705 | 0.061054204 |
| BAD | NR_111951.1 | LINC00869 | 0.925680197 | 2.042106409 | 0.041140977 |
| BAD | NR_111952.1 | LINC00869 | 0.900025082 | 2.039750071 | 0.041375225 |
| BAD | NR_111953.1 | LINC00869 | 0.921688251 | 2.084449225 | 0.037119324 |
| BAD | NR_125849.1 | LOC101928140 | -0.927348567 | -2.072094276 | 0.03825665 |
| BAD | NR_125957.1 | LOC101928626 | -0.8730132 | -1.968673099 | 0.048990642 |
| BAD | NR_126522.1 | EXOC3-AS1 | 0.895683782 | 2.013542953 | 0.044057541 |
| BAD | NR_134520.1 | LOC727993 | 0.921264265 | 2.047215916 | 0.040636889 |
| BAD | NR_134579.1 | LOC105372179 | 0.811216354 | 1.801491595 | 0.071625431 |
| BAD | NR_135024.1 | LOC105369747 | 0.93188057 | 2.069723275 | 0.038478266 |
| BAD | NR_135040.1 | LOC101927038 | 0.853378542 | 1.908198958 | 0.056365506 |
| BAD | NR_135041.1 | LOC101927038 | 0.846925408 | 1.906417235 | 0.056596094 |
| BAD | NR_135097.1 | LOC105369443 | -0.949756547 | -2.118440547 | 0.034137775 |
| BAD | NR_135626.1 | LOC100505585 | 0.806051579 | 1.794427923 | 0.072744893 |
| BAD | NR_144459.1 | ARSD-AS1 | 0.86177309 | 1.953619178 | 0.050746271 |
| BCL2A1 | ENST00000398777.3 | ENSG00000240152.2 | 0.850832624 | 1.911861412 | 0.055893972 |
| BCL2A1 | ENST00000412647.2 | ENSG00000232964.2 | 0.876559436 | 1.96349024 | 0.049589237 |
| BCL2A1 | ENST00000414377.1 | ENSG00000230470.1 | 0.820080517 | 1.857934583 | 0.063178305 |
| BCL2A1 | ENST00000416220.1 | ENSG00000236753.1 | 0.954317513 | 2.12650215 | 0.03346147 |
| BCL2A1 | ENST00000416641.1 | ENSG00000226956.1 | -0.830878239 | -1.845275525 | 0.064997469 |
| BCL2A1 | ENST00000417426.1 | ENSG00000233145.1 | 0.821111012 | 1.813922039 | 0.069689729 |
| BCL2A1 | ENST00000418741.1 | ENSG00000227332.1 | 0.844079403 | 1.893911085 | 0.058236813 |
| BCL2A1 | ENST00000421252.2 | ENSG00000250258.1 | -0.846324119 | -1.879019596 | 0.060241818 |
| BCL2A1 | ENST00000423667.1 | ENSG00000225970.1 | 0.837153777 | 1.870164779 | 0.061460939 |
| BCL2A1 | ENST00000426213.1 | ENSG00000223660.1 | -0.965059994 | -2.144879896 | 0.031962451 |
| BCL2A1 | ENST00000432431.1 | ENSG00000234940.1 | 0.94299317 | 2.11189513 | 0.034695445 |
| BCL2A1 | ENST00000434493.1 | ENSG00000224605.1 | 0.832780208 | 1.866510752 | 0.061969941 |
| BCL2A1 | ENST00000435832.1 | ENSG00000229201.1 | 0.853721834 | 1.920537783 | 0.054790005 |
| BCL2A1 | ENST00000437680.1 | ENSG00000237133.1 | -0.855884292 | -1.911857773 | 0.055894439 |
| BCL2A1 | ENST00000438850.1 | ENSG00000267338.1 | 0.80091775 | 1.808407029 | 0.070543175 |
| BCL2A1 | ENST00000439186.1 | ENSG00000237076.1 | 0.848138917 | 1.917347031 | 0.055193859 |
| BCL2A1 | ENST00000439455.1 | ENSG00000233482.1 | -0.952920711 | -2.118159627 | 0.034161551 |
| BCL2A1 | ENST00000440862.1 | ENSG00000223804.1 | -0.963423424 | -2.142930333 | 0.032118693 |
| BCL2A1 | ENST00000442579.1 | ENSG00000228719.1 | -0.889940362 | -1.9765455 | 0.048093028 |
| BCL2A1 | ENST00000444245.1 | ENSG00000236753.1 | 0.957802706 | 2.137127273 | 0.032587641 |
| BCL2A1 | ENST00000447183.2 | ENSG00000271593.1 | 0.815528185 | 1.836767432 | 0.066244236 |
| BCL2A1 | ENST00000447514.1 | ENSG00000236753.1 | 0.81732725 | 1.826468914 | 0.06777966 |
| BCL2A1 | ENST00000448001.1 | ENSG00000229639.1 | 0.859436509 | 1.884277073 | 0.059527507 |
| BCL2A1 | ENST00000450206.1 | ENSG00000234311.1 | -0.893380436 | -1.985905062 | 0.047043867 |

| | | | | | |
|---|---|---|---|---|---|
| BCL2A1 | ENST00000450365.1 | ENSG00000224404.1 | 0.862204283 | 1.902905886 | 0.057052827 |
| BCL2A1 | ENST00000450848.1 | ENSG00000225539.1 | 0.914789084 | 2.04468592 | 0.040885833 |
| BCL2A1 | ENST00000451575.2 | ENSG00000224251.2 | 0.84305414 | 1.894845906 | 0.058112819 |
| BCL2A1 | ENST00000452553.1 | ENSG00000233973.1 | 0.836129135 | 1.876017744 | 0.060652843 |
| BCL2A1 | ENST00000453878.1 | ENSG00000224850.1 | 0.883933557 | 1.986460772 | 0.046982184 |
| BCL2A1 | ENST00000454515.1 | ENSG00000236753.1 | 0.90920508 | 2.042760231 | 0.041076179 |
| BCL2A1 | ENST00000455238.1 | ENSG00000231413.1 | 0.819164887 | 1.813499925 | 0.06975475 |
| BCL2A1 | ENST00000455416.1 | ENSG00000229337.1 | -0.916076963 | -2.034661987 | 0.041884896 |
| BCL2A1 | ENST00000455699.1 | ENSG00000240996.1 | 0.815103387 | 1.828986773 | 0.06740159 |
| BCL2A1 | ENST00000457169.1 | ENSG00000232408.1 | 0.908612882 | 2.01657468 | 0.043739918 |
| BCL2A1 | ENST00000468444.2 | ENSG00000258525.1 | -0.88659069 | -1.969679558 | 0.048875107 |
| BCL2A1 | ENST00000501405.2 | ENSG00000247402.2 | -0.829095243 | -1.824637511 | 0.068055749 |
| BCL2A1 | ENST00000503938.1 | ENSG00000246095.2 | 0.8902828 | 1.99129294 | 0.046448691 |
| BCL2A1 | ENST00000504017.1 | ENSG00000248388.1 | 0.852072978 | 1.895552241 | 0.058019278 |
| BCL2A1 | ENST00000504344.1 | ENSG00000251438.1 | 0.93063252 | 2.067350602 | 0.038701129 |
| BCL2A1 | ENST00000504795.1 | ENSG00000250723.1 | 0.837466655 | 1.855880445 | 0.063470601 |
| BCL2A1 | ENST00000506058.1 | ENSG00000248261.1 | -0.949236852 | -2.109759034 | 0.034879117 |
| BCL2A1 | ENST00000506059.1 | ENSG00000248311.1 | 0.946606667 | 2.130121851 | 0.033161555 |
| BCL2A1 | ENST00000508081.1 | ENSG00000248254.1 | -0.837289667 | -1.867211675 | 0.061872034 |
| BCL2A1 | ENST00000508845.1 | ENSG00000271724.1 | 0.907795175 | 2.036887411 | 0.041661326 |
| BCL2A1 | ENST00000508936.1 | ENSG00000250582.1 | 0.876706653 | 1.961393453 | 0.049833143 |
| BCL2A1 | ENST00000513023.1 | ENSG00000248809.1 | 0.94398825 | 2.091483615 | 0.036484734 |
| BCL2A1 | ENST00000514802.1 | ENSG00000250190.1 | 0.949471896 | 2.15044213 | 0.031520259 |
| BCL2A1 | ENST00000517716.1 | ENSG00000253515.1 | -0.827548052 | -1.857530661 | 0.063235693 |
| BCL2A1 | ENST00000518416.1 | ENSG00000253901.1 | 0.977955562 | 2.196735235 | 0.028039361 |
| BCL2A1 | ENST00000519660.1 | ENSG00000253416.1 | 0.909220036 | 2.025600219 | 0.042805769 |
| BCL2A1 | ENST00000521378.1 | ENSG00000254222.1 | -0.816808189 | -1.830217064 | 0.067217487 |
| BCL2A1 | ENST00000521953.1 | ENSG00000253214.1 | 0.870145178 | 1.935997241 | 0.052868039 |
| BCL2A1 | ENST00000524275.1 | ENSG00000253507.1 | 0.807237691 | 1.788637328 | 0.073673242 |
| BCL2A1 | ENST00000529160.1 | ENSG00000246790.2 | -0.813357844 | -1.834810251 | 0.066533809 |
| BCL2A1 | ENST00000529837.1 | ENSG00000254687.1 | 0.930084902 | 2.079719503 | 0.037551268 |
| BCL2A1 | ENST00000529875.1 | ENSG00000254404.1 | -0.870173731 | -1.944241801 | 0.051866299 |
| BCL2A1 | ENST00000536529.1 | ENSG00000256422.1 | 0.876535796 | 1.97449286 | 0.048325729 |
| BCL2A1 | ENST00000545593.1 | ENSG00000256972.1 | 0.8768065 | 1.969583803 | 0.048886089 |
| BCL2A1 | ENST00000549329.1 | ENSG00000224189.2 | 0.847441551 | 1.885069823 | 0.059420412 |
| BCL2A1 | ENST00000549756.1 | ENSG00000257769.1 | -0.960080051 | -2.148702836 | 0.031657964 |
| BCL2A1 | ENST00000552156.1 | ENSG00000224189.2 | 0.81160242 | 1.803686742 | 0.071280429 |
| BCL2A1 | ENST00000553464.1 | ENSG00000258418.1 | 0.901045291 | 2.052360505 | 0.04013464 |
| BCL2A1 | ENST00000555966.1 | ENSG00000258843.1 | 0.866166844 | 1.944207226 | 0.051870467 |
| BCL2A1 | ENST00000556458.1 | ENSG00000258504.2 | -0.830362737 | -1.834252315 | 0.066616549 |
| BCL2A1 | ENST00000556786.1 | ENSG00000258525.1 | -0.836159675 | -1.879937734 | 0.060116565 |
| BCL2A1 | ENST00000557903.1 | ENSG00000259182.1 | 0.819114813 | 1.849521931 | 0.064382484 |
| BCL2A1 | ENST00000557965.1 | ENSG00000259681.1 | 0.834235 | 1.872566038 | 0.061128335 |
| BCL2A1 | ENST00000560586.1 | ENSG00000259534.1 | 0.878457518 | 1.977529221 | 0.047981841 |
| BCL2A1 | ENST00000561653.1 | ENSG00000260095.1 | -0.844916217 | -1.890119684 | 0.058741955 |
| BCL2A1 | ENST00000563841.1 | ENSG00000261029.1 | 0.858261398 | 1.924267403 | 0.054321074 |
| BCL2A1 | ENST00000565965.1 | ENSG00000261172.1 | 0.86947366 | 1.976430474 | 0.048106043 |
| BCL2A1 | ENST00000569147.1 | ENSG00000261592.1 | -0.852037861 | -1.895236463 | 0.058061081 |
| BCL2A1 | ENST00000572417.1 | ENSG00000263171.1 | 0.859733639 | 1.931538415 | 0.053416503 |
| BCL2A1 | ENST00000577698.1 | ENSG00000265100.1 | 0.81066965 | 1.802227068 | 0.071509688 |
| BCL2A1 | ENST00000578035.1 | ENSG00000266743.1 | -0.846712798 | -1.895807201 | 0.057985543 |

| | | | | | |
|---|---|---|---|---|---|
| BCL2A1 | ENST00000588177.1 | ENSG00000234899.5 | 0.872569229 | 1.961669239 | 0.049801005 |
| BCL2A1 | ENST00000588799.1 | ENSG00000267275.1 | 0.813330696 | 1.807157367 | 0.070737746 |
| BCL2A1 | ENST00000589777.1 | ENSG00000261040.2 | 0.810457739 | 1.849018726 | 0.064455109 |
| BCL2A1 | ENST00000592431.1 | ENSG00000267475.1 | -0.820105698 | -1.849928429 | 0.064323866 |
| BCL2A1 | ENST00000594762.1 | ENSG00000231898.4 | 0.944804754 | 2.108060206 | 0.035025782 |
| BCL2A1 | ENST00000595409.1 | ENSG00000232729.3 | 0.907521184 | 2.036758207 | 0.041674278 |
| BCL2A1 | ENST00000595972.1 | ENSG00000230333.2 | 0.815292941 | 1.831374918 | 0.067044602 |
| BCL2A1 | ENST00000599524.1 | ENSG00000268240.1 | -0.839636925 | -1.89728297 | 0.057790601 |
| BCL2A1 | ENST00000600489.1 | ENSG00000231898.4 | 0.855477362 | 1.915970034 | 0.05536891 |
| BCL2A1 | ENST00000602435.1 | ENSG00000269952.1 | 0.882867446 | 1.986934471 | 0.046929659 |
| BCL2A1 | ENST00000602507.1 | ENSG00000270069.1 | 0.875421622 | 1.961868292 | 0.04977782 |
| BCL2A1 | ENST00000602592.1 | ENSG00000270049.1 | 0.944968726 | 2.076169665 | 0.037878262 |
| BCL2A1 | ENST00000602790.1 | ENSG00000270000.1 | -0.944019336 | -2.095887893 | 0.036092137 |
| BCL2A1 | ENST00000602835.1 | ENSG00000270096.1 | 0.818652055 | 1.824720329 | 0.068043244 |
| BCL2A1 | ENST00000602872.1 | ENSG00000270067.1 | 0.836931236 | 1.887989181 | 0.059027403 |
| BCL2A1 | ENST00000602954.1 | ENSG00000269906.1 | 0.83708613 | 1.854716308 | 0.063636749 |
| BCL2A1 | ENST00000603474.1 | ENSG00000258929.2 | 0.813572063 | 1.832286019 | 0.066908819 |
| BCL2A1 | ENST00000606048.1 | ENSG00000272343.1 | 0.91396235 | 2.042354573 | 0.041116372 |
| BCL2A1 | ENST00000606457.1 | ENSG00000271830.1 | 0.817266498 | 1.819858804 | 0.06878051 |
| BCL2A1 | ENST00000606757.1 | ENSG00000237188.3 | -0.875508341 | -1.954005963 | 0.050700512 |
| BCL2A1 | ENST00000606898.1 | ENSG00000272094.1 | -0.815371449 | -1.807570456 | 0.07067338 |
| BCL2A1 | ENST00000607594.1 | ENSG00000271766.1 | 0.865972692 | 1.937073865 | 0.052736315 |
| BCL2A1 | ENST00000607769.1 | ENSG00000272438.1 | -0.932610434 | -2.07050266 | 0.038405297 |
| BCL2A1 | ENST00000608476.1 | ENSG00000232675.3 | 0.868238183 | 1.9424398 | 0.052083881 |
| BCL2A1 | ENST00000609270.1 | ENSG00000273073.1 | 0.905750068 | 2.026645439 | 0.042698686 |
| BCL2A1 | ENST00000609610.1 | ENSG00000232675.3 | 0.803283273 | 1.81660617 | 0.069277438 |
| BCL2A1 | ENST00000609612.1 | ENSG00000273424.1 | -0.839401025 | -1.852374264 | 0.063972098 |
| BCL2A1 | ENST00000609701.1 | ENSG00000273284.1 | 0.936679007 | 2.120297247 | 0.033980986 |
| BCL2A1 | ENST00000609775.1 | ENSG00000273232.1 | 0.830499738 | 1.862868096 | 0.062480826 |
| BCL2A1 | ENST00000609890.1 | ENSG00000231898.4 | 0.87104882 | 1.939716096 | 0.052414202 |
| BCL2A1 | ENST00000609952.1 | ENSG00000233766.3 | 0.843655815 | 1.892757962 | 0.058390064 |
| BCL2A1 | NR_024410.1 | LINC00710 | 0.800105533 | 1.801820746 | 0.071573613 |
| BCL2A1 | NR_026932.1 | PDCD4-AS1 | -0.835828268 | -1.877968439 | 0.060385483 |
| BCL2A1 | NR_040061.1 | SRP14-AS1 | -0.903627941 | -2.028988612 | 0.04245945 |
| BCL2A1 | NR_104620.1 | LINC01672 | 0.823891566 | 1.846082229 | 0.064880267 |
| BCL2A1 | NR_108046.1 | LINC00844 | 0.867808856 | 1.937428318 | 0.052693008 |
| BCL2A1 | NR_108077.1 | SMAD1-AS2 | 0.824545096 | 1.857853484 | 0.063189824 |
| BCL2A1 | NR_110370.1 | STAM-AS1 | -0.894898107 | -2.010448382 | 0.044383754 |
| BCL2A1 | NR_110803.1 | LOC101927018 | -0.802986438 | -1.801081379 | 0.071690054 |
| BCL2A1 | NR_134597.1 | LOC105378068 | 0.89580271 | 1.992772105 | 0.046286408 |
| BCL2A1 | NR_134665.1 | LOC105374366 | 0.817336702 | 1.819972196 | 0.068763239 |
| BCL2A1 | NR_135108.1 | LOC105369509 | 0.869038186 | 1.933009265 | 0.053235056 |
| BCL2A1 | NR_136320.1 | LOC105373656 | -0.848293616 | -1.872268824 | 0.061169422 |
| BOP1 | ENST00000411694.1 | ENSG00000225331.1 | 0.812731501 | 1.813239368 | 0.06979491 |
| BOP1 | ENST00000412085.1 | ENSG00000233825.1 | 0.814187668 | 1.813914009 | 0.069690966 |
| BOP1 | ENST00000412759.1 | ENSG00000236933.1 | 0.874036719 | 1.964990576 | 0.049415328 |
| BOP1 | ENST00000415205.1 | ENSG00000182057.4 | 0.939248522 | 2.136175795 | 0.032665088 |
| BOP1 | ENST00000417260.1 | ENSG00000231734.4 | -0.824135328 | -1.841465989 | 0.0655533 |
| BOP1 | ENST00000419662.1 | ENSG00000228265.1 | 0.900732944 | 1.99847365 | 0.045665334 |
| BOP1 | ENST00000422763.1 | ENSG00000231131.2 | -0.864971598 | -1.951744314 | 0.050968569 |
| BOP1 | ENST00000425881.1 | ENSG00000239636.1 | 0.85288797 | 1.903782102 | 0.056938568 |

| | | | | | |
|---|---|---|---|---|---|
| BOP1 | ENST00000426237.2 | ENSG00000235527.2 | 0.813605466 | 1.791360516 | 0.073235461 |
| BOP1 | ENST00000426475.1 | ENSG00000239467.1 | 0.904724873 | 2.045224383 | 0.040832742 |
| BOP1 | ENST00000428765.1 | ENSG00000230107.1 | 0.85919736 | 1.885049789 | 0.059423116 |
| BOP1 | ENST00000429080.1 | ENSG00000233047.1 | -0.892307859 | -1.990471154 | 0.046539059 |
| BOP1 | ENST00000430920.1 | ENSG00000234203.1 | 0.925556558 | 2.067901202 | 0.038649314 |
| BOP1 | ENST00000431730.1 | ENSG00000237401.2 | 0.815208594 | 1.831578318 | 0.06701427 |
| BOP1 | ENST00000433051.1 | ENSG00000233193.1 | 0.845882592 | 1.883359091 | 0.05965172 |
| BOP1 | ENST00000433905.2 | ENSG00000229299.2 | 0.935663055 | 2.100074439 | 0.035722293 |
| BOP1 | ENST00000438190.1 | ENSG00000227214.2 | 0.902294052 | 2.007338075 | 0.044713678 |
| BOP1 | ENST00000438969.2 | ENSG00000228031.2 | -0.833048823 | -1.881743045 | 0.059870912 |
| BOP1 | ENST00000439184.1 | ENSG00000233985.1 | -0.818917697 | -1.832162004 | 0.066927288 |
| BOP1 | ENST00000440595.1 | ENSG00000228265.1 | 0.892058306 | 2.006051526 | 0.044850751 |
| BOP1 | ENST00000441592.2 | ENSG00000224078.8 | 0.929482675 | 2.096350636 | 0.036051098 |
| BOP1 | ENST00000442829.1 | ENSG00000225284.1 | 0.823374524 | 1.844062179 | 0.065174079 |
| BOP1 | ENST00000446562.1 | ENSG00000233896.1 | 0.871132977 | 1.969639573 | 0.048879693 |
| BOP1 | ENST00000447343.2 | ENSG00000229299.2 | 0.929709774 | 2.049722557 | 0.040391513 |
| BOP1 | ENST00000448570.1 | ENSG00000224549.1 | 0.833758439 | 1.867130661 | 0.061883344 |
| BOP1 | ENST00000448858.1 | ENSG00000237734.1 | -0.81584059 | -1.811612447 | 0.0700461 |
| BOP1 | ENST00000451507.1 | ENSG00000229539.1 | 0.90266243 | 2.019819454 | 0.043402119 |
| BOP1 | ENST00000452176.1 | ENSG00000223659.1 | -0.84986619 | -1.912742556 | 0.055781018 |
| BOP1 | ENST00000453051.1 | ENSG00000229407.1 | 0.870959917 | 1.938669139 | 0.052541639 |
| BOP1 | ENST00000457253.1 | ENSG00000225173.1 | 0.803888398 | 1.797228829 | 0.0722993 |
| BOP1 | ENST00000458154.1 | ENSG00000235578.1 | 0.819234107 | 1.810429645 | 0.070229186 |
| BOP1 | ENST00000463255.1 | ENSG00000243305.1 | -0.895338957 | -1.99514647 | 0.046026907 |
| BOP1 | ENST00000468165.1 | ENSG00000239480.1 | 0.910215542 | 2.04573014 | 0.040782929 |
| BOP1 | ENST00000489077.1 | ENSG00000244198.1 | 0.963217361 | 2.163590353 | 0.030495803 |
| BOP1 | ENST00000489090.1 | ENSG00000240045.1 | -0.827089709 | -1.841347487 | 0.065570653 |
| BOP1 | ENST00000498693.1 | ENSG00000244198.1 | 0.883450386 | 1.991202499 | 0.046458629 |
| BOP1 | ENST00000503723.1 | ENSG00000250472.1 | -0.972759786 | -2.196661543 | 0.028044627 |
| BOP1 | ENST00000504916.1 | ENSG00000248112.1 | -0.841877216 | -1.868372432 | 0.061710177 |
| BOP1 | ENST00000506394.1 | ENSG00000251665.1 | 0.818434801 | 1.840416238 | 0.065707151 |
| BOP1 | ENST00000506791.1 | ENSG00000251131.1 | 0.884852929 | 1.963416958 | 0.049597745 |
| BOP1 | ENST00000509036.1 | ENSG00000251131.1 | 0.904595397 | 2.013607014 | 0.04405081 |
| BOP1 | ENST00000509192.1 | ENSG00000250765.1 | 0.871227165 | 1.953109552 | 0.050806615 |
| BOP1 | ENST00000509453.1 | ENSG00000249145.1 | 0.876094469 | 1.967854938 | 0.049084731 |
| BOP1 | ENST00000510570.1 | ENSG00000250438.1 | -0.907240841 | -2.045560715 | 0.04079961 |
| BOP1 | ENST00000517846.1 | ENSG00000254485.1 | 0.922780184 | 2.063125178 | 0.039100737 |
| BOP1 | ENST00000520603.1 | ENSG00000254001.1 | -0.912067215 | -2.02009167 | 0.04337388 |
| BOP1 | ENST00000521653.1 | ENSG00000253301.1 | 0.936032436 | 2.118652334 | 0.034119859 |
| BOP1 | ENST00000523806.1 | ENSG00000253616.1 | 0.803346864 | 1.812679156 | 0.069881321 |
| BOP1 | ENST00000529247.1 | ENSG00000254741.1 | 0.896033231 | 2.003937059 | 0.045076803 |
| BOP1 | ENST00000543275.1 | ENSG00000256944.1 | 0.866636307 | 1.913068673 | 0.055739262 |
| BOP1 | ENST00000543403.1 | ENSG00000256684.1 | -0.850904042 | -1.89424062 | 0.058193079 |
| BOP1 | ENST00000545177.3 | ENSG00000230438.5 | 0.846666822 | 1.880380679 | 0.060056215 |
| BOP1 | ENST00000549806.1 | ENSG00000257252.1 | 0.920345827 | 2.045883105 | 0.040767873 |
| BOP1 | ENST00000558575.1 | ENSG00000259687.1 | 0.832660753 | 1.852049639 | 0.064018695 |
| BOP1 | ENST00000562191.1 | ENSG00000261292.1 | -0.867417445 | -1.963479301 | 0.049590507 |
| BOP1 | ENST00000563610.1 | ENSG00000260051.1 | 0.802309775 | 1.792451086 | 0.073060738 |
| BOP1 | ENST00000563611.1 | ENSG00000261583.1 | 0.958821866 | 2.167267832 | 0.030214438 |
| BOP1 | ENST00000564809.1 | ENSG00000261471.1 | 0.922065773 | 2.062014057 | 0.039206398 |
| BOP1 | ENST00000565735.1 | ENSG00000261213.1 | -0.803262852 | -1.785540204 | 0.074173736 |

| | | | | | |
|---|---|---|---|---|---|
| BOP1 | ENST00000571660.1 | ENSG00000262848.1 | 0.835284402 | 1.879293772 | 0.060204392 |
| BOP1 | ENST00000577853.1 | ENSG00000264207.1 | 0.845157827 | 1.871162016 | 0.061322628 |
| BOP1 | ENST00000578800.1 | ENSG00000264235.1 | 0.925219695 | 2.051042671 | 0.040262791 |
| BOP1 | ENST00000579775.1 | ENSG00000264108.1 | 0.848809812 | 1.904808968 | 0.056804908 |
| BOP1 | ENST00000580622.1 | ENSG00000264634.1 | 0.933982633 | 2.106596225 | 0.035152594 |
| BOP1 | ENST00000582044.1 | ENSG00000263715.2 | 0.990556623 | 2.228066212 | 0.0258761 |
| BOP1 | ENST00000582558.1 | ENSG00000264569.1 | 0.852125729 | 1.882731353 | 0.059736784 |
| BOP1 | ENST00000586694.1 | ENSG00000267141.1 | 0.811963394 | 1.831780017 | 0.066984202 |
| BOP1 | ENST00000588182.2 | ENSG00000267453.2 | 0.873162666 | 1.940083558 | 0.052369536 |
| BOP1 | ENST00000588380.1 | ENSG00000266990.1 | 0.89916102 | 1.983510497 | 0.047310436 |
| BOP1 | ENST00000588402.1 | ENSG00000267006.1 | -0.847657602 | -1.906195702 | 0.05662482 |
| BOP1 | ENST00000589380.1 | ENSG00000267488.1 | 0.843272648 | 1.901266756 | 0.057267081 |
| BOP1 | ENST00000589395.1 | ENSG00000267143.1 | 0.842630871 | 1.894084573 | 0.058213785 |
| BOP1 | ENST00000591174.1 | ENSG00000267289.1 | 0.849060199 | 1.919536329 | 0.054916493 |
| BOP1 | ENST00000592498.1 | ENSG00000267488.1 | 0.842966074 | 1.87647272 | 0.060590397 |
| BOP1 | ENST00000592525.1 | ENSG00000267214.1 | 0.947584653 | 2.135524238 | 0.032718213 |
| BOP1 | ENST00000593139.1 | ENSG00000267042.1 | 0.952741052 | 2.143611926 | 0.032063994 |
| BOP1 | ENST00000594776.1 | ENSG00000269807.1 | 0.815976414 | 1.823087983 | 0.068290066 |
| BOP1 | ENST00000594850.1 | ENSG00000268093.1 | 0.90483755 | 2.021628205 | 0.043214776 |
| BOP1 | ENST00000596091.1 | ENSG00000227733.4 | -0.852079002 | -1.881075244 | 0.059961684 |
| BOP1 | ENST00000596887.1 | ENSG00000237031.3 | -0.93228937 | -2.110664571 | 0.034801153 |
| BOP1 | ENST00000596971.1 | ENSG00000269463.1 | 0.821245442 | 1.858805121 | 0.063054766 |
| BOP1 | ENST00000597169.1 | ENSG00000269720.1 | 0.843259594 | 1.864530996 | 0.062247173 |
| BOP1 | ENST00000597256.1 | ENSG00000267986.1 | 0.956322476 | 2.137037454 | 0.032594945 |
| BOP1 | ENST00000598092.1 | ENSG00000228065.6 | -0.820657361 | -1.840386634 | 0.065711494 |
| BOP1 | ENST00000600242.1 | ENSG00000269583.1 | 0.931084376 | 2.06561983 | 0.03886439 |
| BOP1 | ENST00000600726.1 | ENSG00000267858.1 | 0.876852574 | 1.965217576 | 0.049389061 |
| BOP1 | ENST00000601033.1 | ENSG00000268401.1 | 0.844948108 | 1.87280236 | 0.061095682 |
| BOP1 | ENST00000604142.1 | ENSG00000271308.1 | 0.945494572 | 2.106242292 | 0.035183311 |
| BOP1 | ENST00000604183.1 | ENSG00000271185.1 | 0.952939955 | 2.135199032 | 0.032744756 |
| BOP1 | ENST00000606277.1 | ENSG00000272145.1 | 0.872586437 | 1.941793917 | 0.052162054 |
| BOP1 | ENST00000606441.1 | ENSG00000272277.1 | 0.882282409 | 2.004896805 | 0.04497408 |
| BOP1 | ENST00000606743.1 | ENSG00000272221.1 | 0.832483107 | 1.844788495 | 0.065068312 |
| BOP1 | ENST00000606909.1 | ENSG00000271821.1 | 0.919345622 | 2.05926622 | 0.039468743 |
| BOP1 | ENST00000607224.1 | ENSG00000272521.1 | 0.861478909 | 1.931776737 | 0.053387069 |
| BOP1 | ENST00000607476.1 | ENSG00000272540.1 | 0.967285476 | 2.140077005 | 0.032348544 |
| BOP1 | ENST00000607943.1 | ENSG00000273188.1 | 0.939116908 | 2.110111815 | 0.034848726 |
| BOP1 | ENST00000608367.1 | ENSG00000273361.1 | 0.893466674 | 2.011860613 | 0.044234632 |
| BOP1 | ENST00000608489.1 | ENSG00000272716.1 | 0.831053545 | 1.857066169 | 0.063301741 |
| BOP1 | ENST00000608677.1 | ENSG00000273350.1 | 0.917212422 | 2.047643827 | 0.040594912 |
| BOP1 | NR_026802.1 | FAM74A4 | 0.911864469 | 2.039833518 | 0.04136691 |
| BOP1 | NR_026813.1 | LINC00597 | -0.902497255 | -2.024671557 | 0.042901101 |
| BOP1 | NR_026951.1 | LINC00324 | 0.897588262 | 1.990638505 | 0.046520644 |
| BOP1 | NR_027052.1 | THAP7-AS1 | 0.93994321 | 2.104027214 | 0.035376073 |
| BOP1 | NR_027271.1 | CIRBP-AS1 | 0.862443475 | 1.920819624 | 0.054754452 |
| BOP1 | NR_036658.1 | ZFAS1 | 0.800305524 | 1.771471878 | 0.076482265 |
| BOP1 | NR_037169.1 | LOC100507547 | 0.89422765 | 2.022907101 | 0.043082726 |
| BOP1 | NR_037170.1 | LOC100507547 | 0.857645917 | 1.932702852 | 0.053272814 |
| BOP1 | NR_038421.1 | LINC01220 | 0.922897303 | 2.075457846 | 0.037944122 |
| BOP1 | NR_038923.1 | SSSCA1-AS1 | 0.973740496 | 2.188788171 | 0.02861224 |
| BOP1 | NR_045114.1 | PVRL3-AS1 | -0.898083291 | -2.007405905 | 0.044706461 |

| | | | | | |
|---|---|---|---|---|---|
| BOP1 | NR_046871.1 | LINC00333 | -0.87185596 | -1.958433922 | 0.050179118 |
| BOP1 | NR_047116.1 | HIF1A-AS1 | -0.895993105 | -1.997638967 | 0.045755815 |
| BOP1 | NR_072981.1 | LINC00957 | 0.87709201 | 1.955635499 | 0.050508108 |
| BOP1 | NR_072982.1 | LINC00957 | 0.871875438 | 1.946558807 | 0.051587651 |
| BOP1 | NR_105010.1 | LINC01333 | 0.845334902 | 1.885274707 | 0.059392759 |
| BOP1 | NR_108106.1 | LINC01135 | 0.892344503 | 1.989927728 | 0.046598897 |
| BOP1 | NR_109831.1 | RASSF1-AS1 | 0.902552785 | 2.00939923 | 0.044494812 |
| BOP1 | NR_109885.1 | RALY-AS1 | 0.898568755 | 1.991505173 | 0.046425377 |
| BOP1 | NR_109886.1 | RALY-AS1 | 0.895979074 | 1.992012141 | 0.046369726 |
| BOP1 | NR_110245.1 | LOC101929282 | -0.901606866 | -2.025257414 | 0.042840939 |
| BOP1 | NR_110556.1 | LOC102724890 | -0.818369166 | -1.796523287 | 0.072411333 |
| BOP1 | NR_110630.1 | LOC101927478 | 0.89965236 | 2.031496148 | 0.04220469 |
| BOP1 | NR_110941.1 | MIR762HG | 0.955420894 | 2.125345098 | 0.033557828 |
| BOP1 | NR_110998.1 | FAM74A4 | 0.911864469 | 2.042959378 | 0.04105646 |
| BOP1 | NR_111951.1 | LINC00869 | 0.907474797 | 2.040213966 | 0.041329019 |
| BOP1 | NR_111952.1 | LINC00869 | 0.908637071 | 2.024980717 | 0.042869344 |
| BOP1 | NR_111953.1 | LINC00869 | 0.903086616 | 2.032712354 | 0.042081592 |
| BOP1 | NR_125849.1 | LOC101928140 | -0.839351012 | -1.889815167 | 0.058782684 |
| BOP1 | NR_125957.1 | LOC101928626 | -0.84986619 | -1.886248393 | 0.05926149 |
| BOP1 | NR_126522.1 | EXOC3-AS1 | 0.916292702 | 2.054507154 | 0.039926632 |
| BOP1 | NR_130143.1 | LOC104968399 | 0.908295786 | 2.031549178 | 0.042199316 |
| BOP1 | NR_134520.1 | LOC727993 | 0.885025439 | 1.979879136 | 0.047717111 |
| BOP1 | NR_135024.1 | LOC105369747 | 0.915506176 | 2.045908885 | 0.040765336 |
| BOP1 | NR_135041.1 | LOC101927038 | 0.808761135 | 1.790625462 | 0.073353418 |
| BOP1 | NR_135097.1 | LOC105369443 | -0.850904042 | -1.915342532 | 0.055448835 |
| BOP1 | NR_135584.1 | LOC101927596 | 0.835260791 | 1.86127165 | 0.062705825 |
| BOP1 | NR_136215.1 | VCAN-AS1 | -0.907949612 | -2.03281729 | 0.042070986 |
| C6orf15 | ENST00000318291.4 | ENSG00000177406.4 | 0.931868645 | 2.065328827 | 0.038891898 |
| C6orf15 | ENST00000412759.1 | ENSG00000236933.1 | 0.927843907 | 2.072173733 | 0.038249242 |
| C6orf15 | ENST00000416329.1 | ENSG00000233184.2 | 0.804245564 | 1.775552345 | 0.075806731 |
| C6orf15 | ENST00000417260.1 | ENSG00000231734.4 | -0.841439755 | -1.903842485 | 0.056930701 |
| C6orf15 | ENST00000419662.1 | ENSG00000228265.1 | 0.924978043 | 2.079907156 | 0.03753405 |
| C6orf15 | ENST00000421020.1 | ENSG00000231407.1 | 0.86452487 | 1.928730581 | 0.053764318 |
| C6orf15 | ENST00000421207.1 | ENSG00000231768.1 | 0.906342963 | 2.029439891 | 0.042413506 |
| C6orf15 | ENST00000423428.1 | ENSG00000224048.1 | -0.845375573 | -1.887528835 | 0.059089232 |
| C6orf15 | ENST00000423869.1 | ENSG00000227848.1 | 0.812958618 | 1.826521555 | 0.067771737 |
| C6orf15 | ENST00000425124.1 | ENSG00000232336.1 | 0.903937471 | 2.023916501 | 0.042978744 |
| C6orf15 | ENST00000426237.2 | ENSG00000235527.2 | 0.932999319 | 2.064879441 | 0.038934409 |
| C6orf15 | ENST00000426519.1 | ENSG00000234142.1 | 0.905111548 | 2.043571401 | 0.040995907 |
| C6orf15 | ENST00000433051.1 | ENSG00000233193.1 | 0.801282708 | 1.808081734 | 0.070593781 |
| C6orf15 | ENST00000433905.2 | ENSG00000229299.2 | 0.882659265 | 1.973665341 | 0.048419809 |
| C6orf15 | ENST00000435434.1 | ENSG00000231233.1 | 0.881473758 | 1.957500664 | 0.050288634 |
| C6orf15 | ENST00000435892.1 | ENSG00000233635.2 | 0.836594327 | 1.879779449 | 0.060138142 |
| C6orf15 | ENST00000435992.2 | ENSG00000232675.3 | 0.823911981 | 1.84792821 | 0.064612729 |
| C6orf15 | ENST00000438190.1 | ENSG00000227214.2 | 0.994308503 | 2.251608986 | 0.024346993 |
| C6orf15 | ENST00000440595.1 | ENSG00000228265.1 | 0.808441868 | 1.792525438 | 0.073048838 |
| C6orf15 | ENST00000447343.2 | ENSG00000229299.2 | 0.856573422 | 1.911172942 | 0.05598236 |
| C6orf15 | ENST00000451507.1 | ENSG00000229539.1 | 0.801811538 | 1.789729404 | 0.073497423 |
| C6orf15 | ENST00000452176.1 | ENSG00000223659.1 | -0.913710438 | -2.024107095 | 0.042959134 |
| C6orf15 | ENST00000453051.1 | ENSG00000229407.1 | 0.847766329 | 1.874666955 | 0.060838556 |
| C6orf15 | ENST00000457253.1 | ENSG00000225173.1 | 0.811152014 | 1.808895035 | 0.070467312 |

| | | | | | |
|---|---|---|---|---|---|
| C6orf15 | ENST00000458154.1 | ENSG00000235578.1 | 0.963273962 | 2.118047151 | 0.034171075 |
| C6orf15 | ENST00000458194.1 | ENSG00000226193.1 | 0.880198514 | 1.992205779 | 0.046348484 |
| C6orf15 | ENST00000458364.1 | ENSG00000225655.1 | -0.842796458 | -1.881247522 | 0.059938256 |
| C6orf15 | ENST00000459985.1 | ENSG00000273066.1 | 0.871725385 | 1.943925139 | 0.051904479 |
| C6orf15 | ENST00000463255.1 | ENSG00000243305.1 | -0.802901772 | -1.820573534 | 0.068671709 |
| C6orf15 | ENST00000468165.1 | ENSG00000239480.1 | 0.830558862 | 1.861401959 | 0.062687434 |
| C6orf15 | ENST00000484413.1 | ENSG00000271853.1 | 0.815911392 | 1.838650761 | 0.06596657 |
| C6orf15 | ENST00000489077.1 | ENSG00000244198.1 | 0.979142675 | 2.205881748 | 0.027392281 |
| C6orf15 | ENST00000489557.2 | ENSG00000257045.1 | 0.81623383 | 1.816162837 | 0.069345397 |
| C6orf15 | ENST00000494509.1 | ENSG00000240095.1 | 0.801299598 | 1.777396423 | 0.075503039 |
| C6orf15 | ENST00000498693.1 | ENSG00000244198.1 | 0.982164946 | 2.211559956 | 0.026997085 |
| C6orf15 | ENST00000503723.1 | ENSG00000250472.1 | -0.882572515 | -1.979825146 | 0.04772318 |
| C6orf15 | ENST00000505556.1 | ENSG00000249409.1 | 0.887321572 | 2.012114055 | 0.044207915 |
| C6orf15 | ENST00000506100.1 | ENSG00000249409.1 | 0.879546886 | 1.996860547 | 0.045840334 |
| C6orf15 | ENST00000506791.1 | ENSG00000251131.1 | 0.983422592 | 2.216863817 | 0.026632399 |
| C6orf15 | ENST00000508083.1 | ENSG00000249343.1 | 0.929027715 | 2.111663201 | 0.034715347 |
| C6orf15 | ENST00000509036.1 | ENSG00000251131.1 | 0.96155829 | 2.132268649 | 0.032984768 |
| C6orf15 | ENST00000509192.1 | ENSG00000250765.1 | 0.986224781 | 2.216534487 | 0.026654919 |
| C6orf15 | ENST00000509453.1 | ENSG00000249145.1 | 0.837980089 | 1.84994901 | 0.064320899 |
| C6orf15 | ENST00000510570.1 | ENSG00000250438.1 | -0.817676031 | -1.821166484 | 0.068581553 |
| C6orf15 | ENST00000515128.1 | ENSG00000248215.1 | -0.885931965 | -1.977452679 | 0.047990484 |
| C6orf15 | ENST00000520603.1 | ENSG00000254001.1 | -0.974477113 | -2.18328096 | 0.029015126 |
| C6orf15 | ENST00000522547.1 | ENSG00000253430.1 | -0.845485972 | -1.892350177 | 0.05844434 |
| C6orf15 | ENST00000522600.1 | ENSG00000246582.2 | 0.802958108 | 1.790175349 | 0.073425727 |
| C6orf15 | ENST00000528887.1 | ENSG00000254501.1 | 0.83235496 | 1.875109441 | 0.060777669 |
| C6orf15 | ENST00000543072.1 | ENSG00000256092.2 | -0.877501736 | -1.973519334 | 0.048436424 |
| C6orf15 | ENST00000543275.1 | ENSG00000256944.1 | 0.868114091 | 1.94146392 | 0.052202032 |
| C6orf15 | ENST00000545177.3 | ENSG00000230438.5 | 0.848464737 | 1.907250467 | 0.056488161 |
| C6orf15 | ENST00000548722.2 | ENSG00000257194.2 | -0.850381267 | -1.912743443 | 0.055780905 |
| C6orf15 | ENST00000550263.1 | ENSG00000257605.1 | 0.834391264 | 1.865926283 | 0.06205168 |
| C6orf15 | ENST00000563018.1 | ENSG00000260193.1 | 0.820716685 | 1.837957834 | 0.066068619 |
| C6orf15 | ENST00000563611.1 | ENSG00000261583.1 | 0.904082223 | 2.024529137 | 0.042915737 |
| C6orf15 | ENST00000565823.1 | ENSG00000260686.1 | -0.849932285 | -1.928880634 | 0.053745683 |
| C6orf15 | ENST00000565829.1 | ENSG00000260148.1 | 0.82686325 | 1.855351567 | 0.063546039 |
| C6orf15 | ENST00000566170.1 | ENSG00000261071.1 | 0.820329994 | 1.829123158 | 0.06738116 |
| C6orf15 | ENST00000567395.1 | ENSG00000261090.1 | 0.84124088 | 1.864715415 | 0.062221305 |
| C6orf15 | ENST00000568033.1 | ENSG00000261480.1 | 0.825460283 | 1.837138141 | 0.066189505 |
| C6orf15 | ENST00000569981.1 | ENSG00000238045.5 | 0.909193222 | 2.006906046 | 0.044759668 |
| C6orf15 | ENST00000570493.2 | ENSG00000261898.2 | 0.916202716 | 2.039233483 | 0.041426731 |
| C6orf15 | ENST00000570843.1 | ENSG00000261889.1 | 0.903814946 | 2.005666492 | 0.044891843 |
| C6orf15 | ENST00000570929.1 | ENSG00000262223.2 | 0.908880997 | 2.041338483 | 0.041217194 |
| C6orf15 | ENST00000578800.1 | ENSG00000264235.1 | 0.847330095 | 1.903327476 | 0.056997828 |
| C6orf15 | ENST00000582044.1 | ENSG00000263715.2 | 0.861645708 | 1.944148138 | 0.05187759 |
| C6orf15 | ENST00000582558.1 | ENSG00000264569.1 | 0.932897922 | 2.07320079 | 0.038153597 |
| C6orf15 | ENST00000584705.1 | ENSG00000264569.1 | 0.837586258 | 1.858925499 | 0.063037699 |
| C6orf15 | ENST00000585559.1 | ENSG00000267117.1 | 0.912842278 | 2.061396652 | 0.039265214 |
| C6orf15 | ENST00000586051.1 | ENSG00000267576.1 | 0.808602431 | 1.81664868 | 0.069270925 |
| C6orf15 | ENST00000586694.1 | ENSG00000267141.1 | 0.851238749 | 1.90818077 | 0.056367856 |
| C6orf15 | ENST00000588380.1 | ENSG00000266990.1 | 0.885758816 | 1.985738239 | 0.047062397 |
| C6orf15 | ENST00000591174.1 | ENSG00000267289.1 | 0.929682284 | 2.062838289 | 0.039127995 |
| C6orf15 | ENST00000592400.1 | ENSG00000267735.1 | 0.937841208 | 2.109117881 | 0.034934408 |

| | | | | | |
|---|---|---|---|---|---|
| C6orf15 | ENST00000594590.2 | ENSG00000268199.2 | 0.903838585 | 2.023257384 | 0.043046618 |
| C6orf15 | ENST00000595478.1 | ENSG00000237031.3 | -0.86746591 | -1.935288953 | 0.052954847 |
| C6orf15 | ENST00000596091.1 | ENSG00000227733.4 | -0.828389217 | -1.828435202 | 0.067484262 |
| C6orf15 | ENST00000597169.1 | ENSG00000269720.1 | 0.964012022 | 2.192302167 | 0.028357695 |
| C6orf15 | ENST00000598092.1 | ENSG00000228065.6 | -0.827454722 | -1.859771575 | 0.062917851 |
| C6orf15 | ENST00000599259.1 | ENSG00000269352.1 | 0.889694444 | 2.009159298 | 0.044520243 |
| C6orf15 | ENST00000600534.1 | ENSG00000267858.1 | 0.832415495 | 1.844638158 | 0.065090192 |
| C6orf15 | ENST00000600726.1 | ENSG00000267858.1 | 0.942911618 | 2.090439894 | 0.036578302 |
| C6orf15 | ENST00000601692.1 | ENSG00000267874.1 | -0.81685183 | -1.810489503 | 0.070219911 |
| C6orf15 | ENST00000601735.1 | ENSG00000244513.2 | 0.894692692 | 1.992764985 | 0.046287188 |
| C6orf15 | ENST00000602532.1 | ENSG00000270091.1 | 0.800482269 | 1.806283738 | 0.070874031 |
| C6orf15 | ENST00000602594.1 | ENSG00000269930.1 | -0.860782246 | -1.932995236 | 0.053236785 |
| C6orf15 | ENST00000602949.1 | ENSG00000270030.1 | 0.814065779 | 1.812422988 | 0.069920863 |
| C6orf15 | ENST00000604142.1 | ENSG00000271308.1 | 0.992111468 | 2.215974065 | 0.026693278 |
| C6orf15 | ENST00000604183.1 | ENSG00000271185.1 | 0.830038615 | 1.844674725 | 0.06508487 |
| C6orf15 | ENST00000606277.1 | ENSG00000272145.1 | 0.925732634 | 2.071121913 | 0.038347405 |
| C6orf15 | ENST00000606377.1 | ENSG00000272286.1 | -0.908197293 | -2.016906885 | 0.043705231 |
| C6orf15 | ENST00000606470.1 | ENSG00000271913.1 | 0.88803692 | 1.985434699 | 0.047096129 |
| C6orf15 | ENST00000606743.1 | ENSG00000272221.1 | 0.872217659 | 1.958669431 | 0.050151513 |
| C6orf15 | ENST00000606909.1 | ENSG00000271821.1 | 0.865820804 | 1.962167153 | 0.049743027 |
| C6orf15 | ENST00000607224.1 | ENSG00000272521.1 | 0.875513926 | 1.918374552 | 0.055063535 |
| C6orf15 | ENST00000607476.1 | ENSG00000272540.1 | 0.977144726 | 2.166689991 | 0.0302585 |
| C6orf15 | ENST00000607943.1 | ENSG00000273188.1 | 0.963360621 | 2.139746638 | 0.032375248 |
| C6orf15 | ENST00000608489.1 | ENSG00000272716.1 | 0.835750018 | 1.853407753 | 0.063823937 |
| C6orf15 | ENST00000608677.1 | ENSG00000273350.1 | 0.805667955 | 1.814162655 | 0.069652688 |
| C6orf15 | ENST00000610145.1 | ENSG00000273175.1 | 0.820510774 | 1.827385696 | 0.067641798 |
| C6orf15 | NR_003604.2 | ZFAS1 | 0.933428739 | 2.091432898 | 0.036489276 |
| C6orf15 | NR_003605.1 | ZFAS1 | 0.967632188 | 2.151765224 | 0.03141585 |
| C6orf15 | NR_003606.2 | ZFAS1 | 0.932640584 | 2.098585473 | 0.035853458 |
| C6orf15 | NR_026802.1 | FAM74A4 | 0.935662472 | 2.089944209 | 0.036622812 |
| C6orf15 | NR_026951.1 | LINC00324 | 0.819666938 | 1.816872331 | 0.069236664 |
| C6orf15 | NR_027052.1 | THAP7-AS1 | 0.826758335 | 1.855643436 | 0.063504398 |
| C6orf15 | NR_027271.1 | CIRBP-AS1 | 0.957563259 | 2.120753157 | 0.033942581 |
| C6orf15 | NR_027334.2 | MZF1-AS1 | 0.849508424 | 1.926434706 | 0.054050117 |
| C6orf15 | NR_036480.1 | VPS9D1-AS1 | 0.899185371 | 2.004560696 | 0.045010032 |
| C6orf15 | NR_036658.1 | ZFAS1 | 0.93771363 | 2.086822918 | 0.036904145 |
| C6orf15 | NR_037169.1 | LOC100507547 | 0.932120142 | 2.0980616 | 0.035899704 |
| C6orf15 | NR_037170.1 | LOC100507547 | 0.895616118 | 2.021693011 | 0.043208076 |
| C6orf15 | NR_038421.1 | LINC01220 | 0.885251381 | 1.965580558 | 0.049347082 |
| C6orf15 | NR_038923.1 | SSSCA1-AS1 | 0.840698905 | 1.889600684 | 0.058811386 |
| C6orf15 | NR_044996.1 | HCG23 | 0.804597313 | 1.799530205 | 0.07193485 |
| C6orf15 | NR_045114.1 | PVRL3-AS1 | -0.944595475 | -2.112881169 | 0.03461094 |
| C6orf15 | NR_072981.1 | LINC00957 | 0.968415688 | 2.136142233 | 0.032667822 |
| C6orf15 | NR_072982.1 | LINC00957 | 0.965461249 | 2.161866575 | 0.030628462 |
| C6orf15 | NR_103790.1 | LINC00581 | -0.800441313 | -1.775560228 | 0.07580543 |
| C6orf15 | NR_105010.1 | LINC01333 | 0.977978309 | 2.169875328 | 0.030016292 |
| C6orf15 | NR_108036.1 | CFAP58-AS1 | 0.886521354 | 1.962959242 | 0.04965091 |
| C6orf15 | NR_109885.1 | RALY-AS1 | 0.836103862 | 1.87215646 | 0.061184961 |
| C6orf15 | NR_109886.1 | RALY-AS1 | 0.94310732 | 2.094430249 | 0.036221671 |
| C6orf15 | NR_110245.1 | LOC101929282 | -0.824902569 | -1.849644452 | 0.064364812 |
| C6orf15 | NR_110630.1 | LOC101927478 | 0.84851044 | 1.886876666 | 0.059176916 |

| | | | | | |
|---|---|---|---|---|---|
| C6orf15 | NR_110941.1 | MIR762HG | 0.879930605 | 1.985606648 | 0.047077018 |
| C6orf15 | NR_110998.1 | FAM74A4 | 0.935662472 | 2.078091007 | 0.037700978 |
| C6orf15 | NR_111952.1 | LINC00869 | 0.808477137 | 1.815117167 | 0.069505905 |
| C6orf15 | NR_121189.1 | PGM5P3-AS1 | -0.870192561 | -1.942532941 | 0.052072616 |
| C6orf15 | NR_125957.1 | LOC101928626 | -0.913710438 | -2.04214571 | 0.04113708 |
| C6orf15 | NR_126522.1 | EXOC3-AS1 | 0.912018824 | 2.035881133 | 0.041762293 |
| C6orf15 | NR_130143.1 | LOC104968399 | 0.866780987 | 1.933780661 | 0.053140101 |
| C6orf15 | NR_135024.1 | LOC105369747 | 0.857884815 | 1.902349547 | 0.057125472 |
| C6orf15 | NR_135584.1 | LOC101927596 | 0.853545173 | 1.91875956 | 0.05501477 |
| C6orf15 | NR_144459.1 | ARSD-AS1 | 0.878433198 | 1.960294858 | 0.049961337 |
| CCDC106 | ENST00000413650.1 | ENSG00000230880.2 | 0.81633816 | 1.820363571 | 0.068703656 |
| CCDC106 | ENST00000413991.1 | ENSG00000237614.1 | 0.918720094 | 2.044117966 | 0.040941895 |
| CCDC106 | ENST00000414740.2 | ENSG00000229646.2 | 0.832600382 | 1.879202743 | 0.060216815 |
| CCDC106 | ENST00000416657.1 | ENSG00000235858.1 | 0.914275321 | 2.027632922 | 0.042597726 |
| CCDC106 | ENST00000418972.1 | ENSG00000225044.1 | -0.81866129 | -1.829995957 | 0.067250543 |
| CCDC106 | ENST00000419863.1 | ENSG00000238282.1 | -0.801665425 | -1.792820074 | 0.073001699 |
| CCDC106 | ENST00000420365.1 | ENSG00000225214.1 | 0.903717062 | 2.005382752 | 0.044922144 |
| CCDC106 | ENST00000420981.2 | ENSG00000230438.5 | 0.819619495 | 1.827711128 | 0.067592917 |
| CCDC106 | ENST00000422038.1 | ENSG00000227935.1 | 0.848693012 | 1.905209298 | 0.05675287 |
| CCDC106 | ENST00000424181.1 | ENSG00000224977.1 | 0.825985258 | 1.832406414 | 0.066890893 |
| CCDC106 | ENST00000425371.2 | ENSG00000235872.2 | 0.911691237 | 2.008248843 | 0.044616855 |
| CCDC106 | ENST00000427691.1 | ENSG00000228340.1 | -0.820445914 | -1.839861378 | 0.065788591 |
| CCDC106 | ENST00000429608.1 | ENSG00000237480.1 | 0.907498172 | 2.035945258 | 0.041755853 |
| CCDC106 | ENST00000431290.1 | ENSG00000183822.2 | 0.848554292 | 1.925247774 | 0.054198368 |
| CCDC106 | ENST00000434627.1 | ENSG00000230074.1 | 0.869564343 | 1.943910582 | 0.051906235 |
| CCDC106 | ENST00000435733.1 | ENSG00000226377.1 | 0.84888941 | 1.89671726 | 0.057865264 |
| CCDC106 | ENST00000437330.1 | ENSG00000229203.1 | 0.883378766 | 1.983181305 | 0.047347181 |
| CCDC106 | ENST00000437461.1 | ENSG00000227200.1 | 0.88750629 | 1.97621114 | 0.048130869 |
| CCDC106 | ENST00000442069.1 | ENSG00000225655.1 | -0.905255446 | -2.033064538 | 0.042046003 |
| CCDC106 | ENST00000442850.1 | ENSG00000232600.2 | -0.806353493 | -1.792147758 | 0.0731093 |
| CCDC106 | ENST00000443380.1 | ENSG00000224371.1 | 0.894413752 | 2.014126806 | 0.043996222 |
| CCDC106 | ENST00000447206.1 | ENSG00000230839.1 | 0.80606101 | 1.787757807 | 0.073815091 |
| CCDC106 | ENST00000450109.1 | ENSG00000225376.1 | 0.892938026 | 1.998887738 | 0.045620502 |
| CCDC106 | ENST00000450531.1 | ENSG00000229536.1 | 0.875811993 | 1.961758145 | 0.049790649 |
| CCDC106 | ENST00000452002.1 | ENSG00000236501.1 | -0.809856024 | -1.799090704 | 0.072004334 |
| CCDC106 | ENST00000454530.1 | ENSG00000226649.1 | -0.863227121 | -1.928996102 | 0.053731346 |
| CCDC106 | ENST00000457115.1 | ENSG00000227245.1 | 0.941980504 | 2.10683418 | 0.035131956 |
| CCDC106 | ENST00000457848.1 | ENSG00000226412.1 | 0.846891673 | 1.886917037 | 0.059171485 |
| CCDC106 | ENST00000458194.1 | ENSG00000226193.1 | 0.845539232 | 1.886130656 | 0.05927735 |
| CCDC106 | ENST00000458364.1 | ENSG00000225655.1 | -0.817221669 | -1.832306979 | 0.066905698 |
| CCDC106 | ENST00000459985.1 | ENSG00000273066.1 | 0.870685991 | 1.949738934 | 0.051207243 |
| CCDC106 | ENST00000466431.2 | ENSG00000254485.1 | 0.83139315 | 1.841202631 | 0.06559187 |
| CCDC106 | ENST00000498358.1 | ENSG00000184115.12 | 0.808190997 | 1.817997862 | 0.06906446 |
| CCDC106 | ENST00000505196.1 | ENSG00000248131.1 | 0.860264033 | 1.928493508 | 0.053793771 |
| CCDC106 | ENST00000508188.1 | ENSG00000250999.1 | 0.890269415 | 1.976990601 | 0.048042693 |
| CCDC106 | ENST00000508241.1 | ENSG00000248518.1 | 0.877685664 | 1.952597276 | 0.050867334 |
| CCDC106 | ENST00000517300.1 | ENSG00000254144.2 | 0.885319727 | 1.977671822 | 0.047965741 |
| CCDC106 | ENST00000519189.1 | ENSG00000254344.1 | -0.885083249 | -1.949620474 | 0.051221371 |
| CCDC106 | ENST00000519451.1 | ENSG00000253363.1 | 0.926986404 | 2.058086384 | 0.039581842 |
| CCDC106 | ENST00000519852.1 | ENSG00000253716.1 | 0.954018954 | 2.125944119 | 0.033507913 |
| CCDC106 | ENST00000521207.1 | ENSG00000253716.1 | 0.945564621 | 2.094011428 | 0.036258962 |

| | | | | | |
|---|---|---|---|---|---|
| CCDC106 | ENST00000522600.1 | ENSG00000246582.2 | 0.902441782 | 2.029977295 | 0.042358847 |
| CCDC106 | ENST00000524073.1 | ENSG00000253774.1 | 0.820065221 | 1.835033643 | 0.066500705 |
| CCDC106 | ENST00000524335.1 | ENSG00000253716.1 | 0.937951694 | 2.119487586 | 0.034049282 |
| CCDC106 | ENST00000526154.1 | ENSG00000254511.1 | 0.894126106 | 1.975552579 | 0.048205474 |
| CCDC106 | ENST00000526611.1 | ENSG00000246982.2 | 0.897087707 | 1.987269962 | 0.046892489 |
| CCDC106 | ENST00000526694.1 | ENSG00000231999.2 | 0.890314158 | 1.969292965 | 0.048919459 |
| CCDC106 | ENST00000528000.1 | ENSG00000254804.1 | 0.818267185 | 1.785640055 | 0.074157557 |
| CCDC106 | ENST00000531627.1 | ENSG00000254584.1 | 0.821624933 | 1.851040325 | 0.064163752 |
| CCDC106 | ENST00000532688.1 | ENSG00000255441.1 | 0.922153054 | 2.089772176 | 0.03663827 |
| CCDC106 | ENST00000537269.1 | ENSG00000257084.1 | 0.91809529 | 2.033236188 | 0.042028667 |
| CCDC106 | ENST00000537921.1 | ENSG00000255966.1 | 0.883785941 | 1.984631917 | 0.047185439 |
| CCDC106 | ENST00000543072.1 | ENSG00000256092.2 | -0.802586254 | -1.810492088 | 0.07021951 |
| CCDC106 | ENST00000547750.1 | ENSG00000257886.1 | 0.89169203 | 2.012003382 | 0.04421958 |
| CCDC106 | ENST00000549878.1 | ENSG00000257284.1 | 0.930063212 | 2.085735386 | 0.037002599 |
| CCDC106 | ENST00000554049.1 | ENSG00000258763.1 | 0.832603581 | 1.866257442 | 0.062005356 |
| CCDC106 | ENST00000554430.1 | ENSG00000258646.1 | -0.818507842 | -1.823709363 | 0.068196023 |
| CCDC106 | ENST00000558515.1 | ENSG00000259182.1 | 0.873506746 | 1.958150904 | 0.050212308 |
| CCDC106 | ENST00000558875.1 | ENSG00000259737.2 | 0.810054276 | 1.835115283 | 0.06648861 |
| CCDC106 | ENST00000559569.1 | ENSG00000259760.1 | -0.807621118 | -1.812240618 | 0.069949026 |
| CCDC106 | ENST00000560522.1 | ENSG00000259661.1 | 0.913142027 | 2.046376292 | 0.040719363 |
| CCDC106 | ENST00000563044.1 | ENSG00000260978.1 | 0.875112372 | 1.932763208 | 0.053265375 |
| CCDC106 | ENST00000563806.1 | ENSG00000238045.5 | 0.824540131 | 1.853333217 | 0.063834613 |
| CCDC106 | ENST00000564102.1 | ENSG00000260041.1 | -0.8506735 | -1.891594108 | 0.058545081 |
| CCDC106 | ENST00000565359.1 | ENSG00000260601.1 | 0.855738946 | 1.921836621 | 0.054626319 |
| CCDC106 | ENST00000565823.1 | ENSG00000260686.1 | -0.89393465 | -2.003869966 | 0.045083992 |
| CCDC106 | ENST00000565829.1 | ENSG00000260148.1 | 0.892605947 | 2.006195676 | 0.044835375 |
| CCDC106 | ENST00000567127.1 | ENSG00000260264.1 | -0.80690926 | -1.832788795 | 0.066833986 |
| CCDC106 | ENST00000570843.1 | ENSG00000261889.1 | 0.80068184 | 1.758564943 | 0.078651432 |
| CCDC106 | ENST00000570929.1 | ENSG00000262223.2 | 0.807961578 | 1.822508684 | 0.068377837 |
| CCDC106 | ENST00000571815.1 | ENSG00000262810.1 | 0.869339531 | 1.923307525 | 0.05444144 |
| CCDC106 | ENST00000577064.1 | ENSG00000262823.1 | 0.90153113 | 2.012389839 | 0.044178859 |
| CCDC106 | ENST00000577176.1 | ENSG00000262823.1 | 0.876600078 | 1.970164951 | 0.048819469 |
| CCDC106 | ENST00000578757.1 | ENSG00000175061.13 | 0.800221153 | 1.803330585 | 0.071336312 |
| CCDC106 | ENST00000578936.1 | ENSG00000265547.1 | 0.844938724 | 1.862387044 | 0.062548554 |
| CCDC106 | ENST00000583122.1 | ENSG00000264695.1 | -0.852782871 | -1.880024503 | 0.060104739 |
| CCDC106 | ENST00000583916.1 | ENSG00000264196.1 | 0.848832175 | 1.897388124 | 0.057776732 |
| CCDC106 | ENST00000589233.1 | ENSG00000231616.4 | 0.974527655 | 2.191630206 | 0.028406219 |
| CCDC106 | ENST00000589817.1 | ENSG00000231616.4 | 0.936098833 | 2.080273233 | 0.037500479 |
| CCDC106 | ENST00000590328.1 | ENSG00000256995.2 | -0.837665544 | -1.890501366 | 0.058690938 |
| CCDC106 | ENST00000590368.1 | ENSG00000231616.4 | 0.884198273 | 1.961631064 | 0.049805453 |
| CCDC106 | ENST00000590813.1 | ENSG00000231616.4 | 0.896791537 | 2.019480157 | 0.043437338 |
| CCDC106 | ENST00000590995.1 | ENSG00000267198.1 | 0.801136875 | 1.80686197 | 0.070783803 |
| CCDC106 | ENST00000591103.1 | ENSG00000272895.1 | 0.866816087 | 1.955264337 | 0.050551878 |
| CCDC106 | ENST00000591836.1 | ENSG00000267776.1 | 0.829247276 | 1.866168723 | 0.062017764 |
| CCDC106 | ENST00000593269.1 | ENSG00000236172.2 | 0.823548051 | 1.842850615 | 0.065350824 |
| CCDC106 | ENST00000593632.1 | ENSG00000180279.5 | 0.89494027 | 2.010374266 | 0.044391592 |
| CCDC106 | ENST00000593642.1 | ENSG00000267858.1 | 0.919663746 | 2.082036205 | 0.037339163 |
| CCDC106 | ENST00000594492.1 | ENSG00000250910.3 | 0.853790172 | 1.915538479 | 0.055423867 |
| CCDC106 | ENST00000597309.1 | ENSG00000232098.2 | -0.893508909 | -1.97784677 | 0.047945995 |
| CCDC106 | ENST00000600071.1 | ENSG00000269199.1 | 0.800336692 | 1.831600758 | 0.067010924 |
| CCDC106 | ENST00000600534.1 | ENSG00000267858.1 | 0.88991256 | 2.018071872 | 0.043583777 |

| | | | | | |
|---|---|---|---|---|---|
| CCDC106 | ENST00000600889.1 | ENSG00000232675.3 | 0.869835873 | 1.926975946 | 0.053982628 |
| CCDC106 | ENST00000602532.1 | ENSG00000270091.1 | 0.845389284 | 1.915484209 | 0.055430781 |
| CCDC106 | ENST00000606068.1 | ENSG00000272342.1 | 0.886247816 | 1.985429518 | 0.047096705 |
| CCDC106 | ENST00000606841.1 | ENSG00000272411.1 | 0.901780075 | 1.990058145 | 0.046584531 |
| CCDC106 | ENST00000607014.1 | ENSG00000272345.1 | -0.966262482 | -2.1511382 | 0.031465293 |
| CCDC106 | ENST00000607222.1 | ENSG00000272106.1 | 0.807286875 | 1.811257316 | 0.07010103 |
| CCDC106 | ENST00000608940.1 | ENSG00000272763.1 | 0.869263824 | 1.947936657 | 0.051422543 |
| CCDC106 | ENST00000608952.1 | ENSG00000272689.1 | -0.924736725 | -2.069800003 | 0.038471077 |
| CCDC106 | ENST00000610145.1 | ENSG00000273175.1 | 0.840228691 | 1.878027891 | 0.06037735 |
| CCDC106 | ENST00000610185.1 | ENSG00000273355.1 | -0.916603159 | -2.045708491 | 0.04078506 |
| CCDC106 | NR_003604.2 | ZFAS1 | 0.803506414 | 1.806337889 | 0.070865577 |
| CCDC106 | NR_027334.2 | MZF1-AS1 | 0.87009443 | 1.947299182 | 0.051498877 |
| CCDC106 | NR_046454.1 | LINC00907 | -0.825072512 | -1.835232359 | 0.066471268 |
| CCDC106 | NR_103857.1 | SP2-AS1 | -0.845974463 | -1.899830501 | 0.057455367 |
| CCDC106 | NR_104158.1 | NRG1-IT1 | 0.96304365 | 2.152541287 | 0.031354747 |
| CCDC106 | NR_110635.1 | LINC00687 | 0.862194442 | 1.950174158 | 0.051155365 |
| CCDC106 | NR_110919.1 | LOC101928530 | 0.80499029 | 1.795510343 | 0.072572426 |
| CCDC106 | NR_117098.1 | LINC01353 | 0.876199257 | 1.958723671 | 0.050145157 |
| CCDC106 | NR_121188.1 | PGM5P3-AS1 | -0.896364785 | -2.037697514 | 0.041580193 |
| CCDC106 | NR_121189.1 | PGM5P3-AS1 | -0.838456046 | -1.882540699 | 0.059762639 |
| CCDC106 | NR_121661.1 | ZBTB20-AS5 | -0.923695381 | -2.059495704 | 0.039446776 |
| CCDC106 | NR_135032.1 | LOC105369635 | 0.91809529 | 2.04042352 | 0.041308161 |
| CCDC106 | NR_135644.1 | LOC105371506 | -0.902843853 | -1.981256538 | 0.047562512 |
| CCDC106 | NR_135820.1 | LOC102723727 | 0.896869769 | 2.000313354 | 0.045466438 |
| CCDC106 | NR_138038.1 | LINC00677 | 0.858361062 | 1.933698478 | 0.053150211 |
| CCDC96 | ENST00000318291.4 | ENSG00000177406.4 | 0.952332382 | 2.165405512 | 0.030356644 |
| CCDC96 | ENST00000399186.2 | ENSG00000214888.2 | 0.800725607 | 1.781834279 | 0.07477626 |
| CCDC96 | ENST00000412759.1 | ENSG00000236933.1 | 0.836997772 | 1.88978249 | 0.058787056 |
| CCDC96 | ENST00000419662.1 | ENSG00000228265.1 | 0.852539157 | 1.901399359 | 0.057249723 |
| CCDC96 | ENST00000421020.1 | ENSG00000231407.1 | 0.894617121 | 1.989383829 | 0.046658853 |
| CCDC96 | ENST00000421207.1 | ENSG00000231768.1 | 0.927854904 | 2.085972891 | 0.036981079 |
| CCDC96 | ENST00000423667.1 | ENSG00000225970.1 | 0.821956214 | 1.819863412 | 0.068779808 |
| CCDC96 | ENST00000423869.1 | ENSG00000227848.1 | 0.828728781 | 1.848790015 | 0.06448814 |
| CCDC96 | ENST00000425124.1 | ENSG00000232336.1 | 0.911094309 | 2.043123654 | 0.041040199 |
| CCDC96 | ENST00000425624.1 | ENSG00000223779.4 | 0.937641189 | 2.095417876 | 0.036133862 |
| CCDC96 | ENST00000426237.2 | ENSG00000235527.2 | 0.86085374 | 1.935155722 | 0.052971189 |
| CCDC96 | ENST00000426519.1 | ENSG00000234142.1 | 0.880238323 | 1.961944855 | 0.049768905 |
| CCDC96 | ENST00000426699.1 | ENSG00000229308.1 | 0.922102427 | 2.07442919 | 0.038039469 |
| CCDC96 | ENST00000433344.1 | ENSG00000234083.1 | -0.928304969 | -2.067926546 | 0.038646931 |
| CCDC96 | ENST00000433614.1 | ENSG00000228534.1 | -0.826359889 | -1.864955063 | 0.062187703 |
| CCDC96 | ENST00000435434.1 | ENSG00000231233.1 | 0.954939591 | 2.121770246 | 0.033857036 |
| CCDC96 | ENST00000435892.1 | ENSG00000233635.2 | 0.814677129 | 1.811787363 | 0.070019059 |
| CCDC96 | ENST00000435992.2 | ENSG00000232675.3 | 0.945132255 | 2.115139724 | 0.03441804 |
| CCDC96 | ENST00000436982.2 | ENSG00000235335.2 | -0.816168855 | -1.847703544 | 0.064645241 |
| CCDC96 | ENST00000438107.1 | ENSG00000234449.2 | 0.858180645 | 1.910821477 | 0.056027527 |
| CCDC96 | ENST00000438190.1 | ENSG00000227214.2 | 0.929557605 | 2.08427943 | 0.037134757 |
| CCDC96 | ENST00000438222.1 | ENSG00000238034.1 | 0.836926668 | 1.839056895 | 0.065906818 |
| CCDC96 | ENST00000439186.1 | ENSG00000237076.1 | 0.846579174 | 1.918008087 | 0.055109986 |
| CCDC96 | ENST00000442069.1 | ENSG00000225655.1 | -0.857896245 | -1.945255713 | 0.051744209 |
| CCDC96 | ENST00000447206.1 | ENSG00000230839.1 | 0.80231827 | 1.786143102 | 0.074076091 |
| CCDC96 | ENST00000447514.1 | ENSG00000236753.1 | 0.811332372 | 1.834231331 | 0.066619662 |

| | | | | | |
|---|---|---|---|---|---|
| CCDC96 | ENST00000452176.1 | ENSG00000223659.1 | -0.889982126 | -1.977154537 | 0.048024165 |
| CCDC96 | ENST00000455788.1 | ENSG00000236263.1 | 0.861992691 | 1.924975589 | 0.054232412 |
| CCDC96 | ENST00000457115.1 | ENSG00000227245.1 | 0.828574173 | 1.853999382 | 0.063739248 |
| CCDC96 | ENST00000458154.1 | ENSG00000235578.1 | 0.967509029 | 2.114074357 | 0.034508917 |
| CCDC96 | ENST00000458194.1 | ENSG00000226193.1 | 0.834665677 | 1.864788055 | 0.062211118 |
| CCDC96 | ENST00000458364.1 | ENSG00000225655.1 | -0.91621286 | -2.052546775 | 0.040116554 |
| CCDC96 | ENST00000459985.1 | ENSG00000273066.1 | 0.880848834 | 1.965248334 | 0.049385502 |
| CCDC96 | ENST00000484413.1 | ENSG00000271853.1 | 0.936350752 | 2.089917109 | 0.036625246 |
| CCDC96 | ENST00000489077.1 | ENSG00000244198.1 | 0.868348653 | 1.966221285 | 0.049273055 |
| CCDC96 | ENST00000493123.1 | ENSG00000242428.1 | 0.825015225 | 1.847325592 | 0.064699966 |
| CCDC96 | ENST00000494509.1 | ENSG00000240095.1 | 0.884518953 | 1.97143523 | 0.048674115 |
| CCDC96 | ENST00000498693.1 | ENSG00000244198.1 | 0.921317947 | 2.069029309 | 0.038543336 |
| CCDC96 | ENST00000504891.1 | ENSG00000249388.1 | 0.849121512 | 1.875466192 | 0.060728616 |
| CCDC96 | ENST00000505498.1 | ENSG00000250908.1 | 0.900037085 | 2.021060308 | 0.043273523 |
| CCDC96 | ENST00000505556.1 | ENSG00000249409.1 | 0.900363151 | 2.015144839 | 0.043889475 |
| CCDC96 | ENST00000506100.1 | ENSG00000249409.1 | 0.892977862 | 2.005872298 | 0.044869875 |
| CCDC96 | ENST00000506791.1 | ENSG00000251131.1 | 0.943564679 | 2.113925431 | 0.034521637 |
| CCDC96 | ENST00000508083.1 | ENSG00000249343.1 | 0.949955948 | 2.149421365 | 0.031601013 |
| CCDC96 | ENST00000509036.1 | ENSG00000251131.1 | 0.895921935 | 2.016211157 | 0.0437779 |
| CCDC96 | ENST00000509192.1 | ENSG00000250765.1 | 0.94909558 | 2.139399179 | 0.032403353 |
| CCDC96 | ENST00000515128.1 | ENSG00000248215.1 | -0.871343594 | -1.939074742 | 0.052492238 |
| CCDC96 | ENST00000518473.1 | ENSG00000253985.1 | 0.844550958 | 1.882442695 | 0.059775934 |
| CCDC96 | ENST00000520603.1 | ENSG00000254001.1 | -0.907210639 | -2.029428622 | 0.042414652 |
| CCDC96 | ENST00000521307.1 | ENSG00000253177.1 | 0.908578717 | 2.013225172 | 0.044090946 |
| CCDC96 | ENST00000522704.1 | ENSG00000254135.1 | 0.824479877 | 1.846824016 | 0.06477265 |
| CCDC96 | ENST00000524818.1 | ENSG00000254473.1 | 0.833357173 | 1.842698112 | 0.0653731 |
| CCDC96 | ENST00000526186.1 | ENSG00000254510.1 | 0.861166519 | 1.890399468 | 0.058704555 |
| CCDC96 | ENST00000526611.1 | ENSG00000246982.2 | 0.824182713 | 1.832341314 | 0.066900586 |
| CCDC96 | ENST00000526935.1 | ENSG00000255372.1 | 0.842715668 | 1.860434066 | 0.062824139 |
| CCDC96 | ENST00000528887.1 | ENSG00000254501.1 | 0.902000419 | 2.041638238 | 0.041187429 |
| CCDC96 | ENST00000543072.1 | ENSG00000256092.2 | -0.812222595 | -1.831040838 | 0.067094448 |
| CCDC96 | ENST00000547834.1 | ENSG00000258325.1 | 0.905517952 | 2.021084087 | 0.043271061 |
| CCDC96 | ENST00000548722.2 | ENSG00000257194.2 | -0.882976933 | -1.988021569 | 0.046809305 |
| CCDC96 | ENST00000549878.1 | ENSG00000257284.1 | 0.825615449 | 1.841526462 | 0.065544446 |
| CCDC96 | ENST00000550263.1 | ENSG00000257605.1 | 0.807312054 | 1.8290567 | 0.067391115 |
| CCDC96 | ENST00000556786.1 | ENSG00000258525.1 | -0.82133236 | -1.864179471 | 0.062296505 |
| CCDC96 | ENST00000565823.1 | ENSG00000260686.1 | -0.875004357 | -1.93850663 | 0.052561443 |
| CCDC96 | ENST00000565829.1 | ENSG00000260148.1 | 0.831033373 | 1.847678736 | 0.064648832 |
| CCDC96 | ENST00000566170.1 | ENSG00000261071.1 | 0.823713928 | 1.869991882 | 0.061484945 |
| CCDC96 | ENST00000567395.1 | ENSG00000261090.1 | 0.924197835 | 2.060778922 | 0.039324136 |
| CCDC96 | ENST00000568033.1 | ENSG00000261480.1 | 0.848612421 | 1.881255025 | 0.059937236 |
| CCDC96 | ENST00000569981.1 | ENSG00000238045.5 | 0.956359175 | 2.127683205 | 0.033363358 |
| CCDC96 | ENST00000570493.2 | ENSG00000261898.2 | 0.940121788 | 2.101203755 | 0.035623084 |
| CCDC96 | ENST00000570512.1 | ENSG00000262768.1 | 0.916038679 | 2.088771484 | 0.036728299 |
| CCDC96 | ENST00000570843.1 | ENSG00000261889.1 | 0.879715865 | 1.935234009 | 0.052961586 |
| CCDC96 | ENST00000570929.1 | ENSG00000262223.2 | 0.920600323 | 2.032851437 | 0.042067535 |
| CCDC96 | ENST00000574365.1 | ENSG00000262837.1 | 0.802337471 | 1.776377593 | 0.075670702 |
| CCDC96 | ENST00000575139.1 | ENSG00000263072.1 | 0.811823119 | 1.818474158 | 0.068991693 |
| CCDC96 | ENST00000576554.1 | ENSG00000262413.1 | 0.80377145 | 1.799490394 | 0.071941142 |
| CCDC96 | ENST00000577698.1 | ENSG00000265100.1 | 0.83806645 | 1.879436601 | 0.060184903 |
| CCDC96 | ENST00000578265.1 | ENSG00000214719.7 | 0.847472926 | 1.886324989 | 0.059251174 |

| | | | | | |
|---|---|---|---|---|---|
| CCDC96 | ENST00000578757.1 | ENSG00000175061.13 | 0.824896672 | 1.852511406 | 0.063952421 |
| CCDC96 | ENST00000582558.1 | ENSG00000264569.1 | 0.868434189 | 1.943852912 | 0.051913191 |
| CCDC96 | ENST00000584705.1 | ENSG00000264569.1 | 0.846414589 | 1.879380295 | 0.060192585 |
| CCDC96 | ENST00000584758.1 | ENSG00000265356.1 | 0.806039487 | 1.785991921 | 0.074100566 |
| CCDC96 | ENST00000585559.1 | ENSG00000267117.1 | 0.971950822 | 2.195197317 | 0.028149447 |
| CCDC96 | ENST00000586694.1 | ENSG00000267141.1 | 0.819076681 | 1.831071053 | 0.067089939 |
| CCDC96 | ENST00000588380.1 | ENSG00000266990.1 | 0.811290625 | 1.797435792 | 0.072266463 |
| CCDC96 | ENST00000588799.1 | ENSG00000267275.1 | 0.843755082 | 1.865387207 | 0.062127149 |
| CCDC96 | ENST00000588945.1 | ENSG00000267275.1 | 0.833119861 | 1.891349938 | 0.058577647 |
| CCDC96 | ENST00000591174.1 | ENSG00000267289.1 | 0.881256605 | 1.974691038 | 0.048303221 |
| CCDC96 | ENST00000592400.1 | ENSG00000267735.1 | 0.926786338 | 2.07490502 | 0.037995338 |
| CCDC96 | ENST00000593632.1 | ENSG00000180279.5 | 0.80640398 | 1.814821642 | 0.069551323 |
| CCDC96 | ENST00000594590.2 | ENSG00000268199.2 | 0.834562964 | 1.864641612 | 0.062231656 |
| CCDC96 | ENST00000595478.1 | ENSG00000237031.3 | -0.823128186 | -1.828064191 | 0.067539918 |
| CCDC96 | ENST00000597169.1 | ENSG00000269720.1 | 0.932481435 | 2.071706645 | 0.038292807 |
| CCDC96 | ENST00000597309.1 | ENSG00000232098.2 | -0.838231444 | -1.870552843 | 0.061407086 |
| CCDC96 | ENST00000599259.1 | ENSG00000269352.1 | 0.95205508 | 2.139742344 | 0.032375595 |
| CCDC96 | ENST00000600489.1 | ENSG00000231898.4 | 0.810686265 | 1.789253185 | 0.07357405 |
| CCDC96 | ENST00000600534.1 | ENSG00000267858.1 | 0.854629089 | 1.866405038 | 0.061984719 |
| CCDC96 | ENST00000600726.1 | ENSG00000267858.1 | 0.872480381 | 1.93370099 | 0.053149902 |
| CCDC96 | ENST00000601692.1 | ENSG00000267874.1 | -0.918379577 | -2.06209282 | 0.0391989 |
| CCDC96 | ENST00000601735.1 | ENSG00000244513.2 | 0.920481519 | 2.04829037 | 0.040531556 |
| CCDC96 | ENST00000602594.1 | ENSG00000269930.1 | -0.858307711 | -1.904542713 | 0.056839539 |
| CCDC96 | ENST00000602809.1 | ENSG00000270105.1 | -0.878712336 | -1.965179121 | 0.04939351 |
| CCDC96 | ENST00000602872.1 | ENSG00000270067.1 | 0.803136667 | 1.809569655 | 0.070362549 |
| CCDC96 | ENST00000604142.1 | ENSG00000271308.1 | 0.903481349 | 2.03321908 | 0.042030394 |
| CCDC96 | ENST00000606277.1 | ENSG00000272145.1 | 0.846265146 | 1.872504439 | 0.061136848 |
| CCDC96 | ENST00000606377.1 | ENSG00000272286.1 | -0.914554259 | -2.049217138 | 0.040440887 |
| CCDC96 | ENST00000606470.1 | ENSG00000271913.1 | 0.946707576 | 2.128944343 | 0.033258865 |
| CCDC96 | ENST00000606743.1 | ENSG00000272221.1 | 0.812024582 | 1.797765938 | 0.072214107 |
| CCDC96 | ENST00000607284.1 | ENSG00000272389.1 | 0.828847445 | 1.886280613 | 0.05925715 |
| CCDC96 | ENST00000607476.1 | ENSG00000272540.1 | 0.858794533 | 1.917024085 | 0.055234872 |
| CCDC96 | ENST00000607943.1 | ENSG00000273188.1 | 0.863913463 | 1.947052858 | 0.051528398 |
| CCDC96 | ENST00000609972.1 | ENSG00000230651.3 | 0.920400599 | 2.069691295 | 0.038481262 |
| CCDC96 | ENST00000610145.1 | ENSG00000273175.1 | 0.913486535 | 2.054511764 | 0.039926186 |
| CCDC96 | NR_003604.2 | ZFAS1 | 0.909023469 | 2.031042687 | 0.042250665 |
| CCDC96 | NR_003605.1 | ZFAS1 | 0.979553567 | 2.214031512 | 0.026826611 |
| CCDC96 | NR_003606.2 | ZFAS1 | 0.904778957 | 2.030202995 | 0.04233591 |
| CCDC96 | NR_026802.1 | FAM74A4 | 0.855872892 | 1.904925803 | 0.056789717 |
| CCDC96 | NR_027271.1 | CIRBP-AS1 | 0.899924504 | 1.99105137 | 0.04647524 |
| CCDC96 | NR_027334.2 | MZF1-AS1 | 0.876095836 | 1.941542721 | 0.052192483 |
| CCDC96 | NR_028324.1 | LINC01002 | 0.829205492 | 1.851633532 | 0.064078465 |
| CCDC96 | NR_036480.1 | VPS9D1-AS1 | 0.898779096 | 2.00833924 | 0.044607255 |
| CCDC96 | NR_036658.1 | ZFAS1 | 0.912137973 | 2.038558534 | 0.041494107 |
| CCDC96 | NR_037169.1 | LOC100507547 | 0.860104411 | 1.934746995 | 0.05302135 |
| CCDC96 | NR_037170.1 | LOC100507547 | 0.832724514 | 1.874856096 | 0.060812523 |
| CCDC96 | NR_044996.1 | HCG23 | 0.945408448 | 2.115940264 | 0.034349888 |
| CCDC96 | NR_045114.1 | PVRL3-AS1 | -0.832068348 | -1.863966706 | 0.06232638 |
| CCDC96 | NR_072981.1 | LINC00957 | 0.926821653 | 2.072501933 | 0.038218656 |
| CCDC96 | NR_072982.1 | LINC00957 | 0.923859716 | 2.048427844 | 0.040518096 |
| CCDC96 | NR_103790.1 | LINC00581 | -0.800730568 | -1.806355317 | 0.070862856 |

| | | | | | |
|---|---|---|---|---|---|
| CCDC96 | NR_105010.1 | LINC01333 | 0.951423084 | 2.138434789 | 0.032481471 |
| CCDC96 | NR_108036.1 | CFAP58-AS1 | 0.953903622 | 2.160827348 | 0.030708679 |
| CCDC96 | NR_109886.1 | RALY-AS1 | 0.882964796 | 1.937377456 | 0.05269922 |
| CCDC96 | NR_110998.1 | FAM74A4 | 0.855872892 | 1.917202695 | 0.055212186 |
| CCDC96 | NR_120335.1 | LOC101928414 | 0.878557775 | 1.961599119 | 0.049809175 |
| CCDC96 | NR_121188.1 | PGM5P3-AS1 | -0.87235821 | -1.939139554 | 0.052484347 |
| CCDC96 | NR_121189.1 | PGM5P3-AS1 | -0.935867064 | -2.089287728 | 0.03668183 |
| CCDC96 | NR_125957.1 | LOC101928626 | -0.889982126 | -1.994545966 | 0.046092422 |
| CCDC96 | NR_126166.1 | FAM74A7 | 0.906523404 | 2.042909555 | 0.041061392 |
| CCDC96 | NR_126522.1 | EXOC3-AS1 | 0.827471494 | 1.816476079 | 0.069297374 |
| CCDC96 | NR_138084.1 | HCG24 | 0.885055404 | 1.967451317 | 0.049131203 |
| CCDC96 | NR_144459.1 | ARSD-AS1 | 0.892256706 | 2.007645383 | 0.044680988 |
| CCL3L3 | ENST00000411489.1 | ENSG00000227112.1 | 0.826686329 | 1.835453379 | 0.066438541 |
| CCL3L3 | ENST00000418416.1 | ENSG00000226218.1 | 0.917677307 | 2.072903472 | 0.038181264 |
| CCL3L3 | ENST00000418972.1 | ENSG00000225044.1 | 0.82244045 | 1.854490021 | 0.063669087 |
| CCL3L3 | ENST00000422017.1 | ENSG00000232227.1 | 0.850925739 | 1.874676899 | 0.060837187 |
| CCL3L3 | ENST00000433550.1 | ENSG00000232227.1 | 0.849642512 | 1.896712153 | 0.057865939 |
| CCL3L3 | ENST00000435733.1 | ENSG00000226377.1 | -0.800500195 | -1.782091329 | 0.07473434 |
| CCL3L3 | ENST00000442876.1 | ENSG00000233894.1 | 0.827404562 | 1.856217013 | 0.063422632 |
| CCL3L3 | ENST00000452412.1 | ENSG00000233860.1 | -0.880931898 | -1.96940988 | 0.048906042 |
| CCL3L3 | ENST00000454928.1 | ENSG00000186148.7 | -0.861902418 | -1.935925393 | 0.052876839 |
| CCL3L3 | ENST00000487772.1 | ENSG00000241754.1 | -0.919886808 | -2.040382391 | 0.041312254 |
| CCL3L3 | ENST00000502467.1 | ENSG00000250530.1 | 0.860459621 | 1.911966035 | 0.055880551 |
| CCL3L3 | ENST00000510274.1 | ENSG00000245864.2 | 0.922741941 | 2.091252847 | 0.036505404 |
| CCL3L3 | ENST00000535720.1 | ENSG00000256364.1 | -0.817560395 | -1.836760546 | 0.066245253 |
| CCL3L3 | ENST00000535806.1 | ENSG00000255817.1 | -0.864125535 | -1.915862381 | 0.055382615 |
| CCL3L3 | ENST00000537921.1 | ENSG00000255966.1 | -0.842632289 | -1.905042227 | 0.056774582 |
| CCL3L3 | ENST00000544663.1 | ENSG00000256281.1 | -0.814365584 | -1.825992698 | 0.067851362 |
| CCL3L3 | ENST00000562970.1 | ENSG00000260145.1 | -0.94794661 | -2.119070389 | 0.034084519 |
| CCL3L3 | ENST00000567127.1 | ENSG00000260264.1 | 0.864879936 | 1.918075058 | 0.055101495 |
| CCL3L3 | ENST00000577807.1 | ENSG00000263427.1 | -0.82677606 | -1.849708214 | 0.064355616 |
| CCL3L3 | ENST00000580975.1 | ENSG00000266237.1 | -0.88306575 | -1.990102288 | 0.046579669 |
| CCL3L3 | ENST00000583841.1 | ENSG00000265148.1 | -0.820887008 | -1.841269549 | 0.065582068 |
| CCL3L3 | ENST00000584139.1 | ENSG00000263388.1 | 0.818862831 | 1.822465639 | 0.068384363 |
| CCL3L3 | ENST00000596643.1 | ENSG00000269439.1 | -0.93059673 | -2.097996611 | 0.035905445 |
| CCL3L3 | ENST00000599467.1 | ENSG00000244513.2 | -0.818648717 | -1.81219537 | 0.069956014 |
| CCL3L3 | ENST00000602597.1 | ENSG00000269947.1 | -0.931581918 | -2.086272814 | 0.036953918 |
| CCL3L3 | NR_026962.1 | TTC28-AS1 | -0.828157411 | -1.845088512 | 0.065024664 |
| CCL3L3 | NR_110846.1 | LOC101928674 | -0.891180991 | -1.980894241 | 0.047603135 |
| CCL3L3 | NR_134565.1 | LOC101928807 | 0.937512081 | 2.072363861 | 0.038231521 |
| CCL3L3 | NR_134576.1 | LOC105372672 | -0.818749167 | -1.845120453 | 0.065020019 |
| CCL3L3 | NR_135274.1 | LOC105370619 | -0.855270357 | -1.896946539 | 0.057834994 |
| CCL3L3 | NR_136407.1 | LOC105371430 | -0.884234029 | -1.954910742 | 0.050593606 |
| CCL3L3 | NR_138038.1 | LINC00677 | -0.81928803 | -1.824483068 | 0.068079074 |
| CCL4L2 | ENST00000390540.2 | ENSG00000254140.1 | 0.928904797 | 2.087234388 | 0.036866952 |
| CCL4L2 | ENST00000415106.1 | ENSG00000226733.1 | -0.803178849 | -1.784960147 | 0.074267781 |
| CCL4L2 | ENST00000415205.1 | ENSG00000182057.4 | 0.918667412 | 2.047579803 | 0.04060119 |
| CCL4L2 | ENST00000421006.1 | ENSG00000234548.1 | -0.898174643 | -2.015871972 | 0.043813365 |
| CCL4L2 | ENST00000422763.1 | ENSG00000231131.2 | -0.992556822 | -2.225816282 | 0.02602649 |
| CCL4L2 | ENST00000425881.1 | ENSG00000239636.1 | 0.987913633 | 2.211455294 | 0.027004324 |
| CCL4L2 | ENST00000426475.1 | ENSG00000239467.1 | 0.887877564 | 1.984292073 | 0.047223289 |

| | | | | | |
|---|---|---|---|---|---|
| CCL4L2 | ENST00000427132.1 | ENSG00000232121.1 | 0.889098387 | 1.995832901 | 0.045952114 |
| CCL4L2 | ENST00000431730.1 | ENSG00000237401.2 | 0.833555757 | 1.869956053 | 0.061489921 |
| CCL4L2 | ENST00000433035.1 | ENSG00000230483.1 | 0.800167665 | 1.789127576 | 0.073594272 |
| CCL4L2 | ENST00000434250.1 | ENSG00000234055.1 | 0.910123269 | 1.999581592 | 0.045545463 |
| CCL4L2 | ENST00000437334.1 | ENSG00000226134.1 | 0.841051815 | 1.881223748 | 0.059941489 |
| CCL4L2 | ENST00000437680.1 | ENSG00000237133.1 | 0.850239396 | 1.905322259 | 0.056738194 |
| CCL4L2 | ENST00000438969.2 | ENSG00000228031.2 | -0.886351997 | -1.976079655 | 0.048145756 |
| CCL4L2 | ENST00000439443.1 | ENSG00000236911.2 | 0.863594741 | 1.955902017 | 0.050476698 |
| CCL4L2 | ENST00000440595.1 | ENSG00000228265.1 | 0.842508637 | 1.894471046 | 0.058162514 |
| CCL4L2 | ENST00000441592.2 | ENSG00000224078.8 | 0.900479304 | 2.010744917 | 0.044352407 |
| CCL4L2 | ENST00000446562.1 | ENSG00000233896.1 | 0.936462111 | 2.085688693 | 0.037006831 |
| CCL4L2 | ENST00000448570.1 | ENSG00000224549.1 | 0.848445194 | 1.903214664 | 0.05701254 |
| CCL4L2 | ENST00000448674.1 | ENSG00000235119.1 | 0.853892332 | 1.876001316 | 0.060655099 |
| CCL4L2 | ENST00000450500.1 | ENSG00000225790.1 | 0.939547272 | 2.126803998 | 0.033436372 |
| CCL4L2 | ENST00000451507.1 | ENSG00000229539.1 | 0.872159682 | 1.957103928 | 0.050335251 |
| CCL4L2 | ENST00000452553.1 | ENSG00000233973.1 | -0.923643115 | -2.050363386 | 0.040328983 |
| CCL4L2 | ENST00000455416.1 | ENSG00000229337.1 | 0.924271614 | 2.083495496 | 0.037206082 |
| CCL4L2 | ENST00000456091.1 | ENSG00000226985.1 | 0.910240351 | 2.009902408 | 0.044441519 |
| CCL4L2 | ENST00000457632.1 | ENSG00000234248.1 | -0.800493999 | -1.787184516 | 0.073907671 |
| CCL4L2 | ENST00000485347.1 | ENSG00000239991.1 | 0.81775442 | 1.813212511 | 0.069799051 |
| CCL4L2 | ENST00000488310.1 | ENSG00000240449.1 | 0.902537101 | 2.031006225 | 0.042254363 |
| CCL4L2 | ENST00000489090.1 | ENSG00000240045.1 | -0.857993677 | -1.924528265 | 0.054288401 |
| CCL4L2 | ENST00000503723.1 | ENSG00000250472.1 | -0.831784293 | -1.866084527 | 0.06202954 |
| CCL4L2 | ENST00000508081.1 | ENSG00000248254.1 | 0.850713609 | 1.896602805 | 0.05788038 |
| CCL4L2 | ENST00000509453.1 | ENSG00000249145.1 | 0.822020817 | 1.849985911 | 0.06431558 |
| CCL4L2 | ENST00000509629.1 | ENSG00000250164.1 | 0.80334485 | 1.772267193 | 0.076350214 |
| CCL4L2 | ENST00000510570.1 | ENSG00000250438.1 | -0.804519757 | -1.780051639 | 0.07506751 |
| CCL4L2 | ENST00000513023.1 | ENSG00000248809.1 | -0.835260108 | -1.896457773 | 0.057899539 |
| CCL4L2 | ENST00000521378.1 | ENSG00000254222.1 | 0.885766104 | 1.961463828 | 0.049824941 |
| CCL4L2 | ENST00000521653.1 | ENSG00000253301.1 | 0.928221543 | 2.115162626 | 0.034416089 |
| CCL4L2 | ENST00000523806.1 | ENSG00000253616.1 | 0.849714205 | 1.924234591 | 0.054325185 |
| CCL4L2 | ENST00000524133.1 | ENSG00000253174.2 | 0.814357742 | 1.798728498 | 0.072061639 |
| CCL4L2 | ENST00000529247.1 | ENSG00000254741.1 | 0.808554278 | 1.825377107 | 0.067944141 |
| CCL4L2 | ENST00000535315.1 | ENSG00000250748.2 | -0.861207979 | -1.920466272 | 0.054799029 |
| CCL4L2 | ENST00000543403.1 | ENSG00000256684.1 | -0.888933337 | -2.001656314 | 0.045321708 |
| CCL4L2 | ENST00000549616.1 | ENSG00000258168.1 | -0.856409271 | -1.942842218 | 0.052035225 |
| CCL4L2 | ENST00000552378.1 | ENSG00000257294.1 | 0.849686512 | 1.874345019 | 0.060882886 |
| CCL4L2 | ENST00000552634.1 | ENSG00000257496.1 | 0.866733767 | 1.935556105 | 0.052922091 |
| CCL4L2 | ENST00000555913.1 | ENSG00000259077.1 | -0.920902344 | -2.064547909 | 0.038965796 |
| CCL4L2 | ENST00000557368.1 | ENSG00000258444.1 | 0.971121258 | 2.209194595 | 0.027161109 |
| CCL4L2 | ENST00000558221.1 | ENSG00000259704.1 | 0.84099238 | 1.882874301 | 0.059717405 |
| CCL4L2 | ENST00000562191.1 | ENSG00000261292.1 | -0.89394603 | -2.00356403 | 0.045116782 |
| CCL4L2 | ENST00000564809.1 | ENSG00000261471.1 | 0.873789493 | 1.967282826 | 0.049150614 |
| CCL4L2 | ENST00000565310.1 | ENSG00000261118.1 | 0.80340579 | 1.799282979 | 0.071973929 |
| CCL4L2 | ENST00000565623.1 | ENSG00000261118.1 | 0.831950452 | 1.891682386 | 0.058533312 |
| CCL4L2 | ENST00000565955.1 | ENSG00000261055.1 | 0.853569188 | 1.908805183 | 0.056287228 |
| CCL4L2 | ENST00000568332.1 | ENSG00000260256.1 | 0.805651421 | 1.795512259 | 0.072572121 |
| CCL4L2 | ENST00000571660.1 | ENSG00000262848.1 | 0.93722951 | 2.068038556 | 0.038636398 |
| CCL4L2 | ENST00000572471.1 | ENSG00000262721.1 | 0.837135646 | 1.850635417 | 0.064222021 |
| CCL4L2 | ENST00000573315.1 | ENSG00000270168.1 | 0.952139083 | 2.111535329 | 0.034726325 |
| CCL4L2 | ENST00000576271.1 | ENSG00000263342.1 | 0.879842226 | 1.978868774 | 0.047830783 |

| | | | | | |
|---|---|---|---|---|---|
| CCL4L2 | ENST00000579775.1 | ENSG00000264108.1 | 0.900353618 | 2.042968121 | 0.041055594 |
| CCL4L2 | ENST00000580622.1 | ENSG00000264634.1 | 0.974393239 | 2.17388853 | 0.029713508 |
| CCL4L2 | ENST00000582044.1 | ENSG00000263715.2 | 0.885453367 | 1.975071442 | 0.048260041 |
| CCL4L2 | ENST00000585761.1 | ENSG00000267198.1 | 0.941770002 | 2.097996831 | 0.035905425 |
| CCL4L2 | ENST00000586348.1 | ENSG00000267198.1 | 0.958715294 | 2.14128957 | 0.032250694 |
| CCL4L2 | ENST00000589380.1 | ENSG00000267488.1 | 0.986692151 | 2.23041407 | 0.025719966 |
| CCL4L2 | ENST00000589395.1 | ENSG00000267143.1 | 0.875074852 | 1.947136627 | 0.051518357 |
| CCL4L2 | ENST00000591621.1 | ENSG00000232116.2 | -0.802687614 | -1.809233175 | 0.070414786 |
| CCL4L2 | ENST00000592022.1 | ENSG00000267383.2 | -0.833105257 | -1.842116041 | 0.065458178 |
| CCL4L2 | ENST00000592498.1 | ENSG00000267488.1 | 0.960792091 | 2.144375765 | 0.03200279 |
| CCL4L2 | ENST00000592525.1 | ENSG00000267214.1 | 0.957763775 | 2.144571187 | 0.031987148 |
| CCL4L2 | ENST00000593139.1 | ENSG00000267042.1 | 0.884099178 | 2.02041512 | 0.043340346 |
| CCL4L2 | ENST00000593967.1 | ENSG00000232732.5 | 0.901310703 | 1.998530432 | 0.045659184 |
| CCL4L2 | ENST00000594776.1 | ENSG00000269807.1 | 0.893171134 | 2.006156527 | 0.04483955 |
| CCL4L2 | ENST00000594850.1 | ENSG00000268093.1 | 0.950131285 | 2.106089113 | 0.035196612 |
| CCL4L2 | ENST00000596567.1 | ENSG00000226647.2 | -0.86565228 | -1.957295787 | 0.050312703 |
| CCL4L2 | ENST00000596971.1 | ENSG00000269463.1 | 0.962043518 | 2.155979679 | 0.031085252 |
| CCL4L2 | ENST00000597256.1 | ENSG00000267986.1 | 0.80943664 | 1.814653807 | 0.069577128 |
| CCL4L2 | ENST00000597420.1 | ENSG00000269564.1 | 0.815071468 | 1.816769852 | 0.069252361 |
| CCL4L2 | ENST00000600242.1 | ENSG00000269583.1 | 0.86884301 | 1.935365099 | 0.052945509 |
| CCL4L2 | ENST00000602507.1 | ENSG00000270069.1 | -0.829326247 | -1.839949902 | 0.065775593 |
| CCL4L2 | ENST00000603948.1 | ENSG00000222041.6 | 0.949444735 | 2.088075992 | 0.03679098 |
| CCL4L2 | ENST00000604183.1 | ENSG00000271185.1 | 0.856718545 | 1.92667488 | 0.05402016 |
| CCL4L2 | ENST00000605692.1 | ENSG00000270810.1 | 0.862678606 | 1.944952376 | 0.05178071 |
| CCL4L2 | ENST00000605780.1 | ENSG00000270755.1 | 0.883448031 | 1.960993423 | 0.04987979 |
| CCL4L2 | ENST00000606441.1 | ENSG00000272277.1 | 0.935825009 | 2.129354331 | 0.033224956 |
| CCL4L2 | ENST00000606898.1 | ENSG00000272094.1 | 0.804656347 | 1.763683639 | 0.077785257 |
| CCL4L2 | ENST00000606909.1 | ENSG00000271821.1 | 0.842611005 | 1.87542159 | 0.060734747 |
| CCL4L2 | ENST00000609789.1 | ENSG00000272707.1 | 0.828559237 | 1.856754281 | 0.063346121 |
| CCL4L2 | ENST00000609934.1 | ENSG00000273271.1 | -0.834909006 | -1.866918239 | 0.061913006 |
| CCL4L2 | NR_026774.1 | LINC00239 | 0.803855863 | 1.758398884 | 0.078679663 |
| CCL4L2 | NR_026813.1 | LINC00597 | -0.853304079 | -1.912527037 | 0.055808628 |
| CCL4L2 | NR_026932.1 | PDCD4-AS1 | 0.829243223 | 1.849643504 | 0.064364948 |
| CCL4L2 | NR_031762.2 | HCP5B | 0.830158635 | 1.881729043 | 0.059872814 |
| CCL4L2 | NR_034131.1 | LINC00272 | 0.943098032 | 2.137133125 | 0.032587165 |
| CCL4L2 | NR_038923.1 | SSSCA1-AS1 | 0.804749629 | 1.79139307 | 0.07323024 |
| CCL4L2 | NR_040047.1 | SDCBP2-AS1 | 0.91029996 | 2.094116649 | 0.03624959 |
| CCL4L2 | NR_040049.1 | SDCBP2-AS1 | 0.954192589 | 2.16083747 | 0.030707897 |
| CCL4L2 | NR_046224.1 | LINC00659 | 0.802757655 | 1.789592271 | 0.073519482 |
| CCL4L2 | NR_046871.1 | LINC00333 | -0.849052156 | -1.899979542 | 0.057435804 |
| CCL4L2 | NR_073155.1 | C1orf145 | -0.818167738 | -1.833316088 | 0.066755578 |
| CCL4L2 | NR_102746.1 | ROPN1L-AS1 | 0.886617967 | 1.961035524 | 0.049874879 |
| CCL4L2 | NR_108046.1 | LINC00844 | -0.844431613 | -1.899752395 | 0.057465621 |
| CCL4L2 | NR_108106.1 | LINC01135 | 0.823850605 | 1.830185811 | 0.067222159 |
| CCL4L2 | NR_109831.1 | RASSF1-AS1 | 0.874607551 | 1.971844181 | 0.048627397 |
| CCL4L2 | NR_109885.1 | RALY-AS1 | 0.824578207 | 1.843671488 | 0.065231031 |
| CCL4L2 | NR_110245.1 | LOC101929282 | -0.805469566 | -1.808962237 | 0.07045687 |
| CCL4L2 | NR_110559.1 | LOC101927023 | -0.936655422 | -2.080334025 | 0.037494906 |
| CCL4L2 | NR_110803.1 | LOC101927018 | 0.819994339 | 1.849607538 | 0.064370136 |
| CCL4L2 | NR_110824.1 | LINC01986 | -0.844700907 | -1.890453037 | 0.058697396 |
| CCL4L2 | NR_121624.1 | LOC103352541 | -0.805100771 | -1.813381716 | 0.069772968 |

| | | | | | |
|---|---|---|---|---|---|
| CCL4L2 | NR_125849.1 | LOC101928140 | -0.839928548 | -1.907754693 | 0.056422929 |
| CCL4L2 | NR_131204.1 | XACT | 0.925433946 | 2.070254481 | 0.03842852 |
| CCL4L2 | NR_134520.1 | LOC727993 | 0.930389517 | 2.07495139 | 0.03799104 |
| CCL4L2 | NR_135097.1 | LOC105369443 | -0.888933337 | -2.003622582 | 0.045110505 |
| CCL4L2 | NR_135816.1 | LOC100996664 | 0.80866652 | 1.831592074 | 0.067012218 |
| CCL4L2 | NR_136215.1 | VCAN-AS1 | -0.918204635 | -2.04803923 | 0.040556156 |
| CCL4L2 | NR_136320.1 | LOC105373656 | 0.972779391 | 2.179629976 | 0.029284901 |
| CD58 | ENST00000318291.4 | ENSG00000177406.4 | 0.929022031 | 2.095942788 | 0.036087266 |
| CD58 | ENST00000412759.1 | ENSG00000236933.1 | 0.826907688 | 1.859088417 | 0.063014607 |
| CD58 | ENST00000412809.1 | ENSG00000229938.1 | -0.810410125 | -1.770165554 | 0.076699566 |
| CD58 | ENST00000413353.1 | ENSG00000232893.1 | 0.882054378 | 1.965645302 | 0.049339597 |
| CD58 | ENST00000413650.1 | ENSG00000230880.2 | 0.83424121 | 1.872400453 | 0.061151222 |
| CD58 | ENST00000413991.1 | ENSG00000237614.1 | 0.865736538 | 1.940204419 | 0.052354852 |
| CD58 | ENST00000420365.1 | ENSG00000225214.1 | 0.887080658 | 1.986278055 | 0.047002458 |
| CD58 | ENST00000421207.1 | ENSG00000231768.1 | 0.818670152 | 1.84823066 | 0.064568982 |
| CD58 | ENST00000423667.1 | ENSG00000225970.1 | 0.848410718 | 1.892875328 | 0.058374451 |
| CD58 | ENST00000424735.1 | ENSG00000237457.2 | -0.919897322 | -2.055610633 | 0.039820062 |
| CD58 | ENST00000425624.1 | ENSG00000223779.4 | 0.852627646 | 1.910275621 | 0.056097735 |
| CD58 | ENST00000426237.2 | ENSG00000235527.2 | 0.829053401 | 1.859449938 | 0.062963389 |
| CD58 | ENST00000426519.1 | ENSG00000234142.1 | 0.849703757 | 1.895535116 | 0.058021544 |
| CD58 | ENST00000426699.1 | ENSG00000229308.1 | 0.910997103 | 2.036980196 | 0.041652027 |
| CD58 | ENST00000433344.1 | ENSG00000234083.1 | -0.904679583 | -1.997356995 | 0.045786416 |
| CD58 | ENST00000433614.1 | ENSG00000228534.1 | -0.894007489 | -1.992826864 | 0.046280409 |
| CD58 | ENST00000435434.1 | ENSG00000231233.1 | 0.985693494 | 2.205528319 | 0.027417043 |
| CD58 | ENST00000435892.1 | ENSG00000233635.2 | 0.873649362 | 1.941546341 | 0.052192044 |
| CD58 | ENST00000435992.2 | ENSG00000232675.3 | 0.850754297 | 1.875653842 | 0.060702828 |
| CD58 | ENST00000438107.1 | ENSG00000234449.2 | 0.883952477 | 1.973697524 | 0.048416147 |
| CD58 | ENST00000438190.1 | ENSG00000227214.2 | 0.855257662 | 1.895570829 | 0.058016818 |
| CD58 | ENST00000439186.1 | ENSG00000237076.1 | 0.890588716 | 1.961083753 | 0.049869254 |
| CD58 | ENST00000441875.1 | ENSG00000239203.1 | 0.853167906 | 1.895116583 | 0.058076958 |
| CD58 | ENST00000442069.1 | ENSG00000225655.1 | -0.893678245 | -2.013350096 | 0.044077812 |
| CD58 | ENST00000444665.1 | ENSG00000228852.2 | 0.812532448 | 1.831183938 | 0.067073093 |
| CD58 | ENST00000447206.1 | ENSG00000230839.1 | 0.896244252 | 1.973906262 | 0.048392403 |
| CD58 | ENST00000447514.1 | ENSG00000236753.1 | 0.84439241 | 1.883946959 | 0.05957215 |
| CD58 | ENST00000451656.1 | ENSG00000228417.1 | 0.897713143 | 1.990641681 | 0.046520294 |
| CD58 | ENST00000456715.1 | ENSG00000224893.1 | 0.875978781 | 1.970315564 | 0.048802216 |
| CD58 | ENST00000457115.1 | ENSG00000227245.1 | 0.871437685 | 1.936776519 | 0.052772667 |
| CD58 | ENST00000458154.1 | ENSG00000235578.1 | 0.84405226 | 1.918920917 | 0.054994343 |
| CD58 | ENST00000458194.1 | ENSG00000226193.1 | 0.839524036 | 1.88715696 | 0.059139217 |
| CD58 | ENST00000458364.1 | ENSG00000225655.1 | -0.920562223 | -2.104060011 | 0.035373213 |
| CD58 | ENST00000459985.1 | ENSG00000273066.1 | 0.947838594 | 2.09329627 | 0.036322715 |
| CD58 | ENST00000484413.1 | ENSG00000271853.1 | 0.856517893 | 1.910453254 | 0.05607488 |
| CD58 | ENST00000489557.2 | ENSG00000257045.1 | 0.869733072 | 1.959698267 | 0.050031068 |
| CD58 | ENST00000493123.1 | ENSG00000242428.1 | 0.806574751 | 1.767411198 | 0.077159389 |
| CD58 | ENST00000498693.1 | ENSG00000244198.1 | 0.874261902 | 1.975678853 | 0.048191161 |
| CD58 | ENST00000505196.1 | ENSG00000248131.1 | 0.803200183 | 1.781511855 | 0.07482887 |
| CD58 | ENST00000505498.1 | ENSG00000250908.1 | 0.943340909 | 2.118884505 | 0.034100229 |
| CD58 | ENST00000505556.1 | ENSG00000249409.1 | 0.893102437 | 2.028368597 | 0.042522643 |
| CD58 | ENST00000506100.1 | ENSG00000249409.1 | 0.897740327 | 1.987681616 | 0.046846913 |
| CD58 | ENST00000508083.1 | ENSG00000249343.1 | 0.947532936 | 2.109849919 | 0.034871285 |
| CD58 | ENST00000509192.1 | ENSG00000250765.1 | 0.807545952 | 1.817136051 | 0.069196284 |

| | | | | | |
|---|---|---|---|---|---|
| CD58 | ENST00000515128.1 | ENSG00000248215.1 | -0.803038742 | -1.797762129 | 0.072214711 |
| CD58 | ENST00000519852.1 | ENSG00000253716.1 | 0.830190914 | 1.867884558 | 0.061778164 |
| CD58 | ENST00000521307.1 | ENSG00000253177.1 | 0.853534265 | 1.908419656 | 0.056336998 |
| CD58 | ENST00000522547.1 | ENSG00000253430.1 | -0.819201839 | -1.827719427 | 0.067591671 |
| CD58 | ENST00000522600.1 | ENSG00000246582.2 | 0.888012334 | 1.951441838 | 0.051004509 |
| CD58 | ENST00000522704.1 | ENSG00000254135.1 | 0.839059888 | 1.879405129 | 0.060189197 |
| CD58 | ENST00000524335.1 | ENSG00000253716.1 | 0.827629771 | 1.823016545 | 0.068300885 |
| CD58 | ENST00000524818.1 | ENSG00000254473.1 | 0.872219492 | 1.942240127 | 0.052108037 |
| CD58 | ENST00000526611.1 | ENSG00000246982.2 | 0.956630779 | 2.141355627 | 0.03224537 |
| CD58 | ENST00000526935.1 | ENSG00000255372.1 | 0.836438002 | 1.872963161 | 0.061073472 |
| CD58 | ENST00000528887.1 | ENSG00000254501.1 | 0.968609341 | 2.159772177 | 0.030790311 |
| CD58 | ENST00000536141.1 | ENSG00000256969.1 | 0.824699362 | 1.851322905 | 0.064123113 |
| CD58 | ENST00000537269.1 | ENSG00000257084.1 | 0.871154393 | 1.944105335 | 0.05188275 |
| CD58 | ENST00000543072.1 | ENSG00000256092.2 | -0.873080111 | -1.953961218 | 0.050705804 |
| CD58 | ENST00000544089.1 | ENSG00000256273.1 | 0.823293389 | 1.822292304 | 0.068410645 |
| CD58 | ENST00000547834.1 | ENSG00000258325.1 | 0.863085292 | 1.945021125 | 0.051772436 |
| CD58 | ENST00000549878.1 | ENSG00000257284.1 | 0.917703911 | 2.042110593 | 0.041140562 |
| CD58 | ENST00000552469.1 | ENSG00000258325.1 | 0.88360979 | 1.980700294 | 0.047624894 |
| CD58 | ENST00000556786.1 | ENSG00000258525.1 | -0.904435688 | -2.043228041 | 0.041029869 |
| CD58 | ENST00000565823.1 | ENSG00000260686.1 | -0.916701004 | -2.067844622 | 0.038654636 |
| CD58 | ENST00000565829.1 | ENSG00000260148.1 | 0.922870533 | 2.073374211 | 0.038137467 |
| CD58 | ENST00000570512.1 | ENSG00000262768.1 | 0.898321537 | 1.997788283 | 0.045739618 |
| CD58 | ENST00000570843.1 | ENSG00000261889.1 | 0.92260504 | 2.048879862 | 0.040473864 |
| CD58 | ENST00000570929.1 | ENSG00000262223.2 | 0.965159679 | 2.155797904 | 0.031099449 |
| CD58 | ENST00000575139.1 | ENSG00000263072.1 | 0.808818828 | 1.826758081 | 0.067736151 |
| CD58 | ENST00000576086.1 | ENSG00000262823.1 | 0.890206119 | 1.975153303 | 0.048250753 |
| CD58 | ENST00000576554.1 | ENSG00000262413.1 | 0.902152076 | 1.985486198 | 0.047090404 |
| CD58 | ENST00000577064.1 | ENSG00000262823.1 | 0.934720125 | 2.095903913 | 0.036090716 |
| CD58 | ENST00000577176.1 | ENSG00000262823.1 | 0.913830128 | 2.075954929 | 0.03789812 |
| CD58 | ENST00000577698.1 | ENSG00000265100.1 | 0.922325532 | 2.043208729 | 0.04103178 |
| CD58 | ENST00000585559.1 | ENSG00000267117.1 | 0.862389052 | 1.940789755 | 0.052283784 |
| CD58 | ENST00000586010.1 | ENSG00000267606.1 | 0.873030685 | 1.934441494 | 0.053058869 |
| CD58 | ENST00000586051.1 | ENSG00000267576.1 | 0.828374053 | 1.868797672 | 0.061650969 |
| CD58 | ENST00000591174.1 | ENSG00000267289.1 | 0.861898235 | 1.914424458 | 0.055565943 |
| CD58 | ENST00000592400.1 | ENSG00000267735.1 | 0.863218306 | 1.93029084 | 0.053570811 |
| CD58 | ENST00000593632.1 | ENSG00000180279.5 | 0.838627111 | 1.871918622 | 0.061217863 |
| CD58 | ENST00000594590.2 | ENSG00000268199.2 | 0.893138753 | 1.999947799 | 0.045505901 |
| CD58 | ENST00000597169.1 | ENSG00000269720.1 | 0.800513985 | 1.810800525 | 0.070171735 |
| CD58 | ENST00000597309.1 | ENSG00000232098.2 | -0.952461717 | -2.126689261 | 0.03344591 |
| CD58 | ENST00000599259.1 | ENSG00000269352.1 | 0.899297653 | 1.994768526 | 0.046068132 |
| CD58 | ENST00000600489.1 | ENSG00000231898.4 | 0.812747785 | 1.814939834 | 0.069533156 |
| CD58 | ENST00000600534.1 | ENSG00000267858.1 | 0.940753562 | 2.111418063 | 0.034736394 |
| CD58 | ENST00000600726.1 | ENSG00000267858.1 | 0.844823889 | 1.884794993 | 0.059457521 |
| CD58 | ENST00000601692.1 | ENSG00000267874.1 | -0.846040664 | -1.932199953 | 0.053334831 |
| CD58 | ENST00000602532.1 | ENSG00000270091.1 | 0.81201946 | 1.808937914 | 0.070460649 |
| CD58 | ENST00000602872.1 | ENSG00000270067.1 | 0.900014422 | 2.0101746 | 0.044412713 |
| CD58 | ENST00000607222.1 | ENSG00000272106.1 | 0.849570691 | 1.902364514 | 0.057123517 |
| CD58 | ENST00000609067.1 | ENSG00000272849.1 | 0.803157559 | 1.779303931 | 0.075189947 |
| CD58 | ENST00000609281.1 | ENSG00000273320.1 | 0.833806053 | 1.876655136 | 0.060565375 |
| CD58 | ENST00000609972.1 | ENSG00000230651.3 | 0.892193981 | 1.985076332 | 0.04713598 |
| CD58 | ENST00000609976.1 | ENSG00000272582.1 | 0.804220168 | 1.774269211 | 0.076018631 |

| | | | | | |
|---|---|---|---|---|---|
| CD58 | ENST00000610145.1 | ENSG00000273175.1 | 0.972518426 | 2.165688585 | 0.030334992 |
| CD58 | NR_003604.2 | ZFAS1 | 0.906501935 | 1.996639827 | 0.045864323 |
| CD58 | NR_003605.1 | ZFAS1 | 0.914624117 | 2.080178782 | 0.037509138 |
| CD58 | NR_003606.2 | ZFAS1 | 0.902175505 | 2.00364416 | 0.045108192 |
| CD58 | NR_027271.1 | CIRBP-AS1 | 0.868504689 | 1.929629523 | 0.053652758 |
| CD58 | NR_027334.2 | MZF1-AS1 | 0.95379131 | 2.118732201 | 0.034113105 |
| CD58 | NR_036480.1 | VPS9D1-AS1 | 0.825824226 | 1.822469194 | 0.068383824 |
| CD58 | NR_036658.1 | ZFAS1 | 0.903457401 | 2.008524935 | 0.044587539 |
| CD58 | NR_044996.1 | HCG23 | 0.927159833 | 2.106550017 | 0.035156603 |
| CD58 | NR_104158.1 | NRG1-IT1 | 0.826366445 | 1.865989622 | 0.062042817 |
| CD58 | NR_105010.1 | LINC01333 | 0.866693899 | 1.933084466 | 0.053225793 |
| CD58 | NR_108036.1 | CFAP58-AS1 | 0.987137662 | 2.231530192 | 0.02564603 |
| CD58 | NR_110919.1 | LOC101928530 | 0.924479557 | 2.093427135 | 0.036311042 |
| CD58 | NR_121188.1 | PGM5P3-AS1 | -0.894909681 | -2.006362289 | 0.044817609 |
| CD58 | NR_121189.1 | PGM5P3-AS1 | -0.931973331 | -2.067264564 | 0.038709231 |
| CD58 | NR_126166.1 | FAM74A7 | 0.819953331 | 1.813883776 | 0.069695621 |
| CD58 | NR_135032.1 | LOC105369635 | 0.871154393 | 1.925500637 | 0.054166756 |
| CD58 | NR_135644.1 | LOC105371506 | -0.811050727 | -1.815576767 | 0.06943532 |
| CGA | ENST00000412896.1 | ENSG00000197585.5 | -0.840811009 | -1.891699592 | 0.058531018 |
| CGA | ENST00000413564.1 | ENSG00000224500.1 | -0.811465275 | -1.838965877 | 0.065920205 |
| CGA | ENST00000413989.1 | ENSG00000242628.1 | 0.909111961 | 2.009395252 | 0.044495233 |
| CGA | ENST00000414992.1 | ENSG00000233613.1 | -0.940727645 | -2.115245629 | 0.034409018 |
| CGA | ENST00000417315.1 | ENSG00000242486.1 | -0.813810214 | -1.809752503 | 0.070334177 |
| CGA | ENST00000421006.1 | ENSG00000234548.1 | 0.870535211 | 1.946729832 | 0.051567133 |
| CGA | ENST00000421498.1 | ENSG00000237978.1 | 0.83991995 | 1.869326025 | 0.061577469 |
| CGA | ENST00000421597.1 | ENSG00000227851.1 | 0.848622443 | 1.902561283 | 0.057097815 |
| CGA | ENST00000423925.1 | ENSG00000223536.1 | -0.918109461 | -2.053401775 | 0.040033627 |
| CGA | ENST00000424274.1 | ENSG00000232120.1 | 0.82866911 | 1.855454245 | 0.063531387 |
| CGA | ENST00000425058.1 | ENSG00000226771.1 | 0.859450384 | 1.92970151 | 0.053643833 |
| CGA | ENST00000426125.1 | ENSG00000223653.1 | 0.930597435 | 2.087350484 | 0.036856464 |
| CGA | ENST00000426213.1 | ENSG00000223660.1 | -0.839403696 | -1.880497637 | 0.060040288 |
| CGA | ENST00000426504.1 | ENSG00000234190.1 | -0.852739708 | -1.903121761 | 0.057024659 |
| CGA | ENST00000426929.1 | ENSG00000230184.1 | -0.845792484 | -1.890995893 | 0.058624892 |
| CGA | ENST00000427132.1 | ENSG00000232121.1 | -0.924478262 | -2.083690824 | 0.037188299 |
| CGA | ENST00000428160.1 | ENSG00000236897.1 | 0.839669389 | 1.882965978 | 0.059704979 |
| CGA | ENST00000428440.1 | ENSG00000232827.2 | -0.904485252 | -1.988367627 | 0.046771046 |
| CGA | ENST00000429878.1 | ENSG00000224184.1 | -0.872069128 | -1.960304448 | 0.049960216 |
| CGA | ENST00000430842.1 | ENSG00000230433.1 | -0.877082854 | -1.951551226 | 0.050991509 |
| CGA | ENST00000432957.1 | ENSG00000231534.1 | -0.854913216 | -1.927048462 | 0.053973591 |
| CGA | ENST00000434493.1 | ENSG00000224605.1 | 0.942181079 | 2.098247853 | 0.035883256 |
| CGA | ENST00000435271.1 | ENSG00000231132.1 | -0.837697189 | -1.888232759 | 0.05899471 |
| CGA | ENST00000435357.1 | ENSG00000225444.1 | 0.947207877 | 2.082182456 | 0.037325807 |
| CGA | ENST00000435984.1 | ENSG00000204792.2 | 0.869254772 | 1.992473772 | 0.0463191 |
| CGA | ENST00000436582.1 | ENSG00000236525.1 | -0.842560597 | -1.883680313 | 0.059608231 |
| CGA | ENST00000437334.1 | ENSG00000226134.1 | -0.876835693 | -1.968159221 | 0.049049721 |
| CGA | ENST00000437680.1 | ENSG00000237133.1 | -0.830096144 | -1.855272043 | 0.063557388 |
| CGA | ENST00000440862.1 | ENSG00000223804.1 | -0.829762296 | -1.848240285 | 0.06456759 |
| CGA | ENST00000443162.1 | ENSG00000234183.1 | 0.804196287 | 1.794700661 | 0.072701405 |
| CGA | ENST00000444731.1 | ENSG00000227131.1 | -0.914037268 | -2.0557984 | 0.039801952 |
| CGA | ENST00000445178.1 | ENSG00000234653.1 | 0.89242122 | 2.003908347 | 0.045079879 |
| CGA | ENST00000445617.2 | ENSG00000225751.2 | 0.931301412 | 2.072367152 | 0.038231214 |

| | | | | | |
|---|---|---|---|---|---|
| CGA | ENST00000445631.1 | ENSG00000231052.1 | -0.938157072 | -2.122998493 | 0.033753978 |
| CGA | ENST00000447183.2 | ENSG00000271593.1 | 0.858220714 | 1.902225666 | 0.057141659 |
| CGA | ENST00000448001.1 | ENSG00000229639.1 | 0.913833003 | 2.006571463 | 0.044795312 |
| CGA | ENST00000448650.1 | ENSG00000223536.1 | -0.917529392 | -2.076398192 | 0.037857138 |
| CGA | ENST00000449586.1 | ENSG00000235257.4 | -0.91817694 | -2.040798304 | 0.041270879 |
| CGA | ENST00000449903.1 | ENSG00000223872.1 | -0.850512781 | -1.915472636 | 0.055432256 |
| CGA | ENST00000450206.1 | ENSG00000234311.1 | -0.88224806 | -1.979515479 | 0.047757999 |
| CGA | ENST00000450500.1 | ENSG00000225790.1 | -0.855814701 | -1.977698219 | 0.047962761 |
| CGA | ENST00000452002.1 | ENSG00000236501.1 | -0.831381026 | -1.888751447 | 0.058925141 |
| CGA | ENST00000452553.1 | ENSG00000233973.1 | 0.863250221 | 1.90739355 | 0.056469644 |
| CGA | ENST00000453584.1 | ENSG00000233613.1 | -0.939420625 | -2.092483126 | 0.03639532 |
| CGA | ENST00000453878.1 | ENSG00000224850.1 | 0.941324665 | 2.095559566 | 0.036121279 |
| CGA | ENST00000454709.1 | ENSG00000237280.1 | -0.915940598 | -2.086816079 | 0.036904763 |
| CGA | ENST00000455416.1 | ENSG00000229337.1 | -0.865328808 | -1.932207007 | 0.053333961 |
| CGA | ENST00000457632.1 | ENSG00000234248.1 | 0.864741134 | 1.955670733 | 0.050503955 |
| CGA | ENST00000460993.1 | ENSG00000241231.1 | 0.838718169 | 1.877665373 | 0.060426957 |
| CGA | ENST00000476099.1 | ENSG00000244158.1 | -0.894216054 | -1.978753619 | 0.047843753 |
| CGA | ENST00000477643.1 | ENSG00000241224.2 | -0.87782345 | -1.998052457 | 0.045710973 |
| CGA | ENST00000479233.1 | ENSG00000243150.1 | 0.903058676 | 2.02518908 | 0.042847953 |
| CGA | ENST00000482142.1 | ENSG00000243276.1 | 0.972819358 | 2.196992914 | 0.028020952 |
| CGA | ENST00000485347.1 | ENSG00000239991.1 | -0.971211193 | -2.223370826 | 0.026190805 |
| CGA | ENST00000488040.1 | ENSG00000243176.1 | 0.828218249 | 1.855934505 | 0.063462894 |
| CGA | ENST00000505404.1 | ENSG00000249941.1 | -0.894918368 | -2.015245463 | 0.043878936 |
| CGA | ENST00000505575.1 | ENSG00000248939.1 | -0.838839468 | -1.855403567 | 0.063538618 |
| CGA | ENST00000506059.1 | ENSG00000248311.1 | 0.816982566 | 1.817472522 | 0.069144792 |
| CGA | ENST00000506379.1 | ENSG00000240152.2 | 0.841856878 | 1.871161114 | 0.061322753 |
| CGA | ENST00000506852.1 | ENSG00000250945.1 | 0.872529264 | 1.977263352 | 0.04801187 |
| CGA | ENST00000507558.1 | ENSG00000248445.1 | 0.86777265 | 1.956920028 | 0.050356871 |
| CGA | ENST00000507808.1 | ENSG00000250333.1 | -0.856424131 | -1.910551358 | 0.05606226 |
| CGA | ENST00000508845.1 | ENSG00000271724.1 | 0.907653793 | 2.044261036 | 0.040927766 |
| CGA | ENST00000509629.1 | ENSG00000250164.1 | -0.901684572 | -2.022499817 | 0.043124743 |
| CGA | ENST00000509983.1 | ENSG00000248173.1 | -0.908353726 | -2.030150479 | 0.042341246 |
| CGA | ENST00000510602.1 | ENSG00000249122.1 | -0.885314312 | -1.953710536 | 0.05073546 |
| CGA | ENST00000511234.1 | ENSG00000250865.1 | -0.819022703 | -1.838145374 | 0.066040987 |
| CGA | ENST00000511603.1 | ENSG00000249892.1 | 0.885689008 | 2.008889051 | 0.044548901 |
| CGA | ENST00000514459.1 | ENSG00000248211.1 | -0.978074452 | -2.201002386 | 0.027735855 |
| CGA | ENST00000518260.1 | ENSG00000253628.1 | -0.834600342 | -1.875622964 | 0.060707071 |
| CGA | ENST00000518416.1 | ENSG00000253901.1 | 0.824405616 | 1.853864965 | 0.063758481 |
| CGA | ENST00000518620.1 | ENSG00000253892.1 | -0.979053644 | -2.164439744 | 0.030430616 |
| CGA | ENST00000519038.2 | ENSG00000254054.2 | -0.849725604 | -1.898503623 | 0.057629771 |
| CGA | ENST00000519375.1 | ENSG00000253980.1 | 0.971006836 | 2.167577603 | 0.030190839 |
| CGA | ENST00000519660.1 | ENSG00000253416.1 | 0.920122537 | 2.061072982 | 0.039296078 |
| CGA | ENST00000519844.1 | ENSG00000253824.1 | -0.909256396 | -2.025454552 | 0.042820711 |
| CGA | ENST00000521378.1 | ENSG00000254222.1 | -0.934916615 | -2.103060496 | 0.035460482 |
| CGA | ENST00000521490.1 | ENSG00000253407.1 | -0.907249119 | -2.036180505 | 0.041732233 |
| CGA | ENST00000522190.1 | ENSG00000254165.1 | 0.869047689 | 1.933492553 | 0.05317555 |
| CGA | ENST00000522408.1 | ENSG00000253484.1 | 0.816414975 | 1.841127811 | 0.065602831 |
| CGA | ENST00000524133.1 | ENSG00000253174.2 | -0.974289644 | -2.164949893 | 0.030391523 |
| CGA | ENST00000524309.1 | ENSG00000240915.2 | -0.844360689 | -1.903106096 | 0.057026703 |
| CGA | ENST00000525855.1 | ENSG00000254746.1 | -0.894854006 | -2.014844948 | 0.043920898 |
| CGA | ENST00000528869.1 | ENSG00000255443.1 | 0.931423703 | 2.090652458 | 0.03655923 |

| | | | | | |
|---|---|---|---|---|---|
| CGA | ENST00000529875.1 | ENSG00000254404.1 | -0.814013908 | -1.796481811 | 0.072417923 |
| CGA | ENST00000531071.1 | ENSG00000255248.2 | -0.931317373 | -2.100128815 | 0.035717511 |
| CGA | ENST00000533938.1 | ENSG00000255142.1 | -0.997542618 | -2.272239156 | 0.023072069 |
| CGA | ENST00000535324.1 | ENSG00000255968.1 | 0.805806204 | 1.808237243 | 0.070569584 |
| CGA | ENST00000538294.1 | ENSG00000250748.2 | -0.952986302 | -2.165085487 | 0.030381139 |
| CGA | ENST00000547175.1 | ENSG00000257395.1 | -0.836479549 | -1.860880381 | 0.062761071 |
| CGA | ENST00000548748.1 | ENSG00000258252.1 | -0.970311036 | -2.170346103 | 0.029980637 |
| CGA | ENST00000549616.1 | ENSG00000258168.1 | 0.870371992 | 1.952351354 | 0.050896504 |
| CGA | ENST00000549756.1 | ENSG00000257769.1 | -0.89825787 | -1.995679078 | 0.045968866 |
| CGA | ENST00000550805.1 | ENSG00000244306.5 | -0.920196156 | -2.076010063 | 0.03789302 |
| CGA | ENST00000552558.1 | ENSG00000257947.1 | 0.817936358 | 1.813695292 | 0.06972465 |
| CGA | ENST00000552634.1 | ENSG00000257496.1 | -0.892465744 | -1.961091922 | 0.049868301 |
| CGA | ENST00000553477.1 | ENSG00000259123.1 | 0.881141552 | 1.95424855 | 0.05067183 |
| CGA | ENST00000553668.1 | ENSG00000258733.1 | 0.911345256 | 2.034979484 | 0.041852937 |
| CGA | ENST00000554430.1 | ENSG00000258646.1 | -0.816233271 | -1.819753939 | 0.068796485 |
| CGA | ENST00000555913.1 | ENSG00000259077.1 | 0.914474412 | 2.05330102 | 0.040043392 |
| CGA | ENST00000556458.1 | ENSG00000258504.2 | -0.931262707 | -2.088518824 | 0.036751059 |
| CGA | ENST00000556978.1 | ENSG00000258693.1 | 0.805502328 | 1.801655469 | 0.071599628 |
| CGA | ENST00000557368.1 | ENSG00000258444.1 | -0.818683191 | -1.86991935 | 0.061495018 |
| CGA | ENST00000557855.1 | ENSG00000259176.1 | 0.835822739 | 1.875708645 | 0.060695298 |
| CGA | ENST00000557903.1 | ENSG00000259182.1 | 0.80207524 | 1.757687152 | 0.078800755 |
| CGA | ENST00000558141.1 | ENSG00000259594.1 | 0.837260114 | 1.880009948 | 0.060106722 |
| CGA | ENST00000558221.1 | ENSG00000259704.1 | -0.81856482 | -1.8444777 | 0.065113553 |
| CGA | ENST00000558434.1 | ENSG00000259572.1 | -0.803294671 | -1.81227961 | 0.069943004 |
| CGA | ENST00000560969.1 | ENSG00000259176.1 | 0.819313267 | 1.840621503 | 0.065677044 |
| CGA | ENST00000561847.1 | ENSG00000260293.1 | -0.866658645 | -1.949607405 | 0.05122293 |
| CGA | ENST00000562582.1 | ENSG00000259779.1 | 0.800949074 | 1.786551726 | 0.07400997 |
| CGA | ENST00000563342.1 | ENSG00000259914.1 | 0.849945967 | 1.923945089 | 0.054361467 |
| CGA | ENST00000563570.1 | ENSG00000259961.1 | -0.822487347 | -1.848727498 | 0.064497171 |
| CGA | ENST00000563601.1 | ENSG00000260589.1 | -0.96091165 | -2.160944245 | 0.030699647 |
| CGA | ENST00000565271.1 | ENSG00000261335.1 | -0.926484274 | -2.079952494 | 0.03752989 |
| CGA | ENST00000565623.1 | ENSG00000261118.1 | -0.967329826 | -2.167923487 | 0.030164508 |
| CGA | ENST00000568410.1 | ENSG00000260277.1 | 0.850745491 | 1.908245938 | 0.056359437 |
| CGA | ENST00000569215.1 | ENSG00000260756.1 | 0.877950823 | 1.984408518 | 0.047210317 |
| CGA | ENST00000576271.1 | ENSG00000263342.1 | -0.856033153 | -1.938774129 | 0.052528848 |
| CGA | ENST00000582895.1 | ENSG00000264729.1 | -0.841165898 | -1.887495948 | 0.059093651 |
| CGA | ENST00000585761.1 | ENSG00000267198.1 | -0.837741265 | -1.853198615 | 0.063853896 |
| CGA | ENST00000586297.1 | ENSG00000267633.1 | 0.863659214 | 1.951945997 | 0.050944616 |
| CGA | ENST00000586348.1 | ENSG00000267198.1 | -0.823516779 | -1.822863185 | 0.068324115 |
| CGA | ENST00000587049.1 | ENSG00000235535.3 | -0.928062574 | -2.055390499 | 0.039841302 |
| CGA | ENST00000588177.1 | ENSG00000234899.5 | 0.929932601 | 2.067779372 | 0.038660774 |
| CGA | ENST00000589281.1 | ENSG00000267707.1 | -0.817013045 | -1.803135963 | 0.071366864 |
| CGA | ENST00000591137.1 | ENSG00000267405.1 | 0.80809578 | 1.838864884 | 0.065935062 |
| CGA | ENST00000591621.1 | ENSG00000232116.2 | 0.800394362 | 1.779570528 | 0.075146273 |
| CGA | ENST00000592431.1 | ENSG00000267475.1 | -0.933214428 | -2.119620044 | 0.034038101 |
| CGA | ENST00000592523.1 | ENSG00000226994.3 | 0.85259231 | 1.914417822 | 0.05556679 |
| CGA | ENST00000593967.1 | ENSG00000232732.5 | -0.800828238 | -1.792316535 | 0.073082276 |
| CGA | ENST00000594762.1 | ENSG00000231898.4 | 0.825282911 | 1.828625439 | 0.067455739 |
| CGA | ENST00000596567.1 | ENSG00000226647.2 | 0.815383576 | 1.823783925 | 0.068184745 |
| CGA | ENST00000597420.1 | ENSG00000269564.1 | -0.865317296 | -1.941634741 | 0.052181334 |
| CGA | ENST00000598356.1 | ENSG00000269640.1 | 0.854149968 | 1.908472876 | 0.056330126 |

| | | | | | |
|---|---|---|---|---|---|
| CGA | ENST00000598887.1 | ENSG00000268475.1 | -0.858302406 | -1.91207769 | 0.05586623 |
| CGA | ENST00000599050.1 | ENSG00000268366.1 | 0.87260606 | 1.947450945 | 0.051480695 |
| CGA | ENST00000599387.1 | ENSG00000227733.4 | 0.858769131 | 1.929028666 | 0.053727304 |
| CGA | ENST00000600848.1 | ENSG00000228065.6 | 0.809823449 | 1.79889152 | 0.072035842 |
| CGA | ENST00000602443.1 | ENSG00000270076.1 | 0.915253059 | 2.039603162 | 0.041389867 |
| CGA | ENST00000602485.1 | ENSG00000270163.1 | -0.818187812 | -1.820998097 | 0.068607146 |
| CGA | ENST00000602790.1 | ENSG00000270000.1 | -0.920074735 | -2.067794459 | 0.038659355 |
| CGA | ENST00000603612.1 | ENSG00000270996.1 | 0.856297708 | 1.922368119 | 0.054559454 |
| CGA | ENST00000605021.1 | ENSG00000271401.1 | 0.8535603 | 1.901296797 | 0.057263148 |
| CGA | ENST00000605692.1 | ENSG00000270810.1 | -0.893357142 | -1.989729111 | 0.046620784 |
| CGA | ENST00000606757.1 | ENSG00000237188.3 | -0.814548364 | -1.832007618 | 0.066950286 |
| CGA | ENST00000606898.1 | ENSG00000272094.1 | -0.945089758 | -2.097033408 | 0.035990618 |
| CGA | ENST00000606921.1 | ENSG00000272402.1 | -0.934948646 | -2.112763264 | 0.034621035 |
| CGA | ENST00000606938.1 | ENSG00000272198.1 | -0.828506899 | -1.885086129 | 0.05941821 |
| CGA | ENST00000607594.1 | ENSG00000271766.1 | 0.837171221 | 1.8528391 | 0.063905424 |
| CGA | ENST00000607740.1 | ENSG00000271916.1 | 0.882644163 | 1.993934965 | 0.046159162 |
| CGA | ENST00000608133.1 | ENSG00000273193.1 | 0.826829855 | 1.858874961 | 0.063044864 |
| CGA | ENST00000608173.1 | ENSG00000197099.4 | 0.811879244 | 1.801669011 | 0.071597496 |
| CGA | ENST00000608289.1 | ENSG00000272958.1 | -0.993101303 | -2.231340641 | 0.025658574 |
| CGA | ENST00000608422.1 | ENSG00000272866.1 | 0.839027501 | 1.851624408 | 0.064079776 |
| CGA | ENST00000609182.1 | ENSG00000273248.1 | -0.82691218 | -1.813266036 | 0.069790799 |
| CGA | ENST00000609238.1 | ENSG00000272703.1 | -0.820427863 | -1.842296193 | 0.065431836 |
| CGA | ENST00000609789.1 | ENSG00000272707.1 | -0.879837168 | -1.968008377 | 0.049067074 |
| CGA | ENST00000609890.1 | ENSG00000231898.4 | 0.857311336 | 1.938491063 | 0.05256334 |
| CGA | NR_022011.1 | PWARSN | 0.912074696 | 2.032692024 | 0.042083648 |
| CGA | NR_026932.1 | PDCD4-AS1 | -0.895684012 | -2.009833683 | 0.044448794 |
| CGA | NR_034131.1 | LINC00272 | -0.842211444 | -1.904077028 | 0.056900153 |
| CGA | NR_040047.1 | SDCBP2-AS1 | -0.835560631 | -1.902206343 | 0.057144184 |
| CGA | NR_040049.1 | SDCBP2-AS1 | -0.820467429 | -1.847475488 | 0.064678257 |
| CGA | NR_046556.1 | RBMS3-AS1 | 0.832423339 | 1.884311477 | 0.059522856 |
| CGA | NR_102746.1 | ROPN1L-AS1 | -0.915050017 | -2.033331427 | 0.04201905 |
| CGA | NR_104620.1 | LINC01672 | 0.848974285 | 1.87523449 | 0.060760471 |
| CGA | NR_109975.1 | ARNTL2-AS1 | -0.828473938 | -1.845769799 | 0.064925638 |
| CGA | NR_110284.1 | LOC101927907 | 0.89242122 | 1.987212769 | 0.046898823 |
| CGA | NR_110370.1 | STAM-AS1 | -0.922087093 | -2.072106072 | 0.03825555 |
| CGA | NR_110504.1 | LOC101929572 | -0.972438122 | -2.1916157 | 0.028407267 |
| CGA | NR_120466.1 | LINC01489 | 0.843260183 | 1.872341063 | 0.061159433 |
| CGA | NR_125407.1 | LOC102724604 | 0.860368945 | 1.961848687 | 0.049780103 |
| CGA | NR_125769.1 | LINC01269 | -0.848589488 | -1.880449818 | 0.060046799 |
| CGA | NR_125925.1 | LOC101929448 | 0.873287511 | 1.956940218 | 0.050354497 |
| CGA | NR_126041.1 | LOC101930071 | -0.988947209 | -2.252682561 | 0.024279171 |
| CGA | NR_130916.1 | LOC105274304 | -0.939014619 | -2.07985583 | 0.037538758 |
| CGA | NR_131243.1 | SMCR2 | -0.895742601 | -1.996834902 | 0.045843121 |
| CGA | NR_134632.1 | LOC105373051 | -0.867855508 | -1.928383766 | 0.05380741 |
| CGA | NR_135239.1 | LINC01867 | 0.960713221 | 2.133017152 | 0.032923319 |
| CGA | NR_135549.1 | LOC101929411 | 0.826516021 | 1.841885961 | 0.065491833 |
| CGA | NR_135816.1 | LOC100996664 | -0.871049398 | -1.947432145 | 0.051482947 |
| CGA | NR_136320.1 | LOC105373656 | -0.824801867 | -1.845978979 | 0.064895258 |
| CHCHD1 | ENST00000318291.4 | ENSG00000177406.4 | 0.875262028 | 1.965220032 | 0.049388776 |
| CHCHD1 | ENST00000398777.3 | ENSG00000240152.2 | 0.835728963 | 1.899395893 | 0.057512443 |
| CHCHD1 | ENST00000399186.2 | ENSG00000214888.2 | 0.859700519 | 1.917918915 | 0.055121293 |

| | | | | | |
|---|---|---|---|---|---|
| CHCHD1 | ENST00000413650.1 | ENSG00000230880.2 | 0.860336074 | 1.87993111 | 0.060117467 |
| CHCHD1 | ENST00000413991.1 | ENSG00000237614.1 | 0.80800422 | 1.79909471 | 0.0720037 |
| CHCHD1 | ENST00000417426.1 | ENSG00000233145.1 | 0.879191024 | 1.967930273 | 0.049076061 |
| CHCHD1 | ENST00000420365.1 | ENSG00000225214.1 | 0.816108857 | 1.834567612 | 0.066569781 |
| CHCHD1 | ENST00000421020.1 | ENSG00000231407.1 | 0.836360791 | 1.86608765 | 0.062029104 |
| CHCHD1 | ENST00000421207.1 | ENSG00000231768.1 | 0.914908591 | 2.058113685 | 0.039579222 |
| CHCHD1 | ENST00000423667.1 | ENSG00000225970.1 | 0.929803802 | 2.082049535 | 0.037337945 |
| CHCHD1 | ENST00000423869.1 | ENSG00000227848.1 | 0.852669529 | 1.91987237 | 0.054874023 |
| CHCHD1 | ENST00000424735.1 | ENSG00000237457.2 | -0.836740455 | -1.889850341 | 0.058777979 |
| CHCHD1 | ENST00000425624.1 | ENSG00000223779.4 | 0.961304681 | 2.149012563 | 0.031633404 |
| CHCHD1 | ENST00000426504.1 | ENSG00000234190.1 | -0.813965263 | -1.810838194 | 0.070165902 |
| CHCHD1 | ENST00000426519.1 | ENSG00000234142.1 | 0.846105842 | 1.903672573 | 0.056952841 |
| CHCHD1 | ENST00000426699.1 | ENSG00000229308.1 | 0.980375124 | 2.173943985 | 0.029709342 |
| CHCHD1 | ENST00000427064.1 | ENSG00000238031.1 | 0.814045068 | 1.824644158 | 0.068054745 |
| CHCHD1 | ENST00000433344.1 | ENSG00000234083.1 | -0.95718644 | -2.135242882 | 0.032741176 |
| CHCHD1 | ENST00000433614.1 | ENSG00000228534.1 | -0.953438787 | -2.129325819 | 0.033227313 |
| CHCHD1 | ENST00000435434.1 | ENSG00000231233.1 | 0.965578558 | 2.155623177 | 0.031113101 |
| CHCHD1 | ENST00000435892.1 | ENSG00000233635.2 | 0.835747291 | 1.897977951 | 0.057698987 |
| CHCHD1 | ENST00000435992.2 | ENSG00000232675.3 | 0.946985851 | 2.115455066 | 0.034391181 |
| CHCHD1 | ENST00000436982.2 | ENSG00000235335.2 | -0.894392364 | -2.029737669 | 0.042383212 |
| CHCHD1 | ENST00000438107.1 | ENSG00000234449.2 | 0.950825022 | 2.109197095 | 0.034927573 |
| CHCHD1 | ENST00000438190.1 | ENSG00000227214.2 | 0.820712824 | 1.856356003 | 0.063402831 |
| CHCHD1 | ENST00000438222.1 | ENSG00000238034.1 | 0.85809802 | 1.940075179 | 0.052370554 |
| CHCHD1 | ENST00000439186.1 | ENSG00000237076.1 | 0.964789505 | 2.15801385 | 0.030926755 |
| CHCHD1 | ENST00000441875.1 | ENSG00000239203.1 | 0.837571494 | 1.889630127 | 0.058807445 |
| CHCHD1 | ENST00000442069.1 | ENSG00000225655.1 | -0.872998555 | -1.963520054 | 0.049585777 |
| CHCHD1 | ENST00000444665.1 | ENSG00000228852.2 | 0.835178638 | 1.844285983 | 0.065141473 |
| CHCHD1 | ENST00000447206.1 | ENSG00000230839.1 | 0.845345128 | 1.89834907 | 0.057650114 |
| CHCHD1 | ENST00000447514.1 | ENSG00000236753.1 | 0.923456132 | 2.070243174 | 0.038429578 |
| CHCHD1 | ENST00000447709.1 | ENSG00000237473.1 | 0.802402732 | 1.781133721 | 0.074890608 |
| CHCHD1 | ENST00000450848.1 | ENSG00000225539.1 | 0.902875884 | 2.053779539 | 0.039997034 |
| CHCHD1 | ENST00000451656.1 | ENSG00000228417.1 | 0.889458219 | 1.984084626 | 0.047246407 |
| CHCHD1 | ENST00000455699.1 | ENSG00000240996.1 | 0.847797059 | 1.896931915 | 0.057836925 |
| CHCHD1 | ENST00000457115.1 | ENSG00000227245.1 | 0.813136871 | 1.804220933 | 0.071196679 |
| CHCHD1 | ENST00000457169.1 | ENSG00000232408.1 | 0.856639809 | 1.913589885 | 0.055672579 |
| CHCHD1 | ENST00000458154.1 | ENSG00000235578.1 | 0.875701174 | 1.961026724 | 0.049875906 |
| CHCHD1 | ENST00000458364.1 | ENSG00000225655.1 | -0.936276577 | -2.122820919 | 0.033768861 |
| CHCHD1 | ENST00000458661.2 | ENSG00000236467.3 | 0.81842153 | 1.830922969 | 0.067112041 |
| CHCHD1 | ENST00000459985.1 | ENSG00000273066.1 | 0.852289985 | 1.901897496 | 0.057184557 |
| CHCHD1 | ENST00000469931.2 | ENSG00000272030.1 | 0.82192836 | 1.84527206 | 0.064997973 |
| CHCHD1 | ENST00000484413.1 | ENSG00000271853.1 | 0.965791915 | 2.145222637 | 0.031935051 |
| CHCHD1 | ENST00000493123.1 | ENSG00000242428.1 | 0.875294539 | 1.93400872 | 0.053112056 |
| CHCHD1 | ENST00000494509.1 | ENSG00000240095.1 | 0.81531043 | 1.82650621 | 0.067774047 |
| CHCHD1 | ENST00000498693.1 | ENSG00000244198.1 | 0.828412947 | 1.882930583 | 0.059709776 |
| CHCHD1 | ENST00000501405.2 | ENSG00000247402.2 | -0.810746503 | -1.798344161 | 0.072122486 |
| CHCHD1 | ENST00000502421.1 | ENSG00000250284.1 | -0.818606367 | -1.837022199 | 0.066206618 |
| CHCHD1 | ENST00000503938.1 | ENSG00000246095.2 | 0.846415519 | 1.906918837 | 0.056531098 |
| CHCHD1 | ENST00000504795.1 | ENSG00000250723.1 | 0.840099524 | 1.89386782 | 0.058242557 |
| CHCHD1 | ENST00000504891.1 | ENSG00000249388.1 | 0.902237265 | 2.009976913 | 0.044433632 |
| CHCHD1 | ENST00000505498.1 | ENSG00000250908.1 | 0.946707449 | 2.100328134 | 0.035699986 |
| CHCHD1 | ENST00000505556.1 | ENSG00000249409.1 | 0.906819376 | 2.038037446 | 0.041546188 |

| | | | | | |
|---|---|---|---|---|---|
| CHCHD1 | ENST00000506100.1 | ENSG00000249409.1 | 0.907494244 | 2.018389275 | 0.043550735 |
| CHCHD1 | ENST00000506305.1 | ENSG00000249994.1 | -0.814279573 | -1.824788975 | 0.06803288 |
| CHCHD1 | ENST00000506791.1 | ENSG00000251131.1 | 0.820459847 | 1.823716238 | 0.068194983 |
| CHCHD1 | ENST00000508083.1 | ENSG00000249343.1 | 0.939602734 | 2.148019872 | 0.031712177 |
| CHCHD1 | ENST00000509192.1 | ENSG00000250765.1 | 0.819063704 | 1.845658225 | 0.064941847 |
| CHCHD1 | ENST00000517716.1 | ENSG00000253515.1 | -0.902367158 | -2.031129106 | 0.0422419 |
| CHCHD1 | ENST00000518473.1 | ENSG00000253985.1 | 0.871973342 | 1.951180201 | 0.051035613 |
| CHCHD1 | ENST00000521307.1 | ENSG00000253177.1 | 0.907633812 | 2.015281366 | 0.043875176 |
| CHCHD1 | ENST00000521953.1 | ENSG00000253214.1 | 0.804248919 | 1.822334636 | 0.068404226 |
| CHCHD1 | ENST00000522704.1 | ENSG00000254135.1 | 0.895503038 | 2.00928829 | 0.044506569 |
| CHCHD1 | ENST00000524818.1 | ENSG00000254473.1 | 0.9604369 | 2.142611983 | 0.032144268 |
| CHCHD1 | ENST00000525133.1 | ENSG00000255375.1 | 0.918220737 | 2.057229049 | 0.039664198 |
| CHCHD1 | ENST00000526186.1 | ENSG00000254510.1 | 0.935639485 | 2.110789956 | 0.03479037 |
| CHCHD1 | ENST00000526611.1 | ENSG00000246982.2 | 0.859730275 | 1.935022176 | 0.052987574 |
| CHCHD1 | ENST00000526935.1 | ENSG00000255372.1 | 0.947024999 | 2.130820243 | 0.033103954 |
| CHCHD1 | ENST00000528887.1 | ENSG00000254501.1 | 0.87492269 | 1.971265206 | 0.048693549 |
| CHCHD1 | ENST00000536141.1 | ENSG00000256969.1 | 0.859144295 | 1.932026952 | 0.053356179 |
| CHCHD1 | ENST00000537269.1 | ENSG00000257084.1 | 0.832853813 | 1.852634259 | 0.063934799 |
| CHCHD1 | ENST00000544089.1 | ENSG00000256273.1 | 0.907221567 | 2.01435148 | 0.043972645 |
| CHCHD1 | ENST00000547834.1 | ENSG00000258325.1 | 0.889925443 | 2.014571906 | 0.043949524 |
| CHCHD1 | ENST00000549140.1 | ENSG00000258332.1 | 0.888198135 | 1.993196608 | 0.046239922 |
| CHCHD1 | ENST00000549878.1 | ENSG00000257284.1 | 0.851996807 | 1.901345212 | 0.057256811 |
| CHCHD1 | ENST00000552469.1 | ENSG00000258325.1 | 0.896328393 | 2.011551939 | 0.04426719 |
| CHCHD1 | ENST00000556786.1 | ENSG00000258525.1 | -0.937395093 | -2.074075889 | 0.038072263 |
| CHCHD1 | ENST00000558312.1 | ENSG00000259176.1 | 0.84977176 | 1.923273663 | 0.05444569 |
| CHCHD1 | ENST00000558896.1 | ENSG00000259176.1 | 0.827146003 | 1.834173851 | 0.066628192 |
| CHCHD1 | ENST00000559003.1 | ENSG00000259520.1 | 0.895964091 | 1.979267158 | 0.047785935 |
| CHCHD1 | ENST00000559569.1 | ENSG00000259760.1 | -0.812209662 | -1.803849206 | 0.07125495 |
| CHCHD1 | ENST00000563841.1 | ENSG00000261029.1 | 0.877160133 | 1.927525731 | 0.053914144 |
| CHCHD1 | ENST00000565823.1 | ENSG00000260686.1 | -0.821421928 | -1.824542886 | 0.068070039 |
| CHCHD1 | ENST00000565829.1 | ENSG00000260148.1 | 0.829988682 | 1.894288024 | 0.05818679 |
| CHCHD1 | ENST00000565965.1 | ENSG00000261172.1 | 0.921126931 | 2.050369399 | 0.040328397 |
| CHCHD1 | ENST00000566170.1 | ENSG00000261071.1 | 0.83841589 | 1.858507244 | 0.063097016 |
| CHCHD1 | ENST00000567395.1 | ENSG00000261090.1 | 0.85410044 | 1.91730784 | 0.055198835 |
| CHCHD1 | ENST00000568033.1 | ENSG00000261480.1 | 0.852478563 | 1.894145957 | 0.058205639 |
| CHCHD1 | ENST00000569981.1 | ENSG00000238045.5 | 0.855750296 | 1.912980907 | 0.055750497 |
| CHCHD1 | ENST00000570493.2 | ENSG00000261898.2 | 0.804662286 | 1.810508368 | 0.070216988 |
| CHCHD1 | ENST00000570512.1 | ENSG00000262768.1 | 0.939507487 | 2.116664154 | 0.034288361 |
| CHCHD1 | ENST00000570843.1 | ENSG00000261889.1 | 0.854928176 | 1.880632504 | 0.060021927 |
| CHCHD1 | ENST00000570929.1 | ENSG00000262223.2 | 0.89778734 | 2.015739581 | 0.043827214 |
| CHCHD1 | ENST00000574365.1 | ENSG00000262837.1 | 0.812037361 | 1.824737738 | 0.068040616 |
| CHCHD1 | ENST00000575139.1 | ENSG00000263072.1 | 0.879202452 | 1.976819401 | 0.048062048 |
| CHCHD1 | ENST00000576554.1 | ENSG00000262413.1 | 0.827119726 | 1.861704261 | 0.062644788 |
| CHCHD1 | ENST00000577064.1 | ENSG00000262823.1 | 0.813950086 | 1.822932436 | 0.068313625 |
| CHCHD1 | ENST00000577678.1 | ENSG00000265415.1 | 0.82269175 | 1.845418735 | 0.06497665 |
| CHCHD1 | ENST00000577698.1 | ENSG00000265100.1 | 0.937013966 | 2.093229499 | 0.036328673 |
| CHCHD1 | ENST00000578265.1 | ENSG00000214719.7 | 0.924093592 | 2.066705884 | 0.038761876 |
| CHCHD1 | ENST00000578757.1 | ENSG00000175061.13 | 0.851190031 | 1.898803694 | 0.057590291 |
| CHCHD1 | ENST00000581940.1 | ENSG00000265484.1 | 0.854825205 | 1.918387685 | 0.055061871 |
| CHCHD1 | ENST00000584758.1 | ENSG00000265356.1 | 0.840013655 | 1.883439015 | 0.059640897 |
| CHCHD1 | ENST00000585559.1 | ENSG00000267117.1 | 0.880718993 | 1.980440061 | 0.047654102 |

| | | | | | |
|---|---|---|---|---|---|
| CHCHD1 | ENST00000586010.1 | ENSG00000267606.1 | 0.899730456 | 1.998772512 | 0.045632973 |
| CHCHD1 | ENST00000588799.1 | ENSG00000267275.1 | 0.932477388 | 2.078863516 | 0.037629897 |
| CHCHD1 | ENST00000588945.1 | ENSG00000267275.1 | 0.876722384 | 1.963654619 | 0.049570159 |
| CHCHD1 | ENST00000592400.1 | ENSG00000267735.1 | 0.862571576 | 1.920688074 | 0.054771044 |
| CHCHD1 | ENST00000593632.1 | ENSG00000180279.5 | 0.838243528 | 1.862152471 | 0.062581602 |
| CHCHD1 | ENST00000597169.1 | ENSG00000269720.1 | 0.856422776 | 1.932032103 | 0.053355544 |
| CHCHD1 | ENST00000597309.1 | ENSG00000232098.2 | -0.881491908 | -1.949643572 | 0.051218616 |
| CHCHD1 | ENST00000599259.1 | ENSG00000269352.1 | 0.893708829 | 2.014482466 | 0.043958904 |
| CHCHD1 | ENST00000600489.1 | ENSG00000231898.4 | 0.918305921 | 2.044060849 | 0.040947536 |
| CHCHD1 | ENST00000600534.1 | ENSG00000267858.1 | 0.85815449 | 1.911031046 | 0.056000591 |
| CHCHD1 | ENST00000601692.1 | ENSG00000267874.1 | -0.908870785 | -2.001174068 | 0.045373635 |
| CHCHD1 | ENST00000601735.1 | ENSG00000244513.2 | 0.873660573 | 1.919297984 | 0.054946633 |
| CHCHD1 | ENST00000602592.1 | ENSG00000270049.1 | 0.846564218 | 1.909049898 | 0.056255655 |
| CHCHD1 | ENST00000602809.1 | ENSG00000270105.1 | -0.850068957 | -1.890966952 | 0.058628756 |
| CHCHD1 | ENST00000602872.1 | ENSG00000270067.1 | 0.944974686 | 2.09339865 | 0.036313583 |
| CHCHD1 | ENST00000602954.1 | ENSG00000269906.1 | 0.863906924 | 1.942653867 | 0.052057994 |
| CHCHD1 | ENST00000606377.1 | ENSG00000272286.1 | -0.823610924 | -1.830560859 | 0.067166115 |
| CHCHD1 | ENST00000606457.1 | ENSG00000271830.1 | 0.80953158 | 1.816027886 | 0.069366095 |
| CHCHD1 | ENST00000606470.1 | ENSG00000271913.1 | 0.853168094 | 1.925878746 | 0.054119516 |
| CHCHD1 | ENST00000607222.1 | ENSG00000272106.1 | 0.823582226 | 1.824529734 | 0.068072025 |
| CHCHD1 | ENST00000607284.1 | ENSG00000272389.1 | 0.873501668 | 1.952548375 | 0.050873134 |
| CHCHD1 | ENST00000607876.1 | ENSG00000272848.1 | 0.808150567 | 1.801652439 | 0.071600105 |
| CHCHD1 | ENST00000609067.1 | ENSG00000272849.1 | 0.88957436 | 1.996770402 | 0.04585013 |
| CHCHD1 | ENST00000609610.1 | ENSG00000232675.3 | 0.859265935 | 1.929585517 | 0.053658215 |
| CHCHD1 | ENST00000609725.1 | ENSG00000231898.4 | 0.878958868 | 1.985144144 | 0.047128437 |
| CHCHD1 | ENST00000609972.1 | ENSG00000230651.3 | 0.985012604 | 2.201491566 | 0.027701243 |
| CHCHD1 | ENST00000610145.1 | ENSG00000273175.1 | 0.935670882 | 2.088094543 | 0.036789307 |
| CHCHD1 | ENST00000610161.1 | ENSG00000273059.1 | 0.835404875 | 1.89268194 | 0.058400179 |
| CHCHD1 | NR_003604.2 | ZFAS1 | 0.855851339 | 1.91788268 | 0.055125889 |
| CHCHD1 | NR_003605.1 | ZFAS1 | 0.903730135 | 2.015122068 | 0.043891861 |
| CHCHD1 | NR_003606.2 | ZFAS1 | 0.860686748 | 1.898273486 | 0.057660065 |
| CHCHD1 | NR_027271.1 | CIRBP-AS1 | 0.82112683 | 1.827737264 | 0.067588993 |
| CHCHD1 | NR_027334.2 | MZF1-AS1 | 0.879114452 | 1.939494411 | 0.052441164 |
| CHCHD1 | NR_036658.1 | ZFAS1 | 0.865571123 | 1.947177931 | 0.051513407 |
| CHCHD1 | NR_044996.1 | HCG23 | 0.987758035 | 2.216468698 | 0.026659419 |
| CHCHD1 | NR_046742.2 | ZNF630-AS1 | -0.849990258 | -1.896362147 | 0.057912174 |
| CHCHD1 | NR_103790.1 | LINC00581 | -0.801721248 | -1.798515589 | 0.072095341 |
| CHCHD1 | NR_105010.1 | LINC01333 | 0.877694598 | 1.95470018 | 0.050618469 |
| CHCHD1 | NR_108036.1 | CFAP58-AS1 | 0.949163809 | 2.106880752 | 0.035127917 |
| CHCHD1 | NR_110480.1 | LOC101927079 | 0.871794862 | 1.950753824 | 0.051086337 |
| CHCHD1 | NR_110481.1 | LOC101927079 | 0.84977176 | 1.867060879 | 0.061893087 |
| CHCHD1 | NR_120335.1 | LOC101928414 | 0.946064864 | 2.105423339 | 0.035254474 |
| CHCHD1 | NR_121188.1 | PGM5P3-AS1 | -0.843272486 | -1.904871091 | 0.05679683 |
| CHCHD1 | NR_121189.1 | PGM5P3-AS1 | -0.919802711 | -2.060383546 | 0.039361889 |
| CHCHD1 | NR_126166.1 | FAM74A7 | 0.930349076 | 2.04288782 | 0.041063544 |
| CHCHD1 | NR_134597.1 | LOC105378068 | 0.852933483 | 1.916977058 | 0.055240846 |
| CHCHD1 | NR_135032.1 | LOC105369635 | 0.832853813 | 1.877059517 | 0.060509937 |
| CHCHD1 | NR_138084.1 | HCG24 | 0.909499049 | 2.061743704 | 0.039232143 |
| CSNK1A1L | ENST00000411824.1 | ENSG00000232803.1 | -0.858463909 | -1.90206085 | 0.0571632 |
| CSNK1A1L | ENST00000413645.1 | ENSG00000228798.1 | 0.876885259 | 1.962901967 | 0.049657566 |
| CSNK1A1L | ENST00000415448.1 | ENSG00000228329.1 | 0.952181225 | 2.119023192 | 0.034088507 |

| | | | | | |
|---|---|---|---|---|---|
| CSNK1A1L | ENST00000420498.1 | ENSG00000224985.1 | 0.895856404 | 1.988609555 | 0.046744316 |
| CSNK1A1L | ENST00000427691.1 | ENSG00000228340.1 | -0.867788318 | -1.925729891 | 0.05413811 |
| CSNK1A1L | ENST00000441160.1 | ENSG00000228437.1 | 0.842854266 | 1.90673633 | 0.05655474 |
| CSNK1A1L | ENST00000451648.1 | ENSG00000232803.1 | -0.857931995 | -1.928388395 | 0.053806834 |
| CSNK1A1L | ENST00000454100.1 | ENSG00000236943.2 | -0.838224121 | -1.870562913 | 0.061405689 |
| CSNK1A1L | ENST00000502300.1 | ENSG00000249451.1 | 0.825276933 | 1.839042409 | 0.065908949 |
| CSNK1A1L | ENST00000508199.1 | ENSG00000247810.2 | 0.833521403 | 1.854416008 | 0.063679666 |
| CSNK1A1L | ENST00000508825.1 | ENSG00000250775.1 | -0.833497854 | -1.869306146 | 0.061580234 |
| CSNK1A1L | ENST00000515136.1 | ENSG00000251274.1 | -0.865980247 | -1.928386934 | 0.053807016 |
| CSNK1A1L | ENST00000518590.1 | ENSG00000253986.1 | -0.828717015 | -1.842947844 | 0.065336626 |
| CSNK1A1L | ENST00000520411.1 | ENSG00000253355.1 | -0.805624792 | -1.806009469 | 0.070916861 |
| CSNK1A1L | ENST00000521884.1 | ENSG00000253355.1 | -0.888416695 | -1.985208077 | 0.047121326 |
| CSNK1A1L | ENST00000522524.1 | ENSG00000253342.1 | -0.866792216 | -1.914981044 | 0.055494921 |
| CSNK1A1L | ENST00000524286.1 | ENSG00000253658.1 | 0.893943347 | 2.008943109 | 0.044543167 |
| CSNK1A1L | ENST00000546789.1 | ENSG00000257740.1 | 0.890723853 | 2.008182141 | 0.04462394 |
| CSNK1A1L | ENST00000561215.1 | ENSG00000259611.1 | 0.802848499 | 1.791004503 | 0.073292572 |
| CSNK1A1L | ENST00000568332.1 | ENSG00000260256.1 | -0.800094636 | -1.790005941 | 0.073452956 |
| CSNK1A1L | ENST00000568414.1 | ENSG00000260986.1 | -0.827022045 | -1.830669429 | 0.067149899 |
| CSNK1A1L | ENST00000568756.2 | ENSG00000261760.2 | -0.831282711 | -1.866920594 | 0.061912678 |
| CSNK1A1L | ENST00000573953.1 | ENSG00000261971.2 | -0.867950977 | -1.927813086 | 0.053878379 |
| CSNK1A1L | ENST00000585181.1 | ENSG00000265749.1 | 0.81633496 | 1.823582243 | 0.068215253 |
| CSNK1A1L | ENST00000589754.1 | ENSG00000206129.3 | -0.955831714 | -2.15672165 | 0.031027359 |
| CSNK1A1L | ENST00000591217.1 | ENSG00000231616.4 | 0.859149524 | 1.911648287 | 0.055921321 |
| CSNK1A1L | ENST00000591225.1 | ENSG00000228290.2 | -0.879052515 | -1.968732226 | 0.048983849 |
| CSNK1A1L | ENST00000592681.1 | ENSG00000228290.2 | -0.873576232 | -1.945605907 | 0.051702097 |
| CSNK1A1L | ENST00000593824.1 | ENSG00000268184.1 | 0.884396364 | 1.971500177 | 0.048666693 |
| CSNK1A1L | ENST00000594091.1 | ENSG00000232732.5 | -0.846727833 | -1.891023855 | 0.05862116 |
| CSNK1A1L | ENST00000596497.1 | ENSG00000268530.1 | 0.819494949 | 1.8157008 | 0.069416281 |
| CSNK1A1L | ENST00000600512.1 | ENSG00000269752.1 | 0.918118613 | 2.055449826 | 0.039835577 |
| CSNK1A1L | ENST00000600644.1 | ENSG00000267313.2 | 0.892464499 | 1.979584201 | 0.04775027 |
| CSNK1A1L | ENST00000602614.1 | ENSG00000269957.1 | -0.817070203 | -1.804425965 | 0.071164556 |
| CSNK1A1L | ENST00000606899.1 | ENSG00000272426.1 | 0.948126692 | 2.143745598 | 0.032053276 |
| CSNK1A1L | ENST00000607044.1 | ENSG00000272247.1 | 0.813700781 | 1.825139977 | 0.067979909 |
| CSNK1A1L | ENST00000610044.1 | ENSG00000273160.1 | -0.838465729 | -1.857652208 | 0.063218419 |
| CSNK1A1L | NR_028325.1 | LOC100132062 | 0.890407241 | 1.998585921 | 0.045653175 |
| CSNK1A1L | NR_046369.1 | LOC100131626 | -0.929409422 | -2.060129725 | 0.039386141 |
| CSNK1A1L | NR_046370.1 | LOC100131626 | -0.901154932 | -2.012533339 | 0.044163746 |
| CSNK1A1L | NR_049776.1 | GPC5-AS1 | 0.803844655 | 1.784744344 | 0.074302795 |
| CSNK1A1L | NR_103857.1 | SP2-AS1 | -0.839053874 | -1.901777066 | 0.057200306 |
| CSNK1A1L | NR_108068.1 | LINC00836 | -0.893717027 | -1.965435885 | 0.04936381 |
| CSNK1A1L | NR_109770.1 | TONSL-AS1 | -0.84945356 | -1.915748774 | 0.055397081 |
| CSNK1A1L | NR_133642.1 | DIRC3-AS1 | 0.831331328 | 1.845694524 | 0.064936573 |
| CXCL6 | ENST00000413645.1 | ENSG00000228798.1 | 0.962907625 | 2.165879641 | 0.030320385 |
| CXCL6 | ENST00000416657.1 | ENSG00000235858.1 | 0.823040434 | 1.829732211 | 0.067289992 |
| CXCL6 | ENST00000418972.1 | ENSG00000225044.1 | -0.864174116 | -1.930925098 | 0.053492316 |
| CXCL6 | ENST00000419207.2 | ENSG00000231248.2 | -0.808284478 | -1.787070118 | 0.073926156 |
| CXCL6 | ENST00000420498.1 | ENSG00000224985.1 | 0.922646307 | 2.060653387 | 0.03933612 |
| CXCL6 | ENST00000422038.1 | ENSG00000227935.1 | 0.814673147 | 1.819160003 | 0.068887023 |
| CXCL6 | ENST00000425371.2 | ENSG00000235872.2 | 0.830801189 | 1.852819567 | 0.063908225 |
| CXCL6 | ENST00000426653.1 | ENSG00000235704.1 | 0.892726077 | 1.962032792 | 0.049758667 |
| CXCL6 | ENST00000427691.1 | ENSG00000228340.1 | -0.86090795 | -1.933454707 | 0.053180208 |

| | | | | | |
|---|---|---|---|---|---|
| CXCL6 | ENST00000429608.1 | ENSG00000237480.1 | 0.852417251 | 1.909873383 | 0.056149518 |
| CXCL6 | ENST00000437330.1 | ENSG00000229203.1 | 0.882130444 | 1.950734312 | 0.051088659 |
| CXCL6 | ENST00000448365.1 | ENSG00000231114.1 | 0.815293395 | 1.831970699 | 0.066955786 |
| CXCL6 | ENST00000454928.1 | ENSG00000186148.7 | 0.842216694 | 1.888717781 | 0.058929655 |
| CXCL6 | ENST00000476892.1 | ENSG00000241345.1 | 0.868805184 | 1.96780771 | 0.049090167 |
| CXCL6 | ENST00000502300.1 | ENSG00000249451.1 | 0.9670732 | 2.121564006 | 0.033874368 |
| CXCL6 | ENST00000507997.1 | ENSG00000250551.1 | -0.843117542 | -1.888859874 | 0.058910607 |
| CXCL6 | ENST00000508199.1 | ENSG00000247810.2 | 0.844149912 | 1.92041116 | 0.054805985 |
| CXCL6 | ENST00000508241.1 | ENSG00000248518.1 | 0.927333654 | 2.063943352 | 0.039023088 |
| CXCL6 | ENST00000515136.1 | ENSG00000251274.1 | -0.881497429 | -1.996126042 | 0.045920205 |
| CXCL6 | ENST00000515750.1 | ENSG00000249061.1 | -0.809996213 | -1.818520034 | 0.068984688 |
| CXCL6 | ENST00000522524.1 | ENSG00000253342.1 | -0.888367288 | -1.971833255 | 0.048628645 |
| CXCL6 | ENST00000526154.1 | ENSG00000254511.1 | 0.835665778 | 1.88310669 | 0.059685911 |
| CXCL6 | ENST00000526694.1 | ENSG00000231999.2 | 0.802478113 | 1.781656496 | 0.074805265 |
| CXCL6 | ENST00000531627.1 | ENSG00000254584.1 | 0.837678625 | 1.857300769 | 0.063268375 |
| CXCL6 | ENST00000532688.1 | ENSG00000255441.1 | 0.859534712 | 1.907224872 | 0.056491474 |
| CXCL6 | ENST00000535746.1 | ENSG00000256101.1 | 0.978963685 | 2.171793831 | 0.029871217 |
| CXCL6 | ENST00000537921.1 | ENSG00000255966.1 | 0.805305648 | 1.788209708 | 0.073742181 |
| CXCL6 | ENST00000547750.1 | ENSG00000257886.1 | 0.87046105 | 1.941732821 | 0.052169453 |
| CXCL6 | ENST00000549303.1 | ENSG00000257180.1 | -0.828938758 | -1.874535223 | 0.060856692 |
| CXCL6 | ENST00000554197.1 | ENSG00000197176.3 | 0.931280655 | 2.081125976 | 0.037422377 |
| CXCL6 | ENST00000557232.1 | ENSG00000259054.1 | 0.921680666 | 2.062146212 | 0.039193818 |
| CXCL6 | ENST00000558515.1 | ENSG00000259182.1 | 0.848777845 | 1.894964529 | 0.058097101 |
| CXCL6 | ENST00000561215.1 | ENSG00000259611.1 | 0.938176023 | 2.090623763 | 0.036561804 |
| CXCL6 | ENST00000567127.1 | ENSG00000260264.1 | -0.805643565 | -1.803557847 | 0.071300649 |
| CXCL6 | ENST00000572222.1 | ENSG00000261971.2 | -0.800338728 | -1.800660698 | 0.071756376 |
| CXCL6 | ENST00000583916.1 | ENSG00000264196.1 | 0.818518467 | 1.828864625 | 0.067419891 |
| CXCL6 | ENST00000586399.1 | ENSG00000228430.4 | 0.850829999 | 1.88191427 | 0.059847657 |
| CXCL6 | ENST00000589233.1 | ENSG00000231616.4 | 0.871563582 | 1.954880742 | 0.050597148 |
| CXCL6 | ENST00000589817.1 | ENSG00000231616.4 | 0.900706314 | 2.013270198 | 0.044086212 |
| CXCL6 | ENST00000590255.1 | ENSG00000235779.3 | 0.812592848 | 1.801737578 | 0.071586703 |
| CXCL6 | ENST00000590368.1 | ENSG00000231616.4 | 0.974428211 | 2.186120847 | 0.028806765 |
| CXCL6 | ENST00000590813.1 | ENSG00000231616.4 | 0.947443892 | 2.118925067 | 0.0340968 |
| CXCL6 | ENST00000590995.1 | ENSG00000267198.1 | 0.982655232 | 2.207293604 | 0.027293554 |
| CXCL6 | ENST00000591217.1 | ENSG00000231616.4 | 0.975282332 | 2.179651358 | 0.029283315 |
| CXCL6 | ENST00000591225.1 | ENSG00000228290.2 | -0.841282559 | -1.905663159 | 0.056693922 |
| CXCL6 | ENST00000592622.1 | ENSG00000267546.2 | -0.923717999 | -2.056315979 | 0.039752069 |
| CXCL6 | ENST00000593269.1 | ENSG00000236172.2 | 0.944558531 | 2.140614532 | 0.032305136 |
| CXCL6 | ENST00000593642.1 | ENSG00000267858.1 | 0.851680367 | 1.869168128 | 0.061599427 |
| CXCL6 | ENST00000593824.1 | ENSG00000268184.1 | 0.845634311 | 1.876085836 | 0.060643494 |
| CXCL6 | ENST00000594492.1 | ENSG00000250910.3 | 0.925950467 | 2.062635648 | 0.039147258 |
| CXCL6 | ENST00000596497.1 | ENSG00000268530.1 | 0.903603331 | 2.004580279 | 0.045007937 |
| CXCL6 | ENST00000600512.1 | ENSG00000269752.1 | 0.857453079 | 1.923745087 | 0.054386544 |
| CXCL6 | ENST00000602614.1 | ENSG00000269957.1 | -0.894212748 | -2.015660483 | 0.04383549 |
| CXCL6 | ENST00000602773.1 | ENSG00000270160.1 | -0.946440614 | -2.112489979 | 0.034644445 |
| CXCL6 | ENST00000606841.1 | ENSG00000272411.1 | 0.808652342 | 1.806876772 | 0.070781495 |
| CXCL6 | ENST00000606899.1 | ENSG00000272426.1 | 0.876714061 | 1.970813218 | 0.048745244 |
| CXCL6 | ENST00000607044.1 | ENSG00000272247.1 | 0.932351445 | 2.09950869 | 0.035772083 |
| CXCL6 | ENST00000608264.1 | ENSG00000273473.1 | 0.888647601 | 1.986715658 | 0.046953916 |
| CXCL6 | ENST00000609837.1 | ENSG00000273106.1 | 0.906245341 | 2.046405703 | 0.040716471 |
| CXCL6 | ENST00000609953.1 | ENSG00000272825.1 | 0.828707775 | 1.869361147 | 0.061572586 |

| | | | | | |
|---|---|---|---|---|---|
| CXCL6 | ENST00000610044.1 | ENSG00000273160.1 | -0.82163519 | -1.831163176 | 0.067076191 |
| CXCL6 | ENST00000610185.1 | ENSG00000273355.1 | -0.827877483 | -1.841490746 | 0.065549675 |
| CXCL6 | NR_027401.2 | FAM223A | 0.86978618 | 1.945169184 | 0.051754619 |
| CXCL6 | NR_028325.1 | LOC100132062 | 0.92690817 | 2.066589404 | 0.03877286 |
| CXCL6 | NR_046578.1 | CACNA1C-AS4 | 0.822894016 | 1.835101343 | 0.066490675 |
| CXCL6 | NR_049776.1 | GPC5-AS1 | 0.843402156 | 1.877171673 | 0.060494569 |
| CXCL6 | NR_103857.1 | SP2-AS1 | -0.927623264 | -2.058301359 | 0.039561214 |
| CXCL6 | NR_110635.1 | LINC00687 | 0.801134535 | 1.807328177 | 0.070711125 |
| CXCL6 | NR_121661.1 | ZBTB20-AS5 | -0.945380162 | -2.116659682 | 0.034288741 |
| CXCL6 | NR_135820.1 | LOC102723727 | 0.911657685 | 2.035049144 | 0.041845928 |
| DCXR | ENST00000295549.4 | ENSG00000163364.5 | 0.884409292 | 1.970227539 | 0.048812299 |
| DCXR | ENST00000412896.1 | ENSG00000197585.5 | -0.841710984 | -1.890058517 | 0.058750134 |
| DCXR | ENST00000413645.1 | ENSG00000228798.1 | 0.859241337 | 1.942917919 | 0.052026076 |
| DCXR | ENST00000413887.1 | ENSG00000236948.1 | -0.840736265 | -1.865779034 | 0.062072287 |
| DCXR | ENST00000418972.1 | ENSG00000225044.1 | -0.80974982 | -1.827769013 | 0.067584226 |
| DCXR | ENST00000419296.1 | ENSG00000204588.5 | -0.840806875 | -1.871076708 | 0.06133445 |
| DCXR | ENST00000420315.1 | ENSG00000228072.1 | 0.838525576 | 1.891801324 | 0.058517457 |
| DCXR | ENST00000420465.1 | ENSG00000167355.3 | 0.836004714 | 1.869659862 | 0.061531067 |
| DCXR | ENST00000426444.1 | ENSG00000239395.1 | 0.949482594 | 2.125194606 | 0.033570378 |
| DCXR | ENST00000426653.1 | ENSG00000235704.1 | 0.911096869 | 2.042901121 | 0.041062227 |
| DCXR | ENST00000428160.1 | ENSG00000236897.1 | 0.843166498 | 1.874461724 | 0.060866813 |
| DCXR | ENST00000428853.2 | ENSG00000229206.2 | 0.959254688 | 2.166918601 | 0.030241061 |
| DCXR | ENST00000429630.1 | ENSG00000232533.1 | 0.929037442 | 2.080847309 | 0.037447884 |
| DCXR | ENST00000429666.1 | ENSG00000233755.1 | 0.84541742 | 1.911447558 | 0.05594709 |
| DCXR | ENST00000437330.1 | ENSG00000229203.1 | 0.897115954 | 1.982046381 | 0.04747405 |
| DCXR | ENST00000441029.2 | ENSG00000229188.2 | -0.912520201 | -2.019955029 | 0.043388052 |
| DCXR | ENST00000443123.1 | ENSG00000229457.1 | 0.861115213 | 1.928012171 | 0.053853612 |
| DCXR | ENST00000443306.1 | ENSG00000233891.3 | 0.848136935 | 1.903802745 | 0.056935879 |
| DCXR | ENST00000447111.1 | ENSG00000231903.1 | 0.880407028 | 1.948642772 | 0.0513381 |
| DCXR | ENST00000448365.1 | ENSG00000231114.1 | 0.943606813 | 2.106144294 | 0.03519182 |
| DCXR | ENST00000451697.1 | ENSG00000233823.1 | -0.827581133 | -1.860633936 | 0.062795889 |
| DCXR | ENST00000472596.1 | ENSG00000239774.1 | 0.86121008 | 1.953562741 | 0.050752951 |
| DCXR | ENST00000476892.1 | ENSG00000241345.1 | 0.932805184 | 2.103470732 | 0.035424641 |
| DCXR | ENST00000480904.2 | ENSG00000206573.4 | -0.801294699 | -1.817574226 | 0.069129234 |
| DCXR | ENST00000498199.1 | ENSG00000206573.4 | -0.814901024 | -1.814731978 | 0.069565108 |
| DCXR | ENST00000500498.2 | ENSG00000245311.2 | -0.910972348 | -2.048581467 | 0.040503058 |
| DCXR | ENST00000502300.1 | ENSG00000249451.1 | 0.880393289 | 1.993916992 | 0.046161127 |
| DCXR | ENST00000506420.1 | ENSG00000250034.1 | -0.849658864 | -1.906264085 | 0.056615951 |
| DCXR | ENST00000507997.1 | ENSG00000250551.1 | -0.991869784 | -2.237482268 | 0.025254837 |
| DCXR | ENST00000508241.1 | ENSG00000248518.1 | 0.916735232 | 2.022674791 | 0.043106688 |
| DCXR | ENST00000512563.1 | ENSG00000249547.1 | 0.887819176 | 1.97969166 | 0.047738186 |
| DCXR | ENST00000515750.1 | ENSG00000249061.1 | -0.882437577 | -1.967564716 | 0.049118143 |
| DCXR | ENST00000522426.1 | ENSG00000253538.1 | 0.843528243 | 1.920856277 | 0.054749829 |
| DCXR | ENST00000522524.1 | ENSG00000253342.1 | -0.82613404 | -1.856023083 | 0.063450268 |
| DCXR | ENST00000524073.1 | ENSG00000253774.1 | 0.876567576 | 1.958792353 | 0.050137109 |
| DCXR | ENST00000531627.1 | ENSG00000254584.1 | 0.885943888 | 1.988689468 | 0.046735489 |
| DCXR | ENST00000532249.1 | ENSG00000234899.5 | 0.868250316 | 1.952465695 | 0.05088294 |
| DCXR | ENST00000535746.1 | ENSG00000256101.1 | 0.926203799 | 2.056097157 | 0.039773152 |
| DCXR | ENST00000536412.1 | ENSG00000256072.1 | -0.931296387 | -2.061536661 | 0.03925187 |
| DCXR | ENST00000536492.1 | ENSG00000256237.1 | -0.823647551 | -1.825412762 | 0.067938765 |
| DCXR | ENST00000545642.1 | ENSG00000256342.1 | 0.921748248 | 2.068891027 | 0.038556314 |

| | | | | | |
|---|---|---|---|---|---|
| DCXR | ENST00000547750.1 | ENSG00000257886.1 | 0.876764219 | 1.992203407 | 0.046348745 |
| DCXR | ENST00000548199.1 | ENSG00000257614.1 | 0.907691307 | 2.011773239 | 0.044243846 |
| DCXR | ENST00000551174.1 | ENSG00000257762.1 | -0.922208635 | -2.050795572 | 0.040286859 |
| DCXR | ENST00000554197.1 | ENSG00000197176.3 | 0.883705185 | 1.994251526 | 0.046124574 |
| DCXR | ENST00000557232.1 | ENSG00000259054.1 | 0.867148776 | 1.913930546 | 0.055629031 |
| DCXR | ENST00000558515.1 | ENSG00000259182.1 | 0.822414795 | 1.820369258 | 0.068702791 |
| DCXR | ENST00000561215.1 | ENSG00000259611.1 | 0.896138628 | 1.985645369 | 0.047072715 |
| DCXR | ENST00000562582.1 | ENSG00000259779.1 | 0.853525723 | 1.901148848 | 0.057282519 |
| DCXR | ENST00000562834.1 | ENSG00000261116.1 | 0.827300615 | 1.866741992 | 0.061937627 |
| DCXR | ENST00000563342.1 | ENSG00000259914.1 | 0.816157356 | 1.820387553 | 0.068700007 |
| DCXR | ENST00000565944.1 | ENSG00000260331.1 | 0.877848341 | 1.97870454 | 0.047849282 |
| DCXR | ENST00000568836.1 | ENSG00000259967.1 | 0.974613373 | 2.149665519 | 0.031581682 |
| DCXR | ENST00000570919.1 | ENSG00000263321.1 | 0.954115879 | 2.144201007 | 0.032016784 |
| DCXR | ENST00000577360.1 | ENSG00000264273.1 | 0.918151994 | 2.053736643 | 0.040001188 |
| DCXR | ENST00000578334.1 | ENSG00000265148.1 | 0.841094836 | 1.878431651 | 0.060322139 |
| DCXR | ENST00000586297.1 | ENSG00000267633.1 | 0.853456442 | 1.922474369 | 0.054546096 |
| DCXR | ENST00000586399.1 | ENSG00000228430.4 | 0.980062065 | 2.179305997 | 0.029308944 |
| DCXR | ENST00000589817.1 | ENSG00000231616.4 | 0.825710823 | 1.871885706 | 0.061222418 |
| DCXR | ENST00000590368.1 | ENSG00000231616.4 | 0.894667395 | 1.99577102 | 0.045958853 |
| DCXR | ENST00000590813.1 | ENSG00000231616.4 | 0.813553438 | 1.818622334 | 0.068969068 |
| DCXR | ENST00000590995.1 | ENSG00000267198.1 | 0.872912284 | 1.954755232 | 0.050611968 |
| DCXR | ENST00000591217.1 | ENSG00000231616.4 | 0.866477825 | 1.946183959 | 0.051632646 |
| DCXR | ENST00000592622.1 | ENSG00000267546.2 | -0.967247708 | -2.146792561 | 0.0318098 |
| DCXR | ENST00000593269.1 | ENSG00000236172.2 | 0.892076683 | 1.978934071 | 0.04782343 |
| DCXR | ENST00000594492.1 | ENSG00000250910.3 | 0.888242125 | 2.005804674 | 0.044877092 |
| DCXR | ENST00000596135.1 | ENSG00000269843.1 | 0.858322406 | 1.938373013 | 0.05257773 |
| DCXR | ENST00000596497.1 | ENSG00000268530.1 | 0.867278867 | 1.941595237 | 0.05218612 |
| DCXR | ENST00000598735.1 | ENSG00000268093.1 | 0.870726503 | 1.968350269 | 0.04902775 |
| DCXR | ENST00000600956.1 | ENSG00000232732.5 | -0.832486507 | -1.893656537 | 0.058270614 |
| DCXR | ENST00000601511.1 | ENSG00000244513.2 | 0.832389764 | 1.859162579 | 0.063004097 |
| DCXR | ENST00000602051.1 | ENSG00000227877.2 | -0.819935212 | -1.821756309 | 0.06849197 |
| DCXR | ENST00000602485.1 | ENSG00000270163.1 | -0.853912934 | -1.899772875 | 0.057462932 |
| DCXR | ENST00000602773.1 | ENSG00000270160.1 | -0.954344404 | -2.139611891 | 0.032386145 |
| DCXR | ENST00000606841.1 | ENSG00000272411.1 | 0.807204536 | 1.796518106 | 0.072412156 |
| DCXR | ENST00000606938.1 | ENSG00000272198.1 | -0.840657823 | -1.887110571 | 0.059145455 |
| DCXR | ENST00000607044.1 | ENSG00000272247.1 | 0.904088663 | 2.055426417 | 0.039837836 |
| DCXR | ENST00000607051.1 | ENSG00000271771.1 | 0.850138085 | 1.89881117 | 0.057589308 |
| DCXR | ENST00000607136.1 | ENSG00000267546.2 | -0.84809099 | -1.901407022 | 0.057248721 |
| DCXR | ENST00000607201.1 | ENSG00000272024.1 | -0.893037075 | -1.998825918 | 0.045627193 |
| DCXR | ENST00000607580.1 | ENSG00000272545.1 | -0.852730605 | -1.913800112 | 0.055645701 |
| DCXR | ENST00000609349.1 | ENSG00000272861.1 | 0.811895893 | 1.817360256 | 0.069161969 |
| DCXR | ENST00000609837.1 | ENSG00000273106.1 | 0.819394149 | 1.822695452 | 0.06834953 |
| DCXR | ENST00000609953.1 | ENSG00000272825.1 | 0.962398466 | 2.154825662 | 0.031175479 |
| DCXR | NR_002765.2 | ASAP1-IT1 | 0.809239667 | 1.822836971 | 0.068328086 |
| DCXR | NR_027401.2 | FAM223A | 0.930005609 | 2.0838602 | 0.037172886 |
| DCXR | NR_045637.1 | BOLA3-AS1 | 0.849312358 | 1.883799756 | 0.059592067 |
| DCXR | NR_046556.1 | RBMS3-AS1 | 0.817842006 | 1.823504773 | 0.068226975 |
| DCXR | NR_046578.1 | CACNA1C-AS4 | 0.908787032 | 2.037027507 | 0.041647286 |
| DCXR | NR_046766.1 | ATP2B2-IT2 | 0.853616244 | 1.877930596 | 0.06039066 |
| DCXR | NR_046839.1 | AGBL4-IT1 | -0.859004479 | -1.942494004 | 0.052077325 |
| DCXR | NR_047040.1 | LINC00424 | 0.931931484 | 2.085913163 | 0.03698649 |

| | | | | | |
|---|---|---|---|---|---|
| DCXR | NR_109975.1 | ARNTL2-AS1 | -0.891136986 | -2.017379018 | 0.043655975 |
| DCXR | NR_110007.1 | ADNP-AS1 | 0.923669209 | 2.067718719 | 0.038666481 |
| DCXR | NR_110008.1 | ADNP-AS1 | 0.818890834 | 1.823779003 | 0.068185489 |
| DCXR | NR_110009.1 | ADNP-AS1 | 0.818890834 | 1.81134915 | 0.070086822 |
| DCXR | NR_110053.1 | LOC101927464 | 0.921748248 | 2.077278473 | 0.037775865 |
| DCXR | NR_120527.1 | LOC100506675 | 0.901110457 | 1.995047196 | 0.046037733 |
| DCXR | NR_121661.1 | ZBTB20-AS5 | -0.857996131 | -1.940636325 | 0.052302405 |
| DCXR | NR_126334.1 | LOC101927932 | 0.822522722 | 1.845922953 | 0.064903393 |
| DCXR | NR_135076.1 | LOC102723838 | 0.908685526 | 1.997227142 | 0.045800514 |
| DCXR | NR_135820.1 | LOC102723727 | 0.871533816 | 1.94286273 | 0.052032746 |
| DEFA4 | ENST00000390540.2 | ENSG00000254140.1 | 0.897460231 | 1.998100439 | 0.045705772 |
| DEFA4 | ENST00000413969.1 | ENSG00000224189.2 | -0.833630157 | -1.881378243 | 0.059920484 |
| DEFA4 | ENST00000414992.1 | ENSG00000233613.1 | 0.807247934 | 1.813041462 | 0.069825427 |
| DEFA4 | ENST00000416220.1 | ENSG00000236753.1 | -0.86154465 | -1.956045342 | 0.050459813 |
| DEFA4 | ENST00000416641.1 | ENSG00000226956.1 | 0.829056949 | 1.856962392 | 0.063316505 |
| DEFA4 | ENST00000421006.1 | ENSG00000234548.1 | -0.840030412 | -1.883657733 | 0.059611287 |
| DEFA4 | ENST00000422763.1 | ENSG00000231131.2 | -0.848727214 | -1.899664073 | 0.057477218 |
| DEFA4 | ENST00000425881.1 | ENSG00000239636.1 | 0.814882971 | 1.814830855 | 0.069549907 |
| DEFA4 | ENST00000426213.1 | ENSG00000223660.1 | 0.963035847 | 2.150366012 | 0.031526275 |
| DEFA4 | ENST00000427132.1 | ENSG00000232121.1 | 0.840763822 | 1.907521263 | 0.05645312 |
| DEFA4 | ENST00000428440.1 | ENSG00000232827.2 | 0.828686619 | 1.844941553 | 0.065046041 |
| DEFA4 | ENST00000432431.1 | ENSG00000234940.1 | -0.912850801 | -2.029107849 | 0.042447307 |
| DEFA4 | ENST00000432957.1 | ENSG00000231534.1 | 0.92030925 | 2.021464953 | 0.043231657 |
| DEFA4 | ENST00000434493.1 | ENSG00000224605.1 | -0.802453281 | -1.794247791 | 0.072773627 |
| DEFA4 | ENST00000435832.1 | ENSG00000229201.1 | -0.897544695 | -2.034268514 | 0.04192453 |
| DEFA4 | ENST00000437680.1 | ENSG00000237133.1 | 0.980721273 | 2.199665168 | 0.02783066 |
| DEFA4 | ENST00000438850.1 | ENSG00000267338.1 | -0.92938728 | -2.056098818 | 0.039772992 |
| DEFA4 | ENST00000438969.2 | ENSG00000228031.2 | -0.812075174 | -1.807781757 | 0.070640474 |
| DEFA4 | ENST00000439455.1 | ENSG00000233482.1 | 0.8236435 | 1.820575395 | 0.068671426 |
| DEFA4 | ENST00000440862.1 | ENSG00000223804.1 | 0.846161645 | 1.88786936 | 0.059043491 |
| DEFA4 | ENST00000445631.1 | ENSG00000231052.1 | 0.8127083 | 1.825588329 | 0.067912295 |
| DEFA4 | ENST00000448001.1 | ENSG00000229639.1 | -0.971058758 | -2.150807432 | 0.031491402 |
| DEFA4 | ENST00000450206.1 | ENSG00000234311.1 | 0.910446027 | 2.040387042 | 0.041311791 |
| DEFA4 | ENST00000450500.1 | ENSG00000225790.1 | 0.935192491 | 2.101954305 | 0.035557279 |
| DEFA4 | ENST00000451575.2 | ENSG00000224251.2 | -0.835294996 | -1.846497692 | 0.064819974 |
| DEFA4 | ENST00000452553.1 | ENSG00000233973.1 | -0.88497946 | -1.98545911 | 0.047093415 |
| DEFA4 | ENST00000453878.1 | ENSG00000224850.1 | -0.869995382 | -1.937958546 | 0.05262828 |
| DEFA4 | ENST00000454515.1 | ENSG00000236753.1 | -0.84833569 | -1.853314545 | 0.063837288 |
| DEFA4 | ENST00000454709.1 | ENSG00000237280.1 | 0.818721107 | 1.833035679 | 0.066797265 |
| DEFA4 | ENST00000455416.1 | ENSG00000229337.1 | 0.982311633 | 2.229559543 | 0.025776698 |
| DEFA4 | ENST00000485347.1 | ENSG00000239991.1 | 0.887949697 | 1.987349681 | 0.04688366 |
| DEFA4 | ENST00000502684.1 | ENSG00000251670.1 | 0.848063841 | 1.911094195 | 0.055992477 |
| DEFA4 | ENST00000504344.1 | ENSG00000251438.1 | -0.80270348 | -1.796978718 | 0.072338998 |
| DEFA4 | ENST00000506058.1 | ENSG00000248261.1 | 0.84317845 | 1.889593687 | 0.058812322 |
| DEFA4 | ENST00000506059.1 | ENSG00000248311.1 | -0.920339908 | -2.085340527 | 0.037038401 |
| DEFA4 | ENST00000508081.1 | ENSG00000248254.1 | 0.960647822 | 2.131197852 | 0.033072846 |
| DEFA4 | ENST00000508845.1 | ENSG00000271724.1 | -0.819673658 | -1.830995584 | 0.067101202 |
| DEFA4 | ENST00000513023.1 | ENSG00000248809.1 | -0.962411201 | -2.159996285 | 0.030772957 |
| DEFA4 | ENST00000514459.1 | ENSG00000248211.1 | 0.885032333 | 1.983377992 | 0.047325224 |
| DEFA4 | ENST00000514802.1 | ENSG00000250190.1 | -0.940567548 | -2.104415143 | 0.03534225 |
| DEFA4 | ENST00000518416.1 | ENSG00000253901.1 | -0.940598826 | -2.115005108 | 0.034429512 |

| | | | | | |
|---|---|---|---|---|---|
| DEFA4 | ENST00000518620.1 | ENSG00000253892.1 | 0.821697653 | 1.822289453 | 0.068411078 |
| DEFA4 | ENST00000519660.1 | ENSG00000253416.1 | -0.856792059 | -1.908493836 | 0.056327419 |
| DEFA4 | ENST00000521378.1 | ENSG00000254222.1 | 0.965646242 | 2.140654089 | 0.032301943 |
| DEFA4 | ENST00000523806.1 | ENSG00000253616.1 | 0.818711391 | 1.836989329 | 0.066211471 |
| DEFA4 | ENST00000524133.1 | ENSG00000253174.2 | 0.857262424 | 1.919610941 | 0.054907061 |
| DEFA4 | ENST00000527100.1 | ENSG00000255015.1 | -0.923156624 | -2.055547672 | 0.039826136 |
| DEFA4 | ENST00000529837.1 | ENSG00000254687.1 | -0.814863515 | -1.827945362 | 0.067557752 |
| DEFA4 | ENST00000529875.1 | ENSG00000254404.1 | 0.877720262 | 1.949600746 | 0.051223724 |
| DEFA4 | ENST00000533938.1 | ENSG00000255142.1 | 0.836906892 | 1.846373094 | 0.064838051 |
| DEFA4 | ENST00000535315.1 | ENSG00000250748.2 | -0.889654949 | -2.011442729 | 0.044278714 |
| DEFA4 | ENST00000536529.1 | ENSG00000256422.1 | -0.863406305 | -1.940421956 | 0.05232843 |
| DEFA4 | ENST00000541391.1 | ENSG00000256268.1 | -0.819092184 | -1.820370286 | 0.068702634 |
| DEFA4 | ENST00000549329.1 | ENSG00000224189.2 | -0.803291386 | -1.782736334 | 0.074629234 |
| DEFA4 | ENST00000549616.1 | ENSG00000258168.1 | -0.865206522 | -1.968037079 | 0.049063772 |
| DEFA4 | ENST00000549756.1 | ENSG00000257769.1 | 0.924128089 | 2.063434243 | 0.039071389 |
| DEFA4 | ENST00000552634.1 | ENSG00000257496.1 | 0.806217825 | 1.805667229 | 0.070970334 |
| DEFA4 | ENST00000553464.1 | ENSG00000258418.1 | -0.808642981 | -1.795382297 | 0.072592811 |
| DEFA4 | ENST00000553477.1 | ENSG00000259123.1 | -0.909988057 | -2.050745274 | 0.040291759 |
| DEFA4 | ENST00000555913.1 | ENSG00000259077.1 | -0.891065132 | -1.972529625 | 0.048549178 |
| DEFA4 | ENST00000556458.1 | ENSG00000258504.2 | 0.886693757 | 1.993165526 | 0.046243325 |
| DEFA4 | ENST00000557368.1 | ENSG00000258444.1 | 0.943543488 | 2.107819314 | 0.035046622 |
| DEFA4 | ENST00000557903.1 | ENSG00000259182.1 | -0.843624005 | -1.882616184 | 0.059752401 |
| DEFA4 | ENST00000558221.1 | ENSG00000259704.1 | 0.8142823 | 1.813145933 | 0.069809316 |
| DEFA4 | ENST00000563601.1 | ENSG00000260589.1 | 0.815035234 | 1.817664136 | 0.069115482 |
| DEFA4 | ENST00000565623.1 | ENSG00000261118.1 | 0.817877215 | 1.814291062 | 0.069632927 |
| DEFA4 | ENST00000572417.1 | ENSG00000263171.1 | -0.890533112 | -1.976492475 | 0.048099027 |
| DEFA4 | ENST00000573315.1 | ENSG00000270168.1 | 0.802845731 | 1.782393338 | 0.074685111 |
| DEFA4 | ENST00000578035.1 | ENSG00000266743.1 | 0.928613551 | 2.078783491 | 0.037637255 |
| DEFA4 | ENST00000578349.1 | ENSG00000263688.1 | -0.829477657 | -1.859927392 | 0.062895799 |
| DEFA4 | ENST00000578572.1 | ENSG00000196295.7 | -0.865624559 | -1.939130084 | 0.0524855 |
| DEFA4 | ENST00000579775.1 | ENSG00000264108.1 | 0.824488753 | 1.845497387 | 0.064965218 |
| DEFA4 | ENST00000582348.1 | ENSG00000265148.1 | -0.818519234 | -1.833568852 | 0.066718019 |
| DEFA4 | ENST00000585761.1 | ENSG00000267198.1 | 0.814284833 | 1.838475331 | 0.065992394 |
| DEFA4 | ENST00000586348.1 | ENSG00000267198.1 | 0.810330401 | 1.806634127 | 0.070819345 |
| DEFA4 | ENST00000588177.1 | ENSG00000234899.5 | -0.881874433 | -1.964359109 | 0.049488461 |
| DEFA4 | ENST00000589380.1 | ENSG00000267488.1 | 0.897640904 | 2.00523204 | 0.044938246 |
| DEFA4 | ENST00000591621.1 | ENSG00000232116.2 | -0.828992682 | -1.864739296 | 0.062217956 |
| DEFA4 | ENST00000592022.1 | ENSG00000267383.2 | -0.820537726 | -1.863306641 | 0.062419136 |
| DEFA4 | ENST00000592431.1 | ENSG00000267475.1 | 0.87469051 | 1.923971041 | 0.054358213 |
| DEFA4 | ENST00000592498.1 | ENSG00000267488.1 | 0.884329124 | 1.982772496 | 0.047392848 |
| DEFA4 | ENST00000593486.1 | ENSG00000250910.3 | -0.828985056 | -1.862706976 | 0.062503504 |
| DEFA4 | ENST00000593599.1 | ENSG00000231898.4 | -0.831030401 | -1.866136223 | 0.062022309 |
| DEFA4 | ENST00000594762.1 | ENSG00000231898.4 | -0.856320371 | -1.918486634 | 0.055049335 |
| DEFA4 | ENST00000594850.1 | ENSG00000268093.1 | 0.839090669 | 1.895408401 | 0.058038317 |
| DEFA4 | ENST00000596971.1 | ENSG00000269463.1 | 0.801831163 | 1.797675905 | 0.072228382 |
| DEFA4 | ENST00000602435.1 | ENSG00000269952.1 | -0.813064934 | -1.816325807 | 0.069320409 |
| DEFA4 | ENST00000602507.1 | ENSG00000270069.1 | -0.820015475 | -1.824937754 | 0.068010423 |
| DEFA4 | ENST00000602790.1 | ENSG00000270000.1 | 0.91756428 | 2.040879007 | 0.041262855 |
| DEFA4 | ENST00000605692.1 | ENSG00000270810.1 | 0.844666456 | 1.899822584 | 0.057456406 |
| DEFA4 | ENST00000605780.1 | ENSG00000270755.1 | 0.896810718 | 2.009718601 | 0.04446098 |
| DEFA4 | ENST00000606757.1 | ENSG00000237188.3 | 0.880189426 | 1.94377456 | 0.051922643 |

| | | | | | |
|---|---|---|---|---|---|
| DEFA4 | ENST00000606898.1 | ENSG00000272094.1 | 0.938300664 | 2.106361283 | 0.035172982 |
| DEFA4 | ENST00000607769.1 | ENSG00000272438.1 | 0.834261566 | 1.851833557 | 0.064049727 |
| DEFA4 | ENST00000608289.1 | ENSG00000272958.1 | 0.857698349 | 1.927469363 | 0.053921163 |
| DEFA4 | ENST00000609789.1 | ENSG00000272707.1 | 0.803330427 | 1.802597107 | 0.071451512 |
| DEFA4 | ENST00000609890.1 | ENSG00000231898.4 | -0.89251882 | -2.019309146 | 0.043455098 |
| DEFA4 | ENST00000609934.1 | ENSG00000273271.1 | -0.88656852 | -1.990919821 | 0.046489703 |
| DEFA4 | ENST00000609952.1 | ENSG00000233766.3 | -0.875182884 | -1.988519666 | 0.046754246 |
| DEFA4 | NR_022011.1 | PWARSN | -0.873731111 | -1.957470088 | 0.050292225 |
| DEFA4 | NR_026822.1 | FAM138C | 0.831948975 | 1.857412183 | 0.063252534 |
| DEFA4 | NR_026932.1 | PDCD4-AS1 | 0.841225811 | 1.884255706 | 0.059530396 |
| DEFA4 | NR_033914.1 | LINC00254 | -0.809858254 | -1.786963142 | 0.073943446 |
| DEFA4 | NR_034131.1 | LINC00272 | 0.810590167 | 1.805272191 | 0.071032099 |
| DEFA4 | NR_040049.1 | SDCBP2-AS1 | 0.856229632 | 1.90032282 | 0.057390768 |
| DEFA4 | NR_040061.1 | SRP14-AS1 | 0.857834249 | 1.920064685 | 0.054849729 |
| DEFA4 | NR_102746.1 | ROPN1L-AS1 | 0.817776929 | 1.817584682 | 0.069127635 |
| DEFA4 | NR_104620.1 | LINC01672 | -0.902723328 | -2.015601705 | 0.043841641 |
| DEFA4 | NR_104998.1 | LOC102467225 | -0.843932372 | -1.919216017 | 0.054957001 |
| DEFA4 | NR_108046.1 | LINC00844 | -0.816124967 | -1.828170522 | 0.067523964 |
| DEFA4 | NR_110370.1 | STAM-AS1 | 0.809239794 | 1.79465777 | 0.072708243 |
| DEFA4 | NR_110559.1 | LOC101927023 | -0.810764887 | -1.820307061 | 0.068712257 |
| DEFA4 | NR_110824.1 | LINC01986 | -0.899828754 | -2.007784332 | 0.044666215 |
| DEFA4 | NR_126041.1 | LOC101930071 | 0.877995353 | 1.995677582 | 0.045969029 |
| DEFA4 | NR_131204.1 | XACT | 0.899514221 | 2.010482046 | 0.044380195 |
| DEFA4 | NR_134632.1 | LOC105373051 | 0.837116295 | 1.855631284 | 0.063506131 |
| DEFA4 | NR_135549.1 | LOC101929411 | -0.856251763 | -1.904255874 | 0.056876868 |
| DEFA4 | NR_135840.1 | LOC105376114 | -0.822342512 | -1.841286205 | 0.065579628 |
| DEFA4 | NR_136215.1 | VCAN-AS1 | -0.815180029 | -1.851104324 | 0.064154546 |
| DEFA4 | NR_136320.1 | LOC105373656 | 0.959115486 | 2.151358312 | 0.031447929 |
| DEFB107A | ENST00000362684.1 | ENSG00000228549.2 | 0.852962349 | 1.911013941 | 0.056002789 |
| DEFB107A | ENST00000412896.1 | ENSG00000197585.5 | 0.859686957 | 1.919185077 | 0.054960915 |
| DEFB107A | ENST00000413564.1 | ENSG00000224500.1 | 0.881666832 | 1.958537484 | 0.050166977 |
| DEFB107A | ENST00000413989.1 | ENSG00000242628.1 | -0.914940032 | -2.051894276 | 0.040179938 |
| DEFB107A | ENST00000414992.1 | ENSG00000233613.1 | 0.84328956 | 1.896560714 | 0.05788594 |
| DEFB107A | ENST00000417315.1 | ENSG00000242486.1 | 0.900429032 | 2.018490861 | 0.043540165 |
| DEFB107A | ENST00000420572.2 | ENSG00000233358.2 | -0.804244607 | -1.801949424 | 0.071553363 |
| DEFB107A | ENST00000421498.1 | ENSG00000237978.1 | -0.89550724 | -2.035997393 | 0.041750617 |
| DEFB107A | ENST00000421597.1 | ENSG00000227851.1 | -0.899232452 | -2.026882548 | 0.042674426 |
| DEFB107A | ENST00000422118.1 | ENSG00000231189.1 | 0.841792955 | 1.912528074 | 0.055808495 |
| DEFB107A | ENST00000423925.1 | ENSG00000223536.1 | 0.800505332 | 1.793344764 | 0.072917814 |
| DEFB107A | ENST00000424274.1 | ENSG00000232120.1 | -0.84080955 | -1.861268645 | 0.062706249 |
| DEFB107A | ENST00000424342.1 | ENSG00000234988.1 | 0.828055931 | 1.882581647 | 0.059757086 |
| DEFB107A | ENST00000425058.1 | ENSG00000226771.1 | -0.947523881 | -2.113824406 | 0.034530268 |
| DEFB107A | ENST00000426125.1 | ENSG00000223653.1 | -0.993163055 | -2.204087747 | 0.027518174 |
| DEFB107A | ENST00000426929.1 | ENSG00000230184.1 | 0.950644198 | 2.142773459 | 0.032131294 |
| DEFB107A | ENST00000427064.1 | ENSG00000238031.1 | -0.802895291 | -1.791983236 | 0.073135651 |
| DEFB107A | ENST00000429878.1 | ENSG00000224184.1 | 0.805677136 | 1.794443846 | 0.072742354 |
| DEFB107A | ENST00000429916.1 | ENSG00000227708.1 | -0.892387433 | -2.00951162 | 0.044482904 |
| DEFB107A | ENST00000430247.1 | ENSG00000232855.2 | -0.836591218 | -1.8858752 | 0.059311774 |
| DEFB107A | ENST00000430545.1 | ENSG00000237153.1 | -0.864039847 | -1.932996286 | 0.053236655 |
| DEFB107A | ENST00000430842.1 | ENSG00000230433.1 | 0.916902413 | 2.056233992 | 0.039759967 |
| DEFB107A | ENST00000433249.1 | ENSG00000236556.1 | -0.871622742 | -1.956766079 | 0.050374977 |

| | | | | | |
|---|---|---|---|---|---|
| DEFB107A | ENST00000434292.1 | ENSG00000229796.1 | -0.822352009 | -1.81802254 | 0.069060688 |
| DEFB107A | ENST00000434493.1 | ENSG00000224605.1 | -0.897471658 | -1.999290828 | 0.045576896 |
| DEFB107A | ENST00000435271.1 | ENSG00000231132.1 | 0.819455274 | 1.849790296 | 0.06434378 |
| DEFB107A | ENST00000435357.1 | ENSG00000225444.1 | -0.947062561 | -2.107419967 | 0.035081192 |
| DEFB107A | ENST00000435828.1 | ENSG00000235612.1 | -0.916769171 | -2.048903369 | 0.040471565 |
| DEFB107A | ENST00000436582.1 | ENSG00000236525.1 | 0.845794428 | 1.885172144 | 0.0594066 |
| DEFB107A | ENST00000439529.1 | ENSG00000236526.1 | -0.872879075 | -1.962647114 | 0.049687192 |
| DEFB107A | ENST00000441295.1 | ENSG00000233960.1 | 0.840615535 | 1.854652346 | 0.063645888 |
| DEFB107A | ENST00000441532.1 | ENSG00000234206.1 | 0.812948244 | 1.834994962 | 0.066506436 |
| DEFB107A | ENST00000441666.1 | ENSG00000230379.1 | -0.811208058 | -1.826068957 | 0.067839876 |
| DEFB107A | ENST00000441991.1 | ENSG00000231210.2 | 0.826728009 | 1.843807529 | 0.065211195 |
| DEFB107A | ENST00000442852.1 | ENSG00000237923.1 | 0.910549376 | 2.04563373 | 0.04079242 |
| DEFB107A | ENST00000443066.2 | ENSG00000237633.2 | -0.883625148 | -1.969481782 | 0.048897793 |
| DEFB107A | ENST00000443162.1 | ENSG00000234183.1 | -0.965479638 | -2.141566426 | 0.032228388 |
| DEFB107A | ENST00000444731.1 | ENSG00000227131.1 | 0.922248051 | 2.056445078 | 0.039739635 |
| DEFB107A | ENST00000445178.1 | ENSG00000234653.1 | -0.985804338 | -2.213181699 | 0.026885121 |
| DEFB107A | ENST00000445233.1 | ENSG00000233928.1 | -0.845184919 | -1.901477459 | 0.057239502 |
| DEFB107A | ENST00000445617.2 | ENSG00000225751.2 | -0.855870441 | -1.898710408 | 0.057602562 |
| DEFB107A | ENST00000448086.1 | ENSG00000237571.1 | 0.892387433 | 2.01636525 | 0.043761796 |
| DEFB107A | ENST00000449903.1 | ENSG00000223872.1 | 0.985688864 | 2.224154315 | 0.026138064 |
| DEFB107A | ENST00000451267.1 | ENSG00000230410.1 | 0.810449967 | 1.817585252 | 0.069127547 |
| DEFB107A | ENST00000451828.1 | ENSG00000228549.2 | 0.808046103 | 1.8033407 | 0.071334724 |
| DEFB107A | ENST00000452511.1 | ENSG00000231876.3 | -0.827301888 | -1.860095205 | 0.062872057 |
| DEFB107A | ENST00000453584.1 | ENSG00000233613.1 | 0.956398864 | 2.12768335 | 0.033363346 |
| DEFB107A | ENST00000453878.1 | ENSG00000224850.1 | -0.848226785 | -1.901560946 | 0.057228578 |
| DEFB107A | ENST00000454182.1 | ENSG00000230379.1 | -0.83203391 | -1.871995629 | 0.061207208 |
| DEFB107A | ENST00000454387.1 | ENSG00000223726.1 | 0.864868373 | 1.916952412 | 0.055243978 |
| DEFB107A | ENST00000456999.1 | ENSG00000230690.1 | -0.87058079 | -1.932891893 | 0.053249517 |
| DEFB107A | ENST00000457043.1 | ENSG00000231365.1 | -0.845828099 | -1.883968645 | 0.059569217 |
| DEFB107A | ENST00000457632.1 | ENSG00000234248.1 | -0.843742409 | -1.900974734 | 0.057305322 |
| DEFB107A | ENST00000458082.1 | ENSG00000231210.2 | 0.829070538 | 1.855522111 | 0.063521705 |
| DEFB107A | ENST00000477643.1 | ENSG00000241224.2 | 0.904136603 | 2.019991553 | 0.043384264 |
| DEFB107A | ENST00000479039.1 | ENSG00000241224.2 | 0.948180551 | 2.143262868 | 0.032091997 |
| DEFB107A | ENST00000479233.1 | ENSG00000243150.1 | -0.821932254 | -1.861533064 | 0.062668936 |
| DEFB107A | ENST00000482142.1 | ENSG00000243276.1 | -0.894280927 | -2.019021856 | 0.043484948 |
| DEFB107A | ENST00000483283.1 | ENSG00000240571.1 | -0.846916475 | -1.910461588 | 0.056073808 |
| DEFB107A | ENST00000488040.1 | ENSG00000243176.1 | -0.882688796 | -1.980765348 | 0.047617594 |
| DEFB107A | ENST00000500498.2 | ENSG00000245311.2 | 0.881681157 | 1.951112733 | 0.051043637 |
| DEFB107A | ENST00000503323.1 | ENSG00000249881.1 | 0.801537807 | 1.802108572 | 0.071528325 |
| DEFB107A | ENST00000503470.1 | ENSG00000248559.1 | -0.927145407 | -2.073017946 | 0.038170609 |
| DEFB107A | ENST00000503987.1 | ENSG00000250075.1 | -0.908863944 | -2.026010174 | 0.042763742 |
| DEFB107A | ENST00000505404.1 | ENSG00000249941.1 | 0.975014714 | 2.165531199 | 0.030347029 |
| DEFB107A | ENST00000505575.1 | ENSG00000248939.1 | 0.863884497 | 1.944345007 | 0.051853861 |
| DEFB107A | ENST00000505978.1 | ENSG00000249982.1 | 0.878178 | 1.964145226 | 0.049513253 |
| DEFB107A | ENST00000506305.1 | ENSG00000249994.1 | 0.802511868 | 1.811388921 | 0.07008067 |
| DEFB107A | ENST00000506379.1 | ENSG00000240152.2 | -0.902206274 | -2.033950972 | 0.041956539 |
| DEFB107A | ENST00000506420.1 | ENSG00000250034.1 | 0.864502051 | 1.930369123 | 0.053561118 |
| DEFB107A | ENST00000506852.1 | ENSG00000250945.1 | -0.954765316 | -2.117886404 | 0.034184689 |
| DEFB107A | ENST00000507558.1 | ENSG00000248445.1 | -0.849436282 | -1.861696434 | 0.062645891 |
| DEFB107A | ENST00000509629.1 | ENSG00000250164.1 | 0.849924142 | 1.904058111 | 0.056902616 |
| DEFB107A | ENST00000509983.1 | ENSG00000248173.1 | 0.884153856 | 1.988406943 | 0.046766702 |

| | | | | | |
|---|---|---|---|---|---|
| DEFB107A | ENST00000510001.2 | ENSG00000249196.2 | 0.927363895 | 2.07069752 | 0.038387072 |
| DEFB107A | ENST00000510198.1 | ENSG00000248733.1 | -0.807580245 | -1.789552349 | 0.073525905 |
| DEFB107A | ENST00000510602.1 | ENSG00000249122.1 | 0.912075068 | 2.029144666 | 0.042443558 |
| DEFB107A | ENST00000511603.1 | ENSG00000249892.1 | -0.915369306 | -2.036134145 | 0.041736887 |
| DEFB107A | ENST00000514459.1 | ENSG00000248211.1 | 0.810870221 | 1.810408013 | 0.070232538 |
| DEFB107A | ENST00000518260.1 | ENSG00000253628.1 | 0.868732654 | 1.962297732 | 0.049727831 |
| DEFB107A | ENST00000518620.1 | ENSG00000253892.1 | 0.88809263 | 1.980953477 | 0.047596491 |
| DEFB107A | ENST00000518894.1 | ENSG00000204758.3 | -0.855217573 | -1.92635884 | 0.054059583 |
| DEFB107A | ENST00000519005.1 | ENSG00000253507.1 | -0.804092697 | -1.806094035 | 0.070903652 |
| DEFB107A | ENST00000519375.1 | ENSG00000253980.1 | -0.967275104 | -2.169957639 | 0.030010055 |
| DEFB107A | ENST00000519844.1 | ENSG00000253824.1 | 0.941583634 | 2.107823327 | 0.035046274 |
| DEFB107A | ENST00000520192.1 | ENSG00000253807.1 | -0.802001892 | -1.778440575 | 0.075331524 |
| DEFB107A | ENST00000520849.1 | ENSG00000253553.1 | -0.851395315 | -1.891847617 | 0.058511287 |
| DEFB107A | ENST00000521490.1 | ENSG00000253407.1 | 0.921778886 | 2.050348184 | 0.040330466 |
| DEFB107A | ENST00000521725.1 | ENSG00000253396.1 | -0.802281629 | -1.787162968 | 0.073911153 |
| DEFB107A | ENST00000522190.1 | ENSG00000254165.1 | -0.936426646 | -2.080224594 | 0.037504937 |
| DEFB107A | ENST00000524133.1 | ENSG00000253174.2 | 0.908313918 | 2.041055398 | 0.041245321 |
| DEFB107A | ENST00000525855.1 | ENSG00000254746.1 | 0.921402605 | 2.052429612 | 0.040127929 |
| DEFB107A | ENST00000528869.1 | ENSG00000255443.1 | -0.824412643 | -1.859865436 | 0.062904567 |
| DEFB107A | ENST00000531071.1 | ENSG00000255248.2 | 0.837519398 | 1.861460861 | 0.062679123 |
| DEFB107A | ENST00000533101.1 | ENSG00000255311.1 | -0.877366246 | -1.944217967 | 0.051869172 |
| DEFB107A | ENST00000533938.1 | ENSG00000255142.1 | 0.909002733 | 2.023631961 | 0.043008034 |
| DEFB107A | ENST00000538294.1 | ENSG00000250748.2 | 0.888931409 | 1.993833103 | 0.046170297 |
| DEFB107A | ENST00000545158.1 | ENSG00000256011.1 | -0.880412584 | -1.95827661 | 0.050197564 |
| DEFB107A | ENST00000546135.1 | ENSG00000256670.1 | 0.930387081 | 2.077224108 | 0.03778088 |
| DEFB107A | ENST00000548748.1 | ENSG00000258252.1 | 0.941510133 | 2.103985903 | 0.035379677 |
| DEFB107A | ENST00000549616.1 | ENSG00000258168.1 | -0.800752478 | -1.760001517 | 0.078407549 |
| DEFB107A | ENST00000550805.1 | ENSG00000244306.5 | 0.93309363 | 2.076870805 | 0.037813485 |
| DEFB107A | ENST00000551067.1 | ENSG00000257891.1 | 0.867085523 | 1.944569386 | 0.051826827 |
| DEFB107A | ENST00000552634.1 | ENSG00000257496.1 | 0.84702897 | 1.861892126 | 0.062618297 |
| DEFB107A | ENST00000553348.1 | ENSG00000258829.1 | 0.896468864 | 1.998279597 | 0.045686356 |
| DEFB107A | ENST00000553537.1 | ENSG00000258481.1 | 0.886694474 | 1.992898711 | 0.046272539 |
| DEFB107A | ENST00000553668.1 | ENSG00000258733.1 | -0.869331008 | -1.928577123 | 0.053783381 |
| DEFB107A | ENST00000553954.1 | ENSG00000259052.1 | 0.876128151 | 1.955018334 | 0.050580906 |
| DEFB107A | ENST00000555689.1 | ENSG00000259049.1 | 0.865092745 | 1.921405772 | 0.054680571 |
| DEFB107A | ENST00000556145.1 | ENSG00000258829.1 | 0.947275923 | 2.078296642 | 0.037682046 |
| DEFB107A | ENST00000556458.1 | ENSG00000258504.2 | 0.854889474 | 1.932469209 | 0.053301619 |
| DEFB107A | ENST00000556978.1 | ENSG00000258693.1 | -0.919867308 | -2.039310905 | 0.041419008 |
| DEFB107A | ENST00000558141.1 | ENSG00000259594.1 | -0.845371994 | -1.864859006 | 0.06220117 |
| DEFB107A | ENST00000559041.1 | ENSG00000259713.1 | -0.868338025 | -1.918350933 | 0.055066528 |
| DEFB107A | ENST00000560969.1 | ENSG00000259176.1 | -0.958420884 | -2.125579393 | 0.033538297 |
| DEFB107A | ENST00000561254.1 | ENSG00000259554.1 | -0.800602652 | -1.806844886 | 0.070786468 |
| DEFB107A | ENST00000561847.1 | ENSG00000260293.1 | 0.916266754 | 2.042283823 | 0.041123386 |
| DEFB107A | ENST00000563342.1 | ENSG00000259914.1 | -0.838576685 | -1.865751553 | 0.062076133 |
| DEFB107A | ENST00000563601.1 | ENSG00000260589.1 | 0.845956068 | 1.906181652 | 0.056626642 |
| DEFB107A | ENST00000565271.1 | ENSG00000261335.1 | 0.937292318 | 2.083027082 | 0.037248755 |
| DEFB107A | ENST00000565623.1 | ENSG00000261118.1 | 0.888137415 | 1.971266683 | 0.04869338 |
| DEFB107A | ENST00000565944.1 | ENSG00000260331.1 | -0.86348247 | -1.921033385 | 0.054727499 |
| DEFB107A | ENST00000569215.1 | ENSG00000260756.1 | -0.815000214 | -1.796386598 | 0.072433054 |
| DEFB107A | ENST00000569328.1 | ENSG00000261638.1 | -0.880006449 | -1.96980448 | 0.048860783 |
| DEFB107A | ENST00000569778.1 | ENSG00000260823.1 | 0.818457937 | 1.832828663 | 0.066828055 |

| | | | | | |
|---|---|---|---|---|---|
| DEFB107A | ENST00000570700.1 | ENSG00000263011.1 | 0.887590294 | 1.985666744 | 0.04707034 |
| DEFB107A | ENST00000572608.1 | ENSG00000263305.1 | -0.804855158 | -1.793353499 | 0.072916418 |
| DEFB107A | ENST00000576271.1 | ENSG00000263342.1 | 0.809136852 | 1.800494865 | 0.071782534 |
| DEFB107A | ENST00000577360.1 | ENSG00000264273.1 | -0.823640169 | -1.836459602 | 0.066289712 |
| DEFB107A | ENST00000582895.1 | ENSG00000264729.1 | 0.960063875 | 2.118780868 | 0.03410899 |
| DEFB107A | ENST00000586297.1 | ENSG00000267633.1 | -0.856947615 | -1.917132991 | 0.055221038 |
| DEFB107A | ENST00000587049.1 | ENSG00000235535.3 | 0.935350058 | 2.111695172 | 0.034712603 |
| DEFB107A | ENST00000588177.1 | ENSG00000234899.5 | -0.841279164 | -1.90441828 | 0.05685573 |
| DEFB107A | ENST00000590046.1 | ENSG00000266950.1 | 0.818034267 | 1.841840134 | 0.065498537 |
| DEFB107A | ENST00000591137.1 | ENSG00000267405.1 | -0.837418021 | -1.88039413 | 0.060054383 |
| DEFB107A | ENST00000592431.1 | ENSG00000267475.1 | 0.864139643 | 1.900553353 | 0.05736054 |
| DEFB107A | ENST00000592523.1 | ENSG00000226994.3 | -0.94580751 | -2.129109958 | 0.033245164 |
| DEFB107A | ENST00000595007.1 | ENSG00000231876.3 | -0.849011381 | -1.899096852 | 0.057551743 |
| DEFB107A | ENST00000597680.1 | ENSG00000269574.1 | -0.804415795 | -1.793413138 | 0.072906888 |
| DEFB107A | ENST00000597755.1 | ENSG00000236194.2 | -0.857083598 | -1.938305714 | 0.052585936 |
| DEFB107A | ENST00000598065.1 | ENSG00000231731.3 | 0.844221586 | 1.875621801 | 0.060707231 |
| DEFB107A | ENST00000598131.1 | ENSG00000269043.1 | -0.826116051 | -1.85534778 | 0.063546579 |
| DEFB107A | ENST00000598356.1 | ENSG00000269640.1 | -0.874641341 | -1.985505908 | 0.047088213 |
| DEFB107A | ENST00000598887.1 | ENSG00000268475.1 | 0.910053111 | 2.034056973 | 0.041945851 |
| DEFB107A | ENST00000598950.1 | ENSG00000269736.1 | -0.834235361 | -1.835943306 | 0.066366042 |
| DEFB107A | ENST00000599050.1 | ENSG00000268366.1 | -0.86391072 | -1.972899401 | 0.048507025 |
| DEFB107A | ENST00000599387.1 | ENSG00000227733.4 | -0.874834243 | -1.95436779 | 0.050657737 |
| DEFB107A | ENST00000600848.1 | ENSG00000228065.6 | -0.813035372 | -1.793378254 | 0.072912462 |
| DEFB107A | ENST00000600959.1 | ENSG00000269303.1 | -0.82082133 | -1.810455585 | 0.070225166 |
| DEFB107A | ENST00000602405.1 | ENSG00000269928.1 | 0.899300561 | 1.978879238 | 0.047829604 |
| DEFB107A | ENST00000602443.1 | ENSG00000270076.1 | -0.948339548 | -2.107830068 | 0.035045691 |
| DEFB107A | ENST00000602900.1 | ENSG00000270179.1 | 0.801853219 | 1.775130613 | 0.075876323 |
| DEFB107A | ENST00000603612.1 | ENSG00000270996.1 | -0.907605128 | -1.998480717 | 0.045664568 |
| DEFB107A | ENST00000603949.1 | ENSG00000270332.1 | -0.864957925 | -1.939004826 | 0.052500751 |
| DEFB107A | ENST00000604312.1 | ENSG00000270947.1 | -0.860437084 | -1.928027778 | 0.053851671 |
| DEFB107A | ENST00000605021.1 | ENSG00000271401.1 | -0.901646124 | -1.989486584 | 0.046647521 |
| DEFB107A | ENST00000606482.1 | ENSG00000272416.1 | -0.857257146 | -1.907696603 | 0.056430441 |
| DEFB107A | ENST00000606898.1 | ENSG00000272094.1 | 0.812471754 | 1.839430837 | 0.065851842 |
| DEFB107A | ENST00000606921.1 | ENSG00000272402.1 | 0.845184919 | 1.881299201 | 0.05993123 |
| DEFB107A | ENST00000606938.1 | ENSG00000272198.1 | 0.817520915 | 1.797523129 | 0.07225261 |
| DEFB107A | ENST00000607136.1 | ENSG00000267546.2 | 0.806706028 | 1.786515834 | 0.074015776 |
| DEFB107A | ENST00000607740.1 | ENSG00000271916.1 | -0.830016833 | -1.848245214 | 0.064566877 |
| DEFB107A | ENST00000608133.1 | ENSG00000273193.1 | -0.941218941 | -2.114770448 | 0.034449517 |
| DEFB107A | ENST00000608173.1 | ENSG00000197099.4 | -0.844968568 | -1.899954267 | 0.057439121 |
| DEFB107A | ENST00000608289.1 | ENSG00000272958.1 | 0.907338578 | 2.006878488 | 0.044762603 |
| DEFB107A | ENST00000609182.1 | ENSG00000273248.1 | 0.916038167 | 2.031176124 | 0.042237131 |
| DEFB107A | ENST00000609238.1 | ENSG00000272703.1 | 0.944829968 | 2.109487386 | 0.034902534 |
| DEFB107A | ENST00000609955.1 | ENSG00000273275.1 | -0.833785596 | -1.85863766 | 0.063078515 |
| DEFB107A | NR_002765.2 | ASAP1-IT1 | -0.849855373 | -1.889153213 | 0.058871302 |
| DEFB107A | NR_027067.1 | LINC00114 | 0.864145729 | 1.925763464 | 0.054133915 |
| DEFB107A | NR_027425.1 | FAM66D | 0.842385035 | 1.877634811 | 0.06043114 |
| DEFB107A | NR_046556.1 | RBMS3-AS1 | -0.939448686 | -2.123369336 | 0.033722915 |
| DEFB107A | NR_046748.1 | ARHGAP31-AS1 | -0.844840598 | -1.897089392 | 0.057816141 |
| DEFB107A | NR_102737.1 | LINC00911 | 0.844329597 | 1.893770743 | 0.058255447 |
| DEFB107A | NR_102738.1 | LINC00911 | 0.923309203 | 2.087166994 | 0.036873042 |
| DEFB107A | NR_102746.1 | ROPN1L-AS1 | 0.820325004 | 1.822434344 | 0.068389108 |

| | | | | | |
|---|---|---|---|---|---|
| DEFB107A | NR_109975.1 | ARNTL2-AS1 | 0.860378112 | 1.915472667 | 0.055432252 |
| DEFB107A | NR_110007.1 | ADNP-AS1 | -0.811845941 | -1.826450318 | 0.067782458 |
| DEFB107A | NR_110284.1 | LOC101927907 | -0.985804338 | -2.20414167 | 0.027514382 |
| DEFB107A | NR_110504.1 | LOC101929572 | 0.958006442 | 2.121213285 | 0.033903858 |
| DEFB107A | NR_120330.1 | LOC101928227 | 0.812230758 | 1.80177391 | 0.071580984 |
| DEFB107A | NR_120466.1 | LINC01489 | -0.930286191 | -2.092135999 | 0.036426352 |
| DEFB107A | NR_125407.1 | LOC102724604 | -0.820263698 | -1.840609616 | 0.065678787 |
| DEFB107A | NR_125769.1 | LINC01269 | 0.941628526 | 2.111511589 | 0.034728363 |
| DEFB107A | NR_125925.1 | LOC101929448 | -0.830016833 | -1.884275204 | 0.05952776 |
| DEFB107A | NR_126041.1 | LOC101930071 | 0.839237221 | 1.859959969 | 0.06289119 |
| DEFB107A | NR_126354.1 | LINC01331 | 0.853041699 | 1.888293228 | 0.058986596 |
| DEFB107A | NR_126370.1 | GACAT1 | 0.801026901 | 1.782501022 | 0.074667565 |
| DEFB107A | NR_126409.1 | LINC00376 | -0.89462974 | -2.00364296 | 0.045108321 |
| DEFB107A | NR_130916.1 | LOC105274304 | 0.949053308 | 2.13229354 | 0.032982723 |
| DEFB107A | NR_131243.1 | SMCR2 | 0.926682873 | 2.078977857 | 0.037619386 |
| DEFB107A | NR_133907.1 | HLA-DQB1-AS1 | -0.857712789 | -1.941829105 | 0.052157792 |
| DEFB107A | NR_134610.1 | LOC105375014 | 0.907121904 | 2.020518215 | 0.043329663 |
| DEFB107A | NR_134632.1 | LOC105373051 | 0.839381742 | 1.894633233 | 0.058141009 |
| DEFB107A | NR_135076.1 | LOC102723838 | -0.872643141 | -1.932293274 | 0.053323318 |
| DEFB107A | NR_135239.1 | LINC01867 | -0.947768723 | -2.145034633 | 0.031950078 |
| DEFB107A | NR_136218.1 | MEF2C-AS1 | -0.800580979 | -1.797295675 | 0.072288692 |
| DEFB116 | ENST00000423925.1 | ENSG00000223536.1 | 0.912644048 | 2.017219544 | 0.043672607 |
| DEFB116 | ENST00000425364.1 | ENSG00000231046.1 | 0.808038229 | 1.807316242 | 0.070712985 |
| DEFB116 | ENST00000426213.1 | ENSG00000223660.1 | 0.840582254 | 1.866190083 | 0.062014776 |
| DEFB116 | ENST00000426504.1 | ENSG00000234190.1 | 0.841191206 | 1.860682121 | 0.06278908 |
| DEFB116 | ENST00000427132.1 | ENSG00000232121.1 | 0.913991968 | 2.051187034 | 0.040248736 |
| DEFB116 | ENST00000428440.1 | ENSG00000232827.2 | 0.807651602 | 1.789966642 | 0.073459274 |
| DEFB116 | ENST00000429878.1 | ENSG00000224184.1 | 0.917302381 | 2.038735684 | 0.041476414 |
| DEFB116 | ENST00000431290.1 | ENSG00000183822.2 | -0.807970631 | -1.793842149 | 0.072838367 |
| DEFB116 | ENST00000434250.1 | ENSG00000234055.1 | 0.865711452 | 1.957199175 | 0.050324056 |
| DEFB116 | ENST00000434493.1 | ENSG00000224605.1 | -0.825949018 | -1.866707768 | 0.061942408 |
| DEFB116 | ENST00000435357.1 | ENSG00000225444.1 | -0.840535794 | -1.85310348 | 0.063867528 |
| DEFB116 | ENST00000437334.1 | ENSG00000226134.1 | 0.904525294 | 2.010980836 | 0.044327481 |
| DEFB116 | ENST00000439443.1 | ENSG00000236911.2 | 0.815600999 | 1.842353808 | 0.065423414 |
| DEFB116 | ENST00000439455.1 | ENSG00000233482.1 | 0.920890206 | 2.06338303 | 0.039076251 |
| DEFB116 | ENST00000440518.1 | ENSG00000226571.1 | 0.95034645 | 2.110217753 | 0.034839604 |
| DEFB116 | ENST00000440862.1 | ENSG00000223804.1 | 0.943950439 | 2.123860425 | 0.033681816 |
| DEFB116 | ENST00000444731.1 | ENSG00000227131.1 | 0.835159387 | 1.858402525 | 0.063111874 |
| DEFB116 | ENST00000445617.2 | ENSG00000225751.2 | -0.895702207 | -1.994880129 | 0.046055955 |
| DEFB116 | ENST00000445631.1 | ENSG00000231052.1 | 0.983660085 | 2.207883355 | 0.027252405 |
| DEFB116 | ENST00000448650.1 | ENSG00000223536.1 | 0.925978834 | 2.045988939 | 0.040757459 |
| DEFB116 | ENST00000449586.1 | ENSG00000235257.4 | 0.886649762 | 1.996144763 | 0.045918168 |
| DEFB116 | ENST00000450500.1 | ENSG00000225790.1 | 0.862919685 | 1.959947185 | 0.050001964 |
| DEFB116 | ENST00000452002.1 | ENSG00000236501.1 | 0.858513882 | 1.921629095 | 0.054652445 |
| DEFB116 | ENST00000452553.1 | ENSG00000233973.1 | -0.86257451 | -1.899497284 | 0.057499123 |
| DEFB116 | ENST00000453878.1 | ENSG00000224850.1 | -0.849001878 | -1.916664259 | 0.055280599 |
| DEFB116 | ENST00000454709.1 | ENSG00000237280.1 | 0.917029326 | 2.072517985 | 0.038217161 |
| DEFB116 | ENST00000455416.1 | ENSG00000229337.1 | 0.849442687 | 1.928700248 | 0.053768085 |
| DEFB116 | ENST00000457602.1 | ENSG00000237576.1 | 0.826717072 | 1.833666083 | 0.066703576 |
| DEFB116 | ENST00000479233.1 | ENSG00000243150.1 | -0.879926495 | -1.951722804 | 0.050971124 |
| DEFB116 | ENST00000482142.1 | ENSG00000243276.1 | -0.888498987 | -2.032738365 | 0.042078963 |

| | | | | | |
|---|---|---|---|---|---|
| DEFB116 | ENST00000485347.1 | ENSG00000239991.1 | 0.890018606 | 2.003338274 | 0.045140992 |
| DEFB116 | ENST00000503938.1 | ENSG00000246095.2 | -0.84545228 | -1.893068275 | 0.05834879 |
| DEFB116 | ENST00000507808.1 | ENSG00000250333.1 | 0.895498496 | 2.004943331 | 0.044969106 |
| DEFB116 | ENST00000508845.1 | ENSG00000271724.1 | -0.882992529 | -1.993506818 | 0.046205978 |
| DEFB116 | ENST00000509983.1 | ENSG00000248173.1 | 0.893040113 | 1.994798438 | 0.046064868 |
| DEFB116 | ENST00000511234.1 | ENSG00000250865.1 | 0.820205743 | 1.838234025 | 0.066027928 |
| DEFB116 | ENST00000511603.1 | ENSG00000249892.1 | -0.815006994 | -1.833187994 | 0.066774619 |
| DEFB116 | ENST00000514459.1 | ENSG00000248211.1 | 0.89530464 | 1.998672564 | 0.045643793 |
| DEFB116 | ENST00000518260.1 | ENSG00000253628.1 | 0.816260527 | 1.821136538 | 0.068586104 |
| DEFB116 | ENST00000518416.1 | ENSG00000253901.1 | -0.817477342 | -1.808928567 | 0.070462102 |
| DEFB116 | ENST00000518620.1 | ENSG00000253892.1 | 0.869670675 | 1.937282263 | 0.052710849 |
| DEFB116 | ENST00000519038.2 | ENSG00000254054.2 | 0.8969562 | 2.008639255 | 0.044575405 |
| DEFB116 | ENST00000519660.1 | ENSG00000253416.1 | -0.870678345 | -1.945709592 | 0.051689634 |
| DEFB116 | ENST00000521359.1 | ENSG00000253140.1 | -0.852975853 | -1.927396054 | 0.053930291 |
| DEFB116 | ENST00000522408.1 | ENSG00000253484.1 | -0.808283966 | -1.797768969 | 0.072213627 |
| DEFB116 | ENST00000523703.1 | ENSG00000214803.3 | 0.813867135 | 1.864628718 | 0.062233465 |
| DEFB116 | ENST00000524133.1 | ENSG00000253174.2 | 0.830882906 | 1.864188214 | 0.062295278 |
| DEFB116 | ENST00000524309.1 | ENSG00000240915.2 | 0.855788738 | 1.910742985 | 0.056037618 |
| DEFB116 | ENST00000528869.1 | ENSG00000255443.1 | -0.867799463 | -1.9488057 | 0.051318633 |
| DEFB116 | ENST00000531071.1 | ENSG00000255248.2 | 0.860665477 | 1.915401562 | 0.055441312 |
| DEFB116 | ENST00000531087.1 | ENSG00000254428.1 | 0.894728042 | 2.014510597 | 0.043955953 |
| DEFB116 | ENST00000533938.1 | ENSG00000255142.1 | 0.903299711 | 2.047409265 | 0.040617917 |
| DEFB116 | ENST00000538294.1 | ENSG00000250748.2 | 0.925762374 | 2.113995892 | 0.034515618 |
| DEFB116 | ENST00000548748.1 | ENSG00000258252.1 | 0.859742472 | 1.899985447 | 0.05743503 |
| DEFB116 | ENST00000549756.1 | ENSG00000257769.1 | 0.907752022 | 2.028994389 | 0.042458862 |
| DEFB116 | ENST00000552378.1 | ENSG00000257294.1 | 0.933770979 | 2.104520758 | 0.035333046 |
| DEFB116 | ENST00000553464.1 | ENSG00000258418.1 | -0.946599707 | -2.099746116 | 0.035751181 |
| DEFB116 | ENST00000553668.1 | ENSG00000258733.1 | -0.916052831 | -2.018517442 | 0.0435374 |
| DEFB116 | ENST00000557855.1 | ENSG00000259176.1 | -0.904289735 | -2.017580102 | 0.043635011 |
| DEFB116 | ENST00000560193.1 | ENSG00000259176.1 | -0.876489389 | -1.932685223 | 0.053274987 |
| DEFB116 | ENST00000563601.1 | ENSG00000260589.1 | 0.862415311 | 1.95647456 | 0.050409276 |
| DEFB116 | ENST00000565623.1 | ENSG00000261118.1 | 0.851599774 | 1.933398572 | 0.053187117 |
| DEFB116 | ENST00000566639.1 | ENSG00000261061.1 | 0.911861828 | 2.003212409 | 0.045154494 |
| DEFB116 | ENST00000569147.1 | ENSG00000261592.1 | 0.910009452 | 2.026364982 | 0.042727397 |
| DEFB116 | ENST00000569215.1 | ENSG00000260756.1 | -0.906007248 | -2.043084989 | 0.041044026 |
| DEFB116 | ENST00000586338.1 | ENSG00000219410.4 | 0.877758948 | 1.947411186 | 0.051485458 |
| DEFB116 | ENST00000587850.1 | ENSG00000267683.1 | 0.868865906 | 1.950377395 | 0.051131154 |
| DEFB116 | ENST00000588177.1 | ENSG00000234899.5 | -0.802851777 | -1.803425935 | 0.071321348 |
| DEFB116 | ENST00000589281.1 | ENSG00000267707.1 | 0.819169306 | 1.833273406 | 0.066761922 |
| DEFB116 | ENST00000593967.1 | ENSG00000232732.5 | 0.831011369 | 1.855748489 | 0.063489416 |
| DEFB116 | ENST00000597420.1 | ENSG00000269564.1 | 0.915697188 | 2.017639456 | 0.043628824 |
| DEFB116 | ENST00000599572.1 | ENSG00000233783.3 | 0.868737731 | 1.930614499 | 0.053530743 |
| DEFB116 | ENST00000600848.1 | ENSG00000228065.6 | -0.828383564 | -1.834049535 | 0.066646642 |
| DEFB116 | ENST00000602790.1 | ENSG00000270000.1 | 0.863786644 | 1.908647156 | 0.056307624 |
| DEFB116 | ENST00000603948.1 | ENSG00000222041.6 | 0.828108601 | 1.895982412 | 0.05796237 |
| DEFB116 | ENST00000606757.1 | ENSG00000237188.3 | 0.92241622 | 2.047411328 | 0.040617715 |
| DEFB116 | ENST00000606921.1 | ENSG00000272402.1 | 0.87042346 | 1.97093989 | 0.048730752 |
| DEFB116 | ENST00000607598.1 | ENSG00000272267.1 | 0.840596078 | 1.870792315 | 0.061373873 |
| DEFB116 | ENST00000608289.1 | ENSG00000272958.1 | 0.854011869 | 1.901046949 | 0.057295863 |
| DEFB116 | ENST00000608422.1 | ENSG00000272866.1 | -0.925641325 | -2.049684121 | 0.040395266 |
| DEFB116 | ENST00000609612.1 | ENSG00000273424.1 | 0.863235308 | 1.951197433 | 0.051033564 |

| | | | | | |
|---|---|---|---|---|---|
| DEFB116 | ENST00000609789.1 | ENSG00000272707.1 | 0.860372573 | 1.914892236 | 0.055506248 |
| DEFB116 | NR_026822.1 | FAM138C | 0.802216357 | 1.776097683 | 0.075716818 |
| DEFB116 | NR_026932.1 | PDCD4-AS1 | 0.858436096 | 1.912436855 | 0.055820185 |
| DEFB116 | NR_034131.1 | LINC00272 | 0.805369205 | 1.808935855 | 0.070460969 |
| DEFB116 | NR_040047.1 | SDCBP2-AS1 | 0.85387169 | 1.93003809 | 0.053602118 |
| DEFB116 | NR_040061.1 | SRP14-AS1 | 0.919695329 | 2.047058111 | 0.040652379 |
| DEFB116 | NR_108046.1 | LINC00844 | -0.837806611 | -1.896056806 | 0.057952533 |
| DEFB116 | NR_109985.1 | LOC101927830 | 0.833811197 | 1.899121579 | 0.057548492 |
| DEFB116 | NR_110370.1 | STAM-AS1 | 0.945897297 | 2.128966678 | 0.033257017 |
| DEFB116 | NR_110504.1 | LOC101929572 | 0.810602331 | 1.816652242 | 0.069270379 |
| DEFB116 | NR_125420.1 | LOC101927588 | 0.814375884 | 1.811080238 | 0.070128432 |
| DEFB116 | NR_125925.1 | LOC101929448 | -0.806599287 | -1.817326997 | 0.069167059 |
| DEFB116 | NR_126041.1 | LOC101930071 | 0.904601303 | 2.01664252 | 0.043732832 |
| DEFB116 | NR_126412.1 | SCEL-AS1 | 0.882597293 | 1.976151454 | 0.048137626 |
| DEFB116 | NR_126413.1 | SCEL-AS1 | 0.87289869 | 1.931253074 | 0.053451763 |
| DEFB116 | NR_133942.1 | LOC105377247 | -0.873235978 | -1.920937867 | 0.054739541 |
| DEFB116 | NR_135239.1 | LINC01867 | -0.859627069 | -1.945907373 | 0.051665867 |
| DEFB116 | NR_135816.1 | LOC100996664 | 0.92913005 | 2.064740804 | 0.038947532 |
| DEFB116 | NR_136320.1 | LOC105373656 | 0.813979717 | 1.80674566 | 0.070801945 |
| DHPS | ENST00000416861.1 | ENSG00000227308.2 | 0.895107166 | 2.000117238 | 0.045487606 |
| DHPS | ENST00000417260.1 | ENSG00000231734.4 | -0.80660383 | -1.82626492 | 0.067810367 |
| DHPS | ENST00000419103.1 | ENSG00000227014.1 | 0.881028816 | 1.980926349 | 0.047599534 |
| DHPS | ENST00000422204.1 | ENSG00000238160.1 | 0.898130178 | 2.008947107 | 0.044542743 |
| DHPS | ENST00000431730.1 | ENSG00000237401.2 | 0.866470675 | 1.930112878 | 0.053592853 |
| DHPS | ENST00000433035.1 | ENSG00000230483.1 | 0.945805041 | 2.10690511 | 0.035125806 |
| DHPS | ENST00000437334.1 | ENSG00000226134.1 | 0.866706213 | 1.926294653 | 0.054067593 |
| DHPS | ENST00000439443.1 | ENSG00000236911.2 | 0.919541663 | 2.069080914 | 0.038538494 |
| DHPS | ENST00000445260.2 | ENSG00000231429.2 | -0.83871298 | -1.881070851 | 0.059962282 |
| DHPS | ENST00000446816.1 | ENSG00000204685.5 | 0.874246226 | 1.975352289 | 0.048228183 |
| DHPS | ENST00000451507.1 | ENSG00000229539.1 | 0.803109727 | 1.809919907 | 0.070308209 |
| DHPS | ENST00000451648.1 | ENSG00000232803.1 | 0.845322323 | 1.884856368 | 0.059449232 |
| DHPS | ENST00000454100.1 | ENSG00000236943.2 | 0.866131567 | 1.933535681 | 0.053170242 |
| DHPS | ENST00000454182.1 | ENSG00000230379.1 | -0.927315861 | -2.061000935 | 0.039302951 |
| DHPS | ENST00000454387.1 | ENSG00000223726.1 | 0.847862635 | 1.877738963 | 0.060416884 |
| DHPS | ENST00000454957.1 | ENSG00000224899.1 | -0.873516898 | -1.957312013 | 0.050310796 |
| DHPS | ENST00000457371.1 | ENSG00000237401.2 | 0.89269424 | 1.986921062 | 0.046931145 |
| DHPS | ENST00000458082.1 | ENSG00000231210.2 | 0.823420425 | 1.833588642 | 0.06671508 |
| DHPS | ENST00000458107.3 | ENSG00000248478.2 | -0.861734354 | -1.937193208 | 0.05272173 |
| DHPS | ENST00000479233.1 | ENSG00000243150.1 | -0.874869341 | -1.942893383 | 0.052029042 |
| DHPS | ENST00000480919.1 | ENSG00000242474.1 | 0.927031703 | 2.067029255 | 0.038731397 |
| DHPS | ENST00000487368.1 | ENSG00000273328.1 | 0.9103446 | 2.017421543 | 0.043651541 |
| DHPS | ENST00000488310.1 | ENSG00000240449.1 | 0.839495486 | 1.864691921 | 0.0622246 |
| DHPS | ENST00000500496.2 | ENSG00000245479.2 | 0.849079238 | 1.893073051 | 0.058348155 |
| DHPS | ENST00000507525.1 | ENSG00000250431.1 | -0.964510379 | -2.144769199 | 0.031971305 |
| DHPS | ENST00000507808.1 | ENSG00000250333.1 | 0.840098266 | 1.894019062 | 0.05822248 |
| DHPS | ENST00000509453.1 | ENSG00000249145.1 | 0.808343639 | 1.812458224 | 0.069915423 |
| DHPS | ENST00000511234.1 | ENSG00000250865.1 | 0.932184279 | 2.087916528 | 0.036805365 |
| DHPS | ENST00000513836.1 | ENSG00000251266.1 | -0.928670143 | -2.066648497 | 0.038767287 |
| DHPS | ENST00000518590.1 | ENSG00000253986.1 | 0.835145119 | 1.860942348 | 0.062752319 |
| DHPS | ENST00000519062.1 | ENSG00000253658.1 | -0.833765734 | -1.855332576 | 0.063548749 |
| DHPS | ENST00000520411.1 | ENSG00000253355.1 | 0.857264971 | 1.908076652 | 0.05638131 |

| | | | | | |
|---|---|---|---|---|---|
| DHPS | ENST00000521030.1 | ENSG00000253802.1 | 0.902176849 | 2.010534668 | 0.044374631 |
| DHPS | ENST00000523703.1 | ENSG00000214803.3 | 0.885356905 | 1.999033509 | 0.045604728 |
| DHPS | ENST00000523859.1 | ENSG00000251136.4 | 0.937524705 | 2.117744296 | 0.034196729 |
| DHPS | ENST00000524286.1 | ENSG00000253658.1 | -0.903311117 | -2.014160546 | 0.043992681 |
| DHPS | ENST00000527789.1 | ENSG00000255173.1 | 0.892606991 | 2.007948881 | 0.044648724 |
| DHPS | ENST00000532454.1 | ENSG00000255120.1 | 0.983905471 | 2.186899227 | 0.028749881 |
| DHPS | ENST00000534178.1 | ENSG00000255120.1 | 0.844877029 | 1.880174399 | 0.060084313 |
| DHPS | ENST00000545572.1 | ENSG00000255680.1 | 0.904651381 | 2.014858254 | 0.043919503 |
| DHPS | ENST00000550263.1 | ENSG00000257605.1 | 0.846189335 | 1.882525063 | 0.05976476 |
| DHPS | ENST00000551699.1 | ENSG00000257467.1 | -0.824017815 | -1.840136766 | 0.065748161 |
| DHPS | ENST00000554859.1 | ENSG00000259088.1 | 0.842456613 | 1.885248344 | 0.059396317 |
| DHPS | ENST00000559959.1 | ENSG00000259396.1 | 0.927235322 | 2.034670524 | 0.041884036 |
| DHPS | ENST00000561847.1 | ENSG00000260293.1 | 0.841578 | 1.882593029 | 0.059755542 |
| DHPS | ENST00000562995.1 | ENSG00000261253.1 | 0.883301194 | 1.976796542 | 0.048064633 |
| DHPS | ENST00000565955.1 | ENSG00000261055.1 | 0.829831494 | 1.837169906 | 0.066184817 |
| DHPS | ENST00000568332.1 | ENSG00000260256.1 | 0.86261138 | 1.954449311 | 0.050648104 |
| DHPS | ENST00000570413.1 | ENSG00000263167.1 | 0.849743121 | 1.929170658 | 0.053709679 |
| DHPS | ENST00000571660.1 | ENSG00000262848.1 | 0.827677605 | 1.853222098 | 0.063850532 |
| DHPS | ENST00000572608.1 | ENSG00000263305.1 | -0.877761379 | -1.952629923 | 0.050863463 |
| DHPS | ENST00000586338.1 | ENSG00000219410.4 | 0.835699898 | 1.900907822 | 0.057314087 |
| DHPS | ENST00000586348.1 | ENSG00000267198.1 | 0.800492606 | 1.801452033 | 0.071631661 |
| DHPS | ENST00000587696.1 | ENSG00000225313.2 | 0.929953985 | 2.070377628 | 0.038416995 |
| DHPS | ENST00000587850.1 | ENSG00000267683.1 | 0.891317923 | 1.983913701 | 0.047265461 |
| DHPS | ENST00000589395.1 | ENSG00000267143.1 | 0.853104365 | 1.899330982 | 0.057520972 |
| DHPS | ENST00000590989.1 | ENSG00000267011.1 | 0.986971419 | 2.206244468 | 0.027366887 |
| DHPS | ENST00000591414.1 | ENSG00000267011.1 | 0.963182785 | 2.163106814 | 0.030532965 |
| DHPS | ENST00000592368.1 | ENSG00000267231.1 | 0.929201015 | 2.04178958 | 0.041172408 |
| DHPS | ENST00000593967.1 | ENSG00000232732.5 | 0.871774212 | 1.944818703 | 0.051796802 |
| DHPS | ENST00000597420.1 | ENSG00000269564.1 | 0.826828153 | 1.832140812 | 0.066930444 |
| DHPS | ENST00000599387.1 | ENSG00000227733.4 | -0.88769176 | -1.96933495 | 0.04891464 |
| DHPS | ENST00000600848.1 | ENSG00000228065.6 | -0.865014335 | -1.927965145 | 0.053859462 |
| DHPS | ENST00000602418.1 | ENSG00000232295.3 | 0.848289509 | 1.918261926 | 0.055077808 |
| DHPS | ENST00000602454.1 | ENSG00000270139.1 | -0.836044602 | -1.855574894 | 0.063514175 |
| DHPS | ENST00000602471.1 | ENSG00000270107.1 | 0.835799103 | 1.869999353 | 0.061483908 |
| DHPS | ENST00000603948.1 | ENSG00000222041.6 | 0.820527418 | 1.826362378 | 0.067795695 |
| DHPS | ENST00000605082.1 | ENSG00000270426.1 | 0.91683471 | 2.044682323 | 0.040886187 |
| DHPS | ENST00000606743.1 | ENSG00000272221.1 | 0.823685961 | 1.858939749 | 0.063035679 |
| DHPS | ENST00000606921.1 | ENSG00000272402.1 | 0.828646864 | 1.879535449 | 0.060171418 |
| DHPS | ENST00000606942.1 | ENSG00000271835.1 | -0.915986089 | -2.072367316 | 0.038231199 |
| DHPS | ENST00000607148.1 | ENSG00000272477.1 | -0.820067375 | -1.807324612 | 0.070711681 |
| DHPS | ENST00000608258.1 | ENSG00000229042.2 | -0.951561745 | -2.116778927 | 0.034278615 |
| DHPS | ENST00000609182.1 | ENSG00000273248.1 | 0.837275789 | 1.860005074 | 0.062884808 |
| DHPS | ENST00000610008.1 | ENSG00000272711.1 | 0.992614198 | 2.193699084 | 0.02825705 |
| DHPS | NR_026774.1 | LINC00239 | 0.872770513 | 1.989768134 | 0.046616483 |
| DHPS | NR_028324.1 | LINC01002 | 0.867818845 | 1.947512096 | 0.051473371 |
| DHPS | NR_031762.2 | HCP5B | 0.900349778 | 2.007927921 | 0.044650952 |
| DHPS | NR_034131.1 | LINC00272 | 0.832485598 | 1.878718907 | 0.060282885 |
| DHPS | NR_037170.1 | LOC100507547 | 0.802222743 | 1.793182959 | 0.072943674 |
| DHPS | NR_040047.1 | SDCBP2-AS1 | 0.847158028 | 1.901029631 | 0.057298132 |
| DHPS | NR_047498.1 | LINC00853 | 0.87449349 | 1.944118645 | 0.051881145 |
| DHPS | NR_108085.1 | OVOL1-AS1 | 0.985008119 | 2.208178622 | 0.027231824 |

| | | | | | |
|---|---|---|---|---|---|
| DHPS | NR_109770.1 | TONSL-AS1 | 0.808942046 | 1.813933017 | 0.069688039 |
| DHPS | NR_110930.1 | LOC101927814 | 0.900233649 | 2.008969478 | 0.044540371 |
| DHPS | NR_120371.1 | LINC01585 | 0.825184761 | 1.831304431 | 0.067055117 |
| DHPS | NR_125420.1 | LOC101927588 | 0.893442043 | 1.989140683 | 0.046685677 |
| DHPS | NR_126412.1 | SCEL-AS1 | 0.801028266 | 1.792955502 | 0.072980039 |
| DHPS | NR_133941.1 | LOC105377247 | -0.928670143 | -2.08979886 | 0.036635872 |
| DHPS | NR_133942.1 | LOC105377247 | -0.851266165 | -1.894749299 | 0.058125623 |
| DHPS | NR_134325.1 | LOC102723672 | 0.865095576 | 1.925640834 | 0.054149236 |
| DHPS | NR_135132.1 | HSPC324 | 0.880789082 | 1.989602586 | 0.046634731 |
| DHPS | NR_135626.1 | LOC100505585 | 0.8453306 | 1.897063762 | 0.057819523 |
| DHPS | NR_135816.1 | LOC100996664 | 0.857875013 | 1.902484531 | 0.057107839 |
| DNAJC4 | ENST00000318291.4 | ENSG00000177406.4 | 0.909478411 | 2.042773148 | 0.0410749 |
| DNAJC4 | ENST00000381106.4 | ENSG00000205663.5 | -0.85623478 | -1.912292129 | 0.055838735 |
| DNAJC4 | ENST00000412348.1 | ENSG00000228959.1 | -0.974234555 | -2.172607276 | 0.029809888 |
| DNAJC4 | ENST00000417260.1 | ENSG00000231734.4 | -0.895246835 | -2.03746593 | 0.041603373 |
| DNAJC4 | ENST00000417654.1 | ENSG00000224893.1 | 0.836623553 | 1.879324246 | 0.060200233 |
| DNAJC4 | ENST00000418080.1 | ENSG00000224091.1 | 0.824406464 | 1.84298597 | 0.065331059 |
| DNAJC4 | ENST00000418387.1 | ENSG00000235056.1 | -0.920580594 | -2.038974305 | 0.041452592 |
| DNAJC4 | ENST00000422017.1 | ENSG00000232227.1 | -0.805342007 | -1.834321588 | 0.066606271 |
| DNAJC4 | ENST00000423428.1 | ENSG00000224048.1 | -0.865821386 | -1.916195878 | 0.055340168 |
| DNAJC4 | ENST00000426302.1 | ENSG00000230454.1 | 0.90335602 | 2.006899917 | 0.044760321 |
| DNAJC4 | ENST00000430920.1 | ENSG00000234203.1 | 0.817996282 | 1.831973351 | 0.066955391 |
| DNAJC4 | ENST00000435287.1 | ENSG00000227220.1 | 0.910314682 | 2.024283194 | 0.042941022 |
| DNAJC4 | ENST00000436515.1 | ENSG00000224521.1 | -0.977424571 | -2.19865669 | 0.027902343 |
| DNAJC4 | ENST00000438190.1 | ENSG00000227214.2 | 0.835294627 | 1.874840401 | 0.060814683 |
| DNAJC4 | ENST00000438623.1 | ENSG00000224521.1 | -0.948518937 | -2.100460703 | 0.035688334 |
| DNAJC4 | ENST00000442829.1 | ENSG00000225284.1 | 0.877969341 | 2.006710284 | 0.04478052 |
| DNAJC4 | ENST00000448748.1 | ENSG00000231238.1 | -0.841313986 | -1.85770029 | 0.063211587 |
| DNAJC4 | ENST00000448858.1 | ENSG00000237734.1 | -0.884787318 | -1.978892282 | 0.047828135 |
| DNAJC4 | ENST00000451034.1 | ENSG00000229805.1 | -0.899511004 | -1.987240195 | 0.046895786 |
| DNAJC4 | ENST00000452176.1 | ENSG00000223659.1 | -0.837332373 | -1.86638388 | 0.061987677 |
| DNAJC4 | ENST00000455373.1 | ENSG00000226097.1 | -0.84995282 | -1.893638882 | 0.058272959 |
| DNAJC4 | ENST00000457998.2 | ENSG00000233006.2 | 0.836840187 | 1.863666156 | 0.062368601 |
| DNAJC4 | ENST00000458154.1 | ENSG00000235578.1 | 0.8066793 | 1.808662474 | 0.070503456 |
| DNAJC4 | ENST00000459985.1 | ENSG00000273066.1 | 0.827537922 | 1.845761493 | 0.064926844 |
| DNAJC4 | ENST00000485338.1 | ENSG00000239641.1 | -0.817215406 | -1.816521399 | 0.069290428 |
| DNAJC4 | ENST00000489077.1 | ENSG00000244198.1 | 0.806711418 | 1.811446086 | 0.070071828 |
| DNAJC4 | ENST00000489690.1 | ENSG00000243944.1 | -0.895124843 | -2.004760852 | 0.04498862 |
| DNAJC4 | ENST00000498693.1 | ENSG00000244198.1 | 0.822709602 | 1.827200148 | 0.067669682 |
| DNAJC4 | ENST00000503505.1 | ENSG00000248629.1 | -0.944462632 | -2.102058766 | 0.035548128 |
| DNAJC4 | ENST00000503723.1 | ENSG00000250472.1 | -0.82625934 | -1.862402777 | 0.062546338 |
| DNAJC4 | ENST00000504578.1 | ENSG00000251513.1 | -0.913497055 | -2.075125722 | 0.037974884 |
| DNAJC4 | ENST00000506723.2 | ENSG00000249484.4 | -0.808787105 | -1.81607217 | 0.069359302 |
| DNAJC4 | ENST00000509192.1 | ENSG00000250765.1 | 0.850211447 | 1.920106278 | 0.054844476 |
| DNAJC4 | ENST00000515128.1 | ENSG00000248215.1 | -0.988862464 | -2.202697053 | 0.027616108 |
| DNAJC4 | ENST00000518837.1 | ENSG00000253947.1 | -0.804168874 | -1.820484949 | 0.068685186 |
| DNAJC4 | ENST00000522390.1 | ENSG00000254262.1 | -0.854820151 | -1.925010453 | 0.054228051 |
| DNAJC4 | ENST00000522600.1 | ENSG00000246582.2 | 0.8817974 | 1.972169694 | 0.048590238 |
| DNAJC4 | ENST00000528887.1 | ENSG00000254501.1 | 0.906652808 | 2.046826183 | 0.040675154 |
| DNAJC4 | ENST00000534178.1 | ENSG00000255120.1 | 0.851707501 | 1.909844121 | 0.056153287 |
| DNAJC4 | ENST00000537032.1 | ENSG00000255933.1 | 0.821379428 | 1.848084476 | 0.064590123 |

| | | | | | |
|---|---|---|---|---|---|
| DNAJC4 | ENST00000537850.1 | ENSG00000251002.3 | 0.916059031 | 2.058510784 | 0.039541127 |
| DNAJC4 | ENST00000543275.1 | ENSG00000256944.1 | 0.842274783 | 1.897077959 | 0.05781765 |
| DNAJC4 | ENST00000543494.1 | ENSG00000256514.1 | 0.91982141 | 2.059695732 | 0.039427638 |
| DNAJC4 | ENST00000547834.1 | ENSG00000258325.1 | 0.809371087 | 1.817608613 | 0.069123974 |
| DNAJC4 | ENST00000548210.1 | ENSG00000257784.1 | 0.815723034 | 1.839271455 | 0.065875269 |
| DNAJC4 | ENST00000549683.1 | ENSG00000257953.1 | 0.964481304 | 2.157803502 | 0.030943113 |
| DNAJC4 | ENST00000550263.1 | ENSG00000257605.1 | 0.859426848 | 1.908857033 | 0.056280537 |
| DNAJC4 | ENST00000554431.1 | ENSG00000258616.1 | -0.833705631 | -1.878310974 | 0.060338636 |
| DNAJC4 | ENST00000555460.1 | ENSG00000259042.1 | 0.851870674 | 1.895319981 | 0.058050023 |
| DNAJC4 | ENST00000557602.1 | ENSG00000258616.1 | -0.841077428 | -1.86030908 | 0.06284181 |
| DNAJC4 | ENST00000558575.1 | ENSG00000259687.1 | 0.804450521 | 1.803826551 | 0.071258502 |
| DNAJC4 | ENST00000560963.1 | ENSG00000259370.1 | 0.870749014 | 1.96229948 | 0.049727628 |
| DNAJC4 | ENST00000563018.1 | ENSG00000260193.1 | 0.846322127 | 1.895954887 | 0.05796601 |
| DNAJC4 | ENST00000563610.1 | ENSG00000260051.1 | 0.930485813 | 2.077483227 | 0.037756982 |
| DNAJC4 | ENST00000563611.1 | ENSG00000261583.1 | 0.848093917 | 1.884258926 | 0.05952996 |
| DNAJC4 | ENST00000565823.1 | ENSG00000260686.1 | -0.873993503 | -1.954674086 | 0.050621551 |
| DNAJC4 | ENST00000568659.1 | ENSG00000260004.1 | -0.84726229 | -1.887931876 | 0.059035097 |
| DNAJC4 | ENST00000569981.1 | ENSG00000238045.5 | 0.820191302 | 1.8309651 | 0.067105752 |
| DNAJC4 | ENST00000570493.2 | ENSG00000261898.2 | 0.896837719 | 2.003169013 | 0.04515915 |
| DNAJC4 | ENST00000570929.1 | ENSG00000262223.2 | 0.814285755 | 1.82596134 | 0.067856085 |
| DNAJC4 | ENST00000570974.1 | ENSG00000263300.1 | 0.931471171 | 2.08881741 | 0.036724163 |
| DNAJC4 | ENST00000576086.1 | ENSG00000262823.1 | 0.837983076 | 1.875248364 | 0.060758563 |
| DNAJC4 | ENST00000576554.1 | ENSG00000262413.1 | 0.814560007 | 1.830222691 | 0.067216646 |
| DNAJC4 | ENST00000577176.1 | ENSG00000262823.1 | 0.817761821 | 1.819659331 | 0.0688109 |
| DNAJC4 | ENST00000577853.1 | ENSG00000264207.1 | 0.890069762 | 2.00901656 | 0.044535378 |
| DNAJC4 | ENST00000580729.1 | ENSG00000266176.1 | 0.803355446 | 1.801001568 | 0.071702633 |
| DNAJC4 | ENST00000585559.1 | ENSG00000267117.1 | 0.890382571 | 1.996748181 | 0.045852545 |
| DNAJC4 | ENST00000585810.1 | ENSG00000236172.2 | 0.919944524 | 2.060222598 | 0.039377265 |
| DNAJC4 | ENST00000588402.1 | ENSG00000267006.1 | -0.843774358 | -1.869496066 | 0.06155383 |
| DNAJC4 | ENST00000590328.1 | ENSG00000256995.2 | -0.871512144 | -1.954075836 | 0.050692249 |
| DNAJC4 | ENST00000591174.1 | ENSG00000267289.1 | 0.922636738 | 2.075186559 | 0.037969248 |
| DNAJC4 | ENST00000592720.1 | ENSG00000267232.1 | 0.828782665 | 1.847933935 | 0.064611901 |
| DNAJC4 | ENST00000592816.1 | ENSG00000236172.2 | 0.922896612 | 2.057436548 | 0.039644253 |
| DNAJC4 | ENST00000595478.1 | ENSG00000237031.3 | -0.855502959 | -1.921454865 | 0.054674387 |
| DNAJC4 | ENST00000596091.1 | ENSG00000227733.4 | -0.846465964 | -1.897955903 | 0.057701891 |
| DNAJC4 | ENST00000596887.1 | ENSG00000237031.3 | -0.809009669 | -1.807594786 | 0.07066959 |
| DNAJC4 | ENST00000597680.1 | ENSG00000269574.1 | -0.823067308 | -1.817102157 | 0.069201473 |
| DNAJC4 | ENST00000598092.1 | ENSG00000228065.6 | -0.949036252 | -2.10395042 | 0.035382772 |
| DNAJC4 | ENST00000599259.1 | ENSG00000269352.1 | 0.850470374 | 1.902276176 | 0.057135059 |
| DNAJC4 | ENST00000600007.1 | ENSG00000268655.1 | 0.822846235 | 1.850199005 | 0.064284872 |
| DNAJC4 | ENST00000600716.1 | ENSG00000269487.1 | 0.966415045 | 2.175271371 | 0.029609787 |
| DNAJC4 | ENST00000600726.1 | ENSG00000267858.1 | 0.950866457 | 2.167336378 | 0.030209215 |
| DNAJC4 | ENST00000604142.1 | ENSG00000271308.1 | 0.832896511 | 1.866928416 | 0.061911585 |
| DNAJC4 | ENST00000604183.1 | ENSG00000271185.1 | 0.836775871 | 1.870679261 | 0.061389551 |
| DNAJC4 | ENST00000606010.1 | ENSG00000272249.1 | -0.868516399 | -1.957846555 | 0.050248021 |
| DNAJC4 | ENST00000607148.1 | ENSG00000272477.1 | -0.826917738 | -1.854341798 | 0.063690276 |
| DNAJC4 | ENST00000607476.1 | ENSG00000272540.1 | 0.817123666 | 1.81384646 | 0.069701367 |
| DNAJC4 | ENST00000607549.1 | ENSG00000272293.1 | -0.926092179 | -2.062547611 | 0.03915563 |
| DNAJC4 | ENST00000607943.1 | ENSG00000273188.1 | 0.918864762 | 2.061803234 | 0.039226473 |
| DNAJC4 | ENST00000608259.1 | ENSG00000272627.1 | -0.937349197 | -2.107819486 | 0.035046607 |
| DNAJC4 | ENST00000608489.1 | ENSG00000272716.1 | 0.936799821 | 2.069319322 | 0.038516132 |

| | | | | | |
|---|---|---|---|---|---|
| DNAJC4 | ENST00000609113.1 | ENSG00000272827.1 | 0.899555722 | 2.021466911 | 0.043231454 |
| DNAJC4 | ENST00000609813.1 | ENSG00000272719.1 | 0.907962704 | 2.037771808 | 0.041572759 |
| DNAJC4 | ENST00000609955.1 | ENSG00000273275.1 | -0.808055105 | -1.830407987 | 0.067188954 |
| DNAJC4 | ENST00000609976.1 | ENSG00000272582.1 | 0.915302729 | 2.044693786 | 0.040885057 |
| DNAJC4 | NR_003605.1 | ZFAS1 | 0.860519315 | 1.914678271 | 0.055533546 |
| DNAJC4 | NR_036480.1 | VPS9D1-AS1 | 0.903761342 | 2.021229173 | 0.043256047 |
| DNAJC4 | NR_038421.1 | LINC01220 | 0.901394931 | 2.003126268 | 0.045163737 |
| DNAJC4 | NR_072981.1 | LINC00957 | 0.827890322 | 1.83249383 | 0.06687788 |
| DNAJC4 | NR_072982.1 | LINC00957 | 0.818861768 | 1.854189146 | 0.063712104 |
| DNAJC4 | NR_073552.1 | LOC101059948 | 0.822846235 | 1.836887578 | 0.066226493 |
| DNAJC4 | NR_108036.1 | CFAP58-AS1 | 0.826622301 | 1.849861251 | 0.06433355 |
| DNAJC4 | NR_110117.1 | LOC101927769 | -0.893942861 | -1.998259372 | 0.045688548 |
| DNAJC4 | NR_110245.1 | LOC101929282 | -0.845152042 | -1.871706496 | 0.06124722 |
| DNAJC4 | NR_110556.1 | LOC102724890 | -0.856358694 | -1.916901181 | 0.055250487 |
| DNAJC4 | NR_110630.1 | LOC101927478 | 0.892558371 | 2.006329624 | 0.044821091 |
| DNAJC4 | NR_121188.1 | PGM5P3-AS1 | -0.82245155 | -1.865231249 | 0.062148997 |
| DNAJC4 | NR_125957.1 | LOC101928626 | -0.837332373 | -1.866376366 | 0.061988727 |
| DNAJC4 | NR_135584.1 | LOC101927596 | 0.802703016 | 1.776471064 | 0.075655307 |
| DNAJC4 | NR_136569.1 | LINC01660 | -0.811459956 | -1.804712033 | 0.071119756 |
| DNAJC4 | NR_144459.1 | ARSD-AS1 | 0.964338909 | 2.149758768 | 0.031574301 |
| DYRK1B | ENST00000421019.1 | ENSG00000224397.1 | 0.949569977 | 2.119910503 | 0.034013593 |
| DYRK1B | ENST00000422914.1 | ENSG00000236120.2 | 0.973294028 | 2.17087342 | 0.029940742 |
| DYRK1B | ENST00000425058.1 | ENSG00000226771.1 | -0.908077275 | -2.024557221 | 0.042912851 |
| DYRK1B | ENST00000426030.2 | ENSG00000228686.2 | -0.855820575 | -1.919808594 | 0.054882081 |
| DYRK1B | ENST00000427132.1 | ENSG00000232121.1 | 0.894372661 | 2.021604403 | 0.043217237 |
| DYRK1B | ENST00000427524.1 | ENSG00000236065.2 | 0.851670488 | 1.896885789 | 0.057843013 |
| DYRK1B | ENST00000429878.1 | ENSG00000224184.1 | 0.914859298 | 2.028640494 | 0.042494921 |
| DYRK1B | ENST00000434250.1 | ENSG00000234055.1 | 0.830592124 | 1.854509442 | 0.063666311 |
| DYRK1B | ENST00000434493.1 | ENSG00000224605.1 | -0.802852321 | -1.791443634 | 0.073222132 |
| DYRK1B | ENST00000437334.1 | ENSG00000226134.1 | 0.80524978 | 1.799135016 | 0.071997326 |
| DYRK1B | ENST00000438409.1 | ENSG00000234174.1 | 0.862615614 | 1.92466758 | 0.054270959 |
| DYRK1B | ENST00000439529.1 | ENSG00000236526.1 | -0.827449497 | -1.855095489 | 0.063582592 |
| DYRK1B | ENST00000441809.2 | ENSG00000237445.2 | 0.83289479 | 1.880055065 | 0.060100574 |
| DYRK1B | ENST00000442579.1 | ENSG00000228719.1 | 0.871406227 | 1.945316713 | 0.051736872 |
| DYRK1B | ENST00000442829.1 | ENSG00000225284.1 | 0.818817659 | 1.866708272 | 0.061942338 |
| DYRK1B | ENST00000445617.2 | ENSG00000225751.2 | -0.938655665 | -2.077996987 | 0.037709637 |
| DYRK1B | ENST00000446562.1 | ENSG00000233896.1 | 0.840899476 | 1.877451149 | 0.060456287 |
| DYRK1B | ENST00000448431.1 | ENSG00000232548.1 | -0.836714402 | -1.855759843 | 0.063487797 |
| DYRK1B | ENST00000448674.1 | ENSG00000235119.1 | 0.942741736 | 2.120642955 | 0.03395186 |
| DYRK1B | ENST00000450206.1 | ENSG00000234311.1 | 0.818568407 | 1.841135019 | 0.065601775 |
| DYRK1B | ENST00000451090.1 | ENSG00000235215.2 | -0.8724117 | -1.919830515 | 0.054879311 |
| DYRK1B | ENST00000451267.1 | ENSG00000230410.1 | 0.947130002 | 2.148723171 | 0.031656351 |
| DYRK1B | ENST00000451828.1 | ENSG00000228549.2 | 0.863945888 | 1.937964547 | 0.052627548 |
| DYRK1B | ENST00000452511.1 | ENSG00000231876.3 | -0.85694216 | -1.92177817 | 0.054633676 |
| DYRK1B | ENST00000452553.1 | ENSG00000233973.1 | -0.916868884 | -2.056141517 | 0.039768877 |
| DYRK1B | ENST00000453878.1 | ENSG00000224850.1 | -0.859104687 | -1.930967451 | 0.053487078 |
| DYRK1B | ENST00000454957.1 | ENSG00000224899.1 | -0.809440399 | -1.809600673 | 0.070357735 |
| DYRK1B | ENST00000454965.1 | ENSG00000235435.1 | -0.944308212 | -2.117235597 | 0.034239858 |
| DYRK1B | ENST00000457043.1 | ENSG00000231365.1 | -0.836091693 | -1.869679163 | 0.061528385 |
| DYRK1B | ENST00000457113.1 | ENSG00000227407.1 | 0.90618209 | 2.008409029 | 0.044599844 |
| DYRK1B | ENST00000457632.1 | ENSG00000234248.1 | -0.937132448 | -2.097084446 | 0.035986101 |

| | | | | | |
|---|---|---|---|---|---|
| DYRK1B | ENST00000457856.1 | ENSG00000228549.2 | 0.897473828 | 2.001591539 | 0.04532868 |
| DYRK1B | ENST00000462300.1 | ENSG00000241912.1 | 0.80022205 | 1.802221799 | 0.071510516 |
| DYRK1B | ENST00000479233.1 | ENSG00000243150.1 | -0.806068355 | -1.818532134 | 0.06898284 |
| DYRK1B | ENST00000488040.1 | ENSG00000243176.1 | -0.872199963 | -1.971518181 | 0.048664636 |
| DYRK1B | ENST00000488310.1 | ENSG00000240449.1 | 0.845802399 | 1.915754214 | 0.055396388 |
| DYRK1B | ENST00000506379.1 | ENSG00000240152.2 | -0.894448862 | -1.97196925 | 0.048613117 |
| DYRK1B | ENST00000506852.1 | ENSG00000250945.1 | -0.821631186 | -1.831875435 | 0.066969981 |
| DYRK1B | ENST00000509098.1 | ENSG00000250863.1 | -0.962850877 | -2.147285278 | 0.031770577 |
| DYRK1B | ENST00000509718.1 | ENSG00000251132.1 | 0.969467153 | 2.16041151 | 0.030740827 |
| DYRK1B | ENST00000509983.1 | ENSG00000248173.1 | 0.898710804 | 2.011342436 | 0.044289299 |
| DYRK1B | ENST00000511603.1 | ENSG00000249892.1 | -0.882841468 | -1.967305005 | 0.049148058 |
| DYRK1B | ENST00000511631.1 | ENSG00000250402.1 | 0.86827485 | 1.925099007 | 0.054216973 |
| DYRK1B | ENST00000511917.1 | ENSG00000250062.1 | -0.944487165 | -2.136866811 | 0.032608826 |
| DYRK1B | ENST00000512036.1 | ENSG00000250993.1 | -0.978413547 | -2.171189679 | 0.029916837 |
| DYRK1B | ENST00000515706.1 | ENSG00000250124.1 | -0.920122351 | -2.036667363 | 0.041683387 |
| DYRK1B | ENST00000519005.1 | ENSG00000253507.1 | -0.911455396 | -2.019588242 | 0.043426116 |
| DYRK1B | ENST00000519695.1 | ENSG00000253507.1 | -0.887119355 | -1.97817221 | 0.047909283 |
| DYRK1B | ENST00000521359.1 | ENSG00000253140.1 | -0.911315699 | -2.060135111 | 0.039385626 |
| DYRK1B | ENST00000521490.1 | ENSG00000253407.1 | 0.82758318 | 1.858481573 | 0.063100658 |
| DYRK1B | ENST00000522408.1 | ENSG00000253484.1 | -0.991044065 | -2.217730194 | 0.026573234 |
| DYRK1B | ENST00000524133.1 | ENSG00000253174.2 | 0.848179167 | 1.920302034 | 0.05481976 |
| DYRK1B | ENST00000524275.1 | ENSG00000253507.1 | -0.856236192 | -1.925070776 | 0.054220504 |
| DYRK1B | ENST00000525855.1 | ENSG00000254746.1 | 0.854082135 | 1.926670779 | 0.054020672 |
| DYRK1B | ENST00000527579.1 | ENSG00000254574.1 | 0.952119668 | 2.135680881 | 0.032705434 |
| DYRK1B | ENST00000527757.1 | ENSG00000255109.1 | -0.801865717 | -1.789771357 | 0.073490676 |
| DYRK1B | ENST00000529247.1 | ENSG00000254741.1 | 0.808766343 | 1.798384275 | 0.072116133 |
| DYRK1B | ENST00000529266.1 | ENSG00000254468.1 | -0.906423581 | -2.032262137 | 0.042127125 |
| DYRK1B | ENST00000532123.1 | ENSG00000255555.1 | -0.818250075 | -1.837813422 | 0.066089903 |
| DYRK1B | ENST00000536529.1 | ENSG00000256422.1 | -0.821631186 | -1.825851346 | 0.067872657 |
| DYRK1B | ENST00000539313.1 | ENSG00000256588.1 | -0.927630874 | -2.060389853 | 0.039361286 |
| DYRK1B | ENST00000543403.1 | ENSG00000256684.1 | -0.869543141 | -1.969955479 | 0.048843473 |
| DYRK1B | ENST00000545158.1 | ENSG00000256011.1 | -0.855010037 | -1.919387477 | 0.054935315 |
| DYRK1B | ENST00000548748.1 | ENSG00000258252.1 | 0.85649567 | 1.899466056 | 0.057503225 |
| DYRK1B | ENST00000550279.1 | ENSG00000258338.1 | -0.892057628 | -1.984691882 | 0.047178763 |
| DYRK1B | ENST00000552634.1 | ENSG00000257496.1 | 0.816685887 | 1.821966331 | 0.068460095 |
| DYRK1B | ENST00000553954.1 | ENSG00000259052.1 | 0.88307836 | 1.978316186 | 0.047893048 |
| DYRK1B | ENST00000554431.1 | ENSG00000258616.1 | -0.81198796 | -1.837675467 | 0.066110241 |
| DYRK1B | ENST00000555636.1 | ENSG00000259072.1 | -0.823937637 | -1.868559499 | 0.061684125 |
| DYRK1B | ENST00000555689.1 | ENSG00000259049.1 | 0.876999726 | 1.957610787 | 0.050275701 |
| DYRK1B | ENST00000556458.1 | ENSG00000258504.2 | 0.801023475 | 1.783222674 | 0.074550063 |
| DYRK1B | ENST00000557602.1 | ENSG00000258616.1 | -0.823252556 | -1.829149356 | 0.067377237 |
| DYRK1B | ENST00000557855.1 | ENSG00000259176.1 | -0.874408104 | -1.951573062 | 0.050988914 |
| DYRK1B | ENST00000558221.1 | ENSG00000259704.1 | 0.931313354 | 2.079259496 | 0.037593506 |
| DYRK1B | ENST00000558237.1 | ENSG00000259684.1 | -0.943003549 | -2.093824115 | 0.036275651 |
| DYRK1B | ENST00000559026.1 | ENSG00000259732.1 | -0.814375481 | -1.834469995 | 0.066584258 |
| DYRK1B | ENST00000560268.1 | ENSG00000259287.1 | 0.885597325 | 1.988949595 | 0.046706767 |
| DYRK1B | ENST00000560969.1 | ENSG00000259176.1 | -0.843508104 | -1.868761907 | 0.061655947 |
| DYRK1B | ENST00000562191.1 | ENSG00000261292.1 | -0.842803351 | -1.895133673 | 0.058074695 |
| DYRK1B | ENST00000569215.1 | ENSG00000260756.1 | -0.884959903 | -1.962298859 | 0.0497277 |
| DYRK1B | ENST00000571775.1 | ENSG00000262456.1 | 0.951835279 | 2.122357303 | 0.033807745 |
| DYRK1B | ENST00000576271.1 | ENSG00000263342.1 | 0.858098937 | 1.923325509 | 0.054439183 |

| | | | | | |
|---|---|---|---|---|---|
| DYRK1B | ENST00000580184.1 | ENSG00000264914.1 | 0.863028079 | 1.971691697 | 0.048644812 |
| DYRK1B | ENST00000580311.1 | ENSG00000266803.1 | -0.843822895 | -1.888578917 | 0.058948274 |
| DYRK1B | ENST00000587702.1 | ENSG00000267378.1 | -0.933871411 | -2.09717426 | 0.035978152 |
| DYRK1B | ENST00000588177.1 | ENSG00000234899.5 | -0.830859309 | -1.873399695 | 0.061013212 |
| DYRK1B | ENST00000588384.1 | ENSG00000236172.2 | 0.842053549 | 1.876783229 | 0.06054781 |
| DYRK1B | ENST00000588402.1 | ENSG00000267006.1 | -0.815058981 | -1.825351027 | 0.067948075 |
| DYRK1B | ENST00000592523.1 | ENSG00000226994.3 | -0.833252933 | -1.863776202 | 0.062353139 |
| DYRK1B | ENST00000595007.1 | ENSG00000231876.3 | -0.872837212 | -1.936346513 | 0.052825276 |
| DYRK1B | ENST00000595409.1 | ENSG00000232729.3 | -0.833751449 | -1.85076417 | 0.064203488 |
| DYRK1B | ENST00000595737.1 | ENSG00000228065.6 | -0.893496014 | -1.9892749 | 0.046670869 |
| DYRK1B | ENST00000595972.1 | ENSG00000230333.2 | -0.941930557 | -2.074450349 | 0.038037505 |
| DYRK1B | ENST00000596567.1 | ENSG00000226647.2 | -0.838506804 | -1.856830081 | 0.063335333 |
| DYRK1B | ENST00000597420.1 | ENSG00000269564.1 | 0.86168469 | 1.910709102 | 0.056041974 |
| DYRK1B | ENST00000598887.1 | ENSG00000268475.1 | 0.931482145 | 2.112564415 | 0.034638067 |
| DYRK1B | ENST00000601420.1 | ENSG00000269560.1 | 0.849493997 | 1.890826421 | 0.058647519 |
| DYRK1B | ENST00000602507.1 | ENSG00000270069.1 | -0.848833474 | -1.887017917 | 0.059157916 |
| DYRK1B | ENST00000602790.1 | ENSG00000270000.1 | 0.813347278 | 1.81790551 | 0.069078576 |
| DYRK1B | ENST00000602835.1 | ENSG00000270096.1 | -0.84244373 | -1.881556009 | 0.059896324 |
| DYRK1B | ENST00000605021.1 | ENSG00000271401.1 | -0.920141296 | -2.063805741 | 0.039036139 |
| DYRK1B | ENST00000607594.1 | ENSG00000271766.1 | -0.878985913 | -1.986269471 | 0.047003411 |
| DYRK1B | ENST00000608422.1 | ENSG00000272866.1 | -0.822848264 | -1.853984389 | 0.063741393 |
| DYRK1B | ENST00000609612.1 | ENSG00000273424.1 | 0.809186386 | 1.802626812 | 0.071446843 |
| DYRK1B | ENST00000609775.1 | ENSG00000273232.1 | -0.885468858 | -1.989562426 | 0.046639159 |
| DYRK1B | NR_026932.1 | PDCD4-AS1 | 0.958341965 | 2.141233778 | 0.03225519 |
| DYRK1B | NR_040047.1 | SDCBP2-AS1 | 0.829728372 | 1.854803201 | 0.063624335 |
| DYRK1B | NR_040049.1 | SDCBP2-AS1 | 0.898384458 | 1.976856911 | 0.048057807 |
| DYRK1B | NR_046762.1 | ALMS1-IT1 | -0.897143168 | -2.025478755 | 0.042818228 |
| DYRK1B | NR_047115.1 | PPP2R2B-IT1 | -0.803287394 | -1.801332366 | 0.071650509 |
| DYRK1B | NR_073155.1 | C1orf145 | -0.901716527 | -2.03278264 | 0.042074488 |
| DYRK1B | NR_102703.1 | MAGEA8-AS1 | -0.817241597 | -1.812293873 | 0.069940801 |
| DYRK1B | NR_103830.1 | LINC00587 | -0.944480481 | -2.102357851 | 0.03552194 |
| DYRK1B | NR_108046.1 | LINC00844 | -0.90141622 | -2.021553191 | 0.043222532 |
| DYRK1B | NR_109831.1 | RASSF1-AS1 | 0.804824209 | 1.773424676 | 0.076158363 |
| DYRK1B | NR_110160.1 | LOC100996249 | 0.883118489 | 1.961238576 | 0.049851199 |
| DYRK1B | NR_110803.1 | LOC101927018 | 0.908709051 | 2.023602954 | 0.043011021 |
| DYRK1B | NR_110879.1 | LOC101929064 | -0.968864112 | -2.150704952 | 0.031499495 |
| DYRK1B | NR_125849.1 | LOC101928140 | -0.866293258 | -1.926693213 | 0.054017874 |
| DYRK1B | NR_131243.1 | SMCR2 | 0.917774394 | 2.041218697 | 0.041229094 |
| DYRK1B | NR_131963.1 | LVCAT5 | -0.862495242 | -1.942409702 | 0.052087522 |
| DYRK1B | NR_134520.1 | LOC727993 | 0.828854542 | 1.859275917 | 0.062988039 |
| DYRK1B | NR_134566.1 | LOC105372695 | -0.826888983 | -1.833634637 | 0.066708247 |
| DYRK1B | NR_135097.1 | LOC105369443 | -0.869543141 | -1.946642181 | 0.051577648 |
| ELANE | ENST00000318291.4 | ENSG00000177406.4 | 0.952492443 | 2.139686811 | 0.032380086 |
| ELANE | ENST00000381106.4 | ENSG00000205663.5 | -0.803609656 | -1.817365387 | 0.069161184 |
| ELANE | ENST00000412348.1 | ENSG00000228959.1 | -0.879314844 | -1.965001924 | 0.049414015 |
| ELANE | ENST00000412759.1 | ENSG00000236933.1 | 0.905513263 | 2.034631894 | 0.041887926 |
| ELANE | ENST00000418387.1 | ENSG00000235056.1 | -0.80487617 | -1.79859632 | 0.07208256 |
| ELANE | ENST00000426237.2 | ENSG00000235527.2 | 0.814834716 | 1.801988132 | 0.071547273 |
| ELANE | ENST00000426302.1 | ENSG00000230454.1 | 0.852637764 | 1.90622277 | 0.056621309 |
| ELANE | ENST00000426519.1 | ENSG00000234142.1 | 0.834868731 | 1.84550822 | 0.064963644 |
| ELANE | ENST00000430920.1 | ENSG00000234203.1 | 0.86558989 | 1.942564142 | 0.052068843 |

| | | | | | |
|---|---|---|---|---|---|
| ELANE | ENST00000435434.1 | ENSG00000231233.1 | 0.873472128 | 1.964658612 | 0.049453763 |
| ELANE | ENST00000435892.1 | ENSG00000233635.2 | 0.803575933 | 1.790881369 | 0.073312334 |
| ELANE | ENST00000436515.1 | ENSG00000224521.1 | -0.870177573 | -1.96506874 | 0.049406282 |
| ELANE | ENST00000438190.1 | ENSG00000227214.2 | 0.908436309 | 2.03192326 | 0.042161425 |
| ELANE | ENST00000438623.1 | ENSG00000224521.1 | -0.851463474 | -1.892293355 | 0.058451906 |
| ELANE | ENST00000442069.1 | ENSG00000225655.1 | -0.827285876 | -1.854006648 | 0.063738209 |
| ELANE | ENST00000442829.1 | ENSG00000225284.1 | 0.843461368 | 1.881827228 | 0.059859478 |
| ELANE | ENST00000447206.1 | ENSG00000230839.1 | 0.807512342 | 1.809974143 | 0.070299797 |
| ELANE | ENST00000451034.1 | ENSG00000229805.1 | -0.874312375 | -1.977738824 | 0.047958178 |
| ELANE | ENST00000452176.1 | ENSG00000223659.1 | -0.808574598 | -1.789645528 | 0.073510914 |
| ELANE | ENST00000453051.1 | ENSG00000229407.1 | 0.859436126 | 1.914176304 | 0.055597632 |
| ELANE | ENST00000457115.1 | ENSG00000227245.1 | 0.872244632 | 1.941923709 | 0.052146337 |
| ELANE | ENST00000458154.1 | ENSG00000235578.1 | 0.850171719 | 1.910865006 | 0.056021931 |
| ELANE | ENST00000458194.1 | ENSG00000226193.1 | 0.887752118 | 1.963477857 | 0.049590675 |
| ELANE | ENST00000458364.1 | ENSG00000225655.1 | -0.813266802 | -1.840217509 | 0.06573631 |
| ELANE | ENST00000459985.1 | ENSG00000273066.1 | 0.952426953 | 2.149618728 | 0.031585386 |
| ELANE | ENST00000468165.1 | ENSG00000239480.1 | 0.876366401 | 1.955491794 | 0.050525051 |
| ELANE | ENST00000489077.1 | ENSG00000244198.1 | 0.836124587 | 1.889136008 | 0.058873607 |
| ELANE | ENST00000489557.2 | ENSG00000257045.1 | 0.81325037 | 1.802906805 | 0.071402852 |
| ELANE | ENST00000498693.1 | ENSG00000244198.1 | 0.920509482 | 2.036847817 | 0.041665295 |
| ELANE | ENST00000503723.1 | ENSG00000250472.1 | -0.815224731 | -1.803081059 | 0.071375485 |
| ELANE | ENST00000504578.1 | ENSG00000251513.1 | -0.804315714 | -1.812388498 | 0.069926189 |
| ELANE | ENST00000505556.1 | ENSG00000249409.1 | 0.80414605 | 1.806078119 | 0.070906138 |
| ELANE | ENST00000506791.1 | ENSG00000251131.1 | 0.808360141 | 1.840048686 | 0.06576109 |
| ELANE | ENST00000508083.1 | ENSG00000249343.1 | 0.865999424 | 1.96359971 | 0.049576531 |
| ELANE | ENST00000509036.1 | ENSG00000251131.1 | 0.843547155 | 1.899787068 | 0.057461069 |
| ELANE | ENST00000509192.1 | ENSG00000250765.1 | 0.838464319 | 1.856572084 | 0.063372059 |
| ELANE | ENST00000515128.1 | ENSG00000248215.1 | -0.915322945 | -2.053019045 | 0.04007073 |
| ELANE | ENST00000522600.1 | ENSG00000246582.2 | 0.975039111 | 2.176616137 | 0.02950922 |
| ELANE | ENST00000526611.1 | ENSG00000246982.2 | 0.88319283 | 1.993656672 | 0.046189588 |
| ELANE | ENST00000527086.1 | ENSG00000255182.1 | 0.848441012 | 1.900011977 | 0.057431548 |
| ELANE | ENST00000528887.1 | ENSG00000254501.1 | 0.926979041 | 2.066637391 | 0.038768335 |
| ELANE | ENST00000543072.1 | ENSG00000256092.2 | -0.914093923 | -2.025675285 | 0.042798071 |
| ELANE | ENST00000543275.1 | ENSG00000256944.1 | 0.961637127 | 2.13551985 | 0.032718571 |
| ELANE | ENST00000543494.1 | ENSG00000256514.1 | 0.96447917 | 2.159368012 | 0.030821627 |
| ELANE | ENST00000549683.1 | ENSG00000257953.1 | 0.881570127 | 1.969567923 | 0.048887911 |
| ELANE | ENST00000549878.1 | ENSG00000257284.1 | 0.856136858 | 1.91267997 | 0.055789035 |
| ELANE | ENST00000551135.1 | ENSG00000258294.1 | -0.820488993 | -1.837655978 | 0.066113115 |
| ELANE | ENST00000558575.1 | ENSG00000259687.1 | 0.922190862 | 2.026103819 | 0.042754147 |
| ELANE | ENST00000563018.1 | ENSG00000260193.1 | 0.886839962 | 1.982403387 | 0.047434111 |
| ELANE | ENST00000563610.1 | ENSG00000260051.1 | 0.84903119 | 1.899804541 | 0.057458775 |
| ELANE | ENST00000563611.1 | ENSG00000261583.1 | 0.894592766 | 1.98948298 | 0.046647919 |
| ELANE | ENST00000565823.1 | ENSG00000260686.1 | -0.957656217 | -2.145906383 | 0.031880448 |
| ELANE | ENST00000565829.1 | ENSG00000260148.1 | 0.909041648 | 2.011822433 | 0.044238658 |
| ELANE | ENST00000568659.1 | ENSG00000260004.1 | -0.861217094 | -1.924577517 | 0.054282234 |
| ELANE | ENST00000570493.2 | ENSG00000261898.2 | 0.836388647 | 1.871177712 | 0.061320453 |
| ELANE | ENST00000570843.1 | ENSG00000261889.1 | 0.910595867 | 2.056980718 | 0.039688081 |
| ELANE | ENST00000570929.1 | ENSG00000262223.2 | 0.934548007 | 2.073686723 | 0.038108415 |
| ELANE | ENST00000570974.1 | ENSG00000263300.1 | 0.904749073 | 1.96990977 | 0.048848712 |
| ELANE | ENST00000576086.1 | ENSG00000262823.1 | 0.933263532 | 2.066703948 | 0.038762059 |
| ELANE | ENST00000577064.1 | ENSG00000262823.1 | 0.874703835 | 1.952567455 | 0.050870871 |

| | | | | | |
|---|---|---|---|---|---|
| ELANE | ENST00000577176.1 | ENSG00000262823.1 | 0.889432887 | 2.005961637 | 0.044860341 |
| ELANE | ENST00000577853.1 | ENSG00000264207.1 | 0.817485886 | 1.824115794 | 0.068134568 |
| ELANE | ENST00000578800.1 | ENSG00000264235.1 | 0.819163465 | 1.825905957 | 0.067864429 |
| ELANE | ENST00000578936.1 | ENSG00000265547.1 | 0.813751114 | 1.816122911 | 0.06935152 |
| ELANE | ENST00000585559.1 | ENSG00000267117.1 | 0.833772489 | 1.848654509 | 0.064507716 |
| ELANE | ENST00000588402.1 | ENSG00000267006.1 | -0.835347195 | -1.875892432 | 0.060670052 |
| ELANE | ENST00000589673.1 | ENSG00000267755.1 | 0.826437275 | 1.832791103 | 0.066833643 |
| ELANE | ENST00000590328.1 | ENSG00000256995.2 | -0.890709965 | -2.004988996 | 0.044964223 |
| ELANE | ENST00000591174.1 | ENSG00000267289.1 | 0.983772355 | 2.168336542 | 0.03013309 |
| ELANE | ENST00000593642.1 | ENSG00000267858.1 | 0.81280081 | 1.811765503 | 0.070022438 |
| ELANE | ENST00000594590.2 | ENSG00000268199.2 | 0.927499504 | 2.08936543 | 0.036674841 |
| ELANE | ENST00000596887.1 | ENSG00000237031.3 | -0.862878915 | -1.912082064 | 0.055865669 |
| ELANE | ENST00000597309.1 | ENSG00000232098.2 | -0.856600549 | -1.929643537 | 0.05365102 |
| ELANE | ENST00000598092.1 | ENSG00000228065.6 | -0.922421257 | -2.052131819 | 0.040156853 |
| ELANE | ENST00000599259.1 | ENSG00000269352.1 | 0.885030687 | 1.986579008 | 0.046969069 |
| ELANE | ENST00000600007.1 | ENSG00000268655.1 | 0.811751745 | 1.81477251 | 0.069558877 |
| ELANE | ENST00000600534.1 | ENSG00000267858.1 | 0.909375917 | 2.060228174 | 0.039376733 |
| ELANE | ENST00000600716.1 | ENSG00000269487.1 | 0.909800616 | 2.048814835 | 0.040480225 |
| ELANE | ENST00000600726.1 | ENSG00000267858.1 | 0.980308477 | 2.19816764 | 0.027937162 |
| ELANE | ENST00000602532.1 | ENSG00000270091.1 | 0.847174566 | 1.892363914 | 0.058442511 |
| ELANE | ENST00000604142.1 | ENSG00000271308.1 | 0.872889048 | 1.961545663 | 0.049815404 |
| ELANE | ENST00000607476.1 | ENSG00000272540.1 | 0.879250041 | 1.944276225 | 0.05186215 |
| ELANE | ENST00000607943.1 | ENSG00000273188.1 | 0.931782793 | 2.084516244 | 0.037113234 |
| ELANE | ENST00000608489.1 | ENSG00000272716.1 | 0.871321276 | 1.939103767 | 0.052488704 |
| ELANE | ENST00000609113.1 | ENSG00000272827.1 | 0.9576414 | 2.117688224 | 0.034201481 |
| ELANE | ENST00000609976.1 | ENSG00000272582.1 | 0.934228771 | 2.106763266 | 0.035138105 |
| ELANE | ENST00000610145.1 | ENSG00000273175.1 | 0.871090847 | 1.977783999 | 0.047953079 |
| ELANE | ENST00000610270.1 | ENSG00000272576.1 | -0.868023905 | -1.954810504 | 0.050605441 |
| ELANE | NR_003604.2 | ZFAS1 | 0.922934135 | 2.083058174 | 0.037245921 |
| ELANE | NR_003605.1 | ZFAS1 | 0.911076831 | 2.068487312 | 0.038594222 |
| ELANE | NR_003606.2 | ZFAS1 | 0.902336819 | 2.011484084 | 0.04427435 |
| ELANE | NR_026802.1 | FAM74A4 | 0.849974501 | 1.89846172 | 0.057635286 |
| ELANE | NR_027052.1 | THAP7-AS1 | 0.867441426 | 1.965317656 | 0.049377483 |
| ELANE | NR_027271.1 | CIRBP-AS1 | 0.914374801 | 2.042366234 | 0.041115216 |
| ELANE | NR_027334.2 | MZF1-AS1 | 0.911515486 | 2.02128616 | 0.043250151 |
| ELANE | NR_036480.1 | VPS9D1-AS1 | 0.926905352 | 2.062513881 | 0.039158837 |
| ELANE | NR_036658.1 | ZFAS1 | 0.90325123 | 1.991926753 | 0.046379095 |
| ELANE | NR_038421.1 | LINC01220 | 0.935941362 | 2.089859975 | 0.03663038 |
| ELANE | NR_038923.1 | SSSCA1-AS1 | 0.840409971 | 1.880318769 | 0.060064647 |
| ELANE | NR_073552.1 | LOC101059948 | 0.811751745 | 1.803736445 | 0.071272633 |
| ELANE | NR_104158.1 | NRG1-IT1 | 0.834471221 | 1.847023551 | 0.064743727 |
| ELANE | NR_105010.1 | LINC01333 | 0.861317054 | 1.91673745 | 0.055271295 |
| ELANE | NR_108036.1 | CFAP58-AS1 | 0.90208897 | 2.013887571 | 0.044021339 |
| ELANE | NR_110556.1 | LOC102724890 | -0.832432722 | -1.877202855 | 0.060490296 |
| ELANE | NR_110630.1 | LOC101927478 | 0.812731168 | 1.826583899 | 0.067762356 |
| ELANE | NR_110919.1 | LOC101928530 | 0.806668974 | 1.830381641 | 0.067192891 |
| ELANE | NR_110998.1 | FAM74A4 | 0.849974501 | 1.894437322 | 0.058166987 |
| ELANE | NR_111951.1 | LINC00869 | 0.878089637 | 1.951480674 | 0.050999893 |
| ELANE | NR_111952.1 | LINC00869 | 0.893812758 | 2.020819061 | 0.043298499 |
| ELANE | NR_111953.1 | LINC00869 | 0.868385035 | 1.955173547 | 0.05056259 |
| ELANE | NR_121188.1 | PGM5P3-AS1 | -0.888578745 | -2.001700553 | 0.045316947 |

| | | | | | |
|---|---|---|---|---|---|
| ELANE | NR_121189.1 | PGM5P3-AS1 | -0.877098336 | -1.976212589 | 0.048130705 |
| ELANE | NR_125957.1 | LOC101928626 | -0.808574598 | -1.826378342 | 0.067793292 |
| ELANE | NR_126522.1 | EXOC3-AS1 | 0.850253362 | 1.93082608 | 0.053504564 |
| ELANE | NR_130143.1 | LOC104968399 | 0.835755483 | 1.891932012 | 0.05850004 |
| ELANE | NR_135024.1 | LOC105369747 | 0.825901704 | 1.859108987 | 0.063011692 |
| ELANE | NR_135584.1 | LOC101927596 | 0.808315717 | 1.787222969 | 0.073901458 |
| ELANE | NR_144459.1 | ARSD-AS1 | 0.885667911 | 1.971399999 | 0.048678141 |
| EPOR | ENST00000318291.4 | ENSG00000177406.4 | 0.911934523 | 2.041823018 | 0.04116909 |
| EPOR | ENST00000399186.2 | ENSG00000214888.2 | 0.82058416 | 1.842013354 | 0.065473197 |
| EPOR | ENST00000412085.1 | ENSG00000233825.1 | 0.861446257 | 1.924406676 | 0.054303628 |
| EPOR | ENST00000412759.1 | ENSG00000236933.1 | 0.945007807 | 2.119906763 | 0.034013909 |
| EPOR | ENST00000413650.1 | ENSG00000230880.2 | 0.832119858 | 1.869275639 | 0.061584476 |
| EPOR | ENST00000413991.1 | ENSG00000237614.1 | 0.843852432 | 1.907649528 | 0.056436529 |
| EPOR | ENST00000414740.2 | ENSG00000229646.2 | 0.90459202 | 2.001545741 | 0.04533361 |
| EPOR | ENST00000420365.1 | ENSG00000225214.1 | 0.838096782 | 1.852740427 | 0.063919573 |
| EPOR | ENST00000421020.1 | ENSG00000231407.1 | 0.821263694 | 1.829050447 | 0.067392051 |
| EPOR | ENST00000423869.1 | ENSG00000227848.1 | 0.806072298 | 1.804413458 | 0.071166515 |
| EPOR | ENST00000426237.2 | ENSG00000235527.2 | 0.824416701 | 1.810404614 | 0.070233064 |
| EPOR | ENST00000426519.1 | ENSG00000234142.1 | 0.940597277 | 2.09824755 | 0.035883283 |
| EPOR | ENST00000429796.1 | ENSG00000231858.1 | -0.824693438 | -1.855630037 | 0.063506309 |
| EPOR | ENST00000433614.1 | ENSG00000228534.1 | -0.827530823 | -1.843176161 | 0.065303294 |
| EPOR | ENST00000433876.2 | ENSG00000228423.2 | 0.921308926 | 2.052820499 | 0.04008999 |
| EPOR | ENST00000434627.1 | ENSG00000230074.1 | 0.937676843 | 2.092259731 | 0.036415288 |
| EPOR | ENST00000435434.1 | ENSG00000231233.1 | 0.943649438 | 2.103763337 | 0.035399096 |
| EPOR | ENST00000435892.1 | ENSG00000233635.2 | 0.969426384 | 2.181183877 | 0.029169819 |
| EPOR | ENST00000438107.1 | ENSG00000234449.2 | 0.863653936 | 1.922296227 | 0.054568495 |
| EPOR | ENST00000438190.1 | ENSG00000227214.2 | 0.89570496 | 2.002542784 | 0.045226386 |
| EPOR | ENST00000441875.1 | ENSG00000239203.1 | 0.840572672 | 1.8620261 | 0.062599411 |
| EPOR | ENST00000442069.1 | ENSG00000225655.1 | -0.896948193 | -2.008275649 | 0.044614008 |
| EPOR | ENST00000442850.1 | ENSG00000232600.2 | -0.894217022 | -1.963071878 | 0.049637823 |
| EPOR | ENST00000443380.1 | ENSG00000224371.1 | 0.85400042 | 1.898150919 | 0.057676204 |
| EPOR | ENST00000444665.1 | ENSG00000228852.2 | 0.868912552 | 1.938367923 | 0.052578351 |
| EPOR | ENST00000447206.1 | ENSG00000230839.1 | 0.978536257 | 2.192108997 | 0.028371637 |
| EPOR | ENST00000450109.1 | ENSG00000225376.1 | 0.850136868 | 1.884029248 | 0.059561019 |
| EPOR | ENST00000451656.1 | ENSG00000228417.1 | 0.847051259 | 1.881339214 | 0.05992579 |
| EPOR | ENST00000453051.1 | ENSG00000229407.1 | 0.890423456 | 1.979742202 | 0.047732504 |
| EPOR | ENST00000454530.1 | ENSG00000226649.1 | -0.940393257 | -2.082462278 | 0.037300265 |
| EPOR | ENST00000456715.1 | ENSG00000224893.1 | 0.910379413 | 2.066737591 | 0.038758887 |
| EPOR | ENST00000457115.1 | ENSG00000227245.1 | 0.903922753 | 2.014473227 | 0.043959873 |
| EPOR | ENST00000457848.1 | ENSG00000226412.1 | 0.827519555 | 1.841794762 | 0.065505177 |
| EPOR | ENST00000458154.1 | ENSG00000235578.1 | 0.845391778 | 1.913623766 | 0.055668246 |
| EPOR | ENST00000458194.1 | ENSG00000226193.1 | 0.955863233 | 2.121940597 | 0.033842727 |
| EPOR | ENST00000458364.1 | ENSG00000225655.1 | -0.928325954 | -2.098409714 | 0.035868968 |
| EPOR | ENST00000459985.1 | ENSG00000273066.1 | 0.983374535 | 2.216613207 | 0.026649534 |
| EPOR | ENST00000489557.2 | ENSG00000257045.1 | 0.898703315 | 2.01579417 | 0.043821503 |
| EPOR | ENST00000490013.1 | ENSG00000184115.12 | 0.895641439 | 1.97001598 | 0.048836539 |
| EPOR | ENST00000493123.1 | ENSG00000242428.1 | 0.839945183 | 1.857646516 | 0.063219228 |
| EPOR | ENST00000498693.1 | ENSG00000244198.1 | 0.928769125 | 2.093182789 | 0.036332841 |
| EPOR | ENST00000505196.1 | ENSG00000248131.1 | 0.92208472 | 2.060427481 | 0.039357692 |
| EPOR | ENST00000505498.1 | ENSG00000250908.1 | 0.82044013 | 1.86032899 | 0.062838994 |
| EPOR | ENST00000505556.1 | ENSG00000249409.1 | 0.947347898 | 2.127670467 | 0.033364415 |

| | | | | | |
|---|---|---|---|---|---|
| EPOR | ENST00000506100.1 | ENSG00000249409.1 | 0.95152757 | 2.131097987 | 0.033081071 |
| EPOR | ENST00000508083.1 | ENSG00000249343.1 | 0.95928345 | 2.128959837 | 0.033257583 |
| EPOR | ENST00000508188.1 | ENSG00000250999.1 | 0.858322918 | 1.910283561 | 0.056096713 |
| EPOR | ENST00000509036.1 | ENSG00000251131.1 | 0.849166621 | 1.915645794 | 0.055410196 |
| EPOR | ENST00000514411.1 | ENSG00000250882.1 | 0.865330384 | 1.967065028 | 0.049175714 |
| EPOR | ENST00000514877.1 | ENSG00000248685.1 | 0.88777735 | 1.976072237 | 0.048146596 |
| EPOR | ENST00000517300.1 | ENSG00000254144.2 | 0.880415619 | 1.94978176 | 0.051202136 |
| EPOR | ENST00000519451.1 | ENSG00000253363.1 | 0.85141096 | 1.90960068 | 0.056184647 |
| EPOR | ENST00000519852.1 | ENSG00000253716.1 | 0.88501329 | 1.976129746 | 0.048140084 |
| EPOR | ENST00000521207.1 | ENSG00000253716.1 | 0.913528531 | 2.050375447 | 0.040327807 |
| EPOR | ENST00000522547.1 | ENSG00000253430.1 | -0.907855369 | -2.004607946 | 0.045004976 |
| EPOR | ENST00000522600.1 | ENSG00000246582.2 | 0.926447684 | 2.072392867 | 0.038228818 |
| EPOR | ENST00000524335.1 | ENSG00000253716.1 | 0.907519562 | 2.05025871 | 0.040339192 |
| EPOR | ENST00000526611.1 | ENSG00000246982.2 | 0.948549953 | 2.124461381 | 0.033631582 |
| EPOR | ENST00000528000.1 | ENSG00000254804.1 | 0.913150572 | 2.051660433 | 0.040202675 |
| EPOR | ENST00000528887.1 | ENSG00000254501.1 | 0.884721275 | 1.974346238 | 0.048342387 |
| EPOR | ENST00000536141.1 | ENSG00000256969.1 | 0.888656236 | 1.98185344 | 0.047495646 |
| EPOR | ENST00000537269.1 | ENSG00000257084.1 | 0.936394702 | 2.11213201 | 0.034675128 |
| EPOR | ENST00000543072.1 | ENSG00000256092.2 | -0.982810568 | -2.229685068 | 0.025768358 |
| EPOR | ENST00000543275.1 | ENSG00000256944.1 | 0.903091119 | 2.01586149 | 0.043814462 |
| EPOR | ENST00000543494.1 | ENSG00000256514.1 | 0.816702587 | 1.794241575 | 0.072774619 |
| EPOR | ENST00000545177.3 | ENSG00000230438.5 | 0.804205393 | 1.781211134 | 0.074877965 |
| EPOR | ENST00000549878.1 | ENSG00000257284.1 | 0.917266462 | 2.049909986 | 0.040373216 |
| EPOR | ENST00000552469.1 | ENSG00000258325.1 | 0.812309965 | 1.839857135 | 0.065789215 |
| EPOR | ENST00000554049.1 | ENSG00000258763.1 | 0.836630893 | 1.865728744 | 0.062079326 |
| EPOR | ENST00000554798.1 | ENSG00000258483.1 | 0.826890397 | 1.839896018 | 0.065783505 |
| EPOR | ENST00000558575.1 | ENSG00000259687.1 | 0.835163989 | 1.85204452 | 0.06401943 |
| EPOR | ENST00000559569.1 | ENSG00000259760.1 | -0.813338459 | -1.823001643 | 0.068303142 |
| EPOR | ENST00000565359.1 | ENSG00000260601.1 | 0.825292335 | 1.845036669 | 0.065032205 |
| EPOR | ENST00000565823.1 | ENSG00000260686.1 | -0.933620617 | -2.08744169 | 0.036848227 |
| EPOR | ENST00000565829.1 | ENSG00000260148.1 | 0.992531645 | 2.193960256 | 0.028238267 |
| EPOR | ENST00000570512.1 | ENSG00000262768.1 | 0.801826071 | 1.788698556 | 0.073663376 |
| EPOR | ENST00000570843.1 | ENSG00000261889.1 | 0.991920462 | 2.209423017 | 0.027145232 |
| EPOR | ENST00000570929.1 | ENSG00000262223.2 | 0.985489609 | 2.227984504 | 0.025881548 |
| EPOR | ENST00000571815.1 | ENSG00000262810.1 | 0.873378764 | 1.964634862 | 0.049456514 |
| EPOR | ENST00000574460.1 | ENSG00000263051.1 | 0.90423667 | 2.044297655 | 0.040924151 |
| EPOR | ENST00000575139.1 | ENSG00000263072.1 | 0.863295275 | 1.942859653 | 0.052033118 |
| EPOR | ENST00000576086.1 | ENSG00000262823.1 | 0.901340824 | 2.0233301 | 0.043039126 |
| EPOR | ENST00000577064.1 | ENSG00000262823.1 | 0.965208929 | 2.159881614 | 0.030781835 |
| EPOR | ENST00000577176.1 | ENSG00000262823.1 | 0.871849035 | 1.972788474 | 0.048519667 |
| EPOR | ENST00000578800.1 | ENSG00000264235.1 | 0.811043525 | 1.823890908 | 0.068168567 |
| EPOR | ENST00000578936.1 | ENSG00000265547.1 | 0.887147861 | 2.019985585 | 0.043384883 |
| EPOR | ENST00000581905.1 | ENSG00000264235.1 | 0.893508555 | 1.994146009 | 0.0461361 |
| EPOR | ENST00000585072.1 | ENSG00000263745.1 | 0.817960091 | 1.814887484 | 0.069541202 |
| EPOR | ENST00000586010.1 | ENSG00000267606.1 | 0.824790556 | 1.832174824 | 0.066925378 |
| EPOR | ENST00000586051.1 | ENSG00000267576.1 | 0.937846459 | 2.079755358 | 0.037547978 |
| EPOR | ENST00000589673.1 | ENSG00000267755.1 | 0.829352635 | 1.891625836 | 0.058540851 |
| EPOR | ENST00000591174.1 | ENSG00000267289.1 | 0.906465779 | 2.019709036 | 0.043413577 |
| EPOR | ENST00000593632.1 | ENSG00000180279.5 | 0.90417721 | 2.01360374 | 0.044051153 |
| EPOR | ENST00000594590.2 | ENSG00000268199.2 | 0.974362483 | 2.198173871 | 0.027936718 |
| EPOR | ENST00000595955.1 | ENSG00000268401.1 | 0.859825444 | 1.944277168 | 0.051862036 |

| | | | | | |
|---|---|---|---|---|---|
| EPOR | ENST00000597169.1 | ENSG00000269720.1 | 0.837572758 | 1.901088025 | 0.057290484 |
| EPOR | ENST00000597309.1 | ENSG00000232098.2 | -0.929002433 | -2.08566533 | 0.037008949 |
| EPOR | ENST00000599259.1 | ENSG00000269352.1 | 0.865547835 | 1.930713894 | 0.053518444 |
| EPOR | ENST00000600071.1 | ENSG00000269199.1 | 0.82647069 | 1.868151758 | 0.061740921 |
| EPOR | ENST00000600234.1 | ENSG00000268078.1 | 0.886609557 | 1.951589219 | 0.050986994 |
| EPOR | ENST00000600534.1 | ENSG00000267858.1 | 0.989630336 | 2.241702916 | 0.024980579 |
| EPOR | ENST00000600726.1 | ENSG00000267858.1 | 0.868595871 | 1.948448074 | 0.051361372 |
| EPOR | ENST00000600889.1 | ENSG00000232675.3 | 0.865450624 | 1.920456012 | 0.054800324 |
| EPOR | ENST00000601033.1 | ENSG00000268401.1 | 0.81818763 | 1.836893923 | 0.066225557 |
| EPOR | ENST00000602532.1 | ENSG00000270091.1 | 0.96218236 | 2.123825084 | 0.033684773 |
| EPOR | ENST00000602872.1 | ENSG00000270067.1 | 0.806467559 | 1.814241924 | 0.069640488 |
| EPOR | ENST00000604142.1 | ENSG00000271308.1 | 0.818201809 | 1.8133264 | 0.069781494 |
| EPOR | ENST00000606068.1 | ENSG00000272342.1 | 0.894010884 | 1.997408315 | 0.045780845 |
| EPOR | ENST00000607014.1 | ENSG00000272345.1 | -0.820409733 | -1.848927525 | 0.064468279 |
| EPOR | ENST00000607224.1 | ENSG00000272521.1 | 0.831816638 | 1.859566716 | 0.062946852 |
| EPOR | ENST00000607476.1 | ENSG00000272540.1 | 0.824632121 | 1.855211429 | 0.06356604 |
| EPOR | ENST00000607943.1 | ENSG00000273188.1 | 0.807918148 | 1.817743328 | 0.069103372 |
| EPOR | ENST00000608940.1 | ENSG00000272763.1 | 0.815054974 | 1.819983197 | 0.068761564 |
| EPOR | ENST00000608952.1 | ENSG00000272689.1 | -0.826596533 | -1.831212203 | 0.067068876 |
| EPOR | ENST00000609067.1 | ENSG00000272849.1 | 0.80840252 | 1.838468424 | 0.065993411 |
| EPOR | ENST00000609113.1 | ENSG00000272827.1 | 0.803909143 | 1.822665229 | 0.06835411 |
| EPOR | ENST00000609281.1 | ENSG00000273320.1 | 0.930620368 | 2.079091777 | 0.037608916 |
| EPOR | ENST00000610145.1 | ENSG00000273175.1 | 0.936996134 | 2.109026639 | 0.034942282 |
| EPOR | NR_003604.2 | ZFAS1 | 0.970913138 | 2.156622043 | 0.031035126 |
| EPOR | NR_003605.1 | ZFAS1 | 0.903032032 | 2.079617257 | 0.037560653 |
| EPOR | NR_003606.2 | ZFAS1 | 0.971978668 | 2.16603771 | 0.030308305 |
| EPOR | NR_026802.1 | FAM74A4 | 0.835777321 | 1.860110247 | 0.06286993 |
| EPOR | NR_027271.1 | CIRBP-AS1 | 0.948329409 | 2.120543617 | 0.033960227 |
| EPOR | NR_027334.2 | MZF1-AS1 | 0.990388598 | 2.211163364 | 0.027024526 |
| EPOR | NR_034037.1 | LINC00582 | -0.891502087 | -2.000980194 | 0.045394524 |
| EPOR | NR_036480.1 | VPS9D1-AS1 | 0.837007841 | 1.869891205 | 0.061498927 |
| EPOR | NR_036658.1 | ZFAS1 | 0.968752799 | 2.16365686 | 0.030490694 |
| EPOR | NR_046454.1 | LINC00907 | -0.876055997 | -1.958811004 | 0.050134924 |
| EPOR | NR_103790.1 | LINC00581 | -0.860780508 | -1.893788562 | 0.05825308 |
| EPOR | NR_104158.1 | NRG1-IT1 | 0.933925507 | 2.086452174 | 0.036937683 |
| EPOR | NR_105010.1 | LINC01333 | 0.903145174 | 2.014445767 | 0.043962753 |
| EPOR | NR_108036.1 | CFAP58-AS1 | 0.942837883 | 2.098434143 | 0.035866812 |
| EPOR | NR_110568.1 | LOC101927661 | 0.850298445 | 1.90055479 | 0.057360352 |
| EPOR | NR_110919.1 | LOC101928530 | 0.876711345 | 1.959508741 | 0.050053237 |
| EPOR | NR_110998.1 | FAM74A4 | 0.835777321 | 1.890712503 | 0.058662733 |
| EPOR | NR_117098.1 | LINC01353 | 0.872354496 | 1.925196433 | 0.054204788 |
| EPOR | NR_121188.1 | PGM5P3-AS1 | -0.880177653 | -1.982344139 | 0.047440737 |
| EPOR | NR_121189.1 | PGM5P3-AS1 | -0.925312303 | -2.059227519 | 0.039472448 |
| EPOR | NR_130143.1 | LOC104968399 | 0.858151714 | 1.940505752 | 0.052318256 |
| EPOR | NR_130144.1 | LOC104968399 | 0.893508555 | 2.005369926 | 0.044923514 |
| EPOR | NR_135032.1 | LOC105369635 | 0.936394702 | 2.103201073 | 0.035448197 |
| EPOR | NR_135644.1 | LOC105371506 | -0.943650234 | -2.137782978 | 0.032534361 |
| EXOSC4 | ENST00000318291.4 | ENSG00000177406.4 | 0.875203431 | 1.95448773 | 0.050643565 |
| EXOSC4 | ENST00000413353.1 | ENSG00000232893.1 | 0.873732487 | 1.955236338 | 0.050555181 |
| EXOSC4 | ENST00000413991.1 | ENSG00000237614.1 | 0.864268237 | 1.94682842 | 0.051555309 |
| EXOSC4 | ENST00000416220.1 | ENSG00000236753.1 | 0.836285301 | 1.861123875 | 0.062726685 |

| | | | | | |
|---|---|---|---|---|---|
| EXOSC4 | ENST00000420365.1 | ENSG00000225214.1 | 0.90950557 | 2.00244505 | 0.045236887 |
| EXOSC4 | ENST00000423667.1 | ENSG00000225970.1 | 0.865206429 | 1.931123628 | 0.053467766 |
| EXOSC4 | ENST00000424735.1 | ENSG00000237457.2 | -0.897793651 | -2.010509704 | 0.04437727 |
| EXOSC4 | ENST00000425624.1 | ENSG00000223779.4 | 0.898984879 | 1.992519518 | 0.046314086 |
| EXOSC4 | ENST00000426699.1 | ENSG00000229308.1 | 0.932000858 | 2.065554974 | 0.03887052 |
| EXOSC4 | ENST00000433344.1 | ENSG00000234083.1 | -0.975200929 | -2.176106279 | 0.029547315 |
| EXOSC4 | ENST00000433614.1 | ENSG00000228534.1 | -0.84300605 | -1.870674847 | 0.061390163 |
| EXOSC4 | ENST00000435434.1 | ENSG00000231233.1 | 0.911030417 | 2.022902746 | 0.043083175 |
| EXOSC4 | ENST00000435733.1 | ENSG00000226377.1 | 0.808001164 | 1.810208064 | 0.070263528 |
| EXOSC4 | ENST00000435992.2 | ENSG00000232675.3 | 0.876316262 | 1.982666963 | 0.047404642 |
| EXOSC4 | ENST00000439186.1 | ENSG00000237076.1 | 0.899132866 | 2.011555713 | 0.044266792 |
| EXOSC4 | ENST00000442069.1 | ENSG00000225655.1 | -0.870031062 | -1.932659134 | 0.053278203 |
| EXOSC4 | ENST00000444245.1 | ENSG00000236753.1 | 0.847515071 | 1.881407496 | 0.059916508 |
| EXOSC4 | ENST00000447514.1 | ENSG00000236753.1 | 0.910381186 | 2.035416178 | 0.041809015 |
| EXOSC4 | ENST00000450848.1 | ENSG00000225539.1 | 0.852546381 | 1.884323619 | 0.059521215 |
| EXOSC4 | ENST00000451656.1 | ENSG00000228417.1 | 0.861006324 | 1.943307518 | 0.051979014 |
| EXOSC4 | ENST00000454515.1 | ENSG00000236753.1 | 0.85925001 | 1.923678656 | 0.054394875 |
| EXOSC4 | ENST00000454526.1 | ENSG00000234136.1 | 0.836533024 | 1.87326493 | 0.06103181 |
| EXOSC4 | ENST00000455699.1 | ENSG00000240996.1 | 0.80897259 | 1.802889281 | 0.071405605 |
| EXOSC4 | ENST00000455788.1 | ENSG00000236263.1 | 0.821882348 | 1.816620798 | 0.069275197 |
| EXOSC4 | ENST00000457115.1 | ENSG00000227245.1 | 0.833640717 | 1.865321974 | 0.062136287 |
| EXOSC4 | ENST00000458364.1 | ENSG00000225655.1 | -0.844321736 | -1.890515189 | 0.058689091 |
| EXOSC4 | ENST00000459985.1 | ENSG00000273066.1 | 0.825759254 | 1.870168565 | 0.061460414 |
| EXOSC4 | ENST00000484413.1 | ENSG00000271853.1 | 0.847170838 | 1.875030356 | 0.060788548 |
| EXOSC4 | ENST00000501405.2 | ENSG00000247402.2 | -0.888383747 | -1.979923345 | 0.047712143 |
| EXOSC4 | ENST00000504891.1 | ENSG00000249388.1 | 0.903893062 | 2.048786145 | 0.040483031 |
| EXOSC4 | ENST00000505498.1 | ENSG00000250908.1 | 0.986160037 | 2.215441902 | 0.026729748 |
| EXOSC4 | ENST00000508083.1 | ENSG00000249343.1 | 0.810508381 | 1.820156973 | 0.068735103 |
| EXOSC4 | ENST00000515128.1 | ENSG00000248215.1 | -0.800882919 | -1.796376583 | 0.072434645 |
| EXOSC4 | ENST00000520749.1 | ENSG00000253717.1 | -0.91816556 | -2.056293593 | 0.039754226 |
| EXOSC4 | ENST00000521307.1 | ENSG00000253177.1 | 0.952425157 | 2.140275203 | 0.032332533 |
| EXOSC4 | ENST00000521953.1 | ENSG00000253214.1 | 0.90602228 | 2.028353051 | 0.042524228 |
| EXOSC4 | ENST00000522600.1 | ENSG00000246582.2 | 0.80049492 | 1.783907159 | 0.074438752 |
| EXOSC4 | ENST00000522704.1 | ENSG00000254135.1 | 0.964881035 | 2.131802993 | 0.033023045 |
| EXOSC4 | ENST00000524818.1 | ENSG00000254473.1 | 0.819019039 | 1.826227109 | 0.06781606 |
| EXOSC4 | ENST00000526611.1 | ENSG00000246982.2 | 0.883082847 | 1.978195617 | 0.047906643 |
| EXOSC4 | ENST00000526935.1 | ENSG00000255372.1 | 0.849215703 | 1.934298541 | 0.053076433 |
| EXOSC4 | ENST00000528887.1 | ENSG00000254501.1 | 0.942952773 | 2.111523542 | 0.034727337 |
| EXOSC4 | ENST00000529837.1 | ENSG00000254687.1 | 0.837407966 | 1.864580561 | 0.06224022 |
| EXOSC4 | ENST00000544089.1 | ENSG00000256273.1 | 0.800098843 | 1.808109728 | 0.070589424 |
| EXOSC4 | ENST00000547834.1 | ENSG00000258325.1 | 0.976284234 | 2.179381143 | 0.029303366 |
| EXOSC4 | ENST00000549140.1 | ENSG00000258332.1 | 0.819449994 | 1.840134112 | 0.06574855 |
| EXOSC4 | ENST00000549878.1 | ENSG00000257284.1 | 0.881514505 | 1.966877786 | 0.049197302 |
| EXOSC4 | ENST00000555966.1 | ENSG00000258843.1 | 0.823367941 | 1.829644573 | 0.067303104 |
| EXOSC4 | ENST00000556786.1 | ENSG00000258525.1 | -0.885343199 | -1.987596193 | 0.046856368 |
| EXOSC4 | ENST00000563841.1 | ENSG00000261029.1 | 0.822632698 | 1.844740113 | 0.065075353 |
| EXOSC4 | ENST00000565823.1 | ENSG00000260686.1 | -0.855985252 | -1.921557414 | 0.054661472 |
| EXOSC4 | ENST00000565965.1 | ENSG00000261172.1 | 0.856280022 | 1.911573855 | 0.055930875 |
| EXOSC4 | ENST00000567089.1 | ENSG00000261822.1 | 0.831017944 | 1.845800348 | 0.0649212 |
| EXOSC4 | ENST00000569981.1 | ENSG00000238045.5 | 0.824660003 | 1.849185725 | 0.064430999 |
| EXOSC4 | ENST00000570493.2 | ENSG00000261898.2 | 0.818080054 | 1.823697 | 0.068197893 |

| | | | | | |
|---|---|---|---|---|---|
| EXOSC4 | ENST00000570512.1 | ENSG00000262768.1 | 0.952618455 | 2.154917034 | 0.031168327 |
| EXOSC4 | ENST00000570929.1 | ENSG00000262223.2 | 0.832493332 | 1.880283015 | 0.060069517 |
| EXOSC4 | ENST00000576554.1 | ENSG00000262413.1 | 0.980529795 | 2.209911776 | 0.027111286 |
| EXOSC4 | ENST00000577064.1 | ENSG00000262823.1 | 0.809785314 | 1.789237002 | 0.073576655 |
| EXOSC4 | ENST00000577176.1 | ENSG00000262823.1 | 0.891681424 | 2.013085318 | 0.044105655 |
| EXOSC4 | ENST00000577698.1 | ENSG00000265100.1 | 0.962872225 | 2.158084997 | 0.030921224 |
| EXOSC4 | ENST00000584758.1 | ENSG00000265356.1 | 0.815270425 | 1.816435979 | 0.06930352 |
| EXOSC4 | ENST00000585559.1 | ENSG00000267117.1 | 0.889896 | 1.992082444 | 0.046362013 |
| EXOSC4 | ENST00000586010.1 | ENSG00000267606.1 | 0.833760891 | 1.881164311 | 0.059949571 |
| EXOSC4 | ENST00000588799.1 | ENSG00000267275.1 | 0.880374029 | 1.998968106 | 0.045611805 |
| EXOSC4 | ENST00000588945.1 | ENSG00000267275.1 | 0.806492105 | 1.779688082 | 0.075127022 |
| EXOSC4 | ENST00000596643.1 | ENSG00000269439.1 | 0.837576229 | 1.879190704 | 0.060218459 |
| EXOSC4 | ENST00000597309.1 | ENSG00000232098.2 | -0.906567232 | -2.04046848 | 0.041303687 |
| EXOSC4 | ENST00000599259.1 | ENSG00000269352.1 | 0.896237991 | 1.974858308 | 0.04828423 |
| EXOSC4 | ENST00000600489.1 | ENSG00000231898.4 | 0.917507537 | 2.053228491 | 0.040050422 |
| EXOSC4 | ENST00000600534.1 | ENSG00000267858.1 | 0.806206406 | 1.792782611 | 0.073007691 |
| EXOSC4 | ENST00000601692.1 | ENSG00000267874.1 | -0.851353736 | -1.898452448 | 0.057636506 |
| EXOSC4 | ENST00000602872.1 | ENSG00000270067.1 | 0.858378135 | 1.923481922 | 0.054419555 |
| EXOSC4 | ENST00000603474.1 | ENSG00000258929.2 | 0.803333314 | 1.800684249 | 0.071752662 |
| EXOSC4 | ENST00000606470.1 | ENSG00000271913.1 | 0.812120384 | 1.809936153 | 0.070305689 |
| EXOSC4 | ENST00000607222.1 | ENSG00000272106.1 | 0.897430332 | 1.998857448 | 0.04562378 |
| EXOSC4 | ENST00000607284.1 | ENSG00000272389.1 | 0.820815575 | 1.853538103 | 0.06380527 |
| EXOSC4 | ENST00000607839.1 | ENSG00000272030.1 | 0.848203103 | 1.922847029 | 0.054499264 |
| EXOSC4 | ENST00000607876.1 | ENSG00000272848.1 | 0.827943037 | 1.852682887 | 0.063927824 |
| EXOSC4 | ENST00000608476.1 | ENSG00000232675.3 | 0.84853905 | 1.928576594 | 0.053783447 |
| EXOSC4 | ENST00000609610.1 | ENSG00000232675.3 | 0.867167765 | 1.947747515 | 0.051445182 |
| EXOSC4 | ENST00000609701.1 | ENSG00000273284.1 | 0.839295368 | 1.85863262 | 0.06307923 |
| EXOSC4 | ENST00000609972.1 | ENSG00000230651.3 | 0.885997877 | 1.974098305 | 0.048370566 |
| EXOSC4 | ENST00000610145.1 | ENSG00000273175.1 | 0.925465734 | 2.08534114 | 0.037038346 |
| EXOSC4 | ENST00000610161.1 | ENSG00000273059.1 | 0.849659478 | 1.871815651 | 0.061232112 |
| EXOSC4 | NR_003605.1 | ZFAS1 | 0.840073409 | 1.889805507 | 0.058783977 |
| EXOSC4 | NR_027334.2 | MZF1-AS1 | 0.823135987 | 1.852667592 | 0.063930018 |
| EXOSC4 | NR_036480.1 | VPS9D1-AS1 | 0.804485723 | 1.812533231 | 0.069903844 |
| EXOSC4 | NR_044996.1 | HCG23 | 0.930685964 | 2.086187622 | 0.036961631 |
| EXOSC4 | NR_108036.1 | CFAP58-AS1 | 0.921323579 | 2.080046209 | 0.037521295 |
| EXOSC4 | NR_110919.1 | LOC101928530 | 0.850564408 | 1.89224677 | 0.058458109 |
| EXOSC4 | NR_120335.1 | LOC101928414 | 0.800470066 | 1.78160022 | 0.074814448 |
| EXOSC4 | NR_121188.1 | PGM5P3-AS1 | -0.896565299 | -1.982434626 | 0.047430617 |
| EXOSC4 | NR_121189.1 | PGM5P3-AS1 | -0.883805898 | -1.988175246 | 0.046792312 |
| EXOSC4 | NR_126166.1 | FAM74A7 | 0.907748696 | 2.03332689 | 0.042019508 |
| EXOSC4 | NR_134597.1 | LOC105378068 | 0.858741061 | 1.94192576 | 0.052146088 |
| EXOSC4 | NR_135258.1 | LOC105370489 | 0.897401217 | 1.982131137 | 0.047464565 |
| EXOSC4 | NR_144459.1 | ARSD-AS1 | 0.82724156 | 1.847508905 | 0.064673419 |
| F12 | ENST00000318291.4 | ENSG00000177406.4 | 0.839859295 | 1.892143315 | 0.058471889 |
| F12 | ENST00000411694.1 | ENSG00000225331.1 | 0.860282587 | 1.941754399 | 0.05216684 |
| F12 | ENST00000412348.1 | ENSG00000228959.1 | -0.857287995 | -1.878758909 | 0.06027742 |
| F12 | ENST00000415205.1 | ENSG00000182057.4 | 0.887720046 | 1.987863958 | 0.046826738 |
| F12 | ENST00000417260.1 | ENSG00000231734.4 | -0.951930156 | -2.101573034 | 0.035590694 |
| F12 | ENST00000418387.1 | ENSG00000235056.1 | -0.920509467 | -2.037578708 | 0.041592083 |
| F12 | ENST00000419662.1 | ENSG00000228265.1 | 0.809612446 | 1.816298516 | 0.069324593 |
| F12 | ENST00000423428.1 | ENSG00000224048.1 | -0.834675246 | -1.852933868 | 0.063891838 |

| | | | | | |
|---|---|---|---|---|---|
| F12 | ENST00000426030.2 | ENSG00000228686.2 | -0.939146265 | -2.092512561 | 0.036392689 |
| F12 | ENST00000426302.1 | ENSG00000230454.1 | 0.840527339 | 1.871644005 | 0.061255871 |
| F12 | ENST00000430920.1 | ENSG00000234203.1 | 0.875775443 | 1.949318363 | 0.051257417 |
| F12 | ENST00000431730.1 | ENSG00000237401.2 | 0.931380684 | 2.07608787 | 0.037885825 |
| F12 | ENST00000433035.1 | ENSG00000230483.1 | 0.815790761 | 1.812116589 | 0.069968184 |
| F12 | ENST00000435287.1 | ENSG00000227220.1 | 0.864913977 | 1.949764925 | 0.051204144 |
| F12 | ENST00000436515.1 | ENSG00000224521.1 | -0.898570391 | -1.994432858 | 0.046104771 |
| F12 | ENST00000438190.1 | ENSG00000227214.2 | 0.814671338 | 1.811435541 | 0.070073458 |
| F12 | ENST00000438623.1 | ENSG00000224521.1 | -0.826308754 | -1.886438477 | 0.059235892 |
| F12 | ENST00000442829.1 | ENSG00000225284.1 | 0.943701083 | 2.130233013 | 0.033152381 |
| F12 | ENST00000446562.1 | ENSG00000233896.1 | 0.85505458 | 1.911093498 | 0.055992567 |
| F12 | ENST00000446816.1 | ENSG00000204685.5 | 0.885539116 | 2.007391549 | 0.044707988 |
| F12 | ENST00000448748.1 | ENSG00000231238.1 | -0.856064739 | -1.942267165 | 0.052104766 |
| F12 | ENST00000448858.1 | ENSG00000237734.1 | -0.870268097 | -1.929191903 | 0.053707043 |
| F12 | ENST00000451034.1 | ENSG00000229805.1 | -0.83320038 | -1.896921449 | 0.057838306 |
| F12 | ENST00000451090.1 | ENSG00000235215.2 | -0.811614499 | -1.817488397 | 0.069142363 |
| F12 | ENST00000451507.1 | ENSG00000229539.1 | 0.879746233 | 1.972325331 | 0.04857248 |
| F12 | ENST00000452176.1 | ENSG00000223659.1 | -0.936903695 | -2.108417797 | 0.034994866 |
| F12 | ENST00000455373.1 | ENSG00000226097.1 | -0.821358286 | -1.849798018 | 0.064342667 |
| F12 | ENST00000455788.1 | ENSG00000236263.1 | 0.843632149 | 1.888522442 | 0.058955848 |
| F12 | ENST00000457371.1 | ENSG00000237401.2 | 0.914044007 | 2.053609497 | 0.040013502 |
| F12 | ENST00000458154.1 | ENSG00000235578.1 | 0.830785953 | 1.855773104 | 0.063485906 |
| F12 | ENST00000463255.1 | ENSG00000243305.1 | -0.86939899 | -1.925557039 | 0.054159707 |
| F12 | ENST00000485338.1 | ENSG00000239641.1 | -0.832582639 | -1.864693356 | 0.062224399 |
| F12 | ENST00000488310.1 | ENSG00000240449.1 | 0.852644448 | 1.917050673 | 0.055231494 |
| F12 | ENST00000489077.1 | ENSG00000244198.1 | 0.857279356 | 1.930956265 | 0.053488461 |
| F12 | ENST00000489090.1 | ENSG00000240045.1 | -0.886296303 | -1.969639701 | 0.048879678 |
| F12 | ENST00000489690.1 | ENSG00000243944.1 | -0.970897313 | -2.198868691 | 0.02788726 |
| F12 | ENST00000500496.2 | ENSG00000245479.2 | 0.821907358 | 1.847231724 | 0.064713563 |
| F12 | ENST00000502467.1 | ENSG00000250530.1 | -0.823212192 | -1.841533243 | 0.065543454 |
| F12 | ENST00000503505.1 | ENSG00000248629.1 | -0.8641057 | -1.905488706 | 0.056716574 |
| F12 | ENST00000503723.1 | ENSG00000250472.1 | -0.883476998 | -1.992103148 | 0.046359742 |
| F12 | ENST00000504578.1 | ENSG00000251513.1 | -0.807018948 | -1.809234881 | 0.070414521 |
| F12 | ENST00000506791.1 | ENSG00000251131.1 | 0.836012065 | 1.876379077 | 0.060603246 |
| F12 | ENST00000509036.1 | ENSG00000251131.1 | 0.812533862 | 1.83629788 | 0.066313613 |
| F12 | ENST00000509192.1 | ENSG00000250765.1 | 0.856442215 | 1.922991917 | 0.054481065 |
| F12 | ENST00000509453.1 | ENSG00000249145.1 | 0.841254114 | 1.896262572 | 0.057925333 |
| F12 | ENST00000515128.1 | ENSG00000248215.1 | -0.934115443 | -2.070718321 | 0.038385127 |
| F12 | ENST00000517846.1 | ENSG00000254485.1 | 0.801113943 | 1.786301797 | 0.074050406 |
| F12 | ENST00000518837.1 | ENSG00000253947.1 | -0.811013478 | -1.816963293 | 0.069222734 |
| F12 | ENST00000520603.1 | ENSG00000254001.1 | -0.846950265 | -1.885010418 | 0.059428431 |
| F12 | ENST00000521653.1 | ENSG00000253301.1 | 0.875141819 | 1.97688199 | 0.048054971 |
| F12 | ENST00000529247.1 | ENSG00000254741.1 | 0.900669534 | 2.028982732 | 0.042460049 |
| F12 | ENST00000534178.1 | ENSG00000255120.1 | 0.960727444 | 2.157297952 | 0.030982456 |
| F12 | ENST00000537032.1 | ENSG00000255933.1 | 0.86892846 | 1.962536703 | 0.049700032 |
| F12 | ENST00000537850.1 | ENSG00000251002.3 | 0.875938155 | 1.933601249 | 0.053162174 |
| F12 | ENST00000543403.1 | ENSG00000256684.1 | -0.917063824 | -2.034511556 | 0.041900045 |
| F12 | ENST00000548722.2 | ENSG00000257194.2 | -0.84175418 | -1.862576783 | 0.062521833 |
| F12 | ENST00000549683.1 | ENSG00000257953.1 | 0.806419826 | 1.815134082 | 0.069503307 |
| F12 | ENST00000549806.1 | ENSG00000257252.1 | 0.877746435 | 1.939590661 | 0.052429457 |
| F12 | ENST00000550263.1 | ENSG00000257605.1 | 0.915312757 | 2.029894249 | 0.04236729 |

| | | | | | |
|---|---|---|---|---|---|
| F12 | ENST00000550279.1 | ENSG00000258338.1 | -0.855592538 | -1.909345577 | 0.056217526 |
| F12 | ENST00000554431.1 | ENSG00000258616.1 | -0.970595686 | -2.16149288 | 0.030657286 |
| F12 | ENST00000557602.1 | ENSG00000258616.1 | -0.970597353 | -2.166001559 | 0.030311068 |
| F12 | ENST00000558237.1 | ENSG00000259684.1 | -0.819630812 | -1.829175024 | 0.067373393 |
| F12 | ENST00000560963.1 | ENSG00000259370.1 | 0.871860377 | 1.914688781 | 0.055532205 |
| F12 | ENST00000562191.1 | ENSG00000261292.1 | -0.921756072 | -2.049884105 | 0.040375742 |
| F12 | ENST00000562995.1 | ENSG00000261253.1 | 0.947952363 | 2.119722908 | 0.03402942 |
| F12 | ENST00000563610.1 | ENSG00000260051.1 | 0.920749966 | 2.073585924 | 0.038117784 |
| F12 | ENST00000563611.1 | ENSG00000261583.1 | 0.84317877 | 1.869139854 | 0.06160336 |
| F12 | ENST00000564809.1 | ENSG00000261471.1 | 0.880608211 | 1.970575889 | 0.048772407 |
| F12 | ENST00000569981.1 | ENSG00000238045.5 | 0.861404984 | 1.922239943 | 0.054575573 |
| F12 | ENST00000570493.2 | ENSG00000261898.2 | 0.924639146 | 2.077237508 | 0.037779644 |
| F12 | ENST00000570974.1 | ENSG00000263300.1 | 0.851967347 | 1.934500996 | 0.05305156 |
| F12 | ENST00000576271.1 | ENSG00000263342.1 | 0.810259995 | 1.8200628 | 0.068749442 |
| F12 | ENST00000577853.1 | ENSG00000264207.1 | 0.904090385 | 2.048335145 | 0.040527172 |
| F12 | ENST00000580311.1 | ENSG00000266803.1 | -0.928044586 | -2.063930859 | 0.039024272 |
| F12 | ENST00000582044.1 | ENSG00000263715.2 | 0.860052075 | 1.877975813 | 0.060384474 |
| F12 | ENST00000585559.1 | ENSG00000267117.1 | 0.853222697 | 1.880717733 | 0.060010326 |
| F12 | ENST00000585810.1 | ENSG00000236172.2 | 0.886333497 | 2.009453902 | 0.044489019 |
| F12 | ENST00000588380.1 | ENSG00000266990.1 | 0.92937493 | 2.071593327 | 0.038303383 |
| F12 | ENST00000588402.1 | ENSG00000267006.1 | -0.891564912 | -1.980346288 | 0.047664631 |
| F12 | ENST00000589395.1 | ENSG00000267143.1 | 0.80501588 | 1.797787744 | 0.07221065 |
| F12 | ENST00000591174.1 | ENSG00000267289.1 | 0.877105904 | 1.92980288 | 0.053631266 |
| F12 | ENST00000592525.1 | ENSG00000267214.1 | 0.815774913 | 1.814350482 | 0.069623784 |
| F12 | ENST00000592816.1 | ENSG00000236172.2 | 0.894419532 | 2.012278705 | 0.044190566 |
| F12 | ENST00000596091.1 | ENSG00000227733.4 | -0.888862147 | -1.988765284 | 0.046727116 |
| F12 | ENST00000596887.1 | ENSG00000237031.3 | -0.864499176 | -1.943894484 | 0.051908177 |
| F12 | ENST00000598092.1 | ENSG00000228065.6 | -0.862147877 | -1.922137774 | 0.054588424 |
| F12 | ENST00000599259.1 | ENSG00000269352.1 | 0.812678887 | 1.824422154 | 0.068088275 |
| F12 | ENST00000600242.1 | ENSG00000269583.1 | 0.8231594 | 1.843687669 | 0.065228671 |
| F12 | ENST00000600716.1 | ENSG00000269487.1 | 0.895242267 | 1.991096371 | 0.046470293 |
| F12 | ENST00000600726.1 | ENSG00000267858.1 | 0.893078444 | 1.975594277 | 0.048200747 |
| F12 | ENST00000602594.1 | ENSG00000269930.1 | -0.809117233 | -1.797662214 | 0.072230553 |
| F12 | ENST00000604142.1 | ENSG00000271308.1 | 0.8629811 | 1.933338061 | 0.053194566 |
| F12 | ENST00000604183.1 | ENSG00000271185.1 | 0.921934671 | 2.058470625 | 0.039544978 |
| F12 | ENST00000606470.1 | ENSG00000271913.1 | 0.835981172 | 1.868374518 | 0.061709886 |
| F12 | ENST00000606743.1 | ENSG00000272221.1 | 0.844209086 | 1.88617826 | 0.059270937 |
| F12 | ENST00000606909.1 | ENSG00000271821.1 | 0.857404238 | 1.930158268 | 0.053587231 |
| F12 | ENST00000607148.1 | ENSG00000272477.1 | -0.845437908 | -1.896771851 | 0.057858056 |
| F12 | ENST00000607476.1 | ENSG00000272540.1 | 0.847094926 | 1.878522687 | 0.060309696 |
| F12 | ENST00000607943.1 | ENSG00000273188.1 | 0.918336734 | 2.049767837 | 0.040387092 |
| F12 | ENST00000608258.1 | ENSG00000229042.2 | -0.868057286 | -1.938038852 | 0.052618483 |
| F12 | ENST00000608259.1 | ENSG00000272627.1 | -0.880305706 | -1.989913951 | 0.046600415 |
| F12 | ENST00000608367.1 | ENSG00000273361.1 | 0.944742165 | 2.142435327 | 0.032158468 |
| F12 | ENST00000608489.1 | ENSG00000272716.1 | 0.87803327 | 1.980385502 | 0.047660228 |
| F12 | ENST00000609113.1 | ENSG00000272827.1 | 0.813729501 | 1.851723846 | 0.064065488 |
| F12 | ENST00000609813.1 | ENSG00000272719.1 | 0.903111692 | 1.98740075 | 0.046878005 |
| F12 | ENST00000609955.1 | ENSG00000273275.1 | -0.844587584 | -1.898974952 | 0.057567769 |
| F12 | NR_003605.1 | ZFAS1 | 0.822133735 | 1.820234126 | 0.068723358 |
| F12 | NR_026802.1 | FAM74A4 | 0.830412891 | 1.851191333 | 0.064142032 |
| F12 | NR_026951.1 | LINC00324 | 0.87269786 | 1.953286253 | 0.050785686 |

| | | | | | |
|---|---|---|---|---|---|
| F12 | NR_027052.1 | THAP7-AS1 | 0.810828282 | 1.812191154 | 0.069956665 |
| F12 | NR_028324.1 | LINC01002 | 0.819440916 | 1.828956245 | 0.067406163 |
| F12 | NR_036480.1 | VPS9D1-AS1 | 0.906983254 | 2.049987454 | 0.040365655 |
| F12 | NR_037169.1 | LOC100507547 | 0.878756243 | 1.967815561 | 0.049089263 |
| F12 | NR_037170.1 | LOC100507547 | 0.865000002 | 1.943374252 | 0.051970956 |
| F12 | NR_038421.1 | LINC01220 | 0.870617779 | 1.954049277 | 0.05069539 |
| F12 | NR_038923.1 | SSSCA1-AS1 | 0.86124741 | 1.917018692 | 0.055235557 |
| F12 | NR_047498.1 | LINC00853 | 0.878653805 | 1.945931076 | 0.051663019 |
| F12 | NR_072981.1 | LINC00957 | 0.877676781 | 1.969742763 | 0.048867859 |
| F12 | NR_072982.1 | LINC00957 | 0.866896769 | 1.942800078 | 0.052040318 |
| F12 | NR_109831.1 | RASSF1-AS1 | 0.803556057 | 1.796321034 | 0.072443475 |
| F12 | NR_109886.1 | RALY-AS1 | 0.820627611 | 1.833581032 | 0.06671621 |
| F12 | NR_110117.1 | LOC101927769 | -0.803413967 | -1.800620894 | 0.071762654 |
| F12 | NR_110245.1 | LOC101929282 | -0.867837947 | -1.967421064 | 0.049134688 |
| F12 | NR_110630.1 | LOC101927478 | 0.921949901 | 2.079205342 | 0.037598481 |
| F12 | NR_110998.1 | FAM74A4 | 0.830412891 | 1.847357481 | 0.064695347 |
| F12 | NR_111951.1 | LINC00869 | 0.832812147 | 1.877037463 | 0.060512959 |
| F12 | NR_111952.1 | LINC00869 | 0.810180824 | 1.828132927 | 0.067529604 |
| F12 | NR_111953.1 | LINC00869 | 0.823257144 | 1.858257375 | 0.063132474 |
| F12 | NR_120371.1 | LINC01585 | 0.820324109 | 1.833192162 | 0.066773999 |
| F12 | NR_125849.1 | LOC101928140 | -0.82152586 | -1.844654666 | 0.065087789 |
| F12 | NR_125957.1 | LOC101928626 | -0.936903695 | -2.061907039 | 0.039216587 |
| F12 | NR_126522.1 | EXOC3-AS1 | 0.890878627 | 1.986325008 | 0.046997248 |
| F12 | NR_134520.1 | LOC727993 | 0.863367838 | 1.931660932 | 0.05340137 |
| F12 | NR_135024.1 | LOC105369747 | 0.901967713 | 2.00989934 | 0.044441844 |
| F12 | NR_135097.1 | LOC105369443 | -0.917063824 | -2.047996802 | 0.040560313 |
| F12 | NR_135626.1 | LOC100505585 | 0.840217172 | 1.849616527 | 0.064368839 |
| F12 | NR_144459.1 | ARSD-AS1 | 0.951855817 | 2.127973608 | 0.033339272 |
| FGF22 | ENST00000411694.1 | ENSG00000225331.1 | 0.93264972 | 2.108360646 | 0.034999806 |
| FGF22 | ENST00000415205.1 | ENSG00000182057.4 | 0.839067915 | 1.881485994 | 0.059905839 |
| FGF22 | ENST00000416595.1 | ENSG00000223623.1 | 0.87788878 | 1.961167683 | 0.049859465 |
| FGF22 | ENST00000417260.1 | ENSG00000231734.4 | -0.80165746 | -1.788091574 | 0.073761235 |
| FGF22 | ENST00000419662.1 | ENSG00000228265.1 | 0.870664763 | 1.93192331 | 0.053368972 |
| FGF22 | ENST00000422763.1 | ENSG00000231131.2 | -0.813792883 | -1.810095086 | 0.070281043 |
| FGF22 | ENST00000425881.1 | ENSG00000239636.1 | 0.874661721 | 1.945055924 | 0.051768248 |
| FGF22 | ENST00000426030.2 | ENSG00000228686.2 | -0.912624446 | -2.060260997 | 0.039373596 |
| FGF22 | ENST00000426475.1 | ENSG00000239467.1 | 0.819920062 | 1.824266162 | 0.068111844 |
| FGF22 | ENST00000431730.1 | ENSG00000237401.2 | 0.941972351 | 2.109879726 | 0.034868717 |
| FGF22 | ENST00000433035.1 | ENSG00000230483.1 | 0.926284991 | 2.060635143 | 0.039337861 |
| FGF22 | ENST00000433051.1 | ENSG00000233193.1 | 0.893906115 | 1.979172339 | 0.047796606 |
| FGF22 | ENST00000434250.1 | ENSG00000234055.1 | 0.882916399 | 1.972396248 | 0.04856439 |
| FGF22 | ENST00000439443.1 | ENSG00000236911.2 | 0.863604751 | 1.920878677 | 0.054747005 |
| FGF22 | ENST00000440595.1 | ENSG00000228265.1 | 0.914648711 | 2.056496177 | 0.039734714 |
| FGF22 | ENST00000441592.2 | ENSG00000224078.8 | 0.946422109 | 2.127642909 | 0.033366702 |
| FGF22 | ENST00000442649.1 | ENSG00000234089.1 | -0.858717042 | -1.9130041 | 0.055747528 |
| FGF22 | ENST00000446562.1 | ENSG00000233896.1 | 0.927969822 | 2.063241611 | 0.039089679 |
| FGF22 | ENST00000451507.1 | ENSG00000229539.1 | 0.959013644 | 2.157277394 | 0.030984057 |
| FGF22 | ENST00000452176.1 | ENSG00000223659.1 | -0.886167944 | -1.986446629 | 0.046983753 |
| FGF22 | ENST00000454957.1 | ENSG00000224899.1 | -0.855488771 | -1.910605874 | 0.056055249 |
| FGF22 | ENST00000457371.1 | ENSG00000237401.2 | 0.944507967 | 2.10866771 | 0.034973274 |
| FGF22 | ENST00000463255.1 | ENSG00000243305.1 | -0.969490146 | -2.170640092 | 0.029958389 |

| | | | | | |
|---|---|---|---|---|---|
| FGF22 | ENST00000488310.1 | ENSG00000240449.1 | 0.869519589 | 1.951798302 | 0.050962156 |
| FGF22 | ENST00000489090.1 | ENSG00000240045.1 | -0.983487894 | -2.206734416 | 0.027332619 |
| FGF22 | ENST00000489690.1 | ENSG00000243944.1 | -0.803671078 | -1.808092323 | 0.070592133 |
| FGF22 | ENST00000503571.1 | ENSG00000249592.1 | 0.893680757 | 2.010053642 | 0.044425512 |
| FGF22 | ENST00000504916.1 | ENSG00000248112.1 | -0.833936805 | -1.845136842 | 0.065017635 |
| FGF22 | ENST00000509453.1 | ENSG00000249145.1 | 0.832343801 | 1.842555397 | 0.065393951 |
| FGF22 | ENST00000513836.1 | ENSG00000251266.1 | -0.811910302 | -1.82656298 | 0.067765504 |
| FGF22 | ENST00000517846.1 | ENSG00000254485.1 | 0.84152 | 1.893093842 | 0.058345391 |
| FGF22 | ENST00000520603.1 | ENSG00000254001.1 | -0.808993357 | -1.812696058 | 0.069878713 |
| FGF22 | ENST00000521359.1 | ENSG00000253140.1 | -0.804685828 | -1.808183178 | 0.070577996 |
| FGF22 | ENST00000521653.1 | ENSG00000253301.1 | 0.833581917 | 1.873040169 | 0.061062838 |
| FGF22 | ENST00000529247.1 | ENSG00000254741.1 | 0.897490904 | 2.018592506 | 0.043529591 |
| FGF22 | ENST00000530435.1 | ENSG00000254630.1 | 0.86911093 | 1.944376036 | 0.051850122 |
| FGF22 | ENST00000532454.1 | ENSG00000255120.1 | 0.870980582 | 1.959560241 | 0.050047212 |
| FGF22 | ENST00000534178.1 | ENSG00000255120.1 | 0.841685591 | 1.871720235 | 0.061245319 |
| FGF22 | ENST00000543403.1 | ENSG00000256684.1 | -0.892434663 | -1.956976317 | 0.050350253 |
| FGF22 | ENST00000545254.1 | ENSG00000256633.1 | 0.863187323 | 1.949743816 | 0.051206661 |
| FGF22 | ENST00000548722.2 | ENSG00000257194.2 | -0.804794686 | -1.782268584 | 0.074705443 |
| FGF22 | ENST00000549806.1 | ENSG00000257252.1 | 0.871146857 | 1.942145993 | 0.052119429 |
| FGF22 | ENST00000554431.1 | ENSG00000258616.1 | -0.887592642 | -1.99367457 | 0.04618763 |
| FGF22 | ENST00000557602.1 | ENSG00000258616.1 | -0.874084967 | -1.942243017 | 0.052107688 |
| FGF22 | ENST00000558475.1 | ENSG00000259604.1 | 0.837566248 | 1.874235443 | 0.060897981 |
| FGF22 | ENST00000558568.1 | ENSG00000272639.1 | 0.888462838 | 1.964564575 | 0.049464655 |
| FGF22 | ENST00000559673.1 | ENSG00000259604.1 | 0.902632947 | 1.980825186 | 0.047610881 |
| FGF22 | ENST00000559960.1 | ENSG00000259354.1 | 0.870109382 | 1.938031684 | 0.052619357 |
| FGF22 | ENST00000562191.1 | ENSG00000261292.1 | -0.921624713 | -2.059006611 | 0.039493605 |
| FGF22 | ENST00000562995.1 | ENSG00000261253.1 | 0.891655905 | 1.974282506 | 0.048349629 |
| FGF22 | ENST00000565955.1 | ENSG00000261055.1 | 0.869922712 | 1.958457016 | 0.05017641 |
| FGF22 | ENST00000571660.1 | ENSG00000262848.1 | 0.873503943 | 1.939321133 | 0.052462247 |
| FGF22 | ENST00000580311.1 | ENSG00000266803.1 | -0.909849486 | -2.026235448 | 0.042740663 |
| FGF22 | ENST00000580622.1 | ENSG00000264634.1 | 0.845628299 | 1.910837547 | 0.056025461 |
| FGF22 | ENST00000582044.1 | ENSG00000263715.2 | 0.877824245 | 1.96515523 | 0.049396274 |
| FGF22 | ENST00000583067.1 | ENSG00000266126.1 | -0.801369902 | -1.791751814 | 0.073172731 |
| FGF22 | ENST00000586348.1 | ENSG00000267198.1 | 0.819468368 | 1.832926736 | 0.066813467 |
| FGF22 | ENST00000586694.1 | ENSG00000267141.1 | 0.898870701 | 2.039052591 | 0.041444779 |
| FGF22 | ENST00000587696.1 | ENSG00000225313.2 | 0.894468874 | 2.007034336 | 0.044746007 |
| FGF22 | ENST00000588380.1 | ENSG00000266990.1 | 0.948940876 | 2.121489919 | 0.033880595 |
| FGF22 | ENST00000589395.1 | ENSG00000267143.1 | 0.87038722 | 1.993331575 | 0.046225151 |
| FGF22 | ENST00000592525.1 | ENSG00000267214.1 | 0.856219423 | 1.908710478 | 0.056299451 |
| FGF22 | ENST00000593139.1 | ENSG00000267042.1 | 0.849661776 | 1.901361301 | 0.057254705 |
| FGF22 | ENST00000593967.1 | ENSG00000232732.5 | 0.820803121 | 1.85112223 | 0.064151971 |
| FGF22 | ENST00000596971.1 | ENSG00000269463.1 | 0.805233871 | 1.793029926 | 0.072968139 |
| FGF22 | ENST00000597256.1 | ENSG00000267986.1 | 0.803671078 | 1.792119185 | 0.073113876 |
| FGF22 | ENST00000600242.1 | ENSG00000269583.1 | 0.811534803 | 1.826296777 | 0.06780557 |
| FGF22 | ENST00000602594.1 | ENSG00000269930.1 | -0.884444661 | -1.966732942 | 0.049214007 |
| FGF22 | ENST00000603948.1 | ENSG00000222041.6 | 0.880260657 | 1.966971024 | 0.049186551 |
| FGF22 | ENST00000604183.1 | ENSG00000271185.1 | 0.818345504 | 1.801891467 | 0.071562483 |
| FGF22 | ENST00000606441.1 | ENSG00000272277.1 | 0.928736543 | 2.096348168 | 0.036051317 |
| FGF22 | ENST00000606743.1 | ENSG00000272221.1 | 0.829446863 | 1.859052612 | 0.063019681 |
| FGF22 | ENST00000606909.1 | ENSG00000271821.1 | 0.851546553 | 1.889555294 | 0.058817461 |
| FGF22 | ENST00000606963.1 | ENSG00000272010.1 | -0.824119535 | -1.836605797 | 0.066268111 |

| | | | | | |
|---|---|---|---|---|---|
| FGF22 | ENST00000608159.1 | ENSG00000273093.1 | -0.941176471 | -2.104425041 | 0.035341387 |
| FGF22 | ENST00000608258.1 | ENSG00000229042.2 | -0.832343801 | -1.845780316 | 0.06492411 |
| FGF22 | ENST00000608367.1 | ENSG00000273361.1 | 0.936034619 | 2.103669737 | 0.035407266 |
| FGF22 | ENST00000610008.1 | ENSG00000272711.1 | 0.823994855 | 1.845976627 | 0.064895599 |
| FGF22 | NR_026774.1 | LINC00239 | 0.841156327 | 1.88075424 | 0.060005358 |
| FGF22 | NR_026802.1 | FAM74A4 | 0.802577847 | 1.808381364 | 0.070547166 |
| FGF22 | NR_026813.1 | LINC00597 | -0.804828757 | -1.805122908 | 0.071055451 |
| FGF22 | NR_026951.1 | LINC00324 | 0.928601455 | 2.092993436 | 0.03634974 |
| FGF22 | NR_034131.1 | LINC00272 | 0.803877569 | 1.791644077 | 0.073189998 |
| FGF22 | NR_037169.1 | LOC100507547 | 0.843950888 | 1.8616352 | 0.062654528 |
| FGF22 | NR_037170.1 | LOC100507547 | 0.848290216 | 1.885143462 | 0.059410472 |
| FGF22 | NR_040047.1 | SDCBP2-AS1 | 0.860686664 | 1.930946397 | 0.053489682 |
| FGF22 | NR_040049.1 | SDCBP2-AS1 | 0.804837144 | 1.81804714 | 0.069056928 |
| FGF22 | NR_047498.1 | LINC00853 | 0.826231349 | 1.847716256 | 0.064643401 |
| FGF22 | NR_073155.1 | C1orf145 | -0.916599389 | -2.026852176 | 0.042677533 |
| FGF22 | NR_108085.1 | OVOL1-AS1 | 0.833834102 | 1.872496651 | 0.061137925 |
| FGF22 | NR_109885.1 | RALY-AS1 | 0.909562741 | 2.0192762 | 0.04345852 |
| FGF22 | NR_109886.1 | RALY-AS1 | 0.853539907 | 1.901773338 | 0.057200793 |
| FGF22 | NR_110998.1 | FAM74A4 | 0.802577847 | 1.779318236 | 0.075187603 |
| FGF22 | NR_125957.1 | LOC101928626 | -0.886167944 | -1.974112883 | 0.048368909 |
| FGF22 | NR_126522.1 | EXOC3-AS1 | 0.860342933 | 1.889743777 | 0.058792236 |
| FGF22 | NR_133941.1 | LOC105377247 | -0.811910302 | -1.828921099 | 0.067411429 |
| FGF22 | NR_134252.1 | LOC105379030 | 0.844669092 | 1.899600387 | 0.057485581 |
| FGF22 | NR_134520.1 | LOC727993 | 0.933627263 | 2.104929349 | 0.035297458 |
| FGF22 | NR_134579.1 | LOC105372179 | 0.865780669 | 1.9254897 | 0.054168123 |
| FGF22 | NR_135024.1 | LOC105369747 | 0.885173164 | 1.986133058 | 0.047018552 |
| FGF22 | NR_135097.1 | LOC105369443 | -0.892434663 | -2.001060143 | 0.045385909 |
| FGF22 | NR_135626.1 | LOC100505585 | 0.973591371 | 2.193997168 | 0.028235613 |
| FOSL1 | ENST00000412348.1 | ENSG00000228959.1 | -0.954984214 | -2.137168231 | 0.032584311 |
| FOSL1 | ENST00000418387.1 | ENSG00000235056.1 | -0.87025497 | -1.945091409 | 0.051763978 |
| FOSL1 | ENST00000422017.1 | ENSG00000232227.1 | -0.92155449 | -2.043676401 | 0.040985526 |
| FOSL1 | ENST00000428391.1 | ENSG00000224691.1 | 0.845332459 | 1.912232646 | 0.055846361 |
| FOSL1 | ENST00000432265.1 | ENSG00000231170.1 | 0.856824489 | 1.92111531 | 0.054717172 |
| FOSL1 | ENST00000433550.1 | ENSG00000232227.1 | -0.919246424 | -2.034957246 | 0.041855175 |
| FOSL1 | ENST00000436515.1 | ENSG00000224521.1 | -0.907358274 | -2.01154349 | 0.044268081 |
| FOSL1 | ENST00000438623.1 | ENSG00000224521.1 | -0.893518726 | -2.006102553 | 0.044845308 |
| FOSL1 | ENST00000448748.1 | ENSG00000231238.1 | -0.869318288 | -1.948112105 | 0.051401551 |
| FOSL1 | ENST00000449154.1 | ENSG00000226969.1 | 0.916837322 | 2.040005824 | 0.041349746 |
| FOSL1 | ENST00000451034.1 | ENSG00000229805.1 | -0.870203903 | -1.926135423 | 0.054087467 |
| FOSL1 | ENST00000456499.1 | ENSG00000237640.1 | 0.81061738 | 1.801390542 | 0.071641346 |
| FOSL1 | ENST00000485338.1 | ENSG00000239641.1 | -0.853806272 | -1.907379851 | 0.056471417 |
| FOSL1 | ENST00000502467.1 | ENSG00000250530.1 | -0.827468946 | -1.863276093 | 0.062423432 |
| FOSL1 | ENST00000503505.1 | ENSG00000248629.1 | -0.869520736 | -1.944281089 | 0.051861564 |
| FOSL1 | ENST00000504765.1 | ENSG00000249638.1 | -0.876478285 | -1.963783451 | 0.04955521 |
| FOSL1 | ENST00000512882.2 | ENSG00000251575.2 | -0.864265652 | -1.941948449 | 0.052143341 |
| FOSL1 | ENST00000515128.1 | ENSG00000248215.1 | -0.822286506 | -1.806710207 | 0.070807475 |
| FOSL1 | ENST00000522390.1 | ENSG00000254262.1 | -0.831141998 | -1.85541251 | 0.063537342 |
| FOSL1 | ENST00000522600.1 | ENSG00000246582.2 | 0.841072239 | 1.881223892 | 0.059941469 |
| FOSL1 | ENST00000524808.1 | ENSG00000254812.1 | 0.879658565 | 1.980710418 | 0.047623758 |
| FOSL1 | ENST00000528887.1 | ENSG00000254501.1 | 0.846711416 | 1.899013928 | 0.057562645 |
| FOSL1 | ENST00000537492.1 | ENSG00000256637.2 | -0.806256407 | -1.804929468 | 0.07108572 |

| | | | | | |
|---|---|---|---|---|---|
| FOSL1 | ENST00000539963.1 | ENSG00000256116.1 | 0.942212683 | 2.12451571 | 0.033627044 |
| FOSL1 | ENST00000543494.1 | ENSG00000256514.1 | 0.859679603 | 1.916825126 | 0.055260152 |
| FOSL1 | ENST00000549683.1 | ENSG00000257953.1 | 0.940627713 | 2.103241902 | 0.035444629 |
| FOSL1 | ENST00000553075.1 | ENSG00000257258.1 | -0.848715557 | -1.897969158 | 0.057700145 |
| FOSL1 | ENST00000561699.1 | ENSG00000259813.1 | 0.884207555 | 1.983873162 | 0.047269981 |
| FOSL1 | ENST00000565667.1 | ENSG00000261253.1 | 0.904599691 | 2.041345234 | 0.041216524 |
| FOSL1 | ENST00000567261.1 | ENSG00000261320.1 | -0.825287488 | -1.840097855 | 0.065753872 |
| FOSL1 | ENST00000568659.1 | ENSG00000260004.1 | -0.849898025 | -1.891112448 | 0.058609335 |
| FOSL1 | ENST00000570974.1 | ENSG00000263300.1 | 0.953026774 | 2.126343568 | 0.033474663 |
| FOSL1 | ENST00000576554.1 | ENSG00000262413.1 | 0.874521463 | 1.922396026 | 0.054555945 |
| FOSL1 | ENST00000577176.1 | ENSG00000262823.1 | 0.884466508 | 1.964378927 | 0.049486165 |
| FOSL1 | ENST00000578443.1 | ENSG00000265204.1 | 0.818267876 | 1.827752441 | 0.067586714 |
| FOSL1 | ENST00000580729.1 | ENSG00000266176.1 | 0.887218297 | 1.975891994 | 0.048167011 |
| FOSL1 | ENST00000583122.1 | ENSG00000264695.1 | -0.859639255 | -1.920271657 | 0.054823594 |
| FOSL1 | ENST00000584139.1 | ENSG00000263388.1 | -0.854152935 | -1.926055208 | 0.054097481 |
| FOSL1 | ENST00000590328.1 | ENSG00000256995.2 | -0.929406839 | -2.078172111 | 0.03769351 |
| FOSL1 | ENST00000597357.1 | ENSG00000268309.1 | 0.806776959 | 1.805774487 | 0.070953572 |
| FOSL1 | ENST00000600007.1 | ENSG00000268655.1 | 0.948702544 | 2.1185616 | 0.034127534 |
| FOSL1 | ENST00000600716.1 | ENSG00000269487.1 | 0.948154709 | 2.098804953 | 0.035834098 |
| FOSL1 | ENST00000606010.1 | ENSG00000272249.1 | -0.876699876 | -1.954472743 | 0.050645335 |
| FOSL1 | ENST00000607135.1 | ENSG00000272112.1 | -0.82577097 | -1.824593084 | 0.068062458 |
| FOSL1 | ENST00000607549.1 | ENSG00000272293.1 | -0.908797826 | -2.040638542 | 0.041286768 |
| FOSL1 | ENST00000608259.1 | ENSG00000272627.1 | -0.84458802 | -1.898649714 | 0.057610547 |
| FOSL1 | ENST00000608465.1 | ENSG00000272758.1 | -0.875695733 | -1.975771902 | 0.048180617 |
| FOSL1 | ENST00000608934.1 | ENSG00000273063.1 | -0.824789446 | -1.856569649 | 0.063372405 |
| FOSL1 | ENST00000609113.1 | ENSG00000272827.1 | 0.802810624 | 1.807930599 | 0.070617303 |
| FOSL1 | ENST00000609813.1 | ENSG00000272719.1 | 0.82240835 | 1.842749695 | 0.065365565 |
| FOSL1 | ENST00000609976.1 | ENSG00000272582.1 | 0.866244017 | 1.929668702 | 0.0536479 |
| FOSL1 | NR_024491.1 | LOC100128573 | -0.9050181 | -2.01426899 | 0.0439813 |
| FOSL1 | NR_073552.1 | LOC101059948 | 0.948702544 | 2.122117402 | 0.03382788 |
| FOSL1 | NR_110702.1 | SEMA3B-AS1 | 0.865746801 | 1.928152151 | 0.053836204 |
| FOSL1 | NR_136569.1 | LINC01660 | -0.925550425 | -2.069590368 | 0.038490721 |
| FOSL1 | NR_144459.1 | ARSD-AS1 | 0.804150001 | 1.803108255 | 0.071371215 |
| GABARAP | ENST00000411694.1 | ENSG00000225331.1 | 0.913716659 | 2.060860709 | 0.039316331 |
| GABARAP | ENST00000412085.1 | ENSG00000233825.1 | 0.918596638 | 2.048826477 | 0.040479086 |
| GABARAP | ENST00000412759.1 | ENSG00000236933.1 | 0.898165426 | 2.011475652 | 0.04427524 |
| GABARAP | ENST00000412772.1 | ENSG00000231507.1 | 0.808295428 | 1.8295637 | 0.067315206 |
| GABARAP | ENST00000414740.2 | ENSG00000229646.2 | 0.868277687 | 1.983598301 | 0.047300639 |
| GABARAP | ENST00000419662.1 | ENSG00000228265.1 | 0.906263356 | 2.029257647 | 0.042432055 |
| GABARAP | ENST00000420315.1 | ENSG00000228072.1 | 0.851469904 | 1.881544898 | 0.059897834 |
| GABARAP | ENST00000420981.2 | ENSG00000230438.5 | 0.911540341 | 2.053658889 | 0.040008718 |
| GABARAP | ENST00000421020.1 | ENSG00000231407.1 | 0.850453072 | 1.88217225 | 0.059812632 |
| GABARAP | ENST00000426475.1 | ENSG00000239467.1 | 0.886000827 | 1.989087534 | 0.046691542 |
| GABARAP | ENST00000426519.1 | ENSG00000234142.1 | 0.867606616 | 1.958625892 | 0.050156615 |
| GABARAP | ENST00000430920.1 | ENSG00000234203.1 | 0.895638198 | 2.001474416 | 0.045341288 |
| GABARAP | ENST00000431727.2 | ENSG00000234938.2 | 0.801512761 | 1.769069166 | 0.076882333 |
| GABARAP | ENST00000433036.1 | ENSG00000228989.1 | 0.901275778 | 2.019965376 | 0.043386979 |
| GABARAP | ENST00000433905.2 | ENSG00000229299.2 | 0.806004685 | 1.822762588 | 0.068339356 |
| GABARAP | ENST00000434627.1 | ENSG00000230074.1 | 0.835603023 | 1.840653219 | 0.065672393 |
| GABARAP | ENST00000438190.1 | ENSG00000227214.2 | 0.849114082 | 1.922739496 | 0.054512774 |
| GABARAP | ENST00000439184.1 | ENSG00000233985.1 | -0.932113987 | -2.06745443 | 0.038691354 |

| | | | | | |
|---|---|---|---|---|---|
| GABARAP | ENST00000441592.2 | ENSG00000224078.8 | 0.88004564 | 1.976982646 | 0.048043592 |
| GABARAP | ENST00000442829.1 | ENSG00000225284.1 | 0.801370044 | 1.798250447 | 0.072137328 |
| GABARAP | ENST00000447343.2 | ENSG00000229299.2 | 0.836946632 | 1.883941009 | 0.059572955 |
| GABARAP | ENST00000450072.1 | ENSG00000228486.5 | 0.814233796 | 1.827249942 | 0.067662198 |
| GABARAP | ENST00000452176.1 | ENSG00000223659.1 | -0.844341384 | -1.904379897 | 0.056860725 |
| GABARAP | ENST00000453051.1 | ENSG00000229407.1 | 0.945822151 | 2.111141694 | 0.034760135 |
| GABARAP | ENST00000454530.1 | ENSG00000226649.1 | -0.810032862 | -1.814825963 | 0.069550659 |
| GABARAP | ENST00000457253.1 | ENSG00000225173.1 | 0.96032546 | 2.119751638 | 0.034026996 |
| GABARAP | ENST00000458154.1 | ENSG00000235578.1 | 0.834641457 | 1.854602183 | 0.063653056 |
| GABARAP | ENST00000458194.1 | ENSG00000226193.1 | 0.893789836 | 2.005219966 | 0.044939537 |
| GABARAP | ENST00000463255.1 | ENSG00000243305.1 | -0.930741562 | -2.083605794 | 0.03719604 |
| GABARAP | ENST00000466431.2 | ENSG00000254485.1 | 0.943313252 | 2.114612855 | 0.034462957 |
| GABARAP | ENST00000489077.1 | ENSG00000244198.1 | 0.860937448 | 1.921498515 | 0.054668889 |
| GABARAP | ENST00000490013.1 | ENSG00000184115.12 | 0.828720218 | 1.857446742 | 0.063247622 |
| GABARAP | ENST00000498358.1 | ENSG00000184115.12 | 0.807190481 | 1.828695998 | 0.067445162 |
| GABARAP | ENST00000498693.1 | ENSG00000244198.1 | 0.856195843 | 1.923150285 | 0.054461179 |
| GABARAP | ENST00000503723.1 | ENSG00000250472.1 | -0.811282133 | -1.78531637 | 0.074210015 |
| GABARAP | ENST00000504916.1 | ENSG00000248112.1 | -0.875546464 | -1.946904333 | 0.051546205 |
| GABARAP | ENST00000506394.1 | ENSG00000251665.1 | 0.892436767 | 1.987280394 | 0.046891333 |
| GABARAP | ENST00000506791.1 | ENSG00000251131.1 | 0.831738866 | 1.855137135 | 0.063576646 |
| GABARAP | ENST00000508188.1 | ENSG00000250999.1 | 0.827914925 | 1.865835678 | 0.062064359 |
| GABARAP | ENST00000509036.1 | ENSG00000251131.1 | 0.929963652 | 2.061728481 | 0.039233593 |
| GABARAP | ENST00000517300.1 | ENSG00000254144.2 | 0.862197366 | 1.938662495 | 0.052542448 |
| GABARAP | ENST00000517846.1 | ENSG00000254485.1 | 0.974475417 | 2.172017193 | 0.029854366 |
| GABARAP | ENST00000520603.1 | ENSG00000254001.1 | -0.81490813 | -1.82860972 | 0.067458096 |
| GABARAP | ENST00000524942.1 | ENSG00000255553.1 | 0.821170405 | 1.82757305 | 0.067613654 |
| GABARAP | ENST00000526154.1 | ENSG00000254511.1 | 0.870780682 | 1.9494589 | 0.051240646 |
| GABARAP | ENST00000527757.1 | ENSG00000255109.1 | -0.835456605 | -1.866897993 | 0.061915834 |
| GABARAP | ENST00000528000.1 | ENSG00000254804.1 | 0.810988304 | 1.826622153 | 0.0677566 |
| GABARAP | ENST00000529247.1 | ENSG00000254741.1 | 0.905034946 | 2.02462547 | 0.042905837 |
| GABARAP | ENST00000532688.1 | ENSG00000255441.1 | 0.83025993 | 1.86419224 | 0.062294713 |
| GABARAP | ENST00000534065.1 | ENSG00000254458.1 | 0.836551278 | 1.847197505 | 0.064718521 |
| GABARAP | ENST00000543072.1 | ENSG00000256092.2 | -0.824706309 | -1.833146988 | 0.066780715 |
| GABARAP | ENST00000543275.1 | ENSG00000256944.1 | 0.885038518 | 1.979984321 | 0.047705291 |
| GABARAP | ENST00000545177.3 | ENSG00000230438.5 | 0.975068776 | 2.195346307 | 0.028138766 |
| GABARAP | ENST00000545254.1 | ENSG00000256633.1 | 0.87824351 | 1.976715189 | 0.048073833 |
| GABARAP | ENST00000548722.2 | ENSG00000257194.2 | -0.829758633 | -1.861385798 | 0.062689715 |
| GABARAP | ENST00000549806.1 | ENSG00000257252.1 | 0.959061963 | 2.166412854 | 0.030279653 |
| GABARAP | ENST00000552525.1 | ENSG00000257286.1 | 0.90437976 | 2.011370191 | 0.04428637 |
| GABARAP | ENST00000558515.1 | ENSG00000259182.1 | 0.85106307 | 1.889278456 | 0.058854527 |
| GABARAP | ENST00000563611.1 | ENSG00000261583.1 | 0.834754406 | 1.854722855 | 0.063635813 |
| GABARAP | ENST00000563806.1 | ENSG00000238045.5 | 0.937554407 | 2.109868138 | 0.034869715 |
| GABARAP | ENST00000574460.1 | ENSG00000263051.1 | 0.809602805 | 1.78570114 | 0.074147661 |
| GABARAP | ENST00000578800.1 | ENSG00000264235.1 | 0.980297313 | 2.214447602 | 0.026798003 |
| GABARAP | ENST00000578936.1 | ENSG00000265547.1 | 0.919865297 | 2.079073845 | 0.037610564 |
| GABARAP | ENST00000579154.1 | ENSG00000265908.1 | -0.905140995 | -2.006837914 | 0.044766924 |
| GABARAP | ENST00000582044.1 | ENSG00000263715.2 | 0.889459874 | 2.01056666 | 0.044371249 |
| GABARAP | ENST00000586694.1 | ENSG00000267141.1 | 0.866193817 | 1.905963662 | 0.05665492 |
| GABARAP | ENST00000588182.2 | ENSG00000267453.2 | 0.929574612 | 2.084998951 | 0.037069396 |
| GABARAP | ENST00000588290.1 | ENSG00000267751.1 | 0.921239496 | 2.059767242 | 0.039420798 |
| GABARAP | ENST00000588380.1 | ENSG00000266990.1 | 0.88419167 | 1.980656168 | 0.047629845 |

| | | | | | |
|---|---|---|---|---|---|
| GABARAP | ENST00000591174.1 | ENSG00000267289.1 | 0.849779906 | 1.907602266 | 0.056442642 |
| GABARAP | ENST00000592518.1 | ENSG00000267786.1 | 0.8016479 | 1.811201008 | 0.070109742 |
| GABARAP | ENST00000592525.1 | ENSG00000267214.1 | 0.826197491 | 1.86348611 | 0.062393905 |
| GABARAP | ENST00000593139.1 | ENSG00000267042.1 | 0.931057098 | 2.103965113 | 0.03538149 |
| GABARAP | ENST00000593642.1 | ENSG00000267858.1 | 0.84804759 | 1.910467773 | 0.056073012 |
| GABARAP | ENST00000594492.1 | ENSG00000250910.3 | 0.810518664 | 1.790804781 | 0.073324628 |
| GABARAP | ENST00000595955.1 | ENSG00000268401.1 | 0.897831913 | 2.012601439 | 0.044156575 |
| GABARAP | ENST00000596887.1 | ENSG00000237031.3 | -0.896921757 | -2.041380897 | 0.041212982 |
| GABARAP | ENST00000597169.1 | ENSG00000269720.1 | 0.838461232 | 1.887849151 | 0.059046205 |
| GABARAP | ENST00000597256.1 | ENSG00000267986.1 | 0.962532528 | 2.148550236 | 0.03167007 |
| GABARAP | ENST00000599352.1 | ENSG00000240401.4 | -0.805110808 | -1.781793459 | 0.074782919 |
| GABARAP | ENST00000600242.1 | ENSG00000269583.1 | 0.891235597 | 1.974521673 | 0.048322456 |
| GABARAP | ENST00000600726.1 | ENSG00000267858.1 | 0.802415838 | 1.777555174 | 0.075476941 |
| GABARAP | ENST00000600889.1 | ENSG00000232675.3 | 0.840749099 | 1.876735695 | 0.060554328 |
| GABARAP | ENST00000601033.1 | ENSG00000268401.1 | 0.967076275 | 2.155512485 | 0.031121753 |
| GABARAP | ENST00000602532.1 | ENSG00000270091.1 | 0.83409756 | 1.830273458 | 0.067209058 |
| GABARAP | ENST00000604142.1 | ENSG00000271308.1 | 0.868134156 | 1.950636269 | 0.051100329 |
| GABARAP | ENST00000604183.1 | ENSG00000271185.1 | 0.809267907 | 1.790703511 | 0.073340886 |
| GABARAP | ENST00000606277.1 | ENSG00000272145.1 | 0.883058165 | 2.000794313 | 0.045414561 |
| GABARAP | ENST00000606963.1 | ENSG00000272010.1 | -0.868752695 | -1.931306148 | 0.053445204 |
| GABARAP | ENST00000607476.1 | ENSG00000272540.1 | 0.89416701 | 1.994566338 | 0.046090198 |
| GABARAP | ENST00000607943.1 | ENSG00000273188.1 | 0.839789388 | 1.860841483 | 0.062766565 |
| GABARAP | ENST00000608367.1 | ENSG00000273361.1 | 0.903493885 | 2.023432566 | 0.043028569 |
| GABARAP | ENST00000608677.1 | ENSG00000273350.1 | 0.964124071 | 2.17906275 | 0.029327007 |
| GABARAP | ENST00000608940.1 | ENSG00000272763.1 | 0.903916472 | 2.010096946 | 0.044420929 |
| GABARAP | NR_003604.2 | ZFAS1 | 0.849410947 | 1.877757144 | 0.060414395 |
| GABARAP | NR_003606.2 | ZFAS1 | 0.843858011 | 1.894064204 | 0.058216488 |
| GABARAP | NR_026802.1 | FAM74A4 | 0.961111729 | 2.169941841 | 0.030011252 |
| GABARAP | NR_026813.1 | LINC00597 | -0.88744955 | -1.99090098 | 0.046491774 |
| GABARAP | NR_026951.1 | LINC00324 | 0.964481936 | 2.130200465 | 0.033155067 |
| GABARAP | NR_027052.1 | THAP7-AS1 | 0.941229809 | 2.094901446 | 0.036179754 |
| GABARAP | NR_027271.1 | CIRBP-AS1 | 0.882599378 | 1.965953003 | 0.04930404 |
| GABARAP | NR_036480.1 | VPS9D1-AS1 | 0.836263169 | 1.861816896 | 0.062628904 |
| GABARAP | NR_036658.1 | ZFAS1 | 0.844702845 | 1.887723893 | 0.059063027 |
| GABARAP | NR_038421.1 | LINC01220 | 0.812931858 | 1.834501822 | 0.066579538 |
| GABARAP | NR_038923.1 | SSSCA1-AS1 | 0.907114484 | 2.048351901 | 0.040525531 |
| GABARAP | NR_040096.1 | LOC643339 | 0.896278327 | 1.981175649 | 0.047571579 |
| GABARAP | NR_046713.1 | NAALADL2-AS2 | -0.846337716 | -1.904838369 | 0.056801085 |
| GABARAP | NR_047116.1 | HIF1A-AS1 | -0.883960485 | -1.97551153 | 0.048210128 |
| GABARAP | NR_103790.1 | LINC00581 | -0.836434418 | -1.871073681 | 0.061334869 |
| GABARAP | NR_105010.1 | LINC01333 | 0.847838088 | 1.9260158 | 0.054102401 |
| GABARAP | NR_108106.1 | LINC01135 | 0.883402079 | 1.975214697 | 0.048243789 |
| GABARAP | NR_109831.1 | RASSF1-AS1 | 0.861211377 | 1.908122848 | 0.05637534 |
| GABARAP | NR_109886.1 | RALY-AS1 | 0.894554703 | 1.995072187 | 0.046035007 |
| GABARAP | NR_110941.1 | MIR762HG | 0.823518904 | 1.846618396 | 0.064802466 |
| GABARAP | NR_110998.1 | FAM74A4 | 0.961111729 | 2.136369813 | 0.032649283 |
| GABARAP | NR_111951.1 | LINC00869 | 0.910452149 | 2.022500879 | 0.043124633 |
| GABARAP | NR_111952.1 | LINC00869 | 0.91834257 | 2.05939421 | 0.03945649 |
| GABARAP | NR_111953.1 | LINC00869 | 0.90786504 | 2.039750471 | 0.041375185 |
| GABARAP | NR_117097.1 | LINC01353 | 0.808295428 | 1.830755892 | 0.067136987 |
| GABARAP | NR_125957.1 | LOC101928626 | -0.844341384 | -1.893266766 | 0.058322402 |

| | | | | | |
|---|---|---|---|---|---|
| GABARAP | NR_126522.1 | EXOC3-AS1 | 0.961590838 | 2.168665878 | 0.03010806 |
| GABARAP | NR_130143.1 | LOC104968399 | 0.964036431 | 2.164141198 | 0.030453515 |
| GABARAP | NR_134520.1 | LOC727993 | 0.813079744 | 1.823028985 | 0.068299001 |
| GABARAP | NR_134579.1 | LOC105372179 | 0.944925002 | 2.141739455 | 0.032214454 |
| GABARAP | NR_135024.1 | LOC105369747 | 0.967969952 | 2.133816089 | 0.032857838 |
| GADD45B | ENST00000399186.2 | ENSG00000214888.2 | 0.95326251 | 2.146269669 | 0.03185147 |
| GADD45B | ENST00000412896.1 | ENSG00000197585.5 | -0.906959311 | -2.038410184 | 0.041508929 |
| GADD45B | ENST00000413564.1 | ENSG00000224500.1 | -0.813285202 | -1.828663372 | 0.067450053 |
| GADD45B | ENST00000413650.1 | ENSG00000230880.2 | 0.954244631 | 2.147158465 | 0.031780668 |
| GADD45B | ENST00000413887.1 | ENSG00000236948.1 | -0.843677112 | -1.879634172 | 0.060157953 |
| GADD45B | ENST00000413991.1 | ENSG00000237614.1 | 0.917531012 | 2.038430526 | 0.041506896 |
| GADD45B | ENST00000414740.2 | ENSG00000229646.2 | 0.810652362 | 1.810692501 | 0.070188464 |
| GADD45B | ENST00000416641.1 | ENSG00000226956.1 | -0.815624031 | -1.836136859 | 0.066337418 |
| GADD45B | ENST00000416657.1 | ENSG00000235858.1 | 0.931637716 | 2.073181348 | 0.038155406 |
| GADD45B | ENST00000418972.1 | ENSG00000225044.1 | -0.857107026 | -1.941968331 | 0.052140934 |
| GADD45B | ENST00000419734.1 | ENSG00000234646.1 | -0.805648131 | -1.806513237 | 0.070838208 |
| GADD45B | ENST00000420315.1 | ENSG00000228072.1 | 0.810928984 | 1.812862927 | 0.069852965 |
| GADD45B | ENST00000420365.1 | ENSG00000225214.1 | 0.865820839 | 1.941353225 | 0.052215448 |
| GADD45B | ENST00000420465.1 | ENSG00000167355.3 | 0.960761765 | 2.129275179 | 0.0332315 |
| GADD45B | ENST00000420572.2 | ENSG00000233358.2 | 0.88964668 | 1.990074234 | 0.046582759 |
| GADD45B | ENST00000420981.2 | ENSG00000230438.5 | 0.811226661 | 1.803091027 | 0.07137392 |
| GADD45B | ENST00000421020.1 | ENSG00000231407.1 | 0.824512801 | 1.842642241 | 0.065381262 |
| GADD45B | ENST00000422038.1 | ENSG00000227935.1 | 0.867708591 | 1.92163906 | 0.05465119 |
| GADD45B | ENST00000423925.1 | ENSG00000223536.1 | -0.832696547 | -1.847511486 | 0.064673045 |
| GADD45B | ENST00000424181.1 | ENSG00000224977.1 | 0.928612405 | 2.077639398 | 0.037742585 |
| GADD45B | ENST00000425371.2 | ENSG00000235872.2 | 0.920275302 | 2.059982878 | 0.039400178 |
| GADD45B | ENST00000426504.1 | ENSG00000234190.1 | -0.894522079 | -1.993018213 | 0.046259453 |
| GADD45B | ENST00000426519.1 | ENSG00000234142.1 | 0.813100558 | 1.822901909 | 0.068318249 |
| GADD45B | ENST00000428160.1 | ENSG00000236897.1 | 0.85078394 | 1.919316716 | 0.054944264 |
| GADD45B | ENST00000429630.1 | ENSG00000232533.1 | 0.876806058 | 1.97200287 | 0.048609279 |
| GADD45B | ENST00000429796.1 | ENSG00000231858.1 | -0.818981123 | -1.828033146 | 0.067544577 |
| GADD45B | ENST00000430842.1 | ENSG00000230433.1 | -0.861564075 | -1.93807053 | 0.052614618 |
| GADD45B | ENST00000431290.1 | ENSG00000183822.2 | 0.83459452 | 1.874437958 | 0.060870086 |
| GADD45B | ENST00000433614.1 | ENSG00000228534.1 | -0.838674556 | -1.868527349 | 0.061688602 |
| GADD45B | ENST00000434627.1 | ENSG00000230074.1 | 0.884558514 | 1.982276469 | 0.047448306 |
| GADD45B | ENST00000435733.1 | ENSG00000226377.1 | 0.826713992 | 1.8749656 | 0.060797456 |
| GADD45B | ENST00000435984.1 | ENSG00000204792.2 | 0.860048435 | 1.927113706 | 0.053965461 |
| GADD45B | ENST00000436582.1 | ENSG00000236525.1 | -0.903723204 | -2.028728733 | 0.042485928 |
| GADD45B | ENST00000437330.1 | ENSG00000229203.1 | 0.943105658 | 2.078468333 | 0.037666245 |
| GADD45B | ENST00000437461.1 | ENSG00000227200.1 | 0.880545307 | 1.972150531 | 0.048592425 |
| GADD45B | ENST00000438222.1 | ENSG00000238034.1 | 0.84320862 | 1.89637381 | 0.057910633 |
| GADD45B | ENST00000442069.1 | ENSG00000225655.1 | -0.919515047 | -2.064252592 | 0.038993773 |
| GADD45B | ENST00000442831.1 | ENSG00000229550.1 | -0.948069917 | -2.115160991 | 0.034416228 |
| GADD45B | ENST00000442850.1 | ENSG00000232600.2 | -0.82085769 | -1.835877407 | 0.06637579 |
| GADD45B | ENST00000443306.1 | ENSG00000233891.3 | 0.954716869 | 2.121820137 | 0.033852845 |
| GADD45B | ENST00000443380.1 | ENSG00000224371.1 | 0.916268166 | 2.052696706 | 0.040102002 |
| GADD45B | ENST00000444665.1 | ENSG00000228852.2 | 0.838579809 | 1.911154402 | 0.055984742 |
| GADD45B | ENST00000447183.2 | ENSG00000271593.1 | 0.844131795 | 1.903357625 | 0.056993896 |
| GADD45B | ENST00000447206.1 | ENSG00000230839.1 | 0.842352243 | 1.884019396 | 0.059562352 |
| GADD45B | ENST00000447514.1 | ENSG00000236753.1 | 0.825057466 | 1.836716167 | 0.066251807 |
| GADD45B | ENST00000447709.1 | ENSG00000237473.1 | 0.921871182 | 2.058355131 | 0.039556055 |

| | | | | | |
|---|---|---|---|---|---|
| GADD45B | ENST00000448365.1 | ENSG00000231114.1 | 0.894092294 | 2.006396961 | 0.044813912 |
| GADD45B | ENST00000448650.1 | ENSG00000223536.1 | -0.825548475 | -1.844361413 | 0.065130487 |
| GADD45B | ENST00000449586.1 | ENSG00000235257.4 | -0.835473299 | -1.876914108 | 0.060529867 |
| GADD45B | ENST00000450063.1 | ENSG00000231210.2 | -0.829156403 | -1.831502325 | 0.067025601 |
| GADD45B | ENST00000450109.1 | ENSG00000225376.1 | 0.959594902 | 2.157722421 | 0.03094942 |
| GADD45B | ENST00000451656.1 | ENSG00000228417.1 | 0.887441224 | 1.979616164 | 0.047746675 |
| GADD45B | ENST00000452002.1 | ENSG00000236501.1 | -0.879585647 | -1.972509084 | 0.04855152 |
| GADD45B | ENST00000453579.1 | ENSG00000232529.1 | 0.849013245 | 1.884723081 | 0.059467234 |
| GADD45B | ENST00000454489.1 | ENSG00000231403.1 | 0.847066017 | 1.887032736 | 0.059155923 |
| GADD45B | ENST00000454526.1 | ENSG00000234136.1 | 0.829318434 | 1.861438173 | 0.062682324 |
| GADD45B | ENST00000454530.1 | ENSG00000226649.1 | -0.87058766 | -1.945739303 | 0.051686063 |
| GADD45B | ENST00000454709.1 | ENSG00000237280.1 | -0.812163343 | -1.798760637 | 0.072056552 |
| GADD45B | ENST00000457115.1 | ENSG00000227245.1 | 0.899498719 | 2.015997815 | 0.043800204 |
| GADD45B | ENST00000457848.1 | ENSG00000226412.1 | 0.965362614 | 2.148378098 | 0.031683731 |
| GADD45B | ENST00000458194.1 | ENSG00000226193.1 | 0.818957977 | 1.832089817 | 0.06693804 |
| GADD45B | ENST00000458364.1 | ENSG00000225655.1 | -0.889148717 | -1.99571793 | 0.045964634 |
| GADD45B | ENST00000460993.1 | ENSG00000241231.1 | 0.850400776 | 1.909683795 | 0.056173939 |
| GADD45B | ENST00000472596.1 | ENSG00000239774.1 | 0.932322686 | 2.095580525 | 0.036119418 |
| GADD45B | ENST00000476099.1 | ENSG00000244158.1 | -0.927065047 | -2.066897689 | 0.038743796 |
| GADD45B | ENST00000476892.1 | ENSG00000241345.1 | 0.908186439 | 2.049827517 | 0.040381265 |
| GADD45B | ENST00000482142.1 | ENSG00000243276.1 | 0.80184606 | 1.791663451 | 0.073186893 |
| GADD45B | ENST00000493123.1 | ENSG00000242428.1 | 0.943019889 | 2.124980152 | 0.033588269 |
| GADD45B | ENST00000500498.2 | ENSG00000245311.2 | -0.81018171 | -1.811818198 | 0.070014292 |
| GADD45B | ENST00000502421.1 | ENSG00000250284.1 | -0.838716474 | -1.900772226 | 0.057331853 |
| GADD45B | ENST00000504755.1 | ENSG00000250252.1 | 0.803222927 | 1.763724367 | 0.077778397 |
| GADD45B | ENST00000505196.1 | ENSG00000248131.1 | 0.933127027 | 2.078398704 | 0.037672652 |
| GADD45B | ENST00000505556.1 | ENSG00000249409.1 | 0.824693399 | 1.83275211 | 0.066839444 |
| GADD45B | ENST00000506100.1 | ENSG00000249409.1 | 0.819878916 | 1.834465704 | 0.066584894 |
| GADD45B | ENST00000506305.1 | ENSG00000249994.1 | -0.800532655 | -1.796581875 | 0.072402024 |
| GADD45B | ENST00000507558.1 | ENSG00000248445.1 | 0.880647122 | 1.937493321 | 0.052685069 |
| GADD45B | ENST00000508188.1 | ENSG00000250999.1 | 0.925125705 | 2.114398576 | 0.034481239 |
| GADD45B | ENST00000508241.1 | ENSG00000248518.1 | 0.911136318 | 2.039019055 | 0.041448126 |
| GADD45B | ENST00000515077.1 | ENSG00000251206.1 | 0.940193468 | 2.093726192 | 0.036284378 |
| GADD45B | ENST00000515750.1 | ENSG00000249061.1 | -0.942815834 | -2.105097145 | 0.035282853 |
| GADD45B | ENST00000517300.1 | ENSG00000254144.2 | 0.923056546 | 2.078363382 | 0.037675903 |
| GADD45B | ENST00000518620.1 | ENSG00000253892.1 | -0.80585162 | -1.808948666 | 0.070458979 |
| GADD45B | ENST00000519038.2 | ENSG00000254054.2 | -0.850019676 | -1.902746712 | 0.057073604 |
| GADD45B | ENST00000519189.1 | ENSG00000254344.1 | -0.860751579 | -1.906123768 | 0.05663415 |
| GADD45B | ENST00000519451.1 | ENSG00000253363.1 | 0.962683866 | 2.159858421 | 0.030783631 |
| GADD45B | ENST00000519852.1 | ENSG00000253716.1 | 0.918145822 | 2.035013635 | 0.041849501 |
| GADD45B | ENST00000521207.1 | ENSG00000253716.1 | 0.875388102 | 1.96455105 | 0.049466222 |
| GADD45B | ENST00000524073.1 | ENSG00000253774.1 | 0.97714766 | 2.200708025 | 0.0277567 |
| GADD45B | ENST00000524335.1 | ENSG00000253716.1 | 0.937969244 | 2.089122291 | 0.036696716 |
| GADD45B | ENST00000526154.1 | ENSG00000254511.1 | 0.823478915 | 1.833873018 | 0.066672846 |
| GADD45B | ENST00000526611.1 | ENSG00000246982.2 | 0.839206653 | 1.866143245 | 0.062021327 |
| GADD45B | ENST00000526694.1 | ENSG00000231999.2 | 0.924258547 | 2.048767479 | 0.040484857 |
| GADD45B | ENST00000526935.1 | ENSG00000255372.1 | 0.84008313 | 1.894705313 | 0.058131454 |
| GADD45B | ENST00000528869.1 | ENSG00000255443.1 | 0.849436189 | 1.899257599 | 0.057530615 |
| GADD45B | ENST00000531071.1 | ENSG00000255248.2 | -0.826941056 | -1.863945729 | 0.062329326 |
| GADD45B | ENST00000531627.1 | ENSG00000254584.1 | 0.937905806 | 2.109019549 | 0.034942894 |
| GADD45B | ENST00000532688.1 | ENSG00000255441.1 | 0.897354863 | 2.029372319 | 0.042420382 |

| | | | | | |
|---|---|---|---|---|---|
| GADD45B | ENST00000536141.1 | ENSG00000256969.1 | 0.938302115 | 2.107638188 | 0.035062298 |
| GADD45B | ENST00000537269.1 | ENSG00000257084.1 | 0.954666074 | 2.142975364 | 0.032115077 |
| GADD45B | ENST00000537921.1 | ENSG00000255966.1 | 0.898212609 | 2.015243262 | 0.043879167 |
| GADD45B | ENST00000544089.1 | ENSG00000256273.1 | 0.898847012 | 1.994717101 | 0.046073743 |
| GADD45B | ENST00000545642.1 | ENSG00000256342.1 | 0.906059786 | 2.00942096 | 0.044492509 |
| GADD45B | ENST00000547175.1 | ENSG00000257395.1 | -0.904554411 | -2.024945946 | 0.042872915 |
| GADD45B | ENST00000547750.1 | ENSG00000257886.1 | 0.968614044 | 2.184919857 | 0.028894723 |
| GADD45B | ENST00000549878.1 | ENSG00000257284.1 | 0.898329841 | 2.013808389 | 0.044029655 |
| GADD45B | ENST00000554430.1 | ENSG00000258646.1 | -0.911784348 | -2.062399039 | 0.039169761 |
| GADD45B | ENST00000557412.1 | ENSG00000257621.3 | -0.841144021 | -1.890030424 | 0.058753891 |
| GADD45B | ENST00000558515.1 | ENSG00000259182.1 | 0.857535447 | 1.912764869 | 0.05577816 |
| GADD45B | ENST00000558875.1 | ENSG00000259737.2 | 0.811136667 | 1.831416734 | 0.067038365 |
| GADD45B | ENST00000559569.1 | ENSG00000259760.1 | -0.975649117 | -2.182456755 | 0.02907584 |
| GADD45B | ENST00000560522.1 | ENSG00000259661.1 | 0.968231149 | 2.161342941 | 0.030668858 |
| GADD45B | ENST00000561529.1 | ENSG00000260886.1 | 0.868648104 | 1.928808696 | 0.053754616 |
| GADD45B | ENST00000561567.1 | ENSG00000260177.1 | 0.824326049 | 1.833696913 | 0.066698997 |
| GADD45B | ENST00000562582.1 | ENSG00000259779.1 | 0.814986997 | 1.831139717 | 0.067079692 |
| GADD45B | ENST00000563044.1 | ENSG00000260978.1 | 0.902627967 | 2.016199839 | 0.043779083 |
| GADD45B | ENST00000563601.1 | ENSG00000260589.1 | -0.85662311 | -1.891452972 | 0.058563903 |
| GADD45B | ENST00000565829.1 | ENSG00000260148.1 | 0.843761345 | 1.886923448 | 0.059170623 |
| GADD45B | ENST00000569742.1 | ENSG00000260787.1 | 0.856229005 | 1.913113627 | 0.055733508 |
| GADD45B | ENST00000570512.1 | ENSG00000262768.1 | 0.811711105 | 1.819526307 | 0.068831173 |
| GADD45B | ENST00000571815.1 | ENSG00000262810.1 | 0.949276447 | 2.131507417 | 0.033047362 |
| GADD45B | ENST00000575139.1 | ENSG00000263072.1 | 0.809133567 | 1.786697005 | 0.073986473 |
| GADD45B | ENST00000577064.1 | ENSG00000262823.1 | 0.832959922 | 1.856031718 | 0.063449037 |
| GADD45B | ENST00000577678.1 | ENSG00000265415.1 | 0.877709566 | 1.949964374 | 0.051180365 |
| GADD45B | ENST00000578265.1 | ENSG00000214719.7 | 0.840623633 | 1.87268088 | 0.061112465 |
| GADD45B | ENST00000578757.1 | ENSG00000175061.13 | 0.928883752 | 2.076726058 | 0.03782685 |
| GADD45B | ENST00000581905.1 | ENSG00000264235.1 | 0.83280816 | 1.867956952 | 0.061768071 |
| GADD45B | ENST00000581940.1 | ENSG00000265484.1 | 0.850853644 | 1.92294506 | 0.05448695 |
| GADD45B | ENST00000586010.1 | ENSG00000267606.1 | 0.89580046 | 2.017146898 | 0.043680186 |
| GADD45B | ENST00000586297.1 | ENSG00000267633.1 | 0.81543946 | 1.814094191 | 0.069663226 |
| GADD45B | ENST00000586399.1 | ENSG00000228430.4 | 0.830322406 | 1.899120782 | 0.057548597 |
| GADD45B | ENST00000588835.1 | ENSG00000267476.1 | 0.845697205 | 1.888234706 | 0.058994449 |
| GADD45B | ENST00000589233.1 | ENSG00000231616.4 | 0.895016575 | 2.011998157 | 0.044220131 |
| GADD45B | ENST00000589281.1 | ENSG00000267707.1 | -0.825849234 | -1.842155214 | 0.065452449 |
| GADD45B | ENST00000589817.1 | ENSG00000231616.4 | 0.914476452 | 2.045808002 | 0.040775264 |
| GADD45B | ENST00000590368.1 | ENSG00000231616.4 | 0.849009332 | 1.898119899 | 0.057680289 |
| GADD45B | ENST00000590813.1 | ENSG00000231616.4 | 0.822855092 | 1.84457018 | 0.065100088 |
| GADD45B | ENST00000591103.1 | ENSG00000272895.1 | 0.977419142 | 2.208294552 | 0.027223747 |
| GADD45B | ENST00000591137.1 | ENSG00000267405.1 | 0.883009544 | 1.978299939 | 0.04789488 |
| GADD45B | ENST00000592622.1 | ENSG00000267546.2 | -0.807333246 | -1.821632195 | 0.068510812 |
| GADD45B | ENST00000593632.1 | ENSG00000180279.5 | 0.962003768 | 2.1742186 | 0.029688722 |
| GADD45B | ENST00000594492.1 | ENSG00000250910.3 | 0.832743127 | 1.854637578 | 0.063647998 |
| GADD45B | ENST00000596135.1 | ENSG00000269843.1 | 0.929687724 | 2.100622529 | 0.035674115 |
| GADD45B | ENST00000597309.1 | ENSG00000232098.2 | -0.864232782 | -1.917389884 | 0.055188418 |
| GADD45B | ENST00000600234.1 | ENSG00000268078.1 | 0.812756451 | 1.817148944 | 0.06919431 |
| GADD45B | ENST00000600534.1 | ENSG00000267858.1 | 0.84996599 | 1.885119058 | 0.059413766 |
| GADD45B | ENST00000600889.1 | ENSG00000232675.3 | 0.932451244 | 2.081127342 | 0.037422252 |
| GADD45B | ENST00000600959.1 | ENSG00000269303.1 | 0.80084288 | 1.788273261 | 0.073731932 |
| GADD45B | ENST00000602485.1 | ENSG00000270163.1 | -0.924975264 | -2.057463229 | 0.039641688 |

| | | | | | |
|---|---|---|---|---|---|
| GADD45B | ENST00000602532.1 | ENSG00000270091.1 | 0.81000381 | 1.802322581 | 0.071494668 |
| GADD45B | ENST00000603612.1 | ENSG00000270996.1 | 0.819788592 | 1.825408831 | 0.067939358 |
| GADD45B | ENST00000606068.1 | ENSG00000272342.1 | 0.853408672 | 1.894806505 | 0.058118041 |
| GADD45B | ENST00000606841.1 | ENSG00000272411.1 | 0.986437295 | 2.247296531 | 0.024621082 |
| GADD45B | ENST00000606938.1 | ENSG00000272198.1 | -0.934165959 | -2.090472033 | 0.036575418 |
| GADD45B | ENST00000607014.1 | ENSG00000272345.1 | -0.877408328 | -1.937470509 | 0.052687855 |
| GADD45B | ENST00000607201.1 | ENSG00000272024.1 | -0.90431902 | -2.02613388 | 0.042751067 |
| GADD45B | ENST00000607222.1 | ENSG00000272106.1 | 0.849971391 | 1.888927909 | 0.058901489 |
| GADD45B | ENST00000608173.1 | ENSG00000197099.4 | 0.879354513 | 1.966310175 | 0.049262792 |
| GADD45B | ENST00000608952.1 | ENSG00000272689.1 | -0.821798357 | -1.817690683 | 0.069111422 |
| GADD45B | ENST00000609067.1 | ENSG00000272849.1 | 0.834708756 | 1.860225792 | 0.062853587 |
| GADD45B | ENST00000609610.1 | ENSG00000232675.3 | 0.830092169 | 1.871639168 | 0.061256541 |
| GADD45B | ENST00000609953.1 | ENSG00000272825.1 | 0.850187229 | 1.871389448 | 0.06129112 |
| GADD45B | ENST00000610145.1 | ENSG00000273175.1 | 0.839624094 | 1.874138771 | 0.060911301 |
| GADD45B | ENST00000610185.1 | ENSG00000273355.1 | -0.978447941 | -2.178763769 | 0.029349222 |
| GADD45B | NR_002765.2 | ASAP1-IT1 | 0.811450012 | 1.814035204 | 0.069672306 |
| GADD45B | NR_022011.1 | PWARSN | 0.845174244 | 1.87677455 | 0.060549 |
| GADD45B | NR_027334.2 | MZF1-AS1 | 0.837403586 | 1.858978264 | 0.063030219 |
| GADD45B | NR_045637.1 | BOLA3-AS1 | 0.896466279 | 1.990182327 | 0.046570854 |
| GADD45B | NR_046454.1 | LINC00907 | -0.809722035 | -1.799702936 | 0.071907557 |
| GADD45B | NR_046556.1 | RBMS3-AS1 | 0.809120558 | 1.818467879 | 0.068992652 |
| GADD45B | NR_046578.1 | CACNA1C-AS4 | 0.878829454 | 1.970650332 | 0.048763885 |
| GADD45B | NR_046839.1 | AGBL4-IT1 | -0.87542208 | -1.962734103 | 0.049677078 |
| GADD45B | NR_047040.1 | LINC00424 | 0.901512172 | 2.019710808 | 0.043413393 |
| GADD45B | NR_103790.1 | LINC00581 | -0.874913022 | -1.950667854 | 0.05109657 |
| GADD45B | NR_104158.1 | NRG1-IT1 | 0.886211341 | 1.976413911 | 0.048107917 |
| GADD45B | NR_104620.1 | LINC01672 | 0.809158647 | 1.806292395 | 0.070872679 |
| GADD45B | NR_109975.1 | ARNTL2-AS1 | -0.833755087 | -1.84475264 | 0.06507353 |
| GADD45B | NR_110053.1 | LOC101927464 | 0.906059786 | 2.047367864 | 0.040621979 |
| GADD45B | NR_120330.1 | LOC101928227 | -0.864746651 | -1.971026202 | 0.048720879 |
| GADD45B | NR_120527.1 | LOC100506675 | 0.938623281 | 2.115254037 | 0.034408302 |
| GADD45B | NR_121188.1 | PGM5P3-AS1 | -0.841232416 | -1.90042294 | 0.057377639 |
| GADD45B | NR_121189.1 | PGM5P3-AS1 | -0.849572729 | -1.877929311 | 0.060390836 |
| GADD45B | NR_121661.1 | ZBTB20-AS5 | -0.905564227 | -2.04171807 | 0.041179505 |
| GADD45B | NR_125407.1 | LOC102724604 | 0.869430334 | 1.949102527 | 0.051283182 |
| GADD45B | NR_125925.1 | LOC101929448 | 0.86756036 | 1.920279007 | 0.054822667 |
| GADD45B | NR_130144.1 | LOC104968399 | 0.83280816 | 1.839937769 | 0.065777374 |
| GADD45B | NR_135032.1 | LOC105369635 | 0.954666074 | 2.175126601 | 0.029620631 |
| GADD45B | NR_135644.1 | LOC105371506 | -0.853815757 | -1.898323274 | 0.05765351 |
| GADD45B | NR_135820.1 | LOC102723727 | 0.917724542 | 2.046540738 | 0.040703199 |
| GMNN | ENST00000412772.1 | ENSG00000231507.1 | 0.913696058 | 2.029019753 | 0.042456278 |
| GMNN | ENST00000413645.1 | ENSG00000228798.1 | 0.870017065 | 1.933942705 | 0.053120173 |
| GMNN | ENST00000414740.2 | ENSG00000229646.2 | 0.829708004 | 1.856392263 | 0.063397667 |
| GMNN | ENST00000420498.1 | ENSG00000224985.1 | 0.880514638 | 1.969094054 | 0.048942292 |
| GMNN | ENST00000420572.2 | ENSG00000233358.2 | 0.800065305 | 1.79652946 | 0.072410352 |
| GMNN | ENST00000421617.1 | ENSG00000237342.1 | 0.855316567 | 1.917118207 | 0.055222916 |
| GMNN | ENST00000421866.1 | ENSG00000233875.1 | -0.947143018 | -2.113724335 | 0.034538819 |
| GMNN | ENST00000427691.1 | ENSG00000228340.1 | -0.855295912 | -1.910430647 | 0.056077788 |
| GMNN | ENST00000429608.1 | ENSG00000237480.1 | 0.960049447 | 2.131946892 | 0.033011213 |
| GMNN | ENST00000433036.1 | ENSG00000228989.1 | 0.890595838 | 1.961310359 | 0.04984283 |
| GMNN | ENST00000437330.1 | ENSG00000229203.1 | 0.867056665 | 1.962470893 | 0.049707686 |

| | | | | | |
|---|---|---|---|---|---|
| GMNN | ENST00000442850.1 | ENSG00000232600.2 | -0.831069833 | -1.881924813 | 0.059846225 |
| GMNN | ENST00000443380.1 | ENSG00000224371.1 | 0.873502215 | 1.935972462 | 0.052871074 |
| GMNN | ENST00000445260.2 | ENSG00000231429.2 | 0.80388315 | 1.813063477 | 0.069822031 |
| GMNN | ENST00000446560.1 | ENSG00000229258.1 | 0.947277331 | 2.092003178 | 0.036438231 |
| GMNN | ENST00000448365.1 | ENSG00000231114.1 | 0.833093143 | 1.860210327 | 0.062855775 |
| GMNN | ENST00000448942.1 | ENSG00000237499.2 | 0.831129794 | 1.862181009 | 0.06257758 |
| GMNN | ENST00000450109.1 | ENSG00000225376.1 | 0.801818062 | 1.765212239 | 0.077528102 |
| GMNN | ENST00000455010.1 | ENSG00000233079.1 | 0.956260942 | 2.144233675 | 0.032014168 |
| GMNN | ENST00000458443.1 | ENSG00000238232.1 | 0.858487149 | 1.916488945 | 0.055302889 |
| GMNN | ENST00000466431.2 | ENSG00000254485.1 | 0.801658285 | 1.781085678 | 0.074898455 |
| GMNN | ENST00000490013.1 | ENSG00000184115.12 | 0.825851455 | 1.85973024 | 0.062923701 |
| GMNN | ENST00000498358.1 | ENSG00000184115.12 | 0.926514543 | 2.094498041 | 0.036215637 |
| GMNN | ENST00000502300.1 | ENSG00000249451.1 | 0.906697041 | 2.037042533 | 0.04164578 |
| GMNN | ENST00000503034.1 | ENSG00000248936.1 | 0.838859195 | 1.866181067 | 0.062016037 |
| GMNN | ENST00000508188.1 | ENSG00000250999.1 | 0.832571673 | 1.874942332 | 0.060800657 |
| GMNN | ENST00000508199.1 | ENSG00000247810.2 | 0.940589332 | 2.096577953 | 0.036030953 |
| GMNN | ENST00000508241.1 | ENSG00000248518.1 | 0.837508695 | 1.873977079 | 0.060933584 |
| GMNN | ENST00000512300.1 | ENSG00000248362.1 | 0.943865878 | 2.126197628 | 0.033486808 |
| GMNN | ENST00000515136.1 | ENSG00000251274.1 | -0.886045833 | -1.975457938 | 0.048216203 |
| GMNN | ENST00000519368.1 | ENSG00000253215.1 | 0.834302964 | 1.853982001 | 0.063741735 |
| GMNN | ENST00000519451.1 | ENSG00000253363.1 | 0.833169288 | 1.893894039 | 0.058239076 |
| GMNN | ENST00000521207.1 | ENSG00000253716.1 | 0.841697046 | 1.926291894 | 0.054067937 |
| GMNN | ENST00000521884.1 | ENSG00000253355.1 | -0.814404606 | -1.831989554 | 0.066952977 |
| GMNN | ENST00000522524.1 | ENSG00000253342.1 | -0.8363841 | -1.856900149 | 0.063325361 |
| GMNN | ENST00000524942.1 | ENSG00000255553.1 | 0.892956599 | 2.013425165 | 0.04406992 |
| GMNN | ENST00000528000.1 | ENSG00000254804.1 | 0.833569689 | 1.84354611 | 0.065249316 |
| GMNN | ENST00000532249.1 | ENSG00000234899.5 | 0.900384402 | 2.021785566 | 0.04319851 |
| GMNN | ENST00000532947.1 | ENSG00000255322.1 | 0.897409751 | 1.989670873 | 0.046627203 |
| GMNN | ENST00000535746.1 | ENSG00000256101.1 | 0.902730048 | 1.993461409 | 0.046210945 |
| GMNN | ENST00000547750.1 | ENSG00000257886.1 | 0.819927214 | 1.833699761 | 0.066698574 |
| GMNN | ENST00000550886.1 | ENSG00000257696.1 | 0.882981444 | 1.953968095 | 0.05070499 |
| GMNN | ENST00000554049.1 | ENSG00000258763.1 | 0.877054676 | 1.951575281 | 0.05098865 |
| GMNN | ENST00000554197.1 | ENSG00000197176.3 | 0.923101136 | 2.099512437 | 0.035771753 |
| GMNN | ENST00000557412.1 | ENSG00000257621.3 | -0.865615071 | -1.932090013 | 0.053348397 |
| GMNN | ENST00000558515.1 | ENSG00000259182.1 | 0.910073173 | 2.027481673 | 0.042613177 |
| GMNN | ENST00000558875.1 | ENSG00000259737.2 | 0.941002429 | 2.105417193 | 0.035255008 |
| GMNN | ENST00000561215.1 | ENSG00000259611.1 | 0.876109061 | 1.949717333 | 0.051209819 |
| GMNN | ENST00000561529.1 | ENSG00000260886.1 | 0.882840142 | 1.954837027 | 0.050602309 |
| GMNN | ENST00000563806.1 | ENSG00000238045.5 | 0.806348936 | 1.812349858 | 0.069932155 |
| GMNN | ENST00000565359.1 | ENSG00000260601.1 | 0.885426247 | 1.977872161 | 0.04794313 |
| GMNN | ENST00000569025.1 | ENSG00000246379.2 | -0.83460553 | -1.876702985 | 0.060558813 |
| GMNN | ENST00000569459.1 | ENSG00000261346.1 | 0.927035349 | 2.104681635 | 0.03531903 |
| GMNN | ENST00000569742.1 | ENSG00000260787.1 | 0.86251683 | 1.914444735 | 0.055563354 |
| GMNN | ENST00000578936.1 | ENSG00000265547.1 | 0.825509646 | 1.832580613 | 0.066864963 |
| GMNN | ENST00000583138.1 | ENSG00000263393.1 | 0.925719431 | 2.110148025 | 0.034845608 |
| GMNN | ENST00000583916.1 | ENSG00000264196.1 | 0.836385107 | 1.891762355 | 0.058522651 |
| GMNN | ENST00000586503.1 | ENSG00000267205.1 | -0.843620706 | -1.884870294 | 0.059447352 |
| GMNN | ENST00000588290.1 | ENSG00000267751.1 | 0.877534671 | 1.967653154 | 0.049107959 |
| GMNN | ENST00000588908.1 | ENSG00000267751.1 | 0.943321597 | 2.140113729 | 0.032345577 |
| GMNN | ENST00000589457.1 | ENSG00000267751.1 | 0.92430066 | 2.018626861 | 0.043526017 |
| GMNN | ENST00000589817.1 | ENSG00000231616.4 | 0.83873333 | 1.884897672 | 0.059443654 |

| | | | | | |
|---|---|---|---|---|---|
| GMNN | ENST00000590292.1 | ENSG00000267751.1 | 0.97103364 | 2.16460448 | 0.030417988 |
| GMNN | ENST00000590368.1 | ENSG00000231616.4 | 0.900809986 | 2.006595934 | 0.044792705 |
| GMNN | ENST00000590995.1 | ENSG00000267198.1 | 0.802680382 | 1.813029336 | 0.069827297 |
| GMNN | ENST00000591217.1 | ENSG00000231616.4 | 0.863078663 | 1.935533417 | 0.052924872 |
| GMNN | ENST00000591836.1 | ENSG00000267776.1 | 0.915945064 | 2.053524791 | 0.040021708 |
| GMNN | ENST00000592622.1 | ENSG00000267546.2 | -0.884159552 | -1.979553896 | 0.047753678 |
| GMNN | ENST00000593269.1 | ENSG00000236172.2 | 0.959933005 | 2.171117513 | 0.029922291 |
| GMNN | ENST00000593642.1 | ENSG00000267858.1 | 0.806332735 | 1.785179883 | 0.074232144 |
| GMNN | ENST00000594492.1 | ENSG00000250910.3 | 0.876989017 | 1.972022542 | 0.048607033 |
| GMNN | ENST00000595955.1 | ENSG00000268401.1 | 0.848414124 | 1.871503462 | 0.06127533 |
| GMNN | ENST00000600071.1 | ENSG00000269199.1 | 0.905552635 | 2.032686627 | 0.042084193 |
| GMNN | ENST00000600512.1 | ENSG00000269752.1 | 0.815156277 | 1.809493022 | 0.070374443 |
| GMNN | ENST00000602614.1 | ENSG00000269957.1 | -0.979519438 | -2.194172236 | 0.02822303 |
| GMNN | ENST00000602773.1 | ENSG00000270160.1 | -0.863362529 | -1.894665028 | 0.058136794 |
| GMNN | ENST00000606068.1 | ENSG00000272342.1 | 0.866829145 | 1.919623705 | 0.054905448 |
| GMNN | ENST00000608264.1 | ENSG00000273473.1 | 0.862294234 | 1.92791518 | 0.053865677 |
| GMNN | ENST00000608940.1 | ENSG00000272763.1 | 0.891972869 | 1.968686877 | 0.048989059 |
| GMNN | ENST00000609146.1 | ENSG00000272851.1 | -0.951868978 | -2.089032087 | 0.036704835 |
| GMNN | ENST00000609953.1 | ENSG00000272825.1 | 0.836771441 | 1.898526278 | 0.05762679 |
| GMNN | NR_002765.2 | ASAP1-IT1 | 0.817964031 | 1.843611446 | 0.065239787 |
| GMNN | NR_027401.2 | FAM223A | 0.894938085 | 1.969524028 | 0.048892946 |
| GMNN | NR_028325.1 | LOC100132062 | 0.810400367 | 1.793752602 | 0.072852665 |
| GMNN | NR_040096.1 | LOC643339 | 0.873395409 | 1.926221548 | 0.054076716 |
| GMNN | NR_046370.1 | LOC100131626 | -0.878845346 | -1.98237254 | 0.047437561 |
| GMNN | NR_046454.1 | LINC00907 | -0.846280781 | -1.908401079 | 0.056339397 |
| GMNN | NR_103851.1 | TAT-AS1 | 0.833827323 | 1.859219104 | 0.062996088 |
| GMNN | NR_103857.1 | SP2-AS1 | -0.900883524 | -2.017613261 | 0.043631555 |
| GMNN | NR_110008.1 | ADNP-AS1 | 0.8353072 | 1.863888704 | 0.062337335 |
| GMNN | NR_110009.1 | ADNP-AS1 | 0.8353072 | 1.855754887 | 0.063488504 |
| GMNN | NR_110318.1 | MACROD2-AS1 | 0.923222663 | 2.04782924 | 0.040576734 |
| GMNN | NR_110635.1 | LINC00687 | 0.953904411 | 2.114292894 | 0.034490259 |
| GMNN | NR_117097.1 | LINC01353 | 0.913696058 | 2.051751955 | 0.040193775 |
| GMNN | NR_117098.1 | LINC01353 | 0.899548984 | 2.039282392 | 0.041421852 |
| GMNN | NR_121661.1 | ZBTB20-AS5 | -0.812737947 | -1.823286839 | 0.068259959 |
| GMNN | NR_135820.1 | LOC102723727 | 0.903650516 | 2.021397268 | 0.043238657 |
| GMNN | NR_138041.1 | LINC00384 | 0.827673673 | 1.838168128 | 0.066037635 |
| GNGT1 | ENST00000340585.6 | ENSG00000249429.1 | 0.921590971 | 2.059535244 | 0.039442992 |
| GNGT1 | ENST00000362684.1 | ENSG00000228549.2 | 0.911228439 | 2.031427029 | 0.042211695 |
| GNGT1 | ENST00000412519.1 | ENSG00000227599.1 | 0.893316455 | 2.014236166 | 0.043984744 |
| GNGT1 | ENST00000413887.1 | ENSG00000236948.1 | 0.859048572 | 1.940205673 | 0.052354699 |
| GNGT1 | ENST00000419296.1 | ENSG00000204588.5 | 0.81125033 | 1.813976241 | 0.069681384 |
| GNGT1 | ENST00000419734.1 | ENSG00000234646.1 | 0.882202099 | 1.980820665 | 0.047611389 |
| GNGT1 | ENST00000421617.1 | ENSG00000237342.1 | -0.838165348 | -1.896945248 | 0.057835165 |
| GNGT1 | ENST00000422807.1 | ENSG00000227683.1 | -0.838722079 | -1.897943526 | 0.057703522 |
| GNGT1 | ENST00000424852.1 | ENSG00000229891.1 | 0.907221817 | 2.035407651 | 0.041809872 |
| GNGT1 | ENST00000429681.1 | ENSG00000235236.1 | 0.859996228 | 1.915539117 | 0.055423785 |
| GNGT1 | ENST00000429916.1 | ENSG00000227708.1 | -0.889109907 | -1.98936131 | 0.046661337 |
| GNGT1 | ENST00000430025.1 | ENSG00000233508.1 | 0.942335696 | 2.095348949 | 0.036139984 |
| GNGT1 | ENST00000433249.1 | ENSG00000236556.1 | -0.808435261 | -1.817238863 | 0.069180547 |
| GNGT1 | ENST00000441295.1 | ENSG00000233960.1 | 0.938283105 | 2.103585649 | 0.035414607 |
| GNGT1 | ENST00000441666.1 | ENSG00000230379.1 | -0.903307941 | -2.014622297 | 0.043944239 |

| | | | | | |
|---|---|---|---|---|---|
| GNGT1 | ENST00000441991.1 | ENSG00000231210.2 | 0.977511595 | 2.180130454 | 0.029247793 |
| GNGT1 | ENST00000443066.2 | ENSG00000237633.2 | -0.933310365 | -2.117193653 | 0.034243416 |
| GNGT1 | ENST00000443162.1 | ENSG00000234183.1 | -0.912233981 | -2.041192157 | 0.041231731 |
| GNGT1 | ENST00000448086.1 | ENSG00000237571.1 | 0.920270301 | 2.059660576 | 0.039431001 |
| GNGT1 | ENST00000448491.1 | ENSG00000231212.1 | 0.84726249 | 1.87815831 | 0.060359512 |
| GNGT1 | ENST00000449903.1 | ENSG00000223872.1 | 0.809017541 | 1.784350441 | 0.074366739 |
| GNGT1 | ENST00000450063.1 | ENSG00000231210.2 | 0.820629259 | 1.81595197 | 0.06937774 |
| GNGT1 | ENST00000451697.1 | ENSG00000233823.1 | 0.887762769 | 1.987794975 | 0.04683437 |
| GNGT1 | ENST00000456999.1 | ENSG00000230690.1 | -0.966121684 | -2.152990655 | 0.031319413 |
| GNGT1 | ENST00000457975.2 | ENSG00000236744.2 | 0.825808655 | 1.86568544 | 0.062085388 |
| GNGT1 | ENST00000479039.1 | ENSG00000241224.2 | 0.917021015 | 2.021314484 | 0.043247221 |
| GNGT1 | ENST00000490375.1 | ENSG00000240032.1 | -0.876871157 | -1.985286532 | 0.047112602 |
| GNGT1 | ENST00000503470.1 | ENSG00000248559.1 | -0.957610742 | -2.119815916 | 0.034021573 |
| GNGT1 | ENST00000503987.1 | ENSG00000250075.1 | -0.814583754 | -1.833608784 | 0.066712087 |
| GNGT1 | ENST00000505978.1 | ENSG00000249982.1 | 0.827872893 | 1.840507642 | 0.065693743 |
| GNGT1 | ENST00000506420.1 | ENSG00000250034.1 | 0.853516734 | 1.906229338 | 0.056620457 |
| GNGT1 | ENST00000508004.2 | ENSG00000251661.3 | -0.852652658 | -1.905691686 | 0.056690219 |
| GNGT1 | ENST00000510001.2 | ENSG00000249196.2 | 0.959840955 | 2.169234972 | 0.030064849 |
| GNGT1 | ENST00000514661.1 | ENSG00000247993.2 | -0.847103047 | -1.88336114 | 0.059651443 |
| GNGT1 | ENST00000514737.1 | ENSG00000250597.1 | -0.896870986 | -2.01662477 | 0.043734686 |
| GNGT1 | ENST00000518894.1 | ENSG00000204758.3 | -0.803309734 | -1.812893118 | 0.069848308 |
| GNGT1 | ENST00000519368.1 | ENSG00000253215.1 | -0.815485527 | -1.855574181 | 0.063514277 |
| GNGT1 | ENST00000520838.1 | ENSG00000253404.1 | -0.894202331 | -1.98084847 | 0.047608269 |
| GNGT1 | ENST00000520849.1 | ENSG00000253553.1 | -0.923452665 | -2.058791632 | 0.039514204 |
| GNGT1 | ENST00000521055.1 | ENSG00000253184.1 | 0.861529347 | 1.914463638 | 0.055560941 |
| GNGT1 | ENST00000521403.1 | ENSG00000253603.1 | 0.946850203 | 2.083065452 | 0.037245258 |
| GNGT1 | ENST00000522123.1 | ENSG00000253836.1 | -0.943576502 | -2.077657055 | 0.037740957 |
| GNGT1 | ENST00000532947.1 | ENSG00000255322.1 | -0.813653933 | -1.828014903 | 0.067547315 |
| GNGT1 | ENST00000533101.1 | ENSG00000255311.1 | -0.832386759 | -1.867198306 | 0.0618739 |
| GNGT1 | ENST00000537492.1 | ENSG00000256637.2 | -0.833875354 | -1.87189344 | 0.061221347 |
| GNGT1 | ENST00000540739.1 | ENSG00000249196.2 | 0.898499908 | 2.002461802 | 0.045235087 |
| GNGT1 | ENST00000546135.1 | ENSG00000256670.1 | 0.804670638 | 1.794206481 | 0.072780218 |
| GNGT1 | ENST00000551361.1 | ENSG00000224078.8 | 0.802031351 | 1.779229125 | 0.075202205 |
| GNGT1 | ENST00000553348.1 | ENSG00000258829.1 | 0.958523227 | 2.14295821 | 0.032116454 |
| GNGT1 | ENST00000553537.1 | ENSG00000258481.1 | 0.805561934 | 1.802475666 | 0.0714706 |
| GNGT1 | ENST00000556145.1 | ENSG00000258829.1 | 0.927227625 | 2.093044433 | 0.036345188 |
| GNGT1 | ENST00000556978.1 | ENSG00000258693.1 | -0.875466385 | -1.934811605 | 0.053013419 |
| GNGT1 | ENST00000559041.1 | ENSG00000259713.1 | -0.942689782 | -2.117044029 | 0.034256111 |
| GNGT1 | ENST00000561039.1 | ENSG00000259536.1 | 0.825618245 | 1.80279504 | 0.071420409 |
| GNGT1 | ENST00000561567.1 | ENSG00000260177.1 | -0.819221322 | -1.819465538 | 0.068840436 |
| GNGT1 | ENST00000563449.2 | ENSG00000261613.2 | -0.962419562 | -2.156487814 | 0.031045594 |
| GNGT1 | ENST00000564417.1 | ENSG00000260137.1 | 0.852640596 | 1.885670324 | 0.059339394 |
| GNGT1 | ENST00000569328.1 | ENSG00000261638.1 | -0.897575796 | -1.998056078 | 0.045710581 |
| GNGT1 | ENST00000569778.1 | ENSG00000260823.1 | 0.818461344 | 1.834325316 | 0.066605718 |
| GNGT1 | ENST00000569998.1 | ENSG00000260975.1 | 0.811050658 | 1.799906858 | 0.071875347 |
| GNGT1 | ENST00000571404.1 | ENSG00000262370.1 | -0.904285865 | -1.997709013 | 0.045748216 |
| GNGT1 | ENST00000582895.1 | ENSG00000264729.1 | 0.865231756 | 1.910797298 | 0.056030635 |
| GNGT1 | ENST00000585072.1 | ENSG00000263745.1 | -0.817561086 | -1.818746352 | 0.068950136 |
| GNGT1 | ENST00000585877.1 | ENSG00000267249.1 | 0.860121202 | 1.934299959 | 0.053076258 |
| GNGT1 | ENST00000586503.1 | ENSG00000267205.1 | 0.834194753 | 1.866441732 | 0.061979589 |
| GNGT1 | ENST00000587281.1 | ENSG00000228290.2 | -0.84245788 | -1.879174325 | 0.060220694 |

| | | | | | |
|---|---|---|---|---|---|
| GNGT1 | ENST00000590046.1 | ENSG00000266950.1 | 0.931467326 | 2.054645502 | 0.039913257 |
| GNGT1 | ENST00000593588.1 | ENSG00000269635.1 | -0.877217866 | -1.941865069 | 0.052153437 |
| GNGT1 | ENST00000596473.1 | ENSG00000268650.3 | -0.93773138 | -2.097038586 | 0.03599016 |
| GNGT1 | ENST00000597865.1 | ENSG00000268108.1 | -0.82029267 | -1.83232776 | 0.066902604 |
| GNGT1 | ENST00000598131.1 | ENSG00000269043.1 | -0.864512552 | -1.931281075 | 0.053448302 |
| GNGT1 | ENST00000600959.1 | ENSG00000269303.1 | -0.821140491 | -1.838617783 | 0.065971424 |
| GNGT1 | ENST00000601752.1 | ENSG00000268051.1 | -0.816747734 | -1.823246549 | 0.068266058 |
| GNGT1 | ENST00000602051.1 | ENSG00000227877.2 | 0.843979434 | 1.907892526 | 0.056405108 |
| GNGT1 | ENST00000602900.1 | ENSG00000270179.1 | 0.835758218 | 1.865187376 | 0.062155144 |
| GNGT1 | ENST00000603612.1 | ENSG00000270996.1 | -0.802620559 | -1.794363245 | 0.072755209 |
| GNGT1 | ENST00000606778.1 | ENSG00000271930.1 | -0.908683493 | -2.023187447 | 0.043053825 |
| GNGT1 | ENST00000607136.1 | ENSG00000267546.2 | 0.913950553 | 2.059776698 | 0.039419893 |
| GNGT1 | ENST00000607321.1 | ENSG00000272371.1 | 0.912633088 | 2.059610496 | 0.039435792 |
| GNGT1 | ENST00000607580.1 | ENSG00000272545.1 | 0.861918512 | 1.913554026 | 0.055677164 |
| GNGT1 | ENST00000607600.1 | ENSG00000272114.1 | 0.885617292 | 1.977625771 | 0.04797094 |
| GNGT1 | ENST00000609218.1 | ENSG00000272945.1 | -0.920761067 | -2.042370624 | 0.041114781 |
| GNGT1 | ENST00000609238.1 | ENSG00000272703.1 | 0.877324935 | 1.951719354 | 0.050971533 |
| GNGT1 | ENST00000610034.1 | ENSG00000272912.1 | -0.844031243 | -1.892777578 | 0.058387455 |
| GNGT1 | NR_045637.1 | BOLA3-AS1 | -0.800168813 | -1.776269158 | 0.075688564 |
| GNGT1 | NR_046556.1 | RBMS3-AS1 | -0.840529892 | -1.869604512 | 0.061538758 |
| GNGT1 | NR_046783.1 | KCND3-IT1 | 0.917749919 | 2.050668247 | 0.040299265 |
| GNGT1 | NR_102737.1 | LINC00911 | 0.907507961 | 2.024003993 | 0.042969741 |
| GNGT1 | NR_102738.1 | LINC00911 | 0.907507961 | 2.025856463 | 0.042779496 |
| GNGT1 | NR_103851.1 | TAT-AS1 | -0.86401936 | -1.938804762 | 0.052525116 |
| GNGT1 | NR_110007.1 | ADNP-AS1 | -0.88666132 | -2.006714733 | 0.044780046 |
| GNGT1 | NR_110008.1 | ADNP-AS1 | -0.884478311 | -1.973412394 | 0.048448597 |
| GNGT1 | NR_110009.1 | ADNP-AS1 | -0.884478311 | -1.968805945 | 0.048975379 |
| GNGT1 | NR_120595.1 | LINC01315 | 0.878896961 | 1.965743087 | 0.049328295 |
| GNGT1 | NR_121577.1 | NALT1 | 0.811450301 | 1.812946943 | 0.069840005 |
| GNGT1 | NR_126354.1 | LINC01331 | 0.940757564 | 2.095858929 | 0.036094707 |
| GNGT1 | NR_126370.1 | GACAT1 | 0.864821523 | 1.930297622 | 0.053569972 |
| GNGT1 | NR_131985.1 | CRAT8 | 0.905989463 | 2.021807486 | 0.043196244 |
| GNGT1 | NR_133907.1 | HLA-DQB1-AS1 | -0.893651043 | -1.971198046 | 0.048701227 |
| GNGT1 | NR_134245.1 | LOC105379194 | -0.898103388 | -2.013856111 | 0.044024643 |
| GNGT1 | NR_134910.1 | LOC102725254 | -0.93054733 | -2.083386686 | 0.037215991 |
| GNGT1 | NR_135251.1 | LOC101928143 | 0.843339105 | 1.882031082 | 0.059831796 |
| GPS2 | ENST00000340585.6 | ENSG00000249429.1 | -0.806217708 | -1.793279062 | 0.072928314 |
| GPS2 | ENST00000399186.2 | ENSG00000214888.2 | 0.843684105 | 1.900706908 | 0.057340413 |
| GPS2 | ENST00000411694.1 | ENSG00000225331.1 | 0.875748551 | 1.963789856 | 0.049554467 |
| GPS2 | ENST00000412085.1 | ENSG00000233825.1 | 0.901744372 | 2.015562049 | 0.043845792 |
| GPS2 | ENST00000412759.1 | ENSG00000236933.1 | 0.853176657 | 1.909049045 | 0.056255765 |
| GPS2 | ENST00000412772.1 | ENSG00000231507.1 | 0.814588136 | 1.82643694 | 0.067784472 |
| GPS2 | ENST00000413887.1 | ENSG00000236948.1 | -0.884937056 | -1.985266397 | 0.047114841 |
| GPS2 | ENST00000414740.2 | ENSG00000229646.2 | 0.916104 | 2.075965398 | 0.037897151 |
| GPS2 | ENST00000419662.1 | ENSG00000228265.1 | 0.820186489 | 1.827570222 | 0.067614078 |
| GPS2 | ENST00000419734.1 | ENSG00000234646.1 | -0.846446341 | -1.891903871 | 0.05850379 |
| GPS2 | ENST00000420315.1 | ENSG00000228072.1 | 0.949613421 | 2.119500646 | 0.034048179 |
| GPS2 | ENST00000420465.1 | ENSG00000167355.3 | 0.893854418 | 1.992987203 | 0.046262848 |
| GPS2 | ENST00000420981.2 | ENSG00000230438.5 | 0.981080366 | 2.193667362 | 0.028259332 |
| GPS2 | ENST00000421020.1 | ENSG00000231407.1 | 0.903845652 | 2.026467258 | 0.042716925 |
| GPS2 | ENST00000424181.1 | ENSG00000224977.1 | 0.913153369 | 2.026862776 | 0.042676448 |

| | | | | | |
|---|---|---|---|---|---|
| GPS2 | ENST00000426519.1 | ENSG00000234142.1 | 0.893530675 | 2.037277049 | 0.041622286 |
| GPS2 | ENST00000429608.1 | ENSG00000237480.1 | 0.863628533 | 1.954631153 | 0.050626622 |
| GPS2 | ENST00000429630.1 | ENSG00000232533.1 | 0.935159945 | 2.089427136 | 0.036669291 |
| GPS2 | ENST00000433036.1 | ENSG00000228989.1 | 0.929162352 | 2.089960419 | 0.036621355 |
| GPS2 | ENST00000434627.1 | ENSG00000230074.1 | 0.918515334 | 2.031826192 | 0.042171255 |
| GPS2 | ENST00000435733.1 | ENSG00000226377.1 | 0.822859552 | 1.832313675 | 0.066904701 |
| GPS2 | ENST00000437330.1 | ENSG00000229203.1 | 0.898762886 | 2.050247234 | 0.040340311 |
| GPS2 | ENST00000439184.1 | ENSG00000233985.1 | -0.885294827 | -1.977745301 | 0.047957447 |
| GPS2 | ENST00000442069.1 | ENSG00000225655.1 | -0.822549678 | -1.835745656 | 0.066395282 |
| GPS2 | ENST00000442850.1 | ENSG00000232600.2 | -0.836991712 | -1.875841404 | 0.060677061 |
| GPS2 | ENST00000443306.1 | ENSG00000233891.3 | 0.81493778 | 1.832712581 | 0.066845325 |
| GPS2 | ENST00000443380.1 | ENSG00000224371.1 | 0.854418699 | 1.908464254 | 0.056331239 |
| GPS2 | ENST00000448365.1 | ENSG00000231114.1 | 0.853821245 | 1.905459694 | 0.056720342 |
| GPS2 | ENST00000450063.1 | ENSG00000231210.2 | -0.839704101 | -1.869577644 | 0.061542492 |
| GPS2 | ENST00000450072.1 | ENSG00000228486.5 | 0.816970573 | 1.824634115 | 0.068056262 |
| GPS2 | ENST00000450109.1 | ENSG00000225376.1 | 0.825451906 | 1.872350577 | 0.061158118 |
| GPS2 | ENST00000453051.1 | ENSG00000229407.1 | 0.908262282 | 2.014510849 | 0.043955927 |
| GPS2 | ENST00000454530.1 | ENSG00000226649.1 | -0.892979574 | -2.002980293 | 0.045179404 |
| GPS2 | ENST00000457115.1 | ENSG00000227245.1 | 0.85461932 | 1.92352797 | 0.054413777 |
| GPS2 | ENST00000457253.1 | ENSG00000225173.1 | 0.980651752 | 2.196913631 | 0.028026615 |
| GPS2 | ENST00000458154.1 | ENSG00000235578.1 | 0.807400287 | 1.802447143 | 0.071475084 |
| GPS2 | ENST00000458194.1 | ENSG00000226193.1 | 0.918758768 | 2.053179308 | 0.04005519 |
| GPS2 | ENST00000458364.1 | ENSG00000225655.1 | -0.81973637 | -1.846681916 | 0.064793254 |
| GPS2 | ENST00000463255.1 | ENSG00000243305.1 | -0.822315062 | -1.852744084 | 0.063919048 |
| GPS2 | ENST00000466431.2 | ENSG00000254485.1 | 0.947389593 | 2.108735251 | 0.03496744 |
| GPS2 | ENST00000472596.1 | ENSG00000239774.1 | 0.879240588 | 1.956223887 | 0.050438786 |
| GPS2 | ENST00000476892.1 | ENSG00000241345.1 | 0.861326538 | 1.931541551 | 0.053416116 |
| GPS2 | ENST00000490013.1 | ENSG00000184115.12 | 0.832871261 | 1.85139356 | 0.064112955 |
| GPS2 | ENST00000493123.1 | ENSG00000242428.1 | 0.849092977 | 1.934256355 | 0.053081617 |
| GPS2 | ENST00000498358.1 | ENSG00000184115.12 | 0.828933431 | 1.859641932 | 0.062936203 |
| GPS2 | ENST00000505196.1 | ENSG00000248131.1 | 0.863417193 | 1.928888296 | 0.053744731 |
| GPS2 | ENST00000505556.1 | ENSG00000249409.1 | 0.827881316 | 1.851762238 | 0.064059972 |
| GPS2 | ENST00000506100.1 | ENSG00000249409.1 | 0.811540165 | 1.80197745 | 0.071548953 |
| GPS2 | ENST00000508188.1 | ENSG00000250999.1 | 0.953334383 | 2.132479477 | 0.03296745 |
| GPS2 | ENST00000508241.1 | ENSG00000248518.1 | 0.915453037 | 2.06825443 | 0.038616104 |
| GPS2 | ENST00000509036.1 | ENSG00000251131.1 | 0.873779479 | 1.974122003 | 0.048367872 |
| GPS2 | ENST00000515750.1 | ENSG00000249061.1 | -0.804680361 | -1.815350222 | 0.069470106 |
| GPS2 | ENST00000517300.1 | ENSG00000254144.2 | 0.966046683 | 2.169945382 | 0.030010984 |
| GPS2 | ENST00000517846.1 | ENSG00000254485.1 | 0.858325619 | 1.904740917 | 0.056813757 |
| GPS2 | ENST00000519451.1 | ENSG00000253363.1 | 0.90307757 | 2.040256506 | 0.041324784 |
| GPS2 | ENST00000521207.1 | ENSG00000253716.1 | 0.818334096 | 1.81544532 | 0.069455502 |
| GPS2 | ENST00000524073.1 | ENSG00000253774.1 | 0.84464594 | 1.854369378 | 0.063686333 |
| GPS2 | ENST00000526154.1 | ENSG00000254511.1 | 0.952290472 | 2.148688189 | 0.031659126 |
| GPS2 | ENST00000526694.1 | ENSG00000231999.2 | 0.850033171 | 1.886764249 | 0.059192042 |
| GPS2 | ENST00000528000.1 | ENSG00000254804.1 | 0.847565541 | 1.904956377 | 0.056785742 |
| GPS2 | ENST00000531627.1 | ENSG00000254584.1 | 0.855730053 | 1.916379477 | 0.055316811 |
| GPS2 | ENST00000532249.1 | ENSG00000234899.5 | 0.837674883 | 1.879329805 | 0.060199474 |
| GPS2 | ENST00000532688.1 | ENSG00000255441.1 | 0.956338203 | 2.122343855 | 0.033808873 |
| GPS2 | ENST00000534065.1 | ENSG00000254458.1 | 0.824154784 | 1.830008449 | 0.067248675 |
| GPS2 | ENST00000536141.1 | ENSG00000256969.1 | 0.815878986 | 1.800273535 | 0.071817458 |
| GPS2 | ENST00000537269.1 | ENSG00000257084.1 | 0.84546709 | 1.896228447 | 0.057929843 |

| | | | | | |
|---|---|---|---|---|---|
| GPS2 | ENST00000543072.1 | ENSG00000256092.2 | -0.817795701 | -1.828206233 | 0.067518606 |
| GPS2 | ENST00000543275.1 | ENSG00000256944.1 | 0.821493341 | 1.852232543 | 0.063992438 |
| GPS2 | ENST00000545177.3 | ENSG00000230438.5 | 0.972112109 | 2.172687303 | 0.02980386 |
| GPS2 | ENST00000545254.1 | ENSG00000256633.1 | 0.85065135 | 1.902278102 | 0.057134807 |
| GPS2 | ENST00000545642.1 | ENSG00000256342.1 | 0.853869953 | 1.918292031 | 0.055073992 |
| GPS2 | ENST00000547750.1 | ENSG00000257886.1 | 0.842610519 | 1.892101097 | 0.058477512 |
| GPS2 | ENST00000548722.2 | ENSG00000257194.2 | -0.85231566 | -1.891321396 | 0.058581454 |
| GPS2 | ENST00000549806.1 | ENSG00000257252.1 | 0.843823762 | 1.900069054 | 0.057424058 |
| GPS2 | ENST00000552525.1 | ENSG00000257286.1 | 0.87506782 | 1.958350421 | 0.050188908 |
| GPS2 | ENST00000554197.1 | ENSG00000197176.3 | 0.825860108 | 1.831034591 | 0.06709538 |
| GPS2 | ENST00000558515.1 | ENSG00000259182.1 | 0.960765412 | 2.141358443 | 0.032245143 |
| GPS2 | ENST00000560522.1 | ENSG00000259661.1 | 0.852207559 | 1.880033265 | 0.060103545 |
| GPS2 | ENST00000561529.1 | ENSG00000260886.1 | 0.839964544 | 1.867706783 | 0.061802953 |
| GPS2 | ENST00000561567.1 | ENSG00000260177.1 | 0.817329346 | 1.82141931 | 0.068543142 |
| GPS2 | ENST00000563044.1 | ENSG00000260978.1 | 0.882222728 | 1.961865434 | 0.049778153 |
| GPS2 | ENST00000563806.1 | ENSG00000238045.5 | 0.931967496 | 2.068692404 | 0.03857496 |
| GPS2 | ENST00000565829.1 | ENSG00000260148.1 | 0.819612888 | 1.830120107 | 0.067231981 |
| GPS2 | ENST00000570843.1 | ENSG00000261889.1 | 0.811861395 | 1.829111865 | 0.067382852 |
| GPS2 | ENST00000574460.1 | ENSG00000263051.1 | 0.809789988 | 1.810569495 | 0.070207518 |
| GPS2 | ENST00000578757.1 | ENSG00000175061.13 | 0.844494713 | 1.886786589 | 0.059189036 |
| GPS2 | ENST00000578800.1 | ENSG00000264235.1 | 0.902612369 | 2.012738337 | 0.044142164 |
| GPS2 | ENST00000578936.1 | ENSG00000265547.1 | 0.946536873 | 2.119993522 | 0.034006592 |
| GPS2 | ENST00000579154.1 | ENSG00000265908.1 | -0.827668301 | -1.848848491 | 0.064479693 |
| GPS2 | ENST00000581905.1 | ENSG00000264235.1 | 0.812075022 | 1.82589908 | 0.067865465 |
| GPS2 | ENST00000582386.1 | ENSG00000265174.1 | 0.871670495 | 1.933587803 | 0.053163828 |
| GPS2 | ENST00000583138.1 | ENSG00000263393.1 | 0.818217056 | 1.831288571 | 0.067057483 |
| GPS2 | ENST00000586694.1 | ENSG00000267141.1 | 0.820217944 | 1.84464997 | 0.065088473 |
| GPS2 | ENST00000588182.2 | ENSG00000267453.2 | 0.816633825 | 1.824319649 | 0.068103762 |
| GPS2 | ENST00000588290.1 | ENSG00000267751.1 | 0.928417933 | 2.07262748 | 0.038206962 |
| GPS2 | ENST00000589817.1 | ENSG00000231616.4 | 0.948763022 | 2.14408773 | 0.032025858 |
| GPS2 | ENST00000590368.1 | ENSG00000231616.4 | 0.873498202 | 1.952767917 | 0.050847102 |
| GPS2 | ENST00000590813.1 | ENSG00000231616.4 | 0.889749441 | 1.995866947 | 0.045948407 |
| GPS2 | ENST00000593269.1 | ENSG00000236172.2 | 0.855021625 | 1.928662256 | 0.053772805 |
| GPS2 | ENST00000593588.1 | ENSG00000269635.1 | 0.834324025 | 1.874248004 | 0.060896251 |
| GPS2 | ENST00000593632.1 | ENSG00000180279.5 | 0.890618256 | 1.989865386 | 0.046605766 |
| GPS2 | ENST00000593642.1 | ENSG00000267858.1 | 0.899934576 | 2.004233312 | 0.045045074 |
| GPS2 | ENST00000594492.1 | ENSG00000250910.3 | 0.943640256 | 2.118837329 | 0.034104217 |
| GPS2 | ENST00000595955.1 | ENSG00000268401.1 | 0.895838419 | 2.006736817 | 0.044777694 |
| GPS2 | ENST00000596135.1 | ENSG00000269843.1 | 0.861005546 | 1.922972232 | 0.054483537 |
| GPS2 | ENST00000597256.1 | ENSG00000267986.1 | 0.81615077 | 1.807301661 | 0.070715257 |
| GPS2 | ENST00000600234.1 | ENSG00000268078.1 | 0.846378302 | 1.882233839 | 0.059804273 |
| GPS2 | ENST00000600534.1 | ENSG00000267858.1 | 0.806619318 | 1.820439599 | 0.068692087 |
| GPS2 | ENST00000600889.1 | ENSG00000232675.3 | 0.960681631 | 2.158572724 | 0.030883331 |
| GPS2 | ENST00000601033.1 | ENSG00000268401.1 | 0.947015331 | 2.141201222 | 0.032257815 |
| GPS2 | ENST00000602532.1 | ENSG00000270091.1 | 0.873920334 | 1.957297111 | 0.050312547 |
| GPS2 | ENST00000606068.1 | ENSG00000272342.1 | 0.812462588 | 1.809865199 | 0.070316694 |
| GPS2 | ENST00000606277.1 | ENSG00000272145.1 | 0.819004419 | 1.83366678 | 0.066703473 |
| GPS2 | ENST00000606841.1 | ENSG00000272411.1 | 0.874548549 | 1.960579646 | 0.049928079 |
| GPS2 | ENST00000606963.1 | ENSG00000272010.1 | -0.84660479 | -1.883736589 | 0.059600615 |
| GPS2 | ENST00000607201.1 | ENSG00000272024.1 | -0.915950901 | -2.059396336 | 0.039456287 |
| GPS2 | ENST00000607321.1 | ENSG00000272371.1 | -0.827857085 | -1.844949623 | 0.065044867 |

| | | | | | |
|---|---|---|---|---|---|
| GPS2 | ENST00000607580.1 | ENSG00000272545.1 | -0.876482431 | -1.971294919 | 0.048690152 |
| GPS2 | ENST00000608264.1 | ENSG00000273473.1 | 0.831831434 | 1.85904734 | 0.063020428 |
| GPS2 | ENST00000608677.1 | ENSG00000273350.1 | 0.868796581 | 1.924965453 | 0.05423368 |
| GPS2 | ENST00000608940.1 | ENSG00000272763.1 | 0.942299867 | 2.125680439 | 0.033529877 |
| GPS2 | NR_003604.2 | ZFAS1 | 0.852756534 | 1.89718394 | 0.057803666 |
| GPS2 | NR_003606.2 | ZFAS1 | 0.846728681 | 1.883852685 | 0.059584905 |
| GPS2 | NR_026802.1 | FAM74A4 | 0.908567088 | 2.052319933 | 0.04013858 |
| GPS2 | NR_026951.1 | LINC00324 | 0.884425332 | 1.969948101 | 0.048844319 |
| GPS2 | NR_027052.1 | THAP7-AS1 | 0.817311885 | 1.840194773 | 0.065739647 |
| GPS2 | NR_027271.1 | CIRBP-AS1 | 0.847371678 | 1.896595751 | 0.057881312 |
| GPS2 | NR_036480.1 | VPS9D1-AS1 | 0.820716797 | 1.852226205 | 0.063993347 |
| GPS2 | NR_036658.1 | ZFAS1 | 0.845938647 | 1.896659521 | 0.057872889 |
| GPS2 | NR_040096.1 | LOC643339 | 0.883831353 | 1.968523507 | 0.049007834 |
| GPS2 | NR_045637.1 | BOLA3-AS1 | 0.912998451 | 2.056129863 | 0.03977 |
| GPS2 | NR_046713.1 | NAALADL2-AS2 | -0.871451512 | -1.955334104 | 0.050543648 |
| GPS2 | NR_046839.1 | AGBL4-IT1 | -0.932115105 | -2.089133225 | 0.036695732 |
| GPS2 | NR_103790.1 | LINC00581 | -0.920104171 | -2.054855566 | 0.039892957 |
| GPS2 | NR_103851.1 | TAT-AS1 | 0.838913516 | 1.868790653 | 0.061651946 |
| GPS2 | NR_104158.1 | NRG1-IT1 | 0.811944255 | 1.811167455 | 0.070114934 |
| GPS2 | NR_105010.1 | LINC01333 | 0.810707447 | 1.793670292 | 0.07286581 |
| GPS2 | NR_109886.1 | RALY-AS1 | 0.812824599 | 1.824510737 | 0.068074895 |
| GPS2 | NR_110053.1 | LOC101927464 | 0.853869953 | 1.909361803 | 0.056215435 |
| GPS2 | NR_110998.1 | FAM74A4 | 0.908567088 | 2.039185335 | 0.041431534 |
| GPS2 | NR_111952.1 | LINC00869 | 0.808980092 | 1.80500741 | 0.071073522 |
| GPS2 | NR_117097.1 | LINC01353 | 0.814588136 | 1.797565732 | 0.072245853 |
| GPS2 | NR_117098.1 | LINC01353 | 0.847344455 | 1.903826003 | 0.056932849 |
| GPS2 | NR_121189.1 | PGM5P3-AS1 | -0.81433843 | -1.822498611 | 0.068379364 |
| GPS2 | NR_121661.1 | ZBTB20-AS5 | -0.894969879 | -1.999758685 | 0.045526328 |
| GPS2 | NR_126522.1 | EXOC3-AS1 | 0.894542778 | 1.991426029 | 0.04643407 |
| GPS2 | NR_130143.1 | LOC104968399 | 0.902126398 | 2.022927606 | 0.043080612 |
| GPS2 | NR_130144.1 | LOC104968399 | 0.812075022 | 1.836450874 | 0.066291001 |
| GPS2 | NR_134579.1 | LOC105372179 | 0.943080954 | 2.120409987 | 0.033971485 |
| GPS2 | NR_135024.1 | LOC105369747 | 0.887349735 | 1.984564662 | 0.047192927 |
| GPS2 | NR_135032.1 | LOC105369635 | 0.84546709 | 1.878876988 | 0.060261292 |
| GPS2 | NR_135820.1 | LOC102723727 | 0.827572474 | 1.832329806 | 0.066902299 |
| GPS2 | NR_138038.1 | LINC00677 | 0.805494707 | 1.789080177 | 0.073601904 |
| GSTT1 | ENST00000411824.1 | ENSG00000232803.1 | -0.813593806 | -1.830515859 | 0.067172838 |
| GSTT1 | ENST00000412772.1 | ENSG00000231507.1 | 0.810705525 | 1.808916421 | 0.070463989 |
| GSTT1 | ENST00000413645.1 | ENSG00000228798.1 | 0.909052452 | 2.04961427 | 0.040402087 |
| GSTT1 | ENST00000416657.1 | ENSG00000235858.1 | 0.860396292 | 1.926838451 | 0.053999766 |
| GSTT1 | ENST00000418972.1 | ENSG00000225044.1 | -0.833142795 | -1.865703639 | 0.06208284 |
| GSTT1 | ENST00000420498.1 | ENSG00000224985.1 | 0.911240839 | 2.038946017 | 0.041455416 |
| GSTT1 | ENST00000420981.2 | ENSG00000230438.5 | 0.817272931 | 1.807674615 | 0.070657158 |
| GSTT1 | ENST00000422038.1 | ENSG00000227935.1 | 0.831490683 | 1.866034641 | 0.062036519 |
| GSTT1 | ENST00000425371.2 | ENSG00000235872.2 | 0.870412439 | 1.920579983 | 0.054784681 |
| GSTT1 | ENST00000427691.1 | ENSG00000228340.1 | -0.899733269 | -2.016205701 | 0.043778471 |
| GSTT1 | ENST00000429608.1 | ENSG00000237480.1 | 0.944257271 | 2.129067715 | 0.033248658 |
| GSTT1 | ENST00000429630.1 | ENSG00000232533.1 | 0.819423218 | 1.832518668 | 0.066874183 |
| GSTT1 | ENST00000433036.1 | ENSG00000228989.1 | 0.808614473 | 1.817459135 | 0.06914684 |
| GSTT1 | ENST00000437330.1 | ENSG00000229203.1 | 0.937074474 | 2.100669793 | 0.035669963 |
| GSTT1 | ENST00000437461.1 | ENSG00000227200.1 | 0.817769316 | 1.834506006 | 0.066578917 |

| | | | | | |
|---|---|---|---|---|---|
| GSTT1 | ENST00000443380.1 | ENSG00000224371.1 | 0.884650548 | 1.995055771 | 0.046036798 |
| GSTT1 | ENST00000448365.1 | ENSG00000231114.1 | 0.855198577 | 1.882075724 | 0.059825735 |
| GSTT1 | ENST00000450109.1 | ENSG00000225376.1 | 0.85996187 | 1.91839462 | 0.055060993 |
| GSTT1 | ENST00000455010.1 | ENSG00000233079.1 | 0.801866597 | 1.795080861 | 0.072640818 |
| GSTT1 | ENST00000460993.1 | ENSG00000241231.1 | 0.820791353 | 1.839533884 | 0.065836699 |
| GSTT1 | ENST00000466431.2 | ENSG00000254485.1 | 0.836953885 | 1.871905129 | 0.06121973 |
| GSTT1 | ENST00000476892.1 | ENSG00000241345.1 | 0.858578562 | 1.945929815 | 0.051663171 |
| GSTT1 | ENST00000498358.1 | ENSG00000184115.12 | 0.830576353 | 1.862274402 | 0.062564422 |
| GSTT1 | ENST00000502300.1 | ENSG00000249451.1 | 0.949101192 | 2.115961703 | 0.034348065 |
| GSTT1 | ENST00000507997.1 | ENSG00000250551.1 | -0.812025246 | -1.828648019 | 0.067452354 |
| GSTT1 | ENST00000508188.1 | ENSG00000250999.1 | 0.874573249 | 1.970121004 | 0.048824504 |
| GSTT1 | ENST00000508199.1 | ENSG00000247810.2 | 0.892634001 | 2.004099728 | 0.045059379 |
| GSTT1 | ENST00000508241.1 | ENSG00000248518.1 | 0.945096783 | 2.11762966 | 0.034206444 |
| GSTT1 | ENST00000512300.1 | ENSG00000248362.1 | 0.803425312 | 1.819042433 | 0.068904957 |
| GSTT1 | ENST00000515077.1 | ENSG00000251206.1 | 0.804638469 | 1.782558261 | 0.074658239 |
| GSTT1 | ENST00000515136.1 | ENSG00000251274.1 | -0.8297585 | -1.86570137 | 0.062083158 |
| GSTT1 | ENST00000515750.1 | ENSG00000249061.1 | -0.81708247 | -1.846998404 | 0.064747372 |
| GSTT1 | ENST00000517300.1 | ENSG00000254144.2 | 0.82027898 | 1.830114535 | 0.067232814 |
| GSTT1 | ENST00000519451.1 | ENSG00000253363.1 | 0.905607889 | 2.019372283 | 0.04344854 |
| GSTT1 | ENST00000519852.1 | ENSG00000253716.1 | 0.841780464 | 1.879748606 | 0.060142348 |
| GSTT1 | ENST00000521207.1 | ENSG00000253716.1 | 0.880986612 | 1.974852471 | 0.048284892 |
| GSTT1 | ENST00000521884.1 | ENSG00000253355.1 | -0.805228846 | -1.793955232 | 0.072820315 |
| GSTT1 | ENST00000522524.1 | ENSG00000253342.1 | -0.86790785 | -1.92772375 | 0.053889496 |
| GSTT1 | ENST00000524073.1 | ENSG00000253774.1 | 0.835788409 | 1.871636769 | 0.061256873 |
| GSTT1 | ENST00000524335.1 | ENSG00000253716.1 | 0.839044981 | 1.870383774 | 0.061430544 |
| GSTT1 | ENST00000526154.1 | ENSG00000254511.1 | 0.871522457 | 1.980538947 | 0.047643001 |
| GSTT1 | ENST00000526694.1 | ENSG00000231999.2 | 0.832433223 | 1.846660576 | 0.064796349 |
| GSTT1 | ENST00000531627.1 | ENSG00000254584.1 | 0.844336053 | 1.879378831 | 0.060192785 |
| GSTT1 | ENST00000532688.1 | ENSG00000255441.1 | 0.904697995 | 2.01696977 | 0.043698668 |
| GSTT1 | ENST00000535746.1 | ENSG00000256101.1 | 0.953132817 | 2.121061196 | 0.033916653 |
| GSTT1 | ENST00000537921.1 | ENSG00000255966.1 | 0.821677099 | 1.828278438 | 0.067507774 |
| GSTT1 | ENST00000547750.1 | ENSG00000257886.1 | 0.926941767 | 2.065771509 | 0.03885006 |
| GSTT1 | ENST00000552525.1 | ENSG00000257286.1 | 0.803283696 | 1.797558356 | 0.072247023 |
| GSTT1 | ENST00000554049.1 | ENSG00000258763.1 | 0.812560534 | 1.83102181 | 0.067097288 |
| GSTT1 | ENST00000554197.1 | ENSG00000197176.3 | 0.904352389 | 2.03123617 | 0.042231043 |
| GSTT1 | ENST00000554430.1 | ENSG00000258646.1 | -0.853722242 | -1.909914734 | 0.056144193 |
| GSTT1 | ENST00000557232.1 | ENSG00000259054.1 | 0.807654882 | 1.811904951 | 0.070000884 |
| GSTT1 | ENST00000558515.1 | ENSG00000259182.1 | 0.920181165 | 2.025798097 | 0.042785479 |
| GSTT1 | ENST00000558875.1 | ENSG00000259737.2 | 0.882396987 | 1.978603466 | 0.047860669 |
| GSTT1 | ENST00000560522.1 | ENSG00000259661.1 | 0.842637579 | 1.877454656 | 0.060455807 |
| GSTT1 | ENST00000561215.1 | ENSG00000259611.1 | 0.923647922 | 2.101776172 | 0.035572887 |
| GSTT1 | ENST00000561529.1 | ENSG00000260886.1 | 0.834644205 | 1.887746929 | 0.059059933 |
| GSTT1 | ENST00000563806.1 | ENSG00000238045.5 | 0.83181458 | 1.862005678 | 0.06260229 |
| GSTT1 | ENST00000565359.1 | ENSG00000260601.1 | 0.834166923 | 1.854074087 | 0.063728561 |
| GSTT1 | ENST00000569742.1 | ENSG00000260787.1 | 0.806478349 | 1.777362486 | 0.075508619 |
| GSTT1 | ENST00000578936.1 | ENSG00000265547.1 | 0.808923391 | 1.781668767 | 0.074803263 |
| GSTT1 | ENST00000583916.1 | ENSG00000264196.1 | 0.866857825 | 1.946631792 | 0.051578894 |
| GSTT1 | ENST00000586399.1 | ENSG00000228430.4 | 0.810331417 | 1.801919643 | 0.071558049 |
| GSTT1 | ENST00000588908.1 | ENSG00000267751.1 | 0.819142924 | 1.831694096 | 0.066997009 |
| GSTT1 | ENST00000589233.1 | ENSG00000231616.4 | 0.919945705 | 2.057914895 | 0.039598304 |
| GSTT1 | ENST00000589457.1 | ENSG00000267751.1 | 0.841555984 | 1.874952819 | 0.060799215 |

| | | | | | |
|---|---|---|---|---|---|
| GSTT1 | ENST00000589817.1 | ENSG00000231616.4 | 0.950723633 | 2.108714738 | 0.034969212 |
| GSTT1 | ENST00000590292.1 | ENSG00000267751.1 | 0.836454086 | 1.860944761 | 0.062751978 |
| GSTT1 | ENST00000590368.1 | ENSG00000231616.4 | 0.981283331 | 2.196636668 | 0.028046405 |
| GSTT1 | ENST00000590813.1 | ENSG00000231616.4 | 0.941738177 | 2.110143298 | 0.034846015 |
| GSTT1 | ENST00000590995.1 | ENSG00000267198.1 | 0.935676136 | 2.086234721 | 0.036957367 |
| GSTT1 | ENST00000591103.1 | ENSG00000272895.1 | 0.85091386 | 1.900016075 | 0.05743101 |
| GSTT1 | ENST00000591217.1 | ENSG00000231616.4 | 0.928692374 | 2.074201656 | 0.038060586 |
| GSTT1 | ENST00000591225.1 | ENSG00000228290.2 | -0.801607287 | -1.789375896 | 0.073554298 |
| GSTT1 | ENST00000591836.1 | ENSG00000267776.1 | 0.857385526 | 1.887886955 | 0.059041128 |
| GSTT1 | ENST00000592622.1 | ENSG00000267546.2 | -0.910427055 | -2.059803834 | 0.039417298 |
| GSTT1 | ENST00000593269.1 | ENSG00000236172.2 | 0.958912659 | 2.146976701 | 0.031795137 |
| GSTT1 | ENST00000593642.1 | ENSG00000267858.1 | 0.903829498 | 2.017409644 | 0.043652782 |
| GSTT1 | ENST00000594492.1 | ENSG00000250910.3 | 0.939372923 | 2.081358368 | 0.037401116 |
| GSTT1 | ENST00000596497.1 | ENSG00000268530.1 | 0.855959367 | 1.935386602 | 0.052942872 |
| GSTT1 | ENST00000600071.1 | ENSG00000269199.1 | 0.805888916 | 1.808455834 | 0.070535585 |
| GSTT1 | ENST00000600512.1 | ENSG00000269752.1 | 0.848463048 | 1.890979393 | 0.058627095 |
| GSTT1 | ENST00000600889.1 | ENSG00000232675.3 | 0.83084074 | 1.872247894 | 0.061172316 |
| GSTT1 | ENST00000602614.1 | ENSG00000269957.1 | -0.906986349 | -2.002887039 | 0.045189415 |
| GSTT1 | ENST00000602773.1 | ENSG00000270160.1 | -0.890247913 | -2.00364721 | 0.045107865 |
| GSTT1 | ENST00000606068.1 | ENSG00000272342.1 | 0.848938855 | 1.878804746 | 0.060271159 |
| GSTT1 | ENST00000606841.1 | ENSG00000272411.1 | 0.867879471 | 1.939759375 | 0.05240894 |
| GSTT1 | ENST00000606938.1 | ENSG00000272198.1 | -0.812662777 | -1.819178864 | 0.068884146 |
| GSTT1 | ENST00000607014.1 | ENSG00000272345.1 | -0.864297306 | -1.935738705 | 0.052899711 |
| GSTT1 | ENST00000607044.1 | ENSG00000272247.1 | 0.857656281 | 1.902432053 | 0.057114694 |
| GSTT1 | ENST00000608264.1 | ENSG00000273473.1 | 0.881074 | 1.960744764 | 0.049908804 |
| GSTT1 | ENST00000608940.1 | ENSG00000272763.1 | 0.878071016 | 1.960422234 | 0.049946459 |
| GSTT1 | ENST00000608952.1 | ENSG00000272689.1 | -0.838060904 | -1.858284371 | 0.063128642 |
| GSTT1 | ENST00000609146.1 | ENSG00000272851.1 | -0.84006351 | -1.896299753 | 0.057920419 |
| GSTT1 | ENST00000609837.1 | ENSG00000273106.1 | 0.80108939 | 1.819900302 | 0.068774189 |
| GSTT1 | ENST00000609953.1 | ENSG00000272825.1 | 0.846433464 | 1.894467219 | 0.058163022 |
| GSTT1 | ENST00000610044.1 | ENSG00000273160.1 | -0.844597731 | -1.884849643 | 0.05945014 |
| GSTT1 | ENST00000610185.1 | ENSG00000273355.1 | -0.888312701 | -1.995578033 | 0.045979873 |
| GSTT1 | NR_027401.2 | FAM223A | 0.846760174 | 1.910400504 | 0.056081666 |
| GSTT1 | NR_028325.1 | LOC100132062 | 0.902945352 | 2.014420601 | 0.043965393 |
| GSTT1 | NR_046578.1 | CACNA1C-AS4 | 0.808828537 | 1.790708776 | 0.073340041 |
| GSTT1 | NR_103857.1 | SP2-AS1 | -0.951470794 | -2.134644989 | 0.03279002 |
| GSTT1 | NR_104158.1 | NRG1-IT1 | 0.865132532 | 1.94365237 | 0.051937386 |
| GSTT1 | NR_110318.1 | MACROD2-AS1 | 0.821146316 | 1.837124075 | 0.066191581 |
| GSTT1 | NR_110635.1 | LINC00687 | 0.910202499 | 2.041190952 | 0.041231851 |
| GSTT1 | NR_117097.1 | LINC01353 | 0.810705525 | 1.844355176 | 0.065131395 |
| GSTT1 | NR_117098.1 | LINC01353 | 0.854384501 | 1.929105699 | 0.053717742 |
| GSTT1 | NR_120527.1 | LOC100506675 | 0.809760865 | 1.79956789 | 0.071928895 |
| GSTT1 | NR_121661.1 | ZBTB20-AS5 | -0.962072156 | -2.162834223 | 0.030553932 |
| GSTT1 | NR_135644.1 | LOC105371506 | -0.813371107 | -1.833721952 | 0.066695278 |
| GSTT1 | NR_135820.1 | LOC102723727 | 0.964938494 | 2.120200038 | 0.033989179 |
| GSTT2 | ENST00000318291.4 | ENSG00000177406.4 | 0.844900392 | 1.873138836 | 0.061049216 |
| GSTT2 | ENST00000411694.1 | ENSG00000225331.1 | 0.810352552 | 1.816511748 | 0.069291907 |
| GSTT2 | ENST00000412759.1 | ENSG00000236933.1 | 0.898152203 | 2.011777996 | 0.044243344 |
| GSTT2 | ENST00000415205.1 | ENSG00000182057.4 | 0.930976415 | 2.093658067 | 0.036290451 |
| GSTT2 | ENST00000417260.1 | ENSG00000231734.4 | -0.879151766 | -1.954403951 | 0.050653464 |
| GSTT2 | ENST00000419662.1 | ENSG00000228265.1 | 0.893903397 | 1.973249531 | 0.04846714 |

| | | | | | |
|---|---|---|---|---|---|
| GSTT2 | ENST00000422763.1 | ENSG00000231131.2 | -0.800316275 | -1.801414814 | 0.071637523 |
| GSTT2 | ENST00000423428.1 | ENSG00000224048.1 | -0.847334906 | -1.868972203 | 0.061626682 |
| GSTT2 | ENST00000426237.2 | ENSG00000235527.2 | 0.859521917 | 1.884429092 | 0.059506958 |
| GSTT2 | ENST00000426302.1 | ENSG00000230454.1 | 0.834329652 | 1.891238233 | 0.05859255 |
| GSTT2 | ENST00000426475.1 | ENSG00000239467.1 | 0.81945661 | 1.822302413 | 0.068409112 |
| GSTT2 | ENST00000426519.1 | ENSG00000234142.1 | 0.806012173 | 1.804847318 | 0.071098578 |
| GSTT2 | ENST00000428765.1 | ENSG00000230107.1 | 0.813759281 | 1.823773616 | 0.068186304 |
| GSTT2 | ENST00000429080.1 | ENSG00000233047.1 | -0.893922726 | -2.000395731 | 0.045457549 |
| GSTT2 | ENST00000430920.1 | ENSG00000234203.1 | 0.934997034 | 2.095544104 | 0.036122652 |
| GSTT2 | ENST00000431730.1 | ENSG00000237401.2 | 0.821228979 | 1.841812058 | 0.065502646 |
| GSTT2 | ENST00000433051.1 | ENSG00000233193.1 | 0.801041907 | 1.783025971 | 0.074582076 |
| GSTT2 | ENST00000433905.2 | ENSG00000229299.2 | 0.930352384 | 2.079615887 | 0.037560779 |
| GSTT2 | ENST00000435287.1 | ENSG00000227220.1 | 0.822286774 | 1.82432539 | 0.068102894 |
| GSTT2 | ENST00000438190.1 | ENSG00000227214.2 | 0.942477026 | 2.098911327 | 0.035824718 |
| GSTT2 | ENST00000440595.1 | ENSG00000228265.1 | 0.851376413 | 1.914712429 | 0.055529188 |
| GSTT2 | ENST00000441592.2 | ENSG00000224078.8 | 0.875931039 | 1.947466583 | 0.051478822 |
| GSTT2 | ENST00000442829.1 | ENSG00000225284.1 | 0.862533592 | 1.921986023 | 0.054607516 |
| GSTT2 | ENST00000446562.1 | ENSG00000233896.1 | 0.83435811 | 1.859513779 | 0.062954348 |
| GSTT2 | ENST00000447343.2 | ENSG00000229299.2 | 0.902193294 | 2.039406513 | 0.041409473 |
| GSTT2 | ENST00000448858.1 | ENSG00000237734.1 | -0.86801145 | -1.934704191 | 0.053026606 |
| GSTT2 | ENST00000451507.1 | ENSG00000229539.1 | 0.882547093 | 1.96453911 | 0.049467605 |
| GSTT2 | ENST00000452176.1 | ENSG00000223659.1 | -0.888750756 | -1.98912434 | 0.04668748 |
| GSTT2 | ENST00000453051.1 | ENSG00000229407.1 | 0.871354047 | 1.979801685 | 0.047725817 |
| GSTT2 | ENST00000458154.1 | ENSG00000235578.1 | 0.867379262 | 1.954812517 | 0.050605203 |
| GSTT2 | ENST00000458194.1 | ENSG00000226193.1 | 0.822359616 | 1.858133911 | 0.06315 |
| GSTT2 | ENST00000463255.1 | ENSG00000243305.1 | -0.871880117 | -1.92698242 | 0.053981821 |
| GSTT2 | ENST00000468165.1 | ENSG00000239480.1 | 0.926458182 | 2.076885405 | 0.037812137 |
| GSTT2 | ENST00000489077.1 | ENSG00000244198.1 | 0.978627358 | 2.172970867 | 0.02978251 |
| GSTT2 | ENST00000489090.1 | ENSG00000240045.1 | -0.803276936 | -1.814513374 | 0.069598726 |
| GSTT2 | ENST00000498693.1 | ENSG00000244198.1 | 0.925094304 | 2.072953484 | 0.038176609 |
| GSTT2 | ENST00000503723.1 | ENSG00000250472.1 | -0.981956545 | -2.189851105 | 0.028535037 |
| GSTT2 | ENST00000506723.2 | ENSG00000249484.4 | -0.831665593 | -1.842218943 | 0.065443131 |
| GSTT2 | ENST00000506791.1 | ENSG00000251131.1 | 0.913980056 | 2.02178842 | 0.043198215 |
| GSTT2 | ENST00000509036.1 | ENSG00000251131.1 | 0.919737406 | 2.053378325 | 0.0400359 |
| GSTT2 | ENST00000509192.1 | ENSG00000250765.1 | 0.92037385 | 2.055760244 | 0.039805632 |
| GSTT2 | ENST00000509453.1 | ENSG00000249145.1 | 0.879603764 | 1.95209837 | 0.050926527 |
| GSTT2 | ENST00000510570.1 | ENSG00000250438.1 | -0.866460349 | -1.939582031 | 0.052430506 |
| GSTT2 | ENST00000515128.1 | ENSG00000248215.1 | -0.869598588 | -1.939812078 | 0.052402532 |
| GSTT2 | ENST00000517846.1 | ENSG00000254485.1 | 0.888488083 | 2.034826086 | 0.041868375 |
| GSTT2 | ENST00000520603.1 | ENSG00000254001.1 | -0.932081132 | -2.071073074 | 0.038351968 |
| GSTT2 | ENST00000521653.1 | ENSG00000253301.1 | 0.922678883 | 2.069680723 | 0.038482253 |
| GSTT2 | ENST00000527086.1 | ENSG00000255182.1 | 0.809785379 | 1.788330235 | 0.073722745 |
| GSTT2 | ENST00000529247.1 | ENSG00000254741.1 | 0.883764275 | 1.970325271 | 0.048801104 |
| GSTT2 | ENST00000537850.1 | ENSG00000251002.3 | 0.825138147 | 1.878323871 | 0.060336873 |
| GSTT2 | ENST00000543072.1 | ENSG00000256092.2 | -0.824688135 | -1.83626507 | 0.066318463 |
| GSTT2 | ENST00000543275.1 | ENSG00000256944.1 | 0.906407438 | 2.015831088 | 0.043817642 |
| GSTT2 | ENST00000543403.1 | ENSG00000256684.1 | -0.847467995 | -1.89270927 | 0.058396543 |
| GSTT2 | ENST00000543494.1 | ENSG00000256514.1 | 0.810156346 | 1.821899595 | 0.068470222 |
| GSTT2 | ENST00000545177.3 | ENSG00000230438.5 | 0.845908885 | 1.874016955 | 0.060928088 |
| GSTT2 | ENST00000549806.1 | ENSG00000257252.1 | 0.908059616 | 2.039827033 | 0.041367556 |
| GSTT2 | ENST00000550263.1 | ENSG00000257605.1 | 0.828224121 | 1.84381107 | 0.065210679 |

| | | | | | |
|---|---|---|---|---|---|
| GSTT2 | ENST00000558575.1 | ENSG00000259687.1 | 0.868643988 | 1.930835743 | 0.053503369 |
| GSTT2 | ENST00000562191.1 | ENSG00000261292.1 | -0.859783612 | -1.928028944 | 0.053851526 |
| GSTT2 | ENST00000563018.1 | ENSG00000260193.1 | 0.833893378 | 1.871415609 | 0.061287497 |
| GSTT2 | ENST00000563610.1 | ENSG00000260051.1 | 0.863751465 | 1.938747342 | 0.052532111 |
| GSTT2 | ENST00000563611.1 | ENSG00000261583.1 | 0.980502324 | 2.198769569 | 0.027894311 |
| GSTT2 | ENST00000564809.1 | ENSG00000261471.1 | 0.923048879 | 2.066445436 | 0.03878644 |
| GSTT2 | ENST00000565735.1 | ENSG00000261213.1 | -0.830315874 | -1.87301949 | 0.061065694 |
| GSTT2 | ENST00000569981.1 | ENSG00000238045.5 | 0.803994248 | 1.79634469 | 0.072439714 |
| GSTT2 | ENST00000570493.2 | ENSG00000261898.2 | 0.847844805 | 1.913488918 | 0.055685491 |
| GSTT2 | ENST00000570843.1 | ENSG00000261889.1 | 0.808905102 | 1.821671334 | 0.06850487 |
| GSTT2 | ENST00000577853.1 | ENSG00000264207.1 | 0.887583394 | 1.992515859 | 0.046314487 |
| GSTT2 | ENST00000578800.1 | ENSG00000264235.1 | 0.907284251 | 2.017902949 | 0.04360137 |
| GSTT2 | ENST00000579775.1 | ENSG00000264108.1 | 0.82803842 | 1.865538827 | 0.062105915 |
| GSTT2 | ENST00000580622.1 | ENSG00000264634.1 | 0.881012268 | 1.991557359 | 0.046419646 |
| GSTT2 | ENST00000582044.1 | ENSG00000263715.2 | 0.966406323 | 2.156980109 | 0.031007214 |
| GSTT2 | ENST00000582558.1 | ENSG00000264569.1 | 0.863819593 | 1.94719471 | 0.051511396 |
| GSTT2 | ENST00000586694.1 | ENSG00000267141.1 | 0.805090001 | 1.82174376 | 0.068493875 |
| GSTT2 | ENST00000588182.2 | ENSG00000267453.2 | 0.808134705 | 1.817863953 | 0.069084929 |
| GSTT2 | ENST00000588380.1 | ENSG00000266990.1 | 0.90829165 | 2.018434128 | 0.043546068 |
| GSTT2 | ENST00000588402.1 | ENSG00000267006.1 | -0.873770332 | -1.954122883 | 0.050686686 |
| GSTT2 | ENST00000589395.1 | ENSG00000267143.1 | 0.824142555 | 1.843793995 | 0.065213168 |
| GSTT2 | ENST00000591174.1 | ENSG00000267289.1 | 0.914535348 | 2.09066692 | 0.036557932 |
| GSTT2 | ENST00000592400.1 | ENSG00000267735.1 | 0.818141573 | 1.832936211 | 0.066812058 |
| GSTT2 | ENST00000592525.1 | ENSG00000267214.1 | 0.905910437 | 2.018521647 | 0.043536962 |
| GSTT2 | ENST00000593139.1 | ENSG00000267042.1 | 0.898899401 | 1.988147203 | 0.046795412 |
| GSTT2 | ENST00000594590.2 | ENSG00000268199.2 | 0.849409344 | 1.906448352 | 0.056592061 |
| GSTT2 | ENST00000594776.1 | ENSG00000269807.1 | 0.801190402 | 1.776589564 | 0.075635794 |
| GSTT2 | ENST00000594850.1 | ENSG00000268093.1 | 0.84122265 | 1.84332686 | 0.065281302 |
| GSTT2 | ENST00000595478.1 | ENSG00000237031.3 | -0.822644402 | -1.838229529 | 0.06602859 |
| GSTT2 | ENST00000596091.1 | ENSG00000227733.4 | -0.891114644 | -2.020446691 | 0.043337074 |
| GSTT2 | ENST00000596887.1 | ENSG00000237031.3 | -0.938849714 | -2.124892379 | 0.033595594 |
| GSTT2 | ENST00000597169.1 | ENSG00000269720.1 | 0.862197773 | 1.937709835 | 0.052658633 |
| GSTT2 | ENST00000597256.1 | ENSG00000267986.1 | 0.905490319 | 1.988694482 | 0.046734935 |
| GSTT2 | ENST00000598092.1 | ENSG00000228065.6 | -0.893805557 | -2.005536638 | 0.044905708 |
| GSTT2 | ENST00000600242.1 | ENSG00000269583.1 | 0.901100189 | 2.002685167 | 0.045211092 |
| GSTT2 | ENST00000600726.1 | ENSG00000267858.1 | 0.94413475 | 2.08844458 | 0.03675775 |
| GSTT2 | ENST00000601033.1 | ENSG00000268401.1 | 0.820279391 | 1.838924793 | 0.065926249 |
| GSTT2 | ENST00000604142.1 | ENSG00000271308.1 | 0.972273949 | 2.167524419 | 0.03019489 |
| GSTT2 | ENST00000604183.1 | ENSG00000271185.1 | 0.962688447 | 2.16197178 | 0.030620351 |
| GSTT2 | ENST00000606277.1 | ENSG00000272145.1 | 0.859877609 | 1.937173628 | 0.052724123 |
| GSTT2 | ENST00000606441.1 | ENSG00000272277.1 | 0.832990284 | 1.849961561 | 0.06431909 |
| GSTT2 | ENST00000606743.1 | ENSG00000272221.1 | 0.852956651 | 1.93053633 | 0.053540418 |
| GSTT2 | ENST00000606909.1 | ENSG00000271821.1 | 0.917477542 | 2.037138843 | 0.04163613 |
| GSTT2 | ENST00000607224.1 | ENSG00000272521.1 | 0.852811561 | 1.89327216 | 0.058321685 |
| GSTT2 | ENST00000607476.1 | ENSG00000272540.1 | 0.98442782 | 2.206469983 | 0.02735111 |
| GSTT2 | ENST00000607943.1 | ENSG00000273188.1 | 0.984132574 | 2.252689427 | 0.024278738 |
| GSTT2 | ENST00000608367.1 | ENSG00000273361.1 | 0.896645792 | 1.986601925 | 0.046966528 |
| GSTT2 | ENST00000608489.1 | ENSG00000272716.1 | 0.898970621 | 2.003817376 | 0.045089627 |
| GSTT2 | ENST00000608677.1 | ENSG00000273350.1 | 0.868832907 | 1.9270052 | 0.053978982 |
| GSTT2 | ENST00000609113.1 | ENSG00000272827.1 | 0.85823561 | 1.935864729 | 0.052884271 |
| GSTT2 | NR_003604.2 | ZFAS1 | 0.850936254 | 1.891322181 | 0.05858135 |

| | | | | | |
|---|---|---|---|---|---|
| GSTT2 | NR_003605.1 | ZFAS1 | 0.86509083 | 1.94275063 | 0.052046296 |
| GSTT2 | NR_003606.2 | ZFAS1 | 0.842328821 | 1.879237211 | 0.060212111 |
| GSTT2 | NR_026802.1 | FAM74A4 | 0.922279966 | 2.050414551 | 0.040323994 |
| GSTT2 | NR_026813.1 | LINC00597 | -0.81702406 | -1.805470611 | 0.07100107 |
| GSTT2 | NR_026951.1 | LINC00324 | 0.884335258 | 1.957886336 | 0.050243352 |
| GSTT2 | NR_027052.1 | THAP7-AS1 | 0.937792388 | 2.088226917 | 0.03677737 |
| GSTT2 | NR_027271.1 | CIRBP-AS1 | 0.89894563 | 2.014610848 | 0.04394544 |
| GSTT2 | NR_036480.1 | VPS9D1-AS1 | 0.861117886 | 1.921639029 | 0.054651194 |
| GSTT2 | NR_036658.1 | ZFAS1 | 0.846274034 | 1.892562875 | 0.058416025 |
| GSTT2 | NR_037169.1 | LOC100507547 | 0.915670743 | 2.044377342 | 0.040916284 |
| GSTT2 | NR_037170.1 | LOC100507547 | 0.877987317 | 1.969458279 | 0.048900489 |
| GSTT2 | NR_038421.1 | LINC01220 | 0.963405181 | 2.176756025 | 0.029498776 |
| GSTT2 | NR_038923.1 | SSSCA1-AS1 | 0.969777998 | 2.162152778 | 0.030606402 |
| GSTT2 | NR_045114.1 | PVRL3-AS1 | -0.91719455 | -2.04493975 | 0.040860798 |
| GSTT2 | NR_046871.1 | LINC00333 | -0.861545173 | -1.951979268 | 0.050940666 |
| GSTT2 | NR_047116.1 | HIF1A-AS1 | -0.815388926 | -1.849232379 | 0.064424265 |
| GSTT2 | NR_072981.1 | LINC00957 | 0.91643811 | 2.07616147 | 0.03787902 |
| GSTT2 | NR_072982.1 | LINC00957 | 0.91060924 | 2.025546846 | 0.042811243 |
| GSTT2 | NR_105010.1 | LINC01333 | 0.882667304 | 1.991172338 | 0.046461944 |
| GSTT2 | NR_108106.1 | LINC01135 | 0.857920128 | 1.932804219 | 0.05326032 |
| GSTT2 | NR_109831.1 | RASSF1-AS1 | 0.869300508 | 1.953153787 | 0.050801375 |
| GSTT2 | NR_109885.1 | RALY-AS1 | 0.866981281 | 1.932574242 | 0.053288668 |
| GSTT2 | NR_109886.1 | RALY-AS1 | 0.899317381 | 2.015383746 | 0.043864456 |
| GSTT2 | NR_110245.1 | LOC101929282 | -0.929261858 | -2.077295371 | 0.037774306 |
| GSTT2 | NR_110556.1 | LOC102724890 | -0.866780469 | -1.931237327 | 0.05345371 |
| GSTT2 | NR_110630.1 | LOC101927478 | 0.938577593 | 2.062163779 | 0.039192146 |
| GSTT2 | NR_110941.1 | MIR762HG | 0.921870396 | 2.057237812 | 0.039663356 |
| GSTT2 | NR_110998.1 | FAM74A4 | 0.922279966 | 2.068114571 | 0.038629251 |
| GSTT2 | NR_111951.1 | LINC00869 | 0.916371268 | 2.050715759 | 0.040294635 |
| GSTT2 | NR_111952.1 | LINC00869 | 0.918989719 | 2.028531027 | 0.04250608 |
| GSTT2 | NR_111953.1 | LINC00869 | 0.909302579 | 2.015776149 | 0.043823389 |
| GSTT2 | NR_125849.1 | LOC101928140 | -0.833651138 | -1.862802205 | 0.0624901 |
| GSTT2 | NR_125957.1 | LOC101928626 | -0.888750756 | -1.996381918 | 0.045892367 |
| GSTT2 | NR_126522.1 | EXOC3-AS1 | 0.929596952 | 2.075808726 | 0.037911645 |
| GSTT2 | NR_130143.1 | LOC104968399 | 0.897991922 | 2.013122197 | 0.044101776 |
| GSTT2 | NR_134520.1 | LOC727993 | 0.849437625 | 1.880681614 | 0.060015242 |
| GSTT2 | NR_135024.1 | LOC105369747 | 0.91994999 | 2.04195948 | 0.041155551 |
| GSTT2 | NR_135041.1 | LOC101927038 | 0.810962984 | 1.795241623 | 0.072615212 |
| GSTT2 | NR_135097.1 | LOC105369443 | -0.847467995 | -1.901614764 | 0.057221536 |
| GSTT2 | NR_135584.1 | LOC101927596 | 0.879000703 | 1.987576686 | 0.046858527 |
| GSTT2 | NR_136215.1 | VCAN-AS1 | -0.853337676 | -1.897775162 | 0.057725707 |
| GSTT2 | NR_144459.1 | ARSD-AS1 | 0.844971034 | 1.896552234 | 0.05788706 |
| GTF2H2C | ENST00000414098.2 | ENSG00000234428.2 | -0.8556295 | -1.912094273 | 0.055864103 |
| GTF2H2C | ENST00000416861.1 | ENSG00000227308.2 | 0.888909375 | 2.003308975 | 0.045144135 |
| GTF2H2C | ENST00000417260.1 | ENSG00000231734.4 | -0.892162506 | -1.997573767 | 0.04576289 |
| GTF2H2C | ENST00000418080.1 | ENSG00000224091.1 | 0.820019629 | 1.824841028 | 0.068025022 |
| GTF2H2C | ENST00000419103.1 | ENSG00000227014.1 | 0.90086227 | 2.01174167 | 0.044247176 |
| GTF2H2C | ENST00000422204.1 | ENSG00000238160.1 | 0.865112746 | 1.942036978 | 0.052132624 |
| GTF2H2C | ENST00000423428.1 | ENSG00000224048.1 | -0.866717546 | -1.907269512 | 0.056485696 |
| GTF2H2C | ENST00000425124.1 | ENSG00000232336.1 | 0.868481014 | 1.926343603 | 0.054061484 |
| GTF2H2C | ENST00000431730.1 | ENSG00000237401.2 | 0.828151278 | 1.840464721 | 0.065700039 |

| | | | | | |
|---|---|---|---|---|---|
| GTF2H2C | ENST00000433035.1 | ENSG00000230483.1 | 0.859487392 | 1.935772807 | 0.052895533 |
| GTF2H2C | ENST00000435287.1 | ENSG00000227220.1 | 0.821033896 | 1.824150074 | 0.068129387 |
| GTF2H2C | ENST00000439443.1 | ENSG00000236911.2 | 0.814149105 | 1.810446997 | 0.070226497 |
| GTF2H2C | ENST00000440492.1 | ENSG00000233975.1 | 0.835618744 | 1.86799238 | 0.061763133 |
| GTF2H2C | ENST00000441160.1 | ENSG00000228437.1 | -0.823790733 | -1.834306767 | 0.06660847 |
| GTF2H2C | ENST00000446816.1 | ENSG00000204685.5 | 0.836496645 | 1.885680246 | 0.059338056 |
| GTF2H2C | ENST00000451507.1 | ENSG00000229539.1 | 0.815037526 | 1.811025038 | 0.070136976 |
| GTF2H2C | ENST00000451648.1 | ENSG00000232803.1 | 0.828564815 | 1.864962994 | 0.062186591 |
| GTF2H2C | ENST00000454100.1 | ENSG00000236943.2 | 0.969855363 | 2.163307447 | 0.030517541 |
| GTF2H2C | ENST00000454182.1 | ENSG00000230379.1 | -0.800184841 | -1.793193982 | 0.072941912 |
| GTF2H2C | ENST00000457371.1 | ENSG00000237401.2 | 0.837788449 | 1.864329029 | 0.062275513 |
| GTF2H2C | ENST00000480919.1 | ENSG00000242474.1 | 0.950091264 | 2.110765686 | 0.034792457 |
| GTF2H2C | ENST00000487368.1 | ENSG00000273328.1 | 0.829046366 | 1.836309105 | 0.066311954 |
| GTF2H2C | ENST00000500496.2 | ENSG00000245479.2 | 0.858933565 | 1.925813591 | 0.054127654 |
| GTF2H2C | ENST00000507525.1 | ENSG00000250431.1 | -0.89481238 | -2.024862929 | 0.042881441 |
| GTF2H2C | ENST00000509453.1 | ENSG00000249145.1 | 0.901783759 | 2.03197452 | 0.042156235 |
| GTF2H2C | ENST00000510198.1 | ENSG00000248733.1 | -0.803861698 | -1.798078997 | 0.07216449 |
| GTF2H2C | ENST00000513836.1 | ENSG00000251266.1 | -0.802676072 | -1.789696824 | 0.073502663 |
| GTF2H2C | ENST00000518590.1 | ENSG00000253986.1 | 0.88416959 | 2.00541012 | 0.044919221 |
| GTF2H2C | ENST00000519062.1 | ENSG00000253658.1 | -0.903430475 | -2.048644889 | 0.040496852 |
| GTF2H2C | ENST00000523859.1 | ENSG00000251136.4 | 0.857806569 | 1.925692204 | 0.054142818 |
| GTF2H2C | ENST00000524286.1 | ENSG00000253658.1 | -0.940142015 | -2.055685934 | 0.039812799 |
| GTF2H2C | ENST00000532454.1 | ENSG00000255120.1 | 0.914979783 | 2.009680745 | 0.044464989 |
| GTF2H2C | ENST00000534178.1 | ENSG00000255120.1 | 0.827775716 | 1.854982792 | 0.063598684 |
| GTF2H2C | ENST00000537032.1 | ENSG00000255933.1 | 0.833296373 | 1.856970564 | 0.063315342 |
| GTF2H2C | ENST00000545572.1 | ENSG00000255680.1 | 0.918877797 | 2.076554743 | 0.037842674 |
| GTF2H2C | ENST00000550263.1 | ENSG00000257605.1 | 0.940691209 | 2.105796067 | 0.035222071 |
| GTF2H2C | ENST00000551699.1 | ENSG00000257467.1 | -0.908606981 | -2.039700203 | 0.041380195 |
| GTF2H2C | ENST00000554859.1 | ENSG00000259088.1 | 0.947671182 | 2.121846067 | 0.033850667 |
| GTF2H2C | ENST00000559959.1 | ENSG00000259396.1 | 0.989704445 | 2.22789671 | 0.025887404 |
| GTF2H2C | ENST00000560963.1 | ENSG00000259370.1 | 0.84527564 | 1.879874388 | 0.060125199 |
| GTF2H2C | ENST00000562995.1 | ENSG00000261253.1 | 0.854861758 | 1.917011753 | 0.055236439 |
| GTF2H2C | ENST00000568332.1 | ENSG00000260256.1 | 0.893396638 | 1.992955797 | 0.046266288 |
| GTF2H2C | ENST00000571660.1 | ENSG00000262848.1 | 0.828850568 | 1.855465665 | 0.063529758 |
| GTF2H2C | ENST00000582558.1 | ENSG00000264569.1 | 0.802513137 | 1.805085907 | 0.07106124 |
| GTF2H2C | ENST00000584705.1 | ENSG00000264569.1 | 0.87056884 | 1.955905717 | 0.050476262 |
| GTF2H2C | ENST00000585181.1 | ENSG00000265749.1 | -0.849293935 | -1.903299229 | 0.057001511 |
| GTF2H2C | ENST00000585810.1 | ENSG00000236172.2 | 0.812584435 | 1.822472969 | 0.068383252 |
| GTF2H2C | ENST00000587696.1 | ENSG00000225313.2 | 0.856891162 | 1.924545161 | 0.054286286 |
| GTF2H2C | ENST00000589395.1 | ENSG00000267143.1 | 0.892336133 | 1.988031402 | 0.046808217 |
| GTF2H2C | ENST00000590989.1 | ENSG00000267011.1 | 0.90375019 | 1.988459421 | 0.046760903 |
| GTF2H2C | ENST00000591414.1 | ENSG00000267011.1 | 0.878899368 | 1.979134696 | 0.047800843 |
| GTF2H2C | ENST00000592368.1 | ENSG00000267231.1 | 0.932656248 | 2.081763173 | 0.037364107 |
| GTF2H2C | ENST00000592720.1 | ENSG00000267232.1 | 0.916353777 | 2.020526528 | 0.043328801 |
| GTF2H2C | ENST00000592816.1 | ENSG00000236172.2 | 0.837477186 | 1.892215372 | 0.058462291 |
| GTF2H2C | ENST00000594091.1 | ENSG00000232732.5 | 0.872258964 | 1.946712393 | 0.051569225 |
| GTF2H2C | ENST00000596091.1 | ENSG00000227733.4 | -0.870438813 | -1.942799459 | 0.052040393 |
| GTF2H2C | ENST00000597530.1 | ENSG00000228401.3 | 0.832111201 | 1.830391716 | 0.067191386 |
| GTF2H2C | ENST00000597906.1 | ENSG00000268566.1 | -0.847674116 | -1.901455041 | 0.057242436 |
| GTF2H2C | ENST00000599387.1 | ENSG00000227733.4 | -0.835685985 | -1.864870091 | 0.062199616 |
| GTF2H2C | ENST00000602454.1 | ENSG00000270139.1 | -0.843078919 | -1.883513612 | 0.059630797 |

| | | | | | |
|---|---|---|---|---|---|
| GTF2H2C | ENST00000602471.1 | ENSG00000270107.1 | 0.87537763 | 1.95956445 | 0.05004672 |
| GTF2H2C | ENST00000602949.1 | ENSG00000270030.1 | 0.914983728 | 2.046947297 | 0.040663259 |
| GTF2H2C | ENST00000605082.1 | ENSG00000270426.1 | 0.970093537 | 2.178528499 | 0.029366713 |
| GTF2H2C | ENST00000606482.1 | ENSG00000272416.1 | -0.815663836 | -1.811254464 | 0.070101471 |
| GTF2H2C | ENST00000606743.1 | ENSG00000272221.1 | 0.930563632 | 2.083456967 | 0.03720959 |
| GTF2H2C | ENST00000606909.1 | ENSG00000271821.1 | 0.857957884 | 1.925843294 | 0.054123944 |
| GTF2H2C | ENST00000606942.1 | ENSG00000271835.1 | -0.843013178 | -1.899857785 | 0.057451785 |
| GTF2H2C | ENST00000607148.1 | ENSG00000272477.1 | -0.902445622 | -2.001810641 | 0.045305101 |
| GTF2H2C | ENST00000608258.1 | ENSG00000229042.2 | -0.90944222 | -2.022394035 | 0.043135661 |
| GTF2H2C | ENST00000608759.1 | ENSG00000273464.1 | -0.869101279 | -1.962442623 | 0.049710975 |
| GTF2H2C | ENST00000610008.1 | ENSG00000272711.1 | 0.898444788 | 2.017113435 | 0.043683677 |
| GTF2H2C | NR_026774.1 | LINC00239 | 0.872157331 | 1.960823087 | 0.049899664 |
| GTF2H2C | NR_028324.1 | LINC01002 | 0.929164019 | 2.086499207 | 0.036933427 |
| GTF2H2C | NR_031762.2 | HCP5B | 0.90401043 | 2.012177591 | 0.04420122 |
| GTF2H2C | NR_037169.1 | LOC100507547 | 0.868552426 | 1.943920651 | 0.051905021 |
| GTF2H2C | NR_037170.1 | LOC100507547 | 0.909023128 | 2.038515398 | 0.041498416 |
| GTF2H2C | NR_046369.1 | LOC100131626 | 0.8458718 | 1.884334064 | 0.059519802 |
| GTF2H2C | NR_072981.1 | LINC00957 | 0.828179825 | 1.859267012 | 0.0629893 |
| GTF2H2C | NR_072982.1 | LINC00957 | 0.83642706 | 1.863743823 | 0.062357688 |
| GTF2H2C | NR_108068.1 | LINC00836 | 0.863299311 | 1.94382098 | 0.051917043 |
| GTF2H2C | NR_108085.1 | OVOL1-AS1 | 0.88871395 | 1.981897203 | 0.047490747 |
| GTF2H2C | NR_109770.1 | TONSL-AS1 | 0.924666416 | 2.072926671 | 0.038179104 |
| GTF2H2C | NR_120371.1 | LINC01585 | 0.838064779 | 1.886438433 | 0.059235898 |
| GTF2H2C | NR_133941.1 | LOC105377247 | -0.802676072 | -1.785830035 | 0.074126782 |
| GTF2H2C | NR_134325.1 | LOC102723672 | 0.940996104 | 2.100942465 | 0.035646017 |
| GTF2H2C | NR_135132.1 | HSPC324 | 0.941474671 | 2.113257061 | 0.034578772 |
| HBA2 | ENST00000411694.1 | ENSG00000225331.1 | 0.880382965 | 1.955563301 | 0.05051662 |
| HBA2 | ENST00000412647.2 | ENSG00000232964.2 | -0.810657215 | -1.819056839 | 0.068902759 |
| HBA2 | ENST00000415205.1 | ENSG00000182057.4 | 0.811319331 | 1.806354974 | 0.07086291 |
| HBA2 | ENST00000416595.1 | ENSG00000223623.1 | 0.848648919 | 1.906858314 | 0.056538938 |
| HBA2 | ENST00000421019.1 | ENSG00000224397.1 | 0.843579279 | 1.888331503 | 0.058981461 |
| HBA2 | ENST00000425881.1 | ENSG00000239636.1 | 0.837671967 | 1.881303321 | 0.05993067 |
| HBA2 | ENST00000426030.2 | ENSG00000228686.2 | -0.835902052 | -1.868746474 | 0.061658095 |
| HBA2 | ENST00000426475.1 | ENSG00000239467.1 | 0.896555181 | 2.006708956 | 0.044780662 |
| HBA2 | ENST00000430920.1 | ENSG00000234203.1 | 0.8442466 | 1.888535962 | 0.058954035 |
| HBA2 | ENST00000431727.2 | ENSG00000234938.2 | 0.865360573 | 1.92095816 | 0.054736983 |
| HBA2 | ENST00000431730.1 | ENSG00000237401.2 | 0.814479839 | 1.8099464 | 0.0703041 |
| HBA2 | ENST00000434250.1 | ENSG00000234055.1 | 0.892390354 | 1.996090947 | 0.045924024 |
| HBA2 | ENST00000436132.1 | ENSG00000244125.1 | 0.848285748 | 1.910231169 | 0.056103456 |
| HBA2 | ENST00000439184.1 | ENSG00000233985.1 | -0.808358809 | -1.816342996 | 0.069317774 |
| HBA2 | ENST00000441592.2 | ENSG00000224078.8 | 0.911620164 | 2.057450724 | 0.03964289 |
| HBA2 | ENST00000442829.1 | ENSG00000225284.1 | 0.821447194 | 1.842156565 | 0.065452252 |
| HBA2 | ENST00000445278.1 | ENSG00000223410.1 | -0.858795763 | -1.886921658 | 0.059170864 |
| HBA2 | ENST00000446562.1 | ENSG00000233896.1 | 0.933575998 | 2.074793802 | 0.038005649 |
| HBA2 | ENST00000448674.1 | ENSG00000235119.1 | 0.848709649 | 1.870827062 | 0.061369055 |
| HBA2 | ENST00000451507.1 | ENSG00000229539.1 | 0.830335894 | 1.856942257 | 0.06331937 |
| HBA2 | ENST00000452553.1 | ENSG00000233973.1 | -0.831843371 | -1.883257764 | 0.059665444 |
| HBA2 | ENST00000457113.1 | ENSG00000227407.1 | 0.812444267 | 1.811352928 | 0.070086237 |
| HBA2 | ENST00000463255.1 | ENSG00000243305.1 | -0.925442652 | -2.056960415 | 0.039690034 |
| HBA2 | ENST00000466431.2 | ENSG00000254485.1 | 0.842155366 | 1.891741696 | 0.058525405 |
| HBA2 | ENST00000489090.1 | ENSG00000240045.1 | -0.919350483 | -2.046899853 | 0.040667919 |

| | | | | | |
|---|---|---|---|---|---|
| HBA2 | ENST00000504916.1 | ENSG00000248112.1 | -0.830226459 | -1.838395952 | 0.066004081 |
| HBA2 | ENST00000506394.1 | ENSG00000251665.1 | 0.903751695 | 2.01858677 | 0.043530187 |
| HBA2 | ENST00000508936.1 | ENSG00000250582.1 | -0.813243548 | -1.822875468 | 0.068322254 |
| HBA2 | ENST00000511631.1 | ENSG00000250402.1 | 0.816111066 | 1.810796023 | 0.070172432 |
| HBA2 | ENST00000517846.1 | ENSG00000254485.1 | 0.946573259 | 2.149303514 | 0.031610348 |
| HBA2 | ENST00000521653.1 | ENSG00000253301.1 | 0.825684605 | 1.827626193 | 0.067605672 |
| HBA2 | ENST00000527579.1 | ENSG00000254574.1 | 0.863401886 | 1.944378457 | 0.05184983 |
| HBA2 | ENST00000527757.1 | ENSG00000255109.1 | -0.872134266 | -1.972648084 | 0.048535671 |
| HBA2 | ENST00000529160.1 | ENSG00000246790.2 | 0.896108607 | 2.012499454 | 0.044167314 |
| HBA2 | ENST00000529247.1 | ENSG00000254741.1 | 0.954818751 | 2.144674899 | 0.031978849 |
| HBA2 | ENST00000543403.1 | ENSG00000256684.1 | -0.866329713 | -1.950053145 | 0.051169785 |
| HBA2 | ENST00000545177.3 | ENSG00000230438.5 | 0.804647712 | 1.785267462 | 0.074217944 |
| HBA2 | ENST00000545254.1 | ENSG00000256633.1 | 0.811523624 | 1.802853018 | 0.071411301 |
| HBA2 | ENST00000549806.1 | ENSG00000257252.1 | 0.94043193 | 2.100668693 | 0.035670059 |
| HBA2 | ENST00000552525.1 | ENSG00000257286.1 | 0.874279463 | 1.966808996 | 0.049205235 |
| HBA2 | ENST00000554431.1 | ENSG00000258616.1 | -0.800315386 | -1.780509331 | 0.074992644 |
| HBA2 | ENST00000558568.1 | ENSG00000272639.1 | 0.808683231 | 1.794528335 | 0.07272888 |
| HBA2 | ENST00000559673.1 | ENSG00000259604.1 | 0.865533622 | 1.958912031 | 0.05012309 |
| HBA2 | ENST00000562191.1 | ENSG00000261292.1 | -0.880882864 | -1.978408335 | 0.04788266 |
| HBA2 | ENST00000563806.1 | ENSG00000238045.5 | 0.844119227 | 1.899187395 | 0.057539842 |
| HBA2 | ENST00000564809.1 | ENSG00000261471.1 | 0.81240357 | 1.819437831 | 0.068844659 |
| HBA2 | ENST00000571775.1 | ENSG00000262456.1 | 0.849403061 | 1.89923793 | 0.0575332 |
| HBA2 | ENST00000578800.1 | ENSG00000264235.1 | 0.809136757 | 1.786397969 | 0.074034844 |
| HBA2 | ENST00000579154.1 | ENSG00000265908.1 | -0.883752233 | -1.964107298 | 0.04951765 |
| HBA2 | ENST00000580311.1 | ENSG00000266803.1 | -0.84586585 | -1.890290785 | 0.058719081 |
| HBA2 | ENST00000580622.1 | ENSG00000264634.1 | 0.857749042 | 1.904917322 | 0.056790819 |
| HBA2 | ENST00000582044.1 | ENSG00000263715.2 | 0.851423609 | 1.901449481 | 0.057243164 |
| HBA2 | ENST00000588182.2 | ENSG00000267453.2 | 0.846918338 | 1.878705 | 0.060284785 |
| HBA2 | ENST00000588290.1 | ENSG00000267751.1 | 0.807916825 | 1.784625022 | 0.07432216 |
| HBA2 | ENST00000588380.1 | ENSG00000266990.1 | 0.831184418 | 1.858473566 | 0.063101794 |
| HBA2 | ENST00000588384.1 | ENSG00000236172.2 | 0.864447004 | 1.951740148 | 0.050969063 |
| HBA2 | ENST00000588402.1 | ENSG00000267006.1 | -0.809301364 | -1.809298031 | 0.070404715 |
| HBA2 | ENST00000589380.1 | ENSG00000267488.1 | 0.852296166 | 1.890776284 | 0.058654214 |
| HBA2 | ENST00000592498.1 | ENSG00000267488.1 | 0.858929151 | 1.942829815 | 0.052036724 |
| HBA2 | ENST00000592518.1 | ENSG00000267786.1 | 0.803724938 | 1.777729394 | 0.07544831 |
| HBA2 | ENST00000592525.1 | ENSG00000267214.1 | 0.881839006 | 2.004404404 | 0.045026758 |
| HBA2 | ENST00000593038.1 | ENSG00000267133.1 | 0.86064915 | 1.929299978 | 0.053693633 |
| HBA2 | ENST00000593139.1 | ENSG00000267042.1 | 0.932590278 | 2.073085085 | 0.038164362 |
| HBA2 | ENST00000596887.1 | ENSG00000237031.3 | -0.838895234 | -1.889782077 | 0.058787112 |
| HBA2 | ENST00000597256.1 | ENSG00000267986.1 | 0.890084233 | 1.994526047 | 0.046094596 |
| HBA2 | ENST00000600242.1 | ENSG00000269583.1 | 0.920837162 | 2.077425332 | 0.03776232 |
| HBA2 | ENST00000602507.1 | ENSG00000270069.1 | -0.887298304 | -1.975021448 | 0.048265714 |
| HBA2 | ENST00000603948.1 | ENSG00000222041.6 | 0.807169492 | 1.795402423 | 0.072589607 |
| HBA2 | ENST00000606441.1 | ENSG00000272277.1 | 0.830943034 | 1.854606397 | 0.063652454 |
| HBA2 | ENST00000608159.1 | ENSG00000273093.1 | -0.822821571 | -1.851736029 | 0.064063738 |
| HBA2 | ENST00000608264.1 | ENSG00000273473.1 | 0.80008584 | 1.773205614 | 0.076194642 |
| HBA2 | ENST00000608367.1 | ENSG00000273361.1 | 0.925596387 | 2.071974432 | 0.038267826 |
| HBA2 | ENST00000608677.1 | ENSG00000273350.1 | 0.807933895 | 1.803393102 | 0.0713265 |
| HBA2 | ENST00000609612.1 | ENSG00000273424.1 | 0.824366518 | 1.848199429 | 0.064573498 |
| HBA2 | ENST00000609775.1 | ENSG00000273232.1 | -0.87454013 | -1.963309726 | 0.049610196 |
| HBA2 | NR_026813.1 | LINC00597 | -0.860129369 | -1.909870566 | 0.056149881 |

| | | | | | |
|---|---|---|---|---|---|
| HBA2 | NR_026951.1 | LINC00324 | 0.921761386 | 2.051607701 | 0.040207803 |
| HBA2 | NR_027052.1 | THAP7-AS1 | 0.824668956 | 1.831533113 | 0.06702101 |
| HBA2 | NR_038923.1 | SSSCA1-AS1 | 0.837180966 | 1.866021468 | 0.062038362 |
| HBA2 | NR_040049.1 | SDCBP2-AS1 | 0.86230806 | 1.934024665 | 0.053110095 |
| HBA2 | NR_046713.1 | NAALADL2-AS2 | -0.907830734 | -2.012920953 | 0.044122946 |
| HBA2 | NR_046762.1 | ALMS1-IT1 | -0.836166068 | -1.863807693 | 0.062348715 |
| HBA2 | NR_073155.1 | C1orf145 | -0.902750818 | -2.000045094 | 0.045495395 |
| HBA2 | NR_103841.1 | LINC00539 | 0.81267724 | 1.817186399 | 0.069188577 |
| HBA2 | NR_108046.1 | LINC00844 | -0.885541002 | -1.948516335 | 0.051353212 |
| HBA2 | NR_108077.1 | SMAD1-AS2 | -0.851723143 | -1.892698835 | 0.058397931 |
| HBA2 | NR_108106.1 | LINC01135 | 0.909164163 | 2.0476049 | 0.040598729 |
| HBA2 | NR_109831.1 | RASSF1-AS1 | 0.921364835 | 2.058332277 | 0.039558248 |
| HBA2 | NR_110803.1 | LOC101927018 | 0.883733734 | 1.990219062 | 0.046566809 |
| HBA2 | NR_111951.1 | LINC00869 | 0.824523542 | 1.843434967 | 0.065265529 |
| HBA2 | NR_111953.1 | LINC00869 | 0.827860402 | 1.878381109 | 0.060329048 |
| HBA2 | NR_121624.1 | LOC103352541 | -0.902036849 | -2.006073491 | 0.044848408 |
| HBA2 | NR_125849.1 | LOC101928140 | -0.831532492 | -1.854901688 | 0.063610267 |
| HBA2 | NR_126522.1 | EXOC3-AS1 | 0.828597715 | 1.864646588 | 0.062230958 |
| HBA2 | NR_134520.1 | LOC727993 | 0.940165903 | 2.117946991 | 0.034179557 |
| HBA2 | NR_134579.1 | LOC105372179 | 0.901089783 | 1.999981468 | 0.045502265 |
| HBA2 | NR_135024.1 | LOC105369747 | 0.890827438 | 1.99022875 | 0.046565743 |
| HBA2 | NR_135097.1 | LOC105369443 | -0.866329713 | -1.937937338 | 0.052630868 |
| HBA2 | NR_135626.1 | LOC100505585 | 0.839775214 | 1.898633649 | 0.057612661 |
| HBB | ENST00000420845.1 | ENSG00000232259.1 | 0.821505684 | 1.833536044 | 0.066722893 |
| HBB | ENST00000426653.1 | ENSG00000235704.1 | 0.841675563 | 1.891813899 | 0.058515781 |
| HBB | ENST00000429666.1 | ENSG00000233755.1 | 0.806719332 | 1.829431074 | 0.067335056 |
| HBB | ENST00000431727.2 | ENSG00000234938.2 | 0.800149798 | 1.764371481 | 0.077669456 |
| HBB | ENST00000436132.1 | ENSG00000244125.1 | 0.961383918 | 2.135854801 | 0.032691251 |
| HBB | ENST00000440194.1 | ENSG00000234753.1 | 0.826267972 | 1.830358457 | 0.067196355 |
| HBB | ENST00000443123.1 | ENSG00000229457.1 | 0.951283782 | 2.133320396 | 0.032898452 |
| HBB | ENST00000445278.1 | ENSG00000223410.1 | -0.835496585 | -1.87296168 | 0.061073677 |
| HBB | ENST00000500267.2 | ENSG00000246323.2 | 0.841486637 | 1.867710443 | 0.061802442 |
| HBB | ENST00000512563.1 | ENSG00000249547.1 | 0.809197228 | 1.811288806 | 0.070096157 |
| HBB | ENST00000519412.1 | ENSG00000253214.1 | -0.844631313 | -1.883013333 | 0.059698561 |
| HBB | ENST00000524824.1 | ENSG00000255328.1 | 0.893931087 | 1.988881929 | 0.046714237 |
| HBB | ENST00000536492.1 | ENSG00000256237.1 | -0.92157546 | -2.079485172 | 0.037572779 |
| HBB | ENST00000549303.1 | ENSG00000257180.1 | -0.949314503 | -2.150103067 | 0.031547063 |
| HBB | ENST00000551174.1 | ENSG00000257762.1 | -0.836089104 | -1.879822502 | 0.060132273 |
| HBB | ENST00000554197.1 | ENSG00000197176.3 | 0.844534583 | 1.879998264 | 0.060108315 |
| HBB | ENST00000557232.1 | ENSG00000259054.1 | 0.836480632 | 1.840555593 | 0.06568671 |
| HBB | ENST00000570919.1 | ENSG00000263321.1 | 0.822918849 | 1.857686391 | 0.063213562 |
| HBB | ENST00000572222.1 | ENSG00000261971.2 | -0.88166312 | -1.96045021 | 0.049943192 |
| HBB | ENST00000585703.1 | ENSG00000235779.3 | 0.836299116 | 1.879566395 | 0.060167197 |
| HBB | ENST00000588334.1 | ENSG00000235779.3 | 0.862953173 | 1.945042517 | 0.051769861 |
| HBB | ENST00000590255.1 | ENSG00000235779.3 | 0.826489953 | 1.853177736 | 0.063856888 |
| HBB | ENST00000593038.1 | ENSG00000267133.1 | 0.861962357 | 1.943751832 | 0.051925385 |
| HBB | ENST00000598735.1 | ENSG00000268093.1 | 0.84956751 | 1.921279998 | 0.054696417 |
| HBB | ENST00000599524.1 | ENSG00000268240.1 | 0.807665853 | 1.800507958 | 0.071780468 |
| HBB | ENST00000602773.1 | ENSG00000270160.1 | -0.848787376 | -1.916961014 | 0.055242885 |
| HBB | ENST00000603533.1 | ENSG00000271384.1 | 0.82302492 | 1.850607693 | 0.064226012 |
| HBB | ENST00000607598.1 | ENSG00000272267.1 | 0.829476488 | 1.837146274 | 0.066188304 |

| | | | | | |
|---|---|---|---|---|---|
| HBB | ENST00000609612.1 | ENSG00000273424.1 | 0.801473195 | 1.784909626 | 0.074275977 |
| HBB | ENST00000609837.1 | ENSG00000273106.1 | 0.927894921 | 2.077277267 | 0.037775976 |
| HBB | ENST00000609924.1 | ENSG00000272688.1 | -0.836176028 | -1.867462936 | 0.061836968 |
| HBB | NR_046713.1 | NAALADL2-AS2 | -0.900027988 | -2.003429991 | 0.045131155 |
| HBB | NR_046762.1 | ALMS1-IT1 | -0.836289202 | -1.885684713 | 0.059337454 |
| HBB | NR_108077.1 | SMAD1-AS2 | -0.804127157 | -1.808989653 | 0.070452611 |
| HBB | NR_125839.1 | LOC101927020 | 0.887939159 | 1.980469997 | 0.047650741 |
| HIST1H2AA | ENST00000412519.1 | ENSG00000227599.1 | 0.820235363 | 1.827446204 | 0.067632708 |
| HIST1H2AA | ENST00000412812.1 | ENSG00000225342.1 | -0.812514262 | -1.817132199 | 0.069196874 |
| HIST1H2AA | ENST00000413564.1 | ENSG00000224500.1 | 0.83113998 | 1.8652971 | 0.062139771 |
| HIST1H2AA | ENST00000414098.2 | ENSG00000234428.2 | -0.800747517 | -1.794248128 | 0.072773573 |
| HIST1H2AA | ENST00000416861.1 | ENSG00000227308.2 | 0.87287974 | 1.955789414 | 0.050489966 |
| HIST1H2AA | ENST00000418080.1 | ENSG00000224091.1 | 0.828918287 | 1.847841295 | 0.064625305 |
| HIST1H2AA | ENST00000422118.1 | ENSG00000231189.1 | 0.914601321 | 2.0313408 | 0.042220435 |
| HIST1H2AA | ENST00000422204.1 | ENSG00000238160.1 | 0.863854647 | 1.91650049 | 0.055301421 |
| HIST1H2AA | ENST00000424274.1 | ENSG00000232120.1 | -0.851010092 | -1.90551368 | 0.056713331 |
| HIST1H2AA | ENST00000424342.1 | ENSG00000234988.1 | 0.835877684 | 1.869986199 | 0.061485734 |
| HIST1H2AA | ENST00000424678.1 | ENSG00000229600.1 | -0.89555693 | -2.014097241 | 0.043999325 |
| HIST1H2AA | ENST00000426125.1 | ENSG00000223653.1 | -0.827025514 | -1.860010679 | 0.062884015 |
| HIST1H2AA | ENST00000426929.1 | ENSG00000230184.1 | 0.877106845 | 1.949305813 | 0.051258915 |
| HIST1H2AA | ENST00000429916.1 | ENSG00000227708.1 | -0.908642988 | -2.046900359 | 0.040667869 |
| HIST1H2AA | ENST00000430247.1 | ENSG00000232855.2 | -0.868665512 | -1.926592994 | 0.054030372 |
| HIST1H2AA | ENST00000433126.1 | ENSG00000232803.1 | 0.960014925 | 2.129996296 | 0.033171919 |
| HIST1H2AA | ENST00000435828.1 | ENSG00000235612.1 | -0.953617938 | -2.130365593 | 0.033141442 |
| HIST1H2AA | ENST00000441666.1 | ENSG00000230379.1 | -0.893331083 | -1.979679991 | 0.047739498 |
| HIST1H2AA | ENST00000441991.1 | ENSG00000231210.2 | 0.857458489 | 1.907956477 | 0.056396842 |
| HIST1H2AA | ENST00000442852.1 | ENSG00000237923.1 | 0.894488804 | 2.020377816 | 0.043344213 |
| HIST1H2AA | ENST00000443066.2 | ENSG00000237633.2 | -0.898038804 | -1.975152054 | 0.048250895 |
| HIST1H2AA | ENST00000443162.1 | ENSG00000234183.1 | -0.832461609 | -1.871175879 | 0.061320707 |
| HIST1H2AA | ENST00000445178.1 | ENSG00000234653.1 | -0.868198406 | -1.935342678 | 0.052948258 |
| HIST1H2AA | ENST00000445233.1 | ENSG00000233928.1 | -0.827302319 | -1.858180856 | 0.063143336 |
| HIST1H2AA | ENST00000445260.2 | ENSG00000231429.2 | -0.810374701 | -1.814022818 | 0.069674213 |
| HIST1H2AA | ENST00000447111.1 | ENSG00000231903.1 | -0.831389156 | -1.847258951 | 0.064709619 |
| HIST1H2AA | ENST00000449903.1 | ENSG00000223872.1 | 0.915818639 | 2.047563551 | 0.040602784 |
| HIST1H2AA | ENST00000453584.1 | ENSG00000233613.1 | 0.807323617 | 1.790499497 | 0.073373648 |
| HIST1H2AA | ENST00000454182.1 | ENSG00000230379.1 | -0.830224223 | -1.852251588 | 0.063989704 |
| HIST1H2AA | ENST00000454387.1 | ENSG00000223726.1 | 0.91664575 | 2.018288502 | 0.043561224 |
| HIST1H2AA | ENST00000456499.1 | ENSG00000237640.1 | 0.916195878 | 2.069615551 | 0.038488361 |
| HIST1H2AA | ENST00000456999.1 | ENSG00000230690.1 | -0.855726936 | -1.900773038 | 0.057331747 |
| HIST1H2AA | ENST00000458082.1 | ENSG00000231210.2 | 0.887477696 | 2.001414139 | 0.045347778 |
| HIST1H2AA | ENST00000477643.1 | ENSG00000241224.2 | 0.875809562 | 1.972808883 | 0.048517341 |
| HIST1H2AA | ENST00000479039.1 | ENSG00000241224.2 | 0.89419467 | 2.00217893 | 0.045265491 |
| HIST1H2AA | ENST00000480919.1 | ENSG00000242474.1 | 0.890641339 | 1.990926678 | 0.046488949 |
| HIST1H2AA | ENST00000500498.2 | ENSG00000245311.2 | 0.840438483 | 1.835014589 | 0.066503528 |
| HIST1H2AA | ENST00000503470.1 | ENSG00000248559.1 | -0.905076484 | -2.011570375 | 0.044265245 |
| HIST1H2AA | ENST00000503987.1 | ENSG00000250075.1 | -0.919816919 | -2.039644115 | 0.041385785 |
| HIST1H2AA | ENST00000505404.1 | ENSG00000249941.1 | 0.882363542 | 1.978007097 | 0.047927906 |
| HIST1H2AA | ENST00000506420.1 | ENSG00000250034.1 | 0.976388201 | 2.181112985 | 0.029175061 |
| HIST1H2AA | ENST00000508374.1 | ENSG00000249441.1 | -0.837967258 | -1.878480346 | 0.060315483 |
| HIST1H2AA | ENST00000508687.1 | ENSG00000250538.1 | -0.943396488 | -2.089535777 | 0.036659521 |
| HIST1H2AA | ENST00000510001.2 | ENSG00000249196.2 | 0.825673531 | 1.851273815 | 0.064130171 |

| | | | | | |
|---|---|---|---|---|---|
| HIST1H2AA | ENST00000510198.1 | ENSG00000248733.1 | -0.858909626 | -1.935465217 | 0.052933233 |
| HIST1H2AA | ENST00000510602.1 | ENSG00000249122.1 | 0.853553181 | 1.917605313 | 0.055161076 |
| HIST1H2AA | ENST00000514966.1 | ENSG00000251310.1 | 0.870945166 | 1.947553542 | 0.051468407 |
| HIST1H2AA | ENST00000519062.1 | ENSG00000253658.1 | -0.939216688 | -2.106230425 | 0.035184341 |
| HIST1H2AA | ENST00000519368.1 | ENSG00000253215.1 | -0.813764562 | -1.824607055 | 0.068060348 |
| HIST1H2AA | ENST00000522123.1 | ENSG00000253836.1 | -0.940333666 | -2.102638581 | 0.035497375 |
| HIST1H2AA | ENST00000527297.1 | ENSG00000255229.1 | 0.822361381 | 1.851861294 | 0.064045743 |
| HIST1H2AA | ENST00000532947.1 | ENSG00000255322.1 | -0.810739354 | -1.800674288 | 0.071754233 |
| HIST1H2AA | ENST00000533101.1 | ENSG00000255311.1 | -0.950753623 | -2.119543681 | 0.034044546 |
| HIST1H2AA | ENST00000536412.1 | ENSG00000256072.1 | 0.830259757 | 1.853246375 | 0.063847053 |
| HIST1H2AA | ENST00000537032.1 | ENSG00000255933.1 | 0.815343048 | 1.846200152 | 0.064863149 |
| HIST1H2AA | ENST00000537492.1 | ENSG00000256637.2 | -0.888979621 | -1.993884144 | 0.046164717 |
| HIST1H2AA | ENST00000545158.1 | ENSG00000256011.1 | -0.824370351 | -1.834758224 | 0.066541521 |
| HIST1H2AA | ENST00000545572.1 | ENSG00000255680.1 | 0.89825574 | 1.996268267 | 0.04590473 |
| HIST1H2AA | ENST00000546135.1 | ENSG00000256670.1 | 0.897762807 | 1.99450477 | 0.046096919 |
| HIST1H2AA | ENST00000550886.1 | ENSG00000257696.1 | -0.883026654 | -1.971141272 | 0.048707719 |
| HIST1H2AA | ENST00000551699.1 | ENSG00000257467.1 | -0.843534627 | -1.905742736 | 0.056683592 |
| HIST1H2AA | ENST00000553075.1 | ENSG00000257258.1 | -0.876969858 | -1.965333256 | 0.049375679 |
| HIST1H2AA | ENST00000554859.1 | ENSG00000259088.1 | 0.82182376 | 1.83037596 | 0.06719374 |
| HIST1H2AA | ENST00000556145.1 | ENSG00000258829.1 | 0.840043857 | 1.881956069 | 0.059841981 |
| HIST1H2AA | ENST00000559041.1 | ENSG00000259713.1 | -0.813181278 | -1.823498694 | 0.068227895 |
| HIST1H2AA | ENST00000560963.1 | ENSG00000259370.1 | 0.85953581 | 1.89970817 | 0.057471428 |
| HIST1H2AA | ENST00000561544.1 | ENSG00000261532.1 | 0.921822376 | 2.055245096 | 0.039855337 |
| HIST1H2AA | ENST00000561847.1 | ENSG00000260293.1 | 0.893812058 | 1.991813922 | 0.046391478 |
| HIST1H2AA | ENST00000565944.1 | ENSG00000260331.1 | -0.932336228 | -2.096392764 | 0.036047364 |
| HIST1H2AA | ENST00000566521.1 | ENSG00000261629.1 | -0.805270227 | -1.806300892 | 0.070871353 |
| HIST1H2AA | ENST00000569998.1 | ENSG00000260975.1 | 0.871255701 | 1.957150088 | 0.050329825 |
| HIST1H2AA | ENST00000570413.1 | ENSG00000263167.1 | 0.838602046 | 1.903055454 | 0.05703331 |
| HIST1H2AA | ENST00000570700.1 | ENSG00000263011.1 | 0.883668811 | 1.978995173 | 0.04781655 |
| HIST1H2AA | ENST00000570919.1 | ENSG00000263321.1 | -0.801436317 | -1.786001306 | 0.074099047 |
| HIST1H2AA | ENST00000580729.1 | ENSG00000266176.1 | 0.841277307 | 1.869713245 | 0.061523649 |
| HIST1H2AA | ENST00000582895.1 | ENSG00000264729.1 | 0.857519906 | 1.920453333 | 0.054800662 |
| HIST1H2AA | ENST00000585810.1 | ENSG00000236172.2 | 0.819598713 | 1.847697171 | 0.064646163 |
| HIST1H2AA | ENST00000585877.1 | ENSG00000267249.1 | 0.91025899 | 2.045729495 | 0.040782992 |
| HIST1H2AA | ENST00000590046.1 | ENSG00000266950.1 | 0.924495137 | 2.083462111 | 0.037209122 |
| HIST1H2AA | ENST00000591414.1 | ENSG00000267011.1 | 0.849847344 | 1.901238966 | 0.057270719 |
| HIST1H2AA | ENST00000592816.1 | ENSG00000236172.2 | 0.800540111 | 1.774125382 | 0.076042413 |
| HIST1H2AA | ENST00000594091.1 | ENSG00000232732.5 | 0.821903023 | 1.855847387 | 0.063475314 |
| HIST1H2AA | ENST00000596473.1 | ENSG00000268650.3 | -0.875732544 | -1.967058555 | 0.049176461 |
| HIST1H2AA | ENST00000597680.1 | ENSG00000269574.1 | -0.884915716 | -1.962650495 | 0.049686799 |
| HIST1H2AA | ENST00000597865.1 | ENSG00000268108.1 | -0.843992532 | -1.850001967 | 0.064313266 |
| HIST1H2AA | ENST00000599387.1 | ENSG00000227733.4 | -0.860007088 | -1.936998731 | 0.052745499 |
| HIST1H2AA | ENST00000600956.1 | ENSG00000232732.5 | 0.852079061 | 1.886210608 | 0.05926658 |
| HIST1H2AA | ENST00000602405.1 | ENSG00000269928.1 | 0.884994968 | 1.96439161 | 0.049484695 |
| HIST1H2AA | ENST00000602443.1 | ENSG00000270076.1 | -0.850058682 | -1.895509042 | 0.058024995 |
| HIST1H2AA | ENST00000602454.1 | ENSG00000270139.1 | -0.971222343 | -2.153498673 | 0.031279508 |
| HIST1H2AA | ENST00000602471.1 | ENSG00000270107.1 | 0.939772327 | 2.107298725 | 0.035091694 |
| HIST1H2AA | ENST00000603949.1 | ENSG00000270332.1 | -0.979747873 | -2.195627449 | 0.02811862 |
| HIST1H2AA | ENST00000604312.1 | ENSG00000270947.1 | -0.891628482 | -2.001267583 | 0.045363561 |
| HIST1H2AA | ENST00000606482.1 | ENSG00000272416.1 | -0.892015211 | -1.993402244 | 0.046217418 |
| HIST1H2AA | ENST00000606942.1 | ENSG00000271835.1 | -0.889038988 | -1.997332034 | 0.045789126 |

| | | | | | |
|---|---|---|---|---|---|
| HIST1H2AA | ENST00000607051.1 | ENSG00000271771.1 | -0.966624311 | -2.152167085 | 0.031384197 |
| HIST1H2AA | ENST00000607136.1 | ENSG00000267546.2 | 0.812347279 | 1.822815293 | 0.068331371 |
| HIST1H2AA | ENST00000607148.1 | ENSG00000272477.1 | -0.832389584 | -1.859863967 | 0.062904774 |
| HIST1H2AA | ENST00000608133.1 | ENSG00000273193.1 | -0.863837547 | -1.936567688 | 0.052798211 |
| HIST1H2AA | ENST00000609182.1 | ENSG00000273248.1 | 0.916003092 | 2.039702711 | 0.041379945 |
| HIST1H2AA | ENST00000609955.1 | ENSG00000273275.1 | -0.861731066 | -1.910112066 | 0.056118786 |
| HIST1H2AA | ENST00000610034.1 | ENSG00000272912.1 | -0.905109333 | -2.01574407 | 0.043826745 |
| HIST1H2AA | NR_002765.2 | ASAP1-IT1 | -0.850993908 | -1.884551867 | 0.059490366 |
| HIST1H2AA | NR_024470.1 | SLCO4A1-AS1 | 0.973752742 | 2.154770042 | 0.031179834 |
| HIST1H2AA | NR_027067.1 | LINC00114 | 0.853245825 | 1.928626425 | 0.053777256 |
| HIST1H2AA | NR_027401.2 | FAM223A | -0.821924627 | -1.814136166 | 0.069656765 |
| HIST1H2AA | NR_046556.1 | RBMS3-AS1 | -0.854074336 | -1.946861418 | 0.051551351 |
| HIST1H2AA | NR_102738.1 | LINC00911 | 0.85633302 | 1.919529543 | 0.054917351 |
| HIST1H2AA | NR_109975.1 | ARNTL2-AS1 | 0.808317295 | 1.824291602 | 0.068107999 |
| HIST1H2AA | NR_110007.1 | ADNP-AS1 | -0.845386347 | -1.911595683 | 0.055928074 |
| HIST1H2AA | NR_110284.1 | LOC101927907 | -0.868198406 | -1.946992206 | 0.051535669 |
| HIST1H2AA | NR_125769.1 | LINC01269 | 0.874712087 | 1.925989155 | 0.054105728 |
| HIST1H2AA | NR_126370.1 | GACAT1 | 0.966952392 | 2.154572414 | 0.03119531 |
| HIST1H2AA | NR_126409.1 | LINC00376 | -0.837244903 | -1.889118985 | 0.058875887 |
| HIST1H2AA | NR_133907.1 | HLA-DQB1-AS1 | -0.836376888 | -1.854779432 | 0.06362773 |
| HIST1H2AA | NR_134610.1 | LOC105375014 | 0.894149513 | 1.998603152 | 0.045651309 |
| HIST1H2AA | NR_134910.1 | LOC102725254 | -0.939549882 | -2.105132633 | 0.035279764 |
| HIST1H2AA | NR_135076.1 | LOC102723838 | -0.878908074 | -1.959043983 | 0.050107636 |
| HIST1H2AA | NR_135132.1 | HSPC324 | 0.823957929 | 1.850508911 | 0.064240235 |
| HIST1H2AA | NR_135251.1 | LOC101928143 | 0.849496816 | 1.905584339 | 0.056704156 |
| HIST1H2BA | ENST00000362684.1 | ENSG00000228549.2 | -0.831799458 | -1.847752079 | 0.064638216 |
| HIST1H2BA | ENST00000415448.1 | ENSG00000228329.1 | -0.830175508 | -1.864857836 | 0.062201334 |
| HIST1H2BA | ENST00000416002.1 | ENSG00000230233.1 | -0.924925497 | -2.076965744 | 0.037804721 |
| HIST1H2BA | ENST00000420828.1 | ENSG00000227718.1 | 0.88913186 | 1.977356547 | 0.048001342 |
| HIST1H2BA | ENST00000421252.2 | ENSG00000250258.1 | -0.82962883 | -1.873713751 | 0.06096989 |
| HIST1H2BA | ENST00000422914.1 | ENSG00000236120.2 | -0.872239303 | -1.941830809 | 0.052157586 |
| HIST1H2BA | ENST00000423193.1 | ENSG00000224239.1 | 0.978913215 | 2.181992615 | 0.029110078 |
| HIST1H2BA | ENST00000432743.1 | ENSG00000189229.10 | 0.909260496 | 2.013219747 | 0.044091517 |
| HIST1H2BA | ENST00000435315.2 | ENSG00000226751.2 | 0.817658279 | 1.81132427 | 0.070090671 |
| HIST1H2BA | ENST00000438409.1 | ENSG00000234174.1 | -0.888080281 | -1.977212418 | 0.048017625 |
| HIST1H2BA | ENST00000439529.1 | ENSG00000236526.1 | 0.896376843 | 2.008478019 | 0.04459252 |
| HIST1H2BA | ENST00000441809.2 | ENSG00000237445.2 | -0.822939873 | -1.835846661 | 0.066380338 |
| HIST1H2BA | ENST00000442579.1 | ENSG00000228719.1 | -0.811120261 | -1.838049882 | 0.066055055 |
| HIST1H2BA | ENST00000448431.1 | ENSG00000232548.1 | 0.8239626 | 1.851026029 | 0.064165809 |
| HIST1H2BA | ENST00000450304.1 | ENSG00000237886.1 | -0.845791224 | -1.895566807 | 0.05801735 |
| HIST1H2BA | ENST00000451267.1 | ENSG00000230410.1 | -0.920622597 | -2.042833383 | 0.041068935 |
| HIST1H2BA | ENST00000451828.1 | ENSG00000228549.2 | -0.93551674 | -2.069503216 | 0.03849889 |
| HIST1H2BA | ENST00000454965.1 | ENSG00000235435.1 | 0.923477884 | 2.047160037 | 0.040642374 |
| HIST1H2BA | ENST00000457113.1 | ENSG00000227407.1 | -0.844011593 | -1.887352615 | 0.059112914 |
| HIST1H2BA | ENST00000457856.1 | ENSG00000228549.2 | -0.936013954 | -2.103350318 | 0.035435158 |
| HIST1H2BA | ENST00000462300.1 | ENSG00000241912.1 | -0.951988301 | -2.143910794 | 0.032040035 |
| HIST1H2BA | ENST00000481334.1 | ENSG00000242440.1 | 0.871962662 | 1.945796881 | 0.051679144 |
| HIST1H2BA | ENST00000505978.1 | ENSG00000249982.1 | -0.811750209 | -1.82257039 | 0.068368484 |
| HIST1H2BA | ENST00000508825.1 | ENSG00000250775.1 | 0.870336884 | 1.941647863 | 0.052179744 |
| HIST1H2BA | ENST00000508925.2 | ENSG00000249196.2 | -0.944463793 | -2.102506849 | 0.0355089 |
| HIST1H2BA | ENST00000509098.1 | ENSG00000250863.1 | 0.913058345 | 2.055742081 | 0.039807383 |

| | | | | | |
|---|---|---|---|---|---|
| HIST1H2BA | ENST00000509718.1 | ENSG00000251132.1 | -0.831396027 | -1.857119073 | 0.063294215 |
| HIST1H2BA | ENST00000511917.1 | ENSG00000250062.1 | 0.927506464 | 2.05776947 | 0.039612268 |
| HIST1H2BA | ENST00000512036.1 | ENSG00000250993.1 | 0.853540947 | 1.89862596 | 0.057613673 |
| HIST1H2BA | ENST00000512882.2 | ENSG00000251575.2 | 0.841320012 | 1.890091924 | 0.058745667 |
| HIST1H2BA | ENST00000513179.1 | ENSG00000251580.1 | 0.84807261 | 1.922045054 | 0.054600089 |
| HIST1H2BA | ENST00000515205.1 | ENSG00000251580.1 | 0.810867288 | 1.811569815 | 0.070052693 |
| HIST1H2BA | ENST00000519005.1 | ENSG00000253507.1 | 0.84866072 | 1.912004656 | 0.055875597 |
| HIST1H2BA | ENST00000519695.1 | ENSG00000253507.1 | 0.875319504 | 1.967811212 | 0.049089764 |
| HIST1H2BA | ENST00000521725.1 | ENSG00000253396.1 | 0.800208315 | 1.79538965 | 0.07259164 |
| HIST1H2BA | ENST00000524275.1 | ENSG00000253507.1 | 0.863864509 | 1.93070384 | 0.053519688 |
| HIST1H2BA | ENST00000525097.1 | ENSG00000254530.1 | 0.908508917 | 2.044678195 | 0.040886595 |
| HIST1H2BA | ENST00000529266.1 | ENSG00000254468.1 | 0.964151116 | 2.183177991 | 0.029022705 |
| HIST1H2BA | ENST00000537149.1 | ENSG00000256862.1 | -0.801439249 | -1.781248605 | 0.074871847 |
| HIST1H2BA | ENST00000539313.1 | ENSG00000256588.1 | 0.948358377 | 2.1179439 | 0.034179819 |
| HIST1H2BA | ENST00000545163.1 | ENSG00000256862.1 | -0.89043969 | -1.991422147 | 0.046434496 |
| HIST1H2BA | ENST00000545357.1 | ENSG00000256862.1 | -0.818655466 | -1.839384478 | 0.065858655 |
| HIST1H2BA | ENST00000555636.1 | ENSG00000259072.1 | 0.870415479 | 1.945907917 | 0.051665802 |
| HIST1H2BA | ENST00000555689.1 | ENSG00000259049.1 | -0.801588921 | -1.767576521 | 0.077131726 |
| HIST1H2BA | ENST00000557817.1 | ENSG00000259176.1 | 0.806831316 | 1.816836189 | 0.0692422 |
| HIST1H2BA | ENST00000558237.1 | ENSG00000259684.1 | 0.812433826 | 1.816240643 | 0.069333466 |
| HIST1H2BA | ENST00000559026.1 | ENSG00000259732.1 | 0.924021643 | 2.085847919 | 0.036992401 |
| HIST1H2BA | ENST00000560268.1 | ENSG00000259287.1 | -0.930957188 | -2.081429333 | 0.037394626 |
| HIST1H2BA | ENST00000561039.1 | ENSG00000259536.1 | -0.810327838 | -1.793287995 | 0.072926886 |
| HIST1H2BA | ENST00000561254.1 | ENSG00000259554.1 | 0.833038779 | 1.871000171 | 0.061345057 |
| HIST1H2BA | ENST00000563408.1 | ENSG00000260733.1 | 0.885484547 | 1.977843697 | 0.047946342 |
| HIST1H2BA | ENST00000565441.1 | ENSG00000261013.1 | 0.924701234 | 2.052308132 | 0.040139726 |
| HIST1H2BA | ENST00000566390.1 | ENSG00000260213.1 | 0.821120755 | 1.819498947 | 0.068835343 |
| HIST1H2BA | ENST00000568414.1 | ENSG00000260986.1 | 0.818663173 | 1.816708646 | 0.069261737 |
| HIST1H2BA | ENST00000571775.1 | ENSG00000262456.1 | -0.812436535 | -1.806686713 | 0.070811141 |
| HIST1H2BA | ENST00000572193.1 | ENSG00000261872.1 | 0.990022978 | 2.239132049 | 0.025147325 |
| HIST1H2BA | ENST00000576632.1 | ENSG00000262172.1 | -0.846327801 | -1.873911347 | 0.060942645 |
| HIST1H2BA | ENST00000580085.1 | ENSG00000266490.1 | 0.859030533 | 1.930111986 | 0.053592964 |
| HIST1H2BA | ENST00000580184.1 | ENSG00000264914.1 | -0.934759111 | -2.070167368 | 0.038436674 |
| HIST1H2BA | ENST00000587702.1 | ENSG00000267378.1 | 0.843496758 | 1.874970723 | 0.060796751 |
| HIST1H2BA | ENST00000592523.1 | ENSG00000226994.3 | 0.821072192 | 1.827166883 | 0.067674681 |
| HIST1H2BA | ENST00000594589.1 | ENSG00000269321.1 | -0.80527082 | -1.821234253 | 0.068571256 |
| HIST1H2BA | ENST00000595737.1 | ENSG00000228065.6 | 0.902857439 | 2.053369567 | 0.040036749 |
| HIST1H2BA | ENST00000595972.1 | ENSG00000230333.2 | 0.850775987 | 1.92443073 | 0.054300616 |
| HIST1H2BA | ENST00000597550.1 | ENSG00000269051.1 | 0.802269646 | 1.781578903 | 0.074817927 |
| HIST1H2BA | ENST00000598950.1 | ENSG00000269736.1 | 0.900676498 | 2.007440178 | 0.044702814 |
| HIST1H2BA | ENST00000601420.1 | ENSG00000269560.1 | -0.940511211 | -2.095800033 | 0.036099933 |
| HIST1H2BA | ENST00000602598.1 | ENSG00000269944.1 | 0.830112726 | 1.856561995 | 0.063373495 |
| HIST1H2BA | ENST00000602835.1 | ENSG00000270096.1 | 0.872352975 | 1.978819554 | 0.047836326 |
| HIST1H2BA | ENST00000604464.1 | ENSG00000270462.1 | 0.80407724 | 1.792047204 | 0.073125405 |
| HIST1H2BA | ENST00000605021.1 | ENSG00000271401.1 | 0.843614693 | 1.872017259 | 0.061204216 |
| HIST1H2BA | ENST00000606885.1 | ENSG00000231698.2 | 0.950003752 | 2.132289012 | 0.032983095 |
| HIST1H2BA | ENST00000607135.1 | ENSG00000272112.1 | 0.893348657 | 1.997860016 | 0.045731839 |
| HIST1H2BA | ENST00000607594.1 | ENSG00000271766.1 | 0.839237653 | 1.85816045 | 0.063146233 |
| HIST1H2BA | ENST00000608465.1 | ENSG00000272758.1 | 0.807875811 | 1.822550219 | 0.068371541 |
| HIST1H2BA | ENST00000608509.1 | ENSG00000273245.1 | 0.919802878 | 2.04924296 | 0.040438363 |
| HIST1H2BA | ENST00000609428.1 | ENSG00000273096.1 | 0.949972423 | 2.130638569 | 0.03311893 |

| | | | | | |
|---|---|---|---|---|---|
| HIST1H2BA | NR_046845.1 | DNM3-IT1 | 0.84753879 | 1.915482659 | 0.055430979 |
| HIST1H2BA | NR_047115.1 | PPP2R2B-IT1 | 0.957994596 | 2.149413962 | 0.0316016 |
| HIST1H2BA | NR_102703.1 | MAGEA8-AS1 | 0.947914 | 2.092949431 | 0.036353669 |
| HIST1H2BA | NR_103830.1 | LINC00587 | 0.8821509 | 2.012594364 | 0.04415732 |
| HIST1H2BA | NR_110160.1 | LOC100996249 | -0.895106523 | -2.025796231 | 0.04278567 |
| HIST1H2BA | NR_110731.1 | LINC01232 | -0.900909976 | -2.030243076 | 0.042331837 |
| HIST1H2BA | NR_110879.1 | LOC101929064 | 0.911143836 | 2.02936573 | 0.042421053 |
| HIST1H2BA | NR_131963.1 | LVCAT5 | 0.963591256 | 2.149420137 | 0.031601111 |
| HIST1H2BA | NR_134566.1 | LOC105372695 | 0.962959321 | 2.152212916 | 0.031380589 |
| HIST1H2BA | NR_134664.1 | LOC105374366 | 0.814848862 | 1.818016851 | 0.069061557 |
| HIST1H2BA | NR_134665.1 | LOC105374366 | 0.806810106 | 1.795849678 | 0.072518428 |
| HIST1H2BF | ENST00000340585.6 | ENSG00000249429.1 | 0.830184979 | 1.865848299 | 0.062062593 |
| HIST1H2BF | ENST00000412519.1 | ENSG00000227599.1 | 0.916488304 | 2.065157376 | 0.038908112 |
| HIST1H2BF | ENST00000419296.1 | ENSG00000204588.5 | 0.839175992 | 1.865555764 | 0.062103544 |
| HIST1H2BF | ENST00000422807.1 | ENSG00000227683.1 | -0.844178532 | -1.894772969 | 0.058122486 |
| HIST1H2BF | ENST00000424257.1 | ENSG00000231626.1 | 0.868864279 | 1.933501907 | 0.053174398 |
| HIST1H2BF | ENST00000424852.1 | ENSG00000229891.1 | 0.928067252 | 2.059558436 | 0.039440773 |
| HIST1H2BF | ENST00000428391.1 | ENSG00000224691.1 | 0.939208972 | 2.075227649 | 0.037965441 |
| HIST1H2BF | ENST00000429681.1 | ENSG00000235236.1 | 0.918722519 | 2.064302056 | 0.038989086 |
| HIST1H2BF | ENST00000430025.1 | ENSG00000233508.1 | 0.856681907 | 1.924104547 | 0.05434148 |
| HIST1H2BF | ENST00000432699.1 | ENSG00000233334.2 | -0.856979279 | -1.921390154 | 0.054682539 |
| HIST1H2BF | ENST00000441991.1 | ENSG00000231210.2 | 0.844598926 | 1.875751875 | 0.060689359 |
| HIST1H2BF | ENST00000443066.2 | ENSG00000237633.2 | -0.86350243 | -1.932199217 | 0.053334922 |
| HIST1H2BF | ENST00000451697.1 | ENSG00000233823.1 | 0.877240581 | 1.960136936 | 0.049979787 |
| HIST1H2BF | ENST00000456999.1 | ENSG00000230690.1 | -0.813898265 | -1.814078356 | 0.069665663 |
| HIST1H2BF | ENST00000473329.1 | ENSG00000243849.1 | 0.90548527 | 2.026574485 | 0.042705948 |
| HIST1H2BF | ENST00000486285.1 | ENSG00000241818.1 | -0.830377035 | -1.867627462 | 0.061814016 |
| HIST1H2BF | ENST00000490375.1 | ENSG00000240032.1 | -0.86056873 | -1.931714651 | 0.053394735 |
| HIST1H2BF | ENST00000504765.1 | ENSG00000249638.1 | -0.819760512 | -1.822265874 | 0.068414654 |
| HIST1H2BF | ENST00000514737.1 | ENSG00000250597.1 | -0.945359358 | -2.100579614 | 0.035677885 |
| HIST1H2BF | ENST00000521403.1 | ENSG00000253603.1 | 0.964139812 | 2.163968627 | 0.030466757 |
| HIST1H2BF | ENST00000522123.1 | ENSG00000253836.1 | -0.832297574 | -1.847380478 | 0.064692016 |
| HIST1H2BF | ENST00000524808.1 | ENSG00000254812.1 | 0.883859952 | 1.997080213 | 0.04581647 |
| HIST1H2BF | ENST00000537492.1 | ENSG00000256637.2 | -0.852765246 | -1.902191547 | 0.057146117 |
| HIST1H2BF | ENST00000547547.1 | ENSG00000257241.1 | 0.82161895 | 1.852931723 | 0.063892145 |
| HIST1H2BF | ENST00000551361.1 | ENSG00000224078.8 | 0.808858224 | 1.826252854 | 0.067812183 |
| HIST1H2BF | ENST00000553075.1 | ENSG00000257258.1 | -0.859261161 | -1.932246555 | 0.053329082 |
| HIST1H2BF | ENST00000559041.1 | ENSG00000259713.1 | -0.886440318 | -1.985173022 | 0.047125225 |
| HIST1H2BF | ENST00000561039.1 | ENSG00000259536.1 | 0.883133444 | 1.986719924 | 0.046953443 |
| HIST1H2BF | ENST00000563449.2 | ENSG00000261613.2 | -0.831164331 | -1.856322259 | 0.063407638 |
| HIST1H2BF | ENST00000566390.1 | ENSG00000260213.1 | -0.875913792 | -1.953561536 | 0.050753093 |
| HIST1H2BF | ENST00000567261.1 | ENSG00000261320.1 | -0.825887228 | -1.826632397 | 0.067755059 |
| HIST1H2BF | ENST00000569998.1 | ENSG00000260975.1 | 0.857889899 | 1.915049371 | 0.055486208 |
| HIST1H2BF | ENST00000570022.1 | ENSG00000261399.1 | -0.956680028 | -2.146619094 | 0.031823619 |
| HIST1H2BF | ENST00000571404.1 | ENSG00000262370.1 | -0.917304806 | -2.021974678 | 0.043178968 |
| HIST1H2BF | ENST00000578443.1 | ENSG00000265204.1 | 0.832685133 | 1.833421623 | 0.066739894 |
| HIST1H2BF | ENST00000580729.1 | ENSG00000266176.1 | 0.817403356 | 1.839168289 | 0.065890437 |
| HIST1H2BF | ENST00000585877.1 | ENSG00000267249.1 | 0.817862469 | 1.832206053 | 0.066920727 |
| HIST1H2BF | ENST00000593218.1 | ENSG00000267421.2 | -0.814375703 | -1.812215427 | 0.069952916 |
| HIST1H2BF | ENST00000596473.1 | ENSG00000268650.3 | -0.880840647 | -1.963190314 | 0.049624064 |
| HIST1H2BF | ENST00000597357.1 | ENSG00000268309.1 | 0.853906958 | 1.901009866 | 0.05730072 |

| | | | | | |
|---|---|---|---|---|---|
| HIST1H2BF | ENST00000602051.1 | ENSG00000227877.2 | 0.859637413 | 1.962626135 | 0.049689632 |
| HIST1H2BF | ENST00000602741.1 | ENSG00000270061.1 | 0.879773732 | 1.950701569 | 0.051092557 |
| HIST1H2BF | ENST00000607600.1 | ENSG00000272114.1 | 0.868501542 | 1.931801321 | 0.053384033 |
| HIST1H2BF | ENST00000607991.1 | ENSG00000273076.1 | 0.91280399 | 2.038884686 | 0.041461538 |
| HIST1H2BF | ENST00000608934.1 | ENSG00000273063.1 | -0.845538805 | -1.903085088 | 0.057029443 |
| HIST1H2BF | ENST00000610034.1 | ENSG00000272912.1 | -0.887273581 | -1.980664305 | 0.047628932 |
| HIST1H2BF | NR_026713.1 | FAM182A | -0.83691016 | -1.871309715 | 0.061302165 |
| HIST1H2BF | NR_046783.1 | KCND3-IT1 | 0.890670466 | 1.972799494 | 0.048518411 |
| HIST1H2BF | NR_102738.1 | LINC00911 | 0.816594623 | 1.821540197 | 0.068524782 |
| HIST1H2BF | NR_110702.1 | SEMA3B-AS1 | 0.883402967 | 1.988490976 | 0.046757416 |
| HIST1H2BF | NR_120595.1 | LINC01315 | 0.894778649 | 2.00672893 | 0.044778534 |
| HIST1H2BF | NR_121577.1 | NALT1 | 0.943167299 | 2.127544975 | 0.033374829 |
| HIST1H2BF | NR_125759.1 | PKNOX2-AS1 | 0.827721677 | 1.867980634 | 0.06176477 |
| HIST1H2BF | NR_125774.1 | LINC01170 | -0.868260176 | -1.947523963 | 0.05147195 |
| HIST1H2BF | NR_131985.1 | CRAT8 | 0.929880906 | 2.075659702 | 0.037925435 |
| HIST1H2BF | NR_134910.1 | LOC102725254 | -0.875363816 | -1.944242877 | 0.05186617 |
| HIST1H2BF | NR_135251.1 | LOC101928143 | 0.873579513 | 1.951396504 | 0.051009897 |
| HIST1H4B | ENST00000295549.4 | ENSG00000163364.5 | 0.861448262 | 1.936767147 | 0.052773813 |
| HIST1H4B | ENST00000413969.1 | ENSG00000224189.2 | 0.929967688 | 2.095137673 | 0.036158756 |
| HIST1H4B | ENST00000416641.1 | ENSG00000226956.1 | -0.818084435 | -1.8415631 | 0.065539083 |
| HIST1H4B | ENST00000418621.1 | ENSG00000224731.1 | 0.816013047 | 1.798729496 | 0.072061481 |
| HIST1H4B | ENST00000420830.1 | ENSG00000231512.1 | 0.833827707 | 1.862203166 | 0.062574458 |
| HIST1H4B | ENST00000428769.1 | ENSG00000232738.1 | 0.840371934 | 1.895911373 | 0.057971765 |
| HIST1H4B | ENST00000433174.1 | ENSG00000162947.4 | 0.918696182 | 2.06850243 | 0.038592802 |
| HIST1H4B | ENST00000437680.1 | ENSG00000237133.1 | -0.828831229 | -1.862341371 | 0.062554988 |
| HIST1H4B | ENST00000438850.1 | ENSG00000267338.1 | 0.903918108 | 2.003651083 | 0.04510745 |
| HIST1H4B | ENST00000448001.1 | ENSG00000229639.1 | 0.813630932 | 1.817741558 | 0.069103643 |
| HIST1H4B | ENST00000449749.1 | ENSG00000230834.1 | 0.856940774 | 1.947273959 | 0.051501899 |
| HIST1H4B | ENST00000451556.2 | ENSG00000228386.2 | 0.809558851 | 1.812116203 | 0.069968243 |
| HIST1H4B | ENST00000452412.1 | ENSG00000233860.1 | 0.898051394 | 2.005584352 | 0.044900613 |
| HIST1H4B | ENST00000453579.1 | ENSG00000232529.1 | 0.862306771 | 1.927647211 | 0.053899022 |
| HIST1H4B | ENST00000454489.1 | ENSG00000231403.1 | 0.840689109 | 1.89266913 | 0.058401884 |
| HIST1H4B | ENST00000454928.1 | ENSG00000186148.7 | 0.899281674 | 1.986027383 | 0.047030283 |
| HIST1H4B | ENST00000469846.2 | ENSG00000206573.4 | -0.940899052 | -2.128269529 | 0.033314743 |
| HIST1H4B | ENST00000480904.2 | ENSG00000206573.4 | -0.904106088 | -2.00358891 | 0.045114115 |
| HIST1H4B | ENST00000487772.1 | ENSG00000241754.1 | 0.889659529 | 2.003718987 | 0.045100171 |
| HIST1H4B | ENST00000498199.1 | ENSG00000206573.4 | -0.903558335 | -2.031683116 | 0.042185746 |
| HIST1H4B | ENST00000501133.2 | ENSG00000246560.2 | 0.941305652 | 2.107097287 | 0.035109147 |
| HIST1H4B | ENST00000502684.1 | ENSG00000251670.1 | -0.931735 | -2.081180856 | 0.037417355 |
| HIST1H4B | ENST00000504755.1 | ENSG00000250252.1 | 0.87181275 | 1.945897757 | 0.051667022 |
| HIST1H4B | ENST00000507857.2 | ENSG00000251055.2 | 0.816230924 | 1.815638007 | 0.069425919 |
| HIST1H4B | ENST00000508081.1 | ENSG00000248254.1 | -0.818995257 | -1.819824102 | 0.068785796 |
| HIST1H4B | ENST00000508191.1 | ENSG00000250910.3 | 0.848384056 | 1.885278339 | 0.059392269 |
| HIST1H4B | ENST00000508414.1 | ENSG00000248173.1 | 0.876304984 | 1.949766 | 0.051204016 |
| HIST1H4B | ENST00000514270.1 | ENSG00000249295.1 | 0.867074637 | 1.934757828 | 0.05302002 |
| HIST1H4B | ENST00000521411.2 | ENSG00000253496.2 | 0.954261423 | 2.130754594 | 0.033109365 |
| HIST1H4B | ENST00000523935.1 | ENSG00000253567.1 | 0.883187367 | 1.956827099 | 0.0503678 |
| HIST1H4B | ENST00000527100.1 | ENSG00000255015.1 | 0.93167619 | 2.089859137 | 0.036630455 |
| HIST1H4B | ENST00000531157.1 | ENSG00000254754.1 | 0.965344608 | 2.160959941 | 0.030698434 |
| HIST1H4B | ENST00000535806.1 | ENSG00000255817.1 | 0.973834089 | 2.183667136 | 0.028986716 |
| HIST1H4B | ENST00000540024.1 | ENSG00000255693.1 | -0.898066414 | -1.995906765 | 0.045944072 |

| | | | | | |
|---|---|---|---|---|---|
| HIST1H4B | ENST00000541391.1 | ENSG00000256268.1 | 0.846064491 | 1.891627161 | 0.058540674 |
| HIST1H4B | ENST00000544663.1 | ENSG00000256281.1 | 0.855716963 | 1.922469758 | 0.054546675 |
| HIST1H4B | ENST00000552261.1 | ENSG00000257959.1 | 0.953517491 | 2.141899279 | 0.032201588 |
| HIST1H4B | ENST00000552558.1 | ENSG00000257947.1 | 0.841527698 | 1.862365784 | 0.062551549 |
| HIST1H4B | ENST00000553477.1 | ENSG00000259123.1 | 0.801963317 | 1.797459344 | 0.072262727 |
| HIST1H4B | ENST00000557903.1 | ENSG00000259182.1 | 0.823723529 | 1.823595425 | 0.068213259 |
| HIST1H4B | ENST00000562582.1 | ENSG00000259779.1 | 0.822446832 | 1.846434437 | 0.064829151 |
| HIST1H4B | ENST00000562834.1 | ENSG00000261116.1 | 0.817800219 | 1.827496152 | 0.067625204 |
| HIST1H4B | ENST00000563570.1 | ENSG00000259961.1 | -0.851274805 | -1.89956371 | 0.057490398 |
| HIST1H4B | ENST00000565055.1 | ENSG00000259912.1 | -0.806281254 | -1.797592769 | 0.072241565 |
| HIST1H4B | ENST00000567089.1 | ENSG00000261822.1 | 0.811598891 | 1.803013223 | 0.071386138 |
| HIST1H4B | ENST00000569849.1 | ENSG00000260640.1 | 0.851133187 | 1.902599778 | 0.057092788 |
| HIST1H4B | ENST00000573861.1 | ENSG00000263320.1 | 0.965769514 | 2.121542571 | 0.033876169 |
| HIST1H4B | ENST00000576021.1 | ENSG00000262413.1 | -0.810834986 | -1.792037523 | 0.073126956 |
| HIST1H4B | ENST00000578035.1 | ENSG00000266743.1 | -0.893563394 | -1.963694851 | 0.04956549 |
| HIST1H4B | ENST00000578334.1 | ENSG00000265148.1 | 0.88005403 | 1.960554992 | 0.049930957 |
| HIST1H4B | ENST00000578349.1 | ENSG00000263688.1 | 0.926750194 | 2.064453324 | 0.038974755 |
| HIST1H4B | ENST00000578572.1 | ENSG00000196295.7 | 0.963657083 | 2.168365327 | 0.030130902 |
| HIST1H4B | ENST00000580975.1 | ENSG00000266237.1 | 0.982478392 | 2.165363809 | 0.030359835 |
| HIST1H4B | ENST00000582348.1 | ENSG00000265148.1 | 0.902563007 | 2.008875049 | 0.044550387 |
| HIST1H4B | ENST00000583826.1 | ENSG00000265148.1 | 0.902521572 | 2.025671002 | 0.04279851 |
| HIST1H4B | ENST00000583841.1 | ENSG00000265148.1 | 0.979910436 | 2.222178037 | 0.026271276 |
| HIST1H4B | ENST00000586952.1 | ENSG00000226994.3 | 0.809464253 | 1.788168932 | 0.073748757 |
| HIST1H4B | ENST00000590357.1 | ENSG00000267175.1 | -0.983631452 | -2.22025461 | 0.026401489 |
| HIST1H4B | ENST00000593486.1 | ENSG00000250910.3 | 0.943539358 | 2.09603072 | 0.036079466 |
| HIST1H4B | ENST00000593599.1 | ENSG00000231898.4 | 0.931160508 | 2.067812785 | 0.038657631 |
| HIST1H4B | ENST00000596643.1 | ENSG00000269439.1 | 0.815915047 | 1.818551231 | 0.068979924 |
| HIST1H4B | ENST00000600365.1 | ENSG00000231898.4 | 0.8090968 | 1.802099845 | 0.071529698 |
| HIST1H4B | ENST00000601511.1 | ENSG00000244513.2 | 0.805544582 | 1.819749306 | 0.068797191 |
| HIST1H4B | ENST00000602736.1 | ENSG00000269976.1 | 0.838876059 | 1.889508348 | 0.058823745 |
| HIST1H4B | ENST00000605780.1 | ENSG00000270755.1 | -0.833708033 | -1.876214565 | 0.060625823 |
| HIST1H4B | ENST00000607025.1 | ENSG00000271973.1 | -0.853552833 | -1.93147549 | 0.053424278 |
| HIST1H4B | ENST00000607715.1 | ENSG00000271788.1 | 0.865732634 | 1.908807444 | 0.056286936 |
| HIST1H4B | ENST00000608856.1 | ENSG00000272600.1 | -0.815490423 | -1.79843878 | 0.072107502 |
| HIST1H4B | ENST00000609349.1 | ENSG00000272861.1 | 0.824865144 | 1.851356006 | 0.064118354 |
| HIST1H4B | NR_033914.1 | LINC00254 | 0.859911817 | 1.91077064 | 0.056034062 |
| HIST1H4B | NR_038194.1 | LINC00583 | 0.915254242 | 2.042890408 | 0.041063288 |
| HIST1H4B | NR_038954.1 | LL22NC01-81G9.3 | -0.841907694 | -1.899766164 | 0.057463813 |
| HIST1H4B | NR_040001.2 | LINC01116 | 0.902863714 | 2.029513489 | 0.042406016 |
| HIST1H4B | NR_104618.1 | LINC01017 | 0.864939836 | 1.933090112 | 0.053225098 |
| HIST1H4B | NR_104620.1 | LINC01672 | 0.853781366 | 1.887773941 | 0.059056305 |
| HIST1H4B | NR_104998.1 | LOC102467225 | 0.895395123 | 1.987391861 | 0.046878989 |
| HIST1H4B | NR_110123.1 | GRM7-AS3 | 0.880335128 | 1.973695884 | 0.048416333 |
| HIST1H4B | NR_120566.1 | LOC101928896 | 0.91417151 | 2.014271764 | 0.043981009 |
| HIST1H4B | NR_131186.1 | LOC105377348 | 0.932284463 | 2.10830054 | 0.035005001 |
| HIST1H4B | NR_134565.1 | LOC101928807 | -0.957307415 | -2.150852974 | 0.031487806 |
| HIST1H4B | NR_135274.1 | LOC105370619 | 0.893379677 | 2.009788907 | 0.044453535 |
| HIST1H4B | NR_135549.1 | LOC101929411 | 0.828492862 | 1.871486848 | 0.061277631 |
| HIST1H4B | NR_135840.1 | LOC105376114 | 0.862578526 | 1.939625082 | 0.05242527 |
| HIST1H4B | NR_138419.1 | ARHGEF9-IT1 | 0.806890987 | 1.791457017 | 0.073219986 |
| HIST1H4D | ENST00000398777.3 | ENSG00000240152.2 | 0.810166356 | 1.812588822 | 0.069895263 |

| | | | | | |
|---|---|---|---|---|---|
| HIST1H4D | ENST00000400768.2 | ENSG00000215692.2 | 0.84776381 | 1.89575054 | 0.057993039 |
| HIST1H4D | ENST00000413969.1 | ENSG00000224189.2 | 0.832390523 | 1.86585042 | 0.062062296 |
| HIST1H4D | ENST00000414377.1 | ENSG00000230470.1 | 0.947222714 | 2.145507788 | 0.031912269 |
| HIST1H4D | ENST00000417782.1 | ENSG00000228587.1 | 0.954993046 | 2.111139105 | 0.034760357 |
| HIST1H4D | ENST00000418741.1 | ENSG00000227332.1 | 0.927058715 | 2.095630645 | 0.036114969 |
| HIST1H4D | ENST00000420830.1 | ENSG00000231512.1 | 0.930727464 | 2.074229584 | 0.038057994 |
| HIST1H4D | ENST00000421252.2 | ENSG00000250258.1 | -0.837718134 | -1.871987997 | 0.061208264 |
| HIST1H4D | ENST00000421737.1 | ENSG00000232316.1 | 0.888471903 | 1.985507165 | 0.047088074 |
| HIST1H4D | ENST00000428769.1 | ENSG00000232738.1 | 0.878845606 | 1.962776283 | 0.049672175 |
| HIST1H4D | ENST00000430534.1 | ENSG00000229297.1 | -0.853087489 | -1.90933409 | 0.056219007 |
| HIST1H4D | ENST00000430728.1 | ENSG00000232316.1 | 0.955212854 | 2.111228145 | 0.034752707 |
| HIST1H4D | ENST00000432314.1 | ENSG00000231532.1 | 0.893366024 | 1.980641236 | 0.047631521 |
| HIST1H4D | ENST00000433174.1 | ENSG00000162947.4 | 0.851379117 | 1.916705014 | 0.055275418 |
| HIST1H4D | ENST00000434790.1 | ENSG00000240040.1 | -0.949850984 | -2.101082191 | 0.035633752 |
| HIST1H4D | ENST00000435271.1 | ENSG00000231132.1 | -0.822718831 | -1.836745785 | 0.066247433 |
| HIST1H4D | ENST00000435315.2 | ENSG00000226751.2 | 0.863542486 | 1.926655248 | 0.054022609 |
| HIST1H4D | ENST00000435832.1 | ENSG00000229201.1 | 0.814214761 | 1.823856667 | 0.068173744 |
| HIST1H4D | ENST00000440947.1 | ENSG00000225472.1 | 0.926722998 | 2.107614894 | 0.035064314 |
| HIST1H4D | ENST00000441160.1 | ENSG00000228437.1 | -0.833380876 | -1.849170618 | 0.06443318 |
| HIST1H4D | ENST00000447538.2 | ENSG00000224189.2 | 0.902042253 | 2.029086917 | 0.042449438 |
| HIST1H4D | ENST00000448431.1 | ENSG00000232548.1 | 0.822445673 | 1.825347301 | 0.067948637 |
| HIST1H4D | ENST00000450226.1 | ENSG00000231512.1 | 0.959586493 | 2.164908552 | 0.030394689 |
| HIST1H4D | ENST00000450365.1 | ENSG00000224404.1 | 0.872142845 | 1.974204692 | 0.048358473 |
| HIST1H4D | ENST00000451556.2 | ENSG00000228386.2 | 0.941403768 | 2.091535704 | 0.036480069 |
| HIST1H4D | ENST00000451575.2 | ENSG00000224251.2 | 0.86840704 | 1.959207329 | 0.050088511 |
| HIST1H4D | ENST00000453889.1 | ENSG00000224750.2 | 0.93524305 | 2.088120414 | 0.036786974 |
| HIST1H4D | ENST00000455699.1 | ENSG00000240996.1 | 0.894966329 | 2.002025572 | 0.045281981 |
| HIST1H4D | ENST00000458661.2 | ENSG00000236467.3 | 0.921805679 | 2.078717703 | 0.037643305 |
| HIST1H4D | ENST00000469931.2 | ENSG00000272030.1 | 0.873479183 | 1.975041829 | 0.048263401 |
| HIST1H4D | ENST00000481334.1 | ENSG00000242440.1 | 0.810511062 | 1.833623414 | 0.066709914 |
| HIST1H4D | ENST00000496247.1 | ENSG00000241882.1 | 0.875130396 | 1.974835487 | 0.04828682 |
| HIST1H4D | ENST00000504017.1 | ENSG00000248388.1 | 0.860595219 | 1.905037495 | 0.056775197 |
| HIST1H4D | ENST00000504344.1 | ENSG00000251438.1 | 0.871118083 | 1.973545916 | 0.048433399 |
| HIST1H4D | ENST00000507373.1 | ENSG00000250072.1 | 0.877610361 | 1.983416057 | 0.047320975 |
| HIST1H4D | ENST00000508825.1 | ENSG00000250775.1 | 0.874284507 | 1.946170017 | 0.05163432 |
| HIST1H4D | ENST00000508986.1 | ENSG00000249491.1 | 0.978478414 | 2.178312011 | 0.029382816 |
| HIST1H4D | ENST00000513179.1 | ENSG00000251580.1 | 0.929010688 | 2.057656878 | 0.039623083 |
| HIST1H4D | ENST00000515205.1 | ENSG00000251580.1 | 0.934644223 | 2.084864697 | 0.037081584 |
| HIST1H4D | ENST00000515789.1 | ENSG00000248571.1 | -0.892693292 | -1.973295219 | 0.048461937 |
| HIST1H4D | ENST00000518473.1 | ENSG00000253985.1 | 0.843152493 | 1.915002896 | 0.055492134 |
| HIST1H4D | ENST00000523935.1 | ENSG00000253567.1 | 0.829378675 | 1.844626408 | 0.065091903 |
| HIST1H4D | ENST00000524275.1 | ENSG00000253507.1 | 0.805420884 | 1.801764558 | 0.071582456 |
| HIST1H4D | ENST00000529875.1 | ENSG00000254404.1 | -0.850611178 | -1.899812536 | 0.057457725 |
| HIST1H4D | ENST00000531009.1 | ENSG00000255208.1 | 0.873587068 | 1.979511256 | 0.047758474 |
| HIST1H4D | ENST00000531661.1 | ENSG00000254473.1 | 0.853738367 | 1.913059209 | 0.055740473 |
| HIST1H4D | ENST00000532680.1 | ENSG00000255458.1 | 0.903089627 | 1.973425678 | 0.048447084 |
| HIST1H4D | ENST00000536529.1 | ENSG00000256422.1 | 0.816249884 | 1.836009765 | 0.066356212 |
| HIST1H4D | ENST00000538641.1 | ENSG00000256422.1 | 0.803478288 | 1.792369034 | 0.073073872 |
| HIST1H4D | ENST00000545593.1 | ENSG00000256972.1 | 0.838990048 | 1.856574635 | 0.063371695 |
| HIST1H4D | ENST00000547207.1 | ENSG00000224189.2 | 0.975380314 | 2.167991109 | 0.030159363 |
| HIST1H4D | ENST00000549140.1 | ENSG00000258332.1 | 0.824993769 | 1.884724732 | 0.059467011 |

| | | | | | |
|---|---|---|---|---|---|
| HIST1H4D | ENST00000549329.1 | ENSG00000224189.2 | 0.908578038 | 2.021156264 | 0.043263592 |
| HIST1H4D | ENST00000549487.1 | ENSG00000257126.1 | 0.872993505 | 1.94737451 | 0.051489852 |
| HIST1H4D | ENST00000552156.1 | ENSG00000224189.2 | 0.949240944 | 2.11149636 | 0.03472967 |
| HIST1H4D | ENST00000555636.1 | ENSG00000259072.1 | 0.831738251 | 1.84367256 | 0.065230874 |
| HIST1H4D | ENST00000555966.1 | ENSG00000258843.1 | 0.818001372 | 1.811423201 | 0.070075367 |
| HIST1H4D | ENST00000557965.1 | ENSG00000259681.1 | 0.930471366 | 2.089426188 | 0.036669376 |
| HIST1H4D | ENST00000558896.1 | ENSG00000259176.1 | 0.820926167 | 1.853966058 | 0.063744016 |
| HIST1H4D | ENST00000559003.1 | ENSG00000259520.1 | 0.857582611 | 1.908563714 | 0.056318397 |
| HIST1H4D | ENST00000560586.1 | ENSG00000259534.1 | 0.873499773 | 1.944869974 | 0.05179063 |
| HIST1H4D | ENST00000563841.1 | ENSG00000261029.1 | 0.847747939 | 1.888990754 | 0.058893068 |
| HIST1H4D | ENST00000565722.1 | ENSG00000245768.2 | 0.907656301 | 2.01532854 | 0.043870236 |
| HIST1H4D | ENST00000567089.1 | ENSG00000261822.1 | 0.802112612 | 1.793881432 | 0.072832096 |
| HIST1H4D | ENST00000568243.1 | ENSG00000261521.1 | 0.884524894 | 1.969626784 | 0.04888116 |
| HIST1H4D | ENST00000569456.1 | ENSG00000259955.1 | 0.852084186 | 1.904772302 | 0.056809676 |
| HIST1H4D | ENST00000569849.1 | ENSG00000260640.1 | 0.866420361 | 1.96459405 | 0.049461241 |
| HIST1H4D | ENST00000572417.1 | ENSG00000263171.1 | 0.875158643 | 1.951432596 | 0.051005607 |
| HIST1H4D | ENST00000574365.1 | ENSG00000262837.1 | 0.851107826 | 1.886596811 | 0.059214576 |
| HIST1H4D | ENST00000580085.1 | ENSG00000266490.1 | 0.894108746 | 2.00899024 | 0.044538169 |
| HIST1H4D | ENST00000581362.1 | ENSG00000235300.3 | 0.89731823 | 2.037835374 | 0.041566399 |
| HIST1H4D | ENST00000581996.1 | ENSG00000265778.1 | 0.878750884 | 1.953839405 | 0.050720213 |
| HIST1H4D | ENST00000582348.1 | ENSG00000265148.1 | 0.859847408 | 1.93187538 | 0.053374889 |
| HIST1H4D | ENST00000584758.1 | ENSG00000265356.1 | 0.801679193 | 1.809669382 | 0.070347073 |
| HIST1H4D | ENST00000586952.1 | ENSG00000226994.3 | 0.832300027 | 1.853148497 | 0.063861077 |
| HIST1H4D | ENST00000588945.1 | ENSG00000267275.1 | 0.828647048 | 1.865910411 | 0.062053901 |
| HIST1H4D | ENST00000589777.1 | ENSG00000261040.2 | 0.940648076 | 2.122129839 | 0.033826836 |
| HIST1H4D | ENST00000593861.1 | ENSG00000231898.4 | 0.932653923 | 2.07905331 | 0.037612451 |
| HIST1H4D | ENST00000594589.1 | ENSG00000269321.1 | -0.861904257 | -1.915062059 | 0.05548459 |
| HIST1H4D | ENST00000595892.1 | ENSG00000269640.1 | 0.83361968 | 1.86995585 | 0.061489949 |
| HIST1H4D | ENST00000597550.1 | ENSG00000269051.1 | 0.898014991 | 2.000206975 | 0.045477919 |
| HIST1H4D | ENST00000599143.1 | ENSG00000269349.1 | 0.899508365 | 1.992235188 | 0.046345259 |
| HIST1H4D | ENST00000602835.1 | ENSG00000270096.1 | 0.83118637 | 1.858707992 | 0.06306854 |
| HIST1H4D | ENST00000603474.1 | ENSG00000258929.2 | 0.845504859 | 1.871054615 | 0.061337511 |
| HIST1H4D | ENST00000604464.1 | ENSG00000270462.1 | 0.883091981 | 1.978602191 | 0.047860813 |
| HIST1H4D | ENST00000606457.1 | ENSG00000271830.1 | 0.906303459 | 2.01573723 | 0.04382746 |
| HIST1H4D | ENST00000607715.1 | ENSG00000271788.1 | 0.828125019 | 1.881397647 | 0.059917847 |
| HIST1H4D | ENST00000607769.1 | ENSG00000272438.1 | -0.878392093 | -1.985352373 | 0.047105281 |
| HIST1H4D | ENST00000607876.1 | ENSG00000272848.1 | 0.816635733 | 1.868646328 | 0.061672036 |
| HIST1H4D | ENST00000608085.1 | ENSG00000231898.4 | 0.939063158 | 2.130351821 | 0.033142578 |
| HIST1H4D | ENST00000608509.1 | ENSG00000273245.1 | 0.843057416 | 1.869781291 | 0.061514195 |
| HIST1H4D | ENST00000609725.1 | ENSG00000231898.4 | 0.871226801 | 1.921479855 | 0.05467124 |
| HIST1H4D | ENST00000609952.1 | ENSG00000233766.3 | 0.80875987 | 1.826477115 | 0.067778425 |
| HIST1H4D | NR_024410.1 | LINC00710 | 0.970013215 | 2.177233514 | 0.029463149 |
| HIST1H4D | NR_033371.1 | CDRT7 | 0.959889717 | 2.147983857 | 0.031715038 |
| HIST1H4D | NR_046845.1 | DNM3-IT1 | 0.90383247 | 2.051123711 | 0.040254901 |
| HIST1H4D | NR_102703.1 | MAGEA8-AS1 | 0.817278397 | 1.81472645 | 0.069565958 |
| HIST1H4D | NR_104998.1 | LOC102467225 | 0.814931771 | 1.811921772 | 0.069998285 |
| HIST1H4D | NR_110480.1 | LOC101927079 | 0.804802552 | 1.793629452 | 0.072872332 |
| HIST1H4D | NR_120318.1 | RORA-AS2 | 0.802692148 | 1.807406548 | 0.070698914 |
| HIST1H4D | NR_120502.1 | JARID2-AS1 | -0.817586439 | -1.803396535 | 0.071325961 |
| HIST1H4D | NR_120655.1 | KCNMA1-AS1 | 0.954547078 | 2.160410179 | 0.03074093 |
| HIST1H4D | NR_134265.1 | LINC02103 | 0.946486517 | 2.098309588 | 0.035877806 |

| | | | | | |
|---|---|---|---|---|---|
| HIST1H4D | NR_134597.1 | LOC105378068 | 0.815007029 | 1.843037728 | 0.065323502 |
| HIST1H4D | NR_138419.1 | ARHGEF9-IT1 | 0.837332723 | 1.872187867 | 0.061180617 |
| HLA-A | ENST00000318291.4 | ENSG00000177406.4 | 0.885341161 | 1.974044795 | 0.04837665 |
| HLA-A | ENST00000421020.1 | ENSG00000231407.1 | 0.805173627 | 1.781062537 | 0.074902235 |
| HLA-A | ENST00000421207.1 | ENSG00000231768.1 | 0.918788851 | 2.043651707 | 0.040987967 |
| HLA-A | ENST00000423428.1 | ENSG00000224048.1 | -0.810406131 | -1.811497542 | 0.070063869 |
| HLA-A | ENST00000423667.1 | ENSG00000225970.1 | 0.878328175 | 2.009924772 | 0.044439151 |
| HLA-A | ENST00000425124.1 | ENSG00000232336.1 | 0.930296706 | 2.074570399 | 0.038026368 |
| HLA-A | ENST00000425624.1 | ENSG00000223779.4 | 0.970071065 | 2.183968578 | 0.028964557 |
| HLA-A | ENST00000426237.2 | ENSG00000235527.2 | 0.844044192 | 1.903253286 | 0.057007503 |
| HLA-A | ENST00000426699.1 | ENSG00000229308.1 | 0.947162796 | 2.101572672 | 0.035590726 |
| HLA-A | ENST00000432314.1 | ENSG00000231532.1 | 0.807989243 | 1.804725539 | 0.071117641 |
| HLA-A | ENST00000433344.1 | ENSG00000234083.1 | -0.938886847 | -2.110399309 | 0.034823976 |
| HLA-A | ENST00000435434.1 | ENSG00000231233.1 | 0.904162363 | 2.025301077 | 0.042836458 |
| HLA-A | ENST00000435992.2 | ENSG00000232675.3 | 0.91485858 | 2.040827322 | 0.041267994 |
| HLA-A | ENST00000436982.2 | ENSG00000235335.2 | -0.878309102 | -1.963734267 | 0.049560916 |
| HLA-A | ENST00000438107.1 | ENSG00000234449.2 | 0.820944448 | 1.829637746 | 0.067304126 |
| HLA-A | ENST00000438190.1 | ENSG00000227214.2 | 0.863750586 | 1.911654967 | 0.055920464 |
| HLA-A | ENST00000439186.1 | ENSG00000237076.1 | 0.864572495 | 1.908058099 | 0.056383708 |
| HLA-A | ENST00000452176.1 | ENSG00000223659.1 | -0.835942922 | -1.877816585 | 0.060406261 |
| HLA-A | ENST00000454100.1 | ENSG00000236943.2 | 0.82468146 | 1.837719067 | 0.066103813 |
| HLA-A | ENST00000455788.1 | ENSG00000236263.1 | 0.867593032 | 1.965656428 | 0.049338311 |
| HLA-A | ENST00000458154.1 | ENSG00000235578.1 | 0.908680266 | 2.01588926 | 0.043811557 |
| HLA-A | ENST00000458364.1 | ENSG00000225655.1 | -0.836251273 | -1.863591723 | 0.062379061 |
| HLA-A | ENST00000484413.1 | ENSG00000271853.1 | 0.909187647 | 2.032437787 | 0.042109356 |
| HLA-A | ENST00000489077.1 | ENSG00000244198.1 | 0.815981358 | 1.805497045 | 0.070996937 |
| HLA-A | ENST00000494509.1 | ENSG00000240095.1 | 0.873126002 | 1.960564691 | 0.049929825 |
| HLA-A | ENST00000498693.1 | ENSG00000244198.1 | 0.843533148 | 1.888779 | 0.058921448 |
| HLA-A | ENST00000504891.1 | ENSG00000249388.1 | 0.87408164 | 1.953894701 | 0.050713671 |
| HLA-A | ENST00000505498.1 | ENSG00000250908.1 | 0.878377581 | 1.960502575 | 0.049937077 |
| HLA-A | ENST00000505556.1 | ENSG00000249409.1 | 0.815453013 | 1.814749686 | 0.069562386 |
| HLA-A | ENST00000506100.1 | ENSG00000249409.1 | 0.809582523 | 1.807627065 | 0.070664563 |
| HLA-A | ENST00000506791.1 | ENSG00000251131.1 | 0.902449868 | 2.016572164 | 0.04374018 |
| HLA-A | ENST00000508083.1 | ENSG00000249343.1 | 0.88440899 | 1.986500288 | 0.046977801 |
| HLA-A | ENST00000509036.1 | ENSG00000251131.1 | 0.807377678 | 1.818370364 | 0.069007545 |
| HLA-A | ENST00000509192.1 | ENSG00000250765.1 | 0.930458773 | 2.093852887 | 0.036273087 |
| HLA-A | ENST00000515128.1 | ENSG00000248215.1 | -0.849355728 | -1.880091939 | 0.060095549 |
| HLA-A | ENST00000518473.1 | ENSG00000253985.1 | 0.911849817 | 2.041727759 | 0.041178544 |
| HLA-A | ENST00000520603.1 | ENSG00000254001.1 | -0.877495424 | -1.974638206 | 0.04830922 |
| HLA-A | ENST00000521307.1 | ENSG00000253177.1 | 0.888712217 | 2.017285299 | 0.043665749 |
| HLA-A | ENST00000522704.1 | ENSG00000254135.1 | 0.826771686 | 1.858573719 | 0.063087585 |
| HLA-A | ENST00000524818.1 | ENSG00000254473.1 | 0.820763532 | 1.814184614 | 0.069649308 |
| HLA-A | ENST00000526186.1 | ENSG00000254510.1 | 0.850828142 | 1.897505238 | 0.057761288 |
| HLA-A | ENST00000526935.1 | ENSG00000255372.1 | 0.819134418 | 1.819909668 | 0.068772762 |
| HLA-A | ENST00000528887.1 | ENSG00000254501.1 | 0.865675827 | 1.940422443 | 0.052328371 |
| HLA-A | ENST00000547834.1 | ENSG00000258325.1 | 0.924366458 | 2.069967225 | 0.038455414 |
| HLA-A | ENST00000548722.2 | ENSG00000257194.2 | -0.807693374 | -1.8024414 | 0.071475987 |
| HLA-A | ENST00000550263.1 | ENSG00000257605.1 | 0.834829118 | 1.874374967 | 0.060878761 |
| HLA-A | ENST00000556397.1 | ENSG00000258654.1 | 0.823740038 | 1.826643542 | 0.067753382 |
| HLA-A | ENST00000556786.1 | ENSG00000258525.1 | -0.85342704 | -1.933327992 | 0.053195806 |
| HLA-A | ENST00000559003.1 | ENSG00000259520.1 | 0.841790284 | 1.8729647 | 0.06107326 |

| | | | | | |
|---|---|---|---|---|---|
| HLA-A | ENST00000563841.1 | ENSG00000261029.1 | 0.825913098 | 1.841767186 | 0.065509212 |
| HLA-A | ENST00000564038.1 | ENSG00000261760.2 | 0.814799632 | 1.811144538 | 0.070118481 |
| HLA-A | ENST00000565965.1 | ENSG00000261172.1 | 0.827553848 | 1.868119778 | 0.061745377 |
| HLA-A | ENST00000566170.1 | ENSG00000261071.1 | 0.810890975 | 1.823395118 | 0.068243569 |
| HLA-A | ENST00000567395.1 | ENSG00000261090.1 | 0.92654265 | 2.052133219 | 0.040156717 |
| HLA-A | ENST00000568033.1 | ENSG00000261480.1 | 0.827495575 | 1.857772303 | 0.063201356 |
| HLA-A | ENST00000569981.1 | ENSG00000238045.5 | 0.9226022 | 2.053507916 | 0.040023343 |
| HLA-A | ENST00000570493.2 | ENSG00000261898.2 | 0.916339805 | 2.048559769 | 0.040505182 |
| HLA-A | ENST00000570512.1 | ENSG00000262768.1 | 0.880594228 | 1.967087256 | 0.049173152 |
| HLA-A | ENST00000570929.1 | ENSG00000262223.2 | 0.839607812 | 1.874330674 | 0.060884862 |
| HLA-A | ENST00000574365.1 | ENSG00000262837.1 | 0.809167024 | 1.835451373 | 0.066438838 |
| HLA-A | ENST00000577698.1 | ENSG00000265100.1 | 0.825714092 | 1.839240414 | 0.065879833 |
| HLA-A | ENST00000578265.1 | ENSG00000214719.7 | 0.810505648 | 1.832334805 | 0.066901555 |
| HLA-A | ENST00000582558.1 | ENSG00000264569.1 | 0.872679007 | 1.935465448 | 0.052933205 |
| HLA-A | ENST00000584705.1 | ENSG00000264569.1 | 0.913424151 | 2.06038162 | 0.039362073 |
| HLA-A | ENST00000585559.1 | ENSG00000267117.1 | 0.969534085 | 2.15796823 | 0.030930302 |
| HLA-A | ENST00000588799.1 | ENSG00000267275.1 | 0.89222899 | 1.963363427 | 0.04960396 |
| HLA-A | ENST00000588945.1 | ENSG00000267275.1 | 0.909278501 | 2.054689883 | 0.039908968 |
| HLA-A | ENST00000592400.1 | ENSG00000267735.1 | 0.9369739 | 2.096211471 | 0.036063436 |
| HLA-A | ENST00000592720.1 | ENSG00000267232.1 | 0.845023232 | 1.886549178 | 0.059220988 |
| HLA-A | ENST00000595478.1 | ENSG00000237031.3 | -0.854390507 | -1.900358544 | 0.057386083 |
| HLA-A | ENST00000597169.1 | ENSG00000269720.1 | 0.875949728 | 1.94892486 | 0.051304399 |
| HLA-A | ENST00000599259.1 | ENSG00000269352.1 | 0.888120473 | 2.003500229 | 0.045123623 |
| HLA-A | ENST00000600489.1 | ENSG00000231898.4 | 0.840554384 | 1.879490949 | 0.060177488 |
| HLA-A | ENST00000600726.1 | ENSG00000267858.1 | 0.801660004 | 1.800555736 | 0.071772931 |
| HLA-A | ENST00000601692.1 | ENSG00000267874.1 | -0.975643669 | -2.184719381 | 0.028909428 |
| HLA-A | ENST00000601735.1 | ENSG00000244513.2 | 0.874077462 | 1.93635906 | 0.05282374 |
| HLA-A | ENST00000602594.1 | ENSG00000269930.1 | -0.830544236 | -1.853830963 | 0.063763347 |
| HLA-A | ENST00000602809.1 | ENSG00000270105.1 | -0.827928087 | -1.821476567 | 0.068534446 |
| HLA-A | ENST00000602949.1 | ENSG00000270030.1 | 0.833170998 | 1.875445036 | 0.060731524 |
| HLA-A | ENST00000604142.1 | ENSG00000271308.1 | 0.844956484 | 1.910922983 | 0.056014479 |
| HLA-A | ENST00000606377.1 | ENSG00000272286.1 | -0.879842284 | -1.971080124 | 0.048714712 |
| HLA-A | ENST00000606470.1 | ENSG00000271913.1 | 0.919105591 | 2.03777288 | 0.041572652 |
| HLA-A | ENST00000606743.1 | ENSG00000272221.1 | 0.833959885 | 1.849768665 | 0.064346899 |
| HLA-A | ENST00000607284.1 | ENSG00000272389.1 | 0.916265581 | 2.05540563 | 0.039839842 |
| HLA-A | ENST00000607943.1 | ENSG00000273188.1 | 0.801147734 | 1.778985253 | 0.07524218 |
| HLA-A | ENST00000609725.1 | ENSG00000231898.4 | 0.808867827 | 1.818262042 | 0.069024091 |
| HLA-A | ENST00000609972.1 | ENSG00000230651.3 | 0.94391173 | 2.116836143 | 0.034273757 |
| HLA-A | ENST00000610145.1 | ENSG00000273175.1 | 0.84686233 | 1.873017742 | 0.061065935 |
| HLA-A | ENST00000610161.1 | ENSG00000273059.1 | 0.881762498 | 1.962436502 | 0.049711687 |
| HLA-A | NR_003604.2 | ZFAS1 | 0.810461901 | 1.822577715 | 0.068367373 |
| HLA-A | NR_003605.1 | ZFAS1 | 0.921139387 | 2.059043699 | 0.039490052 |
| HLA-A | NR_003606.2 | ZFAS1 | 0.808069911 | 1.804058388 | 0.071222154 |
| HLA-A | NR_027271.1 | CIRBP-AS1 | 0.803574013 | 1.799261786 | 0.07197728 |
| HLA-A | NR_028324.1 | LINC01002 | 0.867766911 | 1.945988751 | 0.051656091 |
| HLA-A | NR_036480.1 | VPS9D1-AS1 | 0.814240385 | 1.813297199 | 0.069785995 |
| HLA-A | NR_036658.1 | ZFAS1 | 0.817592267 | 1.813351655 | 0.069777601 |
| HLA-A | NR_037169.1 | LOC100507547 | 0.853458757 | 1.907543032 | 0.056450304 |
| HLA-A | NR_037170.1 | LOC100507547 | 0.841977107 | 1.886931189 | 0.059169581 |
| HLA-A | NR_044996.1 | HCG23 | 0.961904877 | 2.128958105 | 0.033257727 |
| HLA-A | NR_045114.1 | PVRL3-AS1 | -0.814701752 | -1.842838985 | 0.065352523 |

| | | | | | |
|---|---|---|---|---|---|
| HLA-A | NR_072981.1 | LINC00957 | 0.912781126 | 2.04747397 | 0.04061157 |
| HLA-A | NR_072982.1 | LINC00957 | 0.914757395 | 2.047476654 | 0.040611307 |
| HLA-A | NR_105010.1 | LINC01333 | 0.882947892 | 1.956050076 | 0.050459255 |
| HLA-A | NR_108036.1 | CFAP58-AS1 | 0.90132692 | 1.974922027 | 0.048276997 |
| HLA-A | NR_109886.1 | RALY-AS1 | 0.820611005 | 1.851496335 | 0.064098181 |
| HLA-A | NR_120335.1 | LOC101928414 | 0.920092116 | 2.048326936 | 0.040527975 |
| HLA-A | NR_121189.1 | PGM5P3-AS1 | -0.857187185 | -1.904610231 | 0.056830756 |
| HLA-A | NR_125957.1 | LOC101928626 | -0.835942922 | -1.853746762 | 0.063775398 |
| HLA-A | NR_126166.1 | FAM74A7 | 0.955161134 | 2.125534249 | 0.033542059 |
| HLA-A | NR_138084.1 | HCG24 | 0.86339591 | 1.946756944 | 0.051563881 |
| HLA-A | NR_144459.1 | ARSD-AS1 | 0.864784468 | 1.933085258 | 0.053225696 |
| HLA-B | ENST00000412085.1 | ENSG00000233825.1 | 0.84084246 | 1.892938074 | 0.058366105 |
| HLA-B | ENST00000412759.1 | ENSG00000236933.1 | 0.892657492 | 1.990752916 | 0.046508058 |
| HLA-B | ENST00000415106.1 | ENSG00000226733.1 | -0.811684852 | -1.816300073 | 0.069324354 |
| HLA-B | ENST00000415205.1 | ENSG00000182057.4 | 0.881888345 | 1.97135969 | 0.048682748 |
| HLA-B | ENST00000419662.1 | ENSG00000228265.1 | 0.882794322 | 1.965892536 | 0.049311025 |
| HLA-B | ENST00000422763.1 | ENSG00000231131.2 | -0.832251188 | -1.846801311 | 0.064775942 |
| HLA-B | ENST00000426237.2 | ENSG00000235527.2 | 0.867345379 | 1.929296152 | 0.053694107 |
| HLA-B | ENST00000426475.1 | ENSG00000239467.1 | 0.893483782 | 2.00173919 | 0.045312789 |
| HLA-B | ENST00000426519.1 | ENSG00000234142.1 | 0.801410925 | 1.810984462 | 0.070143257 |
| HLA-B | ENST00000428765.1 | ENSG00000230107.1 | 0.91438385 | 2.032297416 | 0.042123556 |
| HLA-B | ENST00000429080.1 | ENSG00000233047.1 | -0.906595256 | -2.037065529 | 0.041643476 |
| HLA-B | ENST00000430920.1 | ENSG00000234203.1 | 0.865619487 | 1.904724488 | 0.056815894 |
| HLA-B | ENST00000433051.1 | ENSG00000233193.1 | 0.834310929 | 1.865339727 | 0.0621338 |
| HLA-B | ENST00000433905.2 | ENSG00000229299.2 | 0.975154157 | 2.176682056 | 0.029504298 |
| HLA-B | ENST00000438190.1 | ENSG00000227214.2 | 0.908966929 | 2.036083153 | 0.041742006 |
| HLA-B | ENST00000438969.2 | ENSG00000228031.2 | -0.848573186 | -1.899067781 | 0.057555565 |
| HLA-B | ENST00000439184.1 | ENSG00000233985.1 | -0.829623409 | -1.859430261 | 0.062966176 |
| HLA-B | ENST00000440595.1 | ENSG00000228265.1 | 0.868897933 | 1.944513501 | 0.051833559 |
| HLA-B | ENST00000441592.2 | ENSG00000224078.8 | 0.884661455 | 1.985192208 | 0.047123091 |
| HLA-B | ENST00000447343.2 | ENSG00000229299.2 | 0.97958298 | 2.205799307 | 0.027398055 |
| HLA-B | ENST00000448570.1 | ENSG00000224549.1 | 0.870543812 | 1.910470171 | 0.056072704 |
| HLA-B | ENST00000449463.1 | ENSG00000230309.1 | -0.800422086 | -1.790634002 | 0.073352047 |
| HLA-B | ENST00000451507.1 | ENSG00000229539.1 | 0.841453602 | 1.878456643 | 0.060318723 |
| HLA-B | ENST00000453051.1 | ENSG00000229407.1 | 0.877227413 | 1.945088283 | 0.051764354 |
| HLA-B | ENST00000463255.1 | ENSG00000243305.1 | -0.824596121 | -1.835488022 | 0.066433412 |
| HLA-B | ENST00000468165.1 | ENSG00000239480.1 | 0.91550787 | 2.072157812 | 0.038250726 |
| HLA-B | ENST00000489077.1 | ENSG00000244198.1 | 0.958345642 | 2.133815825 | 0.03285786 |
| HLA-B | ENST00000498693.1 | ENSG00000244198.1 | 0.894389611 | 1.982040422 | 0.047474717 |
| HLA-B | ENST00000503723.1 | ENSG00000250472.1 | -0.944247772 | -2.147065574 | 0.031788062 |
| HLA-B | ENST00000504916.1 | ENSG00000248112.1 | -0.844889573 | -1.895353064 | 0.058045642 |
| HLA-B | ENST00000506791.1 | ENSG00000251131.1 | 0.876898881 | 1.954322134 | 0.050663133 |
| HLA-B | ENST00000509036.1 | ENSG00000251131.1 | 0.89522872 | 1.999901331 | 0.045510919 |
| HLA-B | ENST00000509192.1 | ENSG00000250765.1 | 0.862931263 | 1.932475942 | 0.053300789 |
| HLA-B | ENST00000509453.1 | ENSG00000249145.1 | 0.845825509 | 1.879484831 | 0.060178323 |
| HLA-B | ENST00000510570.1 | ENSG00000250438.1 | -0.953852031 | -2.12507231 | 0.03358058 |
| HLA-B | ENST00000510922.1 | ENSG00000250777.1 | -0.805922764 | -1.793261054 | 0.072931192 |
| HLA-B | ENST00000517846.1 | ENSG00000254485.1 | 0.869438318 | 1.97885762 | 0.047832039 |
| HLA-B | ENST00000520603.1 | ENSG00000254001.1 | -0.903860854 | -2.026614617 | 0.04270184 |
| HLA-B | ENST00000521653.1 | ENSG00000253301.1 | 0.877518264 | 1.968493852 | 0.049011243 |
| HLA-B | ENST00000522547.1 | ENSG00000253430.1 | -0.8155957 | -1.833526172 | 0.06672436 |

| | | | | | |
|---|---|---|---|---|---|
| HLA-B | ENST00000529247.1 | ENSG00000254741.1 | 0.80261368 | 1.822502398 | 0.06837879 |
| HLA-B | ENST00000543072.1 | ENSG00000256092.2 | -0.812613672 | -1.794934113 | 0.072664198 |
| HLA-B | ENST00000543275.1 | ENSG00000256944.1 | 0.84525751 | 1.87889863 | 0.060258336 |
| HLA-B | ENST00000545177.3 | ENSG00000230438.5 | 0.812746716 | 1.834182596 | 0.066626894 |
| HLA-B | ENST00000549806.1 | ENSG00000257252.1 | 0.847290061 | 1.918874216 | 0.055000254 |
| HLA-B | ENST00000558575.1 | ENSG00000259687.1 | 0.825228421 | 1.833445468 | 0.066736351 |
| HLA-B | ENST00000563611.1 | ENSG00000261583.1 | 0.947288911 | 2.124990727 | 0.033587387 |
| HLA-B | ENST00000564809.1 | ENSG00000261471.1 | 0.858423429 | 1.92132749 | 0.054690434 |
| HLA-B | ENST00000578800.1 | ENSG00000264235.1 | 0.918361073 | 2.059395157 | 0.039456399 |
| HLA-B | ENST00000579775.1 | ENSG00000264108.1 | 0.817944831 | 1.83947038 | 0.065846031 |
| HLA-B | ENST00000580622.1 | ENSG00000264634.1 | 0.897956037 | 2.021255837 | 0.043253288 |
| HLA-B | ENST00000582044.1 | ENSG00000263715.2 | 0.958689902 | 2.133073144 | 0.032918726 |
| HLA-B | ENST00000582558.1 | ENSG00000264569.1 | 0.875014404 | 1.978412866 | 0.047882149 |
| HLA-B | ENST00000588182.2 | ENSG00000267453.2 | 0.874183917 | 1.96243933 | 0.049711358 |
| HLA-B | ENST00000588380.1 | ENSG00000266990.1 | 0.83141727 | 1.86662785 | 0.061953576 |
| HLA-B | ENST00000589380.1 | ENSG00000267488.1 | 0.802619307 | 1.796020737 | 0.072491219 |
| HLA-B | ENST00000589395.1 | ENSG00000267143.1 | 0.800662619 | 1.782470174 | 0.074672591 |
| HLA-B | ENST00000591174.1 | ENSG00000267289.1 | 0.811835462 | 1.811076149 | 0.070129065 |
| HLA-B | ENST00000592498.1 | ENSG00000267488.1 | 0.812866633 | 1.815836872 | 0.069395399 |
| HLA-B | ENST00000592525.1 | ENSG00000267214.1 | 0.900516068 | 2.008422449 | 0.044598419 |
| HLA-B | ENST00000593139.1 | ENSG00000267042.1 | 0.916509282 | 2.034720723 | 0.041878982 |
| HLA-B | ENST00000593218.1 | ENSG00000267421.2 | 0.850560657 | 1.881326719 | 0.059927489 |
| HLA-B | ENST00000594590.2 | ENSG00000268199.2 | 0.834993198 | 1.86538154 | 0.062127943 |
| HLA-B | ENST00000594776.1 | ENSG00000269807.1 | 0.8004742 | 1.789770268 | 0.073490851 |
| HLA-B | ENST00000594850.1 | ENSG00000268093.1 | 0.900549009 | 2.004308455 | 0.045037029 |
| HLA-B | ENST00000596091.1 | ENSG00000227733.4 | -0.807036281 | -1.808720722 | 0.070494402 |
| HLA-B | ENST00000596887.1 | ENSG00000237031.3 | -0.878303936 | -1.968100988 | 0.049056419 |
| HLA-B | ENST00000596971.1 | ENSG00000269463.1 | 0.802174971 | 1.803373604 | 0.07132956 |
| HLA-B | ENST00000597169.1 | ENSG00000269720.1 | 0.863206116 | 1.933477182 | 0.053177441 |
| HLA-B | ENST00000597256.1 | ENSG00000267986.1 | 0.942410559 | 2.082119783 | 0.03733153 |
| HLA-B | ENST00000599352.1 | ENSG00000240401.4 | -0.869142795 | -1.924657275 | 0.054272249 |
| HLA-B | ENST00000600242.1 | ENSG00000269583.1 | 0.870857032 | 1.958279381 | 0.050197239 |
| HLA-B | ENST00000600726.1 | ENSG00000267858.1 | 0.846245709 | 1.897800122 | 0.057722417 |
| HLA-B | ENST00000601033.1 | ENSG00000268401.1 | 0.848082058 | 1.889659651 | 0.058803494 |
| HLA-B | ENST00000604142.1 | ENSG00000271308.1 | 0.938434933 | 2.101414555 | 0.035604591 |
| HLA-B | ENST00000604183.1 | ENSG00000271185.1 | 0.895349351 | 2.001127214 | 0.045378682 |
| HLA-B | ENST00000606277.1 | ENSG00000272145.1 | 0.903894542 | 2.021240566 | 0.043254868 |
| HLA-B | ENST00000606441.1 | ENSG00000272277.1 | 0.834691417 | 1.8860958 | 0.059282046 |
| HLA-B | ENST00000606743.1 | ENSG00000272221.1 | 0.801974985 | 1.815914549 | 0.069383481 |
| HLA-B | ENST00000606909.1 | ENSG00000271821.1 | 0.891043773 | 2.000160023 | 0.045482987 |
| HLA-B | ENST00000607052.1 | ENSG00000271870.1 | -0.80318219 | -1.811813942 | 0.07001495 |
| HLA-B | ENST00000607224.1 | ENSG00000272521.1 | 0.934496894 | 2.085154809 | 0.03705525 |
| HLA-B | ENST00000607476.1 | ENSG00000272540.1 | 0.964398647 | 2.185325237 | 0.028865008 |
| HLA-B | ENST00000607943.1 | ENSG00000273188.1 | 0.909156956 | 2.002792484 | 0.045199567 |
| HLA-B | ENST00000608677.1 | ENSG00000273350.1 | 0.931139808 | 2.070123198 | 0.038440809 |
| HLA-B | NR_003604.2 | ZFAS1 | 0.80708569 | 1.820192033 | 0.068729766 |
| HLA-B | NR_003606.2 | ZFAS1 | 0.815913601 | 1.827147322 | 0.067677622 |
| HLA-B | NR_024321.1 | LINC00115 | 0.854684716 | 1.931253386 | 0.053451725 |
| HLA-B | NR_026802.1 | FAM74A4 | 0.888731853 | 2.022502432 | 0.043124473 |
| HLA-B | NR_026813.1 | LINC00597 | -0.906147438 | -2.030765454 | 0.042278793 |
| HLA-B | NR_026951.1 | LINC00324 | 0.828235407 | 1.844940065 | 0.065046258 |

| | | | | | |
|---|---|---|---|---|---|
| HLA-B | NR_027052.1 | THAP7-AS1 | 0.909012821 | 2.046249813 | 0.040731799 |
| HLA-B | NR_027271.1 | CIRBP-AS1 | 0.873367652 | 1.985999846 | 0.047033341 |
| HLA-B | NR_036658.1 | ZFAS1 | 0.816903884 | 1.848932837 | 0.064467511 |
| HLA-B | NR_037169.1 | LOC100507547 | 0.867255019 | 1.965128032 | 0.049399421 |
| HLA-B | NR_037170.1 | LOC100507547 | 0.82518887 | 1.842936628 | 0.065338264 |
| HLA-B | NR_038421.1 | LINC01220 | 0.894376805 | 2.0310402 | 0.042250917 |
| HLA-B | NR_038923.1 | SSSCA1-AS1 | 0.935757963 | 2.106219956 | 0.03518525 |
| HLA-B | NR_045114.1 | PVRL3-AS1 | -0.934521847 | -2.120467909 | 0.033966605 |
| HLA-B | NR_046871.1 | LINC00333 | -0.868337801 | -1.929346253 | 0.053687891 |
| HLA-B | NR_047116.1 | HIF1A-AS1 | -0.921494425 | -2.099262176 | 0.035793796 |
| HLA-B | NR_051987.1 | LINC00499 | 0.863354933 | 1.919865697 | 0.054874866 |
| HLA-B | NR_072981.1 | LINC00957 | 0.856117972 | 1.92032707 | 0.054816599 |
| HLA-B | NR_072982.1 | LINC00957 | 0.854494546 | 1.902673813 | 0.057083121 |
| HLA-B | NR_105010.1 | LINC01333 | 0.856940884 | 1.909681136 | 0.056174281 |
| HLA-B | NR_108106.1 | LINC01135 | 0.835477709 | 1.871988114 | 0.061208248 |
| HLA-B | NR_109831.1 | RASSF1-AS1 | 0.835530311 | 1.844227327 | 0.065150017 |
| HLA-B | NR_109885.1 | RALY-AS1 | 0.876038466 | 1.94368308 | 0.05193368 |
| HLA-B | NR_109886.1 | RALY-AS1 | 0.878956941 | 1.982820333 | 0.047387502 |
| HLA-B | NR_110245.1 | LOC101929282 | -0.867414818 | -1.927051003 | 0.053973274 |
| HLA-B | NR_110630.1 | LOC101927478 | 0.848499399 | 1.897995134 | 0.057696723 |
| HLA-B | NR_110941.1 | MIR762HG | 0.98684969 | 2.204553764 | 0.027485423 |
| HLA-B | NR_110998.1 | FAM74A4 | 0.888731853 | 1.964048286 | 0.049524493 |
| HLA-B | NR_111951.1 | LINC00869 | 0.857600899 | 1.913783257 | 0.055647856 |
| HLA-B | NR_111952.1 | LINC00869 | 0.871727843 | 1.963534836 | 0.049584061 |
| HLA-B | NR_111953.1 | LINC00869 | 0.853142643 | 1.89163679 | 0.058539391 |
| HLA-B | NR_126522.1 | EXOC3-AS1 | 0.866543585 | 1.950612395 | 0.051103172 |
| HLA-B | NR_130143.1 | LOC104968399 | 0.91489618 | 2.048548479 | 0.040506287 |
| HLA-B | NR_135024.1 | LOC105369747 | 0.848794392 | 1.925273883 | 0.054195103 |
| HLA-B | NR_135584.1 | LOC101927596 | 0.862100045 | 1.920182793 | 0.054834814 |
| HLA-B | NR_136215.1 | VCAN-AS1 | -0.909190796 | -2.07069968 | 0.03838687 |
| HLA-C | ENST00000381106.4 | ENSG00000205663.5 | -0.843114983 | -1.87686134 | 0.060537101 |
| HLA-C | ENST00000412348.1 | ENSG00000228959.1 | -0.871430114 | -1.951805978 | 0.050961244 |
| HLA-C | ENST00000415205.1 | ENSG00000182057.4 | 0.844324878 | 1.874407346 | 0.060874302 |
| HLA-C | ENST00000417260.1 | ENSG00000231734.4 | -0.800606846 | -1.799135977 | 0.071997174 |
| HLA-C | ENST00000418387.1 | ENSG00000235056.1 | -0.876540743 | -1.951238245 | 0.051028711 |
| HLA-C | ENST00000423428.1 | ENSG00000224048.1 | -0.803499473 | -1.810114327 | 0.07027806 |
| HLA-C | ENST00000426302.1 | ENSG00000230454.1 | 0.984284709 | 2.193969948 | 0.02823757 |
| HLA-C | ENST00000429080.1 | ENSG00000233047.1 | -0.858314123 | -1.927525549 | 0.053914167 |
| HLA-C | ENST00000430920.1 | ENSG00000234203.1 | 0.923734 | 2.061400646 | 0.039264833 |
| HLA-C | ENST00000433905.2 | ENSG00000229299.2 | 0.825988945 | 1.834800495 | 0.066535255 |
| HLA-C | ENST00000435287.1 | ENSG00000227220.1 | 0.859066559 | 1.909188342 | 0.0562378 |
| HLA-C | ENST00000436515.1 | ENSG00000224521.1 | -0.908876653 | -2.028114794 | 0.042548533 |
| HLA-C | ENST00000438623.1 | ENSG00000224521.1 | -0.926973288 | -2.054694589 | 0.039908513 |
| HLA-C | ENST00000442017.1 | ENSG00000229660.1 | 0.820616192 | 1.854476497 | 0.06367102 |
| HLA-C | ENST00000442829.1 | ENSG00000225284.1 | 0.896748127 | 2.027889385 | 0.042571539 |
| HLA-C | ENST00000448858.1 | ENSG00000237734.1 | -0.926226556 | -2.078192217 | 0.037691659 |
| HLA-C | ENST00000450696.1 | ENSG00000235146.2 | -0.801841498 | -1.804855413 | 0.07109731 |
| HLA-C | ENST00000451034.1 | ENSG00000229805.1 | -0.95429401 | -2.139731006 | 0.032376512 |
| HLA-C | ENST00000451090.1 | ENSG00000235215.2 | -0.903640078 | -2.038818792 | 0.041468116 |
| HLA-C | ENST00000455373.1 | ENSG00000226097.1 | -0.909533048 | -2.034585228 | 0.041892625 |
| HLA-C | ENST00000457043.1 | ENSG00000231365.1 | -0.804664237 | -1.79028724 | 0.073407746 |

| | | | | | |
|---|---|---|---|---|---|
| HLA-C | ENST00000457998.2 | ENSG00000233006.2 | 0.912029173 | 2.028167668 | 0.042543138 |
| HLA-C | ENST00000468165.1 | ENSG00000239480.1 | 0.938534161 | 2.107621576 | 0.035063736 |
| HLA-C | ENST00000503505.1 | ENSG00000248629.1 | -0.860922161 | -1.911768466 | 0.055905898 |
| HLA-C | ENST00000503723.1 | ENSG00000250472.1 | -0.898406159 | -1.992746051 | 0.046289262 |
| HLA-C | ENST00000504578.1 | ENSG00000251513.1 | -0.862251836 | -1.957312802 | 0.050310703 |
| HLA-C | ENST00000506723.2 | ENSG00000249484.4 | -0.890466267 | -1.989119547 | 0.046688009 |
| HLA-C | ENST00000515128.1 | ENSG00000248215.1 | -0.873650483 | -1.944142557 | 0.051878262 |
| HLA-C | ENST00000518837.1 | ENSG00000253947.1 | -0.845681549 | -1.875730716 | 0.060692266 |
| HLA-C | ENST00000521294.1 | ENSG00000253664.1 | 0.893215712 | 1.984268427 | 0.047225924 |
| HLA-C | ENST00000521653.1 | ENSG00000253301.1 | 0.843000289 | 1.90301215 | 0.05703896 |
| HLA-C | ENST00000522281.1 | ENSG00000253376.1 | -0.846383995 | -1.884258049 | 0.059530079 |
| HLA-C | ENST00000522300.1 | ENSG00000249484.4 | -0.884162917 | -2.002533692 | 0.045227363 |
| HLA-C | ENST00000522390.1 | ENSG00000254262.1 | -0.903359311 | -2.002519091 | 0.045228932 |
| HLA-C | ENST00000522600.1 | ENSG00000246582.2 | 0.833179192 | 1.890895884 | 0.058638244 |
| HLA-C | ENST00000523806.1 | ENSG00000253616.1 | 0.844892252 | 1.893506232 | 0.05829058 |
| HLA-C | ENST00000527086.1 | ENSG00000255182.1 | 0.943765654 | 2.125107395 | 0.033577653 |
| HLA-C | ENST00000527274.2 | ENSG00000255517.2 | 0.892898305 | 2.004409394 | 0.045026224 |
| HLA-C | ENST00000527757.1 | ENSG00000255109.1 | -0.823377358 | -1.871305698 | 0.061302721 |
| HLA-C | ENST00000535914.1 | ENSG00000256894.1 | -0.822836508 | -1.838631718 | 0.065969373 |
| HLA-C | ENST00000537850.1 | ENSG00000251002.3 | 0.893274267 | 1.988295186 | 0.046779053 |
| HLA-C | ENST00000543275.1 | ENSG00000256944.1 | 0.900203184 | 2.060039142 | 0.039394799 |
| HLA-C | ENST00000543494.1 | ENSG00000256514.1 | 0.963513555 | 2.145269682 | 0.031931291 |
| HLA-C | ENST00000548210.1 | ENSG00000257784.1 | 0.95897798 | 2.153902849 | 0.031247791 |
| HLA-C | ENST00000549683.1 | ENSG00000257953.1 | 0.879272603 | 1.96562193 | 0.049342299 |
| HLA-C | ENST00000550279.1 | ENSG00000258338.1 | -0.90205239 | -2.014224302 | 0.043985989 |
| HLA-C | ENST00000551135.1 | ENSG00000258294.1 | -0.963361985 | -2.161353246 | 0.030668063 |
| HLA-C | ENST00000552541.1 | ENSG00000258294.1 | -0.887557178 | -1.975371914 | 0.048225958 |
| HLA-C | ENST00000555460.1 | ENSG00000259042.1 | 0.879992861 | 1.97810794 | 0.047916531 |
| HLA-C | ENST00000558237.1 | ENSG00000259684.1 | -0.802425527 | -1.763353332 | 0.077840916 |
| HLA-C | ENST00000558575.1 | ENSG00000259687.1 | 0.944455497 | 2.133659831 | 0.032870637 |
| HLA-C | ENST00000563018.1 | ENSG00000260193.1 | 0.918449997 | 2.044305904 | 0.040923336 |
| HLA-C | ENST00000563610.1 | ENSG00000260051.1 | 0.96055183 | 2.140379282 | 0.032324127 |
| HLA-C | ENST00000563611.1 | ENSG00000261583.1 | 0.9377232 | 2.115545034 | 0.034383521 |
| HLA-C | ENST00000563855.1 | ENSG00000260658.1 | -0.845681549 | -1.884658953 | 0.059475898 |
| HLA-C | ENST00000564809.1 | ENSG00000261471.1 | 0.911139497 | 2.06097868 | 0.039305074 |
| HLA-C | ENST00000565667.1 | ENSG00000261253.1 | 0.815877794 | 1.815654972 | 0.069423315 |
| HLA-C | ENST00000565735.1 | ENSG00000261213.1 | -0.933642269 | -2.061248898 | 0.039279301 |
| HLA-C | ENST00000565798.2 | ENSG00000259786.2 | -0.802982717 | -1.801775939 | 0.071580665 |
| HLA-C | ENST00000566449.1 | ENSG00000259791.1 | -0.802937847 | -1.791964218 | 0.073138698 |
| HLA-C | ENST00000568659.1 | ENSG00000260004.1 | -0.94567016 | -2.148894382 | 0.031642773 |
| HLA-C | ENST00000569313.1 | ENSG00000261604.1 | -0.854396175 | -1.92055522 | 0.054787805 |
| HLA-C | ENST00000570974.1 | ENSG00000263300.1 | 0.850129623 | 1.908561674 | 0.05631866 |
| HLA-C | ENST00000573260.1 | ENSG00000262482.1 | -0.811702188 | -1.813277939 | 0.069788964 |
| HLA-C | ENST00000576086.1 | ENSG00000262823.1 | 0.855241583 | 1.899309147 | 0.057523841 |
| HLA-C | ENST00000577853.1 | ENSG00000264207.1 | 0.958346452 | 2.143314945 | 0.032087818 |
| HLA-C | ENST00000579775.1 | ENSG00000264108.1 | 0.844845616 | 1.890206406 | 0.05873036 |
| HLA-C | ENST00000585810.1 | ENSG00000236172.2 | 0.803639352 | 1.796016476 | 0.072491897 |
| HLA-C | ENST00000588402.1 | ENSG00000267006.1 | -0.937674524 | -2.097652899 | 0.035935819 |
| HLA-C | ENST00000588842.1 | ENSG00000235779.3 | -0.870737862 | -1.94832182 | 0.051376468 |
| HLA-C | ENST00000589673.1 | ENSG00000267755.1 | 0.820530955 | 1.843979718 | 0.065186096 |
| HLA-C | ENST00000590328.1 | ENSG00000256995.2 | -0.849816575 | -1.90425872 | 0.056876498 |

| | | | | | |
|---|---|---|---|---|---|
| HLA-C | ENST00000591174.1 | ENSG00000267289.1 | 0.876953599 | 1.981030933 | 0.047587805 |
| HLA-C | ENST00000592518.1 | ENSG00000267786.1 | 0.847966843 | 1.898165005 | 0.057674349 |
| HLA-C | ENST00000592816.1 | ENSG00000236172.2 | 0.810919894 | 1.824194873 | 0.068122616 |
| HLA-C | ENST00000593175.1 | ENSG00000229036.3 | -0.894745508 | -2.019016886 | 0.043485464 |
| HLA-C | ENST00000594590.2 | ENSG00000268199.2 | 0.804855007 | 1.792595015 | 0.073037704 |
| HLA-C | ENST00000596091.1 | ENSG00000227733.4 | -0.823065374 | -1.841625594 | 0.065529935 |
| HLA-C | ENST00000596567.1 | ENSG00000226647.2 | -0.82791289 | -1.855001272 | 0.063596045 |
| HLA-C | ENST00000596887.1 | ENSG00000237031.3 | -0.924170336 | -2.096575804 | 0.036031143 |
| HLA-C | ENST00000597755.1 | ENSG00000236194.2 | -0.826343706 | -1.845286919 | 0.064995812 |
| HLA-C | ENST00000598092.1 | ENSG00000228065.6 | -0.983530663 | -2.176708561 | 0.029502319 |
| HLA-C | ENST00000600007.1 | ENSG00000268655.1 | 0.833369345 | 1.843973443 | 0.065187011 |
| HLA-C | ENST00000600242.1 | ENSG00000269583.1 | 0.832891571 | 1.842043603 | 0.065468772 |
| HLA-C | ENST00000600716.1 | ENSG00000269487.1 | 0.862720087 | 1.954615912 | 0.050628422 |
| HLA-C | ENST00000600726.1 | ENSG00000267858.1 | 0.925890492 | 2.071243358 | 0.03833606 |
| HLA-C | ENST00000602881.1 | ENSG00000269965.1 | -0.832737733 | -1.87239347 | 0.061152188 |
| HLA-C | ENST00000604183.1 | ENSG00000271185.1 | 0.904150698 | 2.020962951 | 0.0432836 |
| HLA-C | ENST00000606010.1 | ENSG00000272249.1 | -0.84562166 | -1.901657557 | 0.057215938 |
| HLA-C | ENST00000606855.1 | ENSG00000245937.3 | 0.806196209 | 1.825538239 | 0.067919846 |
| HLA-C | ENST00000606869.1 | ENSG00000272349.1 | -0.825111934 | -1.867137477 | 0.061882392 |
| HLA-C | ENST00000607119.1 | ENSG00000272541.1 | -0.842781542 | -1.891823707 | 0.058514473 |
| HLA-C | ENST00000607476.1 | ENSG00000272540.1 | 0.829479538 | 1.856352218 | 0.063403371 |
| HLA-C | ENST00000607549.1 | ENSG00000272293.1 | -0.801622138 | -1.810788594 | 0.070173583 |
| HLA-C | ENST00000607943.1 | ENSG00000273188.1 | 0.905738511 | 2.047844416 | 0.040575247 |
| HLA-C | ENST00000608259.1 | ENSG00000272627.1 | -0.903921944 | -2.033344426 | 0.042017738 |
| HLA-C | ENST00000608465.1 | ENSG00000272758.1 | -0.836803108 | -1.858750355 | 0.063062532 |
| HLA-C | ENST00000608489.1 | ENSG00000272716.1 | 0.951073239 | 2.130460294 | 0.033133631 |
| HLA-C | ENST00000609113.1 | ENSG00000272827.1 | 0.973847034 | 2.188330976 | 0.028645502 |
| HLA-C | ENST00000609807.1 | ENSG00000272700.1 | -0.845168417 | -1.893085682 | 0.058346476 |
| HLA-C | ENST00000609976.1 | ENSG00000272582.1 | 0.936795547 | 2.086917761 | 0.036895569 |
| HLA-C | ENST00000610270.1 | ENSG00000272576.1 | -0.96224553 | -2.158705884 | 0.030872992 |
| HLA-C | NR_027052.1 | THAP7-AS1 | 0.882178669 | 1.971636459 | 0.048651122 |
| HLA-C | NR_027402.1 | FAM223B | 0.851398696 | 1.890076129 | 0.058747779 |
| HLA-C | NR_038421.1 | LINC01220 | 0.973272144 | 2.144516524 | 0.031991523 |
| HLA-C | NR_038923.1 | SSSCA1-AS1 | 0.903997527 | 2.01929783 | 0.043456273 |
| HLA-C | NR_046871.1 | LINC00333 | -0.864553439 | -1.92758351 | 0.053906952 |
| HLA-C | NR_073552.1 | LOC101059948 | 0.833369345 | 1.852180541 | 0.063999902 |
| HLA-C | NR_108106.1 | LINC01135 | 0.821000168 | 1.822595236 | 0.068364718 |
| HLA-C | NR_109831.1 | RASSF1-AS1 | 0.822112317 | 1.84412082 | 0.065165534 |
| HLA-C | NR_109877.1 | LINC01470 | -0.899800033 | -2.023963218 | 0.042973936 |
| HLA-C | NR_110117.1 | LOC101927769 | -0.878927771 | -1.961722195 | 0.049794836 |
| HLA-C | NR_110245.1 | LOC101929282 | -0.905758728 | -2.065173108 | 0.038906624 |
| HLA-C | NR_110556.1 | LOC102724890 | -0.971321558 | -2.197279768 | 0.028000472 |
| HLA-C | NR_110630.1 | LOC101927478 | 0.909912948 | 2.056088107 | 0.039774024 |
| HLA-C | NR_111951.1 | LINC00869 | 0.910867771 | 2.015061186 | 0.043898239 |
| HLA-C | NR_111952.1 | LINC00869 | 0.904764796 | 2.004909491 | 0.044972724 |
| HLA-C | NR_111953.1 | LINC00869 | 0.907102731 | 2.046978959 | 0.040660151 |
| HLA-C | NR_125849.1 | LOC101928140 | -0.885346739 | -1.967038269 | 0.049178799 |
| HLA-C | NR_133930.1 | LOC105375556 | -0.813425212 | -1.830763302 | 0.06713588 |
| HLA-C | NR_135040.1 | LOC101927038 | 0.936541708 | 2.10997614 | 0.034860411 |
| HLA-C | NR_135041.1 | LOC101927038 | 0.919545802 | 2.046338689 | 0.04072306 |
| HLA-C | NR_135584.1 | LOC101927596 | 0.87516039 | 1.996444951 | 0.045885512 |

| | | | | | |
|---|---|---|---|---|---|
| HLA-C | NR_144459.1 | ARSD-AS1 | 0.800665957 | 1.827690827 | 0.067595966 |
| HLA-DPA1 | ENST00000318291.4 | ENSG00000177406.4 | 0.803610287 | 1.781732511 | 0.074792862 |
| HLA-DPA1 | ENST00000421207.1 | ENSG00000231768.1 | 0.880994053 | 1.995124117 | 0.046029345 |
| HLA-DPA1 | ENST00000423667.1 | ENSG00000225970.1 | 0.922849701 | 2.048757441 | 0.040485839 |
| HLA-DPA1 | ENST00000425124.1 | ENSG00000232336.1 | 0.896499763 | 2.00759774 | 0.044686055 |
| HLA-DPA1 | ENST00000425624.1 | ENSG00000223779.4 | 0.989910805 | 2.211526276 | 0.026999414 |
| HLA-DPA1 | ENST00000426699.1 | ENSG00000229308.1 | 0.964503755 | 2.14917594 | 0.031620456 |
| HLA-DPA1 | ENST00000432314.1 | ENSG00000231532.1 | 0.870666739 | 1.963955886 | 0.049535208 |
| HLA-DPA1 | ENST00000433344.1 | ENSG00000234083.1 | -0.950084743 | -2.125729746 | 0.033525769 |
| HLA-DPA1 | ENST00000435434.1 | ENSG00000231233.1 | 0.852598805 | 1.917633064 | 0.055157555 |
| HLA-DPA1 | ENST00000435992.2 | ENSG00000232675.3 | 0.901992026 | 2.003993586 | 0.045070747 |
| HLA-DPA1 | ENST00000436982.2 | ENSG00000235335.2 | -0.911162516 | -2.04975452 | 0.040388392 |
| HLA-DPA1 | ENST00000438107.1 | ENSG00000234449.2 | 0.800064466 | 1.796502148 | 0.072414691 |
| HLA-DPA1 | ENST00000439186.1 | ENSG00000237076.1 | 0.896064967 | 2.018762875 | 0.043511871 |
| HLA-DPA1 | ENST00000440492.1 | ENSG00000233975.1 | 0.840861328 | 1.876660306 | 0.060564666 |
| HLA-DPA1 | ENST00000447514.1 | ENSG00000236753.1 | 0.825678539 | 1.84561026 | 0.064948816 |
| HLA-DPA1 | ENST00000450848.1 | ENSG00000225539.1 | 0.82343143 | 1.819590729 | 0.068821355 |
| HLA-DPA1 | ENST00000454100.1 | ENSG00000236943.2 | 0.84214775 | 1.919463007 | 0.054925764 |
| HLA-DPA1 | ENST00000455699.1 | ENSG00000240996.1 | 0.878726129 | 1.966522992 | 0.049238229 |
| HLA-DPA1 | ENST00000455788.1 | ENSG00000236263.1 | 0.857507816 | 1.927938137 | 0.053862821 |
| HLA-DPA1 | ENST00000458154.1 | ENSG00000235578.1 | 0.834871209 | 1.852025421 | 0.064022173 |
| HLA-DPA1 | ENST00000458661.2 | ENSG00000236467.3 | 0.8656582 | 1.948903229 | 0.051306982 |
| HLA-DPA1 | ENST00000484413.1 | ENSG00000271853.1 | 0.900417181 | 2.03926927 | 0.041423161 |
| HLA-DPA1 | ENST00000494509.1 | ENSG00000240095.1 | 0.86304925 | 1.954028515 | 0.050697845 |
| HLA-DPA1 | ENST00000504891.1 | ENSG00000249388.1 | 0.920426537 | 2.098509679 | 0.035860146 |
| HLA-DPA1 | ENST00000505498.1 | ENSG00000250908.1 | 0.872638888 | 1.968773669 | 0.048979087 |
| HLA-DPA1 | ENST00000506791.1 | ENSG00000251131.1 | 0.821869476 | 1.842095372 | 0.065461201 |
| HLA-DPA1 | ENST00000507373.1 | ENSG00000250072.1 | 0.815069353 | 1.781589398 | 0.074816215 |
| HLA-DPA1 | ENST00000508083.1 | ENSG00000249343.1 | 0.812253973 | 1.814707643 | 0.06956885 |
| HLA-DPA1 | ENST00000509192.1 | ENSG00000250765.1 | 0.853216559 | 1.925714697 | 0.054140008 |
| HLA-DPA1 | ENST00000518473.1 | ENSG00000253985.1 | 0.965574858 | 2.160229228 | 0.030754929 |
| HLA-DPA1 | ENST00000521307.1 | ENSG00000253177.1 | 0.887387111 | 2.002139537 | 0.045269726 |
| HLA-DPA1 | ENST00000521953.1 | ENSG00000253214.1 | 0.828669166 | 1.8652292 | 0.062149284 |
| HLA-DPA1 | ENST00000522704.1 | ENSG00000254135.1 | 0.859882525 | 1.925605976 | 0.054153592 |
| HLA-DPA1 | ENST00000524818.1 | ENSG00000254473.1 | 0.835140157 | 1.882989439 | 0.059701799 |
| HLA-DPA1 | ENST00000525133.1 | ENSG00000255375.1 | 0.813289088 | 1.803114085 | 0.0713703 |
| HLA-DPA1 | ENST00000526186.1 | ENSG00000254510.1 | 0.850970822 | 1.902905269 | 0.057052907 |
| HLA-DPA1 | ENST00000526935.1 | ENSG00000255372.1 | 0.838554894 | 1.859406126 | 0.062969594 |
| HLA-DPA1 | ENST00000528887.1 | ENSG00000254501.1 | 0.809051322 | 1.824444767 | 0.068084859 |
| HLA-DPA1 | ENST00000532680.1 | ENSG00000255458.1 | 0.846347286 | 1.904472607 | 0.056848661 |
| HLA-DPA1 | ENST00000547834.1 | ENSG00000258325.1 | 0.93108295 | 2.07726614 | 0.037777002 |
| HLA-DPA1 | ENST00000549140.1 | ENSG00000258332.1 | 0.841401068 | 1.921679105 | 0.054646148 |
| HLA-DPA1 | ENST00000554679.1 | ENSG00000258837.1 | 0.864680543 | 1.915893659 | 0.055378633 |
| HLA-DPA1 | ENST00000554859.1 | ENSG00000259088.1 | 0.813525823 | 1.824236339 | 0.06811635 |
| HLA-DPA1 | ENST00000556397.1 | ENSG00000258654.1 | 0.820478114 | 1.821681917 | 0.068503263 |
| HLA-DPA1 | ENST00000556786.1 | ENSG00000258525.1 | -0.879007442 | -1.992535988 | 0.046312281 |
| HLA-DPA1 | ENST00000558896.1 | ENSG00000259176.1 | 0.84965152 | 1.887097512 | 0.059147211 |
| HLA-DPA1 | ENST00000559003.1 | ENSG00000259520.1 | 0.905523221 | 2.028811854 | 0.042477457 |
| HLA-DPA1 | ENST00000563841.1 | ENSG00000261029.1 | 0.90519741 | 2.032499977 | 0.042103066 |
| HLA-DPA1 | ENST00000564038.1 | ENSG00000261760.2 | 0.815770241 | 1.847795711 | 0.064631902 |
| HLA-DPA1 | ENST00000565965.1 | ENSG00000261172.1 | 0.888320172 | 1.999255128 | 0.045580757 |

| | | | | | |
|---|---|---|---|---|---|
| HLA-DPA1 | ENST00000567395.1 | ENSG00000261090.1 | 0.916167252 | 2.039545509 | 0.041395614 |
| HLA-DPA1 | ENST00000569981.1 | ENSG00000238045.5 | 0.874633105 | 1.973999216 | 0.048381832 |
| HLA-DPA1 | ENST00000570493.2 | ENSG00000261898.2 | 0.858347251 | 1.914213928 | 0.055592827 |
| HLA-DPA1 | ENST00000570512.1 | ENSG00000262768.1 | 0.870629862 | 1.950955383 | 0.051062354 |
| HLA-DPA1 | ENST00000574365.1 | ENSG00000262837.1 | 0.844083933 | 1.874642458 | 0.060841928 |
| HLA-DPA1 | ENST00000577698.1 | ENSG00000265100.1 | 0.839956604 | 1.880629508 | 0.060022335 |
| HLA-DPA1 | ENST00000578265.1 | ENSG00000214719.7 | 0.814989195 | 1.791382515 | 0.073231933 |
| HLA-DPA1 | ENST00000582558.1 | ENSG00000264569.1 | 0.807594446 | 1.813975966 | 0.069681426 |
| HLA-DPA1 | ENST00000584705.1 | ENSG00000264569.1 | 0.891794919 | 1.968240231 | 0.049040403 |
| HLA-DPA1 | ENST00000584758.1 | ENSG00000265356.1 | 0.830864617 | 1.85111123 | 0.064153553 |
| HLA-DPA1 | ENST00000585559.1 | ENSG00000267117.1 | 0.929763589 | 2.094216859 | 0.036240667 |
| HLA-DPA1 | ENST00000588799.1 | ENSG00000267275.1 | 0.947201602 | 2.113131734 | 0.034589494 |
| HLA-DPA1 | ENST00000588945.1 | ENSG00000267275.1 | 0.96864671 | 2.156205849 | 0.031067595 |
| HLA-DPA1 | ENST00000589777.1 | ENSG00000261040.2 | 0.803722525 | 1.802996749 | 0.071388725 |
| HLA-DPA1 | ENST00000592400.1 | ENSG00000267735.1 | 0.879547789 | 1.963837642 | 0.049548923 |
| HLA-DPA1 | ENST00000592720.1 | ENSG00000267232.1 | 0.838452375 | 1.904612855 | 0.056830414 |
| HLA-DPA1 | ENST00000593861.1 | ENSG00000231898.4 | 0.808139916 | 1.826187026 | 0.067822095 |
| HLA-DPA1 | ENST00000597530.1 | ENSG00000228401.3 | 0.840422569 | 1.863144944 | 0.062441876 |
| HLA-DPA1 | ENST00000597550.1 | ENSG00000269051.1 | 0.826906429 | 1.820337296 | 0.068707655 |
| HLA-DPA1 | ENST00000599143.1 | ENSG00000269349.1 | 0.825635168 | 1.871937039 | 0.061215315 |
| HLA-DPA1 | ENST00000599259.1 | ENSG00000269352.1 | 0.828396239 | 1.868655596 | 0.061670746 |
| HLA-DPA1 | ENST00000600489.1 | ENSG00000231898.4 | 0.895910575 | 2.039762838 | 0.041373953 |
| HLA-DPA1 | ENST00000601692.1 | ENSG00000267874.1 | -0.981378276 | -2.204698808 | 0.027475237 |
| HLA-DPA1 | ENST00000601735.1 | ENSG00000244513.2 | 0.827542697 | 1.872401685 | 0.061151052 |
| HLA-DPA1 | ENST00000602809.1 | ENSG00000270105.1 | -0.807945186 | -1.785347519 | 0.074204966 |
| HLA-DPA1 | ENST00000602872.1 | ENSG00000270067.1 | 0.800626791 | 1.786005669 | 0.07409834 |
| HLA-DPA1 | ENST00000602954.1 | ENSG00000269906.1 | 0.836751397 | 1.875691101 | 0.060697709 |
| HLA-DPA1 | ENST00000606377.1 | ENSG00000272286.1 | -0.829836097 | -1.868818426 | 0.06164808 |
| HLA-DPA1 | ENST00000606457.1 | ENSG00000271830.1 | 0.829596019 | 1.833078169 | 0.066790947 |
| HLA-DPA1 | ENST00000606470.1 | ENSG00000271913.1 | 0.880974295 | 1.966331513 | 0.049260329 |
| HLA-DPA1 | ENST00000607284.1 | ENSG00000272389.1 | 0.96001368 | 2.126961933 | 0.033423246 |
| HLA-DPA1 | ENST00000607839.1 | ENSG00000272030.1 | 0.850139977 | 1.861447311 | 0.062681035 |
| HLA-DPA1 | ENST00000609701.1 | ENSG00000273284.1 | 0.811704374 | 1.822202457 | 0.068424272 |
| HLA-DPA1 | ENST00000609725.1 | ENSG00000231898.4 | 0.860631688 | 1.931550639 | 0.053414993 |
| HLA-DPA1 | ENST00000609972.1 | ENSG00000230651.3 | 0.9605797 | 2.150328159 | 0.031529267 |
| HLA-DPA1 | ENST00000610161.1 | ENSG00000273059.1 | 0.951545391 | 2.13536845 | 0.032730926 |
| HLA-DPA1 | NR_003605.1 | ZFAS1 | 0.843866459 | 1.894906004 | 0.058104856 |
| HLA-DPA1 | NR_028324.1 | LINC01002 | 0.858719104 | 1.927246125 | 0.053948964 |
| HLA-DPA1 | NR_044996.1 | HCG23 | 0.96706279 | 2.184622254 | 0.028916555 |
| HLA-DPA1 | NR_072981.1 | LINC00957 | 0.839343314 | 1.873944578 | 0.060938064 |
| HLA-DPA1 | NR_072982.1 | LINC00957 | 0.84368058 | 1.907797267 | 0.056417424 |
| HLA-DPA1 | NR_108036.1 | CFAP58-AS1 | 0.844214288 | 1.89177406 | 0.058521091 |
| HLA-DPA1 | NR_110480.1 | LOC101927079 | 0.802144982 | 1.795210741 | 0.07262013 |
| HLA-DPA1 | NR_120335.1 | LOC101928414 | 0.954005091 | 2.129811132 | 0.033187209 |
| HLA-DPA1 | NR_120655.1 | KCNMA1-AS1 | 0.822792816 | 1.827188066 | 0.067671498 |
| HLA-DPA1 | NR_126166.1 | FAM74A7 | 0.989079977 | 2.198829658 | 0.027890037 |
| HLA-DPA1 | NR_134325.1 | LOC102723672 | 0.840526834 | 1.870555113 | 0.061406771 |
| HLA-DPA1 | NR_134597.1 | LOC105378068 | 0.842640838 | 1.900936796 | 0.057310292 |
| HLA-DPA1 | NR_135258.1 | LOC105370489 | 0.851208519 | 1.908031145 | 0.056387191 |
| HLA-DPA1 | NR_138084.1 | HCG24 | 0.856924676 | 1.930121461 | 0.05359179 |
| HLA-DQA1 | ENST00000362684.1 | ENSG00000228549.2 | -0.900227979 | -2.017079518 | 0.043687216 |

| | | | | | |
|---|---|---|---|---|---|
| HLA-DQA1 | ENST00000412896.1 | ENSG00000197585.5 | -0.803817038 | -1.775236631 | 0.075858824 |
| HLA-DQA1 | ENST00000413564.1 | ENSG00000224500.1 | -0.82628721 | -1.841204557 | 0.065591588 |
| HLA-DQA1 | ENST00000413989.1 | ENSG00000242628.1 | 0.92302186 | 2.041315658 | 0.041219462 |
| HLA-DQA1 | ENST00000417315.1 | ENSG00000242486.1 | -0.909189102 | -2.033526505 | 0.041999359 |
| HLA-DQA1 | ENST00000421498.1 | ENSG00000237978.1 | 0.931189675 | 2.103895194 | 0.03538759 |
| HLA-DQA1 | ENST00000421597.1 | ENSG00000227851.1 | 0.900976375 | 2.035277441 | 0.041822965 |
| HLA-DQA1 | ENST00000424342.1 | ENSG00000234988.1 | -0.829706726 | -1.824801373 | 0.068031009 |
| HLA-DQA1 | ENST00000425058.1 | ENSG00000226771.1 | 0.968582484 | 2.172640522 | 0.029807384 |
| HLA-DQA1 | ENST00000426125.1 | ENSG00000223653.1 | 0.974932725 | 2.166686866 | 0.030258739 |
| HLA-DQA1 | ENST00000426929.1 | ENSG00000230184.1 | -0.923713541 | -2.058531942 | 0.039539098 |
| HLA-DQA1 | ENST00000427064.1 | ENSG00000238031.1 | 0.830394837 | 1.879731281 | 0.06014471 |
| HLA-DQA1 | ENST00000429878.1 | ENSG00000224184.1 | -0.817729469 | -1.807348401 | 0.070707974 |
| HLA-DQA1 | ENST00000429916.1 | ENSG00000227708.1 | 0.877374461 | 1.965011501 | 0.049412906 |
| HLA-DQA1 | ENST00000430247.1 | ENSG00000232855.2 | 0.817398348 | 1.825539773 | 0.067919615 |
| HLA-DQA1 | ENST00000430545.1 | ENSG00000237153.1 | 0.833812724 | 1.879005326 | 0.060243766 |
| HLA-DQA1 | ENST00000430842.1 | ENSG00000230433.1 | -0.894913951 | -2.010498418 | 0.044378463 |
| HLA-DQA1 | ENST00000432265.1 | ENSG00000231170.1 | -0.80275101 | -1.777882153 | 0.075423212 |
| HLA-DQA1 | ENST00000433249.1 | ENSG00000236556.1 | 0.881967282 | 1.989879767 | 0.046604182 |
| HLA-DQA1 | ENST00000434292.1 | ENSG00000229796.1 | 0.802903441 | 1.789829549 | 0.073481317 |
| HLA-DQA1 | ENST00000434493.1 | ENSG00000224605.1 | 0.918211754 | 2.061750831 | 0.039231464 |
| HLA-DQA1 | ENST00000435271.1 | ENSG00000231132.1 | -0.860291985 | -1.923435053 | 0.054425436 |
| HLA-DQA1 | ENST00000435357.1 | ENSG00000225444.1 | 0.916227892 | 2.038485927 | 0.041501361 |
| HLA-DQA1 | ENST00000435828.1 | ENSG00000235612.1 | 0.876289691 | 1.952848497 | 0.05083755 |
| HLA-DQA1 | ENST00000436582.1 | ENSG00000236525.1 | -0.826614145 | -1.86477064 | 0.06221356 |
| HLA-DQA1 | ENST00000439529.1 | ENSG00000236526.1 | 0.928603467 | 2.086499419 | 0.036933408 |
| HLA-DQA1 | ENST00000441295.1 | ENSG00000233960.1 | -0.854886425 | -1.896730038 | 0.057863577 |
| HLA-DQA1 | ENST00000441991.1 | ENSG00000231210.2 | -0.817422894 | -1.821986621 | 0.068457016 |
| HLA-DQA1 | ENST00000442852.1 | ENSG00000237923.1 | -0.880591155 | -1.958691151 | 0.050148967 |
| HLA-DQA1 | ENST00000443066.2 | ENSG00000237633.2 | 0.892185443 | 2.000109552 | 0.045488436 |
| HLA-DQA1 | ENST00000443162.1 | ENSG00000234183.1 | 0.960772582 | 2.157082489 | 0.030999238 |
| HLA-DQA1 | ENST00000444731.1 | ENSG00000227131.1 | -0.887562016 | -1.959809294 | 0.050018085 |
| HLA-DQA1 | ENST00000445178.1 | ENSG00000234653.1 | 0.965470103 | 2.178922 | 0.029337463 |
| HLA-DQA1 | ENST00000445233.1 | ENSG00000233928.1 | 0.815663516 | 1.813298982 | 0.06978572 |
| HLA-DQA1 | ENST00000445617.2 | ENSG00000225751.2 | 0.863275677 | 1.948504545 | 0.051354622 |
| HLA-DQA1 | ENST00000448086.1 | ENSG00000237571.1 | -0.928758032 | -2.061853351 | 0.0392217 |
| HLA-DQA1 | ENST00000449903.1 | ENSG00000223872.1 | -0.964511001 | -2.169787364 | 0.030022958 |
| HLA-DQA1 | ENST00000451267.1 | ENSG00000230410.1 | -0.87285866 | -1.936742693 | 0.052776804 |
| HLA-DQA1 | ENST00000451828.1 | ENSG00000228549.2 | -0.873362893 | -1.962688029 | 0.049682435 |
| HLA-DQA1 | ENST00000452511.1 | ENSG00000231876.3 | 0.850421288 | 1.889215272 | 0.058862989 |
| HLA-DQA1 | ENST00000453584.1 | ENSG00000233613.1 | -0.922563155 | -2.06990231 | 0.038461494 |
| HLA-DQA1 | ENST00000453878.1 | ENSG00000224850.1 | 0.87383453 | 1.937945561 | 0.052629865 |
| HLA-DQA1 | ENST00000456999.1 | ENSG00000230690.1 | 0.867178535 | 1.953750162 | 0.050730771 |
| HLA-DQA1 | ENST00000457043.1 | ENSG00000231365.1 | 0.871642845 | 1.932640189 | 0.053280538 |
| HLA-DQA1 | ENST00000457632.1 | ENSG00000234248.1 | 0.869278147 | 1.942814612 | 0.052038562 |
| HLA-DQA1 | ENST00000457856.1 | ENSG00000228549.2 | -0.864488449 | -1.940344813 | 0.052337799 |
| HLA-DQA1 | ENST00000477643.1 | ENSG00000241224.2 | -0.838267109 | -1.886279315 | 0.059257325 |
| HLA-DQA1 | ENST00000479039.1 | ENSG00000241224.2 | -0.93375626 | -2.108084153 | 0.035023711 |
| HLA-DQA1 | ENST00000482142.1 | ENSG00000243276.1 | 0.872344644 | 1.956733044 | 0.050378863 |
| HLA-DQA1 | ENST00000483283.1 | ENSG00000240571.1 | 0.816969049 | 1.82283789 | 0.068327947 |
| HLA-DQA1 | ENST00000488040.1 | ENSG00000243176.1 | 0.916909153 | 2.032549774 | 0.04209803 |
| HLA-DQA1 | ENST00000500498.2 | ENSG00000245311.2 | -0.827317709 | -1.860397097 | 0.062829365 |

| | | | | | |
|---|---|---|---|---|---|
| HLA-DQA1 | ENST00000502421.1 | ENSG00000250284.1 | -0.808558894 | -1.835960875 | 0.066363443 |
| HLA-DQA1 | ENST00000503323.1 | ENSG00000249881.1 | -0.842932187 | -1.885507433 | 0.059361362 |
| HLA-DQA1 | ENST00000503470.1 | ENSG00000248559.1 | 0.912313787 | 2.050145747 | 0.04035021 |
| HLA-DQA1 | ENST00000503987.1 | ENSG00000250075.1 | 0.905120112 | 2.028554576 | 0.042503679 |
| HLA-DQA1 | ENST00000505404.1 | ENSG00000249941.1 | -0.937680937 | -2.119845406 | 0.034019085 |
| HLA-DQA1 | ENST00000505575.1 | ENSG00000248939.1 | -0.885088995 | -1.979020985 | 0.047813644 |
| HLA-DQA1 | ENST00000505978.1 | ENSG00000249982.1 | -0.912047554 | -2.04006869 | 0.041343485 |
| HLA-DQA1 | ENST00000506305.1 | ENSG00000249994.1 | -0.829091349 | -1.87115672 | 0.061323362 |
| HLA-DQA1 | ENST00000506379.1 | ENSG00000240152.2 | 0.921468124 | 2.043870724 | 0.04096632 |
| HLA-DQA1 | ENST00000506420.1 | ENSG00000250034.1 | -0.808535854 | -1.805054333 | 0.07106618 |
| HLA-DQA1 | ENST00000506852.1 | ENSG00000250945.1 | 0.982692757 | 2.204946941 | 0.027457818 |
| HLA-DQA1 | ENST00000507558.1 | ENSG00000248445.1 | 0.80017783 | 1.79979957 | 0.071892292 |
| HLA-DQA1 | ENST00000508925.2 | ENSG00000249196.2 | -0.812948717 | -1.821537886 | 0.068525133 |
| HLA-DQA1 | ENST00000509098.1 | ENSG00000250863.1 | 0.806920644 | 1.77039473 | 0.076661407 |
| HLA-DQA1 | ENST00000509629.1 | ENSG00000250164.1 | -0.804101943 | -1.808374756 | 0.070548194 |
| HLA-DQA1 | ENST00000509983.1 | ENSG00000248173.1 | -0.8886172 | -1.978006631 | 0.047927959 |
| HLA-DQA1 | ENST00000510001.2 | ENSG00000249196.2 | -0.938583901 | -2.111910823 | 0.034694099 |
| HLA-DQA1 | ENST00000510602.1 | ENSG00000249122.1 | -0.854001199 | -1.885422462 | 0.059372824 |
| HLA-DQA1 | ENST00000511603.1 | ENSG00000249892.1 | 0.942899201 | 2.11118417 | 0.034756485 |
| HLA-DQA1 | ENST00000518260.1 | ENSG00000253628.1 | -0.86173455 | -1.923002982 | 0.054479675 |
| HLA-DQA1 | ENST00000518620.1 | ENSG00000253892.1 | -0.869488218 | -1.926505578 | 0.054041276 |
| HLA-DQA1 | ENST00000518894.1 | ENSG00000204758.3 | 0.835286512 | 1.854512075 | 0.063665934 |
| HLA-DQA1 | ENST00000519005.1 | ENSG00000253507.1 | 0.855954681 | 1.877441814 | 0.060457565 |
| HLA-DQA1 | ENST00000519375.1 | ENSG00000253980.1 | 0.954322943 | 2.112900219 | 0.034609309 |
| HLA-DQA1 | ENST00000519695.1 | ENSG00000253507.1 | 0.818669403 | 1.811474408 | 0.070067447 |
| HLA-DQA1 | ENST00000519844.1 | ENSG00000253824.1 | -0.929030599 | -2.060120118 | 0.039387059 |
| HLA-DQA1 | ENST00000520192.1 | ENSG00000253807.1 | 0.810787305 | 1.79977264 | 0.071896546 |
| HLA-DQA1 | ENST00000520849.1 | ENSG00000253553.1 | 0.850540847 | 1.896146594 | 0.057940663 |
| HLA-DQA1 | ENST00000521490.1 | ENSG00000253407.1 | -0.951701772 | -2.099359556 | 0.035785217 |
| HLA-DQA1 | ENST00000521725.1 | ENSG00000253396.1 | 0.848134127 | 1.918215332 | 0.055083713 |
| HLA-DQA1 | ENST00000522190.1 | ENSG00000254165.1 | 0.929369194 | 2.068608197 | 0.038582868 |
| HLA-DQA1 | ENST00000522408.1 | ENSG00000253484.1 | 0.813479404 | 1.821935506 | 0.068464772 |
| HLA-DQA1 | ENST00000524133.1 | ENSG00000253174.2 | -0.900457908 | -2.007468001 | 0.044699854 |
| HLA-DQA1 | ENST00000524275.1 | ENSG00000253507.1 | 0.828515319 | 1.840676922 | 0.065668918 |
| HLA-DQA1 | ENST00000525855.1 | ENSG00000254746.1 | -0.90163663 | -2.039986893 | 0.041351631 |
| HLA-DQA1 | ENST00000533101.1 | ENSG00000255311.1 | 0.816352003 | 1.842156363 | 0.065452281 |
| HLA-DQA1 | ENST00000533938.1 | ENSG00000255142.1 | -0.890094899 | -1.994257758 | 0.046123893 |
| HLA-DQA1 | ENST00000535324.1 | ENSG00000255968.1 | 0.839503172 | 1.88379494 | 0.059592718 |
| HLA-DQA1 | ENST00000538294.1 | ENSG00000250748.2 | -0.879874747 | -1.997996471 | 0.045717043 |
| HLA-DQA1 | ENST00000545158.1 | ENSG00000256011.1 | 0.883073971 | 1.970424029 | 0.048789794 |
| HLA-DQA1 | ENST00000546135.1 | ENSG00000256670.1 | -0.922665005 | -2.067454549 | 0.038691343 |
| HLA-DQA1 | ENST00000548748.1 | ENSG00000258252.1 | -0.929551085 | -2.049902262 | 0.040373969 |
| HLA-DQA1 | ENST00000549616.1 | ENSG00000258168.1 | 0.801733193 | 1.813001147 | 0.069831644 |
| HLA-DQA1 | ENST00000550805.1 | ENSG00000244306.5 | -0.943239996 | -2.088180153 | 0.036781587 |
| HLA-DQA1 | ENST00000551067.1 | ENSG00000257891.1 | -0.860913718 | -1.920412206 | 0.054805853 |
| HLA-DQA1 | ENST00000552634.1 | ENSG00000257496.1 | -0.828044829 | -1.848020976 | 0.064599308 |
| HLA-DQA1 | ENST00000553348.1 | ENSG00000258829.1 | -0.912218255 | -2.035019261 | 0.041848935 |
| HLA-DQA1 | ENST00000553537.1 | ENSG00000258481.1 | -0.906363292 | -2.012622894 | 0.044154317 |
| HLA-DQA1 | ENST00000553668.1 | ENSG00000258733.1 | 0.851419115 | 1.88309365 | 0.059687678 |
| HLA-DQA1 | ENST00000553954.1 | ENSG00000259052.1 | -0.900936391 | -2.007962996 | 0.044647224 |
| HLA-DQA1 | ENST00000555689.1 | ENSG00000259049.1 | -0.898747917 | -2.017417586 | 0.043651954 |

| | | | | | |
|---|---|---|---|---|---|
| HLA-DQA1 | ENST00000556145.1 | ENSG00000258829.1 | -0.944439219 | -2.105968902 | 0.035207054 |
| HLA-DQA1 | ENST00000556458.1 | ENSG00000258504.2 | -0.873099041 | -1.98009198 | 0.047693194 |
| HLA-DQA1 | ENST00000556978.1 | ENSG00000258693.1 | 0.918743903 | 2.060494788 | 0.039351264 |
| HLA-DQA1 | ENST00000557855.1 | ENSG00000259176.1 | 0.804492018 | 1.806171463 | 0.070891561 |
| HLA-DQA1 | ENST00000558141.1 | ENSG00000259594.1 | 0.87034628 | 1.945215116 | 0.051749093 |
| HLA-DQA1 | ENST00000559041.1 | ENSG00000259713.1 | 0.897394116 | 2.007788679 | 0.044665752 |
| HLA-DQA1 | ENST00000560969.1 | ENSG00000259176.1 | 0.980409679 | 2.187960828 | 0.028672456 |
| HLA-DQA1 | ENST00000561254.1 | ENSG00000259554.1 | 0.849798132 | 1.898451996 | 0.057636566 |
| HLA-DQA1 | ENST00000561847.1 | ENSG00000260293.1 | -0.859684968 | -1.930787049 | 0.053509393 |
| HLA-DQA1 | ENST00000563601.1 | ENSG00000260589.1 | -0.818235832 | -1.852145377 | 0.06400495 |
| HLA-DQA1 | ENST00000565271.1 | ENSG00000261335.1 | -0.951868211 | -2.120026548 | 0.034003807 |
| HLA-DQA1 | ENST00000565623.1 | ENSG00000261118.1 | -0.855088707 | -1.943267075 | 0.051983897 |
| HLA-DQA1 | ENST00000569215.1 | ENSG00000260756.1 | 0.804825869 | 1.827029955 | 0.067695266 |
| HLA-DQA1 | ENST00000569328.1 | ENSG00000261638.1 | 0.908800618 | 2.044687896 | 0.040885638 |
| HLA-DQA1 | ENST00000569778.1 | ENSG00000260823.1 | -0.831827496 | -1.868227281 | 0.061730398 |
| HLA-DQA1 | ENST00000570700.1 | ENSG00000263011.1 | -0.860352567 | -1.917442518 | 0.055181737 |
| HLA-DQA1 | ENST00000573260.1 | ENSG00000262482.1 | 0.816431581 | 1.836950974 | 0.066217133 |
| HLA-DQA1 | ENST00000581996.1 | ENSG00000265778.1 | 0.801276055 | 1.765287961 | 0.077515382 |
| HLA-DQA1 | ENST00000582895.1 | ENSG00000264729.1 | -0.931682186 | -2.091994305 | 0.036439025 |
| HLA-DQA1 | ENST00000586297.1 | ENSG00000267633.1 | 0.8049857 | 1.807632644 | 0.070663694 |
| HLA-DQA1 | ENST00000587049.1 | ENSG00000235535.3 | -0.891597551 | -2.000771476 | 0.045417023 |
| HLA-DQA1 | ENST00000588177.1 | ENSG00000234899.5 | 0.867664606 | 1.944857152 | 0.051792173 |
| HLA-DQA1 | ENST00000591137.1 | ENSG00000267405.1 | 0.833954342 | 1.856511679 | 0.06338066 |
| HLA-DQA1 | ENST00000592431.1 | ENSG00000267475.1 | -0.878570991 | -1.968211911 | 0.04904366 |
| HLA-DQA1 | ENST00000592523.1 | ENSG00000226994.3 | 0.978278116 | 2.198076474 | 0.027943657 |
| HLA-DQA1 | ENST00000595007.1 | ENSG00000231876.3 | 0.866063889 | 1.916263927 | 0.05533151 |
| HLA-DQA1 | ENST00000597755.1 | ENSG00000236194.2 | 0.87257024 | 1.959817175 | 0.050017163 |
| HLA-DQA1 | ENST00000598065.1 | ENSG00000231731.3 | -0.864724153 | -1.917231353 | 0.055208547 |
| HLA-DQA1 | ENST00000598131.1 | ENSG00000269043.1 | 0.804906339 | 1.786929735 | 0.073948846 |
| HLA-DQA1 | ENST00000598356.1 | ENSG00000269640.1 | 0.904936131 | 2.028756233 | 0.042483125 |
| HLA-DQA1 | ENST00000598887.1 | ENSG00000268475.1 | -0.921555921 | -2.088502483 | 0.036752532 |
| HLA-DQA1 | ENST00000598950.1 | ENSG00000269736.1 | 0.896807083 | 2.008238148 | 0.044617991 |
| HLA-DQA1 | ENST00000599050.1 | ENSG00000268366.1 | 0.833630296 | 1.855721123 | 0.063493318 |
| HLA-DQA1 | ENST00000599387.1 | ENSG00000227733.4 | 0.817020199 | 1.821504972 | 0.068530132 |
| HLA-DQA1 | ENST00000600959.1 | ENSG00000269303.1 | 0.834734744 | 1.85438035 | 0.063684764 |
| HLA-DQA1 | ENST00000601420.1 | ENSG00000269560.1 | -0.810612359 | -1.821970935 | 0.068459396 |
| HLA-DQA1 | ENST00000602405.1 | ENSG00000269928.1 | -0.880994012 | -1.960936124 | 0.049886475 |
| HLA-DQA1 | ENST00000602443.1 | ENSG00000270076.1 | 0.912556008 | 2.057927868 | 0.039597058 |
| HLA-DQA1 | ENST00000602881.1 | ENSG00000269965.1 | 0.832379802 | 1.847669489 | 0.06465017 |
| HLA-DQA1 | ENST00000602900.1 | ENSG00000270179.1 | -0.824987669 | -1.83177906 | 0.066984344 |
| HLA-DQA1 | ENST00000603612.1 | ENSG00000270996.1 | 0.898712574 | 2.023371157 | 0.043034896 |
| HLA-DQA1 | ENST00000603949.1 | ENSG00000270332.1 | 0.806218212 | 1.816415258 | 0.069306696 |
| HLA-DQA1 | ENST00000604312.1 | ENSG00000270947.1 | 0.847999317 | 1.885655312 | 0.059341418 |
| HLA-DQA1 | ENST00000605021.1 | ENSG00000271401.1 | 0.941122633 | 2.117467544 | 0.034220187 |
| HLA-DQA1 | ENST00000606898.1 | ENSG00000272094.1 | -0.80485161 | -1.830162691 | 0.067225615 |
| HLA-DQA1 | ENST00000607594.1 | ENSG00000271766.1 | 0.840904428 | 1.894331566 | 0.058181014 |
| HLA-DQA1 | ENST00000608133.1 | ENSG00000273193.1 | 0.923888103 | 2.076560645 | 0.037842128 |
| HLA-DQA1 | ENST00000608173.1 | ENSG00000197099.4 | 0.846801412 | 1.929938756 | 0.053614427 |
| HLA-DQA1 | ENST00000608289.1 | ENSG00000272958.1 | -0.889490847 | -1.998888015 | 0.045620472 |
| HLA-DQA1 | ENST00000609182.1 | ENSG00000273248.1 | -0.871829429 | -1.951718222 | 0.050971668 |
| HLA-DQA1 | ENST00000609238.1 | ENSG00000272703.1 | -0.95033221 | -2.106486798 | 0.035162089 |

| | | | | | |
|---|---|---|---|---|---|
| HLA-DQA1 | ENST00000609955.1 | ENSG00000273275.1 | 0.819137156 | 1.84394546 | 0.065191089 |
| HLA-DQA1 | NR_026932.1 | PDCD4-AS1 | -0.817191532 | -1.830110153 | 0.067233469 |
| HLA-DQA1 | NR_027067.1 | LINC00114 | -0.870128638 | -1.942057652 | 0.052130121 |
| HLA-DQA1 | NR_027425.1 | FAM66D | -0.87786214 | -1.977007519 | 0.04804078 |
| HLA-DQA1 | NR_046556.1 | RBMS3-AS1 | 0.901453504 | 2.019751037 | 0.043409218 |
| HLA-DQA1 | NR_046748.1 | ARHGAP31-AS1 | 0.862915685 | 1.912024784 | 0.055873015 |
| HLA-DQA1 | NR_102737.1 | LINC00911 | -0.846388337 | -1.866240897 | 0.06200767 |
| HLA-DQA1 | NR_102738.1 | LINC00911 | -0.942825311 | -2.136737329 | 0.032619362 |
| HLA-DQA1 | NR_110284.1 | LOC101927907 | 0.965470103 | 2.136744483 | 0.03261878 |
| HLA-DQA1 | NR_110504.1 | LOC101929572 | -0.926012611 | -2.088222567 | 0.036777763 |
| HLA-DQA1 | NR_110731.1 | LINC01232 | -0.852554826 | -1.91039861 | 0.05608191 |
| HLA-DQA1 | NR_120330.1 | LOC101928227 | -0.805393761 | -1.807339908 | 0.070709297 |
| HLA-DQA1 | NR_120466.1 | LINC01489 | 0.962015462 | 2.13491351 | 0.032768076 |
| HLA-DQA1 | NR_125769.1 | LINC01269 | -0.907506397 | -2.020765641 | 0.043304031 |
| HLA-DQA1 | NR_126041.1 | LOC101930071 | -0.801590627 | -1.76370205 | 0.077782156 |
| HLA-DQA1 | NR_126354.1 | LINC01331 | -0.833616924 | -1.840122327 | 0.06575028 |
| HLA-DQA1 | NR_126409.1 | LINC00376 | 0.913636762 | 2.040969127 | 0.041253896 |
| HLA-DQA1 | NR_130916.1 | LOC105274304 | -0.960111653 | -2.156202378 | 0.031067866 |
| HLA-DQA1 | NR_131243.1 | SMCR2 | -0.929103879 | -2.050027061 | 0.04036179 |
| HLA-DQA1 | NR_133907.1 | HLA-DQB1-AS1 | 0.865742618 | 1.937452039 | 0.052690111 |
| HLA-DQA1 | NR_134610.1 | LOC105375014 | -0.873636518 | -1.965334721 | 0.049375509 |
| HLA-DQA1 | NR_134632.1 | LOC105373051 | -0.854236174 | -1.904585876 | 0.056833924 |
| HLA-DQA1 | NR_135076.1 | LOC102723838 | 0.814638517 | 1.79416304 | 0.07278715 |
| HLA-DQA1 | NR_135239.1 | LINC01867 | 0.922165702 | 2.077407513 | 0.037763963 |
| HLA-DRB1 | ENST00000295549.4 | ENSG00000163364.5 | 0.831578328 | 1.864788814 | 0.062211012 |
| HLA-DRB1 | ENST00000413645.1 | ENSG00000228798.1 | 0.91996992 | 2.083841907 | 0.03717455 |
| HLA-DRB1 | ENST00000416657.1 | ENSG00000235858.1 | 0.822805557 | 1.834457271 | 0.066586145 |
| HLA-DRB1 | ENST00000418972.1 | ENSG00000225044.1 | -0.911310284 | -2.01021916 | 0.044407998 |
| HLA-DRB1 | ENST00000420315.1 | ENSG00000228072.1 | 0.82205208 | 1.81317383 | 0.069805015 |
| HLA-DRB1 | ENST00000420465.1 | ENSG00000167355.3 | 0.817701426 | 1.825702797 | 0.067895042 |
| HLA-DRB1 | ENST00000420498.1 | ENSG00000224985.1 | 0.852255162 | 1.903856126 | 0.056928924 |
| HLA-DRB1 | ENST00000420981.2 | ENSG00000230438.5 | 0.860173696 | 1.909703713 | 0.056171373 |
| HLA-DRB1 | ENST00000424181.1 | ENSG00000224977.1 | 0.817421841 | 1.815143857 | 0.069501805 |
| HLA-DRB1 | ENST00000425371.2 | ENSG00000235872.2 | 0.812300351 | 1.816477028 | 0.069297229 |
| HLA-DRB1 | ENST00000426444.1 | ENSG00000239395.1 | 0.824563312 | 1.82949898 | 0.067324892 |
| HLA-DRB1 | ENST00000426653.1 | ENSG00000235704.1 | 0.941502356 | 2.111289647 | 0.034747423 |
| HLA-DRB1 | ENST00000428853.2 | ENSG00000229206.2 | 0.868289873 | 1.938701445 | 0.052537703 |
| HLA-DRB1 | ENST00000429608.1 | ENSG00000237480.1 | 0.81697245 | 1.824631141 | 0.068056711 |
| HLA-DRB1 | ENST00000429630.1 | ENSG00000232533.1 | 0.908007863 | 2.023512453 | 0.043020341 |
| HLA-DRB1 | ENST00000433036.1 | ENSG00000228989.1 | 0.802796256 | 1.782139136 | 0.074726545 |
| HLA-DRB1 | ENST00000437330.1 | ENSG00000229203.1 | 0.924883669 | 2.07271463 | 0.038198845 |
| HLA-DRB1 | ENST00000441029.2 | ENSG00000229188.2 | -0.813443137 | -1.839889938 | 0.065784398 |
| HLA-DRB1 | ENST00000443306.1 | ENSG00000233891.3 | 0.825472111 | 1.857298602 | 0.063268683 |
| HLA-DRB1 | ENST00000448365.1 | ENSG00000231114.1 | 0.894937214 | 2.009604649 | 0.044473049 |
| HLA-DRB1 | ENST00000454928.1 | ENSG00000186148.7 | 0.856671733 | 1.92216996 | 0.054584375 |
| HLA-DRB1 | ENST00000472596.1 | ENSG00000239774.1 | 0.838365956 | 1.888562527 | 0.058950472 |
| HLA-DRB1 | ENST00000476892.1 | ENSG00000241345.1 | 0.952046112 | 2.131258419 | 0.033067859 |
| HLA-DRB1 | ENST00000502300.1 | ENSG00000249451.1 | 0.934710154 | 2.0870614 | 0.036882585 |
| HLA-DRB1 | ENST00000507997.1 | ENSG00000250551.1 | -0.921664373 | -2.045395247 | 0.040815907 |
| HLA-DRB1 | ENST00000508188.1 | ENSG00000250999.1 | 0.818055032 | 1.820094629 | 0.068744595 |
| HLA-DRB1 | ENST00000508241.1 | ENSG00000248518.1 | 0.97231373 | 2.172117269 | 0.029846819 |

| | | | | | |
|---|---|---|---|---|---|
| HLA-DRB1 | ENST00000515750.1 | ENSG00000249061.1 | -0.894856093 | -1.987901154 | 0.046822623 |
| HLA-DRB1 | ENST00000519451.1 | ENSG00000253363.1 | 0.828774016 | 1.869053696 | 0.061615344 |
| HLA-DRB1 | ENST00000522524.1 | ENSG00000253342.1 | -0.841931597 | -1.876036894 | 0.060650214 |
| HLA-DRB1 | ENST00000524073.1 | ENSG00000253774.1 | 0.862403532 | 1.953526526 | 0.050757237 |
| HLA-DRB1 | ENST00000526154.1 | ENSG00000254511.1 | 0.867316246 | 1.948540666 | 0.051350304 |
| HLA-DRB1 | ENST00000526694.1 | ENSG00000231999.2 | 0.851784593 | 1.877648497 | 0.060429267 |
| HLA-DRB1 | ENST00000531627.1 | ENSG00000254584.1 | 0.919449147 | 2.032397388 | 0.042113442 |
| HLA-DRB1 | ENST00000532249.1 | ENSG00000234899.5 | 0.811952319 | 1.838440607 | 0.065997506 |
| HLA-DRB1 | ENST00000532688.1 | ENSG00000255441.1 | 0.898367432 | 2.007440256 | 0.044702806 |
| HLA-DRB1 | ENST00000535720.1 | ENSG00000256364.1 | 0.842493639 | 1.906312131 | 0.056609721 |
| HLA-DRB1 | ENST00000535746.1 | ENSG00000256101.1 | 0.977512595 | 2.162240569 | 0.030599638 |
| HLA-DRB1 | ENST00000536412.1 | ENSG00000256072.1 | -0.845582429 | -1.898819706 | 0.057588185 |
| HLA-DRB1 | ENST00000537921.1 | ENSG00000255966.1 | 0.829670792 | 1.879215852 | 0.060215026 |
| HLA-DRB1 | ENST00000545642.1 | ENSG00000256342.1 | 0.905417731 | 2.020763519 | 0.043304251 |
| HLA-DRB1 | ENST00000547750.1 | ENSG00000257886.1 | 0.905619974 | 2.016087821 | 0.043790793 |
| HLA-DRB1 | ENST00000548199.1 | ENSG00000257614.1 | 0.813974055 | 1.803752856 | 0.071270059 |
| HLA-DRB1 | ENST00000549303.1 | ENSG00000257180.1 | -0.878054068 | -1.986169375 | 0.04701452 |
| HLA-DRB1 | ENST00000554197.1 | ENSG00000197176.3 | 0.938700233 | 2.142288656 | 0.032170261 |
| HLA-DRB1 | ENST00000557232.1 | ENSG00000259054.1 | 0.923442465 | 2.06516316 | 0.038907565 |
| HLA-DRB1 | ENST00000558515.1 | ENSG00000259182.1 | 0.882732158 | 1.967578958 | 0.049116503 |
| HLA-DRB1 | ENST00000560522.1 | ENSG00000259661.1 | 0.830757748 | 1.864861856 | 0.06220077 |
| HLA-DRB1 | ENST00000561215.1 | ENSG00000259611.1 | 0.918991324 | 2.081011768 | 0.037432829 |
| HLA-DRB1 | ENST00000562582.1 | ENSG00000259779.1 | 0.800607683 | 1.787949511 | 0.073784154 |
| HLA-DRB1 | ENST00000567127.1 | ENSG00000260264.1 | -0.836210766 | -1.872158808 | 0.061184636 |
| HLA-DRB1 | ENST00000568836.1 | ENSG00000259967.1 | 0.870794688 | 1.943598612 | 0.051943873 |
| HLA-DRB1 | ENST00000570919.1 | ENSG00000263321.1 | 0.837486005 | 1.845842682 | 0.064915051 |
| HLA-DRB1 | ENST00000572222.1 | ENSG00000261971.2 | -0.800750102 | -1.81867314 | 0.068961312 |
| HLA-DRB1 | ENST00000578334.1 | ENSG00000265148.1 | 0.806043419 | 1.82060384 | 0.068667099 |
| HLA-DRB1 | ENST00000586399.1 | ENSG00000228430.4 | 0.931839496 | 2.102674291 | 0.035494251 |
| HLA-DRB1 | ENST00000589233.1 | ENSG00000231616.4 | 0.840801959 | 1.885515453 | 0.05936028 |
| HLA-DRB1 | ENST00000589817.1 | ENSG00000231616.4 | 0.927288779 | 2.06546934 | 0.038878614 |
| HLA-DRB1 | ENST00000590368.1 | ENSG00000231616.4 | 0.97912245 | 2.207918978 | 0.027249922 |
| HLA-DRB1 | ENST00000590813.1 | ENSG00000231616.4 | 0.954847372 | 2.127800194 | 0.033353653 |
| HLA-DRB1 | ENST00000590995.1 | ENSG00000267198.1 | 0.95768549 | 2.154643684 | 0.031189728 |
| HLA-DRB1 | ENST00000591217.1 | ENSG00000231616.4 | 0.935522364 | 2.07941158 | 0.037579537 |
| HLA-DRB1 | ENST00000592622.1 | ENSG00000267546.2 | -0.955977779 | -2.115946124 | 0.03434939 |
| HLA-DRB1 | ENST00000593269.1 | ENSG00000236172.2 | 0.946159713 | 2.143946215 | 0.032037197 |
| HLA-DRB1 | ENST00000593642.1 | ENSG00000267858.1 | 0.823920538 | 1.847316966 | 0.064701215 |
| HLA-DRB1 | ENST00000594492.1 | ENSG00000250910.3 | 0.962415101 | 2.155324963 | 0.031136414 |
| HLA-DRB1 | ENST00000596135.1 | ENSG00000269843.1 | 0.822620593 | 1.799442529 | 0.071948707 |
| HLA-DRB1 | ENST00000596497.1 | ENSG00000268530.1 | 0.883128622 | 1.968294399 | 0.049034174 |
| HLA-DRB1 | ENST00000598735.1 | ENSG00000268093.1 | 0.833668986 | 1.85900892 | 0.063025874 |
| HLA-DRB1 | ENST00000602485.1 | ENSG00000270163.1 | -0.801118941 | -1.805490462 | 0.070997967 |
| HLA-DRB1 | ENST00000602614.1 | ENSG00000269957.1 | -0.834544396 | -1.863593123 | 0.062378864 |
| HLA-DRB1 | ENST00000602773.1 | ENSG00000270160.1 | -0.967126968 | -2.151413509 | 0.031443576 |
| HLA-DRB1 | ENST00000606841.1 | ENSG00000272411.1 | 0.867453777 | 1.934226211 | 0.053085321 |
| HLA-DRB1 | ENST00000607044.1 | ENSG00000272247.1 | 0.925181036 | 2.10500341 | 0.035291011 |
| HLA-DRB1 | ENST00000607201.1 | ENSG00000272024.1 | -0.846368289 | -1.881963993 | 0.059840905 |
| HLA-DRB1 | ENST00000608264.1 | ENSG00000273473.1 | 0.854118779 | 1.917040329 | 0.055232808 |
| HLA-DRB1 | ENST00000609837.1 | ENSG00000273106.1 | 0.928980374 | 2.091232349 | 0.036507241 |
| HLA-DRB1 | ENST00000609953.1 | ENSG00000272825.1 | 0.898222464 | 2.023261775 | 0.043046166 |

| | | | | | |
|---|---|---|---|---|---|
| HLA-DRB1 | ENST00000610185.1 | ENSG00000273355.1 | -0.844046812 | -1.882251073 | 0.059801934 |
| HLA-DRB1 | NR_027401.2 | FAM223A | 0.886370352 | 1.972585082 | 0.048542854 |
| HLA-DRB1 | NR_028325.1 | LOC100132062 | 0.855257268 | 1.915450338 | 0.055435097 |
| HLA-DRB1 | NR_046578.1 | CACNA1C-AS4 | 0.87776755 | 1.974094557 | 0.048370992 |
| HLA-DRB1 | NR_046713.1 | NAALADL2-AS2 | -0.854569754 | -1.92892412 | 0.053740283 |
| HLA-DRB1 | NR_046839.1 | AGBL4-IT1 | -0.849942532 | -1.894343995 | 0.058179365 |
| HLA-DRB1 | NR_047040.1 | LINC00424 | 0.849916662 | 1.915561653 | 0.055420914 |
| HLA-DRB1 | NR_049776.1 | GPC5-AS1 | 0.819374294 | 1.845948033 | 0.064899751 |
| HLA-DRB1 | NR_103857.1 | SP2-AS1 | -0.854739988 | -1.939076586 | 0.052492013 |
| HLA-DRB1 | NR_110053.1 | LOC101927464 | 0.905417731 | 2.002014229 | 0.045283201 |
| HLA-DRB1 | NR_120527.1 | LOC100506675 | 0.873569933 | 1.952363309 | 0.050895086 |
| HLA-DRB1 | NR_121661.1 | ZBTB20-AS5 | -0.963555564 | -2.160272523 | 0.030751579 |
| HLA-DRB1 | NR_135820.1 | LOC102723727 | 0.916007762 | 2.06562989 | 0.03886344 |
| HLA-DRB5 | ENST00000295549.4 | ENSG00000163364.5 | 0.881556687 | 1.960165278 | 0.049976475 |
| HLA-DRB5 | ENST00000413645.1 | ENSG00000228798.1 | 0.950553259 | 2.104989672 | 0.035292207 |
| HLA-DRB5 | ENST00000418972.1 | ENSG00000225044.1 | -0.811617696 | -1.797778759 | 0.072212075 |
| HLA-DRB5 | ENST00000419207.2 | ENSG00000231248.2 | -0.81304061 | -1.814610104 | 0.069583849 |
| HLA-DRB5 | ENST00000420044.1 | ENSG00000225956.1 | 0.834925018 | 1.866640526 | 0.061951804 |
| HLA-DRB5 | ENST00000420498.1 | ENSG00000224985.1 | 0.86832353 | 1.942975404 | 0.05201913 |
| HLA-DRB5 | ENST00000426653.1 | ENSG00000235704.1 | 0.960723266 | 2.107987457 | 0.035032074 |
| HLA-DRB5 | ENST00000428853.2 | ENSG00000229206.2 | 0.855512221 | 1.906211561 | 0.056622763 |
| HLA-DRB5 | ENST00000443123.1 | ENSG00000229457.1 | 0.832501906 | 1.885548914 | 0.059355767 |
| HLA-DRB5 | ENST00000449749.1 | ENSG00000230834.1 | 0.84743569 | 1.9125672 | 0.055803482 |
| HLA-DRB5 | ENST00000454928.1 | ENSG00000186148.7 | 0.853103387 | 1.909219321 | 0.056233805 |
| HLA-DRB5 | ENST00000476892.1 | ENSG00000241345.1 | 0.837294717 | 1.881874772 | 0.059853021 |
| HLA-DRB5 | ENST00000502300.1 | ENSG00000249451.1 | 0.918665426 | 2.06886784 | 0.03855849 |
| HLA-DRB5 | ENST00000507997.1 | ENSG00000250551.1 | -0.890007901 | -1.988650717 | 0.046739769 |
| HLA-DRB5 | ENST00000508241.1 | ENSG00000248518.1 | 0.834014283 | 1.851953302 | 0.064032529 |
| HLA-DRB5 | ENST00000512563.1 | ENSG00000249547.1 | 0.884392 | 2.002086423 | 0.045275437 |
| HLA-DRB5 | ENST00000515136.1 | ENSG00000251274.1 | -0.827433346 | -1.869807175 | 0.0615106 |
| HLA-DRB5 | ENST00000518339.1 | ENSG00000253470.1 | 0.803045374 | 1.796819163 | 0.072364333 |
| HLA-DRB5 | ENST00000522524.1 | ENSG00000253342.1 | -0.877547345 | -1.965010552 | 0.049413016 |
| HLA-DRB5 | ENST00000524824.1 | ENSG00000255328.1 | 0.86495938 | 1.937518528 | 0.052681991 |
| HLA-DRB5 | ENST00000535746.1 | ENSG00000256101.1 | 0.935907213 | 2.086708082 | 0.03691453 |
| HLA-DRB5 | ENST00000536412.1 | ENSG00000256072.1 | -0.863201729 | -1.940677073 | 0.052297459 |
| HLA-DRB5 | ENST00000536492.1 | ENSG00000256237.1 | -0.907659977 | -2.003287056 | 0.045146486 |
| HLA-DRB5 | ENST00000548199.1 | ENSG00000257614.1 | 0.845800221 | 1.878578839 | 0.060302023 |
| HLA-DRB5 | ENST00000549303.1 | ENSG00000257180.1 | -0.859873188 | -1.924030058 | 0.054350816 |
| HLA-DRB5 | ENST00000554197.1 | ENSG00000197176.3 | 0.876480818 | 1.972009989 | 0.048608466 |
| HLA-DRB5 | ENST00000557232.1 | ENSG00000259054.1 | 0.988456521 | 2.184123175 | 0.028953198 |
| HLA-DRB5 | ENST00000561215.1 | ENSG00000259611.1 | 0.910077281 | 2.031935924 | 0.042160143 |
| HLA-DRB5 | ENST00000562582.1 | ENSG00000259779.1 | 0.804942175 | 1.790455465 | 0.073380721 |
| HLA-DRB5 | ENST00000562834.1 | ENSG00000261116.1 | 0.881757636 | 1.980623345 | 0.047633529 |
| HLA-DRB5 | ENST00000568836.1 | ENSG00000259967.1 | 0.862217171 | 1.903402106 | 0.056988096 |
| HLA-DRB5 | ENST00000570919.1 | ENSG00000263321.1 | 0.895951259 | 1.97366917 | 0.048419373 |
| HLA-DRB5 | ENST00000572222.1 | ENSG00000261971.2 | -0.955347481 | -2.123266258 | 0.033731546 |
| HLA-DRB5 | ENST00000573953.1 | ENSG00000261971.2 | -0.919674831 | -2.02756852 | 0.042604305 |
| HLA-DRB5 | ENST00000577360.1 | ENSG00000264273.1 | 0.801575938 | 1.785983583 | 0.074101916 |
| HLA-DRB5 | ENST00000578334.1 | ENSG00000265148.1 | 0.894507021 | 1.972589075 | 0.048542399 |
| HLA-DRB5 | ENST00000583826.1 | ENSG00000265148.1 | 0.838677509 | 1.879226713 | 0.060213544 |
| HLA-DRB5 | ENST00000585703.1 | ENSG00000235779.3 | 0.839788456 | 1.888092077 | 0.05901359 |

| | | | | | |
|---|---|---|---|---|---|
| HLA-DRB5 | ENST00000586399.1 | ENSG00000228430.4 | 0.899417761 | 2.03733502 | 0.041616481 |
| HLA-DRB5 | ENST00000588334.1 | ENSG00000235779.3 | 0.89531672 | 2.01531191 | 0.043871978 |
| HLA-DRB5 | ENST00000590255.1 | ENSG00000235779.3 | 0.89736409 | 1.996312602 | 0.045899907 |
| HLA-DRB5 | ENST00000590368.1 | ENSG00000231616.4 | 0.872656461 | 1.924125417 | 0.054338865 |
| HLA-DRB5 | ENST00000590813.1 | ENSG00000231616.4 | 0.825689872 | 1.845973081 | 0.064896114 |
| HLA-DRB5 | ENST00000590995.1 | ENSG00000267198.1 | 0.952985634 | 2.126138381 | 0.033491739 |
| HLA-DRB5 | ENST00000591217.1 | ENSG00000231616.4 | 0.951130631 | 2.141666291 | 0.032220345 |
| HLA-DRB5 | ENST00000592622.1 | ENSG00000267546.2 | -0.90819279 | -2.024379639 | 0.042931105 |
| HLA-DRB5 | ENST00000593269.1 | ENSG00000236172.2 | 0.848536222 | 1.897176694 | 0.057804622 |
| HLA-DRB5 | ENST00000593824.1 | ENSG00000268184.1 | 0.867461034 | 1.940893419 | 0.052271206 |
| HLA-DRB5 | ENST00000594091.1 | ENSG00000232732.5 | -0.820118593 | -1.846293572 | 0.064849591 |
| HLA-DRB5 | ENST00000594492.1 | ENSG00000250910.3 | 0.817879137 | 1.821443648 | 0.068539445 |
| HLA-DRB5 | ENST00000596497.1 | ENSG00000268530.1 | 0.926797657 | 2.072374933 | 0.038230489 |
| HLA-DRB5 | ENST00000600512.1 | ENSG00000269752.1 | 0.81198982 | 1.792718322 | 0.073017975 |
| HLA-DRB5 | ENST00000600956.1 | ENSG00000232732.5 | -0.841203065 | -1.910425282 | 0.056078478 |
| HLA-DRB5 | ENST00000602773.1 | ENSG00000270160.1 | -0.960425408 | -2.140315785 | 0.032329255 |
| HLA-DRB5 | ENST00000606899.1 | ENSG00000272426.1 | 0.861037538 | 1.917917684 | 0.05512145 |
| HLA-DRB5 | ENST00000607044.1 | ENSG00000272247.1 | 0.976662401 | 2.174070451 | 0.029699845 |
| HLA-DRB5 | ENST00000609837.1 | ENSG00000273106.1 | 0.91764026 | 2.060539213 | 0.039347021 |
| HLA-DRB5 | ENST00000609953.1 | ENSG00000272825.1 | 0.81475999 | 1.816391343 | 0.069310362 |
| HLA-DRB5 | NR_027401.2 | FAM223A | 0.885006009 | 1.99401805 | 0.046150082 |
| HLA-DRB5 | NR_028325.1 | LOC100132062 | 0.881541412 | 1.979546661 | 0.047754492 |
| HLA-DRB5 | NR_040001.2 | LINC01116 | 0.821139868 | 1.826019705 | 0.067847294 |
| HLA-DRB5 | NR_046578.1 | CACNA1C-AS4 | 0.841562328 | 1.869424114 | 0.061563832 |
| HLA-DRB5 | NR_046766.1 | ATP2B2-IT2 | 0.805485597 | 1.780124654 | 0.075055563 |
| HLA-DRB5 | NR_049776.1 | GPC5-AS1 | 0.945812252 | 2.13283137 | 0.032938562 |
| HLA-DRB5 | NR_103857.1 | SP2-AS1 | -0.816446412 | -1.82768208 | 0.067597279 |
| HLA-DRB5 | NR_121661.1 | ZBTB20-AS5 | -0.82455184 | -1.867997195 | 0.061762462 |
| HLA-DRB5 | NR_131186.1 | LOC105377348 | 0.825920653 | 1.85257592 | 0.063943166 |
| HLA-DRB5 | NR_136178.1 | LOC101928166 | 0.84957788 | 1.894230224 | 0.058194458 |
| HSPA1A | ENST00000415205.1 | ENSG00000182057.4 | 0.883637638 | 1.984069869 | 0.047248052 |
| HSPA1A | ENST00000417260.1 | ENSG00000231734.4 | -0.935439746 | -2.068312578 | 0.03861064 |
| HSPA1A | ENST00000419662.1 | ENSG00000228265.1 | 0.912286328 | 2.062216283 | 0.039187149 |
| HSPA1A | ENST00000421207.1 | ENSG00000231768.1 | 0.807511665 | 1.802505076 | 0.071465977 |
| HSPA1A | ENST00000422763.1 | ENSG00000231131.2 | -0.806431175 | -1.832513857 | 0.066874899 |
| HSPA1A | ENST00000423428.1 | ENSG00000224048.1 | -0.907214256 | -2.024075636 | 0.04296237 |
| HSPA1A | ENST00000425124.1 | ENSG00000232336.1 | 0.948566296 | 2.112449405 | 0.034647921 |
| HSPA1A | ENST00000425881.1 | ENSG00000239636.1 | 0.817697147 | 1.852507639 | 0.063952962 |
| HSPA1A | ENST00000426237.2 | ENSG00000235527.2 | 0.835430533 | 1.897943059 | 0.057703583 |
| HSPA1A | ENST00000429080.1 | ENSG00000233047.1 | -0.819924633 | -1.847540525 | 0.06466884 |
| HSPA1A | ENST00000431730.1 | ENSG00000237401.2 | 0.877067374 | 1.9618108 | 0.049784516 |
| HSPA1A | ENST00000433035.1 | ENSG00000230483.1 | 0.817114791 | 1.814919313 | 0.06953631 |
| HSPA1A | ENST00000433051.1 | ENSG00000233193.1 | 0.90799305 | 2.030649288 | 0.042290584 |
| HSPA1A | ENST00000433905.2 | ENSG00000229299.2 | 0.808623108 | 1.800523092 | 0.071778081 |
| HSPA1A | ENST00000435287.1 | ENSG00000227220.1 | 0.842533055 | 1.881892448 | 0.05985062 |
| HSPA1A | ENST00000438190.1 | ENSG00000227214.2 | 0.882792977 | 1.990159456 | 0.046573373 |
| HSPA1A | ENST00000440595.1 | ENSG00000228265.1 | 0.940343823 | 2.074038615 | 0.038075725 |
| HSPA1A | ENST00000441592.2 | ENSG00000224078.8 | 0.873049411 | 1.946265595 | 0.051622844 |
| HSPA1A | ENST00000442649.1 | ENSG00000234089.1 | -0.809479994 | -1.809123727 | 0.070431784 |
| HSPA1A | ENST00000448858.1 | ENSG00000237734.1 | -0.816651727 | -1.794175187 | 0.072785211 |
| HSPA1A | ENST00000451507.1 | ENSG00000229539.1 | 0.9459683 | 2.113675708 | 0.034542975 |

| | | | | | |
|---|---|---|---|---|---|
| HSPA1A | ENST00000452176.1 | ENSG00000223659.1 | -0.909470056 | -2.036795017 | 0.041670588 |
| HSPA1A | ENST00000454100.1 | ENSG00000236943.2 | 0.85035308 | 1.898124833 | 0.05767964 |
| HSPA1A | ENST00000456091.1 | ENSG00000226985.1 | 0.828719993 | 1.837748712 | 0.066099442 |
| HSPA1A | ENST00000457371.1 | ENSG00000237401.2 | 0.8452598 | 1.907909988 | 0.056402851 |
| HSPA1A | ENST00000458154.1 | ENSG00000235578.1 | 0.867505578 | 1.93158168 | 0.053411159 |
| HSPA1A | ENST00000463255.1 | ENSG00000243305.1 | -0.850128796 | -1.868838976 | 0.06164522 |
| HSPA1A | ENST00000489077.1 | ENSG00000244198.1 | 0.956749511 | 2.154769785 | 0.031179854 |
| HSPA1A | ENST00000489090.1 | ENSG00000240045.1 | -0.823042709 | -1.846870639 | 0.064765891 |
| HSPA1A | ENST00000498693.1 | ENSG00000244198.1 | 0.83942744 | 1.857414853 | 0.063252155 |
| HSPA1A | ENST00000503723.1 | ENSG00000250472.1 | -0.911991195 | -2.059681184 | 0.039429029 |
| HSPA1A | ENST00000506791.1 | ENSG00000251131.1 | 0.943819242 | 2.117659989 | 0.034203874 |
| HSPA1A | ENST00000509036.1 | ENSG00000251131.1 | 0.866146314 | 1.910453452 | 0.056074854 |
| HSPA1A | ENST00000509192.1 | ENSG00000250765.1 | 0.957540452 | 2.122106548 | 0.033828792 |
| HSPA1A | ENST00000509453.1 | ENSG00000249145.1 | 0.980472696 | 2.17953025 | 0.0292923 |
| HSPA1A | ENST00000510570.1 | ENSG00000250438.1 | -0.867425668 | -1.937116386 | 0.052731118 |
| HSPA1A | ENST00000515128.1 | ENSG00000248215.1 | -0.846573672 | -1.893292429 | 0.058318991 |
| HSPA1A | ENST00000520603.1 | ENSG00000254001.1 | -0.976789273 | -2.195582892 | 0.028121812 |
| HSPA1A | ENST00000521653.1 | ENSG00000253301.1 | 0.861795179 | 1.929033823 | 0.053726663 |
| HSPA1A | ENST00000532454.1 | ENSG00000255120.1 | 0.80508339 | 1.809886482 | 0.070313393 |
| HSPA1A | ENST00000534178.1 | ENSG00000255120.1 | 0.818149823 | 1.850029762 | 0.06430926 |
| HSPA1A | ENST00000537850.1 | ENSG00000251002.3 | 0.811854213 | 1.819314143 | 0.068863517 |
| HSPA1A | ENST00000543403.1 | ENSG00000256684.1 | -0.811287292 | -1.806402403 | 0.070855506 |
| HSPA1A | ENST00000550263.1 | ENSG00000257605.1 | 0.939179843 | 2.096709954 | 0.036019259 |
| HSPA1A | ENST00000551699.1 | ENSG00000257467.1 | -0.812786338 | -1.823632144 | 0.068207704 |
| HSPA1A | ENST00000559959.1 | ENSG00000259396.1 | 0.83315128 | 1.898033486 | 0.057691671 |
| HSPA1A | ENST00000562191.1 | ENSG00000261292.1 | -0.838623639 | -1.898294377 | 0.057657314 |
| HSPA1A | ENST00000562995.1 | ENSG00000261253.1 | 0.85469638 | 1.909565556 | 0.056189174 |
| HSPA1A | ENST00000563611.1 | ENSG00000261583.1 | 0.849195448 | 1.892198236 | 0.058464573 |
| HSPA1A | ENST00000564809.1 | ENSG00000261471.1 | 0.804082523 | 1.796359955 | 0.072437288 |
| HSPA1A | ENST00000565955.1 | ENSG00000261055.1 | 0.83262276 | 1.850104456 | 0.064298496 |
| HSPA1A | ENST00000567395.1 | ENSG00000261090.1 | 0.834926047 | 1.873183767 | 0.061043013 |
| HSPA1A | ENST00000568332.1 | ENSG00000260256.1 | 0.813148868 | 1.797844521 | 0.07220165 |
| HSPA1A | ENST00000569981.1 | ENSG00000238045.5 | 0.859190819 | 1.941337921 | 0.052217303 |
| HSPA1A | ENST00000570493.2 | ENSG00000261898.2 | 0.89495273 | 1.960904797 | 0.04989013 |
| HSPA1A | ENST00000571660.1 | ENSG00000262848.1 | 0.892512694 | 1.987511316 | 0.046865763 |
| HSPA1A | ENST00000573315.1 | ENSG00000270168.1 | 0.816338949 | 1.800724762 | 0.071746273 |
| HSPA1A | ENST00000580622.1 | ENSG00000264634.1 | 0.836119115 | 1.862980383 | 0.062465026 |
| HSPA1A | ENST00000582044.1 | ENSG00000263715.2 | 0.917930999 | 2.052820885 | 0.040089952 |
| HSPA1A | ENST00000582558.1 | ENSG00000264569.1 | 0.962405085 | 2.161249543 | 0.030676068 |
| HSPA1A | ENST00000584705.1 | ENSG00000264569.1 | 0.876429528 | 1.975680715 | 0.04819095 |
| HSPA1A | ENST00000585559.1 | ENSG00000267117.1 | 0.859131267 | 1.906380691 | 0.056600832 |
| HSPA1A | ENST00000586348.1 | ENSG00000267198.1 | 0.804585198 | 1.794911164 | 0.072667855 |
| HSPA1A | ENST00000586694.1 | ENSG00000267141.1 | 0.860909121 | 1.964990772 | 0.049415306 |
| HSPA1A | ENST00000588380.1 | ENSG00000266990.1 | 0.925420283 | 2.095061129 | 0.036165559 |
| HSPA1A | ENST00000589395.1 | ENSG00000267143.1 | 0.945230863 | 2.117724373 | 0.034198417 |
| HSPA1A | ENST00000592400.1 | ENSG00000267735.1 | 0.862276291 | 1.937356129 | 0.052701825 |
| HSPA1A | ENST00000592525.1 | ENSG00000267214.1 | 0.835892863 | 1.891566394 | 0.058548777 |
| HSPA1A | ENST00000592816.1 | ENSG00000236172.2 | 0.803617039 | 1.787880419 | 0.073795303 |
| HSPA1A | ENST00000594776.1 | ENSG00000269807.1 | 0.814218976 | 1.827072208 | 0.067688913 |
| HSPA1A | ENST00000595478.1 | ENSG00000237031.3 | -0.864824691 | -1.915145661 | 0.05547393 |
| HSPA1A | ENST00000596091.1 | ENSG00000227733.4 | -0.934245873 | -2.113812279 | 0.034531304 |

| | | | | | |
|---|---|---|---|---|---|
| HSPA1A | ENST00000596971.1 | ENSG00000269463.1 | 0.825345903 | 1.833570858 | 0.066717721 |
| HSPA1A | ENST00000597169.1 | ENSG00000269720.1 | 0.86989187 | 1.97781447 | 0.04794964 |
| HSPA1A | ENST00000597906.1 | ENSG00000268566.1 | -0.86994857 | -1.950946202 | 0.051063446 |
| HSPA1A | ENST00000600726.1 | ENSG00000267858.1 | 0.8330342 | 1.846528459 | 0.064815511 |
| HSPA1A | ENST00000601735.1 | ENSG00000244513.2 | 0.811059341 | 1.827399097 | 0.067639785 |
| HSPA1A | ENST00000602594.1 | ENSG00000269930.1 | -0.90063778 | -2.008846856 | 0.044553377 |
| HSPA1A | ENST00000602949.1 | ENSG00000270030.1 | 0.906037821 | 2.00763045 | 0.044682576 |
| HSPA1A | ENST00000604142.1 | ENSG00000271308.1 | 0.939069488 | 2.082857351 | 0.037264228 |
| HSPA1A | ENST00000604183.1 | ENSG00000271185.1 | 0.884259235 | 1.954413372 | 0.050652351 |
| HSPA1A | ENST00000605082.1 | ENSG00000270426.1 | 0.875918209 | 1.972405235 | 0.048563365 |
| HSPA1A | ENST00000606277.1 | ENSG00000272145.1 | 0.841894767 | 1.878579985 | 0.060301866 |
| HSPA1A | ENST00000606377.1 | ENSG00000272286.1 | -0.882822866 | -1.979446852 | 0.047765718 |
| HSPA1A | ENST00000606441.1 | ENSG00000272277.1 | 0.898156209 | 2.027228726 | 0.042639027 |
| HSPA1A | ENST00000606470.1 | ENSG00000271913.1 | 0.849063631 | 1.886345056 | 0.059248471 |
| HSPA1A | ENST00000606743.1 | ENSG00000272221.1 | 0.988987713 | 2.214313239 | 0.026807239 |
| HSPA1A | ENST00000606909.1 | ENSG00000271821.1 | 0.984598628 | 2.178192687 | 0.029391694 |
| HSPA1A | ENST00000607148.1 | ENSG00000272477.1 | -0.830752534 | -1.868607394 | 0.061677456 |
| HSPA1A | ENST00000607476.1 | ENSG00000272540.1 | 0.913872961 | 2.035954925 | 0.041754882 |
| HSPA1A | ENST00000607943.1 | ENSG00000273188.1 | 0.9093534 | 2.044958924 | 0.040858908 |
| HSPA1A | ENST00000608088.1 | ENSG00000272632.1 | -0.802056837 | -1.807559431 | 0.070675097 |
| HSPA1A | ENST00000608258.1 | ENSG00000229042.2 | -0.800757596 | -1.789358253 | 0.073557138 |
| HSPA1A | ENST00000608367.1 | ENSG00000273361.1 | 0.822511938 | 1.822563134 | 0.068369584 |
| HSPA1A | ENST00000608489.1 | ENSG00000272716.1 | 0.832293434 | 1.851136444 | 0.064149926 |
| HSPA1A | ENST00000608759.1 | ENSG00000273464.1 | -0.807554132 | -1.814559593 | 0.069591617 |
| HSPA1A | NR_003605.1 | ZFAS1 | 0.832828178 | 1.896492034 | 0.057895012 |
| HSPA1A | NR_026774.1 | LINC00239 | 0.866814345 | 1.918504985 | 0.055047011 |
| HSPA1A | NR_026802.1 | FAM74A4 | 0.839657877 | 1.907478367 | 0.05645867 |
| HSPA1A | NR_026951.1 | LINC00324 | 0.813184644 | 1.807588554 | 0.070670561 |
| HSPA1A | NR_028324.1 | LINC01002 | 0.889806938 | 1.980650949 | 0.047630431 |
| HSPA1A | NR_031762.2 | HCP5B | 0.822841449 | 1.856733226 | 0.063349118 |
| HSPA1A | NR_037169.1 | LOC100507547 | 0.998427084 | 2.241591164 | 0.024987808 |
| HSPA1A | NR_037170.1 | LOC100507547 | 0.994658868 | 2.202730244 | 0.027613767 |
| HSPA1A | NR_038421.1 | LINC01220 | 0.811245278 | 1.815664265 | 0.069421889 |
| HSPA1A | NR_038923.1 | SSSCA1-AS1 | 0.815177451 | 1.802602528 | 0.07145066 |
| HSPA1A | NR_045114.1 | PVRL3-AS1 | -0.921636036 | -2.051953281 | 0.040174203 |
| HSPA1A | NR_072981.1 | LINC00957 | 0.979907946 | 2.202544075 | 0.027626899 |
| HSPA1A | NR_072982.1 | LINC00957 | 0.981604262 | 2.183661968 | 0.028987096 |
| HSPA1A | NR_105010.1 | LINC01333 | 0.846432252 | 1.933213698 | 0.053209878 |
| HSPA1A | NR_109885.1 | RALY-AS1 | 0.957194298 | 2.151124418 | 0.031466381 |
| HSPA1A | NR_109886.1 | RALY-AS1 | 0.91941838 | 2.103128839 | 0.035454509 |
| HSPA1A | NR_110245.1 | LOC101929282 | -0.887600674 | -1.975377447 | 0.04822533 |
| HSPA1A | NR_110630.1 | LOC101927478 | 0.906861041 | 2.020159783 | 0.043366816 |
| HSPA1A | NR_110941.1 | MIR762HG | 0.89546536 | 2.013413251 | 0.044071173 |
| HSPA1A | NR_110998.1 | FAM74A4 | 0.839657877 | 1.873933094 | 0.060939647 |
| HSPA1A | NR_125957.1 | LOC101928626 | -0.909470056 | -2.029331357 | 0.042424552 |
| HSPA1A | NR_126522.1 | EXOC3-AS1 | 0.849078724 | 1.90982915 | 0.056155215 |
| HSPA1A | NR_134252.1 | LOC105379030 | 0.844489474 | 1.891121071 | 0.058608184 |
| HSPA1A | NR_134520.1 | LOC727993 | 0.804992914 | 1.784632709 | 0.074320912 |
| HSPA1A | NR_135024.1 | LOC105369747 | 0.816409003 | 1.821287289 | 0.068563198 |
| HSPA1A | NR_135097.1 | LOC105369443 | -0.811287292 | -1.813291805 | 0.069786827 |
| HSPA1A | NR_135584.1 | LOC101927596 | 0.815480341 | 1.852283345 | 0.063985146 |

| | | | | | |
|---|---|---|---|---|---|
| HSPA1A | NR_144459.1 | ARSD-AS1 | 0.847434748 | 1.898973467 | 0.057567965 |
| IFITM1 | ENST00000340585.6 | ENSG00000249429.1 | 0.882623759 | 1.961161494 | 0.049860187 |
| IFITM1 | ENST00000412519.1 | ENSG00000227599.1 | 0.870755557 | 1.939957952 | 0.0523848 |
| IFITM1 | ENST00000412647.2 | ENSG00000232964.2 | 0.804768315 | 1.804367146 | 0.07117377 |
| IFITM1 | ENST00000413311.1 | ENSG00000226276.1 | 0.809943144 | 1.816000671 | 0.069370269 |
| IFITM1 | ENST00000416595.1 | ENSG00000223623.1 | -0.861588493 | -1.929671063 | 0.053647608 |
| IFITM1 | ENST00000419296.1 | ENSG00000204588.5 | 0.854256921 | 1.908298766 | 0.056352612 |
| IFITM1 | ENST00000424257.1 | ENSG00000231626.1 | 0.975003518 | 2.173785516 | 0.029721247 |
| IFITM1 | ENST00000424852.1 | ENSG00000229891.1 | 0.91497598 | 2.063014582 | 0.039111243 |
| IFITM1 | ENST00000426475.1 | ENSG00000239467.1 | -0.914427402 | -2.010583333 | 0.044369486 |
| IFITM1 | ENST00000429681.1 | ENSG00000235236.1 | 0.913393755 | 2.029524117 | 0.042404935 |
| IFITM1 | ENST00000430025.1 | ENSG00000233508.1 | 0.86879293 | 1.937737362 | 0.052655273 |
| IFITM1 | ENST00000431727.2 | ENSG00000234938.2 | -0.927950511 | -2.071847029 | 0.038279709 |
| IFITM1 | ENST00000432699.1 | ENSG00000233334.2 | -0.977236871 | -2.207879724 | 0.027252659 |
| IFITM1 | ENST00000433036.1 | ENSG00000228989.1 | -0.817930336 | -1.862969068 | 0.062466618 |
| IFITM1 | ENST00000439184.1 | ENSG00000233985.1 | 0.940402734 | 2.117755127 | 0.034195811 |
| IFITM1 | ENST00000441592.2 | ENSG00000224078.8 | -0.819545847 | -1.81953425 | 0.068829962 |
| IFITM1 | ENST00000442649.1 | ENSG00000234089.1 | 0.862979067 | 1.934186619 | 0.053090187 |
| IFITM1 | ENST00000448570.1 | ENSG00000224549.1 | -0.895727804 | -2.004177622 | 0.045051037 |
| IFITM1 | ENST00000450072.1 | ENSG00000228486.5 | -0.921705855 | -2.013649352 | 0.044046361 |
| IFITM1 | ENST00000450480.1 | ENSG00000231295.1 | 0.867656579 | 1.926640972 | 0.054024389 |
| IFITM1 | ENST00000451697.1 | ENSG00000233823.1 | 0.822212262 | 1.823690968 | 0.068198805 |
| IFITM1 | ENST00000504916.1 | ENSG00000248112.1 | 0.949989625 | 2.141893729 | 0.032202035 |
| IFITM1 | ENST00000510922.1 | ENSG00000250777.1 | 0.828963743 | 1.834500797 | 0.06657969 |
| IFITM1 | ENST00000514737.1 | ENSG00000250597.1 | -0.885390525 | -1.992584995 | 0.04630691 |
| IFITM1 | ENST00000521403.1 | ENSG00000253603.1 | 0.85878317 | 1.924386423 | 0.054306165 |
| IFITM1 | ENST00000530435.1 | ENSG00000254630.1 | -0.840771895 | -1.866293558 | 0.062000306 |
| IFITM1 | ENST00000534065.1 | ENSG00000254458.1 | -0.926157426 | -2.062446811 | 0.039165217 |
| IFITM1 | ENST00000545254.1 | ENSG00000256633.1 | -0.876386141 | -1.958068101 | 0.050222022 |
| IFITM1 | ENST00000571404.1 | ENSG00000262370.1 | -0.866604335 | -1.928847067 | 0.053749851 |
| IFITM1 | ENST00000579154.1 | ENSG00000265908.1 | 0.96995383 | 2.138845377 | 0.032448193 |
| IFITM1 | ENST00000583067.1 | ENSG00000266126.1 | 0.95177408 | 2.108053858 | 0.035026331 |
| IFITM1 | ENST00000588182.2 | ENSG00000267453.2 | -0.857157632 | -1.91348534 | 0.055685948 |
| IFITM1 | ENST00000588290.1 | ENSG00000267751.1 | -0.81427583 | -1.811824318 | 0.070013346 |
| IFITM1 | ENST00000593139.1 | ENSG00000267042.1 | -0.82511871 | -1.832699065 | 0.066847336 |
| IFITM1 | ENST00000593218.1 | ENSG00000267421.2 | -0.925584017 | -2.087908151 | 0.036806121 |
| IFITM1 | ENST00000597256.1 | ENSG00000267986.1 | -0.848946887 | -1.912970877 | 0.055751781 |
| IFITM1 | ENST00000602741.1 | ENSG00000270061.1 | 0.835526087 | 1.877425196 | 0.060459841 |
| IFITM1 | ENST00000606963.1 | ENSG00000272010.1 | 0.867201005 | 1.908123738 | 0.056375225 |
| IFITM1 | ENST00000608677.1 | ENSG00000273350.1 | -0.856942406 | -1.936507432 | 0.052805583 |
| IFITM1 | NR_026713.1 | FAM182A | -0.881508886 | -1.979540536 | 0.04775518 |
| IFITM1 | NR_026813.1 | LINC00597 | 0.929120339 | 2.070065398 | 0.038446221 |
| IFITM1 | NR_046224.1 | LINC00659 | -0.893217205 | -1.999242401 | 0.045582133 |
| IFITM1 | NR_047116.1 | HIF1A-AS1 | 0.92311541 | 2.074749066 | 0.038009797 |
| IFITM1 | NR_051987.1 | LINC00499 | -0.80912018 | -1.802680586 | 0.071438393 |
| IFITM1 | NR_108077.1 | SMAD1-AS2 | 0.803355772 | 1.822891504 | 0.068319825 |
| IFITM1 | NR_110008.1 | ADNP-AS1 | -0.819857105 | -1.838076054 | 0.066051199 |
| IFITM1 | NR_110009.1 | ADNP-AS1 | -0.819857105 | -1.834188886 | 0.066625961 |
| IFITM1 | NR_120595.1 | LINC01315 | 0.927537227 | 2.112214689 | 0.034668039 |
| IFITM1 | NR_121577.1 | NALT1 | 0.825585619 | 1.854033522 | 0.063734364 |
| IFITM1 | NR_121624.1 | LOC103352541 | 0.84858648 | 1.892878886 | 0.058373978 |

| | | | | | |
|---|---|---|---|---|---|
| IFITM1 | NR_125774.1 | LINC01170 | -0.867856303 | -1.953783674 | 0.050726806 |
| IFITM1 | NR_131985.1 | CRAT8 | 0.884635274 | 1.990758564 | 0.046507437 |
| IFITM2 | ENST00000412519.1 | ENSG00000227599.1 | -0.846921444 | -1.897696204 | 0.057736113 |
| IFITM2 | ENST00000416595.1 | ENSG00000223623.1 | 0.839909175 | 1.874666619 | 0.060838602 |
| IFITM2 | ENST00000419296.1 | ENSG00000204588.5 | -0.851746666 | -1.877995792 | 0.060381741 |
| IFITM2 | ENST00000423380.1 | ENSG00000230537.1 | 0.840819091 | 1.866048761 | 0.062034544 |
| IFITM2 | ENST00000424257.1 | ENSG00000231626.1 | -0.967218826 | -2.145269275 | 0.031931324 |
| IFITM2 | ENST00000424852.1 | ENSG00000229891.1 | -0.873598601 | -1.998119213 | 0.045703737 |
| IFITM2 | ENST00000428391.1 | ENSG00000224691.1 | -0.825545093 | -1.834247908 | 0.066617203 |
| IFITM2 | ENST00000429681.1 | ENSG00000235236.1 | -0.868188067 | -1.916145891 | 0.055346528 |
| IFITM2 | ENST00000432699.1 | ENSG00000233334.2 | 0.939821818 | 2.08659657 | 0.036924618 |
| IFITM2 | ENST00000439072.1 | ENSG00000224516.1 | -0.835500542 | -1.861239032 | 0.062710429 |
| IFITM2 | ENST00000442649.1 | ENSG00000234089.1 | -0.802918425 | -1.827628837 | 0.067605275 |
| IFITM2 | ENST00000450072.1 | ENSG00000228486.5 | 0.836090022 | 1.873461095 | 0.06100474 |
| IFITM2 | ENST00000450480.1 | ENSG00000231295.1 | -0.852902673 | -1.893507486 | 0.058290414 |
| IFITM2 | ENST00000451697.1 | ENSG00000233823.1 | -0.841463315 | -1.873426081 | 0.061009571 |
| IFITM2 | ENST00000473329.1 | ENSG00000243849.1 | -0.90074404 | -2.035442642 | 0.041806354 |
| IFITM2 | ENST00000486285.1 | ENSG00000241818.1 | 0.946444521 | 2.114126224 | 0.034504488 |
| IFITM2 | ENST00000504916.1 | ENSG00000248112.1 | -0.833827707 | -1.876472799 | 0.060590386 |
| IFITM2 | ENST00000510922.1 | ENSG00000250777.1 | -0.807850568 | -1.801472649 | 0.071628414 |
| IFITM2 | ENST00000514737.1 | ENSG00000250597.1 | 0.894948409 | 1.962463136 | 0.049708588 |
| IFITM2 | ENST00000521403.1 | ENSG00000253603.1 | -0.889620379 | -1.979269748 | 0.047785644 |
| IFITM2 | ENST00000524808.1 | ENSG00000254812.1 | -0.884800821 | -1.947879609 | 0.05142937 |
| IFITM2 | ENST00000530435.1 | ENSG00000254630.1 | 0.838629339 | 1.866510622 | 0.061969959 |
| IFITM2 | ENST00000534065.1 | ENSG00000254458.1 | 0.827677029 | 1.830926314 | 0.067111542 |
| IFITM2 | ENST00000567261.1 | ENSG00000261320.1 | 0.812746433 | 1.827071008 | 0.067689094 |
| IFITM2 | ENST00000569998.1 | ENSG00000260975.1 | -0.80178921 | -1.778227357 | 0.075366522 |
| IFITM2 | ENST00000570022.1 | ENSG00000261399.1 | 0.949565572 | 2.091705018 | 0.036464911 |
| IFITM2 | ENST00000571404.1 | ENSG00000262370.1 | 0.808650964 | 1.811270095 | 0.070099052 |
| IFITM2 | ENST00000578443.1 | ENSG00000265204.1 | -0.901269654 | -2.031552996 | 0.042198929 |
| IFITM2 | ENST00000583067.1 | ENSG00000266126.1 | -0.910528483 | -2.030689921 | 0.042286459 |
| IFITM2 | ENST00000593218.1 | ENSG00000267421.2 | 0.830456331 | 1.86000937 | 0.0628842 |
| IFITM2 | ENST00000597357.1 | ENSG00000268309.1 | -0.913319303 | -2.031940261 | 0.042159704 |
| IFITM2 | ENST00000602051.1 | ENSG00000227877.2 | -0.847039604 | -1.886482862 | 0.059229916 |
| IFITM2 | ENST00000602741.1 | ENSG00000270061.1 | -0.901919707 | -2.010709691 | 0.04435613 |
| IFITM2 | ENST00000603533.1 | ENSG00000271384.1 | 0.868532486 | 1.938576737 | 0.052552899 |
| IFITM2 | ENST00000607991.1 | ENSG00000273076.1 | -0.92812441 | -2.057486127 | 0.039639488 |
| IFITM2 | ENST00000608934.1 | ENSG00000273063.1 | 0.828800048 | 1.864454897 | 0.06225785 |
| IFITM2 | NR_026713.1 | FAM182A | 0.965747296 | 2.18780429 | 0.028683861 |
| IFITM2 | NR_046224.1 | LINC00659 | 0.898541555 | 2.013123398 | 0.044101649 |
| IFITM2 | NR_120595.1 | LINC01315 | -0.846811816 | -1.908760083 | 0.056293048 |
| IFITM2 | NR_121577.1 | NALT1 | -0.926389665 | -2.079779929 | 0.037545723 |
| IFITM2 | NR_121624.1 | LOC103352541 | -0.802739368 | -1.80076391 | 0.0717401 |
| IFITM2 | NR_125759.1 | PKNOX2-AS1 | -0.878709213 | -1.978556314 | 0.047865982 |
| IFITM2 | NR_125774.1 | LINC01170 | 0.827097766 | 1.83613783 | 0.066337275 |
| IFITM2 | NR_131985.1 | CRAT8 | -0.840442428 | -1.861903884 | 0.062616639 |
| IFNB1 | ENST00000295549.4 | ENSG00000163364.5 | 0.964435783 | 2.181851159 | 0.029120519 |
| IFNB1 | ENST00000414098.2 | ENSG00000234428.2 | 0.815040623 | 1.834616017 | 0.066562604 |
| IFNB1 | ENST00000414896.1 | ENSG00000223374.1 | 0.817631644 | 1.836665182 | 0.066259338 |
| IFNB1 | ENST00000418621.1 | ENSG00000224731.1 | 0.850253939 | 1.887908436 | 0.059038244 |
| IFNB1 | ENST00000420044.1 | ENSG00000225956.1 | 0.91947359 | 2.074255401 | 0.038055597 |

| | | | | | |
|---|---|---|---|---|---|
| IFNB1 | ENST00000421597.1 | ENSG00000227851.1 | 0.801221273 | 1.787844264 | 0.073801137 |
| IFNB1 | ENST00000423796.1 | ENSG00000235146.2 | 0.938275067 | 2.093062937 | 0.036343537 |
| IFNB1 | ENST00000424274.1 | ENSG00000232120.1 | 0.854673618 | 1.913569176 | 0.055675227 |
| IFNB1 | ENST00000424342.1 | ENSG00000234988.1 | -0.832776287 | -1.876051222 | 0.060648247 |
| IFNB1 | ENST00000424678.1 | ENSG00000229600.1 | 0.871530996 | 1.963734832 | 0.049560851 |
| IFNB1 | ENST00000426444.1 | ENSG00000239395.1 | 0.928744543 | 2.111710506 | 0.034711287 |
| IFNB1 | ENST00000426653.1 | ENSG00000235704.1 | 0.881689975 | 1.952039271 | 0.050933542 |
| IFNB1 | ENST00000428160.1 | ENSG00000236897.1 | 0.895896223 | 2.010447644 | 0.044383832 |
| IFNB1 | ENST00000428853.2 | ENSG00000229206.2 | 0.968387522 | 2.167242607 | 0.03021636 |
| IFNB1 | ENST00000429666.1 | ENSG00000233755.1 | 0.852016412 | 1.893364403 | 0.058309426 |
| IFNB1 | ENST00000430247.1 | ENSG00000232855.2 | 0.863299325 | 1.910107779 | 0.056119338 |
| IFNB1 | ENST00000430751.1 | ENSG00000232222.1 | -0.872336888 | -1.960231633 | 0.049968723 |
| IFNB1 | ENST00000434292.1 | ENSG00000229796.1 | 0.891817306 | 1.992950912 | 0.046266823 |
| IFNB1 | ENST00000438173.2 | ENSG00000227733.4 | 0.862302523 | 1.926324653 | 0.054063849 |
| IFNB1 | ENST00000441029.2 | ENSG00000229188.2 | -0.924344947 | -2.057093225 | 0.039677259 |
| IFNB1 | ENST00000442017.1 | ENSG00000229660.1 | -0.872495364 | -1.940872666 | 0.052273724 |
| IFNB1 | ENST00000442852.1 | ENSG00000237923.1 | -0.900817167 | -2.004986955 | 0.044964442 |
| IFNB1 | ENST00000443123.1 | ENSG00000229457.1 | 0.812961117 | 1.813271131 | 0.069790014 |
| IFNB1 | ENST00000445233.1 | ENSG00000233928.1 | 0.921838325 | 2.060670269 | 0.039334508 |
| IFNB1 | ENST00000447111.1 | ENSG00000231903.1 | 0.976630964 | 2.173670541 | 0.029729887 |
| IFNB1 | ENST00000449749.1 | ENSG00000230834.1 | 0.89616988 | 1.991879335 | 0.046384299 |
| IFNB1 | ENST00000450227.1 | ENSG00000229941.1 | 0.862846594 | 1.946678057 | 0.051573344 |
| IFNB1 | ENST00000450696.1 | ENSG00000235146.2 | 0.815961547 | 1.854679147 | 0.063642058 |
| IFNB1 | ENST00000453584.1 | ENSG00000233613.1 | -0.820039662 | -1.834236504 | 0.066618895 |
| IFNB1 | ENST00000469846.2 | ENSG00000206573.4 | -0.862686561 | -1.925528434 | 0.054163282 |
| IFNB1 | ENST00000476892.1 | ENSG00000241345.1 | 0.808180935 | 1.823791054 | 0.068183667 |
| IFNB1 | ENST00000480904.2 | ENSG00000206573.4 | -0.919503074 | -2.048771835 | 0.040484431 |
| IFNB1 | ENST00000483283.1 | ENSG00000240571.1 | 0.926177028 | 2.09400984 | 0.036259104 |
| IFNB1 | ENST00000498199.1 | ENSG00000206573.4 | -0.911537323 | -2.045101753 | 0.040844827 |
| IFNB1 | ENST00000500498.2 | ENSG00000245311.2 | -0.900520648 | -2.011551847 | 0.0442672 |
| IFNB1 | ENST00000501133.2 | ENSG00000246560.2 | 0.881981545 | 1.986391146 | 0.046989909 |
| IFNB1 | ENST00000504755.1 | ENSG00000250252.1 | 0.810157738 | 1.809216461 | 0.070417381 |
| IFNB1 | ENST00000506420.1 | ENSG00000250034.1 | -0.813796988 | -1.806148363 | 0.070895168 |
| IFNB1 | ENST00000507997.1 | ENSG00000250551.1 | -0.917533563 | -2.036055977 | 0.041744735 |
| IFNB1 | ENST00000508191.1 | ENSG00000250910.3 | 0.8688983 | 1.931685905 | 0.053398286 |
| IFNB1 | ENST00000508823.1 | ENSG00000250716.1 | 0.905463335 | 2.022297841 | 0.043145592 |
| IFNB1 | ENST00000509629.1 | ENSG00000250164.1 | -0.812507692 | -1.819266747 | 0.068870744 |
| IFNB1 | ENST00000510198.1 | ENSG00000248733.1 | 0.849138245 | 1.883145751 | 0.059680618 |
| IFNB1 | ENST00000512563.1 | ENSG00000249547.1 | 0.920985119 | 2.052758207 | 0.040096034 |
| IFNB1 | ENST00000518339.1 | ENSG00000253470.1 | 0.916766893 | 2.038289901 | 0.041520949 |
| IFNB1 | ENST00000520192.1 | ENSG00000253807.1 | 0.8527861 | 1.888700908 | 0.058931917 |
| IFNB1 | ENST00000522190.1 | ENSG00000254165.1 | 0.817102356 | 1.826415243 | 0.067787737 |
| IFNB1 | ENST00000522426.1 | ENSG00000253538.1 | 0.978170036 | 2.187927692 | 0.02867487 |
| IFNB1 | ENST00000531977.1 | ENSG00000224023.6 | 0.892485769 | 2.02242216 | 0.043132758 |
| IFNB1 | ENST00000535914.1 | ENSG00000256894.1 | 0.809567122 | 1.816676495 | 0.069266663 |
| IFNB1 | ENST00000536412.1 | ENSG00000256072.1 | -0.920780159 | -2.043621557 | 0.040990948 |
| IFNB1 | ENST00000536492.1 | ENSG00000256237.1 | -0.86324115 | -1.923305219 | 0.05444173 |
| IFNB1 | ENST00000540024.1 | ENSG00000255693.1 | -0.817736903 | -1.817568726 | 0.069130075 |
| IFNB1 | ENST00000545642.1 | ENSG00000256342.1 | 0.815464858 | 1.826373167 | 0.067794071 |
| IFNB1 | ENST00000546135.1 | ENSG00000256670.1 | -0.818354924 | -1.848098634 | 0.064588075 |
| IFNB1 | ENST00000548199.1 | ENSG00000257614.1 | 0.950800021 | 2.099969268 | 0.035731545 |

| | | | | | |
|---|---|---|---|---|---|
| IFNB1 | ENST00000551067.1 | ENSG00000257891.1 | -0.911871604 | -2.038064044 | 0.041543528 |
| IFNB1 | ENST00000551174.1 | ENSG00000257762.1 | -0.857054459 | -1.913186191 | 0.055724221 |
| IFNB1 | ENST00000552558.1 | ENSG00000257947.1 | 0.898301599 | 2.006913933 | 0.044758828 |
| IFNB1 | ENST00000553537.1 | ENSG00000258481.1 | -0.807298141 | -1.814601552 | 0.069585164 |
| IFNB1 | ENST00000557232.1 | ENSG00000259054.1 | 0.838017875 | 1.846878377 | 0.064764769 |
| IFNB1 | ENST00000558618.1 | ENSG00000259209.1 | -0.853073353 | -1.909474476 | 0.056200911 |
| IFNB1 | ENST00000562582.1 | ENSG00000259779.1 | 0.915488297 | 2.078150713 | 0.03769548 |
| IFNB1 | ENST00000562834.1 | ENSG00000261116.1 | 0.955323673 | 2.124042558 | 0.033666585 |
| IFNB1 | ENST00000563342.1 | ENSG00000259914.1 | 0.933871135 | 2.103966745 | 0.035381348 |
| IFNB1 | ENST00000563570.1 | ENSG00000259961.1 | -0.898836333 | -1.99142738 | 0.046433922 |
| IFNB1 | ENST00000565944.1 | ENSG00000260331.1 | 0.835760937 | 1.86736591 | 0.061850507 |
| IFNB1 | ENST00000568410.1 | ENSG00000260277.1 | 0.854099194 | 1.904216303 | 0.056882019 |
| IFNB1 | ENST00000568836.1 | ENSG00000259967.1 | 0.958740129 | 2.156057357 | 0.031079187 |
| IFNB1 | ENST00000569778.1 | ENSG00000260823.1 | -0.837955228 | -1.86920649 | 0.061594092 |
| IFNB1 | ENST00000570919.1 | ENSG00000263321.1 | 0.9405279 | 2.120261379 | 0.033984009 |
| IFNB1 | ENST00000572222.1 | ENSG00000261971.2 | -0.805452838 | -1.790026398 | 0.073449668 |
| IFNB1 | ENST00000573861.1 | ENSG00000263320.1 | 0.855786765 | 1.896665436 | 0.057872108 |
| IFNB1 | ENST00000576021.1 | ENSG00000262413.1 | -0.899489087 | -1.993272679 | 0.046231596 |
| IFNB1 | ENST00000577360.1 | ENSG00000264273.1 | 0.971993059 | 2.18762635 | 0.028696831 |
| IFNB1 | ENST00000578334.1 | ENSG00000265148.1 | 0.946735227 | 2.11510099 | 0.034421341 |
| IFNB1 | ENST00000583826.1 | ENSG00000265148.1 | 0.893327631 | 2.026076556 | 0.04275694 |
| IFNB1 | ENST00000585684.1 | ENSG00000267057.1 | 0.915581936 | 2.063058847 | 0.039107038 |
| IFNB1 | ENST00000586297.1 | ENSG00000267633.1 | 0.906746787 | 2.047977929 | 0.040562162 |
| IFNB1 | ENST00000586399.1 | ENSG00000228430.4 | 0.933069586 | 2.081584709 | 0.037380419 |
| IFNB1 | ENST00000589983.1 | ENSG00000267057.1 | 0.941111144 | 2.123298959 | 0.033728808 |
| IFNB1 | ENST00000592022.1 | ENSG00000267383.2 | 0.813161616 | 1.846911423 | 0.064759979 |
| IFNB1 | ENST00000592045.1 | ENSG00000267057.1 | 0.944074861 | 2.102975968 | 0.03546787 |
| IFNB1 | ENST00000592622.1 | ENSG00000267546.2 | -0.824295144 | -1.85995301 | 0.062892174 |
| IFNB1 | ENST00000593486.1 | ENSG00000250910.3 | 0.82365456 | 1.812079052 | 0.069973983 |
| IFNB1 | ENST00000593599.1 | ENSG00000231898.4 | 0.860500291 | 1.891199716 | 0.058597689 |
| IFNB1 | ENST00000596497.1 | ENSG00000268530.1 | 0.842273732 | 1.87555065 | 0.060717008 |
| IFNB1 | ENST00000598065.1 | ENSG00000231731.3 | -0.82136385 | -1.854640978 | 0.063647512 |
| IFNB1 | ENST00000599050.1 | ENSG00000268366.1 | 0.907024447 | 2.023229919 | 0.043049448 |
| IFNB1 | ENST00000600365.1 | ENSG00000231898.4 | 0.833680672 | 1.858385578 | 0.063114279 |
| IFNB1 | ENST00000600956.1 | ENSG00000232732.5 | -0.808874921 | -1.798626747 | 0.072077743 |
| IFNB1 | ENST00000601511.1 | ENSG00000244513.2 | 0.868448806 | 1.951900683 | 0.050949997 |
| IFNB1 | ENST00000602485.1 | ENSG00000270163.1 | -0.840956811 | -1.871400664 | 0.061289567 |
| IFNB1 | ENST00000602620.1 | ENSG00000215386.6 | 0.836732141 | 1.869045361 | 0.061616504 |
| IFNB1 | ENST00000602736.1 | ENSG00000269976.1 | 0.805152645 | 1.807611191 | 0.070667035 |
| IFNB1 | ENST00000602773.1 | ENSG00000270160.1 | -0.811318194 | -1.817232584 | 0.069181508 |
| IFNB1 | ENST00000603949.1 | ENSG00000270332.1 | 0.831831014 | 1.86531351 | 0.062137473 |
| IFNB1 | ENST00000606482.1 | ENSG00000272416.1 | 0.824850566 | 1.851799208 | 0.064054661 |
| IFNB1 | ENST00000606869.1 | ENSG00000272349.1 | 0.820283497 | 1.834287066 | 0.066611393 |
| IFNB1 | ENST00000607044.1 | ENSG00000272247.1 | 0.861661324 | 1.926037405 | 0.054099704 |
| IFNB1 | ENST00000607051.1 | ENSG00000271771.1 | 0.819560214 | 1.869669702 | 0.061529699 |
| IFNB1 | ENST00000608088.1 | ENSG00000272632.1 | 0.802900893 | 1.78971211 | 0.073500205 |
| IFNB1 | ENST00000608133.1 | ENSG00000273193.1 | 0.821561116 | 1.839048151 | 0.065908104 |
| IFNB1 | ENST00000609349.1 | ENSG00000272861.1 | 0.932844258 | 2.095288752 | 0.036145332 |
| IFNB1 | ENST00000609807.1 | ENSG00000272700.1 | 0.841038187 | 1.869040554 | 0.061617172 |
| IFNB1 | ENST00000609953.1 | ENSG00000272825.1 | 0.833953618 | 1.869820774 | 0.061508711 |
| IFNB1 | NR_027401.2 | FAM223A | 0.803014723 | 1.788577878 | 0.073682823 |

| | | | | | |
|---|---|---|---|---|---|
| IFNB1 | NR_027402.1 | FAM223B | -0.837412291 | -1.876838902 | 0.060540177 |
| IFNB1 | NR_027440.1 | LOC100272217 | -0.933509886 | -2.094021945 | 0.036258025 |
| IFNB1 | NR_038194.1 | LINC00583 | 0.874487254 | 1.961555916 | 0.049814209 |
| IFNB1 | NR_040001.2 | LINC01116 | 0.925019649 | 2.057132917 | 0.039673442 |
| IFNB1 | NR_046578.1 | CACNA1C-AS4 | 0.897395499 | 2.001896546 | 0.045295859 |
| IFNB1 | NR_046748.1 | ARHGAP31-AS1 | 0.841605453 | 1.879887058 | 0.060123472 |
| IFNB1 | NR_046766.1 | ATP2B2-IT2 | 0.973564912 | 2.176114634 | 0.02954669 |
| IFNB1 | NR_047040.1 | LINC00424 | 0.867888748 | 1.961450553 | 0.049826488 |
| IFNB1 | NR_047698.1 | VWC2L-IT1 | 0.911589282 | 2.034005528 | 0.041951038 |
| IFNB1 | NR_049776.1 | GPC5-AS1 | 0.821170386 | 1.824967933 | 0.068005869 |
| IFNB1 | NR_104618.1 | LINC01017 | 0.896702601 | 2.004587852 | 0.045007126 |
| IFNB1 | NR_109975.1 | ARNTL2-AS1 | -0.880308963 | -1.950176235 | 0.051155117 |
| IFNB1 | NR_110007.1 | ADNP-AS1 | 0.845815922 | 1.886558998 | 0.059219666 |
| IFNB1 | NR_110053.1 | LOC101927464 | 0.815464858 | 1.830967825 | 0.067105346 |
| IFNB1 | NR_110123.1 | GRM7-AS3 | 0.808953373 | 1.835209502 | 0.066474654 |
| IFNB1 | NR_120330.1 | LOC101928227 | -0.802029213 | -1.794506614 | 0.072732344 |
| IFNB1 | NR_120527.1 | LOC100506675 | 0.848123433 | 1.891624611 | 0.058541014 |
| IFNB1 | NR_120566.1 | LOC101928896 | 0.879636142 | 1.950827762 | 0.051077538 |
| IFNB1 | NR_125769.1 | LINC01269 | -0.885186816 | -1.995439514 | 0.045994965 |
| IFNB1 | NR_126334.1 | LOC101927932 | 0.825823796 | 1.864865706 | 0.06220023 |
| IFNB1 | NR_131186.1 | LOC105377348 | 0.877020277 | 1.961831357 | 0.049782122 |
| IFNB1 | NR_134573.1 | GS1-124K5.4 | 0.915508432 | 2.038864198 | 0.041463583 |
| IFNB1 | NR_134610.1 | LOC105375014 | -0.895322439 | -1.986853805 | 0.0469386 |
| IFNB1 | NR_135076.1 | LOC102723838 | 0.933356837 | 2.076347839 | 0.037861792 |
| IFNB1 | NR_135549.1 | LOC101929411 | 0.811202766 | 1.816302293 | 0.069324014 |
| IFNB1 | NR_136178.1 | LOC101928166 | 0.931123781 | 2.057079937 | 0.039678537 |
| IFNB1 | NR_136218.1 | MEF2C-AS1 | 0.864368317 | 1.896791576 | 0.057855452 |
| IGSF6 | ENST00000412809.1 | ENSG00000229938.1 | 0.826216088 | 1.859370933 | 0.062974579 |
| IGSF6 | ENST00000413353.1 | ENSG00000232893.1 | -0.882934831 | -1.979688286 | 0.047738566 |
| IGSF6 | ENST00000413650.1 | ENSG00000230880.2 | -0.834634658 | -1.882652041 | 0.059747539 |
| IGSF6 | ENST00000413991.1 | ENSG00000237614.1 | -0.813455663 | -1.800045004 | 0.071853532 |
| IGSF6 | ENST00000422697.1 | ENSG00000236414.1 | 0.839626994 | 1.88048995 | 0.060041335 |
| IGSF6 | ENST00000423925.1 | ENSG00000223536.1 | 0.850213786 | 1.921944634 | 0.054612725 |
| IGSF6 | ENST00000424241.1 | ENSG00000237311.1 | 0.901260387 | 2.012545005 | 0.044162518 |
| IGSF6 | ENST00000424735.1 | ENSG00000237457.2 | 0.882441097 | 1.987229586 | 0.046896961 |
| IGSF6 | ENST00000425364.1 | ENSG00000231046.1 | 0.859017704 | 1.931664837 | 0.053400888 |
| IGSF6 | ENST00000426504.1 | ENSG00000234190.1 | 0.87161997 | 1.923691358 | 0.054393282 |
| IGSF6 | ENST00000429796.1 | ENSG00000231858.1 | 0.887599011 | 1.975623832 | 0.048197397 |
| IGSF6 | ENST00000431290.1 | ENSG00000183822.2 | -0.850395045 | -1.886691849 | 0.059201785 |
| IGSF6 | ENST00000432244.1 | ENSG00000234265.1 | -0.83967628 | -1.859550806 | 0.062949105 |
| IGSF6 | ENST00000433614.1 | ENSG00000228534.1 | 0.858105437 | 1.94129946 | 0.052221965 |
| IGSF6 | ENST00000433876.2 | ENSG00000228423.2 | -0.85466053 | -1.90952499 | 0.056194401 |
| IGSF6 | ENST00000435434.1 | ENSG00000231233.1 | -0.85360949 | -1.910187664 | 0.056109055 |
| IGSF6 | ENST00000435892.1 | ENSG00000233635.2 | -0.881694609 | -1.991718653 | 0.046401936 |
| IGSF6 | ENST00000437308.1 | ENSG00000233154.1 | -0.879528249 | -1.955148588 | 0.050565535 |
| IGSF6 | ENST00000437461.1 | ENSG00000227200.1 | -0.830074972 | -1.835361843 | 0.066452093 |
| IGSF6 | ENST00000438107.1 | ENSG00000234449.2 | -0.857776528 | -1.915263546 | 0.055458902 |
| IGSF6 | ENST00000441875.1 | ENSG00000239203.1 | -0.920661776 | -2.063598317 | 0.039055817 |
| IGSF6 | ENST00000442850.1 | ENSG00000232600.2 | 0.809307299 | 1.810035222 | 0.070290325 |
| IGSF6 | ENST00000444665.1 | ENSG00000228852.2 | -0.900719837 | -2.023679297 | 0.04300316 |
| IGSF6 | ENST00000447206.1 | ENSG00000230839.1 | -0.916303113 | -2.027818976 | 0.042578727 |

| | | | | | |
|---|---|---|---|---|---|
| IGSF6 | ENST00000448650.1 | ENSG00000223536.1 | 0.843489619 | 1.880101078 | 0.060094304 |
| IGSF6 | ENST00000450109.1 | ENSG00000225376.1 | -0.802591314 | -1.801125564 | 0.071683091 |
| IGSF6 | ENST00000450531.1 | ENSG00000229536.1 | -0.811965857 | -1.809277141 | 0.070407959 |
| IGSF6 | ENST00000451656.1 | ENSG00000228417.1 | -0.891009077 | -1.976356055 | 0.048114465 |
| IGSF6 | ENST00000452002.1 | ENSG00000236501.1 | 0.881621134 | 2.010492816 | 0.044379056 |
| IGSF6 | ENST00000454530.1 | ENSG00000226649.1 | 0.813847272 | 1.84184413 | 0.065497953 |
| IGSF6 | ENST00000456715.1 | ENSG00000224893.1 | -0.98399581 | -2.18266317 | 0.029060624 |
| IGSF6 | ENST00000457848.1 | ENSG00000226412.1 | -0.841024475 | -1.907248396 | 0.056488429 |
| IGSF6 | ENST00000458364.1 | ENSG00000225655.1 | 0.801688569 | 1.783685111 | 0.074474847 |
| IGSF6 | ENST00000459985.1 | ENSG00000273066.1 | -0.847239317 | -1.892318013 | 0.058448622 |
| IGSF6 | ENST00000489557.2 | ENSG00000257045.1 | -0.892066949 | -1.996263927 | 0.045905202 |
| IGSF6 | ENST00000502421.1 | ENSG00000250284.1 | 0.811434939 | 1.787513658 | 0.073854507 |
| IGSF6 | ENST00000503938.1 | ENSG00000246095.2 | -0.840802833 | -1.853901385 | 0.06375327 |
| IGSF6 | ENST00000504795.1 | ENSG00000250723.1 | -0.806086257 | -1.810346188 | 0.070242119 |
| IGSF6 | ENST00000505196.1 | ENSG00000248131.1 | -0.837715347 | -1.882753803 | 0.05973374 |
| IGSF6 | ENST00000505556.1 | ENSG00000249409.1 | -0.818099094 | -1.846547214 | 0.064812791 |
| IGSF6 | ENST00000506100.1 | ENSG00000249409.1 | -0.837299721 | -1.859590182 | 0.062943529 |
| IGSF6 | ENST00000508083.1 | ENSG00000249343.1 | -0.835113423 | -1.869969589 | 0.061488041 |
| IGSF6 | ENST00000514411.1 | ENSG00000250882.1 | -0.859384975 | -1.909092284 | 0.056250188 |
| IGSF6 | ENST00000514877.1 | ENSG00000248685.1 | -0.803356722 | -1.798829411 | 0.072045669 |
| IGSF6 | ENST00000518260.1 | ENSG00000253628.1 | 0.806636236 | 1.798579738 | 0.072085185 |
| IGSF6 | ENST00000519038.2 | ENSG00000254054.2 | 0.88678519 | 1.9758146 | 0.048175779 |
| IGSF6 | ENST00000519852.1 | ENSG00000253716.1 | -0.864651962 | -1.914370383 | 0.055572847 |
| IGSF6 | ENST00000521207.1 | ENSG00000253716.1 | -0.833966817 | -1.842832849 | 0.065353419 |
| IGSF6 | ENST00000522547.1 | ENSG00000253430.1 | 0.848670541 | 1.906388993 | 0.056599756 |
| IGSF6 | ENST00000524335.1 | ENSG00000253716.1 | -0.877795824 | -1.969960825 | 0.04884286 |
| IGSF6 | ENST00000524818.1 | ENSG00000254473.1 | -0.82766214 | -1.85927008 | 0.062988866 |
| IGSF6 | ENST00000526611.1 | ENSG00000246982.2 | -0.893991144 | -1.978779108 | 0.047840882 |
| IGSF6 | ENST00000528818.1 | ENSG00000232995.3 | -0.859751236 | -1.946246127 | 0.051625181 |
| IGSF6 | ENST00000531087.1 | ENSG00000254428.1 | 0.862000604 | 1.919615301 | 0.05490651 |
| IGSF6 | ENST00000531136.1 | ENSG00000255558.1 | 0.834740813 | 1.857044187 | 0.063304868 |
| IGSF6 | ENST00000536141.1 | ENSG00000256969.1 | -0.824130332 | -1.825842179 | 0.067874038 |
| IGSF6 | ENST00000537269.1 | ENSG00000257084.1 | -0.844991223 | -1.869495686 | 0.061553883 |
| IGSF6 | ENST00000543072.1 | ENSG00000256092.2 | 0.838711622 | 1.890412446 | 0.05870282 |
| IGSF6 | ENST00000548731.1 | ENSG00000257809.1 | -0.823945186 | -1.840148524 | 0.065746435 |
| IGSF6 | ENST00000549878.1 | ENSG00000257284.1 | -0.818276328 | -1.829693236 | 0.067295823 |
| IGSF6 | ENST00000552469.1 | ENSG00000258325.1 | -0.912415334 | -2.055801027 | 0.039801699 |
| IGSF6 | ENST00000553464.1 | ENSG00000258418.1 | -0.80778121 | -1.796178006 | 0.072466212 |
| IGSF6 | ENST00000554049.1 | ENSG00000258763.1 | -0.824442372 | -1.833749989 | 0.066691114 |
| IGSF6 | ENST00000554798.1 | ENSG00000258483.1 | -0.891610367 | -1.984105863 | 0.04724404 |
| IGSF6 | ENST00000556786.1 | ENSG00000258525.1 | 0.838170164 | 1.880090112 | 0.060095798 |
| IGSF6 | ENST00000560193.1 | ENSG00000259176.1 | -0.829115096 | -1.823258237 | 0.068264288 |
| IGSF6 | ENST00000564102.1 | ENSG00000260041.1 | 0.860450351 | 1.926469935 | 0.054045722 |
| IGSF6 | ENST00000565359.1 | ENSG00000260601.1 | -0.802874917 | -1.796942144 | 0.072344805 |
| IGSF6 | ENST00000565829.1 | ENSG00000260148.1 | -0.878782465 | -1.934285818 | 0.053077996 |
| IGSF6 | ENST00000570158.1 | ENSG00000260937.1 | 0.939740929 | 2.08967502 | 0.036647002 |
| IGSF6 | ENST00000570843.1 | ENSG00000261889.1 | -0.857304765 | -1.928327697 | 0.053814379 |
| IGSF6 | ENST00000570929.1 | ENSG00000262223.2 | -0.859818713 | -1.927055477 | 0.053972717 |
| IGSF6 | ENST00000571815.1 | ENSG00000262810.1 | -0.88031629 | -1.997748674 | 0.045743914 |
| IGSF6 | ENST00000575139.1 | ENSG00000263072.1 | -0.803370138 | -1.785386981 | 0.074198569 |
| IGSF6 | ENST00000576086.1 | ENSG00000262823.1 | -0.856728527 | -1.905625567 | 0.056698803 |

| | | | | | |
|---|---|---|---|---|---|
| IGSF6 | ENST00000577064.1 | ENSG00000262823.1 | -0.928378774 | -2.053129433 | 0.040060026 |
| IGSF6 | ENST00000577176.1 | ENSG00000262823.1 | -0.823000817 | -1.838112641 | 0.066045809 |
| IGSF6 | ENST00000577698.1 | ENSG00000265100.1 | -0.810321878 | -1.823281385 | 0.068260784 |
| IGSF6 | ENST00000581905.1 | ENSG00000264235.1 | -0.82703278 | -1.884574604 | 0.059487294 |
| IGSF6 | ENST00000586010.1 | ENSG00000267606.1 | -0.868390806 | -1.908530334 | 0.056322706 |
| IGSF6 | ENST00000586051.1 | ENSG00000267576.1 | -0.887499384 | -1.994726193 | 0.046072751 |
| IGSF6 | ENST00000589281.1 | ENSG00000267707.1 | 0.827288254 | 1.838901113 | 0.065929732 |
| IGSF6 | ENST00000594590.2 | ENSG00000268199.2 | -0.846087491 | -1.886823013 | 0.059184135 |
| IGSF6 | ENST00000597309.1 | ENSG00000232098.2 | 0.870575679 | 1.939460995 | 0.05244523 |
| IGSF6 | ENST00000599572.1 | ENSG00000233783.3 | 0.864693167 | 1.928005442 | 0.053854449 |
| IGSF6 | ENST00000600534.1 | ENSG00000267858.1 | -0.879844831 | -1.95592927 | 0.050473487 |
| IGSF6 | ENST00000602532.1 | ENSG00000270091.1 | -0.821634142 | -1.806721829 | 0.070805662 |
| IGSF6 | ENST00000602872.1 | ENSG00000270067.1 | -0.87762406 | -1.9779476 | 0.047934618 |
| IGSF6 | ENST00000606068.1 | ENSG00000272342.1 | -0.830619446 | -1.85345482 | 0.063817196 |
| IGSF6 | ENST00000606374.1 | ENSG00000272312.1 | 0.827105973 | 1.83561386 | 0.066414786 |
| IGSF6 | ENST00000607222.1 | ENSG00000272106.1 | -0.80034818 | -1.769957184 | 0.076734274 |
| IGSF6 | ENST00000608422.1 | ENSG00000272866.1 | -0.805333075 | -1.801780921 | 0.071579881 |
| IGSF6 | ENST00000608952.1 | ENSG00000272689.1 | 0.850956477 | 1.915034562 | 0.055488096 |
| IGSF6 | ENST00000609067.1 | ENSG00000272849.1 | -0.830881927 | -1.856786585 | 0.063341523 |
| IGSF6 | ENST00000609281.1 | ENSG00000273320.1 | -0.943196915 | -2.113781301 | 0.034533951 |
| IGSF6 | ENST00000610145.1 | ENSG00000273175.1 | -0.847974213 | -1.921400494 | 0.054681236 |
| IGSF6 | NR_027334.2 | MZF1-AS1 | -0.8829118 | -1.952998867 | 0.050819729 |
| IGSF6 | NR_034037.1 | LINC00582 | 0.858371708 | 1.949532812 | 0.051231828 |
| IGSF6 | NR_034111.1 | TRAF3IP2-AS1 | -0.901180362 | -2.011933368 | 0.044226961 |
| IGSF6 | NR_046454.1 | LINC00907 | 0.85049191 | 1.906320747 | 0.056608604 |
| IGSF6 | NR_104158.1 | NRG1-IT1 | -0.848895598 | -1.886782336 | 0.059189608 |
| IGSF6 | NR_108036.1 | CFAP58-AS1 | -0.840260376 | -1.859883912 | 0.062901952 |
| IGSF6 | NR_109870.1 | LINC01723 | -0.836526162 | -1.887889163 | 0.059040832 |
| IGSF6 | NR_110370.1 | STAM-AS1 | 0.817434836 | 1.828698831 | 0.067444738 |
| IGSF6 | NR_110568.1 | LOC101927661 | -0.841759449 | -1.859082326 | 0.06301547 |
| IGSF6 | NR_110919.1 | LOC101928530 | -0.931531253 | -2.081972512 | 0.037344981 |
| IGSF6 | NR_130144.1 | LOC104968399 | -0.82703278 | -1.863313577 | 0.062418161 |
| IGSF6 | NR_133658.1 | HTR3E-AS1 | 0.830859536 | 1.852082384 | 0.064013994 |
| IGSF6 | NR_134273.1 | LOC101929544 | -0.81642814 | -1.849848751 | 0.064335352 |
| IGSF6 | NR_135032.1 | LOC105369635 | -0.844991223 | -1.870036676 | 0.061478725 |
| IGSF6 | NR_135644.1 | LOC105371506 | 0.887951788 | 1.985689025 | 0.047067864 |
| IGSF6 | NR_135679.1 | LOC105370829 | 0.813395671 | 1.820108375 | 0.068742503 |
| IL10RB | ENST00000381106.4 | ENSG00000205663.5 | -0.944107672 | -2.069375754 | 0.03851084 |
| IL10RB | ENST00000381475.3 | ENSG00000215863.2 | -0.957966767 | -2.135999079 | 0.032679489 |
| IL10RB | ENST00000412348.1 | ENSG00000228959.1 | -0.892999231 | -2.022450166 | 0.043129867 |
| IL10RB | ENST00000417654.1 | ENSG00000224893.1 | 0.914241134 | 2.035181246 | 0.041832639 |
| IL10RB | ENST00000424735.1 | ENSG00000237457.2 | -0.829296991 | -1.865294104 | 0.062140191 |
| IL10RB | ENST00000426302.1 | ENSG00000230454.1 | 0.831762653 | 1.844720467 | 0.065078212 |
| IL10RB | ENST00000436515.1 | ENSG00000224521.1 | -0.862610268 | -1.923218035 | 0.054452673 |
| IL10RB | ENST00000437859.2 | ENSG00000235122.3 | -0.913862002 | -2.063225483 | 0.03909121 |
| IL10RB | ENST00000438623.1 | ENSG00000224521.1 | -0.913235149 | -2.030989272 | 0.042256083 |
| IL10RB | ENST00000449154.1 | ENSG00000226969.1 | 0.950348127 | 2.10616 | 0.035190456 |
| IL10RB | ENST00000450696.1 | ENSG00000235146.2 | -0.846682813 | -1.899692396 | 0.057473499 |
| IL10RB | ENST00000451034.1 | ENSG00000229805.1 | -0.817217901 | -1.825795172 | 0.067881121 |
| IL10RB | ENST00000457998.2 | ENSG00000233006.2 | 0.930904147 | 2.092559375 | 0.036388506 |
| IL10RB | ENST00000503505.1 | ENSG00000248629.1 | -0.84303497 | -1.891009951 | 0.058623016 |

| | | | | | |
|---|---|---|---|---|---|
| IL10RB | ENST00000504578.1 | ENSG00000251513.1 | -0.855454959 | -1.932936426 | 0.05324403 |
| IL10RB | ENST00000515128.1 | ENSG00000248215.1 | -0.813033518 | -1.804978188 | 0.071078095 |
| IL10RB | ENST00000522600.1 | ENSG00000246582.2 | 0.815104748 | 1.798808972 | 0.072048904 |
| IL10RB | ENST00000524808.1 | ENSG00000254812.1 | 0.81205304 | 1.825591989 | 0.067911743 |
| IL10RB | ENST00000528887.1 | ENSG00000254501.1 | 0.872328565 | 1.972276574 | 0.048578042 |
| IL10RB | ENST00000543494.1 | ENSG00000256514.1 | 0.906257827 | 2.019451778 | 0.043440285 |
| IL10RB | ENST00000548210.1 | ENSG00000257784.1 | 0.836230862 | 1.854789047 | 0.063626357 |
| IL10RB | ENST00000549683.1 | ENSG00000257953.1 | 0.943168196 | 2.119302703 | 0.034064893 |
| IL10RB | ENST00000551135.1 | ENSG00000258294.1 | -0.838905159 | -1.875525426 | 0.060720475 |
| IL10RB | ENST00000555460.1 | ENSG00000259042.1 | 0.904909896 | 2.056870273 | 0.039698706 |
| IL10RB | ENST00000563018.1 | ENSG00000260193.1 | 0.891163158 | 1.990716086 | 0.046512109 |
| IL10RB | ENST00000565667.1 | ENSG00000261253.1 | 0.915805323 | 2.034085528 | 0.041942973 |
| IL10RB | ENST00000565798.2 | ENSG00000259786.2 | -0.879150612 | -1.93958274 | 0.05243042 |
| IL10RB | ENST00000567067.1 | ENSG00000261600.1 | -0.92249312 | -2.079477862 | 0.037573451 |
| IL10RB | ENST00000568659.1 | ENSG00000260004.1 | -0.910434872 | -2.050087851 | 0.040355859 |
| IL10RB | ENST00000570974.1 | ENSG00000263300.1 | 0.804325687 | 1.781267747 | 0.074868721 |
| IL10RB | ENST00000576086.1 | ENSG00000262823.1 | 0.936828783 | 2.102732831 | 0.03548913 |
| IL10RB | ENST00000576554.1 | ENSG00000262413.1 | 0.818755068 | 1.828282284 | 0.067507197 |
| IL10RB | ENST00000577176.1 | ENSG00000262823.1 | 0.852493938 | 1.896591594 | 0.057881861 |
| IL10RB | ENST00000578443.1 | ENSG00000265204.1 | 0.815531797 | 1.83446885 | 0.066584428 |
| IL10RB | ENST00000580729.1 | ENSG00000266176.1 | 0.839674448 | 1.864167808 | 0.062298143 |
| IL10RB | ENST00000598092.1 | ENSG00000228065.6 | -0.874021896 | -1.969201367 | 0.048929972 |
| IL10RB | ENST00000600007.1 | ENSG00000268655.1 | 0.867011965 | 1.951765675 | 0.050966031 |
| IL10RB | ENST00000600716.1 | ENSG00000269487.1 | 0.818344573 | 1.813131703 | 0.06981151 |
| IL10RB | ENST00000600726.1 | ENSG00000267858.1 | 0.800414321 | 1.800968843 | 0.071707791 |
| IL10RB | ENST00000602620.1 | ENSG00000215386.6 | -0.847743257 | -1.887605026 | 0.059078995 |
| IL10RB | ENST00000606010.1 | ENSG00000272249.1 | -0.817015038 | -1.834155371 | 0.066630934 |
| IL10RB | ENST00000607549.1 | ENSG00000272293.1 | -0.87565512 | -1.97311612 | 0.048482334 |
| IL10RB | ENST00000607665.1 | ENSG00000272254.1 | -0.839138192 | -1.867748592 | 0.061797122 |
| IL10RB | ENST00000608259.1 | ENSG00000272627.1 | -0.824185581 | -1.826077973 | 0.067838518 |
| IL10RB | ENST00000608465.1 | ENSG00000272758.1 | -0.81497325 | -1.798834373 | 0.072044884 |
| IL10RB | ENST00000608489.1 | ENSG00000272716.1 | 0.828375139 | 1.874756614 | 0.060826214 |
| IL10RB | ENST00000608934.1 | ENSG00000273063.1 | -0.882230842 | -1.951158855 | 0.051038152 |
| IL10RB | ENST00000609113.1 | ENSG00000272827.1 | 0.831189627 | 1.858287737 | 0.063128164 |
| IL10RB | ENST00000609976.1 | ENSG00000272582.1 | 0.971217205 | 2.178479697 | 0.029370342 |
| IL10RB | ENST00000610270.1 | ENSG00000272576.1 | -0.812991775 | -1.829901724 | 0.067264636 |
| IL10RB | NR_073552.1 | LOC101059948 | 0.867011965 | 1.933397747 | 0.053187219 |
| IL10RB | NR_110117.1 | LOC101927769 | -0.843740902 | -1.913912745 | 0.055631306 |
| IL10RB | NR_110556.1 | LOC102724890 | -0.8161865 | -1.844292015 | 0.065140595 |
| IL10RB | NR_110919.1 | LOC101928530 | 0.836716431 | 1.877191827 | 0.060491807 |
| IL17C | ENST00000318291.4 | ENSG00000177406.4 | 0.969601679 | 2.137898188 | 0.032525007 |
| IL17C | ENST00000411694.1 | ENSG00000225331.1 | 0.812690722 | 1.830735321 | 0.067140058 |
| IL17C | ENST00000412759.1 | ENSG00000236933.1 | 0.942500232 | 2.111797804 | 0.034703796 |
| IL17C | ENST00000417260.1 | ENSG00000231734.4 | -0.823449902 | -1.839356711 | 0.065862737 |
| IL17C | ENST00000419662.1 | ENSG00000228265.1 | 0.914583732 | 2.064760953 | 0.038945624 |
| IL17C | ENST00000421020.1 | ENSG00000231407.1 | 0.904107983 | 2.041894549 | 0.041161993 |
| IL17C | ENST00000421207.1 | ENSG00000231768.1 | 0.879003992 | 1.949785681 | 0.051201669 |
| IL17C | ENST00000423428.1 | ENSG00000224048.1 | -0.804939605 | -1.781718645 | 0.074795125 |
| IL17C | ENST00000423869.1 | ENSG00000227848.1 | 0.810625899 | 1.814649003 | 0.069577867 |
| IL17C | ENST00000425124.1 | ENSG00000232336.1 | 0.870768176 | 1.97020737 | 0.048814609 |
| IL17C | ENST00000426237.2 | ENSG00000235527.2 | 0.895851172 | 1.964013966 | 0.049528472 |

| | | | | | |
|---|---|---|---|---|---|
| IL17C | ENST00000426519.1 | ENSG00000234142.1 | 0.928999711 | 2.046884296 | 0.040669446 |
| IL17C | ENST00000430920.1 | ENSG00000234203.1 | 0.811702275 | 1.782612097 | 0.074649469 |
| IL17C | ENST00000433905.2 | ENSG00000229299.2 | 0.846660642 | 1.890483248 | 0.058693359 |
| IL17C | ENST00000434627.1 | ENSG00000230074.1 | 0.815035624 | 1.826241165 | 0.067813943 |
| IL17C | ENST00000435434.1 | ENSG00000231233.1 | 0.915616163 | 2.048812437 | 0.040480459 |
| IL17C | ENST00000435892.1 | ENSG00000233635.2 | 0.856820746 | 1.905434402 | 0.056723627 |
| IL17C | ENST00000435992.2 | ENSG00000232675.3 | 0.85684655 | 1.902996013 | 0.057041065 |
| IL17C | ENST00000438190.1 | ENSG00000227214.2 | 0.991760539 | 2.21021285 | 0.027090394 |
| IL17C | ENST00000442069.1 | ENSG00000225655.1 | -0.837053559 | -1.893351781 | 0.058311103 |
| IL17C | ENST00000447206.1 | ENSG00000230839.1 | 0.830524995 | 1.837056926 | 0.066201492 |
| IL17C | ENST00000447343.2 | ENSG00000229299.2 | 0.817696048 | 1.805398253 | 0.071012384 |
| IL17C | ENST00000452176.1 | ENSG00000223659.1 | -0.928948825 | -2.083421339 | 0.037212835 |
| IL17C | ENST00000453051.1 | ENSG00000229407.1 | 0.872166068 | 1.932988131 | 0.05323766 |
| IL17C | ENST00000457115.1 | ENSG00000227245.1 | 0.84627512 | 1.888417213 | 0.058969962 |
| IL17C | ENST00000457253.1 | ENSG00000225173.1 | 0.84321253 | 1.877210471 | 0.060489253 |
| IL17C | ENST00000458154.1 | ENSG00000235578.1 | 0.978544801 | 2.181341102 | 0.029158196 |
| IL17C | ENST00000458194.1 | ENSG00000226193.1 | 0.918602143 | 2.037094202 | 0.041640603 |
| IL17C | ENST00000458364.1 | ENSG00000225655.1 | -0.88870598 | -1.982474456 | 0.047426164 |
| IL17C | ENST00000459985.1 | ENSG00000273066.1 | 0.920330245 | 2.055309642 | 0.039849107 |
| IL17C | ENST00000463255.1 | ENSG00000243305.1 | -0.808217247 | -1.797927988 | 0.07218842 |
| IL17C | ENST00000468165.1 | ENSG00000239480.1 | 0.821505685 | 1.821299196 | 0.068561389 |
| IL17C | ENST00000484413.1 | ENSG00000271853.1 | 0.839909524 | 1.859475836 | 0.062959721 |
| IL17C | ENST00000489077.1 | ENSG00000244198.1 | 0.955372382 | 2.136136955 | 0.032668252 |
| IL17C | ENST00000489557.2 | ENSG00000257045.1 | 0.802722298 | 1.790233204 | 0.073416429 |
| IL17C | ENST00000494509.1 | ENSG00000240095.1 | 0.811630862 | 1.807477004 | 0.070687937 |
| IL17C | ENST00000498693.1 | ENSG00000244198.1 | 0.987425875 | 2.221897051 | 0.026290264 |
| IL17C | ENST00000503723.1 | ENSG00000250472.1 | -0.854399418 | -1.914876619 | 0.05550824 |
| IL17C | ENST00000505556.1 | ENSG00000249409.1 | 0.908491961 | 2.015026055 | 0.043901919 |
| IL17C | ENST00000506100.1 | ENSG00000249409.1 | 0.899297596 | 2.038196576 | 0.041530278 |
| IL17C | ENST00000506791.1 | ENSG00000251131.1 | 0.971035835 | 2.216821212 | 0.026635311 |
| IL17C | ENST00000508083.1 | ENSG00000249343.1 | 0.947301785 | 2.116467253 | 0.034305087 |
| IL17C | ENST00000509036.1 | ENSG00000251131.1 | 0.968745096 | 2.174031756 | 0.02970275 |
| IL17C | ENST00000509192.1 | ENSG00000250765.1 | 0.967373595 | 2.187540276 | 0.028703106 |
| IL17C | ENST00000515128.1 | ENSG00000248215.1 | -0.901410084 | -1.997259334 | 0.045797018 |
| IL17C | ENST00000517300.1 | ENSG00000254144.2 | 0.826276349 | 1.850411594 | 0.064254249 |
| IL17C | ENST00000520603.1 | ENSG00000254001.1 | -0.947944092 | -2.118103189 | 0.03416633 |
| IL17C | ENST00000521307.1 | ENSG00000253177.1 | 0.80380392 | 1.817008448 | 0.06921582 |
| IL17C | ENST00000522547.1 | ENSG00000253430.1 | -0.828296574 | -1.85563215 | 0.063506008 |
| IL17C | ENST00000522600.1 | ENSG00000246582.2 | 0.867587334 | 1.948323076 | 0.051376318 |
| IL17C | ENST00000526611.1 | ENSG00000246982.2 | 0.81332533 | 1.830788488 | 0.067132119 |
| IL17C | ENST00000528887.1 | ENSG00000254501.1 | 0.875146274 | 1.967853188 | 0.049084932 |
| IL17C | ENST00000543072.1 | ENSG00000256092.2 | -0.904284997 | -2.046571027 | 0.040700222 |
| IL17C | ENST00000543275.1 | ENSG00000256944.1 | 0.902186679 | 2.032769055 | 0.042075861 |
| IL17C | ENST00000545177.3 | ENSG00000230438.5 | 0.883466101 | 1.959589086 | 0.050043838 |
| IL17C | ENST00000548722.2 | ENSG00000257194.2 | -0.892867324 | -2.0102266 | 0.044407211 |
| IL17C | ENST00000549806.1 | ENSG00000257252.1 | 0.804771867 | 1.78176298 | 0.074787892 |
| IL17C | ENST00000549878.1 | ENSG00000257284.1 | 0.811448816 | 1.792173448 | 0.073105187 |
| IL17C | ENST00000550263.1 | ENSG00000257605.1 | 0.811235641 | 1.820680025 | 0.06865551 |
| IL17C | ENST00000558575.1 | ENSG00000259687.1 | 0.804090934 | 1.79983674 | 0.071886421 |
| IL17C | ENST00000563018.1 | ENSG00000260193.1 | 0.807455342 | 1.7969409 | 0.072345003 |
| IL17C | ENST00000563611.1 | ENSG00000261583.1 | 0.89090707 | 1.989287974 | 0.046669426 |

| | | | | | |
|---|---|---|---|---|---|
| IL17C | ENST00000565823.1 | ENSG00000260686.1 | -0.911604946 | -2.046831684 | 0.040674614 |
| IL17C | ENST00000565829.1 | ENSG00000260148.1 | 0.878282214 | 1.970906623 | 0.048734557 |
| IL17C | ENST00000567395.1 | ENSG00000261090.1 | 0.844394039 | 1.884545922 | 0.059491169 |
| IL17C | ENST00000569981.1 | ENSG00000238045.5 | 0.930635608 | 2.062651646 | 0.039145737 |
| IL17C | ENST00000570493.2 | ENSG00000261898.2 | 0.932348265 | 2.104435728 | 0.035340456 |
| IL17C | ENST00000570512.1 | ENSG00000262768.1 | 0.820510245 | 1.848070599 | 0.06459213 |
| IL17C | ENST00000570843.1 | ENSG00000261889.1 | 0.931503853 | 2.094317962 | 0.036231665 |
| IL17C | ENST00000570929.1 | ENSG00000262223.2 | 0.942950177 | 2.123077714 | 0.03374734 |
| IL17C | ENST00000578800.1 | ENSG00000264235.1 | 0.860641082 | 1.920370502 | 0.054811117 |
| IL17C | ENST00000578936.1 | ENSG00000265547.1 | 0.811358886 | 1.815654173 | 0.069423438 |
| IL17C | ENST00000582044.1 | ENSG00000263715.2 | 0.831920541 | 1.842647519 | 0.065380491 |
| IL17C | ENST00000582558.1 | ENSG00000264569.1 | 0.880362755 | 1.950552192 | 0.051110339 |
| IL17C | ENST00000585559.1 | ENSG00000267117.1 | 0.9248839 | 2.103960952 | 0.035381853 |
| IL17C | ENST00000586051.1 | ENSG00000267576.1 | 0.808008208 | 1.782319284 | 0.07469718 |
| IL17C | ENST00000586694.1 | ENSG00000267141.1 | 0.852866361 | 1.946777532 | 0.051561412 |
| IL17C | ENST00000588380.1 | ENSG00000266990.1 | 0.888000429 | 1.98868675 | 0.046735789 |
| IL17C | ENST00000591174.1 | ENSG00000267289.1 | 0.964962115 | 2.177539784 | 0.029440317 |
| IL17C | ENST00000592400.1 | ENSG00000267735.1 | 0.90545057 | 2.026949655 | 0.042667562 |
| IL17C | ENST00000593632.1 | ENSG00000180279.5 | 0.809440874 | 1.796960545 | 0.072341884 |
| IL17C | ENST00000594590.2 | ENSG00000268199.2 | 0.917916025 | 2.054107031 | 0.039965334 |
| IL17C | ENST00000595478.1 | ENSG00000237031.3 | -0.828990381 | -1.869195921 | 0.061595562 |
| IL17C | ENST00000596887.1 | ENSG00000237031.3 | -0.812081782 | -1.83441729 | 0.066592075 |
| IL17C | ENST00000597169.1 | ENSG00000269720.1 | 0.954490192 | 2.124855139 | 0.033598702 |
| IL17C | ENST00000597309.1 | ENSG00000232098.2 | -0.807570511 | -1.833899135 | 0.066668968 |
| IL17C | ENST00000598092.1 | ENSG00000228065.6 | -0.831091672 | -1.838801344 | 0.06594441 |
| IL17C | ENST00000599259.1 | ENSG00000269352.1 | 0.937093832 | 2.082133643 | 0.037330264 |
| IL17C | ENST00000600234.1 | ENSG00000268078.1 | 0.801066874 | 1.781165343 | 0.074885443 |
| IL17C | ENST00000600534.1 | ENSG00000267858.1 | 0.885095614 | 1.960656937 | 0.049919056 |
| IL17C | ENST00000600726.1 | ENSG00000267858.1 | 0.95964401 | 2.155346717 | 0.031134713 |
| IL17C | ENST00000601033.1 | ENSG00000268401.1 | 0.809464294 | 1.789010542 | 0.073613118 |
| IL17C | ENST00000601735.1 | ENSG00000244513.2 | 0.895390016 | 1.995007378 | 0.046042075 |
| IL17C | ENST00000602532.1 | ENSG00000270091.1 | 0.838715031 | 1.875724612 | 0.060693105 |
| IL17C | ENST00000602594.1 | ENSG00000269930.1 | -0.853754488 | -1.901762195 | 0.057202251 |
| IL17C | ENST00000602809.1 | ENSG00000270105.1 | -0.817154212 | -1.843554256 | 0.065248128 |
| IL17C | ENST00000604142.1 | ENSG00000271308.1 | 0.979453176 | 2.208355995 | 0.027219467 |
| IL17C | ENST00000604183.1 | ENSG00000271185.1 | 0.813149926 | 1.800383276 | 0.07180014 |
| IL17C | ENST00000606277.1 | ENSG00000272145.1 | 0.907563403 | 2.002627735 | 0.045217261 |
| IL17C | ENST00000606377.1 | ENSG00000272286.1 | -0.900421944 | -1.999835466 | 0.045518034 |
| IL17C | ENST00000606470.1 | ENSG00000271913.1 | 0.907609962 | 2.035654809 | 0.04178503 |
| IL17C | ENST00000606743.1 | ENSG00000272221.1 | 0.825473015 | 1.837145141 | 0.066188472 |
| IL17C | ENST00000606909.1 | ENSG00000271821.1 | 0.814358941 | 1.799481465 | 0.071942553 |
| IL17C | ENST00000607224.1 | ENSG00000272521.1 | 0.841407297 | 1.877017198 | 0.060515737 |
| IL17C | ENST00000607476.1 | ENSG00000272540.1 | 0.964493445 | 2.148993097 | 0.031634947 |
| IL17C | ENST00000607943.1 | ENSG00000273188.1 | 0.962219832 | 2.182354781 | 0.029083359 |
| IL17C | ENST00000608367.1 | ENSG00000273361.1 | 0.808200577 | 1.799404764 | 0.071954677 |
| IL17C | ENST00000608489.1 | ENSG00000272716.1 | 0.822778086 | 1.83592132 | 0.066369294 |
| IL17C | ENST00000609113.1 | ENSG00000272827.1 | 0.812080872 | 1.84538844 | 0.064981054 |
| IL17C | ENST00000610145.1 | ENSG00000273175.1 | 0.875514975 | 1.954120013 | 0.050687026 |
| IL17C | NR_003604.2 | ZFAS1 | 0.963115564 | 2.187974769 | 0.02867144 |
| IL17C | NR_003605.1 | ZFAS1 | 0.988919717 | 2.180035766 | 0.02925481 |
| IL17C | NR_003606.2 | ZFAS1 | 0.956642008 | 2.128421628 | 0.033302142 |

| | | | | | |
|---|---|---|---|---|---|
| IL17C | NR_026802.1 | FAM74A4 | 0.951894396 | 2.144673101 | 0.031978993 |
| IL17C | NR_026951.1 | LINC00324 | 0.839903549 | 1.886909727 | 0.059172469 |
| IL17C | NR_027052.1 | THAP7-AS1 | 0.84232398 | 1.873970854 | 0.060934442 |
| IL17C | NR_027271.1 | CIRBP-AS1 | 0.973619792 | 2.168981346 | 0.0300841 |
| IL17C | NR_027334.2 | MZF1-AS1 | 0.89801568 | 2.033400072 | 0.04201212 |
| IL17C | NR_036480.1 | VPS9D1-AS1 | 0.946026295 | 2.096267585 | 0.036058461 |
| IL17C | NR_036658.1 | ZFAS1 | 0.961345084 | 2.176344398 | 0.029529518 |
| IL17C | NR_037169.1 | LOC100507547 | 0.896993505 | 1.991893924 | 0.046382698 |
| IL17C | NR_037170.1 | LOC100507547 | 0.855128879 | 1.897945461 | 0.057703267 |
| IL17C | NR_038421.1 | LINC01220 | 0.888128773 | 2.007731182 | 0.044671865 |
| IL17C | NR_038923.1 | SSSCA1-AS1 | 0.837855843 | 1.871238784 | 0.061311991 |
| IL17C | NR_044996.1 | HCG23 | 0.817364505 | 1.851673142 | 0.064072773 |
| IL17C | NR_045114.1 | PVRL3-AS1 | -0.894822628 | -1.99831061 | 0.045682996 |
| IL17C | NR_072981.1 | LINC00957 | 0.946180402 | 2.118534983 | 0.034129785 |
| IL17C | NR_072982.1 | LINC00957 | 0.939603263 | 2.083241436 | 0.037229222 |
| IL17C | NR_103790.1 | LINC00581 | -0.830599278 | -1.873925305 | 0.060940721 |
| IL17C | NR_105010.1 | LINC01333 | 0.982985171 | 2.196433189 | 0.028060952 |
| IL17C | NR_108036.1 | CFAP58-AS1 | 0.923979756 | 2.06628149 | 0.038801908 |
| IL17C | NR_109886.1 | RALY-AS1 | 0.934692888 | 2.104455578 | 0.035338726 |
| IL17C | NR_110630.1 | LOC101927478 | 0.824605557 | 1.845879272 | 0.064909737 |
| IL17C | NR_110941.1 | MIR762HG | 0.824853465 | 1.850152844 | 0.064291523 |
| IL17C | NR_110998.1 | FAM74A4 | 0.951894396 | 2.132427735 | 0.032971699 |
| IL17C | NR_111951.1 | LINC00869 | 0.812154415 | 1.827281738 | 0.06765742 |
| IL17C | NR_111952.1 | LINC00869 | 0.834182388 | 1.871908586 | 0.061219252 |
| IL17C | NR_121188.1 | PGM5P3-AS1 | -0.867385675 | -1.962046153 | 0.049757111 |
| IL17C | NR_121189.1 | PGM5P3-AS1 | -0.921339473 | -2.060702125 | 0.039331467 |
| IL17C | NR_125957.1 | LOC101928626 | -0.928948825 | -2.065856637 | 0.038842018 |
| IL17C | NR_126522.1 | EXOC3-AS1 | 0.934022673 | 2.099330966 | 0.035787736 |
| IL17C | NR_130143.1 | LOC104968399 | 0.880129229 | 1.971408695 | 0.048677147 |
| IL17C | NR_135024.1 | LOC105369747 | 0.884181219 | 1.961986295 | 0.04976408 |
| IL17C | NR_135584.1 | LOC101927596 | 0.810733678 | 1.827234257 | 0.067664555 |
| IL17C | NR_144459.1 | ARSD-AS1 | 0.904718892 | 2.034014237 | 0.04195016 |
| IRF7 | ENST00000318291.4 | ENSG00000177406.4 | 0.869629199 | 1.948705621 | 0.05133059 |
| IRF7 | ENST00000412348.1 | ENSG00000228959.1 | -0.926144354 | -2.088603153 | 0.036743461 |
| IRF7 | ENST00000415205.1 | ENSG00000182057.4 | 0.855673126 | 1.922183172 | 0.054582714 |
| IRF7 | ENST00000417260.1 | ENSG00000231734.4 | -0.914458375 | -2.05865244 | 0.039527545 |
| IRF7 | ENST00000418387.1 | ENSG00000235056.1 | -0.958321772 | -2.169179095 | 0.03006909 |
| IRF7 | ENST00000422017.1 | ENSG00000232227.1 | -0.823081371 | -1.83146752 | 0.067030791 |
| IRF7 | ENST00000423428.1 | ENSG00000224048.1 | -0.824449453 | -1.837615799 | 0.066119039 |
| IRF7 | ENST00000426030.2 | ENSG00000228686.2 | -0.880674055 | -1.961403591 | 0.049831961 |
| IRF7 | ENST00000426302.1 | ENSG00000230454.1 | 0.911736086 | 2.04126469 | 0.041224525 |
| IRF7 | ENST00000430920.1 | ENSG00000234203.1 | 0.904966372 | 2.036339908 | 0.041716235 |
| IRF7 | ENST00000431730.1 | ENSG00000237401.2 | 0.849737722 | 1.921291168 | 0.05469501 |
| IRF7 | ENST00000435287.1 | ENSG00000227220.1 | 0.876579676 | 1.947685222 | 0.051452639 |
| IRF7 | ENST00000436515.1 | ENSG00000224521.1 | -0.953077156 | -2.117840846 | 0.034188549 |
| IRF7 | ENST00000438190.1 | ENSG00000227214.2 | 0.820090457 | 1.848326244 | 0.064555161 |
| IRF7 | ENST00000438623.1 | ENSG00000224521.1 | -0.904805438 | -2.017971527 | 0.043594227 |
| IRF7 | ENST00000442829.1 | ENSG00000225284.1 | 0.965038946 | 2.170057168 | 0.030002515 |
| IRF7 | ENST00000446816.1 | ENSG00000204685.5 | 0.836889124 | 1.855446887 | 0.063532437 |
| IRF7 | ENST00000448748.1 | ENSG00000231238.1 | -0.866729744 | -1.942758115 | 0.052045391 |
| IRF7 | ENST00000448858.1 | ENSG00000237734.1 | -0.89302984 | -1.981758701 | 0.047506254 |

| | | | | | |
|---|---|---|---|---|---|
| IRF7 | ENST00000451034.1 | ENSG00000229805.1 | -0.921968019 | -2.073498862 | 0.038125877 |
| IRF7 | ENST00000451090.1 | ENSG00000235215.2 | -0.863166381 | -1.912671475 | 0.055790123 |
| IRF7 | ENST00000452176.1 | ENSG00000223659.1 | -0.884311459 | -1.991050736 | 0.04647531 |
| IRF7 | ENST00000455373.1 | ENSG00000226097.1 | -0.862702162 | -1.963579368 | 0.049578892 |
| IRF7 | ENST00000457043.1 | ENSG00000231365.1 | -0.819190726 | -1.827317031 | 0.067652116 |
| IRF7 | ENST00000457371.1 | ENSG00000237401.2 | 0.814926354 | 1.810870016 | 0.070160975 |
| IRF7 | ENST00000458154.1 | ENSG00000235578.1 | 0.803212482 | 1.799630097 | 0.071919066 |
| IRF7 | ENST00000468165.1 | ENSG00000239480.1 | 0.802671835 | 1.783413571 | 0.074519006 |
| IRF7 | ENST00000485338.1 | ENSG00000239641.1 | -0.842551488 | -1.895960075 | 0.057965324 |
| IRF7 | ENST00000489077.1 | ENSG00000244198.1 | 0.829799996 | 1.857860527 | 0.063188823 |
| IRF7 | ENST00000489690.1 | ENSG00000243944.1 | -0.951318263 | -2.168521713 | 0.030119014 |
| IRF7 | ENST00000498693.1 | ENSG00000244198.1 | 0.80378806 | 1.776817367 | 0.075598293 |
| IRF7 | ENST00000502467.1 | ENSG00000250530.1 | -0.823044869 | -1.853621206 | 0.063793372 |
| IRF7 | ENST00000503505.1 | ENSG00000248629.1 | -0.907946073 | -2.018206285 | 0.043569782 |
| IRF7 | ENST00000503723.1 | ENSG00000250472.1 | -0.876258428 | -1.973301233 | 0.048461253 |
| IRF7 | ENST00000504578.1 | ENSG00000251513.1 | -0.843092055 | -1.883361303 | 0.059651421 |
| IRF7 | ENST00000509192.1 | ENSG00000250765.1 | 0.827906735 | 1.857573153 | 0.063229654 |
| IRF7 | ENST00000515128.1 | ENSG00000248215.1 | -0.955520672 | -2.121650951 | 0.03386706 |
| IRF7 | ENST00000518837.1 | ENSG00000253947.1 | -0.816187375 | -1.856042351 | 0.063447522 |
| IRF7 | ENST00000521653.1 | ENSG00000253301.1 | 0.845600451 | 1.899406732 | 0.057511019 |
| IRF7 | ENST00000522390.1 | ENSG00000254262.1 | -0.872896515 | -1.968447253 | 0.0490166 |
| IRF7 | ENST00000522600.1 | ENSG00000246582.2 | 0.843222902 | 1.878185386 | 0.060355809 |
| IRF7 | ENST00000527086.1 | ENSG00000255182.1 | 0.851274355 | 1.906979449 | 0.056523249 |
| IRF7 | ENST00000528887.1 | ENSG00000254501.1 | 0.800617753 | 1.793791758 | 0.072846413 |
| IRF7 | ENST00000529247.1 | ENSG00000254741.1 | 0.875459137 | 1.940761638 | 0.052287196 |
| IRF7 | ENST00000534178.1 | ENSG00000255120.1 | 0.913495402 | 2.045230101 | 0.040832178 |
| IRF7 | ENST00000537032.1 | ENSG00000255933.1 | 0.849820065 | 1.926980415 | 0.053982071 |
| IRF7 | ENST00000537850.1 | ENSG00000251002.3 | 0.897529712 | 2.020557379 | 0.043325605 |
| IRF7 | ENST00000543275.1 | ENSG00000256944.1 | 0.860415705 | 1.929994538 | 0.053607515 |
| IRF7 | ENST00000543403.1 | ENSG00000256684.1 | -0.87121305 | -1.947112048 | 0.051521303 |
| IRF7 | ENST00000543494.1 | ENSG00000256514.1 | 0.888602829 | 2.003163167 | 0.045159778 |
| IRF7 | ENST00000548210.1 | ENSG00000257784.1 | 0.812461074 | 1.802368763 | 0.071487406 |
| IRF7 | ENST00000549683.1 | ENSG00000257953.1 | 0.889642816 | 1.980426237 | 0.047655654 |
| IRF7 | ENST00000549806.1 | ENSG00000257252.1 | 0.862032788 | 1.938078225 | 0.05261368 |
| IRF7 | ENST00000550263.1 | ENSG00000257605.1 | 0.860935408 | 1.922343175 | 0.054562591 |
| IRF7 | ENST00000550279.1 | ENSG00000258338.1 | -0.89356999 | -2.013878122 | 0.044022331 |
| IRF7 | ENST00000554431.1 | ENSG00000258616.1 | -0.929665388 | -2.09111989 | 0.036517318 |
| IRF7 | ENST00000557602.1 | ENSG00000258616.1 | -0.934057856 | -2.073234158 | 0.038150493 |
| IRF7 | ENST00000558237.1 | ENSG00000259684.1 | -0.850212917 | -1.893786047 | 0.058253414 |
| IRF7 | ENST00000558575.1 | ENSG00000259687.1 | 0.811818208 | 1.806831083 | 0.070788621 |
| IRF7 | ENST00000560963.1 | ENSG00000259370.1 | 0.860317162 | 1.895740768 | 0.057994332 |
| IRF7 | ENST00000561699.1 | ENSG00000259813.1 | 0.80847548 | 1.798800534 | 0.072050239 |
| IRF7 | ENST00000562191.1 | ENSG00000261292.1 | -0.865082301 | -1.923691579 | 0.054393254 |
| IRF7 | ENST00000562995.1 | ENSG00000261253.1 | 0.879897874 | 1.934118533 | 0.053098556 |
| IRF7 | ENST00000563610.1 | ENSG00000260051.1 | 0.961128664 | 2.133036887 | 0.0329217 |
| IRF7 | ENST00000563611.1 | ENSG00000261583.1 | 0.872443652 | 1.965760757 | 0.049326253 |
| IRF7 | ENST00000564809.1 | ENSG00000261471.1 | 0.884242753 | 1.986132473 | 0.047018616 |
| IRF7 | ENST00000565823.1 | ENSG00000260686.1 | -0.837274335 | -1.851033943 | 0.06416467 |
| IRF7 | ENST00000568659.1 | ENSG00000260004.1 | -0.815431383 | -1.832120483 | 0.066933472 |
| IRF7 | ENST00000569981.1 | ENSG00000238045.5 | 0.816406222 | 1.830644369 | 0.067153641 |
| IRF7 | ENST00000570493.2 | ENSG00000261898.2 | 0.891032401 | 1.966806995 | 0.049205466 |

| | | | | | |
|---|---|---|---|---|---|
| IRF7 | ENST00000570974.1 | ENSG00000263300.1 | 0.927291722 | 2.071243493 | 0.038336047 |
| IRF7 | ENST00000577853.1 | ENSG00000264207.1 | 0.934858173 | 2.086216253 | 0.036959039 |
| IRF7 | ENST00000580311.1 | ENSG00000266803.1 | -0.871406424 | -1.954385548 | 0.050655639 |
| IRF7 | ENST00000582044.1 | ENSG00000263715.2 | 0.813691371 | 1.833325787 | 0.066754137 |
| IRF7 | ENST00000585559.1 | ENSG00000267117.1 | 0.835916767 | 1.863828501 | 0.062345792 |
| IRF7 | ENST00000585810.1 | ENSG00000236172.2 | 0.895032274 | 2.013979034 | 0.044011735 |
| IRF7 | ENST00000588380.1 | ENSG00000266990.1 | 0.858876331 | 1.950168395 | 0.051156051 |
| IRF7 | ENST00000588402.1 | ENSG00000267006.1 | -0.930732348 | -2.039891721 | 0.041361112 |
| IRF7 | ENST00000590328.1 | ENSG00000256995.2 | -0.875726595 | -1.936532331 | 0.052802537 |
| IRF7 | ENST00000591174.1 | ENSG00000267289.1 | 0.918066996 | 2.061328885 | 0.039271674 |
| IRF7 | ENST00000592816.1 | ENSG00000236172.2 | 0.897333874 | 2.007533389 | 0.044692899 |
| IRF7 | ENST00000595007.1 | ENSG00000231876.3 | -0.805481626 | -1.79212232 | 0.073113374 |
| IRF7 | ENST00000596091.1 | ENSG00000227733.4 | -0.859731903 | -1.936236696 | 0.052838718 |
| IRF7 | ENST00000596887.1 | ENSG00000237031.3 | -0.894436878 | -2.021573619 | 0.04322042 |
| IRF7 | ENST00000598092.1 | ENSG00000228065.6 | -0.926044196 | -2.096757628 | 0.036015036 |
| IRF7 | ENST00000599259.1 | ENSG00000269352.1 | 0.819781505 | 1.842263194 | 0.065436661 |
| IRF7 | ENST00000600242.1 | ENSG00000269583.1 | 0.817409933 | 1.805673002 | 0.070969432 |
| IRF7 | ENST00000600716.1 | ENSG00000269487.1 | 0.954808613 | 2.119368151 | 0.034059366 |
| IRF7 | ENST00000600726.1 | ENSG00000267858.1 | 0.934591555 | 2.090613788 | 0.036562699 |
| IRF7 | ENST00000604142.1 | ENSG00000271308.1 | 0.845163674 | 1.854349751 | 0.063689139 |
| IRF7 | ENST00000604183.1 | ENSG00000271185.1 | 0.911521892 | 2.032585841 | 0.042094383 |
| IRF7 | ENST00000606010.1 | ENSG00000272249.1 | -0.85267884 | -1.93127273 | 0.053449334 |
| IRF7 | ENST00000607148.1 | ENSG00000272477.1 | -0.818544028 | -1.838116438 | 0.06604525 |
| IRF7 | ENST00000607476.1 | ENSG00000272540.1 | 0.840261891 | 1.856872535 | 0.063329291 |
| IRF7 | ENST00000607549.1 | ENSG00000272293.1 | -0.855764088 | -1.906190044 | 0.056625553 |
| IRF7 | ENST00000607943.1 | ENSG00000273188.1 | 0.928870182 | 2.026552631 | 0.042708185 |
| IRF7 | ENST00000608259.1 | ENSG00000272627.1 | -0.926015047 | -2.039331842 | 0.04141692 |
| IRF7 | ENST00000608367.1 | ENSG00000273361.1 | 0.904560691 | 2.014480284 | 0.043959133 |
| IRF7 | ENST00000608489.1 | ENSG00000272716.1 | 0.916240983 | 2.031352861 | 0.042219213 |
| IRF7 | ENST00000609113.1 | ENSG00000272827.1 | 0.905649951 | 2.019194415 | 0.043467017 |
| IRF7 | ENST00000609813.1 | ENSG00000272719.1 | 0.901026449 | 2.01035882 | 0.044393226 |
| IRF7 | ENST00000609955.1 | ENSG00000273275.1 | -0.837360441 | -1.856751462 | 0.063346522 |
| IRF7 | ENST00000609976.1 | ENSG00000272582.1 | 0.838453456 | 1.859040767 | 0.06302136 |
| IRF7 | NR_003605.1 | ZFAS1 | 0.826710318 | 1.831552506 | 0.067018118 |
| IRF7 | NR_026802.1 | FAM74A4 | 0.809204927 | 1.798068591 | 0.072166139 |
| IRF7 | NR_026951.1 | LINC00324 | 0.820302449 | 1.826863099 | 0.067720356 |
| IRF7 | NR_027052.1 | THAP7-AS1 | 0.841298126 | 1.872109741 | 0.061191423 |
| IRF7 | NR_036480.1 | VPS9D1-AS1 | 0.916104932 | 2.056299991 | 0.039753609 |
| IRF7 | NR_037169.1 | LOC100507547 | 0.809744316 | 1.814060802 | 0.069668365 |
| IRF7 | NR_038421.1 | LINC01220 | 0.917555443 | 2.047743658 | 0.040585124 |
| IRF7 | NR_038923.1 | SSSCA1-AS1 | 0.872400879 | 1.933715157 | 0.053148159 |
| IRF7 | NR_047498.1 | LINC00853 | 0.806014682 | 1.816424539 | 0.069305274 |
| IRF7 | NR_072981.1 | LINC00957 | 0.827564099 | 1.867996595 | 0.061762546 |
| IRF7 | NR_072982.1 | LINC00957 | 0.815128502 | 1.7965915 | 0.072400495 |
| IRF7 | NR_109831.1 | RASSF1-AS1 | 0.800293647 | 1.790684915 | 0.073343872 |
| IRF7 | NR_110117.1 | LOC101927769 | -0.855160869 | -1.912760657 | 0.0557787 |
| IRF7 | NR_110245.1 | LOC101929282 | -0.869506164 | -1.928447428 | 0.053799497 |
| IRF7 | NR_110556.1 | LOC102724890 | -0.847993178 | -1.894975249 | 0.058095681 |
| IRF7 | NR_110630.1 | LOC101927478 | 0.918755347 | 2.058754668 | 0.039517746 |
| IRF7 | NR_110998.1 | FAM74A4 | 0.809204927 | 1.825262891 | 0.067961367 |
| IRF7 | NR_111951.1 | LINC00869 | 0.875085902 | 1.989828395 | 0.046609842 |

| | | | | | |
|---|---|---|---|---|---|
| IRF7 | NR_111952.1 | LINC00869 | 0.857519847 | 1.923169508 | 0.054458765 |
| IRF7 | NR_111953.1 | LINC00869 | 0.866463925 | 1.931689006 | 0.053397903 |
| IRF7 | NR_125849.1 | LOC101928140 | -0.843207726 | -1.896616961 | 0.05787851 |
| IRF7 | NR_125957.1 | LOC101928626 | -0.884311459 | -1.966525984 | 0.049237884 |
| IRF7 | NR_126522.1 | EXOC3-AS1 | 0.864612224 | 1.905294763 | 0.056741766 |
| IRF7 | NR_135024.1 | LOC105369747 | 0.876640758 | 1.963450425 | 0.04959386 |
| IRF7 | NR_135040.1 | LOC101927038 | 0.846416628 | 1.887987829 | 0.059027584 |
| IRF7 | NR_135097.1 | LOC105369443 | -0.87121305 | -1.95421843 | 0.050675391 |
| IRF7 | NR_136569.1 | LINC01660 | -0.820802111 | -1.819680495 | 0.068807675 |
| IRF7 | NR_138038.1 | LINC00677 | 0.814620462 | 1.822553305 | 0.068371073 |
| IRF7 | NR_144459.1 | ARSD-AS1 | 0.950242175 | 2.117269427 | 0.034236988 |
| JUNB | ENST00000381106.4 | ENSG00000205663.5 | -0.827811682 | -1.850734061 | 0.064207821 |
| JUNB | ENST00000414098.2 | ENSG00000234428.2 | -0.822003162 | -1.806404278 | 0.070855214 |
| JUNB | ENST00000414896.1 | ENSG00000223374.1 | -0.864993089 | -1.91584241 | 0.055385158 |
| JUNB | ENST00000415106.1 | ENSG00000226733.1 | -0.892224177 | -1.987402583 | 0.046877802 |
| JUNB | ENST00000415205.1 | ENSG00000182057.4 | 0.949999713 | 2.154230173 | 0.031222126 |
| JUNB | ENST00000417260.1 | ENSG00000231734.4 | -0.90982071 | -2.046914146 | 0.040666515 |
| JUNB | ENST00000418387.1 | ENSG00000235056.1 | -0.828620324 | -1.843417124 | 0.065268132 |
| JUNB | ENST00000421006.1 | ENSG00000234548.1 | -0.831292913 | -1.880624464 | 0.060023021 |
| JUNB | ENST00000422763.1 | ENSG00000231131.2 | -0.807867171 | -1.813449868 | 0.069762464 |
| JUNB | ENST00000423428.1 | ENSG00000224048.1 | -0.920801162 | -2.063428879 | 0.039071899 |
| JUNB | ENST00000423796.1 | ENSG00000235146.2 | -0.801848664 | -1.796486067 | 0.072417247 |
| JUNB | ENST00000426237.2 | ENSG00000235527.2 | 0.805059521 | 1.792953666 | 0.072980333 |
| JUNB | ENST00000426302.1 | ENSG00000230454.1 | 0.946490068 | 2.118274675 | 0.034151812 |
| JUNB | ENST00000429080.1 | ENSG00000233047.1 | -0.951823063 | -2.150323889 | 0.031529604 |
| JUNB | ENST00000430920.1 | ENSG00000234203.1 | 0.884972293 | 1.946047974 | 0.051648977 |
| JUNB | ENST00000431730.1 | ENSG00000237401.2 | 0.815818909 | 1.829194612 | 0.067370459 |
| JUNB | ENST00000433905.2 | ENSG00000229299.2 | 0.86362696 | 1.940311126 | 0.05234189 |
| JUNB | ENST00000434292.1 | ENSG00000229796.1 | -0.837435037 | -1.872568217 | 0.061128034 |
| JUNB | ENST00000435287.1 | ENSG00000227220.1 | 0.937369965 | 2.087940661 | 0.036803188 |
| JUNB | ENST00000436515.1 | ENSG00000224521.1 | -0.86779992 | -1.934314159 | 0.053074513 |
| JUNB | ENST00000438173.2 | ENSG00000227733.4 | -0.887058907 | -1.988296019 | 0.046778961 |
| JUNB | ENST00000438190.1 | ENSG00000227214.2 | 0.807154091 | 1.810844135 | 0.070164982 |
| JUNB | ENST00000438623.1 | ENSG00000224521.1 | -0.875014826 | -1.944296424 | 0.051859716 |
| JUNB | ENST00000438969.2 | ENSG00000228031.2 | -0.879928202 | -1.964999349 | 0.049414313 |
| JUNB | ENST00000442017.1 | ENSG00000229660.1 | 0.868423903 | 1.935878591 | 0.052882572 |
| JUNB | ENST00000442829.1 | ENSG00000225284.1 | 0.850507718 | 1.921178069 | 0.054709262 |
| JUNB | ENST00000446562.1 | ENSG00000233896.1 | 0.808441792 | 1.796880465 | 0.072354599 |
| JUNB | ENST00000446816.1 | ENSG00000204685.5 | 0.803843194 | 1.793306316 | 0.072923958 |
| JUNB | ENST00000448858.1 | ENSG00000237734.1 | -0.986326882 | -2.187913768 | 0.028675884 |
| JUNB | ENST00000450696.1 | ENSG00000235146.2 | -0.837949277 | -1.877976727 | 0.060384349 |
| JUNB | ENST00000451034.1 | ENSG00000229805.1 | -0.839885276 | -1.861582016 | 0.06266203 |
| JUNB | ENST00000451090.1 | ENSG00000235215.2 | -0.872308563 | -1.933535814 | 0.053170226 |
| JUNB | ENST00000451507.1 | ENSG00000229539.1 | 0.809402835 | 1.815363753 | 0.069468028 |
| JUNB | ENST00000455373.1 | ENSG00000226097.1 | -0.94155944 | -2.093695671 | 0.036287099 |
| JUNB | ENST00000457998.2 | ENSG00000233006.2 | 0.874226906 | 1.960432749 | 0.049945231 |
| JUNB | ENST00000468165.1 | ENSG00000239480.1 | 0.907572465 | 2.041602807 | 0.041190947 |
| JUNB | ENST00000488310.1 | ENSG00000240449.1 | 0.822617402 | 1.853333198 | 0.063834616 |
| JUNB | ENST00000489077.1 | ENSG00000244198.1 | 0.871703573 | 1.957043097 | 0.050342402 |
| JUNB | ENST00000503505.1 | ENSG00000248629.1 | -0.857330802 | -1.930731506 | 0.053516265 |
| JUNB | ENST00000503723.1 | ENSG00000250472.1 | -0.972659118 | -2.177696347 | 0.029428651 |

| | | | | | |
|---|---|---|---|---|---|
| JUNB | ENST00000504578.1 | ENSG00000251513.1 | -0.912103152 | -2.057655007 | 0.039623262 |
| JUNB | ENST00000506723.2 | ENSG00000249484.4 | -0.972051651 | -2.155542125 | 0.031119436 |
| JUNB | ENST00000509192.1 | ENSG00000250765.1 | 0.826602689 | 1.859061168 | 0.063018469 |
| JUNB | ENST00000509453.1 | ENSG00000249145.1 | 0.870627373 | 1.959978907 | 0.049998256 |
| JUNB | ENST00000510570.1 | ENSG00000250438.1 | -0.803652113 | -1.779446728 | 0.075166551 |
| JUNB | ENST00000515128.1 | ENSG00000248215.1 | -0.875529078 | -1.961953201 | 0.049767933 |
| JUNB | ENST00000518837.1 | ENSG00000253947.1 | -0.950885525 | -2.117899984 | 0.034183539 |
| JUNB | ENST00000520603.1 | ENSG00000254001.1 | -0.805436479 | -1.812592893 | 0.069894635 |
| JUNB | ENST00000521294.1 | ENSG00000253664.1 | 0.880906536 | 1.97616362 | 0.048136249 |
| JUNB | ENST00000521653.1 | ENSG00000253301.1 | 0.94297773 | 2.115773457 | 0.03436408 |
| JUNB | ENST00000522281.1 | ENSG00000253376.1 | -0.93632694 | -2.07964297 | 0.037558293 |
| JUNB | ENST00000522300.1 | ENSG00000249484.4 | -0.918264488 | -2.055622721 | 0.039818896 |
| JUNB | ENST00000522390.1 | ENSG00000254262.1 | -0.845690398 | -1.888076024 | 0.059015745 |
| JUNB | ENST00000523806.1 | ENSG00000253616.1 | 0.86193577 | 1.927721243 | 0.053889808 |
| JUNB | ENST00000527086.1 | ENSG00000255182.1 | 0.826586257 | 1.848393838 | 0.064545389 |
| JUNB | ENST00000527274.2 | ENSG00000255517.2 | 0.923495686 | 2.059618612 | 0.039435015 |
| JUNB | ENST00000529247.1 | ENSG00000254741.1 | 0.801883452 | 1.774662288 | 0.075953666 |
| JUNB | ENST00000531977.1 | ENSG00000224023.6 | -0.886691345 | -1.992055984 | 0.046364916 |
| JUNB | ENST00000534178.1 | ENSG00000255120.1 | 0.800384886 | 1.773012198 | 0.076226686 |
| JUNB | ENST00000535914.1 | ENSG00000256894.1 | -0.93230931 | -2.058748891 | 0.0395183 |
| JUNB | ENST00000537032.1 | ENSG00000255933.1 | 0.894948333 | 2.011724415 | 0.044248996 |
| JUNB | ENST00000537850.1 | ENSG00000251002.3 | 0.952527816 | 2.168491865 | 0.030121283 |
| JUNB | ENST00000543403.1 | ENSG00000256684.1 | -0.872334289 | -1.932969333 | 0.053239976 |
| JUNB | ENST00000543494.1 | ENSG00000256514.1 | 0.830736797 | 1.849775069 | 0.064345976 |
| JUNB | ENST00000548210.1 | ENSG00000257784.1 | 0.895759834 | 2.016233288 | 0.043775587 |
| JUNB | ENST00000549616.1 | ENSG00000258168.1 | -0.843784463 | -1.883832161 | 0.059587682 |
| JUNB | ENST00000549683.1 | ENSG00000257953.1 | 0.802769925 | 1.77857889 | 0.075308828 |
| JUNB | ENST00000550263.1 | ENSG00000257605.1 | 0.846582758 | 1.889318813 | 0.058849122 |
| JUNB | ENST00000550279.1 | ENSG00000258338.1 | -0.863911193 | -1.944491675 | 0.051836188 |
| JUNB | ENST00000551135.1 | ENSG00000258294.1 | -0.894926229 | -2.018189563 | 0.043571523 |
| JUNB | ENST00000551699.1 | ENSG00000257467.1 | -0.857390724 | -1.902195397 | 0.057145614 |
| JUNB | ENST00000552541.1 | ENSG00000258294.1 | -0.915269467 | -2.045186228 | 0.040836502 |
| JUNB | ENST00000552634.1 | ENSG00000257496.1 | 0.85508241 | 1.923545772 | 0.054411544 |
| JUNB | ENST00000555460.1 | ENSG00000259042.1 | 0.85309738 | 1.899544418 | 0.057492932 |
| JUNB | ENST00000555913.1 | ENSG00000259077.1 | -0.817684521 | -1.815142006 | 0.069502089 |
| JUNB | ENST00000558575.1 | ENSG00000259687.1 | 0.836894699 | 1.859075738 | 0.063016404 |
| JUNB | ENST00000560963.1 | ENSG00000259370.1 | 0.810638486 | 1.832888764 | 0.066819115 |
| JUNB | ENST00000562191.1 | ENSG00000261292.1 | -0.861775924 | -1.93405739 | 0.053106072 |
| JUNB | ENST00000562995.1 | ENSG00000261253.1 | 0.811252575 | 1.799894354 | 0.071877321 |
| JUNB | ENST00000563018.1 | ENSG00000260193.1 | 0.884945458 | 1.965164562 | 0.049395194 |
| JUNB | ENST00000563610.1 | ENSG00000260051.1 | 0.949948002 | 2.140632926 | 0.032303651 |
| JUNB | ENST00000563611.1 | ENSG00000261583.1 | 0.949558277 | 2.093134555 | 0.036337145 |
| JUNB | ENST00000564809.1 | ENSG00000261471.1 | 0.956095085 | 2.113443261 | 0.034562847 |
| JUNB | ENST00000565310.1 | ENSG00000261118.1 | 0.929825239 | 2.068036473 | 0.038636594 |
| JUNB | ENST00000565735.1 | ENSG00000261213.1 | -0.966348255 | -2.164382468 | 0.030435008 |
| JUNB | ENST00000566449.1 | ENSG00000259791.1 | -0.902884066 | -2.038562753 | 0.041493686 |
| JUNB | ENST00000568410.1 | ENSG00000260277.1 | -0.800656246 | -1.799393341 | 0.071956482 |
| JUNB | ENST00000568659.1 | ENSG00000260004.1 | -0.800252641 | -1.765647768 | 0.077454961 |
| JUNB | ENST00000570700.1 | ENSG00000263011.1 | 0.812274978 | 1.832209562 | 0.066920205 |
| JUNB | ENST00000571660.1 | ENSG00000262848.1 | 0.804075028 | 1.793873874 | 0.072833302 |
| JUNB | ENST00000576021.1 | ENSG00000262413.1 | 0.831362595 | 1.854323736 | 0.063692858 |

| | | | | | |
|---|---|---|---|---|---|
| JUNB | ENST00000576271.1 | ENSG00000263342.1 | 0.848594199 | 1.901173215 | 0.057279328 |
| JUNB | ENST00000577853.1 | ENSG00000264207.1 | 0.974241344 | 2.17781403 | 0.029419885 |
| JUNB | ENST00000579775.1 | ENSG00000264108.1 | 0.927514804 | 2.048628794 | 0.040498427 |
| JUNB | ENST00000580622.1 | ENSG00000264634.1 | 0.85493457 | 1.90017034 | 0.057410769 |
| JUNB | ENST00000582044.1 | ENSG00000263715.2 | 0.887267815 | 2.011994336 | 0.044220534 |
| JUNB | ENST00000585684.1 | ENSG00000267057.1 | -0.823188967 | -1.8390507 | 0.065907729 |
| JUNB | ENST00000585761.1 | ENSG00000267198.1 | 0.858274989 | 1.927133488 | 0.053962996 |
| JUNB | ENST00000585810.1 | ENSG00000236172.2 | 0.854529675 | 1.915216619 | 0.055464884 |
| JUNB | ENST00000586348.1 | ENSG00000267198.1 | 0.823662742 | 1.857102905 | 0.063296515 |
| JUNB | ENST00000588402.1 | ENSG00000267006.1 | -0.888537803 | -1.971565178 | 0.048659266 |
| JUNB | ENST00000588842.1 | ENSG00000235779.3 | -0.910906609 | -2.03735579 | 0.041614401 |
| JUNB | ENST00000589395.1 | ENSG00000267143.1 | 0.815650488 | 1.830643482 | 0.067153774 |
| JUNB | ENST00000591174.1 | ENSG00000267289.1 | 0.80576849 | 1.800341547 | 0.071806724 |
| JUNB | ENST00000591621.1 | ENSG00000232116.2 | -0.860867515 | -1.922449666 | 0.054549201 |
| JUNB | ENST00000592022.1 | ENSG00000267383.2 | -0.841922234 | -1.892410975 | 0.058436245 |
| JUNB | ENST00000592525.1 | ENSG00000267214.1 | 0.879408532 | 2.016866947 | 0.0437094 |
| JUNB | ENST00000592816.1 | ENSG00000236172.2 | 0.875468101 | 1.941594283 | 0.052186235 |
| JUNB | ENST00000593175.1 | ENSG00000229036.3 | -0.875119251 | -1.948298768 | 0.051379224 |
| JUNB | ENST00000594776.1 | ENSG00000269807.1 | 0.914294679 | 2.035681398 | 0.041782358 |
| JUNB | ENST00000594850.1 | ENSG00000268093.1 | 0.837581473 | 1.864095237 | 0.062308331 |
| JUNB | ENST00000595478.1 | ENSG00000237031.3 | -0.87905906 | -1.967995507 | 0.049068555 |
| JUNB | ENST00000596091.1 | ENSG00000227733.4 | -0.955815115 | -2.142709957 | 0.032136395 |
| JUNB | ENST00000596567.1 | ENSG00000226647.2 | -0.910509771 | -2.046802916 | 0.040677439 |
| JUNB | ENST00000596887.1 | ENSG00000237031.3 | -0.888523616 | -1.972349733 | 0.048569696 |
| JUNB | ENST00000597680.1 | ENSG00000269574.1 | -0.813526961 | -1.81270123 | 0.069877915 |
| JUNB | ENST00000597755.1 | ENSG00000236194.2 | -0.820256591 | -1.829065076 | 0.06738986 |
| JUNB | ENST00000597906.1 | ENSG00000268566.1 | -0.822104658 | -1.827968737 | 0.067554244 |
| JUNB | ENST00000598092.1 | ENSG00000228065.6 | -0.957834418 | -2.137489279 | 0.032558217 |
| JUNB | ENST00000600242.1 | ENSG00000269583.1 | 0.849341339 | 1.893330496 | 0.058313932 |
| JUNB | ENST00000600726.1 | ENSG00000267858.1 | 0.88911426 | 1.981117873 | 0.047578056 |
| JUNB | ENST00000602949.1 | ENSG00000270030.1 | 0.841427783 | 1.881784029 | 0.059865345 |
| JUNB | ENST00000604142.1 | ENSG00000271308.1 | 0.851273882 | 1.938367158 | 0.052578444 |
| JUNB | ENST00000604183.1 | ENSG00000271185.1 | 0.968515041 | 2.150510784 | 0.031514834 |
| JUNB | ENST00000606743.1 | ENSG00000272221.1 | 0.805069634 | 1.808735313 | 0.070492134 |
| JUNB | ENST00000606869.1 | ENSG00000272349.1 | -0.882827964 | -1.963227227 | 0.049619777 |
| JUNB | ENST00000606909.1 | ENSG00000271821.1 | 0.88847264 | 2.001225446 | 0.0453681 |
| JUNB | ENST00000607148.1 | ENSG00000272477.1 | -0.877338886 | -1.961679991 | 0.049799753 |
| JUNB | ENST00000607476.1 | ENSG00000272540.1 | 0.867230782 | 1.929327602 | 0.053690205 |
| JUNB | ENST00000607943.1 | ENSG00000273188.1 | 0.923504538 | 2.084407417 | 0.037123124 |
| JUNB | ENST00000608088.1 | ENSG00000272632.1 | -0.886553367 | -1.978260636 | 0.047899311 |
| JUNB | ENST00000608259.1 | ENSG00000272627.1 | -0.898913372 | -2.03286469 | 0.042066195 |
| JUNB | ENST00000608489.1 | ENSG00000272716.1 | 0.978666404 | 2.199898986 | 0.027814063 |
| JUNB | ENST00000608759.1 | ENSG00000273464.1 | -0.865571589 | -1.949742491 | 0.051206819 |
| JUNB | ENST00000609113.1 | ENSG00000272827.1 | 0.854071056 | 1.923498724 | 0.054417447 |
| JUNB | ENST00000609807.1 | ENSG00000272700.1 | -0.921880525 | -2.044132324 | 0.040940477 |
| JUNB | ENST00000609976.1 | ENSG00000272582.1 | 0.819242675 | 1.825793986 | 0.067881299 |
| JUNB | ENST00000610270.1 | ENSG00000272576.1 | -0.843072602 | -1.895982199 | 0.057962398 |
| JUNB | NR_027052.1 | THAP7-AS1 | 0.835131218 | 1.871920539 | 0.061217598 |
| JUNB | NR_027402.1 | FAM223B | 0.887171212 | 1.972353444 | 0.048569273 |
| JUNB | NR_033914.1 | LINC00254 | -0.807596312 | -1.80816427 | 0.070580938 |
| JUNB | NR_037169.1 | LOC100507547 | 0.838406575 | 1.894164945 | 0.05820312 |

| | | | | | |
|---|---|---|---|---|---|
| JUNB | NR_037170.1 | LOC100507547 | 0.814053868 | 1.823251304 | 0.068265338 |
| JUNB | NR_038421.1 | LINC01220 | 0.949796177 | 2.111963106 | 0.034689614 |
| JUNB | NR_038923.1 | SSSCA1-AS1 | 0.911480983 | 2.041995597 | 0.041151968 |
| JUNB | NR_045114.1 | PVRL3-AS1 | -0.858656043 | -1.924269134 | 0.054320857 |
| JUNB | NR_046871.1 | LINC00333 | -0.946484093 | -2.140377995 | 0.032324231 |
| JUNB | NR_047698.1 | VWC2L-IT1 | -0.836617203 | -1.880233653 | 0.060076241 |
| JUNB | NR_072981.1 | LINC00957 | 0.819654626 | 1.848779387 | 0.064489675 |
| JUNB | NR_072982.1 | LINC00957 | 0.817966198 | 1.842516459 | 0.065399641 |
| JUNB | NR_108106.1 | LINC01135 | 0.801486959 | 1.786029092 | 0.074094548 |
| JUNB | NR_109831.1 | RASSF1-AS1 | 0.835153582 | 1.871180077 | 0.061320125 |
| JUNB | NR_109877.1 | LINC01470 | -0.93443322 | -2.103790991 | 0.035396683 |
| JUNB | NR_110117.1 | LOC101927769 | -0.898164778 | -2.036335407 | 0.041716687 |
| JUNB | NR_110245.1 | LOC101929282 | -0.995540997 | -2.231193585 | 0.025668309 |
| JUNB | NR_110556.1 | LOC102724890 | -0.972587738 | -2.174377704 | 0.029676781 |
| JUNB | NR_110630.1 | LOC101927478 | 0.989064389 | 2.207011161 | 0.02731328 |
| JUNB | NR_110941.1 | MIR762HG | 0.818521801 | 1.832849107 | 0.066825014 |
| JUNB | NR_111951.1 | LINC00869 | 0.842430452 | 1.896266744 | 0.057924781 |
| JUNB | NR_111952.1 | LINC00869 | 0.829149261 | 1.851955266 | 0.064032247 |
| JUNB | NR_111953.1 | LINC00869 | 0.837018603 | 1.883264505 | 0.059664531 |
| JUNB | NR_125849.1 | LOC101928140 | -0.884682728 | -1.98776173 | 0.046838048 |
| JUNB | NR_131204.1 | XACT | 0.848670611 | 1.923514301 | 0.054415492 |
| JUNB | NR_134520.1 | LOC727993 | 0.812511702 | 1.815621592 | 0.069428439 |
| JUNB | NR_135040.1 | LOC101927038 | 0.89777727 | 2.003403405 | 0.045134006 |
| JUNB | NR_135041.1 | LOC101927038 | 0.839683619 | 1.899119846 | 0.05754872 |
| JUNB | NR_135097.1 | LOC105369443 | -0.872334289 | -1.939716241 | 0.052414185 |
| JUNB | NR_135584.1 | LOC101927596 | 0.929934369 | 2.100812807 | 0.035657402 |
| JUNB | NR_136215.1 | VCAN-AS1 | -0.857252033 | -1.942299925 | 0.052100802 |
| JUNB | NR_144459.1 | ARSD-AS1 | 0.805258112 | 1.778804703 | 0.075271786 |
| KISS1R | ENST00000318291.4 | ENSG00000177406.4 | 0.81456976 | 1.820879401 | 0.068625191 |
| KISS1R | ENST00000399186.2 | ENSG00000214888.2 | 0.948329857 | 2.099708409 | 0.035754499 |
| KISS1R | ENST00000412085.1 | ENSG00000233825.1 | 0.804103663 | 1.805447125 | 0.071004743 |
| KISS1R | ENST00000412759.1 | ENSG00000236933.1 | 0.811170156 | 1.816039213 | 0.069364357 |
| KISS1R | ENST00000412896.1 | ENSG00000197585.5 | -0.824258329 | -1.859599492 | 0.062942211 |
| KISS1R | ENST00000413650.1 | ENSG00000230880.2 | 0.920388499 | 2.03669159 | 0.041680958 |
| KISS1R | ENST00000413887.1 | ENSG00000236948.1 | -0.838287663 | -1.874979689 | 0.060795518 |
| KISS1R | ENST00000413991.1 | ENSG00000237614.1 | 0.917755427 | 2.057756183 | 0.039613544 |
| KISS1R | ENST00000414740.2 | ENSG00000229646.2 | 0.890699716 | 2.003176066 | 0.045158394 |
| KISS1R | ENST00000416657.1 | ENSG00000235858.1 | 0.904295435 | 2.005635695 | 0.044895131 |
| KISS1R | ENST00000418972.1 | ENSG00000225044.1 | -0.842745362 | -1.879440182 | 0.060184414 |
| KISS1R | ENST00000419734.1 | ENSG00000234646.1 | -0.817676658 | -1.857744522 | 0.063205303 |
| KISS1R | ENST00000420315.1 | ENSG00000228072.1 | 0.840911179 | 1.875115402 | 0.060776849 |
| KISS1R | ENST00000420365.1 | ENSG00000225214.1 | 0.882256513 | 1.980111123 | 0.047691044 |
| KISS1R | ENST00000420465.1 | ENSG00000167355.3 | 0.943910736 | 2.155181154 | 0.031147661 |
| KISS1R | ENST00000420572.2 | ENSG00000233358.2 | 0.845207205 | 1.906968628 | 0.05652465 |
| KISS1R | ENST00000420981.2 | ENSG00000230438.5 | 0.885373279 | 1.978084793 | 0.047919142 |
| KISS1R | ENST00000421020.1 | ENSG00000231407.1 | 0.89069753 | 2.014779163 | 0.043927793 |
| KISS1R | ENST00000422038.1 | ENSG00000227935.1 | 0.803237165 | 1.802588369 | 0.071452885 |
| KISS1R | ENST00000424181.1 | ENSG00000224977.1 | 0.944358518 | 2.134536059 | 0.032798925 |
| KISS1R | ENST00000425371.2 | ENSG00000235872.2 | 0.88213576 | 1.987043501 | 0.046917576 |
| KISS1R | ENST00000426504.1 | ENSG00000234190.1 | -0.835576185 | -1.841132389 | 0.06560216 |
| KISS1R | ENST00000426519.1 | ENSG00000234142.1 | 0.894025429 | 2.006909406 | 0.04475931 |

| | | | | | |
|---|---|---|---|---|---|
| KISS1R | ENST00000429608.1 | ENSG00000237480.1 | 0.859453176 | 1.924463135 | 0.054296557 |
| KISS1R | ENST00000429630.1 | ENSG00000232533.1 | 0.878012232 | 1.974367011 | 0.048340026 |
| KISS1R | ENST00000431290.1 | ENSG00000183822.2 | 0.802227624 | 1.790576093 | 0.073361346 |
| KISS1R | ENST00000433614.1 | ENSG00000228534.1 | -0.814131662 | -1.837109002 | 0.066193806 |
| KISS1R | ENST00000434627.1 | ENSG00000230074.1 | 0.945833475 | 2.114038 | 0.034512022 |
| KISS1R | ENST00000435434.1 | ENSG00000231233.1 | 0.839214995 | 1.853981661 | 0.063741784 |
| KISS1R | ENST00000435733.1 | ENSG00000226377.1 | 0.879232975 | 1.974244094 | 0.048353994 |
| KISS1R | ENST00000435892.1 | ENSG00000233635.2 | 0.862966022 | 1.957366847 | 0.050304353 |
| KISS1R | ENST00000435992.2 | ENSG00000232675.3 | 0.808389612 | 1.796026621 | 0.072490283 |
| KISS1R | ENST00000436582.1 | ENSG00000236525.1 | -0.84114395 | -1.882891483 | 0.059715076 |
| KISS1R | ENST00000437330.1 | ENSG00000229203.1 | 0.937951988 | 2.100362782 | 0.03569694 |
| KISS1R | ENST00000437461.1 | ENSG00000227200.1 | 0.846998735 | 1.875718688 | 0.060693918 |
| KISS1R | ENST00000438222.1 | ENSG00000238034.1 | 0.84021294 | 1.870699189 | 0.061386787 |
| KISS1R | ENST00000442069.1 | ENSG00000225655.1 | -0.957768968 | -2.128588075 | 0.033288356 |
| KISS1R | ENST00000442831.1 | ENSG00000229550.1 | -0.871572873 | -1.941584773 | 0.052187388 |
| KISS1R | ENST00000442850.1 | ENSG00000232600.2 | -0.870066457 | -1.954134697 | 0.05068529 |
| KISS1R | ENST00000443306.1 | ENSG00000233891.3 | 0.905602576 | 2.034784561 | 0.041872555 |
| KISS1R | ENST00000443380.1 | ENSG00000224371.1 | 0.934501058 | 2.109735937 | 0.034881107 |
| KISS1R | ENST00000444665.1 | ENSG00000228852.2 | 0.826604874 | 1.853379287 | 0.063828014 |
| KISS1R | ENST00000447206.1 | ENSG00000230839.1 | 0.893188048 | 2.014481358 | 0.04395902 |
| KISS1R | ENST00000447709.1 | ENSG00000237473.1 | 0.861838032 | 1.950710149 | 0.051091535 |
| KISS1R | ENST00000448365.1 | ENSG00000231114.1 | 0.86260437 | 1.930123724 | 0.05359151 |
| KISS1R | ENST00000450063.1 | ENSG00000231210.2 | -0.83073451 | -1.845780126 | 0.064924137 |
| KISS1R | ENST00000450109.1 | ENSG00000225376.1 | 0.953975752 | 2.154815776 | 0.031176253 |
| KISS1R | ENST00000451656.1 | ENSG00000228417.1 | 0.863210079 | 1.905623569 | 0.056699062 |
| KISS1R | ENST00000452002.1 | ENSG00000236501.1 | -0.830111755 | -1.854652554 | 0.063645858 |
| KISS1R | ENST00000453051.1 | ENSG00000229407.1 | 0.814501455 | 1.818934286 | 0.068921456 |
| KISS1R | ENST00000454530.1 | ENSG00000226649.1 | -0.930355091 | -2.102827483 | 0.035480853 |
| KISS1R | ENST00000457115.1 | ENSG00000227245.1 | 0.959006248 | 2.149773314 | 0.03157315 |
| KISS1R | ENST00000457253.1 | ENSG00000225173.1 | 0.861369684 | 1.916911879 | 0.055249128 |
| KISS1R | ENST00000457848.1 | ENSG00000226412.1 | 0.929544388 | 2.068776747 | 0.038567041 |
| KISS1R | ENST00000458194.1 | ENSG00000226193.1 | 0.914939506 | 2.063110573 | 0.039102124 |
| KISS1R | ENST00000458364.1 | ENSG00000225655.1 | -0.930815264 | -2.095660121 | 0.036112352 |
| KISS1R | ENST00000459985.1 | ENSG00000273066.1 | 0.886271081 | 1.952474025 | 0.050881952 |
| KISS1R | ENST00000466431.2 | ENSG00000254485.1 | 0.817026803 | 1.831757464 | 0.066987563 |
| KISS1R | ENST00000472596.1 | ENSG00000239774.1 | 0.907956999 | 2.062981173 | 0.039114417 |
| KISS1R | ENST00000476099.1 | ENSG00000244158.1 | -0.83510558 | -1.864329486 | 0.062275449 |
| KISS1R | ENST00000476892.1 | ENSG00000241345.1 | 0.880767327 | 1.971466027 | 0.048670595 |
| KISS1R | ENST00000484413.1 | ENSG00000271853.1 | 0.803556728 | 1.789066745 | 0.073604067 |
| KISS1R | ENST00000490013.1 | ENSG00000184115.12 | 0.801487059 | 1.792744402 | 0.073013803 |
| KISS1R | ENST00000493123.1 | ENSG00000242428.1 | 0.946751339 | 2.132005956 | 0.033006357 |
| KISS1R | ENST00000505196.1 | ENSG00000248131.1 | 0.956598927 | 2.135498399 | 0.032720321 |
| KISS1R | ENST00000505556.1 | ENSG00000249409.1 | 0.884147525 | 1.976021264 | 0.048152369 |
| KISS1R | ENST00000506100.1 | ENSG00000249409.1 | 0.877027393 | 1.963727386 | 0.049561715 |
| KISS1R | ENST00000507558.1 | ENSG00000248445.1 | 0.809953843 | 1.810533039 | 0.070213166 |
| KISS1R | ENST00000508083.1 | ENSG00000249343.1 | 0.840655076 | 1.907802005 | 0.056416812 |
| KISS1R | ENST00000508188.1 | ENSG00000250999.1 | 0.965444538 | 2.163736245 | 0.030484598 |
| KISS1R | ENST00000508241.1 | ENSG00000248518.1 | 0.916763156 | 2.050595996 | 0.040306307 |
| KISS1R | ENST00000514877.1 | ENSG00000248685.1 | 0.810556207 | 1.808560324 | 0.070519337 |
| KISS1R | ENST00000515077.1 | ENSG00000251206.1 | 0.897549596 | 2.000026838 | 0.045497366 |
| KISS1R | ENST00000515750.1 | ENSG00000249061.1 | -0.893671644 | -2.003665958 | 0.045105855 |

| | | | | | |
|---|---|---|---|---|---|
| KISS1R | ENST00000517300.1 | ENSG00000254144.2 | 0.976402954 | 2.176502555 | 0.029517703 |
| KISS1R | ENST00000519189.1 | ENSG00000254344.1 | -0.822352372 | -1.842996253 | 0.065329558 |
| KISS1R | ENST00000519451.1 | ENSG00000253363.1 | 0.979588353 | 2.204016831 | 0.02752316 |
| KISS1R | ENST00000519852.1 | ENSG00000253716.1 | 0.930506759 | 2.076403013 | 0.037856693 |
| KISS1R | ENST00000521207.1 | ENSG00000253716.1 | 0.924641433 | 2.065217188 | 0.038902455 |
| KISS1R | ENST00000522600.1 | ENSG00000246582.2 | 0.841450662 | 1.903666564 | 0.056953624 |
| KISS1R | ENST00000524073.1 | ENSG00000253774.1 | 0.936564811 | 2.077165387 | 0.037786297 |
| KISS1R | ENST00000524335.1 | ENSG00000253716.1 | 0.950152333 | 2.12569877 | 0.033528349 |
| KISS1R | ENST00000526154.1 | ENSG00000254511.1 | 0.902531749 | 2.009645398 | 0.044468733 |
| KISS1R | ENST00000526611.1 | ENSG00000246982.2 | 0.890542971 | 2.004698552 | 0.044995283 |
| KISS1R | ENST00000526694.1 | ENSG00000231999.2 | 0.93355499 | 2.065024934 | 0.038920641 |
| KISS1R | ENST00000526935.1 | ENSG00000255372.1 | 0.809387341 | 1.835496176 | 0.066432205 |
| KISS1R | ENST00000528000.1 | ENSG00000254804.1 | 0.846334373 | 1.872420139 | 0.061148501 |
| KISS1R | ENST00000531627.1 | ENSG00000254584.1 | 0.910842063 | 2.037366581 | 0.04161332 |
| KISS1R | ENST00000532688.1 | ENSG00000255441.1 | 0.949548859 | 2.123088604 | 0.033746428 |
| KISS1R | ENST00000536141.1 | ENSG00000256969.1 | 0.938443572 | 2.115344993 | 0.034400554 |
| KISS1R | ENST00000537269.1 | ENSG00000257084.1 | 0.98128273 | 2.215015721 | 0.026758985 |
| KISS1R | ENST00000537921.1 | ENSG00000255966.1 | 0.892087093 | 1.999082723 | 0.045599404 |
| KISS1R | ENST00000543072.1 | ENSG00000256092.2 | -0.842655617 | -1.878135833 | 0.060362586 |
| KISS1R | ENST00000544089.1 | ENSG00000256273.1 | 0.853370252 | 1.88378624 | 0.059593896 |
| KISS1R | ENST00000545177.3 | ENSG00000230438.5 | 0.854439751 | 1.931609654 | 0.053407703 |
| KISS1R | ENST00000545642.1 | ENSG00000256342.1 | 0.870054977 | 1.950654359 | 0.051098176 |
| KISS1R | ENST00000547175.1 | ENSG00000257395.1 | -0.813205991 | -1.844186208 | 0.065156008 |
| KISS1R | ENST00000547750.1 | ENSG00000257886.1 | 0.941113966 | 2.119327377 | 0.03406281 |
| KISS1R | ENST00000548722.2 | ENSG00000257194.2 | -0.814643355 | -1.806822976 | 0.070789885 |
| KISS1R | ENST00000549878.1 | ENSG00000257284.1 | 0.940220162 | 2.102753266 | 0.035487343 |
| KISS1R | ENST00000554049.1 | ENSG00000258763.1 | 0.801801186 | 1.791742365 | 0.073174245 |
| KISS1R | ENST00000554430.1 | ENSG00000258646.1 | -0.846168237 | -1.89914191 | 0.05754582 |
| KISS1R | ENST00000557412.1 | ENSG00000257621.3 | -0.830431264 | -1.870849555 | 0.061365937 |
| KISS1R | ENST00000558515.1 | ENSG00000259182.1 | 0.91067001 | 2.052170916 | 0.040153055 |
| KISS1R | ENST00000558875.1 | ENSG00000259737.2 | 0.807037215 | 1.797252333 | 0.07229557 |
| KISS1R | ENST00000559569.1 | ENSG00000259760.1 | -0.943672553 | -2.098825337 | 0.0358323 |
| KISS1R | ENST00000560522.1 | ENSG00000259661.1 | 0.968838706 | 2.154830043 | 0.031175136 |
| KISS1R | ENST00000561529.1 | ENSG00000260886.1 | 0.874613007 | 1.967172222 | 0.049163359 |
| KISS1R | ENST00000561567.1 | ENSG00000260177.1 | 0.812738081 | 1.800887317 | 0.071720642 |
| KISS1R | ENST00000563044.1 | ENSG00000260978.1 | 0.937004403 | 2.109167046 | 0.034930165 |
| KISS1R | ENST00000565823.1 | ENSG00000260686.1 | -0.88527912 | -1.953737332 | 0.050732289 |
| KISS1R | ENST00000565829.1 | ENSG00000260148.1 | 0.920405212 | 2.039170478 | 0.041433016 |
| KISS1R | ENST00000569742.1 | ENSG00000260787.1 | 0.836943148 | 1.892273804 | 0.058454509 |
| KISS1R | ENST00000570512.1 | ENSG00000262768.1 | 0.835579578 | 1.856370815 | 0.063400722 |
| KISS1R | ENST00000570843.1 | ENSG00000261889.1 | 0.86753994 | 1.940715929 | 0.052292743 |
| KISS1R | ENST00000570929.1 | ENSG00000262223.2 | 0.859069216 | 1.910311764 | 0.056093084 |
| KISS1R | ENST00000571815.1 | ENSG00000262810.1 | 0.932950698 | 2.08336694 | 0.037217789 |
| KISS1R | ENST00000575139.1 | ENSG00000263072.1 | 0.823667244 | 1.843794554 | 0.065213087 |
| KISS1R | ENST00000577064.1 | ENSG00000262823.1 | 0.886719396 | 1.992893282 | 0.046273134 |
| KISS1R | ENST00000577678.1 | ENSG00000265415.1 | 0.843804831 | 1.873360074 | 0.061018679 |
| KISS1R | ENST00000578265.1 | ENSG00000214719.7 | 0.821050282 | 1.842190696 | 0.065447261 |
| KISS1R | ENST00000578757.1 | ENSG00000175061.13 | 0.935289372 | 2.097963041 | 0.03590841 |
| KISS1R | ENST00000578936.1 | ENSG00000265547.1 | 0.886785609 | 1.979736848 | 0.047733106 |
| KISS1R | ENST00000581905.1 | ENSG00000264235.1 | 0.862285837 | 1.932938017 | 0.053243834 |
| KISS1R | ENST00000581940.1 | ENSG00000265484.1 | 0.837220377 | 1.877773522 | 0.060412154 |

| | | | | | |
|---|---|---|---|---|---|
| KISS1R | ENST00000582386.1 | ENSG00000265174.1 | 0.808230695 | 1.807855674 | 0.070628966 |
| KISS1R | ENST00000586010.1 | ENSG00000267606.1 | 0.859832504 | 1.92616888 | 0.05408329 |
| KISS1R | ENST00000589233.1 | ENSG00000231616.4 | 0.91658558 | 2.025049196 | 0.042862313 |
| KISS1R | ENST00000589817.1 | ENSG00000231616.4 | 0.953729365 | 2.132747674 | 0.032945431 |
| KISS1R | ENST00000590368.1 | ENSG00000231616.4 | 0.864826541 | 1.928841978 | 0.053750483 |
| KISS1R | ENST00000590813.1 | ENSG00000231616.4 | 0.866187782 | 1.938791756 | 0.0525267 |
| KISS1R | ENST00000591103.1 | ENSG00000272895.1 | 0.921393104 | 2.03952936 | 0.041397224 |
| KISS1R | ENST00000591137.1 | ENSG00000267405.1 | 0.824319687 | 1.843134212 | 0.065309417 |
| KISS1R | ENST00000593269.1 | ENSG00000236172.2 | 0.805838623 | 1.815315115 | 0.069475497 |
| KISS1R | ENST00000593588.1 | ENSG00000269635.1 | 0.803915465 | 1.798936352 | 0.072028749 |
| KISS1R | ENST00000593632.1 | ENSG00000180279.5 | 0.988700668 | 2.230326032 | 0.025725806 |
| KISS1R | ENST00000593642.1 | ENSG00000267858.1 | 0.851991963 | 1.920783976 | 0.054758947 |
| KISS1R | ENST00000594492.1 | ENSG00000250910.3 | 0.87341756 | 1.964257518 | 0.049500236 |
| KISS1R | ENST00000595955.1 | ENSG00000268401.1 | 0.81544307 | 1.840462653 | 0.065700342 |
| KISS1R | ENST00000596135.1 | ENSG00000269843.1 | 0.897590845 | 1.984387245 | 0.047212687 |
| KISS1R | ENST00000597309.1 | ENSG00000232098.2 | -0.903698148 | -2.009934334 | 0.044438139 |
| KISS1R | ENST00000599259.1 | ENSG00000269352.1 | 0.851101326 | 1.921564601 | 0.054660566 |
| KISS1R | ENST00000600234.1 | ENSG00000268078.1 | 0.855863993 | 1.927463796 | 0.053921856 |
| KISS1R | ENST00000600534.1 | ENSG00000267858.1 | 0.922054677 | 2.080281979 | 0.037499677 |
| KISS1R | ENST00000600889.1 | ENSG00000232675.3 | 0.972237687 | 2.193972072 | 0.028237417 |
| KISS1R | ENST00000601033.1 | ENSG00000268401.1 | 0.811115669 | 1.81461966 | 0.069582379 |
| KISS1R | ENST00000602485.1 | ENSG00000270163.1 | -0.828358882 | -1.869895787 | 0.061498291 |
| KISS1R | ENST00000602532.1 | ENSG00000270091.1 | 0.895018277 | 1.996466454 | 0.045883174 |
| KISS1R | ENST00000602809.1 | ENSG00000270105.1 | -0.817242362 | -1.831497228 | 0.067026361 |
| KISS1R | ENST00000606068.1 | ENSG00000272342.1 | 0.895325562 | 1.990438445 | 0.046542658 |
| KISS1R | ENST00000606841.1 | ENSG00000272411.1 | 0.975804941 | 2.160480864 | 0.030735463 |
| KISS1R | ENST00000606938.1 | ENSG00000272198.1 | -0.858837012 | -1.938634632 | 0.052545843 |
| KISS1R | ENST00000607014.1 | ENSG00000272345.1 | -0.891144863 | -1.988604272 | 0.046744899 |
| KISS1R | ENST00000607201.1 | ENSG00000272024.1 | -0.896310306 | -2.016181654 | 0.043780984 |
| KISS1R | ENST00000607222.1 | ENSG00000272106.1 | 0.81881937 | 1.833893282 | 0.066669837 |
| KISS1R | ENST00000608940.1 | ENSG00000272763.1 | 0.864797607 | 1.925008143 | 0.054228339 |
| KISS1R | ENST00000608952.1 | ENSG00000272689.1 | -0.83787755 | -1.896509101 | 0.057892758 |
| KISS1R | ENST00000609067.1 | ENSG00000272849.1 | 0.804605474 | 1.805018103 | 0.071071849 |
| KISS1R | ENST00000609281.1 | ENSG00000273320.1 | 0.800291704 | 1.765130848 | 0.077541777 |
| KISS1R | ENST00000610145.1 | ENSG00000273175.1 | 0.890173394 | 1.981340159 | 0.04755314 |
| KISS1R | ENST00000610185.1 | ENSG00000273355.1 | -0.941582442 | -2.09341092 | 0.036312488 |
| KISS1R | NR_003604.2 | ZFAS1 | 0.876990748 | 1.973766833 | 0.048408262 |
| KISS1R | NR_003606.2 | ZFAS1 | 0.869530177 | 1.930593846 | 0.0535333 |
| KISS1R | NR_027271.1 | CIRBP-AS1 | 0.826283193 | 1.840416663 | 0.065707089 |
| KISS1R | NR_027334.2 | MZF1-AS1 | 0.910361049 | 2.061582315 | 0.039247519 |
| KISS1R | NR_036480.1 | VPS9D1-AS1 | 0.811961584 | 1.819518011 | 0.068832438 |
| KISS1R | NR_036658.1 | ZFAS1 | 0.867485017 | 1.928969297 | 0.053734674 |
| KISS1R | NR_045637.1 | BOLA3-AS1 | 0.894330388 | 2.006958776 | 0.044754053 |
| KISS1R | NR_046454.1 | LINC00907 | -0.844737975 | -1.885267163 | 0.059393777 |
| KISS1R | NR_046839.1 | AGBL4-IT1 | -0.883361313 | -1.962118883 | 0.049748645 |
| KISS1R | NR_047040.1 | LINC00424 | 0.82569474 | 1.859482369 | 0.062958796 |
| KISS1R | NR_103790.1 | LINC00581 | -0.915804533 | -2.043118689 | 0.04104069 |
| KISS1R | NR_104158.1 | NRG1-IT1 | 0.938744252 | 2.084770901 | 0.037090101 |
| KISS1R | NR_108036.1 | CFAP58-AS1 | 0.831781498 | 1.849370414 | 0.064404345 |
| KISS1R | NR_110053.1 | LOC101927464 | 0.870054977 | 1.96478066 | 0.04943963 |
| KISS1R | NR_110635.1 | LINC00687 | 0.819510947 | 1.823953732 | 0.068159067 |

| | | | | | |
|---|---|---|---|---|---|
| KISS1R | NR_117098.1 | LINC01353 | 0.84720687 | 1.895603671 | 0.058012471 |
| KISS1R | NR_120527.1 | LOC100506675 | 0.862510585 | 1.915223836 | 0.055463964 |
| KISS1R | NR_121188.1 | PGM5P3-AS1 | -0.908195525 | -2.026179407 | 0.042746403 |
| KISS1R | NR_121189.1 | PGM5P3-AS1 | -0.91395287 | -2.036250729 | 0.041725185 |
| KISS1R | NR_121661.1 | ZBTB20-AS5 | -0.922119787 | -2.04228503 | 0.041123266 |
| KISS1R | NR_130144.1 | LOC104968399 | 0.862285837 | 1.943268713 | 0.0519837 |
| KISS1R | NR_135032.1 | LOC105369635 | 0.98128273 | 2.183079819 | 0.029029932 |
| KISS1R | NR_135644.1 | LOC105371506 | -0.904640467 | -2.036725009 | 0.041677607 |
| KISS1R | NR_135820.1 | LOC102723727 | 0.903172826 | 2.005896909 | 0.044867248 |
| KISS1R | NR_138038.1 | LINC00677 | 0.823115573 | 1.850489131 | 0.064243083 |
| KLF2 | ENST00000318291.4 | ENSG00000177406.4 | 0.882694567 | 1.949152813 | 0.051277178 |
| KLF2 | ENST00000399186.2 | ENSG00000214888.2 | 0.956380492 | 2.12459214 | 0.03362066 |
| KLF2 | ENST00000411694.1 | ENSG00000225331.1 | 0.83133037 | 1.885920251 | 0.059305702 |
| KLF2 | ENST00000412759.1 | ENSG00000236933.1 | 0.871394577 | 1.940313513 | 0.0523416 |
| KLF2 | ENST00000413549.1 | ENSG00000231609.1 | 0.814641231 | 1.806088598 | 0.070904502 |
| KLF2 | ENST00000413650.1 | ENSG00000230880.2 | 0.843422516 | 1.864552587 | 0.062244144 |
| KLF2 | ENST00000413887.1 | ENSG00000236948.1 | -0.846243184 | -1.87981249 | 0.060133638 |
| KLF2 | ENST00000414740.2 | ENSG00000229646.2 | 0.832688133 | 1.88237201 | 0.059785523 |
| KLF2 | ENST00000416329.1 | ENSG00000233184.2 | 0.821510611 | 1.835536464 | 0.066426241 |
| KLF2 | ENST00000419662.1 | ENSG00000228265.1 | 0.892282578 | 2.020906023 | 0.043289494 |
| KLF2 | ENST00000419734.1 | ENSG00000234646.1 | -0.832110659 | -1.856766839 | 0.063344333 |
| KLF2 | ENST00000420315.1 | ENSG00000228072.1 | 0.886783396 | 1.957226692 | 0.050320822 |
| KLF2 | ENST00000420465.1 | ENSG00000167355.3 | 0.90535825 | 2.037271928 | 0.041622799 |
| KLF2 | ENST00000420981.2 | ENSG00000230438.5 | 0.872919557 | 1.96664589 | 0.049224049 |
| KLF2 | ENST00000421020.1 | ENSG00000231407.1 | 0.98801721 | 2.218995576 | 0.026487026 |
| KLF2 | ENST00000421207.1 | ENSG00000231768.1 | 0.870781496 | 1.952829935 | 0.05083975 |
| KLF2 | ENST00000423869.1 | ENSG00000227848.1 | 0.938721769 | 2.12123474 | 0.033902053 |
| KLF2 | ENST00000424181.1 | ENSG00000224977.1 | 0.938145341 | 2.097215553 | 0.035974498 |
| KLF2 | ENST00000425624.1 | ENSG00000223779.4 | 0.802136259 | 1.776585626 | 0.075636442 |
| KLF2 | ENST00000426519.1 | ENSG00000234142.1 | 0.963167688 | 2.17392024 | 0.029711126 |
| KLF2 | ENST00000429630.1 | ENSG00000232533.1 | 0.825852921 | 1.842653174 | 0.065379665 |
| KLF2 | ENST00000433614.1 | ENSG00000228534.1 | -0.837746347 | -1.888537856 | 0.058953781 |
| KLF2 | ENST00000434627.1 | ENSG00000230074.1 | 0.895672863 | 2.010078582 | 0.044422872 |
| KLF2 | ENST00000435434.1 | ENSG00000231233.1 | 0.894436049 | 1.990432234 | 0.046543342 |
| KLF2 | ENST00000435733.1 | ENSG00000226377.1 | 0.864013427 | 1.944925219 | 0.051783979 |
| KLF2 | ENST00000435892.1 | ENSG00000233635.2 | 0.882485484 | 1.958843216 | 0.050131151 |
| KLF2 | ENST00000435992.2 | ENSG00000232675.3 | 0.940626194 | 2.128470547 | 0.033298089 |
| KLF2 | ENST00000438107.1 | ENSG00000234449.2 | 0.8757375 | 1.939175503 | 0.052479971 |
| KLF2 | ENST00000438190.1 | ENSG00000227214.2 | 0.869840297 | 1.965089951 | 0.049403827 |
| KLF2 | ENST00000438222.1 | ENSG00000238034.1 | 0.934756855 | 2.090204762 | 0.03659941 |
| KLF2 | ENST00000442069.1 | ENSG00000225655.1 | -0.918473454 | -2.07799232 | 0.037710066 |
| KLF2 | ENST00000443306.1 | ENSG00000233891.3 | 0.847141552 | 1.873981551 | 0.060932968 |
| KLF2 | ENST00000444665.1 | ENSG00000228852.2 | 0.813898155 | 1.827489838 | 0.067626153 |
| KLF2 | ENST00000447206.1 | ENSG00000230839.1 | 0.871070299 | 1.937403405 | 0.052696051 |
| KLF2 | ENST00000447514.1 | ENSG00000236753.1 | 0.804849301 | 1.826152915 | 0.067827231 |
| KLF2 | ENST00000447709.1 | ENSG00000237473.1 | 0.851966357 | 1.904544871 | 0.056839259 |
| KLF2 | ENST00000450063.1 | ENSG00000231210.2 | -0.917478968 | -2.063590703 | 0.03905654 |
| KLF2 | ENST00000450109.1 | ENSG00000225376.1 | 0.800096607 | 1.791526764 | 0.073208804 |
| KLF2 | ENST00000452176.1 | ENSG00000223659.1 | -0.855070092 | -1.922943541 | 0.054487141 |
| KLF2 | ENST00000453051.1 | ENSG00000229407.1 | 0.827374862 | 1.847800608 | 0.064631193 |
| KLF2 | ENST00000454530.1 | ENSG00000226649.1 | -0.86430063 | -1.944796411 | 0.051799486 |

| | | | | | |
|---|---|---|---|---|---|
| KLF2 | ENST00000457115.1 | ENSG00000227245.1 | 0.895382151 | 1.992689578 | 0.046295449 |
| KLF2 | ENST00000457253.1 | ENSG00000225173.1 | 0.930317525 | 2.099762215 | 0.035749764 |
| KLF2 | ENST00000458154.1 | ENSG00000235578.1 | 0.948430427 | 2.110390593 | 0.034824726 |
| KLF2 | ENST00000458194.1 | ENSG00000226193.1 | 0.925604016 | 2.076781246 | 0.037821754 |
| KLF2 | ENST00000458364.1 | ENSG00000225655.1 | -0.965036081 | -2.175984697 | 0.029556405 |
| KLF2 | ENST00000459985.1 | ENSG00000273066.1 | 0.867181764 | 1.95340075 | 0.050772127 |
| KLF2 | ENST00000463297.1 | ENSG00000243486.1 | -0.808065189 | -1.811091606 | 0.070126673 |
| KLF2 | ENST00000472596.1 | ENSG00000239774.1 | 0.887290173 | 2.009808239 | 0.044451488 |
| KLF2 | ENST00000484413.1 | ENSG00000271853.1 | 0.942818012 | 2.0990597 | 0.035811638 |
| KLF2 | ENST00000493123.1 | ENSG00000242428.1 | 0.967379873 | 2.159555212 | 0.030807119 |
| KLF2 | ENST00000494509.1 | ENSG00000240095.1 | 0.881734248 | 2.000109791 | 0.04548841 |
| KLF2 | ENST00000498693.1 | ENSG00000244198.1 | 0.884470278 | 1.977000624 | 0.04804156 |
| KLF2 | ENST00000505196.1 | ENSG00000248131.1 | 0.887645938 | 1.979858668 | 0.047719412 |
| KLF2 | ENST00000505498.1 | ENSG00000250908.1 | 0.81027372 | 1.826505002 | 0.067774229 |
| KLF2 | ENST00000505556.1 | ENSG00000249409.1 | 0.957699352 | 2.171498895 | 0.029893481 |
| KLF2 | ENST00000506100.1 | ENSG00000249409.1 | 0.946051331 | 2.124801684 | 0.033603164 |
| KLF2 | ENST00000506791.1 | ENSG00000251131.1 | 0.888965438 | 1.995682191 | 0.045968527 |
| KLF2 | ENST00000508083.1 | ENSG00000249343.1 | 0.930667509 | 2.091271398 | 0.036503742 |
| KLF2 | ENST00000508188.1 | ENSG00000250999.1 | 0.87825685 | 1.938579978 | 0.052552504 |
| KLF2 | ENST00000509036.1 | ENSG00000251131.1 | 0.932812063 | 2.082849609 | 0.037264934 |
| KLF2 | ENST00000509192.1 | ENSG00000250765.1 | 0.811986229 | 1.815969889 | 0.069374991 |
| KLF2 | ENST00000514877.1 | ENSG00000248685.1 | 0.837043722 | 1.835247654 | 0.066469003 |
| KLF2 | ENST00000517300.1 | ENSG00000254144.2 | 0.949071997 | 2.113832881 | 0.034529544 |
| KLF2 | ENST00000519451.1 | ENSG00000253363.1 | 0.840860778 | 1.874263832 | 0.06089407 |
| KLF2 | ENST00000520603.1 | ENSG00000254001.1 | -0.824300462 | -1.839457737 | 0.065847889 |
| KLF2 | ENST00000521307.1 | ENSG00000253177.1 | 0.848928294 | 1.907497658 | 0.056456174 |
| KLF2 | ENST00000524073.1 | ENSG00000253774.1 | 0.823263754 | 1.842993124 | 0.065330014 |
| KLF2 | ENST00000524818.1 | ENSG00000254473.1 | 0.824218303 | 1.850819686 | 0.064195498 |
| KLF2 | ENST00000526154.1 | ENSG00000254511.1 | 0.834306505 | 1.838311825 | 0.06601647 |
| KLF2 | ENST00000526186.1 | ENSG00000254510.1 | 0.875507769 | 1.94851141 | 0.051353801 |
| KLF2 | ENST00000526611.1 | ENSG00000246982.2 | 0.805862888 | 1.791816574 | 0.073162353 |
| KLF2 | ENST00000526694.1 | ENSG00000231999.2 | 0.846326952 | 1.903317187 | 0.05699917 |
| KLF2 | ENST00000526935.1 | ENSG00000255372.1 | 0.873984412 | 1.941948401 | 0.052143347 |
| KLF2 | ENST00000531627.1 | ENSG00000254584.1 | 0.808523097 | 1.799086846 | 0.072004944 |
| KLF2 | ENST00000532688.1 | ENSG00000255441.1 | 0.857531461 | 1.938948343 | 0.052507629 |
| KLF2 | ENST00000536141.1 | ENSG00000256969.1 | 0.923098824 | 2.072837851 | 0.038187372 |
| KLF2 | ENST00000537269.1 | ENSG00000257084.1 | 0.900916835 | 2.025472671 | 0.042818852 |
| KLF2 | ENST00000543072.1 | ENSG00000256092.2 | -0.842082261 | -1.878839387 | 0.060266427 |
| KLF2 | ENST00000544089.1 | ENSG00000256273.1 | 0.850547554 | 1.888357966 | 0.05897791 |
| KLF2 | ENST00000545177.3 | ENSG00000230438.5 | 0.919162918 | 2.03983344 | 0.041366918 |
| KLF2 | ENST00000545254.1 | ENSG00000256633.1 | 0.822442097 | 1.852908873 | 0.063895421 |
| KLF2 | ENST00000545642.1 | ENSG00000256342.1 | 0.803789285 | 1.808594581 | 0.070514011 |
| KLF2 | ENST00000548722.2 | ENSG00000257194.2 | -0.930935 | -2.066719102 | 0.03876063 |
| KLF2 | ENST00000549140.1 | ENSG00000258332.1 | 0.815901551 | 1.825457755 | 0.067931981 |
| KLF2 | ENST00000549878.1 | ENSG00000257284.1 | 0.857236686 | 1.935624946 | 0.052913653 |
| KLF2 | ENST00000555342.1 | ENSG00000259048.1 | -0.861616492 | -1.906592706 | 0.05657335 |
| KLF2 | ENST00000559569.1 | ENSG00000259760.1 | -0.882222712 | -1.976553586 | 0.048092113 |
| KLF2 | ENST00000560522.1 | ENSG00000259661.1 | 0.871086101 | 1.951146948 | 0.051039568 |
| KLF2 | ENST00000561567.1 | ENSG00000260177.1 | 0.906666287 | 2.023895323 | 0.042980923 |
| KLF2 | ENST00000563044.1 | ENSG00000260978.1 | 0.906102381 | 2.012955319 | 0.04411933 |
| KLF2 | ENST00000564417.1 | ENSG00000260137.1 | -0.867985187 | -1.926694056 | 0.054017769 |

| | | | | | |
|---|---|---|---|---|---|
| KLF2 | ENST00000565823.1 | ENSG00000260686.1 | -0.857941915 | -1.918535225 | 0.05504318 |
| KLF2 | ENST00000565829.1 | ENSG00000260148.1 | 0.870670497 | 1.915548904 | 0.055422539 |
| KLF2 | ENST00000566170.1 | ENSG00000261071.1 | 0.826780886 | 1.850452967 | 0.064248291 |
| KLF2 | ENST00000567395.1 | ENSG00000261090.1 | 0.867206276 | 1.918428391 | 0.055056714 |
| KLF2 | ENST00000568033.1 | ENSG00000261480.1 | 0.885687749 | 1.975493494 | 0.048212172 |
| KLF2 | ENST00000569981.1 | ENSG00000238045.5 | 0.901100166 | 2.031865315 | 0.042167293 |
| KLF2 | ENST00000570493.2 | ENSG00000261898.2 | 0.82239951 | 1.820924125 | 0.068618391 |
| KLF2 | ENST00000570512.1 | ENSG00000262768.1 | 0.884932713 | 1.978113467 | 0.047915908 |
| KLF2 | ENST00000570843.1 | ENSG00000261889.1 | 0.893399141 | 2.013286967 | 0.044084449 |
| KLF2 | ENST00000570929.1 | ENSG00000262223.2 | 0.887627851 | 2.003108484 | 0.045165646 |
| KLF2 | ENST00000574365.1 | ENSG00000262837.1 | 0.830202643 | 1.88301573 | 0.059698236 |
| KLF2 | ENST00000575139.1 | ENSG00000263072.1 | 0.910650913 | 2.050543306 | 0.040311442 |
| KLF2 | ENST00000577678.1 | ENSG00000265415.1 | 0.896461854 | 2.004627539 | 0.04500288 |
| KLF2 | ENST00000578265.1 | ENSG00000214719.7 | 0.926881045 | 2.078173555 | 0.037693377 |
| KLF2 | ENST00000578757.1 | ENSG00000175061.13 | 0.956472149 | 2.121495869 | 0.033880095 |
| KLF2 | ENST00000578936.1 | ENSG00000265547.1 | 0.839272456 | 1.867857869 | 0.061781885 |
| KLF2 | ENST00000581905.1 | ENSG00000264235.1 | 0.849125298 | 1.899738234 | 0.05746748 |
| KLF2 | ENST00000581940.1 | ENSG00000265484.1 | 0.942514778 | 2.107995916 | 0.035031343 |
| KLF2 | ENST00000582386.1 | ENSG00000265174.1 | 0.923242377 | 2.060926505 | 0.039310052 |
| KLF2 | ENST00000584758.1 | ENSG00000265356.1 | 0.837168914 | 1.857101139 | 0.063296766 |
| KLF2 | ENST00000585072.1 | ENSG00000263745.1 | 0.827462814 | 1.818005458 | 0.069063299 |
| KLF2 | ENST00000585559.1 | ENSG00000267117.1 | 0.808465771 | 1.777917163 | 0.075417461 |
| KLF2 | ENST00000586010.1 | ENSG00000267606.1 | 0.80461729 | 1.790724885 | 0.073337454 |
| KLF2 | ENST00000586694.1 | ENSG00000267141.1 | 0.893241034 | 2.010299223 | 0.044399529 |
| KLF2 | ENST00000588380.1 | ENSG00000266990.1 | 0.805155965 | 1.783645853 | 0.07448123 |
| KLF2 | ENST00000589817.1 | ENSG00000231616.4 | 0.814612514 | 1.836480449 | 0.066286631 |
| KLF2 | ENST00000591137.1 | ENSG00000267405.1 | 0.801665166 | 1.800110873 | 0.071843133 |
| KLF2 | ENST00000591174.1 | ENSG00000267289.1 | 0.833924907 | 1.867848475 | 0.061783195 |
| KLF2 | ENST00000593588.1 | ENSG00000269635.1 | 0.860724267 | 1.900513016 | 0.057365829 |
| KLF2 | ENST00000593632.1 | ENSG00000180279.5 | 0.947391153 | 2.114380578 | 0.034482775 |
| KLF2 | ENST00000594590.2 | ENSG00000268199.2 | 0.802933072 | 1.792452007 | 0.07306059 |
| KLF2 | ENST00000596135.1 | ENSG00000269843.1 | 0.880235522 | 1.997480905 | 0.045772967 |
| KLF2 | ENST00000597169.1 | ENSG00000269720.1 | 0.93765249 | 2.07876591 | 0.037638872 |
| KLF2 | ENST00000597309.1 | ENSG00000232098.2 | -0.829298746 | -1.843047183 | 0.065322122 |
| KLF2 | ENST00000599259.1 | ENSG00000269352.1 | 0.922624948 | 2.047517691 | 0.040607282 |
| KLF2 | ENST00000600234.1 | ENSG00000268078.1 | 0.908811849 | 2.032847853 | 0.042067897 |
| KLF2 | ENST00000600534.1 | ENSG00000267858.1 | 0.880757412 | 1.986303967 | 0.046999582 |
| KLF2 | ENST00000600889.1 | ENSG00000232675.3 | 0.928035028 | 2.078661288 | 0.037648493 |
| KLF2 | ENST00000601033.1 | ENSG00000268401.1 | 0.824187915 | 1.82647758 | 0.067778355 |
| KLF2 | ENST00000601735.1 | ENSG00000244513.2 | 0.964434458 | 2.166345368 | 0.030284805 |
| KLF2 | ENST00000602532.1 | ENSG00000270091.1 | 0.849723752 | 1.917780034 | 0.055138908 |
| KLF2 | ENST00000602594.1 | ENSG00000269930.1 | -0.859798708 | -1.924812793 | 0.054252783 |
| KLF2 | ENST00000602809.1 | ENSG00000270105.1 | -0.947835672 | -2.122303558 | 0.033812255 |
| KLF2 | ENST00000602900.1 | ENSG00000270179.1 | -0.800506179 | -1.802027522 | 0.071541076 |
| KLF2 | ENST00000604142.1 | ENSG00000271308.1 | 0.835788637 | 1.853070133 | 0.063872307 |
| KLF2 | ENST00000606277.1 | ENSG00000272145.1 | 0.907017406 | 2.016115249 | 0.043787926 |
| KLF2 | ENST00000606377.1 | ENSG00000272286.1 | -0.924635915 | -2.067492384 | 0.038687781 |
| KLF2 | ENST00000606470.1 | ENSG00000271913.1 | 0.901020253 | 2.013514872 | 0.044060492 |
| KLF2 | ENST00000606841.1 | ENSG00000272411.1 | 0.865612772 | 1.936235508 | 0.052838863 |
| KLF2 | ENST00000606963.1 | ENSG00000272010.1 | -0.851725823 | -1.901940593 | 0.057178921 |
| KLF2 | ENST00000607201.1 | ENSG00000272024.1 | -0.850786009 | -1.909947279 | 0.056140002 |

| | | | | | |
|---|---|---|---|---|---|
| KLF2 | ENST00000607321.1 | ENSG00000272371.1 | -0.812633466 | -1.813968779 | 0.069682533 |
| KLF2 | ENST00000607476.1 | ENSG00000272540.1 | 0.809853049 | 1.794286508 | 0.072767451 |
| KLF2 | ENST00000607580.1 | ENSG00000272545.1 | -0.823507559 | -1.836335528 | 0.066308048 |
| KLF2 | ENST00000609067.1 | ENSG00000272849.1 | 0.848636035 | 1.886081592 | 0.05928396 |
| KLF2 | ENST00000609218.1 | ENSG00000272945.1 | 0.803328391 | 1.798781583 | 0.072053238 |
| KLF2 | ENST00000609725.1 | ENSG00000231898.4 | 0.808370713 | 1.813071901 | 0.069820732 |
| KLF2 | ENST00000609972.1 | ENSG00000230651.3 | 0.802676879 | 1.770701885 | 0.076610289 |
| KLF2 | ENST00000610145.1 | ENSG00000273175.1 | 0.885559729 | 1.986048369 | 0.047027953 |
| KLF2 | NR_003604.2 | ZFAS1 | 0.927709484 | 2.068158638 | 0.038625108 |
| KLF2 | NR_003605.1 | ZFAS1 | 0.922910281 | 2.060589596 | 0.03934221 |
| KLF2 | NR_003606.2 | ZFAS1 | 0.929587101 | 2.073556376 | 0.03812053 |
| KLF2 | NR_026802.1 | FAM74A4 | 0.919076829 | 2.070761287 | 0.03838111 |
| KLF2 | NR_026951.1 | LINC00324 | 0.806497046 | 1.804653174 | 0.071128972 |
| KLF2 | NR_027271.1 | CIRBP-AS1 | 0.912120051 | 2.047081074 | 0.040650125 |
| KLF2 | NR_027334.2 | MZF1-AS1 | 0.886617693 | 1.987018478 | 0.046920349 |
| KLF2 | NR_036480.1 | VPS9D1-AS1 | 0.875885475 | 1.960209165 | 0.049971348 |
| KLF2 | NR_036658.1 | ZFAS1 | 0.933330785 | 2.09020582 | 0.036599315 |
| KLF2 | NR_044996.1 | HCG23 | 0.806897173 | 1.796628472 | 0.072394621 |
| KLF2 | NR_045637.1 | BOLA3-AS1 | 0.877379002 | 1.963175488 | 0.049625787 |
| KLF2 | NR_046742.2 | ZNF630-AS1 | -0.931975913 | -2.113024774 | 0.034598647 |
| KLF2 | NR_046839.1 | AGBL4-IT1 | -0.897823887 | -2.023594108 | 0.043011932 |
| KLF2 | NR_103790.1 | LINC00581 | -0.972618277 | -2.166817735 | 0.030248755 |
| KLF2 | NR_105010.1 | LINC01333 | 0.942277064 | 2.076878286 | 0.037812794 |
| KLF2 | NR_108036.1 | CFAP58-AS1 | 0.878911429 | 1.962298719 | 0.049727716 |
| KLF2 | NR_109886.1 | RALY-AS1 | 0.904342777 | 2.034661606 | 0.041884934 |
| KLF2 | NR_110053.1 | LOC101927464 | 0.803789285 | 1.805597641 | 0.070981211 |
| KLF2 | NR_110998.1 | FAM74A4 | 0.919076829 | 2.047391443 | 0.040619666 |
| KLF2 | NR_121188.1 | PGM5P3-AS1 | -0.878610751 | -1.968222443 | 0.049042449 |
| KLF2 | NR_121189.1 | PGM5P3-AS1 | -0.946422054 | -2.129102297 | 0.033245798 |
| KLF2 | NR_125957.1 | LOC101928626 | -0.855070092 | -1.901391665 | 0.05725073 |
| KLF2 | NR_126522.1 | EXOC3-AS1 | 0.879121745 | 1.966216855 | 0.049273566 |
| KLF2 | NR_130143.1 | LOC104968399 | 0.828451982 | 1.845245294 | 0.065001864 |
| KLF2 | NR_130144.1 | LOC104968399 | 0.849125298 | 1.900679781 | 0.057343968 |
| KLF2 | NR_134252.1 | LOC105379030 | 0.822801623 | 1.840025846 | 0.065764443 |
| KLF2 | NR_134579.1 | LOC105372179 | 0.820466294 | 1.813301802 | 0.069785286 |
| KLF2 | NR_135024.1 | LOC105369747 | 0.815388439 | 1.797539266 | 0.07225005 |
| KLF2 | NR_135032.1 | LOC105369635 | 0.900916835 | 1.993161953 | 0.046243716 |
| KLF2 | NR_138084.1 | HCG24 | 0.930669082 | 2.098525396 | 0.035858759 |
| KLRB1 | ENST00000398777.3 | ENSG00000240152.2 | 0.905535777 | 1.996965199 | 0.045828964 |
| KLRB1 | ENST00000414377.1 | ENSG00000230470.1 | 0.878594054 | 1.972144147 | 0.048593154 |
| KLRB1 | ENST00000416220.1 | ENSG00000236753.1 | 0.824025995 | 1.843334323 | 0.065280213 |
| KLRB1 | ENST00000416641.1 | ENSG00000226956.1 | -0.821674115 | -1.843969034 | 0.065187653 |
| KLRB1 | ENST00000417426.1 | ENSG00000233145.1 | 0.841426914 | 1.879545953 | 0.060169985 |
| KLRB1 | ENST00000417782.1 | ENSG00000228587.1 | 0.911728134 | 2.065747608 | 0.038852317 |
| KLRB1 | ENST00000418741.1 | ENSG00000227332.1 | 0.918540031 | 2.054021772 | 0.039973584 |
| KLRB1 | ENST00000420830.1 | ENSG00000231512.1 | 0.836156721 | 1.870973979 | 0.061348688 |
| KLRB1 | ENST00000421252.2 | ENSG00000250258.1 | -0.883235067 | -1.951516027 | 0.050995692 |
| KLRB1 | ENST00000423667.1 | ENSG00000225970.1 | 0.875467415 | 1.948547809 | 0.05134945 |
| KLRB1 | ENST00000425624.1 | ENSG00000223779.4 | 0.91627214 | 2.077468543 | 0.037758336 |
| KLRB1 | ENST00000426699.1 | ENSG00000229308.1 | 0.879182144 | 1.992835024 | 0.046279515 |
| KLRB1 | ENST00000428769.1 | ENSG00000232738.1 | 0.881739694 | 1.970290891 | 0.048805042 |

| | | | | | |
|---|---|---|---|---|---|
| KLRB1 | ENST00000430728.1 | ENSG00000232316.1 | 0.806445235 | 1.792573683 | 0.073041118 |
| KLRB1 | ENST00000432314.1 | ENSG00000231532.1 | 0.932755958 | 2.105006496 | 0.035290743 |
| KLRB1 | ENST00000433344.1 | ENSG00000234083.1 | -0.863988822 | -1.943444545 | 0.05196247 |
| KLRB1 | ENST00000434790.1 | ENSG00000240040.1 | -0.944675447 | -2.112932718 | 0.034606527 |
| KLRB1 | ENST00000435992.2 | ENSG00000232675.3 | 0.83958682 | 1.870665557 | 0.061391452 |
| KLRB1 | ENST00000436982.2 | ENSG00000235335.2 | -0.850728174 | -1.928636128 | 0.053776051 |
| KLRB1 | ENST00000438222.1 | ENSG00000238034.1 | 0.81389835 | 1.826787003 | 0.067731801 |
| KLRB1 | ENST00000439186.1 | ENSG00000237076.1 | 0.871150686 | 1.897657871 | 0.057741166 |
| KLRB1 | ENST00000440947.1 | ENSG00000225472.1 | 0.881560877 | 1.980270884 | 0.047673099 |
| KLRB1 | ENST00000441160.1 | ENSG00000228437.1 | -0.815786231 | -1.821785469 | 0.068487543 |
| KLRB1 | ENST00000444245.1 | ENSG00000236753.1 | 0.834027427 | 1.864447796 | 0.062258846 |
| KLRB1 | ENST00000447514.1 | ENSG00000236753.1 | 0.861570521 | 1.923469786 | 0.054421077 |
| KLRB1 | ENST00000447538.2 | ENSG00000224189.2 | 0.902973059 | 2.028965474 | 0.042461807 |
| KLRB1 | ENST00000450226.1 | ENSG00000231512.1 | 0.928684287 | 2.066976228 | 0.038736394 |
| KLRB1 | ENST00000450365.1 | ENSG00000224404.1 | 0.821039193 | 1.831616756 | 0.067008539 |
| KLRB1 | ENST00000450848.1 | ENSG00000225539.1 | 0.897032234 | 2.026232743 | 0.04274094 |
| KLRB1 | ENST00000451556.2 | ENSG00000228386.2 | 0.823124141 | 1.837183593 | 0.066182797 |
| KLRB1 | ENST00000451575.2 | ENSG00000224251.2 | 0.837751241 | 1.851027334 | 0.064165621 |
| KLRB1 | ENST00000453889.1 | ENSG00000224750.2 | 0.812836114 | 1.823520762 | 0.068224556 |
| KLRB1 | ENST00000454526.1 | ENSG00000234136.1 | 0.831472501 | 1.819490082 | 0.068836695 |
| KLRB1 | ENST00000455238.1 | ENSG00000231413.1 | 0.849455563 | 1.928868212 | 0.053747225 |
| KLRB1 | ENST00000455699.1 | ENSG00000240996.1 | 0.991264827 | 2.253430569 | 0.024232014 |
| KLRB1 | ENST00000457169.1 | ENSG00000232408.1 | 0.911621794 | 2.014820209 | 0.043923491 |
| KLRB1 | ENST00000458661.2 | ENSG00000236467.3 | 0.97814464 | 2.239616313 | 0.025115843 |
| KLRB1 | ENST00000468444.2 | ENSG00000258525.1 | -0.866748856 | -1.918983433 | 0.054986431 |
| KLRB1 | ENST00000469931.2 | ENSG00000272030.1 | 0.930261361 | 2.108190676 | 0.035014499 |
| KLRB1 | ENST00000481334.1 | ENSG00000242440.1 | 0.867351312 | 1.930647709 | 0.053526634 |
| KLRB1 | ENST00000484413.1 | ENSG00000271853.1 | 0.850737411 | 1.916051712 | 0.055358514 |
| KLRB1 | ENST00000494509.1 | ENSG00000240095.1 | 0.812981806 | 1.817270887 | 0.069175645 |
| KLRB1 | ENST00000496247.1 | ENSG00000241882.1 | 0.813925294 | 1.822831242 | 0.068328954 |
| KLRB1 | ENST00000501405.2 | ENSG00000247402.2 | -0.818662225 | -1.797358443 | 0.072278734 |
| KLRB1 | ENST00000504017.1 | ENSG00000248388.1 | 0.859743816 | 1.925915075 | 0.054114979 |
| KLRB1 | ENST00000504344.1 | ENSG00000251438.1 | 0.944625207 | 2.109330798 | 0.034916038 |
| KLRB1 | ENST00000504891.1 | ENSG00000249388.1 | 0.950715592 | 2.099602099 | 0.035763858 |
| KLRB1 | ENST00000506059.1 | ENSG00000248311.1 | 0.834764349 | 1.874861559 | 0.060811772 |
| KLRB1 | ENST00000507373.1 | ENSG00000250072.1 | 0.946235767 | 2.125379491 | 0.03355496 |
| KLRB1 | ENST00000508825.1 | ENSG00000250775.1 | 0.818974097 | 1.820025976 | 0.068755049 |
| KLRB1 | ENST00000508986.1 | ENSG00000249491.1 | 0.896988102 | 2.026413103 | 0.042722469 |
| KLRB1 | ENST00000513179.1 | ENSG00000251580.1 | 0.926302154 | 2.078859945 | 0.037630225 |
| KLRB1 | ENST00000515205.1 | ENSG00000251580.1 | 0.932060669 | 2.069615449 | 0.03848837 |
| KLRB1 | ENST00000517716.1 | ENSG00000253515.1 | -0.867820277 | -1.975195988 | 0.048245911 |
| KLRB1 | ENST00000518473.1 | ENSG00000253985.1 | 0.968942874 | 2.171037318 | 0.029928352 |
| KLRB1 | ENST00000521307.1 | ENSG00000253177.1 | 0.822559063 | 1.858184084 | 0.063142877 |
| KLRB1 | ENST00000521953.1 | ENSG00000253214.1 | 0.889567747 | 1.969897294 | 0.048850143 |
| KLRB1 | ENST00000522704.1 | ENSG00000254135.1 | 0.860093172 | 1.941060519 | 0.052250937 |
| KLRB1 | ENST00000524818.1 | ENSG00000254473.1 | 0.824362016 | 1.872310899 | 0.061163604 |
| KLRB1 | ENST00000525133.1 | ENSG00000255375.1 | 0.864020366 | 1.925355575 | 0.054184889 |
| KLRB1 | ENST00000526186.1 | ENSG00000254510.1 | 0.820687253 | 1.829907574 | 0.067263761 |
| KLRB1 | ENST00000526935.1 | ENSG00000255372.1 | 0.868999781 | 1.954717145 | 0.050616465 |
| KLRB1 | ENST00000529837.1 | ENSG00000254687.1 | 0.914067394 | 2.032252139 | 0.042128137 |
| KLRB1 | ENST00000531661.1 | ENSG00000254473.1 | 0.885347573 | 1.971201844 | 0.048700793 |

| | | | | | |
|---|---|---|---|---|---|
| KLRB1 | ENST00000532680.1 | ENSG00000255458.1 | 0.92850177 | 2.075510532 | 0.037939244 |
| KLRB1 | ENST00000545593.1 | ENSG00000256972.1 | 0.903330826 | 2.003360054 | 0.045138656 |
| KLRB1 | ENST00000547207.1 | ENSG00000224189.2 | 0.90277064 | 1.993830462 | 0.046170585 |
| KLRB1 | ENST00000547834.1 | ENSG00000258325.1 | 0.810720644 | 1.811200123 | 0.070109879 |
| KLRB1 | ENST00000549140.1 | ENSG00000258332.1 | 0.938079683 | 2.113030722 | 0.034598138 |
| KLRB1 | ENST00000549329.1 | ENSG00000224189.2 | 0.874962951 | 1.960342572 | 0.049955763 |
| KLRB1 | ENST00000549487.1 | ENSG00000257126.1 | 0.900389229 | 2.019254639 | 0.04346076 |
| KLRB1 | ENST00000552156.1 | ENSG00000224189.2 | 0.911617824 | 2.003927002 | 0.045077881 |
| KLRB1 | ENST00000554679.1 | ENSG00000258837.1 | 0.838226368 | 1.883580125 | 0.059621792 |
| KLRB1 | ENST00000555966.1 | ENSG00000258843.1 | 0.913798763 | 2.058625067 | 0.03953017 |
| KLRB1 | ENST00000557903.1 | ENSG00000259182.1 | 0.817875458 | 1.842678311 | 0.065375993 |
| KLRB1 | ENST00000557965.1 | ENSG00000259681.1 | 0.936100383 | 2.071968218 | 0.038268405 |
| KLRB1 | ENST00000558312.1 | ENSG00000259176.1 | 0.806390671 | 1.7840499 | 0.074415557 |
| KLRB1 | ENST00000558896.1 | ENSG00000259176.1 | 0.897268522 | 1.988677602 | 0.0467368 |
| KLRB1 | ENST00000559003.1 | ENSG00000259520.1 | 0.971867935 | 2.196942328 | 0.028024565 |
| KLRB1 | ENST00000560586.1 | ENSG00000259534.1 | 0.931913725 | 2.028431173 | 0.042516261 |
| KLRB1 | ENST00000563841.1 | ENSG00000261029.1 | 0.979241472 | 2.178327947 | 0.02938163 |
| KLRB1 | ENST00000565965.1 | ENSG00000261172.1 | 0.950132587 | 2.172375026 | 0.029827387 |
| KLRB1 | ENST00000567089.1 | ENSG00000261822.1 | 0.894719514 | 2.021287323 | 0.043250031 |
| KLRB1 | ENST00000567395.1 | ENSG00000261090.1 | 0.82938941 | 1.837796284 | 0.066092429 |
| KLRB1 | ENST00000570512.1 | ENSG00000262768.1 | 0.801410467 | 1.794440241 | 0.072742929 |
| KLRB1 | ENST00000572417.1 | ENSG00000263171.1 | 0.818693031 | 1.810439234 | 0.0702277 |
| KLRB1 | ENST00000574365.1 | ENSG00000262837.1 | 0.914834533 | 2.033409897 | 0.042011128 |
| KLRB1 | ENST00000578265.1 | ENSG00000214719.7 | 0.840990871 | 1.84331834 | 0.065282545 |
| KLRB1 | ENST00000580085.1 | ENSG00000266490.1 | 0.885781295 | 1.974807173 | 0.048290035 |
| KLRB1 | ENST00000581362.1 | ENSG00000235300.3 | 0.903781755 | 2.035646238 | 0.041785891 |
| KLRB1 | ENST00000582348.1 | ENSG00000265148.1 | 0.83232813 | 1.849808091 | 0.064341214 |
| KLRB1 | ENST00000584758.1 | ENSG00000265356.1 | 0.903196237 | 2.033800546 | 0.041971709 |
| KLRB1 | ENST00000586952.1 | ENSG00000226994.3 | 0.802498141 | 1.817004113 | 0.069216484 |
| KLRB1 | ENST00000588799.1 | ENSG00000267275.1 | 0.962696364 | 2.150369076 | 0.031526032 |
| KLRB1 | ENST00000588945.1 | ENSG00000267275.1 | 0.968041008 | 2.176052497 | 0.029551335 |
| KLRB1 | ENST00000589777.1 | ENSG00000261040.2 | 0.975984118 | 2.162203651 | 0.030602482 |
| KLRB1 | ENST00000593861.1 | ENSG00000231898.4 | 0.95321167 | 2.130084958 | 0.0331646 |
| KLRB1 | ENST00000594762.1 | ENSG00000231898.4 | 0.824845593 | 1.807807632 | 0.070636445 |
| KLRB1 | ENST00000595892.1 | ENSG00000269640.1 | 0.838110359 | 1.846570718 | 0.064809381 |
| KLRB1 | ENST00000597550.1 | ENSG00000269051.1 | 0.929875163 | 2.042530152 | 0.041098972 |
| KLRB1 | ENST00000599143.1 | ENSG00000269349.1 | 0.944925194 | 2.12415441 | 0.033657234 |
| KLRB1 | ENST00000600489.1 | ENSG00000231898.4 | 0.93392703 | 2.118657573 | 0.034119416 |
| KLRB1 | ENST00000601692.1 | ENSG00000267874.1 | -0.810501564 | -1.826631161 | 0.067755245 |
| KLRB1 | ENST00000602592.1 | ENSG00000270049.1 | 0.885345354 | 1.972042901 | 0.048604709 |
| KLRB1 | ENST00000602954.1 | ENSG00000269906.1 | 0.877462444 | 1.96824049 | 0.049040374 |
| KLRB1 | ENST00000603474.1 | ENSG00000258929.2 | 0.924919913 | 2.038320062 | 0.041517935 |
| KLRB1 | ENST00000604464.1 | ENSG00000270462.1 | 0.859869414 | 1.910256498 | 0.056100196 |
| KLRB1 | ENST00000606457.1 | ENSG00000271830.1 | 0.965470383 | 2.125832397 | 0.033517217 |
| KLRB1 | ENST00000607284.1 | ENSG00000272389.1 | 0.865453439 | 1.930856402 | 0.053500813 |
| KLRB1 | ENST00000607769.1 | ENSG00000272438.1 | -0.901187257 | -1.998267055 | 0.045687715 |
| KLRB1 | ENST00000607876.1 | ENSG00000272848.1 | 0.911240776 | 2.048052549 | 0.040554851 |
| KLRB1 | ENST00000608085.1 | ENSG00000231898.4 | 0.966247139 | 2.170847569 | 0.029942697 |
| KLRB1 | ENST00000608476.1 | ENSG00000232675.3 | 0.902791781 | 2.000257083 | 0.045472511 |
| KLRB1 | ENST00000608509.1 | ENSG00000273245.1 | 0.84308862 | 1.864823313 | 0.062206174 |
| KLRB1 | ENST00000609270.1 | ENSG00000273073.1 | 0.823562395 | 1.832277751 | 0.06691005 |

| | | | | | |
|---|---|---|---|---|---|
| KLRB1 | ENST00000609610.1 | ENSG00000232675.3 | 0.863029648 | 1.958809774 | 0.050135069 |
| KLRB1 | ENST00000609701.1 | ENSG00000273284.1 | 0.913383419 | 2.037531681 | 0.04159679 |
| KLRB1 | ENST00000609725.1 | ENSG00000231898.4 | 0.956784499 | 2.15301869 | 0.03131721 |
| KLRB1 | ENST00000609972.1 | ENSG00000230651.3 | 0.887522272 | 1.982373588 | 0.047437443 |
| KLRB1 | ENST00000610161.1 | ENSG00000273059.1 | 0.946777251 | 2.090052978 | 0.036613041 |
| KLRB1 | NR_024410.1 | LINC00710 | 0.94151127 | 2.072493441 | 0.038219447 |
| KLRB1 | NR_033371.1 | CDRT7 | 0.942543944 | 2.102367583 | 0.035521089 |
| KLRB1 | NR_044996.1 | HCG23 | 0.856832821 | 1.917427235 | 0.055183677 |
| KLRB1 | NR_104998.1 | LOC102467225 | 0.827670407 | 1.840483461 | 0.06569729 |
| KLRB1 | NR_110480.1 | LOC101927079 | 0.898529091 | 2.005040415 | 0.044958727 |
| KLRB1 | NR_110481.1 | LOC101927079 | 0.806390671 | 1.802292359 | 0.07149942 |
| KLRB1 | NR_120335.1 | LOC101928414 | 0.929357327 | 2.106458661 | 0.03516453 |
| KLRB1 | NR_120655.1 | KCNMA1-AS1 | 0.986718997 | 2.212275104 | 0.026947662 |
| KLRB1 | NR_126166.1 | FAM74A7 | 0.930489392 | 2.05519553 | 0.039860123 |
| KLRB1 | NR_134265.1 | LINC02103 | 0.877345072 | 1.965532313 | 0.049352659 |
| KLRB1 | NR_134597.1 | LOC105378068 | 0.949038909 | 2.125005819 | 0.033586127 |
| KLRB1 | NR_134664.1 | LOC105374366 | 0.80337541 | 1.805768764 | 0.070954466 |
| KLRB1 | NR_135108.1 | LOC105369509 | 0.880043455 | 1.958600549 | 0.050159585 |
| KLRB1 | NR_135258.1 | LOC105370489 | 0.904316216 | 2.003702574 | 0.04510193 |
| KLRB1 | NR_135840.1 | LOC105376114 | 0.803070275 | 1.803967943 | 0.071236333 |
| KLRB1 | NR_138084.1 | HCG24 | 0.83547686 | 1.855039459 | 0.063590592 |
| KRTAP10-2 | ENST00000295549.4 | ENSG00000163364.5 | 0.866322392 | 1.926685343 | 0.054018856 |
| KRTAP10-2 | ENST00000411824.1 | ENSG00000232803.1 | -0.845338934 | -1.899126796 | 0.057547807 |
| KRTAP10-2 | ENST00000413645.1 | ENSG00000228798.1 | 0.88510633 | 1.941962529 | 0.052141637 |
| KRTAP10-2 | ENST00000416657.1 | ENSG00000235858.1 | 0.916943129 | 2.05933498 | 0.03946216 |
| KRTAP10-2 | ENST00000418972.1 | ENSG00000225044.1 | -0.888229438 | -2.009137787 | 0.044522523 |
| KRTAP10-2 | ENST00000419207.2 | ENSG00000231248.2 | -0.954849606 | -2.182831051 | 0.029048254 |
| KRTAP10-2 | ENST00000419863.1 | ENSG00000238282.1 | -0.801585632 | -1.796091941 | 0.072479896 |
| KRTAP10-2 | ENST00000420498.1 | ENSG00000224985.1 | 0.893979101 | 1.991682665 | 0.046405887 |
| KRTAP10-2 | ENST00000422038.1 | ENSG00000227935.1 | 0.961357418 | 2.144904116 | 0.031960514 |
| KRTAP10-2 | ENST00000425371.2 | ENSG00000235872.2 | 0.9378626 | 2.107149989 | 0.03510458 |
| KRTAP10-2 | ENST00000426653.1 | ENSG00000235704.1 | 0.905989732 | 2.025060177 | 0.042861185 |
| KRTAP10-2 | ENST00000427691.1 | ENSG00000228340.1 | -0.842456647 | -1.894309877 | 0.058183891 |
| KRTAP10-2 | ENST00000428160.1 | ENSG00000236897.1 | 0.873858103 | 1.95305135 | 0.050813511 |
| KRTAP10-2 | ENST00000428440.1 | ENSG00000232827.2 | -0.873417117 | -1.936804337 | 0.052769266 |
| KRTAP10-2 | ENST00000428853.2 | ENSG00000229206.2 | 0.82725178 | 1.848799422 | 0.064486781 |
| KRTAP10-2 | ENST00000432957.1 | ENSG00000231534.1 | -0.833009772 | -1.86448532 | 0.062253581 |
| KRTAP10-2 | ENST00000435984.1 | ENSG00000204792.2 | 0.803759873 | 1.795326661 | 0.07260167 |
| KRTAP10-2 | ENST00000437330.1 | ENSG00000229203.1 | 0.834479898 | 1.881097684 | 0.059958632 |
| KRTAP10-2 | ENST00000442831.1 | ENSG00000229550.1 | -0.875488049 | -1.948506777 | 0.051354355 |
| KRTAP10-2 | ENST00000449749.1 | ENSG00000230834.1 | 0.822050042 | 1.844174004 | 0.065157786 |
| KRTAP10-2 | ENST00000454928.1 | ENSG00000186148.7 | 0.942824194 | 2.098944352 | 0.035821806 |
| KRTAP10-2 | ENST00000460993.1 | ENSG00000241231.1 | 0.857477664 | 1.894621797 | 0.058142525 |
| KRTAP10-2 | ENST00000476099.1 | ENSG00000244158.1 | -0.842836635 | -1.882839611 | 0.059722107 |
| KRTAP10-2 | ENST00000476892.1 | ENSG00000241345.1 | 0.867085623 | 1.953242847 | 0.050790826 |
| KRTAP10-2 | ENST00000480904.2 | ENSG00000206573.4 | -0.811708555 | -1.7913977 | 0.073229498 |
| KRTAP10-2 | ENST00000501133.2 | ENSG00000246560.2 | 0.892148145 | 1.993308696 | 0.046227655 |
| KRTAP10-2 | ENST00000502300.1 | ENSG00000249451.1 | 0.906415544 | 2.001925069 | 0.045292791 |
| KRTAP10-2 | ENST00000502684.1 | ENSG00000251670.1 | -0.856604959 | -1.919037463 | 0.054979593 |
| KRTAP10-2 | ENST00000507997.1 | ENSG00000250551.1 | -0.837918872 | -1.847806665 | 0.064630316 |
| KRTAP10-2 | ENST00000508241.1 | ENSG00000248518.1 | 0.857233098 | 1.911249513 | 0.055972524 |

| | | | | | |
|---|---|---|---|---|---|
| KRTAP10-2 | ENST00000515750.1 | ENSG00000249061.1 | -0.879175041 | -1.97624415 | 0.048127132 |
| KRTAP10-2 | ENST00000519189.1 | ENSG00000254344.1 | -0.838184452 | -1.891587886 | 0.058545911 |
| KRTAP10-2 | ENST00000521411.2 | ENSG00000253496.2 | 0.810754501 | 1.814670222 | 0.069574604 |
| KRTAP10-2 | ENST00000522524.1 | ENSG00000253342.1 | -0.903822186 | -2.030973519 | 0.042257681 |
| KRTAP10-2 | ENST00000524073.1 | ENSG00000253774.1 | 0.824261259 | 1.859215592 | 0.062996586 |
| KRTAP10-2 | ENST00000524309.1 | ENSG00000240915.2 | -0.81308898 | -1.804788341 | 0.07110781 |
| KRTAP10-2 | ENST00000526694.1 | ENSG00000231999.2 | 0.822124886 | 1.822108604 | 0.068438509 |
| KRTAP10-2 | ENST00000531627.1 | ENSG00000254584.1 | 0.864510684 | 1.925188613 | 0.054205766 |
| KRTAP10-2 | ENST00000535746.1 | ENSG00000256101.1 | 0.884270137 | 1.974286516 | 0.048349173 |
| KRTAP10-2 | ENST00000536412.1 | ENSG00000256072.1 | -0.862487302 | -1.916408181 | 0.05531316 |
| KRTAP10-2 | ENST00000537921.1 | ENSG00000255966.1 | 0.865984653 | 1.940551301 | 0.052312726 |
| KRTAP10-2 | ENST00000545642.1 | ENSG00000256342.1 | 0.809706435 | 1.824475044 | 0.068080286 |
| KRTAP10-2 | ENST00000546789.1 | ENSG00000257740.1 | 0.805923394 | 1.800773535 | 0.071738582 |
| KRTAP10-2 | ENST00000547750.1 | ENSG00000257886.1 | 0.882211314 | 1.973253124 | 0.048466731 |
| KRTAP10-2 | ENST00000548199.1 | ENSG00000257614.1 | 0.874260051 | 1.957473339 | 0.050291843 |
| KRTAP10-2 | ENST00000552558.1 | ENSG00000257947.1 | 0.877922288 | 1.974363258 | 0.048340453 |
| KRTAP10-2 | ENST00000554430.1 | ENSG00000258646.1 | -0.85638273 | -1.931084618 | 0.053472589 |
| KRTAP10-2 | ENST00000557232.1 | ENSG00000259054.1 | 0.915639703 | 2.060055898 | 0.039393197 |
| KRTAP10-2 | ENST00000558434.1 | ENSG00000259572.1 | -0.898268959 | -2.002123235 | 0.045271479 |
| KRTAP10-2 | ENST00000560522.1 | ENSG00000259661.1 | 0.821817862 | 1.843657052 | 0.065233136 |
| KRTAP10-2 | ENST00000561215.1 | ENSG00000259611.1 | 0.922505321 | 2.056829216 | 0.039702657 |
| KRTAP10-2 | ENST00000562582.1 | ENSG00000259779.1 | 0.918434477 | 2.060423193 | 0.039358102 |
| KRTAP10-2 | ENST00000563342.1 | ENSG00000259914.1 | 0.821671794 | 1.808101074 | 0.070590771 |
| KRTAP10-2 | ENST00000563570.1 | ENSG00000259961.1 | -0.904811611 | -2.02963202 | 0.042393958 |
| KRTAP10-2 | ENST00000577360.1 | ENSG00000264273.1 | 0.807344585 | 1.811760288 | 0.070023244 |
| KRTAP10-2 | ENST00000578334.1 | ENSG00000265148.1 | 0.881949381 | 1.986249812 | 0.047005592 |
| KRTAP10-2 | ENST00000580975.1 | ENSG00000266237.1 | 0.819317744 | 1.815893271 | 0.069386746 |
| KRTAP10-2 | ENST00000586297.1 | ENSG00000267633.1 | 0.848127882 | 1.892247461 | 0.058458017 |
| KRTAP10-2 | ENST00000586399.1 | ENSG00000228430.4 | 0.885951015 | 1.940841612 | 0.052277492 |
| KRTAP10-2 | ENST00000589233.1 | ENSG00000231616.4 | 0.872399103 | 1.936858972 | 0.052762585 |
| KRTAP10-2 | ENST00000589817.1 | ENSG00000231616.4 | 0.80175144 | 1.788741237 | 0.073656499 |
| KRTAP10-2 | ENST00000590364.1 | ENSG00000267613.1 | -0.920193974 | -2.038090473 | 0.041540886 |
| KRTAP10-2 | ENST00000590368.1 | ENSG00000231616.4 | 0.869653017 | 1.96356949 | 0.049580038 |
| KRTAP10-2 | ENST00000590813.1 | ENSG00000231616.4 | 0.833827622 | 1.874215076 | 0.060900787 |
| KRTAP10-2 | ENST00000590995.1 | ENSG00000267198.1 | 0.963099902 | 2.153439573 | 0.031284148 |
| KRTAP10-2 | ENST00000591103.1 | ENSG00000272895.1 | 0.875285123 | 1.961839575 | 0.049781165 |
| KRTAP10-2 | ENST00000591217.1 | ENSG00000231616.4 | 0.916290894 | 2.062473678 | 0.039162661 |
| KRTAP10-2 | ENST00000592622.1 | ENSG00000267546.2 | -0.864968026 | -1.934109405 | 0.053099678 |
| KRTAP10-2 | ENST00000593599.1 | ENSG00000231898.4 | 0.834446084 | 1.8610336 | 0.062739432 |
| KRTAP10-2 | ENST00000593824.1 | ENSG00000268184.1 | 0.943519607 | 2.133825592 | 0.03285706 |
| KRTAP10-2 | ENST00000596497.1 | ENSG00000268530.1 | 0.959031336 | 2.153929723 | 0.031245684 |
| KRTAP10-2 | ENST00000600512.1 | ENSG00000269752.1 | 0.877425917 | 1.966200552 | 0.049275449 |
| KRTAP10-2 | ENST00000602485.1 | ENSG00000270163.1 | -0.863910146 | -1.942284099 | 0.052102717 |
| KRTAP10-2 | ENST00000602773.1 | ENSG00000270160.1 | -0.841873157 | -1.873514009 | 0.06099744 |
| KRTAP10-2 | ENST00000606841.1 | ENSG00000272411.1 | 0.838735578 | 1.888072107 | 0.059016271 |
| KRTAP10-2 | ENST00000606899.1 | ENSG00000272426.1 | 0.873095712 | 1.992479314 | 0.046318493 |
| KRTAP10-2 | ENST00000606938.1 | ENSG00000272198.1 | -0.835574387 | -1.868044346 | 0.06175589 |
| KRTAP10-2 | ENST00000607044.1 | ENSG00000272247.1 | 0.939871517 | 2.106807666 | 0.035134255 |
| KRTAP10-2 | ENST00000609953.1 | ENSG00000272825.1 | 0.805562965 | 1.802272624 | 0.071502524 |
| KRTAP10-2 | ENST00000610044.1 | ENSG00000273160.1 | -0.905589915 | -2.030021546 | 0.042354349 |
| KRTAP10-2 | ENST00000610185.1 | ENSG00000273355.1 | -0.911102024 | -2.04007238 | 0.041343117 |

| | | | | | |
|---|---|---|---|---|---|
| KRTAP10-2 | NR_026822.1 | FAM138C | -0.802015727 | -1.804069909 | 0.071220348 |
| KRTAP10-2 | NR_028325.1 | LOC100132062 | 0.944946409 | 2.13104181 | 0.033085698 |
| KRTAP10-2 | NR_040001.2 | LINC01116 | 0.809432498 | 1.820702584 | 0.068652079 |
| KRTAP10-2 | NR_046578.1 | CACNA1C-AS4 | 0.937142091 | 2.107003332 | 0.035117291 |
| KRTAP10-2 | NR_046766.1 | ATP2B2-IT2 | 0.835283442 | 1.892540319 | 0.058419027 |
| KRTAP10-2 | NR_047040.1 | LINC00424 | 0.807645941 | 1.806737367 | 0.070803239 |
| KRTAP10-2 | NR_049776.1 | GPC5-AS1 | 0.90594237 | 2.016681709 | 0.04372874 |
| KRTAP10-2 | NR_103857.1 | SP2-AS1 | -0.899013803 | -1.997255243 | 0.045797463 |
| KRTAP10-2 | NR_109975.1 | ARNTL2-AS1 | -0.843680484 | -1.882872618 | 0.059717633 |
| KRTAP10-2 | NR_110053.1 | LOC101927464 | 0.809706435 | 1.837312397 | 0.066163791 |
| KRTAP10-2 | NR_120527.1 | LOC100506675 | 0.893284987 | 1.995726294 | 0.045963723 |
| KRTAP10-2 | NR_121661.1 | ZBTB20-AS5 | -0.881975546 | -1.998326566 | 0.045681267 |
| KRTAP10-2 | NR_125407.1 | LOC102724604 | 0.817715732 | 1.827490246 | 0.067626091 |
| KRTAP10-2 | NR_131186.1 | LOC105377348 | 0.91131936 | 2.037666075 | 0.041583339 |
| KRTAP10-2 | NR_133642.1 | DIRC3-AS1 | 0.852827039 | 1.900546705 | 0.057361412 |
| KRTAP10-2 | NR_135274.1 | LOC105370619 | 0.800539994 | 1.787218101 | 0.073902245 |
| KRTAP10-2 | NR_135820.1 | LOC102723727 | 0.877258182 | 1.985218038 | 0.047120218 |
| KRTAP9-6 | ENST00000412085.1 | ENSG00000233825.1 | 0.867451548 | 1.928409792 | 0.053804175 |
| KRTAP9-6 | ENST00000412772.1 | ENSG00000231507.1 | 0.927209693 | 2.07622479 | 0.037873166 |
| KRTAP9-6 | ENST00000414740.2 | ENSG00000229646.2 | 0.921581066 | 2.035040503 | 0.041846798 |
| KRTAP9-6 | ENST00000420572.2 | ENSG00000233358.2 | 0.81253305 | 1.823538727 | 0.068221837 |
| KRTAP9-6 | ENST00000420981.2 | ENSG00000230438.5 | 0.831398257 | 1.86120436 | 0.062715323 |
| KRTAP9-6 | ENST00000421866.1 | ENSG00000233875.1 | -0.823827237 | -1.854075055 | 0.063728423 |
| KRTAP9-6 | ENST00000427691.1 | ENSG00000228340.1 | -0.832769495 | -1.855166139 | 0.063572505 |
| KRTAP9-6 | ENST00000429608.1 | ENSG00000237480.1 | 0.98924246 | 2.200830786 | 0.027748005 |
| KRTAP9-6 | ENST00000431290.1 | ENSG00000183822.2 | 0.818772372 | 1.835939191 | 0.066366651 |
| KRTAP9-6 | ENST00000433036.1 | ENSG00000228989.1 | 0.857551924 | 1.897188888 | 0.057803013 |
| KRTAP9-6 | ENST00000433876.2 | ENSG00000228423.2 | 0.813340113 | 1.782889638 | 0.07460427 |
| KRTAP9-6 | ENST00000434627.1 | ENSG00000230074.1 | 0.914147602 | 2.014725937 | 0.043933373 |
| KRTAP9-6 | ENST00000437330.1 | ENSG00000229203.1 | 0.911790486 | 2.025676684 | 0.042797927 |
| KRTAP9-6 | ENST00000437461.1 | ENSG00000227200.1 | 0.829934466 | 1.863315211 | 0.062417931 |
| KRTAP9-6 | ENST00000442069.1 | ENSG00000225655.1 | -0.811141829 | -1.825848132 | 0.067873141 |
| KRTAP9-6 | ENST00000442850.1 | ENSG00000232600.2 | -0.898676715 | -2.016505226 | 0.043747172 |
| KRTAP9-6 | ENST00000443380.1 | ENSG00000224371.1 | 0.944874206 | 2.084057408 | 0.037154946 |
| KRTAP9-6 | ENST00000447206.1 | ENSG00000230839.1 | 0.819633422 | 1.849986302 | 0.064315524 |
| KRTAP9-6 | ENST00000448365.1 | ENSG00000231114.1 | 0.81451564 | 1.798902731 | 0.072034069 |
| KRTAP9-6 | ENST00000450109.1 | ENSG00000225376.1 | 0.905732602 | 2.002674045 | 0.045212286 |
| KRTAP9-6 | ENST00000450531.1 | ENSG00000229536.1 | 0.805693986 | 1.805659657 | 0.070971518 |
| KRTAP9-6 | ENST00000453051.1 | ENSG00000229407.1 | 0.860613993 | 1.917503711 | 0.05517397 |
| KRTAP9-6 | ENST00000454530.1 | ENSG00000226649.1 | -0.91742397 | -2.038973143 | 0.041452708 |
| KRTAP9-6 | ENST00000455010.1 | ENSG00000233079.1 | 0.842023044 | 1.837643649 | 0.066114932 |
| KRTAP9-6 | ENST00000457115.1 | ENSG00000227245.1 | 0.866053327 | 1.955782663 | 0.050490762 |
| KRTAP9-6 | ENST00000457848.1 | ENSG00000226412.1 | 0.808229477 | 1.796622757 | 0.072395529 |
| KRTAP9-6 | ENST00000458194.1 | ENSG00000226193.1 | 0.871466825 | 1.941846047 | 0.052155741 |
| KRTAP9-6 | ENST00000459985.1 | ENSG00000273066.1 | 0.831481525 | 1.841301768 | 0.065577349 |
| KRTAP9-6 | ENST00000466431.2 | ENSG00000254485.1 | 0.895494183 | 2.014060709 | 0.04400316 |
| KRTAP9-6 | ENST00000490013.1 | ENSG00000184115.12 | 0.899361583 | 1.992329827 | 0.046334881 |
| KRTAP9-6 | ENST00000498358.1 | ENSG00000184115.12 | 0.938531722 | 2.118324819 | 0.034147568 |
| KRTAP9-6 | ENST00000502300.1 | ENSG00000249451.1 | 0.832313336 | 1.848246591 | 0.064566678 |
| KRTAP9-6 | ENST00000503034.1 | ENSG00000248936.1 | 0.82600394 | 1.848706837 | 0.064500156 |
| KRTAP9-6 | ENST00000505196.1 | ENSG00000248131.1 | 0.879220624 | 1.940164722 | 0.052359674 |

| | | | | | |
|---|---|---|---|---|---|
| KRTAP9-6 | ENST00000508188.1 | ENSG00000250999.1 | 0.934128262 | 2.073390214 | 0.038135979 |
| KRTAP9-6 | ENST00000508199.1 | ENSG00000247810.2 | 0.873655031 | 1.94029861 | 0.05234341 |
| KRTAP9-6 | ENST00000508241.1 | ENSG00000248518.1 | 0.88987186 | 1.987004357 | 0.046921914 |
| KRTAP9-6 | ENST00000515077.1 | ENSG00000251206.1 | 0.820783431 | 1.832651408 | 0.066854428 |
| KRTAP9-6 | ENST00000517300.1 | ENSG00000254144.2 | 0.889687918 | 1.987342126 | 0.046884496 |
| KRTAP9-6 | ENST00000519451.1 | ENSG00000253363.1 | 0.943080557 | 2.107890053 | 0.035040501 |
| KRTAP9-6 | ENST00000519852.1 | ENSG00000253716.1 | 0.897397999 | 2.003396011 | 0.045134799 |
| KRTAP9-6 | ENST00000521207.1 | ENSG00000253716.1 | 0.969678849 | 2.174778067 | 0.029646751 |
| KRTAP9-6 | ENST00000522600.1 | ENSG00000246582.2 | 0.85260606 | 1.917824831 | 0.055133226 |
| KRTAP9-6 | ENST00000524073.1 | ENSG00000253774.1 | 0.800976078 | 1.76984767 | 0.07675252 |
| KRTAP9-6 | ENST00000524335.1 | ENSG00000253716.1 | 0.907964182 | 2.029081923 | 0.042449947 |
| KRTAP9-6 | ENST00000524942.1 | ENSG00000255553.1 | 0.858411437 | 1.914652539 | 0.05553683 |
| KRTAP9-6 | ENST00000526154.1 | ENSG00000254511.1 | 0.880549051 | 1.975612154 | 0.048198721 |
| KRTAP9-6 | ENST00000526611.1 | ENSG00000246982.2 | 0.811174493 | 1.838099188 | 0.066047791 |
| KRTAP9-6 | ENST00000528000.1 | ENSG00000254804.1 | 0.916928174 | 2.067657936 | 0.0386722 |
| KRTAP9-6 | ENST00000532688.1 | ENSG00000255441.1 | 0.903735422 | 2.012121359 | 0.044207146 |
| KRTAP9-6 | ENST00000535746.1 | ENSG00000256101.1 | 0.843489546 | 1.883662786 | 0.059610603 |
| KRTAP9-6 | ENST00000537269.1 | ENSG00000257084.1 | 0.880516049 | 1.967506333 | 0.049124867 |
| KRTAP9-6 | ENST00000543072.1 | ENSG00000256092.2 | -0.8429472 | -1.884882932 | 0.059445645 |
| KRTAP9-6 | ENST00000543275.1 | ENSG00000256944.1 | 0.841657606 | 1.856927779 | 0.06332143 |
| KRTAP9-6 | ENST00000547750.1 | ENSG00000257886.1 | 0.886574801 | 1.997202814 | 0.045803156 |
| KRTAP9-6 | ENST00000548731.1 | ENSG00000257809.1 | 0.816880351 | 1.796061398 | 0.072484753 |
| KRTAP9-6 | ENST00000549878.1 | ENSG00000257284.1 | 0.833856563 | 1.8543214 | 0.063693192 |
| KRTAP9-6 | ENST00000552525.1 | ENSG00000257286.1 | 0.851202562 | 1.91351159 | 0.055682591 |
| KRTAP9-6 | ENST00000554049.1 | ENSG00000258763.1 | 0.916132044 | 2.01163467 | 0.044258462 |
| KRTAP9-6 | ENST00000554197.1 | ENSG00000197176.3 | 0.832063064 | 1.850069125 | 0.064303587 |
| KRTAP9-6 | ENST00000554430.1 | ENSG00000258646.1 | -0.815492338 | -1.822804892 | 0.068332947 |
| KRTAP9-6 | ENST00000557412.1 | ENSG00000257621.3 | -0.844644028 | -1.892635872 | 0.05840631 |
| KRTAP9-6 | ENST00000558515.1 | ENSG00000259182.1 | 0.950074473 | 2.148385618 | 0.031683134 |
| KRTAP9-6 | ENST00000558875.1 | ENSG00000259737.2 | 0.90364887 | 1.993539128 | 0.046202443 |
| KRTAP9-6 | ENST00000560522.1 | ENSG00000259661.1 | 0.824962882 | 1.837396884 | 0.066151326 |
| KRTAP9-6 | ENST00000561529.1 | ENSG00000260886.1 | 0.888799638 | 2.000077946 | 0.045491848 |
| KRTAP9-6 | ENST00000563806.1 | ENSG00000238045.5 | 0.894268745 | 2.038881548 | 0.041461851 |
| KRTAP9-6 | ENST00000565359.1 | ENSG00000260601.1 | 0.930894086 | 2.085744267 | 0.037001794 |
| KRTAP9-6 | ENST00000565823.1 | ENSG00000260686.1 | -0.824784565 | -1.857208016 | 0.063281565 |
| KRTAP9-6 | ENST00000565829.1 | ENSG00000260148.1 | 0.877744623 | 1.992979838 | 0.046263655 |
| KRTAP9-6 | ENST00000569459.1 | ENSG00000261346.1 | 0.820962813 | 1.850282949 | 0.064272779 |
| KRTAP9-6 | ENST00000569742.1 | ENSG00000260787.1 | 0.839829634 | 1.872524116 | 0.061134129 |
| KRTAP9-6 | ENST00000570843.1 | ENSG00000261889.1 | 0.806798329 | 1.810751906 | 0.070179264 |
| KRTAP9-6 | ENST00000571815.1 | ENSG00000262810.1 | 0.846314794 | 1.875772323 | 0.06068655 |
| KRTAP9-6 | ENST00000574460.1 | ENSG00000263051.1 | 0.833425049 | 1.862412635 | 0.06254495 |
| KRTAP9-6 | ENST00000577064.1 | ENSG00000262823.1 | 0.85130316 | 1.895290382 | 0.058053942 |
| KRTAP9-6 | ENST00000578936.1 | ENSG00000265547.1 | 0.928313926 | 2.120896435 | 0.033930519 |
| KRTAP9-6 | ENST00000583138.1 | ENSG00000263393.1 | 0.898306843 | 2.043636148 | 0.040989505 |
| KRTAP9-6 | ENST00000583916.1 | ENSG00000264196.1 | 0.894784716 | 2.035958905 | 0.041754482 |
| KRTAP9-6 | ENST00000588290.1 | ENSG00000267751.1 | 0.857716256 | 1.935818162 | 0.052889976 |
| KRTAP9-6 | ENST00000588908.1 | ENSG00000267751.1 | 0.892297711 | 1.984812368 | 0.047165351 |
| KRTAP9-6 | ENST00000589233.1 | ENSG00000231616.4 | 0.886843354 | 1.968024005 | 0.049065276 |
| KRTAP9-6 | ENST00000589457.1 | ENSG00000267751.1 | 0.861265291 | 1.899158526 | 0.057543636 |
| KRTAP9-6 | ENST00000589673.1 | ENSG00000267755.1 | 0.847005242 | 1.88354851 | 0.059626072 |
| KRTAP9-6 | ENST00000589817.1 | ENSG00000231616.4 | 0.942764812 | 2.092961563 | 0.036352586 |

| | | | | | |
|---|---|---|---|---|---|
| KRTAP9-6 | ENST00000590292.1 | ENSG00000267751.1 | 0.859618347 | 1.922882007 | 0.05449487 |
| KRTAP9-6 | ENST00000590368.1 | ENSG00000231616.4 | 0.914928101 | 2.048118008 | 0.040548438 |
| KRTAP9-6 | ENST00000590813.1 | ENSG00000231616.4 | 0.880399639 | 1.964522423 | 0.049469539 |
| KRTAP9-6 | ENST00000591103.1 | ENSG00000272895.1 | 0.816288885 | 1.823390406 | 0.068244282 |
| KRTAP9-6 | ENST00000591836.1 | ENSG00000267776.1 | 0.912591018 | 2.033820497 | 0.041969697 |
| KRTAP9-6 | ENST00000592622.1 | ENSG00000267546.2 | -0.806080535 | -1.808915024 | 0.070464206 |
| KRTAP9-6 | ENST00000593269.1 | ENSG00000236172.2 | 0.909416881 | 2.009283197 | 0.044507109 |
| KRTAP9-6 | ENST00000593632.1 | ENSG00000180279.5 | 0.854798008 | 1.913104408 | 0.055734688 |
| KRTAP9-6 | ENST00000593642.1 | ENSG00000267858.1 | 0.934631245 | 2.098374362 | 0.035872088 |
| KRTAP9-6 | ENST00000594492.1 | ENSG00000250910.3 | 0.903902142 | 2.016744738 | 0.043722159 |
| KRTAP9-6 | ENST00000595955.1 | ENSG00000268401.1 | 0.912287133 | 2.026349035 | 0.04272903 |
| KRTAP9-6 | ENST00000600071.1 | ENSG00000269199.1 | 0.917853794 | 2.028213865 | 0.042538425 |
| KRTAP9-6 | ENST00000600534.1 | ENSG00000267858.1 | 0.857302301 | 1.917206756 | 0.05521167 |
| KRTAP9-6 | ENST00000600889.1 | ENSG00000232675.3 | 0.895413924 | 2.008266819 | 0.044614946 |
| KRTAP9-6 | ENST00000601033.1 | ENSG00000268401.1 | 0.853415879 | 1.918577079 | 0.055037879 |
| KRTAP9-6 | ENST00000602532.1 | ENSG00000270091.1 | 0.904355353 | 1.999223738 | 0.045584151 |
| KRTAP9-6 | ENST00000602614.1 | ENSG00000269957.1 | -0.851683796 | -1.913547329 | 0.055678021 |
| KRTAP9-6 | ENST00000606068.1 | ENSG00000272342.1 | 0.948508384 | 2.102099714 | 0.035544542 |
| KRTAP9-6 | ENST00000606841.1 | ENSG00000272411.1 | 0.844505531 | 1.887847644 | 0.059046407 |
| KRTAP9-6 | ENST00000607014.1 | ENSG00000272345.1 | -0.889659982 | -2.010854686 | 0.044340808 |
| KRTAP9-6 | ENST00000608264.1 | ENSG00000273473.1 | 0.859939555 | 1.899836707 | 0.057454552 |
| KRTAP9-6 | ENST00000608940.1 | ENSG00000272763.1 | 0.958158165 | 2.156361611 | 0.03105544 |
| KRTAP9-6 | ENST00000608952.1 | ENSG00000272689.1 | -0.893222514 | -1.992600587 | 0.046305201 |
| KRTAP9-6 | ENST00000609146.1 | ENSG00000272851.1 | -0.929999308 | -2.036571389 | 0.041693012 |
| KRTAP9-6 | ENST00000610185.1 | ENSG00000273355.1 | -0.840967511 | -1.866647822 | 0.061950785 |
| KRTAP9-6 | NR_027334.2 | MZF1-AS1 | 0.831940872 | 1.862424059 | 0.062543341 |
| KRTAP9-6 | NR_040096.1 | LOC643339 | 0.898532079 | 2.017800579 | 0.043612035 |
| KRTAP9-6 | NR_046454.1 | LINC00907 | -0.905398108 | -2.047567156 | 0.04060243 |
| KRTAP9-6 | NR_046571.1 | POTEH-AS1 | 0.828786182 | 1.838660729 | 0.065965103 |
| KRTAP9-6 | NR_103851.1 | TAT-AS1 | 0.802190728 | 1.782986669 | 0.074588473 |
| KRTAP9-6 | NR_103857.1 | SP2-AS1 | -0.868698324 | -1.94482798 | 0.051795685 |
| KRTAP9-6 | NR_104158.1 | NRG1-IT1 | 0.955964552 | 2.131434043 | 0.033053401 |
| KRTAP9-6 | NR_110318.1 | MACROD2-AS1 | 0.872198051 | 1.961583833 | 0.049810956 |
| KRTAP9-6 | NR_110635.1 | LINC00687 | 0.946336269 | 2.122956017 | 0.033757538 |
| KRTAP9-6 | NR_117097.1 | LINC01353 | 0.927209693 | 2.053870653 | 0.039988212 |
| KRTAP9-6 | NR_117098.1 | LINC01353 | 0.967265165 | 2.171887721 | 0.029864133 |
| KRTAP9-6 | NR_121661.1 | ZBTB20-AS5 | -0.90485229 | -2.030166251 | 0.042339643 |
| KRTAP9-6 | NR_130143.1 | LOC104968399 | 0.808590734 | 1.854484004 | 0.063669947 |
| KRTAP9-6 | NR_135032.1 | LOC105369635 | 0.880516049 | 1.961108229 | 0.049866399 |
| KRTAP9-6 | NR_135644.1 | LOC105371506 | -0.933836208 | -2.102142207 | 0.035540821 |
| KRTAP9-6 | NR_135820.1 | LOC102723727 | 0.924642635 | 2.079813224 | 0.037542668 |
| KRTAP9-7 | ENST00000412085.1 | ENSG00000233825.1 | 0.823883485 | 1.845493001 | 0.064965856 |
| KRTAP9-7 | ENST00000412759.1 | ENSG00000236933.1 | 0.82952237 | 1.848613112 | 0.064513698 |
| KRTAP9-7 | ENST00000412772.1 | ENSG00000231507.1 | 0.801525397 | 1.807851758 | 0.070629576 |
| KRTAP9-7 | ENST00000413650.1 | ENSG00000230880.2 | 0.813862867 | 1.823840963 | 0.068176119 |
| KRTAP9-7 | ENST00000414740.2 | ENSG00000229646.2 | 0.886479537 | 2.002940542 | 0.045183671 |
| KRTAP9-7 | ENST00000419734.1 | ENSG00000234646.1 | -0.804420332 | -1.78731244 | 0.073887005 |
| KRTAP9-7 | ENST00000420572.2 | ENSG00000233358.2 | 0.856987929 | 1.919253106 | 0.05495231 |
| KRTAP9-7 | ENST00000421617.1 | ENSG00000237342.1 | 0.837093475 | 1.859299127 | 0.062984751 |
| KRTAP9-7 | ENST00000423925.1 | ENSG00000223536.1 | -0.8313351 | -1.839456703 | 0.065848041 |
| KRTAP9-7 | ENST00000424241.1 | ENSG00000237311.1 | -0.913821301 | -2.058567022 | 0.039535735 |

| | | | | | |
|---|---|---|---|---|---|
| KRTAP9-7 | ENST00000425364.1 | ENSG00000231046.1 | -0.89459889 | -2.010129481 | 0.044417487 |
| KRTAP9-7 | ENST00000426504.1 | ENSG00000234190.1 | -0.842066342 | -1.880985918 | 0.059973834 |
| KRTAP9-7 | ENST00000426519.1 | ENSG00000234142.1 | 0.830830126 | 1.854746654 | 0.063632413 |
| KRTAP9-7 | ENST00000429796.1 | ENSG00000231858.1 | -0.933811666 | -2.078857743 | 0.037630428 |
| KRTAP9-7 | ENST00000430545.1 | ENSG00000237153.1 | 0.85388291 | 1.896863773 | 0.05784592 |
| KRTAP9-7 | ENST00000431290.1 | ENSG00000183822.2 | 0.85770642 | 1.941817077 | 0.052159249 |
| KRTAP9-7 | ENST00000432244.1 | ENSG00000234265.1 | 0.910420976 | 2.023413241 | 0.04303056 |
| KRTAP9-7 | ENST00000432559.2 | ENSG00000228229.2 | 0.853502039 | 1.883897344 | 0.059578863 |
| KRTAP9-7 | ENST00000433876.2 | ENSG00000228423.2 | 0.9630945 | 2.160909321 | 0.030702345 |
| KRTAP9-7 | ENST00000434627.1 | ENSG00000230074.1 | 0.900882784 | 1.999583786 | 0.045545226 |
| KRTAP9-7 | ENST00000435892.1 | ENSG00000233635.2 | 0.942727839 | 2.110638098 | 0.03480343 |
| KRTAP9-7 | ENST00000436582.1 | ENSG00000236525.1 | -0.831835793 | -1.87472924 | 0.060829982 |
| KRTAP9-7 | ENST00000437308.1 | ENSG00000233154.1 | 0.804698705 | 1.796595891 | 0.072399797 |
| KRTAP9-7 | ENST00000437461.1 | ENSG00000227200.1 | 0.826040619 | 1.840671513 | 0.065669711 |
| KRTAP9-7 | ENST00000438107.1 | ENSG00000234449.2 | 0.829934701 | 1.843864952 | 0.065202824 |
| KRTAP9-7 | ENST00000438488.1 | ENSG00000223812.1 | 0.838647613 | 1.845606645 | 0.064949341 |
| KRTAP9-7 | ENST00000441532.1 | ENSG00000234206.1 | -0.887373943 | -2.000937545 | 0.045399121 |
| KRTAP9-7 | ENST00000441875.1 | ENSG00000239203.1 | 0.87451451 | 1.964079731 | 0.049520846 |
| KRTAP9-7 | ENST00000442850.1 | ENSG00000232600.2 | -0.946033082 | -2.138527066 | 0.03247399 |
| KRTAP9-7 | ENST00000443380.1 | ENSG00000224371.1 | 0.900907542 | 2.010343913 | 0.044394802 |
| KRTAP9-7 | ENST00000444665.1 | ENSG00000228852.2 | 0.926340485 | 2.070111218 | 0.038441931 |
| KRTAP9-7 | ENST00000447206.1 | ENSG00000230839.1 | 0.9595033 | 2.12105293 | 0.033917348 |
| KRTAP9-7 | ENST00000448650.1 | ENSG00000223536.1 | -0.813433565 | -1.814055151 | 0.069669235 |
| KRTAP9-7 | ENST00000448942.1 | ENSG00000237499.2 | 0.818693122 | 1.847904389 | 0.064616175 |
| KRTAP9-7 | ENST00000450109.1 | ENSG00000225376.1 | 0.891749184 | 2.001808124 | 0.045305372 |
| KRTAP9-7 | ENST00000451656.1 | ENSG00000228417.1 | 0.816004524 | 1.827095877 | 0.067685355 |
| KRTAP9-7 | ENST00000452002.1 | ENSG00000236501.1 | -0.874964078 | -1.950624823 | 0.051101692 |
| KRTAP9-7 | ENST00000454530.1 | ENSG00000226649.1 | -0.926843244 | -2.047877573 | 0.040571997 |
| KRTAP9-7 | ENST00000456715.1 | ENSG00000224893.1 | 0.962297219 | 2.160654765 | 0.030722018 |
| KRTAP9-7 | ENST00000457848.1 | ENSG00000226412.1 | 0.86874526 | 1.964383157 | 0.049485675 |
| KRTAP9-7 | ENST00000458194.1 | ENSG00000226193.1 | 0.84429425 | 1.884958384 | 0.059435457 |
| KRTAP9-7 | ENST00000458364.1 | ENSG00000225655.1 | -0.801579953 | -1.814608609 | 0.069584078 |
| KRTAP9-7 | ENST00000458443.1 | ENSG00000238232.1 | 0.877646253 | 1.948442969 | 0.051361983 |
| KRTAP9-7 | ENST00000459985.1 | ENSG00000273066.1 | 0.850288904 | 1.857650171 | 0.063218709 |
| KRTAP9-7 | ENST00000489557.2 | ENSG00000257045.1 | 0.873908598 | 1.962951669 | 0.04965179 |
| KRTAP9-7 | ENST00000490013.1 | ENSG00000184115.12 | 0.900527938 | 2.021043595 | 0.043275252 |
| KRTAP9-7 | ENST00000498358.1 | ENSG00000184115.12 | 0.810005689 | 1.804195763 | 0.071200623 |
| KRTAP9-7 | ENST00000502421.1 | ENSG00000250284.1 | -0.824364771 | -1.825326398 | 0.067951789 |
| KRTAP9-7 | ENST00000503034.1 | ENSG00000248936.1 | 0.929123596 | 2.072143347 | 0.038252075 |
| KRTAP9-7 | ENST00000505196.1 | ENSG00000248131.1 | 0.921284875 | 2.078648588 | 0.037649662 |
| KRTAP9-7 | ENST00000505556.1 | ENSG00000249409.1 | 0.858342395 | 1.9300562 | 0.053599875 |
| KRTAP9-7 | ENST00000506100.1 | ENSG00000249409.1 | 0.874435632 | 1.975766317 | 0.04818125 |
| KRTAP9-7 | ENST00000507558.1 | ENSG00000248445.1 | 0.838305421 | 1.87157869 | 0.061264914 |
| KRTAP9-7 | ENST00000508004.2 | ENSG00000251661.3 | 0.861036725 | 1.913056416 | 0.05574083 |
| KRTAP9-7 | ENST00000508083.1 | ENSG00000249343.1 | 0.833408622 | 1.868343594 | 0.061714194 |
| KRTAP9-7 | ENST00000508188.1 | ENSG00000250999.1 | 0.82826385 | 1.864115933 | 0.062305426 |
| KRTAP9-7 | ENST00000514411.1 | ENSG00000250882.1 | 0.942790624 | 2.125693521 | 0.033528787 |
| KRTAP9-7 | ENST00000514877.1 | ENSG00000248685.1 | 0.92010614 | 2.029728728 | 0.042384121 |
| KRTAP9-7 | ENST00000515077.1 | ENSG00000251206.1 | 0.838864913 | 1.857915331 | 0.063181039 |
| KRTAP9-7 | ENST00000518260.1 | ENSG00000253628.1 | -0.845535934 | -1.876415601 | 0.060598234 |
| KRTAP9-7 | ENST00000518894.1 | ENSG00000204758.3 | 0.848527066 | 1.896294624 | 0.057921097 |

| | | | | | |
|---|---|---|---|---|---|
| KRTAP9-7 | ENST00000519038.2 | ENSG00000254054.2 | -0.852568599 | -1.908433966 | 0.05633515 |
| KRTAP9-7 | ENST00000519368.1 | ENSG00000253215.1 | 0.838768853 | 1.88263293 | 0.059750131 |
| KRTAP9-7 | ENST00000519451.1 | ENSG00000253363.1 | 0.844026981 | 1.884235978 | 0.059533063 |
| KRTAP9-7 | ENST00000519852.1 | ENSG00000253716.1 | 0.869723182 | 1.947890371 | 0.051428082 |
| KRTAP9-7 | ENST00000521207.1 | ENSG00000253716.1 | 0.917538668 | 2.056456299 | 0.039738554 |
| KRTAP9-7 | ENST00000522547.1 | ENSG00000253430.1 | -0.900964132 | -1.994700046 | 0.046075604 |
| KRTAP9-7 | ENST00000524335.1 | ENSG00000253716.1 | 0.909314564 | 2.035807493 | 0.04176969 |
| KRTAP9-7 | ENST00000526611.1 | ENSG00000246982.2 | 0.849186895 | 1.936739362 | 0.052777212 |
| KRTAP9-7 | ENST00000528000.1 | ENSG00000254804.1 | 0.925619095 | 2.058322322 | 0.039559203 |
| KRTAP9-7 | ENST00000528818.1 | ENSG00000232995.3 | 0.947007233 | 2.126500734 | 0.033461588 |
| KRTAP9-7 | ENST00000531136.1 | ENSG00000255558.1 | -0.939023352 | -2.086715581 | 0.036913852 |
| KRTAP9-7 | ENST00000536141.1 | ENSG00000256969.1 | 0.869733817 | 1.956667704 | 0.050386549 |
| KRTAP9-7 | ENST00000537269.1 | ENSG00000257084.1 | 0.879889525 | 1.968750993 | 0.048981692 |
| KRTAP9-7 | ENST00000543072.1 | ENSG00000256092.2 | -0.906431802 | -2.010675054 | 0.044359791 |
| KRTAP9-7 | ENST00000548731.1 | ENSG00000257809.1 | 0.8573861 | 1.889491829 | 0.058825957 |
| KRTAP9-7 | ENST00000552469.1 | ENSG00000258325.1 | 0.818513387 | 1.814140263 | 0.069656134 |
| KRTAP9-7 | ENST00000554049.1 | ENSG00000258763.1 | 0.937562986 | 2.076348402 | 0.03786174 |
| KRTAP9-7 | ENST00000554798.1 | ENSG00000258483.1 | 0.95727548 | 2.145993977 | 0.031873459 |
| KRTAP9-7 | ENST00000557412.1 | ENSG00000257621.3 | -0.859485073 | -1.881411322 | 0.059915988 |
| KRTAP9-7 | ENST00000558875.1 | ENSG00000259737.2 | 0.831691314 | 1.85794297 | 0.063177113 |
| KRTAP9-7 | ENST00000559569.1 | ENSG00000259760.1 | -0.806483326 | -1.802764846 | 0.071425153 |
| KRTAP9-7 | ENST00000561529.1 | ENSG00000260886.1 | 0.867045279 | 1.965772052 | 0.049324947 |
| KRTAP9-7 | ENST00000564102.1 | ENSG00000260041.1 | -0.818685488 | -1.84308484 | 0.065316624 |
| KRTAP9-7 | ENST00000565359.1 | ENSG00000260601.1 | 0.909565925 | 2.047972162 | 0.040562727 |
| KRTAP9-7 | ENST00000565829.1 | ENSG00000260148.1 | 0.908951653 | 2.013428756 | 0.044069543 |
| KRTAP9-7 | ENST00000569742.1 | ENSG00000260787.1 | 0.838593839 | 1.84984756 | 0.064335524 |
| KRTAP9-7 | ENST00000570158.1 | ENSG00000260937.1 | -0.885356945 | -1.991466039 | 0.046429675 |
| KRTAP9-7 | ENST00000570843.1 | ENSG00000261889.1 | 0.894897753 | 1.946951641 | 0.051540533 |
| KRTAP9-7 | ENST00000570929.1 | ENSG00000262223.2 | 0.853744525 | 1.89164681 | 0.058538055 |
| KRTAP9-7 | ENST00000571815.1 | ENSG00000262810.1 | 0.914561026 | 2.037794548 | 0.041570484 |
| KRTAP9-7 | ENST00000573414.1 | ENSG00000263072.1 | 0.859430012 | 1.92406287 | 0.054346703 |
| KRTAP9-7 | ENST00000574460.1 | ENSG00000263051.1 | 0.923234374 | 2.044593358 | 0.040894965 |
| KRTAP9-7 | ENST00000575139.1 | ENSG00000263072.1 | 0.848141248 | 1.896504277 | 0.057893395 |
| KRTAP9-7 | ENST00000576086.1 | ENSG00000262823.1 | 0.810396981 | 1.815845932 | 0.069394009 |
| KRTAP9-7 | ENST00000577064.1 | ENSG00000262823.1 | 0.910111211 | 2.038847521 | 0.041465248 |
| KRTAP9-7 | ENST00000578936.1 | ENSG00000265547.1 | 0.824672663 | 1.839126446 | 0.06589659 |
| KRTAP9-7 | ENST00000581905.1 | ENSG00000264235.1 | 0.934669166 | 2.074206453 | 0.038060141 |
| KRTAP9-7 | ENST00000583138.1 | ENSG00000263393.1 | 0.843619612 | 1.895302149 | 0.058052384 |
| KRTAP9-7 | ENST00000585072.1 | ENSG00000263745.1 | 0.865757735 | 1.95279939 | 0.050843371 |
| KRTAP9-7 | ENST00000586010.1 | ENSG00000267606.1 | 0.80275219 | 1.807355408 | 0.070706882 |
| KRTAP9-7 | ENST00000586051.1 | ENSG00000267576.1 | 0.948875401 | 2.124901342 | 0.033594846 |
| KRTAP9-7 | ENST00000589281.1 | ENSG00000267707.1 | -0.875042796 | -1.942663526 | 0.052056826 |
| KRTAP9-7 | ENST00000589673.1 | ENSG00000267755.1 | 0.82235079 | 1.799451602 | 0.071947273 |
| KRTAP9-7 | ENST00000591137.1 | ENSG00000267405.1 | 0.80242284 | 1.794502511 | 0.072732998 |
| KRTAP9-7 | ENST00000591836.1 | ENSG00000267776.1 | 0.883815104 | 1.998674429 | 0.045643591 |
| KRTAP9-7 | ENST00000593632.1 | ENSG00000180279.5 | 0.816951427 | 1.818928667 | 0.068922314 |
| KRTAP9-7 | ENST00000594590.2 | ENSG00000268199.2 | 0.886235283 | 1.964216352 | 0.049505007 |
| KRTAP9-7 | ENST00000595508.1 | ENSG00000269749.1 | 0.840012705 | 1.887364475 | 0.05911132 |
| KRTAP9-7 | ENST00000595955.1 | ENSG00000268401.1 | 0.830110251 | 1.84761442 | 0.064658142 |
| KRTAP9-7 | ENST00000597309.1 | ENSG00000232098.2 | -0.818160282 | -1.824641538 | 0.068055141 |
| KRTAP9-7 | ENST00000598131.1 | ENSG00000269043.1 | 0.869465645 | 1.929403571 | 0.053680781 |

| | | | | | |
|---|---|---|---|---|---|
| KRTAP9-7 | ENST00000599352.1 | ENSG00000240401.4 | -0.817555571 | -1.815654176 | 0.069423438 |
| KRTAP9-7 | ENST00000600071.1 | ENSG00000269199.1 | 0.917284613 | 2.085315696 | 0.037040654 |
| KRTAP9-7 | ENST00000600234.1 | ENSG00000268078.1 | 0.876942586 | 1.945503624 | 0.051714394 |
| KRTAP9-7 | ENST00000600534.1 | ENSG00000267858.1 | 0.893383376 | 2.037062538 | 0.041643775 |
| KRTAP9-7 | ENST00000600889.1 | ENSG00000232675.3 | 0.806519031 | 1.805740036 | 0.070958956 |
| KRTAP9-7 | ENST00000601752.1 | ENSG00000268051.1 | 0.831135914 | 1.846802366 | 0.064775789 |
| KRTAP9-7 | ENST00000602532.1 | ENSG00000270091.1 | 0.926481167 | 2.094297098 | 0.036233523 |
| KRTAP9-7 | ENST00000606068.1 | ENSG00000272342.1 | 0.943716341 | 2.104154216 | 0.035364997 |
| KRTAP9-7 | ENST00000606374.1 | ENSG00000272312.1 | -0.813597697 | -1.801557606 | 0.071615036 |
| KRTAP9-7 | ENST00000608952.1 | ENSG00000272689.1 | -0.86188834 | -1.934878181 | 0.053005246 |
| KRTAP9-7 | ENST00000609067.1 | ENSG00000272849.1 | 0.82668853 | 1.835880019 | 0.066375403 |
| KRTAP9-7 | ENST00000609146.1 | ENSG00000272851.1 | -0.820910577 | -1.821225001 | 0.068572662 |
| KRTAP9-7 | ENST00000609281.1 | ENSG00000273320.1 | 0.989351389 | 2.224283877 | 0.026129351 |
| KRTAP9-7 | NR_003604.2 | ZFAS1 | 0.830456395 | 1.851893127 | 0.064041171 |
| KRTAP9-7 | NR_003606.2 | ZFAS1 | 0.848082989 | 1.874571859 | 0.060851648 |
| KRTAP9-7 | NR_027271.1 | CIRBP-AS1 | 0.802459329 | 1.78850009 | 0.073695361 |
| KRTAP9-7 | NR_027334.2 | MZF1-AS1 | 0.886989973 | 1.958174863 | 0.050209498 |
| KRTAP9-7 | NR_034037.1 | LINC00582 | -0.968791457 | -2.173238283 | 0.029762388 |
| KRTAP9-7 | NR_034111.1 | TRAF3IP2-AS1 | 0.941561921 | 2.091515059 | 0.036481918 |
| KRTAP9-7 | NR_036658.1 | ZFAS1 | 0.83839199 | 1.893638135 | 0.058273058 |
| KRTAP9-7 | NR_046454.1 | LINC00907 | -0.964311293 | -2.143549923 | 0.032068967 |
| KRTAP9-7 | NR_103851.1 | TAT-AS1 | 0.810715977 | 1.794787724 | 0.072687527 |
| KRTAP9-7 | NR_104158.1 | NRG1-IT1 | 0.902623393 | 2.038624389 | 0.041487529 |
| KRTAP9-7 | NR_109870.1 | LINC01723 | 0.917269685 | 2.065452406 | 0.038880214 |
| KRTAP9-7 | NR_110318.1 | MACROD2-AS1 | 0.860176575 | 1.880467132 | 0.060044442 |
| KRTAP9-7 | NR_110568.1 | LOC101927661 | 0.943502352 | 2.108632575 | 0.034976309 |
| KRTAP9-7 | NR_110635.1 | LINC00687 | 0.842599696 | 1.872901476 | 0.061081991 |
| KRTAP9-7 | NR_110919.1 | LOC101928530 | 0.832342803 | 1.867376198 | 0.061849071 |
| KRTAP9-7 | NR_117097.1 | LINC01353 | 0.801525397 | 1.813177243 | 0.069804489 |
| KRTAP9-7 | NR_117098.1 | LINC01353 | 0.876515331 | 1.944868059 | 0.05179086 |
| KRTAP9-7 | NR_120423.1 | LOC101929140 | -0.842548424 | -1.900234412 | 0.057402364 |
| KRTAP9-7 | NR_125925.1 | LOC101929448 | 0.843351489 | 1.895117065 | 0.058076894 |
| KRTAP9-7 | NR_126380.1 | LINC01072 | 0.853145142 | 1.907986159 | 0.056393005 |
| KRTAP9-7 | NR_130144.1 | LOC104968399 | 0.934669166 | 2.108118376 | 0.035020751 |
| KRTAP9-7 | NR_133658.1 | HTR3E-AS1 | -0.876409197 | -1.952497008 | 0.050879226 |
| KRTAP9-7 | NR_134273.1 | LOC101929544 | 0.806075198 | 1.783345984 | 0.07453 |
| KRTAP9-7 | NR_135032.1 | LOC105369635 | 0.879889525 | 1.935341311 | 0.052948426 |
| KRTAP9-7 | NR_135644.1 | LOC105371506 | -0.960699808 | -2.178858999 | 0.029342144 |
| KRTAP9-7 | NR_138041.1 | LINC00384 | 0.894707518 | 2.033462949 | 0.042005773 |
| LAT2 | ENST00000398777.3 | ENSG00000240152.2 | 0.901333236 | 2.030707485 | 0.042284677 |
| LAT2 | ENST00000413650.1 | ENSG00000230880.2 | 0.813361659 | 1.804989545 | 0.071076318 |
| LAT2 | ENST00000413969.1 | ENSG00000224189.2 | 0.821213521 | 1.839092913 | 0.065901521 |
| LAT2 | ENST00000414377.1 | ENSG00000230470.1 | 0.85646987 | 1.878030779 | 0.060376955 |
| LAT2 | ENST00000416220.1 | ENSG00000236753.1 | 0.908999004 | 2.071912818 | 0.038273572 |
| LAT2 | ENST00000416641.1 | ENSG00000226956.1 | -0.938364697 | -2.097012071 | 0.035992507 |
| LAT2 | ENST00000417426.1 | ENSG00000233145.1 | 0.917205699 | 2.029324493 | 0.04242525 |
| LAT2 | ENST00000417782.1 | ENSG00000228587.1 | 0.910228659 | 2.031801801 | 0.042173725 |
| LAT2 | ENST00000418741.1 | ENSG00000227332.1 | 0.896318007 | 2.002833433 | 0.04519517 |
| LAT2 | ENST00000420830.1 | ENSG00000231512.1 | 0.841096604 | 1.889792968 | 0.058785654 |
| LAT2 | ENST00000421252.2 | ENSG00000250258.1 | -0.877012764 | -1.972700581 | 0.048529686 |
| LAT2 | ENST00000423667.1 | ENSG00000225970.1 | 0.887020985 | 1.957484919 | 0.050290483 |

| | | | | | |
|---|---|---|---|---|---|
| LAT2 | ENST00000425624.1 | ENSG00000223779.4 | 0.904531125 | 2.015934983 | 0.043806775 |
| LAT2 | ENST00000426213.1 | ENSG00000223660.1 | -0.852965489 | -1.897841355 | 0.057716984 |
| LAT2 | ENST00000426699.1 | ENSG00000229308.1 | 0.903981467 | 2.008678281 | 0.044571264 |
| LAT2 | ENST00000428769.1 | ENSG00000232738.1 | 0.886689424 | 2.026322723 | 0.042731724 |
| LAT2 | ENST00000432314.1 | ENSG00000231532.1 | 0.837930521 | 1.897390902 | 0.057776366 |
| LAT2 | ENST00000433344.1 | ENSG00000234083.1 | -0.904869502 | -2.022553545 | 0.043119198 |
| LAT2 | ENST00000433614.1 | ENSG00000228534.1 | -0.886982331 | -1.974031333 | 0.04837818 |
| LAT2 | ENST00000434790.1 | ENSG00000240040.1 | -0.87531302 | -1.965969994 | 0.049302077 |
| LAT2 | ENST00000435992.2 | ENSG00000232675.3 | 0.879279927 | 1.939926276 | 0.05238865 |
| LAT2 | ENST00000436982.2 | ENSG00000235335.2 | -0.802108995 | -1.820592365 | 0.068668844 |
| LAT2 | ENST00000438222.1 | ENSG00000238034.1 | 0.872047223 | 1.956973737 | 0.050350556 |
| LAT2 | ENST00000439186.1 | ENSG00000237076.1 | 0.929519766 | 2.050645517 | 0.04030148 |
| LAT2 | ENST00000440947.1 | ENSG00000225472.1 | 0.860545894 | 1.949533323 | 0.051231767 |
| LAT2 | ENST00000444245.1 | ENSG00000236753.1 | 0.89664445 | 1.965663001 | 0.049337551 |
| LAT2 | ENST00000447183.2 | ENSG00000271593.1 | 0.898091058 | 2.011403533 | 0.04428285 |
| LAT2 | ENST00000447514.1 | ENSG00000236753.1 | 0.96063928 | 2.15646863 | 0.031047091 |
| LAT2 | ENST00000447538.2 | ENSG00000224189.2 | 0.848497324 | 1.886473944 | 0.059231116 |
| LAT2 | ENST00000447709.1 | ENSG00000237473.1 | 0.884503123 | 1.964650427 | 0.049454711 |
| LAT2 | ENST00000450226.1 | ENSG00000231512.1 | 0.915744884 | 2.024378761 | 0.042931195 |
| LAT2 | ENST00000450848.1 | ENSG00000225539.1 | 0.969927342 | 2.176187198 | 0.029541266 |
| LAT2 | ENST00000451556.2 | ENSG00000228386.2 | 0.81023154 | 1.804432105 | 0.071163594 |
| LAT2 | ENST00000451575.2 | ENSG00000224251.2 | 0.831034638 | 1.877923112 | 0.060391684 |
| LAT2 | ENST00000451656.1 | ENSG00000228417.1 | 0.824695676 | 1.827561298 | 0.067615419 |
| LAT2 | ENST00000452412.1 | ENSG00000233860.1 | 0.868321156 | 1.932342331 | 0.053317267 |
| LAT2 | ENST00000453579.1 | ENSG00000232529.1 | 0.825479279 | 1.850403951 | 0.06425535 |
| LAT2 | ENST00000454489.1 | ENSG00000231403.1 | 0.916704128 | 2.065012241 | 0.038921842 |
| LAT2 | ENST00000454515.1 | ENSG00000236753.1 | 0.8449173 | 1.87421059 | 0.060901405 |
| LAT2 | ENST00000454526.1 | ENSG00000234136.1 | 0.942954436 | 2.102165053 | 0.03553882 |
| LAT2 | ENST00000455238.1 | ENSG00000231413.1 | 0.829433174 | 1.827041992 | 0.067693456 |
| LAT2 | ENST00000455699.1 | ENSG00000240996.1 | 0.988690144 | 2.232083856 | 0.025609421 |
| LAT2 | ENST00000457169.1 | ENSG00000232408.1 | 0.955019423 | 2.16858449 | 0.030114244 |
| LAT2 | ENST00000458661.2 | ENSG00000236467.3 | 0.924676465 | 2.092965865 | 0.036352202 |
| LAT2 | ENST00000468444.2 | ENSG00000258525.1 | -0.82390192 | -1.844120149 | 0.065165632 |
| LAT2 | ENST00000469931.2 | ENSG00000272030.1 | 0.966426992 | 2.16883757 | 0.030095018 |
| LAT2 | ENST00000484413.1 | ENSG00000271853.1 | 0.889570667 | 1.992373165 | 0.046330129 |
| LAT2 | ENST00000501405.2 | ENSG00000247402.2 | -0.936954941 | -2.094400137 | 0.036224351 |
| LAT2 | ENST00000503938.1 | ENSG00000246095.2 | 0.802333865 | 1.780122524 | 0.075055911 |
| LAT2 | ENST00000504017.1 | ENSG00000248388.1 | 0.835359034 | 1.86775103 | 0.061796782 |
| LAT2 | ENST00000504344.1 | ENSG00000251438.1 | 0.95604664 | 2.145587753 | 0.031905883 |
| LAT2 | ENST00000504891.1 | ENSG00000249388.1 | 0.970512225 | 2.166661813 | 0.03026065 |
| LAT2 | ENST00000505498.1 | ENSG00000250908.1 | 0.871046802 | 1.956012653 | 0.050463664 |
| LAT2 | ENST00000506059.1 | ENSG00000248311.1 | 0.930472606 | 2.0623314 | 0.039176196 |
| LAT2 | ENST00000507373.1 | ENSG00000250072.1 | 0.93667532 | 2.085393968 | 0.037033554 |
| LAT2 | ENST00000508845.1 | ENSG00000271724.1 | 0.850572648 | 1.887886814 | 0.059041147 |
| LAT2 | ENST00000513179.1 | ENSG00000251580.1 | 0.855339101 | 1.919182512 | 0.05496124 |
| LAT2 | ENST00000514802.1 | ENSG00000250190.1 | 0.814315509 | 1.825300561 | 0.067955686 |
| LAT2 | ENST00000515205.1 | ENSG00000251580.1 | 0.824092927 | 1.852872051 | 0.0639007 |
| LAT2 | ENST00000517716.1 | ENSG00000253515.1 | -0.94081828 | -2.101679579 | 0.035581354 |
| LAT2 | ENST00000518416.1 | ENSG00000253901.1 | 0.897605808 | 1.992680739 | 0.046296418 |
| LAT2 | ENST00000518473.1 | ENSG00000253985.1 | 0.904562766 | 2.018788285 | 0.043509229 |
| LAT2 | ENST00000519660.1 | ENSG00000253416.1 | 0.862772978 | 1.929222528 | 0.053703243 |

| | | | | | |
|---|---|---|---|---|---|
| LAT2 | ENST00000520749.1 | ENSG00000253717.1 | -0.829612465 | -1.851319659 | 0.06412358 |
| LAT2 | ENST00000521307.1 | ENSG00000253177.1 | 0.882117498 | 1.95202177 | 0.05093562 |
| LAT2 | ENST00000521953.1 | ENSG00000253214.1 | 0.935543723 | 2.081436605 | 0.037393961 |
| LAT2 | ENST00000522704.1 | ENSG00000254135.1 | 0.940195119 | 2.0665004 | 0.038781255 |
| LAT2 | ENST00000524818.1 | ENSG00000254473.1 | 0.898493363 | 2.033378376 | 0.04201431 |
| LAT2 | ENST00000525133.1 | ENSG00000255375.1 | 0.909299206 | 2.043975285 | 0.040955989 |
| LAT2 | ENST00000526186.1 | ENSG00000254510.1 | 0.835031344 | 1.862989705 | 0.062463715 |
| LAT2 | ENST00000526935.1 | ENSG00000255372.1 | 0.947738773 | 2.105236491 | 0.035270727 |
| LAT2 | ENST00000529837.1 | ENSG00000254687.1 | 0.963414942 | 2.152893016 | 0.031327087 |
| LAT2 | ENST00000529875.1 | ENSG00000254404.1 | -0.828790884 | -1.85423239 | 0.06370592 |
| LAT2 | ENST00000531661.1 | ENSG00000254473.1 | 0.946321719 | 2.122165998 | 0.033823801 |
| LAT2 | ENST00000535324.1 | ENSG00000255968.1 | 0.814944524 | 1.846407977 | 0.06483299 |
| LAT2 | ENST00000544089.1 | ENSG00000256273.1 | 0.900975116 | 1.986013694 | 0.047031803 |
| LAT2 | ENST00000545593.1 | ENSG00000256972.1 | 0.88276146 | 2.002454527 | 0.045235869 |
| LAT2 | ENST00000547175.1 | ENSG00000257395.1 | -0.8398272 | -1.874950926 | 0.060799475 |
| LAT2 | ENST00000547207.1 | ENSG00000224189.2 | 0.816799724 | 1.807489401 | 0.070686006 |
| LAT2 | ENST00000547834.1 | ENSG00000258325.1 | 0.83670741 | 1.841166176 | 0.06559721 |
| LAT2 | ENST00000549140.1 | ENSG00000258332.1 | 0.982896684 | 2.185872685 | 0.028824921 |
| LAT2 | ENST00000549329.1 | ENSG00000224189.2 | 0.888387848 | 1.996328306 | 0.045898199 |
| LAT2 | ENST00000549487.1 | ENSG00000257126.1 | 0.807704586 | 1.778394848 | 0.075339028 |
| LAT2 | ENST00000549756.1 | ENSG00000257769.1 | -0.845271943 | -1.89794334 | 0.057703546 |
| LAT2 | ENST00000552156.1 | ENSG00000224189.2 | 0.89704352 | 1.984429014 | 0.047208034 |
| LAT2 | ENST00000555966.1 | ENSG00000258843.1 | 0.964148568 | 2.174633766 | 0.029657572 |
| LAT2 | ENST00000556786.1 | ENSG00000258525.1 | -0.850642416 | -1.896236981 | 0.057928715 |
| LAT2 | ENST00000557903.1 | ENSG00000259182.1 | 0.916912322 | 2.060332209 | 0.039366793 |
| LAT2 | ENST00000557965.1 | ENSG00000259681.1 | 0.871657414 | 1.953983965 | 0.050703114 |
| LAT2 | ENST00000558141.1 | ENSG00000259594.1 | 0.807161176 | 1.802077414 | 0.071533227 |
| LAT2 | ENST00000558312.1 | ENSG00000259176.1 | 0.835525987 | 1.87762257 | 0.060432816 |
| LAT2 | ENST00000558896.1 | ENSG00000259176.1 | 0.829717247 | 1.868409725 | 0.061704983 |
| LAT2 | ENST00000559003.1 | ENSG00000259520.1 | 0.951490375 | 2.139754007 | 0.032374652 |
| LAT2 | ENST00000560586.1 | ENSG00000259534.1 | 0.922122273 | 2.059553297 | 0.039441265 |
| LAT2 | ENST00000563841.1 | ENSG00000261029.1 | 0.972312109 | 2.132353947 | 0.03297776 |
| LAT2 | ENST00000565965.1 | ENSG00000261172.1 | 0.984862439 | 2.169987849 | 0.030007766 |
| LAT2 | ENST00000567089.1 | ENSG00000261822.1 | 0.95514127 | 2.118236554 | 0.034155039 |
| LAT2 | ENST00000570512.1 | ENSG00000262768.1 | 0.886022558 | 1.993327221 | 0.046225627 |
| LAT2 | ENST00000572417.1 | ENSG00000263171.1 | 0.8521115 | 1.919301593 | 0.054946177 |
| LAT2 | ENST00000574365.1 | ENSG00000262837.1 | 0.89366051 | 2.002365449 | 0.045245442 |
| LAT2 | ENST00000577698.1 | ENSG00000265100.1 | 0.901327471 | 2.038723256 | 0.041477655 |
| LAT2 | ENST00000578035.1 | ENSG00000266743.1 | -0.845587781 | -1.884246156 | 0.059531687 |
| LAT2 | ENST00000578265.1 | ENSG00000214719.7 | 0.899243193 | 1.997593721 | 0.045760725 |
| LAT2 | ENST00000578349.1 | ENSG00000263688.1 | 0.83410292 | 1.861498631 | 0.062673794 |
| LAT2 | ENST00000578757.1 | ENSG00000175061.13 | 0.811675114 | 1.825878431 | 0.067868576 |
| LAT2 | ENST00000581362.1 | ENSG00000235300.3 | 0.87990127 | 1.961936833 | 0.049769839 |
| LAT2 | ENST00000581996.1 | ENSG00000265778.1 | 0.807909308 | 1.794591951 | 0.072718736 |
| LAT2 | ENST00000582348.1 | ENSG00000265148.1 | 0.87475227 | 1.956904134 | 0.05035874 |
| LAT2 | ENST00000584758.1 | ENSG00000265356.1 | 0.934166248 | 2.112493145 | 0.034644173 |
| LAT2 | ENST00000586010.1 | ENSG00000267606.1 | 0.85578733 | 1.897836896 | 0.057717571 |
| LAT2 | ENST00000588799.1 | ENSG00000267275.1 | 0.966214204 | 2.185322212 | 0.02886523 |
| LAT2 | ENST00000588835.1 | ENSG00000267476.1 | 0.815278602 | 1.833105856 | 0.06678683 |
| LAT2 | ENST00000588945.1 | ENSG00000267275.1 | 0.907316807 | 2.033151579 | 0.042037211 |
| LAT2 | ENST00000589777.1 | ENSG00000261040.2 | 0.934402876 | 2.091014801 | 0.036526737 |

| | | | | | |
|---|---|---|---|---|---|
| LAT2 | ENST00000593861.1 | ENSG00000231898.4 | 0.861304276 | 1.894127187 | 0.05820813 |
| LAT2 | ENST00000594762.1 | ENSG00000231898.4 | 0.86318301 | 1.920466941 | 0.054798945 |
| LAT2 | ENST00000595892.1 | ENSG00000269640.1 | 0.889700821 | 1.975280129 | 0.048236367 |
| LAT2 | ENST00000597550.1 | ENSG00000269051.1 | 0.848926354 | 1.917840751 | 0.055131207 |
| LAT2 | ENST00000599143.1 | ENSG00000269349.1 | 0.881892818 | 1.97621105 | 0.048130879 |
| LAT2 | ENST00000600489.1 | ENSG00000231898.4 | 0.982162934 | 2.183052758 | 0.029031925 |
| LAT2 | ENST00000602592.1 | ENSG00000270049.1 | 0.940191093 | 2.076421182 | 0.037855014 |
| LAT2 | ENST00000602790.1 | ENSG00000270000.1 | -0.804103121 | -1.78852439 | 0.073691444 |
| LAT2 | ENST00000602809.1 | ENSG00000270105.1 | -0.816537573 | -1.820983465 | 0.06860937 |
| LAT2 | ENST00000602872.1 | ENSG00000270067.1 | 0.884277828 | 1.965564911 | 0.049348891 |
| LAT2 | ENST00000602954.1 | ENSG00000269906.1 | 0.866584171 | 1.960305768 | 0.049960062 |
| LAT2 | ENST00000603474.1 | ENSG00000258929.2 | 0.937488509 | 2.096260651 | 0.036059075 |
| LAT2 | ENST00000606457.1 | ENSG00000271830.1 | 0.932115294 | 2.106969917 | 0.035120187 |
| LAT2 | ENST00000607222.1 | ENSG00000272106.1 | 0.827239929 | 1.840734312 | 0.065660503 |
| LAT2 | ENST00000607284.1 | ENSG00000272389.1 | 0.810075731 | 1.827721429 | 0.06759137 |
| LAT2 | ENST00000607715.1 | ENSG00000271788.1 | 0.834968398 | 1.854475183 | 0.063671207 |
| LAT2 | ENST00000607769.1 | ENSG00000272438.1 | -0.905589979 | -2.012840884 | 0.044131371 |
| LAT2 | ENST00000607876.1 | ENSG00000272848.1 | 0.961971565 | 2.147414213 | 0.03176032 |
| LAT2 | ENST00000608085.1 | ENSG00000231898.4 | 0.964170668 | 2.11897564 | 0.034092526 |
| LAT2 | ENST00000608476.1 | ENSG00000232675.3 | 0.975051566 | 2.218996825 | 0.026486941 |
| LAT2 | ENST00000609610.1 | ENSG00000232675.3 | 0.964487869 | 2.152360764 | 0.031368951 |
| LAT2 | ENST00000609701.1 | ENSG00000273284.1 | 0.94737902 | 2.094538058 | 0.036212076 |
| LAT2 | ENST00000609725.1 | ENSG00000231898.4 | 0.958191149 | 2.151921091 | 0.03140357 |
| LAT2 | ENST00000609890.1 | ENSG00000231898.4 | 0.901969975 | 2.015656563 | 0.043835901 |
| LAT2 | ENST00000609972.1 | ENSG00000230651.3 | 0.901890977 | 1.993898545 | 0.046163143 |
| LAT2 | ENST00000610161.1 | ENSG00000273059.1 | 0.895539251 | 1.983859062 | 0.047271554 |
| LAT2 | NR_022011.1 | PWARSN | 0.805395859 | 1.782343119 | 0.074693295 |
| LAT2 | NR_024410.1 | LINC00710 | 0.896569417 | 1.98546534 | 0.047092723 |
| LAT2 | NR_033371.1 | CDRT7 | 0.830645394 | 1.865160438 | 0.062158919 |
| LAT2 | NR_044996.1 | HCG23 | 0.881651671 | 1.956524336 | 0.050403419 |
| LAT2 | NR_104620.1 | LINC01672 | 0.841264084 | 1.880990758 | 0.059973176 |
| LAT2 | NR_104998.1 | LOC102467225 | 0.91000443 | 2.03187465 | 0.042166347 |
| LAT2 | NR_110480.1 | LOC101927079 | 0.911973047 | 2.064499132 | 0.038970416 |
| LAT2 | NR_110481.1 | LOC101927079 | 0.835525987 | 1.885775523 | 0.059325211 |
| LAT2 | NR_120335.1 | LOC101928414 | 0.905021785 | 2.021507888 | 0.043227217 |
| LAT2 | NR_120655.1 | KCNMA1-AS1 | 0.949639447 | 2.140133217 | 0.032344002 |
| LAT2 | NR_126166.1 | FAM74A7 | 0.909902212 | 2.042308164 | 0.041120973 |
| LAT2 | NR_134597.1 | LOC105378068 | 0.990919671 | 2.25859589 | 0.023908534 |
| LAT2 | NR_135108.1 | LOC105369509 | 0.85800382 | 1.896693643 | 0.057868383 |
| LAT2 | NR_135258.1 | LOC105370489 | 0.921581725 | 2.045243339 | 0.040830874 |
| LAT2 | NR_135840.1 | LOC105376114 | 0.903612802 | 2.020068503 | 0.043376282 |
| LAT2 | NR_138084.1 | HCG24 | 0.836068384 | 1.872115522 | 0.061190623 |
| LCE1D | ENST00000413645.1 | ENSG00000228798.1 | 0.945238495 | 2.115658235 | 0.034373885 |
| LCE1D | ENST00000420498.1 | ENSG00000224985.1 | 0.871393928 | 1.959621342 | 0.050040065 |
| LCE1D | ENST00000436132.1 | ENSG00000244125.1 | 0.830995114 | 1.848159096 | 0.064579331 |
| LCE1D | ENST00000440492.1 | ENSG00000233975.1 | -0.824203882 | -1.854117815 | 0.063722307 |
| LCE1D | ENST00000443123.1 | ENSG00000229457.1 | 0.814149966 | 1.832255521 | 0.06691336 |
| LCE1D | ENST00000446560.1 | ENSG00000229258.1 | 0.917154619 | 2.086683617 | 0.036916743 |
| LCE1D | ENST00000454262.2 | ENSG00000229923.2 | -0.830315313 | -1.848537387 | 0.064524641 |
| LCE1D | ENST00000502300.1 | ENSG00000249451.1 | 0.913974568 | 2.039532131 | 0.041396948 |
| LCE1D | ENST00000507997.1 | ENSG00000250551.1 | -0.851046318 | -1.895153952 | 0.058072009 |

| | | | | | |
|---|---|---|---|---|---|
| LCE1D | ENST00000508374.1 | ENSG00000249441.1 | 0.85044177 | 1.885930745 | 0.059304288 |
| LCE1D | ENST00000512300.1 | ENSG00000248362.1 | 0.841387288 | 1.891519044 | 0.058555091 |
| LCE1D | ENST00000512563.1 | ENSG00000249547.1 | 0.881673428 | 1.981422855 | 0.047543873 |
| LCE1D | ENST00000515136.1 | ENSG00000251274.1 | -0.935043824 | -2.075072455 | 0.03797982 |
| LCE1D | ENST00000521660.1 | ENSG00000253455.1 | -0.87654111 | -1.944313996 | 0.051857598 |
| LCE1D | ENST00000522524.1 | ENSG00000253342.1 | -0.863763114 | -1.913094399 | 0.055735969 |
| LCE1D | ENST00000524824.1 | ENSG00000255328.1 | 0.93508143 | 2.089147847 | 0.036694416 |
| LCE1D | ENST00000527297.1 | ENSG00000255229.1 | -0.910150496 | -2.059173077 | 0.039477661 |
| LCE1D | ENST00000532249.1 | ENSG00000234899.5 | 0.839029483 | 1.898854934 | 0.057583552 |
| LCE1D | ENST00000535746.1 | ENSG00000256101.1 | 0.926991302 | 2.097401932 | 0.03595801 |
| LCE1D | ENST00000536412.1 | ENSG00000256072.1 | -0.819044792 | -1.823884172 | 0.068169585 |
| LCE1D | ENST00000550886.1 | ENSG00000257696.1 | 0.886871623 | 1.983912851 | 0.047265556 |
| LCE1D | ENST00000554197.1 | ENSG00000197176.3 | 0.945246918 | 2.113645775 | 0.034545533 |
| LCE1D | ENST00000557232.1 | ENSG00000259054.1 | 0.851515152 | 1.916015803 | 0.055363084 |
| LCE1D | ENST00000561215.1 | ENSG00000259611.1 | 0.889438436 | 2.004961538 | 0.044967159 |
| LCE1D | ENST00000570919.1 | ENSG00000263321.1 | 0.885759172 | 1.993463586 | 0.046210707 |
| LCE1D | ENST00000572222.1 | ENSG00000261971.2 | -0.882866081 | -1.950048632 | 0.051170323 |
| LCE1D | ENST00000573953.1 | ENSG00000261971.2 | -0.841375337 | -1.904842982 | 0.056800485 |
| LCE1D | ENST00000590292.1 | ENSG00000267751.1 | 0.820621426 | 1.813741724 | 0.069717498 |
| LCE1D | ENST00000590368.1 | ENSG00000231616.4 | 0.849307892 | 1.887319839 | 0.05911732 |
| LCE1D | ENST00000590995.1 | ENSG00000267198.1 | 0.859318921 | 1.936936521 | 0.052753103 |
| LCE1D | ENST00000591217.1 | ENSG00000231616.4 | 0.921918798 | 2.053988768 | 0.039976779 |
| LCE1D | ENST00000592622.1 | ENSG00000267546.2 | -0.907625562 | -2.012190132 | 0.044199898 |
| LCE1D | ENST00000593269.1 | ENSG00000236172.2 | 0.901925164 | 1.995686309 | 0.045968078 |
| LCE1D | ENST00000594091.1 | ENSG00000232732.5 | -0.863863959 | -1.943171081 | 0.051995491 |
| LCE1D | ENST00000594492.1 | ENSG00000250910.3 | 0.816239905 | 1.813857594 | 0.069699653 |
| LCE1D | ENST00000596497.1 | ENSG00000268530.1 | 0.838504524 | 1.88640548 | 0.059240335 |
| LCE1D | ENST00000600512.1 | ENSG00000269752.1 | 0.804057712 | 1.787994819 | 0.073776844 |
| LCE1D | ENST00000600956.1 | ENSG00000232732.5 | -0.932238188 | -2.071052302 | 0.038353909 |
| LCE1D | ENST00000602614.1 | ENSG00000269957.1 | -0.919268663 | -2.068716724 | 0.038572677 |
| LCE1D | ENST00000602773.1 | ENSG00000270160.1 | -0.961633847 | -2.137536061 | 0.032554416 |
| LCE1D | ENST00000607044.1 | ENSG00000272247.1 | 0.894411817 | 1.951526948 | 0.050994394 |
| LCE1D | ENST00000607051.1 | ENSG00000271771.1 | 0.843353444 | 1.884373503 | 0.059514471 |
| LCE1D | ENST00000608264.1 | ENSG00000273473.1 | 0.806780549 | 1.823572421 | 0.068216739 |
| LCE1D | ENST00000609837.1 | ENSG00000273106.1 | 0.805608634 | 1.789571156 | 0.073522879 |
| LCE1D | ENST00000609953.1 | ENSG00000272825.1 | 0.820043007 | 1.828813011 | 0.067427625 |
| LCE1D | NR_027401.2 | FAM223A | 0.947106756 | 2.100393932 | 0.035694202 |
| LCE1D | NR_028325.1 | LOC100132062 | 0.82202731 | 1.834648285 | 0.066557819 |
| LCE1D | NR_046369.1 | LOC100131626 | -0.889406713 | -2.000741976 | 0.045420203 |
| LCE1D | NR_046370.1 | LOC100131626 | -0.918476335 | -2.057255816 | 0.039661625 |
| LCE1D | NR_103857.1 | SP2-AS1 | -0.81270641 | -1.824872757 | 0.068020233 |
| LCE1D | NR_108068.1 | LINC00836 | -0.842500006 | -1.882731021 | 0.059736829 |
| LCE1D | NR_109770.1 | TONSL-AS1 | -0.804539008 | -1.781318149 | 0.074860491 |
| LST1 | ENST00000411694.1 | ENSG00000225331.1 | 0.802953304 | 1.787567648 | 0.073845789 |
| LST1 | ENST00000412759.1 | ENSG00000236933.1 | 0.866841185 | 1.930637461 | 0.053527902 |
| LST1 | ENST00000415106.1 | ENSG00000226733.1 | -0.80043149 | -1.779333475 | 0.075185106 |
| LST1 | ENST00000415205.1 | ENSG00000182057.4 | 0.943447551 | 2.101662597 | 0.035582842 |
| LST1 | ENST00000417260.1 | ENSG00000231734.4 | -0.834631206 | -1.855075155 | 0.063585495 |
| LST1 | ENST00000419662.1 | ENSG00000228265.1 | 0.899453117 | 2.034612931 | 0.041889835 |
| LST1 | ENST00000422763.1 | ENSG00000231131.2 | -0.871447804 | -1.952409236 | 0.050889637 |
| LST1 | ENST00000425881.1 | ENSG00000239636.1 | 0.858347315 | 1.883867784 | 0.059582862 |

| | | | | | |
|---|---|---|---|---|---|
| LST1 | ENST00000426237.2 | ENSG00000235527.2 | 0.823325381 | 1.857098821 | 0.063297096 |
| LST1 | ENST00000426475.1 | ENSG00000239467.1 | 0.899033232 | 2.01972445 | 0.043411977 |
| LST1 | ENST00000428765.1 | ENSG00000230107.1 | 0.860466855 | 1.916054647 | 0.05535814 |
| LST1 | ENST00000429080.1 | ENSG00000233047.1 | -0.901842946 | -2.022514376 | 0.04312324 |
| LST1 | ENST00000430920.1 | ENSG00000234203.1 | 0.91722729 | 2.029329517 | 0.042424739 |
| LST1 | ENST00000431730.1 | ENSG00000237401.2 | 0.819994298 | 1.80823542 | 0.070569868 |
| LST1 | ENST00000433051.1 | ENSG00000233193.1 | 0.850751492 | 1.93353274 | 0.053170604 |
| LST1 | ENST00000433905.2 | ENSG00000229299.2 | 0.937652309 | 2.084273166 | 0.037135327 |
| LST1 | ENST00000438190.1 | ENSG00000227214.2 | 0.902874931 | 2.037398131 | 0.041610161 |
| LST1 | ENST00000438969.2 | ENSG00000228031.2 | -0.844229948 | -1.895073545 | 0.058082659 |
| LST1 | ENST00000439184.1 | ENSG00000233985.1 | -0.807275967 | -1.792981362 | 0.072975904 |
| LST1 | ENST00000440595.1 | ENSG00000228265.1 | 0.898490049 | 2.025442165 | 0.042821982 |
| LST1 | ENST00000441592.2 | ENSG00000224078.8 | 0.92841422 | 2.083466629 | 0.03720871 |
| LST1 | ENST00000442829.1 | ENSG00000225284.1 | 0.81576249 | 1.823037009 | 0.068297786 |
| LST1 | ENST00000446562.1 | ENSG00000233896.1 | 0.869445113 | 1.976894556 | 0.04805355 |
| LST1 | ENST00000447343.2 | ENSG00000229299.2 | 0.92953479 | 2.085638808 | 0.037011353 |
| LST1 | ENST00000448570.1 | ENSG00000224549.1 | 0.831889614 | 1.877154341 | 0.060496943 |
| LST1 | ENST00000448858.1 | ENSG00000237734.1 | -0.825442934 | -1.837463422 | 0.066141511 |
| LST1 | ENST00000451507.1 | ENSG00000229539.1 | 0.90786894 | 2.034288211 | 0.041922545 |
| LST1 | ENST00000452176.1 | ENSG00000223659.1 | -0.849070613 | -1.902878512 | 0.057056399 |
| LST1 | ENST00000453051.1 | ENSG00000229407.1 | 0.858133267 | 1.895091008 | 0.058080346 |
| LST1 | ENST00000458154.1 | ENSG00000235578.1 | 0.81804074 | 1.828825848 | 0.067425702 |
| LST1 | ENST00000463255.1 | ENSG00000243305.1 | -0.889065989 | -1.974719334 | 0.048300008 |
| LST1 | ENST00000468165.1 | ENSG00000239480.1 | 0.907967254 | 2.014438707 | 0.043963494 |
| LST1 | ENST00000489077.1 | ENSG00000244198.1 | 0.96691703 | 2.153278977 | 0.03129676 |
| LST1 | ENST00000489090.1 | ENSG00000240045.1 | -0.825723289 | -1.859449422 | 0.062963462 |
| LST1 | ENST00000498693.1 | ENSG00000244198.1 | 0.881805206 | 1.993070734 | 0.046253702 |
| LST1 | ENST00000503723.1 | ENSG00000250472.1 | -0.976924751 | -2.16709576 | 0.030227553 |
| LST1 | ENST00000504916.1 | ENSG00000248112.1 | -0.836891664 | -1.859168235 | 0.063003296 |
| LST1 | ENST00000506394.1 | ENSG00000251665.1 | 0.801185572 | 1.784014275 | 0.074421346 |
| LST1 | ENST00000506723.2 | ENSG00000249484.4 | -0.801517295 | -1.814692028 | 0.069571251 |
| LST1 | ENST00000506791.1 | ENSG00000251131.1 | 0.888296392 | 1.984035145 | 0.047251922 |
| LST1 | ENST00000509036.1 | ENSG00000251131.1 | 0.90019269 | 2.012351601 | 0.044182887 |
| LST1 | ENST00000509192.1 | ENSG00000250765.1 | 0.878902225 | 1.966752709 | 0.049211727 |
| LST1 | ENST00000509453.1 | ENSG00000249145.1 | 0.889633778 | 1.958437991 | 0.050178641 |
| LST1 | ENST00000510570.1 | ENSG00000250438.1 | -0.916441022 | -2.019969469 | 0.043386555 |
| LST1 | ENST00000517846.1 | ENSG00000254485.1 | 0.910319345 | 2.037516409 | 0.041598319 |
| LST1 | ENST00000520603.1 | ENSG00000254001.1 | -0.918182791 | -2.069478513 | 0.038501206 |
| LST1 | ENST00000521653.1 | ENSG00000253301.1 | 0.939203814 | 2.08812724 | 0.036786358 |
| LST1 | ENST00000523806.1 | ENSG00000253616.1 | 0.801201807 | 1.771113184 | 0.076541882 |
| LST1 | ENST00000529247.1 | ENSG00000254741.1 | 0.886810718 | 1.980895659 | 0.047602976 |
| LST1 | ENST00000543275.1 | ENSG00000256944.1 | 0.856729101 | 1.920073192 | 0.054848655 |
| LST1 | ENST00000543403.1 | ENSG00000256684.1 | -0.851804431 | -1.908235217 | 0.056360822 |
| LST1 | ENST00000545177.3 | ENSG00000230438.5 | 0.833312919 | 1.87860297 | 0.060298725 |
| LST1 | ENST00000549806.1 | ENSG00000257252.1 | 0.909235276 | 2.052249862 | 0.040145386 |
| LST1 | ENST00000558575.1 | ENSG00000259687.1 | 0.824910437 | 1.854910429 | 0.063609018 |
| LST1 | ENST00000562191.1 | ENSG00000261292.1 | -0.86839723 | -1.934889899 | 0.053003808 |
| LST1 | ENST00000563610.1 | ENSG00000260051.1 | 0.804145995 | 1.821444131 | 0.068539372 |
| LST1 | ENST00000563611.1 | ENSG00000261583.1 | 0.959367375 | 2.124103647 | 0.033661478 |
| LST1 | ENST00000564809.1 | ENSG00000261471.1 | 0.922111868 | 2.055614592 | 0.03981968 |
| LST1 | ENST00000565735.1 | ENSG00000261213.1 | -0.809893827 | -1.816805901 | 0.069246839 |

| | | | | | |
|---|---|---|---|---|---|
| LST1 | ENST00000571660.1 | ENSG00000262848.1 | 0.846439293 | 1.88917951 | 0.058867779 |
| LST1 | ENST00000577853.1 | ENSG00000264207.1 | 0.847924884 | 1.877996495 | 0.060381645 |
| LST1 | ENST00000578800.1 | ENSG00000264235.1 | 0.913551959 | 2.053806034 | 0.039994469 |
| LST1 | ENST00000579775.1 | ENSG00000264108.1 | 0.853729255 | 1.901364213 | 0.057254324 |
| LST1 | ENST00000580622.1 | ENSG00000264634.1 | 0.936280676 | 2.107851352 | 0.035043849 |
| LST1 | ENST00000582044.1 | ENSG00000263715.2 | 0.991437643 | 2.224284182 | 0.02612933 |
| LST1 | ENST00000582558.1 | ENSG00000264569.1 | 0.864036758 | 1.928315141 | 0.05381594 |
| LST1 | ENST00000586694.1 | ENSG00000267141.1 | 0.809053672 | 1.797863012 | 0.072198719 |
| LST1 | ENST00000588182.2 | ENSG00000267453.2 | 0.859362207 | 1.89748589 | 0.057763839 |
| LST1 | ENST00000588380.1 | ENSG00000266990.1 | 0.897951636 | 2.012528641 | 0.044164241 |
| LST1 | ENST00000588402.1 | ENSG00000267006.1 | -0.840360537 | -1.863161651 | 0.062439527 |
| LST1 | ENST00000589380.1 | ENSG00000267488.1 | 0.843254532 | 1.870067125 | 0.061474497 |
| LST1 | ENST00000589395.1 | ENSG00000267143.1 | 0.855689156 | 1.915285685 | 0.05545608 |
| LST1 | ENST00000591174.1 | ENSG00000267289.1 | 0.843441364 | 1.861278317 | 0.062704884 |
| LST1 | ENST00000592498.1 | ENSG00000267488.1 | 0.839928899 | 1.864839659 | 0.062203883 |
| LST1 | ENST00000592525.1 | ENSG00000267214.1 | 0.947919439 | 2.122637845 | 0.033784211 |
| LST1 | ENST00000593139.1 | ENSG00000267042.1 | 0.945706019 | 2.130165116 | 0.033157984 |
| LST1 | ENST00000594776.1 | ENSG00000269807.1 | 0.830505668 | 1.856292475 | 0.063411881 |
| LST1 | ENST00000594850.1 | ENSG00000268093.1 | 0.907187001 | 2.039283241 | 0.041421767 |
| LST1 | ENST00000596091.1 | ENSG00000227733.4 | -0.865506145 | -1.934238211 | 0.053083846 |
| LST1 | ENST00000596887.1 | ENSG00000237031.3 | -0.924263674 | -2.072881284 | 0.038183329 |
| LST1 | ENST00000596971.1 | ENSG00000269463.1 | 0.831606473 | 1.871255301 | 0.061309703 |
| LST1 | ENST00000597169.1 | ENSG00000269720.1 | 0.843826143 | 1.872258087 | 0.061170906 |
| LST1 | ENST00000597256.1 | ENSG00000267986.1 | 0.947920844 | 2.118502775 | 0.03413251 |
| LST1 | ENST00000598092.1 | ENSG00000228065.6 | -0.824476398 | -1.866298673 | 0.06199959 |
| LST1 | ENST00000600242.1 | ENSG00000269583.1 | 0.923347408 | 2.065820965 | 0.038845388 |
| LST1 | ENST00000600726.1 | ENSG00000267858.1 | 0.87621378 | 1.947069125 | 0.051526448 |
| LST1 | ENST00000601033.1 | ENSG00000268401.1 | 0.829147503 | 1.867079889 | 0.061890432 |
| LST1 | ENST00000604142.1 | ENSG00000271308.1 | 0.947656125 | 2.138138919 | 0.03250547 |
| LST1 | ENST00000604183.1 | ENSG00000271185.1 | 0.955069491 | 2.106664611 | 0.035146662 |
| LST1 | ENST00000606277.1 | ENSG00000272145.1 | 0.870851307 | 1.942189047 | 0.052114219 |
| LST1 | ENST00000606441.1 | ENSG00000272277.1 | 0.888369758 | 1.981036496 | 0.047587181 |
| LST1 | ENST00000606743.1 | ENSG00000272221.1 | 0.846056541 | 1.88462697 | 0.059480218 |
| LST1 | ENST00000606909.1 | ENSG00000271821.1 | 0.930185669 | 2.073531581 | 0.038122835 |
| LST1 | ENST00000607224.1 | ENSG00000272521.1 | 0.862060959 | 1.916591624 | 0.055289833 |
| LST1 | ENST00000607476.1 | ENSG00000272540.1 | 0.967614906 | 2.166520403 | 0.030271442 |
| LST1 | ENST00000607943.1 | ENSG00000273188.1 | 0.940639492 | 2.138666165 | 0.032462715 |
| LST1 | ENST00000608367.1 | ENSG00000273361.1 | 0.886429687 | 1.984031603 | 0.047252317 |
| LST1 | ENST00000608489.1 | ENSG00000272716.1 | 0.8392571 | 1.886888691 | 0.059175298 |
| LST1 | ENST00000608677.1 | ENSG00000273350.1 | 0.907037831 | 2.032697167 | 0.042083128 |
| LST1 | NR_026802.1 | FAM74A4 | 0.904555745 | 2.033386775 | 0.042013462 |
| LST1 | NR_026813.1 | LINC00597 | -0.897687721 | -2.028002786 | 0.042559964 |
| LST1 | NR_026951.1 | LINC00324 | 0.887981289 | 1.970188825 | 0.048816734 |
| LST1 | NR_027052.1 | THAP7-AS1 | 0.929923789 | 2.093251767 | 0.036326686 |
| LST1 | NR_027271.1 | CIRBP-AS1 | 0.857136315 | 1.9496446 | 0.051218494 |
| LST1 | NR_037169.1 | LOC100507547 | 0.903855486 | 2.015486507 | 0.043853698 |
| LST1 | NR_037170.1 | LOC100507547 | 0.869280901 | 1.976219657 | 0.048129904 |
| LST1 | NR_038421.1 | LINC01220 | 0.921904341 | 2.054940718 | 0.039884731 |
| LST1 | NR_038923.1 | SSSCA1-AS1 | 0.968962464 | 2.178831105 | 0.029344217 |
| LST1 | NR_045114.1 | PVRL3-AS1 | -0.908391626 | -2.0473628 | 0.040622476 |
| LST1 | NR_046871.1 | LINC00333 | -0.878942638 | -1.949385822 | 0.051249366 |

| | | | | | |
|---|---|---|---|---|---|
| LST1 | NR_047116.1 | HIF1A-AS1 | -0.892035793 | -2.00339139 | 0.045135295 |
| LST1 | NR_072981.1 | LINC00957 | 0.885154547 | 1.988353746 | 0.046772581 |
| LST1 | NR_072982.1 | LINC00957 | 0.880818966 | 1.977423777 | 0.047993749 |
| LST1 | NR_105010.1 | LINC01333 | 0.844001466 | 1.870927444 | 0.061355138 |
| LST1 | NR_108106.1 | LINC01135 | 0.881218177 | 1.947299287 | 0.051498864 |
| LST1 | NR_109831.1 | RASSF1-AS1 | 0.894328506 | 2.006787492 | 0.044772295 |
| LST1 | NR_109885.1 | RALY-AS1 | 0.905558522 | 2.018984778 | 0.043488802 |
| LST1 | NR_109886.1 | RALY-AS1 | 0.895410776 | 2.000415606 | 0.045455405 |
| LST1 | NR_110245.1 | LOC101929282 | -0.91085954 | -2.047168596 | 0.040641534 |
| LST1 | NR_110556.1 | LOC102724890 | -0.824042173 | -1.825444712 | 0.067933947 |
| LST1 | NR_110630.1 | LOC101927478 | 0.907710658 | 2.039381002 | 0.041412017 |
| LST1 | NR_110941.1 | MIR762HG | 0.960347227 | 2.139375219 | 0.032405292 |
| LST1 | NR_110998.1 | FAM74A4 | 0.904555745 | 2.036141048 | 0.041736194 |
| LST1 | NR_111951.1 | LINC00869 | 0.896508745 | 2.017427404 | 0.04365093 |
| LST1 | NR_111952.1 | LINC00869 | 0.897519185 | 1.995234244 | 0.046017338 |
| LST1 | NR_111953.1 | LINC00869 | 0.891756203 | 1.999502919 | 0.045553966 |
| LST1 | NR_125849.1 | LOC101928140 | -0.83430827 | -1.880261921 | 0.06007239 |
| LST1 | NR_125957.1 | LOC101928626 | -0.849070613 | -1.926579592 | 0.054032044 |
| LST1 | NR_126522.1 | EXOC3-AS1 | 0.908616054 | 2.022643641 | 0.043109901 |
| LST1 | NR_130143.1 | LOC104968399 | 0.897225613 | 2.00019804 | 0.045478883 |
| LST1 | NR_134520.1 | LOC727993 | 0.882800997 | 1.954075293 | 0.050692314 |
| LST1 | NR_135024.1 | LOC105369747 | 0.905872734 | 2.036412106 | 0.041708991 |
| LST1 | NR_135097.1 | LOC105369443 | -0.851804431 | -1.919589953 | 0.054909714 |
| LST1 | NR_135584.1 | LOC101927596 | 0.846406354 | 1.881812212 | 0.059861517 |
| LST1 | NR_136215.1 | VCAN-AS1 | -0.909433931 | -2.059711124 | 0.039426165 |
| LTB | ENST00000318291.4 | ENSG00000177406.4 | 0.871727817 | 1.938981717 | 0.052503564 |
| LTB | ENST00000416329.1 | ENSG00000233184.2 | 0.82848094 | 1.862864386 | 0.062481349 |
| LTB | ENST00000417260.1 | ENSG00000231734.4 | -0.871899247 | -1.950851318 | 0.051074735 |
| LTB | ENST00000419662.1 | ENSG00000228265.1 | 0.873787531 | 1.955238445 | 0.050554933 |
| LTB | ENST00000421020.1 | ENSG00000231407.1 | 0.810101546 | 1.783689341 | 0.074474159 |
| LTB | ENST00000421207.1 | ENSG00000231768.1 | 0.917396853 | 2.05542286 | 0.039838179 |
| LTB | ENST00000423428.1 | ENSG00000224048.1 | -0.878760059 | -1.963938682 | 0.049537203 |
| LTB | ENST00000425124.1 | ENSG00000232336.1 | 0.98163485 | 2.226619544 | 0.025972712 |
| LTB | ENST00000425624.1 | ENSG00000223779.4 | 0.901640802 | 2.030742699 | 0.042281102 |
| LTB | ENST00000426237.2 | ENSG00000235527.2 | 0.873213355 | 1.94774507 | 0.051445474 |
| LTB | ENST00000426699.1 | ENSG00000229308.1 | 0.85030923 | 1.891600385 | 0.058544245 |
| LTB | ENST00000433051.1 | ENSG00000233193.1 | 0.80238952 | 1.797773033 | 0.072212983 |
| LTB | ENST00000433344.1 | ENSG00000234083.1 | -0.846785643 | -1.910414619 | 0.05607985 |
| LTB | ENST00000435287.1 | ENSG00000227220.1 | 0.801452585 | 1.803553542 | 0.071301325 |
| LTB | ENST00000435434.1 | ENSG00000231233.1 | 0.84037055 | 1.877294246 | 0.060477776 |
| LTB | ENST00000435992.2 | ENSG00000232675.3 | 0.865179418 | 1.959902953 | 0.050007134 |
| LTB | ENST00000436982.2 | ENSG00000235335.2 | -0.809170028 | -1.802907455 | 0.07140275 |
| LTB | ENST00000438190.1 | ENSG00000227214.2 | 0.904885641 | 2.034196977 | 0.041931739 |
| LTB | ENST00000452176.1 | ENSG00000223659.1 | -0.900109815 | -2.011014281 | 0.044323948 |
| LTB | ENST00000454100.1 | ENSG00000236943.2 | 0.876118642 | 1.987807756 | 0.046832956 |
| LTB | ENST00000455788.1 | ENSG00000236263.1 | 0.869255431 | 1.93574542 | 0.052898889 |
| LTB | ENST00000458154.1 | ENSG00000235578.1 | 0.930053432 | 2.092259756 | 0.036415286 |
| LTB | ENST00000484413.1 | ENSG00000271853.1 | 0.852011122 | 1.90354063 | 0.056970037 |
| LTB | ENST00000489077.1 | ENSG00000244198.1 | 0.906127829 | 2.013296317 | 0.044083466 |
| LTB | ENST00000494509.1 | ENSG00000240095.1 | 0.885122606 | 1.985891441 | 0.047045379 |
| LTB | ENST00000498693.1 | ENSG00000244198.1 | 0.873832636 | 1.958620369 | 0.050157262 |

| | | | | | |
|---|---|---|---|---|---|
| LTB | ENST00000504578.1 | ENSG00000251513.1 | -0.80174753 | -1.791788219 | 0.073166897 |
| LTB | ENST00000506791.1 | ENSG00000251131.1 | 0.958477331 | 2.126065971 | 0.033497767 |
| LTB | ENST00000508083.1 | ENSG00000249343.1 | 0.855070905 | 1.880620027 | 0.060023626 |
| LTB | ENST00000509036.1 | ENSG00000251131.1 | 0.865078806 | 1.927835547 | 0.053875585 |
| LTB | ENST00000509192.1 | ENSG00000250765.1 | 0.979615397 | 2.190210314 | 0.028508988 |
| LTB | ENST00000509453.1 | ENSG00000249145.1 | 0.865393076 | 1.935070884 | 0.052981598 |
| LTB | ENST00000515128.1 | ENSG00000248215.1 | -0.875385602 | -1.966404644 | 0.049251887 |
| LTB | ENST00000518473.1 | ENSG00000253985.1 | 0.852638628 | 1.909507884 | 0.056196606 |
| LTB | ENST00000520603.1 | ENSG00000254001.1 | -0.956167012 | -2.118352961 | 0.034145186 |
| LTB | ENST00000521307.1 | ENSG00000253177.1 | 0.814207005 | 1.807849829 | 0.070629876 |
| LTB | ENST00000528887.1 | ENSG00000254501.1 | 0.800801005 | 1.784716882 | 0.074307251 |
| LTB | ENST00000547834.1 | ENSG00000258325.1 | 0.848692113 | 1.921253853 | 0.054699712 |
| LTB | ENST00000548722.2 | ENSG00000257194.2 | -0.828841815 | -1.864133254 | 0.062302994 |
| LTB | ENST00000550263.1 | ENSG00000257605.1 | 0.910029248 | 2.040844654 | 0.041266271 |
| LTB | ENST00000554859.1 | ENSG00000259088.1 | 0.818937333 | 1.837342588 | 0.066159337 |
| LTB | ENST00000556397.1 | ENSG00000258654.1 | 0.811404766 | 1.801722371 | 0.071589097 |
| LTB | ENST00000566170.1 | ENSG00000261071.1 | 0.816776643 | 1.836299847 | 0.066313322 |
| LTB | ENST00000567395.1 | ENSG00000261090.1 | 0.933445179 | 2.07480916 | 0.038004225 |
| LTB | ENST00000568033.1 | ENSG00000261480.1 | 0.835085038 | 1.865909382 | 0.062054045 |
| LTB | ENST00000569981.1 | ENSG00000238045.5 | 0.935834644 | 2.089249083 | 0.036685307 |
| LTB | ENST00000570493.2 | ENSG00000261898.2 | 0.943811842 | 2.119697522 | 0.034031562 |
| LTB | ENST00000570929.1 | ENSG00000262223.2 | 0.804620816 | 1.792347084 | 0.073077386 |
| LTB | ENST00000582558.1 | ENSG00000264569.1 | 0.949874126 | 2.103718009 | 0.035403053 |
| LTB | ENST00000584705.1 | ENSG00000264569.1 | 0.937907745 | 2.115206729 | 0.034412332 |
| LTB | ENST00000585559.1 | ENSG00000267117.1 | 0.96295964 | 2.162722966 | 0.030562494 |
| LTB | ENST00000586694.1 | ENSG00000267141.1 | 0.836424061 | 1.873332556 | 0.061022477 |
| LTB | ENST00000588380.1 | ENSG00000266990.1 | 0.852856302 | 1.910311894 | 0.056093067 |
| LTB | ENST00000588945.1 | ENSG00000267275.1 | 0.836520461 | 1.899292558 | 0.057526021 |
| LTB | ENST00000591174.1 | ENSG00000267289.1 | 0.811004056 | 1.81609357 | 0.06935602 |
| LTB | ENST00000592400.1 | ENSG00000267735.1 | 0.944713612 | 2.119037546 | 0.034087294 |
| LTB | ENST00000592720.1 | ENSG00000267232.1 | 0.858679041 | 1.91189097 | 0.05589018 |
| LTB | ENST00000595478.1 | ENSG00000237031.3 | -0.889282484 | -1.969515737 | 0.048893897 |
| LTB | ENST00000596091.1 | ENSG00000227733.4 | -0.836397046 | -1.870701299 | 0.061386495 |
| LTB | ENST00000597169.1 | ENSG00000269720.1 | 0.913125339 | 2.051258975 | 0.040241733 |
| LTB | ENST00000599259.1 | ENSG00000269352.1 | 0.8617394 | 1.944532989 | 0.051831211 |
| LTB | ENST00000600726.1 | ENSG00000267858.1 | 0.839421598 | 1.894061895 | 0.058216795 |
| LTB | ENST00000601692.1 | ENSG00000267874.1 | -0.92939235 | -2.101648372 | 0.035584089 |
| LTB | ENST00000601735.1 | ENSG00000244513.2 | 0.891022699 | 1.993071244 | 0.046253646 |
| LTB | ENST00000602594.1 | ENSG00000269930.1 | -0.899608859 | -2.000034133 | 0.045496578 |
| LTB | ENST00000602809.1 | ENSG00000270105.1 | -0.807143364 | -1.830913595 | 0.067113441 |
| LTB | ENST00000602949.1 | ENSG00000270030.1 | 0.896280775 | 2.009152138 | 0.044521002 |
| LTB | ENST00000604142.1 | ENSG00000271308.1 | 0.916741358 | 2.054679212 | 0.039909999 |
| LTB | ENST00000605082.1 | ENSG00000270426.1 | 0.864759562 | 1.912432484 | 0.055820745 |
| LTB | ENST00000606277.1 | ENSG00000272145.1 | 0.836459804 | 1.883841661 | 0.059586396 |
| LTB | ENST00000606377.1 | ENSG00000272286.1 | -0.923724967 | -2.075757547 | 0.037916381 |
| LTB | ENST00000606470.1 | ENSG00000271913.1 | 0.930881771 | 2.079654731 | 0.037557213 |
| LTB | ENST00000606743.1 | ENSG00000272221.1 | 0.934684949 | 2.07712512 | 0.037790013 |
| LTB | ENST00000606909.1 | ENSG00000271821.1 | 0.861676909 | 1.907959624 | 0.056396435 |
| LTB | ENST00000607284.1 | ENSG00000272389.1 | 0.836181822 | 1.855235765 | 0.063562566 |
| LTB | ENST00000607476.1 | ENSG00000272540.1 | 0.867553733 | 1.945870202 | 0.051670333 |
| LTB | ENST00000607943.1 | ENSG00000273188.1 | 0.872530589 | 1.925172386 | 0.054207795 |

| | | | | | |
|---|---|---|---|---|---|
| LTB | ENST00000609972.1 | ENSG00000230651.3 | 0.855602789 | 1.912874815 | 0.05576408 |
| LTB | NR_003604.2 | ZFAS1 | 0.808296567 | 1.774183336 | 0.07603283 |
| LTB | NR_003605.1 | ZFAS1 | 0.921104528 | 2.08295624 | 0.037255213 |
| LTB | NR_003606.2 | ZFAS1 | 0.806092228 | 1.761120319 | 0.078218041 |
| LTB | NR_026802.1 | FAM74A4 | 0.81738249 | 1.835203875 | 0.066475487 |
| LTB | NR_027271.1 | CIRBP-AS1 | 0.827109544 | 1.876314517 | 0.060612105 |
| LTB | NR_028324.1 | LINC01002 | 0.917022399 | 2.047571032 | 0.04060205 |
| LTB | NR_036480.1 | VPS9D1-AS1 | 0.830819745 | 1.8530004 | 0.063882301 |
| LTB | NR_036658.1 | ZFAS1 | 0.816407051 | 1.835748979 | 0.06639479 |
| LTB | NR_037169.1 | LOC100507547 | 0.949526345 | 2.122844691 | 0.033766868 |
| LTB | NR_037170.1 | LOC100507547 | 0.941672671 | 2.112826653 | 0.034615608 |
| LTB | NR_044996.1 | HCG23 | 0.87698597 | 1.9475689 | 0.051466568 |
| LTB | NR_045114.1 | PVRL3-AS1 | -0.891538497 | -1.991585083 | 0.046416601 |
| LTB | NR_072981.1 | LINC00957 | 0.978753938 | 2.178147881 | 0.029395029 |
| LTB | NR_072982.1 | LINC00957 | 0.980982204 | 2.20252736 | 0.027628079 |
| LTB | NR_105010.1 | LINC01333 | 0.904927316 | 2.0395037 | 0.041399782 |
| LTB | NR_108036.1 | CFAP58-AS1 | 0.841819585 | 1.885343492 | 0.059383478 |
| LTB | NR_109885.1 | RALY-AS1 | 0.831978018 | 1.855966182 | 0.063458378 |
| LTB | NR_109886.1 | RALY-AS1 | 0.898253285 | 2.004262763 | 0.045041921 |
| LTB | NR_110998.1 | FAM74A4 | 0.81738249 | 1.813144391 | 0.069809554 |
| LTB | NR_120335.1 | LOC101928414 | 0.845294951 | 1.866295112 | 0.062000088 |
| LTB | NR_121189.1 | PGM5P3-AS1 | -0.810789021 | -1.803528967 | 0.07130518 |
| LTB | NR_125957.1 | LOC101928626 | -0.900109815 | -1.97807639 | 0.04792009 |
| LTB | NR_126166.1 | FAM74A7 | 0.878882537 | 1.978984535 | 0.047817748 |
| LTB | NR_126522.1 | EXOC3-AS1 | 0.802268383 | 1.771032876 | 0.076555235 |
| LTB | NR_134252.1 | LOC105379030 | 0.802899803 | 1.78835616 | 0.073718565 |
| LTB | NR_134325.1 | LOC102723672 | 0.811369152 | 1.819576308 | 0.068823552 |
| LTB | NR_138084.1 | HCG24 | 0.835134082 | 1.878758059 | 0.060277536 |
| LTB | NR_144459.1 | ARSD-AS1 | 0.887544768 | 1.975039202 | 0.048263699 |
| LUM | ENST00000411694.1 | ENSG00000225331.1 | -0.806778959 | -1.818172366 | 0.069037792 |
| LUM | ENST00000415205.1 | ENSG00000182057.4 | -0.845801177 | -1.883147327 | 0.059680405 |
| LUM | ENST00000417260.1 | ENSG00000231734.4 | 0.897018351 | 2.02778825 | 0.042581864 |
| LUM | ENST00000418387.1 | ENSG00000235056.1 | 0.845568413 | 1.894684015 | 0.058134277 |
| LUM | ENST00000425881.1 | ENSG00000239636.1 | -0.814496512 | -1.804911987 | 0.071088456 |
| LUM | ENST00000426030.2 | ENSG00000228686.2 | 0.987326577 | 2.208359918 | 0.027219194 |
| LUM | ENST00000427132.1 | ENSG00000232121.1 | -0.823457638 | -1.838036251 | 0.066057064 |
| LUM | ENST00000431730.1 | ENSG00000237401.2 | -0.98251331 | -2.211667168 | 0.026989671 |
| LUM | ENST00000433035.1 | ENSG00000230483.1 | -0.952341312 | -2.151610498 | 0.031428045 |
| LUM | ENST00000434250.1 | ENSG00000234055.1 | -0.829250856 | -1.875625392 | 0.060706737 |
| LUM | ENST00000437334.1 | ENSG00000226134.1 | -0.860327008 | -1.949311513 | 0.051258234 |
| LUM | ENST00000439443.1 | ENSG00000236911.2 | -0.886075018 | -1.969538777 | 0.048891254 |
| LUM | ENST00000442829.1 | ENSG00000225284.1 | -0.849924241 | -1.900640872 | 0.057349068 |
| LUM | ENST00000446562.1 | ENSG00000233896.1 | -0.897410223 | -2.000204561 | 0.04547818 |
| LUM | ENST00000446816.1 | ENSG00000204685.5 | -0.955035678 | -2.144636751 | 0.031981902 |
| LUM | ENST00000448748.1 | ENSG00000231238.1 | 0.838367791 | 1.863618496 | 0.062375298 |
| LUM | ENST00000451507.1 | ENSG00000229539.1 | -0.892291614 | -2.001240655 | 0.045366462 |
| LUM | ENST00000452176.1 | ENSG00000223659.1 | 0.835123153 | 1.860808764 | 0.062771187 |
| LUM | ENST00000452511.1 | ENSG00000231876.3 | 0.830078907 | 1.866273846 | 0.062003062 |
| LUM | ENST00000454182.1 | ENSG00000230379.1 | 0.851129339 | 1.927346905 | 0.053936412 |
| LUM | ENST00000454957.1 | ENSG00000224899.1 | 0.937094489 | 2.123318607 | 0.033727162 |
| LUM | ENST00000457371.1 | ENSG00000237401.2 | -0.97647975 | -2.179400079 | 0.02930196 |

| | | | | | |
|---|---|---|---|---|---|
| LUM | ENST00000463255.1 | ENSG00000243305.1 | 0.833865916 | 1.857059796 | 0.063302647 |
| LUM | ENST00000479233.1 | ENSG00000243150.1 | 0.855610807 | 1.934529261 | 0.053048088 |
| LUM | ENST00000485338.1 | ENSG00000239641.1 | 0.836249585 | 1.868522399 | 0.061689291 |
| LUM | ENST00000488310.1 | ENSG00000240449.1 | -0.944944267 | -2.093279478 | 0.036324213 |
| LUM | ENST00000489090.1 | ENSG00000240045.1 | 0.948881854 | 2.124620734 | 0.033618272 |
| LUM | ENST00000489690.1 | ENSG00000243944.1 | 0.948151945 | 2.153876133 | 0.031249887 |
| LUM | ENST00000500496.2 | ENSG00000245479.2 | -0.841490501 | -1.876538075 | 0.060581432 |
| LUM | ENST00000502467.1 | ENSG00000250530.1 | 0.824140922 | 1.838412331 | 0.066001669 |
| LUM | ENST00000507525.1 | ENSG00000250431.1 | 0.813202949 | 1.807684965 | 0.070655546 |
| LUM | ENST00000509453.1 | ENSG00000249145.1 | -0.817832308 | -1.806297199 | 0.070871929 |
| LUM | ENST00000513836.1 | ENSG00000251266.1 | 0.946943292 | 2.12122667 | 0.033902732 |
| LUM | ENST00000521359.1 | ENSG00000253140.1 | 0.879218606 | 1.977824582 | 0.047948499 |
| LUM | ENST00000521653.1 | ENSG00000253301.1 | -0.836708398 | -1.873381224 | 0.061015761 |
| LUM | ENST00000525855.1 | ENSG00000254746.1 | -0.854392766 | -1.940407168 | 0.052330226 |
| LUM | ENST00000529247.1 | ENSG00000254741.1 | -0.851082037 | -1.897150164 | 0.057808122 |
| LUM | ENST00000532454.1 | ENSG00000255120.1 | -0.933029592 | -2.101326253 | 0.035612337 |
| LUM | ENST00000534178.1 | ENSG00000255120.1 | -0.976670506 | -2.182923196 | 0.029041466 |
| LUM | ENST00000537032.1 | ENSG00000255933.1 | -0.879028057 | -1.97785369 | 0.047945215 |
| LUM | ENST00000543403.1 | ENSG00000256684.1 | 0.941864142 | 2.082483182 | 0.037298358 |
| LUM | ENST00000545158.1 | ENSG00000256011.1 | 0.860573994 | 1.89832521 | 0.057653255 |
| LUM | ENST00000545572.1 | ENSG00000255680.1 | -0.85716219 | -1.92670035 | 0.054016984 |
| LUM | ENST00000550263.1 | ENSG00000257605.1 | -0.872389401 | -1.952204991 | 0.050913872 |
| LUM | ENST00000554431.1 | ENSG00000258616.1 | 0.973798644 | 2.174662743 | 0.029655399 |
| LUM | ENST00000557602.1 | ENSG00000258616.1 | 0.974189811 | 2.140589074 | 0.032307191 |
| LUM | ENST00000558237.1 | ENSG00000259684.1 | 0.823269453 | 1.836782171 | 0.066242059 |
| LUM | ENST00000558475.1 | ENSG00000259604.1 | -0.841681301 | -1.878652585 | 0.060291946 |
| LUM | ENST00000559673.1 | ENSG00000259604.1 | -0.841478108 | -1.882663673 | 0.059745961 |
| LUM | ENST00000560963.1 | ENSG00000259370.1 | -0.86018526 | -1.935949444 | 0.052873893 |
| LUM | ENST00000562191.1 | ENSG00000261292.1 | 0.947334142 | 2.098068582 | 0.035899087 |
| LUM | ENST00000562995.1 | ENSG00000261253.1 | -0.990484529 | -2.222881384 | 0.026223799 |
| LUM | ENST00000563610.1 | ENSG00000260051.1 | -0.809643837 | -1.805592832 | 0.070981963 |
| LUM | ENST00000564809.1 | ENSG00000261471.1 | -0.80435041 | -1.80380755 | 0.071261482 |
| LUM | ENST00000569215.1 | ENSG00000260756.1 | 0.838404845 | 1.884726134 | 0.059466822 |
| LUM | ENST00000570413.1 | ENSG00000263167.1 | -0.825594863 | -1.866413518 | 0.061983533 |
| LUM | ENST00000570700.1 | ENSG00000263011.1 | -0.839538381 | -1.871564711 | 0.061266849 |
| LUM | ENST00000571660.1 | ENSG00000262848.1 | -0.84113419 | -1.871964437 | 0.061211524 |
| LUM | ENST00000572608.1 | ENSG00000263305.1 | 0.842158713 | 1.876762246 | 0.060550687 |
| LUM | ENST00000576271.1 | ENSG00000263342.1 | -0.894119211 | -2.012658019 | 0.044150619 |
| LUM | ENST00000577853.1 | ENSG00000264207.1 | -0.804284416 | -1.802056804 | 0.071536469 |
| LUM | ENST00000580311.1 | ENSG00000266803.1 | 0.970237008 | 2.148324141 | 0.031688014 |
| LUM | ENST00000585761.1 | ENSG00000267198.1 | -0.824332542 | -1.858797925 | 0.063055787 |
| LUM | ENST00000585810.1 | ENSG00000236172.2 | -0.826118022 | -1.836646187 | 0.066262144 |
| LUM | ENST00000586338.1 | ENSG00000219410.4 | -0.807867328 | -1.793506279 | 0.072892008 |
| LUM | ENST00000586348.1 | ENSG00000267198.1 | -0.852165619 | -1.930233622 | 0.053577897 |
| LUM | ENST00000587696.1 | ENSG00000225313.2 | -0.846950734 | -1.871342878 | 0.061297571 |
| LUM | ENST00000587702.1 | ENSG00000267378.1 | 0.803352681 | 1.800343304 | 0.071806447 |
| LUM | ENST00000587850.1 | ENSG00000267683.1 | -0.823161655 | -1.844586589 | 0.065097699 |
| LUM | ENST00000588380.1 | ENSG00000266990.1 | -0.865958597 | -1.937896213 | 0.052635886 |
| LUM | ENST00000589395.1 | ENSG00000267143.1 | -0.842516997 | -1.883878319 | 0.059581437 |
| LUM | ENST00000590989.1 | ENSG00000267011.1 | -0.844258966 | -1.885705487 | 0.059334653 |
| LUM | ENST00000591414.1 | ENSG00000267011.1 | -0.845944384 | -1.876384775 | 0.060602464 |

| | | | | | |
|---|---|---|---|---|---|
| LUM | ENST00000592816.1 | ENSG00000236172.2 | -0.827200821 | -1.839973985 | 0.065772057 |
| LUM | ENST00000593967.1 | ENSG00000232732.5 | -0.815235018 | -1.813496295 | 0.06975531 |
| LUM | ENST00000595007.1 | ENSG00000231876.3 | 0.853936866 | 1.904365159 | 0.056862643 |
| LUM | ENST00000596091.1 | ENSG00000227733.4 | 0.824271278 | 1.882536762 | 0.059763173 |
| LUM | ENST00000597420.1 | ENSG00000269564.1 | -0.866407992 | -1.93766397 | 0.052664232 |
| LUM | ENST00000598887.1 | ENSG00000268475.1 | -0.834327918 | -1.851229147 | 0.064136594 |
| LUM | ENST00000599387.1 | ENSG00000227733.4 | 0.813612143 | 1.82374049 | 0.068191314 |
| LUM | ENST00000600848.1 | ENSG00000228065.6 | 0.841190889 | 1.892858703 | 0.058376662 |
| LUM | ENST00000602405.1 | ENSG00000269928.1 | -0.861419153 | -1.899388109 | 0.057513466 |
| LUM | ENST00000603948.1 | ENSG00000222041.6 | -0.867425268 | -1.943595032 | 0.051944305 |
| LUM | ENST00000604183.1 | ENSG00000271185.1 | -0.833376455 | -1.864392218 | 0.062266645 |
| LUM | ENST00000606441.1 | ENSG00000272277.1 | -0.834121806 | -1.837214715 | 0.066178204 |
| LUM | ENST00000606743.1 | ENSG00000272221.1 | -0.806502015 | -1.791354201 | 0.073236473 |
| LUM | ENST00000606909.1 | ENSG00000271821.1 | -0.813873199 | -1.832914016 | 0.066815359 |
| LUM | ENST00000606942.1 | ENSG00000271835.1 | 0.826580626 | 1.844131425 | 0.065163989 |
| LUM | ENST00000607148.1 | ENSG00000272477.1 | 0.833726171 | 1.861662458 | 0.062650683 |
| LUM | ENST00000608159.1 | ENSG00000273093.1 | 0.894213836 | 1.999067104 | 0.045601094 |
| LUM | ENST00000608258.1 | ENSG00000229042.2 | 0.954689536 | 2.096377173 | 0.036048746 |
| LUM | ENST00000608367.1 | ENSG00000273361.1 | -0.89379887 | -2.010419121 | 0.044386848 |
| LUM | ENST00000609182.1 | ENSG00000273248.1 | -0.806122432 | -1.813338801 | 0.069779582 |
| LUM | ENST00000609813.1 | ENSG00000272719.1 | -0.868326954 | -1.945459054 | 0.051719753 |
| LUM | ENST00000609955.1 | ENSG00000273275.1 | 0.879241754 | 1.984549245 | 0.047194644 |
| LUM | ENST00000610008.1 | ENSG00000272711.1 | -0.900619931 | -2.026372804 | 0.042726596 |
| LUM | NR_028324.1 | LINC01002 | -0.815912026 | -1.822640764 | 0.068357817 |
| LUM | NR_034131.1 | LINC00272 | -0.827969164 | -1.869307623 | 0.061580028 |
| LUM | NR_037170.1 | LOC100507547 | -0.813752677 | -1.828585422 | 0.067461738 |
| LUM | NR_040047.1 | SDCBP2-AS1 | -0.901362587 | -1.991337687 | 0.046443775 |
| LUM | NR_040049.1 | SDCBP2-AS1 | -0.827299897 | -1.825451882 | 0.067932866 |
| LUM | NR_047498.1 | LINC00853 | -0.97129565 | -2.178881749 | 0.029340454 |
| LUM | NR_073155.1 | C1orf145 | 0.930324997 | 2.061206565 | 0.039283337 |
| LUM | NR_108085.1 | OVOL1-AS1 | -0.916171943 | -2.064643127 | 0.038956779 |
| LUM | NR_110630.1 | LOC101927478 | -0.820817888 | -1.818571377 | 0.068976848 |
| LUM | NR_120371.1 | LINC01585 | -0.829571765 | -1.861194214 | 0.062716755 |
| LUM | NR_125957.1 | LOC101928626 | 0.835123153 | 1.875349324 | 0.060744682 |
| LUM | NR_131243.1 | SMCR2 | -0.829806517 | -1.844086038 | 0.065170602 |
| LUM | NR_133941.1 | LOC105377247 | 0.946943292 | 2.128533746 | 0.033292855 |
| LUM | NR_134520.1 | LOC727993 | -0.894256951 | -2.013851432 | 0.044025134 |
| LUM | NR_135097.1 | LOC105369443 | 0.941864142 | 2.109531445 | 0.034898735 |
| LUM | NR_135626.1 | LOC100505585 | -0.944729821 | -2.107221038 | 0.035098424 |
| LUM | NR_135816.1 | LOC100996664 | -0.802679061 | -1.809318089 | 0.0704016 |
| LUM | NR_144459.1 | ARSD-AS1 | -0.813327458 | -1.817251319 | 0.06917864 |
| MGST2 | ENST00000411824.1 | ENSG00000232803.1 | 0.907854359 | 2.046644841 | 0.040692969 |
| MGST2 | ENST00000412896.1 | ENSG00000197585.5 | 0.889088643 | 1.965698643 | 0.049333432 |
| MGST2 | ENST00000413564.1 | ENSG00000224500.1 | 0.899987077 | 2.014756704 | 0.043930148 |
| MGST2 | ENST00000419207.2 | ENSG00000231248.2 | 0.801839171 | 1.79488013 | 0.0726728 |
| MGST2 | ENST00000419863.1 | ENSG00000238282.1 | 0.885538084 | 1.972231677 | 0.048583165 |
| MGST2 | ENST00000420498.1 | ENSG00000224985.1 | -0.86807395 | -1.946877509 | 0.051549422 |
| MGST2 | ENST00000420572.2 | ENSG00000233358.2 | -0.941059347 | -2.108573429 | 0.034981418 |
| MGST2 | ENST00000421617.1 | ENSG00000237342.1 | -0.849020677 | -1.877345222 | 0.060470794 |
| MGST2 | ENST00000421866.1 | ENSG00000233875.1 | 0.851302218 | 1.900231021 | 0.057402809 |
| MGST2 | ENST00000422038.1 | ENSG00000227935.1 | -0.801846529 | -1.790258646 | 0.073412341 |

| | | | | | |
|---|---|---|---|---|---|
| MGST2 | ENST00000422118.1 | ENSG00000231189.1 | 0.862720985 | 1.92860549 | 0.053779857 |
| MGST2 | ENST00000422204.1 | ENSG00000238160.1 | 0.806062179 | 1.796482108 | 0.072417876 |
| MGST2 | ENST00000423925.1 | ENSG00000223536.1 | 0.863033318 | 1.906187807 | 0.056625844 |
| MGST2 | ENST00000425371.2 | ENSG00000235872.2 | -0.823863468 | -1.830296611 | 0.067205598 |
| MGST2 | ENST00000426504.1 | ENSG00000234190.1 | 0.808206601 | 1.782891442 | 0.074603976 |
| MGST2 | ENST00000427691.1 | ENSG00000228340.1 | 0.909405679 | 2.046541469 | 0.040703127 |
| MGST2 | ENST00000428440.1 | ENSG00000232827.2 | 0.846303099 | 1.886967605 | 0.059164683 |
| MGST2 | ENST00000429608.1 | ENSG00000237480.1 | -0.87657015 | -1.914910652 | 0.055503899 |
| MGST2 | ENST00000429796.1 | ENSG00000231858.1 | 0.805776297 | 1.812507351 | 0.069907839 |
| MGST2 | ENST00000430545.1 | ENSG00000237153.1 | -0.82574439 | -1.858737489 | 0.063064357 |
| MGST2 | ENST00000431290.1 | ENSG00000183822.2 | -0.96667345 | -2.182391533 | 0.029080649 |
| MGST2 | ENST00000432244.1 | ENSG00000234265.1 | -0.92510986 | -2.050744463 | 0.040291839 |
| MGST2 | ENST00000434627.1 | ENSG00000230074.1 | -0.800439871 | -1.799527656 | 0.071935253 |
| MGST2 | ENST00000435984.1 | ENSG00000204792.2 | -0.961862056 | -2.130941411 | 0.033093969 |
| MGST2 | ENST00000436582.1 | ENSG00000236525.1 | 0.823270021 | 1.825310506 | 0.067954186 |
| MGST2 | ENST00000437330.1 | ENSG00000229203.1 | -0.876216674 | -1.961336914 | 0.049839734 |
| MGST2 | ENST00000437461.1 | ENSG00000227200.1 | -0.952503969 | -2.123797835 | 0.033687052 |
| MGST2 | ENST00000441532.1 | ENSG00000234206.1 | 0.904584093 | 2.030126273 | 0.042343706 |
| MGST2 | ENST00000442850.1 | ENSG00000232600.2 | 0.85526994 | 1.906619634 | 0.056569861 |
| MGST2 | ENST00000443380.1 | ENSG00000224371.1 | -0.934904041 | -2.100993748 | 0.035641515 |
| MGST2 | ENST00000444731.1 | ENSG00000227131.1 | 0.838755963 | 1.878313921 | 0.060338233 |
| MGST2 | ENST00000445260.2 | ENSG00000231429.2 | -0.918598042 | -2.07450267 | 0.038032651 |
| MGST2 | ENST00000448365.1 | ENSG00000231114.1 | -0.826206176 | -1.859234777 | 0.062993867 |
| MGST2 | ENST00000448650.1 | ENSG00000223536.1 | 0.878099455 | 1.990926218 | 0.046488999 |
| MGST2 | ENST00000448942.1 | ENSG00000237499.2 | -0.883350433 | -1.988298742 | 0.04677866 |
| MGST2 | ENST00000449586.1 | ENSG00000235257.4 | 0.920954211 | 2.071934157 | 0.038271582 |
| MGST2 | ENST00000450109.1 | ENSG00000225376.1 | -0.93393309 | -2.063093302 | 0.039103764 |
| MGST2 | ENST00000450531.1 | ENSG00000229536.1 | -0.84211059 | -1.857821461 | 0.063194372 |
| MGST2 | ENST00000451648.1 | ENSG00000232803.1 | 0.869224706 | 1.951606592 | 0.05098493 |
| MGST2 | ENST00000452002.1 | ENSG00000236501.1 | 0.934733699 | 2.08803523 | 0.036794657 |
| MGST2 | ENST00000454387.1 | ENSG00000223726.1 | 0.823624269 | 1.856151902 | 0.06343191 |
| MGST2 | ENST00000454530.1 | ENSG00000226649.1 | 0.824032311 | 1.864715572 | 0.062221283 |
| MGST2 | ENST00000454709.1 | ENSG00000237280.1 | 0.865422021 | 1.932992155 | 0.053237164 |
| MGST2 | ENST00000455010.1 | ENSG00000233079.1 | -0.840691843 | -1.881933093 | 0.0598451 |
| MGST2 | ENST00000457848.1 | ENSG00000226412.1 | -0.898088475 | -1.999114925 | 0.045595921 |
| MGST2 | ENST00000458082.1 | ENSG00000231210.2 | 0.812172243 | 1.799829352 | 0.071887588 |
| MGST2 | ENST00000458107.3 | ENSG00000248478.2 | -0.866935046 | -1.961222814 | 0.049853037 |
| MGST2 | ENST00000458443.1 | ENSG00000238232.1 | -0.837842408 | -1.864724275 | 0.062220062 |
| MGST2 | ENST00000460993.1 | ENSG00000241231.1 | -0.956015264 | -2.149132281 | 0.031623915 |
| MGST2 | ENST00000476099.1 | ENSG00000244158.1 | 0.867733248 | 1.958809454 | 0.050135106 |
| MGST2 | ENST00000477643.1 | ENSG00000241224.2 | 0.808131639 | 1.803795693 | 0.071263341 |
| MGST2 | ENST00000487368.1 | ENSG00000273328.1 | 0.880335219 | 1.963511861 | 0.049586728 |
| MGST2 | ENST00000502300.1 | ENSG00000249451.1 | -0.848616492 | -1.865781748 | 0.062071907 |
| MGST2 | ENST00000503034.1 | ENSG00000248936.1 | -0.842174296 | -1.891046058 | 0.058618196 |
| MGST2 | ENST00000505196.1 | ENSG00000248131.1 | -0.848063241 | -1.920545644 | 0.054789014 |
| MGST2 | ENST00000507558.1 | ENSG00000248445.1 | -0.925017543 | -2.041166723 | 0.041234258 |
| MGST2 | ENST00000507808.1 | ENSG00000250333.1 | 0.865386255 | 1.906220937 | 0.056621547 |
| MGST2 | ENST00000508188.1 | ENSG00000250999.1 | -0.830293564 | -1.871898536 | 0.061220642 |
| MGST2 | ENST00000508199.1 | ENSG00000247810.2 | -0.854618855 | -1.926071944 | 0.054095391 |
| MGST2 | ENST00000510602.1 | ENSG00000249122.1 | 0.864843853 | 1.935507929 | 0.052927997 |
| MGST2 | ENST00000511234.1 | ENSG00000250865.1 | 0.808162703 | 1.811772606 | 0.07002134 |

| | | | | | |
|---|---|---|---|---|---|
| MGST2 | ENST00000512300.1 | ENSG00000248362.1 | -0.867640213 | -1.948808247 | 0.051318329 |
| MGST2 | ENST00000515077.1 | ENSG00000251206.1 | -0.917622781 | -2.075534072 | 0.037937064 |
| MGST2 | ENST00000519038.2 | ENSG00000254054.2 | 0.90439358 | 2.03857813 | 0.04149215 |
| MGST2 | ENST00000519189.1 | ENSG00000254344.1 | 0.857923518 | 1.915304592 | 0.05545367 |
| MGST2 | ENST00000519368.1 | ENSG00000253215.1 | -0.885488852 | -2.004855568 | 0.04497849 |
| MGST2 | ENST00000519451.1 | ENSG00000253363.1 | -0.896860524 | -2.015285942 | 0.043874697 |
| MGST2 | ENST00000519852.1 | ENSG00000253716.1 | -0.906607818 | -2.048991547 | 0.040462941 |
| MGST2 | ENST00000520411.1 | ENSG00000253355.1 | 0.830033356 | 1.855900965 | 0.063467675 |
| MGST2 | ENST00000521207.1 | ENSG00000253716.1 | -0.916740879 | -2.067190393 | 0.038716217 |
| MGST2 | ENST00000521884.1 | ENSG00000253355.1 | 0.868998074 | 1.952721732 | 0.050852577 |
| MGST2 | ENST00000522524.1 | ENSG00000253342.1 | 0.857968842 | 1.91574023 | 0.055398169 |
| MGST2 | ENST00000523703.1 | ENSG00000214803.3 | 0.843283678 | 1.90112949 | 0.057285054 |
| MGST2 | ENST00000524073.1 | ENSG00000253774.1 | -0.818638333 | -1.831197778 | 0.067071028 |
| MGST2 | ENST00000524309.1 | ENSG00000240915.2 | 0.888074712 | 1.971084579 | 0.048714202 |
| MGST2 | ENST00000524335.1 | ENSG00000253716.1 | -0.918053247 | -2.046104467 | 0.040746094 |
| MGST2 | ENST00000528000.1 | ENSG00000254804.1 | -0.80487462 | -1.802597328 | 0.071451477 |
| MGST2 | ENST00000528818.1 | ENSG00000232995.3 | -0.800121623 | -1.782586061 | 0.074653711 |
| MGST2 | ENST00000528869.1 | ENSG00000255443.1 | -0.912105245 | -2.021675872 | 0.043209848 |
| MGST2 | ENST00000531071.1 | ENSG00000255248.2 | 0.915422985 | 2.038165389 | 0.041533396 |
| MGST2 | ENST00000531136.1 | ENSG00000255558.1 | 0.839211127 | 1.875266191 | 0.060756112 |
| MGST2 | ENST00000532947.1 | ENSG00000255322.1 | -0.837965066 | -1.88909312 | 0.058879352 |
| MGST2 | ENST00000533101.1 | ENSG00000255311.1 | -0.801143148 | -1.784459201 | 0.074349079 |
| MGST2 | ENST00000537269.1 | ENSG00000257084.1 | -0.826981891 | -1.870265218 | 0.061446997 |
| MGST2 | ENST00000546789.1 | ENSG00000257740.1 | -0.838985583 | -1.860862313 | 0.062763623 |
| MGST2 | ENST00000547750.1 | ENSG00000257886.1 | -0.900299278 | -2.009608431 | 0.044472648 |
| MGST2 | ENST00000548731.1 | ENSG00000257809.1 | -0.862198859 | -1.896134135 | 0.05794231 |
| MGST2 | ENST00000554049.1 | ENSG00000258763.1 | -0.934019638 | -2.070758915 | 0.038381331 |
| MGST2 | ENST00000554430.1 | ENSG00000258646.1 | 0.96686446 | 2.15422195 | 0.03122277 |
| MGST2 | ENST00000557412.1 | ENSG00000257621.3 | 0.903829746 | 2.029145933 | 0.042443429 |
| MGST2 | ENST00000558434.1 | ENSG00000259572.1 | 0.858549976 | 1.952851107 | 0.050837241 |
| MGST2 | ENST00000558515.1 | ENSG00000259182.1 | -0.807984899 | -1.824680321 | 0.068049285 |
| MGST2 | ENST00000558875.1 | ENSG00000259737.2 | -0.964850222 | -2.170929915 | 0.029936471 |
| MGST2 | ENST00000559569.1 | ENSG00000259760.1 | 0.802952469 | 1.787837637 | 0.073802207 |
| MGST2 | ENST00000561215.1 | ENSG00000259611.1 | -0.866992165 | -1.940674152 | 0.052297814 |
| MGST2 | ENST00000561529.1 | ENSG00000260886.1 | -0.902292476 | -2.007288285 | 0.044718976 |
| MGST2 | ENST00000563601.1 | ENSG00000260589.1 | 0.80420303 | 1.798033827 | 0.072171647 |
| MGST2 | ENST00000564102.1 | ENSG00000260041.1 | 0.887562835 | 1.989776667 | 0.046615543 |
| MGST2 | ENST00000565359.1 | ENSG00000260601.1 | -0.927970945 | -2.09082615 | 0.036543651 |
| MGST2 | ENST00000569459.1 | ENSG00000261346.1 | -0.804684545 | -1.796054871 | 0.072485791 |
| MGST2 | ENST00000569742.1 | ENSG00000260787.1 | -0.906545105 | -1.997256734 | 0.045797301 |
| MGST2 | ENST00000571815.1 | ENSG00000262810.1 | -0.914614864 | -2.049429707 | 0.040420115 |
| MGST2 | ENST00000573414.1 | ENSG00000263072.1 | -0.861403319 | -1.90938406 | 0.056212566 |
| MGST2 | ENST00000577064.1 | ENSG00000262823.1 | -0.800304654 | -1.801887876 | 0.071563048 |
| MGST2 | ENST00000583138.1 | ENSG00000263393.1 | -0.814010628 | -1.845726035 | 0.064931995 |
| MGST2 | ENST00000587049.1 | ENSG00000235535.3 | 0.855187004 | 1.899513885 | 0.057496943 |
| MGST2 | ENST00000589281.1 | ENSG00000267707.1 | 0.971606033 | 2.178362926 | 0.029379028 |
| MGST2 | ENST00000589457.1 | ENSG00000267751.1 | -0.811202875 | -1.815458379 | 0.069453497 |
| MGST2 | ENST00000590368.1 | ENSG00000231616.4 | -0.815606917 | -1.793021951 | 0.072969414 |
| MGST2 | ENST00000591103.1 | ENSG00000272895.1 | -0.910302314 | -2.044275898 | 0.040926299 |
| MGST2 | ENST00000591836.1 | ENSG00000267776.1 | -0.970762089 | -2.178131068 | 0.02939628 |
| MGST2 | ENST00000592622.1 | ENSG00000267546.2 | 0.816860187 | 1.840238772 | 0.06573319 |

| | | | | | |
|---|---|---|---|---|---|
| MGST2 | ENST00000593269.1 | ENSG00000236172.2 | -0.822720431 | -1.83829497 | 0.066018952 |
| MGST2 | ENST00000599572.1 | ENSG00000233783.3 | 0.818948339 | 1.7998506 | 0.071884232 |
| MGST2 | ENST00000600071.1 | ENSG00000269199.1 | -0.889488279 | -1.997388455 | 0.045783001 |
| MGST2 | ENST00000600512.1 | ENSG00000269752.1 | -0.860703195 | -1.940495111 | 0.052319548 |
| MGST2 | ENST00000602614.1 | ENSG00000269957.1 | 0.830085522 | 1.868946329 | 0.061630282 |
| MGST2 | ENST00000606068.1 | ENSG00000272342.1 | -0.923893432 | -2.049159975 | 0.040446474 |
| MGST2 | ENST00000606921.1 | ENSG00000272402.1 | 0.831897675 | 1.86142013 | 0.06268487 |
| MGST2 | ENST00000606938.1 | ENSG00000272198.1 | 0.905980951 | 2.015792571 | 0.043821671 |
| MGST2 | ENST00000607014.1 | ENSG00000272345.1 | 0.888631487 | 1.973869239 | 0.048396613 |
| MGST2 | ENST00000607740.1 | ENSG00000271916.1 | -0.915935375 | -2.070854266 | 0.038372417 |
| MGST2 | ENST00000608952.1 | ENSG00000272689.1 | 0.935029735 | 2.088865156 | 0.036719863 |
| MGST2 | ENST00000609146.1 | ENSG00000272851.1 | 0.848716599 | 1.871379606 | 0.061292484 |
| MGST2 | ENST00000609953.1 | ENSG00000272825.1 | -0.824776829 | -1.816115014 | 0.069352731 |
| MGST2 | ENST00000610044.1 | ENSG00000273160.1 | 0.906638432 | 2.046910943 | 0.04066683 |
| MGST2 | ENST00000610185.1 | ENSG00000273355.1 | 0.870752712 | 1.943546317 | 0.051950185 |
| MGST2 | NR_002765.2 | ASAP1-IT1 | -0.905488607 | -2.028281383 | 0.042531538 |
| MGST2 | NR_028325.1 | LOC100132062 | -0.845125068 | -1.899920822 | 0.057443511 |
| MGST2 | NR_046454.1 | LINC00907 | 0.918039693 | 2.034703246 | 0.041880741 |
| MGST2 | NR_103857.1 | SP2-AS1 | 0.92337986 | 2.058466252 | 0.039545398 |
| MGST2 | NR_104158.1 | NRG1-IT1 | -0.88722106 | -2.003550573 | 0.045118225 |
| MGST2 | NR_109870.1 | LINC01723 | -0.9259076 | -2.068633867 | 0.038580457 |
| MGST2 | NR_109975.1 | ARNTL2-AS1 | 0.825977171 | 1.849171602 | 0.064433038 |
| MGST2 | NR_110318.1 | MACROD2-AS1 | -0.948711286 | -2.135009115 | 0.032760266 |
| MGST2 | NR_110635.1 | LINC00687 | -0.962001816 | -2.16064275 | 0.030722946 |
| MGST2 | NR_117098.1 | LINC01353 | -0.840725542 | -1.864347643 | 0.0622729 |
| MGST2 | NR_125407.1 | LOC102724604 | -0.948699808 | -2.09487287 | 0.036182295 |
| MGST2 | NR_125420.1 | LOC101927588 | 0.833991518 | 1.872268358 | 0.061169486 |
| MGST2 | NR_125925.1 | LOC101929448 | -0.939623359 | -2.10495864 | 0.035294909 |
| MGST2 | NR_126412.1 | SCEL-AS1 | 0.843691839 | 1.906696919 | 0.056559846 |
| MGST2 | NR_126413.1 | SCEL-AS1 | 0.85321173 | 1.908661203 | 0.056305811 |
| MGST2 | NR_135032.1 | LOC105369635 | -0.826981891 | -1.861830803 | 0.062626943 |
| MGST2 | NR_135644.1 | LOC105371506 | 0.896402346 | 2.008201781 | 0.044621854 |
| MGST2 | NR_135820.1 | LOC102723727 | -0.932302328 | -2.084022444 | 0.037158126 |
| MGST2 | NR_138041.1 | LINC00384 | -0.821122664 | -1.825282642 | 0.067958388 |
| MICA | ENST00000318291.4 | ENSG00000177406.4 | 0.904232296 | 2.029269754 | 0.042430822 |
| MICA | ENST00000412085.1 | ENSG00000233825.1 | 0.850401692 | 1.908145373 | 0.05637243 |
| MICA | ENST00000412759.1 | ENSG00000236933.1 | 0.961931198 | 2.164921182 | 0.030393722 |
| MICA | ENST00000414740.2 | ENSG00000229646.2 | 0.807148105 | 1.824109232 | 0.06813556 |
| MICA | ENST00000415205.1 | ENSG00000182057.4 | 0.802043918 | 1.75322649 | 0.079563138 |
| MICA | ENST00000419662.1 | ENSG00000228265.1 | 0.928550436 | 2.090201552 | 0.036599698 |
| MICA | ENST00000421020.1 | ENSG00000231407.1 | 0.851149097 | 1.901471456 | 0.057240288 |
| MICA | ENST00000421207.1 | ENSG00000231768.1 | 0.874831622 | 1.954496274 | 0.050642555 |
| MICA | ENST00000423428.1 | ENSG00000224048.1 | -0.800740522 | -1.805430315 | 0.071007371 |
| MICA | ENST00000423869.1 | ENSG00000227848.1 | 0.815391867 | 1.823631667 | 0.068207776 |
| MICA | ENST00000425124.1 | ENSG00000232336.1 | 0.849147513 | 1.900679596 | 0.057343993 |
| MICA | ENST00000426237.2 | ENSG00000235527.2 | 0.925351647 | 2.073153203 | 0.038158024 |
| MICA | ENST00000426519.1 | ENSG00000234142.1 | 0.921475813 | 2.061260789 | 0.039278167 |
| MICA | ENST00000429080.1 | ENSG00000233047.1 | -0.81833203 | -1.856229104 | 0.063420909 |
| MICA | ENST00000430920.1 | ENSG00000234203.1 | 0.842945224 | 1.861266592 | 0.062706538 |
| MICA | ENST00000433051.1 | ENSG00000233193.1 | 0.80409745 | 1.802750939 | 0.071427338 |
| MICA | ENST00000433905.2 | ENSG00000229299.2 | 0.930648524 | 2.066815273 | 0.038751564 |

| | | | | | |
|---|---|---|---|---|---|
| MICA | ENST00000434627.1 | ENSG00000230074.1 | 0.810325526 | 1.820706444 | 0.068651492 |
| MICA | ENST00000435434.1 | ENSG00000231233.1 | 0.844691257 | 1.867695599 | 0.061804512 |
| MICA | ENST00000435892.1 | ENSG00000233635.2 | 0.861680395 | 1.941568993 | 0.0521893 |
| MICA | ENST00000438190.1 | ENSG00000227214.2 | 0.994838939 | 2.219954615 | 0.026421849 |
| MICA | ENST00000440595.1 | ENSG00000228265.1 | 0.814495824 | 1.830720663 | 0.067142247 |
| MICA | ENST00000440714.1 | ENSG00000237609.1 | 0.825910877 | 1.860285501 | 0.062845144 |
| MICA | ENST00000441592.2 | ENSG00000224078.8 | 0.800679028 | 1.802283662 | 0.071500788 |
| MICA | ENST00000447206.1 | ENSG00000230839.1 | 0.816119645 | 1.828929049 | 0.067410238 |
| MICA | ENST00000447343.2 | ENSG00000229299.2 | 0.916901785 | 2.064178279 | 0.039000816 |
| MICA | ENST00000449463.1 | ENSG00000230309.1 | -0.817561112 | -1.844577059 | 0.065099087 |
| MICA | ENST00000451507.1 | ENSG00000229539.1 | 0.80087253 | 1.801222589 | 0.071667803 |
| MICA | ENST00000452176.1 | ENSG00000223659.1 | -0.887027561 | -2.003153602 | 0.045160804 |
| MICA | ENST00000453051.1 | ENSG00000229407.1 | 0.907943391 | 2.044670435 | 0.04088736 |
| MICA | ENST00000457253.1 | ENSG00000225173.1 | 0.848602058 | 1.88299414 | 0.059701162 |
| MICA | ENST00000458154.1 | ENSG00000235578.1 | 0.938955285 | 2.094189603 | 0.036243094 |
| MICA | ENST00000458194.1 | ENSG00000226193.1 | 0.908773001 | 2.042381821 | 0.041113672 |
| MICA | ENST00000458364.1 | ENSG00000225655.1 | -0.82144941 | -1.837355818 | 0.066157385 |
| MICA | ENST00000459985.1 | ENSG00000273066.1 | 0.869602266 | 1.921005105 | 0.054731064 |
| MICA | ENST00000463255.1 | ENSG00000243305.1 | -0.823300863 | -1.842028963 | 0.065470913 |
| MICA | ENST00000468165.1 | ENSG00000239480.1 | 0.883470745 | 1.978598041 | 0.04786128 |
| MICA | ENST00000489077.1 | ENSG00000244198.1 | 0.984534578 | 2.223779509 | 0.026163283 |
| MICA | ENST00000489557.2 | ENSG00000257045.1 | 0.827488829 | 1.834094788 | 0.066639925 |
| MICA | ENST00000498693.1 | ENSG00000244198.1 | 0.98851818 | 2.2294237 | 0.025785727 |
| MICA | ENST00000503723.1 | ENSG00000250472.1 | -0.904821926 | -2.006545987 | 0.044798027 |
| MICA | ENST00000505556.1 | ENSG00000249409.1 | 0.889178824 | 1.997271895 | 0.045795655 |
| MICA | ENST00000506100.1 | ENSG00000249409.1 | 0.882250909 | 1.976302579 | 0.048120518 |
| MICA | ENST00000506791.1 | ENSG00000251131.1 | 0.96478815 | 2.124024509 | 0.033668094 |
| MICA | ENST00000508083.1 | ENSG00000249343.1 | 0.914982383 | 2.0688606 | 0.03855917 |
| MICA | ENST00000509036.1 | ENSG00000251131.1 | 0.972212796 | 2.184736772 | 0.028908152 |
| MICA | ENST00000509192.1 | ENSG00000250765.1 | 0.955331631 | 2.137200188 | 0.032581713 |
| MICA | ENST00000509453.1 | ENSG00000249145.1 | 0.817044353 | 1.846841211 | 0.064770157 |
| MICA | ENST00000510570.1 | ENSG00000250438.1 | -0.854124058 | -1.905647055 | 0.056696013 |
| MICA | ENST00000514877.1 | ENSG00000248685.1 | 0.801225947 | 1.795413944 | 0.072587772 |
| MICA | ENST00000515128.1 | ENSG00000248215.1 | -0.841475751 | -1.872943066 | 0.061076247 |
| MICA | ENST00000517846.1 | ENSG00000254485.1 | 0.819443988 | 1.826331016 | 0.067800416 |
| MICA | ENST00000520603.1 | ENSG00000254001.1 | -0.958238994 | -2.127167504 | 0.033406168 |
| MICA | ENST00000522547.1 | ENSG00000253430.1 | -0.875396835 | -1.937445888 | 0.052690862 |
| MICA | ENST00000522600.1 | ENSG00000246582.2 | 0.804042081 | 1.797734958 | 0.072219019 |
| MICA | ENST00000543072.1 | ENSG00000256092.2 | -0.910414909 | -2.038203706 | 0.041529565 |
| MICA | ENST00000543275.1 | ENSG00000256944.1 | 0.907005411 | 2.009148746 | 0.044521361 |
| MICA | ENST00000545177.3 | ENSG00000230438.5 | 0.881543332 | 1.952873624 | 0.050834572 |
| MICA | ENST00000548722.2 | ENSG00000257194.2 | -0.822275124 | -1.847905868 | 0.064615962 |
| MICA | ENST00000549806.1 | ENSG00000257252.1 | 0.829575667 | 1.807663404 | 0.070658904 |
| MICA | ENST00000558575.1 | ENSG00000259687.1 | 0.836970544 | 1.87321406 | 0.061038832 |
| MICA | ENST00000563018.1 | ENSG00000260193.1 | 0.828103601 | 1.835904102 | 0.066371841 |
| MICA | ENST00000563611.1 | ENSG00000261583.1 | 0.935986606 | 2.07814539 | 0.03769597 |
| MICA | ENST00000565823.1 | ENSG00000260686.1 | -0.83707079 | -1.876240167 | 0.060622309 |
| MICA | ENST00000565829.1 | ENSG00000260148.1 | 0.839381152 | 1.868637807 | 0.061673222 |
| MICA | ENST00000566170.1 | ENSG00000261071.1 | 0.806834576 | 1.801322101 | 0.071652126 |
| MICA | ENST00000568033.1 | ENSG00000261480.1 | 0.805550962 | 1.802985078 | 0.071390558 |
| MICA | ENST00000569981.1 | ENSG00000238045.5 | 0.858299507 | 1.91830785 | 0.055071988 |

| | | | | | |
|---|---|---|---|---|---|
| MICA | ENST00000570493.2 | ENSG00000261898.2 | 0.863016578 | 1.942370822 | 0.052092225 |
| MICA | ENST00000570843.1 | ENSG00000261889.1 | 0.916845195 | 2.060072906 | 0.039391572 |
| MICA | ENST00000570929.1 | ENSG00000262223.2 | 0.90052941 | 1.993977695 | 0.046154492 |
| MICA | ENST00000574460.1 | ENSG00000263051.1 | 0.826148134 | 1.822789557 | 0.06833527 |
| MICA | ENST00000578800.1 | ENSG00000264235.1 | 0.911431588 | 2.041420203 | 0.041209078 |
| MICA | ENST00000578936.1 | ENSG00000265547.1 | 0.831953072 | 1.861962108 | 0.062608431 |
| MICA | ENST00000582044.1 | ENSG00000263715.2 | 0.89589565 | 2.008713021 | 0.044567577 |
| MICA | ENST00000582558.1 | ENSG00000264569.1 | 0.909445116 | 2.058542993 | 0.039538039 |
| MICA | ENST00000585559.1 | ENSG00000267117.1 | 0.845204147 | 1.867229253 | 0.06186958 |
| MICA | ENST00000586051.1 | ENSG00000267576.1 | 0.847847652 | 1.885958021 | 0.059300612 |
| MICA | ENST00000586694.1 | ENSG00000267141.1 | 0.841513514 | 1.877950259 | 0.06038797 |
| MICA | ENST00000588380.1 | ENSG00000266990.1 | 0.877890834 | 1.970700274 | 0.048758169 |
| MICA | ENST00000591174.1 | ENSG00000267289.1 | 0.930935928 | 2.07445881 | 0.03803672 |
| MICA | ENST00000592400.1 | ENSG00000267735.1 | 0.898350486 | 1.998098888 | 0.04570594 |
| MICA | ENST00000593139.1 | ENSG00000267042.1 | 0.812618521 | 1.797830053 | 0.072203943 |
| MICA | ENST00000594590.2 | ENSG00000268199.2 | 0.927693178 | 2.099457667 | 0.035776576 |
| MICA | ENST00000595478.1 | ENSG00000237031.3 | -0.822111709 | -1.863444809 | 0.062399711 |
| MICA | ENST00000595955.1 | ENSG00000268401.1 | 0.808989581 | 1.802896153 | 0.071404525 |
| MICA | ENST00000596091.1 | ENSG00000227733.4 | -0.80235666 | -1.801602381 | 0.071607986 |
| MICA | ENST00000596887.1 | ENSG00000237031.3 | -0.84938612 | -1.883170852 | 0.059677218 |
| MICA | ENST00000597169.1 | ENSG00000269720.1 | 0.957662047 | 2.123755123 | 0.033690625 |
| MICA | ENST00000597256.1 | ENSG00000267986.1 | 0.862228972 | 1.933323236 | 0.053196391 |
| MICA | ENST00000598092.1 | ENSG00000228065.6 | -0.825890491 | -1.838253615 | 0.066025043 |
| MICA | ENST00000599259.1 | ENSG00000269352.1 | 0.849993136 | 1.885248954 | 0.059396234 |
| MICA | ENST00000599352.1 | ENSG00000240401.4 | -0.817183127 | -1.834473328 | 0.066583763 |
| MICA | ENST00000600234.1 | ENSG00000268078.1 | 0.823117487 | 1.851248455 | 0.064133818 |
| MICA | ENST00000600534.1 | ENSG00000267858.1 | 0.835271677 | 1.877534919 | 0.060444816 |
| MICA | ENST00000600726.1 | ENSG00000267858.1 | 0.939921261 | 2.104406193 | 0.03534303 |
| MICA | ENST00000601033.1 | ENSG00000268401.1 | 0.85599884 | 1.915872581 | 0.055381316 |
| MICA | ENST00000601735.1 | ENSG00000244513.2 | 0.87038792 | 1.95598376 | 0.050467067 |
| MICA | ENST00000602532.1 | ENSG00000270091.1 | 0.848484497 | 1.905448342 | 0.056721816 |
| MICA | ENST00000602594.1 | ENSG00000269930.1 | -0.826164815 | -1.856313058 | 0.063408949 |
| MICA | ENST00000604142.1 | ENSG00000271308.1 | 0.993181213 | 2.210433933 | 0.027075061 |
| MICA | ENST00000604183.1 | ENSG00000271185.1 | 0.852018366 | 1.894317346 | 0.0581829 |
| MICA | ENST00000606277.1 | ENSG00000272145.1 | 0.945358302 | 2.132259455 | 0.032985523 |
| MICA | ENST00000606377.1 | ENSG00000272286.1 | -0.879006635 | -1.964803272 | 0.049437011 |
| MICA | ENST00000606470.1 | ENSG00000271913.1 | 0.832855242 | 1.875825126 | 0.060679297 |
| MICA | ENST00000606743.1 | ENSG00000272221.1 | 0.830672201 | 1.878936412 | 0.060253176 |
| MICA | ENST00000606909.1 | ENSG00000271821.1 | 0.857141989 | 1.896132661 | 0.057942505 |
| MICA | ENST00000607224.1 | ENSG00000272521.1 | 0.922411569 | 2.076821094 | 0.037818075 |
| MICA | ENST00000607476.1 | ENSG00000272540.1 | 0.994377393 | 2.234384165 | 0.025457807 |
| MICA | ENST00000607943.1 | ENSG00000273188.1 | 0.964356026 | 2.149046264 | 0.031630733 |
| MICA | ENST00000608367.1 | ENSG00000273361.1 | 0.805650743 | 1.782396259 | 0.074684635 |
| MICA | ENST00000608489.1 | ENSG00000272716.1 | 0.822496893 | 1.858636433 | 0.063078689 |
| MICA | ENST00000608677.1 | ENSG00000273350.1 | 0.880355651 | 1.964803956 | 0.049436932 |
| MICA | ENST00000609113.1 | ENSG00000272827.1 | 0.803022099 | 1.792072674 | 0.073121326 |
| MICA | NR_003604.2 | ZFAS1 | 0.941124092 | 2.077137612 | 0.03778886 |
| MICA | NR_003605.1 | ZFAS1 | 0.941137018 | 2.126400726 | 0.033469907 |
| MICA | NR_003606.2 | ZFAS1 | 0.943155816 | 2.102066191 | 0.035547478 |
| MICA | NR_024321.1 | LINC00115 | 0.810897066 | 1.800175333 | 0.071832958 |
| MICA | NR_026802.1 | FAM74A4 | 0.955718112 | 2.160594203 | 0.0307267 |

| | | | | | |
|---|---|---|---|---|---|
| MICA | NR_026951.1 | LINC00324 | 0.847076271 | 1.888871335 | 0.058909071 |
| MICA | NR_027052.1 | THAP7-AS1 | 0.888055386 | 1.97790805 | 0.047939081 |
| MICA | NR_027271.1 | CIRBP-AS1 | 0.972948188 | 2.168061058 | 0.030154041 |
| MICA | NR_027334.2 | MZF1-AS1 | 0.847192443 | 1.89400804 | 0.058223943 |
| MICA | NR_036480.1 | VPS9D1-AS1 | 0.879905739 | 1.960692978 | 0.049914849 |
| MICA | NR_036658.1 | ZFAS1 | 0.945974239 | 2.119265361 | 0.034068047 |
| MICA | NR_037169.1 | LOC100507547 | 0.904436779 | 2.017536308 | 0.043639576 |
| MICA | NR_037170.1 | LOC100507547 | 0.859039658 | 1.915880179 | 0.055380349 |
| MICA | NR_038421.1 | LINC01220 | 0.910365477 | 2.031092273 | 0.042245635 |
| MICA | NR_038923.1 | SSSCA1-AS1 | 0.891106171 | 1.99586575 | 0.045948538 |
| MICA | NR_045114.1 | PVRL3-AS1 | -0.944507519 | -2.130122857 | 0.033161472 |
| MICA | NR_047116.1 | HIF1A-AS1 | -0.811746527 | -1.829194648 | 0.067370454 |
| MICA | NR_072981.1 | LINC00957 | 0.935249528 | 2.077177315 | 0.037785197 |
| MICA | NR_072982.1 | LINC00957 | 0.93097939 | 2.07172108 | 0.03829146 |
| MICA | NR_103790.1 | LINC00581 | -0.821157966 | -1.831000175 | 0.067100517 |
| MICA | NR_105010.1 | LINC01333 | 0.972353095 | 2.157517995 | 0.030965327 |
| MICA | NR_108036.1 | CFAP58-AS1 | 0.849250295 | 1.923575325 | 0.054407836 |
| MICA | NR_109885.1 | RALY-AS1 | 0.836729343 | 1.834794456 | 0.06653615 |
| MICA | NR_109886.1 | RALY-AS1 | 0.940479492 | 2.095669014 | 0.036111562 |
| MICA | NR_110245.1 | LOC101929282 | -0.829408448 | -1.888169482 | 0.059003201 |
| MICA | NR_110630.1 | LOC101927478 | 0.842097576 | 1.871362424 | 0.061294863 |
| MICA | NR_110941.1 | MIR762HG | 0.91784029 | 2.056432119 | 0.039740883 |
| MICA | NR_110998.1 | FAM74A4 | 0.955718112 | 2.142702438 | 0.032137 |
| MICA | NR_111951.1 | LINC00869 | 0.843997019 | 1.894427761 | 0.058168254 |
| MICA | NR_111952.1 | LINC00869 | 0.867520013 | 1.956392998 | 0.050418876 |
| MICA | NR_111953.1 | LINC00869 | 0.833522936 | 1.856502147 | 0.063382017 |
| MICA | NR_121189.1 | PGM5P3-AS1 | -0.842940383 | -1.890701671 | 0.058664179 |
| MICA | NR_125957.1 | LOC101928626 | -0.887027561 | -1.971098427 | 0.048712619 |
| MICA | NR_126522.1 | EXOC3-AS1 | 0.928349957 | 2.072945794 | 0.038177325 |
| MICA | NR_130143.1 | LOC104968399 | 0.926671821 | 2.073971035 | 0.038082001 |
| MICA | NR_135024.1 | LOC105369747 | 0.884514523 | 1.979108439 | 0.047803799 |
| MICA | NR_135584.1 | LOC101927596 | 0.858185089 | 1.91416862 | 0.055598614 |
| MICA | NR_144459.1 | ARSD-AS1 | 0.826698633 | 1.870904877 | 0.061358267 |
| MPLKIP | ENST00000399186.2 | ENSG00000214888.2 | 0.841211251 | 1.892968688 | 0.058362034 |
| MPLKIP | ENST00000400768.2 | ENSG00000215692.2 | 0.84745949 | 1.882656193 | 0.059746976 |
| MPLKIP | ENST00000411489.1 | ENSG00000227112.1 | -0.822870728 | -1.831132053 | 0.067080835 |
| MPLKIP | ENST00000416641.1 | ENSG00000226956.1 | -0.83337239 | -1.861315582 | 0.062699624 |
| MPLKIP | ENST00000418972.1 | ENSG00000225044.1 | -0.848465235 | -1.896844563 | 0.057848456 |
| MPLKIP | ENST00000420766.1 | ENSG00000228679.1 | 0.92347773 | 2.038438282 | 0.041506121 |
| MPLKIP | ENST00000421020.1 | ENSG00000231407.1 | 0.864912601 | 1.938356705 | 0.052579719 |
| MPLKIP | ENST00000424181.1 | ENSG00000224977.1 | 0.898602771 | 2.022669834 | 0.043107199 |
| MPLKIP | ENST00000428769.1 | ENSG00000232738.1 | 0.849826114 | 1.903568436 | 0.056966413 |
| MPLKIP | ENST00000429666.1 | ENSG00000233755.1 | 0.804767369 | 1.811380053 | 0.070082042 |
| MPLKIP | ENST00000432314.1 | ENSG00000231532.1 | 0.840826934 | 1.878783901 | 0.060274006 |
| MPLKIP | ENST00000434790.1 | ENSG00000240040.1 | -0.872976187 | -1.943204231 | 0.051991487 |
| MPLKIP | ENST00000435733.1 | ENSG00000226377.1 | 0.897830677 | 2.022441721 | 0.043130739 |
| MPLKIP | ENST00000435992.2 | ENSG00000232675.3 | 0.900968311 | 2.0093209 | 0.044503113 |
| MPLKIP | ENST00000438222.1 | ENSG00000238034.1 | 0.961377211 | 2.15289686 | 0.031326785 |
| MPLKIP | ENST00000442069.1 | ENSG00000225655.1 | -0.806387809 | -1.808868781 | 0.070471391 |
| MPLKIP | ENST00000442831.1 | ENSG00000229550.1 | -0.808077668 | -1.850615848 | 0.064224838 |
| MPLKIP | ENST00000443306.1 | ENSG00000233891.3 | 0.838178831 | 1.870436198 | 0.061423269 |

| | | | | | |
|---|---|---|---|---|---|
| MPLKIP | ENST00000447514.1 | ENSG00000236753.1 | 0.827162386 | 1.839219541 | 0.065882902 |
| MPLKIP | ENST00000447709.1 | ENSG00000237473.1 | 0.883627246 | 2.008549464 | 0.044584935 |
| MPLKIP | ENST00000449457.1 | ENSG00000224034.1 | -0.823874775 | -1.852801913 | 0.063910756 |
| MPLKIP | ENST00000450226.1 | ENSG00000231512.1 | 0.863382136 | 1.9347583 | 0.053019963 |
| MPLKIP | ENST00000452412.1 | ENSG00000233860.1 | 0.881925814 | 1.959286249 | 0.050079273 |
| MPLKIP | ENST00000453579.1 | ENSG00000232529.1 | 0.856121423 | 1.935419698 | 0.052938814 |
| MPLKIP | ENST00000454489.1 | ENSG00000231403.1 | 0.874047922 | 1.947555341 | 0.051468192 |
| MPLKIP | ENST00000454526.1 | ENSG00000234136.1 | 0.884449096 | 1.996515433 | 0.045877848 |
| MPLKIP | ENST00000455699.1 | ENSG00000240996.1 | 0.823127728 | 1.836649378 | 0.066261673 |
| MPLKIP | ENST00000455788.1 | ENSG00000236263.1 | 0.863224349 | 1.91592278 | 0.055374925 |
| MPLKIP | ENST00000469931.2 | ENSG00000272030.1 | 0.862393785 | 1.939581937 | 0.052430518 |
| MPLKIP | ENST00000472596.1 | ENSG00000239774.1 | 0.841897727 | 1.877566608 | 0.060440477 |
| MPLKIP | ENST00000484413.1 | ENSG00000271853.1 | 0.859718106 | 1.917046287 | 0.055232051 |
| MPLKIP | ENST00000493123.1 | ENSG00000242428.1 | 0.83511076 | 1.86218073 | 0.06257762 |
| MPLKIP | ENST00000494509.1 | ENSG00000240095.1 | 0.913919748 | 2.028191196 | 0.042540738 |
| MPLKIP | ENST00000501405.2 | ENSG00000247402.2 | -0.822870728 | -1.850827082 | 0.064194434 |
| MPLKIP | ENST00000504755.1 | ENSG00000250252.1 | 0.909796579 | 2.051212622 | 0.040246245 |
| MPLKIP | ENST00000504891.1 | ENSG00000249388.1 | 0.884920349 | 1.991987278 | 0.046372454 |
| MPLKIP | ENST00000507373.1 | ENSG00000250072.1 | 0.85102625 | 1.902249584 | 0.057138533 |
| MPLKIP | ENST00000515750.1 | ENSG00000249061.1 | -0.828600205 | -1.851846452 | 0.064047875 |
| MPLKIP | ENST00000519506.1 | ENSG00000253103.1 | 0.867369532 | 1.931885169 | 0.053373681 |
| MPLKIP | ENST00000521307.1 | ENSG00000253177.1 | 0.897277737 | 2.001735155 | 0.045313223 |
| MPLKIP | ENST00000522704.1 | ENSG00000254135.1 | 0.833657609 | 1.859054093 | 0.063019471 |
| MPLKIP | ENST00000526694.1 | ENSG00000231999.2 | 0.880985836 | 1.962880762 | 0.049660031 |
| MPLKIP | ENST00000526935.1 | ENSG00000255372.1 | 0.832071853 | 1.854980685 | 0.063598985 |
| MPLKIP | ENST00000531627.1 | ENSG00000254584.1 | 0.836599208 | 1.881235845 | 0.059939844 |
| MPLKIP | ENST00000531661.1 | ENSG00000254473.1 | 0.897078401 | 2.008968465 | 0.044540478 |
| MPLKIP | ENST00000535720.1 | ENSG00000256364.1 | 0.924587294 | 2.097960522 | 0.035908633 |
| MPLKIP | ENST00000537921.1 | ENSG00000255966.1 | 0.858993444 | 1.922884907 | 0.054494506 |
| MPLKIP | ENST00000545642.1 | ENSG00000256342.1 | 0.820842843 | 1.836829387 | 0.066235086 |
| MPLKIP | ENST00000548722.2 | ENSG00000257194.2 | -0.890551375 | -1.983295581 | 0.047334423 |
| MPLKIP | ENST00000549140.1 | ENSG00000258332.1 | 0.897149306 | 2.001827645 | 0.045303272 |
| MPLKIP | ENST00000554451.1 | ENSG00000258683.1 | 0.88334477 | 1.963098658 | 0.049634711 |
| MPLKIP | ENST00000557903.1 | ENSG00000259182.1 | 0.803803752 | 1.790710716 | 0.073339729 |
| MPLKIP | ENST00000560522.1 | ENSG00000259661.1 | 0.848769864 | 1.898511684 | 0.05762871 |
| MPLKIP | ENST00000563044.1 | ENSG00000260978.1 | 0.898858568 | 2.022273704 | 0.043148084 |
| MPLKIP | ENST00000567089.1 | ENSG00000261822.1 | 0.891142543 | 1.991253422 | 0.046453033 |
| MPLKIP | ENST00000567127.1 | ENSG00000260264.1 | -0.847469882 | -1.916317881 | 0.055324646 |
| MPLKIP | ENST00000567395.1 | ENSG00000261090.1 | 0.873114913 | 1.915052135 | 0.055485855 |
| MPLKIP | ENST00000569981.1 | ENSG00000238045.5 | 0.847404057 | 1.882463994 | 0.059773044 |
| MPLKIP | ENST00000570512.1 | ENSG00000262768.1 | 0.872554409 | 1.94505285 | 0.051768618 |
| MPLKIP | ENST00000574365.1 | ENSG00000262837.1 | 0.947906375 | 2.115613519 | 0.034377691 |
| MPLKIP | ENST00000577807.1 | ENSG00000263427.1 | 0.992143119 | 2.240461298 | 0.025060991 |
| MPLKIP | ENST00000578265.1 | ENSG00000214719.7 | 0.851044697 | 1.900229899 | 0.057402956 |
| MPLKIP | ENST00000578757.1 | ENSG00000175061.13 | 0.914509061 | 2.031953846 | 0.042158329 |
| MPLKIP | ENST00000580975.1 | ENSG00000266237.1 | 0.823577107 | 1.847058692 | 0.064738635 |
| MPLKIP | ENST00000581362.1 | ENSG00000235300.3 | 0.917455006 | 2.066612554 | 0.038770677 |
| MPLKIP | ENST00000581940.1 | ENSG00000265484.1 | 0.80325241 | 1.7991096 | 0.072001345 |
| MPLKIP | ENST00000582386.1 | ENSG00000265174.1 | 0.893825091 | 1.990687557 | 0.046515248 |
| MPLKIP | ENST00000583841.1 | ENSG00000265148.1 | 0.823988152 | 1.859090543 | 0.063014306 |
| MPLKIP | ENST00000584758.1 | ENSG00000265356.1 | 0.971972796 | 2.161446848 | 0.030660839 |

| | | | | | |
|---|---|---|---|---|---|
| MPLKIP | ENST00000588835.1 | ENSG00000267476.1 | 0.836476394 | 1.899431514 | 0.057507763 |
| MPLKIP | ENST00000595892.1 | ENSG00000269640.1 | 0.801454547 | 1.813473754 | 0.069758783 |
| MPLKIP | ENST00000596135.1 | ENSG00000269843.1 | 0.836791545 | 1.879507029 | 0.060175295 |
| MPLKIP | ENST00000596643.1 | ENSG00000269439.1 | 0.828850031 | 1.849433874 | 0.064395188 |
| MPLKIP | ENST00000599259.1 | ENSG00000269352.1 | 0.821700957 | 1.824453816 | 0.068083492 |
| MPLKIP | ENST00000599467.1 | ENSG00000244513.2 | 0.994474261 | 2.214990273 | 0.026760732 |
| MPLKIP | ENST00000600365.1 | ENSG00000231898.4 | 0.819708515 | 1.838453922 | 0.065995546 |
| MPLKIP | ENST00000601511.1 | ENSG00000244513.2 | 0.858275915 | 1.925523171 | 0.05416394 |
| MPLKIP | ENST00000601735.1 | ENSG00000244513.2 | 0.806314495 | 1.821401389 | 0.068545864 |
| MPLKIP | ENST00000602594.1 | ENSG00000269930.1 | -0.809865362 | -1.806292469 | 0.070872668 |
| MPLKIP | ENST00000602809.1 | ENSG00000270105.1 | -0.9574148 | -2.157950329 | 0.030931694 |
| MPLKIP | ENST00000606470.1 | ENSG00000271913.1 | 0.872328477 | 1.938422329 | 0.052571718 |
| MPLKIP | ENST00000606841.1 | ENSG00000272411.1 | 0.809037933 | 1.814937083 | 0.069533579 |
| MPLKIP | ENST00000607715.1 | ENSG00000271788.1 | 0.812268845 | 1.806741011 | 0.07080267 |
| MPLKIP | ENST00000607876.1 | ENSG00000272848.1 | 0.941386192 | 2.100000689 | 0.035728781 |
| MPLKIP | ENST00000608085.1 | ENSG00000231898.4 | 0.860049086 | 1.941071175 | 0.052249645 |
| MPLKIP | ENST00000608476.1 | ENSG00000232675.3 | 0.820118344 | 1.810008837 | 0.070294417 |
| MPLKIP | ENST00000608856.1 | ENSG00000272600.1 | -0.934522729 | -2.094835036 | 0.036185659 |
| MPLKIP | ENST00000609610.1 | ENSG00000232675.3 | 0.874501641 | 1.961835245 | 0.049781669 |
| MPLKIP | ENST00000609725.1 | ENSG00000231898.4 | 0.85876564 | 1.920121888 | 0.054842505 |
| MPLKIP | NR_046742.2 | ZNF630-AS1 | -0.804769692 | -1.81929709 | 0.068866117 |
| MPLKIP | NR_046839.1 | AGBL4-IT1 | -0.830792398 | -1.869859331 | 0.061503355 |
| MPLKIP | NR_104998.1 | LOC102467225 | 0.826910024 | 1.846977035 | 0.064750469 |
| MPLKIP | NR_110053.1 | LOC101927464 | 0.820842843 | 1.833207131 | 0.066771774 |
| MPLKIP | NR_120318.1 | RORA-AS2 | 0.913837142 | 2.03610026 | 0.041740289 |
| MPLKIP | NR_134565.1 | LOC101928807 | -0.825166776 | -1.860832948 | 0.062767771 |
| MPLKIP | NR_135274.1 | LOC105370619 | 0.931547549 | 2.100786483 | 0.035659714 |
| MPLKIP | NR_135840.1 | LOC105376114 | 0.836435938 | 1.838595596 | 0.06597469 |
| MPLKIP | NR_138038.1 | LINC00677 | 0.803309642 | 1.810874862 | 0.070160225 |
| MPLKIP | NR_138084.1 | HCG24 | 0.823651109 | 1.83080881 | 0.067129085 |
| MPLKIP | NR_138419.1 | ARHGEF9-IT1 | 0.800893616 | 1.783411746 | 0.074519303 |
| MRPL34 | ENST00000412812.1 | ENSG00000225342.1 | 0.864058425 | 1.952760067 | 0.050848032 |
| MRPL34 | ENST00000412896.1 | ENSG00000197585.5 | -0.820170227 | -1.831464605 | 0.067031226 |
| MRPL34 | ENST00000413564.1 | ENSG00000224500.1 | -0.906073224 | -2.018655453 | 0.043523043 |
| MRPL34 | ENST00000414740.2 | ENSG00000229646.2 | 0.847908976 | 1.866580217 | 0.061960232 |
| MRPL34 | ENST00000419734.1 | ENSG00000234646.1 | -0.827526546 | -1.863272888 | 0.062423883 |
| MRPL34 | ENST00000420572.2 | ENSG00000233358.2 | 0.936218726 | 2.100486348 | 0.03568608 |
| MRPL34 | ENST00000421617.1 | ENSG00000237342.1 | 0.924029255 | 2.05826937 | 0.039564283 |
| MRPL34 | ENST00000421866.1 | ENSG00000233875.1 | -0.855915542 | -1.878424671 | 0.060323093 |
| MRPL34 | ENST00000423925.1 | ENSG00000223536.1 | -0.877360171 | -1.970441381 | 0.048787807 |
| MRPL34 | ENST00000424241.1 | ENSG00000237311.1 | -0.914744952 | -2.040792293 | 0.041271477 |
| MRPL34 | ENST00000425364.1 | ENSG00000231046.1 | -0.94504624 | -2.133352932 | 0.032895785 |
| MRPL34 | ENST00000426504.1 | ENSG00000234190.1 | -0.839199808 | -1.863140075 | 0.062442561 |
| MRPL34 | ENST00000429796.1 | ENSG00000231858.1 | -0.942650217 | -2.097206986 | 0.035975256 |
| MRPL34 | ENST00000430545.1 | ENSG00000237153.1 | 0.946344699 | 2.147943727 | 0.031718226 |
| MRPL34 | ENST00000431290.1 | ENSG00000183822.2 | 0.887390731 | 1.983281722 | 0.04733597 |
| MRPL34 | ENST00000432244.1 | ENSG00000234265.1 | 0.977818064 | 2.186201663 | 0.028800855 |
| MRPL34 | ENST00000432559.2 | ENSG00000228229.2 | 0.933880242 | 2.055929011 | 0.039789359 |
| MRPL34 | ENST00000433876.2 | ENSG00000228423.2 | 0.914151975 | 2.019251663 | 0.043461069 |
| MRPL34 | ENST00000434627.1 | ENSG00000230074.1 | 0.851496191 | 1.929843862 | 0.053626187 |
| MRPL34 | ENST00000435892.1 | ENSG00000233635.2 | 0.859909338 | 1.956168338 | 0.050445327 |

| | | | | | |
|---|---|---|---|---|---|
| MRPL34 | ENST00000435984.1 | ENSG00000204792.2 | 0.872450419 | 1.932030666 | 0.053355721 |
| MRPL34 | ENST00000436582.1 | ENSG00000236525.1 | -0.872896893 | -1.937440597 | 0.052691508 |
| MRPL34 | ENST00000437461.1 | ENSG00000227200.1 | 0.842904125 | 1.889555116 | 0.058817485 |
| MRPL34 | ENST00000438488.1 | ENSG00000223812.1 | 0.862998555 | 1.940267932 | 0.052347136 |
| MRPL34 | ENST00000441532.1 | ENSG00000234206.1 | -0.973612216 | -2.157471709 | 0.030968929 |
| MRPL34 | ENST00000441666.1 | ENSG00000230379.1 | 0.848086159 | 1.872639923 | 0.061118124 |
| MRPL34 | ENST00000442850.1 | ENSG00000232600.2 | -0.938170329 | -2.109416747 | 0.034908625 |
| MRPL34 | ENST00000443380.1 | ENSG00000224371.1 | 0.918129343 | 2.03884246 | 0.041465753 |
| MRPL34 | ENST00000444665.1 | ENSG00000228852.2 | 0.901302323 | 2.021139748 | 0.043265301 |
| MRPL34 | ENST00000444731.1 | ENSG00000227131.1 | -0.886086102 | -1.974024036 | 0.04837901 |
| MRPL34 | ENST00000445260.2 | ENSG00000231429.2 | 0.882398455 | 1.974721083 | 0.048299809 |
| MRPL34 | ENST00000447206.1 | ENSG00000230839.1 | 0.883407098 | 1.961008829 | 0.049877993 |
| MRPL34 | ENST00000448650.1 | ENSG00000223536.1 | -0.86102909 | -1.936276523 | 0.052833843 |
| MRPL34 | ENST00000448942.1 | ENSG00000237499.2 | 0.893005446 | 1.972175455 | 0.048589581 |
| MRPL34 | ENST00000449586.1 | ENSG00000235257.4 | -0.871381257 | -1.937857898 | 0.052640562 |
| MRPL34 | ENST00000450109.1 | ENSG00000225376.1 | 0.907410386 | 2.057026166 | 0.039683709 |
| MRPL34 | ENST00000452002.1 | ENSG00000236501.1 | -0.899203222 | -2.022418862 | 0.043133098 |
| MRPL34 | ENST00000454387.1 | ENSG00000223726.1 | -0.806676357 | -1.805140981 | 0.071052624 |
| MRPL34 | ENST00000454530.1 | ENSG00000226649.1 | -0.884481496 | -1.973280157 | 0.048463652 |
| MRPL34 | ENST00000456715.1 | ENSG00000224893.1 | 0.881262036 | 1.943381281 | 0.051970107 |
| MRPL34 | ENST00000457602.1 | ENSG00000237576.1 | -0.860318758 | -1.933213161 | 0.053209944 |
| MRPL34 | ENST00000457848.1 | ENSG00000226412.1 | 0.876187749 | 1.98089863 | 0.047602643 |
| MRPL34 | ENST00000458082.1 | ENSG00000231210.2 | -0.867173157 | -1.932740246 | 0.053268205 |
| MRPL34 | ENST00000458107.3 | ENSG00000248478.2 | 0.842197897 | 1.896160505 | 0.057938824 |
| MRPL34 | ENST00000458443.1 | ENSG00000238232.1 | 0.940963363 | 2.110679262 | 0.03479989 |
| MRPL34 | ENST00000460993.1 | ENSG00000241231.1 | 0.823129506 | 1.823373669 | 0.068246815 |
| MRPL34 | ENST00000490013.1 | ENSG00000184115.12 | 0.853733053 | 1.918783876 | 0.055011691 |
| MRPL34 | ENST00000502421.1 | ENSG00000250284.1 | -0.825513599 | -1.850016864 | 0.064311119 |
| MRPL34 | ENST00000503034.1 | ENSG00000248936.1 | 0.955975806 | 2.143364236 | 0.032083862 |
| MRPL34 | ENST00000505196.1 | ENSG00000248131.1 | 0.893106111 | 2.001890043 | 0.045296559 |
| MRPL34 | ENST00000505844.1 | ENSG00000248455.1 | 0.812916528 | 1.811261626 | 0.070100363 |
| MRPL34 | ENST00000507558.1 | ENSG00000248445.1 | 0.919779279 | 2.062900326 | 0.039122099 |
| MRPL34 | ENST00000508004.2 | ENSG00000251661.3 | 0.893292575 | 2.007853589 | 0.044658852 |
| MRPL34 | ENST00000508188.1 | ENSG00000250999.1 | 0.815291322 | 1.84258987 | 0.065388914 |
| MRPL34 | ENST00000510602.1 | ENSG00000249122.1 | -0.84304276 | -1.902149083 | 0.057151667 |
| MRPL34 | ENST00000514411.1 | ENSG00000250882.1 | 0.888742572 | 1.975395935 | 0.048223234 |
| MRPL34 | ENST00000514877.1 | ENSG00000248685.1 | 0.885135135 | 1.974619221 | 0.048311376 |
| MRPL34 | ENST00000515077.1 | ENSG00000251206.1 | 0.899384174 | 2.012746131 | 0.044141344 |
| MRPL34 | ENST00000518260.1 | ENSG00000253628.1 | -0.909082921 | -2.052811066 | 0.040090905 |
| MRPL34 | ENST00000518894.1 | ENSG00000204758.3 | 0.933594346 | 2.081149177 | 0.037420254 |
| MRPL34 | ENST00000519038.2 | ENSG00000254054.2 | -0.871637236 | -1.957212653 | 0.050322472 |
| MRPL34 | ENST00000519368.1 | ENSG00000253215.1 | 0.941262369 | 2.101381861 | 0.035607459 |
| MRPL34 | ENST00000519451.1 | ENSG00000253363.1 | 0.844590745 | 1.90177108 | 0.057201089 |
| MRPL34 | ENST00000519844.1 | ENSG00000253824.1 | -0.841963136 | -1.876262051 | 0.060619305 |
| MRPL34 | ENST00000519852.1 | ENSG00000253716.1 | 0.830451961 | 1.854006392 | 0.063738246 |
| MRPL34 | ENST00000520838.1 | ENSG00000253404.1 | 0.847974093 | 1.916814704 | 0.055261476 |
| MRPL34 | ENST00000520849.1 | ENSG00000253553.1 | 0.827138021 | 1.829884259 | 0.067267248 |
| MRPL34 | ENST00000521207.1 | ENSG00000253716.1 | 0.888220944 | 1.975175649 | 0.048248218 |
| MRPL34 | ENST00000523703.1 | ENSG00000214803.3 | -0.827347262 | -1.866056769 | 0.062033423 |
| MRPL34 | ENST00000524335.1 | ENSG00000253716.1 | 0.879645328 | 1.975113725 | 0.048255244 |
| MRPL34 | ENST00000528000.1 | ENSG00000254804.1 | 0.887229839 | 1.960678754 | 0.049916509 |

| | | | | | |
|---|---|---|---|---|---|
| MRPL34 | ENST00000528818.1 | ENSG00000232995.3 | 0.975474328 | 2.170320675 | 0.029982561 |
| MRPL34 | ENST00000528869.1 | ENSG00000255443.1 | 0.853776248 | 1.909624781 | 0.056181542 |
| MRPL34 | ENST00000531071.1 | ENSG00000255248.2 | -0.855165766 | -1.93051127 | 0.05354352 |
| MRPL34 | ENST00000531136.1 | ENSG00000255558.1 | -0.985135424 | -2.159769266 | 0.030790536 |
| MRPL34 | ENST00000532947.1 | ENSG00000255322.1 | 0.886355482 | 1.97572764 | 0.048185633 |
| MRPL34 | ENST00000533101.1 | ENSG00000255311.1 | 0.810960947 | 1.800158132 | 0.071835673 |
| MRPL34 | ENST00000536141.1 | ENSG00000256969.1 | 0.832134464 | 1.850864099 | 0.064189107 |
| MRPL34 | ENST00000537269.1 | ENSG00000257084.1 | 0.819915316 | 1.819557993 | 0.068826344 |
| MRPL34 | ENST00000538294.1 | ENSG00000250748.2 | -0.811878169 | -1.799067603 | 0.072007988 |
| MRPL34 | ENST00000548731.1 | ENSG00000257809.1 | 0.844406582 | 1.882709987 | 0.059739681 |
| MRPL34 | ENST00000553668.1 | ENSG00000258733.1 | 0.838109341 | 1.845236633 | 0.065003124 |
| MRPL34 | ENST00000554049.1 | ENSG00000258763.1 | 0.952733369 | 2.139675206 | 0.032381024 |
| MRPL34 | ENST00000554430.1 | ENSG00000258646.1 | -0.853881296 | -1.930889758 | 0.053496687 |
| MRPL34 | ENST00000554798.1 | ENSG00000258483.1 | 0.934718496 | 2.059900803 | 0.039408025 |
| MRPL34 | ENST00000556978.1 | ENSG00000258693.1 | 0.828486983 | 1.863343704 | 0.062413925 |
| MRPL34 | ENST00000557412.1 | ENSG00000257621.3 | -0.932915413 | -2.090820495 | 0.036544158 |
| MRPL34 | ENST00000558875.1 | ENSG00000259737.2 | 0.9142416 | 2.045662188 | 0.040789618 |
| MRPL34 | ENST00000561529.1 | ENSG00000260886.1 | 0.919431882 | 2.06474551 | 0.038947086 |
| MRPL34 | ENST00000565359.1 | ENSG00000260601.1 | 0.916413494 | 2.057163106 | 0.039670539 |
| MRPL34 | ENST00000565829.1 | ENSG00000260148.1 | 0.802687548 | 1.788583485 | 0.07368192 |
| MRPL34 | ENST00000569459.1 | ENSG00000261346.1 | 0.849036206 | 1.899614256 | 0.05748376 |
| MRPL34 | ENST00000569742.1 | ENSG00000260787.1 | 0.9188506 | 2.044468755 | 0.040907261 |
| MRPL34 | ENST00000570158.1 | ENSG00000260937.1 | -0.803916063 | -1.793069082 | 0.072961879 |
| MRPL34 | ENST00000571815.1 | ENSG00000262810.1 | 0.910019633 | 2.012398559 | 0.04417794 |
| MRPL34 | ENST00000573414.1 | ENSG00000263072.1 | 0.944760341 | 2.11476626 | 0.034449874 |
| MRPL34 | ENST00000574460.1 | ENSG00000263051.1 | 0.87439996 | 1.947299971 | 0.051498782 |
| MRPL34 | ENST00000577064.1 | ENSG00000262823.1 | 0.811937412 | 1.816201515 | 0.069339466 |
| MRPL34 | ENST00000581905.1 | ENSG00000264235.1 | 0.914496513 | 2.0267221 | 0.042690841 |
| MRPL34 | ENST00000582895.1 | ENSG00000264729.1 | -0.804381029 | -1.823176174 | 0.068276712 |
| MRPL34 | ENST00000583138.1 | ENSG00000263393.1 | 0.867860399 | 1.941836959 | 0.052156841 |
| MRPL34 | ENST00000585072.1 | ENSG00000263745.1 | 0.840649501 | 1.87890459 | 0.060257522 |
| MRPL34 | ENST00000586051.1 | ENSG00000267576.1 | 0.860139471 | 1.924838613 | 0.054249552 |
| MRPL34 | ENST00000587049.1 | ENSG00000235535.3 | -0.847879417 | -1.919600623 | 0.054908365 |
| MRPL34 | ENST00000589281.1 | ENSG00000267707.1 | -0.944072536 | -2.093211034 | 0.03633032 |
| MRPL34 | ENST00000591137.1 | ENSG00000267405.1 | 0.829162774 | 1.870098846 | 0.061470093 |
| MRPL34 | ENST00000591836.1 | ENSG00000267776.1 | 0.938643472 | 2.109817428 | 0.034874085 |
| MRPL34 | ENST00000598131.1 | ENSG00000269043.1 | 0.939159632 | 2.110561803 | 0.034809994 |
| MRPL34 | ENST00000600071.1 | ENSG00000269199.1 | 0.934272864 | 2.074782455 | 0.038006701 |
| MRPL34 | ENST00000600234.1 | ENSG00000268078.1 | 0.831414962 | 1.861313582 | 0.062699906 |
| MRPL34 | ENST00000600959.1 | ENSG00000269303.1 | 0.814248788 | 1.840674072 | 0.065669336 |
| MRPL34 | ENST00000601752.1 | ENSG00000268051.1 | 0.913868379 | 2.060455244 | 0.03935504 |
| MRPL34 | ENST00000602532.1 | ENSG00000270091.1 | 0.856570678 | 1.903090848 | 0.057028692 |
| MRPL34 | ENST00000603612.1 | ENSG00000270996.1 | 0.863469425 | 1.931890478 | 0.053373025 |
| MRPL34 | ENST00000606068.1 | ENSG00000272342.1 | 0.939083393 | 2.088909429 | 0.036715877 |
| MRPL34 | ENST00000606778.1 | ENSG00000271930.1 | 0.810301872 | 1.807959948 | 0.070612734 |
| MRPL34 | ENST00000606938.1 | ENSG00000272198.1 | -0.816533897 | -1.815421804 | 0.069459113 |
| MRPL34 | ENST00000607136.1 | ENSG00000267546.2 | -0.800678227 | -1.814072934 | 0.069666498 |
| MRPL34 | ENST00000607740.1 | ENSG00000271916.1 | 0.810252152 | 1.827743795 | 0.067588012 |
| MRPL34 | ENST00000608952.1 | ENSG00000272689.1 | -0.843844002 | -1.896601906 | 0.057880499 |
| MRPL34 | ENST00000609146.1 | ENSG00000272851.1 | -0.841464301 | -1.87244269 | 0.061145384 |
| MRPL34 | ENST00000609238.1 | ENSG00000272703.1 | -0.803237419 | -1.794317631 | 0.072762486 |

| | | | | | |
|---|---|---|---|---|---|
| MRPL34 | ENST00000609281.1 | ENSG00000273320.1 | 0.943186353 | 2.117944221 | 0.034179792 |
| MRPL34 | NR_002765.2 | ASAP1-IT1 | 0.884265658 | 1.986645968 | 0.046961643 |
| MRPL34 | NR_034037.1 | LINC00582 | -0.950900362 | -2.146351493 | 0.031844946 |
| MRPL34 | NR_034111.1 | TRAF3IP2-AS1 | 0.950612172 | 2.133789132 | 0.032860046 |
| MRPL34 | NR_046454.1 | LINC00907 | -0.971315202 | -2.190478185 | 0.028489575 |
| MRPL34 | NR_103445.2 | RBFADN | -0.835585586 | -1.874363324 | 0.060880365 |
| MRPL34 | NR_103851.1 | TAT-AS1 | 0.866938934 | 1.952576968 | 0.050869743 |
| MRPL34 | NR_104158.1 | NRG1-IT1 | 0.849115964 | 1.91743981 | 0.055182081 |
| MRPL34 | NR_109870.1 | LINC01723 | 0.985995386 | 2.19884982 | 0.027888603 |
| MRPL34 | NR_110318.1 | MACROD2-AS1 | 0.940604532 | 2.0840068 | 0.037159549 |
| MRPL34 | NR_110568.1 | LOC101927661 | 0.932310752 | 2.052210509 | 0.040149209 |
| MRPL34 | NR_110635.1 | LINC00687 | 0.896751261 | 1.960921501 | 0.049888181 |
| MRPL34 | NR_110930.1 | LOC101927814 | -0.80956313 | -1.8111306 | 0.070120638 |
| MRPL34 | NR_117098.1 | LINC01353 | 0.848599052 | 1.89668011 | 0.05787017 |
| MRPL34 | NR_120423.1 | LOC101929140 | -0.866005601 | -1.956047489 | 0.05045956 |
| MRPL34 | NR_125407.1 | LOC102724604 | 0.855870607 | 1.929868659 | 0.053623114 |
| MRPL34 | NR_125420.1 | LOC101927588 | -0.824828433 | -1.871055079 | 0.061337447 |
| MRPL34 | NR_125925.1 | LOC101929448 | 0.924744304 | 2.074321972 | 0.038049419 |
| MRPL34 | NR_126354.1 | LINC01331 | -0.851295714 | -1.903133971 | 0.057023066 |
| MRPL34 | NR_126380.1 | LINC01072 | 0.830510353 | 1.863696 | 0.062364407 |
| MRPL34 | NR_126412.1 | SCEL-AS1 | -0.80140418 | -1.802077099 | 0.071533276 |
| MRPL34 | NR_126413.1 | SCEL-AS1 | -0.818227178 | -1.838241347 | 0.06602685 |
| MRPL34 | NR_130144.1 | LOC104968399 | 0.914496513 | 2.04125092 | 0.041225893 |
| MRPL34 | NR_133658.1 | HTR3E-AS1 | -0.932038918 | -2.085246716 | 0.037046911 |
| MRPL34 | NR_134245.1 | LOC105379194 | 0.854740644 | 1.939070224 | 0.052492788 |
| MRPL34 | NR_134273.1 | LOC101929544 | 0.834802533 | 1.872669053 | 0.061114099 |
| MRPL34 | NR_135032.1 | LOC105369635 | 0.819915316 | 1.822442864 | 0.068387816 |
| MRPL34 | NR_135644.1 | LOC105371506 | -0.921658964 | -2.079007161 | 0.037616692 |
| MRPL34 | NR_138041.1 | LINC00384 | 0.946028038 | 2.138736175 | 0.032457041 |
| MRPL53 | ENST00000295549.4 | ENSG00000163364.5 | 0.836552795 | 1.866342327 | 0.061993487 |
| MRPL53 | ENST00000411804.1 | ENSG00000227415.1 | 0.899176181 | 2.007436791 | 0.044703175 |
| MRPL53 | ENST00000413969.1 | ENSG00000224189.2 | 0.839804511 | 1.857464056 | 0.06324516 |
| MRPL53 | ENST00000414896.1 | ENSG00000223374.1 | 0.838844666 | 1.872667758 | 0.061114278 |
| MRPL53 | ENST00000415106.1 | ENSG00000226733.1 | 0.955655076 | 2.133636931 | 0.032872512 |
| MRPL53 | ENST00000418621.1 | ENSG00000224731.1 | 0.926201493 | 2.050213069 | 0.040343643 |
| MRPL53 | ENST00000420044.1 | ENSG00000225956.1 | 0.869061644 | 1.947698348 | 0.051451068 |
| MRPL53 | ENST00000420830.1 | ENSG00000231512.1 | 0.818821057 | 1.827567822 | 0.067614439 |
| MRPL53 | ENST00000421006.1 | ENSG00000234548.1 | 0.867298814 | 1.928028754 | 0.05385155 |
| MRPL53 | ENST00000423796.1 | ENSG00000235146.2 | 0.86209234 | 1.930254794 | 0.053575275 |
| MRPL53 | ENST00000428160.1 | ENSG00000236897.1 | 0.806287997 | 1.77395377 | 0.076070798 |
| MRPL53 | ENST00000429080.1 | ENSG00000233047.1 | 0.88499877 | 2.008258837 | 0.044615794 |
| MRPL53 | ENST00000433174.1 | ENSG00000162947.4 | 0.859769395 | 1.931620455 | 0.053406369 |
| MRPL53 | ENST00000434292.1 | ENSG00000229796.1 | 0.937473001 | 2.093672731 | 0.036289144 |
| MRPL53 | ENST00000435832.1 | ENSG00000229201.1 | 0.820732133 | 1.833198442 | 0.066773066 |
| MRPL53 | ENST00000437680.1 | ENSG00000237133.1 | -0.877346338 | -1.956252686 | 0.050435395 |
| MRPL53 | ENST00000438173.2 | ENSG00000227733.4 | 0.899613383 | 2.034481541 | 0.041903068 |
| MRPL53 | ENST00000438969.2 | ENSG00000228031.2 | 0.923363792 | 2.078697025 | 0.037645207 |
| MRPL53 | ENST00000440038.2 | ENSG00000237094.7 | -0.877640493 | -1.965417774 | 0.049365904 |
| MRPL53 | ENST00000442017.1 | ENSG00000229660.1 | -0.875329026 | -1.959474522 | 0.050057241 |
| MRPL53 | ENST00000445233.1 | ENSG00000233928.1 | 0.870910293 | 1.933663532 | 0.05315451 |
| MRPL53 | ENST00000448001.1 | ENSG00000229639.1 | 0.826240117 | 1.837416257 | 0.066148469 |

| | | | | | |
|---|---|---|---|---|---|
| MRPL53 | ENST00000448858.1 | ENSG00000237734.1 | 0.809099598 | 1.814731046 | 0.069565252 |
| MRPL53 | ENST00000449749.1 | ENSG00000230834.1 | 0.888692147 | 2.027028109 | 0.042659538 |
| MRPL53 | ENST00000450696.1 | ENSG00000235146.2 | 0.819608339 | 1.84173839 | 0.065513426 |
| MRPL53 | ENST00000451556.2 | ENSG00000228386.2 | 0.804891723 | 1.817949198 | 0.069071898 |
| MRPL53 | ENST00000455373.1 | ENSG00000226097.1 | 0.815702321 | 1.82809839 | 0.067534787 |
| MRPL53 | ENST00000469846.2 | ENSG00000206573.4 | -0.875498727 | -1.936924866 | 0.052754528 |
| MRPL53 | ENST00000480904.2 | ENSG00000206573.4 | -0.882928638 | -1.962965015 | 0.049650239 |
| MRPL53 | ENST00000483283.1 | ENSG00000240571.1 | 0.922620518 | 2.072989009 | 0.038173302 |
| MRPL53 | ENST00000498199.1 | ENSG00000206573.4 | -0.828962818 | -1.849034367 | 0.06445285 |
| MRPL53 | ENST00000501133.2 | ENSG00000246560.2 | 0.905783515 | 2.015737133 | 0.043827471 |
| MRPL53 | ENST00000502684.1 | ENSG00000251670.1 | -0.853175494 | -1.926943044 | 0.053986728 |
| MRPL53 | ENST00000505575.1 | ENSG00000248939.1 | -0.833798151 | -1.855076684 | 0.063585277 |
| MRPL53 | ENST00000506723.2 | ENSG00000249484.4 | 0.886919409 | 2.001484979 | 0.045340151 |
| MRPL53 | ENST00000507857.2 | ENSG00000251055.2 | 0.861633022 | 1.913562844 | 0.055676036 |
| MRPL53 | ENST00000508191.1 | ENSG00000250910.3 | 0.942682464 | 2.095546914 | 0.036122403 |
| MRPL53 | ENST00000508414.1 | ENSG00000248173.1 | 0.860252588 | 1.920459852 | 0.05479984 |
| MRPL53 | ENST00000508823.1 | ENSG00000250716.1 | 0.923228507 | 2.08107938 | 0.037426641 |
| MRPL53 | ENST00000509629.1 | ENSG00000250164.1 | -0.876416535 | -1.948033088 | 0.051411004 |
| MRPL53 | ENST00000514270.1 | ENSG00000249295.1 | 0.877879412 | 1.960090141 | 0.049985255 |
| MRPL53 | ENST00000518339.1 | ENSG00000253470.1 | 0.880451649 | 1.97262584 | 0.048538207 |
| MRPL53 | ENST00000518837.1 | ENSG00000253947.1 | 0.840705026 | 1.868175078 | 0.061737671 |
| MRPL53 | ENST00000520192.1 | ENSG00000253807.1 | 0.910711692 | 2.025932041 | 0.042771749 |
| MRPL53 | ENST00000521294.1 | ENSG00000253664.1 | -0.917996181 | -2.055650634 | 0.039816203 |
| MRPL53 | ENST00000521378.1 | ENSG00000254222.1 | -0.871125757 | -1.958101701 | 0.05021808 |
| MRPL53 | ENST00000521411.2 | ENSG00000253496.2 | 0.891691936 | 2.00989913 | 0.044441866 |
| MRPL53 | ENST00000522281.1 | ENSG00000253376.1 | 0.881256501 | 1.969771896 | 0.048864519 |
| MRPL53 | ENST00000522300.1 | ENSG00000249484.4 | 0.950885888 | 2.140328614 | 0.032328219 |
| MRPL53 | ENST00000522426.1 | ENSG00000253538.1 | 0.897996074 | 1.990249106 | 0.046563501 |
| MRPL53 | ENST00000523806.1 | ENSG00000253616.1 | -0.870831987 | -1.933372321 | 0.053190348 |
| MRPL53 | ENST00000523935.1 | ENSG00000253567.1 | 0.91261131 | 2.012364169 | 0.044181563 |
| MRPL53 | ENST00000527100.1 | ENSG00000255015.1 | 0.873946928 | 1.960043166 | 0.049990745 |
| MRPL53 | ENST00000527274.2 | ENSG00000255517.2 | -0.949348132 | -2.117810116 | 0.034191152 |
| MRPL53 | ENST00000527727.1 | ENSG00000255227.1 | 0.850095355 | 1.900775289 | 0.057331452 |
| MRPL53 | ENST00000531157.1 | ENSG00000254754.1 | 0.92014372 | 2.060829936 | 0.039319267 |
| MRPL53 | ENST00000531977.1 | ENSG00000224023.6 | 0.94030284 | 2.08429808 | 0.037133062 |
| MRPL53 | ENST00000535315.1 | ENSG00000250748.2 | 0.870921642 | 1.935904777 | 0.052879365 |
| MRPL53 | ENST00000535914.1 | ENSG00000256894.1 | 0.875239508 | 1.958677195 | 0.050150603 |
| MRPL53 | ENST00000538641.1 | ENSG00000256422.1 | 0.843149549 | 1.905952867 | 0.056656321 |
| MRPL53 | ENST00000540024.1 | ENSG00000255693.1 | -0.890711386 | -1.982192936 | 0.047457651 |
| MRPL53 | ENST00000541391.1 | ENSG00000256268.1 | 0.985680679 | 2.193467716 | 0.028273698 |
| MRPL53 | ENST00000548210.1 | ENSG00000257784.1 | -0.821502875 | -1.842248663 | 0.065438785 |
| MRPL53 | ENST00000549616.1 | ENSG00000258168.1 | 0.910460382 | 2.027266255 | 0.042635191 |
| MRPL53 | ENST00000550805.1 | ENSG00000244306.5 | -0.817792677 | -1.835201061 | 0.066475904 |
| MRPL53 | ENST00000551135.1 | ENSG00000258294.1 | 0.829277032 | 1.842881459 | 0.06534632 |
| MRPL53 | ENST00000552261.1 | ENSG00000257959.1 | 0.950621781 | 2.115130105 | 0.03441886 |
| MRPL53 | ENST00000552541.1 | ENSG00000258294.1 | 0.956688355 | 2.126423736 | 0.033467993 |
| MRPL53 | ENST00000552558.1 | ENSG00000257947.1 | 0.904781906 | 2.036142799 | 0.041736018 |
| MRPL53 | ENST00000552634.1 | ENSG00000257496.1 | -0.837159243 | -1.877330763 | 0.060472774 |
| MRPL53 | ENST00000553477.1 | ENSG00000259123.1 | 0.930010427 | 2.086477746 | 0.036935369 |
| MRPL53 | ENST00000555913.1 | ENSG00000259077.1 | 0.853824865 | 1.917575124 | 0.055164907 |
| MRPL53 | ENST00000556458.1 | ENSG00000258504.2 | -0.815890078 | -1.810052142 | 0.070287702 |

| | | | | | |
|---|---|---|---|---|---|
| MRPL53 | ENST00000557368.1 | ENSG00000258444.1 | -0.85270049 | -1.912637075 | 0.05579453 |
| MRPL53 | ENST00000562582.1 | ENSG00000259779.1 | 0.825701011 | 1.844058577 | 0.065174604 |
| MRPL53 | ENST00000562834.1 | ENSG00000261116.1 | 0.842956203 | 1.90260698 | 0.057091848 |
| MRPL53 | ENST00000563342.1 | ENSG00000259914.1 | 0.878329937 | 1.976449315 | 0.048103911 |
| MRPL53 | ENST00000563570.1 | ENSG00000259961.1 | -0.87151357 | -1.959125609 | 0.050098078 |
| MRPL53 | ENST00000563855.1 | ENSG00000260658.1 | 0.893597064 | 1.988829609 | 0.046720013 |
| MRPL53 | ENST00000565055.1 | ENSG00000259912.1 | -0.882969847 | -1.981182189 | 0.047570846 |
| MRPL53 | ENST00000565310.1 | ENSG00000261118.1 | -0.887680572 | -2.005907322 | 0.044866137 |
| MRPL53 | ENST00000565735.1 | ENSG00000261213.1 | 0.914994297 | 2.051465624 | 0.040221624 |
| MRPL53 | ENST00000566449.1 | ENSG00000259791.1 | 0.848317769 | 1.883907048 | 0.05957755 |
| MRPL53 | ENST00000568410.1 | ENSG00000260277.1 | 0.909758618 | 2.047393351 | 0.040619479 |
| MRPL53 | ENST00000569849.1 | ENSG00000260640.1 | 0.89122217 | 1.987918653 | 0.046820687 |
| MRPL53 | ENST00000572417.1 | ENSG00000263171.1 | 0.817224271 | 1.845889474 | 0.064908255 |
| MRPL53 | ENST00000573861.1 | ENSG00000263320.1 | 0.925577491 | 2.089643232 | 0.03664986 |
| MRPL53 | ENST00000576021.1 | ENSG00000262413.1 | -0.975944009 | -2.174860982 | 0.029640536 |
| MRPL53 | ENST00000578035.1 | ENSG00000266743.1 | -0.824973809 | -1.856949908 | 0.063318281 |
| MRPL53 | ENST00000578334.1 | ENSG00000265148.1 | 0.855839839 | 1.91959645 | 0.054908893 |
| MRPL53 | ENST00000578349.1 | ENSG00000263688.1 | 0.857755877 | 1.922451646 | 0.054548952 |
| MRPL53 | ENST00000578572.1 | ENSG00000196295.7 | 0.914175765 | 2.028625149 | 0.042496485 |
| MRPL53 | ENST00000579775.1 | ENSG00000264108.1 | -0.915417689 | -2.053461778 | 0.040027813 |
| MRPL53 | ENST00000583826.1 | ENSG00000265148.1 | 0.83389222 | 1.876527307 | 0.060582909 |
| MRPL53 | ENST00000583841.1 | ENSG00000265148.1 | 0.827635714 | 1.826749016 | 0.067737515 |
| MRPL53 | ENST00000585684.1 | ENSG00000267057.1 | 0.92496721 | 2.050056701 | 0.040358898 |
| MRPL53 | ENST00000588842.1 | ENSG00000235779.3 | 0.873208523 | 1.958770782 | 0.050139637 |
| MRPL53 | ENST00000589983.1 | ENSG00000267057.1 | 0.888205324 | 1.967308287 | 0.04914768 |
| MRPL53 | ENST00000590357.1 | ENSG00000267175.1 | -0.879107793 | -1.974681088 | 0.04830435 |
| MRPL53 | ENST00000591469.1 | ENSG00000267374.1 | 0.918236829 | 2.033190413 | 0.042033289 |
| MRPL53 | ENST00000591621.1 | ENSG00000232116.2 | 0.96259548 | 2.16010891 | 0.030764239 |
| MRPL53 | ENST00000592022.1 | ENSG00000267383.2 | 0.95553033 | 2.14800366 | 0.031713465 |
| MRPL53 | ENST00000592045.1 | ENSG00000267057.1 | 0.873249308 | 1.959270986 | 0.05008106 |
| MRPL53 | ENST00000592431.1 | ENSG00000267475.1 | -0.812956751 | -1.800886766 | 0.071720729 |
| MRPL53 | ENST00000593175.1 | ENSG00000229036.3 | 0.902184081 | 1.998317907 | 0.045682205 |
| MRPL53 | ENST00000593486.1 | ENSG00000250910.3 | 0.92690944 | 2.068825578 | 0.038562457 |
| MRPL53 | ENST00000593599.1 | ENSG00000231898.4 | 0.925488366 | 2.077020571 | 0.037799661 |
| MRPL53 | ENST00000594776.1 | ENSG00000269807.1 | -0.897285589 | -2.015596035 | 0.043842235 |
| MRPL53 | ENST00000594850.1 | ENSG00000268093.1 | -0.805632086 | -1.804609332 | 0.071135837 |
| MRPL53 | ENST00000596567.1 | ENSG00000226647.2 | 0.852931545 | 1.901314671 | 0.057260809 |
| MRPL53 | ENST00000598065.1 | ENSG00000231731.3 | -0.840138101 | -1.877103146 | 0.060503958 |
| MRPL53 | ENST00000599050.1 | ENSG00000268366.1 | 0.874492464 | 1.925688058 | 0.054143336 |
| MRPL53 | ENST00000602736.1 | ENSG00000269976.1 | 0.925855323 | 2.054701421 | 0.039907853 |
| MRPL53 | ENST00000605692.1 | ENSG00000270810.1 | -0.878153652 | -1.953306993 | 0.050783229 |
| MRPL53 | ENST00000605780.1 | ENSG00000270755.1 | -0.831088724 | -1.86415519 | 0.062299914 |
| MRPL53 | ENST00000606869.1 | ENSG00000272349.1 | 0.966007374 | 2.17817778 | 0.029392804 |
| MRPL53 | ENST00000606898.1 | ENSG00000272094.1 | -0.874863809 | -1.975943038 | 0.048161229 |
| MRPL53 | ENST00000607025.1 | ENSG00000271973.1 | -0.823016821 | -1.845983855 | 0.06489455 |
| MRPL53 | ENST00000607715.1 | ENSG00000271788.1 | 0.839367701 | 1.853474967 | 0.063814311 |
| MRPL53 | ENST00000608088.1 | ENSG00000272632.1 | 0.812683502 | 1.823056946 | 0.068294766 |
| MRPL53 | ENST00000608943.1 | ENSG00000273368.1 | 0.862091364 | 1.907882768 | 0.05640637 |
| MRPL53 | ENST00000609349.1 | ENSG00000272861.1 | 0.844907361 | 1.901554645 | 0.057229402 |
| MRPL53 | ENST00000609807.1 | ENSG00000272700.1 | 0.882328197 | 1.977701813 | 0.047962356 |
| MRPL53 | NR_027402.1 | FAM223B | -0.932964159 | -2.071324117 | 0.038328517 |

| | | | | | |
|---|---|---|---|---|---|
| MRPL53 | NR_027440.1 | LOC100272217 | -0.859674207 | -1.921204447 | 0.054705938 |
| MRPL53 | NR_033914.1 | LINC00254 | 0.996899358 | 2.231482245 | 0.025649202 |
| MRPL53 | NR_038194.1 | LINC00583 | 0.898952871 | 1.998413427 | 0.045671857 |
| MRPL53 | NR_040001.2 | LINC01116 | 0.919637181 | 2.075836864 | 0.037909042 |
| MRPL53 | NR_046766.1 | ATP2B2-IT2 | 0.889015672 | 2.000486337 | 0.045447774 |
| MRPL53 | NR_046871.1 | LINC00333 | 0.925491445 | 2.083312503 | 0.037222748 |
| MRPL53 | NR_047698.1 | VWC2L-IT1 | 0.971441701 | 2.199887196 | 0.027814899 |
| MRPL53 | NR_103776.1 | CHRM3-AS2 | 0.801072788 | 1.803271129 | 0.071345645 |
| MRPL53 | NR_104618.1 | LINC01017 | 0.856473246 | 1.929683648 | 0.053646047 |
| MRPL53 | NR_109877.1 | LINC01470 | 0.933512358 | 2.103269821 | 0.03544219 |
| MRPL53 | NR_110123.1 | GRM7-AS3 | 0.954155491 | 2.127428302 | 0.033384513 |
| MRPL53 | NR_110245.1 | LOC101929282 | 0.812952682 | 1.80452098 | 0.071149673 |
| MRPL53 | NR_110556.1 | LOC102724890 | 0.851385964 | 1.906091987 | 0.056638272 |
| MRPL53 | NR_110824.1 | LINC01986 | 0.91078959 | 2.031347592 | 0.042219747 |
| MRPL53 | NR_120566.1 | LOC101928896 | 0.892079578 | 2.027271896 | 0.042634614 |
| MRPL53 | NR_125769.1 | LINC01269 | -0.802774148 | -1.785754216 | 0.074139063 |
| MRPL53 | NR_131186.1 | LOC105377348 | 0.881047237 | 1.994591193 | 0.046087485 |
| MRPL53 | NR_131204.1 | XACT | -0.931998666 | -2.09398582 | 0.036261243 |
| MRPL53 | NR_133930.1 | LOC105375556 | 0.814595859 | 1.809309051 | 0.070403004 |
| MRPL53 | NR_134573.1 | GS1-124K5.4 | 0.818113675 | 1.84640471 | 0.064833464 |
| MRPL53 | NR_134610.1 | LOC105375014 | -0.803147575 | -1.780887267 | 0.074930869 |
| MRPL53 | NR_134632.1 | LOC105373051 | -0.903438993 | -2.01356883 | 0.044054822 |
| MRPL53 | NR_135549.1 | LOC101929411 | 0.965094408 | 2.147360923 | 0.031764559 |
| MRPL53 | NR_136178.1 | LOC101928166 | 0.851291787 | 1.908698277 | 0.056301026 |
| MRPL53 | NR_136215.1 | VCAN-AS1 | 0.831527778 | 1.860162859 | 0.062862488 |
| MRPL53 | NR_136218.1 | MEF2C-AS1 | 0.960077526 | 2.130131221 | 0.033160781 |
| MSRB1 | ENST00000318291.4 | ENSG00000177406.4 | 0.813983323 | 1.82712638 | 0.06768077 |
| MSRB1 | ENST00000381106.4 | ENSG00000205663.5 | -0.975560479 | -2.16196384 | 0.030620963 |
| MSRB1 | ENST00000381475.3 | ENSG00000215863.2 | -0.890536265 | -1.984225906 | 0.047230662 |
| MSRB1 | ENST00000412348.1 | ENSG00000228959.1 | -0.862349988 | -1.941919044 | 0.052146902 |
| MSRB1 | ENST00000412809.1 | ENSG00000229938.1 | -0.843290204 | -1.897248156 | 0.057795194 |
| MSRB1 | ENST00000417654.1 | ENSG00000224893.1 | 0.981427348 | 2.19032116 | 0.028500953 |
| MSRB1 | ENST00000423428.1 | ENSG00000224048.1 | -0.881787324 | -1.964100678 | 0.049518418 |
| MSRB1 | ENST00000424735.1 | ENSG00000237457.2 | -0.848390562 | -1.890616063 | 0.058675614 |
| MSRB1 | ENST00000426237.2 | ENSG00000235527.2 | 0.862863232 | 1.945617597 | 0.051700692 |
| MSRB1 | ENST00000435287.1 | ENSG00000227220.1 | 0.881717447 | 1.949128737 | 0.051280053 |
| MSRB1 | ENST00000435434.1 | ENSG00000231233.1 | 0.800026672 | 1.785342093 | 0.074205845 |
| MSRB1 | ENST00000436515.1 | ENSG00000224521.1 | -0.848888502 | -1.875122269 | 0.060775905 |
| MSRB1 | ENST00000438623.1 | ENSG00000224521.1 | -0.872706243 | -1.963624133 | 0.049573697 |
| MSRB1 | ENST00000448858.1 | ENSG00000237734.1 | -0.802009251 | -1.795533492 | 0.072568742 |
| MSRB1 | ENST00000449154.1 | ENSG00000226969.1 | 0.820185748 | 1.8485935 | 0.064516532 |
| MSRB1 | ENST00000450696.1 | ENSG00000235146.2 | -0.899687195 | -1.992671002 | 0.046297485 |
| MSRB1 | ENST00000457998.2 | ENSG00000233006.2 | 0.897002761 | 1.981982907 | 0.047481154 |
| MSRB1 | ENST00000489557.2 | ENSG00000257045.1 | 0.830734188 | 1.85254664 | 0.063947367 |
| MSRB1 | ENST00000503505.1 | ENSG00000248629.1 | -0.853279902 | -1.923704621 | 0.054391618 |
| MSRB1 | ENST00000504578.1 | ENSG00000251513.1 | -0.945205731 | -2.106648594 | 0.035148051 |
| MSRB1 | ENST00000506723.2 | ENSG00000249484.4 | -0.810630778 | -1.796108859 | 0.072477206 |
| MSRB1 | ENST00000509192.1 | ENSG00000250765.1 | 0.812904839 | 1.802238755 | 0.07150785 |
| MSRB1 | ENST00000510941.1 | ENSG00000251339.1 | -0.867839144 | -1.975810529 | 0.04817624 |
| MSRB1 | ENST00000515128.1 | ENSG00000248215.1 | -0.894137119 | -1.999629548 | 0.045540281 |
| MSRB1 | ENST00000528887.1 | ENSG00000254501.1 | 0.908534348 | 2.040227856 | 0.041327636 |

| | | | | | |
|---|---|---|---|---|---|
| MSRB1 | ENST00000537850.1 | ENSG00000251002.3 | 0.855045267 | 1.903116098 | 0.057025398 |
| MSRB1 | ENST00000543494.1 | ENSG00000256514.1 | 0.821198595 | 1.838313951 | 0.066016156 |
| MSRB1 | ENST00000549683.1 | ENSG00000257953.1 | 0.914859635 | 2.041562914 | 0.041194907 |
| MSRB1 | ENST00000555460.1 | ENSG00000259042.1 | 0.858821863 | 1.92586453 | 0.054121291 |
| MSRB1 | ENST00000563018.1 | ENSG00000260193.1 | 0.912453179 | 2.034480368 | 0.041903186 |
| MSRB1 | ENST00000564038.1 | ENSG00000261760.2 | 0.861829084 | 1.914699241 | 0.05553087 |
| MSRB1 | ENST00000566449.1 | ENSG00000259791.1 | -0.864069787 | -1.919594634 | 0.054909122 |
| MSRB1 | ENST00000567067.1 | ENSG00000261600.1 | -0.88364039 | -1.973239623 | 0.048468268 |
| MSRB1 | ENST00000576086.1 | ENSG00000262823.1 | 0.877158122 | 1.970429738 | 0.04878914 |
| MSRB1 | ENST00000576554.1 | ENSG00000262413.1 | 0.826547859 | 1.855420411 | 0.063536215 |
| MSRB1 | ENST00000584705.1 | ENSG00000264569.1 | 0.845464013 | 1.894283249 | 0.058187423 |
| MSRB1 | ENST00000585559.1 | ENSG00000267117.1 | 0.838748087 | 1.887850625 | 0.059046007 |
| MSRB1 | ENST00000585810.1 | ENSG00000236172.2 | 0.825222515 | 1.834124407 | 0.066635529 |
| MSRB1 | ENST00000592400.1 | ENSG00000267735.1 | 0.891539414 | 1.987685075 | 0.046846531 |
| MSRB1 | ENST00000592720.1 | ENSG00000267232.1 | 0.825491675 | 1.839672984 | 0.065816262 |
| MSRB1 | ENST00000592816.1 | ENSG00000236172.2 | 0.840670654 | 1.889830134 | 0.058780682 |
| MSRB1 | ENST00000595478.1 | ENSG00000237031.3 | -0.94102909 | -2.084020496 | 0.037158303 |
| MSRB1 | ENST00000598092.1 | ENSG00000228065.6 | -0.880382398 | -1.9707165 | 0.048756312 |
| MSRB1 | ENST00000600726.1 | ENSG00000267858.1 | 0.852400567 | 1.923496886 | 0.054417677 |
| MSRB1 | ENST00000601692.1 | ENSG00000267874.1 | -0.857284635 | -1.907667137 | 0.056434252 |
| MSRB1 | ENST00000602620.1 | ENSG00000215386.6 | -0.903786852 | -2.03578395 | 0.041772055 |
| MSRB1 | ENST00000602949.1 | ENSG00000270030.1 | 0.840584748 | 1.872601466 | 0.061123439 |
| MSRB1 | ENST00000607549.1 | ENSG00000272293.1 | -0.873533938 | -1.934598049 | 0.05303964 |
| MSRB1 | ENST00000607839.1 | ENSG00000272030.1 | 0.812515058 | 1.786750367 | 0.073977844 |
| MSRB1 | ENST00000607943.1 | ENSG00000273188.1 | 0.806250293 | 1.795512999 | 0.072572004 |
| MSRB1 | ENST00000608088.1 | ENSG00000272632.1 | -0.843602851 | -1.865507561 | 0.062110293 |
| MSRB1 | ENST00000608259.1 | ENSG00000272627.1 | -0.815705346 | -1.810403622 | 0.070233218 |
| MSRB1 | ENST00000608489.1 | ENSG00000272716.1 | 0.883418148 | 1.966438708 | 0.049247956 |
| MSRB1 | ENST00000609807.1 | ENSG00000272700.1 | -0.835516365 | -1.855721229 | 0.063493303 |
| MSRB1 | ENST00000609924.1 | ENSG00000272688.1 | 0.855005247 | 1.932135693 | 0.05334276 |
| MSRB1 | ENST00000609976.1 | ENSG00000272582.1 | 0.904947119 | 2.030469438 | 0.042308845 |
| MSRB1 | NR_108036.1 | CFAP58-AS1 | 0.824993966 | 1.865286555 | 0.062141249 |
| MSRB1 | NR_110117.1 | LOC101927769 | -0.850721403 | -1.912121483 | 0.055860614 |
| MSRB1 | NR_110556.1 | LOC102724890 | -0.813136 | -1.816473743 | 0.069297732 |
| MSRB1 | NR_134573.1 | GS1-124K5.4 | -0.841075414 | -1.878154577 | 0.060360022 |
| MSRB1 | NR_135584.1 | LOC101927596 | 0.872826002 | 1.963927646 | 0.049538483 |
| MSRB1 | NR_135679.1 | LOC105370829 | -0.801908771 | -1.770810171 | 0.076592274 |
| MSRB1 | NR_144459.1 | ARSD-AS1 | 0.818467129 | 1.821789402 | 0.068486946 |
| NANOS3 | ENST00000414896.1 | ENSG00000223374.1 | -0.814678864 | -1.816762586 | 0.069253474 |
| NANOS3 | ENST00000416329.1 | ENSG00000233184.2 | 0.807227819 | 1.799033825 | 0.07201333 |
| NANOS3 | ENST00000417260.1 | ENSG00000231734.4 | -0.805204217 | -1.787386863 | 0.073874983 |
| NANOS3 | ENST00000421207.1 | ENSG00000231768.1 | 0.847803497 | 1.891048854 | 0.058617823 |
| NANOS3 | ENST00000423428.1 | ENSG00000224048.1 | -0.872466234 | -1.938479463 | 0.052564754 |
| NANOS3 | ENST00000425124.1 | ENSG00000232336.1 | 0.941232302 | 2.100132283 | 0.035717206 |
| NANOS3 | ENST00000425624.1 | ENSG00000223779.4 | 0.806652415 | 1.825034847 | 0.067995771 |
| NANOS3 | ENST00000426237.2 | ENSG00000235527.2 | 0.818948469 | 1.825764393 | 0.067885759 |
| NANOS3 | ENST00000436982.2 | ENSG00000235335.2 | -0.826072841 | -1.862654803 | 0.062510849 |
| NANOS3 | ENST00000440492.1 | ENSG00000233975.1 | 0.841135856 | 1.880177427 | 0.060083901 |
| NANOS3 | ENST00000441160.1 | ENSG00000228437.1 | -0.842961976 | -1.877626004 | 0.060432346 |
| NANOS3 | ENST00000446107.1 | ENSG00000227029.1 | 0.819016914 | 1.848398287 | 0.064544746 |
| NANOS3 | ENST00000454100.1 | ENSG00000236943.2 | 0.953440307 | 2.142491513 | 0.032153951 |

| | | | | | |
|---|---|---|---|---|---|
| NANOS3 | ENST00000506791.1 | ENSG00000251131.1 | 0.832359246 | 1.848668713 | 0.064505664 |
| NANOS3 | ENST00000509192.1 | ENSG00000250765.1 | 0.880234951 | 1.981264698 | 0.047561597 |
| NANOS3 | ENST00000509453.1 | ENSG00000249145.1 | 0.879612352 | 1.984105706 | 0.047244057 |
| NANOS3 | ENST00000518473.1 | ENSG00000253985.1 | 0.822942748 | 1.817649106 | 0.069117781 |
| NANOS3 | ENST00000520603.1 | ENSG00000254001.1 | -0.866268524 | -1.930923873 | 0.053492467 |
| NANOS3 | ENST00000524286.1 | ENSG00000253658.1 | -0.839815946 | -1.872560805 | 0.061129058 |
| NANOS3 | ENST00000550263.1 | ENSG00000257605.1 | 0.866770738 | 1.945118287 | 0.051760743 |
| NANOS3 | ENST00000554859.1 | ENSG00000259088.1 | 0.857866984 | 1.918816051 | 0.055007618 |
| NANOS3 | ENST00000556397.1 | ENSG00000258654.1 | 0.886795212 | 1.97661908 | 0.048084704 |
| NANOS3 | ENST00000558618.1 | ENSG00000259209.1 | 0.825474776 | 1.851523527 | 0.064094273 |
| NANOS3 | ENST00000559959.1 | ENSG00000259396.1 | 0.871471853 | 1.959590167 | 0.050043712 |
| NANOS3 | ENST00000567395.1 | ENSG00000261090.1 | 0.818143954 | 1.833915776 | 0.066666497 |
| NANOS3 | ENST00000568332.1 | ENSG00000260256.1 | 0.805053099 | 1.815269781 | 0.069482461 |
| NANOS3 | ENST00000568756.2 | ENSG00000261760.2 | 0.86420829 | 1.950972973 | 0.051060261 |
| NANOS3 | ENST00000582558.1 | ENSG00000264569.1 | 0.936114571 | 2.067125007 | 0.038722376 |
| NANOS3 | ENST00000584705.1 | ENSG00000264569.1 | 0.97810274 | 2.179220807 | 0.029315269 |
| NANOS3 | ENST00000585559.1 | ENSG00000267117.1 | 0.821147947 | 1.81979024 | 0.068790955 |
| NANOS3 | ENST00000587693.1 | ENSG00000267373.1 | -0.85558992 | -1.888793495 | 0.058919505 |
| NANOS3 | ENST00000588945.1 | ENSG00000267275.1 | 0.817835673 | 1.824632176 | 0.068056554 |
| NANOS3 | ENST00000589395.1 | ENSG00000267143.1 | 0.824206771 | 1.850113773 | 0.064297153 |
| NANOS3 | ENST00000592400.1 | ENSG00000267735.1 | 0.895428431 | 1.993309909 | 0.046227522 |
| NANOS3 | ENST00000592720.1 | ENSG00000267232.1 | 0.841605329 | 1.890863242 | 0.058642602 |
| NANOS3 | ENST00000594091.1 | ENSG00000232732.5 | 0.825332275 | 1.846802334 | 0.064775794 |
| NANOS3 | ENST00000595478.1 | ENSG00000237031.3 | -0.875568614 | -1.962993307 | 0.049646952 |
| NANOS3 | ENST00000596091.1 | ENSG00000227733.4 | -0.82117792 | -1.844446115 | 0.065118152 |
| NANOS3 | ENST00000597530.1 | ENSG00000228401.3 | 0.809565624 | 1.793442679 | 0.072902168 |
| NANOS3 | ENST00000597906.1 | ENSG00000268566.1 | -0.892146551 | -1.974679454 | 0.048304536 |
| NANOS3 | ENST00000601692.1 | ENSG00000267874.1 | -0.898859214 | -2.034748751 | 0.04187616 |
| NANOS3 | ENST00000602949.1 | ENSG00000270030.1 | 0.942078242 | 2.114716385 | 0.034454127 |
| NANOS3 | ENST00000605082.1 | ENSG00000270426.1 | 0.910253023 | 2.022107849 | 0.043165211 |
| NANOS3 | ENST00000606743.1 | ENSG00000272221.1 | 0.928380683 | 2.072703541 | 0.038199878 |
| NANOS3 | ENST00000606909.1 | ENSG00000271821.1 | 0.850311718 | 1.895001431 | 0.058092212 |
| NANOS3 | ENST00000607284.1 | ENSG00000272389.1 | 0.84619619 | 1.885583588 | 0.059351091 |
| NANOS3 | ENST00000608088.1 | ENSG00000272632.1 | -0.821805814 | -1.83946496 | 0.065846827 |
| NANOS3 | NR_026774.1 | LINC00239 | 0.804747192 | 1.790925803 | 0.073305202 |
| NANOS3 | NR_028324.1 | LINC01002 | 0.870849676 | 1.936929139 | 0.052754006 |
| NANOS3 | NR_037169.1 | LOC100507547 | 0.896037503 | 1.995921949 | 0.045942419 |
| NANOS3 | NR_037170.1 | LOC100507547 | 0.912558129 | 2.049357551 | 0.040427165 |
| NANOS3 | NR_045114.1 | PVRL3-AS1 | -0.860161495 | -1.936060493 | 0.052860292 |
| NANOS3 | NR_046369.1 | LOC100131626 | 0.877740007 | 1.960397165 | 0.049949387 |
| NANOS3 | NR_072981.1 | LINC00957 | 0.890692672 | 1.987960664 | 0.046816041 |
| NANOS3 | NR_072982.1 | LINC00957 | 0.903324962 | 2.018552518 | 0.04353375 |
| NANOS3 | NR_109770.1 | TONSL-AS1 | 0.880142125 | 1.946943215 | 0.051541543 |
| NANOS3 | NR_109885.1 | RALY-AS1 | 0.80913233 | 1.806808039 | 0.070792215 |
| NANOS3 | NR_134325.1 | LOC102723672 | 0.882470882 | 2.015393441 | 0.043863441 |
| NANOS3 | NR_135132.1 | HSPC324 | 0.822104226 | 1.833771643 | 0.066687899 |
| NCF1 | ENST00000418621.1 | ENSG00000224731.1 | -0.857573846 | -1.934734304 | 0.053022909 |
| NCF1 | ENST00000421597.1 | ENSG00000227851.1 | -0.823588288 | -1.862332063 | 0.062556299 |
| NCF1 | ENST00000422807.1 | ENSG00000227683.1 | -0.938767197 | -2.093238961 | 0.036327828 |
| NCF1 | ENST00000423796.1 | ENSG00000235146.2 | -0.860976122 | -1.9219523 | 0.05461176 |
| NCF1 | ENST00000424342.1 | ENSG00000234988.1 | 0.9894496 | 2.240389213 | 0.025065666 |

| | | | | | |
|---|---|---|---|---|---|
| NCF1 | ENST00000424678.1 | ENSG00000229600.1 | -0.830897086 | -1.845922699 | 0.06490343 |
| NCF1 | ENST00000425058.1 | ENSG00000226771.1 | -0.812816432 | -1.808940201 | 0.070460294 |
| NCF1 | ENST00000426302.1 | ENSG00000230454.1 | 0.812221155 | 1.804252884 | 0.071191672 |
| NCF1 | ENST00000430247.1 | ENSG00000232855.2 | -0.956072087 | -2.124797313 | 0.033603529 |
| NCF1 | ENST00000430751.1 | ENSG00000232222.1 | 0.804342878 | 1.798992051 | 0.072019938 |
| NCF1 | ENST00000434292.1 | ENSG00000229796.1 | -0.908531247 | -2.034416241 | 0.041909646 |
| NCF1 | ENST00000435828.1 | ENSG00000235612.1 | -0.854715329 | -1.931241792 | 0.053453158 |
| NCF1 | ENST00000438623.1 | ENSG00000224521.1 | -0.83565695 | -1.877932193 | 0.060390442 |
| NCF1 | ENST00000439072.1 | ENSG00000224516.1 | 0.823588288 | 1.843840171 | 0.065206436 |
| NCF1 | ENST00000440038.2 | ENSG00000237094.7 | 0.843211904 | 1.874333414 | 0.060884485 |
| NCF1 | ENST00000442017.1 | ENSG00000229660.1 | 0.930712782 | 2.068763311 | 0.038568303 |
| NCF1 | ENST00000442852.1 | ENSG00000237923.1 | 0.931157052 | 2.067154288 | 0.038719618 |
| NCF1 | ENST00000443066.2 | ENSG00000237633.2 | -0.832923599 | -1.890729105 | 0.058660515 |
| NCF1 | ENST00000445233.1 | ENSG00000233928.1 | -0.930088477 | -2.061342571 | 0.039270369 |
| NCF1 | ENST00000447111.1 | ENSG00000231903.1 | -0.901477645 | -2.021412639 | 0.043237067 |
| NCF1 | ENST00000449154.1 | ENSG00000226969.1 | 0.819451228 | 1.844486019 | 0.065112342 |
| NCF1 | ENST00000449903.1 | ENSG00000223872.1 | 0.819496204 | 1.822774887 | 0.068337493 |
| NCF1 | ENST00000451090.1 | ENSG00000235215.2 | -0.810049419 | -1.815539444 | 0.06944105 |
| NCF1 | ENST00000451697.1 | ENSG00000233823.1 | 0.806782562 | 1.787346781 | 0.073881457 |
| NCF1 | ENST00000455373.1 | ENSG00000226097.1 | -0.871794107 | -1.947555703 | 0.051468148 |
| NCF1 | ENST00000456499.1 | ENSG00000237640.1 | 0.843683268 | 1.876957681 | 0.060523894 |
| NCF1 | ENST00000457043.1 | ENSG00000231365.1 | -0.885951862 | -1.96847922 | 0.049012925 |
| NCF1 | ENST00000457998.2 | ENSG00000233006.2 | 0.847278004 | 1.885497336 | 0.059362724 |
| NCF1 | ENST00000479039.1 | ENSG00000241224.2 | 0.875975453 | 1.93611786 | 0.052853268 |
| NCF1 | ENST00000483283.1 | ENSG00000240571.1 | -0.894025921 | -2.003625565 | 0.045110185 |
| NCF1 | ENST00000503323.1 | ENSG00000249881.1 | 0.852547025 | 1.909525699 | 0.05619431 |
| NCF1 | ENST00000503470.1 | ENSG00000248559.1 | -0.826210363 | -1.84737126 | 0.064693352 |
| NCF1 | ENST00000503505.1 | ENSG00000248629.1 | -0.801824236 | -1.799758309 | 0.07189881 |
| NCF1 | ENST00000503987.1 | ENSG00000250075.1 | -0.91729752 | -2.051136505 | 0.040253655 |
| NCF1 | ENST00000504765.1 | ENSG00000249638.1 | -0.905100263 | -2.014042701 | 0.044005051 |
| NCF1 | ENST00000505575.1 | ENSG00000248939.1 | 0.866751148 | 1.932517122 | 0.053295711 |
| NCF1 | ENST00000505978.1 | ENSG00000249982.1 | 0.848960488 | 1.883569155 | 0.059623277 |
| NCF1 | ENST00000506379.1 | ENSG00000240152.2 | -0.856929958 | -1.918871665 | 0.055000577 |
| NCF1 | ENST00000506852.1 | ENSG00000250945.1 | -0.843580624 | -1.887575229 | 0.059082998 |
| NCF1 | ENST00000508191.1 | ENSG00000250910.3 | -0.813908212 | -1.832176562 | 0.06692512 |
| NCF1 | ENST00000508687.1 | ENSG00000250538.1 | -0.884413832 | -1.992832256 | 0.046279818 |
| NCF1 | ENST00000508823.1 | ENSG00000250716.1 | -0.896032583 | -1.999529592 | 0.045551083 |
| NCF1 | ENST00000512882.2 | ENSG00000251575.2 | -0.880994461 | -1.97748739 | 0.047986564 |
| NCF1 | ENST00000518837.1 | ENSG00000253947.1 | -0.800930466 | -1.821388343 | 0.068547846 |
| NCF1 | ENST00000520192.1 | ENSG00000253807.1 | -0.958431844 | -2.137355031 | 0.032569126 |
| NCF1 | ENST00000521725.1 | ENSG00000253396.1 | -0.957052256 | -2.14671813 | 0.031815728 |
| NCF1 | ENST00000522190.1 | ENSG00000254165.1 | -0.838872855 | -1.879331724 | 0.060199213 |
| NCF1 | ENST00000522281.1 | ENSG00000253376.1 | -0.852955924 | -1.90423178 | 0.056880005 |
| NCF1 | ENST00000522300.1 | ENSG00000249484.4 | -0.86135934 | -1.929941493 | 0.053614087 |
| NCF1 | ENST00000522390.1 | ENSG00000254262.1 | -0.884854354 | -1.996957941 | 0.045829752 |
| NCF1 | ENST00000522426.1 | ENSG00000253538.1 | -0.848932468 | -1.891970425 | 0.058494921 |
| NCF1 | ENST00000527274.2 | ENSG00000255517.2 | 0.834069044 | 1.859767586 | 0.062918415 |
| NCF1 | ENST00000527727.1 | ENSG00000255227.1 | -0.822492675 | -1.833145692 | 0.066780908 |
| NCF1 | ENST00000531977.1 | ENSG00000224023.6 | -0.837358644 | -1.881741559 | 0.059871114 |
| NCF1 | ENST00000535914.1 | ENSG00000256894.1 | -0.827010855 | -1.850602519 | 0.064226757 |
| NCF1 | ENST00000538641.1 | ENSG00000256422.1 | -0.808197371 | -1.820805758 | 0.068636389 |

| | | | | | |
|---|---|---|---|---|---|
| NCF1 | ENST00000546135.1 | ENSG00000256670.1 | 0.961851651 | 2.155906755 | 0.031090947 |
| NCF1 | ENST00000548210.1 | ENSG00000257784.1 | 0.868132924 | 1.942741482 | 0.052047402 |
| NCF1 | ENST00000550805.1 | ENSG00000244306.5 | 0.853005267 | 1.88794889 | 0.059032812 |
| NCF1 | ENST00000551067.1 | ENSG00000257891.1 | 0.968795155 | 2.134826014 | 0.032775225 |
| NCF1 | ENST00000553075.1 | ENSG00000257258.1 | -0.839934178 | -1.863020753 | 0.062459347 |
| NCF1 | ENST00000553537.1 | ENSG00000258481.1 | 0.955047737 | 2.124312367 | 0.033644032 |
| NCF1 | ENST00000553954.1 | ENSG00000259052.1 | 0.85778645 | 1.932979393 | 0.053238736 |
| NCF1 | ENST00000555460.1 | ENSG00000259042.1 | 0.924350539 | 2.08008786 | 0.037517475 |
| NCF1 | ENST00000556145.1 | ENSG00000258829.1 | 0.846665119 | 1.877919668 | 0.060392155 |
| NCF1 | ENST00000559041.1 | ENSG00000259713.1 | -0.872059986 | -1.924382615 | 0.054306642 |
| NCF1 | ENST00000560969.1 | ENSG00000259176.1 | -0.888685738 | -1.983995619 | 0.047256328 |
| NCF1 | ENST00000561039.1 | ENSG00000259536.1 | 0.881059281 | 1.957249473 | 0.050318145 |
| NCF1 | ENST00000561254.1 | ENSG00000259554.1 | -0.933829711 | -2.086587203 | 0.036925465 |
| NCF1 | ENST00000563855.1 | ENSG00000260658.1 | -0.896348175 | -2.012294705 | 0.04418888 |
| NCF1 | ENST00000569778.1 | ENSG00000260823.1 | 0.939884885 | 2.125632849 | 0.033533842 |
| NCF1 | ENST00000570700.1 | ENSG00000263011.1 | 0.811023248 | 1.79721381 | 0.072301683 |
| NCF1 | ENST00000573260.1 | ENSG00000262482.1 | -0.962535687 | -2.168456486 | 0.030123972 |
| NCF1 | ENST00000577360.1 | ENSG00000264273.1 | -0.815593125 | -1.805344575 | 0.071020778 |
| NCF1 | ENST00000580729.1 | ENSG00000266176.1 | 0.879964312 | 1.973217962 | 0.048470735 |
| NCF1 | ENST00000585684.1 | ENSG00000267057.1 | -0.903460558 | -2.030497367 | 0.042306009 |
| NCF1 | ENST00000588842.1 | ENSG00000235779.3 | -0.918162814 | -2.051461632 | 0.040222012 |
| NCF1 | ENST00000589983.1 | ENSG00000267057.1 | -0.812055367 | -1.824993258 | 0.068002047 |
| NCF1 | ENST00000591469.1 | ENSG00000267374.1 | -0.883494565 | -1.971743918 | 0.048638847 |
| NCF1 | ENST00000591621.1 | ENSG00000232116.2 | -0.81225724 | -1.801513585 | 0.071621968 |
| NCF1 | ENST00000592045.1 | ENSG00000267057.1 | -0.862641661 | -1.914431091 | 0.055565096 |
| NCF1 | ENST00000597680.1 | ENSG00000269574.1 | -0.90932134 | -2.045550183 | 0.040800647 |
| NCF1 | ENST00000597755.1 | ENSG00000236194.2 | -0.945008875 | -2.100596634 | 0.03567639 |
| NCF1 | ENST00000598065.1 | ENSG00000231731.3 | 0.980540506 | 2.15056238 | 0.031510757 |
| NCF1 | ENST00000599050.1 | ENSG00000268366.1 | -0.811077765 | -1.819209438 | 0.068879484 |
| NCF1 | ENST00000602051.1 | ENSG00000227877.2 | 0.82714949 | 1.863542107 | 0.062386034 |
| NCF1 | ENST00000602881.1 | ENSG00000269965.1 | -0.949337125 | -2.099027629 | 0.035814465 |
| NCF1 | ENST00000603949.1 | ENSG00000270332.1 | -0.807022964 | -1.831715492 | 0.066993819 |
| NCF1 | ENST00000604312.1 | ENSG00000270947.1 | -0.943313959 | -2.114650751 | 0.034459725 |
| NCF1 | ENST00000606010.1 | ENSG00000272249.1 | -0.894778649 | -1.993066979 | 0.046254113 |
| NCF1 | ENST00000607135.1 | ENSG00000272112.1 | -0.802532129 | -1.808649255 | 0.070505511 |
| NCF1 | ENST00000607665.1 | ENSG00000272254.1 | -0.869206666 | -1.916576334 | 0.055291777 |
| NCF1 | ENST00000608133.1 | ENSG00000273193.1 | -0.920052396 | -2.070145802 | 0.038438693 |
| NCF1 | ENST00000608259.1 | ENSG00000272627.1 | -0.825991009 | -1.848997949 | 0.064458109 |
| NCF1 | ENST00000608465.1 | ENSG00000272758.1 | -0.890648875 | -1.965351262 | 0.049373596 |
| NCF1 | ENST00000608934.1 | ENSG00000273063.1 | -0.87713568 | -1.946166268 | 0.05163477 |
| NCF1 | ENST00000609349.1 | ENSG00000272861.1 | -0.804421045 | -1.799970636 | 0.071865275 |
| NCF1 | ENST00000609807.1 | ENSG00000272700.1 | -0.8335664 | -1.861507229 | 0.062672581 |
| NCF1 | NR_027067.1 | LINC00114 | 0.927495519 | 2.062886502 | 0.039123413 |
| NCF1 | NR_027402.1 | FAM223B | 0.916745979 | 2.06891125 | 0.038554416 |
| NCF1 | NR_027425.1 | FAM66D | 0.978155921 | 2.173056566 | 0.02977606 |
| NCF1 | NR_027440.1 | LOC100272217 | 0.945046047 | 2.109773724 | 0.034877851 |
| NCF1 | NR_046748.1 | ARHGAP31-AS1 | -0.967434944 | -2.146558988 | 0.031828408 |
| NCF1 | NR_047698.1 | VWC2L-IT1 | -0.807459616 | -1.837578792 | 0.066124496 |
| NCF1 | NR_102738.1 | LINC00911 | 0.897782497 | 2.013597278 | 0.044051832 |
| NCF1 | NR_109877.1 | LINC01470 | -0.863959622 | -1.946201602 | 0.051630528 |
| NCF1 | NR_110117.1 | LOC101927769 | -0.877064842 | -1.968047809 | 0.049062537 |

| | | | | | |
|---|---|---|---|---|---|
| NCF1 | NR_110731.1 | LINC01232 | 0.90629256 | 2.021375753 | 0.043240883 |
| NCF1 | NR_120466.1 | LINC01489 | -0.877343405 | -1.984203538 | 0.047233154 |
| NCF1 | NR_125769.1 | LINC01269 | 0.886827979 | 1.985265502 | 0.04711494 |
| NCF1 | NR_126334.1 | LOC101927932 | -0.822353204 | -1.846248513 | 0.06485613 |
| NCF1 | NR_126409.1 | LINC00376 | -0.889595667 | -1.996799431 | 0.045846975 |
| NCF1 | NR_130916.1 | LOC105274304 | 0.814598739 | 1.82534115 | 0.067949564 |
| NCF1 | NR_134573.1 | GS1-124K5.4 | -0.82886749 | -1.87198675 | 0.061208437 |
| NCF1 | NR_134610.1 | LOC105375014 | 0.917433866 | 2.052683102 | 0.040103322 |
| NCF1 | NR_134632.1 | LOC105373051 | 0.839840831 | 1.862783167 | 0.062492779 |
| NCF1 | NR_135040.1 | LOC101927038 | 0.84317779 | 1.88301452 | 0.0596984 |
| NCF1 | NR_135076.1 | LOC102723838 | -0.805153424 | -1.777690689 | 0.07545467 |
| NCF1 | NR_136218.1 | MEF2C-AS1 | -0.87997337 | -1.976687917 | 0.048076917 |
| NLGN4Y | ENST00000390540.2 | ENSG00000254140.1 | 0.817337897 | 1.839533253 | 0.065836792 |
| NLGN4Y | ENST00000414992.1 | ENSG00000233613.1 | 0.893504625 | 1.986129642 | 0.047018931 |
| NLGN4Y | ENST00000419863.1 | ENSG00000238282.1 | 0.821583836 | 1.833845935 | 0.066676867 |
| NLGN4Y | ENST00000421006.1 | ENSG00000234548.1 | -0.854012611 | -1.888981215 | 0.058894346 |
| NLGN4Y | ENST00000422763.1 | ENSG00000231131.2 | -0.839286576 | -1.877527707 | 0.060445804 |
| NLGN4Y | ENST00000423925.1 | ENSG00000223536.1 | 0.846799682 | 1.91919188 | 0.054960055 |
| NLGN4Y | ENST00000425881.1 | ENSG00000239636.1 | 0.860685099 | 1.911334597 | 0.055961596 |
| NLGN4Y | ENST00000427132.1 | ENSG00000232121.1 | 0.918874032 | 2.03645739 | 0.041704448 |
| NLGN4Y | ENST00000428440.1 | ENSG00000232827.2 | 0.91034094 | 2.022462372 | 0.043128607 |
| NLGN4Y | ENST00000429878.1 | ENSG00000224184.1 | 0.812195797 | 1.825901391 | 0.067865117 |
| NLGN4Y | ENST00000431290.1 | ENSG00000183822.2 | -0.819686775 | -1.852593845 | 0.063940595 |
| NLGN4Y | ENST00000432957.1 | ENSG00000231534.1 | 0.893197737 | 2.009620587 | 0.044471361 |
| NLGN4Y | ENST00000433035.1 | ENSG00000230483.1 | 0.807726784 | 1.815908899 | 0.069384348 |
| NLGN4Y | ENST00000434250.1 | ENSG00000234055.1 | 0.876276134 | 2.008209647 | 0.044621018 |
| NLGN4Y | ENST00000435357.1 | ENSG00000225444.1 | -0.843924939 | -1.872372487 | 0.061155089 |
| NLGN4Y | ENST00000435984.1 | ENSG00000204792.2 | -0.834836574 | -1.868137489 | 0.061742909 |
| NLGN4Y | ENST00000437334.1 | ENSG00000226134.1 | 0.943215337 | 2.129726062 | 0.033194236 |
| NLGN4Y | ENST00000437461.1 | ENSG00000227200.1 | -0.801671681 | -1.782032413 | 0.074743946 |
| NLGN4Y | ENST00000439443.1 | ENSG00000236911.2 | 0.920899852 | 2.093821081 | 0.036275921 |
| NLGN4Y | ENST00000440518.1 | ENSG00000226571.1 | 0.879130629 | 1.963351766 | 0.049605314 |
| NLGN4Y | ENST00000440862.1 | ENSG00000223804.1 | 0.810643483 | 1.823666899 | 0.068202446 |
| NLGN4Y | ENST00000445617.2 | ENSG00000225751.2 | -0.847629089 | -1.898662634 | 0.057608847 |
| NLGN4Y | ENST00000445631.1 | ENSG00000231052.1 | 0.967617272 | 2.171572908 | 0.029887892 |
| NLGN4Y | ENST00000448001.1 | ENSG00000229639.1 | -0.83574481 | -1.882932421 | 0.059709527 |
| NLGN4Y | ENST00000448650.1 | ENSG00000223536.1 | 0.883318679 | 1.935915415 | 0.052878061 |
| NLGN4Y | ENST00000449586.1 | ENSG00000235257.4 | 0.886620695 | 1.986022019 | 0.047030879 |
| NLGN4Y | ENST00000450500.1 | ENSG00000225790.1 | 0.960692027 | 2.171444642 | 0.029897577 |
| NLGN4Y | ENST00000451648.1 | ENSG00000232803.1 | 0.807850794 | 1.813562104 | 0.069745169 |
| NLGN4Y | ENST00000452002.1 | ENSG00000236501.1 | 0.822753351 | 1.879827549 | 0.060131585 |
| NLGN4Y | ENST00000452553.1 | ENSG00000233973.1 | -0.84976777 | -1.870596732 | 0.061400998 |
| NLGN4Y | ENST00000454709.1 | ENSG00000237280.1 | 0.927678356 | 2.096877425 | 0.036004427 |
| NLGN4Y | ENST00000455416.1 | ENSG00000229337.1 | 0.856348839 | 1.928357102 | 0.053810724 |
| NLGN4Y | ENST00000456091.1 | ENSG00000226985.1 | 0.86750019 | 1.936558033 | 0.052799392 |
| NLGN4Y | ENST00000460993.1 | ENSG00000241231.1 | -0.806054438 | -1.770786457 | 0.076596219 |
| NLGN4Y | ENST00000476099.1 | ENSG00000244158.1 | 0.804315285 | 1.811099281 | 0.070125485 |
| NLGN4Y | ENST00000479233.1 | ENSG00000243150.1 | -0.919935319 | -2.056299383 | 0.039753668 |
| NLGN4Y | ENST00000482142.1 | ENSG00000243276.1 | -0.842749828 | -1.900820046 | 0.057325588 |
| NLGN4Y | ENST00000485347.1 | ENSG00000239991.1 | 0.954972784 | 2.145478359 | 0.03191462 |
| NLGN4Y | ENST00000487368.1 | ENSG00000273328.1 | 0.834387519 | 1.849551125 | 0.064378273 |

| | | | | | |
|---|---|---|---|---|---|
| NLGN4Y | ENST00000488310.1 | ENSG00000240449.1 | 0.829951142 | 1.820790605 | 0.068638693 |
| NLGN4Y | ENST00000507808.1 | ENSG00000250333.1 | 0.954219496 | 2.164498583 | 0.030426105 |
| NLGN4Y | ENST00000509629.1 | ENSG00000250164.1 | 0.809530608 | 1.826625017 | 0.067756169 |
| NLGN4Y | ENST00000509983.1 | ENSG00000248173.1 | 0.807962186 | 1.785438481 | 0.074190222 |
| NLGN4Y | ENST00000511234.1 | ENSG00000250865.1 | 0.894374127 | 1.96237272 | 0.049719106 |
| NLGN4Y | ENST00000514459.1 | ENSG00000248211.1 | 0.945144514 | 2.12164189 | 0.033867822 |
| NLGN4Y | ENST00000518620.1 | ENSG00000253892.1 | 0.834181028 | 1.886557073 | 0.059219925 |
| NLGN4Y | ENST00000519038.2 | ENSG00000254054.2 | 0.854012611 | 1.895875098 | 0.057976563 |
| NLGN4Y | ENST00000521378.1 | ENSG00000254222.1 | 0.859516859 | 1.929859702 | 0.053624224 |
| NLGN4Y | ENST00000523703.1 | ENSG00000214803.3 | 0.859433757 | 1.934923931 | 0.052999631 |
| NLGN4Y | ENST00000524133.1 | ENSG00000253174.2 | 0.844736791 | 1.930974745 | 0.053486176 |
| NLGN4Y | ENST00000524309.1 | ENSG00000240915.2 | 0.937596636 | 2.103868972 | 0.035389878 |
| NLGN4Y | ENST00000528869.1 | ENSG00000255443.1 | -0.874854859 | -1.970303301 | 0.04880362 |
| NLGN4Y | ENST00000531071.1 | ENSG00000255248.2 | 0.880237696 | 1.961975477 | 0.049765339 |
| NLGN4Y | ENST00000533938.1 | ENSG00000255142.1 | 0.888567521 | 2.007758058 | 0.044669008 |
| NLGN4Y | ENST00000538294.1 | ENSG00000250748.2 | 0.841571375 | 1.872029426 | 0.061202533 |
| NLGN4Y | ENST00000546789.1 | ENSG00000257740.1 | -0.804984472 | -1.797227171 | 0.072299563 |
| NLGN4Y | ENST00000548748.1 | ENSG00000258252.1 | 0.847268899 | 1.90709077 | 0.056508834 |
| NLGN4Y | ENST00000549756.1 | ENSG00000257769.1 | 0.835555524 | 1.88655167 | 0.059220652 |
| NLGN4Y | ENST00000552378.1 | ENSG00000257294.1 | 0.949843484 | 2.146040655 | 0.031869735 |
| NLGN4Y | ENST00000553464.1 | ENSG00000258418.1 | -0.862591668 | -1.950325026 | 0.051137392 |
| NLGN4Y | ENST00000553668.1 | ENSG00000258733.1 | -0.845972098 | -1.865553172 | 0.062103906 |
| NLGN4Y | ENST00000555913.1 | ENSG00000259077.1 | -0.858301587 | -1.907294221 | 0.056482498 |
| NLGN4Y | ENST00000557368.1 | ENSG00000258444.1 | 0.852085923 | 1.919936327 | 0.054865943 |
| NLGN4Y | ENST00000558434.1 | ENSG00000259572.1 | 0.885936635 | 1.981288127 | 0.047558971 |
| NLGN4Y | ENST00000563601.1 | ENSG00000260589.1 | 0.836561998 | 1.839545902 | 0.065834933 |
| NLGN4Y | ENST00000565623.1 | ENSG00000261118.1 | 0.908503233 | 2.024404421 | 0.042928557 |
| NLGN4Y | ENST00000565955.1 | ENSG00000261055.1 | 0.839790079 | 1.880022619 | 0.060104995 |
| NLGN4Y | ENST00000566639.1 | ENSG00000261061.1 | 0.867909395 | 1.92566488 | 0.054146232 |
| NLGN4Y | ENST00000568332.1 | ENSG00000260256.1 | 0.849191828 | 1.874284761 | 0.060891187 |
| NLGN4Y | ENST00000569147.1 | ENSG00000261592.1 | 0.851382447 | 1.915075886 | 0.055482827 |
| NLGN4Y | ENST00000569215.1 | ENSG00000260756.1 | -0.860950091 | -1.960585179 | 0.049927433 |
| NLGN4Y | ENST00000571660.1 | ENSG00000262848.1 | 0.847232325 | 1.911574057 | 0.05593085 |
| NLGN4Y | ENST00000572471.1 | ENSG00000262721.1 | 0.914427402 | 2.019569223 | 0.04342809 |
| NLGN4Y | ENST00000573315.1 | ENSG00000270168.1 | 0.908828217 | 2.030296273 | 0.042326433 |
| NLGN4Y | ENST00000576271.1 | ENSG00000263342.1 | 0.815366258 | 1.846919126 | 0.064758862 |
| NLGN4Y | ENST00000585181.1 | ENSG00000265749.1 | -0.832583855 | -1.858065692 | 0.063159686 |
| NLGN4Y | ENST00000585761.1 | ENSG00000267198.1 | 0.865683525 | 1.899623042 | 0.057482606 |
| NLGN4Y | ENST00000586338.1 | ENSG00000219410.4 | 0.832562798 | 1.85422615 | 0.063706812 |
| NLGN4Y | ENST00000586348.1 | ENSG00000267198.1 | 0.890813329 | 1.979978303 | 0.047705967 |
| NLGN4Y | ENST00000587049.1 | ENSG00000235535.3 | 0.800269324 | 1.77120306 | 0.076526941 |
| NLGN4Y | ENST00000587850.1 | ENSG00000267683.1 | 0.885386706 | 1.968134717 | 0.049052539 |
| NLGN4Y | ENST00000589281.1 | ENSG00000267707.1 | 0.817181901 | 1.837634091 | 0.066116342 |
| NLGN4Y | ENST00000590364.1 | ENSG00000267613.1 | 0.80977633 | 1.80888226 | 0.070469297 |
| NLGN4Y | ENST00000592368.1 | ENSG00000267231.1 | 0.839161258 | 1.874956167 | 0.060798754 |
| NLGN4Y | ENST00000593967.1 | ENSG00000232732.5 | 0.955867077 | 2.133131437 | 0.032913946 |
| NLGN4Y | ENST00000596971.1 | ENSG00000269463.1 | 0.862082467 | 1.941421454 | 0.052207178 |
| NLGN4Y | ENST00000597420.1 | ENSG00000269564.1 | 0.910750836 | 2.030723675 | 0.042283033 |
| NLGN4Y | ENST00000599387.1 | ENSG00000227733.4 | -0.820289721 | -1.867801085 | 0.061789802 |
| NLGN4Y | ENST00000599572.1 | ENSG00000233783.3 | 0.846768686 | 1.864421787 | 0.062262496 |
| NLGN4Y | ENST00000600848.1 | ENSG00000228065.6 | -0.802368756 | -1.795189458 | 0.072623519 |

| | | | | | |
|---|---|---|---|---|---|
| NLGN4Y | ENST00000603948.1 | ENSG00000222041.6 | 0.923385077 | 2.045164635 | 0.04083863 |
| NLGN4Y | ENST00000605692.1 | ENSG00000270810.1 | 0.852322729 | 1.888484361 | 0.058960956 |
| NLGN4Y | ENST00000606757.1 | ENSG00000237188.3 | 0.91538573 | 2.013808109 | 0.044029684 |
| NLGN4Y | ENST00000606898.1 | ENSG00000272094.1 | 0.803947206 | 1.797271073 | 0.072292596 |
| NLGN4Y | ENST00000606921.1 | ENSG00000272402.1 | 0.933947571 | 2.046857076 | 0.04067212 |
| NLGN4Y | ENST00000607740.1 | ENSG00000271916.1 | -0.858332508 | -1.918115099 | 0.055096418 |
| NLGN4Y | ENST00000608289.1 | ENSG00000272958.1 | 0.848677733 | 1.942732007 | 0.052048547 |
| NLGN4Y | ENST00000609789.1 | ENSG00000272707.1 | 0.980306075 | 2.188199271 | 0.02865509 |
| NLGN4Y | NR_026774.1 | LINC00239 | 0.804223437 | 1.826247933 | 0.067812924 |
| NLGN4Y | NR_026822.1 | FAM138C | 0.858525756 | 1.945266919 | 0.051742861 |
| NLGN4Y | NR_031762.2 | HCP5B | 0.872713012 | 1.955177166 | 0.050562163 |
| NLGN4Y | NR_034131.1 | LINC00272 | 0.938383857 | 2.143016233 | 0.032111795 |
| NLGN4Y | NR_040047.1 | SDCBP2-AS1 | 0.918068291 | 2.045809359 | 0.040775131 |
| NLGN4Y | NR_040049.1 | SDCBP2-AS1 | 0.825965402 | 1.891359737 | 0.058576339 |
| NLGN4Y | NR_040061.1 | SRP14-AS1 | 0.874191356 | 1.93319202 | 0.053212547 |
| NLGN4Y | NR_102746.1 | ROPN1L-AS1 | 0.892757729 | 1.996066535 | 0.045926681 |
| NLGN4Y | NR_109985.1 | LOC101927830 | 0.910181612 | 2.011424679 | 0.044280619 |
| NLGN4Y | NR_110370.1 | STAM-AS1 | 0.829384285 | 1.868965087 | 0.061627672 |
| NLGN4Y | NR_110504.1 | LOC101929572 | 0.836490622 | 1.880272136 | 0.060070999 |
| NLGN4Y | NR_110559.1 | LOC101927023 | -0.907595071 | -2.044270849 | 0.040926797 |
| NLGN4Y | NR_125420.1 | LOC101927588 | 0.857933403 | 1.902460336 | 0.057111 |
| NLGN4Y | NR_126041.1 | LOC101930071 | 0.934198733 | 2.124304535 | 0.033644687 |
| NLGN4Y | NR_126412.1 | SCEL-AS1 | 0.913500278 | 2.070743589 | 0.038382764 |
| NLGN4Y | NR_126413.1 | SCEL-AS1 | 0.891064671 | 1.987346099 | 0.046884056 |
| NLGN4Y | NR_133942.1 | LOC105377247 | -0.835572749 | -1.866845493 | 0.061923167 |
| NLGN4Y | NR_135239.1 | LINC01867 | -0.849523004 | -1.893680011 | 0.058267496 |
| NLGN4Y | NR_135816.1 | LOC100996664 | 0.962496251 | 2.147856261 | 0.031725176 |
| NLGN4Y | NR_136320.1 | LOC105373656 | 0.862975824 | 1.926885472 | 0.053993904 |
| NTS | ENST00000340585.6 | ENSG00000249429.1 | 0.856838486 | 1.913769284 | 0.055649642 |
| NTS | ENST00000362684.1 | ENSG00000228549.2 | 0.812017119 | 1.831177671 | 0.067074028 |
| NTS | ENST00000399186.2 | ENSG00000214888.2 | -0.852871182 | -1.888833609 | 0.058914128 |
| NTS | ENST00000412085.1 | ENSG00000233825.1 | -0.802840031 | -1.787581241 | 0.073843594 |
| NTS | ENST00000412896.1 | ENSG00000197585.5 | 0.840091054 | 1.879782032 | 0.06013779 |
| NTS | ENST00000413564.1 | ENSG00000224500.1 | 0.877970169 | 1.966064311 | 0.049291182 |
| NTS | ENST00000413650.1 | ENSG00000230880.2 | -0.837725443 | -1.884804093 | 0.059456292 |
| NTS | ENST00000413887.1 | ENSG00000236948.1 | 0.907433278 | 2.036974459 | 0.041652602 |
| NTS | ENST00000413989.1 | ENSG00000242628.1 | -0.867361524 | -1.955787246 | 0.050490222 |
| NTS | ENST00000414740.2 | ENSG00000229646.2 | -0.863125809 | -1.927703006 | 0.053892078 |
| NTS | ENST00000416401.1 | ENSG00000237756.1 | 0.872872158 | 1.924523599 | 0.054288986 |
| NTS | ENST00000417315.1 | ENSG00000242486.1 | 0.838015898 | 1.874530254 | 0.060857376 |
| NTS | ENST00000417426.1 | ENSG00000233145.1 | -0.801481697 | -1.797482821 | 0.072259003 |
| NTS | ENST00000419734.1 | ENSG00000234646.1 | 0.968643062 | 2.161847541 | 0.03062993 |
| NTS | ENST00000420465.1 | ENSG00000167355.3 | -0.863268976 | -1.903103375 | 0.057027058 |
| NTS | ENST00000420572.2 | ENSG00000233358.2 | -0.905267554 | -2.025113196 | 0.042855742 |
| NTS | ENST00000421498.1 | ENSG00000237978.1 | -0.836582269 | -1.87318705 | 0.06104256 |
| NTS | ENST00000421617.1 | ENSG00000237342.1 | -0.900081129 | -1.989999966 | 0.046590939 |
| NTS | ENST00000423869.1 | ENSG00000227848.1 | -0.913970136 | -2.035256313 | 0.041825089 |
| NTS | ENST00000423925.1 | ENSG00000223536.1 | 0.822624895 | 1.845403994 | 0.064978793 |
| NTS | ENST00000424241.1 | ENSG00000237311.1 | 0.937406189 | 2.080534397 | 0.037476544 |
| NTS | ENST00000426504.1 | ENSG00000234190.1 | 0.862757018 | 1.92024703 | 0.054826704 |
| NTS | ENST00000426519.1 | ENSG00000234142.1 | -0.858712301 | -1.932003503 | 0.053359074 |

| | | | | | |
|---|---|---|---|---|---|
| NTS | ENST00000427064.1 | ENSG00000238031.1 | -0.913797786 | -2.058784116 | 0.039514924 |
| NTS | ENST00000429796.1 | ENSG00000231858.1 | 0.968452188 | 2.157369725 | 0.030976868 |
| NTS | ENST00000430025.1 | ENSG00000233508.1 | 0.851999903 | 1.913529256 | 0.055680332 |
| NTS | ENST00000430545.1 | ENSG00000237153.1 | -0.901442045 | -2.027441478 | 0.042617284 |
| NTS | ENST00000430842.1 | ENSG00000230433.1 | 0.833740456 | 1.843427961 | 0.065266551 |
| NTS | ENST00000432244.1 | ENSG00000234265.1 | -0.801531798 | -1.780214867 | 0.075040803 |
| NTS | ENST00000432559.2 | ENSG00000228229.2 | -0.840582633 | -1.896721324 | 0.057864728 |
| NTS | ENST00000433614.1 | ENSG00000228534.1 | 0.811655967 | 1.828771405 | 0.06743386 |
| NTS | ENST00000433876.2 | ENSG00000228423.2 | -0.884852206 | -1.96516924 | 0.049394653 |
| NTS | ENST00000434627.1 | ENSG00000230074.1 | -0.891932632 | -2.010484122 | 0.044379975 |
| NTS | ENST00000435892.1 | ENSG00000233635.2 | -0.915049567 | -2.044181444 | 0.040935626 |
| NTS | ENST00000436582.1 | ENSG00000236525.1 | 0.94244099 | 2.077825719 | 0.037725414 |
| NTS | ENST00000438107.1 | ENSG00000234449.2 | -0.88747309 | -1.954779216 | 0.050609135 |
| NTS | ENST00000440714.1 | ENSG00000237609.1 | -0.872075058 | -1.958125879 | 0.050215244 |
| NTS | ENST00000441295.1 | ENSG00000233960.1 | 0.898052917 | 2.010041911 | 0.044426753 |
| NTS | ENST00000441532.1 | ENSG00000234206.1 | 0.908837243 | 2.030995562 | 0.042255445 |
| NTS | ENST00000441666.1 | ENSG00000230379.1 | -0.834591052 | -1.859368817 | 0.062974879 |
| NTS | ENST00000441875.1 | ENSG00000239203.1 | -0.803379677 | -1.831611009 | 0.067009395 |
| NTS | ENST00000441991.1 | ENSG00000231210.2 | 0.85799422 | 1.899218387 | 0.057535768 |
| NTS | ENST00000442850.1 | ENSG00000232600.2 | 0.915502021 | 2.024372224 | 0.042931867 |
| NTS | ENST00000443162.1 | ENSG00000234183.1 | -0.88224006 | -1.993351812 | 0.046222937 |
| NTS | ENST00000443380.1 | ENSG00000224371.1 | -0.878988349 | -1.948107571 | 0.051402093 |
| NTS | ENST00000444665.1 | ENSG00000228852.2 | -0.963182738 | -2.15797022 | 0.030930147 |
| NTS | ENST00000444731.1 | ENSG00000227131.1 | 0.836838782 | 1.881135045 | 0.059953551 |
| NTS | ENST00000447206.1 | ENSG00000230839.1 | -0.906921982 | -2.019020272 | 0.043485113 |
| NTS | ENST00000448086.1 | ENSG00000237571.1 | 0.842694365 | 1.87681053 | 0.060544067 |
| NTS | ENST00000448365.1 | ENSG00000231114.1 | -0.821485266 | -1.831638697 | 0.067005267 |
| NTS | ENST00000448491.1 | ENSG00000231212.1 | 0.81164441 | 1.803385395 | 0.07132771 |
| NTS | ENST00000449463.1 | ENSG00000230309.1 | 0.894242116 | 1.983699671 | 0.04728933 |
| NTS | ENST00000450063.1 | ENSG00000231210.2 | 0.93725023 | 2.093340005 | 0.036318814 |
| NTS | ENST00000450109.1 | ENSG00000225376.1 | -0.892433339 | -1.982450585 | 0.047428833 |
| NTS | ENST00000454530.1 | ENSG00000226649.1 | 0.898998715 | 2.015244906 | 0.043878994 |
| NTS | ENST00000456715.1 | ENSG00000224893.1 | -0.805911027 | -1.805030185 | 0.071069959 |
| NTS | ENST00000456999.1 | ENSG00000230690.1 | -0.861228416 | -1.935606344 | 0.052915933 |
| NTS | ENST00000457848.1 | ENSG00000226412.1 | -0.879772877 | -1.972124198 | 0.04859543 |
| NTS | ENST00000457975.2 | ENSG00000236744.2 | 0.92755487 | 2.065584131 | 0.038867764 |
| NTS | ENST00000458194.1 | ENSG00000226193.1 | -0.812012956 | -1.829803461 | 0.067279333 |
| NTS | ENST00000458364.1 | ENSG00000225655.1 | 0.813981203 | 1.822707274 | 0.068347738 |
| NTS | ENST00000458443.1 | ENSG00000238232.1 | -0.873655585 | -1.974820178 | 0.048288558 |
| NTS | ENST00000490013.1 | ENSG00000184115.12 | -0.803165065 | -1.772179535 | 0.07636476 |
| NTS | ENST00000493123.1 | ENSG00000242428.1 | -0.857711178 | -1.904461817 | 0.056850065 |
| NTS | ENST00000502421.1 | ENSG00000250284.1 | 0.944608415 | 2.13942026 | 0.032401648 |
| NTS | ENST00000503034.1 | ENSG00000248936.1 | -0.847141997 | -1.884317594 | 0.059522029 |
| NTS | ENST00000503470.1 | ENSG00000248559.1 | -0.828866135 | -1.860750087 | 0.062779477 |
| NTS | ENST00000505196.1 | ENSG00000248131.1 | -0.933530227 | -2.076280328 | 0.037868032 |
| NTS | ENST00000505556.1 | ENSG00000249409.1 | -0.886696162 | -1.952876942 | 0.050834179 |
| NTS | ENST00000506100.1 | ENSG00000249409.1 | -0.895277749 | -2.017898488 | 0.043601835 |
| NTS | ENST00000506305.1 | ENSG00000249994.1 | 0.910537687 | 2.04893842 | 0.040468137 |
| NTS | ENST00000507558.1 | ENSG00000248445.1 | -0.885231197 | -1.992396374 | 0.046327585 |
| NTS | ENST00000508004.2 | ENSG00000251661.3 | -0.954922238 | -2.111287641 | 0.034747596 |
| NTS | ENST00000508083.1 | ENSG00000249343.1 | -0.80461951 | -1.800522739 | 0.071778136 |

| | | | | | |
|---|---|---|---|---|---|
| NTS | ENST00000508188.1 | ENSG00000250999.1 | -0.86693887 | -1.945387244 | 0.051728389 |
| NTS | ENST00000510001.2 | ENSG00000249196.2 | 0.862665464 | 1.891039902 | 0.058619018 |
| NTS | ENST00000514411.1 | ENSG00000250882.1 | -0.842564434 | -1.88540734 | 0.059374864 |
| NTS | ENST00000514661.1 | ENSG00000247993.2 | -0.888818496 | -2.000746566 | 0.045419708 |
| NTS | ENST00000514877.1 | ENSG00000248685.1 | -0.964641684 | -2.152920935 | 0.031324893 |
| NTS | ENST00000515077.1 | ENSG00000251206.1 | -0.914642787 | -2.016131404 | 0.043786237 |
| NTS | ENST00000517300.1 | ENSG00000254144.2 | -0.827758514 | -1.838757665 | 0.065950838 |
| NTS | ENST00000518260.1 | ENSG00000253628.1 | 0.907036537 | 2.012834968 | 0.044131994 |
| NTS | ENST00000518894.1 | ENSG00000204758.3 | -0.904103225 | -2.043387576 | 0.041014086 |
| NTS | ENST00000519368.1 | ENSG00000253215.1 | -0.892178337 | -1.979784723 | 0.047727724 |
| NTS | ENST00000519451.1 | ENSG00000253363.1 | -0.854165248 | -1.897489767 | 0.057763328 |
| NTS | ENST00000519844.1 | ENSG00000253824.1 | 0.896245339 | 2.021173365 | 0.043261822 |
| NTS | ENST00000520838.1 | ENSG00000253404.1 | -0.865359447 | -1.918755172 | 0.055015326 |
| NTS | ENST00000520849.1 | ENSG00000253553.1 | -0.974839692 | -2.163627433 | 0.030492954 |
| NTS | ENST00000521055.1 | ENSG00000253184.1 | 0.864152414 | 1.92689943 | 0.053992165 |
| NTS | ENST00000522547.1 | ENSG00000253430.1 | 0.850053576 | 1.892941972 | 0.058365587 |
| NTS | ENST00000524073.1 | ENSG00000253774.1 | -0.826624646 | -1.847277652 | 0.06470691 |
| NTS | ENST00000524335.1 | ENSG00000253716.1 | -0.816072738 | -1.840030154 | 0.06576381 |
| NTS | ENST00000524818.1 | ENSG00000254473.1 | -0.803043065 | -1.812960785 | 0.06983787 |
| NTS | ENST00000526186.1 | ENSG00000254510.1 | -0.829840822 | -1.860196359 | 0.06285775 |
| NTS | ENST00000528000.1 | ENSG00000254804.1 | -0.843785641 | -1.891481379 | 0.058560114 |
| NTS | ENST00000528818.1 | ENSG00000232995.3 | -0.935990362 | -2.09745804 | 0.035953048 |
| NTS | ENST00000531136.1 | ENSG00000255558.1 | 0.878310066 | 1.980655343 | 0.047629938 |
| NTS | ENST00000532947.1 | ENSG00000255322.1 | -0.836894561 | -1.876666319 | 0.060563842 |
| NTS | ENST00000535324.1 | ENSG00000255968.1 | -0.818997535 | -1.8245593 | 0.06806756 |
| NTS | ENST00000536141.1 | ENSG00000256969.1 | -0.938833925 | -2.122016146 | 0.033836382 |
| NTS | ENST00000537269.1 | ENSG00000257084.1 | -0.848724682 | -1.881917831 | 0.059847173 |
| NTS | ENST00000540739.1 | ENSG00000249196.2 | 0.889348471 | 2.001978032 | 0.045287094 |
| NTS | ENST00000544089.1 | ENSG00000256273.1 | -0.817807513 | -1.822663715 | 0.068354339 |
| NTS | ENST00000553348.1 | ENSG00000258829.1 | 0.882080548 | 1.991699524 | 0.046404036 |
| NTS | ENST00000556145.1 | ENSG00000258829.1 | 0.846852159 | 1.885421612 | 0.059372939 |
| NTS | ENST00000556978.1 | ENSG00000258693.1 | -0.948561702 | -2.122229992 | 0.033818429 |
| NTS | ENST00000557412.1 | ENSG00000257621.3 | 0.917033499 | 2.0475024 | 0.040608781 |
| NTS | ENST00000558141.1 | ENSG00000259594.1 | -0.868978803 | -1.932287863 | 0.053323986 |
| NTS | ENST00000558312.1 | ENSG00000259176.1 | -0.850437941 | -1.889730218 | 0.05879405 |
| NTS | ENST00000559569.1 | ENSG00000259760.1 | 0.896716982 | 2.003302156 | 0.045144866 |
| NTS | ENST00000561529.1 | ENSG00000260886.1 | -0.912169877 | -2.056532573 | 0.03973121 |
| NTS | ENST00000561567.1 | ENSG00000260177.1 | -0.916940699 | -2.03416666 | 0.041934795 |
| NTS | ENST00000563449.2 | ENSG00000261613.2 | -0.91551132 | -2.033212013 | 0.042031108 |
| NTS | ENST00000564417.1 | ENSG00000260137.1 | 0.938183829 | 2.11958325 | 0.034041206 |
| NTS | ENST00000566170.1 | ENSG00000261071.1 | -0.814524881 | -1.78849249 | 0.073696586 |
| NTS | ENST00000568033.1 | ENSG00000261480.1 | -0.805492553 | -1.814258468 | 0.069637942 |
| NTS | ENST00000569328.1 | ENSG00000261638.1 | -0.801513424 | -1.796752252 | 0.07237496 |
| NTS | ENST00000569459.1 | ENSG00000261346.1 | -0.801119769 | -1.802103311 | 0.071529153 |
| NTS | ENST00000569742.1 | ENSG00000260787.1 | -0.910876434 | -2.055732371 | 0.03980832 |
| NTS | ENST00000570843.1 | ENSG00000261889.1 | -0.81263754 | -1.825176366 | 0.067974419 |
| NTS | ENST00000571815.1 | ENSG00000262810.1 | -0.886032231 | -2.00531531 | 0.044929349 |
| NTS | ENST00000574460.1 | ENSG00000263051.1 | -0.871340265 | -1.936913464 | 0.052755922 |
| NTS | ENST00000575139.1 | ENSG00000263072.1 | -0.955319456 | -2.131949551 | 0.033010994 |
| NTS | ENST00000577678.1 | ENSG00000265415.1 | -0.934933325 | -2.113097228 | 0.034592447 |
| NTS | ENST00000578265.1 | ENSG00000214719.7 | -0.808612769 | -1.789332909 | 0.073561217 |

| | | | | | |
|---|---|---|---|---|---|
| NTS | ENST00000581905.1 | ENSG00000264235.1 | -0.979846284 | -2.21930868 | 0.026465731 |
| NTS | ENST00000581940.1 | ENSG00000265484.1 | -0.883295113 | -2.002357872 | 0.045246256 |
| NTS | ENST00000582895.1 | ENSG00000264729.1 | 0.876059634 | 1.92553108 | 0.054162952 |
| NTS | ENST00000585072.1 | ENSG00000263745.1 | -0.979936633 | -2.195517496 | 0.028126497 |
| NTS | ENST00000586051.1 | ENSG00000267576.1 | -0.85834595 | -1.910130879 | 0.056116364 |
| NTS | ENST00000587049.1 | ENSG00000235535.3 | 0.814513079 | 1.81965682 | 0.068811283 |
| NTS | ENST00000587281.1 | ENSG00000228290.2 | -0.808606355 | -1.792626906 | 0.073032601 |
| NTS | ENST00000590046.1 | ENSG00000266950.1 | 0.801590109 | 1.800335301 | 0.07180771 |
| NTS | ENST00000591137.1 | ENSG00000267405.1 | -0.952335152 | -2.131028129 | 0.033086825 |
| NTS | ENST00000593588.1 | ENSG00000269635.1 | -0.968307367 | -2.16434145 | 0.030438154 |
| NTS | ENST00000593632.1 | ENSG00000180279.5 | -0.84472817 | -1.875555255 | 0.060716375 |
| NTS | ENST00000598131.1 | ENSG00000269043.1 | -0.956486883 | -2.16705713 | 0.030230498 |
| NTS | ENST00000598356.1 | ENSG00000269640.1 | -0.852117385 | -1.894772471 | 0.058122552 |
| NTS | ENST00000600071.1 | ENSG00000269199.1 | -0.81819848 | -1.835977964 | 0.066360916 |
| NTS | ENST00000600234.1 | ENSG00000268078.1 | -0.964465301 | -2.168309554 | 0.030135142 |
| NTS | ENST00000600889.1 | ENSG00000232675.3 | -0.873909902 | -1.970075073 | 0.048829767 |
| NTS | ENST00000600959.1 | ENSG00000269303.1 | -0.971650314 | -2.182708421 | 0.029057289 |
| NTS | ENST00000601752.1 | ENSG00000268051.1 | -0.849398384 | -1.906104172 | 0.056636691 |
| NTS | ENST00000602532.1 | ENSG00000270091.1 | -0.858175435 | -1.909866793 | 0.056150367 |
| NTS | ENST00000602900.1 | ENSG00000270179.1 | 0.868474078 | 1.919258176 | 0.054951668 |
| NTS | ENST00000603612.1 | ENSG00000270996.1 | -0.94346298 | -2.129547591 | 0.033208982 |
| NTS | ENST00000606068.1 | ENSG00000272342.1 | -0.852893662 | -1.909042073 | 0.056256664 |
| NTS | ENST00000606374.1 | ENSG00000272312.1 | 0.856112043 | 1.935568712 | 0.052920545 |
| NTS | ENST00000606778.1 | ENSG00000271930.1 | -0.86681843 | -1.961833128 | 0.049781915 |
| NTS | ENST00000606938.1 | ENSG00000272198.1 | 0.826287417 | 1.870102135 | 0.061469636 |
| NTS | ENST00000607136.1 | ENSG00000267546.2 | 0.910626459 | 2.028569446 | 0.042502163 |
| NTS | ENST00000607201.1 | ENSG00000272024.1 | 0.869358932 | 1.949180423 | 0.051273882 |
| NTS | ENST00000607321.1 | ENSG00000272371.1 | 0.953840025 | 2.132236065 | 0.032987445 |
| NTS | ENST00000607580.1 | ENSG00000272545.1 | 0.855945647 | 1.94742546 | 0.051483748 |
| NTS | ENST00000608173.1 | ENSG00000197099.4 | -0.883708307 | -1.991700276 | 0.046403953 |
| NTS | ENST00000609067.1 | ENSG00000272849.1 | -0.930190404 | -2.069750974 | 0.038475671 |
| NTS | ENST00000609218.1 | ENSG00000272945.1 | -0.900559843 | -2.02886694 | 0.042471845 |
| NTS | ENST00000609238.1 | ENSG00000272703.1 | 0.913456932 | 2.04054415 | 0.041296158 |
| NTS | ENST00000609281.1 | ENSG00000273320.1 | -0.924290662 | -2.079182587 | 0.037600572 |
| NTS | NR_002765.2 | ASAP1-IT1 | -0.851169284 | -1.901860751 | 0.057189361 |
| NTS | NR_003606.2 | ZFAS1 | -0.806538827 | -1.812856671 | 0.069853931 |
| NTS | NR_034037.1 | LINC00582 | 0.945116935 | 2.095725725 | 0.036106528 |
| NTS | NR_034111.1 | TRAF3IP2-AS1 | -0.898474871 | -2.02342082 | 0.043029779 |
| NTS | NR_045637.1 | BOLA3-AS1 | -0.904436353 | -2.039122925 | 0.041437761 |
| NTS | NR_046454.1 | LINC00907 | 0.873666187 | 1.94321173 | 0.051990581 |
| NTS | NR_046556.1 | RBMS3-AS1 | -0.861019549 | -1.936235618 | 0.05283885 |
| NTS | NR_046742.2 | ZNF630-AS1 | 0.851825105 | 1.906009879 | 0.056648924 |
| NTS | NR_102737.1 | LINC00911 | 0.936198288 | 2.114426236 | 0.034478879 |
| NTS | NR_103790.1 | LINC00581 | 0.910380851 | 2.031985556 | 0.042155118 |
| NTS | NR_103851.1 | TAT-AS1 | -0.925674053 | -2.06439009 | 0.038980745 |
| NTS | NR_109870.1 | LINC01723 | -0.835976007 | -1.861631701 | 0.062655022 |
| NTS | NR_110008.1 | ADNP-AS1 | -0.800780328 | -1.812769916 | 0.069867316 |
| NTS | NR_110009.1 | ADNP-AS1 | -0.800780328 | -1.783116311 | 0.074567372 |
| NTS | NR_110481.1 | LOC101927079 | -0.850437941 | -1.898357218 | 0.057649041 |
| NTS | NR_110568.1 | LOC101927661 | -0.968643062 | -2.172555731 | 0.029813771 |
| NTS | NR_125925.1 | LOC101929448 | -0.859408598 | -1.923560954 | 0.054409639 |

| | | | | | |
|---|---|---|---|---|---|
| NTS | NR_126354.1 | LINC01331 | 0.955876684 | 2.118995036 | 0.034090886 |
| NTS | NR_126380.1 | LINC01072 | -0.908175606 | -2.002011044 | 0.045283544 |
| NTS | NR_130144.1 | LOC104968399 | -0.979846284 | -2.189892486 | 0.028532035 |
| NTS | NR_134245.1 | LOC105379194 | -0.877861283 | -1.976370228 | 0.048112861 |
| NTS | NR_135032.1 | LOC105369635 | -0.848724682 | -1.88686346 | 0.059178693 |
| NTS | NR_135644.1 | LOC105371506 | 0.834653031 | 1.853280836 | 0.063842116 |
| NTS | NR_138041.1 | LINC00384 | -0.901875347 | -2.008689566 | 0.044570066 |
| OPN1MW2 | ENST00000318291.4 | ENSG00000177406.4 | 0.90733333 | 2.038019098 | 0.041548023 |
| OPN1MW2 | ENST00000399186.2 | ENSG00000214888.2 | 0.878447727 | 1.953464875 | 0.050764535 |
| OPN1MW2 | ENST00000412759.1 | ENSG00000236933.1 | 0.890679665 | 2.01672424 | 0.043724299 |
| OPN1MW2 | ENST00000413650.1 | ENSG00000230880.2 | 0.853316902 | 1.891272715 | 0.058587949 |
| OPN1MW2 | ENST00000416329.1 | ENSG00000233184.2 | 0.803644949 | 1.801370343 | 0.071644527 |
| OPN1MW2 | ENST00000417426.1 | ENSG00000233145.1 | 0.819804028 | 1.809820536 | 0.070323622 |
| OPN1MW2 | ENST00000421020.1 | ENSG00000231407.1 | 0.878904021 | 1.968180162 | 0.049047312 |
| OPN1MW2 | ENST00000421207.1 | ENSG00000231768.1 | 0.942078256 | 2.074884553 | 0.037997236 |
| OPN1MW2 | ENST00000423667.1 | ENSG00000225970.1 | 0.824854895 | 1.84533742 | 0.06498847 |
| OPN1MW2 | ENST00000423869.1 | ENSG00000227848.1 | 0.919867347 | 2.044057242 | 0.040947893 |
| OPN1MW2 | ENST00000424241.1 | ENSG00000237311.1 | -0.816057268 | -1.856616373 | 0.063365753 |
| OPN1MW2 | ENST00000425624.1 | ENSG00000223779.4 | 0.875226696 | 1.953153567 | 0.050801401 |
| OPN1MW2 | ENST00000426237.2 | ENSG00000235527.2 | 0.863155619 | 1.920491897 | 0.054795796 |
| OPN1MW2 | ENST00000426504.1 | ENSG00000234190.1 | -0.80805652 | -1.823438192 | 0.06823705 |
| OPN1MW2 | ENST00000426519.1 | ENSG00000234142.1 | 0.945832575 | 2.126387224 | 0.033471031 |
| OPN1MW2 | ENST00000426699.1 | ENSG00000229308.1 | 0.898490905 | 2.012810713 | 0.044134547 |
| OPN1MW2 | ENST00000427064.1 | ENSG00000238031.1 | 0.816540089 | 1.814729237 | 0.06956553 |
| OPN1MW2 | ENST00000429796.1 | ENSG00000231858.1 | -0.824285894 | -1.820517929 | 0.068680168 |
| OPN1MW2 | ENST00000433344.1 | ENSG00000234083.1 | -0.866933769 | -1.941098174 | 0.052246371 |
| OPN1MW2 | ENST00000433614.1 | ENSG00000228534.1 | -0.918674662 | -2.055472893 | 0.039833351 |
| OPN1MW2 | ENST00000433876.2 | ENSG00000228423.2 | 0.822477505 | 1.822240521 | 0.068418499 |
| OPN1MW2 | ENST00000434627.1 | ENSG00000230074.1 | 0.863005383 | 1.947577246 | 0.051465569 |
| OPN1MW2 | ENST00000435434.1 | ENSG00000231233.1 | 0.97511863 | 2.179899498 | 0.029264912 |
| OPN1MW2 | ENST00000435892.1 | ENSG00000233635.2 | 0.944822905 | 2.112697293 | 0.034626685 |
| OPN1MW2 | ENST00000435992.2 | ENSG00000232675.3 | 0.90838987 | 2.027775889 | 0.042583126 |
| OPN1MW2 | ENST00000436982.2 | ENSG00000235335.2 | -0.830395674 | -1.857561222 | 0.06323135 |
| OPN1MW2 | ENST00000438107.1 | ENSG00000234449.2 | 0.964582714 | 2.143368247 | 0.032083541 |
| OPN1MW2 | ENST00000438190.1 | ENSG00000227214.2 | 0.911682782 | 2.057265203 | 0.039660723 |
| OPN1MW2 | ENST00000438222.1 | ENSG00000238034.1 | 0.819540779 | 1.802722081 | 0.071431873 |
| OPN1MW2 | ENST00000439186.1 | ENSG00000237076.1 | 0.878900686 | 1.964413426 | 0.049482167 |
| OPN1MW2 | ENST00000440714.1 | ENSG00000237609.1 | 0.856141636 | 1.927868611 | 0.053871471 |
| OPN1MW2 | ENST00000441875.1 | ENSG00000239203.1 | 0.886060925 | 2.002834747 | 0.045195029 |
| OPN1MW2 | ENST00000442069.1 | ENSG00000225655.1 | -0.884696224 | -1.987224018 | 0.046897577 |
| OPN1MW2 | ENST00000444665.1 | ENSG00000228852.2 | 0.89468067 | 2.008983973 | 0.044538833 |
| OPN1MW2 | ENST00000447206.1 | ENSG00000230839.1 | 0.938992534 | 2.145768703 | 0.031891437 |
| OPN1MW2 | ENST00000447514.1 | ENSG00000236753.1 | 0.832074902 | 1.850829291 | 0.064194116 |
| OPN1MW2 | ENST00000449463.1 | ENSG00000230309.1 | -0.838253676 | -1.862694178 | 0.062505305 |
| OPN1MW2 | ENST00000450063.1 | ENSG00000231210.2 | -0.826625566 | -1.848822872 | 0.064483394 |
| OPN1MW2 | ENST00000451656.1 | ENSG00000228417.1 | 0.859197914 | 1.924959993 | 0.054234363 |
| OPN1MW2 | ENST00000454530.1 | ENSG00000226649.1 | -0.851419379 | -1.922017431 | 0.054603565 |
| OPN1MW2 | ENST00000456715.1 | ENSG00000224893.1 | 0.857112891 | 1.922195178 | 0.054581203 |
| OPN1MW2 | ENST00000457115.1 | ENSG00000227245.1 | 0.848789906 | 1.904604093 | 0.056831554 |
| OPN1MW2 | ENST00000457975.2 | ENSG00000236744.2 | -0.824341615 | -1.853713314 | 0.063780186 |
| OPN1MW2 | ENST00000458154.1 | ENSG00000235578.1 | 0.916191734 | 2.060701213 | 0.039331554 |

| | | | | | |
|---|---|---|---|---|---|
| OPN1MW2 | ENST00000458194.1 | ENSG00000226193.1 | 0.900041417 | 1.982548385 | 0.047417898 |
| OPN1MW2 | ENST00000458364.1 | ENSG00000225655.1 | -0.962781049 | -2.137497114 | 0.03255758 |
| OPN1MW2 | ENST00000459985.1 | ENSG00000273066.1 | 0.91909846 | 2.080307835 | 0.037497307 |
| OPN1MW2 | ENST00000484413.1 | ENSG00000271853.1 | 0.935039339 | 2.086588447 | 0.036925352 |
| OPN1MW2 | ENST00000489077.1 | ENSG00000244198.1 | 0.805716056 | 1.794847296 | 0.072678033 |
| OPN1MW2 | ENST00000489557.2 | ENSG00000257045.1 | 0.856810997 | 1.906224687 | 0.056621061 |
| OPN1MW2 | ENST00000493123.1 | ENSG00000242428.1 | 0.898605983 | 1.993083204 | 0.046252337 |
| OPN1MW2 | ENST00000498693.1 | ENSG00000244198.1 | 0.926308688 | 2.0578766 | 0.039601981 |
| OPN1MW2 | ENST00000502421.1 | ENSG00000250284.1 | -0.840595675 | -1.855883905 | 0.063470108 |
| OPN1MW2 | ENST00000505196.1 | ENSG00000248131.1 | 0.873636998 | 1.95102384 | 0.05105421 |
| OPN1MW2 | ENST00000505498.1 | ENSG00000250908.1 | 0.879616717 | 1.964523115 | 0.049469458 |
| OPN1MW2 | ENST00000505556.1 | ENSG00000249409.1 | 0.980139909 | 2.19708538 | 0.028014349 |
| OPN1MW2 | ENST00000506100.1 | ENSG00000249409.1 | 0.981807363 | 2.215294083 | 0.026739886 |
| OPN1MW2 | ENST00000506305.1 | ENSG00000249994.1 | -0.814558827 | -1.808541173 | 0.070522315 |
| OPN1MW2 | ENST00000506791.1 | ENSG00000251131.1 | 0.877832383 | 1.973014119 | 0.048493954 |
| OPN1MW2 | ENST00000508083.1 | ENSG00000249343.1 | 0.993060228 | 2.211877377 | 0.026975139 |
| OPN1MW2 | ENST00000509036.1 | ENSG00000251131.1 | 0.87902939 | 1.988075562 | 0.046803334 |
| OPN1MW2 | ENST00000509192.1 | ENSG00000250765.1 | 0.860091768 | 1.916312645 | 0.055325312 |
| OPN1MW2 | ENST00000514877.1 | ENSG00000248685.1 | 0.868832447 | 1.951503959 | 0.050997126 |
| OPN1MW2 | ENST00000517300.1 | ENSG00000254144.2 | 0.843005956 | 1.891316292 | 0.058582135 |
| OPN1MW2 | ENST00000517716.1 | ENSG00000253515.1 | -0.833930205 | -1.85411281 | 0.063723022 |
| OPN1MW2 | ENST00000520603.1 | ENSG00000254001.1 | -0.821953911 | -1.836750751 | 0.066246699 |
| OPN1MW2 | ENST00000521307.1 | ENSG00000253177.1 | 0.829611031 | 1.861119346 | 0.062727325 |
| OPN1MW2 | ENST00000522547.1 | ENSG00000253430.1 | -0.900879034 | -2.00195545 | 0.045289523 |
| OPN1MW2 | ENST00000522600.1 | ENSG00000246582.2 | 0.803084728 | 1.80220831 | 0.071512638 |
| OPN1MW2 | ENST00000524335.1 | ENSG00000253716.1 | 0.804358703 | 1.800897684 | 0.071719008 |
| OPN1MW2 | ENST00000524818.1 | ENSG00000254473.1 | 0.918135626 | 2.049479071 | 0.040415292 |
| OPN1MW2 | ENST00000525133.1 | ENSG00000255375.1 | 0.86448507 | 1.949024577 | 0.05129249 |
| OPN1MW2 | ENST00000526186.1 | ENSG00000254510.1 | 0.925688977 | 2.068710353 | 0.038573275 |
| OPN1MW2 | ENST00000526611.1 | ENSG00000246982.2 | 0.881814198 | 1.988751058 | 0.046728687 |
| OPN1MW2 | ENST00000526935.1 | ENSG00000255372.1 | 0.886896683 | 1.967398362 | 0.049137303 |
| OPN1MW2 | ENST00000528887.1 | ENSG00000254501.1 | 0.863640361 | 1.920689593 | 0.054770852 |
| OPN1MW2 | ENST00000536141.1 | ENSG00000256969.1 | 0.912309167 | 2.044392665 | 0.040914771 |
| OPN1MW2 | ENST00000537269.1 | ENSG00000257084.1 | 0.889806403 | 1.995385688 | 0.046000831 |
| OPN1MW2 | ENST00000543072.1 | ENSG00000256092.2 | -0.899424212 | -1.997500461 | 0.045770844 |
| OPN1MW2 | ENST00000544089.1 | ENSG00000256273.1 | 0.868393191 | 1.927944708 | 0.053862004 |
| OPN1MW2 | ENST00000549878.1 | ENSG00000257284.1 | 0.866118023 | 1.951513187 | 0.050996029 |
| OPN1MW2 | ENST00000552469.1 | ENSG00000258325.1 | 0.895065658 | 1.980533587 | 0.047643603 |
| OPN1MW2 | ENST00000556786.1 | ENSG00000258525.1 | -0.856789998 | -1.902763339 | 0.057071433 |
| OPN1MW2 | ENST00000558312.1 | ENSG00000259176.1 | 0.827546109 | 1.840880133 | 0.065639126 |
| OPN1MW2 | ENST00000559569.1 | ENSG00000259760.1 | -0.837614368 | -1.86953528 | 0.06154838 |
| OPN1MW2 | ENST00000561567.1 | ENSG00000260177.1 | 0.800565812 | 1.815649776 | 0.069424113 |
| OPN1MW2 | ENST00000564417.1 | ENSG00000260137.1 | -0.827899903 | -1.859809126 | 0.062912536 |
| OPN1MW2 | ENST00000565823.1 | ENSG00000260686.1 | -0.867019271 | -1.911272391 | 0.055969585 |
| OPN1MW2 | ENST00000565829.1 | ENSG00000260148.1 | 0.915114749 | 2.048705933 | 0.040490879 |
| OPN1MW2 | ENST00000565965.1 | ENSG00000261172.1 | 0.800480925 | 1.792402664 | 0.073068488 |
| OPN1MW2 | ENST00000566170.1 | ENSG00000261071.1 | 0.887514259 | 1.984059998 | 0.047249152 |
| OPN1MW2 | ENST00000567395.1 | ENSG00000261090.1 | 0.808745862 | 1.784199605 | 0.074391236 |
| OPN1MW2 | ENST00000568033.1 | ENSG00000261480.1 | 0.888013366 | 1.971303618 | 0.048689158 |
| OPN1MW2 | ENST00000569981.1 | ENSG00000238045.5 | 0.846396176 | 1.918291986 | 0.055073998 |
| OPN1MW2 | ENST00000570512.1 | ENSG00000262768.1 | 0.882224463 | 1.953135613 | 0.050803528 |

| | | | | | |
|---|---|---|---|---|---|
| OPN1MW2 | ENST00000570843.1 | ENSG00000261889.1 | 0.951615583 | 2.165038308 | 0.030384752 |
| OPN1MW2 | ENST00000570929.1 | ENSG00000262223.2 | 0.95902656 | 2.135123359 | 0.032750935 |
| OPN1MW2 | ENST00000571815.1 | ENSG00000262810.1 | 0.81288473 | 1.827322742 | 0.067651258 |
| OPN1MW2 | ENST00000574460.1 | ENSG00000263051.1 | 0.802003586 | 1.795685653 | 0.072544525 |
| OPN1MW2 | ENST00000575139.1 | ENSG00000263072.1 | 0.942825903 | 2.113173038 | 0.03458596 |
| OPN1MW2 | ENST00000577064.1 | ENSG00000262823.1 | 0.866761753 | 1.91314058 | 0.055730058 |
| OPN1MW2 | ENST00000577678.1 | ENSG00000265415.1 | 0.85249305 | 1.911144367 | 0.055986031 |
| OPN1MW2 | ENST00000577698.1 | ENSG00000265100.1 | 0.854600471 | 1.871106232 | 0.061330358 |
| OPN1MW2 | ENST00000578265.1 | ENSG00000214719.7 | 0.895973807 | 1.964876584 | 0.049428524 |
| OPN1MW2 | ENST00000578757.1 | ENSG00000175061.13 | 0.84826858 | 1.892275635 | 0.058454266 |
| OPN1MW2 | ENST00000581905.1 | ENSG00000264235.1 | 0.868083179 | 1.937481908 | 0.052686463 |
| OPN1MW2 | ENST00000581940.1 | ENSG00000265484.1 | 0.87465701 | 1.951747644 | 0.050968173 |
| OPN1MW2 | ENST00000585072.1 | ENSG00000263745.1 | 0.866798423 | 1.96124395 | 0.049850572 |
| OPN1MW2 | ENST00000585559.1 | ENSG00000267117.1 | 0.848408483 | 1.913904352 | 0.055632378 |
| OPN1MW2 | ENST00000586010.1 | ENSG00000267606.1 | 0.867410404 | 1.923720678 | 0.054389605 |
| OPN1MW2 | ENST00000586051.1 | ENSG00000267576.1 | 0.883435241 | 1.985643522 | 0.04707292 |
| OPN1MW2 | ENST00000588799.1 | ENSG00000267275.1 | 0.808761427 | 1.815450751 | 0.069454668 |
| OPN1MW2 | ENST00000591174.1 | ENSG00000267289.1 | 0.842892714 | 1.883484199 | 0.059634779 |
| OPN1MW2 | ENST00000592400.1 | ENSG00000267735.1 | 0.887271346 | 2.000945852 | 0.045398226 |
| OPN1MW2 | ENST00000593632.1 | ENSG00000180279.5 | 0.890176122 | 1.969991938 | 0.048839295 |
| OPN1MW2 | ENST00000594590.2 | ENSG00000268199.2 | 0.903870314 | 2.035601268 | 0.04179041 |
| OPN1MW2 | ENST00000597169.1 | ENSG00000269720.1 | 0.92933265 | 2.080180687 | 0.037508963 |
| OPN1MW2 | ENST00000597309.1 | ENSG00000232098.2 | -0.886192967 | -1.96129969 | 0.049844073 |
| OPN1MW2 | ENST00000599259.1 | ENSG00000269352.1 | 0.897593171 | 1.999307172 | 0.045575129 |
| OPN1MW2 | ENST00000600234.1 | ENSG00000268078.1 | 0.902741311 | 2.018837941 | 0.043504066 |
| OPN1MW2 | ENST00000600534.1 | ENSG00000267858.1 | 0.927223097 | 2.056906071 | 0.039695262 |
| OPN1MW2 | ENST00000600726.1 | ENSG00000267858.1 | 0.812445476 | 1.786066066 | 0.074088562 |
| OPN1MW2 | ENST00000600889.1 | ENSG00000232675.3 | 0.822301884 | 1.842869414 | 0.065348079 |
| OPN1MW2 | ENST00000600959.1 | ENSG00000269303.1 | 0.812491167 | 1.79743131 | 0.072267174 |
| OPN1MW2 | ENST00000601692.1 | ENSG00000267874.1 | -0.837329828 | -1.868907559 | 0.061635677 |
| OPN1MW2 | ENST00000601735.1 | ENSG00000244513.2 | 0.905502552 | 2.022329171 | 0.043142357 |
| OPN1MW2 | ENST00000602532.1 | ENSG00000270091.1 | 0.863261681 | 1.916736048 | 0.055271473 |
| OPN1MW2 | ENST00000602809.1 | ENSG00000270105.1 | -0.826072334 | -1.852113142 | 0.064009578 |
| OPN1MW2 | ENST00000602872.1 | ENSG00000270067.1 | 0.894173871 | 2.007776981 | 0.044666996 |
| OPN1MW2 | ENST00000604142.1 | ENSG00000271308.1 | 0.849491732 | 1.910249145 | 0.056101142 |
| OPN1MW2 | ENST00000606277.1 | ENSG00000272145.1 | 0.871727676 | 1.944705312 | 0.051810456 |
| OPN1MW2 | ENST00000606374.1 | ENSG00000272312.1 | -0.847204408 | -1.879683289 | 0.060151254 |
| OPN1MW2 | ENST00000606377.1 | ENSG00000272286.1 | -0.851574625 | -1.892850781 | 0.058377716 |
| OPN1MW2 | ENST00000606470.1 | ENSG00000271913.1 | 0.834010616 | 1.834596256 | 0.066565534 |
| OPN1MW2 | ENST00000607224.1 | ENSG00000272521.1 | 0.816329617 | 1.805971166 | 0.070922844 |
| OPN1MW2 | ENST00000607476.1 | ENSG00000272540.1 | 0.823328431 | 1.840159369 | 0.065744843 |
| OPN1MW2 | ENST00000609067.1 | ENSG00000272849.1 | 0.904859959 | 2.0181739 | 0.043573153 |
| OPN1MW2 | ENST00000609281.1 | ENSG00000273320.1 | 0.880603381 | 1.985769972 | 0.047058871 |
| OPN1MW2 | ENST00000609972.1 | ENSG00000230651.3 | 0.916196426 | 2.064545766 | 0.038965999 |
| OPN1MW2 | ENST00000610145.1 | ENSG00000273175.1 | 0.938230798 | 2.121216002 | 0.033903629 |
| OPN1MW2 | NR_003604.2 | ZFAS1 | 0.945890986 | 2.120033919 | 0.034003185 |
| OPN1MW2 | NR_003605.1 | ZFAS1 | 0.941718998 | 2.111017696 | 0.034770791 |
| OPN1MW2 | NR_003606.2 | ZFAS1 | 0.955635899 | 2.161074567 | 0.03068958 |
| OPN1MW2 | NR_026802.1 | FAM74A4 | 0.836407119 | 1.868909667 | 0.061635383 |
| OPN1MW2 | NR_027271.1 | CIRBP-AS1 | 0.928963728 | 2.078235813 | 0.037687645 |
| OPN1MW2 | NR_027334.2 | MZF1-AS1 | 0.941740818 | 2.125148314 | 0.033574239 |

| | | | | | |
|---|---|---|---|---|---|
| OPN1MW2 | NR_034037.1 | LINC00582 | -0.819589933 | -1.859777075 | 0.062917072 |
| OPN1MW2 | NR_036480.1 | VPS9D1-AS1 | 0.819338213 | 1.812240829 | 0.069948993 |
| OPN1MW2 | NR_036658.1 | ZFAS1 | 0.957263372 | 2.138348577 | 0.032488463 |
| OPN1MW2 | NR_044996.1 | HCG23 | 0.921506264 | 2.087702721 | 0.036824659 |
| OPN1MW2 | NR_046742.2 | ZNF630-AS1 | -0.863109791 | -1.937952682 | 0.052628996 |
| OPN1MW2 | NR_072981.1 | LINC00957 | 0.812241561 | 1.829518447 | 0.067321978 |
| OPN1MW2 | NR_072982.1 | LINC00957 | 0.809146311 | 1.804934691 | 0.071084902 |
| OPN1MW2 | NR_103790.1 | LINC00581 | -0.894294388 | -2.005064379 | 0.044956165 |
| OPN1MW2 | NR_105010.1 | LINC01333 | 0.952344628 | 2.151870693 | 0.03140754 |
| OPN1MW2 | NR_108036.1 | CFAP58-AS1 | 0.959052898 | 2.144531025 | 0.031990362 |
| OPN1MW2 | NR_109886.1 | RALY-AS1 | 0.8242326 | 1.862885205 | 0.062478419 |
| OPN1MW2 | NR_110480.1 | LOC101927079 | 0.80232523 | 1.810707191 | 0.070186189 |
| OPN1MW2 | NR_110481.1 | LOC101927079 | 0.827546109 | 1.858393809 | 0.063113111 |
| OPN1MW2 | NR_110568.1 | LOC101927661 | 0.824505336 | 1.809254836 | 0.070411422 |
| OPN1MW2 | NR_110998.1 | FAM74A4 | 0.836407119 | 1.886690088 | 0.059202022 |
| OPN1MW2 | NR_120335.1 | LOC101928414 | 0.86741777 | 1.941262154 | 0.052226488 |
| OPN1MW2 | NR_121188.1 | PGM5P3-AS1 | -0.850212859 | -1.907937315 | 0.056399319 |
| OPN1MW2 | NR_121189.1 | PGM5P3-AS1 | -0.940538176 | -2.090915063 | 0.036535679 |
| OPN1MW2 | NR_126166.1 | FAM74A7 | 0.818063984 | 1.846570157 | 0.064809463 |
| OPN1MW2 | NR_130144.1 | LOC104968399 | 0.868083179 | 1.948384059 | 0.051369026 |
| OPN1MW2 | NR_135032.1 | LOC105369635 | 0.889806403 | 1.981727436 | 0.047509755 |
| OPN1MW2 | NR_135644.1 | LOC105371506 | -0.804516123 | -1.805221037 | 0.0710401 |
| OPN1MW2 | NR_138084.1 | HCG24 | 0.897871087 | 2.004367533 | 0.045030705 |
| OR10AG1 | ENST00000414098.2 | ENSG00000234428.2 | 0.83455098 | 1.893317747 | 0.058315626 |
| OR10AG1 | ENST00000414896.1 | ENSG00000223374.1 | 0.811186298 | 1.819128877 | 0.06889177 |
| OR10AG1 | ENST00000415106.1 | ENSG00000226733.1 | 0.871164949 | 1.94194858 | 0.052143325 |
| OR10AG1 | ENST00000415205.1 | ENSG00000182057.4 | -0.954283542 | -2.130585896 | 0.033123273 |
| OR10AG1 | ENST00000417260.1 | ENSG00000231734.4 | 0.88166038 | 1.988059503 | 0.04680511 |
| OR10AG1 | ENST00000418387.1 | ENSG00000235056.1 | 0.861229542 | 1.931208944 | 0.053457218 |
| OR10AG1 | ENST00000421006.1 | ENSG00000234548.1 | 0.881370133 | 1.962080253 | 0.049753142 |
| OR10AG1 | ENST00000422763.1 | ENSG00000231131.2 | 0.830076292 | 1.861096566 | 0.062730541 |
| OR10AG1 | ENST00000423428.1 | ENSG00000224048.1 | 0.859318687 | 1.898605996 | 0.057616299 |
| OR10AG1 | ENST00000425881.1 | ENSG00000239636.1 | -0.822318371 | -1.846897788 | 0.064761955 |
| OR10AG1 | ENST00000426302.1 | ENSG00000230454.1 | -0.949329994 | -2.105289164 | 0.035266145 |
| OR10AG1 | ENST00000427524.1 | ENSG00000236065.2 | -0.835287762 | -1.853650846 | 0.063789128 |
| OR10AG1 | ENST00000429080.1 | ENSG00000233047.1 | 0.906137184 | 2.023716636 | 0.042999316 |
| OR10AG1 | ENST00000430247.1 | ENSG00000232855.2 | 0.827597101 | 1.846266505 | 0.064853519 |
| OR10AG1 | ENST00000430920.1 | ENSG00000234203.1 | -0.872261367 | -1.934186228 | 0.053090235 |
| OR10AG1 | ENST00000431730.1 | ENSG00000237401.2 | -0.84151116 | -1.886235319 | 0.059263251 |
| OR10AG1 | ENST00000434292.1 | ENSG00000229796.1 | 0.885968411 | 1.984175432 | 0.047236286 |
| OR10AG1 | ENST00000435287.1 | ENSG00000227220.1 | -0.906073058 | -2.033538386 | 0.041998159 |
| OR10AG1 | ENST00000436515.1 | ENSG00000224521.1 | 0.856058987 | 1.916898689 | 0.055250804 |
| OR10AG1 | ENST00000438173.2 | ENSG00000227733.4 | 0.898146037 | 2.02774276 | 0.042586509 |
| OR10AG1 | ENST00000438623.1 | ENSG00000224521.1 | 0.864201888 | 1.92489342 | 0.054242693 |
| OR10AG1 | ENST00000438969.2 | ENSG00000228031.2 | 0.86930793 | 1.967273324 | 0.049151709 |
| OR10AG1 | ENST00000442017.1 | ENSG00000229660.1 | -0.886293495 | -1.976611288 | 0.048085585 |
| OR10AG1 | ENST00000442829.1 | ENSG00000225284.1 | -0.869518997 | -1.90515269 | 0.056760226 |
| OR10AG1 | ENST00000445233.1 | ENSG00000233928.1 | 0.83741549 | 1.883684776 | 0.059607627 |
| OR10AG1 | ENST00000446562.1 | ENSG00000233896.1 | -0.852755088 | -1.894206007 | 0.058197671 |
| OR10AG1 | ENST00000446816.1 | ENSG00000204685.5 | -0.858861672 | -1.936919266 | 0.052755213 |
| OR10AG1 | ENST00000448674.1 | ENSG00000235119.1 | -0.85390216 | -1.886145574 | 0.05927534 |

| | | | | | |
|---|---|---|---|---|---|
| OR10AG1 | ENST00000448858.1 | ENSG00000237734.1 | 0.979954698 | 2.186992523 | 0.02874307 |
| OR10AG1 | ENST00000451034.1 | ENSG00000229805.1 | 0.855056544 | 1.906183943 | 0.056626345 |
| OR10AG1 | ENST00000451090.1 | ENSG00000235215.2 | 0.938101445 | 2.099841747 | 0.035742765 |
| OR10AG1 | ENST00000455373.1 | ENSG00000226097.1 | 0.970501358 | 2.194828239 | 0.028175921 |
| OR10AG1 | ENST00000457043.1 | ENSG00000231365.1 | 0.864846171 | 1.915456877 | 0.055434264 |
| OR10AG1 | ENST00000457632.1 | ENSG00000234248.1 | 0.886921072 | 1.977211128 | 0.04801777 |
| OR10AG1 | ENST00000457998.2 | ENSG00000233006.2 | -0.835000473 | -1.855645859 | 0.063504053 |
| OR10AG1 | ENST00000468165.1 | ENSG00000239480.1 | -0.84641116 | -1.866851858 | 0.061922278 |
| OR10AG1 | ENST00000483283.1 | ENSG00000240571.1 | 0.835883207 | 1.85210959 | 0.064010088 |
| OR10AG1 | ENST00000488310.1 | ENSG00000240449.1 | -0.884676297 | -1.992560891 | 0.046309551 |
| OR10AG1 | ENST00000489690.1 | ENSG00000243944.1 | 0.816440602 | 1.810296845 | 0.070249766 |
| OR10AG1 | ENST00000503505.1 | ENSG00000248629.1 | 0.8544939 | 1.90770911 | 0.056428824 |
| OR10AG1 | ENST00000503723.1 | ENSG00000250472.1 | 0.932335935 | 2.081714087 | 0.037368593 |
| OR10AG1 | ENST00000504578.1 | ENSG00000251513.1 | 0.856846241 | 1.922888929 | 0.054494 |
| OR10AG1 | ENST00000506379.1 | ENSG00000240152.2 | 0.892182825 | 1.974783272 | 0.048292748 |
| OR10AG1 | ENST00000506723.2 | ENSG00000249484.4 | 0.950981911 | 2.128948204 | 0.033258546 |
| OR10AG1 | ENST00000509453.1 | ENSG00000249145.1 | -0.827086708 | -1.845720915 | 0.064932739 |
| OR10AG1 | ENST00000509629.1 | ENSG00000250164.1 | -0.856063652 | -1.91942506 | 0.054930562 |
| OR10AG1 | ENST00000515128.1 | ENSG00000248215.1 | 0.812550884 | 1.812388312 | 0.069926218 |
| OR10AG1 | ENST00000518837.1 | ENSG00000253947.1 | 0.966243674 | 2.189175257 | 0.028584104 |
| OR10AG1 | ENST00000519005.1 | ENSG00000253507.1 | 0.827252813 | 1.856400468 | 0.063396498 |
| OR10AG1 | ENST00000520192.1 | ENSG00000253807.1 | 0.845888796 | 1.882013106 | 0.059834236 |
| OR10AG1 | ENST00000521294.1 | ENSG00000253664.1 | -0.907466835 | -2.029242029 | 0.042433645 |
| OR10AG1 | ENST00000521653.1 | ENSG00000253301.1 | -0.953688715 | -2.119235636 | 0.034070558 |
| OR10AG1 | ENST00000522281.1 | ENSG00000253376.1 | 0.957207129 | 2.162953045 | 0.030544791 |
| OR10AG1 | ENST00000522300.1 | ENSG00000249484.4 | 0.932417788 | 2.040349121 | 0.041315566 |
| OR10AG1 | ENST00000522390.1 | ENSG00000254262.1 | 0.895796866 | 2.030460774 | 0.042309724 |
| OR10AG1 | ENST00000523806.1 | ENSG00000253616.1 | -0.90578353 | -2.025693324 | 0.042796221 |
| OR10AG1 | ENST00000524133.1 | ENSG00000253174.2 | -0.815070643 | -1.834325598 | 0.066605677 |
| OR10AG1 | ENST00000525855.1 | ENSG00000254746.1 | -0.842409239 | -1.874796267 | 0.060820757 |
| OR10AG1 | ENST00000527086.1 | ENSG00000255182.1 | -0.835998328 | -1.863911232 | 0.062334171 |
| OR10AG1 | ENST00000527274.2 | ENSG00000255517.2 | -0.947532273 | -2.110356036 | 0.0348277 |
| OR10AG1 | ENST00000527727.1 | ENSG00000255227.1 | 0.826769751 | 1.844898969 | 0.065052237 |
| OR10AG1 | ENST00000529247.1 | ENSG00000254741.1 | -0.819782463 | -1.819161177 | 0.068886844 |
| OR10AG1 | ENST00000531977.1 | ENSG00000224023.6 | 0.885515249 | 1.97851484 | 0.047870656 |
| OR10AG1 | ENST00000534178.1 | ENSG00000255120.1 | -0.822078549 | -1.830994208 | 0.067101408 |
| OR10AG1 | ENST00000535914.1 | ENSG00000256894.1 | 0.944818144 | 2.099768705 | 0.035749192 |
| OR10AG1 | ENST00000537032.1 | ENSG00000255933.1 | -0.927514173 | -2.082754921 | 0.037273568 |
| OR10AG1 | ENST00000537850.1 | ENSG00000251002.3 | -0.938295288 | -2.117011512 | 0.034258871 |
| OR10AG1 | ENST00000538641.1 | ENSG00000256422.1 | 0.822811607 | 1.873830332 | 0.060953814 |
| OR10AG1 | ENST00000541391.1 | ENSG00000256268.1 | 0.824605792 | 1.86273969 | 0.062498899 |
| OR10AG1 | ENST00000543403.1 | ENSG00000256684.1 | 0.918242503 | 2.032354893 | 0.042117741 |
| OR10AG1 | ENST00000548210.1 | ENSG00000257784.1 | -0.909142647 | -2.039390667 | 0.041411053 |
| OR10AG1 | ENST00000549616.1 | ENSG00000258168.1 | 0.925377629 | 2.086379309 | 0.036944278 |
| OR10AG1 | ENST00000550263.1 | ENSG00000257605.1 | -0.804010246 | -1.774182326 | 0.076032997 |
| OR10AG1 | ENST00000550279.1 | ENSG00000258338.1 | 0.921368595 | 2.068608605 | 0.03858283 |
| OR10AG1 | ENST00000551135.1 | ENSG00000258294.1 | 0.875995783 | 1.960032396 | 0.049992004 |
| OR10AG1 | ENST00000551699.1 | ENSG00000257467.1 | 0.860189529 | 1.909659197 | 0.056177108 |
| OR10AG1 | ENST00000552541.1 | ENSG00000258294.1 | 0.913011049 | 2.0321051 | 0.042143017 |
| OR10AG1 | ENST00000552634.1 | ENSG00000257496.1 | -0.933860743 | -2.089777954 | 0.036637751 |
| OR10AG1 | ENST00000553954.1 | ENSG00000259052.1 | -0.861064052 | -1.936904027 | 0.052757076 |

| | | | | | |
|---|---|---|---|---|---|
| OR10AG1 | ENST00000554431.1 | ENSG00000258616.1 | 0.804045398 | 1.809226502 | 0.070415822 |
| OR10AG1 | ENST00000555460.1 | ENSG00000259042.1 | -0.852074228 | -1.886636508 | 0.059209233 |
| OR10AG1 | ENST00000555913.1 | ENSG00000259077.1 | 0.898996543 | 1.985640826 | 0.04707322 |
| OR10AG1 | ENST00000557368.1 | ENSG00000258444.1 | -0.827682914 | -1.876404366 | 0.060599776 |
| OR10AG1 | ENST00000557602.1 | ENSG00000258616.1 | 0.821650635 | 1.844650994 | 0.065088324 |
| OR10AG1 | ENST00000558221.1 | ENSG00000259704.1 | -0.898364028 | -2.033295205 | 0.042022707 |
| OR10AG1 | ENST00000558237.1 | ENSG00000259684.1 | 0.853734025 | 1.907230834 | 0.056490702 |
| OR10AG1 | ENST00000560963.1 | ENSG00000259370.1 | -0.824374 | -1.81963143 | 0.068815152 |
| OR10AG1 | ENST00000562191.1 | ENSG00000261292.1 | 0.89875586 | 2.018656126 | 0.043522973 |
| OR10AG1 | ENST00000562995.1 | ENSG00000261253.1 | -0.836858163 | -1.87159643 | 0.061262458 |
| OR10AG1 | ENST00000563610.1 | ENSG00000260051.1 | -0.959524683 | -2.136659216 | 0.032625719 |
| OR10AG1 | ENST00000563611.1 | ENSG00000261583.1 | -0.886258391 | -2.002500995 | 0.045230876 |
| OR10AG1 | ENST00000563855.1 | ENSG00000260658.1 | 0.822335042 | 1.843082522 | 0.065316963 |
| OR10AG1 | ENST00000564809.1 | ENSG00000261471.1 | -0.970010434 | -2.143781393 | 0.032050407 |
| OR10AG1 | ENST00000565310.1 | ENSG00000261118.1 | -0.955138728 | -2.143653274 | 0.032060679 |
| OR10AG1 | ENST00000565735.1 | ENSG00000261213.1 | 0.971544552 | 2.19668146 | 0.028043204 |
| OR10AG1 | ENST00000566449.1 | ENSG00000259791.1 | 0.839959381 | 1.876732039 | 0.060554829 |
| OR10AG1 | ENST00000568410.1 | ENSG00000260277.1 | 0.847126845 | 1.878202184 | 0.060353512 |
| OR10AG1 | ENST00000569313.1 | ENSG00000261604.1 | 0.846785544 | 1.886093858 | 0.059282308 |
| OR10AG1 | ENST00000570700.1 | ENSG00000263011.1 | -0.878220743 | -1.96556095 | 0.049349349 |
| OR10AG1 | ENST00000571660.1 | ENSG00000262848.1 | -0.813809867 | -1.833279944 | 0.06676095 |
| OR10AG1 | ENST00000573260.1 | ENSG00000262482.1 | 0.804978527 | 1.780797678 | 0.074945509 |
| OR10AG1 | ENST00000576021.1 | ENSG00000262413.1 | -0.826333712 | -1.850023409 | 0.064310176 |
| OR10AG1 | ENST00000576271.1 | ENSG00000263342.1 | -0.924064272 | -2.077501449 | 0.037755302 |
| OR10AG1 | ENST00000577853.1 | ENSG00000264207.1 | -0.985923837 | -2.222479652 | 0.026250908 |
| OR10AG1 | ENST00000579775.1 | ENSG00000264108.1 | -0.953296571 | -2.11852218 | 0.034130869 |
| OR10AG1 | ENST00000580622.1 | ENSG00000264634.1 | -0.861994118 | -1.942191232 | 0.052113954 |
| OR10AG1 | ENST00000582044.1 | ENSG00000263715.2 | -0.851612118 | -1.916392357 | 0.055315173 |
| OR10AG1 | ENST00000585684.1 | ENSG00000267057.1 | 0.827930649 | 1.820296179 | 0.068713913 |
| OR10AG1 | ENST00000585761.1 | ENSG00000267198.1 | -0.905394183 | -2.027700489 | 0.042590826 |
| OR10AG1 | ENST00000585810.1 | ENSG00000236172.2 | -0.85138805 | -1.902275786 | 0.05713511 |
| OR10AG1 | ENST00000586348.1 | ENSG00000267198.1 | -0.873351525 | -1.957513822 | 0.050287088 |
| OR10AG1 | ENST00000588402.1 | ENSG00000267006.1 | 0.910449268 | 2.033144349 | 0.042037941 |
| OR10AG1 | ENST00000588842.1 | ENSG00000235779.3 | 0.941121239 | 2.08566596 | 0.037008892 |
| OR10AG1 | ENST00000589380.1 | ENSG00000267488.1 | -0.817519198 | -1.823596795 | 0.068213051 |
| OR10AG1 | ENST00000591621.1 | ENSG00000232116.2 | 0.920680294 | 2.052896095 | 0.040082656 |
| OR10AG1 | ENST00000592022.1 | ENSG00000267383.2 | 0.865866048 | 1.948139975 | 0.051398217 |
| OR10AG1 | ENST00000592525.1 | ENSG00000267214.1 | -0.888469546 | -1.991862855 | 0.046386108 |
| OR10AG1 | ENST00000592816.1 | ENSG00000236172.2 | -0.862412607 | -1.947457934 | 0.051479858 |
| OR10AG1 | ENST00000593175.1 | ENSG00000229036.3 | 0.907191737 | 2.052939888 | 0.040078408 |
| OR10AG1 | ENST00000594776.1 | ENSG00000269807.1 | -0.917784999 | -2.057234442 | 0.03966368 |
| OR10AG1 | ENST00000594850.1 | ENSG00000268093.1 | -0.838496981 | -1.86688967 | 0.061916997 |
| OR10AG1 | ENST00000595007.1 | ENSG00000231876.3 | 0.801791322 | 1.775376687 | 0.075835711 |
| OR10AG1 | ENST00000596091.1 | ENSG00000227733.4 | 0.925536176 | 2.051655822 | 0.040203123 |
| OR10AG1 | ENST00000596567.1 | ENSG00000226647.2 | 0.975371789 | 2.164627226 | 0.030416244 |
| OR10AG1 | ENST00000596887.1 | ENSG00000237031.3 | 0.872696811 | 1.951883589 | 0.050952027 |
| OR10AG1 | ENST00000597680.1 | ENSG00000269574.1 | 0.842936076 | 1.884042066 | 0.059559286 |
| OR10AG1 | ENST00000597755.1 | ENSG00000236194.2 | 0.890196336 | 1.980400288 | 0.047658568 |
| OR10AG1 | ENST00000598092.1 | ENSG00000228065.6 | 0.914296816 | 2.047409926 | 0.040617853 |
| OR10AG1 | ENST00000598887.1 | ENSG00000268475.1 | -0.85188157 | -1.907915248 | 0.056402171 |
| OR10AG1 | ENST00000600242.1 | ENSG00000269583.1 | -0.862742992 | -1.920164924 | 0.054837071 |

| | | | | | |
|---|---|---|---|---|---|
| OR10AG1 | ENST00000600726.1 | ENSG00000267858.1 | -0.804530115 | -1.77150834 | 0.076476207 |
| OR10AG1 | ENST00000602405.1 | ENSG00000269928.1 | -0.826882635 | -1.844915429 | 0.065049842 |
| OR10AG1 | ENST00000602443.1 | ENSG00000270076.1 | 0.817222683 | 1.821565203 | 0.068520985 |
| OR10AG1 | ENST00000602881.1 | ENSG00000269965.1 | 0.832902311 | 1.862128679 | 0.062584954 |
| OR10AG1 | ENST00000604183.1 | ENSG00000271185.1 | -0.95790151 | -2.126951311 | 0.033424129 |
| OR10AG1 | ENST00000604312.1 | ENSG00000270947.1 | 0.836154498 | 1.856575243 | 0.063371609 |
| OR10AG1 | ENST00000605692.1 | ENSG00000270810.1 | -0.849638863 | -1.91503596 | 0.055487918 |
| OR10AG1 | ENST00000606010.1 | ENSG00000272249.1 | 0.839318064 | 1.879826645 | 0.060131708 |
| OR10AG1 | ENST00000606482.1 | ENSG00000272416.1 | 0.820799638 | 1.81350998 | 0.069753201 |
| OR10AG1 | ENST00000606869.1 | ENSG00000272349.1 | 0.862422155 | 1.922421455 | 0.054552748 |
| OR10AG1 | ENST00000606909.1 | ENSG00000271821.1 | -0.841905289 | -1.877064775 | 0.060509217 |
| OR10AG1 | ENST00000607148.1 | ENSG00000272477.1 | 0.876777433 | 1.973328659 | 0.04845813 |
| OR10AG1 | ENST00000607943.1 | ENSG00000273188.1 | -0.846133422 | -1.892235832 | 0.058459566 |
| OR10AG1 | ENST00000608088.1 | ENSG00000272632.1 | 0.832465014 | 1.86733114 | 0.061855359 |
| OR10AG1 | ENST00000608133.1 | ENSG00000273193.1 | 0.850412576 | 1.899771429 | 0.057463122 |
| OR10AG1 | ENST00000608259.1 | ENSG00000272627.1 | 0.909448144 | 2.051543586 | 0.04021404 |
| OR10AG1 | ENST00000608489.1 | ENSG00000272716.1 | -0.938134924 | -2.095367403 | 0.036138345 |
| OR10AG1 | ENST00000608759.1 | ENSG00000273464.1 | 0.878354981 | 1.968651731 | 0.048993098 |
| OR10AG1 | ENST00000609113.1 | ENSG00000272827.1 | -0.815698103 | -1.821682395 | 0.068503191 |
| OR10AG1 | ENST00000609807.1 | ENSG00000272700.1 | 0.902897818 | 2.012906159 | 0.044124503 |
| OR10AG1 | ENST00000609955.1 | ENSG00000273275.1 | 0.856869137 | 1.933584901 | 0.053164185 |
| OR10AG1 | ENST00000610270.1 | ENSG00000272576.1 | 0.820047527 | 1.841687493 | 0.065520875 |
| OR10AG1 | NR_027067.1 | LINC00114 | -0.845694229 | -1.873313353 | 0.061025127 |
| OR10AG1 | NR_027402.1 | FAM223B | -0.917568459 | -2.027735368 | 0.042587264 |
| OR10AG1 | NR_033914.1 | LINC00254 | 0.836036015 | 1.868835693 | 0.061645677 |
| OR10AG1 | NR_034131.1 | LINC00272 | -0.802114043 | -1.812027696 | 0.069981917 |
| OR10AG1 | NR_038421.1 | LINC01220 | -0.895541534 | -2.020648888 | 0.043316124 |
| OR10AG1 | NR_038923.1 | SSSCA1-AS1 | -0.882193197 | -1.985651559 | 0.047072027 |
| OR10AG1 | NR_040049.1 | SDCBP2-AS1 | -0.865887918 | -1.934439349 | 0.053059132 |
| OR10AG1 | NR_046871.1 | LINC00333 | 0.935967776 | 2.100892609 | 0.035650394 |
| OR10AG1 | NR_047698.1 | VWC2L-IT1 | 0.838177133 | 1.871765493 | 0.061239054 |
| OR10AG1 | NR_102746.1 | ROPN1L-AS1 | -0.865912756 | -1.912532693 | 0.055807903 |
| OR10AG1 | NR_108106.1 | LINC01135 | -0.820447163 | -1.808301541 | 0.070559582 |
| OR10AG1 | NR_109831.1 | RASSF1-AS1 | -0.864015249 | -1.923380577 | 0.054432272 |
| OR10AG1 | NR_109877.1 | LINC01470 | 0.944330776 | 2.091388628 | 0.036493241 |
| OR10AG1 | NR_110117.1 | LOC101927769 | 0.901883931 | 2.02572567 | 0.042792905 |
| OR10AG1 | NR_110245.1 | LOC101929282 | 0.969493416 | 2.17381332 | 0.029719158 |
| OR10AG1 | NR_110556.1 | LOC102724890 | 0.947710562 | 2.118090913 | 0.034167369 |
| OR10AG1 | NR_110630.1 | LOC101927478 | -0.963483801 | -2.144689433 | 0.031977686 |
| OR10AG1 | NR_110824.1 | LINC01986 | 0.833432728 | 1.850992893 | 0.064170575 |
| OR10AG1 | NR_111951.1 | LINC00869 | -0.818861495 | -1.822605487 | 0.068363164 |
| OR10AG1 | NR_111953.1 | LINC00869 | -0.816679403 | -1.832479374 | 0.066880032 |
| OR10AG1 | NR_125849.1 | LOC101928140 | 0.934751985 | 2.086204712 | 0.036960084 |
| OR10AG1 | NR_131204.1 | XACT | -0.879122226 | -1.961196949 | 0.049856053 |
| OR10AG1 | NR_131243.1 | SMCR2 | -0.826174602 | -1.859172665 | 0.063002668 |
| OR10AG1 | NR_134520.1 | LOC727993 | -0.850431058 | -1.904311261 | 0.056869659 |
| OR10AG1 | NR_134610.1 | LOC105375014 | -0.803909126 | -1.783055801 | 0.07457722 |
| OR10AG1 | NR_134632.1 | LOC105373051 | -0.863181708 | -1.940815415 | 0.052280671 |
| OR10AG1 | NR_135040.1 | LOC101927038 | -0.945995761 | -2.114219177 | 0.034496552 |
| OR10AG1 | NR_135041.1 | LOC101927038 | -0.856662588 | -1.917641653 | 0.055156465 |
| OR10AG1 | NR_135097.1 | LOC105369443 | 0.918242503 | 2.051611419 | 0.040207442 |

| | | | | | |
|---|---|---|---|---|---|
| OR10AG1 | NR_135584.1 | LOC101927596 | -0.843640247 | -1.883102837 | 0.059686433 |
| OR10AG1 | NR_136215.1 | VCAN-AS1 | 0.85163068 | 1.924236074 | 0.054324999 |
| OR10AG1 | NR_136218.1 | MEF2C-AS1 | 0.827016201 | 1.855624485 | 0.063507101 |
| OR13C2 | ENST00000411489.1 | ENSG00000227112.1 | 0.815844608 | 1.81727392 | 0.069175181 |
| OR13C2 | ENST00000412445.1 | ENSG00000238042.1 | -0.873139539 | -1.92433587 | 0.054312497 |
| OR13C2 | ENST00000412812.1 | ENSG00000225342.1 | 0.871912542 | 1.96215875 | 0.049744005 |
| OR13C2 | ENST00000418416.1 | ENSG00000226218.1 | 0.961938288 | 2.140429394 | 0.032320081 |
| OR13C2 | ENST00000428391.1 | ENSG00000224691.1 | -0.831676519 | -1.843540514 | 0.065250132 |
| OR13C2 | ENST00000429916.1 | ENSG00000227708.1 | 0.875519705 | 1.976449611 | 0.048103877 |
| OR13C2 | ENST00000432265.1 | ENSG00000231170.1 | -0.815467535 | -1.842097136 | 0.065460943 |
| OR13C2 | ENST00000433249.1 | ENSG00000236556.1 | 0.824929261 | 1.879435919 | 0.060184996 |
| OR13C2 | ENST00000437864.1 | ENSG00000231521.1 | 0.964826628 | 2.164825557 | 0.030401047 |
| OR13C2 | ENST00000438488.1 | ENSG00000223812.1 | 0.806740992 | 1.821367173 | 0.068551062 |
| OR13C2 | ENST00000441295.1 | ENSG00000233960.1 | -0.801891102 | -1.803507352 | 0.071308572 |
| OR13C2 | ENST00000441666.1 | ENSG00000230379.1 | 0.817811037 | 1.835642175 | 0.066410595 |
| OR13C2 | ENST00000441991.1 | ENSG00000231210.2 | -0.828366169 | -1.845508408 | 0.064963616 |
| OR13C2 | ENST00000442876.1 | ENSG00000233894.1 | 0.99467055 | 2.211347876 | 0.027011756 |
| OR13C2 | ENST00000443066.2 | ENSG00000237633.2 | 0.822575882 | 1.839362615 | 0.065861869 |
| OR13C2 | ENST00000448748.1 | ENSG00000231238.1 | 0.835437139 | 1.839174027 | 0.065889594 |
| OR13C2 | ENST00000450779.1 | ENSG00000228858.1 | 0.970466539 | 2.16174177 | 0.030638086 |
| OR13C2 | ENST00000452511.1 | ENSG00000231876.3 | 0.805808872 | 1.787156408 | 0.073912213 |
| OR13C2 | ENST00000456999.1 | ENSG00000230690.1 | 0.82862367 | 1.852715322 | 0.063923173 |
| OR13C2 | ENST00000485338.1 | ENSG00000239641.1 | 0.858453039 | 1.885909148 | 0.059307199 |
| OR13C2 | ENST00000490375.1 | ENSG00000240032.1 | 0.938280935 | 2.076239901 | 0.037871769 |
| OR13C2 | ENST00000502467.1 | ENSG00000250530.1 | 0.85032064 | 1.909815015 | 0.056157035 |
| OR13C2 | ENST00000510274.1 | ENSG00000245864.2 | 0.90635081 | 2.019944716 | 0.043389122 |
| OR13C2 | ENST00000514966.1 | ENSG00000251310.1 | -0.800898279 | -1.802837162 | 0.071413792 |
| OR13C2 | ENST00000528607.1 | ENSG00000254604.1 | 0.984806509 | 2.197496409 | 0.027985013 |
| OR13C2 | ENST00000532123.1 | ENSG00000255555.1 | 0.879184368 | 2.003402541 | 0.045134099 |
| OR13C2 | ENST00000537492.1 | ENSG00000256637.2 | 0.894543248 | 1.977418493 | 0.047994345 |
| OR13C2 | ENST00000545158.1 | ENSG00000256011.1 | 0.801695161 | 1.778871009 | 0.075260912 |
| OR13C2 | ENST00000545357.1 | ENSG00000256862.1 | -0.832107582 | -1.834477472 | 0.066583149 |
| OR13C2 | ENST00000547547.1 | ENSG00000257241.1 | -0.839651618 | -1.898418894 | 0.057640923 |
| OR13C2 | ENST00000550138.1 | ENSG00000257467.1 | 0.950265757 | 2.115703065 | 0.03437007 |
| OR13C2 | ENST00000557817.1 | ENSG00000259176.1 | 0.853776147 | 1.922720133 | 0.054515207 |
| OR13C2 | ENST00000561544.1 | ENSG00000261532.1 | -0.916220596 | -2.037626905 | 0.041587259 |
| OR13C2 | ENST00000562970.1 | ENSG00000260145.1 | -0.871860808 | -1.977443922 | 0.047991473 |
| OR13C2 | ENST00000566521.1 | ENSG00000261629.1 | 0.957732334 | 2.132344119 | 0.032978568 |
| OR13C2 | ENST00000569328.1 | ENSG00000261638.1 | 0.814066521 | 1.830680142 | 0.067148299 |
| OR13C2 | ENST00000570413.1 | ENSG00000263167.1 | -0.904539544 | -2.041621405 | 0.0411891 |
| OR13C2 | ENST00000584139.1 | ENSG00000263388.1 | 0.92652038 | 2.120772205 | 0.033940977 |
| OR13C2 | ENST00000585877.1 | ENSG00000267249.1 | -0.937773531 | -2.06727852 | 0.038707917 |
| OR13C2 | ENST00000590046.1 | ENSG00000266950.1 | -0.826446734 | -1.850418961 | 0.064253188 |
| OR13C2 | ENST00000593568.1 | ENSG00000228065.6 | -0.871912542 | -1.927533894 | 0.053913128 |
| OR13C2 | ENST00000596473.1 | ENSG00000268650.3 | 0.873475283 | 1.968612046 | 0.048997658 |
| OR13C2 | ENST00000597865.1 | ENSG00000268108.1 | 0.934650826 | 2.098085963 | 0.035897552 |
| OR13C2 | ENST00000601752.1 | ENSG00000268051.1 | 0.819912576 | 1.810675167 | 0.070191149 |
| OR13C2 | ENST00000602418.1 | ENSG00000232295.3 | -0.843917546 | -1.882911851 | 0.059712315 |
| OR13C2 | ENST00000602597.1 | ENSG00000269947.1 | -0.923277331 | -2.053013923 | 0.040071227 |
| OR13C2 | ENST00000606942.1 | ENSG00000271835.1 | 0.83188766 | 1.851413744 | 0.064110053 |
| OR13C2 | ENST00000607600.1 | ENSG00000272114.1 | -0.952717303 | -2.088342645 | 0.036766937 |

| | | | | | |
|---|---|---|---|---|---|
| OR13C2 | NR_026962.1 | TTC28-AS1 | -0.905863119 | -2.025124151 | 0.042854618 |
| OR13C2 | NR_103445.2 | RBFADN | -0.890296687 | -1.996216378 | 0.045910376 |
| OR13C2 | NR_110702.1 | SEMA3B-AS1 | -0.8842519 | -1.987412582 | 0.046876694 |
| OR13C2 | NR_110846.1 | LOC101928674 | -0.979428075 | -2.207065255 | 0.027309501 |
| OR13C2 | NR_125875.1 | TBX18-AS1 | 0.845486602 | 1.881760669 | 0.059868518 |
| OR13C2 | NR_125876.1 | TBX18-AS1 | 0.811365833 | 1.816453615 | 0.069300817 |
| OR13C2 | NR_133907.1 | HLA-DQB1-AS1 | 0.905298756 | 2.054568288 | 0.039920721 |
| OR13C2 | NR_134576.1 | LOC105372672 | -0.928870723 | -2.065338706 | 0.038890964 |
| OR13C2 | NR_134910.1 | LOC102725254 | 0.826672449 | 1.864005869 | 0.06232088 |
| OR13C2 | NR_135251.1 | LOC101928143 | -0.945012687 | -2.124541627 | 0.033624879 |
| OR13C5 | ENST00000381106.4 | ENSG00000205663.5 | 0.864397938 | 1.91257086 | 0.055803013 |
| OR13C5 | ENST00000381475.3 | ENSG00000215863.2 | 0.943259899 | 2.105770969 | 0.035224252 |
| OR13C5 | ENST00000412809.1 | ENSG00000229938.1 | 0.855416904 | 1.925286657 | 0.054193506 |
| OR13C5 | ENST00000417654.1 | ENSG00000224893.1 | -0.864858365 | -1.931928768 | 0.053368299 |
| OR13C5 | ENST00000420845.1 | ENSG00000232259.1 | 0.842539023 | 1.906349475 | 0.056604879 |
| OR13C5 | ENST00000423796.1 | ENSG00000235146.2 | 0.816228522 | 1.823226785 | 0.06826905 |
| OR13C5 | ENST00000430751.1 | ENSG00000232222.1 | -0.926730729 | -2.067368103 | 0.038699481 |
| OR13C5 | ENST00000437308.1 | ENSG00000233154.1 | -0.82215708 | -1.844430723 | 0.065120393 |
| OR13C5 | ENST00000437859.2 | ENSG00000235122.3 | 0.894551369 | 1.996764301 | 0.045850793 |
| OR13C5 | ENST00000439072.1 | ENSG00000224516.1 | -0.833132467 | -1.877884896 | 0.060396913 |
| OR13C5 | ENST00000443123.1 | ENSG00000229457.1 | 0.911562555 | 2.04449997 | 0.04090418 |
| OR13C5 | ENST00000450227.1 | ENSG00000229941.1 | 0.866778142 | 1.940493388 | 0.052319757 |
| OR13C5 | ENST00000450696.1 | ENSG00000235146.2 | 0.870606008 | 1.945871643 | 0.05167016 |
| OR13C5 | ENST00000457998.2 | ENSG00000233006.2 | -0.821366267 | -1.843593212 | 0.065242446 |
| OR13C5 | ENST00000510941.1 | ENSG00000251339.1 | 0.872922026 | 1.967375231 | 0.049139968 |
| OR13C5 | ENST00000512563.1 | ENSG00000249547.1 | 0.854002186 | 1.912476195 | 0.055815143 |
| OR13C5 | ENST00000518339.1 | ENSG00000253470.1 | 0.838737573 | 1.83913403 | 0.065895475 |
| OR13C5 | ENST00000519412.1 | ENSG00000253214.1 | -0.933768827 | -2.098359348 | 0.035873413 |
| OR13C5 | ENST00000536492.1 | ENSG00000256237.1 | -0.889108449 | -1.982768226 | 0.047393325 |
| OR13C5 | ENST00000551174.1 | ENSG00000257762.1 | -0.815392503 | -1.828519115 | 0.06747168 |
| OR13C5 | ENST00000562834.1 | ENSG00000261116.1 | 0.821733926 | 1.862419155 | 0.062544031 |
| OR13C5 | ENST00000567067.1 | ENSG00000261600.1 | 0.927702402 | 2.068946922 | 0.038551068 |
| OR13C5 | ENST00000570919.1 | ENSG00000263321.1 | 0.810020944 | 1.81350014 | 0.069754717 |
| OR13C5 | ENST00000589983.1 | ENSG00000267057.1 | 0.87113083 | 1.951634431 | 0.050981622 |
| OR13C5 | ENST00000592045.1 | ENSG00000267057.1 | 0.841765284 | 1.896280883 | 0.057922913 |
| OR13C5 | ENST00000602620.1 | ENSG00000215386.6 | 0.904738842 | 2.026180248 | 0.042746317 |
| OR13C5 | ENST00000603533.1 | ENSG00000271384.1 | 0.905152375 | 2.026469809 | 0.042716664 |
| OR13C5 | ENST00000608934.1 | ENSG00000273063.1 | 0.810930759 | 1.826324096 | 0.067801458 |
| OR13C5 | ENST00000609924.1 | ENSG00000272688.1 | -0.968449879 | -2.155719825 | 0.031105549 |
| OR13C5 | NR_110275.1 | LOC101927795 | -0.866409877 | -1.961311177 | 0.049842734 |
| OR13C5 | NR_125839.1 | LOC101927020 | 0.955817428 | 2.124939319 | 0.033591677 |
| OR13C5 | NR_134573.1 | GS1-124K5.4 | 0.83889537 | 1.892486584 | 0.058426179 |
| OR13C5 | NR_135679.1 | LOC105370829 | 0.862473777 | 1.92103826 | 0.054726884 |
| OR2T4 | ENST00000399186.2 | ENSG00000214888.2 | 0.981058821 | 2.191396777 | 0.028423092 |
| OR2T4 | ENST00000413650.1 | ENSG00000230880.2 | 0.937266204 | 2.085135712 | 0.037056983 |
| OR2T4 | ENST00000413991.1 | ENSG00000237614.1 | 0.887022453 | 1.996299816 | 0.045901298 |
| OR2T4 | ENST00000416641.1 | ENSG00000226956.1 | -0.90214417 | -2.035309745 | 0.041819716 |
| OR2T4 | ENST00000416657.1 | ENSG00000235858.1 | 0.879916938 | 1.995511107 | 0.045987164 |
| OR2T4 | ENST00000417426.1 | ENSG00000233145.1 | 0.877778059 | 1.953512885 | 0.050758852 |
| OR2T4 | ENST00000418972.1 | ENSG00000225044.1 | -0.845306707 | -1.87763224 | 0.060431492 |
| OR2T4 | ENST00000420315.1 | ENSG00000228072.1 | 0.81328322 | 1.8202993 | 0.068713438 |

| | | | | | |
|---|---|---|---|---|---|
| OR2T4 | ENST00000420365.1 | ENSG00000225214.1 | 0.860439179 | 1.906076199 | 0.05664032 |
| OR2T4 | ENST00000420465.1 | ENSG00000167355.3 | 0.929487891 | 2.070357095 | 0.038418917 |
| OR2T4 | ENST00000420766.1 | ENSG00000228679.1 | 0.809665678 | 1.815003436 | 0.069523381 |
| OR2T4 | ENST00000421020.1 | ENSG00000231407.1 | 0.908630979 | 2.031155729 | 0.0422392 |
| OR2T4 | ENST00000423869.1 | ENSG00000227848.1 | 0.822916316 | 1.834417898 | 0.066591985 |
| OR2T4 | ENST00000424181.1 | ENSG00000224977.1 | 0.957328048 | 2.140601886 | 0.032306157 |
| OR2T4 | ENST00000425371.2 | ENSG00000235872.2 | 0.837652184 | 1.847293265 | 0.064704649 |
| OR2T4 | ENST00000426504.1 | ENSG00000234190.1 | -0.849568562 | -1.868493432 | 0.061693325 |
| OR2T4 | ENST00000426519.1 | ENSG00000234142.1 | 0.823402593 | 1.829482036 | 0.067327428 |
| OR2T4 | ENST00000427064.1 | ENSG00000238031.1 | 0.825470235 | 1.841519205 | 0.065545509 |
| OR2T4 | ENST00000429630.1 | ENSG00000232533.1 | 0.809766071 | 1.805933573 | 0.070928716 |
| OR2T4 | ENST00000433344.1 | ENSG00000234083.1 | -0.838409198 | -1.874298404 | 0.060889307 |
| OR2T4 | ENST00000433614.1 | ENSG00000228534.1 | -0.896592208 | -1.998961429 | 0.045612527 |
| OR2T4 | ENST00000435434.1 | ENSG00000231233.1 | 0.845102845 | 1.893599125 | 0.05827824 |
| OR2T4 | ENST00000435733.1 | ENSG00000226377.1 | 0.894464609 | 1.995255877 | 0.04601498 |
| OR2T4 | ENST00000435992.2 | ENSG00000232675.3 | 0.933839424 | 2.090811998 | 0.03654492 |
| OR2T4 | ENST00000436582.1 | ENSG00000236525.1 | -0.827735221 | -1.863247199 | 0.062427495 |
| OR2T4 | ENST00000437330.1 | ENSG00000229203.1 | 0.802222771 | 1.803842503 | 0.071256001 |
| OR2T4 | ENST00000438107.1 | ENSG00000234449.2 | 0.840707347 | 1.877963242 | 0.060386194 |
| OR2T4 | ENST00000438222.1 | ENSG00000238034.1 | 0.974514694 | 2.183976287 | 0.028963991 |
| OR2T4 | ENST00000439186.1 | ENSG00000237076.1 | 0.818869715 | 1.857411305 | 0.063252659 |
| OR2T4 | ENST00000442069.1 | ENSG00000225655.1 | -0.939938261 | -2.106742994 | 0.035139863 |
| OR2T4 | ENST00000442831.1 | ENSG00000229550.1 | -0.927322882 | -2.084433062 | 0.037120793 |
| OR2T4 | ENST00000443306.1 | ENSG00000233891.3 | 0.944480678 | 2.119195116 | 0.034073981 |
| OR2T4 | ENST00000447183.2 | ENSG00000271593.1 | 0.893024198 | 1.987960508 | 0.046816058 |
| OR2T4 | ENST00000447514.1 | ENSG00000236753.1 | 0.93253979 | 2.092580031 | 0.036386661 |
| OR2T4 | ENST00000447709.1 | ENSG00000237473.1 | 0.97995756 | 2.190331938 | 0.028500172 |
| OR2T4 | ENST00000450063.1 | ENSG00000231210.2 | -0.840830861 | -1.886019793 | 0.059292287 |
| OR2T4 | ENST00000450109.1 | ENSG00000225376.1 | 0.82511301 | 1.830382585 | 0.06719275 |
| OR2T4 | ENST00000450848.1 | ENSG00000225539.1 | 0.840700584 | 1.891361494 | 0.058576105 |
| OR2T4 | ENST00000451656.1 | ENSG00000228417.1 | 0.884465489 | 2.002751702 | 0.045203946 |
| OR2T4 | ENST00000453579.1 | ENSG00000232529.1 | 0.898047528 | 2.005368425 | 0.044923675 |
| OR2T4 | ENST00000454489.1 | ENSG00000231403.1 | 0.927178065 | 2.061526789 | 0.03925281 |
| OR2T4 | ENST00000454526.1 | ENSG00000234136.1 | 0.931912362 | 2.089179577 | 0.036691561 |
| OR2T4 | ENST00000455699.1 | ENSG00000240996.1 | 0.807122549 | 1.822179933 | 0.068427688 |
| OR2T4 | ENST00000457115.1 | ENSG00000227245.1 | 0.893249475 | 2.002179518 | 0.045265428 |
| OR2T4 | ENST00000457848.1 | ENSG00000226412.1 | 0.87519267 | 1.93501463 | 0.0529885 |
| OR2T4 | ENST00000458154.1 | ENSG00000235578.1 | 0.808268555 | 1.833360216 | 0.06674902 |
| OR2T4 | ENST00000458364.1 | ENSG00000225655.1 | -0.93375562 | -2.087275833 | 0.036863208 |
| OR2T4 | ENST00000469931.2 | ENSG00000272030.1 | 0.895428685 | 1.979749121 | 0.047731726 |
| OR2T4 | ENST00000472596.1 | ENSG00000239774.1 | 0.929569743 | 2.06939256 | 0.038509264 |
| OR2T4 | ENST00000476099.1 | ENSG00000244158.1 | -0.833559598 | -1.853924568 | 0.063749952 |
| OR2T4 | ENST00000476892.1 | ENSG00000241345.1 | 0.851879924 | 1.910192106 | 0.056108483 |
| OR2T4 | ENST00000484413.1 | ENSG00000271853.1 | 0.930298575 | 2.072029361 | 0.038262703 |
| OR2T4 | ENST00000493123.1 | ENSG00000242428.1 | 0.975885499 | 2.159536958 | 0.030808533 |
| OR2T4 | ENST00000494509.1 | ENSG00000240095.1 | 0.822626564 | 1.85309372 | 0.063868927 |
| OR2T4 | ENST00000501405.2 | ENSG00000247402.2 | -0.887919172 | -1.999143565 | 0.045592823 |
| OR2T4 | ENST00000502421.1 | ENSG00000250284.1 | -0.825295775 | -1.850595068 | 0.06422783 |
| OR2T4 | ENST00000504755.1 | ENSG00000250252.1 | 0.868685894 | 1.941172748 | 0.052237327 |
| OR2T4 | ENST00000504891.1 | ENSG00000249388.1 | 0.868550239 | 1.959296852 | 0.050078032 |
| OR2T4 | ENST00000505196.1 | ENSG00000248131.1 | 0.848231475 | 1.892244807 | 0.058458371 |

| | | | | | |
|---|---|---|---|---|---|
| OR2T4 | ENST00000505498.1 | ENSG00000250908.1 | 0.871082197 | 1.941504682 | 0.052197092 |
| OR2T4 | ENST00000505556.1 | ENSG00000249409.1 | 0.849579701 | 1.898706713 | 0.057603048 |
| OR2T4 | ENST00000506100.1 | ENSG00000249409.1 | 0.838723701 | 1.87846226 | 0.060317955 |
| OR2T4 | ENST00000506305.1 | ENSG00000249994.1 | -0.835883358 | -1.894849619 | 0.058112327 |
| OR2T4 | ENST00000508083.1 | ENSG00000249343.1 | 0.817787832 | 1.827265887 | 0.067659802 |
| OR2T4 | ENST00000508188.1 | ENSG00000250999.1 | 0.820014736 | 1.832665693 | 0.066852302 |
| OR2T4 | ENST00000515750.1 | ENSG00000249061.1 | -0.910104684 | -2.046078082 | 0.040748689 |
| OR2T4 | ENST00000517300.1 | ENSG00000254144.2 | 0.886587147 | 1.961476981 | 0.049823408 |
| OR2T4 | ENST00000517716.1 | ENSG00000253515.1 | -0.894638615 | -2.003980097 | 0.045072192 |
| OR2T4 | ENST00000519451.1 | ENSG00000253363.1 | 0.841030465 | 1.879557031 | 0.060168474 |
| OR2T4 | ENST00000519852.1 | ENSG00000253716.1 | 0.80596066 | 1.795515296 | 0.072571638 |
| OR2T4 | ENST00000520749.1 | ENSG00000253717.1 | -0.828262428 | -1.87350148 | 0.060999169 |
| OR2T4 | ENST00000521307.1 | ENSG00000253177.1 | 0.90077003 | 2.033482861 | 0.042003763 |
| OR2T4 | ENST00000522704.1 | ENSG00000254135.1 | 0.875458114 | 1.970237633 | 0.048811142 |
| OR2T4 | ENST00000524073.1 | ENSG00000253774.1 | 0.902928483 | 2.02223293 | 0.043152294 |
| OR2T4 | ENST00000524335.1 | ENSG00000253716.1 | 0.823243363 | 1.834300609 | 0.066609384 |
| OR2T4 | ENST00000524818.1 | ENSG00000254473.1 | 0.866495841 | 1.941940609 | 0.052144291 |
| OR2T4 | ENST00000525133.1 | ENSG00000255375.1 | 0.845164649 | 1.891607047 | 0.058543356 |
| OR2T4 | ENST00000526186.1 | ENSG00000254510.1 | 0.82992183 | 1.808081967 | 0.070593744 |
| OR2T4 | ENST00000526611.1 | ENSG00000246982.2 | 0.815393623 | 1.84620958 | 0.06486178 |
| OR2T4 | ENST00000526694.1 | ENSG00000231999.2 | 0.92993335 | 2.082892996 | 0.037260978 |
| OR2T4 | ENST00000526935.1 | ENSG00000255372.1 | 0.949619328 | 2.122549965 | 0.033791582 |
| OR2T4 | ENST00000531627.1 | ENSG00000254584.1 | 0.90541687 | 2.039094919 | 0.041440555 |
| OR2T4 | ENST00000531661.1 | ENSG00000254473.1 | 0.90030664 | 2.030247814 | 0.042331356 |
| OR2T4 | ENST00000532688.1 | ENSG00000255441.1 | 0.844205729 | 1.862270604 | 0.062564957 |
| OR2T4 | ENST00000535720.1 | ENSG00000256364.1 | 0.845157204 | 1.902193789 | 0.057145825 |
| OR2T4 | ENST00000536141.1 | ENSG00000256969.1 | 0.914893617 | 2.042967087 | 0.041055696 |
| OR2T4 | ENST00000537269.1 | ENSG00000257084.1 | 0.903126829 | 2.026875015 | 0.042675196 |
| OR2T4 | ENST00000537921.1 | ENSG00000255966.1 | 0.89989669 | 2.004134198 | 0.045055687 |
| OR2T4 | ENST00000544089.1 | ENSG00000256273.1 | 0.945753318 | 2.136233053 | 0.032660423 |
| OR2T4 | ENST00000545642.1 | ENSG00000256342.1 | 0.880189983 | 1.969224123 | 0.04892736 |
| OR2T4 | ENST00000547175.1 | ENSG00000257395.1 | -0.920716388 | -2.039548585 | 0.041395307 |
| OR2T4 | ENST00000547750.1 | ENSG00000257886.1 | 0.834883561 | 1.869115021 | 0.061606814 |
| OR2T4 | ENST00000548722.2 | ENSG00000257194.2 | -0.854773048 | -1.898863173 | 0.057582469 |
| OR2T4 | ENST00000549140.1 | ENSG00000258332.1 | 0.928914086 | 2.096437993 | 0.036043355 |
| OR2T4 | ENST00000549878.1 | ENSG00000257284.1 | 0.888239399 | 1.968452486 | 0.049015998 |
| OR2T4 | ENST00000557903.1 | ENSG00000259182.1 | 0.865248606 | 1.915585415 | 0.055417888 |
| OR2T4 | ENST00000558141.1 | ENSG00000259594.1 | 0.813503063 | 1.827320452 | 0.067651602 |
| OR2T4 | ENST00000558312.1 | ENSG00000259176.1 | 0.814745704 | 1.811757696 | 0.070023644 |
| OR2T4 | ENST00000559569.1 | ENSG00000259760.1 | -0.943977098 | -2.084109301 | 0.037150226 |
| OR2T4 | ENST00000560522.1 | ENSG00000259661.1 | 0.954149407 | 2.172603307 | 0.029810187 |
| OR2T4 | ENST00000561567.1 | ENSG00000260177.1 | 0.8506621 | 1.916825561 | 0.055260096 |
| OR2T4 | ENST00000563044.1 | ENSG00000260978.1 | 0.938341397 | 2.096226835 | 0.036062074 |
| OR2T4 | ENST00000565965.1 | ENSG00000261172.1 | 0.836594992 | 1.873671346 | 0.060975738 |
| OR2T4 | ENST00000567089.1 | ENSG00000261822.1 | 0.855246716 | 1.918220797 | 0.05508302 |
| OR2T4 | ENST00000569981.1 | ENSG00000238045.5 | 0.806569638 | 1.770974602 | 0.076564925 |
| OR2T4 | ENST00000570512.1 | ENSG00000262768.1 | 0.933838131 | 2.093720383 | 0.036284896 |
| OR2T4 | ENST00000571815.1 | ENSG00000262810.1 | 0.843181602 | 1.883893038 | 0.059579445 |
| OR2T4 | ENST00000574365.1 | ENSG00000262837.1 | 0.868406379 | 1.959475757 | 0.050057096 |
| OR2T4 | ENST00000575139.1 | ENSG00000263072.1 | 0.830022712 | 1.87461106 | 0.060846251 |
| OR2T4 | ENST00000577678.1 | ENSG00000265415.1 | 0.901347499 | 2.021432109 | 0.043235054 |

| | | | | | |
|---|---|---|---|---|---|
| OR2T4 | ENST00000577698.1 | ENSG00000265100.1 | 0.860736315 | 1.901262242 | 0.057267672 |
| OR2T4 | ENST00000577807.1 | ENSG00000263427.1 | 0.868720047 | 1.947942385 | 0.051421857 |
| OR2T4 | ENST00000578265.1 | ENSG00000214719.7 | 0.952315094 | 2.164126683 | 0.030454628 |
| OR2T4 | ENST00000578757.1 | ENSG00000175061.13 | 0.991702324 | 2.215899222 | 0.026698405 |
| OR2T4 | ENST00000581362.1 | ENSG00000235300.3 | 0.846078059 | 1.89392113 | 0.058235479 |
| OR2T4 | ENST00000581940.1 | ENSG00000265484.1 | 0.923605514 | 2.073682876 | 0.038108773 |
| OR2T4 | ENST00000582386.1 | ENSG00000265174.1 | 0.883515578 | 1.976068596 | 0.048147009 |
| OR2T4 | ENST00000584758.1 | ENSG00000265356.1 | 0.928639329 | 2.085896886 | 0.036987964 |
| OR2T4 | ENST00000586010.1 | ENSG00000267606.1 | 0.906365606 | 2.025119085 | 0.042855138 |
| OR2T4 | ENST00000588835.1 | ENSG00000267476.1 | 0.904747846 | 2.025322652 | 0.042834244 |
| OR2T4 | ENST00000589233.1 | ENSG00000231616.4 | 0.81482622 | 1.834877612 | 0.066523826 |
| OR2T4 | ENST00000589817.1 | ENSG00000231616.4 | 0.810749989 | 1.835161164 | 0.066481813 |
| OR2T4 | ENST00000591103.1 | ENSG00000272895.1 | 0.865599724 | 1.959345628 | 0.050072324 |
| OR2T4 | ENST00000591137.1 | ENSG00000267405.1 | 0.845881308 | 1.918678749 | 0.055025003 |
| OR2T4 | ENST00000593632.1 | ENSG00000180279.5 | 0.942281092 | 2.102881254 | 0.035476151 |
| OR2T4 | ENST00000595892.1 | ENSG00000269640.1 | 0.866469055 | 1.936765926 | 0.052773963 |
| OR2T4 | ENST00000596135.1 | ENSG00000269843.1 | 0.929002434 | 2.09856804 | 0.035854996 |
| OR2T4 | ENST00000596643.1 | ENSG00000269439.1 | 0.814856978 | 1.827689575 | 0.067596154 |
| OR2T4 | ENST00000597309.1 | ENSG00000232098.2 | -0.860531279 | -1.925680342 | 0.0541443 |
| OR2T4 | ENST00000599259.1 | ENSG00000269352.1 | 0.870931949 | 1.938665451 | 0.052542088 |
| OR2T4 | ENST00000599467.1 | ENSG00000244513.2 | 0.877431156 | 1.957855162 | 0.050247011 |
| OR2T4 | ENST00000600489.1 | ENSG00000231898.4 | 0.844955384 | 1.902757345 | 0.057072215 |
| OR2T4 | ENST00000600534.1 | ENSG00000267858.1 | 0.818279949 | 1.851237989 | 0.064135323 |
| OR2T4 | ENST00000600889.1 | ENSG00000232675.3 | 0.868789698 | 1.935666854 | 0.052908517 |
| OR2T4 | ENST00000601511.1 | ENSG00000244513.2 | 0.812814932 | 1.808918553 | 0.070463658 |
| OR2T4 | ENST00000601735.1 | ENSG00000244513.2 | 0.840525493 | 1.880428119 | 0.060049754 |
| OR2T4 | ENST00000602485.1 | ENSG00000270163.1 | -0.859782644 | -1.936141313 | 0.052850396 |
| OR2T4 | ENST00000602809.1 | ENSG00000270105.1 | -0.937861061 | -2.104860188 | 0.03530348 |
| OR2T4 | ENST00000602872.1 | ENSG00000270067.1 | 0.849133105 | 1.902977679 | 0.057043458 |
| OR2T4 | ENST00000602900.1 | ENSG00000270179.1 | -0.809964708 | -1.802097194 | 0.071530115 |
| OR2T4 | ENST00000606470.1 | ENSG00000271913.1 | 0.817104384 | 1.805276926 | 0.071031359 |
| OR2T4 | ENST00000606841.1 | ENSG00000272411.1 | 0.942062225 | 2.102589628 | 0.035501657 |
| OR2T4 | ENST00000607201.1 | ENSG00000272024.1 | -0.829681483 | -1.868895562 | 0.061637346 |
| OR2T4 | ENST00000607222.1 | ENSG00000272106.1 | 0.859530239 | 1.94675415 | 0.051564216 |
| OR2T4 | ENST00000607715.1 | ENSG00000271788.1 | 0.801832678 | 1.796523805 | 0.07241125 |
| OR2T4 | ENST00000607876.1 | ENSG00000272848.1 | 0.908882774 | 2.007762715 | 0.044668513 |
| OR2T4 | ENST00000608085.1 | ENSG00000231898.4 | 0.829508955 | 1.854767862 | 0.063629383 |
| OR2T4 | ENST00000608173.1 | ENSG00000197099.4 | 0.880564839 | 1.950490194 | 0.051117721 |
| OR2T4 | ENST00000608476.1 | ENSG00000232675.3 | 0.841001193 | 1.858539002 | 0.06309251 |
| OR2T4 | ENST00000608856.1 | ENSG00000272600.1 | -0.84322438 | -1.875078715 | 0.060781895 |
| OR2T4 | ENST00000609067.1 | ENSG00000272849.1 | 0.856734324 | 1.925406165 | 0.054178565 |
| OR2T4 | ENST00000609610.1 | ENSG00000232675.3 | 0.94167457 | 2.069852132 | 0.038466194 |
| OR2T4 | ENST00000609725.1 | ENSG00000231898.4 | 0.876262168 | 1.939278511 | 0.052467434 |
| OR2T4 | ENST00000609890.1 | ENSG00000231898.4 | 0.850202306 | 1.883029219 | 0.059696408 |
| OR2T4 | ENST00000609972.1 | ENSG00000230651.3 | 0.802162955 | 1.788996398 | 0.073615396 |
| OR2T4 | ENST00000610145.1 | ENSG00000273175.1 | 0.8813253 | 1.945151882 | 0.051756701 |
| OR2T4 | ENST00000610185.1 | ENSG00000273355.1 | -0.888759595 | -1.977673632 | 0.047965537 |
| OR2T4 | NR_022011.1 | PWARSN | 0.830624219 | 1.862075162 | 0.062592497 |
| OR2T4 | NR_027334.2 | MZF1-AS1 | 0.8206566 | 1.835586801 | 0.066418791 |
| OR2T4 | NR_044996.1 | HCG23 | 0.801605288 | 1.788594644 | 0.073680121 |
| OR2T4 | NR_045637.1 | BOLA3-AS1 | 0.83546387 | 1.896942733 | 0.057835497 |

| | | | | | |
|---|---|---|---|---|---|
| OR2T4 | NR_046742.2 | ZNF630-AS1 | -0.895903915 | -2.013242692 | 0.044089104 |
| OR2T4 | NR_046839.1 | AGBL4-IT1 | -0.880663453 | -1.98022792 | 0.047677924 |
| OR2T4 | NR_047040.1 | LINC00424 | 0.815204553 | 1.840093321 | 0.065754538 |
| OR2T4 | NR_103790.1 | LINC00581 | -0.876749571 | -1.954611605 | 0.05062893 |
| OR2T4 | NR_104620.1 | LINC01672 | 0.827287313 | 1.839447931 | 0.06584933 |
| OR2T4 | NR_104998.1 | LOC102467225 | 0.817891185 | 1.808662664 | 0.070503427 |
| OR2T4 | NR_108036.1 | CFAP58-AS1 | 0.826236689 | 1.833041921 | 0.066796337 |
| OR2T4 | NR_110053.1 | LOC101927464 | 0.880189983 | 1.965649023 | 0.049339167 |
| OR2T4 | NR_110480.1 | LOC101927079 | 0.815017367 | 1.815997789 | 0.069370711 |
| OR2T4 | NR_110481.1 | LOC101927079 | 0.814745704 | 1.828929011 | 0.067410243 |
| OR2T4 | NR_120330.1 | LOC101928227 | -0.878945209 | -1.952144304 | 0.050921074 |
| OR2T4 | NR_120527.1 | LOC100506675 | 0.862685438 | 1.906465911 | 0.056589784 |
| OR2T4 | NR_121188.1 | PGM5P3-AS1 | -0.880388762 | -1.973319248 | 0.048459201 |
| OR2T4 | NR_121189.1 | PGM5P3-AS1 | -0.90726461 | -2.022018875 | 0.043174402 |
| OR2T4 | NR_134597.1 | LOC105378068 | 0.810769571 | 1.82976391 | 0.06728525 |
| OR2T4 | NR_135032.1 | LOC105369635 | 0.903126829 | 2.00114934 | 0.045376299 |
| OR2T4 | NR_135274.1 | LOC105370619 | 0.865040816 | 1.92435264 | 0.054310397 |
| OR2T4 | NR_135840.1 | LOC105376114 | 0.873005061 | 1.950649915 | 0.051098705 |
| OR2T4 | NR_138084.1 | HCG24 | 0.878015211 | 1.964508221 | 0.049471184 |
| OR4C11 | ENST00000412445.1 | ENSG00000238042.1 | -0.922636906 | -2.073305429 | 0.038143864 |
| OR4C11 | ENST00000412812.1 | ENSG00000225342.1 | 0.90127134 | 2.029199425 | 0.042437982 |
| OR4C11 | ENST00000416401.1 | ENSG00000237756.1 | -0.911986937 | -2.050215224 | 0.040343433 |
| OR4C11 | ENST00000421617.1 | ENSG00000237342.1 | 0.828516482 | 1.855101964 | 0.063581667 |
| OR4C11 | ENST00000424241.1 | ENSG00000237311.1 | -0.835640675 | -1.862222561 | 0.062571726 |
| OR4C11 | ENST00000425364.1 | ENSG00000231046.1 | -0.893197817 | -1.99365759 | 0.046189487 |
| OR4C11 | ENST00000429796.1 | ENSG00000231858.1 | -0.830596392 | -1.866098205 | 0.062027627 |
| OR4C11 | ENST00000430545.1 | ENSG00000237153.1 | 0.859995032 | 1.904529352 | 0.056841278 |
| OR4C11 | ENST00000432244.1 | ENSG00000234265.1 | 0.817398436 | 1.827446094 | 0.067632724 |
| OR4C11 | ENST00000432559.2 | ENSG00000228229.2 | 0.901508525 | 2.025368655 | 0.042829524 |
| OR4C11 | ENST00000432711.1 | ENSG00000234919.1 | -0.800993702 | -1.812233804 | 0.069950078 |
| OR4C11 | ENST00000433249.1 | ENSG00000236556.1 | 0.848173788 | 1.908460767 | 0.056331689 |
| OR4C11 | ENST00000433876.2 | ENSG00000228423.2 | 0.882376595 | 1.992718411 | 0.04629229 |
| OR4C11 | ENST00000437864.1 | ENSG00000231521.1 | 0.863810075 | 1.937863961 | 0.052639822 |
| OR4C11 | ENST00000438488.1 | ENSG00000223812.1 | 0.988156174 | 2.185116023 | 0.028880341 |
| OR4C11 | ENST00000440714.1 | ENSG00000237609.1 | 0.854352299 | 1.903664365 | 0.05695391 |
| OR4C11 | ENST00000441295.1 | ENSG00000233960.1 | -0.875418097 | -1.984328494 | 0.047219232 |
| OR4C11 | ENST00000441532.1 | ENSG00000234206.1 | -0.83041225 | -1.886527189 | 0.059223948 |
| OR4C11 | ENST00000441666.1 | ENSG00000230379.1 | 0.842421718 | 1.860637978 | 0.062795318 |
| OR4C11 | ENST00000441875.1 | ENSG00000239203.1 | 0.818337788 | 1.859233432 | 0.062994058 |
| OR4C11 | ENST00000441991.1 | ENSG00000231210.2 | -0.828957896 | -1.874405133 | 0.060874607 |
| OR4C11 | ENST00000442850.1 | ENSG00000232600.2 | -0.812717872 | -1.836915718 | 0.066222338 |
| OR4C11 | ENST00000449463.1 | ENSG00000230309.1 | -0.867691287 | -1.943924554 | 0.05190455 |
| OR4C11 | ENST00000456715.1 | ENSG00000224893.1 | 0.830521585 | 1.856200793 | 0.063424943 |
| OR4C11 | ENST00000456999.1 | ENSG00000230690.1 | 0.829298799 | 1.852220258 | 0.063994201 |
| OR4C11 | ENST00000457602.1 | ENSG00000237576.1 | -0.864365189 | -1.954455914 | 0.050647324 |
| OR4C11 | ENST00000458443.1 | ENSG00000238232.1 | 0.87981749 | 1.94717671 | 0.051513553 |
| OR4C11 | ENST00000474656.1 | ENSG00000244198.1 | 0.886607421 | 1.963866764 | 0.049545545 |
| OR4C11 | ENST00000503034.1 | ENSG00000248936.1 | 0.894946334 | 2.003326189 | 0.045142288 |
| OR4C11 | ENST00000508004.2 | ENSG00000251661.3 | 0.933091523 | 2.071290004 | 0.038331703 |
| OR4C11 | ENST00000514411.1 | ENSG00000250882.1 | 0.944037167 | 2.121647184 | 0.033867377 |
| OR4C11 | ENST00000514877.1 | ENSG00000248685.1 | 0.860536856 | 1.913782029 | 0.055648013 |

| | | | | | |
|---|---|---|---|---|---|
| OR4C11 | ENST00000515504.1 | ENSG00000249588.1 | -0.906706099 | -2.043549306 | 0.040998092 |
| OR4C11 | ENST00000518260.1 | ENSG00000253628.1 | -0.835759339 | -1.845029679 | 0.065033221 |
| OR4C11 | ENST00000518894.1 | ENSG00000204758.3 | 0.890174948 | 1.984314916 | 0.047220744 |
| OR4C11 | ENST00000519368.1 | ENSG00000253215.1 | 0.813590711 | 1.827086975 | 0.067686693 |
| OR4C11 | ENST00000520838.1 | ENSG00000253404.1 | 0.857788178 | 1.931946281 | 0.053366137 |
| OR4C11 | ENST00000522547.1 | ENSG00000253430.1 | -0.863442967 | -1.906419501 | 0.056595801 |
| OR4C11 | ENST00000524094.1 | ENSG00000248555.2 | -0.890632986 | -1.97847953 | 0.047874636 |
| OR4C11 | ENST00000528607.1 | ENSG00000254604.1 | 0.832810548 | 1.869805633 | 0.061510814 |
| OR4C11 | ENST00000528818.1 | ENSG00000232995.3 | 0.937533754 | 2.070263655 | 0.038427661 |
| OR4C11 | ENST00000531136.1 | ENSG00000255558.1 | -0.933912474 | -2.086732191 | 0.03691235 |
| OR4C11 | ENST00000540739.1 | ENSG00000249196.2 | -0.835493823 | -1.856499093 | 0.063382452 |
| OR4C11 | ENST00000547547.1 | ENSG00000257241.1 | -0.806703249 | -1.758252312 | 0.078704588 |
| OR4C11 | ENST00000550138.1 | ENSG00000257467.1 | 0.897853821 | 2.000614732 | 0.045433925 |
| OR4C11 | ENST00000551361.1 | ENSG00000224078.8 | -0.816567829 | -1.839122307 | 0.065897199 |
| OR4C11 | ENST00000554798.1 | ENSG00000258483.1 | 0.919634128 | 2.043884599 | 0.040964949 |
| OR4C11 | ENST00000560134.1 | ENSG00000259176.1 | 0.85938785 | 1.932197482 | 0.053335136 |
| OR4C11 | ENST00000563449.2 | ENSG00000261613.2 | 0.864306446 | 1.915172092 | 0.05547056 |
| OR4C11 | ENST00000570158.1 | ENSG00000260937.1 | -0.827470623 | -1.833903486 | 0.066668322 |
| OR4C11 | ENST00000573414.1 | ENSG00000263072.1 | 0.854076744 | 1.897279083 | 0.057791114 |
| OR4C11 | ENST00000574460.1 | ENSG00000263051.1 | 0.849168383 | 1.939696425 | 0.052416594 |
| OR4C11 | ENST00000581905.1 | ENSG00000264235.1 | 0.829228872 | 1.854648926 | 0.063646377 |
| OR4C11 | ENST00000585072.1 | ENSG00000263745.1 | 0.851534136 | 1.915154666 | 0.055472782 |
| OR4C11 | ENST00000586051.1 | ENSG00000267576.1 | 0.872979308 | 1.9308807 | 0.053497807 |
| OR4C11 | ENST00000587281.1 | ENSG00000228290.2 | 0.879828619 | 1.980270832 | 0.047673104 |
| OR4C11 | ENST00000595508.1 | ENSG00000269749.1 | 0.930107899 | 2.087410675 | 0.036851028 |
| OR4C11 | ENST00000597865.1 | ENSG00000268108.1 | 0.858318355 | 1.946552916 | 0.051588358 |
| OR4C11 | ENST00000598131.1 | ENSG00000269043.1 | 0.898617397 | 1.995842568 | 0.045951062 |
| OR4C11 | ENST00000598220.1 | ENSG00000268777.1 | 0.807526372 | 1.817151758 | 0.069193879 |
| OR4C11 | ENST00000599352.1 | ENSG00000240401.4 | -0.865211771 | -1.92393782 | 0.054362378 |
| OR4C11 | ENST00000601752.1 | ENSG00000268051.1 | 0.948430559 | 2.120047395 | 0.034002049 |
| OR4C11 | ENST00000606374.1 | ENSG00000272312.1 | -0.843482379 | -1.884245678 | 0.059531751 |
| OR4C11 | ENST00000606778.1 | ENSG00000271930.1 | 0.82158099 | 1.816810817 | 0.069246086 |
| OR4C11 | ENST00000607052.1 | ENSG00000271870.1 | -0.838708648 | -1.883790664 | 0.059593297 |
| OR4C11 | ENST00000607224.1 | ENSG00000272521.1 | 0.808961572 | 1.809460661 | 0.070379466 |
| OR4C11 | ENST00000607600.1 | ENSG00000272114.1 | -0.824650872 | -1.868060124 | 0.061753691 |
| OR4C11 | ENST00000609281.1 | ENSG00000273320.1 | 0.86704929 | 1.931967408 | 0.053363529 |
| OR4C11 | NR_024321.1 | LINC00115 | 0.839683562 | 1.860402214 | 0.062828642 |
| OR4C11 | NR_034037.1 | LINC00582 | -0.901742674 | -2.024850208 | 0.042882748 |
| OR4C11 | NR_034111.1 | TRAF3IP2-AS1 | 0.932819523 | 2.102437364 | 0.035514981 |
| OR4C11 | NR_046454.1 | LINC00907 | -0.808058498 | -1.81100327 | 0.070140346 |
| OR4C11 | NR_103445.2 | RBFADN | -0.918848388 | -2.060718911 | 0.039329864 |
| OR4C11 | NR_109870.1 | LINC01723 | 0.830473904 | 1.847223745 | 0.064714719 |
| OR4C11 | NR_110568.1 | LOC101927661 | 0.923270188 | 2.054972649 | 0.039881647 |
| OR4C11 | NR_125875.1 | TBX18-AS1 | 0.934685685 | 2.108256794 | 0.035008783 |
| OR4C11 | NR_125876.1 | TBX18-AS1 | 0.945239968 | 2.126904693 | 0.033428003 |
| OR4C11 | NR_126380.1 | LINC01072 | 0.948370276 | 2.115404343 | 0.0343955 |
| OR4C11 | NR_126389.1 | LINC00428 | -0.856785038 | -1.931307875 | 0.05344499 |
| OR4C11 | NR_130144.1 | LOC104968399 | 0.829228872 | 1.828247802 | 0.06751237 |
| OR4C11 | NR_133658.1 | HTR3E-AS1 | -0.916694336 | -2.042171625 | 0.04113451 |
| OR4C11 | NR_133907.1 | HLA-DQB1-AS1 | 0.811785366 | 1.802404595 | 0.071481773 |
| OR4C11 | NR_134245.1 | LOC105379194 | 0.862500206 | 1.94814359 | 0.051397784 |

| | | | | | |
|---|---|---|---|---|---|
| OR4C11 | NR_138041.1 | LINC00384 | 0.899398336 | 2.010618049 | 0.044365816 |
| OR4S2 | ENST00000412812.1 | ENSG00000225342.1 | 0.919688849 | 2.050417023 | 0.040323753 |
| OR4S2 | ENST00000416401.1 | ENSG00000237756.1 | -0.942393566 | -2.098029373 | 0.035902551 |
| OR4S2 | ENST00000422697.1 | ENSG00000236414.1 | -0.832211532 | -1.841423122 | 0.065559577 |
| OR4S2 | ENST00000424241.1 | ENSG00000237311.1 | -0.902242169 | -2.048151257 | 0.040545181 |
| OR4S2 | ENST00000425364.1 | ENSG00000231046.1 | -0.956277014 | -2.123757627 | 0.033690416 |
| OR4S2 | ENST00000426929.1 | ENSG00000230184.1 | -0.843619631 | -1.887755179 | 0.059058825 |
| OR4S2 | ENST00000429796.1 | ENSG00000231858.1 | -0.865105233 | -1.940406459 | 0.052330312 |
| OR4S2 | ENST00000429878.1 | ENSG00000224184.1 | -0.865845628 | -1.949365093 | 0.05125184 |
| OR4S2 | ENST00000429916.1 | ENSG00000227708.1 | 0.831640039 | 1.842455976 | 0.06540848 |
| OR4S2 | ENST00000430545.1 | ENSG00000237153.1 | 0.91634268 | 2.069042037 | 0.038542142 |
| OR4S2 | ENST00000432244.1 | ENSG00000234265.1 | 0.854573833 | 1.907675631 | 0.056433153 |
| OR4S2 | ENST00000432559.2 | ENSG00000228229.2 | 0.96890056 | 2.168973104 | 0.030084726 |
| OR4S2 | ENST00000432711.1 | ENSG00000234919.1 | -0.926634599 | -2.090061661 | 0.036612261 |
| OR4S2 | ENST00000433249.1 | ENSG00000236556.1 | 0.944755253 | 2.146929002 | 0.031798934 |
| OR4S2 | ENST00000437308.1 | ENSG00000233154.1 | 0.806149583 | 1.78342485 | 0.074517171 |
| OR4S2 | ENST00000437864.1 | ENSG00000231521.1 | 0.887197598 | 2.002373076 | 0.045244622 |
| OR4S2 | ENST00000438488.1 | ENSG00000223812.1 | 0.883338269 | 1.955656982 | 0.050505575 |
| OR4S2 | ENST00000441295.1 | ENSG00000233960.1 | -0.877468574 | -1.980660478 | 0.047629362 |
| OR4S2 | ENST00000441532.1 | ENSG00000234206.1 | -0.847447758 | -1.901934221 | 0.057179755 |
| OR4S2 | ENST00000441666.1 | ENSG00000230379.1 | 0.800678227 | 1.796879493 | 0.072354753 |
| OR4S2 | ENST00000441875.1 | ENSG00000239203.1 | 0.835100266 | 1.885033475 | 0.059425318 |
| OR4S2 | ENST00000444665.1 | ENSG00000228852.2 | 0.812704636 | 1.84066307 | 0.065670949 |
| OR4S2 | ENST00000444731.1 | ENSG00000227131.1 | -0.842994347 | -1.892451039 | 0.058430911 |
| OR4S2 | ENST00000456715.1 | ENSG00000224893.1 | 0.828652332 | 1.809632267 | 0.070352833 |
| OR4S2 | ENST00000456999.1 | ENSG00000230690.1 | 0.82182431 | 1.822660364 | 0.068354847 |
| OR4S2 | ENST00000457602.1 | ENSG00000237576.1 | -0.97367266 | -2.156645031 | 0.031033333 |
| OR4S2 | ENST00000458082.1 | ENSG00000231210.2 | -0.814899407 | -1.847842268 | 0.064625164 |
| OR4S2 | ENST00000474656.1 | ENSG00000244198.1 | 0.944247443 | 2.101650827 | 0.035583874 |
| OR4S2 | ENST00000488040.1 | ENSG00000243176.1 | 0.883736944 | 1.977198203 | 0.048019231 |
| OR4S2 | ENST00000505844.1 | ENSG00000248455.1 | 0.835461879 | 1.859772535 | 0.062917715 |
| OR4S2 | ENST00000508004.2 | ENSG00000251661.3 | 0.924564394 | 2.082770458 | 0.037272152 |
| OR4S2 | ENST00000509983.1 | ENSG00000248173.1 | -0.866495494 | -1.946315363 | 0.051616869 |
| OR4S2 | ENST00000511603.1 | ENSG00000249892.1 | 0.837911304 | 1.867253084 | 0.061866254 |
| OR4S2 | ENST00000514411.1 | ENSG00000250882.1 | 0.83563572 | 1.897736812 | 0.057730761 |
| OR4S2 | ENST00000515504.1 | ENSG00000249588.1 | -0.942223415 | -2.094190153 | 0.036243045 |
| OR4S2 | ENST00000518260.1 | ENSG00000253628.1 | -0.933593882 | -2.077760431 | 0.03773143 |
| OR4S2 | ENST00000518894.1 | ENSG00000204758.3 | 0.94269176 | 2.137120696 | 0.032588176 |
| OR4S2 | ENST00000519844.1 | ENSG00000253824.1 | -0.805750688 | -1.83089568 | 0.067116115 |
| OR4S2 | ENST00000528607.1 | ENSG00000254604.1 | 0.833737721 | 1.859429002 | 0.062966354 |
| OR4S2 | ENST00000528818.1 | ENSG00000232995.3 | 0.928909648 | 2.088105294 | 0.036788338 |
| OR4S2 | ENST00000531136.1 | ENSG00000255558.1 | -0.911996583 | -2.046393605 | 0.040717661 |
| OR4S2 | ENST00000537149.1 | ENSG00000256862.1 | -0.801218964 | -1.782057249 | 0.074739896 |
| OR4S2 | ENST00000538294.1 | ENSG00000250748.2 | -0.842163253 | -1.899892012 | 0.057447292 |
| OR4S2 | ENST00000550138.1 | ENSG00000257467.1 | 0.887134257 | 1.984182483 | 0.047235501 |
| OR4S2 | ENST00000553668.1 | ENSG00000258733.1 | 0.897008009 | 1.990082558 | 0.046581842 |
| OR4S2 | ENST00000554798.1 | ENSG00000258483.1 | 0.857546262 | 1.917900069 | 0.055123683 |
| OR4S2 | ENST00000555689.1 | ENSG00000259049.1 | -0.89221147 | -1.992966708 | 0.046265093 |
| OR4S2 | ENST00000557817.1 | ENSG00000259176.1 | 0.822066102 | 1.837338609 | 0.066159924 |
| OR4S2 | ENST00000557855.1 | ENSG00000259176.1 | 0.918514717 | 2.019117479 | 0.043475011 |
| OR4S2 | ENST00000560134.1 | ENSG00000259176.1 | 0.948445933 | 2.128981707 | 0.033255774 |

| | | | | | |
|---|---|---|---|---|---|
| OR4S2 | ENST00000560193.1 | ENSG00000259176.1 | 0.952633418 | 2.147291665 | 0.031770069 |
| OR4S2 | ENST00000569215.1 | ENSG00000260756.1 | 0.841699278 | 1.85738434 | 0.063256493 |
| OR4S2 | ENST00000569328.1 | ENSG00000261638.1 | 0.86205056 | 1.928671973 | 0.053771598 |
| OR4S2 | ENST00000570158.1 | ENSG00000260937.1 | -0.850430446 | -1.893911525 | 0.058236754 |
| OR4S2 | ENST00000572608.1 | ENSG00000263305.1 | 0.883278675 | 1.986669989 | 0.04695898 |
| OR4S2 | ENST00000573414.1 | ENSG00000263072.1 | 0.868468042 | 1.929228955 | 0.053702445 |
| OR4S2 | ENST00000586338.1 | ENSG00000219410.4 | -0.86050394 | -1.915705182 | 0.055402632 |
| OR4S2 | ENST00000595508.1 | ENSG00000269749.1 | 0.810890095 | 1.815649754 | 0.069424116 |
| OR4S2 | ENST00000597865.1 | ENSG00000268108.1 | 0.877883494 | 1.969332513 | 0.04891492 |
| OR4S2 | ENST00000598131.1 | ENSG00000269043.1 | 0.894269021 | 1.984603881 | 0.04718856 |
| OR4S2 | ENST00000600848.1 | ENSG00000228065.6 | 0.882150529 | 1.965429868 | 0.049364505 |
| OR4S2 | ENST00000601752.1 | ENSG00000268051.1 | 0.883606415 | 1.983249813 | 0.047339532 |
| OR4S2 | ENST00000602418.1 | ENSG00000232295.3 | -0.833186342 | -1.868348122 | 0.061713563 |
| OR4S2 | ENST00000603612.1 | ENSG00000270996.1 | 0.807547971 | 1.822364917 | 0.068399634 |
| OR4S2 | ENST00000606374.1 | ENSG00000272312.1 | -0.868300213 | -1.970354093 | 0.048797803 |
| OR4S2 | ENST00000608422.1 | ENSG00000272866.1 | 0.902951551 | 2.0330046 | 0.042052058 |
| OR4S2 | ENST00000609238.1 | ENSG00000272703.1 | -0.834377434 | -1.886687959 | 0.059202308 |
| OR4S2 | ENST00000609281.1 | ENSG00000273320.1 | 0.830191763 | 1.863711777 | 0.062362191 |
| OR4S2 | NR_034037.1 | LINC00582 | -0.813610708 | -1.845178234 | 0.065011616 |
| OR4S2 | NR_034111.1 | TRAF3IP2-AS1 | 0.959745467 | 2.155674874 | 0.031109062 |
| OR4S2 | NR_103445.2 | RBFADN | -0.84786625 | -1.886174055 | 0.059271504 |
| OR4S2 | NR_109870.1 | LINC01723 | 0.86099904 | 1.934450583 | 0.053057752 |
| OR4S2 | NR_110568.1 | LOC101927661 | 0.859117491 | 1.91991382 | 0.054868786 |
| OR4S2 | NR_110930.1 | LOC101927814 | -0.845981268 | -1.905807759 | 0.056675152 |
| OR4S2 | NR_125875.1 | TBX18-AS1 | 0.962152004 | 2.145241794 | 0.03193352 |
| OR4S2 | NR_125876.1 | TBX18-AS1 | 0.963689491 | 2.186274349 | 0.02879554 |
| OR4S2 | NR_126380.1 | LINC01072 | 0.904384478 | 2.030324576 | 0.042323558 |
| OR4S2 | NR_126389.1 | LINC00428 | -0.872031714 | -1.942718235 | 0.052050212 |
| OR4S2 | NR_133658.1 | HTR3E-AS1 | -0.9725925 | -2.175437783 | 0.029597326 |
| OR4S2 | NR_133907.1 | HLA-DQB1-AS1 | 0.855633981 | 1.897290676 | 0.057789585 |
| OR4S2 | NR_133942.1 | LOC105377247 | 0.887627012 | 1.972832705 | 0.048514626 |
| OR4S2 | NR_134273.1 | LOC101929544 | 0.847333657 | 1.870555873 | 0.061406666 |
| OR6C70 | ENST00000412348.1 | ENSG00000228959.1 | 0.879597733 | 1.929466969 | 0.053672917 |
| OR6C70 | ENST00000418387.1 | ENSG00000235056.1 | 0.95509351 | 2.16932357 | 0.030058127 |
| OR6C70 | ENST00000422017.1 | ENSG00000232227.1 | 0.95217839 | 2.130525359 | 0.033128264 |
| OR6C70 | ENST00000422914.1 | ENSG00000236120.2 | -0.824337041 | -1.837734275 | 0.066101571 |
| OR6C70 | ENST00000426030.2 | ENSG00000228686.2 | 0.824880094 | 1.847235039 | 0.064713083 |
| OR6C70 | ENST00000432265.1 | ENSG00000231170.1 | -0.95913996 | -2.173889341 | 0.029713447 |
| OR6C70 | ENST00000433550.1 | ENSG00000232227.1 | 0.898925533 | 2.012256599 | 0.044192895 |
| OR6C70 | ENST00000436515.1 | ENSG00000224521.1 | 0.878942811 | 1.94063933 | 0.05230204 |
| OR6C70 | ENST00000438409.1 | ENSG00000234174.1 | -0.828781102 | -1.876183229 | 0.060630124 |
| OR6C70 | ENST00000438623.1 | ENSG00000224521.1 | 0.830281437 | 1.842946132 | 0.065336876 |
| OR6C70 | ENST00000442829.1 | ENSG00000225284.1 | -0.867804707 | -1.954074163 | 0.050692447 |
| OR6C70 | ENST00000448748.1 | ENSG00000231238.1 | 0.966817811 | 2.138684692 | 0.032461213 |
| OR6C70 | ENST00000450779.1 | ENSG00000228858.1 | 0.843543247 | 1.876674084 | 0.060562777 |
| OR6C70 | ENST00000451034.1 | ENSG00000229805.1 | 0.880612946 | 1.964426119 | 0.049480696 |
| OR6C70 | ENST00000451090.1 | ENSG00000235215.2 | 0.838256765 | 1.874428899 | 0.060871333 |
| OR6C70 | ENST00000451267.1 | ENSG00000230410.1 | -0.812782837 | -1.837490944 | 0.066137452 |
| OR6C70 | ENST00000451828.1 | ENSG00000228549.2 | -0.843114017 | -1.86885395 | 0.061643137 |
| OR6C70 | ENST00000452511.1 | ENSG00000231876.3 | 0.944285012 | 2.121158792 | 0.033908442 |
| OR6C70 | ENST00000454965.1 | ENSG00000235435.1 | 0.818753077 | 1.84339455 | 0.065271425 |

| | | | | | |
|---|---|---|---|---|---|
| OR6C70 | ENST00000457043.1 | ENSG00000231365.1 | 0.902423819 | 2.024085518 | 0.042961353 |
| OR6C70 | ENST00000457856.1 | ENSG00000228549.2 | -0.834619954 | -1.851328049 | 0.064122373 |
| OR6C70 | ENST00000485338.1 | ENSG00000239641.1 | 0.965984232 | 2.143434518 | 0.032078224 |
| OR6C70 | ENST00000489690.1 | ENSG00000243944.1 | 0.902423819 | 1.98055648 | 0.047641034 |
| OR6C70 | ENST00000490375.1 | ENSG00000240032.1 | 0.802119642 | 1.792193978 | 0.073101899 |
| OR6C70 | ENST00000502467.1 | ENSG00000250530.1 | 0.953963622 | 2.183392712 | 0.029006902 |
| OR6C70 | ENST00000503505.1 | ENSG00000248629.1 | 0.848924495 | 1.905029677 | 0.056776213 |
| OR6C70 | ENST00000503987.1 | ENSG00000250075.1 | 0.802078915 | 1.794559133 | 0.072723969 |
| OR6C70 | ENST00000504765.1 | ENSG00000249638.1 | 0.886070254 | 1.965552365 | 0.049350341 |
| OR6C70 | ENST00000505978.1 | ENSG00000249982.1 | -0.8906264 | -1.961289515 | 0.04984526 |
| OR6C70 | ENST00000508925.2 | ENSG00000249196.2 | -0.825927356 | -1.868476392 | 0.061695698 |
| OR6C70 | ENST00000509718.1 | ENSG00000251132.1 | -0.852487837 | -1.897267108 | 0.057792694 |
| OR6C70 | ENST00000512036.1 | ENSG00000250993.1 | 0.813433838 | 1.830676582 | 0.06714883 |
| OR6C70 | ENST00000512882.2 | ENSG00000251575.2 | 0.917642453 | 2.063729958 | 0.039043327 |
| OR6C70 | ENST00000522390.1 | ENSG00000254262.1 | 0.889253673 | 1.979532698 | 0.047756062 |
| OR6C70 | ENST00000532123.1 | ENSG00000255555.1 | 0.95217164 | 2.181241579 | 0.029165553 |
| OR6C70 | ENST00000534178.1 | ENSG00000255120.1 | -0.834388486 | -1.865512889 | 0.062109547 |
| OR6C70 | ENST00000537492.1 | ENSG00000256637.2 | 0.842692457 | 1.901581083 | 0.057225943 |
| OR6C70 | ENST00000539963.1 | ENSG00000256116.1 | -0.848295452 | -1.874599441 | 0.06084785 |
| OR6C70 | ENST00000545158.1 | ENSG00000256011.1 | 0.869922299 | 1.953672707 | 0.050739936 |
| OR6C70 | ENST00000545357.1 | ENSG00000256862.1 | -0.85900472 | -1.890945303 | 0.058631646 |
| OR6C70 | ENST00000549683.1 | ENSG00000257953.1 | -0.814323435 | -1.808706888 | 0.070496552 |
| OR6C70 | ENST00000550279.1 | ENSG00000258338.1 | 0.841272393 | 1.858166074 | 0.063145434 |
| OR6C70 | ENST00000553954.1 | ENSG00000259052.1 | -0.888957925 | -1.96774589 | 0.049097283 |
| OR6C70 | ENST00000554431.1 | ENSG00000258616.1 | 0.8599999 | 1.935229391 | 0.052962153 |
| OR6C70 | ENST00000557602.1 | ENSG00000258616.1 | 0.870731544 | 1.924635445 | 0.054274982 |
| OR6C70 | ENST00000557817.1 | ENSG00000259176.1 | 0.806103591 | 1.788421639 | 0.073708008 |
| OR6C70 | ENST00000558237.1 | ENSG00000259684.1 | 0.920139968 | 2.051951707 | 0.040174356 |
| OR6C70 | ENST00000560963.1 | ENSG00000259370.1 | -0.80800898 | -1.804476269 | 0.071156676 |
| OR6C70 | ENST00000561254.1 | ENSG00000259554.1 | 0.844003547 | 1.903355315 | 0.056994198 |
| OR6C70 | ENST00000561699.1 | ENSG00000259813.1 | -0.953787062 | -2.132267121 | 0.032984893 |
| OR6C70 | ENST00000563610.1 | ENSG00000260051.1 | -0.836102627 | -1.883599174 | 0.059619214 |
| OR6C70 | ENST00000570974.1 | ENSG00000263300.1 | -0.914147326 | -2.049303035 | 0.040432492 |
| OR6C70 | ENST00000571775.1 | ENSG00000262456.1 | -0.832729725 | -1.89549201 | 0.058027249 |
| OR6C70 | ENST00000580311.1 | ENSG00000266803.1 | 0.834546507 | 1.86694694 | 0.061908998 |
| OR6C70 | ENST00000580729.1 | ENSG00000266176.1 | -0.827920249 | -1.838672374 | 0.065963389 |
| OR6C70 | ENST00000584139.1 | ENSG00000263388.1 | 0.903534485 | 2.016116118 | 0.043787835 |
| OR6C70 | ENST00000585810.1 | ENSG00000236172.2 | -0.810438428 | -1.81953561 | 0.068829755 |
| OR6C70 | ENST00000587702.1 | ENSG00000267378.1 | 0.94071432 | 2.116116962 | 0.034334861 |
| OR6C70 | ENST00000588402.1 | ENSG00000267006.1 | 0.805712485 | 1.82793094 | 0.067559917 |
| OR6C70 | ENST00000590328.1 | ENSG00000256995.2 | 0.883261325 | 2.026571801 | 0.042706223 |
| OR6C70 | ENST00000595007.1 | ENSG00000231876.3 | 0.926935385 | 2.070159913 | 0.038437372 |
| OR6C70 | ENST00000597755.1 | ENSG00000236194.2 | 0.818059621 | 1.840085483 | 0.065755688 |
| OR6C70 | ENST00000600007.1 | ENSG00000268655.1 | -0.865169157 | -1.952985144 | 0.050821356 |
| OR6C70 | ENST00000600716.1 | ENSG00000269487.1 | -0.911140908 | -2.007088401 | 0.044740251 |
| OR6C70 | ENST00000602405.1 | ENSG00000269928.1 | -0.809556365 | -1.806312645 | 0.070869518 |
| OR6C70 | ENST00000602881.1 | ENSG00000269965.1 | 0.817881836 | 1.807896168 | 0.070622662 |
| OR6C70 | ENST00000606010.1 | ENSG00000272249.1 | 0.90056241 | 2.056231311 | 0.039760226 |
| OR6C70 | ENST00000607119.1 | ENSG00000272541.1 | 0.800272248 | 1.775205073 | 0.075864032 |
| OR6C70 | ENST00000607135.1 | ENSG00000272112.1 | 0.925888295 | 2.054753176 | 0.039902851 |
| OR6C70 | ENST00000607549.1 | ENSG00000272293.1 | 0.834566818 | 1.837060907 | 0.066200904 |

| | | | | | |
|---|---|---|---|---|---|
| OR6C70 | ENST00000608259.1 | ENSG00000272627.1 | 0.859044164 | 1.926098239 | 0.054092109 |
| OR6C70 | ENST00000608465.1 | ENSG00000272758.1 | 0.88010695 | 1.974205306 | 0.048358403 |
| OR6C70 | ENST00000609813.1 | ENSG00000272719.1 | -0.873936239 | -1.928010611 | 0.053853806 |
| OR6C70 | ENST00000609955.1 | ENSG00000273275.1 | 0.855201805 | 1.872729638 | 0.061105728 |
| OR6C70 | NR_024491.1 | LOC100128573 | 0.806742347 | 1.755115938 | 0.079239479 |
| OR6C70 | NR_026962.1 | TTC28-AS1 | -0.905013072 | -2.008449137 | 0.044595586 |
| OR6C70 | NR_027067.1 | LINC00114 | -0.846425623 | -1.90403921 | 0.056905078 |
| OR6C70 | NR_047498.1 | LINC00853 | -0.824725388 | -1.835753462 | 0.066394127 |
| OR6C70 | NR_073552.1 | LOC101059948 | -0.865169157 | -1.932983518 | 0.053238228 |
| OR6C70 | NR_103830.1 | LINC00587 | 0.912388207 | 2.065728408 | 0.038854131 |
| OR6C70 | NR_110702.1 | SEMA3B-AS1 | -0.803941002 | -1.795671587 | 0.072546763 |
| OR6C70 | NR_110731.1 | LINC01232 | -0.831129566 | -1.872595941 | 0.061124202 |
| OR6C70 | NR_126409.1 | LINC00376 | 0.886239629 | 1.990558853 | 0.046529408 |
| OR6C70 | NR_134576.1 | LOC105372672 | -0.805687502 | -1.789124621 | 0.073594748 |
| OR6C70 | NR_136569.1 | LINC01660 | 0.987636542 | 2.236309692 | 0.025331492 |
| OR8H2 | ENST00000400768.2 | ENSG00000215692.2 | 0.894469292 | 2.015024761 | 0.043902055 |
| OR8H2 | ENST00000413969.1 | ENSG00000224189.2 | 0.909636647 | 2.02447926 | 0.042920864 |
| OR8H2 | ENST00000414377.1 | ENSG00000230470.1 | 0.951563994 | 2.124716441 | 0.033610281 |
| OR8H2 | ENST00000416641.1 | ENSG00000226956.1 | -0.802807701 | -1.790543855 | 0.073366524 |
| OR8H2 | ENST00000417782.1 | ENSG00000228587.1 | 0.97734153 | 2.197239128 | 0.028003372 |
| OR8H2 | ENST00000418621.1 | ENSG00000224731.1 | 0.883246938 | 1.95321264 | 0.050794404 |
| OR8H2 | ENST00000418741.1 | ENSG00000227332.1 | 0.90567117 | 2.02305927 | 0.043067037 |
| OR8H2 | ENST00000420830.1 | ENSG00000231512.1 | 0.98242545 | 2.181596077 | 0.029139357 |
| OR8H2 | ENST00000421252.2 | ENSG00000250258.1 | -0.8031829 | -1.800822348 | 0.071730885 |
| OR8H2 | ENST00000421737.1 | ENSG00000232316.1 | 0.942539569 | 2.096962995 | 0.035996851 |
| OR8H2 | ENST00000428769.1 | ENSG00000232738.1 | 0.908351578 | 2.011721565 | 0.044249296 |
| OR8H2 | ENST00000430534.1 | ENSG00000229297.1 | -0.836717494 | -1.854935219 | 0.063605478 |
| OR8H2 | ENST00000430728.1 | ENSG00000232316.1 | 0.972397438 | 2.183309527 | 0.029013023 |
| OR8H2 | ENST00000432314.1 | ENSG00000231532.1 | 0.835723552 | 1.863184061 | 0.062436375 |
| OR8H2 | ENST00000433174.1 | ENSG00000162947.4 | 0.937881878 | 2.056012693 | 0.039781293 |
| OR8H2 | ENST00000434790.1 | ENSG00000240040.1 | -0.926834309 | -2.062912798 | 0.039120914 |
| OR8H2 | ENST00000435271.1 | ENSG00000231132.1 | -0.868487658 | -1.952806537 | 0.050842524 |
| OR8H2 | ENST00000435315.2 | ENSG00000226751.2 | 0.826504237 | 1.843951237 | 0.065190247 |
| OR8H2 | ENST00000435832.1 | ENSG00000229201.1 | 0.847868045 | 1.892164095 | 0.058469121 |
| OR8H2 | ENST00000440038.2 | ENSG00000237094.7 | -0.824589791 | -1.832991425 | 0.066803846 |
| OR8H2 | ENST00000440947.1 | ENSG00000225472.1 | 0.930956077 | 2.033884282 | 0.041963264 |
| OR8H2 | ENST00000447538.2 | ENSG00000224189.2 | 0.882577175 | 1.959827176 | 0.050015994 |
| OR8H2 | ENST00000448431.1 | ENSG00000232548.1 | 0.814426961 | 1.827207205 | 0.067668621 |
| OR8H2 | ENST00000450226.1 | ENSG00000231512.1 | 0.98259738 | 2.217930393 | 0.026559579 |
| OR8H2 | ENST00000450365.1 | ENSG00000224404.1 | 0.839804995 | 1.884577231 | 0.059486939 |
| OR8H2 | ENST00000451556.2 | ENSG00000228386.2 | 0.991331031 | 2.222093335 | 0.026276999 |
| OR8H2 | ENST00000451575.2 | ENSG00000224251.2 | 0.873202475 | 1.951815027 | 0.05096017 |
| OR8H2 | ENST00000452412.1 | ENSG00000233860.1 | 0.806042243 | 1.799385502 | 0.071957721 |
| OR8H2 | ENST00000453579.1 | ENSG00000232529.1 | 0.816438454 | 1.826994728 | 0.067700562 |
| OR8H2 | ENST00000453889.1 | ENSG00000224750.2 | 0.89586536 | 2.012438347 | 0.04417375 |
| OR8H2 | ENST00000455699.1 | ENSG00000240996.1 | 0.871388108 | 1.95631873 | 0.050427619 |
| OR8H2 | ENST00000458661.2 | ENSG00000236467.3 | 0.865722308 | 1.967503686 | 0.049125171 |
| OR8H2 | ENST00000469846.2 | ENSG00000206573.4 | -0.805386192 | -1.8072039 | 0.070730493 |
| OR8H2 | ENST00000469931.2 | ENSG00000272030.1 | 0.896727354 | 2.00329619 | 0.045145506 |
| OR8H2 | ENST00000503323.1 | ENSG00000249881.1 | -0.803910875 | -1.792361546 | 0.07307507 |
| OR8H2 | ENST00000504017.1 | ENSG00000248388.1 | 0.838438012 | 1.873884872 | 0.060946295 |

| | | | | | |
|---|---|---|---|---|---|
| OR8H2 | ENST00000504344.1 | ENSG00000251438.1 | 0.849901053 | 1.917632142 | 0.055157672 |
| OR8H2 | ENST00000505575.1 | ENSG00000248939.1 | -0.843001601 | -1.89013217 | 0.058740286 |
| OR8H2 | ENST00000506059.1 | ENSG00000248311.1 | 0.802502818 | 1.800493822 | 0.071782698 |
| OR8H2 | ENST00000507373.1 | ENSG00000250072.1 | 0.86911764 | 1.94441057 | 0.05184596 |
| OR8H2 | ENST00000508191.1 | ENSG00000250910.3 | 0.819272659 | 1.827694882 | 0.067595357 |
| OR8H2 | ENST00000508823.1 | ENSG00000250716.1 | 0.820507487 | 1.817149267 | 0.069194261 |
| OR8H2 | ENST00000508986.1 | ENSG00000249491.1 | 0.914703707 | 2.045039254 | 0.040850988 |
| OR8H2 | ENST00000513179.1 | ENSG00000251580.1 | 0.869057435 | 1.947070258 | 0.051526312 |
| OR8H2 | ENST00000515205.1 | ENSG00000251580.1 | 0.852022936 | 1.921732892 | 0.054639376 |
| OR8H2 | ENST00000515789.1 | ENSG00000248571.1 | -0.900859798 | -2.013626487 | 0.044048763 |
| OR8H2 | ENST00000523935.1 | ENSG00000253567.1 | 0.927883207 | 2.086837703 | 0.036902808 |
| OR8H2 | ENST00000524275.1 | ENSG00000253507.1 | 0.808499991 | 1.765820619 | 0.077425948 |
| OR8H2 | ENST00000527727.1 | ENSG00000255227.1 | 0.833522672 | 1.856269125 | 0.063415208 |
| OR8H2 | ENST00000529875.1 | ENSG00000254404.1 | -0.885649804 | -1.962681737 | 0.049683167 |
| OR8H2 | ENST00000531009.1 | ENSG00000255208.1 | 0.867011735 | 1.950048877 | 0.051170294 |
| OR8H2 | ENST00000531661.1 | ENSG00000254473.1 | 0.911619333 | 2.044461796 | 0.040907948 |
| OR8H2 | ENST00000536529.1 | ENSG00000256422.1 | 0.825038504 | 1.833958048 | 0.066660222 |
| OR8H2 | ENST00000538641.1 | ENSG00000256422.1 | 0.839714743 | 1.861342064 | 0.062695887 |
| OR8H2 | ENST00000541391.1 | ENSG00000256268.1 | 0.813552489 | 1.826448471 | 0.067782736 |
| OR8H2 | ENST00000547207.1 | ENSG00000224189.2 | 0.952913382 | 2.10964558 | 0.034888895 |
| OR8H2 | ENST00000549140.1 | ENSG00000258332.1 | 0.83945207 | 1.882869818 | 0.059718012 |
| OR8H2 | ENST00000549329.1 | ENSG00000224189.2 | 0.926834309 | 2.068547309 | 0.038588587 |
| OR8H2 | ENST00000552156.1 | ENSG00000224189.2 | 0.950898515 | 2.119871503 | 0.034016883 |
| OR8H2 | ENST00000552261.1 | ENSG00000257959.1 | 0.84771858 | 1.929030669 | 0.053727055 |
| OR8H2 | ENST00000554451.1 | ENSG00000258683.1 | 0.850853782 | 1.898505284 | 0.057629552 |
| OR8H2 | ENST00000555636.1 | ENSG00000259072.1 | 0.836912672 | 1.888041787 | 0.059020341 |
| OR8H2 | ENST00000555966.1 | ENSG00000258843.1 | 0.835939876 | 1.892432091 | 0.058433434 |
| OR8H2 | ENST00000557903.1 | ENSG00000259182.1 | 0.867366456 | 1.963584776 | 0.049578264 |
| OR8H2 | ENST00000557965.1 | ENSG00000259681.1 | 0.871559507 | 1.932415352 | 0.053308261 |
| OR8H2 | ENST00000559003.1 | ENSG00000259520.1 | 0.805311244 | 1.800355689 | 0.071804493 |
| OR8H2 | ENST00000560586.1 | ENSG00000259534.1 | 0.846971514 | 1.870045348 | 0.061477521 |
| OR8H2 | ENST00000565722.1 | ENSG00000245768.2 | 0.919208163 | 2.045461567 | 0.040809374 |
| OR8H2 | ENST00000567089.1 | ENSG00000261822.1 | 0.840734882 | 1.897462814 | 0.057766882 |
| OR8H2 | ENST00000568243.1 | ENSG00000261521.1 | 0.845241145 | 1.906752073 | 0.0565527 |
| OR8H2 | ENST00000569849.1 | ENSG00000260640.1 | 0.950167181 | 2.163073375 | 0.030535537 |
| OR8H2 | ENST00000572417.1 | ENSG00000263171.1 | 0.91830392 | 2.055471262 | 0.039833509 |
| OR8H2 | ENST00000574365.1 | ENSG00000262837.1 | 0.846780638 | 1.889890479 | 0.058772609 |
| OR8H2 | ENST00000578035.1 | ENSG00000266743.1 | -0.849829159 | -1.906542076 | 0.056579912 |
| OR8H2 | ENST00000578349.1 | ENSG00000263688.1 | 0.888737932 | 1.99158907 | 0.046416164 |
| OR8H2 | ENST00000578572.1 | ENSG00000196295.7 | 0.843434 | 1.857392727 | 0.063255301 |
| OR8H2 | ENST00000580085.1 | ENSG00000266490.1 | 0.801465889 | 1.7791279 | 0.075218796 |
| OR8H2 | ENST00000581362.1 | ENSG00000235300.3 | 0.906709623 | 2.042160443 | 0.041135619 |
| OR8H2 | ENST00000581996.1 | ENSG00000265778.1 | 0.929146591 | 2.105895044 | 0.03521347 |
| OR8H2 | ENST00000582348.1 | ENSG00000265148.1 | 0.920941617 | 2.073045897 | 0.038168008 |
| OR8H2 | ENST00000583841.1 | ENSG00000265148.1 | 0.800188958 | 1.786345761 | 0.074043292 |
| OR8H2 | ENST00000584758.1 | ENSG00000265356.1 | 0.824296627 | 1.853213042 | 0.063851829 |
| OR8H2 | ENST00000586952.1 | ENSG00000226994.3 | 0.850824586 | 1.936247651 | 0.052837377 |
| OR8H2 | ENST00000588835.1 | ENSG00000267476.1 | 0.817567367 | 1.830720449 | 0.067142279 |
| OR8H2 | ENST00000589777.1 | ENSG00000261040.2 | 0.903341684 | 2.032668332 | 0.042086043 |
| OR8H2 | ENST00000590357.1 | ENSG00000267175.1 | -0.801434683 | -1.793259959 | 0.072931367 |
| OR8H2 | ENST00000591469.1 | ENSG00000267374.1 | 0.880624383 | 1.978411128 | 0.047882345 |

| | | | | | |
|---|---|---|---|---|---|
| OR8H2 | ENST00000593486.1 | ENSG00000250910.3 | 0.832040514 | 1.869863715 | 0.061502746 |
| OR8H2 | ENST00000593861.1 | ENSG00000231898.4 | 0.849954044 | 1.892714072 | 0.058395904 |
| OR8H2 | ENST00000594589.1 | ENSG00000269321.1 | -0.879357197 | -1.961914679 | 0.049772418 |
| OR8H2 | ENST00000595892.1 | ENSG00000269640.1 | 0.883930626 | 1.976273196 | 0.048123844 |
| OR8H2 | ENST00000597550.1 | ENSG00000269051.1 | 0.826280231 | 1.8417918 | 0.06550561 |
| OR8H2 | ENST00000599143.1 | ENSG00000269349.1 | 0.855364158 | 1.902786415 | 0.057068421 |
| OR8H2 | ENST00000600365.1 | ENSG00000231898.4 | 0.88957742 | 2.008039731 | 0.04463907 |
| OR8H2 | ENST00000602835.1 | ENSG00000270096.1 | 0.821281371 | 1.854022794 | 0.063735899 |
| OR8H2 | ENST00000603474.1 | ENSG00000258929.2 | 0.842645973 | 1.859760009 | 0.062919488 |
| OR8H2 | ENST00000604464.1 | ENSG00000270462.1 | 0.822129277 | 1.857344076 | 0.063262217 |
| OR8H2 | ENST00000606457.1 | ENSG00000271830.1 | 0.857203071 | 1.940236591 | 0.052350944 |
| OR8H2 | ENST00000607715.1 | ENSG00000271788.1 | 0.922590633 | 2.05679776 | 0.039705684 |
| OR8H2 | ENST00000607769.1 | ENSG00000272438.1 | -0.862082243 | -1.911444336 | 0.055947504 |
| OR8H2 | ENST00000607876.1 | ENSG00000272848.1 | 0.851364569 | 1.904420564 | 0.056855433 |
| OR8H2 | ENST00000608085.1 | ENSG00000231898.4 | 0.944578314 | 2.135472424 | 0.03272244 |
| OR8H2 | ENST00000608476.1 | ENSG00000232675.3 | 0.814980003 | 1.827895275 | 0.06756527 |
| OR8H2 | ENST00000609725.1 | ENSG00000231898.4 | 0.858552304 | 1.902652356 | 0.057085923 |
| OR8H2 | ENST00000609952.1 | ENSG00000233766.3 | 0.820749799 | 1.810634601 | 0.070197432 |
| OR8H2 | NR_024410.1 | LINC00710 | 0.949614343 | 2.120406546 | 0.033971775 |
| OR8H2 | NR_033371.1 | CDRT7 | 0.88676548 | 1.977105036 | 0.048029759 |
| OR8H2 | NR_038194.1 | LINC00583 | 0.825913962 | 1.827102123 | 0.067684416 |
| OR8H2 | NR_046845.1 | DNM3-IT1 | 0.904423398 | 2.021766143 | 0.043200517 |
| OR8H2 | NR_104618.1 | LINC01017 | 0.838878575 | 1.871149744 | 0.061324328 |
| OR8H2 | NR_104998.1 | LOC102467225 | 0.892140729 | 1.965203895 | 0.049390643 |
| OR8H2 | NR_120318.1 | RORA-AS2 | 0.858823957 | 1.899479291 | 0.057501487 |
| OR8H2 | NR_120655.1 | KCNMA1-AS1 | 0.922086052 | 2.057816335 | 0.039607767 |
| OR8H2 | NR_134265.1 | LINC02103 | 0.858266185 | 1.919051775 | 0.054977782 |
| OR8H2 | NR_134597.1 | LOC105378068 | 0.806661154 | 1.804046937 | 0.071223949 |
| OR8H2 | NR_135549.1 | LOC101929411 | 0.808664895 | 1.787948364 | 0.073784339 |
| OR8H2 | NR_135840.1 | LOC105376114 | 0.871187022 | 1.931504993 | 0.053420632 |
| OR8H2 | NR_138419.1 | ARHGEF9-IT1 | 0.883347874 | 1.970742359 | 0.048753353 |
| OR9K2 | ENST00000417315.1 | ENSG00000242486.1 | -0.819010078 | -1.835526955 | 0.066427649 |
| OR9K2 | ENST00000418621.1 | ENSG00000224731.1 | 0.946116163 | 2.106761888 | 0.035138225 |
| OR9K2 | ENST00000420830.1 | ENSG00000231512.1 | 0.81023981 | 1.859375012 | 0.062974001 |
| OR9K2 | ENST00000421006.1 | ENSG00000234548.1 | 0.811360982 | 1.792955482 | 0.072980043 |
| OR9K2 | ENST00000421597.1 | ENSG00000227851.1 | 0.881462238 | 1.977580826 | 0.047976014 |
| OR9K2 | ENST00000423796.1 | ENSG00000235146.2 | 0.826993739 | 1.848861268 | 0.064477848 |
| OR9K2 | ENST00000424342.1 | ENSG00000234988.1 | -0.884126342 | -1.981324071 | 0.047554943 |
| OR9K2 | ENST00000425058.1 | ENSG00000226771.1 | 0.861345644 | 1.907064393 | 0.056512249 |
| OR9K2 | ENST00000426125.1 | ENSG00000223653.1 | 0.840592769 | 1.909347666 | 0.056217257 |
| OR9K2 | ENST00000426302.1 | ENSG00000230454.1 | -0.823785202 | -1.836927153 | 0.06622065 |
| OR9K2 | ENST00000427524.1 | ENSG00000236065.2 | -0.883162612 | -1.986600757 | 0.046966657 |
| OR9K2 | ENST00000428160.1 | ENSG00000236897.1 | 0.800611397 | 1.802942408 | 0.07139726 |
| OR9K2 | ENST00000430247.1 | ENSG00000232855.2 | 0.890003493 | 2.007593702 | 0.044686484 |
| OR9K2 | ENST00000434292.1 | ENSG00000229796.1 | 0.962687284 | 2.146809137 | 0.03180848 |
| OR9K2 | ENST00000434493.1 | ENSG00000224605.1 | 0.828944138 | 1.855109141 | 0.063580643 |
| OR9K2 | ENST00000435271.1 | ENSG00000231132.1 | -0.920462956 | -2.035581654 | 0.041792382 |
| OR9K2 | ENST00000435828.1 | ENSG00000235612.1 | 0.814934199 | 1.821543616 | 0.068524263 |
| OR9K2 | ENST00000435832.1 | ENSG00000229201.1 | 0.857960352 | 1.927898358 | 0.05386777 |
| OR9K2 | ENST00000437680.1 | ENSG00000237133.1 | -0.805384676 | -1.787706203 | 0.07382342 |
| OR9K2 | ENST00000438173.2 | ENSG00000227733.4 | 0.851072638 | 1.891633104 | 0.058539882 |

| | | | | | |
|---|---|---|---|---|---|
| OR9K2 | ENST00000439529.1 | ENSG00000236526.1 | 0.823474247 | 1.857549754 | 0.06323298 |
| OR9K2 | ENST00000440038.2 | ENSG00000237094.7 | -0.871118834 | -1.934834429 | 0.053010617 |
| OR9K2 | ENST00000442017.1 | ENSG00000229660.1 | -0.912858535 | -2.064635149 | 0.038957535 |
| OR9K2 | ENST00000442852.1 | ENSG00000237923.1 | -0.902293773 | -2.021813227 | 0.043195651 |
| OR9K2 | ENST00000445233.1 | ENSG00000233928.1 | 0.92395878 | 2.067458909 | 0.038690932 |
| OR9K2 | ENST00000447111.1 | ENSG00000231903.1 | 0.832458929 | 1.852344342 | 0.063976392 |
| OR9K2 | ENST00000448431.1 | ENSG00000232548.1 | 0.83532286 | 1.879552322 | 0.060169116 |
| OR9K2 | ENST00000448858.1 | ENSG00000237734.1 | 0.808867036 | 1.776930733 | 0.075579637 |
| OR9K2 | ENST00000449903.1 | ENSG00000223872.1 | -0.807179878 | -1.780047463 | 0.075068193 |
| OR9K2 | ENST00000450206.1 | ENSG00000234311.1 | -0.871544356 | -1.929779292 | 0.05363419 |
| OR9K2 | ENST00000451090.1 | ENSG00000235215.2 | 0.872929918 | 1.977823924 | 0.047948574 |
| OR9K2 | ENST00000451267.1 | ENSG00000230410.1 | -0.818180722 | -1.833617872 | 0.066710737 |
| OR9K2 | ENST00000451556.2 | ENSG00000228386.2 | 0.812623677 | 1.82809862 | 0.067534752 |
| OR9K2 | ENST00000453584.1 | ENSG00000233613.1 | -0.878630725 | -1.958686892 | 0.050149466 |
| OR9K2 | ENST00000453878.1 | ENSG00000224850.1 | 0.83772288 | 1.889980311 | 0.058760593 |
| OR9K2 | ENST00000455373.1 | ENSG00000226097.1 | 0.889859949 | 2.013851223 | 0.044025156 |
| OR9K2 | ENST00000457043.1 | ENSG00000231365.1 | 0.856737314 | 1.913821722 | 0.055642939 |
| OR9K2 | ENST00000457632.1 | ENSG00000234248.1 | 0.897733795 | 1.980436914 | 0.047654456 |
| OR9K2 | ENST00000480904.2 | ENSG00000206573.4 | -0.820672599 | -1.82655036 | 0.067767403 |
| OR9K2 | ENST00000483283.1 | ENSG00000240571.1 | 0.945928954 | 2.121639183 | 0.033868049 |
| OR9K2 | ENST00000503323.1 | ENSG00000249881.1 | -0.825901806 | -1.834558386 | 0.066571149 |
| OR9K2 | ENST00000503987.1 | ENSG00000250075.1 | 0.822390317 | 1.821636834 | 0.068510108 |
| OR9K2 | ENST00000505575.1 | ENSG00000248939.1 | -0.959597023 | -2.183524316 | 0.02899722 |
| OR9K2 | ENST00000506379.1 | ENSG00000240152.2 | 0.929862837 | 2.074166474 | 0.038063853 |
| OR9K2 | ENST00000506723.2 | ENSG00000249484.4 | 0.823992283 | 1.838311772 | 0.066016477 |
| OR9K2 | ENST00000506852.1 | ENSG00000250945.1 | 0.897409333 | 2.018926719 | 0.043494837 |
| OR9K2 | ENST00000508191.1 | ENSG00000250910.3 | 0.878744727 | 1.952594516 | 0.050867661 |
| OR9K2 | ENST00000508823.1 | ENSG00000250716.1 | 0.952963446 | 2.115491432 | 0.034388084 |
| OR9K2 | ENST00000509629.1 | ENSG00000250164.1 | -0.87379948 | -1.966917314 | 0.049192744 |
| OR9K2 | ENST00000518837.1 | ENSG00000253947.1 | 0.849399067 | 1.910213778 | 0.056105694 |
| OR9K2 | ENST00000519005.1 | ENSG00000253507.1 | 0.910746711 | 2.028730579 | 0.04248574 |
| OR9K2 | ENST00000519375.1 | ENSG00000253980.1 | 0.877626101 | 1.941604054 | 0.052185052 |
| OR9K2 | ENST00000519695.1 | ENSG00000253507.1 | 0.864945771 | 1.932474746 | 0.053300936 |
| OR9K2 | ENST00000520192.1 | ENSG00000253807.1 | 0.985209303 | 2.192089591 | 0.028373038 |
| OR9K2 | ENST00000521294.1 | ENSG00000253664.1 | -0.896267339 | -2.033125436 | 0.042039852 |
| OR9K2 | ENST00000521378.1 | ENSG00000254222.1 | -0.849920552 | -1.907820768 | 0.056414386 |
| OR9K2 | ENST00000521490.1 | ENSG00000253407.1 | -0.850684502 | -1.924190132 | 0.054330756 |
| OR9K2 | ENST00000521725.1 | ENSG00000253396.1 | 0.953413875 | 2.092588929 | 0.036385866 |
| OR9K2 | ENST00000522190.1 | ENSG00000254165.1 | 0.882491871 | 1.975607529 | 0.048199245 |
| OR9K2 | ENST00000522281.1 | ENSG00000253376.1 | 0.895149214 | 2.021608043 | 0.04321686 |
| OR9K2 | ENST00000522300.1 | ENSG00000249484.4 | 0.930777149 | 2.085741364 | 0.037002057 |
| OR9K2 | ENST00000522390.1 | ENSG00000254262.1 | 0.852725968 | 1.913869099 | 0.055636884 |
| OR9K2 | ENST00000522426.1 | ENSG00000253538.1 | 0.88448143 | 1.983341137 | 0.047329337 |
| OR9K2 | ENST00000523806.1 | ENSG00000253616.1 | -0.844356667 | -1.873612965 | 0.06098379 |
| OR9K2 | ENST00000523935.1 | ENSG00000253567.1 | 0.87088418 | 1.930987932 | 0.053484545 |
| OR9K2 | ENST00000524133.1 | ENSG00000253174.2 | -0.879830863 | -1.950609828 | 0.051103477 |
| OR9K2 | ENST00000524275.1 | ENSG00000253507.1 | 0.869715418 | 1.936366064 | 0.052822883 |
| OR9K2 | ENST00000525855.1 | ENSG00000254746.1 | -0.808347952 | -1.816290348 | 0.069325845 |
| OR9K2 | ENST00000527274.2 | ENSG00000255517.2 | -0.933848201 | -2.094826841 | 0.036186388 |
| OR9K2 | ENST00000527727.1 | ENSG00000255227.1 | 0.952190599 | 2.129570005 | 0.03320713 |
| OR9K2 | ENST00000529875.1 | ENSG00000254404.1 | -0.871686298 | -1.967296348 | 0.049149056 |

| | | | | | |
|---|---|---|---|---|---|
| OR9K2 | ENST00000531977.1 | ENSG00000224023.6 | 0.882491871 | 1.960243696 | 0.049967313 |
| OR9K2 | ENST00000535914.1 | ENSG00000256894.1 | 0.868708024 | 1.965767342 | 0.049325492 |
| OR9K2 | ENST00000536529.1 | ENSG00000256422.1 | 0.833612461 | 1.809697348 | 0.070342734 |
| OR9K2 | ENST00000538641.1 | ENSG00000256422.1 | 0.92987132 | 2.070287709 | 0.03842541 |
| OR9K2 | ENST00000541391.1 | ENSG00000256268.1 | 0.924757966 | 2.077412631 | 0.037763491 |
| OR9K2 | ENST00000546135.1 | ENSG00000256670.1 | -0.89689237 | -2.014896725 | 0.043915471 |
| OR9K2 | ENST00000548210.1 | ENSG00000257784.1 | -0.881014202 | -1.952765639 | 0.050847372 |
| OR9K2 | ENST00000548748.1 | ENSG00000258252.1 | -0.808879213 | -1.826609191 | 0.067758551 |
| OR9K2 | ENST00000549329.1 | ENSG00000224189.2 | 0.830009507 | 1.855052906 | 0.063588672 |
| OR9K2 | ENST00000549616.1 | ENSG00000258168.1 | 0.942179849 | 2.124739912 | 0.033608321 |
| OR9K2 | ENST00000550805.1 | ENSG00000244306.5 | -0.959799017 | -2.160449553 | 0.030737885 |
| OR9K2 | ENST00000551067.1 | ENSG00000257891.1 | -0.918328459 | -2.048159993 | 0.040544325 |
| OR9K2 | ENST00000551135.1 | ENSG00000258294.1 | 0.814778681 | 1.826249943 | 0.067812622 |
| OR9K2 | ENST00000552261.1 | ENSG00000257959.1 | 0.821681713 | 1.842704554 | 0.065372159 |
| OR9K2 | ENST00000552541.1 | ENSG00000258294.1 | 0.880655936 | 1.968403617 | 0.049021616 |
| OR9K2 | ENST00000552558.1 | ENSG00000257947.1 | 0.809933187 | 1.809252794 | 0.070411739 |
| OR9K2 | ENST00000552634.1 | ENSG00000257496.1 | -0.887058418 | -1.972866755 | 0.048510745 |
| OR9K2 | ENST00000553477.1 | ENSG00000259123.1 | 0.935848005 | 2.103084953 | 0.035458344 |
| OR9K2 | ENST00000553537.1 | ENSG00000258481.1 | -0.912888162 | -2.034068465 | 0.041944693 |
| OR9K2 | ENST00000553954.1 | ENSG00000259052.1 | -0.858385064 | -1.905946581 | 0.056657137 |
| OR9K2 | ENST00000555460.1 | ENSG00000259042.1 | -0.835742956 | -1.849637046 | 0.06436588 |
| OR9K2 | ENST00000555636.1 | ENSG00000259072.1 | 0.895127867 | 1.995310896 | 0.046008982 |
| OR9K2 | ENST00000555913.1 | ENSG00000259077.1 | 0.858479445 | 1.898455812 | 0.057636063 |
| OR9K2 | ENST00000556458.1 | ENSG00000258504.2 | -0.920965401 | -2.056320294 | 0.039751653 |
| OR9K2 | ENST00000558221.1 | ENSG00000259704.1 | -0.872423121 | -1.948855876 | 0.051312639 |
| OR9K2 | ENST00000560969.1 | ENSG00000259176.1 | 0.895546658 | 2.000792706 | 0.045414734 |
| OR9K2 | ENST00000561254.1 | ENSG00000259554.1 | 0.827147136 | 1.854662893 | 0.063644381 |
| OR9K2 | ENST00000563342.1 | ENSG00000259914.1 | 0.87347504 | 1.95999146 | 0.049996788 |
| OR9K2 | ENST00000563855.1 | ENSG00000260658.1 | 0.934680098 | 2.085574568 | 0.037017177 |
| OR9K2 | ENST00000565271.1 | ENSG00000261335.1 | -0.870388398 | -1.936311108 | 0.052829609 |
| OR9K2 | ENST00000565310.1 | ENSG00000261118.1 | -0.868283033 | -1.915169393 | 0.055470904 |
| OR9K2 | ENST00000565722.1 | ENSG00000245768.2 | 0.814694603 | 1.829006559 | 0.067398626 |
| OR9K2 | ENST00000565735.1 | ENSG00000261213.1 | 0.885728098 | 1.97348106 | 0.04844078 |
| OR9K2 | ENST00000568410.1 | ENSG00000260277.1 | 0.861276705 | 1.914328397 | 0.055578208 |
| OR9K2 | ENST00000569778.1 | ENSG00000260823.1 | -0.851686996 | -1.899542949 | 0.057493125 |
| OR9K2 | ENST00000569849.1 | ENSG00000260640.1 | 0.892643184 | 2.01156271 | 0.044266054 |
| OR9K2 | ENST00000570700.1 | ENSG00000263011.1 | -0.813847734 | -1.811908188 | 0.070000384 |
| OR9K2 | ENST00000572417.1 | ENSG00000263171.1 | 0.833343868 | 1.852580732 | 0.063942476 |
| OR9K2 | ENST00000573260.1 | ENSG00000262482.1 | 0.935749354 | 2.089342128 | 0.036676937 |
| OR9K2 | ENST00000576021.1 | ENSG00000262413.1 | -0.867734315 | -1.921384246 | 0.054683283 |
| OR9K2 | ENST00000577360.1 | ENSG00000264273.1 | 0.822419268 | 1.857577833 | 0.063228989 |
| OR9K2 | ENST00000577853.1 | ENSG00000264207.1 | -0.80433948 | -1.799651511 | 0.071915682 |
| OR9K2 | ENST00000579775.1 | ENSG00000264108.1 | -0.848023996 | -1.89871367 | 0.057602133 |
| OR9K2 | ENST00000581996.1 | ENSG00000265778.1 | 0.914181163 | 2.028032593 | 0.042556922 |
| OR9K2 | ENST00000585684.1 | ENSG00000267057.1 | 0.905229413 | 2.019635833 | 0.043421175 |
| OR9K2 | ENST00000586297.1 | ENSG00000267633.1 | 0.816833917 | 1.808219495 | 0.070572346 |
| OR9K2 | ENST00000588177.1 | ENSG00000234899.5 | 0.877619502 | 1.935859104 | 0.05288496 |
| OR9K2 | ENST00000588842.1 | ENSG00000235779.3 | 0.930360407 | 2.100113389 | 0.035718868 |
| OR9K2 | ENST00000591469.1 | ENSG00000267374.1 | 0.970436307 | 2.180567584 | 0.029215415 |
| OR9K2 | ENST00000591621.1 | ENSG00000232116.2 | 0.960314397 | 2.136092256 | 0.032671895 |
| OR9K2 | ENST00000592022.1 | ENSG00000267383.2 | 0.84494754 | 1.895169969 | 0.058069887 |

| | | | | | |
|---|---|---|---|---|---|
| OR9K2 | ENST00000592045.1 | ENSG00000267057.1 | 0.829422489 | 1.832031543 | 0.066946721 |
| OR9K2 | ENST00000592431.1 | ENSG00000267475.1 | -0.918096904 | -2.042021472 | 0.041149401 |
| OR9K2 | ENST00000592523.1 | ENSG00000226994.3 | 0.820535157 | 1.834760946 | 0.066541118 |
| OR9K2 | ENST00000593175.1 | ENSG00000229036.3 | 0.893757135 | 1.99337502 | 0.046220397 |
| OR9K2 | ENST00000593486.1 | ENSG00000250910.3 | 0.844273454 | 1.898250477 | 0.057663094 |
| OR9K2 | ENST00000593599.1 | ENSG00000231898.4 | 0.829436863 | 1.854971109 | 0.063600352 |
| OR9K2 | ENST00000594589.1 | ENSG00000269321.1 | -0.903606114 | -2.012333421 | 0.044184802 |
| OR9K2 | ENST00000596567.1 | ENSG00000226647.2 | 0.900691264 | 1.995978381 | 0.045936276 |
| OR9K2 | ENST00000597680.1 | ENSG00000269574.1 | 0.817256703 | 1.811041524 | 0.070134424 |
| OR9K2 | ENST00000597755.1 | ENSG00000236194.2 | 0.920713412 | 2.079145529 | 0.037603976 |
| OR9K2 | ENST00000598065.1 | ENSG00000231731.3 | -0.978418077 | -2.183201783 | 0.029020954 |
| OR9K2 | ENST00000598887.1 | ENSG00000268475.1 | -0.843935841 | -1.872457004 | 0.061143405 |
| OR9K2 | ENST00000598950.1 | ENSG00000269736.1 | 0.811639502 | 1.847609924 | 0.064658793 |
| OR9K2 | ENST00000599050.1 | ENSG00000268366.1 | 0.906323331 | 2.029256799 | 0.042432141 |
| OR9K2 | ENST00000600365.1 | ENSG00000231898.4 | 0.803225225 | 1.794168899 | 0.072786215 |
| OR9K2 | ENST00000602443.1 | ENSG00000270076.1 | 0.860158894 | 1.90938999 | 0.056211801 |
| OR9K2 | ENST00000602835.1 | ENSG00000270096.1 | 0.838523649 | 1.875997189 | 0.060655666 |
| OR9K2 | ENST00000602881.1 | ENSG00000269965.1 | 0.914999643 | 2.033794296 | 0.04197234 |
| OR9K2 | ENST00000604312.1 | ENSG00000270947.1 | 0.867879898 | 1.946813648 | 0.05155708 |
| OR9K2 | ENST00000605692.1 | ENSG00000270810.1 | -0.829658579 | -1.888796533 | 0.058919098 |
| OR9K2 | ENST00000606869.1 | ENSG00000272349.1 | 0.853561313 | 1.906568677 | 0.056576464 |
| OR9K2 | ENST00000606898.1 | ENSG00000272094.1 | -0.895939267 | -1.982466242 | 0.047427082 |
| OR9K2 | ENST00000607715.1 | ENSG00000271788.1 | 0.844696284 | 1.862929551 | 0.062472179 |
| OR9K2 | ENST00000608133.1 | ENSG00000273193.1 | 0.931075158 | 2.080719478 | 0.03745959 |
| OR9K2 | ENST00000608289.1 | ENSG00000272958.1 | -0.832828753 | -1.877680606 | 0.060424871 |
| OR9K2 | ENST00000609349.1 | ENSG00000272861.1 | 0.819360592 | 1.849225649 | 0.064425237 |
| OR9K2 | ENST00000609807.1 | ENSG00000272700.1 | 0.839536149 | 1.879644775 | 0.060156507 |
| OR9K2 | NR_027067.1 | LINC00114 | -0.858607797 | -1.941671192 | 0.052176918 |
| OR9K2 | NR_027402.1 | FAM223B | -0.956699493 | -2.150291474 | 0.031532166 |
| OR9K2 | NR_027425.1 | FAM66D | -0.941202281 | -2.101127398 | 0.035629784 |
| OR9K2 | NR_027440.1 | LOC100272217 | -0.929537517 | -2.093301603 | 0.03632224 |
| OR9K2 | NR_033914.1 | LINC00254 | 0.905107228 | 2.006420133 | 0.044811442 |
| OR9K2 | NR_038194.1 | LINC00583 | 0.847518962 | 1.88308491 | 0.059688862 |
| OR9K2 | NR_046748.1 | ARHGAP31-AS1 | 0.922355748 | 2.099264664 | 0.035793577 |
| OR9K2 | NR_046766.1 | ATP2B2-IT2 | 0.846937739 | 1.898168339 | 0.05767391 |
| OR9K2 | NR_046845.1 | DNM3-IT1 | 0.87407489 | 1.948308972 | 0.051378004 |
| OR9K2 | NR_046871.1 | LINC00333 | 0.803609806 | 1.811345701 | 0.070087355 |
| OR9K2 | NR_047698.1 | VWC2L-IT1 | 0.881914696 | 1.95020216 | 0.051152028 |
| OR9K2 | NR_102703.1 | MAGEA8-AS1 | 0.829723659 | 1.84185448 | 0.065496438 |
| OR9K2 | NR_104618.1 | LINC01017 | 0.840153999 | 1.877388621 | 0.06046485 |
| OR9K2 | NR_109877.1 | LINC01470 | 0.920263625 | 2.059893527 | 0.039408721 |
| OR9K2 | NR_110117.1 | LOC101927769 | 0.812291877 | 1.803021473 | 0.071384842 |
| OR9K2 | NR_110123.1 | GRM7-AS3 | 0.800985522 | 1.802750606 | 0.071427391 |
| OR9K2 | NR_110504.1 | LOC101929572 | -0.818005225 | -1.798801128 | 0.072050145 |
| OR9K2 | NR_110556.1 | LOC102724890 | 0.814498164 | 1.835330108 | 0.066456793 |
| OR9K2 | NR_110731.1 | LINC01232 | -0.852754377 | -1.907602903 | 0.05644256 |
| OR9K2 | NR_110824.1 | LINC01986 | 0.853505759 | 1.882323425 | 0.059792116 |
| OR9K2 | NR_120330.1 | LOC101928227 | -0.802048903 | -1.790635071 | 0.073351875 |
| OR9K2 | NR_120466.1 | LINC01489 | 0.92734198 | 2.050102748 | 0.040354405 |
| OR9K2 | NR_125769.1 | LINC01269 | -0.904273715 | -2.029317162 | 0.042425996 |
| OR9K2 | NR_126409.1 | LINC00376 | 0.820813168 | 1.858629007 | 0.063079743 |

| | | | | | |
|---|---|---|---|---|---|
| OR9K2 | NR_130916.1 | LOC105274304 | -0.922460162 | -2.061153969 | 0.039288353 |
| OR9K2 | NR_131204.1 | XACT | -0.810328926 | -1.820065897 | 0.06874897 |
| OR9K2 | NR_131243.1 | SMCR2 | -0.8283223 | -1.856162165 | 0.063430447 |
| OR9K2 | NR_134610.1 | LOC105375014 | -0.89968408 | -2.000410089 | 0.045456 |
| OR9K2 | NR_134632.1 | LOC105373051 | -0.980028008 | -2.188148303 | 0.028658801 |
| OR9K2 | NR_135040.1 | LOC101927038 | -0.899307105 | -2.034471562 | 0.041904073 |
| OR9K2 | NR_135549.1 | LOC101929411 | 0.954833719 | 2.103111758 | 0.035456001 |
| OR9K2 | NR_136218.1 | MEF2C-AS1 | 0.981979093 | 2.194615596 | 0.028191184 |
| PANO1 | ENST00000318291.4 | ENSG00000177406.4 | 0.812433317 | 1.813915711 | 0.069690704 |
| PANO1 | ENST00000398777.3 | ENSG00000240152.2 | 0.807633264 | 1.819958084 | 0.068765388 |
| PANO1 | ENST00000412809.1 | ENSG00000229938.1 | -0.814846123 | -1.827273835 | 0.067658607 |
| PANO1 | ENST00000413353.1 | ENSG00000232893.1 | 0.814390347 | 1.802262943 | 0.071504046 |
| PANO1 | ENST00000416220.1 | ENSG00000236753.1 | 0.808328498 | 1.808674728 | 0.070501551 |
| PANO1 | ENST00000421207.1 | ENSG00000231768.1 | 0.830196189 | 1.857410454 | 0.06325278 |
| PANO1 | ENST00000423667.1 | ENSG00000225970.1 | 0.966115736 | 2.178887247 | 0.029340045 |
| PANO1 | ENST00000424735.1 | ENSG00000237457.2 | -0.8693755 | -1.957132196 | 0.050331928 |
| PANO1 | ENST00000425624.1 | ENSG00000223779.4 | 0.97630048 | 2.205023429 | 0.02745245 |
| PANO1 | ENST00000426699.1 | ENSG00000229308.1 | 0.989386301 | 2.258239868 | 0.023930709 |
| PANO1 | ENST00000433344.1 | ENSG00000234083.1 | -0.974212019 | -2.16529472 | 0.030365122 |
| PANO1 | ENST00000433614.1 | ENSG00000228534.1 | -0.854482086 | -1.907297412 | 0.056482085 |
| PANO1 | ENST00000435434.1 | ENSG00000231233.1 | 0.889060205 | 1.965472259 | 0.049359603 |
| PANO1 | ENST00000435992.2 | ENSG00000232675.3 | 0.878461062 | 1.961854456 | 0.049779432 |
| PANO1 | ENST00000436982.2 | ENSG00000235335.2 | -0.897668637 | -1.991682392 | 0.046405917 |
| PANO1 | ENST00000438107.1 | ENSG00000234449.2 | 0.822149707 | 1.875493461 | 0.060724868 |
| PANO1 | ENST00000439186.1 | ENSG00000237076.1 | 0.947525345 | 2.098664664 | 0.035846472 |
| PANO1 | ENST00000440492.1 | ENSG00000233975.1 | 0.821319619 | 1.832538303 | 0.066871261 |
| PANO1 | ENST00000444245.1 | ENSG00000236753.1 | 0.863880863 | 1.941453941 | 0.052203241 |
| PANO1 | ENST00000447514.1 | ENSG00000236753.1 | 0.875602905 | 1.957949997 | 0.05023588 |
| PANO1 | ENST00000450848.1 | ENSG00000225539.1 | 0.881254261 | 1.974574565 | 0.048316448 |
| PANO1 | ENST00000455238.1 | ENSG00000231413.1 | 0.834462078 | 1.866071492 | 0.062031364 |
| PANO1 | ENST00000455699.1 | ENSG00000240996.1 | 0.8735681 | 1.973906572 | 0.048392367 |
| PANO1 | ENST00000457169.1 | ENSG00000232408.1 | 0.821257612 | 1.851306651 | 0.06412545 |
| PANO1 | ENST00000458364.1 | ENSG00000225655.1 | -0.800925125 | -1.744351364 | 0.08109784 |
| PANO1 | ENST00000458661.2 | ENSG00000236467.3 | 0.822882561 | 1.868639915 | 0.061672929 |
| PANO1 | ENST00000468444.2 | ENSG00000258525.1 | -0.819609995 | -1.815018881 | 0.069521008 |
| PANO1 | ENST00000484413.1 | ENSG00000271853.1 | 0.880169672 | 1.957558003 | 0.050281899 |
| PANO1 | ENST00000503938.1 | ENSG00000246095.2 | 0.817038786 | 1.810982753 | 0.070143521 |
| PANO1 | ENST00000504344.1 | ENSG00000251438.1 | 0.805212092 | 1.819846251 | 0.068782422 |
| PANO1 | ENST00000504795.1 | ENSG00000250723.1 | 0.812348901 | 1.833621849 | 0.066710147 |
| PANO1 | ENST00000504891.1 | ENSG00000249388.1 | 0.918108732 | 2.072047305 | 0.03826103 |
| PANO1 | ENST00000505498.1 | ENSG00000250908.1 | 0.929861794 | 2.09710992 | 0.035983846 |
| PANO1 | ENST00000507373.1 | ENSG00000250072.1 | 0.80559724 | 1.809068059 | 0.070440431 |
| PANO1 | ENST00000508083.1 | ENSG00000249343.1 | 0.821135778 | 1.810202052 | 0.070264459 |
| PANO1 | ENST00000517716.1 | ENSG00000253515.1 | -0.808979114 | -1.80668916 | 0.070810759 |
| PANO1 | ENST00000518473.1 | ENSG00000253985.1 | 0.918508034 | 2.016624276 | 0.043734738 |
| PANO1 | ENST00000521307.1 | ENSG00000253177.1 | 0.895534143 | 2.038774953 | 0.041472493 |
| PANO1 | ENST00000521953.1 | ENSG00000253214.1 | 0.89003754 | 1.976032885 | 0.048151053 |
| PANO1 | ENST00000522704.1 | ENSG00000254135.1 | 0.908725873 | 2.016110931 | 0.043788377 |
| PANO1 | ENST00000524818.1 | ENSG00000254473.1 | 0.873882155 | 1.941111677 | 0.052244733 |
| PANO1 | ENST00000525133.1 | ENSG00000255375.1 | 0.82743728 | 1.833330046 | 0.066753504 |
| PANO1 | ENST00000526186.1 | ENSG00000254510.1 | 0.826741642 | 1.866475747 | 0.061974834 |

| | | | | | |
|---|---|---|---|---|---|
| PANO1 | ENST00000526935.1 | ENSG00000255372.1 | 0.859562983 | 1.948030381 | 0.051411328 |
| PANO1 | ENST00000528887.1 | ENSG00000254501.1 | 0.872600329 | 1.935523344 | 0.052926107 |
| PANO1 | ENST00000529837.1 | ENSG00000254687.1 | 0.846004822 | 1.886570401 | 0.059218131 |
| PANO1 | ENST00000547834.1 | ENSG00000258325.1 | 0.950300347 | 2.126741645 | 0.033441555 |
| PANO1 | ENST00000549140.1 | ENSG00000258332.1 | 0.838134235 | 1.867215244 | 0.061871536 |
| PANO1 | ENST00000552469.1 | ENSG00000258325.1 | 0.837123284 | 1.853806637 | 0.063766829 |
| PANO1 | ENST00000554679.1 | ENSG00000258837.1 | 0.872993535 | 1.939204139 | 0.052476486 |
| PANO1 | ENST00000555966.1 | ENSG00000258843.1 | 0.80012753 | 1.776989371 | 0.075569989 |
| PANO1 | ENST00000556786.1 | ENSG00000258525.1 | -0.9497467 | -2.123155043 | 0.033740862 |
| PANO1 | ENST00000559003.1 | ENSG00000259520.1 | 0.888631978 | 1.931694795 | 0.053397188 |
| PANO1 | ENST00000563841.1 | ENSG00000261029.1 | 0.916782833 | 2.055028677 | 0.039876235 |
| PANO1 | ENST00000564038.1 | ENSG00000261760.2 | 0.851509698 | 1.887285351 | 0.059121956 |
| PANO1 | ENST00000565965.1 | ENSG00000261172.1 | 0.915655422 | 2.039714643 | 0.041378756 |
| PANO1 | ENST00000567395.1 | ENSG00000261090.1 | 0.833125295 | 1.84524085 | 0.065002511 |
| PANO1 | ENST00000569981.1 | ENSG00000238045.5 | 0.817237657 | 1.839918109 | 0.065780261 |
| PANO1 | ENST00000570493.2 | ENSG00000261898.2 | 0.804790441 | 1.824154344 | 0.068128742 |
| PANO1 | ENST00000570512.1 | ENSG00000262768.1 | 0.89402491 | 1.998912944 | 0.045617774 |
| PANO1 | ENST00000576554.1 | ENSG00000262413.1 | 0.87462439 | 1.938697173 | 0.052538223 |
| PANO1 | ENST00000577698.1 | ENSG00000265100.1 | 0.918032712 | 2.042987957 | 0.04105363 |
| PANO1 | ENST00000578265.1 | ENSG00000214719.7 | 0.800152102 | 1.802886331 | 0.071406068 |
| PANO1 | ENST00000584705.1 | ENSG00000264569.1 | 0.836609634 | 1.872313542 | 0.061163238 |
| PANO1 | ENST00000585559.1 | ENSG00000267117.1 | 0.901896629 | 2.01893531 | 0.043493944 |
| PANO1 | ENST00000588799.1 | ENSG00000267275.1 | 0.95775408 | 2.156805556 | 0.031020818 |
| PANO1 | ENST00000588945.1 | ENSG00000267275.1 | 0.936902704 | 2.12110598 | 0.033912885 |
| PANO1 | ENST00000592400.1 | ENSG00000267735.1 | 0.858155106 | 1.925508805 | 0.054165736 |
| PANO1 | ENST00000597530.1 | ENSG00000228401.3 | 0.800571658 | 1.787930256 | 0.073787261 |
| PANO1 | ENST00000599259.1 | ENSG00000269352.1 | 0.826004454 | 1.838370655 | 0.066007806 |
| PANO1 | ENST00000600489.1 | ENSG00000231898.4 | 0.934534257 | 2.095714037 | 0.036107566 |
| PANO1 | ENST00000601692.1 | ENSG00000267874.1 | -0.971263259 | -2.176183141 | 0.029541569 |
| PANO1 | ENST00000602592.1 | ENSG00000270049.1 | 0.82794413 | 1.850901136 | 0.064183777 |
| PANO1 | ENST00000602872.1 | ENSG00000270067.1 | 0.873369479 | 1.969042202 | 0.048948245 |
| PANO1 | ENST00000602954.1 | ENSG00000269906.1 | 0.854378842 | 1.893093636 | 0.058345418 |
| PANO1 | ENST00000606457.1 | ENSG00000271830.1 | 0.811541943 | 1.823023765 | 0.068299792 |
| PANO1 | ENST00000606470.1 | ENSG00000271913.1 | 0.815447428 | 1.821352302 | 0.068553321 |
| PANO1 | ENST00000607284.1 | ENSG00000272389.1 | 0.964722463 | 2.155753414 | 0.031102925 |
| PANO1 | ENST00000607839.1 | ENSG00000272030.1 | 0.924775481 | 2.073832671 | 0.038094854 |
| PANO1 | ENST00000608476.1 | ENSG00000232675.3 | 0.812131451 | 1.842603367 | 0.065386942 |
| PANO1 | ENST00000609610.1 | ENSG00000232675.3 | 0.810487733 | 1.803309727 | 0.071339586 |
| PANO1 | ENST00000609701.1 | ENSG00000273284.1 | 0.873796538 | 1.965258913 | 0.049384278 |
| PANO1 | ENST00000609725.1 | ENSG00000231898.4 | 0.82704019 | 1.864480474 | 0.062254261 |
| PANO1 | ENST00000609972.1 | ENSG00000230651.3 | 0.971718539 | 2.17129474 | 0.0299089 |
| PANO1 | ENST00000610145.1 | ENSG00000273175.1 | 0.851272521 | 1.876883231 | 0.0605341 |
| PANO1 | ENST00000610161.1 | ENSG00000273059.1 | 0.943999 | 2.072168992 | 0.038249684 |
| PANO1 | NR_003605.1 | ZFAS1 | 0.826839559 | 1.853714781 | 0.063779976 |
| PANO1 | NR_044996.1 | HCG23 | 0.986330341 | 2.218140705 | 0.02654524 |
| PANO1 | NR_108036.1 | CFAP58-AS1 | 0.883526161 | 1.965033005 | 0.049410418 |
| PANO1 | NR_120335.1 | LOC101928414 | 0.934075682 | 2.094657606 | 0.03620144 |
| PANO1 | NR_121189.1 | PGM5P3-AS1 | -0.813693536 | -1.808212038 | 0.070573506 |
| PANO1 | NR_126166.1 | FAM74A7 | 0.9794696 | 2.21697958 | 0.026624487 |
| PANO1 | NR_134597.1 | LOC105378068 | 0.881836076 | 1.995839882 | 0.045951354 |
| PANO1 | NR_135258.1 | LOC105370489 | 0.880862643 | 1.968925676 | 0.048961627 |

| | | | | | |
|---|---|---|---|---|---|
| PFDN5 | ENST00000340585.6 | ENSG00000249429.1 | -0.800272351 | -1.776339898 | 0.075676911 |
| PFDN5 | ENST00000412085.1 | ENSG00000233825.1 | 0.969756011 | 2.201432747 | 0.027705403 |
| PFDN5 | ENST00000412759.1 | ENSG00000236933.1 | 0.929367316 | 2.033971619 | 0.041954457 |
| PFDN5 | ENST00000412772.1 | ENSG00000231507.1 | 0.920933485 | 2.067189697 | 0.038716283 |
| PFDN5 | ENST00000414740.2 | ENSG00000229646.2 | 0.944526268 | 2.084819547 | 0.037085684 |
| PFDN5 | ENST00000419734.1 | ENSG00000234646.1 | -0.825963751 | -1.860114357 | 0.062869348 |
| PFDN5 | ENST00000421617.1 | ENSG00000237342.1 | 0.820477665 | 1.829525158 | 0.067320974 |
| PFDN5 | ENST00000426519.1 | ENSG00000234142.1 | 0.861550951 | 1.922618909 | 0.054527928 |
| PFDN5 | ENST00000428765.1 | ENSG00000230107.1 | 0.846427086 | 1.90831667 | 0.0563503 |
| PFDN5 | ENST00000429608.1 | ENSG00000237480.1 | 0.817125139 | 1.827836494 | 0.067574094 |
| PFDN5 | ENST00000433036.1 | ENSG00000228989.1 | 0.823636024 | 1.842061794 | 0.065466111 |
| PFDN5 | ENST00000433876.2 | ENSG00000228423.2 | 0.972938786 | 2.116807008 | 0.03427623 |
| PFDN5 | ENST00000433905.2 | ENSG00000229299.2 | 0.889652657 | 1.981517164 | 0.047533307 |
| PFDN5 | ENST00000434627.1 | ENSG00000230074.1 | 0.895764078 | 1.990638258 | 0.046520671 |
| PFDN5 | ENST00000435892.1 | ENSG00000233635.2 | 0.905768667 | 1.986168682 | 0.047014597 |
| PFDN5 | ENST00000438190.1 | ENSG00000227214.2 | 0.815953154 | 1.801262495 | 0.071661516 |
| PFDN5 | ENST00000438488.1 | ENSG00000223812.1 | 0.8730692 | 1.930737627 | 0.053515507 |
| PFDN5 | ENST00000440714.1 | ENSG00000237609.1 | 0.842160934 | 1.877245601 | 0.06048444 |
| PFDN5 | ENST00000442850.1 | ENSG00000232600.2 | -0.932176576 | -2.073450441 | 0.038130379 |
| PFDN5 | ENST00000443380.1 | ENSG00000224371.1 | 0.822181894 | 1.871529315 | 0.061271751 |
| PFDN5 | ENST00000447206.1 | ENSG00000230839.1 | 0.873569649 | 1.966768618 | 0.049209892 |
| PFDN5 | ENST00000447343.2 | ENSG00000229299.2 | 0.91396178 | 2.074870508 | 0.037998538 |
| PFDN5 | ENST00000449463.1 | ENSG00000230309.1 | -0.865676505 | -1.93077037 | 0.053511456 |
| PFDN5 | ENST00000453051.1 | ENSG00000229407.1 | 0.946539476 | 2.120676907 | 0.033949001 |
| PFDN5 | ENST00000454530.1 | ENSG00000226649.1 | -0.912350236 | -2.047976343 | 0.040562317 |
| PFDN5 | ENST00000456715.1 | ENSG00000224893.1 | 0.804565726 | 1.779661953 | 0.075131301 |
| PFDN5 | ENST00000458194.1 | ENSG00000226193.1 | 0.888382774 | 1.997546832 | 0.045765812 |
| PFDN5 | ENST00000458443.1 | ENSG00000238232.1 | 0.882857825 | 1.965732253 | 0.049329547 |
| PFDN5 | ENST00000468165.1 | ENSG00000239480.1 | 0.868344319 | 1.956439627 | 0.050413388 |
| PFDN5 | ENST00000489557.2 | ENSG00000257045.1 | 0.839792613 | 1.88368029 | 0.059608234 |
| PFDN5 | ENST00000490013.1 | ENSG00000184115.12 | 0.980004992 | 2.24499018 | 0.024768763 |
| PFDN5 | ENST00000498358.1 | ENSG00000184115.12 | 0.916950406 | 2.056376625 | 0.039746228 |
| PFDN5 | ENST00000498693.1 | ENSG00000244198.1 | 0.848790043 | 1.917016386 | 0.05523585 |
| PFDN5 | ENST00000503034.1 | ENSG00000248936.1 | 0.922457897 | 2.060194632 | 0.039379938 |
| PFDN5 | ENST00000505196.1 | ENSG00000248131.1 | 0.831224234 | 1.885602592 | 0.059348528 |
| PFDN5 | ENST00000505556.1 | ENSG00000249409.1 | 0.823723616 | 1.833765443 | 0.06668882 |
| PFDN5 | ENST00000506100.1 | ENSG00000249409.1 | 0.830180233 | 1.874461385 | 0.06086686 |
| PFDN5 | ENST00000508188.1 | ENSG00000250999.1 | 0.824321513 | 1.860771267 | 0.062776485 |
| PFDN5 | ENST00000514411.1 | ENSG00000250882.1 | 0.943750481 | 2.129763492 | 0.033191144 |
| PFDN5 | ENST00000514877.1 | ENSG00000248685.1 | 0.925569844 | 2.064644891 | 0.038956612 |
| PFDN5 | ENST00000520838.1 | ENSG00000253404.1 | 0.834830781 | 1.841967716 | 0.065479872 |
| PFDN5 | ENST00000521207.1 | ENSG00000253716.1 | 0.83250483 | 1.899853655 | 0.057452327 |
| PFDN5 | ENST00000522547.1 | ENSG00000253430.1 | -0.917205848 | -2.055602354 | 0.039820861 |
| PFDN5 | ENST00000524094.1 | ENSG00000248555.2 | -0.81079671 | -1.803235902 | 0.071351174 |
| PFDN5 | ENST00000524942.1 | ENSG00000255553.1 | 0.865952948 | 1.917445994 | 0.055181296 |
| PFDN5 | ENST00000528000.1 | ENSG00000254804.1 | 0.965494673 | 2.137009505 | 0.032597219 |
| PFDN5 | ENST00000528818.1 | ENSG00000232995.3 | 0.826765825 | 1.836954385 | 0.06621663 |
| PFDN5 | ENST00000531136.1 | ENSG00000255558.1 | -0.829642569 | -1.865448136 | 0.062118615 |
| PFDN5 | ENST00000543072.1 | ENSG00000256092.2 | -0.933457897 | -2.07526663 | 0.03796183 |
| PFDN5 | ENST00000543275.1 | ENSG00000256944.1 | 0.877108112 | 1.97649204 | 0.048099076 |
| PFDN5 | ENST00000554049.1 | ENSG00000258763.1 | 0.85994664 | 1.903778462 | 0.056939043 |

| | | | | | |
|---|---|---|---|---|---|
| PFDN5 | ENST00000554798.1 | ENSG00000258483.1 | 0.8821648 | 1.998711623 | 0.045639565 |
| PFDN5 | ENST00000558515.1 | ENSG00000259182.1 | 0.801639221 | 1.785551274 | 0.074171942 |
| PFDN5 | ENST00000558575.1 | ENSG00000259687.1 | 0.873070361 | 1.974244777 | 0.048353917 |
| PFDN5 | ENST00000561529.1 | ENSG00000260886.1 | 0.825810309 | 1.869512831 | 0.0615515 |
| PFDN5 | ENST00000563611.1 | ENSG00000261583.1 | 0.812740479 | 1.849010779 | 0.064456256 |
| PFDN5 | ENST00000565359.1 | ENSG00000260601.1 | 0.837334481 | 1.839340032 | 0.065865188 |
| PFDN5 | ENST00000565829.1 | ENSG00000260148.1 | 0.843184147 | 1.84527934 | 0.064996914 |
| PFDN5 | ENST00000569025.1 | ENSG00000246379.2 | -0.808446015 | -1.835368749 | 0.066451071 |
| PFDN5 | ENST00000569459.1 | ENSG00000261346.1 | 0.842729244 | 1.876808477 | 0.060544348 |
| PFDN5 | ENST00000570843.1 | ENSG00000261889.1 | 0.882520084 | 1.964449342 | 0.049478006 |
| PFDN5 | ENST00000570929.1 | ENSG00000262223.2 | 0.803632469 | 1.786436904 | 0.074028545 |
| PFDN5 | ENST00000574460.1 | ENSG00000263051.1 | 0.989812907 | 2.180666364 | 0.029208103 |
| PFDN5 | ENST00000578800.1 | ENSG00000264235.1 | 0.910781144 | 1.997112168 | 0.045812999 |
| PFDN5 | ENST00000578936.1 | ENSG00000265547.1 | 0.925274054 | 2.059194941 | 0.039475568 |
| PFDN5 | ENST00000581905.1 | ENSG00000264235.1 | 0.891686182 | 2.004976098 | 0.044965602 |
| PFDN5 | ENST00000583138.1 | ENSG00000263393.1 | 0.928913212 | 2.097871959 | 0.035916458 |
| PFDN5 | ENST00000585072.1 | ENSG00000263745.1 | 0.839119889 | 1.894496511 | 0.058159137 |
| PFDN5 | ENST00000586051.1 | ENSG00000267576.1 | 0.943854175 | 2.123384332 | 0.033721659 |
| PFDN5 | ENST00000586503.1 | ENSG00000267205.1 | -0.807297871 | -1.830636688 | 0.067154789 |
| PFDN5 | ENST00000587281.1 | ENSG00000228290.2 | 0.868325608 | 1.918395556 | 0.055060874 |
| PFDN5 | ENST00000588182.2 | ENSG00000267453.2 | 0.871932773 | 1.941160548 | 0.052238807 |
| PFDN5 | ENST00000588290.1 | ENSG00000267751.1 | 0.834979327 | 1.824385955 | 0.068093744 |
| PFDN5 | ENST00000588908.1 | ENSG00000267751.1 | 0.814775237 | 1.773498373 | 0.076146161 |
| PFDN5 | ENST00000589673.1 | ENSG00000267755.1 | 0.923503376 | 2.095940661 | 0.036087455 |
| PFDN5 | ENST00000592413.1 | ENSG00000266933.1 | 0.893523441 | 1.998554453 | 0.045656583 |
| PFDN5 | ENST00000593588.1 | ENSG00000269635.1 | 0.808530973 | 1.822912438 | 0.068316654 |
| PFDN5 | ENST00000594590.2 | ENSG00000268199.2 | 0.915965427 | 2.05607496 | 0.039775291 |
| PFDN5 | ENST00000595508.1 | ENSG00000269749.1 | 0.877168662 | 1.96562393 | 0.049342068 |
| PFDN5 | ENST00000595955.1 | ENSG00000268401.1 | 0.959641426 | 2.104948638 | 0.035295779 |
| PFDN5 | ENST00000597256.1 | ENSG00000267986.1 | 0.826492988 | 1.857386439 | 0.063256194 |
| PFDN5 | ENST00000599352.1 | ENSG00000240401.4 | -0.987123889 | -2.159237047 | 0.030831781 |
| PFDN5 | ENST00000600071.1 | ENSG00000269199.1 | 0.913585046 | 2.052968626 | 0.04007562 |
| PFDN5 | ENST00000600234.1 | ENSG00000268078.1 | 0.879226388 | 1.954581065 | 0.050632538 |
| PFDN5 | ENST00000600534.1 | ENSG00000267858.1 | 0.810474262 | 1.8134699 | 0.069759377 |
| PFDN5 | ENST00000600889.1 | ENSG00000232675.3 | 0.801565118 | 1.810224399 | 0.070260995 |
| PFDN5 | ENST00000601033.1 | ENSG00000268401.1 | 0.920437668 | 2.072131702 | 0.038253161 |
| PFDN5 | ENST00000601752.1 | ENSG00000268051.1 | 0.814872236 | 1.830494708 | 0.067175997 |
| PFDN5 | ENST00000602532.1 | ENSG00000270091.1 | 0.943098448 | 2.156178015 | 0.031069768 |
| PFDN5 | ENST00000606068.1 | ENSG00000272342.1 | 0.878071739 | 1.959235912 | 0.050085165 |
| PFDN5 | ENST00000606277.1 | ENSG00000272145.1 | 0.815139146 | 1.834540839 | 0.066573751 |
| PFDN5 | ENST00000606778.1 | ENSG00000271930.1 | 0.835769588 | 1.898605315 | 0.057616389 |
| PFDN5 | ENST00000607052.1 | ENSG00000271870.1 | -0.967703579 | -2.186883945 | 0.028750997 |
| PFDN5 | ENST00000607224.1 | ENSG00000272521.1 | 0.933736882 | 2.087797641 | 0.036816092 |
| PFDN5 | ENST00000607476.1 | ENSG00000272540.1 | 0.821840163 | 1.835418761 | 0.066443666 |
| PFDN5 | ENST00000608677.1 | ENSG00000273350.1 | 0.901715637 | 1.980872402 | 0.047605585 |
| PFDN5 | ENST00000608940.1 | ENSG00000272763.1 | 0.872342715 | 1.960376001 | 0.049951859 |
| PFDN5 | ENST00000609146.1 | ENSG00000272851.1 | -0.877120487 | -1.952127535 | 0.050923065 |
| PFDN5 | ENST00000609281.1 | ENSG00000273320.1 | 0.87864479 | 1.977201955 | 0.048018807 |
| PFDN5 | NR_003604.2 | ZFAS1 | 0.851471035 | 1.920200911 | 0.054832527 |
| PFDN5 | NR_003606.2 | ZFAS1 | 0.86832118 | 1.940593424 | 0.052307612 |
| PFDN5 | NR_024321.1 | LINC00115 | 0.823334773 | 1.840696437 | 0.065666056 |

| | | | | | |
|---|---|---|---|---|---|
| PFDN5 | NR_026802.1 | FAM74A4 | 0.803127527 | 1.808371653 | 0.070548677 |
| PFDN5 | NR_027052.1 | THAP7-AS1 | 0.860352506 | 1.931892607 | 0.053372762 |
| PFDN5 | NR_027271.1 | CIRBP-AS1 | 0.876820141 | 1.956163684 | 0.050445875 |
| PFDN5 | NR_027334.2 | MZF1-AS1 | 0.802221641 | 1.813332706 | 0.069780522 |
| PFDN5 | NR_034037.1 | LINC00582 | -0.934487 | -2.104065013 | 0.035372776 |
| PFDN5 | NR_036658.1 | ZFAS1 | 0.859977778 | 1.917456833 | 0.05517992 |
| PFDN5 | NR_040096.1 | LOC643339 | 0.945838981 | 2.115765531 | 0.034364754 |
| PFDN5 | NR_046454.1 | LINC00907 | -0.890006392 | -1.992071683 | 0.046363193 |
| PFDN5 | NR_103851.1 | TAT-AS1 | 0.849226976 | 1.891027237 | 0.058620708 |
| PFDN5 | NR_110568.1 | LOC101927661 | 0.896577232 | 2.020401058 | 0.043341804 |
| PFDN5 | NR_110998.1 | FAM74A4 | 0.803127527 | 1.764691882 | 0.077615564 |
| PFDN5 | NR_111951.1 | LINC00869 | 0.808362512 | 1.807000585 | 0.070762188 |
| PFDN5 | NR_111952.1 | LINC00869 | 0.851478222 | 1.910269947 | 0.056098465 |
| PFDN5 | NR_111953.1 | LINC00869 | 0.807473715 | 1.799601738 | 0.071923547 |
| PFDN5 | NR_117097.1 | LINC01353 | 0.920933485 | 2.050280022 | 0.040337113 |
| PFDN5 | NR_117098.1 | LINC01353 | 0.917397337 | 2.059974971 | 0.039400934 |
| PFDN5 | NR_126380.1 | LINC01072 | 0.81873598 | 1.842571293 | 0.065391629 |
| PFDN5 | NR_130143.1 | LOC104968399 | 0.936845493 | 2.088089008 | 0.036789806 |
| PFDN5 | NR_130144.1 | LOC104968399 | 0.891686182 | 1.992099563 | 0.046360135 |
| PFDN5 | NR_134245.1 | LOC105379194 | 0.83901729 | 1.91181496 | 0.055899932 |
| PFDN5 | NR_135644.1 | LOC105371506 | -0.869473822 | -1.951918966 | 0.050947826 |
| PFDN5 | NR_138041.1 | LINC00384 | 0.898392586 | 2.015573508 | 0.043844592 |
| PGLS | ENST00000318291.4 | ENSG00000177406.4 | 0.832803528 | 1.861363291 | 0.062692891 |
| PGLS | ENST00000340585.6 | ENSG00000249429.1 | -0.804935385 | -1.814717169 | 0.069567385 |
| PGLS | ENST00000399186.2 | ENSG00000214888.2 | 0.808235776 | 1.823904893 | 0.068166452 |
| PGLS | ENST00000411694.1 | ENSG00000225331.1 | 0.910568414 | 2.05496773 | 0.039882122 |
| PGLS | ENST00000412085.1 | ENSG00000233825.1 | 0.917577418 | 2.076611633 | 0.037837418 |
| PGLS | ENST00000412759.1 | ENSG00000236933.1 | 0.930673628 | 2.07518569 | 0.037969328 |
| PGLS | ENST00000413887.1 | ENSG00000236948.1 | -0.828413318 | -1.868551164 | 0.061685286 |
| PGLS | ENST00000414740.2 | ENSG00000229646.2 | 0.895846497 | 1.9979762 | 0.04571924 |
| PGLS | ENST00000419662.1 | ENSG00000228265.1 | 0.94528058 | 2.12122398 | 0.033902958 |
| PGLS | ENST00000419734.1 | ENSG00000234646.1 | -0.827796417 | -1.857470421 | 0.063244256 |
| PGLS | ENST00000420315.1 | ENSG00000228072.1 | 0.900871102 | 2.021889178 | 0.043187802 |
| PGLS | ENST00000420465.1 | ENSG00000167355.3 | 0.808311222 | 1.791660582 | 0.073187352 |
| PGLS | ENST00000420981.2 | ENSG00000230438.5 | 0.923137581 | 2.073397491 | 0.038135302 |
| PGLS | ENST00000421020.1 | ENSG00000231407.1 | 0.932874439 | 2.07879992 | 0.037635744 |
| PGLS | ENST00000423869.1 | ENSG00000227848.1 | 0.856097902 | 1.921309154 | 0.054692744 |
| PGLS | ENST00000424181.1 | ENSG00000224977.1 | 0.848137319 | 1.895776021 | 0.057989668 |
| PGLS | ENST00000426475.1 | ENSG00000239467.1 | 0.811547222 | 1.814939231 | 0.069533249 |
| PGLS | ENST00000426519.1 | ENSG00000234142.1 | 0.945087543 | 2.137514036 | 0.032556205 |
| PGLS | ENST00000429630.1 | ENSG00000232533.1 | 0.824222189 | 1.832107292 | 0.066935437 |
| PGLS | ENST00000430920.1 | ENSG00000234203.1 | 0.815777116 | 1.832568051 | 0.066866833 |
| PGLS | ENST00000433036.1 | ENSG00000228989.1 | 0.861865114 | 1.934906794 | 0.053001734 |
| PGLS | ENST00000433051.1 | ENSG00000233193.1 | 0.810175112 | 1.808616809 | 0.070510555 |
| PGLS | ENST00000434627.1 | ENSG00000230074.1 | 0.895371141 | 2.016667711 | 0.043730202 |
| PGLS | ENST00000435892.1 | ENSG00000233635.2 | 0.844669357 | 1.876749099 | 0.06055249 |
| PGLS | ENST00000438190.1 | ENSG00000227214.2 | 0.896943808 | 2.003312309 | 0.045143777 |
| PGLS | ENST00000439184.1 | ENSG00000233985.1 | -0.932303526 | -2.077056562 | 0.037796339 |
| PGLS | ENST00000441592.2 | ENSG00000224078.8 | 0.827989718 | 1.862980732 | 0.062464977 |
| PGLS | ENST00000442649.1 | ENSG00000234089.1 | -0.811522566 | -1.830029465 | 0.067245533 |
| PGLS | ENST00000442850.1 | ENSG00000232600.2 | -0.800911301 | -1.795313431 | 0.072603776 |

| | | | | | |
|---|---|---|---|---|---|
| PGLS | ENST00000447206.1 | ENSG00000230839.1 | 0.806078941 | 1.801993611 | 0.071546411 |
| PGLS | ENST00000447343.2 | ENSG00000229299.2 | 0.824703016 | 1.847863665 | 0.064622068 |
| PGLS | ENST00000450063.1 | ENSG00000231210.2 | -0.847218434 | -1.864300532 | 0.062279512 |
| PGLS | ENST00000450072.1 | ENSG00000228486.5 | 0.821973962 | 1.838945541 | 0.065923197 |
| PGLS | ENST00000452176.1 | ENSG00000223659.1 | -0.874871669 | -1.959583403 | 0.050044503 |
| PGLS | ENST00000453051.1 | ENSG00000229407.1 | 0.944027523 | 2.123318925 | 0.033727136 |
| PGLS | ENST00000454530.1 | ENSG00000226649.1 | -0.86787048 | -1.958742364 | 0.050142966 |
| PGLS | ENST00000457115.1 | ENSG00000227245.1 | 0.816180983 | 1.837250071 | 0.066172987 |
| PGLS | ENST00000457253.1 | ENSG00000225173.1 | 0.990037238 | 2.219005323 | 0.026486362 |
| PGLS | ENST00000458154.1 | ENSG00000235578.1 | 0.910626823 | 2.027446447 | 0.042616776 |
| PGLS | ENST00000458194.1 | ENSG00000226193.1 | 0.942823187 | 2.106726758 | 0.035141271 |
| PGLS | ENST00000458364.1 | ENSG00000225655.1 | -0.840610298 | -1.886464328 | 0.059232411 |
| PGLS | ENST00000459985.1 | ENSG00000273066.1 | 0.821914555 | 1.840982689 | 0.065624096 |
| PGLS | ENST00000463255.1 | ENSG00000243305.1 | -0.903997497 | -1.994478252 | 0.046099814 |
| PGLS | ENST00000466431.2 | ENSG00000254485.1 | 0.900459612 | 2.016440926 | 0.04375389 |
| PGLS | ENST00000489077.1 | ENSG00000244198.1 | 0.8782852 | 1.968899229 | 0.048964664 |
| PGLS | ENST00000490013.1 | ENSG00000184115.12 | 0.831947762 | 1.8389306 | 0.065925394 |
| PGLS | ENST00000493123.1 | ENSG00000242428.1 | 0.826620925 | 1.848951405 | 0.06446483 |
| PGLS | ENST00000498693.1 | ENSG00000244198.1 | 0.906667541 | 2.060611365 | 0.039340132 |
| PGLS | ENST00000504916.1 | ENSG00000248112.1 | -0.860718431 | -1.96018567 | 0.049974093 |
| PGLS | ENST00000505196.1 | ENSG00000248131.1 | 0.821417856 | 1.822033677 | 0.068449876 |
| PGLS | ENST00000505556.1 | ENSG00000249409.1 | 0.886150246 | 1.981112829 | 0.047578622 |
| PGLS | ENST00000506100.1 | ENSG00000249409.1 | 0.871075404 | 1.941570104 | 0.052189165 |
| PGLS | ENST00000506791.1 | ENSG00000251131.1 | 0.893199985 | 2.001431966 | 0.045345859 |
| PGLS | ENST00000508083.1 | ENSG00000249343.1 | 0.850393533 | 1.875602833 | 0.060709837 |
| PGLS | ENST00000508188.1 | ENSG00000250999.1 | 0.879875899 | 1.940604056 | 0.052306322 |
| PGLS | ENST00000509036.1 | ENSG00000251131.1 | 0.973403065 | 2.176757138 | 0.029498693 |
| PGLS | ENST00000509192.1 | ENSG00000250765.1 | 0.810423801 | 1.817350974 | 0.069163389 |
| PGLS | ENST00000514877.1 | ENSG00000248685.1 | 0.834091743 | 1.84196497 | 0.065480274 |
| PGLS | ENST00000517300.1 | ENSG00000254144.2 | 0.926385269 | 2.069881973 | 0.038463399 |
| PGLS | ENST00000517846.1 | ENSG00000254485.1 | 0.902946933 | 2.027312953 | 0.042630418 |
| PGLS | ENST00000520603.1 | ENSG00000254001.1 | -0.859778485 | -1.889140696 | 0.058872979 |
| PGLS | ENST00000526154.1 | ENSG00000254511.1 | 0.874580857 | 1.941963346 | 0.052141538 |
| PGLS | ENST00000528000.1 | ENSG00000254804.1 | 0.831850305 | 1.865772995 | 0.062073132 |
| PGLS | ENST00000529247.1 | ENSG00000254741.1 | 0.817014277 | 1.839764019 | 0.06580289 |
| PGLS | ENST00000532688.1 | ENSG00000255441.1 | 0.858370423 | 1.92561263 | 0.054152761 |
| PGLS | ENST00000534065.1 | ENSG00000254458.1 | 0.8683994 | 1.943271496 | 0.051983364 |
| PGLS | ENST00000543072.1 | ENSG00000256092.2 | -0.868960873 | -1.963143232 | 0.049629533 |
| PGLS | ENST00000543275.1 | ENSG00000256944.1 | 0.870168417 | 1.938640868 | 0.052545084 |
| PGLS | ENST00000545177.3 | ENSG00000230438.5 | 0.991789903 | 2.213861186 | 0.026838329 |
| PGLS | ENST00000545254.1 | ENSG00000256633.1 | 0.901779068 | 2.000901116 | 0.045403047 |
| PGLS | ENST00000548722.2 | ENSG00000257194.2 | -0.890350194 | -1.979173199 | 0.047796509 |
| PGLS | ENST00000549806.1 | ENSG00000257252.1 | 0.891983691 | 1.964494465 | 0.049472778 |
| PGLS | ENST00000552525.1 | ENSG00000257286.1 | 0.822153278 | 1.845582123 | 0.064952904 |
| PGLS | ENST00000555342.1 | ENSG00000259048.1 | -0.833597544 | -1.856458694 | 0.063388205 |
| PGLS | ENST00000558515.1 | ENSG00000259182.1 | 0.858076907 | 1.886058465 | 0.059287077 |
| PGLS | ENST00000561567.1 | ENSG00000260177.1 | 0.818482141 | 1.84140275 | 0.06556256 |
| PGLS | ENST00000563044.1 | ENSG00000260978.1 | 0.816730553 | 1.829182247 | 0.067372311 |
| PGLS | ENST00000563806.1 | ENSG00000238045.5 | 0.886163984 | 1.977615193 | 0.047972134 |
| PGLS | ENST00000564417.1 | ENSG00000260137.1 | -0.808845757 | -1.771188426 | 0.076529373 |
| PGLS | ENST00000565823.1 | ENSG00000260686.1 | -0.812344945 | -1.830602672 | 0.067159869 |

| | | | | | |
|---|---|---|---|---|---|
| PGLS | ENST00000565829.1 | ENSG00000260148.1 | 0.826730154 | 1.847376154 | 0.064692643 |
| PGLS | ENST00000569981.1 | ENSG00000238045.5 | 0.828492484 | 1.872620703 | 0.06112078 |
| PGLS | ENST00000570843.1 | ENSG00000261889.1 | 0.870453147 | 1.955008958 | 0.050582013 |
| PGLS | ENST00000570929.1 | ENSG00000262223.2 | 0.828462171 | 1.866947654 | 0.061908898 |
| PGLS | ENST00000574460.1 | ENSG00000263051.1 | 0.83948805 | 1.870268409 | 0.061446554 |
| PGLS | ENST00000578757.1 | ENSG00000175061.13 | 0.809717429 | 1.833589399 | 0.066714967 |
| PGLS | ENST00000578800.1 | ENSG00000264235.1 | 0.957180857 | 2.173033261 | 0.029777814 |
| PGLS | ENST00000578936.1 | ENSG00000265547.1 | 0.928496067 | 2.083127162 | 0.037239634 |
| PGLS | ENST00000579154.1 | ENSG00000265908.1 | -0.84617557 | -1.881147544 | 0.059951851 |
| PGLS | ENST00000581905.1 | ENSG00000264235.1 | 0.814549201 | 1.809364742 | 0.070394357 |
| PGLS | ENST00000582044.1 | ENSG00000263715.2 | 0.840094784 | 1.876642829 | 0.060567063 |
| PGLS | ENST00000582386.1 | ENSG00000265174.1 | 0.859739462 | 1.92294329 | 0.054487172 |
| PGLS | ENST00000586694.1 | ENSG00000267141.1 | 0.914219474 | 2.046217894 | 0.040734938 |
| PGLS | ENST00000588182.2 | ENSG00000267453.2 | 0.856892945 | 1.899551037 | 0.057492063 |
| PGLS | ENST00000588290.1 | ENSG00000267751.1 | 0.873837031 | 1.954412091 | 0.050652502 |
| PGLS | ENST00000588380.1 | ENSG00000266990.1 | 0.886355871 | 1.971519501 | 0.048664485 |
| PGLS | ENST00000589817.1 | ENSG00000231616.4 | 0.824256919 | 1.846044956 | 0.064885678 |
| PGLS | ENST00000591174.1 | ENSG00000267289.1 | 0.869320612 | 1.940587087 | 0.052308382 |
| PGLS | ENST00000593139.1 | ENSG00000267042.1 | 0.848362284 | 1.92567068 | 0.054145507 |
| PGLS | ENST00000593588.1 | ENSG00000269635.1 | 0.840679448 | 1.870362317 | 0.061433521 |
| PGLS | ENST00000593632.1 | ENSG00000180279.5 | 0.848309869 | 1.914759271 | 0.055523211 |
| PGLS | ENST00000593642.1 | ENSG00000267858.1 | 0.813861588 | 1.830902409 | 0.067115111 |
| PGLS | ENST00000594492.1 | ENSG00000250910.3 | 0.802321712 | 1.788256028 | 0.073734711 |
| PGLS | ENST00000594590.2 | ENSG00000268199.2 | 0.827908762 | 1.860970409 | 0.062748356 |
| PGLS | ENST00000595955.1 | ENSG00000268401.1 | 0.885261229 | 1.986774555 | 0.046947386 |
| PGLS | ENST00000596887.1 | ENSG00000237031.3 | -0.817287381 | -1.819361244 | 0.068856335 |
| PGLS | ENST00000597169.1 | ENSG00000269720.1 | 0.921111617 | 2.078783716 | 0.037637234 |
| PGLS | ENST00000597256.1 | ENSG00000267986.1 | 0.906152406 | 2.066289747 | 0.038801129 |
| PGLS | ENST00000599259.1 | ENSG00000269352.1 | 0.832098011 | 1.886883547 | 0.059175991 |
| PGLS | ENST00000599352.1 | ENSG00000240401.4 | -0.801393215 | -1.796954554 | 0.072342835 |
| PGLS | ENST00000600234.1 | ENSG00000268078.1 | 0.880209525 | 1.954632812 | 0.050626426 |
| PGLS | ENST00000600534.1 | ENSG00000267858.1 | 0.816615073 | 1.818306994 | 0.069017224 |
| PGLS | ENST00000600726.1 | ENSG00000267858.1 | 0.811632023 | 1.813410749 | 0.069768493 |
| PGLS | ENST00000600889.1 | ENSG00000232675.3 | 0.907706663 | 2.064832857 | 0.038938818 |
| PGLS | ENST00000601033.1 | ENSG00000268401.1 | 0.958519386 | 2.141738786 | 0.032214508 |
| PGLS | ENST00000601735.1 | ENSG00000244513.2 | 0.890123158 | 1.96493936 | 0.049421256 |
| PGLS | ENST00000602532.1 | ENSG00000270091.1 | 0.878464688 | 1.945624442 | 0.051699869 |
| PGLS | ENST00000602594.1 | ENSG00000269930.1 | -0.843778121 | -1.922890604 | 0.05449379 |
| PGLS | ENST00000602809.1 | ENSG00000270105.1 | -0.81591839 | -1.811996348 | 0.069986761 |
| PGLS | ENST00000604142.1 | ENSG00000271308.1 | 0.896972235 | 2.005451031 | 0.044914851 |
| PGLS | ENST00000606277.1 | ENSG00000272145.1 | 0.944448837 | 2.133456358 | 0.032887308 |
| PGLS | ENST00000606377.1 | ENSG00000272286.1 | -0.88031737 | -1.975196363 | 0.048245869 |
| PGLS | ENST00000606470.1 | ENSG00000271913.1 | 0.815426992 | 1.814399558 | 0.069616234 |
| PGLS | ENST00000606963.1 | ENSG00000272010.1 | -0.909982569 | -2.055010485 | 0.039877992 |
| PGLS | ENST00000607201.1 | ENSG00000272024.1 | -0.822316031 | -1.840670276 | 0.065669892 |
| PGLS | ENST00000607224.1 | ENSG00000272521.1 | 0.814960706 | 1.814245371 | 0.069639958 |
| PGLS | ENST00000607476.1 | ENSG00000272540.1 | 0.905577796 | 2.046545361 | 0.040702744 |
| PGLS | ENST00000607580.1 | ENSG00000272545.1 | -0.824304292 | -1.832102742 | 0.066936115 |
| PGLS | ENST00000607943.1 | ENSG00000273188.1 | 0.839464522 | 1.870657283 | 0.061392599 |
| PGLS | ENST00000608367.1 | ENSG00000273361.1 | 0.847019407 | 1.88903567 | 0.058887049 |
| PGLS | ENST00000608677.1 | ENSG00000273350.1 | 0.941500857 | 2.089245443 | 0.036685635 |

| | | | | | |
|---|---|---|---|---|---|
| PGLS | ENST00000608940.1 | ENSG00000272763.1 | 0.88229468 | 1.964440345 | 0.049479048 |
| PGLS | NR_003604.2 | ZFAS1 | 0.914061374 | 2.07385863 | 0.038092442 |
| PGLS | NR_003605.1 | ZFAS1 | 0.872398091 | 1.952771542 | 0.050846672 |
| PGLS | NR_003606.2 | ZFAS1 | 0.914149168 | 2.058853815 | 0.039508245 |
| PGLS | NR_026802.1 | FAM74A4 | 0.985785613 | 2.209949486 | 0.027108668 |
| PGLS | NR_026813.1 | LINC00597 | -0.833886943 | -1.853400876 | 0.063824922 |
| PGLS | NR_026951.1 | LINC00324 | 0.939148923 | 2.102450883 | 0.035513798 |
| PGLS | NR_027052.1 | THAP7-AS1 | 0.882813269 | 1.986974928 | 0.046925175 |
| PGLS | NR_027271.1 | CIRBP-AS1 | 0.933771197 | 2.110276826 | 0.034834518 |
| PGLS | NR_027334.2 | MZF1-AS1 | 0.814787959 | 1.806358814 | 0.07086231 |
| PGLS | NR_036480.1 | VPS9D1-AS1 | 0.870601662 | 1.948891692 | 0.05130836 |
| PGLS | NR_036658.1 | ZFAS1 | 0.915833887 | 2.047579232 | 0.040601246 |
| PGLS | NR_038923.1 | SSSCA1-AS1 | 0.838136418 | 1.869189927 | 0.061596395 |
| PGLS | NR_040096.1 | LOC643339 | 0.863434431 | 1.90828549 | 0.056354327 |
| PGLS | NR_045637.1 | BOLA3-AS1 | 0.844706591 | 1.895123022 | 0.058076105 |
| PGLS | NR_046839.1 | AGBL4-IT1 | -0.859702553 | -1.929794829 | 0.053632265 |
| PGLS | NR_047116.1 | HIF1A-AS1 | -0.857217537 | -1.913926703 | 0.055629522 |
| PGLS | NR_072981.1 | LINC00957 | 0.809443922 | 1.802133619 | 0.071524385 |
| PGLS | NR_103790.1 | LINC00581 | -0.929811917 | -2.077179801 | 0.037784967 |
| PGLS | NR_105010.1 | LINC01333 | 0.9241327 | 2.080668384 | 0.03746427 |
| PGLS | NR_109886.1 | RALY-AS1 | 0.941151774 | 2.131662788 | 0.033034578 |
| PGLS | NR_110941.1 | MIR762HG | 0.816104182 | 1.803957343 | 0.071237994 |
| PGLS | NR_110998.1 | FAM74A4 | 0.985785613 | 2.232446188 | 0.025585488 |
| PGLS | NR_111951.1 | LINC00869 | 0.837480122 | 1.875025915 | 0.060789158 |
| PGLS | NR_111952.1 | LINC00869 | 0.858397705 | 1.926447751 | 0.05404849 |
| PGLS | NR_111953.1 | LINC00869 | 0.830720909 | 1.864647496 | 0.062230831 |
| PGLS | NR_121189.1 | PGM5P3-AS1 | -0.841586526 | -1.905889683 | 0.05666452 |
| PGLS | NR_125957.1 | LOC101928626 | -0.874871669 | -1.958690885 | 0.050148999 |
| PGLS | NR_126522.1 | EXOC3-AS1 | 0.965629657 | 2.172837908 | 0.029792519 |
| PGLS | NR_130143.1 | LOC104968399 | 0.959099606 | 2.158583238 | 0.030882514 |
| PGLS | NR_130144.1 | LOC104968399 | 0.814549201 | 1.827428863 | 0.067635313 |
| PGLS | NR_134252.1 | LOC105379030 | 0.846484742 | 1.91266905 | 0.055790434 |
| PGLS | NR_134579.1 | LOC105372179 | 0.928062021 | 2.070355403 | 0.038419075 |
| PGLS | NR_135024.1 | LOC105369747 | 0.942809888 | 2.089112869 | 0.036697564 |
| PIP | ENST00000398777.3 | ENSG00000240152.2 | -0.927368115 | -2.02965491 | 0.042391629 |
| PIP | ENST00000417426.1 | ENSG00000233145.1 | -0.829815246 | -1.828223879 | 0.067515959 |
| PIP | ENST00000418741.1 | ENSG00000227332.1 | -0.82938874 | -1.859747978 | 0.062921191 |
| PIP | ENST00000421207.1 | ENSG00000231768.1 | -0.873139963 | -1.923898164 | 0.054367349 |
| PIP | ENST00000421252.2 | ENSG00000250258.1 | 0.872881775 | 1.954955598 | 0.050588311 |
| PIP | ENST00000422697.1 | ENSG00000236414.1 | 0.807721559 | 1.812677498 | 0.069881577 |
| PIP | ENST00000423667.1 | ENSG00000225970.1 | -0.962911197 | -2.163785445 | 0.03048082 |
| PIP | ENST00000425124.1 | ENSG00000232336.1 | -0.801989422 | -1.793965547 | 0.072818668 |
| PIP | ENST00000425624.1 | ENSG00000223779.4 | -0.955188488 | -2.071211639 | 0.038339022 |
| PIP | ENST00000426699.1 | ENSG00000229308.1 | -0.945475018 | -2.13308412 | 0.032917826 |
| PIP | ENST00000432314.1 | ENSG00000231532.1 | -0.832280649 | -1.876435657 | 0.060595482 |
| PIP | ENST00000433344.1 | ENSG00000234083.1 | 0.879836834 | 1.952097863 | 0.050926587 |
| PIP | ENST00000433614.1 | ENSG00000228534.1 | 0.829043593 | 1.865864255 | 0.06206036 |
| PIP | ENST00000435992.2 | ENSG00000232675.3 | -0.827855283 | -1.817540324 | 0.06913442 |
| PIP | ENST00000436982.2 | ENSG00000235335.2 | 0.983333051 | 2.196366597 | 0.028065714 |
| PIP | ENST00000438107.1 | ENSG00000234449.2 | -0.838526672 | -1.842479931 | 0.065404979 |
| PIP | ENST00000439186.1 | ENSG00000237076.1 | -0.922639973 | -2.036146953 | 0.041735601 |

| | | | | | |
|---|---|---|---|---|---|
| PIP | ENST00000444245.1 | ENSG00000236753.1 | -0.816436046 | -1.784053927 | 0.074414903 |
| PIP | ENST00000447514.1 | ENSG00000236753.1 | -0.813049573 | -1.833106863 | 0.066786681 |
| PIP | ENST00000450848.1 | ENSG00000225539.1 | -0.87550163 | -1.980732617 | 0.047621267 |
| PIP | ENST00000455699.1 | ENSG00000240996.1 | -0.902795372 | -1.993963849 | 0.046156005 |
| PIP | ENST00000457169.1 | ENSG00000232408.1 | -0.887310415 | -2.002318134 | 0.045250527 |
| PIP | ENST00000458661.2 | ENSG00000236467.3 | -0.939890071 | -2.105512184 | 0.035246748 |
| PIP | ENST00000468444.2 | ENSG00000258525.1 | 0.876627734 | 1.931653362 | 0.053402305 |
| PIP | ENST00000469931.2 | ENSG00000272030.1 | -0.816436046 | -1.830278595 | 0.06720829 |
| PIP | ENST00000481334.1 | ENSG00000242440.1 | -0.924342908 | -2.05477227 | 0.039901006 |
| PIP | ENST00000484413.1 | ENSG00000271853.1 | -0.86381994 | -1.933787415 | 0.053139271 |
| PIP | ENST00000503938.1 | ENSG00000246095.2 | -0.852406085 | -1.91158814 | 0.055929042 |
| PIP | ENST00000504344.1 | ENSG00000251438.1 | -0.853941224 | -1.928160195 | 0.053835204 |
| PIP | ENST00000504795.1 | ENSG00000250723.1 | -0.882926181 | -1.97822943 | 0.04790283 |
| PIP | ENST00000504891.1 | ENSG00000249388.1 | -0.87902514 | -1.998572308 | 0.045654649 |
| PIP | ENST00000508825.1 | ENSG00000250775.1 | -0.810445017 | -1.80590283 | 0.070933519 |
| PIP | ENST00000513179.1 | ENSG00000251580.1 | -0.885682223 | -1.974634316 | 0.048309662 |
| PIP | ENST00000515205.1 | ENSG00000251580.1 | -0.898656874 | -2.02857637 | 0.042501457 |
| PIP | ENST00000517716.1 | ENSG00000253515.1 | 0.850657446 | 1.881019756 | 0.059969232 |
| PIP | ENST00000518473.1 | ENSG00000253985.1 | -0.959258991 | -2.144938842 | 0.031957737 |
| PIP | ENST00000521953.1 | ENSG00000253214.1 | -0.812089919 | -1.811953261 | 0.069993419 |
| PIP | ENST00000524818.1 | ENSG00000254473.1 | -0.883724291 | -1.968672553 | 0.048990705 |
| PIP | ENST00000525133.1 | ENSG00000255375.1 | -0.896153222 | -1.995339756 | 0.046005837 |
| PIP | ENST00000526186.1 | ENSG00000254510.1 | -0.898874367 | -2.003731516 | 0.045098828 |
| PIP | ENST00000526935.1 | ENSG00000255372.1 | -0.847621057 | -1.879980709 | 0.060110707 |
| PIP | ENST00000529837.1 | ENSG00000254687.1 | -0.820507683 | -1.835556978 | 0.066423205 |
| PIP | ENST00000532680.1 | ENSG00000255458.1 | -0.873376544 | -1.919657168 | 0.054901218 |
| PIP | ENST00000547834.1 | ENSG00000258325.1 | -0.814026798 | -1.773557134 | 0.076136433 |
| PIP | ENST00000549140.1 | ENSG00000258332.1 | -0.846952462 | -1.889316537 | 0.058849427 |
| PIP | ENST00000549487.1 | ENSG00000257126.1 | -0.868809503 | -1.964242727 | 0.04950195 |
| PIP | ENST00000552469.1 | ENSG00000258325.1 | -0.844297301 | -1.896061142 | 0.05795196 |
| PIP | ENST00000554679.1 | ENSG00000258837.1 | -0.927973964 | -2.158070831 | 0.030922325 |
| PIP | ENST00000556397.1 | ENSG00000258654.1 | -0.887119087 | -1.992368432 | 0.046330648 |
| PIP | ENST00000556786.1 | ENSG00000258525.1 | 0.910916652 | 2.023379398 | 0.043034047 |
| PIP | ENST00000557965.1 | ENSG00000259681.1 | -0.844826963 | -1.897450382 | 0.057768522 |
| PIP | ENST00000558312.1 | ENSG00000259176.1 | -0.836963295 | -1.887403761 | 0.05910604 |
| PIP | ENST00000558896.1 | ENSG00000259176.1 | -0.944808147 | -2.111064047 | 0.034766807 |
| PIP | ENST00000559003.1 | ENSG00000259520.1 | -0.965754916 | -2.135192332 | 0.032745303 |
| PIP | ENST00000560586.1 | ENSG00000259534.1 | -0.803865323 | -1.776423561 | 0.07566313 |
| PIP | ENST00000563408.1 | ENSG00000260733.1 | -0.821492791 | -1.833730508 | 0.066694008 |
| PIP | ENST00000563841.1 | ENSG00000261029.1 | -0.950808241 | -2.107254526 | 0.035095523 |
| PIP | ENST00000564038.1 | ENSG00000261760.2 | -0.852059445 | -1.881675936 | 0.059880029 |
| PIP | ENST00000565965.1 | ENSG00000261172.1 | -0.927928575 | -2.069007794 | 0.038545355 |
| PIP | ENST00000566170.1 | ENSG00000261071.1 | -0.822899556 | -1.856522961 | 0.063379053 |
| PIP | ENST00000567395.1 | ENSG00000261090.1 | -0.817776248 | -1.808256819 | 0.070566539 |
| PIP | ENST00000568033.1 | ENSG00000261480.1 | -0.821002104 | -1.827912888 | 0.067562626 |
| PIP | ENST00000568756.2 | ENSG00000261760.2 | -0.84252811 | -1.900552133 | 0.0573607 |
| PIP | ENST00000574365.1 | ENSG00000262837.1 | -0.803870501 | -1.777474654 | 0.075490177 |
| PIP | ENST00000577698.1 | ENSG00000265100.1 | -0.807903562 | -1.816023332 | 0.069366793 |
| PIP | ENST00000578265.1 | ENSG00000214719.7 | -0.82364553 | -1.856776495 | 0.063342959 |
| PIP | ENST00000580085.1 | ENSG00000266490.1 | -0.869962026 | -1.925921943 | 0.054114121 |
| PIP | ENST00000584705.1 | ENSG00000264569.1 | -0.829583683 | -1.84449978 | 0.065110338 |

| | | | | | |
|---|---|---|---|---|---|
| PIP | ENST00000588799.1 | ENSG00000267275.1 | -0.960895575 | -2.101348483 | 0.035610387 |
| PIP | ENST00000588945.1 | ENSG00000267275.1 | -0.974660846 | -2.175702386 | 0.029577522 |
| PIP | ENST00000589777.1 | ENSG00000261040.2 | -0.850737271 | -1.869743917 | 0.061519388 |
| PIP | ENST00000592100.1 | ENSG00000226686.3 | 0.85210305 | 1.917179001 | 0.055215195 |
| PIP | ENST00000592400.1 | ENSG00000267735.1 | -0.816048596 | -1.824159209 | 0.068128006 |
| PIP | ENST00000593861.1 | ENSG00000231898.4 | -0.908909206 | -2.032408786 | 0.042112289 |
| PIP | ENST00000597530.1 | ENSG00000228401.3 | -0.807128944 | -1.791041161 | 0.07328669 |
| PIP | ENST00000597550.1 | ENSG00000269051.1 | -0.922911286 | -2.057148737 | 0.039671921 |
| PIP | ENST00000599143.1 | ENSG00000269349.1 | -0.802061544 | -1.818348082 | 0.069010948 |
| PIP | ENST00000599524.1 | ENSG00000268240.1 | 0.806073548 | 1.818653759 | 0.068964271 |
| PIP | ENST00000600489.1 | ENSG00000231898.4 | -0.895651253 | -2.002841147 | 0.045194342 |
| PIP | ENST00000601692.1 | ENSG00000267874.1 | 0.942214281 | 2.106551307 | 0.035156491 |
| PIP | ENST00000602592.1 | ENSG00000270049.1 | -0.87583744 | -1.939714862 | 0.052414352 |
| PIP | ENST00000602598.1 | ENSG00000269944.1 | -0.807060365 | -1.774625118 | 0.075959807 |
| PIP | ENST00000602872.1 | ENSG00000270067.1 | -0.839319664 | -1.863755339 | 0.06235607 |
| PIP | ENST00000602954.1 | ENSG00000269906.1 | -0.955462108 | -2.160021311 | 0.03077102 |
| PIP | ENST00000604464.1 | ENSG00000270462.1 | -0.831411873 | -1.878825626 | 0.060268307 |
| PIP | ENST00000606457.1 | ENSG00000271830.1 | -0.932113568 | -2.135926796 | 0.032685381 |
| PIP | ENST00000607284.1 | ENSG00000272389.1 | -0.970889154 | -2.205555769 | 0.027415119 |
| PIP | ENST00000607769.1 | ENSG00000272438.1 | 0.801016988 | 1.804624444 | 0.07113347 |
| PIP | ENST00000607839.1 | ENSG00000272030.1 | -0.861082032 | -1.919676212 | 0.054898811 |
| PIP | ENST00000608085.1 | ENSG00000231898.4 | -0.828943822 | -1.84054374 | 0.065688449 |
| PIP | ENST00000608509.1 | ENSG00000273245.1 | -0.874255917 | -1.954214623 | 0.050675841 |
| PIP | ENST00000609270.1 | ENSG00000273073.1 | -0.814883095 | -1.83527503 | 0.066464949 |
| PIP | ENST00000609428.1 | ENSG00000273096.1 | -0.810410807 | -1.813295098 | 0.069786319 |
| PIP | ENST00000609701.1 | ENSG00000273284.1 | -0.854822152 | -1.890017719 | 0.05875559 |
| PIP | ENST00000609725.1 | ENSG00000231898.4 | -0.893192668 | -1.99975821 | 0.045526379 |
| PIP | ENST00000609972.1 | ENSG00000230651.3 | -0.96388699 | -2.150670528 | 0.031502214 |
| PIP | ENST00000610007.1 | ENSG00000272660.1 | -0.857659649 | -1.929394483 | 0.053681908 |
| PIP | ENST00000610161.1 | ENSG00000273059.1 | -0.934229617 | -2.076383061 | 0.037858537 |
| PIP | NR_033371.1 | CDRT7 | -0.873585739 | -1.965611821 | 0.049343468 |
| PIP | NR_044996.1 | HCG23 | -0.936707629 | -2.092898059 | 0.036358256 |
| PIP | NR_110480.1 | LOC101927079 | -0.900194961 | -2.020550075 | 0.043326362 |
| PIP | NR_110481.1 | LOC101927079 | -0.836963295 | -1.886283126 | 0.059256812 |
| PIP | NR_120335.1 | LOC101928414 | -0.98620526 | -2.213654808 | 0.026852534 |
| PIP | NR_120655.1 | KCNMA1-AS1 | -0.894531319 | -1.981676599 | 0.047515448 |
| PIP | NR_126166.1 | FAM74A7 | -0.948363351 | -2.071502528 | 0.038311858 |
| PIP | NR_134597.1 | LOC105378068 | -0.869164896 | -1.955491027 | 0.050525141 |
| PIP | NR_134664.1 | LOC105374366 | -0.919247638 | -2.093604877 | 0.036295193 |
| PIP | NR_134665.1 | LOC105374366 | -0.90942407 | -2.045533549 | 0.040802285 |
| PIP | NR_138084.1 | HCG24 | -0.854943952 | -1.911665154 | 0.055919156 |
| POLD4 | ENST00000390540.2 | ENSG00000254140.1 | 0.858072119 | 1.91651864 | 0.055299113 |
| POLD4 | ENST00000415205.1 | ENSG00000182057.4 | 0.944905152 | 2.126424837 | 0.033467902 |
| POLD4 | ENST00000419662.1 | ENSG00000228265.1 | 0.851988472 | 1.902792835 | 0.057067583 |
| POLD4 | ENST00000422763.1 | ENSG00000231131.2 | -0.956857965 | -2.115060382 | 0.034424801 |
| POLD4 | ENST00000425881.1 | ENSG00000239636.1 | 0.951430045 | 2.15064417 | 0.031504296 |
| POLD4 | ENST00000426475.1 | ENSG00000239467.1 | 0.963934729 | 2.16114594 | 0.030684068 |
| POLD4 | ENST00000428765.1 | ENSG00000230107.1 | 0.829794367 | 1.867296031 | 0.061860259 |
| POLD4 | ENST00000429080.1 | ENSG00000233047.1 | -0.847357871 | -1.902446425 | 0.057112817 |
| POLD4 | ENST00000430920.1 | ENSG00000234203.1 | 0.858874815 | 1.909621306 | 0.05618199 |
| POLD4 | ENST00000431730.1 | ENSG00000237401.2 | 0.847707828 | 1.902211489 | 0.057143511 |

| | | | | | |
|---|---|---|---|---|---|
| POLD4 | ENST00000433051.1 | ENSG00000233193.1 | 0.881161877 | 1.989404131 | 0.046656614 |
| POLD4 | ENST00000433905.2 | ENSG00000229299.2 | 0.849405731 | 1.890586992 | 0.058679498 |
| POLD4 | ENST00000434250.1 | ENSG00000234055.1 | 0.827368558 | 1.86507318 | 0.062171147 |
| POLD4 | ENST00000438969.2 | ENSG00000228031.2 | -0.859953102 | -1.947774025 | 0.051442008 |
| POLD4 | ENST00000439184.1 | ENSG00000233985.1 | -0.820736274 | -1.82884681 | 0.06742256 |
| POLD4 | ENST00000440595.1 | ENSG00000228265.1 | 0.932881143 | 2.081765775 | 0.037363869 |
| POLD4 | ENST00000441592.2 | ENSG00000224078.8 | 0.978876421 | 2.180638564 | 0.02921016 |
| POLD4 | ENST00000442649.1 | ENSG00000234089.1 | -0.842616947 | -1.891336272 | 0.05857947 |
| POLD4 | ENST00000446562.1 | ENSG00000233896.1 | 0.934239987 | 2.10115057 | 0.035627751 |
| POLD4 | ENST00000447343.2 | ENSG00000229299.2 | 0.86347986 | 1.919520565 | 0.054918486 |
| POLD4 | ENST00000448570.1 | ENSG00000224549.1 | 0.904780595 | 2.023322012 | 0.043039959 |
| POLD4 | ENST00000451507.1 | ENSG00000229539.1 | 0.935868762 | 2.066424872 | 0.038788379 |
| POLD4 | ENST00000456091.1 | ENSG00000226985.1 | 0.870178904 | 1.963657896 | 0.049569778 |
| POLD4 | ENST00000463255.1 | ENSG00000243305.1 | -0.899259108 | -2.000142351 | 0.045484895 |
| POLD4 | ENST00000468165.1 | ENSG00000239480.1 | 0.803403612 | 1.788264509 | 0.073733343 |
| POLD4 | ENST00000488310.1 | ENSG00000240449.1 | 0.838946544 | 1.865794647 | 0.062070102 |
| POLD4 | ENST00000489077.1 | ENSG00000244198.1 | 0.882288253 | 1.991225631 | 0.046456087 |
| POLD4 | ENST00000489090.1 | ENSG00000240045.1 | -0.884621275 | -1.97633832 | 0.048116472 |
| POLD4 | ENST00000503723.1 | ENSG00000250472.1 | -0.923481194 | -2.062520951 | 0.039158165 |
| POLD4 | ENST00000504916.1 | ENSG00000248112.1 | -0.895597614 | -1.998259325 | 0.045688553 |
| POLD4 | ENST00000509036.1 | ENSG00000251131.1 | 0.80134626 | 1.758923148 | 0.078590563 |
| POLD4 | ENST00000509453.1 | ENSG00000249145.1 | 0.878677835 | 1.965269701 | 0.049383031 |
| POLD4 | ENST00000510570.1 | ENSG00000250438.1 | -0.90171268 | -2.02791163 | 0.042569268 |
| POLD4 | ENST00000510922.1 | ENSG00000250777.1 | -0.813331919 | -1.834909702 | 0.06651907 |
| POLD4 | ENST00000517846.1 | ENSG00000254485.1 | 0.906365079 | 2.012137359 | 0.044205459 |
| POLD4 | ENST00000520603.1 | ENSG00000254001.1 | -0.838524827 | -1.862246159 | 0.062568401 |
| POLD4 | ENST00000521653.1 | ENSG00000253301.1 | 0.947492309 | 2.122474924 | 0.033797876 |
| POLD4 | ENST00000523806.1 | ENSG00000253616.1 | 0.814430836 | 1.830784935 | 0.06713265 |
| POLD4 | ENST00000529247.1 | ENSG00000254741.1 | 0.890448991 | 1.99982042 | 0.045519659 |
| POLD4 | ENST00000530435.1 | ENSG00000254630.1 | 0.844181083 | 1.885807419 | 0.059320911 |
| POLD4 | ENST00000535315.1 | ENSG00000250748.2 | -0.809887907 | -1.805330711 | 0.071022947 |
| POLD4 | ENST00000543403.1 | ENSG00000256684.1 | -0.881595154 | -1.97183883 | 0.048628008 |
| POLD4 | ENST00000549806.1 | ENSG00000257252.1 | 0.88910983 | 1.968686555 | 0.048989096 |
| POLD4 | ENST00000557368.1 | ENSG00000258444.1 | 0.868123485 | 1.921265022 | 0.054698304 |
| POLD4 | ENST00000562191.1 | ENSG00000261292.1 | -0.900539074 | -2.000712103 | 0.045423424 |
| POLD4 | ENST00000563611.1 | ENSG00000261583.1 | 0.862987317 | 1.911742912 | 0.055909177 |
| POLD4 | ENST00000564809.1 | ENSG00000261471.1 | 0.90404263 | 2.033218306 | 0.042030472 |
| POLD4 | ENST00000565955.1 | ENSG00000261055.1 | 0.844433191 | 1.897614428 | 0.057746892 |
| POLD4 | ENST00000571660.1 | ENSG00000262848.1 | 0.915472159 | 2.056512249 | 0.039733167 |
| POLD4 | ENST00000573315.1 | ENSG00000270168.1 | 0.878334695 | 1.949106431 | 0.051282716 |
| POLD4 | ENST00000578800.1 | ENSG00000264235.1 | 0.846667382 | 1.921278311 | 0.05469663 |
| POLD4 | ENST00000579154.1 | ENSG00000265908.1 | -0.834298585 | -1.886128508 | 0.05927764 |
| POLD4 | ENST00000579775.1 | ENSG00000264108.1 | 0.864102994 | 1.953446026 | 0.050766767 |
| POLD4 | ENST00000580622.1 | ENSG00000264634.1 | 0.988627646 | 2.200115033 | 0.027798735 |
| POLD4 | ENST00000582044.1 | ENSG00000263715.2 | 0.984465386 | 2.200813144 | 0.027749255 |
| POLD4 | ENST00000583067.1 | ENSG00000266126.1 | -0.813780142 | -1.825717259 | 0.067892862 |
| POLD4 | ENST00000585761.1 | ENSG00000267198.1 | 0.854205151 | 1.913847929 | 0.055639589 |
| POLD4 | ENST00000586348.1 | ENSG00000267198.1 | 0.878585357 | 1.966565331 | 0.049233344 |
| POLD4 | ENST00000588182.2 | ENSG00000267453.2 | 0.861929285 | 1.912856953 | 0.055766368 |
| POLD4 | ENST00000588380.1 | ENSG00000266990.1 | 0.865666737 | 1.926818231 | 0.054002287 |
| POLD4 | ENST00000589380.1 | ENSG00000267488.1 | 0.939667963 | 2.098596246 | 0.035852508 |

| | | | | | |
|---|---|---|---|---|---|
| POLD4 | ENST00000589395.1 | ENSG00000267143.1 | 0.891481899 | 2.005999003 | 0.044856354 |
| POLD4 | ENST00000592498.1 | ENSG00000267488.1 | 0.931869359 | 2.080634699 | 0.037467355 |
| POLD4 | ENST00000592525.1 | ENSG00000267214.1 | 0.984473748 | 2.216791257 | 0.026637359 |
| POLD4 | ENST00000593139.1 | ENSG00000267042.1 | 0.97253648 | 2.183615002 | 0.02899055 |
| POLD4 | ENST00000593218.1 | ENSG00000267421.2 | 0.836602822 | 1.877284734 | 0.060479079 |
| POLD4 | ENST00000594776.1 | ENSG00000269807.1 | 0.846494725 | 1.886982371 | 0.059162697 |
| POLD4 | ENST00000594850.1 | ENSG00000268093.1 | 0.954037509 | 2.118584319 | 0.034125612 |
| POLD4 | ENST00000596091.1 | ENSG00000227733.4 | -0.810732518 | -1.82799766 | 0.067549903 |
| POLD4 | ENST00000596887.1 | ENSG00000237031.3 | -0.866131161 | -1.916519779 | 0.055298968 |
| POLD4 | ENST00000596971.1 | ENSG00000269463.1 | 0.927138975 | 2.076289384 | 0.037867195 |
| POLD4 | ENST00000597256.1 | ENSG00000267986.1 | 0.94737473 | 2.110613938 | 0.034805509 |
| POLD4 | ENST00000600242.1 | ENSG00000269583.1 | 0.931029175 | 2.086969139 | 0.036890924 |
| POLD4 | ENST00000603948.1 | ENSG00000222041.6 | 0.866734044 | 1.921246193 | 0.054700677 |
| POLD4 | ENST00000604142.1 | ENSG00000271308.1 | 0.843215012 | 1.891697843 | 0.058531251 |
| POLD4 | ENST00000604183.1 | ENSG00000271185.1 | 0.920515578 | 2.048644203 | 0.040496919 |
| POLD4 | ENST00000605780.1 | ENSG00000270755.1 | 0.821681542 | 1.839055184 | 0.06590707 |
| POLD4 | ENST00000606441.1 | ENSG00000272277.1 | 0.957283412 | 2.155753138 | 0.031102946 |
| POLD4 | ENST00000606743.1 | ENSG00000272221.1 | 0.810215914 | 1.821611633 | 0.068513935 |
| POLD4 | ENST00000606909.1 | ENSG00000271821.1 | 0.915893649 | 2.055723536 | 0.039809172 |
| POLD4 | ENST00000607476.1 | ENSG00000272540.1 | 0.869259602 | 1.944881814 | 0.051789204 |
| POLD4 | ENST00000607943.1 | ENSG00000273188.1 | 0.824416375 | 1.824534567 | 0.068071295 |
| POLD4 | ENST00000608367.1 | ENSG00000273361.1 | 0.872862296 | 1.97420798 | 0.048358099 |
| POLD4 | ENST00000608677.1 | ENSG00000273350.1 | 0.877555555 | 1.971853732 | 0.048626306 |
| POLD4 | NR_026774.1 | LINC00239 | 0.804131494 | 1.790014788 | 0.073451534 |
| POLD4 | NR_026802.1 | FAM74A4 | 0.816391447 | 1.849721361 | 0.06435372 |
| POLD4 | NR_026813.1 | LINC00597 | -0.953060373 | -2.129510755 | 0.033212026 |
| POLD4 | NR_026951.1 | LINC00324 | 0.8712367 | 1.934298849 | 0.053076395 |
| POLD4 | NR_027052.1 | THAP7-AS1 | 0.860943559 | 1.919369207 | 0.054937625 |
| POLD4 | NR_034131.1 | LINC00272 | 0.838575827 | 1.900280449 | 0.057396326 |
| POLD4 | NR_037169.1 | LOC100507547 | 0.848227478 | 1.870983861 | 0.061347318 |
| POLD4 | NR_037170.1 | LOC100507547 | 0.828048174 | 1.842524564 | 0.065398457 |
| POLD4 | NR_038421.1 | LINC01220 | 0.80721184 | 1.807558473 | 0.070675246 |
| POLD4 | NR_038923.1 | SSSCA1-AS1 | 0.920517361 | 2.073813776 | 0.038096609 |
| POLD4 | NR_040047.1 | SDCBP2-AS1 | 0.804441323 | 1.80347377 | 0.071313841 |
| POLD4 | NR_040049.1 | SDCBP2-AS1 | 0.867720083 | 1.941867576 | 0.052153134 |
| POLD4 | NR_045114.1 | PVRL3-AS1 | -0.807245852 | -1.830994938 | 0.067101299 |
| POLD4 | NR_046871.1 | LINC00333 | -0.853869822 | -1.92354187 | 0.054412033 |
| POLD4 | NR_047116.1 | HIF1A-AS1 | -0.9241822 | -2.049138731 | 0.040448551 |
| POLD4 | NR_051987.1 | LINC00499 | 0.812246732 | 1.807214928 | 0.070728774 |
| POLD4 | NR_108106.1 | LINC01135 | 0.887396841 | 1.969734592 | 0.048868796 |
| POLD4 | NR_109831.1 | RASSF1-AS1 | 0.913944396 | 2.043213519 | 0.041031306 |
| POLD4 | NR_109885.1 | RALY-AS1 | 0.925536799 | 2.078238174 | 0.037687428 |
| POLD4 | NR_109886.1 | RALY-AS1 | 0.832833632 | 1.875998807 | 0.060655444 |
| POLD4 | NR_110245.1 | LOC101929282 | -0.851421807 | -1.90917727 | 0.056239227 |
| POLD4 | NR_110559.1 | LOC101927023 | -0.870871509 | -1.956958216 | 0.050352381 |
| POLD4 | NR_110630.1 | LOC101927478 | 0.840238257 | 1.879814185 | 0.060133406 |
| POLD4 | NR_110941.1 | MIR762HG | 0.93063076 | 2.072036411 | 0.038262046 |
| POLD4 | NR_110998.1 | FAM74A4 | 0.816391447 | 1.833135912 | 0.066782362 |
| POLD4 | NR_111951.1 | LINC00869 | 0.825018287 | 1.846567503 | 0.064809848 |
| POLD4 | NR_111952.1 | LINC00869 | 0.812473183 | 1.817089681 | 0.069203382 |
| POLD4 | NR_111953.1 | LINC00869 | 0.824114616 | 1.849549001 | 0.064378579 |

| | | | | | |
|---|---|---|---|---|---|
| POLD4 | NR_125849.1 | LOC101928140 | -0.836059198 | -1.853566642 | 0.063801184 |
| POLD4 | NR_126522.1 | EXOC3-AS1 | 0.836229887 | 1.864180869 | 0.062296309 |
| POLD4 | NR_130143.1 | LOC104968399 | 0.80966081 | 1.7900528 | 0.073445424 |
| POLD4 | NR_131204.1 | XACT | 0.843468787 | 1.863051829 | 0.062454975 |
| POLD4 | NR_134520.1 | LOC727993 | 0.941097151 | 2.094177952 | 0.036244131 |
| POLD4 | NR_135024.1 | LOC105369747 | 0.856883927 | 1.904295459 | 0.056871715 |
| POLD4 | NR_135097.1 | LOC105369443 | -0.881595154 | -1.950352946 | 0.051134066 |
| POLD4 | NR_136215.1 | VCAN-AS1 | -0.934668174 | -2.105509031 | 0.035247022 |
| POLD4 | NR_136320.1 | LOC105373656 | 0.857619055 | 1.913905686 | 0.055632208 |
| POLR2J3 | ENST00000400768.2 | ENSG00000215692.2 | 0.889415534 | 2.00651097 | 0.04480176 |
| POLR2J3 | ENST00000411489.1 | ENSG00000227112.1 | -0.827420664 | -1.848445957 | 0.064537856 |
| POLR2J3 | ENST00000420766.1 | ENSG00000228679.1 | 0.883071124 | 1.994017922 | 0.046150096 |
| POLR2J3 | ENST00000420845.1 | ENSG00000232259.1 | 0.929882136 | 2.097520009 | 0.035947568 |
| POLR2J3 | ENST00000421737.1 | ENSG00000232316.1 | 0.801736778 | 1.78832953 | 0.073722858 |
| POLR2J3 | ENST00000426653.1 | ENSG00000235704.1 | 0.802373173 | 1.782503332 | 0.074667188 |
| POLR2J3 | ENST00000429666.1 | ENSG00000233755.1 | 0.920923 | 2.060641255 | 0.039337278 |
| POLR2J3 | ENST00000430751.1 | ENSG00000232222.1 | -0.848812621 | -1.897959146 | 0.057701464 |
| POLR2J3 | ENST00000437095.1 | ENSG00000237087.1 | 0.978879879 | 2.184349751 | 0.028936558 |
| POLR2J3 | ENST00000450227.1 | ENSG00000229941.1 | 0.906538249 | 1.994702849 | 0.046075299 |
| POLR2J3 | ENST00000469846.2 | ENSG00000206573.4 | -0.864601384 | -1.946210041 | 0.051629514 |
| POLR2J3 | ENST00000487772.1 | ENSG00000241754.1 | 0.830617814 | 1.864347299 | 0.062272949 |
| POLR2J3 | ENST00000498199.1 | ENSG00000206573.4 | -0.867204775 | -1.919349876 | 0.05494007 |
| POLR2J3 | ENST00000500267.2 | ENSG00000246323.2 | 0.95079978 | 2.117437325 | 0.034222749 |
| POLR2J3 | ENST00000504755.1 | ENSG00000250252.1 | 0.823977978 | 1.84764192 | 0.064654161 |
| POLR2J3 | ENST00000508414.1 | ENSG00000248173.1 | 0.835903232 | 1.878682488 | 0.06028786 |
| POLR2J3 | ENST00000519506.1 | ENSG00000253103.1 | 0.804727951 | 1.78495658 | 0.07426836 |
| POLR2J3 | ENST00000519750.1 | ENSG00000241956.5 | 0.906592998 | 2.064687335 | 0.038952594 |
| POLR2J3 | ENST00000535720.1 | ENSG00000256364.1 | 0.866385514 | 1.921541818 | 0.054663436 |
| POLR2J3 | ENST00000535806.1 | ENSG00000255817.1 | 0.87206933 | 1.924072109 | 0.054345546 |
| POLR2J3 | ENST00000536492.1 | ENSG00000256237.1 | -0.867963047 | -1.929816736 | 0.053629549 |
| POLR2J3 | ENST00000549303.1 | ENSG00000257180.1 | -0.8555054 | -1.908700196 | 0.056300778 |
| POLR2J3 | ENST00000554451.1 | ENSG00000258683.1 | 0.923449301 | 2.048477531 | 0.040513232 |
| POLR2J3 | ENST00000558475.1 | ENSG00000259604.1 | 0.826543624 | 1.833122705 | 0.066784325 |
| POLR2J3 | ENST00000558568.1 | ENSG00000272639.1 | 0.849395329 | 1.891606246 | 0.058543463 |
| POLR2J3 | ENST00000559673.1 | ENSG00000259604.1 | 0.829895756 | 1.854899658 | 0.063610557 |
| POLR2J3 | ENST00000562834.1 | ENSG00000261116.1 | 0.800813658 | 1.781585531 | 0.074816846 |
| POLR2J3 | ENST00000562970.1 | ENSG00000260145.1 | 0.84998319 | 1.895164246 | 0.058070645 |
| POLR2J3 | ENST00000577807.1 | ENSG00000263427.1 | 0.829401569 | 1.856056775 | 0.063445466 |
| POLR2J3 | ENST00000583826.1 | ENSG00000265148.1 | 0.880678842 | 1.966985333 | 0.049184902 |
| POLR2J3 | ENST00000583841.1 | ENSG00000265148.1 | 0.834010352 | 1.875939155 | 0.060663635 |
| POLR2J3 | ENST00000585703.1 | ENSG00000235779.3 | 0.851819469 | 1.879428809 | 0.060185966 |
| POLR2J3 | ENST00000588334.1 | ENSG00000235779.3 | 0.867389123 | 1.953621943 | 0.050745944 |
| POLR2J3 | ENST00000590255.1 | ENSG00000235779.3 | 0.805724209 | 1.814290327 | 0.06963304 |
| POLR2J3 | ENST00000590357.1 | ENSG00000267175.1 | -0.801860793 | -1.781280792 | 0.074866591 |
| POLR2J3 | ENST00000591178.1 | ENSG00000235779.3 | 0.84047714 | 1.896558588 | 0.057886221 |
| POLR2J3 | ENST00000598735.1 | ENSG00000268093.1 | 0.857734068 | 1.901505913 | 0.057235779 |
| POLR2J3 | ENST00000599467.1 | ENSG00000244513.2 | 0.818126773 | 1.858025097 | 0.06316545 |
| POLR2J3 | ENST00000600365.1 | ENSG00000231898.4 | 0.817653465 | 1.815094115 | 0.069509447 |
| POLR2J3 | ENST00000601511.1 | ENSG00000244513.2 | 0.82628139 | 1.835471621 | 0.06643584 |
| POLR2J3 | ENST00000608159.1 | ENSG00000273093.1 | -0.810780206 | -1.832059071 | 0.06694262 |
| POLR2J3 | ENST00000608275.1 | ENSG00000273343.1 | 0.969231798 | 2.188155424 | 0.028658283 |

| | | | | | |
|---|---|---|---|---|---|
| POLR2J3 | ENST00000608856.1 | ENSG00000272600.1 | -0.889220144 | -1.990157641 | 0.046573573 |
| POLR2J3 | ENST00000609349.1 | ENSG00000272861.1 | 0.820726548 | 1.871804098 | 0.061233711 |
| POLR2J3 | ENST00000609837.1 | ENSG00000273106.1 | 0.800914204 | 1.813651543 | 0.06973139 |
| POLR2J3 | NR_120318.1 | RORA-AS2 | 0.888227607 | 1.990481605 | 0.046537908 |
| POLR2J3 | NR_120566.1 | LOC101928896 | 0.865885781 | 1.939641637 | 0.052423257 |
| POLR2J3 | NR_125839.1 | LOC101927020 | 0.803229992 | 1.797573631 | 0.0722446 |
| POLR2J3 | NR_126389.1 | LINC00428 | 0.859973248 | 1.921667173 | 0.05464765 |
| POLR2J3 | NR_126391.1 | LINC01054 | 0.862294375 | 1.946500185 | 0.051594686 |
| POLR2J3 | NR_135274.1 | LOC105370619 | 0.837267481 | 1.858055235 | 0.063161171 |
| POLR2J3 | NR_136407.1 | LOC105371430 | 0.868602709 | 1.938561497 | 0.052554756 |
| POLR2J3 | NR_138419.1 | ARHGEF9-IT1 | 0.866626691 | 1.933434556 | 0.053182688 |
| PPP1R18 | ENST00000415205.1 | ENSG00000182057.4 | 0.902542733 | 2.017159888 | 0.043678831 |
| PPP1R18 | ENST00000417260.1 | ENSG00000231734.4 | -0.928428636 | -2.049947268 | 0.040369577 |
| PPP1R18 | ENST00000419662.1 | ENSG00000228265.1 | 0.924622417 | 2.080095655 | 0.03751676 |
| PPP1R18 | ENST00000421207.1 | ENSG00000231768.1 | 0.806760462 | 1.788084805 | 0.073762327 |
| PPP1R18 | ENST00000422763.1 | ENSG00000231131.2 | -0.821341632 | -1.837295975 | 0.066166214 |
| PPP1R18 | ENST00000423428.1 | ENSG00000224048.1 | -0.899864784 | -1.992717808 | 0.046292356 |
| PPP1R18 | ENST00000425124.1 | ENSG00000232336.1 | 0.93153224 | 2.088703422 | 0.036734429 |
| PPP1R18 | ENST00000425881.1 | ENSG00000239636.1 | 0.827917899 | 1.854935672 | 0.063605413 |
| PPP1R18 | ENST00000426237.2 | ENSG00000235527.2 | 0.850516793 | 1.894418024 | 0.058169546 |
| PPP1R18 | ENST00000429080.1 | ENSG00000233047.1 | -0.844859951 | -1.849961745 | 0.064319063 |
| PPP1R18 | ENST00000431730.1 | ENSG00000237401.2 | 0.873157014 | 1.961161854 | 0.049860145 |
| PPP1R18 | ENST00000433051.1 | ENSG00000233193.1 | 0.907383115 | 2.03349993 | 0.042002041 |
| PPP1R18 | ENST00000433905.2 | ENSG00000229299.2 | 0.846185293 | 1.889876676 | 0.058774456 |
| PPP1R18 | ENST00000435287.1 | ENSG00000227220.1 | 0.838338962 | 1.843716516 | 0.065224465 |
| PPP1R18 | ENST00000438190.1 | ENSG00000227214.2 | 0.904687497 | 2.031654099 | 0.042188686 |
| PPP1R18 | ENST00000440595.1 | ENSG00000228265.1 | 0.941710231 | 2.117807297 | 0.034191391 |
| PPP1R18 | ENST00000441592.2 | ENSG00000224078.8 | 0.890911581 | 1.996753967 | 0.045851916 |
| PPP1R18 | ENST00000442649.1 | ENSG00000234089.1 | -0.813778424 | -1.816327131 | 0.069320206 |
| PPP1R18 | ENST00000446562.1 | ENSG00000233896.1 | 0.812876645 | 1.798006886 | 0.072175917 |
| PPP1R18 | ENST00000447343.2 | ENSG00000229299.2 | 0.821958771 | 1.83781569 | 0.066089569 |
| PPP1R18 | ENST00000448858.1 | ENSG00000237734.1 | -0.827883857 | -1.847176222 | 0.064721604 |
| PPP1R18 | ENST00000451507.1 | ENSG00000229539.1 | 0.947508653 | 2.123011555 | 0.033752884 |
| PPP1R18 | ENST00000452176.1 | ENSG00000223659.1 | -0.913316251 | -2.035274907 | 0.04182322 |
| PPP1R18 | ENST00000454100.1 | ENSG00000236943.2 | 0.807498489 | 1.804794016 | 0.071106921 |
| PPP1R18 | ENST00000456091.1 | ENSG00000226985.1 | 0.821023484 | 1.843817609 | 0.065209726 |
| PPP1R18 | ENST00000457371.1 | ENSG00000237401.2 | 0.830873109 | 1.864777859 | 0.062212548 |
| PPP1R18 | ENST00000458154.1 | ENSG00000235578.1 | 0.876664409 | 1.951371475 | 0.051012872 |
| PPP1R18 | ENST00000463255.1 | ENSG00000243305.1 | -0.867612643 | -1.935544923 | 0.052923462 |
| PPP1R18 | ENST00000489077.1 | ENSG00000244198.1 | 0.974101187 | 2.168995025 | 0.030083062 |
| PPP1R18 | ENST00000489090.1 | ENSG00000240045.1 | -0.829040176 | -1.845529881 | 0.064960496 |
| PPP1R18 | ENST00000498693.1 | ENSG00000244198.1 | 0.865839349 | 1.942699449 | 0.052052483 |
| PPP1R18 | ENST00000503723.1 | ENSG00000250472.1 | -0.935627796 | -2.074840224 | 0.038001345 |
| PPP1R18 | ENST00000506791.1 | ENSG00000251131.1 | 0.950601472 | 2.123156542 | 0.033740736 |
| PPP1R18 | ENST00000509036.1 | ENSG00000251131.1 | 0.889433838 | 1.990036729 | 0.04658689 |
| PPP1R18 | ENST00000509192.1 | ENSG00000250765.1 | 0.960122475 | 2.136524436 | 0.032636691 |
| PPP1R18 | ENST00000509453.1 | ENSG00000249145.1 | 0.973669139 | 2.168049786 | 0.030154899 |
| PPP1R18 | ENST00000510570.1 | ENSG00000250438.1 | -0.886770409 | -1.981404945 | 0.04754588 |
| PPP1R18 | ENST00000515128.1 | ENSG00000248215.1 | -0.850054692 | -1.883027241 | 0.059696676 |
| PPP1R18 | ENST00000520603.1 | ENSG00000254001.1 | -0.981675111 | -2.193861452 | 0.028245371 |
| PPP1R18 | ENST00000521653.1 | ENSG00000253301.1 | 0.88335552 | 1.968089877 | 0.049057697 |

| | | | | | |
|---|---|---|---|---|---|
| PPP1R18 | ENST00000534178.1 | ENSG00000255120.1 | 0.807997717 | 1.833369551 | 0.066747633 |
| PPP1R18 | ENST00000537850.1 | ENSG00000251002.3 | 0.813097974 | 1.806971849 | 0.070766669 |
| PPP1R18 | ENST00000543403.1 | ENSG00000256684.1 | -0.824171192 | -1.82801023 | 0.067548016 |
| PPP1R18 | ENST00000549806.1 | ENSG00000257252.1 | 0.800233975 | 1.775089203 | 0.075883159 |
| PPP1R18 | ENST00000550263.1 | ENSG00000257605.1 | 0.921964236 | 2.071423716 | 0.038319216 |
| PPP1R18 | ENST00000562191.1 | ENSG00000261292.1 | -0.850059915 | -1.893511068 | 0.058289938 |
| PPP1R18 | ENST00000562995.1 | ENSG00000261253.1 | 0.844660881 | 1.887464013 | 0.059097942 |
| PPP1R18 | ENST00000563611.1 | ENSG00000261583.1 | 0.883321507 | 1.989954943 | 0.046595899 |
| PPP1R18 | ENST00000564809.1 | ENSG00000261471.1 | 0.833781326 | 1.866067853 | 0.062031873 |
| PPP1R18 | ENST00000565955.1 | ENSG00000261055.1 | 0.818171558 | 1.850097053 | 0.064299563 |
| PPP1R18 | ENST00000567395.1 | ENSG00000261090.1 | 0.815092181 | 1.827165949 | 0.067674822 |
| PPP1R18 | ENST00000569981.1 | ENSG00000238045.5 | 0.854485905 | 1.912420332 | 0.055822302 |
| PPP1R18 | ENST00000570493.2 | ENSG00000261898.2 | 0.889758689 | 1.969829028 | 0.048857969 |
| PPP1R18 | ENST00000571660.1 | ENSG00000262848.1 | 0.888541739 | 1.985375096 | 0.047102755 |
| PPP1R18 | ENST00000573315.1 | ENSG00000270168.1 | 0.811595185 | 1.81819998 | 0.069033573 |
| PPP1R18 | ENST00000580622.1 | ENSG00000264634.1 | 0.860148567 | 1.940162344 | 0.052359963 |
| PPP1R18 | ENST00000582044.1 | ENSG00000263715.2 | 0.941909297 | 2.118797654 | 0.034107571 |
| PPP1R18 | ENST00000582558.1 | ENSG00000264569.1 | 0.959108534 | 2.155366396 | 0.031133174 |
| PPP1R18 | ENST00000584705.1 | ENSG00000264569.1 | 0.854341144 | 1.906464338 | 0.056589988 |
| PPP1R18 | ENST00000585559.1 | ENSG00000267117.1 | 0.849220278 | 1.919315022 | 0.054944478 |
| PPP1R18 | ENST00000586348.1 | ENSG00000267198.1 | 0.80465344 | 1.793352036 | 0.072916652 |
| PPP1R18 | ENST00000586694.1 | ENSG00000267141.1 | 0.864839284 | 1.935349238 | 0.052947454 |
| PPP1R18 | ENST00000588380.1 | ENSG00000266990.1 | 0.933129851 | 2.108081636 | 0.035023929 |
| PPP1R18 | ENST00000589395.1 | ENSG00000267143.1 | 0.935784232 | 2.081843626 | 0.037356755 |
| PPP1R18 | ENST00000591174.1 | ENSG00000267289.1 | 0.812096248 | 1.821175497 | 0.068580184 |
| PPP1R18 | ENST00000592400.1 | ENSG00000267735.1 | 0.862260151 | 1.922247281 | 0.05457465 |
| PPP1R18 | ENST00000592525.1 | ENSG00000267214.1 | 0.862933029 | 1.939624079 | 0.052425392 |
| PPP1R18 | ENST00000593139.1 | ENSG00000267042.1 | 0.81549025 | 1.82988197 | 0.06726759 |
| PPP1R18 | ENST00000594776.1 | ENSG00000269807.1 | 0.822151148 | 1.849200267 | 0.0644289 |
| PPP1R18 | ENST00000595478.1 | ENSG00000237031.3 | -0.860703529 | -1.920749721 | 0.054763268 |
| PPP1R18 | ENST00000596091.1 | ENSG00000227733.4 | -0.932125097 | -2.097344266 | 0.035963111 |
| PPP1R18 | ENST00000596971.1 | ENSG00000269463.1 | 0.829301976 | 1.846279742 | 0.064851598 |
| PPP1R18 | ENST00000597169.1 | ENSG00000269720.1 | 0.882982386 | 1.986155537 | 0.047016056 |
| PPP1R18 | ENST00000597256.1 | ENSG00000267986.1 | 0.811870705 | 1.834040002 | 0.066648056 |
| PPP1R18 | ENST00000597906.1 | ENSG00000268566.1 | -0.858139352 | -1.895895662 | 0.057973843 |
| PPP1R18 | ENST00000600726.1 | ENSG00000267858.1 | 0.858012673 | 1.92783549 | 0.053875592 |
| PPP1R18 | ENST00000601735.1 | ENSG00000244513.2 | 0.813440674 | 1.822337595 | 0.068403777 |
| PPP1R18 | ENST00000602594.1 | ENSG00000269930.1 | -0.892298486 | -1.992117101 | 0.046358211 |
| PPP1R18 | ENST00000602949.1 | ENSG00000270030.1 | 0.887459977 | 1.977430573 | 0.047992981 |
| PPP1R18 | ENST00000604142.1 | ENSG00000271308.1 | 0.957255451 | 2.137685963 | 0.03254224 |
| PPP1R18 | ENST00000604183.1 | ENSG00000271185.1 | 0.907357956 | 2.070216424 | 0.038432082 |
| PPP1R18 | ENST00000605082.1 | ENSG00000270426.1 | 0.836888404 | 1.854682904 | 0.063641521 |
| PPP1R18 | ENST00000606277.1 | ENSG00000272145.1 | 0.863429872 | 1.941073457 | 0.052249368 |
| PPP1R18 | ENST00000606377.1 | ENSG00000272286.1 | -0.880361393 | -1.962910633 | 0.049656559 |
| PPP1R18 | ENST00000606441.1 | ENSG00000272277.1 | 0.902091254 | 2.048316214 | 0.040529025 |
| PPP1R18 | ENST00000606470.1 | ENSG00000271913.1 | 0.840613788 | 1.900142835 | 0.057414378 |
| PPP1R18 | ENST00000606743.1 | ENSG00000272221.1 | 0.974630308 | 2.167714947 | 0.030180381 |
| PPP1R18 | ENST00000606909.1 | ENSG00000271821.1 | 0.985098948 | 2.221686035 | 0.026304531 |
| PPP1R18 | ENST00000607148.1 | ENSG00000272477.1 | -0.81293866 | -1.81769973 | 0.069110039 |
| PPP1R18 | ENST00000607476.1 | ENSG00000272540.1 | 0.939791341 | 2.073829162 | 0.03809518 |
| PPP1R18 | ENST00000607943.1 | ENSG00000273188.1 | 0.930978729 | 2.047288827 | 0.040629734 |

| | | | | | |
|---|---|---|---|---|---|
| PPP1R18 | ENST00000608367.1 | ENSG00000273361.1 | 0.844349297 | 1.883688286 | 0.059607152 |
| PPP1R18 | ENST00000608489.1 | ENSG00000272716.1 | 0.846351207 | 1.891089559 | 0.05861239 |
| PPP1R18 | NR_003605.1 | ZFAS1 | 0.845342309 | 1.89847141 | 0.057634011 |
| PPP1R18 | NR_026774.1 | LINC00239 | 0.844680721 | 1.891234093 | 0.058593102 |
| PPP1R18 | NR_026802.1 | FAM74A4 | 0.867800189 | 1.940576488 | 0.052309668 |
| PPP1R18 | NR_026951.1 | LINC00324 | 0.83859189 | 1.833113695 | 0.066785665 |
| PPP1R18 | NR_027271.1 | CIRBP-AS1 | 0.818403511 | 1.860390711 | 0.062830268 |
| PPP1R18 | NR_028324.1 | LINC01002 | 0.85898476 | 1.914400407 | 0.055569014 |
| PPP1R18 | NR_036480.1 | VPS9D1-AS1 | 0.802268746 | 1.806559057 | 0.070831058 |
| PPP1R18 | NR_037169.1 | LOC100507547 | 0.994676979 | 2.220013432 | 0.026417856 |
| PPP1R18 | NR_037170.1 | LOC100507547 | 0.984159539 | 2.22327229 | 0.026197445 |
| PPP1R18 | NR_038421.1 | LINC01220 | 0.845730197 | 1.888452187 | 0.058965271 |
| PPP1R18 | NR_038923.1 | SSSCA1-AS1 | 0.854866127 | 1.914520558 | 0.055553675 |
| PPP1R18 | NR_045114.1 | PVRL3-AS1 | -0.934505153 | -2.073042345 | 0.038168339 |
| PPP1R18 | NR_072981.1 | LINC00957 | 0.978298947 | 2.198713631 | 0.027898291 |
| PPP1R18 | NR_072982.1 | LINC00957 | 0.978789746 | 2.174190399 | 0.029690839 |
| PPP1R18 | NR_105010.1 | LINC01333 | 0.864849427 | 1.94019711 | 0.05235574 |
| PPP1R18 | NR_109885.1 | RALY-AS1 | 0.957643236 | 2.103901694 | 0.035387023 |
| PPP1R18 | NR_109886.1 | RALY-AS1 | 0.930460586 | 2.083119793 | 0.037240306 |
| PPP1R18 | NR_110245.1 | LOC101929282 | -0.902304969 | -2.019191988 | 0.043467269 |
| PPP1R18 | NR_110630.1 | LOC101927478 | 0.918253321 | 2.071827008 | 0.038281577 |
| PPP1R18 | NR_110941.1 | MIR762HG | 0.919462419 | 2.029676127 | 0.042389471 |
| PPP1R18 | NR_110998.1 | FAM74A4 | 0.867800189 | 1.945810886 | 0.05167746 |
| PPP1R18 | NR_125957.1 | LOC101928626 | -0.913316251 | -2.043214548 | 0.041031204 |
| PPP1R18 | NR_126522.1 | EXOC3-AS1 | 0.874974888 | 1.946437774 | 0.051602176 |
| PPP1R18 | NR_134252.1 | LOC105379030 | 0.842589427 | 1.85938209 | 0.062972999 |
| PPP1R18 | NR_134520.1 | LOC727993 | 0.824295188 | 1.863482095 | 0.062394469 |
| PPP1R18 | NR_135024.1 | LOC105369747 | 0.845920682 | 1.910955547 | 0.056010294 |
| PPP1R18 | NR_135097.1 | LOC105369443 | -0.824171192 | -1.851049155 | 0.064162482 |
| PPP1R18 | NR_135584.1 | LOC101927596 | 0.834895706 | 1.839199159 | 0.065885898 |
| PPP1R18 | NR_144459.1 | ARSD-AS1 | 0.846436358 | 1.882763357 | 0.059732445 |
| PPP1R27 | ENST00000318291.4 | ENSG00000177406.4 | 0.84614551 | 1.866379689 | 0.061988263 |
| PPP1R27 | ENST00000412085.1 | ENSG00000233825.1 | 0.911880893 | 2.007015192 | 0.044748045 |
| PPP1R27 | ENST00000412759.1 | ENSG00000236933.1 | 0.964035395 | 2.173231726 | 0.029762881 |
| PPP1R27 | ENST00000412772.1 | ENSG00000231507.1 | 0.844137113 | 1.888919269 | 0.058902647 |
| PPP1R27 | ENST00000414740.2 | ENSG00000229646.2 | 0.860805153 | 1.883275508 | 0.059663041 |
| PPP1R27 | ENST00000415205.1 | ENSG00000182057.4 | 0.809097449 | 1.799787261 | 0.071894236 |
| PPP1R27 | ENST00000419662.1 | ENSG00000228265.1 | 0.803379721 | 1.795295445 | 0.07260664 |
| PPP1R27 | ENST00000426237.2 | ENSG00000235527.2 | 0.862611252 | 1.911022848 | 0.056001645 |
| PPP1R27 | ENST00000426302.1 | ENSG00000230454.1 | 0.814967783 | 1.830801235 | 0.067130216 |
| PPP1R27 | ENST00000426519.1 | ENSG00000234142.1 | 0.863146027 | 1.938281807 | 0.052588851 |
| PPP1R27 | ENST00000428765.1 | ENSG00000230107.1 | 0.857001322 | 1.962434252 | 0.049711948 |
| PPP1R27 | ENST00000429080.1 | ENSG00000233047.1 | -0.850882072 | -1.922728714 | 0.054514129 |
| PPP1R27 | ENST00000430920.1 | ENSG00000234203.1 | 0.928611145 | 2.092066365 | 0.036432579 |
| PPP1R27 | ENST00000433876.2 | ENSG00000228423.2 | 0.853068266 | 1.903139816 | 0.057022304 |
| PPP1R27 | ENST00000433905.2 | ENSG00000229299.2 | 0.953904589 | 2.159262633 | 0.030829797 |
| PPP1R27 | ENST00000434627.1 | ENSG00000230074.1 | 0.824970068 | 1.834434769 | 0.066589482 |
| PPP1R27 | ENST00000435892.1 | ENSG00000233635.2 | 0.838680066 | 1.877126392 | 0.060500773 |
| PPP1R27 | ENST00000438190.1 | ENSG00000227214.2 | 0.933423549 | 2.092227472 | 0.036418172 |
| PPP1R27 | ENST00000442829.1 | ENSG00000225284.1 | 0.805853551 | 1.782646581 | 0.074643852 |
| PPP1R27 | ENST00000447206.1 | ENSG00000230839.1 | 0.803217788 | 1.793551144 | 0.07288484 |

| | | | | | |
|---|---|---|---|---|---|
| PPP1R27 | ENST00000447343.2 | ENSG00000229299.2 | 0.931724616 | 2.071362408 | 0.038324941 |
| PPP1R27 | ENST00000453051.1 | ENSG00000229407.1 | 0.954632026 | 2.135837738 | 0.032692642 |
| PPP1R27 | ENST00000454530.1 | ENSG00000226649.1 | -0.823496311 | -1.82822605 | 0.067515633 |
| PPP1R27 | ENST00000457253.1 | ENSG00000225173.1 | 0.800122581 | 1.781810618 | 0.07478012 |
| PPP1R27 | ENST00000458154.1 | ENSG00000235578.1 | 0.816969369 | 1.830997407 | 0.06710093 |
| PPP1R27 | ENST00000458194.1 | ENSG00000226193.1 | 0.903312809 | 2.013701566 | 0.044040876 |
| PPP1R27 | ENST00000459985.1 | ENSG00000273066.1 | 0.86812246 | 1.957933813 | 0.05023778 |
| PPP1R27 | ENST00000466431.2 | ENSG00000254485.1 | 0.828993653 | 1.883454926 | 0.059638743 |
| PPP1R27 | ENST00000468165.1 | ENSG00000239480.1 | 0.975741678 | 2.178657661 | 0.029357109 |
| PPP1R27 | ENST00000489077.1 | ENSG00000244198.1 | 0.912092503 | 2.055068093 | 0.039872428 |
| PPP1R27 | ENST00000489557.2 | ENSG00000257045.1 | 0.838543126 | 1.858949667 | 0.063034273 |
| PPP1R27 | ENST00000490013.1 | ENSG00000184115.12 | 0.898333062 | 2.016811912 | 0.043715145 |
| PPP1R27 | ENST00000498358.1 | ENSG00000184115.12 | 0.829753091 | 1.864713512 | 0.062221572 |
| PPP1R27 | ENST00000498693.1 | ENSG00000244198.1 | 0.94209203 | 2.071259517 | 0.03833455 |
| PPP1R27 | ENST00000503723.1 | ENSG00000250472.1 | -0.907959742 | -2.037991367 | 0.041550796 |
| PPP1R27 | ENST00000505556.1 | ENSG00000249409.1 | 0.807130398 | 1.812804203 | 0.069862025 |
| PPP1R27 | ENST00000506100.1 | ENSG00000249409.1 | 0.803907739 | 1.781697525 | 0.074798571 |
| PPP1R27 | ENST00000506394.1 | ENSG00000251665.1 | 0.821108723 | 1.827121315 | 0.067681531 |
| PPP1R27 | ENST00000506791.1 | ENSG00000251131.1 | 0.833408708 | 1.848104011 | 0.064587298 |
| PPP1R27 | ENST00000508083.1 | ENSG00000249343.1 | 0.831096709 | 1.841610505 | 0.065532143 |
| PPP1R27 | ENST00000509036.1 | ENSG00000251131.1 | 0.893694572 | 1.992171222 | 0.046352275 |
| PPP1R27 | ENST00000509192.1 | ENSG00000250765.1 | 0.833881398 | 1.882196178 | 0.059809384 |
| PPP1R27 | ENST00000514411.1 | ENSG00000250882.1 | 0.807619782 | 1.809959955 | 0.070301997 |
| PPP1R27 | ENST00000517846.1 | ENSG00000254485.1 | 0.866331281 | 1.929700428 | 0.053643967 |
| PPP1R27 | ENST00000520603.1 | ENSG00000254001.1 | -0.828817152 | -1.873574196 | 0.060989137 |
| PPP1R27 | ENST00000521653.1 | ENSG00000253301.1 | 0.806851171 | 1.809337261 | 0.070398624 |
| PPP1R27 | ENST00000522547.1 | ENSG00000253430.1 | -0.853107301 | -1.901942653 | 0.057178652 |
| PPP1R27 | ENST00000522600.1 | ENSG00000246582.2 | 0.854310759 | 1.922878174 | 0.054495351 |
| PPP1R27 | ENST00000524942.1 | ENSG00000255553.1 | 0.817493664 | 1.832598724 | 0.066862268 |
| PPP1R27 | ENST00000527086.1 | ENSG00000255182.1 | 0.867696036 | 1.914548835 | 0.055550066 |
| PPP1R27 | ENST00000527757.1 | ENSG00000255109.1 | -0.801950767 | -1.79281918 | 0.073001842 |
| PPP1R27 | ENST00000528000.1 | ENSG00000254804.1 | 0.868853357 | 1.938808998 | 0.0525246 |
| PPP1R27 | ENST00000529247.1 | ENSG00000254741.1 | 0.80473294 | 1.811239893 | 0.070103726 |
| PPP1R27 | ENST00000543072.1 | ENSG00000256092.2 | -0.938001902 | -2.121335675 | 0.033893564 |
| PPP1R27 | ENST00000543275.1 | ENSG00000256944.1 | 0.975938489 | 2.189309418 | 0.028574358 |
| PPP1R27 | ENST00000543494.1 | ENSG00000256514.1 | 0.880747957 | 1.964331782 | 0.049491628 |
| PPP1R27 | ENST00000545177.3 | ENSG00000230438.5 | 0.848731107 | 1.905030193 | 0.056776146 |
| PPP1R27 | ENST00000549806.1 | ENSG00000257252.1 | 0.864728696 | 1.945983036 | 0.051656777 |
| PPP1R27 | ENST00000551135.1 | ENSG00000258294.1 | -0.843521123 | -1.887434555 | 0.059101901 |
| PPP1R27 | ENST00000558575.1 | ENSG00000259687.1 | 0.96487518 | 2.182328995 | 0.029085261 |
| PPP1R27 | ENST00000563018.1 | ENSG00000260193.1 | 0.884341316 | 1.977681113 | 0.047964692 |
| PPP1R27 | ENST00000563611.1 | ENSG00000261583.1 | 0.972578982 | 2.172756042 | 0.029798683 |
| PPP1R27 | ENST00000563806.1 | ENSG00000238045.5 | 0.836020192 | 1.831520365 | 0.067022911 |
| PPP1R27 | ENST00000564809.1 | ENSG00000261471.1 | 0.8480212 | 1.898597804 | 0.057617377 |
| PPP1R27 | ENST00000565823.1 | ENSG00000260686.1 | -0.831631551 | -1.856214599 | 0.063422976 |
| PPP1R27 | ENST00000565829.1 | ENSG00000260148.1 | 0.852012913 | 1.922362543 | 0.054560155 |
| PPP1R27 | ENST00000570843.1 | ENSG00000261889.1 | 0.901218451 | 2.020547616 | 0.043326616 |
| PPP1R27 | ENST00000570929.1 | ENSG00000262223.2 | 0.867983177 | 1.973352163 | 0.048455454 |
| PPP1R27 | ENST00000574460.1 | ENSG00000263051.1 | 0.884498389 | 1.946579777 | 0.051585135 |
| PPP1R27 | ENST00000576086.1 | ENSG00000262823.1 | 0.83600179 | 1.86740989 | 0.06184437 |
| PPP1R27 | ENST00000577853.1 | ENSG00000264207.1 | 0.816200149 | 1.806221712 | 0.070883715 |

| | | | | | |
|---|---|---|---|---|---|
| PPP1R27 | ENST00000578800.1 | ENSG00000264235.1 | 0.947894858 | 2.109391956 | 0.034910763 |
| PPP1R27 | ENST00000578936.1 | ENSG00000265547.1 | 0.88847081 | 1.991582792 | 0.046416853 |
| PPP1R27 | ENST00000582044.1 | ENSG00000263715.2 | 0.870407984 | 1.937348409 | 0.052702769 |
| PPP1R27 | ENST00000586051.1 | ENSG00000267576.1 | 0.865326836 | 1.935363944 | 0.052945651 |
| PPP1R27 | ENST00000588182.2 | ENSG00000267453.2 | 0.857173366 | 1.913033965 | 0.055743704 |
| PPP1R27 | ENST00000588402.1 | ENSG00000267006.1 | -0.846886547 | -1.882770239 | 0.059731512 |
| PPP1R27 | ENST00000589673.1 | ENSG00000267755.1 | 0.902826389 | 2.005442904 | 0.044915719 |
| PPP1R27 | ENST00000591174.1 | ENSG00000267289.1 | 0.930594042 | 2.083448268 | 0.037210382 |
| PPP1R27 | ENST00000592413.1 | ENSG00000266933.1 | 0.871557905 | 1.942206968 | 0.05211205 |
| PPP1R27 | ENST00000592518.1 | ENSG00000267786.1 | 0.823119714 | 1.835923757 | 0.066368934 |
| PPP1R27 | ENST00000593139.1 | ENSG00000267042.1 | 0.845851502 | 1.877786373 | 0.060410395 |
| PPP1R27 | ENST00000594590.2 | ENSG00000268199.2 | 0.947672983 | 2.138553721 | 0.032471829 |
| PPP1R27 | ENST00000595955.1 | ENSG00000268401.1 | 0.902512432 | 2.061206241 | 0.039283368 |
| PPP1R27 | ENST00000596887.1 | ENSG00000237031.3 | -0.936570774 | -2.097514793 | 0.035948029 |
| PPP1R27 | ENST00000597169.1 | ENSG00000269720.1 | 0.828108483 | 1.844709597 | 0.065079794 |
| PPP1R27 | ENST00000597256.1 | ENSG00000267986.1 | 0.888259369 | 1.997556484 | 0.045764765 |
| PPP1R27 | ENST00000598092.1 | ENSG00000228065.6 | -0.879690839 | -1.972441139 | 0.04855927 |
| PPP1R27 | ENST00000599352.1 | ENSG00000240401.4 | -0.88513739 | -1.995107708 | 0.046031134 |
| PPP1R27 | ENST00000600242.1 | ENSG00000269583.1 | 0.858868217 | 1.922566369 | 0.054534531 |
| PPP1R27 | ENST00000600534.1 | ENSG00000267858.1 | 0.8324926 | 1.869551996 | 0.061546057 |
| PPP1R27 | ENST00000600726.1 | ENSG00000267858.1 | 0.940525321 | 2.122298717 | 0.033812661 |
| PPP1R27 | ENST00000601033.1 | ENSG00000268401.1 | 0.896043663 | 1.99615614 | 0.04591693 |
| PPP1R27 | ENST00000602532.1 | ENSG00000270091.1 | 0.889545395 | 1.99798859 | 0.045717897 |
| PPP1R27 | ENST00000604142.1 | ENSG00000271308.1 | 0.921064841 | 2.074241485 | 0.038056889 |
| PPP1R27 | ENST00000604183.1 | ENSG00000271185.1 | 0.870557325 | 1.923633439 | 0.054400547 |
| PPP1R27 | ENST00000606277.1 | ENSG00000272145.1 | 0.838390114 | 1.882214759 | 0.059806863 |
| PPP1R27 | ENST00000607052.1 | ENSG00000271870.1 | -0.85771133 | -1.918287909 | 0.055074515 |
| PPP1R27 | ENST00000607224.1 | ENSG00000272521.1 | 0.913688168 | 2.054712861 | 0.039906747 |
| PPP1R27 | ENST00000607476.1 | ENSG00000272540.1 | 0.954515145 | 2.130149694 | 0.033159257 |
| PPP1R27 | ENST00000607943.1 | ENSG00000273188.1 | 0.940825295 | 2.118424694 | 0.034139116 |
| PPP1R27 | ENST00000608489.1 | ENSG00000272716.1 | 0.839511237 | 1.891546066 | 0.058551488 |
| PPP1R27 | ENST00000608677.1 | ENSG00000273350.1 | 0.896150384 | 2.016285524 | 0.043770128 |
| PPP1R27 | ENST00000608940.1 | ENSG00000272763.1 | 0.841701481 | 1.900059378 | 0.057425328 |
| PPP1R27 | ENST00000609113.1 | ENSG00000272827.1 | 0.919291991 | 2.068249042 | 0.038616611 |
| PPP1R27 | ENST00000609976.1 | ENSG00000272582.1 | 0.822287536 | 1.873048013 | 0.061061755 |
| PPP1R27 | ENST00000610270.1 | ENSG00000272576.1 | -0.886568104 | -1.993017318 | 0.046259551 |
| PPP1R27 | NR_003604.2 | ZFAS1 | 0.907580538 | 2.023778675 | 0.042992929 |
| PPP1R27 | NR_003605.1 | ZFAS1 | 0.847586962 | 1.92681668 | 0.05400248 |
| PPP1R27 | NR_003606.2 | ZFAS1 | 0.904279606 | 2.008496475 | 0.04459056 |
| PPP1R27 | NR_026802.1 | FAM74A4 | 0.901943437 | 2.002292978 | 0.045253231 |
| PPP1R27 | NR_026951.1 | LINC00324 | 0.806117796 | 1.808606436 | 0.070512168 |
| PPP1R27 | NR_027052.1 | THAP7-AS1 | 0.966357961 | 2.147845043 | 0.031726067 |
| PPP1R27 | NR_027271.1 | CIRBP-AS1 | 0.939045036 | 2.103988694 | 0.035379433 |
| PPP1R27 | NR_027334.2 | MZF1-AS1 | 0.83231639 | 1.842315762 | 0.065428976 |
| PPP1R27 | NR_036480.1 | VPS9D1-AS1 | 0.830381425 | 1.860626532 | 0.062796935 |
| PPP1R27 | NR_036658.1 | ZFAS1 | 0.902263918 | 2.047645345 | 0.040594763 |
| PPP1R27 | NR_038421.1 | LINC01220 | 0.965078104 | 2.165340075 | 0.030361652 |
| PPP1R27 | NR_038923.1 | SSSCA1-AS1 | 0.947762183 | 2.128018356 | 0.033335562 |
| PPP1R27 | NR_040096.1 | LOC643339 | 0.879724418 | 2.004921127 | 0.04497148 |
| PPP1R27 | NR_045114.1 | PVRL3-AS1 | -0.866608366 | -1.938904222 | 0.052513002 |
| PPP1R27 | NR_046871.1 | LINC00333 | -0.81861484 | -1.812311135 | 0.069938135 |

| | | | | | |
|---|---|---|---|---|---|
| PPP1R27 | NR_105010.1 | LINC01333 | 0.87638477 | 1.951353448 | 0.051015015 |
| PPP1R27 | NR_108106.1 | LINC01135 | 0.851941363 | 1.897343399 | 0.057782631 |
| PPP1R27 | NR_109831.1 | RASSF1-AS1 | 0.825884132 | 1.802192944 | 0.071515054 |
| PPP1R27 | NR_109886.1 | RALY-AS1 | 0.812370081 | 1.840314521 | 0.065722075 |
| PPP1R27 | NR_110245.1 | LOC101929282 | -0.834307082 | -1.83278016 | 0.066835271 |
| PPP1R27 | NR_110556.1 | LOC102724890 | -0.856196441 | -1.906886091 | 0.05653534 |
| PPP1R27 | NR_110630.1 | LOC101927478 | 0.831164155 | 1.883705832 | 0.059604777 |
| PPP1R27 | NR_110941.1 | MIR762HG | 0.86604146 | 1.94448192 | 0.051837364 |
| PPP1R27 | NR_110998.1 | FAM74A4 | 0.901943437 | 2.006274575 | 0.044826961 |
| PPP1R27 | NR_111951.1 | LINC00869 | 0.944100003 | 2.119823663 | 0.034020919 |
| PPP1R27 | NR_111952.1 | LINC00869 | 0.963629048 | 2.146469533 | 0.031835537 |
| PPP1R27 | NR_111953.1 | LINC00869 | 0.939527488 | 2.087500112 | 0.036842951 |
| PPP1R27 | NR_117097.1 | LINC01353 | 0.844137113 | 1.865619551 | 0.062094612 |
| PPP1R27 | NR_117098.1 | LINC01353 | 0.840287094 | 1.869714477 | 0.061523478 |
| PPP1R27 | NR_126522.1 | EXOC3-AS1 | 0.882840238 | 1.977708666 | 0.047961582 |
| PPP1R27 | NR_130143.1 | LOC104968399 | 0.955216786 | 2.144963144 | 0.031955794 |
| PPP1R27 | NR_135024.1 | LOC105369747 | 0.868020938 | 1.936918024 | 0.052755365 |
| PPP1R27 | NR_135041.1 | LOC101927038 | 0.863256076 | 1.908521432 | 0.056323856 |
| PPP1R27 | NR_135584.1 | LOC101927596 | 0.865305834 | 1.94607142 | 0.051646161 |
| PPP1R27 | NR_136215.1 | VCAN-AS1 | -0.814153417 | -1.806785477 | 0.070795734 |
| PPP1R35 | ENST00000318291.4 | ENSG00000177406.4 | 0.831020049 | 1.861697282 | 0.062645772 |
| PPP1R35 | ENST00000417260.1 | ENSG00000231734.4 | -0.956768382 | -2.156158577 | 0.031071285 |
| PPP1R35 | ENST00000418080.1 | ENSG00000224091.1 | 0.854371377 | 1.890312953 | 0.058716117 |
| PPP1R35 | ENST00000419103.1 | ENSG00000227014.1 | 0.823667887 | 1.837611441 | 0.066119682 |
| PPP1R35 | ENST00000419662.1 | ENSG00000228265.1 | 0.832762595 | 1.861469902 | 0.062677847 |
| PPP1R35 | ENST00000423428.1 | ENSG00000224048.1 | -0.912541102 | -2.036160197 | 0.041734272 |
| PPP1R35 | ENST00000425124.1 | ENSG00000232336.1 | 0.951819671 | 2.147406055 | 0.031760969 |
| PPP1R35 | ENST00000425624.1 | ENSG00000223779.4 | 0.817214577 | 1.828836895 | 0.067424046 |
| PPP1R35 | ENST00000431730.1 | ENSG00000237401.2 | 0.853538711 | 1.902961043 | 0.057045629 |
| PPP1R35 | ENST00000435287.1 | ENSG00000227220.1 | 0.875573989 | 1.963400806 | 0.04959962 |
| PPP1R35 | ENST00000438190.1 | ENSG00000227214.2 | 0.832534326 | 1.853147484 | 0.063861222 |
| PPP1R35 | ENST00000440595.1 | ENSG00000228265.1 | 0.811969829 | 1.804830881 | 0.07110115 |
| PPP1R35 | ENST00000446816.1 | ENSG00000204685.5 | 0.810065427 | 1.807542852 | 0.07067768 |
| PPP1R35 | ENST00000451507.1 | ENSG00000229539.1 | 0.857644725 | 1.886855016 | 0.059179829 |
| PPP1R35 | ENST00000452176.1 | ENSG00000223659.1 | -0.92428337 | -2.073056482 | 0.038167023 |
| PPP1R35 | ENST00000454100.1 | ENSG00000236943.2 | 0.902457379 | 2.027483243 | 0.042613016 |
| PPP1R35 | ENST00000455788.1 | ENSG00000236263.1 | 0.92287341 | 2.075544985 | 0.037936054 |
| PPP1R35 | ENST00000457371.1 | ENSG00000237401.2 | 0.87894743 | 1.954120259 | 0.050686997 |
| PPP1R35 | ENST00000458154.1 | ENSG00000235578.1 | 0.875799526 | 1.968953686 | 0.04895841 |
| PPP1R35 | ENST00000480919.1 | ENSG00000242474.1 | 0.812346588 | 1.818172539 | 0.069037766 |
| PPP1R35 | ENST00000489077.1 | ENSG00000244198.1 | 0.870219921 | 1.936534429 | 0.05280228 |
| PPP1R35 | ENST00000489690.1 | ENSG00000243944.1 | -0.850974432 | -1.900935489 | 0.057310463 |
| PPP1R35 | ENST00000494509.1 | ENSG00000240095.1 | 0.860138941 | 1.911033181 | 0.056000317 |
| PPP1R35 | ENST00000500496.2 | ENSG00000245479.2 | 0.902155318 | 2.002829811 | 0.045195559 |
| PPP1R35 | ENST00000503723.1 | ENSG00000250472.1 | -0.804636917 | -1.816839534 | 0.069241687 |
| PPP1R35 | ENST00000504578.1 | ENSG00000251513.1 | -0.842008292 | -1.868990078 | 0.061624195 |
| PPP1R35 | ENST00000506791.1 | ENSG00000251131.1 | 0.907227351 | 2.030802272 | 0.042275057 |
| PPP1R35 | ENST00000509036.1 | ENSG00000251131.1 | 0.800880425 | 1.790407885 | 0.073388364 |
| PPP1R35 | ENST00000509192.1 | ENSG00000250765.1 | 0.942755061 | 2.119347583 | 0.034061103 |
| PPP1R35 | ENST00000509453.1 | ENSG00000249145.1 | 0.90688269 | 2.017457758 | 0.043647765 |
| PPP1R35 | ENST00000515128.1 | ENSG00000248215.1 | -0.917540351 | -2.049695154 | 0.040394188 |

| | | | | | |
|---|---|---|---|---|---|
| PPP1R35 | ENST00000520603.1 | ENSG00000254001.1 | -0.920339584 | -2.041466482 | 0.041204482 |
| PPP1R35 | ENST00000532454.1 | ENSG00000255120.1 | 0.840631457 | 1.905538382 | 0.056710123 |
| PPP1R35 | ENST00000534178.1 | ENSG00000255120.1 | 0.887196686 | 1.980007806 | 0.047702652 |
| PPP1R35 | ENST00000537032.1 | ENSG00000255933.1 | 0.815265201 | 1.817540682 | 0.069134365 |
| PPP1R35 | ENST00000537850.1 | ENSG00000251002.3 | 0.838076243 | 1.852272895 | 0.063986646 |
| PPP1R35 | ENST00000545572.1 | ENSG00000255680.1 | 0.845458998 | 1.920209141 | 0.054831487 |
| PPP1R35 | ENST00000547834.1 | ENSG00000258325.1 | 0.837753303 | 1.869841577 | 0.061505821 |
| PPP1R35 | ENST00000548722.2 | ENSG00000257194.2 | -0.824290696 | -1.844241098 | 0.065148011 |
| PPP1R35 | ENST00000550263.1 | ENSG00000257605.1 | 0.983622158 | 2.206530265 | 0.027346894 |
| PPP1R35 | ENST00000551699.1 | ENSG00000257467.1 | -0.840739767 | -1.865933375 | 0.062050687 |
| PPP1R35 | ENST00000554431.1 | ENSG00000258616.1 | -0.82493189 | -1.834328167 | 0.066605296 |
| PPP1R35 | ENST00000554859.1 | ENSG00000259088.1 | 0.899658125 | 2.03053625 | 0.04230206 |
| PPP1R35 | ENST00000557602.1 | ENSG00000258616.1 | -0.821495031 | -1.824001238 | 0.068151885 |
| PPP1R35 | ENST00000559959.1 | ENSG00000259396.1 | 0.878329454 | 1.955540038 | 0.050519362 |
| PPP1R35 | ENST00000560963.1 | ENSG00000259370.1 | 0.859943795 | 1.918763969 | 0.055014212 |
| PPP1R35 | ENST00000562995.1 | ENSG00000261253.1 | 0.883357855 | 1.980487003 | 0.047648832 |
| PPP1R35 | ENST00000567395.1 | ENSG00000261090.1 | 0.905050044 | 2.034430069 | 0.041908253 |
| PPP1R35 | ENST00000569981.1 | ENSG00000238045.5 | 0.92449757 | 2.063139035 | 0.03909942 |
| PPP1R35 | ENST00000570493.2 | ENSG00000261898.2 | 0.964639224 | 2.148321271 | 0.031688242 |
| PPP1R35 | ENST00000582558.1 | ENSG00000264569.1 | 0.888142542 | 1.984182607 | 0.047235487 |
| PPP1R35 | ENST00000584705.1 | ENSG00000264569.1 | 0.899058864 | 2.032565966 | 0.042096393 |
| PPP1R35 | ENST00000585559.1 | ENSG00000267117.1 | 0.953231787 | 2.146927545 | 0.03179905 |
| PPP1R35 | ENST00000585810.1 | ENSG00000236172.2 | 0.866341898 | 1.924685371 | 0.054268732 |
| PPP1R35 | ENST00000586694.1 | ENSG00000267141.1 | 0.817260465 | 1.819612588 | 0.068818023 |
| PPP1R35 | ENST00000588380.1 | ENSG00000266990.1 | 0.889147536 | 1.980878958 | 0.047604849 |
| PPP1R35 | ENST00000589395.1 | ENSG00000267143.1 | 0.853917646 | 1.89204647 | 0.05848479 |
| PPP1R35 | ENST00000592400.1 | ENSG00000267735.1 | 0.855039638 | 1.947779347 | 0.051441371 |
| PPP1R35 | ENST00000592720.1 | ENSG00000267232.1 | 0.932453373 | 2.062716452 | 0.039139576 |
| PPP1R35 | ENST00000592816.1 | ENSG00000236172.2 | 0.891086865 | 2.016839654 | 0.043712249 |
| PPP1R35 | ENST00000595478.1 | ENSG00000237031.3 | -0.874657692 | -1.976121383 | 0.048141031 |
| PPP1R35 | ENST00000596091.1 | ENSG00000227733.4 | -0.903168216 | -2.047700736 | 0.040589332 |
| PPP1R35 | ENST00000597169.1 | ENSG00000269720.1 | 0.811912141 | 1.844522912 | 0.06510697 |
| PPP1R35 | ENST00000599259.1 | ENSG00000269352.1 | 0.824514405 | 1.835309724 | 0.066459811 |
| PPP1R35 | ENST00000600726.1 | ENSG00000267858.1 | 0.826671694 | 1.842544067 | 0.065395607 |
| PPP1R35 | ENST00000601692.1 | ENSG00000267874.1 | -0.855536446 | -1.918746571 | 0.055016415 |
| PPP1R35 | ENST00000602594.1 | ENSG00000269930.1 | -0.892781035 | -2.010539817 | 0.044374086 |
| PPP1R35 | ENST00000602949.1 | ENSG00000270030.1 | 0.913314095 | 2.049524603 | 0.040410845 |
| PPP1R35 | ENST00000604142.1 | ENSG00000271308.1 | 0.873595456 | 1.961688405 | 0.049798773 |
| PPP1R35 | ENST00000605082.1 | ENSG00000270426.1 | 0.914855285 | 2.039545526 | 0.041395612 |
| PPP1R35 | ENST00000606377.1 | ENSG00000272286.1 | -0.863183056 | -1.921468818 | 0.05467263 |
| PPP1R35 | ENST00000606470.1 | ENSG00000271913.1 | 0.917837768 | 2.023392094 | 0.043032739 |
| PPP1R35 | ENST00000606743.1 | ENSG00000272221.1 | 0.958974402 | 2.162071789 | 0.030612643 |
| PPP1R35 | ENST00000606909.1 | ENSG00000271821.1 | 0.893974465 | 1.990875148 | 0.046494615 |
| PPP1R35 | ENST00000607148.1 | ENSG00000272477.1 | -0.876872183 | -1.940219569 | 0.052353011 |
| PPP1R35 | ENST00000607476.1 | ENSG00000272540.1 | 0.821833455 | 1.83443719 | 0.066589123 |
| PPP1R35 | ENST00000607943.1 | ENSG00000273188.1 | 0.868412348 | 1.952591613 | 0.050868006 |
| PPP1R35 | ENST00000608258.1 | ENSG00000229042.2 | -0.888352635 | -2.007385441 | 0.044708638 |
| PPP1R35 | ENST00000608489.1 | ENSG00000272716.1 | 0.822826832 | 1.857969598 | 0.063173332 |
| PPP1R35 | ENST00000609813.1 | ENSG00000272719.1 | 0.860580564 | 1.926982497 | 0.053981811 |
| PPP1R35 | NR_003605.1 | ZFAS1 | 0.859362322 | 1.919544385 | 0.054915475 |
| PPP1R35 | NR_028324.1 | LINC01002 | 0.965722409 | 2.178275841 | 0.029385507 |

| | | | | | |
|---|---|---|---|---|---|
| PPP1R35 | NR_036480.1 | VPS9D1-AS1 | 0.834846073 | 1.878604012 | 0.060298583 |
| PPP1R35 | NR_037169.1 | LOC100507547 | 0.955742274 | 2.134276885 | 0.032820122 |
| PPP1R35 | NR_037170.1 | LOC100507547 | 0.961867346 | 2.140796479 | 0.032290454 |
| PPP1R35 | NR_045114.1 | PVRL3-AS1 | -0.819953898 | -1.806604219 | 0.070824011 |
| PPP1R35 | NR_072981.1 | LINC00957 | 0.962483551 | 2.135584069 | 0.032713331 |
| PPP1R35 | NR_072982.1 | LINC00957 | 0.962301737 | 2.167935647 | 0.030163583 |
| PPP1R35 | NR_105010.1 | LINC01333 | 0.810085641 | 1.800551369 | 0.07177362 |
| PPP1R35 | NR_109885.1 | RALY-AS1 | 0.840821796 | 1.883290706 | 0.059660982 |
| PPP1R35 | NR_109886.1 | RALY-AS1 | 0.854226514 | 1.908893672 | 0.05627581 |
| PPP1R35 | NR_110245.1 | LOC101929282 | -0.812124438 | -1.811839555 | 0.070010991 |
| PPP1R35 | NR_110630.1 | LOC101927478 | 0.863081345 | 1.917438297 | 0.055182273 |
| PPP1R35 | NR_120371.1 | LINC01585 | 0.894859321 | 2.007041236 | 0.044745272 |
| PPP1R35 | NR_125957.1 | LOC101928626 | -0.92428337 | -2.051870582 | 0.040182242 |
| PPP1R35 | NR_126166.1 | FAM74A7 | 0.815895331 | 1.830136011 | 0.067229603 |
| PPP1R35 | NR_134325.1 | LOC102723672 | 0.859266242 | 1.927307531 | 0.053941316 |
| PPP1R35 | NR_144459.1 | ARSD-AS1 | 0.937925159 | 2.103805709 | 0.035395399 |
| PRAMEF5 | ENST00000416861.1 | ENSG00000227308.2 | 0.820652829 | 1.824793339 | 0.068032221 |
| PRAMEF5 | ENST00000419103.1 | ENSG00000227014.1 | 0.963665564 | 2.136145689 | 0.032667541 |
| PRAMEF5 | ENST00000425124.1 | ENSG00000232336.1 | 0.881273962 | 1.982158036 | 0.047461556 |
| PRAMEF5 | ENST00000425624.1 | ENSG00000223779.4 | 0.810714299 | 1.799198031 | 0.071987361 |
| PRAMEF5 | ENST00000432314.1 | ENSG00000231532.1 | 0.904316319 | 2.037486551 | 0.041601308 |
| PRAMEF5 | ENST00000434790.1 | ENSG00000240040.1 | -0.807545286 | -1.807366507 | 0.070705153 |
| PRAMEF5 | ENST00000440492.1 | ENSG00000233975.1 | 0.897785489 | 2.029601754 | 0.042397037 |
| PRAMEF5 | ENST00000441160.1 | ENSG00000228437.1 | -0.944918732 | -2.098064971 | 0.035899406 |
| PRAMEF5 | ENST00000454100.1 | ENSG00000236943.2 | 0.987445715 | 2.220178233 | 0.026406671 |
| PRAMEF5 | ENST00000455788.1 | ENSG00000236263.1 | 0.809265514 | 1.820225537 | 0.068724666 |
| PRAMEF5 | ENST00000480919.1 | ENSG00000242474.1 | 0.833275707 | 1.864454377 | 0.062257923 |
| PRAMEF5 | ENST00000500496.2 | ENSG00000245479.2 | 0.84051262 | 1.888055213 | 0.059018539 |
| PRAMEF5 | ENST00000507525.1 | ENSG00000250431.1 | -0.856956825 | -1.909800072 | 0.05615896 |
| PRAMEF5 | ENST00000515205.1 | ENSG00000251580.1 | 0.800329027 | 1.776084036 | 0.075719067 |
| PRAMEF5 | ENST00000518473.1 | ENSG00000253985.1 | 0.89037664 | 2.013323311 | 0.044080628 |
| PRAMEF5 | ENST00000519062.1 | ENSG00000253658.1 | -0.812996473 | -1.808023787 | 0.070602799 |
| PRAMEF5 | ENST00000521030.1 | ENSG00000253802.1 | 0.802477072 | 1.818858787 | 0.068932977 |
| PRAMEF5 | ENST00000523859.1 | ENSG00000251136.4 | 0.826667013 | 1.84977711 | 0.064345681 |
| PRAMEF5 | ENST00000524286.1 | ENSG00000253658.1 | -0.895457781 | -1.980617682 | 0.047634164 |
| PRAMEF5 | ENST00000532680.1 | ENSG00000255458.1 | 0.925644272 | 2.062581334 | 0.039152423 |
| PRAMEF5 | ENST00000550263.1 | ENSG00000257605.1 | 0.837302513 | 1.863370383 | 0.062410174 |
| PRAMEF5 | ENST00000554679.1 | ENSG00000258837.1 | 0.811619954 | 1.847818216 | 0.064628645 |
| PRAMEF5 | ENST00000554859.1 | ENSG00000259088.1 | 0.92240484 | 2.054515807 | 0.039925795 |
| PRAMEF5 | ENST00000559959.1 | ENSG00000259396.1 | 0.897000681 | 2.01680062 | 0.043716324 |
| PRAMEF5 | ENST00000567395.1 | ENSG00000261090.1 | 0.839003722 | 1.877463593 | 0.060454583 |
| PRAMEF5 | ENST00000584705.1 | ENSG00000264569.1 | 0.876198838 | 1.96710374 | 0.049171252 |
| PRAMEF5 | ENST00000588945.1 | ENSG00000267275.1 | 0.879497635 | 2.005187402 | 0.044943016 |
| PRAMEF5 | ENST00000590989.1 | ENSG00000267011.1 | 0.82464778 | 1.841039709 | 0.06561574 |
| PRAMEF5 | ENST00000592720.1 | ENSG00000267232.1 | 0.876452617 | 1.939755515 | 0.052409409 |
| PRAMEF5 | ENST00000594091.1 | ENSG00000232732.5 | 0.814794064 | 1.829504703 | 0.067324035 |
| PRAMEF5 | ENST00000597530.1 | ENSG00000228401.3 | 0.935342999 | 2.084137217 | 0.037147688 |
| PRAMEF5 | ENST00000599143.1 | ENSG00000269349.1 | 0.828526679 | 1.882761132 | 0.059732747 |
| PRAMEF5 | ENST00000601692.1 | ENSG00000267874.1 | -0.845774097 | -1.892154526 | 0.058470395 |
| PRAMEF5 | ENST00000602949.1 | ENSG00000270030.1 | 0.837524678 | 1.855415113 | 0.063536971 |
| PRAMEF5 | ENST00000605082.1 | ENSG00000270426.1 | 0.960712653 | 2.161771279 | 0.03063581 |

| | | | | | |
|---|---|---|---|---|---|
| PRAMEF5 | ENST00000606743.1 | ENSG00000272221.1 | 0.851199656 | 1.900082815 | 0.057422253 |
| PRAMEF5 | ENST00000607284.1 | ENSG00000272389.1 | 0.836799161 | 1.895193653 | 0.058066751 |
| PRAMEF5 | ENST00000610161.1 | ENSG00000273059.1 | 0.86796771 | 1.986759526 | 0.046949052 |
| PRAMEF5 | NR_028324.1 | LINC01002 | 0.925445691 | 2.054457658 | 0.039931417 |
| PRAMEF5 | NR_033371.1 | CDRT7 | 0.82559642 | 1.83010563 | 0.067234145 |
| PRAMEF5 | NR_037170.1 | LOC100507547 | 0.827823795 | 1.892674285 | 0.058401198 |
| PRAMEF5 | NR_046369.1 | LOC100131626 | 0.906039748 | 2.018082698 | 0.043582649 |
| PRAMEF5 | NR_046370.1 | LOC100131626 | 0.845279701 | 1.899771829 | 0.057463069 |
| PRAMEF5 | NR_108068.1 | LINC00836 | 0.810120843 | 1.804747486 | 0.071114205 |
| PRAMEF5 | NR_109770.1 | TONSL-AS1 | 0.958598163 | 2.12817137 | 0.033322878 |
| PRAMEF5 | NR_120371.1 | LINC01585 | 0.824899022 | 1.837596882 | 0.066121829 |
| PRAMEF5 | NR_126166.1 | FAM74A7 | 0.837087658 | 1.869491217 | 0.061554504 |
| PRAMEF5 | NR_134265.1 | LINC02103 | 0.821725185 | 1.847096597 | 0.064733142 |
| PRAMEF5 | NR_134325.1 | LOC102723672 | 0.994020153 | 2.235657123 | 0.02537424 |
| PRAMEF5 | NR_135132.1 | HSPC324 | 0.93552845 | 2.109163299 | 0.034930489 |
| PRKACA | ENST00000318291.4 | ENSG00000177406.4 | 0.846203969 | 1.899097272 | 0.057551688 |
| PRKACA | ENST00000412348.1 | ENSG00000228959.1 | -0.936052355 | -2.078715834 | 0.037643477 |
| PRKACA | ENST00000418080.1 | ENSG00000224091.1 | 0.860261399 | 1.914230158 | 0.055590754 |
| PRKACA | ENST00000418387.1 | ENSG00000235056.1 | -0.838690152 | -1.890049337 | 0.058751362 |
| PRKACA | ENST00000420365.1 | ENSG00000225214.1 | 0.843787012 | 1.889640339 | 0.058806078 |
| PRKACA | ENST00000422017.1 | ENSG00000232227.1 | -0.921902101 | -2.049164778 | 0.040446005 |
| PRKACA | ENST00000433344.1 | ENSG00000234083.1 | -0.817722125 | -1.827483055 | 0.067627172 |
| PRKACA | ENST00000433550.1 | ENSG00000232227.1 | -0.880821107 | -2.00366383 | 0.045106083 |
| PRKACA | ENST00000435733.1 | ENSG00000226377.1 | 0.835221458 | 1.862723047 | 0.062501242 |
| PRKACA | ENST00000436515.1 | ENSG00000224521.1 | -0.883340118 | -1.988597019 | 0.046745701 |
| PRKACA | ENST00000438623.1 | ENSG00000224521.1 | -0.826937136 | -1.846639456 | 0.064799412 |
| PRKACA | ENST00000448748.1 | ENSG00000231238.1 | -0.900348854 | -2.007920677 | 0.044651722 |
| PRKACA | ENST00000449154.1 | ENSG00000226969.1 | 0.802032736 | 1.782943786 | 0.074595455 |
| PRKACA | ENST00000455788.1 | ENSG00000236263.1 | 0.827465307 | 1.851616249 | 0.064080948 |
| PRKACA | ENST00000457115.1 | ENSG00000227245.1 | 0.804380034 | 1.790460957 | 0.073379838 |
| PRKACA | ENST00000485338.1 | ENSG00000239641.1 | -0.873015032 | -1.949491751 | 0.051236727 |
| PRKACA | ENST00000489690.1 | ENSG00000243944.1 | -0.826146267 | -1.844417701 | 0.06512229 |
| PRKACA | ENST00000502467.1 | ENSG00000250530.1 | -0.876679563 | -1.945907845 | 0.05166581 |
| PRKACA | ENST00000503505.1 | ENSG00000248629.1 | -0.847438669 | -1.904646348 | 0.056826058 |
| PRKACA | ENST00000505498.1 | ENSG00000250908.1 | 0.848203607 | 1.912107366 | 0.055862424 |
| PRKACA | ENST00000515128.1 | ENSG00000248215.1 | -0.873972837 | -1.940536727 | 0.052314495 |
| PRKACA | ENST00000520749.1 | ENSG00000253717.1 | -0.825597298 | -1.855662463 | 0.063501684 |
| PRKACA | ENST00000521307.1 | ENSG00000253177.1 | 0.856433042 | 1.923420814 | 0.054427222 |
| PRKACA | ENST00000522600.1 | ENSG00000246582.2 | 0.841093119 | 1.864536168 | 0.062246447 |
| PRKACA | ENST00000522704.1 | ENSG00000254135.1 | 0.817327798 | 1.822315446 | 0.068407136 |
| PRKACA | ENST00000528887.1 | ENSG00000254501.1 | 0.897051321 | 1.990607486 | 0.046524057 |
| PRKACA | ENST00000539963.1 | ENSG00000256116.1 | 0.828286577 | 1.831425137 | 0.067037112 |
| PRKACA | ENST00000547834.1 | ENSG00000258325.1 | 0.895008339 | 1.982619645 | 0.047409931 |
| PRKACA | ENST00000549683.1 | ENSG00000257953.1 | 0.90613067 | 2.045293894 | 0.040825892 |
| PRKACA | ENST00000549878.1 | ENSG00000257284.1 | 0.811194632 | 1.799060413 | 0.072009125 |
| PRKACA | ENST00000561699.1 | ENSG00000259813.1 | 0.817231345 | 1.860419533 | 0.062826193 |
| PRKACA | ENST00000565823.1 | ENSG00000260686.1 | -0.854296101 | -1.920276933 | 0.054822928 |
| PRKACA | ENST00000570493.2 | ENSG00000261898.2 | 0.823911012 | 1.847223636 | 0.064714735 |
| PRKACA | ENST00000570512.1 | ENSG00000262768.1 | 0.825694374 | 1.84295262 | 0.065335929 |
| PRKACA | ENST00000570974.1 | ENSG00000263300.1 | 0.933414936 | 2.061468434 | 0.039258372 |
| PRKACA | ENST00000576554.1 | ENSG00000262413.1 | 0.935208253 | 2.100206978 | 0.035710638 |

| | | | | | |
|---|---|---|---|---|---|
| PRKACA | ENST00000577176.1 | ENSG00000262823.1 | 0.884922109 | 1.974695793 | 0.048302681 |
| PRKACA | ENST00000580729.1 | ENSG00000266176.1 | 0.808885646 | 1.820909216 | 0.068620658 |
| PRKACA | ENST00000583122.1 | ENSG00000264695.1 | -0.831378939 | -1.847776074 | 0.064634744 |
| PRKACA | ENST00000584139.1 | ENSG00000263388.1 | -0.889003104 | -2.005370938 | 0.044923406 |
| PRKACA | ENST00000585559.1 | ENSG00000267117.1 | 0.837014929 | 1.861638244 | 0.062654099 |
| PRKACA | ENST00000585810.1 | ENSG00000236172.2 | 0.813838253 | 1.794449227 | 0.072741496 |
| PRKACA | ENST00000590328.1 | ENSG00000256995.2 | -0.867714729 | -1.946051428 | 0.051648562 |
| PRKACA | ENST00000596643.1 | ENSG00000269439.1 | 0.844707389 | 1.865820005 | 0.062066552 |
| PRKACA | ENST00000597309.1 | ENSG00000232098.2 | -0.808056737 | -1.788113946 | 0.073757626 |
| PRKACA | ENST00000599259.1 | ENSG00000269352.1 | 0.848988313 | 1.911492255 | 0.055941351 |
| PRKACA | ENST00000600007.1 | ENSG00000268655.1 | 0.824253914 | 1.860130515 | 0.062867063 |
| PRKACA | ENST00000600716.1 | ENSG00000269487.1 | 0.948754562 | 2.135527405 | 0.032717954 |
| PRKACA | ENST00000607549.1 | ENSG00000272293.1 | -0.889245227 | -1.983364553 | 0.047326724 |
| PRKACA | ENST00000608259.1 | ENSG00000272627.1 | -0.800708979 | -1.787210251 | 0.073903513 |
| PRKACA | ENST00000609813.1 | ENSG00000272719.1 | 0.864576221 | 1.95638844 | 0.050419413 |
| PRKACA | ENST00000610145.1 | ENSG00000273175.1 | 0.807148804 | 1.798282252 | 0.072132291 |
| PRKACA | NR_024491.1 | LOC100128573 | -0.812265897 | -1.799722925 | 0.071904399 |
| PRKACA | NR_036480.1 | VPS9D1-AS1 | 0.847322515 | 1.892440186 | 0.058432356 |
| PRKACA | NR_073552.1 | LOC101059948 | 0.824253914 | 1.845348238 | 0.064986898 |
| PRKACA | NR_108036.1 | CFAP58-AS1 | 0.803636044 | 1.800142071 | 0.071838208 |
| PRKACA | NR_110702.1 | SEMA3B-AS1 | 0.861843184 | 1.943260258 | 0.051984721 |
| PRKACA | NR_120371.1 | LINC01585 | 0.820342217 | 1.804852462 | 0.071097772 |
| PRKACA | NR_121188.1 | PGM5P3-AS1 | -0.877158875 | -1.943470483 | 0.051959338 |
| PRKACA | NR_134576.1 | LOC105372672 | 0.822417555 | 1.822279937 | 0.068412521 |
| PRKACA | NR_136569.1 | LINC01660 | -0.86754237 | -1.972168652 | 0.048590357 |
| PRKACA | NR_138038.1 | LINC00677 | 0.852779428 | 1.92611084 | 0.054090535 |
| PRKACA | NR_144459.1 | ARSD-AS1 | 0.896824313 | 2.005933019 | 0.044863395 |
| PSMA3 | ENST00000414098.2 | ENSG00000234428.2 | 0.950746026 | 2.142319148 | 0.032167809 |
| PSMA3 | ENST00000414896.1 | ENSG00000223374.1 | 0.965414262 | 2.185848559 | 0.028826687 |
| PSMA3 | ENST00000415106.1 | ENSG00000226733.1 | 0.879875247 | 1.935723819 | 0.052901536 |
| PSMA3 | ENST00000415205.1 | ENSG00000182057.4 | -0.805344081 | -1.816942488 | 0.06922592 |
| PSMA3 | ENST00000417260.1 | ENSG00000231734.4 | 0.836070466 | 1.855510973 | 0.063523294 |
| PSMA3 | ENST00000419207.2 | ENSG00000231248.2 | -0.818836326 | -1.82907825 | 0.067387887 |
| PSMA3 | ENST00000420044.1 | ENSG00000225956.1 | 0.929268231 | 2.098959768 | 0.035820447 |
| PSMA3 | ENST00000421006.1 | ENSG00000234548.1 | 0.886133222 | 1.996815966 | 0.045845178 |
| PSMA3 | ENST00000423428.1 | ENSG00000224048.1 | 0.918815055 | 2.100845925 | 0.035654494 |
| PSMA3 | ENST00000423796.1 | ENSG00000235146.2 | 0.826663704 | 1.837750604 | 0.066099163 |
| PSMA3 | ENST00000424274.1 | ENSG00000232120.1 | 0.859419469 | 1.913271598 | 0.055713292 |
| PSMA3 | ENST00000424678.1 | ENSG00000229600.1 | 0.850507529 | 1.888915281 | 0.058903181 |
| PSMA3 | ENST00000429080.1 | ENSG00000233047.1 | 0.868987781 | 1.925148507 | 0.054210782 |
| PSMA3 | ENST00000435287.1 | ENSG00000227220.1 | -0.859825365 | -1.900629522 | 0.057350556 |
| PSMA3 | ENST00000438173.2 | ENSG00000227733.4 | 0.924159522 | 2.079105802 | 0.037607627 |
| PSMA3 | ENST00000438969.2 | ENSG00000228031.2 | 0.869660355 | 1.962619209 | 0.049690437 |
| PSMA3 | ENST00000448858.1 | ENSG00000237734.1 | 0.834607731 | 1.897004804 | 0.057827304 |
| PSMA3 | ENST00000449749.1 | ENSG00000230834.1 | 0.858972133 | 1.934106025 | 0.053100093 |
| PSMA3 | ENST00000454100.1 | ENSG00000236943.2 | -0.816181202 | -1.837619702 | 0.066118464 |
| PSMA3 | ENST00000456091.1 | ENSG00000226985.1 | -0.860492795 | -1.89897445 | 0.057567835 |
| PSMA3 | ENST00000480919.1 | ENSG00000242474.1 | -0.827075312 | -1.818842469 | 0.068935467 |
| PSMA3 | ENST00000503723.1 | ENSG00000250472.1 | 0.81332914 | 1.814296374 | 0.06963211 |
| PSMA3 | ENST00000504578.1 | ENSG00000251513.1 | 0.83019459 | 1.852798977 | 0.063911177 |
| PSMA3 | ENST00000506723.2 | ENSG00000249484.4 | 0.884978964 | 1.962331388 | 0.049723915 |

| | | | | | |
|---|---|---|---|---|---|
| PSMA3 | ENST00000507857.2 | ENSG00000251055.2 | 0.917220693 | 2.070404401 | 0.03841449 |
| PSMA3 | ENST00000509453.1 | ENSG00000249145.1 | -0.923320545 | -2.096327282 | 0.036053168 |
| PSMA3 | ENST00000509629.1 | ENSG00000250164.1 | -0.847955852 | -1.906800588 | 0.056546415 |
| PSMA3 | ENST00000510198.1 | ENSG00000248733.1 | 0.873485531 | 1.925926569 | 0.054113543 |
| PSMA3 | ENST00000510570.1 | ENSG00000250438.1 | 0.840321259 | 1.876812837 | 0.06054375 |
| PSMA3 | ENST00000518339.1 | ENSG00000253470.1 | 0.811788872 | 1.831746832 | 0.066989148 |
| PSMA3 | ENST00000518590.1 | ENSG00000253986.1 | -0.918220901 | -2.042168432 | 0.041134827 |
| PSMA3 | ENST00000518837.1 | ENSG00000253947.1 | 0.876523065 | 1.979854257 | 0.047719908 |
| PSMA3 | ENST00000522281.1 | ENSG00000253376.1 | 0.852704936 | 1.947659076 | 0.05145577 |
| PSMA3 | ENST00000524286.1 | ENSG00000253658.1 | 0.842531397 | 1.891073709 | 0.058614505 |
| PSMA3 | ENST00000531977.1 | ENSG00000224023.6 | 0.867772519 | 1.942764097 | 0.052044668 |
| PSMA3 | ENST00000535914.1 | ENSG00000256894.1 | 0.886904988 | 1.973241128 | 0.048468097 |
| PSMA3 | ENST00000537032.1 | ENSG00000255933.1 | -0.819809962 | -1.848750058 | 0.064493912 |
| PSMA3 | ENST00000537850.1 | ENSG00000251002.3 | -0.827541884 | -1.83567787 | 0.066405313 |
| PSMA3 | ENST00000540024.1 | ENSG00000255693.1 | -0.836607217 | -1.886919868 | 0.059171104 |
| PSMA3 | ENST00000550263.1 | ENSG00000257605.1 | -0.841047186 | -1.88870289 | 0.058931651 |
| PSMA3 | ENST00000551699.1 | ENSG00000257467.1 | 0.913699418 | 2.054885472 | 0.039890068 |
| PSMA3 | ENST00000552558.1 | ENSG00000257947.1 | 0.817430677 | 1.817743884 | 0.069103287 |
| PSMA3 | ENST00000558618.1 | ENSG00000259209.1 | -0.957009018 | -2.137654325 | 0.032544809 |
| PSMA3 | ENST00000559959.1 | ENSG00000259396.1 | -0.892792896 | -1.987022333 | 0.046919922 |
| PSMA3 | ENST00000565310.1 | ENSG00000261118.1 | -0.898717034 | -2.026427738 | 0.042720971 |
| PSMA3 | ENST00000566449.1 | ENSG00000259791.1 | 0.911505867 | 2.048653754 | 0.040495984 |
| PSMA3 | ENST00000568332.1 | ENSG00000260256.1 | -0.927137623 | -2.0650799 | 0.038915441 |
| PSMA3 | ENST00000568410.1 | ENSG00000260277.1 | 0.88686722 | 1.969502238 | 0.048895446 |
| PSMA3 | ENST00000571660.1 | ENSG00000262848.1 | -0.861584084 | -1.934491075 | 0.053052778 |
| PSMA3 | ENST00000573315.1 | ENSG00000270168.1 | -0.861303231 | -1.932131338 | 0.053343297 |
| PSMA3 | ENST00000576021.1 | ENSG00000262413.1 | -0.83305795 | -1.851788907 | 0.064056141 |
| PSMA3 | ENST00000582558.1 | ENSG00000264569.1 | -0.834219593 | -1.884102212 | 0.059551151 |
| PSMA3 | ENST00000584705.1 | ENSG00000264569.1 | -0.876501447 | -1.93552277 | 0.052926177 |
| PSMA3 | ENST00000585181.1 | ENSG00000265749.1 | 0.939350824 | 2.107964652 | 0.035034047 |
| PSMA3 | ENST00000585761.1 | ENSG00000267198.1 | -0.863435307 | -1.936824872 | 0.052766754 |
| PSMA3 | ENST00000586348.1 | ENSG00000267198.1 | -0.829058083 | -1.849063487 | 0.064448646 |
| PSMA3 | ENST00000589395.1 | ENSG00000267143.1 | -0.89496125 | -2.003681824 | 0.045104154 |
| PSMA3 | ENST00000592022.1 | ENSG00000267383.2 | 0.894462393 | 2.00775698 | 0.044669122 |
| PSMA3 | ENST00000592368.1 | ENSG00000267231.1 | -0.893519344 | -2.014212764 | 0.0439872 |
| PSMA3 | ENST00000593824.1 | ENSG00000268184.1 | 0.843177663 | 1.880449687 | 0.060046817 |
| PSMA3 | ENST00000594091.1 | ENSG00000232732.5 | -0.888221914 | -1.976912933 | 0.048051473 |
| PSMA3 | ENST00000594776.1 | ENSG00000269807.1 | -0.93239823 | -2.089293959 | 0.03668127 |
| PSMA3 | ENST00000595478.1 | ENSG00000237031.3 | 0.878517679 | 1.947081867 | 0.051524921 |
| PSMA3 | ENST00000596091.1 | ENSG00000227733.4 | 0.920903344 | 2.055994519 | 0.039783044 |
| PSMA3 | ENST00000596971.1 | ENSG00000269463.1 | -0.831746043 | -1.853302378 | 0.063839031 |
| PSMA3 | ENST00000597906.1 | ENSG00000268566.1 | 0.996214316 | 2.210712797 | 0.027055732 |
| PSMA3 | ENST00000602471.1 | ENSG00000270107.1 | -0.822303696 | -1.827793175 | 0.067580598 |
| PSMA3 | ENST00000602949.1 | ENSG00000270030.1 | -0.945745443 | -2.126444875 | 0.033466235 |
| PSMA3 | ENST00000605692.1 | ENSG00000270810.1 | -0.870535284 | -1.949782592 | 0.051202037 |
| PSMA3 | ENST00000606482.1 | ENSG00000272416.1 | 0.866208854 | 1.952467095 | 0.050882774 |
| PSMA3 | ENST00000606743.1 | ENSG00000272221.1 | -0.877236555 | -1.948510177 | 0.051353948 |
| PSMA3 | ENST00000606869.1 | ENSG00000272349.1 | 0.800940934 | 1.781255829 | 0.074870667 |
| PSMA3 | ENST00000606909.1 | ENSG00000271821.1 | -0.898466259 | -2.010895018 | 0.044336547 |
| PSMA3 | ENST00000607148.1 | ENSG00000272477.1 | 0.877238529 | 1.961887779 | 0.049775551 |
| PSMA3 | ENST00000608088.1 | ENSG00000272632.1 | 0.953615859 | 2.145425566 | 0.031918837 |

| | | | | | |
|---|---|---|---|---|---|
| PSMA3 | ENST00000608489.1 | ENSG00000272716.1 | -0.802478916 | -1.799924587 | 0.071872547 |
| PSMA3 | ENST00000608759.1 | ENSG00000273464.1 | 0.95427624 | 2.163554193 | 0.03049858 |
| PSMA3 | ENST00000609807.1 | ENSG00000272700.1 | 0.86828093 | 1.952940129 | 0.05082669 |
| PSMA3 | NR_026774.1 | LINC00239 | -0.81502793 | -1.838244869 | 0.066026331 |
| PSMA3 | NR_031762.2 | HCP5B | -0.904537576 | -2.032123655 | 0.042141139 |
| PSMA3 | NR_034131.1 | LINC00272 | -0.813824792 | -1.818336053 | 0.069012786 |
| PSMA3 | NR_037169.1 | LOC100507547 | -0.838139769 | -1.878730008 | 0.060281368 |
| PSMA3 | NR_037170.1 | LOC100507547 | -0.854484981 | -1.916871487 | 0.05525426 |
| PSMA3 | NR_045114.1 | PVRL3-AS1 | 0.847844317 | 1.913964713 | 0.055624665 |
| PSMA3 | NR_046871.1 | LINC00333 | 0.819399327 | 1.823703726 | 0.068196875 |
| PSMA3 | NR_047698.1 | VWC2L-IT1 | 0.841418046 | 1.882409436 | 0.059780446 |
| PSMA3 | NR_102746.1 | ROPN1L-AS1 | -0.825765397 | -1.840573016 | 0.065684155 |
| PSMA3 | NR_108068.1 | LINC00836 | -0.839672067 | -1.890917833 | 0.058635313 |
| PSMA3 | NR_110245.1 | LOC101929282 | 0.877733824 | 1.977799109 | 0.047951374 |
| PSMA3 | NR_110630.1 | LOC101927478 | -0.849977058 | -1.911922946 | 0.055886078 |
| PSMA3 | NR_131204.1 | XACT | -0.818228617 | -1.824723957 | 0.068042696 |
| PSMA3 | NR_133642.1 | DIRC3-AS1 | 0.93558984 | 2.111276268 | 0.034748573 |
| PSMA3 | NR_134573.1 | GS1-124K5.4 | 0.836078867 | 1.868216137 | 0.06173195 |
| PSMA3 | NR_135584.1 | LOC101927596 | -0.845687307 | -1.871643266 | 0.061255973 |
| PSMA3 | NR_136178.1 | LOC101928166 | 0.907164995 | 2.039158833 | 0.041434178 |
| PSMB9 | ENST00000415205.1 | ENSG00000182057.4 | 0.945230543 | 2.114655221 | 0.034459344 |
| PSMB9 | ENST00000417260.1 | ENSG00000231734.4 | -0.915645832 | -2.070711842 | 0.038385733 |
| PSMB9 | ENST00000419662.1 | ENSG00000228265.1 | 0.884591125 | 1.995505808 | 0.045987741 |
| PSMB9 | ENST00000421006.1 | ENSG00000234548.1 | -0.812233609 | -1.843198633 | 0.065300014 |
| PSMB9 | ENST00000422763.1 | ENSG00000231131.2 | -0.909984535 | -2.056633346 | 0.039721508 |
| PSMB9 | ENST00000423428.1 | ENSG00000224048.1 | -0.869326905 | -1.954689267 | 0.050619758 |
| PSMB9 | ENST00000425124.1 | ENSG00000232336.1 | 0.871500482 | 1.971931631 | 0.048617412 |
| PSMB9 | ENST00000425881.1 | ENSG00000239636.1 | 0.917212546 | 2.059797307 | 0.039417922 |
| PSMB9 | ENST00000426475.1 | ENSG00000239467.1 | 0.82810569 | 1.841519027 | 0.065545535 |
| PSMB9 | ENST00000429080.1 | ENSG00000233047.1 | -0.859331412 | -1.931361426 | 0.053438372 |
| PSMB9 | ENST00000431730.1 | ENSG00000237401.2 | 0.914074012 | 2.042549821 | 0.041097023 |
| PSMB9 | ENST00000433035.1 | ENSG00000230483.1 | 0.858310603 | 1.9239877 | 0.054356125 |
| PSMB9 | ENST00000433051.1 | ENSG00000233193.1 | 0.914883693 | 2.043064812 | 0.041046023 |
| PSMB9 | ENST00000433905.2 | ENSG00000229299.2 | 0.820544757 | 1.831805871 | 0.066980348 |
| PSMB9 | ENST00000435287.1 | ENSG00000227220.1 | 0.816195098 | 1.846264109 | 0.064853866 |
| PSMB9 | ENST00000438190.1 | ENSG00000227214.2 | 0.825254641 | 1.854648972 | 0.06364637 |
| PSMB9 | ENST00000438969.2 | ENSG00000228031.2 | -0.834780069 | -1.856660131 | 0.063359523 |
| PSMB9 | ENST00000439443.1 | ENSG00000236911.2 | 0.827434601 | 1.84253615 | 0.065396764 |
| PSMB9 | ENST00000440595.1 | ENSG00000228265.1 | 0.965505957 | 2.136446877 | 0.032643007 |
| PSMB9 | ENST00000441592.2 | ENSG00000224078.8 | 0.934064608 | 2.053528779 | 0.040021322 |
| PSMB9 | ENST00000442649.1 | ENSG00000234089.1 | -0.828004639 | -1.843561357 | 0.065247092 |
| PSMB9 | ENST00000446562.1 | ENSG00000233896.1 | 0.885386313 | 1.985575713 | 0.047080455 |
| PSMB9 | ENST00000447343.2 | ENSG00000229299.2 | 0.802330788 | 1.788787906 | 0.073648979 |
| PSMB9 | ENST00000448858.1 | ENSG00000237734.1 | -0.834894929 | -1.845135142 | 0.065017882 |
| PSMB9 | ENST00000451507.1 | ENSG00000229539.1 | 0.978453409 | 2.218136469 | 0.026545529 |
| PSMB9 | ENST00000452176.1 | ENSG00000223659.1 | -0.86353844 | -1.912979996 | 0.055750613 |
| PSMB9 | ENST00000456091.1 | ENSG00000226985.1 | 0.900256141 | 2.006659212 | 0.044785962 |
| PSMB9 | ENST00000457371.1 | ENSG00000237401.2 | 0.846880766 | 1.891752558 | 0.058523957 |
| PSMB9 | ENST00000463255.1 | ENSG00000243305.1 | -0.875385919 | -1.977523999 | 0.047982431 |
| PSMB9 | ENST00000488310.1 | ENSG00000240449.1 | 0.873322112 | 1.962577777 | 0.049695255 |
| PSMB9 | ENST00000489077.1 | ENSG00000244198.1 | 0.934527097 | 2.0834565 | 0.037209633 |

| | | | | | |
|---|---|---|---|---|---|
| PSMB9 | ENST00000489090.1 | ENSG00000240045.1 | -0.882692251 | -1.971409761 | 0.048677025 |
| PSMB9 | ENST00000503723.1 | ENSG00000250472.1 | -0.9400668 | -2.110949892 | 0.034776619 |
| PSMB9 | ENST00000504916.1 | ENSG00000248112.1 | -0.800998794 | -1.813034187 | 0.069826549 |
| PSMB9 | ENST00000506723.2 | ENSG00000249484.4 | -0.807962017 | -1.799911452 | 0.071874621 |
| PSMB9 | ENST00000506791.1 | ENSG00000251131.1 | 0.883208752 | 1.997867692 | 0.045731006 |
| PSMB9 | ENST00000509036.1 | ENSG00000251131.1 | 0.822861941 | 1.814169988 | 0.069651559 |
| PSMB9 | ENST00000509192.1 | ENSG00000250765.1 | 0.890956882 | 1.989941872 | 0.046597339 |
| PSMB9 | ENST00000509453.1 | ENSG00000249145.1 | 0.986980461 | 2.197951996 | 0.027952527 |
| PSMB9 | ENST00000510570.1 | ENSG00000250438.1 | -0.898251965 | -2.025719112 | 0.042793577 |
| PSMB9 | ENST00000518837.1 | ENSG00000253947.1 | -0.814512753 | -1.808167672 | 0.070580408 |
| PSMB9 | ENST00000520603.1 | ENSG00000254001.1 | -0.933556725 | -2.116437859 | 0.034307585 |
| PSMB9 | ENST00000521653.1 | ENSG00000253301.1 | 0.931937988 | 2.078198752 | 0.037691057 |
| PSMB9 | ENST00000529247.1 | ENSG00000254741.1 | 0.821407687 | 1.823889349 | 0.068168802 |
| PSMB9 | ENST00000530435.1 | ENSG00000254630.1 | 0.824140944 | 1.841575497 | 0.065537268 |
| PSMB9 | ENST00000532454.1 | ENSG00000255120.1 | 0.812825467 | 1.831584123 | 0.067013404 |
| PSMB9 | ENST00000534178.1 | ENSG00000255120.1 | 0.814080742 | 1.82212161 | 0.068436536 |
| PSMB9 | ENST00000537032.1 | ENSG00000255933.1 | 0.8064357 | 1.802356618 | 0.071489316 |
| PSMB9 | ENST00000537850.1 | ENSG00000251002.3 | 0.80166549 | 1.795152188 | 0.072629456 |
| PSMB9 | ENST00000543403.1 | ENSG00000256684.1 | -0.882101283 | -1.965449744 | 0.049362207 |
| PSMB9 | ENST00000549806.1 | ENSG00000257252.1 | 0.80919759 | 1.794787816 | 0.072687513 |
| PSMB9 | ENST00000550263.1 | ENSG00000257605.1 | 0.897412547 | 2.0226117 | 0.043113197 |
| PSMB9 | ENST00000551699.1 | ENSG00000257467.1 | -0.809809858 | -1.828065597 | 0.067539707 |
| PSMB9 | ENST00000559959.1 | ENSG00000259396.1 | 0.806630508 | 1.823863872 | 0.068172655 |
| PSMB9 | ENST00000562191.1 | ENSG00000261292.1 | -0.904395358 | -2.010734324 | 0.044353527 |
| PSMB9 | ENST00000562995.1 | ENSG00000261253.1 | 0.86975727 | 1.964297086 | 0.049495649 |
| PSMB9 | ENST00000563611.1 | ENSG00000261583.1 | 0.860471935 | 1.92229767 | 0.054568313 |
| PSMB9 | ENST00000564809.1 | ENSG00000261471.1 | 0.872177737 | 1.940938826 | 0.052265698 |
| PSMB9 | ENST00000565310.1 | ENSG00000261118.1 | 0.814205851 | 1.827935847 | 0.06755918 |
| PSMB9 | ENST00000565955.1 | ENSG00000261055.1 | 0.886829862 | 1.983074694 | 0.047359087 |
| PSMB9 | ENST00000568332.1 | ENSG00000260256.1 | 0.854326881 | 1.900181822 | 0.057409263 |
| PSMB9 | ENST00000570493.2 | ENSG00000261898.2 | 0.815051426 | 1.81874028 | 0.068951063 |
| PSMB9 | ENST00000571660.1 | ENSG00000262848.1 | 0.956718336 | 2.157748904 | 0.030947359 |
| PSMB9 | ENST00000573315.1 | ENSG00000270168.1 | 0.903464365 | 2.032755578 | 0.042077223 |
| PSMB9 | ENST00000576271.1 | ENSG00000263342.1 | 0.816920889 | 1.819970173 | 0.068763547 |
| PSMB9 | ENST00000577853.1 | ENSG00000264207.1 | 0.813353814 | 1.828979803 | 0.067402634 |
| PSMB9 | ENST00000579775.1 | ENSG00000264108.1 | 0.811266474 | 1.796257804 | 0.072453525 |
| PSMB9 | ENST00000580622.1 | ENSG00000264634.1 | 0.927630289 | 2.066243201 | 0.038805522 |
| PSMB9 | ENST00000582044.1 | ENSG00000263715.2 | 0.962331597 | 2.147260465 | 0.031772551 |
| PSMB9 | ENST00000582558.1 | ENSG00000264569.1 | 0.913448873 | 2.055228157 | 0.039856973 |
| PSMB9 | ENST00000585761.1 | ENSG00000267198.1 | 0.88432277 | 1.973900168 | 0.048393096 |
| PSMB9 | ENST00000586348.1 | ENSG00000267198.1 | 0.89676719 | 2.016786194 | 0.04371783 |
| PSMB9 | ENST00000586694.1 | ENSG00000267141.1 | 0.826241926 | 1.847378946 | 0.064692238 |
| PSMB9 | ENST00000588380.1 | ENSG00000266990.1 | 0.91488543 | 2.063363739 | 0.039078082 |
| PSMB9 | ENST00000589380.1 | ENSG00000267488.1 | 0.820896936 | 1.848054814 | 0.064594414 |
| PSMB9 | ENST00000589395.1 | ENSG00000267143.1 | 0.976962985 | 2.207662763 | 0.02726779 |
| PSMB9 | ENST00000592368.1 | ENSG00000267231.1 | 0.813570314 | 1.833259844 | 0.066763938 |
| PSMB9 | ENST00000592525.1 | ENSG00000267214.1 | 0.922599576 | 2.060484974 | 0.039352201 |
| PSMB9 | ENST00000593139.1 | ENSG00000267042.1 | 0.857319983 | 1.96507297 | 0.049405793 |
| PSMB9 | ENST00000593967.1 | ENSG00000232732.5 | 0.830153553 | 1.880178615 | 0.060083739 |
| PSMB9 | ENST00000594776.1 | ENSG00000269807.1 | 0.882831945 | 1.979415239 | 0.047769274 |
| PSMB9 | ENST00000594850.1 | ENSG00000268093.1 | 0.855892041 | 1.90492485 | 0.056789841 |

| | | | | | |
|---|---|---|---|---|---|
| PSMB9 | ENST00000595478.1 | ENSG00000237031.3 | -0.800565118 | -1.7718915 | 0.07641257 |
| PSMB9 | ENST00000596091.1 | ENSG00000227733.4 | -0.934920788 | -2.089055735 | 0.036702706 |
| PSMB9 | ENST00000596971.1 | ENSG00000269463.1 | 0.914345329 | 2.045550123 | 0.040800653 |
| PSMB9 | ENST00000597256.1 | ENSG00000267986.1 | 0.829286379 | 1.858354534 | 0.063118684 |
| PSMB9 | ENST00000597906.1 | ENSG00000268566.1 | -0.873945057 | -1.954418054 | 0.050651797 |
| PSMB9 | ENST00000600242.1 | ENSG00000269583.1 | 0.822792499 | 1.854030571 | 0.063734786 |
| PSMB9 | ENST00000602594.1 | ENSG00000269930.1 | -0.847541002 | -1.894789818 | 0.058120253 |
| PSMB9 | ENST00000602949.1 | ENSG00000270030.1 | 0.854984406 | 1.902452477 | 0.057112026 |
| PSMB9 | ENST00000603948.1 | ENSG00000222041.6 | 0.859567562 | 1.920166484 | 0.054836874 |
| PSMB9 | ENST00000604142.1 | ENSG00000271308.1 | 0.902238926 | 2.023127833 | 0.04305997 |
| PSMB9 | ENST00000604183.1 | ENSG00000271185.1 | 0.92703112 | 2.067299015 | 0.038705987 |
| PSMB9 | ENST00000605082.1 | ENSG00000270426.1 | 0.822321679 | 1.82793634 | 0.067559106 |
| PSMB9 | ENST00000606277.1 | ENSG00000272145.1 | 0.80201033 | 1.8042733 | 0.071188473 |
| PSMB9 | ENST00000606377.1 | ENSG00000272286.1 | -0.808742993 | -1.817209358 | 0.069185063 |
| PSMB9 | ENST00000606441.1 | ENSG00000272277.1 | 0.962002871 | 2.105750915 | 0.035225995 |
| PSMB9 | ENST00000606743.1 | ENSG00000272221.1 | 0.959142482 | 2.160184145 | 0.030758417 |
| PSMB9 | ENST00000606909.1 | ENSG00000271821.1 | 0.997061452 | 2.232394287 | 0.025588915 |
| PSMB9 | ENST00000607148.1 | ENSG00000272477.1 | -0.81886352 | -1.819938641 | 0.06876835 |
| PSMB9 | ENST00000607476.1 | ENSG00000272540.1 | 0.893817464 | 2.003129303 | 0.045163411 |
| PSMB9 | ENST00000607943.1 | ENSG00000273188.1 | 0.883183878 | 1.983547786 | 0.047306275 |
| PSMB9 | ENST00000608367.1 | ENSG00000273361.1 | 0.854344216 | 1.875455645 | 0.060730066 |
| PSMB9 | ENST00000608489.1 | ENSG00000272716.1 | 0.819496107 | 1.836893059 | 0.066225684 |
| PSMB9 | ENST00000608759.1 | ENSG00000273464.1 | -0.840676764 | -1.859817689 | 0.062911324 |
| PSMB9 | NR_026774.1 | LINC00239 | 0.896391207 | 2.00556748 | 0.044902414 |
| PSMB9 | NR_026802.1 | FAM74A4 | 0.81095467 | 1.849718677 | 0.064354107 |
| PSMB9 | NR_026813.1 | LINC00597 | -0.828681326 | -1.828131301 | 0.067529848 |
| PSMB9 | NR_026951.1 | LINC00324 | 0.83097067 | 1.876539586 | 0.060581224 |
| PSMB9 | NR_028324.1 | LINC01002 | 0.815975889 | 1.815412896 | 0.069460481 |
| PSMB9 | NR_031762.2 | HCP5B | 0.869432688 | 1.950903172 | 0.051068565 |
| PSMB9 | NR_034131.1 | LINC00272 | 0.864291887 | 1.928659004 | 0.053773209 |
| PSMB9 | NR_037169.1 | LOC100507547 | 0.967639838 | 2.132059355 | 0.033001967 |
| PSMB9 | NR_037170.1 | LOC100507547 | 0.964705397 | 2.167866883 | 0.030168816 |
| PSMB9 | NR_038421.1 | LINC01220 | 0.814563697 | 1.815785445 | 0.069403291 |
| PSMB9 | NR_038923.1 | SSSCA1-AS1 | 0.860978265 | 1.928932985 | 0.053739182 |
| PSMB9 | NR_040047.1 | SDCBP2-AS1 | 0.812706774 | 1.824954044 | 0.068007965 |
| PSMB9 | NR_045114.1 | PVRL3-AS1 | -0.888837063 | -1.978359842 | 0.047888127 |
| PSMB9 | NR_046871.1 | LINC00333 | -0.825778795 | -1.850442366 | 0.064249818 |
| PSMB9 | NR_047116.1 | HIF1A-AS1 | -0.813572138 | -1.825011929 | 0.067999229 |
| PSMB9 | NR_072981.1 | LINC00957 | 0.924001389 | 2.044616705 | 0.040892661 |
| PSMB9 | NR_072982.1 | LINC00957 | 0.925005067 | 2.07605469 | 0.037888893 |
| PSMB9 | NR_109885.1 | RALY-AS1 | 0.972498438 | 2.200150637 | 0.027796209 |
| PSMB9 | NR_109886.1 | RALY-AS1 | 0.881298284 | 1.964893249 | 0.049426594 |
| PSMB9 | NR_110245.1 | LOC101929282 | -0.912048277 | -2.058331512 | 0.039558321 |
| PSMB9 | NR_110559.1 | LOC101927023 | -0.831484131 | -1.875940518 | 0.060663448 |
| PSMB9 | NR_110630.1 | LOC101927478 | 0.918506967 | 2.067100534 | 0.038724682 |
| PSMB9 | NR_110941.1 | MIR762HG | 0.917381348 | 2.054517839 | 0.039925599 |
| PSMB9 | NR_110998.1 | FAM74A4 | 0.81095467 | 1.818898096 | 0.068926978 |
| PSMB9 | NR_125957.1 | LOC101928626 | -0.86353844 | -1.944354167 | 0.051852757 |
| PSMB9 | NR_126522.1 | EXOC3-AS1 | 0.833005562 | 1.853127362 | 0.063864106 |
| PSMB9 | NR_134252.1 | LOC105379030 | 0.818891056 | 1.804864751 | 0.071095849 |
| PSMB9 | NR_134520.1 | LOC727993 | 0.891362462 | 1.987708635 | 0.046843923 |

| | | | | | |
|---|---|---|---|---|---|
| PSMB9 | NR_135024.1 | LOC105369747 | 0.824795648 | 1.85778982 | 0.063198867 |
| PSMB9 | NR_135097.1 | LOC105369443 | -0.882101283 | -1.974787367 | 0.048292283 |
| PSMB9 | NR_136215.1 | VCAN-AS1 | -0.832068279 | -1.849015376 | 0.064455593 |
| PTGDS | ENST00000400768.2 | ENSG00000215692.2 | 0.816729465 | 1.827686865 | 0.067596561 |
| PTGDS | ENST00000411489.1 | ENSG00000227112.1 | -0.878356439 | -1.984688981 | 0.047179085 |
| PTGDS | ENST00000411694.1 | ENSG00000225331.1 | 0.871636831 | 1.929631747 | 0.053652482 |
| PTGDS | ENST00000420766.1 | ENSG00000228679.1 | 0.902078139 | 2.034637972 | 0.041887314 |
| PTGDS | ENST00000421020.1 | ENSG00000231407.1 | 0.866117835 | 1.920273395 | 0.054823375 |
| PTGDS | ENST00000424181.1 | ENSG00000224977.1 | 0.834592227 | 1.852023382 | 0.064022465 |
| PTGDS | ENST00000432314.1 | ENSG00000231532.1 | 0.854679168 | 1.935125214 | 0.052974932 |
| PTGDS | ENST00000434790.1 | ENSG00000240040.1 | -0.83931361 | -1.90245472 | 0.057111733 |
| PTGDS | ENST00000435733.1 | ENSG00000226377.1 | 0.888422333 | 1.990990046 | 0.046481982 |
| PTGDS | ENST00000435992.2 | ENSG00000232675.3 | 0.869782703 | 1.939604583 | 0.052427763 |
| PTGDS | ENST00000438222.1 | ENSG00000238034.1 | 0.897189101 | 1.981644738 | 0.047519016 |
| PTGDS | ENST00000452176.1 | ENSG00000223659.1 | -0.881966428 | -1.9472571 | 0.051503919 |
| PTGDS | ENST00000455788.1 | ENSG00000236263.1 | 0.940770685 | 2.102491673 | 0.035510228 |
| PTGDS | ENST00000457371.1 | ENSG00000237401.2 | 0.845677127 | 1.892974891 | 0.058361209 |
| PTGDS | ENST00000458154.1 | ENSG00000235578.1 | 0.824245984 | 1.826957652 | 0.067706137 |
| PTGDS | ENST00000484413.1 | ENSG00000271853.1 | 0.807866061 | 1.823822816 | 0.068178863 |
| PTGDS | ENST00000494509.1 | ENSG00000240095.1 | 0.948205149 | 2.124382301 | 0.033638189 |
| PTGDS | ENST00000500496.2 | ENSG00000245479.2 | 0.865752877 | 1.936192936 | 0.052844075 |
| PTGDS | ENST00000503571.1 | ENSG00000249592.1 | 0.857778897 | 1.903133816 | 0.057023087 |
| PTGDS | ENST00000504891.1 | ENSG00000249388.1 | 0.824651537 | 1.844826094 | 0.06506284 |
| PTGDS | ENST00000519506.1 | ENSG00000253103.1 | 0.928412629 | 2.070215192 | 0.038432197 |
| PTGDS | ENST00000521307.1 | ENSG00000253177.1 | 0.870629199 | 1.959924242 | 0.050004646 |
| PTGDS | ENST00000526694.1 | ENSG00000231999.2 | 0.807822205 | 1.807238945 | 0.070725031 |
| PTGDS | ENST00000527789.1 | ENSG00000255173.1 | 0.835887325 | 1.8982947 | 0.057657272 |
| PTGDS | ENST00000535720.1 | ENSG00000256364.1 | 0.877617308 | 1.986915583 | 0.046931753 |
| PTGDS | ENST00000548722.2 | ENSG00000257194.2 | -0.934247852 | -2.124161791 | 0.033656617 |
| PTGDS | ENST00000554451.1 | ENSG00000258683.1 | 0.866238704 | 1.943347974 | 0.051974129 |
| PTGDS | ENST00000558475.1 | ENSG00000259604.1 | 0.850953737 | 1.896865327 | 0.057845715 |
| PTGDS | ENST00000558568.1 | ENSG00000272639.1 | 0.819023283 | 1.847193274 | 0.064719134 |
| PTGDS | ENST00000559673.1 | ENSG00000259604.1 | 0.845320711 | 1.897747886 | 0.057729301 |
| PTGDS | ENST00000559960.1 | ENSG00000259354.1 | 0.808759711 | 1.785246594 | 0.074221327 |
| PTGDS | ENST00000563044.1 | ENSG00000260978.1 | 0.856881301 | 1.928639452 | 0.053775638 |
| PTGDS | ENST00000567127.1 | ENSG00000260264.1 | -0.844481616 | -1.903630065 | 0.05695838 |
| PTGDS | ENST00000567395.1 | ENSG00000261090.1 | 0.917545885 | 2.066883651 | 0.038745119 |
| PTGDS | ENST00000569981.1 | ENSG00000238045.5 | 0.909170996 | 2.03842969 | 0.04150698 |
| PTGDS | ENST00000570493.2 | ENSG00000261898.2 | 0.863622769 | 1.94503248 | 0.051771069 |
| PTGDS | ENST00000570512.1 | ENSG00000262768.1 | 0.821075794 | 1.851174195 | 0.064144497 |
| PTGDS | ENST00000574365.1 | ENSG00000262837.1 | 0.913180654 | 2.030745163 | 0.042280852 |
| PTGDS | ENST00000577807.1 | ENSG00000263427.1 | 0.958449126 | 2.142770046 | 0.032131568 |
| PTGDS | ENST00000578757.1 | ENSG00000175061.13 | 0.836349149 | 1.860762147 | 0.062777773 |
| PTGDS | ENST00000581362.1 | ENSG00000235300.3 | 0.850548856 | 1.907653157 | 0.05643606 |
| PTGDS | ENST00000582386.1 | ENSG00000265174.1 | 0.861979009 | 1.920524207 | 0.054791718 |
| PTGDS | ENST00000584758.1 | ENSG00000265356.1 | 0.908782846 | 2.007008153 | 0.044748795 |
| PTGDS | ENST00000585559.1 | ENSG00000267117.1 | 0.807053409 | 1.807982907 | 0.070609161 |
| PTGDS | ENST00000586694.1 | ENSG00000267141.1 | 0.878522553 | 1.974528857 | 0.04832164 |
| PTGDS | ENST00000588380.1 | ENSG00000266990.1 | 0.836486129 | 1.870167741 | 0.061460528 |
| PTGDS | ENST00000599259.1 | ENSG00000269352.1 | 0.837291028 | 1.86440438 | 0.062264938 |
| PTGDS | ENST00000599467.1 | ENSG00000244513.2 | 0.965234004 | 2.150273243 | 0.031533608 |

| | | | | | |
|---|---|---|---|---|---|
| PTGDS | ENST00000601735.1 | ENSG00000244513.2 | 0.817807298 | 1.817490757 | 0.069142002 |
| PTGDS | ENST00000602594.1 | ENSG00000269930.1 | -0.902091461 | -2.00081451 | 0.045412383 |
| PTGDS | ENST00000602809.1 | ENSG00000270105.1 | -0.939022822 | -2.098814418 | 0.035833263 |
| PTGDS | ENST00000606377.1 | ENSG00000272286.1 | -0.851003845 | -1.913352851 | 0.055702896 |
| PTGDS | ENST00000606470.1 | ENSG00000271913.1 | 0.927908538 | 2.049812075 | 0.040382773 |
| PTGDS | ENST00000607876.1 | ENSG00000272848.1 | 0.845329921 | 1.903939546 | 0.056918058 |
| PTGDS | ENST00000608159.1 | ENSG00000273093.1 | -0.861447419 | -1.932316701 | 0.053320428 |
| PTGDS | ENST00000608856.1 | ENSG00000272600.1 | -0.859577363 | -1.962617967 | 0.049690581 |
| PTGDS | NR_028324.1 | LINC01002 | 0.86859818 | 1.950273894 | 0.051143482 |
| PTGDS | NR_036480.1 | VPS9D1-AS1 | 0.815637941 | 1.844891788 | 0.065053281 |
| PTGDS | NR_120318.1 | RORA-AS2 | 0.881965375 | 1.986404269 | 0.046988453 |
| PTGDS | NR_120371.1 | LINC01585 | 0.869911004 | 1.936643555 | 0.05278893 |
| PTGDS | NR_125957.1 | LOC101928626 | -0.881966428 | -1.99551155 | 0.045987116 |
| PTGDS | NR_135274.1 | LOC105370619 | 0.835081244 | 1.86023703 | 0.062851998 |
| PTGDS | NR_135626.1 | LOC100505585 | 0.829499744 | 1.842146373 | 0.065453742 |
| PTGDS | NR_138038.1 | LINC00677 | 0.828993896 | 1.869333073 | 0.061576489 |
| PYDC2 | ENST00000381106.4 | ENSG00000205663.5 | -0.848065372 | -1.918765098 | 0.055014069 |
| PYDC2 | ENST00000411804.1 | ENSG00000227415.1 | -0.865708339 | -1.93553599 | 0.052924557 |
| PYDC2 | ENST00000414896.1 | ENSG00000223374.1 | -0.806050358 | -1.819131415 | 0.068891383 |
| PYDC2 | ENST00000415106.1 | ENSG00000226733.1 | -0.833295703 | -1.888215218 | 0.058997064 |
| PYDC2 | ENST00000418621.1 | ENSG00000224731.1 | -0.87797946 | -1.967228724 | 0.049156848 |
| PYDC2 | ENST00000423796.1 | ENSG00000235146.2 | -0.920237246 | -2.050750026 | 0.040291297 |
| PYDC2 | ENST00000424342.1 | ENSG00000234988.1 | 0.926900305 | 2.07015185 | 0.038438127 |
| PYDC2 | ENST00000424678.1 | ENSG00000229600.1 | -0.860912108 | -1.942037126 | 0.052132606 |
| PYDC2 | ENST00000426302.1 | ENSG00000230454.1 | 0.927024811 | 2.035033813 | 0.041847471 |
| PYDC2 | ENST00000429080.1 | ENSG00000233047.1 | -0.818276624 | -1.827989455 | 0.067551134 |
| PYDC2 | ENST00000430247.1 | ENSG00000232855.2 | -0.945758046 | -2.119759511 | 0.034026331 |
| PYDC2 | ENST00000434292.1 | ENSG00000229796.1 | -0.964775512 | -2.145854916 | 0.031884556 |
| PYDC2 | ENST00000435287.1 | ENSG00000227220.1 | 0.849588391 | 1.880972895 | 0.059975606 |
| PYDC2 | ENST00000435828.1 | ENSG00000235612.1 | -0.824932539 | -1.851894906 | 0.064040916 |
| PYDC2 | ENST00000436515.1 | ENSG00000224521.1 | -0.817869187 | -1.801683014 | 0.071595292 |
| PYDC2 | ENST00000438173.2 | ENSG00000227733.4 | -0.898309185 | -2.013357592 | 0.044077024 |
| PYDC2 | ENST00000438623.1 | ENSG00000224521.1 | -0.890466906 | -2.022810706 | 0.043092667 |
| PYDC2 | ENST00000440038.2 | ENSG00000237094.7 | 0.909090909 | 2.03102463 | 0.042252496 |
| PYDC2 | ENST00000442017.1 | ENSG00000229660.1 | 0.981856987 | 2.198138682 | 0.027939225 |
| PYDC2 | ENST00000442852.1 | ENSG00000237923.1 | 0.899996655 | 2.02307535 | 0.043065379 |
| PYDC2 | ENST00000445233.1 | ENSG00000233928.1 | -0.947528232 | -2.108375655 | 0.034998509 |
| PYDC2 | ENST00000447111.1 | ENSG00000231903.1 | -0.862237793 | -1.914660007 | 0.055535877 |
| PYDC2 | ENST00000448858.1 | ENSG00000237734.1 | -0.912080812 | -2.046704932 | 0.040687065 |
| PYDC2 | ENST00000450696.1 | ENSG00000235146.2 | -0.878254305 | -1.954663222 | 0.050622834 |
| PYDC2 | ENST00000451034.1 | ENSG00000229805.1 | -0.823793773 | -1.861487293 | 0.062675393 |
| PYDC2 | ENST00000451090.1 | ENSG00000235215.2 | -0.88302548 | -2.005899551 | 0.044866966 |
| PYDC2 | ENST00000455373.1 | ENSG00000226097.1 | -0.955093465 | -2.125315011 | 0.033560337 |
| PYDC2 | ENST00000457043.1 | ENSG00000231365.1 | -0.869048189 | -1.927489358 | 0.053918673 |
| PYDC2 | ENST00000457632.1 | ENSG00000234248.1 | -0.808046939 | -1.809545156 | 0.070366351 |
| PYDC2 | ENST00000457998.2 | ENSG00000233006.2 | 0.941225818 | 2.115350275 | 0.034400105 |
| PYDC2 | ENST00000483283.1 | ENSG00000240571.1 | -0.930695264 | -2.099106478 | 0.035807515 |
| PYDC2 | ENST00000503505.1 | ENSG00000248629.1 | -0.842207583 | -1.886667131 | 0.059205111 |
| PYDC2 | ENST00000503987.1 | ENSG00000250075.1 | -0.838703787 | -1.885568109 | 0.059353178 |
| PYDC2 | ENST00000504578.1 | ENSG00000251513.1 | -0.849881014 | -1.935803439 | 0.05289178 |
| PYDC2 | ENST00000504765.1 | ENSG00000249638.1 | -0.80887273 | -1.806936211 | 0.070772226 |

| | | | | | |
|---|---|---|---|---|---|
| PYDC2 | ENST00000505575.1 | ENSG00000248939.1 | 0.829055001 | 1.851563484 | 0.064088531 |
| PYDC2 | ENST00000506379.1 | ENSG00000240152.2 | -0.869747099 | -1.932390609 | 0.053311312 |
| PYDC2 | ENST00000506723.2 | ENSG00000249484.4 | -0.928432635 | -2.082726978 | 0.037276117 |
| PYDC2 | ENST00000508191.1 | ENSG00000250910.3 | -0.884920202 | -1.979870098 | 0.047718127 |
| PYDC2 | ENST00000508687.1 | ENSG00000250538.1 | -0.809209624 | -1.795177505 | 0.072625423 |
| PYDC2 | ENST00000508823.1 | ENSG00000250716.1 | -0.902389315 | -2.003299177 | 0.045145186 |
| PYDC2 | ENST00000509629.1 | ENSG00000250164.1 | 0.823226349 | 1.834573587 | 0.066568895 |
| PYDC2 | ENST00000512882.2 | ENSG00000251575.2 | -0.815284054 | -1.851379917 | 0.064114916 |
| PYDC2 | ENST00000518339.1 | ENSG00000253470.1 | -0.827227119 | -1.848933209 | 0.064467458 |
| PYDC2 | ENST00000518837.1 | ENSG00000253947.1 | -0.915526865 | -2.046465483 | 0.040710595 |
| PYDC2 | ENST00000519005.1 | ENSG00000253507.1 | -0.81498485 | -1.80757768 | 0.070672255 |
| PYDC2 | ENST00000520192.1 | ENSG00000253807.1 | -0.980748652 | -2.206909691 | 0.027320369 |
| PYDC2 | ENST00000521294.1 | ENSG00000253664.1 | 0.911134169 | 2.032339274 | 0.042119321 |
| PYDC2 | ENST00000521725.1 | ENSG00000253396.1 | -0.9246419 | -2.04802645 | 0.040557408 |
| PYDC2 | ENST00000522281.1 | ENSG00000253376.1 | -0.952193843 | -2.137151411 | 0.032585679 |
| PYDC2 | ENST00000522300.1 | ENSG00000249484.4 | -0.98034275 | -2.211262116 | 0.027017691 |
| PYDC2 | ENST00000522390.1 | ENSG00000254262.1 | -0.909662879 | -2.026675373 | 0.042695623 |
| PYDC2 | ENST00000522426.1 | ENSG00000253538.1 | -0.852111526 | -1.892912693 | 0.058369481 |
| PYDC2 | ENST00000523806.1 | ENSG00000253616.1 | 0.819754764 | 1.821010005 | 0.068605336 |
| PYDC2 | ENST00000523935.1 | ENSG00000253567.1 | -0.802430511 | -1.77831187 | 0.075352648 |
| PYDC2 | ENST00000527274.2 | ENSG00000255517.2 | 0.964941196 | 2.100733388 | 0.035664377 |
| PYDC2 | ENST00000527727.1 | ENSG00000255227.1 | -0.871785996 | -1.958477896 | 0.050173962 |
| PYDC2 | ENST00000531977.1 | ENSG00000224023.6 | -0.947572449 | -2.121319951 | 0.033894887 |
| PYDC2 | ENST00000535914.1 | ENSG00000256894.1 | -0.93349798 | -2.079309203 | 0.03758894 |
| PYDC2 | ENST00000537032.1 | ENSG00000255933.1 | 0.831567838 | 1.869657516 | 0.061531393 |
| PYDC2 | ENST00000537850.1 | ENSG00000251002.3 | 0.887877852 | 2.00180075 | 0.045306165 |
| PYDC2 | ENST00000538641.1 | ENSG00000256422.1 | -0.873363975 | -1.94704857 | 0.051528912 |
| PYDC2 | ENST00000541391.1 | ENSG00000256268.1 | -0.886287097 | -1.996513725 | 0.045878033 |
| PYDC2 | ENST00000546135.1 | ENSG00000256670.1 | 0.890002421 | 1.976308509 | 0.048119847 |
| PYDC2 | ENST00000548210.1 | ENSG00000257784.1 | 0.960242503 | 2.167772056 | 0.030176034 |
| PYDC2 | ENST00000549616.1 | ENSG00000258168.1 | -0.877788039 | -1.961623426 | 0.049806343 |
| PYDC2 | ENST00000550279.1 | ENSG00000258338.1 | -0.80171544 | -1.800556579 | 0.071772798 |
| PYDC2 | ENST00000550805.1 | ENSG00000244306.5 | 0.837082068 | 1.851605659 | 0.06408247 |
| PYDC2 | ENST00000551067.1 | ENSG00000257891.1 | 0.893548553 | 1.995429798 | 0.045996024 |
| PYDC2 | ENST00000551135.1 | ENSG00000258294.1 | -0.921212341 | -2.078180436 | 0.037692743 |
| PYDC2 | ENST00000552541.1 | ENSG00000258294.1 | -0.933523845 | -2.117129403 | 0.034248867 |
| PYDC2 | ENST00000552634.1 | ENSG00000257496.1 | 0.839832566 | 1.879417672 | 0.060187485 |
| PYDC2 | ENST00000553477.1 | ENSG00000259123.1 | -0.816387736 | -1.830297438 | 0.067205474 |
| PYDC2 | ENST00000553537.1 | ENSG00000258481.1 | 0.838644612 | 1.87397571 | 0.060933773 |
| PYDC2 | ENST00000553954.1 | ENSG00000259052.1 | 0.84016805 | 1.875577537 | 0.060713313 |
| PYDC2 | ENST00000555460.1 | ENSG00000259042.1 | 0.957596269 | 2.1470608 | 0.031788442 |
| PYDC2 | ENST00000560969.1 | ENSG00000259176.1 | -0.815481128 | -1.779527289 | 0.075153355 |
| PYDC2 | ENST00000561254.1 | ENSG00000259554.1 | -0.829925666 | -1.853739638 | 0.063776418 |
| PYDC2 | ENST00000563018.1 | ENSG00000260193.1 | 0.818842181 | 1.853089809 | 0.063869487 |
| PYDC2 | ENST00000563342.1 | ENSG00000259914.1 | -0.801166836 | -1.773841241 | 0.076089414 |
| PYDC2 | ENST00000563610.1 | ENSG00000260051.1 | 0.872446758 | 1.932315327 | 0.053320598 |
| PYDC2 | ENST00000563855.1 | ENSG00000260658.1 | -0.958780102 | -2.167736335 | 0.030178753 |
| PYDC2 | ENST00000565310.1 | ENSG00000261118.1 | 0.901751404 | 2.026267276 | 0.042737403 |
| PYDC2 | ENST00000565735.1 | ENSG00000261213.1 | -0.949738158 | -2.092377016 | 0.036404803 |
| PYDC2 | ENST00000566449.1 | ENSG00000259791.1 | -0.859167481 | -1.901622962 | 0.057220464 |
| PYDC2 | ENST00000567067.1 | ENSG00000261600.1 | -0.8412308 | -1.855476553 | 0.063528204 |

| | | | | | |
|---|---|---|---|---|---|
| PYDC2 | ENST00000568410.1 | ENSG00000260277.1 | -0.833626147 | -1.88125543 | 0.059937181 |
| PYDC2 | ENST00000568659.1 | ENSG00000260004.1 | -0.866365994 | -1.918219283 | 0.055083212 |
| PYDC2 | ENST00000569778.1 | ENSG00000260823.1 | 0.801724401 | 1.793045688 | 0.072965619 |
| PYDC2 | ENST00000570700.1 | ENSG00000263011.1 | 0.828951079 | 1.848608957 | 0.064514298 |
| PYDC2 | ENST00000573260.1 | ENSG00000262482.1 | -0.939823109 | -2.090975021 | 0.036530303 |
| PYDC2 | ENST00000576021.1 | ENSG00000262413.1 | 0.912176405 | 2.039747259 | 0.041375505 |
| PYDC2 | ENST00000577853.1 | ENSG00000264207.1 | 0.885037959 | 1.98175164 | 0.047507044 |
| PYDC2 | ENST00000579775.1 | ENSG00000264108.1 | 0.850253534 | 1.898599681 | 0.05761713 |
| PYDC2 | ENST00000580729.1 | ENSG00000266176.1 | 0.801093146 | 1.798302636 | 0.072129062 |
| PYDC2 | ENST00000585684.1 | ENSG00000267057.1 | -0.969968762 | -2.132337061 | 0.032979147 |
| PYDC2 | ENST00000588842.1 | ENSG00000235779.3 | -0.985375257 | -2.194919333 | 0.028169385 |
| PYDC2 | ENST00000589983.1 | ENSG00000267057.1 | -0.883734246 | -1.97170701 | 0.048643063 |
| PYDC2 | ENST00000591469.1 | ENSG00000267374.1 | -0.917132176 | -2.044718078 | 0.04088266 |
| PYDC2 | ENST00000591621.1 | ENSG00000232116.2 | -0.92449163 | -2.029802791 | 0.042376589 |
| PYDC2 | ENST00000592022.1 | ENSG00000267383.2 | -0.833280974 | -1.851051435 | 0.064162154 |
| PYDC2 | ENST00000592045.1 | ENSG00000267057.1 | -0.922012067 | -2.060576245 | 0.039343485 |
| PYDC2 | ENST00000593175.1 | ENSG00000229036.3 | -0.902655589 | -2.00597859 | 0.044858532 |
| PYDC2 | ENST00000594776.1 | ENSG00000269807.1 | 0.802698173 | 1.783237483 | 0.074547653 |
| PYDC2 | ENST00000596567.1 | ENSG00000226647.2 | -0.880324172 | -1.976045561 | 0.048149617 |
| PYDC2 | ENST00000597680.1 | ENSG00000269574.1 | -0.903983298 | -2.00647781 | 0.044805294 |
| PYDC2 | ENST00000597755.1 | ENSG00000236194.2 | -0.941449077 | -2.10383819 | 0.035392564 |
| PYDC2 | ENST00000598065.1 | ENSG00000231731.3 | 0.949961675 | 2.121491204 | 0.033880487 |
| PYDC2 | ENST00000598092.1 | ENSG00000228065.6 | -0.871538506 | -1.948356266 | 0.051372349 |
| PYDC2 | ENST00000599050.1 | ENSG00000268366.1 | -0.804054411 | -1.807426243 | 0.070695845 |
| PYDC2 | ENST00000602620.1 | ENSG00000215386.6 | -0.857954545 | -1.927323119 | 0.053939374 |
| PYDC2 | ENST00000602736.1 | ENSG00000269976.1 | -0.837340099 | -1.88597282 | 0.059298617 |
| PYDC2 | ENST00000602881.1 | ENSG00000269965.1 | -0.928517539 | -2.090715852 | 0.036553543 |
| PYDC2 | ENST00000604312.1 | ENSG00000270947.1 | -0.920151331 | -2.074903101 | 0.037995516 |
| PYDC2 | ENST00000606010.1 | ENSG00000272249.1 | -0.881286048 | -1.949471888 | 0.051239097 |
| PYDC2 | ENST00000606482.1 | ENSG00000272416.1 | -0.811254862 | -1.814013895 | 0.069675587 |
| PYDC2 | ENST00000606855.1 | ENSG00000245937.3 | 0.82255024 | 1.830773607 | 0.067134341 |
| PYDC2 | ENST00000606869.1 | ENSG00000272349.1 | -0.926278404 | -2.04996441 | 0.040367904 |
| PYDC2 | ENST00000608088.1 | ENSG00000272632.1 | -0.827484327 | -1.8582262 | 0.063136899 |
| PYDC2 | ENST00000608133.1 | ENSG00000273193.1 | -0.907344845 | -2.022424335 | 0.043132533 |
| PYDC2 | ENST00000608259.1 | ENSG00000272627.1 | -0.883739725 | -2.005291135 | 0.044931932 |
| PYDC2 | ENST00000608465.1 | ENSG00000272758.1 | -0.854715115 | -1.946206503 | 0.051629939 |
| PYDC2 | ENST00000608489.1 | ENSG00000272716.1 | 0.876719992 | 1.95269327 | 0.050855952 |
| PYDC2 | ENST00000609807.1 | ENSG00000272700.1 | -0.94759967 | -2.120166768 | 0.033991984 |
| PYDC2 | ENST00000609976.1 | ENSG00000272582.1 | 0.812403131 | 1.796317181 | 0.072444087 |
| PYDC2 | ENST00000610270.1 | ENSG00000272576.1 | -0.8301397 | -1.850572651 | 0.064231057 |
| PYDC2 | NR_027067.1 | LINC00114 | 0.89680696 | 2.006791289 | 0.044771891 |
| PYDC2 | NR_027402.1 | FAM223B | 0.994008455 | 2.224348413 | 0.026125012 |
| PYDC2 | NR_027425.1 | FAM66D | 0.929639347 | 2.046383477 | 0.040718656 |
| PYDC2 | NR_027440.1 | LOC100272217 | 0.909342368 | 2.021582958 | 0.043219454 |
| PYDC2 | NR_033914.1 | LINC00254 | -0.885580048 | -1.992956444 | 0.046266217 |
| PYDC2 | NR_046748.1 | ARHGAP31-AS1 | -0.87114481 | -1.951419031 | 0.051007219 |
| PYDC2 | NR_046766.1 | ATP2B2-IT2 | -0.81418713 | -1.827804113 | 0.067578956 |
| PYDC2 | NR_046871.1 | LINC00333 | -0.853724441 | -1.909674478 | 0.056175139 |
| PYDC2 | NR_047698.1 | VWC2L-IT1 | -0.923211636 | -2.05107794 | 0.040259357 |
| PYDC2 | NR_109877.1 | LINC01470 | -0.982657583 | -2.20099467 | 0.027736401 |
| PYDC2 | NR_110117.1 | LOC101927769 | -0.929600516 | -2.096443725 | 0.036042847 |

| | | | | | |
|---|---|---|---|---|---|
| PYDC2 | NR_110123.1 | GRM7-AS3 | -0.841647302 | -1.882802415 | 0.05972715 |
| PYDC2 | NR_110245.1 | LOC101929282 | -0.846114112 | -1.898704574 | 0.05760333 |
| PYDC2 | NR_110556.1 | LOC102724890 | -0.932346102 | -2.090805774 | 0.036545478 |
| PYDC2 | NR_110630.1 | LOC101927478 | 0.818612779 | 1.853056134 | 0.063874313 |
| PYDC2 | NR_110731.1 | LINC01232 | 0.821282268 | 1.812610035 | 0.069891989 |
| PYDC2 | NR_125769.1 | LINC01269 | 0.860831414 | 1.915993978 | 0.055365862 |
| PYDC2 | NR_126409.1 | LINC00376 | -0.816892655 | -1.836511423 | 0.066282054 |
| PYDC2 | NR_134573.1 | GS1-124K5.4 | -0.893250522 | -2.021632011 | 0.043214382 |
| PYDC2 | NR_134610.1 | LOC105375014 | 0.900773343 | 2.029500103 | 0.042407379 |
| PYDC2 | NR_134632.1 | LOC105373051 | 0.89041221 | 1.989072375 | 0.046693215 |
| PYDC2 | NR_135040.1 | LOC101927038 | 0.92975317 | 2.104048792 | 0.035374191 |
| PYDC2 | NR_135549.1 | LOC101929411 | -0.870432686 | -1.940797154 | 0.052282886 |
| PYDC2 | NR_135584.1 | LOC101927596 | 0.808625443 | 1.810332149 | 0.070244294 |
| PYDC2 | NR_136178.1 | LOC101928166 | -0.802634082 | -1.798864959 | 0.072040045 |
| PYDC2 | NR_136218.1 | MEF2C-AS1 | -0.929815179 | -2.069525173 | 0.038496832 |
| RMRP | ENST00000413549.1 | ENSG00000231609.1 | -0.932787642 | -2.086851376 | 0.036901571 |
| RMRP | ENST00000416329.1 | ENSG00000233184.2 | -0.939036383 | -2.1245916 | 0.033620705 |
| RMRP | ENST00000416595.1 | ENSG00000223623.1 | -0.80391429 | -1.794550734 | 0.072725309 |
| RMRP | ENST00000419662.1 | ENSG00000228265.1 | -0.878655172 | -1.941685526 | 0.052175182 |
| RMRP | ENST00000420315.1 | ENSG00000228072.1 | -0.824079632 | -1.849179764 | 0.06443186 |
| RMRP | ENST00000423869.1 | ENSG00000227848.1 | -0.857558033 | -1.897714873 | 0.057733652 |
| RMRP | ENST00000424257.1 | ENSG00000231626.1 | 0.821927101 | 1.855861557 | 0.063473294 |
| RMRP | ENST00000424852.1 | ENSG00000229891.1 | 0.868615875 | 1.957719933 | 0.050262885 |
| RMRP | ENST00000433051.1 | ENSG00000233193.1 | -0.917069663 | -2.043910175 | 0.040962422 |
| RMRP | ENST00000439184.1 | ENSG00000233985.1 | 0.87474247 | 1.942324108 | 0.052097876 |
| RMRP | ENST00000440595.1 | ENSG00000228265.1 | -0.836905354 | -1.883791784 | 0.059593145 |
| RMRP | ENST00000442649.1 | ENSG00000234089.1 | 0.960235397 | 2.165602754 | 0.030341556 |
| RMRP | ENST00000448491.1 | ENSG00000231212.1 | 0.951810232 | 2.112497142 | 0.034643831 |
| RMRP | ENST00000449457.1 | ENSG00000224034.1 | 0.81734601 | 1.848414904 | 0.064542344 |
| RMRP | ENST00000449473.1 | ENSG00000233184.2 | -0.977135674 | -2.146222762 | 0.03185521 |
| RMRP | ENST00000450063.1 | ENSG00000231210.2 | 0.851508121 | 1.885161966 | 0.059407974 |
| RMRP | ENST00000450072.1 | ENSG00000228486.5 | -0.899275287 | -2.000966119 | 0.045396041 |
| RMRP | ENST00000450480.1 | ENSG00000231295.1 | 0.966976076 | 2.179006792 | 0.029331164 |
| RMRP | ENST00000457253.1 | ENSG00000225173.1 | -0.803363761 | -1.812161057 | 0.069961314 |
| RMRP | ENST00000457975.2 | ENSG00000236744.2 | 0.80095737 | 1.8029478 | 0.071396413 |
| RMRP | ENST00000463297.1 | ENSG00000243486.1 | 0.962957969 | 2.098290735 | 0.035879471 |
| RMRP | ENST00000473329.1 | ENSG00000243849.1 | 0.867220862 | 1.94550789 | 0.051713881 |
| RMRP | ENST00000494509.1 | ENSG00000240095.1 | -0.809249915 | -1.8050582 | 0.071065575 |
| RMRP | ENST00000503571.1 | ENSG00000249592.1 | -0.862539791 | -1.918389188 | 0.055061681 |
| RMRP | ENST00000504916.1 | ENSG00000248112.1 | 0.903768089 | 2.033835727 | 0.041968161 |
| RMRP | ENST00000510922.1 | ENSG00000250777.1 | 0.922246589 | 2.054504478 | 0.03992689 |
| RMRP | ENST00000514661.1 | ENSG00000247993.2 | -0.884876745 | -1.965742121 | 0.049328407 |
| RMRP | ENST00000514737.1 | ENSG00000250597.1 | -0.888003728 | -1.957682175 | 0.050267318 |
| RMRP | ENST00000521055.1 | ENSG00000253184.1 | 0.892177017 | 1.989372178 | 0.046660138 |
| RMRP | ENST00000530435.1 | ENSG00000254630.1 | -0.88865015 | -2.001546712 | 0.045333505 |
| RMRP | ENST00000531009.1 | ENSG00000255208.1 | -0.806769111 | -1.822536869 | 0.068373565 |
| RMRP | ENST00000534065.1 | ENSG00000254458.1 | -0.953063017 | -2.144252756 | 0.03201264 |
| RMRP | ENST00000545254.1 | ENSG00000256633.1 | -0.937526437 | -2.103115891 | 0.03545564 |
| RMRP | ENST00000555342.1 | ENSG00000259048.1 | 0.985502135 | 2.217371891 | 0.026597689 |
| RMRP | ENST00000559960.1 | ENSG00000259354.1 | -0.850248573 | -1.880420431 | 0.060050801 |
| RMRP | ENST00000561567.1 | ENSG00000260177.1 | -0.86040574 | -1.929871689 | 0.053622738 |

| | | | | | |
|---|---|---|---|---|---|
| RMRP | ENST00000563639.2 | ENSG00000260457.2 | 0.840380837 | 1.889091435 | 0.058879578 |
| RMRP | ENST00000564417.1 | ENSG00000260137.1 | 0.873922308 | 1.955687736 | 0.05050195 |
| RMRP | ENST00000566170.1 | ENSG00000261071.1 | -0.841511156 | -1.876549908 | 0.060579808 |
| RMRP | ENST00000568033.1 | ENSG00000261480.1 | -0.895697438 | -1.994985208 | 0.046044493 |
| RMRP | ENST00000574387.1 | ENSG00000262370.1 | -0.929502795 | -2.077705253 | 0.037736515 |
| RMRP | ENST00000579050.1 | ENSG00000264290.1 | -0.882374036 | -1.972884281 | 0.048508748 |
| RMRP | ENST00000581940.1 | ENSG00000265484.1 | -0.817346924 | -1.812634888 | 0.069888153 |
| RMRP | ENST00000582386.1 | ENSG00000265174.1 | -0.869039642 | -1.923324719 | 0.054439282 |
| RMRP | ENST00000583067.1 | ENSG00000266126.1 | 0.927450473 | 2.055749854 | 0.039806634 |
| RMRP | ENST00000586694.1 | ENSG00000267141.1 | -0.896454111 | -2.008135222 | 0.044628924 |
| RMRP | ENST00000593218.1 | ENSG00000267421.2 | -0.816580071 | -1.821320244 | 0.068558191 |
| RMRP | ENST00000593588.1 | ENSG00000269635.1 | -0.803233105 | -1.808916505 | 0.070463976 |
| RMRP | ENST00000597169.1 | ENSG00000269720.1 | -0.834408773 | -1.85812488 | 0.063151282 |
| RMRP | ENST00000601735.1 | ENSG00000244513.2 | -0.88174933 | -1.976536112 | 0.04809409 |
| RMRP | ENST00000602594.1 | ENSG00000269930.1 | 0.864737892 | 1.949204632 | 0.051270992 |
| RMRP | ENST00000602598.1 | ENSG00000269944.1 | -0.859577721 | -1.940805173 | 0.052281913 |
| RMRP | ENST00000602900.1 | ENSG00000270179.1 | 0.812322933 | 1.822013989 | 0.068452863 |
| RMRP | ENST00000604464.1 | ENSG00000270462.1 | -0.816642789 | -1.824418501 | 0.068088827 |
| RMRP | ENST00000604793.1 | ENSG00000228486.5 | -0.922920795 | -2.079902088 | 0.037534515 |
| RMRP | ENST00000606277.1 | ENSG00000272145.1 | -0.878908135 | -1.996629002 | 0.0458655 |
| RMRP | ENST00000606377.1 | ENSG00000272286.1 | 0.885896372 | 2.001461366 | 0.045342693 |
| RMRP | ENST00000606963.1 | ENSG00000272010.1 | 0.962375455 | 2.1106217 | 0.034804841 |
| RMRP | ENST00000607580.1 | ENSG00000272545.1 | 0.826909434 | 1.843261071 | 0.065290902 |
| RMRP | ENST00000607991.1 | ENSG00000273076.1 | 0.849182688 | 1.871244251 | 0.061311234 |
| RMRP | ENST00000609218.1 | ENSG00000272945.1 | -0.88059068 | -1.995155711 | 0.0460259 |
| RMRP | ENST00000610007.1 | ENSG00000272660.1 | -0.836090286 | -1.873133935 | 0.061049892 |
| RMRP | NR_046742.2 | ZNF630-AS1 | 0.860106204 | 1.916353064 | 0.055320171 |
| RMRP | NR_046783.1 | KCND3-IT1 | 0.915955627 | 2.037045793 | 0.041645453 |
| RMRP | NR_047116.1 | HIF1A-AS1 | 0.828459295 | 1.84242987 | 0.065412296 |
| RMRP | NR_109885.1 | RALY-AS1 | -0.83270787 | -1.863658117 | 0.062369731 |
| RMRP | NR_109886.1 | RALY-AS1 | -0.861766778 | -1.933288788 | 0.053200632 |
| RMRP | NR_110808.1 | LOC101927557 | 0.865354126 | 1.955857637 | 0.050481927 |
| RMRP | NR_120595.1 | LINC01315 | 0.836916531 | 1.874465201 | 0.060866334 |
| RMRP | NR_121577.1 | NALT1 | 0.856795901 | 1.915561378 | 0.05542095 |
| RMRP | NR_125759.1 | PKNOX2-AS1 | 0.840741132 | 1.896316589 | 0.057918194 |
| RMRP | NR_134252.1 | LOC105379030 | -0.972786754 | -2.157227693 | 0.030987928 |
| RMRP | NR_138084.1 | HCG24 | -0.845456478 | -1.903278941 | 0.057004157 |
| RNASE2 | ENST00000340585.6 | ENSG00000249429.1 | -0.891777196 | -1.991561191 | 0.046419225 |
| RNASE2 | ENST00000412085.1 | ENSG00000233825.1 | 0.941138288 | 2.135675448 | 0.032705877 |
| RNASE2 | ENST00000412759.1 | ENSG00000236933.1 | 0.936753894 | 2.078233022 | 0.037687902 |
| RNASE2 | ENST00000413887.1 | ENSG00000236948.1 | -0.849096706 | -1.890981152 | 0.05862686 |
| RNASE2 | ENST00000414740.2 | ENSG00000229646.2 | 0.911380994 | 2.040205218 | 0.04132989 |
| RNASE2 | ENST00000416329.1 | ENSG00000233184.2 | 0.832213163 | 1.836397296 | 0.066298919 |
| RNASE2 | ENST00000419662.1 | ENSG00000228265.1 | 0.900653277 | 2.02391988 | 0.042978396 |
| RNASE2 | ENST00000419734.1 | ENSG00000234646.1 | -0.902048532 | -2.040063439 | 0.041344008 |
| RNASE2 | ENST00000420315.1 | ENSG00000228072.1 | 0.829811238 | 1.846245674 | 0.064856542 |
| RNASE2 | ENST00000421020.1 | ENSG00000231407.1 | 0.845467253 | 1.886562949 | 0.059219134 |
| RNASE2 | ENST00000421207.1 | ENSG00000231768.1 | 0.827690471 | 1.83979327 | 0.065798594 |
| RNASE2 | ENST00000423869.1 | ENSG00000227848.1 | 0.909441233 | 2.035154205 | 0.041835359 |
| RNASE2 | ENST00000424852.1 | ENSG00000229891.1 | -0.836108922 | -1.863272997 | 0.062423867 |
| RNASE2 | ENST00000426475.1 | ENSG00000239467.1 | 0.812613319 | 1.858137683 | 0.063149465 |

| | | | | | |
|---|---|---|---|---|---|
| RNASE2 | ENST00000426519.1 | ENSG00000234142.1 | 0.942728083 | 2.100976206 | 0.035643055 |
| RNASE2 | ENST00000429681.1 | ENSG00000235236.1 | -0.808021933 | -1.794356149 | 0.072756341 |
| RNASE2 | ENST00000430025.1 | ENSG00000233508.1 | -0.863092894 | -1.911522191 | 0.055937508 |
| RNASE2 | ENST00000433036.1 | ENSG00000228989.1 | 0.83425271 | 1.856589156 | 0.063369628 |
| RNASE2 | ENST00000433876.2 | ENSG00000228423.2 | 0.874371654 | 1.936945245 | 0.052752037 |
| RNASE2 | ENST00000433905.2 | ENSG00000229299.2 | 0.857192647 | 1.923377249 | 0.054432689 |
| RNASE2 | ENST00000434627.1 | ENSG00000230074.1 | 0.896437528 | 2.007151587 | 0.044733525 |
| RNASE2 | ENST00000435892.1 | ENSG00000233635.2 | 0.90087619 | 2.036303198 | 0.041719919 |
| RNASE2 | ENST00000438190.1 | ENSG00000227214.2 | 0.88701262 | 1.998959996 | 0.045612682 |
| RNASE2 | ENST00000439184.1 | ENSG00000233985.1 | -0.93873837 | -2.099988296 | 0.035729871 |
| RNASE2 | ENST00000440714.1 | ENSG00000237609.1 | 0.899053563 | 2.008009065 | 0.044642328 |
| RNASE2 | ENST00000442649.1 | ENSG00000234089.1 | -0.806870083 | -1.7887106 | 0.073661435 |
| RNASE2 | ENST00000442850.1 | ENSG00000232600.2 | -0.858765156 | -1.917970819 | 0.055114711 |
| RNASE2 | ENST00000447206.1 | ENSG00000230839.1 | 0.849360691 | 1.88757029 | 0.059083662 |
| RNASE2 | ENST00000447343.2 | ENSG00000229299.2 | 0.906337449 | 2.044705069 | 0.040883944 |
| RNASE2 | ENST00000448491.1 | ENSG00000231212.1 | -0.824726801 | -1.819983492 | 0.068761519 |
| RNASE2 | ENST00000449463.1 | ENSG00000230309.1 | -0.920311134 | -2.061921738 | 0.039215188 |
| RNASE2 | ENST00000450063.1 | ENSG00000231210.2 | -0.885956781 | -1.988561891 | 0.046749581 |
| RNASE2 | ENST00000453051.1 | ENSG00000229407.1 | 0.935964084 | 2.053421771 | 0.04003169 |
| RNASE2 | ENST00000454530.1 | ENSG00000226649.1 | -0.884637967 | -2.034420544 | 0.041909212 |
| RNASE2 | ENST00000457253.1 | ENSG00000225173.1 | 0.93069901 | 2.094097117 | 0.03625133 |
| RNASE2 | ENST00000457975.2 | ENSG00000236744.2 | -0.807090071 | -1.832216605 | 0.066919156 |
| RNASE2 | ENST00000458154.1 | ENSG00000235578.1 | 0.845897036 | 1.898951383 | 0.057570869 |
| RNASE2 | ENST00000458194.1 | ENSG00000226193.1 | 0.917502453 | 2.060348346 | 0.039365251 |
| RNASE2 | ENST00000458364.1 | ENSG00000225655.1 | -0.801662289 | -1.802483661 | 0.071469343 |
| RNASE2 | ENST00000489077.1 | ENSG00000244198.1 | 0.864442167 | 1.929831614 | 0.053627705 |
| RNASE2 | ENST00000490013.1 | ENSG00000184115.12 | 0.873748071 | 1.92538605 | 0.05418108 |
| RNASE2 | ENST00000498693.1 | ENSG00000244198.1 | 0.900119366 | 2.001679032 | 0.045319263 |
| RNASE2 | ENST00000504916.1 | ENSG00000248112.1 | -0.86817768 | -1.92347417 | 0.054420527 |
| RNASE2 | ENST00000505196.1 | ENSG00000248131.1 | 0.837665149 | 1.897546652 | 0.057755828 |
| RNASE2 | ENST00000505556.1 | ENSG00000249409.1 | 0.904454617 | 1.998882595 | 0.045621058 |
| RNASE2 | ENST00000506100.1 | ENSG00000249409.1 | 0.899210814 | 2.025536419 | 0.042812313 |
| RNASE2 | ENST00000506791.1 | ENSG00000251131.1 | 0.860332184 | 1.890642959 | 0.058672022 |
| RNASE2 | ENST00000508083.1 | ENSG00000249343.1 | 0.856094686 | 1.921571634 | 0.054659681 |
| RNASE2 | ENST00000508188.1 | ENSG00000250999.1 | 0.850983513 | 1.902198664 | 0.057145187 |
| RNASE2 | ENST00000509036.1 | ENSG00000251131.1 | 0.935545041 | 2.08804899 | 0.036793416 |
| RNASE2 | ENST00000514411.1 | ENSG00000250882.1 | 0.813565921 | 1.821739176 | 0.068494571 |
| RNASE2 | ENST00000514661.1 | ENSG00000247993.2 | 0.889773779 | 1.971864764 | 0.048625047 |
| RNASE2 | ENST00000514877.1 | ENSG00000248685.1 | 0.930116618 | 2.066764715 | 0.03875633 |
| RNASE2 | ENST00000517300.1 | ENSG00000254144.2 | 0.866560186 | 1.941356915 | 0.052215 |
| RNASE2 | ENST00000517846.1 | ENSG00000254485.1 | 0.823427405 | 1.852667241 | 0.063930068 |
| RNASE2 | ENST00000520603.1 | ENSG00000254001.1 | -0.835184147 | -1.865273175 | 0.062143123 |
| RNASE2 | ENST00000521055.1 | ENSG00000253184.1 | -0.814890745 | -1.806644497 | 0.070817727 |
| RNASE2 | ENST00000522547.1 | ENSG00000253430.1 | -0.883471685 | -1.976689794 | 0.048076705 |
| RNASE2 | ENST00000524094.1 | ENSG00000248555.2 | -0.819851682 | -1.822073476 | 0.068443838 |
| RNASE2 | ENST00000528000.1 | ENSG00000254804.1 | 0.871033953 | 1.975832164 | 0.048173789 |
| RNASE2 | ENST00000534065.1 | ENSG00000254458.1 | 0.868603378 | 1.944264487 | 0.051863565 |
| RNASE2 | ENST00000536141.1 | ENSG00000256969.1 | 0.810126745 | 1.808300047 | 0.070559815 |
| RNASE2 | ENST00000543072.1 | ENSG00000256092.2 | -0.885944592 | -1.965386575 | 0.049369512 |
| RNASE2 | ENST00000543275.1 | ENSG00000256944.1 | 0.821722951 | 1.840829373 | 0.065646567 |
| RNASE2 | ENST00000545177.3 | ENSG00000230438.5 | 0.913741538 | 2.038139293 | 0.041536005 |

| | | | | | |
|---|---|---|---|---|---|
| RNASE2 | ENST00000545254.1 | ENSG00000256633.1 | 0.845783957 | 1.895583387 | 0.058015156 |
| RNASE2 | ENST00000551361.1 | ENSG00000224078.8 | -0.833276277 | -1.873259733 | 0.061032527 |
| RNASE2 | ENST00000555342.1 | ENSG00000259048.1 | -0.861762915 | -1.902355403 | 0.057124707 |
| RNASE2 | ENST00000558515.1 | ENSG00000259182.1 | 0.807012068 | 1.80510091 | 0.071058893 |
| RNASE2 | ENST00000561567.1 | ENSG00000260177.1 | 0.850471583 | 1.909357561 | 0.056215981 |
| RNASE2 | ENST00000563449.2 | ENSG00000261613.2 | 0.824079992 | 1.83693952 | 0.066218824 |
| RNASE2 | ENST00000564417.1 | ENSG00000260137.1 | -0.897551693 | -2.013927535 | 0.044017142 |
| RNASE2 | ENST00000565829.1 | ENSG00000260148.1 | 0.807728234 | 1.799421917 | 0.071951965 |
| RNASE2 | ENST00000566170.1 | ENSG00000261071.1 | 0.843140989 | 1.888740104 | 0.058926662 |
| RNASE2 | ENST00000568033.1 | ENSG00000261480.1 | 0.849730039 | 1.88594875 | 0.059301861 |
| RNASE2 | ENST00000570843.1 | ENSG00000261889.1 | 0.878285692 | 1.958263588 | 0.050199091 |
| RNASE2 | ENST00000570929.1 | ENSG00000262223.2 | 0.809689678 | 1.79409565 | 0.072797903 |
| RNASE2 | ENST00000574460.1 | ENSG00000263051.1 | 0.916254895 | 2.066051949 | 0.038823575 |
| RNASE2 | ENST00000575139.1 | ENSG00000263072.1 | 0.870149578 | 1.94218814 | 0.052114328 |
| RNASE2 | ENST00000578800.1 | ENSG00000264235.1 | 0.937164325 | 2.069578719 | 0.038491813 |
| RNASE2 | ENST00000578936.1 | ENSG00000265547.1 | 0.910074425 | 2.02507434 | 0.042859731 |
| RNASE2 | ENST00000579154.1 | ENSG00000265908.1 | -0.837390273 | -1.87714478 | 0.060498253 |
| RNASE2 | ENST00000581905.1 | ENSG00000264235.1 | 0.894735016 | 1.987917491 | 0.046820816 |
| RNASE2 | ENST00000581940.1 | ENSG00000265484.1 | 0.802205944 | 1.777515762 | 0.07548342 |
| RNASE2 | ENST00000582044.1 | ENSG00000263715.2 | 0.802788036 | 1.802779644 | 0.071422828 |
| RNASE2 | ENST00000583138.1 | ENSG00000263393.1 | 0.80994892 | 1.837244638 | 0.066173789 |
| RNASE2 | ENST00000585072.1 | ENSG00000263745.1 | 0.896988904 | 1.984172727 | 0.047236588 |
| RNASE2 | ENST00000586051.1 | ENSG00000267576.1 | 0.865803601 | 1.92508274 | 0.054219008 |
| RNASE2 | ENST00000586694.1 | ENSG00000267141.1 | 0.829901152 | 1.834558966 | 0.066571063 |
| RNASE2 | ENST00000587281.1 | ENSG00000228290.2 | 0.881091882 | 1.963709635 | 0.049563775 |
| RNASE2 | ENST00000588182.2 | ENSG00000267453.2 | 0.86586622 | 1.930241927 | 0.053576869 |
| RNASE2 | ENST00000588290.1 | ENSG00000267751.1 | 0.839928052 | 1.910456529 | 0.056074458 |
| RNASE2 | ENST00000593139.1 | ENSG00000267042.1 | 0.807381998 | 1.845342065 | 0.064987795 |
| RNASE2 | ENST00000593218.1 | ENSG00000267421.2 | 0.863358732 | 1.910834877 | 0.056025804 |
| RNASE2 | ENST00000593588.1 | ENSG00000269635.1 | 0.916603706 | 2.057884099 | 0.03960126 |
| RNASE2 | ENST00000594590.2 | ENSG00000268199.2 | 0.858280934 | 1.922920057 | 0.05449009 |
| RNASE2 | ENST00000595955.1 | ENSG00000268401.1 | 0.894344425 | 2.001368943 | 0.045352645 |
| RNASE2 | ENST00000597169.1 | ENSG00000269720.1 | 0.9242987 | 2.059922086 | 0.03940599 |
| RNASE2 | ENST00000597256.1 | ENSG00000267986.1 | 0.891224683 | 1.997181674 | 0.045805451 |
| RNASE2 | ENST00000599352.1 | ENSG00000240401.4 | -0.911579825 | -2.038047015 | 0.041545231 |
| RNASE2 | ENST00000600234.1 | ENSG00000268078.1 | 0.944666894 | 2.099727025 | 0.035752861 |
| RNASE2 | ENST00000600889.1 | ENSG00000232675.3 | 0.872339279 | 1.950552499 | 0.051110302 |
| RNASE2 | ENST00000601033.1 | ENSG00000268401.1 | 0.946292473 | 2.124712885 | 0.033610578 |
| RNASE2 | ENST00000601735.1 | ENSG00000244513.2 | 0.866316354 | 1.924293075 | 0.054317858 |
| RNASE2 | ENST00000602532.1 | ENSG00000270091.1 | 0.896942303 | 1.991311657 | 0.046446635 |
| RNASE2 | ENST00000604142.1 | ENSG00000271308.1 | 0.873538185 | 1.932360736 | 0.053314997 |
| RNASE2 | ENST00000606277.1 | ENSG00000272145.1 | 0.971998107 | 2.171373082 | 0.029902982 |
| RNASE2 | ENST00000606377.1 | ENSG00000272286.1 | -0.835459338 | -1.870211555 | 0.061454446 |
| RNASE2 | ENST00000606778.1 | ENSG00000271930.1 | 0.817456101 | 1.81229143 | 0.069941178 |
| RNASE2 | ENST00000606963.1 | ENSG00000272010.1 | -0.878821929 | -1.964157143 | 0.049511871 |
| RNASE2 | ENST00000607052.1 | ENSG00000271870.1 | -0.814889835 | -1.815600861 | 0.069431622 |
| RNASE2 | ENST00000607201.1 | ENSG00000272024.1 | -0.801774728 | -1.786941222 | 0.073946989 |
| RNASE2 | ENST00000607224.1 | ENSG00000272521.1 | 0.920518002 | 2.055101204 | 0.039869231 |
| RNASE2 | ENST00000607321.1 | ENSG00000272371.1 | -0.87169805 | -1.943227584 | 0.051988667 |
| RNASE2 | ENST00000607476.1 | ENSG00000272540.1 | 0.892530149 | 1.983276592 | 0.047336543 |
| RNASE2 | ENST00000607580.1 | ENSG00000272545.1 | -0.834378892 | -1.8513509 | 0.064119088 |

| | | | | | |
|---|---|---|---|---|---|
| RNASE2 | ENST00000608677.1 | ENSG00000273350.1 | 0.959008942 | 2.146080717 | 0.031866539 |
| RNASE2 | ENST00000608940.1 | ENSG00000272763.1 | 0.831570614 | 1.863554651 | 0.062384271 |
| RNASE2 | ENST00000609218.1 | ENSG00000272945.1 | 0.838277935 | 1.874473261 | 0.060865224 |
| RNASE2 | NR_003604.2 | ZFAS1 | 0.89069024 | 1.993420286 | 0.046215444 |
| RNASE2 | NR_003605.1 | ZFAS1 | 0.815669132 | 1.832614936 | 0.066859855 |
| RNASE2 | NR_003606.2 | ZFAS1 | 0.908775957 | 2.044831892 | 0.040871434 |
| RNASE2 | NR_024321.1 | LINC00115 | 0.828775129 | 1.853487531 | 0.063812512 |
| RNASE2 | NR_026802.1 | FAM74A4 | 0.928436076 | 2.078767604 | 0.037638716 |
| RNASE2 | NR_026813.1 | LINC00597 | -0.85367174 | -1.90770578 | 0.056429254 |
| RNASE2 | NR_026951.1 | LINC00324 | 0.82212262 | 1.848653851 | 0.064507811 |
| RNASE2 | NR_027052.1 | THAP7-AS1 | 0.83515815 | 1.889908552 | 0.058770192 |
| RNASE2 | NR_027271.1 | CIRBP-AS1 | 0.923257472 | 2.068986661 | 0.038547338 |
| RNASE2 | NR_034037.1 | LINC00582 | -0.860283696 | -1.911971542 | 0.055879844 |
| RNASE2 | NR_036658.1 | ZFAS1 | 0.907137544 | 2.040327562 | 0.041317711 |
| RNASE2 | NR_040096.1 | LOC643339 | 0.871909574 | 1.946579072 | 0.05158522 |
| RNASE2 | NR_045114.1 | PVRL3-AS1 | -0.804081709 | -1.800720776 | 0.071746901 |
| RNASE2 | NR_045637.1 | BOLA3-AS1 | 0.842656071 | 1.883559471 | 0.059624588 |
| RNASE2 | NR_047116.1 | HIF1A-AS1 | -0.904628197 | -2.033890878 | 0.041962599 |
| RNASE2 | NR_103790.1 | LINC00581 | -0.92831899 | -2.090827704 | 0.036543512 |
| RNASE2 | NR_103851.1 | TAT-AS1 | 0.850289113 | 1.892152147 | 0.058470712 |
| RNASE2 | NR_105010.1 | LINC01333 | 0.911998641 | 2.018191599 | 0.043571311 |
| RNASE2 | NR_109886.1 | RALY-AS1 | 0.895716468 | 2.002924866 | 0.045185354 |
| RNASE2 | NR_110568.1 | LOC101927661 | 0.855463866 | 1.895595817 | 0.058013511 |
| RNASE2 | NR_110941.1 | MIR762HG | 0.873276209 | 1.950157304 | 0.051157373 |
| RNASE2 | NR_110998.1 | FAM74A4 | 0.928436076 | 2.099495528 | 0.035773242 |
| RNASE2 | NR_120595.1 | LINC01315 | -0.869578696 | -1.945373083 | 0.051730092 |
| RNASE2 | NR_126522.1 | EXOC3-AS1 | 0.871122021 | 1.956408298 | 0.050417075 |
| RNASE2 | NR_130143.1 | LOC104968399 | 0.955338875 | 2.146973109 | 0.031795423 |
| RNASE2 | NR_130144.1 | LOC104968399 | 0.894735016 | 2.01732837 | 0.043661257 |
| RNASE2 | NR_131985.1 | CRAT8 | -0.809260017 | -1.803104727 | 0.071371769 |
| RNASE2 | NR_134252.1 | LOC105379030 | 0.824622229 | 1.843497512 | 0.065256405 |
| RNASE2 | NR_135024.1 | LOC105369747 | 0.828270133 | 1.849382698 | 0.064402572 |
| RPL3L | ENST00000318291.4 | ENSG00000177406.4 | 0.900624682 | 2.01842183 | 0.043547348 |
| RPL3L | ENST00000411694.1 | ENSG00000225331.1 | 0.919341977 | 2.088805315 | 0.036725252 |
| RPL3L | ENST00000418387.1 | ENSG00000235056.1 | -0.821021728 | -1.834560105 | 0.066570894 |
| RPL3L | ENST00000419662.1 | ENSG00000228265.1 | 0.812691222 | 1.813745881 | 0.069716858 |
| RPL3L | ENST00000420981.2 | ENSG00000230438.5 | 0.865678385 | 1.920883216 | 0.054746432 |
| RPL3L | ENST00000421020.1 | ENSG00000231407.1 | 0.89190924 | 1.98631241 | 0.046998646 |
| RPL3L | ENST00000422017.1 | ENSG00000232227.1 | -0.852197826 | -1.902067344 | 0.057162351 |
| RPL3L | ENST00000424181.1 | ENSG00000224977.1 | 0.827053093 | 1.841447092 | 0.065556067 |
| RPL3L | ENST00000426030.2 | ENSG00000228686.2 | -0.850227046 | -1.892474658 | 0.058427767 |
| RPL3L | ENST00000435733.1 | ENSG00000226377.1 | 0.949406806 | 2.113917747 | 0.034522293 |
| RPL3L | ENST00000435992.2 | ENSG00000232675.3 | 0.818905744 | 1.82701841 | 0.067697001 |
| RPL3L | ENST00000442069.1 | ENSG00000225655.1 | -0.842445141 | -1.899624114 | 0.057482465 |
| RPL3L | ENST00000442829.1 | ENSG00000225284.1 | 0.86892667 | 1.921218032 | 0.054704226 |
| RPL3L | ENST00000448748.1 | ENSG00000231238.1 | -0.840144073 | -1.868263984 | 0.061725284 |
| RPL3L | ENST00000452176.1 | ENSG00000223659.1 | -0.945183435 | -2.122815384 | 0.033769325 |
| RPL3L | ENST00000455788.1 | ENSG00000236263.1 | 0.899503706 | 1.993814595 | 0.04617232 |
| RPL3L | ENST00000457115.1 | ENSG00000227245.1 | 0.872849407 | 1.957036065 | 0.050343228 |
| RPL3L | ENST00000457253.1 | ENSG00000225173.1 | 0.809125013 | 1.774107624 | 0.07604535 |
| RPL3L | ENST00000457371.1 | ENSG00000237401.2 | 0.843541705 | 1.863361553 | 0.062411415 |

| | | | | | |
|---|---|---|---|---|---|
| RPL3L | ENST00000458154.1 | ENSG00000235578.1 | 0.888353565 | 1.9889238 | 0.046709614 |
| RPL3L | ENST00000459985.1 | ENSG00000273066.1 | 0.802067309 | 1.802351041 | 0.071490193 |
| RPL3L | ENST00000463255.1 | ENSG00000243305.1 | -0.849804859 | -1.883760452 | 0.059597385 |
| RPL3L | ENST00000466431.2 | ENSG00000254485.1 | 0.813701298 | 1.812253739 | 0.069946999 |
| RPL3L | ENST00000489690.1 | ENSG00000243944.1 | -0.893437347 | -2.022867466 | 0.043086814 |
| RPL3L | ENST00000494509.1 | ENSG00000240095.1 | 0.826474219 | 1.834202758 | 0.066623902 |
| RPL3L | ENST00000500496.2 | ENSG00000245479.2 | 0.812365435 | 1.817017804 | 0.069214387 |
| RPL3L | ENST00000502467.1 | ENSG00000250530.1 | -0.856546726 | -1.925538617 | 0.05416201 |
| RPL3L | ENST00000506791.1 | ENSG00000251131.1 | 0.819552607 | 1.82589845 | 0.06786556 |
| RPL3L | ENST00000509036.1 | ENSG00000251131.1 | 0.842526337 | 1.888471258 | 0.058962713 |
| RPL3L | ENST00000515128.1 | ENSG00000248215.1 | -0.860529272 | -1.90965401 | 0.056177776 |
| RPL3L | ENST00000521307.1 | ENSG00000253177.1 | 0.853491814 | 1.899541052 | 0.057493374 |
| RPL3L | ENST00000522600.1 | ENSG00000246582.2 | 0.83296087 | 1.859957322 | 0.062891564 |
| RPL3L | ENST00000526154.1 | ENSG00000254511.1 | 0.89375 | 1.984806717 | 0.04716598 |
| RPL3L | ENST00000526694.1 | ENSG00000231999.2 | 0.841285005 | 1.875927118 | 0.060665288 |
| RPL3L | ENST00000529247.1 | ENSG00000254741.1 | 0.825300334 | 1.848303712 | 0.064558419 |
| RPL3L | ENST00000532688.1 | ENSG00000255441.1 | 0.846504506 | 1.875598546 | 0.060710426 |
| RPL3L | ENST00000534178.1 | ENSG00000255120.1 | 0.848305502 | 1.893392925 | 0.058305636 |
| RPL3L | ENST00000537921.1 | ENSG00000255966.1 | 0.804617278 | 1.792373728 | 0.07307312 |
| RPL3L | ENST00000545177.3 | ENSG00000230438.5 | 0.863293164 | 1.938051186 | 0.052616978 |
| RPL3L | ENST00000548722.2 | ENSG00000257194.2 | -0.960178845 | -2.124260063 | 0.033648403 |
| RPL3L | ENST00000549806.1 | ENSG00000257252.1 | 0.838755605 | 1.856411602 | 0.063394912 |
| RPL3L | ENST00000549878.1 | ENSG00000257284.1 | 0.801287183 | 1.799358842 | 0.071961936 |
| RPL3L | ENST00000554431.1 | ENSG00000258616.1 | -0.901341838 | -2.019202618 | 0.043466164 |
| RPL3L | ENST00000557602.1 | ENSG00000258616.1 | -0.888810071 | -2.010063675 | 0.04442445 |
| RPL3L | ENST00000558475.1 | ENSG00000259604.1 | 0.873120837 | 1.94953425 | 0.051231657 |
| RPL3L | ENST00000559673.1 | ENSG00000259604.1 | 0.839317614 | 1.872613442 | 0.061121784 |
| RPL3L | ENST00000562995.1 | ENSG00000261253.1 | 0.807017591 | 1.781585983 | 0.074816772 |
| RPL3L | ENST00000563044.1 | ENSG00000260978.1 | 0.900122101 | 2.011966805 | 0.044223436 |
| RPL3L | ENST00000563806.1 | ENSG00000238045.5 | 0.801061051 | 1.797907358 | 0.07219169 |
| RPL3L | ENST00000565823.1 | ENSG00000260686.1 | -0.885617559 | -1.990804169 | 0.046502421 |
| RPL3L | ENST00000567127.1 | ENSG00000260264.1 | -0.895870123 | -1.982192301 | 0.047457722 |
| RPL3L | ENST00000567395.1 | ENSG00000261090.1 | 0.818740842 | 1.860571951 | 0.062804649 |
| RPL3L | ENST00000569981.1 | ENSG00000238045.5 | 0.924770376 | 2.074351862 | 0.038046645 |
| RPL3L | ENST00000570493.2 | ENSG00000261898.2 | 0.928504059 | 2.094184722 | 0.036243528 |
| RPL3L | ENST00000570512.1 | ENSG00000262768.1 | 0.8263391 | 1.865529759 | 0.062107185 |
| RPL3L | ENST00000570974.1 | ENSG00000263300.1 | 0.87832227 | 1.96726533 | 0.04915263 |
| RPL3L | ENST00000577807.1 | ENSG00000263427.1 | 0.804314299 | 1.803335693 | 0.07133551 |
| RPL3L | ENST00000578757.1 | ENSG00000175061.13 | 0.809059895 | 1.789892857 | 0.073471137 |
| RPL3L | ENST00000580311.1 | ENSG00000266803.1 | -0.879122664 | -1.974807545 | 0.048289993 |
| RPL3L | ENST00000585559.1 | ENSG00000267117.1 | 0.863794604 | 1.925284481 | 0.054193778 |
| RPL3L | ENST00000586694.1 | ENSG00000267141.1 | 0.840555475 | 1.880474267 | 0.06004347 |
| RPL3L | ENST00000588380.1 | ENSG00000266990.1 | 0.899417157 | 1.999242176 | 0.045582157 |
| RPL3L | ENST00000590328.1 | ENSG00000256995.2 | -0.813893919 | -1.788613658 | 0.073677057 |
| RPL3L | ENST00000591174.1 | ENSG00000267289.1 | 0.892885733 | 1.985344755 | 0.047106128 |
| RPL3L | ENST00000593642.1 | ENSG00000267858.1 | 0.832374545 | 1.874160379 | 0.060908323 |
| RPL3L | ENST00000599259.1 | ENSG00000269352.1 | 0.929112748 | 2.05052812 | 0.040312923 |
| RPL3L | ENST00000599467.1 | ENSG00000244513.2 | 0.822241893 | 1.827577285 | 0.067613018 |
| RPL3L | ENST00000600716.1 | ENSG00000269487.1 | 0.892474262 | 2.005720167 | 0.044886112 |
| RPL3L | ENST00000600726.1 | ENSG00000267858.1 | 0.841102204 | 1.877022015 | 0.060515077 |
| RPL3L | ENST00000602594.1 | ENSG00000269930.1 | -0.835442924 | -1.882563019 | 0.059759612 |

| | | | | | |
|---|---|---|---|---|---|
| RPL3L | ENST00000602809.1 | ENSG00000270105.1 | -0.859890528 | -1.944552611 | 0.051828848 |
| RPL3L | ENST00000604142.1 | ENSG00000271308.1 | 0.803560022 | 1.79745108 | 0.072264038 |
| RPL3L | ENST00000606377.1 | ENSG00000272286.1 | -0.80092743 | -1.820333439 | 0.068708242 |
| RPL3L | ENST00000606470.1 | ENSG00000271913.1 | 0.910067083 | 2.029455636 | 0.042411903 |
| RPL3L | ENST00000607943.1 | ENSG00000273188.1 | 0.83002689 | 1.856923932 | 0.063321977 |
| RPL3L | ENST00000608367.1 | ENSG00000273361.1 | 0.896234902 | 1.996246147 | 0.045907137 |
| RPL3L | NR_003604.2 | ZFAS1 | 0.810031764 | 1.807431859 | 0.07069497 |
| RPL3L | NR_003605.1 | ZFAS1 | 0.874609645 | 1.948439767 | 0.051362365 |
| RPL3L | NR_026802.1 | FAM74A4 | 0.866141797 | 1.949764678 | 0.051204173 |
| RPL3L | NR_026951.1 | LINC00324 | 0.883313395 | 1.985537848 | 0.047084664 |
| RPL3L | NR_036480.1 | VPS9D1-AS1 | 0.971365607 | 2.187539085 | 0.028703193 |
| RPL3L | NR_047498.1 | LINC00853 | 0.80510984 | 1.813020795 | 0.069828614 |
| RPL3L | NR_072981.1 | LINC00957 | 0.80598709 | 1.791168902 | 0.073266195 |
| RPL3L | NR_105010.1 | LINC01333 | 0.802986522 | 1.803330677 | 0.071336298 |
| RPL3L | NR_109886.1 | RALY-AS1 | 0.826825519 | 1.836327266 | 0.06630927 |
| RPL3L | NR_110998.1 | FAM74A4 | 0.866141797 | 1.936005504 | 0.052867027 |
| RPL3L | NR_120371.1 | LINC01585 | 0.823502715 | 1.814884525 | 0.069541657 |
| RPL3L | NR_121188.1 | PGM5P3-AS1 | -0.906443371 | -2.007023828 | 0.044747126 |
| RPL3L | NR_121189.1 | PGM5P3-AS1 | -0.865576151 | -1.933038278 | 0.053231483 |
| RPL3L | NR_125957.1 | LOC101928626 | -0.945183435 | -2.116569062 | 0.034296438 |
| RPL3L | NR_126522.1 | EXOC3-AS1 | 0.911774247 | 2.040914797 | 0.041259297 |
| RPL3L | NR_134579.1 | LOC105372179 | 0.865436176 | 1.896324618 | 0.057917133 |
| RPL3L | NR_135024.1 | LOC105369747 | 0.90711791 | 2.021407526 | 0.043237596 |
| RPL3L | NR_138038.1 | LINC00677 | 0.959375 | 2.139189453 | 0.032420328 |
| RPL3L | NR_144459.1 | ARSD-AS1 | 0.928755817 | 2.06995835 | 0.038456245 |
| RPS17 | ENST00000412085.1 | ENSG00000233825.1 | 0.909684072 | 2.026574831 | 0.042705913 |
| RPS17 | ENST00000412759.1 | ENSG00000236933.1 | 0.932404647 | 2.105521379 | 0.035245948 |
| RPS17 | ENST00000412772.1 | ENSG00000231507.1 | 0.815731087 | 1.806718661 | 0.070806157 |
| RPS17 | ENST00000414740.2 | ENSG00000229646.2 | 0.830518783 | 1.8829746 | 0.05970381 |
| RPS17 | ENST00000415205.1 | ENSG00000182057.4 | 0.869473275 | 1.971484083 | 0.048668532 |
| RPS17 | ENST00000419662.1 | ENSG00000228265.1 | 0.852892017 | 1.898739164 | 0.05759878 |
| RPS17 | ENST00000426237.2 | ENSG00000235527.2 | 0.815279751 | 1.833086578 | 0.066789697 |
| RPS17 | ENST00000426475.1 | ENSG00000239467.1 | 0.877411494 | 1.966911156 | 0.049193454 |
| RPS17 | ENST00000426519.1 | ENSG00000234142.1 | 0.831125439 | 1.825787873 | 0.067882221 |
| RPS17 | ENST00000428765.1 | ENSG00000230107.1 | 0.893302118 | 2.009957826 | 0.044435653 |
| RPS17 | ENST00000429080.1 | ENSG00000233047.1 | -0.866335737 | -1.928648333 | 0.053774534 |
| RPS17 | ENST00000430920.1 | ENSG00000234203.1 | 0.941928695 | 2.074197073 | 0.038061012 |
| RPS17 | ENST00000433905.2 | ENSG00000229299.2 | 0.958368912 | 2.129765265 | 0.033190998 |
| RPS17 | ENST00000438190.1 | ENSG00000227214.2 | 0.906349955 | 2.014948567 | 0.043910038 |
| RPS17 | ENST00000439184.1 | ENSG00000233985.1 | -0.830274427 | -1.849478953 | 0.064388684 |
| RPS17 | ENST00000441592.2 | ENSG00000224078.8 | 0.85269549 | 1.899597288 | 0.057485988 |
| RPS17 | ENST00000442829.1 | ENSG00000225284.1 | 0.807835135 | 1.794459119 | 0.072739918 |
| RPS17 | ENST00000447343.2 | ENSG00000229299.2 | 0.957031174 | 2.136162439 | 0.032666176 |
| RPS17 | ENST00000448570.1 | ENSG00000224549.1 | 0.819035328 | 1.821043279 | 0.068600278 |
| RPS17 | ENST00000453051.1 | ENSG00000229407.1 | 0.945479754 | 2.104436634 | 0.035340377 |
| RPS17 | ENST00000457253.1 | ENSG00000225173.1 | 0.831595699 | 1.862252943 | 0.062567445 |
| RPS17 | ENST00000458194.1 | ENSG00000226193.1 | 0.862048714 | 1.893507244 | 0.058290446 |
| RPS17 | ENST00000463255.1 | ENSG00000243305.1 | -0.835129937 | -1.848358909 | 0.064550439 |
| RPS17 | ENST00000466431.2 | ENSG00000254485.1 | 0.840969345 | 1.884430756 | 0.059506733 |
| RPS17 | ENST00000468165.1 | ENSG00000239480.1 | 0.955151853 | 2.167252517 | 0.030215605 |
| RPS17 | ENST00000489077.1 | ENSG00000244198.1 | 0.927919097 | 2.045057076 | 0.040849231 |

| | | | | | |
|---|---|---|---|---|---|
| RPS17 | ENST00000490013.1 | ENSG00000184115.12 | 0.858375021 | 1.933826474 | 0.053134467 |
| RPS17 | ENST00000498358.1 | ENSG00000184115.12 | 0.800650324 | 1.799223201 | 0.071983381 |
| RPS17 | ENST00000498693.1 | ENSG00000244198.1 | 0.905997576 | 2.029759772 | 0.042380964 |
| RPS17 | ENST00000503723.1 | ENSG00000250472.1 | -0.933683644 | -2.093860549 | 0.036272405 |
| RPS17 | ENST00000506394.1 | ENSG00000251665.1 | 0.865595702 | 1.947772732 | 0.051442163 |
| RPS17 | ENST00000506791.1 | ENSG00000251131.1 | 0.839782442 | 1.891887459 | 0.058505977 |
| RPS17 | ENST00000509036.1 | ENSG00000251131.1 | 0.901695802 | 1.995296345 | 0.046010568 |
| RPS17 | ENST00000509192.1 | ENSG00000250765.1 | 0.819221176 | 1.822475271 | 0.068382903 |
| RPS17 | ENST00000510570.1 | ENSG00000250438.1 | -0.863201137 | -1.920518821 | 0.054792398 |
| RPS17 | ENST00000517846.1 | ENSG00000254485.1 | 0.930752996 | 2.069234122 | 0.038524122 |
| RPS17 | ENST00000520603.1 | ENSG00000254001.1 | -0.850201469 | -1.899719973 | 0.057469878 |
| RPS17 | ENST00000521653.1 | ENSG00000253301.1 | 0.87101609 | 1.935862141 | 0.052884588 |
| RPS17 | ENST00000522547.1 | ENSG00000253430.1 | -0.800211337 | -1.774565498 | 0.075969658 |
| RPS17 | ENST00000523806.1 | ENSG00000253616.1 | 0.808274193 | 1.826272306 | 0.067809255 |
| RPS17 | ENST00000524942.1 | ENSG00000255553.1 | 0.825614687 | 1.837291815 | 0.066166828 |
| RPS17 | ENST00000527086.1 | ENSG00000255182.1 | 0.840058159 | 1.867752383 | 0.061796593 |
| RPS17 | ENST00000527757.1 | ENSG00000255109.1 | -0.830435997 | -1.865861174 | 0.062060791 |
| RPS17 | ENST00000528000.1 | ENSG00000254804.1 | 0.81993338 | 1.832316556 | 0.066904272 |
| RPS17 | ENST00000529247.1 | ENSG00000254741.1 | 0.866158412 | 1.935635126 | 0.052912405 |
| RPS17 | ENST00000543072.1 | ENSG00000256092.2 | -0.872670166 | -1.946783498 | 0.051560696 |
| RPS17 | ENST00000543275.1 | ENSG00000256944.1 | 0.931936432 | 2.103604296 | 0.035412979 |
| RPS17 | ENST00000545177.3 | ENSG00000230438.5 | 0.870139494 | 1.990500847 | 0.046535791 |
| RPS17 | ENST00000549806.1 | ENSG00000257252.1 | 0.914639002 | 2.052215089 | 0.040148764 |
| RPS17 | ENST00000552525.1 | ENSG00000257286.1 | 0.820137436 | 1.825783208 | 0.067882924 |
| RPS17 | ENST00000558575.1 | ENSG00000259687.1 | 0.912912012 | 2.058992753 | 0.039494933 |
| RPS17 | ENST00000563018.1 | ENSG00000260193.1 | 0.801534166 | 1.790505809 | 0.073372634 |
| RPS17 | ENST00000563611.1 | ENSG00000261583.1 | 0.965284247 | 2.163607843 | 0.030494459 |
| RPS17 | ENST00000563806.1 | ENSG00000238045.5 | 0.84700455 | 1.908281983 | 0.05635478 |
| RPS17 | ENST00000564809.1 | ENSG00000261471.1 | 0.886781034 | 1.999299379 | 0.045575971 |
| RPS17 | ENST00000570843.1 | ENSG00000261889.1 | 0.829232097 | 1.87696936 | 0.060522293 |
| RPS17 | ENST00000574460.1 | ENSG00000263051.1 | 0.849600287 | 1.903304146 | 0.05700087 |
| RPS17 | ENST00000577853.1 | ENSG00000264207.1 | 0.810520739 | 1.800986913 | 0.071704943 |
| RPS17 | ENST00000578800.1 | ENSG00000264235.1 | 0.9717475 | 2.17436903 | 0.029677432 |
| RPS17 | ENST00000578936.1 | ENSG00000265547.1 | 0.870284566 | 1.951929392 | 0.050946588 |
| RPS17 | ENST00000579154.1 | ENSG00000265908.1 | -0.804561617 | -1.822155425 | 0.068431406 |
| RPS17 | ENST00000579775.1 | ENSG00000264108.1 | 0.813549115 | 1.823750913 | 0.068189738 |
| RPS17 | ENST00000580622.1 | ENSG00000264634.1 | 0.86640095 | 1.946093461 | 0.051643514 |
| RPS17 | ENST00000582044.1 | ENSG00000263715.2 | 0.939277117 | 2.123587699 | 0.033704635 |
| RPS17 | ENST00000588182.2 | ENSG00000267453.2 | 0.925303699 | 2.100827938 | 0.035656073 |
| RPS17 | ENST00000588290.1 | ENSG00000267751.1 | 0.817393118 | 1.818373797 | 0.06900702 |
| RPS17 | ENST00000588380.1 | ENSG00000266990.1 | 0.825746533 | 1.816887162 | 0.069234393 |
| RPS17 | ENST00000588402.1 | ENSG00000267006.1 | -0.850690759 | -1.896220759 | 0.057930859 |
| RPS17 | ENST00000589673.1 | ENSG00000267755.1 | 0.842232271 | 1.885597485 | 0.059349217 |
| RPS17 | ENST00000591174.1 | ENSG00000267289.1 | 0.876655921 | 1.944449847 | 0.051841228 |
| RPS17 | ENST00000592413.1 | ENSG00000266933.1 | 0.853309662 | 1.905429062 | 0.05672432 |
| RPS17 | ENST00000592498.1 | ENSG00000267488.1 | 0.808365974 | 1.819838494 | 0.068783604 |
| RPS17 | ENST00000592518.1 | ENSG00000267786.1 | 0.833574183 | 1.85358906 | 0.063797974 |
| RPS17 | ENST00000592525.1 | ENSG00000267214.1 | 0.890173755 | 2.015288766 | 0.043874401 |
| RPS17 | ENST00000593139.1 | ENSG00000267042.1 | 0.935329721 | 2.092055244 | 0.036433574 |
| RPS17 | ENST00000594590.2 | ENSG00000268199.2 | 0.8759142 | 1.980357445 | 0.047663378 |
| RPS17 | ENST00000594850.1 | ENSG00000268093.1 | 0.86881707 | 1.929033423 | 0.053726713 |

| | | | | | |
|---|---|---|---|---|---|
| RPS17 | ENST00000595955.1 | ENSG00000268401.1 | 0.887461283 | 2.007029938 | 0.044746475 |
| RPS17 | ENST00000596887.1 | ENSG00000237031.3 | -0.950862476 | -2.082999181 | 0.037251298 |
| RPS17 | ENST00000597169.1 | ENSG00000269720.1 | 0.828918986 | 1.86065505 | 0.062792905 |
| RPS17 | ENST00000597256.1 | ENSG00000267986.1 | 0.962446156 | 2.172338293 | 0.029830156 |
| RPS17 | ENST00000598092.1 | ENSG00000228065.6 | -0.821248173 | -1.812142451 | 0.069964189 |
| RPS17 | ENST00000599352.1 | ENSG00000240401.4 | -0.889517311 | -1.994240652 | 0.046125761 |
| RPS17 | ENST00000600242.1 | ENSG00000269583.1 | 0.92285248 | 2.074992938 | 0.037987189 |
| RPS17 | ENST00000600726.1 | ENSG00000267858.1 | 0.887692913 | 1.997365134 | 0.045785533 |
| RPS17 | ENST00000601033.1 | ENSG00000268401.1 | 0.916251473 | 2.042551096 | 0.041096896 |
| RPS17 | ENST00000602532.1 | ENSG00000270091.1 | 0.833080541 | 1.844829111 | 0.065062401 |
| RPS17 | ENST00000604142.1 | ENSG00000271308.1 | 0.921551093 | 2.060151379 | 0.039384071 |
| RPS17 | ENST00000604183.1 | ENSG00000271185.1 | 0.90482233 | 2.034131843 | 0.041938304 |
| RPS17 | ENST00000606277.1 | ENSG00000272145.1 | 0.867639798 | 1.952200517 | 0.050914403 |
| RPS17 | ENST00000606909.1 | ENSG00000271821.1 | 0.81588351 | 1.813272633 | 0.069789782 |
| RPS17 | ENST00000607052.1 | ENSG00000271870.1 | -0.855532621 | -1.943004774 | 0.052015582 |
| RPS17 | ENST00000607224.1 | ENSG00000272521.1 | 0.907085689 | 2.022110087 | 0.04316498 |
| RPS17 | ENST00000607476.1 | ENSG00000272540.1 | 0.95875966 | 2.153180186 | 0.03130452 |
| RPS17 | ENST00000607943.1 | ENSG00000273188.1 | 0.922423393 | 2.074499156 | 0.038032977 |
| RPS17 | ENST00000608367.1 | ENSG00000273361.1 | 0.845328234 | 1.911138665 | 0.055986763 |
| RPS17 | ENST00000608677.1 | ENSG00000273350.1 | 0.952134601 | 2.092248192 | 0.03641632 |
| RPS17 | ENST00000608940.1 | ENSG00000272763.1 | 0.828866029 | 1.858868746 | 0.063045745 |
| RPS17 | ENST00000609113.1 | ENSG00000272827.1 | 0.854131721 | 1.898027061 | 0.057692517 |
| RPS17 | ENST00000610270.1 | ENSG00000272576.1 | -0.824998043 | -1.857030216 | 0.063306855 |
| RPS17 | NR_003604.2 | ZFAS1 | 0.852031102 | 1.912921686 | 0.055758079 |
| RPS17 | NR_003606.2 | ZFAS1 | 0.851618497 | 1.900845368 | 0.05732227 |
| RPS17 | NR_026802.1 | FAM74A4 | 0.915754258 | 2.030084722 | 0.042347928 |
| RPS17 | NR_026813.1 | LINC00597 | -0.876678824 | -1.940443895 | 0.052325766 |
| RPS17 | NR_026951.1 | LINC00324 | 0.867037909 | 1.937729315 | 0.052656255 |
| RPS17 | NR_027052.1 | THAP7-AS1 | 0.979079577 | 2.185574545 | 0.028846747 |
| RPS17 | NR_027271.1 | CIRBP-AS1 | 0.902726362 | 2.009089385 | 0.044527655 |
| RPS17 | NR_036658.1 | ZFAS1 | 0.850687963 | 1.888661686 | 0.058937176 |
| RPS17 | NR_038421.1 | LINC01220 | 0.93767491 | 2.089350649 | 0.03667617 |
| RPS17 | NR_038923.1 | SSSCA1-AS1 | 0.97621466 | 2.163067574 | 0.030535983 |
| RPS17 | NR_040096.1 | LOC643339 | 0.880260323 | 1.980082638 | 0.047694244 |
| RPS17 | NR_045114.1 | PVRL3-AS1 | -0.865371558 | -1.923575732 | 0.054407785 |
| RPS17 | NR_046871.1 | LINC00333 | -0.848475864 | -1.910161544 | 0.056112417 |
| RPS17 | NR_047116.1 | HIF1A-AS1 | -0.881231101 | -1.979059348 | 0.047809325 |
| RPS17 | NR_072981.1 | LINC00957 | 0.803868128 | 1.791244766 | 0.073254025 |
| RPS17 | NR_105010.1 | LINC01333 | 0.851610271 | 1.895645655 | 0.058006916 |
| RPS17 | NR_108106.1 | LINC01135 | 0.910009212 | 2.009975398 | 0.044433793 |
| RPS17 | NR_109831.1 | RASSF1-AS1 | 0.893941156 | 1.989504848 | 0.046645507 |
| RPS17 | NR_109886.1 | RALY-AS1 | 0.849993188 | 1.894628827 | 0.058141593 |
| RPS17 | NR_110245.1 | LOC101929282 | -0.84460223 | -1.896525497 | 0.057890592 |
| RPS17 | NR_110556.1 | LOC102724890 | -0.818772273 | -1.848650221 | 0.064508336 |
| RPS17 | NR_110630.1 | LOC101927478 | 0.836338162 | 1.881006314 | 0.05997106 |
| RPS17 | NR_110941.1 | MIR762HG | 0.923571267 | 2.060880815 | 0.039314412 |
| RPS17 | NR_110998.1 | FAM74A4 | 0.915754258 | 2.049183811 | 0.040444144 |
| RPS17 | NR_111951.1 | LINC00869 | 0.947637076 | 2.091548934 | 0.036478885 |
| RPS17 | NR_111952.1 | LINC00869 | 0.959399963 | 2.161566215 | 0.030651628 |
| RPS17 | NR_111953.1 | LINC00869 | 0.945102716 | 2.132716004 | 0.03294803 |
| RPS17 | NR_117097.1 | LINC01353 | 0.815731087 | 1.826238441 | 0.067814353 |

| | | | | | |
|---|---|---|---|---|---|
| RPS17 | NR_125849.1 | LOC101928140 | -0.824546308 | -1.849205609 | 0.064428129 |
| RPS17 | NR_126522.1 | EXOC3-AS1 | 0.9045725 | 1.999256114 | 0.04558065 |
| RPS17 | NR_130143.1 | LOC104968399 | 0.964503742 | 2.173401218 | 0.029750133 |
| RPS17 | NR_134520.1 | LOC727993 | 0.804593038 | 1.791850784 | 0.073156871 |
| RPS17 | NR_135024.1 | LOC105369747 | 0.902709162 | 2.031797669 | 0.042174143 |
| RPS17 | NR_135041.1 | LOC101927038 | 0.862660379 | 1.92886225 | 0.053747965 |
| RPS17 | NR_135584.1 | LOC101927596 | 0.826635882 | 1.85402261 | 0.063735925 |
| RPS17 | NR_136215.1 | VCAN-AS1 | -0.890800999 | -2.024058959 | 0.042964086 |
| RTP4 | ENST00000412348.1 | ENSG00000228959.1 | -0.806482564 | -1.804939465 | 0.071084155 |
| RTP4 | ENST00000416002.1 | ENSG00000230233.1 | 0.821731096 | 1.85383952 | 0.063762123 |
| RTP4 | ENST00000422807.1 | ENSG00000227683.1 | -0.869662319 | -1.966958577 | 0.049187986 |
| RTP4 | ENST00000424342.1 | ENSG00000234988.1 | 0.83977609 | 1.879799189 | 0.060135451 |
| RTP4 | ENST00000428391.1 | ENSG00000224691.1 | 0.918436238 | 2.055468114 | 0.039833812 |
| RTP4 | ENST00000432265.1 | ENSG00000231170.1 | 0.872187346 | 1.938898913 | 0.052513648 |
| RTP4 | ENST00000433550.1 | ENSG00000232227.1 | -0.835290343 | -1.857142715 | 0.063290852 |
| RTP4 | ENST00000438623.1 | ENSG00000224521.1 | -0.804221408 | -1.821592943 | 0.068516773 |
| RTP4 | ENST00000439072.1 | ENSG00000224516.1 | 0.803376848 | 1.802381186 | 0.071485453 |
| RTP4 | ENST00000442435.2 | ENSG00000237781.2 | 0.818705027 | 1.838781681 | 0.065947304 |
| RTP4 | ENST00000443066.2 | ENSG00000237633.2 | -0.842327852 | -1.897478011 | 0.057764878 |
| RTP4 | ENST00000449154.1 | ENSG00000226969.1 | 0.936521347 | 2.08569991 | 0.037005815 |
| RTP4 | ENST00000456499.1 | ENSG00000237640.1 | 0.83775266 | 1.875434802 | 0.060732931 |
| RTP4 | ENST00000457043.1 | ENSG00000231365.1 | -0.803530834 | -1.784217241 | 0.074388372 |
| RTP4 | ENST00000473329.1 | ENSG00000243849.1 | 0.865883144 | 1.931053405 | 0.053476448 |
| RTP4 | ENST00000486285.1 | ENSG00000241818.1 | -0.815938018 | -1.844121092 | 0.065165495 |
| RTP4 | ENST00000503987.1 | ENSG00000250075.1 | -0.81485479 | -1.807954086 | 0.070613647 |
| RTP4 | ENST00000504765.1 | ENSG00000249638.1 | -0.953121609 | -2.135196705 | 0.032744946 |
| RTP4 | ENST00000505978.1 | ENSG00000249982.1 | 0.856824971 | 1.933447342 | 0.053181114 |
| RTP4 | ENST00000508687.1 | ENSG00000250538.1 | -0.803854229 | -1.790830815 | 0.073320449 |
| RTP4 | ENST00000508925.2 | ENSG00000249196.2 | 0.872700682 | 1.953210773 | 0.050794625 |
| RTP4 | ENST00000512882.2 | ENSG00000251575.2 | -0.925999671 | -2.075085504 | 0.037978611 |
| RTP4 | ENST00000521403.1 | ENSG00000253603.1 | 0.821886125 | 1.843061137 | 0.065320085 |
| RTP4 | ENST00000522390.1 | ENSG00000254262.1 | -0.816295784 | -1.830129354 | 0.067230599 |
| RTP4 | ENST00000524808.1 | ENSG00000254812.1 | 0.960056136 | 2.151443525 | 0.031441209 |
| RTP4 | ENST00000537492.1 | ENSG00000256637.2 | -0.850064269 | -1.920103257 | 0.054844858 |
| RTP4 | ENST00000539963.1 | ENSG00000256116.1 | 0.911618699 | 2.053763425 | 0.039998594 |
| RTP4 | ENST00000545357.1 | ENSG00000256862.1 | 0.82522984 | 1.831796154 | 0.066981797 |
| RTP4 | ENST00000549683.1 | ENSG00000257953.1 | 0.809517137 | 1.791690496 | 0.073182558 |
| RTP4 | ENST00000553075.1 | ENSG00000257258.1 | -0.901523675 | -2.015462214 | 0.043856241 |
| RTP4 | ENST00000559041.1 | ENSG00000259713.1 | -0.879930628 | -1.959815267 | 0.050017386 |
| RTP4 | ENST00000561039.1 | ENSG00000259536.1 | 0.960909132 | 2.151441419 | 0.031441375 |
| RTP4 | ENST00000561254.1 | ENSG00000259554.1 | -0.933606019 | -2.099798342 | 0.035746584 |
| RTP4 | ENST00000565441.1 | ENSG00000261013.1 | -0.911007788 | -2.038997475 | 0.04145028 |
| RTP4 | ENST00000565667.1 | ENSG00000261253.1 | 0.910443935 | 2.040062037 | 0.041344147 |
| RTP4 | ENST00000566390.1 | ENSG00000260213.1 | -0.899810518 | -2.003884162 | 0.04508247 |
| RTP4 | ENST00000567261.1 | ENSG00000261320.1 | -0.933865056 | -2.103783619 | 0.035397326 |
| RTP4 | ENST00000570022.1 | ENSG00000261399.1 | -0.943516607 | -2.1220426 | 0.033834161 |
| RTP4 | ENST00000573260.1 | ENSG00000262482.1 | -0.830260981 | -1.850717808 | 0.064210161 |
| RTP4 | ENST00000576632.1 | ENSG00000262172.1 | 0.820352733 | 1.831340164 | 0.067049786 |
| RTP4 | ENST00000578443.1 | ENSG00000265204.1 | 0.845371714 | 1.871188153 | 0.061319006 |
| RTP4 | ENST00000580729.1 | ENSG00000266176.1 | 0.923379697 | 2.071462293 | 0.038315615 |
| RTP4 | ENST00000597357.1 | ENSG00000268309.1 | 0.840844182 | 1.892526016 | 0.058420931 |

| | | | | | |
|---|---|---|---|---|---|
| RTP4 | ENST00000600007.1 | ENSG00000268655.1 | 0.899892359 | 2.003646103 | 0.045107984 |
| RTP4 | ENST00000602881.1 | ENSG00000269965.1 | -0.848520901 | -1.891492198 | 0.058558672 |
| RTP4 | ENST00000606010.1 | ENSG00000272249.1 | -0.869889859 | -1.929388616 | 0.053682636 |
| RTP4 | ENST00000606885.1 | ENSG00000231698.2 | -0.84336146 | -1.873816758 | 0.060955686 |
| RTP4 | ENST00000607135.1 | ENSG00000272112.1 | -0.920423519 | -2.057895839 | 0.039600133 |
| RTP4 | ENST00000607549.1 | ENSG00000272293.1 | -0.821852252 | -1.833082623 | 0.066790285 |
| RTP4 | ENST00000607665.1 | ENSG00000272254.1 | -0.91054818 | -2.029141528 | 0.042443877 |
| RTP4 | ENST00000607991.1 | ENSG00000273076.1 | 0.865067479 | 1.932076624 | 0.053350049 |
| RTP4 | ENST00000608465.1 | ENSG00000272758.1 | -0.920152841 | -2.041330157 | 0.041218022 |
| RTP4 | ENST00000608934.1 | ENSG00000273063.1 | -0.947840099 | -2.126945169 | 0.033424639 |
| RTP4 | NR_024491.1 | LOC100128573 | -0.863419496 | -1.961297902 | 0.049844282 |
| RTP4 | NR_027067.1 | LINC00114 | 0.821340575 | 1.846203793 | 0.064862621 |
| RTP4 | NR_027425.1 | FAM66D | 0.837094863 | 1.892355344 | 0.058443652 |
| RTP4 | NR_073552.1 | LOC101059948 | 0.899892359 | 2.0082899 | 0.044612495 |
| RTP4 | NR_102738.1 | LINC00911 | 0.845709404 | 1.884595825 | 0.059484426 |
| RTP4 | NR_110702.1 | SEMA3B-AS1 | 0.866668655 | 1.932233993 | 0.053330631 |
| RTP4 | NR_110731.1 | LINC01232 | 0.880812128 | 1.98916427 | 0.046683074 |
| RTP4 | NR_121577.1 | NALT1 | 0.850052601 | 1.901778501 | 0.057200118 |
| RTP4 | NR_125759.1 | PKNOX2-AS1 | 0.825947484 | 1.832438875 | 0.066886061 |
| RTP4 | NR_126409.1 | LINC00376 | -0.831411726 | -1.866419809 | 0.061982654 |
| RTP4 | NR_136569.1 | LINC01660 | -0.889223864 | -1.977208975 | 0.048018014 |
| S1PR4 | ENST00000318291.4 | ENSG00000177406.4 | 0.953262796 | 2.142514703 | 0.032152087 |
| S1PR4 | ENST00000399186.2 | ENSG00000214888.2 | 0.837231458 | 1.866240157 | 0.062007773 |
| S1PR4 | ENST00000412759.1 | ENSG00000236933.1 | 0.892639001 | 2.039321285 | 0.041417973 |
| S1PR4 | ENST00000416329.1 | ENSG00000233184.2 | 0.81773747 | 1.817369379 | 0.069160573 |
| S1PR4 | ENST00000419662.1 | ENSG00000228265.1 | 0.869362894 | 1.930013208 | 0.053605201 |
| S1PR4 | ENST00000421020.1 | ENSG00000231407.1 | 0.911836836 | 2.054597744 | 0.039917874 |
| S1PR4 | ENST00000421207.1 | ENSG00000231768.1 | 0.946364587 | 2.097264881 | 0.035970134 |
| S1PR4 | ENST00000423869.1 | ENSG00000227848.1 | 0.881384559 | 1.962749226 | 0.04967532 |
| S1PR4 | ENST00000425124.1 | ENSG00000232336.1 | 0.883174527 | 2.001861719 | 0.045299606 |
| S1PR4 | ENST00000425624.1 | ENSG00000223779.4 | 0.904747621 | 2.003647647 | 0.045107818 |
| S1PR4 | ENST00000426237.2 | ENSG00000235527.2 | 0.883764546 | 1.951205513 | 0.051032603 |
| S1PR4 | ENST00000426519.1 | ENSG00000234142.1 | 0.932100458 | 2.089273868 | 0.036683077 |
| S1PR4 | ENST00000426699.1 | ENSG00000229308.1 | 0.899258855 | 2.025188543 | 0.042848008 |
| S1PR4 | ENST00000433344.1 | ENSG00000234083.1 | -0.893390881 | -2.034559715 | 0.041895194 |
| S1PR4 | ENST00000433614.1 | ENSG00000228534.1 | -0.848969933 | -1.894695776 | 0.058132718 |
| S1PR4 | ENST00000434627.1 | ENSG00000230074.1 | 0.809938007 | 1.833567867 | 0.066718166 |
| S1PR4 | ENST00000435434.1 | ENSG00000231233.1 | 0.967899211 | 2.171925843 | 0.029861257 |
| S1PR4 | ENST00000435892.1 | ENSG00000233635.2 | 0.884226553 | 1.962995192 | 0.049646733 |
| S1PR4 | ENST00000435992.2 | ENSG00000232675.3 | 0.934396742 | 2.066351295 | 0.038795321 |
| S1PR4 | ENST00000436982.2 | ENSG00000235335.2 | -0.808381825 | -1.773904484 | 0.076078951 |
| S1PR4 | ENST00000438107.1 | ENSG00000234449.2 | 0.897815187 | 1.993493162 | 0.046207472 |
| S1PR4 | ENST00000438190.1 | ENSG00000227214.2 | 0.95359463 | 2.140066471 | 0.032349395 |
| S1PR4 | ENST00000438222.1 | ENSG00000238034.1 | 0.833700905 | 1.862777345 | 0.062493599 |
| S1PR4 | ENST00000439186.1 | ENSG00000237076.1 | 0.837922906 | 1.850622696 | 0.064223852 |
| S1PR4 | ENST00000442069.1 | ENSG00000225655.1 | -0.875336 | -1.940853493 | 0.05227605 |
| S1PR4 | ENST00000447206.1 | ENSG00000230839.1 | 0.869142864 | 1.941646278 | 0.052179936 |
| S1PR4 | ENST00000447514.1 | ENSG00000236753.1 | 0.8002361 | 1.80018297 | 0.071831752 |
| S1PR4 | ENST00000452176.1 | ENSG00000223659.1 | -0.871633931 | -1.955856446 | 0.050482067 |
| S1PR4 | ENST00000457115.1 | ENSG00000227245.1 | 0.849720135 | 1.896348027 | 0.057914039 |
| S1PR4 | ENST00000458154.1 | ENSG00000235578.1 | 0.972230372 | 2.142595995 | 0.032145553 |

| | | | | | |
|---|---|---|---|---|---|
| S1PR4 | ENST00000458194.1 | ENSG00000226193.1 | 0.889889108 | 2.007436691 | 0.044703185 |
| S1PR4 | ENST00000458364.1 | ENSG00000225655.1 | -0.94366614 | -2.115153568 | 0.034416861 |
| S1PR4 | ENST00000459985.1 | ENSG00000273066.1 | 0.912543364 | 2.023104628 | 0.043062362 |
| S1PR4 | ENST00000484413.1 | ENSG00000271853.1 | 0.937408882 | 2.085635372 | 0.037011665 |
| S1PR4 | ENST00000489077.1 | ENSG00000244198.1 | 0.883305572 | 1.971294881 | 0.048690157 |
| S1PR4 | ENST00000489557.2 | ENSG00000257045.1 | 0.812057328 | 1.800245881 | 0.071821822 |
| S1PR4 | ENST00000493123.1 | ENSG00000242428.1 | 0.861418331 | 1.928386419 | 0.05380708 |
| S1PR4 | ENST00000494509.1 | ENSG00000240095.1 | 0.855536892 | 1.924950621 | 0.054235536 |
| S1PR4 | ENST00000498693.1 | ENSG00000244198.1 | 0.953383704 | 2.138894948 | 0.032444177 |
| S1PR4 | ENST00000504891.1 | ENSG00000249388.1 | 0.804455351 | 1.792134741 | 0.073111385 |
| S1PR4 | ENST00000505498.1 | ENSG00000250908.1 | 0.881599013 | 2.002230502 | 0.045259947 |
| S1PR4 | ENST00000505556.1 | ENSG00000249409.1 | 0.94887018 | 2.113305696 | 0.034574612 |
| S1PR4 | ENST00000506100.1 | ENSG00000249409.1 | 0.943861857 | 2.098596186 | 0.035852513 |
| S1PR4 | ENST00000506791.1 | ENSG00000251131.1 | 0.948180709 | 2.125669425 | 0.033530794 |
| S1PR4 | ENST00000508083.1 | ENSG00000249343.1 | 0.98170524 | 2.168712047 | 0.030104552 |
| S1PR4 | ENST00000509036.1 | ENSG00000251131.1 | 0.925731403 | 2.066181185 | 0.038811375 |
| S1PR4 | ENST00000509192.1 | ENSG00000250765.1 | 0.940709165 | 2.119332846 | 0.034062348 |
| S1PR4 | ENST00000515128.1 | ENSG00000248215.1 | -0.835588191 | -1.877935639 | 0.06038997 |
| S1PR4 | ENST00000517300.1 | ENSG00000254144.2 | 0.822683069 | 1.852047575 | 0.064018992 |
| S1PR4 | ENST00000520603.1 | ENSG00000254001.1 | -0.907547609 | -2.027706954 | 0.042590166 |
| S1PR4 | ENST00000521307.1 | ENSG00000253177.1 | 0.873901918 | 1.964044398 | 0.049524943 |
| S1PR4 | ENST00000522547.1 | ENSG00000253430.1 | -0.844773526 | -1.870072436 | 0.06147376 |
| S1PR4 | ENST00000522600.1 | ENSG00000246582.2 | 0.819131731 | 1.816927813 | 0.069228167 |
| S1PR4 | ENST00000524818.1 | ENSG00000254473.1 | 0.852206799 | 1.907394405 | 0.056469533 |
| S1PR4 | ENST00000526186.1 | ENSG00000254510.1 | 0.885168615 | 1.984117268 | 0.047242768 |
| S1PR4 | ENST00000526611.1 | ENSG00000246982.2 | 0.848566833 | 1.895653116 | 0.058005929 |
| S1PR4 | ENST00000526935.1 | ENSG00000255372.1 | 0.847814571 | 1.894144203 | 0.058205872 |
| S1PR4 | ENST00000528887.1 | ENSG00000254501.1 | 0.889839656 | 1.994823286 | 0.046062157 |
| S1PR4 | ENST00000536141.1 | ENSG00000256969.1 | 0.835977953 | 1.889807356 | 0.058783729 |
| S1PR4 | ENST00000537269.1 | ENSG00000257084.1 | 0.832950277 | 1.837904747 | 0.066076443 |
| S1PR4 | ENST00000543072.1 | ENSG00000256092.2 | -0.873490935 | -1.961687361 | 0.049798894 |
| S1PR4 | ENST00000543275.1 | ENSG00000256944.1 | 0.800661815 | 1.769560331 | 0.076800412 |
| S1PR4 | ENST00000545177.3 | ENSG00000230438.5 | 0.816664819 | 1.806954804 | 0.070769326 |
| S1PR4 | ENST00000547834.1 | ENSG00000258325.1 | 0.851079376 | 1.932051075 | 0.053353202 |
| S1PR4 | ENST00000548722.2 | ENSG00000257194.2 | -0.874452527 | -1.9238047 | 0.054379068 |
| S1PR4 | ENST00000549878.1 | ENSG00000257284.1 | 0.845335623 | 1.883372606 | 0.05964989 |
| S1PR4 | ENST00000552469.1 | ENSG00000258325.1 | 0.815624852 | 1.847734601 | 0.064640746 |
| S1PR4 | ENST00000556786.1 | ENSG00000258525.1 | -0.812189835 | -1.816800144 | 0.069247721 |
| S1PR4 | ENST00000565823.1 | ENSG00000260686.1 | -0.888929531 | -1.969028066 | 0.048949868 |
| S1PR4 | ENST00000565829.1 | ENSG00000260148.1 | 0.880483084 | 1.939151414 | 0.052482904 |
| S1PR4 | ENST00000566170.1 | ENSG00000261071.1 | 0.862315447 | 1.916147537 | 0.055346319 |
| S1PR4 | ENST00000567395.1 | ENSG00000261090.1 | 0.890628152 | 2.000165636 | 0.045482381 |
| S1PR4 | ENST00000568033.1 | ENSG00000261480.1 | 0.878783268 | 1.97760113 | 0.047973722 |
| S1PR4 | ENST00000569981.1 | ENSG00000238045.5 | 0.932225412 | 2.114479385 | 0.034474344 |
| S1PR4 | ENST00000570493.2 | ENSG00000261898.2 | 0.904975197 | 2.008284167 | 0.044613103 |
| S1PR4 | ENST00000570512.1 | ENSG00000262768.1 | 0.899063653 | 2.02657298 | 0.042706102 |
| S1PR4 | ENST00000570843.1 | ENSG00000261889.1 | 0.92933278 | 2.087716578 | 0.036823408 |
| S1PR4 | ENST00000570929.1 | ENSG00000262223.2 | 0.951323332 | 2.117096374 | 0.034251669 |
| S1PR4 | ENST00000575139.1 | ENSG00000263072.1 | 0.872720246 | 1.933354368 | 0.053192559 |
| S1PR4 | ENST00000577064.1 | ENSG00000262823.1 | 0.812847263 | 1.807547926 | 0.070676889 |
| S1PR4 | ENST00000577698.1 | ENSG00000265100.1 | 0.825240932 | 1.855458453 | 0.063530787 |

| | | | | | |
|---|---|---|---|---|---|
| S1PR4 | ENST00000578265.1 | ENSG00000214719.7 | 0.863927733 | 1.937642578 | 0.052666844 |
| S1PR4 | ENST00000578757.1 | ENSG00000175061.13 | 0.841160921 | 1.918678559 | 0.055025027 |
| S1PR4 | ENST00000581940.1 | ENSG00000265484.1 | 0.831133676 | 1.869611425 | 0.061537798 |
| S1PR4 | ENST00000582558.1 | ENSG00000264569.1 | 0.867859701 | 1.945853078 | 0.051672391 |
| S1PR4 | ENST00000584705.1 | ENSG00000264569.1 | 0.808133153 | 1.783983118 | 0.074426408 |
| S1PR4 | ENST00000585559.1 | ENSG00000267117.1 | 0.934893664 | 2.075672444 | 0.037924256 |
| S1PR4 | ENST00000586051.1 | ENSG00000267576.1 | 0.815072076 | 1.84555108 | 0.064957415 |
| S1PR4 | ENST00000586694.1 | ENSG00000267141.1 | 0.822325698 | 1.828100517 | 0.067534467 |
| S1PR4 | ENST00000588380.1 | ENSG00000266990.1 | 0.800651606 | 1.788110906 | 0.073758116 |
| S1PR4 | ENST00000588799.1 | ENSG00000267275.1 | 0.808269936 | 1.804024022 | 0.071227541 |
| S1PR4 | ENST00000591174.1 | ENSG00000267289.1 | 0.896254906 | 2.005177563 | 0.044944068 |
| S1PR4 | ENST00000592400.1 | ENSG00000267735.1 | 0.925069563 | 2.032187064 | 0.042134722 |
| S1PR4 | ENST00000593632.1 | ENSG00000180279.5 | 0.851316188 | 1.902543271 | 0.057100168 |
| S1PR4 | ENST00000594590.2 | ENSG00000268199.2 | 0.887292728 | 1.961930797 | 0.049770542 |
| S1PR4 | ENST00000597169.1 | ENSG00000269720.1 | 0.959174542 | 2.170883306 | 0.029939995 |
| S1PR4 | ENST00000597309.1 | ENSG00000232098.2 | -0.856410925 | -1.921866636 | 0.054622541 |
| S1PR4 | ENST00000599259.1 | ENSG00000269352.1 | 0.945111448 | 2.109980749 | 0.034860014 |
| S1PR4 | ENST00000600234.1 | ENSG00000268078.1 | 0.837817209 | 1.889204006 | 0.058864498 |
| S1PR4 | ENST00000600534.1 | ENSG00000267858.1 | 0.896923765 | 2.005392457 | 0.044921107 |
| S1PR4 | ENST00000600726.1 | ENSG00000267858.1 | 0.879679776 | 1.9473672 | 0.051490727 |
| S1PR4 | ENST00000601692.1 | ENSG00000267874.1 | -0.88081229 | -1.958282111 | 0.050196919 |
| S1PR4 | ENST00000601735.1 | ENSG00000244513.2 | 0.935433251 | 2.093012755 | 0.036348016 |
| S1PR4 | ENST00000602532.1 | ENSG00000270091.1 | 0.814330153 | 1.825812985 | 0.067878437 |
| S1PR4 | ENST00000602594.1 | ENSG00000269930.1 | -0.840624547 | -1.881793839 | 0.059864013 |
| S1PR4 | ENST00000602809.1 | ENSG00000270105.1 | -0.869460352 | -1.96806415 | 0.049060657 |
| S1PR4 | ENST00000602872.1 | ENSG00000270067.1 | 0.819707962 | 1.841375819 | 0.065566504 |
| S1PR4 | ENST00000604142.1 | ENSG00000271308.1 | 0.918841848 | 2.075762894 | 0.037915886 |
| S1PR4 | ENST00000606277.1 | ENSG00000272145.1 | 0.891876606 | 2.010550364 | 0.044372971 |
| S1PR4 | ENST00000606377.1 | ENSG00000272286.1 | -0.915149905 | -2.056554129 | 0.039729134 |
| S1PR4 | ENST00000606470.1 | ENSG00000271913.1 | 0.919951068 | 2.07180196 | 0.038283914 |
| S1PR4 | ENST00000607224.1 | ENSG00000272521.1 | 0.800145696 | 1.795714784 | 0.072539889 |
| S1PR4 | ENST00000607476.1 | ENSG00000272540.1 | 0.885509588 | 1.968902941 | 0.048964238 |
| S1PR4 | ENST00000607943.1 | ENSG00000273188.1 | 0.869593327 | 1.956133455 | 0.050449435 |
| S1PR4 | ENST00000609067.1 | ENSG00000272849.1 | 0.809517214 | 1.814569838 | 0.069590041 |
| S1PR4 | ENST00000609972.1 | ENSG00000230651.3 | 0.907187482 | 2.027201378 | 0.042641822 |
| S1PR4 | ENST00000610145.1 | ENSG00000273175.1 | 0.925902399 | 2.052680687 | 0.040103556 |
| S1PR4 | NR_003604.2 | ZFAS1 | 0.948467295 | 2.105127235 | 0.035280234 |
| S1PR4 | NR_003605.1 | ZFAS1 | 0.985374694 | 2.215170224 | 0.026748383 |
| S1PR4 | NR_003606.2 | ZFAS1 | 0.949483563 | 2.117199615 | 0.03424291 |
| S1PR4 | NR_026802.1 | FAM74A4 | 0.888243021 | 1.979288466 | 0.047783537 |
| S1PR4 | NR_027271.1 | CIRBP-AS1 | 0.941871865 | 2.113516383 | 0.034556594 |
| S1PR4 | NR_027334.2 | MZF1-AS1 | 0.914837233 | 2.048858729 | 0.040475931 |
| S1PR4 | NR_036480.1 | VPS9D1-AS1 | 0.894145099 | 1.993639075 | 0.046191512 |
| S1PR4 | NR_036658.1 | ZFAS1 | 0.954536036 | 2.104848725 | 0.035304478 |
| S1PR4 | NR_037169.1 | LOC100507547 | 0.840198356 | 1.860306721 | 0.062842143 |
| S1PR4 | NR_037170.1 | LOC100507547 | 0.80236169 | 1.790519573 | 0.073370424 |
| S1PR4 | NR_044996.1 | HCG23 | 0.926709914 | 2.065514457 | 0.038874349 |
| S1PR4 | NR_045114.1 | PVRL3-AS1 | -0.849790538 | -1.898960393 | 0.057569684 |
| S1PR4 | NR_046742.2 | ZNF630-AS1 | -0.832173245 | -1.867892567 | 0.061777047 |
| S1PR4 | NR_072981.1 | LINC00957 | 0.910960676 | 2.040724213 | 0.041278247 |
| S1PR4 | NR_072982.1 | LINC00957 | 0.907347407 | 2.034573725 | 0.041893783 |

| | | | | | |
|---|---|---|---|---|---|
| S1PR4 | NR_103790.1 | LINC00581 | -0.860767247 | -1.911085303 | 0.05599362 |
| S1PR4 | NR_105010.1 | LINC01333 | 0.978440426 | 2.22806489 | 0.025876188 |
| S1PR4 | NR_108036.1 | CFAP58-AS1 | 0.962285976 | 2.149572036 | 0.031589082 |
| S1PR4 | NR_109886.1 | RALY-AS1 | 0.896735624 | 2.012647865 | 0.044151688 |
| S1PR4 | NR_110998.1 | FAM74A4 | 0.888243021 | 1.970131508 | 0.048823301 |
| S1PR4 | NR_120335.1 | LOC101928414 | 0.862271972 | 1.943626501 | 0.051940508 |
| S1PR4 | NR_121188.1 | PGM5P3-AS1 | -0.873897552 | -1.960770953 | 0.049905748 |
| S1PR4 | NR_121189.1 | PGM5P3-AS1 | -0.949483654 | -2.129154501 | 0.03324148 |
| S1PR4 | NR_125957.1 | LOC101928626 | -0.871633931 | -1.938534876 | 0.052558 |
| S1PR4 | NR_126166.1 | FAM74A7 | 0.85899033 | 1.942637085 | 0.052060023 |
| S1PR4 | NR_126522.1 | EXOC3-AS1 | 0.846855098 | 1.900817111 | 0.057325972 |
| S1PR4 | NR_135032.1 | LOC105369635 | 0.832950277 | 1.900654448 | 0.057347289 |
| S1PR4 | NR_138084.1 | HCG24 | 0.896523921 | 2.01766029 | 0.043626653 |
| S1PR4 | NR_144459.1 | ARSD-AS1 | 0.850811773 | 1.881414386 | 0.059915571 |
| SIGLEC14 | ENST00000318291.4 | ENSG00000177406.4 | 0.877394285 | 1.969470485 | 0.048899089 |
| SIGLEC14 | ENST00000411694.1 | ENSG00000225331.1 | 0.81146586 | 1.790432489 | 0.073384411 |
| SIGLEC14 | ENST00000412348.1 | ENSG00000228959.1 | -0.862811056 | -1.930042636 | 0.053601555 |
| SIGLEC14 | ENST00000412759.1 | ENSG00000236933.1 | 0.815344489 | 1.797650868 | 0.072232352 |
| SIGLEC14 | ENST00000415205.1 | ENSG00000182057.4 | 0.917969372 | 2.030149111 | 0.042341385 |
| SIGLEC14 | ENST00000417260.1 | ENSG00000231734.4 | -0.949260603 | -2.107858688 | 0.035043215 |
| SIGLEC14 | ENST00000418387.1 | ENSG00000235056.1 | -0.89012889 | -1.988820796 | 0.046720986 |
| SIGLEC14 | ENST00000419662.1 | ENSG00000228265.1 | 0.82921897 | 1.859694131 | 0.062928813 |
| SIGLEC14 | ENST00000423428.1 | ENSG00000224048.1 | -0.888529803 | -1.995478004 | 0.045990771 |
| SIGLEC14 | ENST00000426030.2 | ENSG00000228686.2 | -0.856350991 | -1.929993992 | 0.053607582 |
| SIGLEC14 | ENST00000426302.1 | ENSG00000230454.1 | 0.896899837 | 2.009931335 | 0.044438457 |
| SIGLEC14 | ENST00000429080.1 | ENSG00000233047.1 | -0.828064205 | -1.844256015 | 0.065145838 |
| SIGLEC14 | ENST00000430920.1 | ENSG00000234203.1 | 0.919642158 | 2.058220767 | 0.039568946 |
| SIGLEC14 | ENST00000431730.1 | ENSG00000237401.2 | 0.878436929 | 1.972163913 | 0.048590898 |
| SIGLEC14 | ENST00000433905.2 | ENSG00000229299.2 | 0.815214677 | 1.831970589 | 0.066955803 |
| SIGLEC14 | ENST00000435287.1 | ENSG00000227220.1 | 0.902848287 | 2.002046896 | 0.045279688 |
| SIGLEC14 | ENST00000436515.1 | ENSG00000224521.1 | -0.906923719 | -2.050852537 | 0.040281309 |
| SIGLEC14 | ENST00000438190.1 | ENSG00000227214.2 | 0.894433457 | 1.987527551 | 0.046863966 |
| SIGLEC14 | ENST00000438623.1 | ENSG00000224521.1 | -0.862096346 | -1.916207291 | 0.055338716 |
| SIGLEC14 | ENST00000441592.2 | ENSG00000224078.8 | 0.803095986 | 1.786414955 | 0.074032096 |
| SIGLEC14 | ENST00000442829.1 | ENSG00000225284.1 | 0.930326973 | 2.067228698 | 0.038712609 |
| SIGLEC14 | ENST00000446562.1 | ENSG00000233896.1 | 0.829357491 | 1.867312114 | 0.061858015 |
| SIGLEC14 | ENST00000446816.1 | ENSG00000204685.5 | 0.821784387 | 1.849230743 | 0.064424501 |
| SIGLEC14 | ENST00000448858.1 | ENSG00000237734.1 | -0.920002405 | -2.043362684 | 0.041016549 |
| SIGLEC14 | ENST00000451034.1 | ENSG00000229805.1 | -0.859181578 | -1.913638044 | 0.05566642 |
| SIGLEC14 | ENST00000451090.1 | ENSG00000235215.2 | -0.821297927 | -1.853033594 | 0.063877544 |
| SIGLEC14 | ENST00000451507.1 | ENSG00000229539.1 | 0.865105616 | 1.924292417 | 0.054317941 |
| SIGLEC14 | ENST00000452176.1 | ENSG00000223659.1 | -0.916998873 | -2.044825833 | 0.040872032 |
| SIGLEC14 | ENST00000455373.1 | ENSG00000226097.1 | -0.855453159 | -1.878506292 | 0.060311937 |
| SIGLEC14 | ENST00000457371.1 | ENSG00000237401.2 | 0.826662365 | 1.837312072 | 0.066163839 |
| SIGLEC14 | ENST00000458154.1 | ENSG00000235578.1 | 0.855698406 | 1.917410631 | 0.055185785 |
| SIGLEC14 | ENST00000463255.1 | ENSG00000243305.1 | -0.842650482 | -1.910147287 | 0.056114252 |
| SIGLEC14 | ENST00000468165.1 | ENSG00000239480.1 | 0.860730599 | 1.930221474 | 0.053579402 |
| SIGLEC14 | ENST00000488310.1 | ENSG00000240449.1 | 0.808569723 | 1.820958489 | 0.068613167 |
| SIGLEC14 | ENST00000489077.1 | ENSG00000244198.1 | 0.923688305 | 2.073893044 | 0.038089245 |
| SIGLEC14 | ENST00000489090.1 | ENSG00000240045.1 | -0.825730878 | -1.853691738 | 0.063783274 |
| SIGLEC14 | ENST00000489690.1 | ENSG00000243944.1 | -0.908455754 | -2.036235642 | 0.041726699 |

| | | | | | |
|---|---|---|---|---|---|
| SIGLEC14 | ENST00000498693.1 | ENSG00000244198.1 | 0.871706333 | 1.93335347 | 0.053192669 |
| SIGLEC14 | ENST00000503505.1 | ENSG00000248629.1 | -0.867086233 | -1.907263125 | 0.056486523 |
| SIGLEC14 | ENST00000503723.1 | ENSG00000250472.1 | -0.949625086 | -2.13902604 | 0.032433559 |
| SIGLEC14 | ENST00000504578.1 | ENSG00000251513.1 | -0.870101082 | -1.938409411 | 0.052573293 |
| SIGLEC14 | ENST00000506723.2 | ENSG00000249484.4 | -0.849411575 | -1.926036797 | 0.054099779 |
| SIGLEC14 | ENST00000506791.1 | ENSG00000251131.1 | 0.876684006 | 1.983477341 | 0.047314136 |
| SIGLEC14 | ENST00000509036.1 | ENSG00000251131.1 | 0.85897812 | 1.913863927 | 0.055637545 |
| SIGLEC14 | ENST00000509192.1 | ENSG00000250765.1 | 0.908263327 | 2.045421987 | 0.040813273 |
| SIGLEC14 | ENST00000509453.1 | ENSG00000249145.1 | 0.864996242 | 1.936986147 | 0.052747037 |
| SIGLEC14 | ENST00000515128.1 | ENSG00000248215.1 | -0.955564086 | -2.133151867 | 0.03291227 |
| SIGLEC14 | ENST00000517846.1 | ENSG00000254485.1 | 0.82327433 | 1.839844196 | 0.065791115 |
| SIGLEC14 | ENST00000518837.1 | ENSG00000253947.1 | -0.848538365 | -1.886698564 | 0.059200881 |
| SIGLEC14 | ENST00000520603.1 | ENSG00000254001.1 | -0.89067655 | -2.020099524 | 0.043373065 |
| SIGLEC14 | ENST00000521653.1 | ENSG00000253301.1 | 0.905739913 | 2.017515032 | 0.043641794 |
| SIGLEC14 | ENST00000522281.1 | ENSG00000253376.1 | -0.803881146 | -1.796914339 | 0.07234922 |
| SIGLEC14 | ENST00000522390.1 | ENSG00000254262.1 | -0.807289477 | -1.802599474 | 0.07145114 |
| SIGLEC14 | ENST00000522600.1 | ENSG00000246582.2 | 0.806767201 | 1.79697065 | 0.072340279 |
| SIGLEC14 | ENST00000527086.1 | ENSG00000255182.1 | 0.822522859 | 1.861081751 | 0.062732633 |
| SIGLEC14 | ENST00000529247.1 | ENSG00000254741.1 | 0.885405535 | 1.983037389 | 0.047363253 |
| SIGLEC14 | ENST00000534178.1 | ENSG00000255120.1 | 0.894328497 | 2.006599148 | 0.044792362 |
| SIGLEC14 | ENST00000537032.1 | ENSG00000255933.1 | 0.854641375 | 1.918663094 | 0.055026985 |
| SIGLEC14 | ENST00000537850.1 | ENSG00000251002.3 | 0.910824557 | 2.043062184 | 0.041046283 |
| SIGLEC14 | ENST00000543275.1 | ENSG00000256944.1 | 0.875720479 | 1.951172058 | 0.051036582 |
| SIGLEC14 | ENST00000543403.1 | ENSG00000256684.1 | -0.8889304 | -1.975390811 | 0.048223815 |
| SIGLEC14 | ENST00000543494.1 | ENSG00000256514.1 | 0.857302639 | 1.920672599 | 0.054772996 |
| SIGLEC14 | ENST00000548722.2 | ENSG00000257194.2 | -0.806198945 | -1.789486199 | 0.073536548 |
| SIGLEC14 | ENST00000549683.1 | ENSG00000257953.1 | 0.838336362 | 1.874212675 | 0.060901118 |
| SIGLEC14 | ENST00000549806.1 | ENSG00000257252.1 | 0.882956401 | 1.970295344 | 0.048804532 |
| SIGLEC14 | ENST00000550263.1 | ENSG00000257605.1 | 0.903733817 | 2.024143896 | 0.042955348 |
| SIGLEC14 | ENST00000550279.1 | ENSG00000258338.1 | -0.856558117 | -1.913228913 | 0.055718754 |
| SIGLEC14 | ENST00000554431.1 | ENSG00000258616.1 | -0.899759807 | -1.98505745 | 0.04713808 |
| SIGLEC14 | ENST00000557602.1 | ENSG00000258616.1 | -0.902297243 | -2.025495605 | 0.042816499 |
| SIGLEC14 | ENST00000558575.1 | ENSG00000259687.1 | 0.829096249 | 1.856756985 | 0.063345736 |
| SIGLEC14 | ENST00000560963.1 | ENSG00000259370.1 | 0.834729618 | 1.868161009 | 0.061739632 |
| SIGLEC14 | ENST00000562191.1 | ENSG00000261292.1 | -0.891981948 | -2.010773039 | 0.044349435 |
| SIGLEC14 | ENST00000562995.1 | ENSG00000261253.1 | 0.885317973 | 1.979883594 | 0.04771661 |
| SIGLEC14 | ENST00000563018.1 | ENSG00000260193.1 | 0.818352507 | 1.840263433 | 0.065729571 |
| SIGLEC14 | ENST00000563610.1 | ENSG00000260051.1 | 0.943742391 | 2.132496804 | 0.032966027 |
| SIGLEC14 | ENST00000563611.1 | ENSG00000261583.1 | 0.935903826 | 2.094486524 | 0.036216662 |
| SIGLEC14 | ENST00000564809.1 | ENSG00000261471.1 | 0.919708859 | 2.074976448 | 0.037988718 |
| SIGLEC14 | ENST00000565735.1 | ENSG00000261213.1 | -0.836046032 | -1.841869849 | 0.06549419 |
| SIGLEC14 | ENST00000565823.1 | ENSG00000260686.1 | -0.813469192 | -1.827809838 | 0.067578096 |
| SIGLEC14 | ENST00000569981.1 | ENSG00000238045.5 | 0.844919863 | 1.871260079 | 0.061309041 |
| SIGLEC14 | ENST00000570493.2 | ENSG00000261898.2 | 0.911571334 | 2.03620586 | 0.041729688 |
| SIGLEC14 | ENST00000570974.1 | ENSG00000263300.1 | 0.840317521 | 1.87676422 | 0.060550416 |
| SIGLEC14 | ENST00000577853.1 | ENSG00000264207.1 | 0.939351954 | 2.11448806 | 0.034473604 |
| SIGLEC14 | ENST00000578800.1 | ENSG00000264235.1 | 0.807167082 | 1.809770504 | 0.070331384 |
| SIGLEC14 | ENST00000580311.1 | ENSG00000266803.1 | -0.835685736 | -1.892946474 | 0.058364988 |
| SIGLEC14 | ENST00000580622.1 | ENSG00000264634.1 | 0.807520864 | 1.797334108 | 0.072282595 |
| SIGLEC14 | ENST00000582044.1 | ENSG00000263715.2 | 0.903037233 | 2.034053725 | 0.041946179 |
| SIGLEC14 | ENST00000585559.1 | ENSG00000267117.1 | 0.861090902 | 1.924943006 | 0.054236489 |

| | | | | | |
|---|---|---|---|---|---|
| SIGLEC14 | ENST00000585810.1 | ENSG00000236172.2 | 0.87300092 | 1.935560051 | 0.052921607 |
| SIGLEC14 | ENST00000588380.1 | ENSG00000266990.1 | 0.909236496 | 2.021592856 | 0.043218431 |
| SIGLEC14 | ENST00000588402.1 | ENSG00000267006.1 | -0.91085289 | -2.046777571 | 0.040679929 |
| SIGLEC14 | ENST00000589395.1 | ENSG00000267143.1 | 0.806153596 | 1.823012318 | 0.068301525 |
| SIGLEC14 | ENST00000591174.1 | ENSG00000267289.1 | 0.927328449 | 2.084854236 | 0.037082534 |
| SIGLEC14 | ENST00000592525.1 | ENSG00000267214.1 | 0.848710174 | 1.892400109 | 0.058437692 |
| SIGLEC14 | ENST00000592816.1 | ENSG00000236172.2 | 0.889923286 | 1.98024847 | 0.047675616 |
| SIGLEC14 | ENST00000593139.1 | ENSG00000267042.1 | 0.80747578 | 1.79536906 | 0.072594919 |
| SIGLEC14 | ENST00000595478.1 | ENSG00000237031.3 | -0.845511388 | -1.864271544 | 0.062283581 |
| SIGLEC14 | ENST00000596091.1 | ENSG00000227733.4 | -0.923597285 | -2.083968189 | 0.037163061 |
| SIGLEC14 | ENST00000596567.1 | ENSG00000226647.2 | -0.800872638 | -1.789411615 | 0.07354855 |
| SIGLEC14 | ENST00000596887.1 | ENSG00000237031.3 | -0.915084324 | -2.051693942 | 0.040199416 |
| SIGLEC14 | ENST00000598092.1 | ENSG00000228065.6 | -0.936306389 | -2.07826642 | 0.037684828 |
| SIGLEC14 | ENST00000599259.1 | ENSG00000269352.1 | 0.818010588 | 1.81345483 | 0.069761699 |
| SIGLEC14 | ENST00000600242.1 | ENSG00000269583.1 | 0.855578949 | 1.914979698 | 0.055495093 |
| SIGLEC14 | ENST00000600716.1 | ENSG00000269487.1 | 0.883101272 | 1.988106232 | 0.046799942 |
| SIGLEC14 | ENST00000600726.1 | ENSG00000267858.1 | 0.958315672 | 2.135770702 | 0.032698108 |
| SIGLEC14 | ENST00000602949.1 | ENSG00000270030.1 | 0.807259286 | 1.809965717 | 0.070301104 |
| SIGLEC14 | ENST00000604142.1 | ENSG00000271308.1 | 0.927156593 | 2.063045284 | 0.039108326 |
| SIGLEC14 | ENST00000604183.1 | ENSG00000271185.1 | 0.959610883 | 2.157912108 | 0.030934666 |
| SIGLEC14 | ENST00000606470.1 | ENSG00000271913.1 | 0.810989527 | 1.808968551 | 0.070455889 |
| SIGLEC14 | ENST00000606743.1 | ENSG00000272221.1 | 0.853826043 | 1.908112376 | 0.056376693 |
| SIGLEC14 | ENST00000606909.1 | ENSG00000271821.1 | 0.888915828 | 1.998399772 | 0.045673336 |
| SIGLEC14 | ENST00000607148.1 | ENSG00000272477.1 | -0.84331952 | -1.891807169 | 0.058516678 |
| SIGLEC14 | ENST00000607476.1 | ENSG00000272540.1 | 0.92346746 | 2.067348317 | 0.038701345 |
| SIGLEC14 | ENST00000607943.1 | ENSG00000273188.1 | 0.978323245 | 2.205185125 | 0.027441107 |
| SIGLEC14 | ENST00000608259.1 | ENSG00000272627.1 | -0.889596984 | -2.010916696 | 0.044334256 |
| SIGLEC14 | ENST00000608367.1 | ENSG00000273361.1 | 0.91604885 | 2.017284672 | 0.043665814 |
| SIGLEC14 | ENST00000608489.1 | ENSG00000272716.1 | 0.943762979 | 2.090865169 | 0.036540152 |
| SIGLEC14 | ENST00000609113.1 | ENSG00000272827.1 | 0.88410484 | 1.981206293 | 0.047568144 |
| SIGLEC14 | ENST00000609813.1 | ENSG00000272719.1 | 0.853988871 | 1.902502601 | 0.057105479 |
| SIGLEC14 | ENST00000609976.1 | ENSG00000272582.1 | 0.81325776 | 1.816089698 | 0.069356614 |
| SIGLEC14 | NR_003604.2 | ZFAS1 | 0.806473481 | 1.797143186 | 0.072312891 |
| SIGLEC14 | NR_003605.1 | ZFAS1 | 0.866168128 | 1.941034837 | 0.052254052 |
| SIGLEC14 | NR_026802.1 | FAM74A4 | 0.867221036 | 1.913784377 | 0.055647713 |
| SIGLEC14 | NR_026951.1 | LINC00324 | 0.856454155 | 1.913970994 | 0.055623862 |
| SIGLEC14 | NR_027052.1 | THAP7-AS1 | 0.878537341 | 1.976134718 | 0.048139521 |
| SIGLEC14 | NR_027271.1 | CIRBP-AS1 | 0.836984751 | 1.864959922 | 0.062187022 |
| SIGLEC14 | NR_036480.1 | VPS9D1-AS1 | 0.90779043 | 2.019463084 | 0.043439111 |
| SIGLEC14 | NR_037169.1 | LOC100507547 | 0.902478237 | 2.012944305 | 0.044120489 |
| SIGLEC14 | NR_037170.1 | LOC100507547 | 0.875561064 | 1.968302815 | 0.049033206 |
| SIGLEC14 | NR_038421.1 | LINC01220 | 0.952023849 | 2.114761057 | 0.034450318 |
| SIGLEC14 | NR_038923.1 | SSSCA1-AS1 | 0.922571951 | 2.061475623 | 0.039257687 |
| SIGLEC14 | NR_045114.1 | PVRL3-AS1 | -0.83894438 | -1.867001006 | 0.061901447 |
| SIGLEC14 | NR_046871.1 | LINC00333 | -0.803614839 | -1.794712184 | 0.072699568 |
| SIGLEC14 | NR_072981.1 | LINC00957 | 0.909561722 | 2.035301846 | 0.041820511 |
| SIGLEC14 | NR_072982.1 | LINC00957 | 0.901363862 | 2.009647352 | 0.044468526 |
| SIGLEC14 | NR_105010.1 | LINC01333 | 0.827039175 | 1.853208202 | 0.063852522 |
| SIGLEC14 | NR_108106.1 | LINC01135 | 0.803583616 | 1.789818746 | 0.073483055 |
| SIGLEC14 | NR_109831.1 | RASSF1-AS1 | 0.830264261 | 1.885814233 | 0.059319992 |
| SIGLEC14 | NR_109885.1 | RALY-AS1 | 0.802418533 | 1.789994165 | 0.073454849 |

| | | | | | |
|---|---|---|---|---|---|
| SIGLEC14 | NR_109886.1 | RALY-AS1 | 0.843215892 | 1.902471197 | 0.057109581 |
| SIGLEC14 | NR_110117.1 | LOC101927769 | -0.836252985 | -1.864316215 | 0.062277311 |
| SIGLEC14 | NR_110245.1 | LOC101929282 | -0.932300097 | -2.084370979 | 0.037126436 |
| SIGLEC14 | NR_110556.1 | LOC102724890 | -0.87858245 | -1.976711516 | 0.048074248 |
| SIGLEC14 | NR_110630.1 | LOC101927478 | 0.965615988 | 2.167980078 | 0.030160202 |
| SIGLEC14 | NR_110998.1 | FAM74A4 | 0.867221036 | 1.968207661 | 0.049044149 |
| SIGLEC14 | NR_111951.1 | LINC00869 | 0.88721017 | 2.002129714 | 0.045270783 |
| SIGLEC14 | NR_111952.1 | LINC00869 | 0.87690278 | 1.945429499 | 0.051723307 |
| SIGLEC14 | NR_111953.1 | LINC00869 | 0.878005504 | 1.96557154 | 0.049348124 |
| SIGLEC14 | NR_125849.1 | LOC101928140 | -0.844909133 | -1.8833928 | 0.059647155 |
| SIGLEC14 | NR_125957.1 | LOC101928626 | -0.916998873 | -2.063906464 | 0.039026586 |
| SIGLEC14 | NR_126522.1 | EXOC3-AS1 | 0.903910991 | 2.048921933 | 0.040469749 |
| SIGLEC14 | NR_134520.1 | LOC727993 | 0.840702561 | 1.868815218 | 0.061648527 |
| SIGLEC14 | NR_135024.1 | LOC105369747 | 0.902581182 | 1.997496315 | 0.045771294 |
| SIGLEC14 | NR_135040.1 | LOC101927038 | 0.817264388 | 1.826510818 | 0.067773353 |
| SIGLEC14 | NR_135097.1 | LOC105369443 | -0.8889304 | -1.988857251 | 0.046716961 |
| SIGLEC14 | NR_135584.1 | LOC101927596 | 0.839603018 | 1.867919104 | 0.061773348 |
| SIGLEC14 | NR_144459.1 | ARSD-AS1 | 0.941366241 | 2.102222863 | 0.035533758 |
| STUB1 | ENST00000318291.4 | ENSG00000177406.4 | 0.871303293 | 1.958049444 | 0.050224211 |
| STUB1 | ENST00000399186.2 | ENSG00000214888.2 | 0.896848764 | 2.008016385 | 0.044641551 |
| STUB1 | ENST00000411694.1 | ENSG00000225331.1 | 0.877704911 | 1.976581649 | 0.048088938 |
| STUB1 | ENST00000419662.1 | ENSG00000228265.1 | 0.864074276 | 1.930720774 | 0.053517593 |
| STUB1 | ENST00000420315.1 | ENSG00000228072.1 | 0.832989318 | 1.884082583 | 0.059553806 |
| STUB1 | ENST00000420465.1 | ENSG00000167355.3 | 0.821810154 | 1.82896232 | 0.067405253 |
| STUB1 | ENST00000420766.1 | ENSG00000228679.1 | 0.865793603 | 1.949518435 | 0.051233543 |
| STUB1 | ENST00000420981.2 | ENSG00000230438.5 | 0.852933943 | 1.923732673 | 0.0543881 |
| STUB1 | ENST00000421020.1 | ENSG00000231407.1 | 0.974124953 | 2.16822144 | 0.030141842 |
| STUB1 | ENST00000423869.1 | ENSG00000227848.1 | 0.812753878 | 1.83977516 | 0.065801254 |
| STUB1 | ENST00000424181.1 | ENSG00000224977.1 | 0.931768013 | 2.099887922 | 0.035738701 |
| STUB1 | ENST00000425124.1 | ENSG00000232336.1 | 0.821939556 | 1.829268337 | 0.067359419 |
| STUB1 | ENST00000425624.1 | ENSG00000223779.4 | 0.842910547 | 1.88478369 | 0.059459048 |
| STUB1 | ENST00000426519.1 | ENSG00000234142.1 | 0.846214867 | 1.906909591 | 0.056532296 |
| STUB1 | ENST00000433344.1 | ENSG00000234083.1 | -0.852160006 | -1.91560688 | 0.055415153 |
| STUB1 | ENST00000435434.1 | ENSG00000231233.1 | 0.840550227 | 1.907198422 | 0.056494898 |
| STUB1 | ENST00000435733.1 | ENSG00000226377.1 | 0.934758758 | 2.061792444 | 0.039227501 |
| STUB1 | ENST00000435992.2 | ENSG00000232675.3 | 0.966076128 | 2.137143479 | 0.032586323 |
| STUB1 | ENST00000438222.1 | ENSG00000238034.1 | 0.969383275 | 2.159298698 | 0.030827001 |
| STUB1 | ENST00000442069.1 | ENSG00000225655.1 | -0.894861349 | -1.995421667 | 0.04599691 |
| STUB1 | ENST00000443306.1 | ENSG00000233891.3 | 0.81020154 | 1.787692623 | 0.073825612 |
| STUB1 | ENST00000447514.1 | ENSG00000236753.1 | 0.824105373 | 1.827495358 | 0.067625323 |
| STUB1 | ENST00000447709.1 | ENSG00000237473.1 | 0.845317071 | 1.895476554 | 0.058029295 |
| STUB1 | ENST00000452176.1 | ENSG00000223659.1 | -0.910937152 | -2.050811636 | 0.040285294 |
| STUB1 | ENST00000454526.1 | ENSG00000234136.1 | 0.806448297 | 1.773901419 | 0.076079458 |
| STUB1 | ENST00000455788.1 | ENSG00000236263.1 | 0.901674601 | 1.999548122 | 0.045549081 |
| STUB1 | ENST00000457115.1 | ENSG00000227245.1 | 0.869048622 | 1.93232472 | 0.053319439 |
| STUB1 | ENST00000457253.1 | ENSG00000225173.1 | 0.854285386 | 1.914999013 | 0.055492629 |
| STUB1 | ENST00000458154.1 | ENSG00000235578.1 | 0.935937489 | 2.096431965 | 0.036043889 |
| STUB1 | ENST00000458194.1 | ENSG00000226193.1 | 0.808433005 | 1.821442725 | 0.068539586 |
| STUB1 | ENST00000458364.1 | ENSG00000225655.1 | -0.9030559 | -2.038482559 | 0.041501697 |
| STUB1 | ENST00000472596.1 | ENSG00000239774.1 | 0.843418841 | 1.935875204 | 0.052882987 |
| STUB1 | ENST00000484413.1 | ENSG00000271853.1 | 0.933532552 | 2.094837622 | 0.036185429 |

| | | | | | |
|---|---|---|---|---|---|
| STUB1 | ENST00000493123.1 | ENSG00000242428.1 | 0.904466966 | 2.041382029 | 0.041212869 |
| STUB1 | ENST00000494509.1 | ENSG00000240095.1 | 0.960819279 | 2.155276429 | 0.031140209 |
| STUB1 | ENST00000504891.1 | ENSG00000249388.1 | 0.866728341 | 1.935321741 | 0.052950826 |
| STUB1 | ENST00000505498.1 | ENSG00000250908.1 | 0.834351422 | 1.817205209 | 0.069185698 |
| STUB1 | ENST00000505556.1 | ENSG00000249409.1 | 0.836786526 | 1.873690959 | 0.060973033 |
| STUB1 | ENST00000506100.1 | ENSG00000249409.1 | 0.816278037 | 1.822247697 | 0.06841741 |
| STUB1 | ENST00000506791.1 | ENSG00000251131.1 | 0.864587453 | 1.931505986 | 0.05342051 |
| STUB1 | ENST00000508083.1 | ENSG00000249343.1 | 0.835676311 | 1.848153283 | 0.064580172 |
| STUB1 | ENST00000509036.1 | ENSG00000251131.1 | 0.869515473 | 1.937393312 | 0.052697284 |
| STUB1 | ENST00000509192.1 | ENSG00000250765.1 | 0.800146385 | 1.783403375 | 0.074520664 |
| STUB1 | ENST00000517300.1 | ENSG00000254144.2 | 0.860755147 | 1.909887619 | 0.056147685 |
| STUB1 | ENST00000519506.1 | ENSG00000253103.1 | 0.814618627 | 1.831670771 | 0.067000486 |
| STUB1 | ENST00000520603.1 | ENSG00000254001.1 | -0.804194357 | -1.778135177 | 0.075381656 |
| STUB1 | ENST00000521307.1 | ENSG00000253177.1 | 0.930518127 | 2.094235225 | 0.036239031 |
| STUB1 | ENST00000522704.1 | ENSG00000254135.1 | 0.82391841 | 1.815390243 | 0.069463959 |
| STUB1 | ENST00000526154.1 | ENSG00000254511.1 | 0.828403446 | 1.87157327 | 0.061265664 |
| STUB1 | ENST00000526694.1 | ENSG00000231999.2 | 0.87979273 | 1.962393374 | 0.049716704 |
| STUB1 | ENST00000526935.1 | ENSG00000255372.1 | 0.855808188 | 1.924428644 | 0.054300877 |
| STUB1 | ENST00000531627.1 | ENSG00000254584.1 | 0.803826038 | 1.809926051 | 0.070307256 |
| STUB1 | ENST00000532688.1 | ENSG00000255441.1 | 0.830941796 | 1.876142062 | 0.060635775 |
| STUB1 | ENST00000535720.1 | ENSG00000256364.1 | 0.850490962 | 1.893795112 | 0.058252211 |
| STUB1 | ENST00000537921.1 | ENSG00000255966.1 | 0.824377208 | 1.819354512 | 0.068857362 |
| STUB1 | ENST00000545177.3 | ENSG00000230438.5 | 0.859573171 | 1.941319319 | 0.052219558 |
| STUB1 | ENST00000547834.1 | ENSG00000258325.1 | 0.829329349 | 1.851034471 | 0.064164594 |
| STUB1 | ENST00000548722.2 | ENSG00000257194.2 | -0.981277919 | -2.185009532 | 0.028888148 |
| STUB1 | ENST00000549140.1 | ENSG00000258332.1 | 0.861561294 | 1.918701196 | 0.05502216 |
| STUB1 | ENST00000549878.1 | ENSG00000257284.1 | 0.820138461 | 1.860765371 | 0.062777318 |
| STUB1 | ENST00000560522.1 | ENSG00000259661.1 | 0.865400647 | 1.942335867 | 0.052096454 |
| STUB1 | ENST00000563044.1 | ENSG00000260978.1 | 0.935310195 | 2.080272879 | 0.037500511 |
| STUB1 | ENST00000565823.1 | ENSG00000260686.1 | -0.829421721 | -1.875572301 | 0.060714033 |
| STUB1 | ENST00000567127.1 | ENSG00000260264.1 | -0.828181747 | -1.862760066 | 0.062496031 |
| STUB1 | ENST00000567395.1 | ENSG00000261090.1 | 0.942232209 | 2.093481217 | 0.036306219 |
| STUB1 | ENST00000569981.1 | ENSG00000238045.5 | 0.959662375 | 2.139410136 | 0.032402467 |
| STUB1 | ENST00000570493.2 | ENSG00000261898.2 | 0.89762421 | 1.997694441 | 0.045749797 |
| STUB1 | ENST00000570512.1 | ENSG00000262768.1 | 0.923722066 | 2.038157532 | 0.041534181 |
| STUB1 | ENST00000574365.1 | ENSG00000262837.1 | 0.920283412 | 2.072205944 | 0.038246239 |
| STUB1 | ENST00000577807.1 | ENSG00000263427.1 | 0.910549676 | 2.057218914 | 0.039665173 |
| STUB1 | ENST00000578265.1 | ENSG00000214719.7 | 0.891358608 | 1.991650337 | 0.046409436 |
| STUB1 | ENST00000578757.1 | ENSG00000175061.13 | 0.950961194 | 2.123388796 | 0.033721285 |
| STUB1 | ENST00000581362.1 | ENSG00000235300.3 | 0.857784164 | 1.913230655 | 0.055718531 |
| STUB1 | ENST00000581940.1 | ENSG00000265484.1 | 0.864403684 | 1.927281874 | 0.053944511 |
| STUB1 | ENST00000582386.1 | ENSG00000265174.1 | 0.921889897 | 2.064917763 | 0.038930782 |
| STUB1 | ENST00000584758.1 | ENSG00000265356.1 | 0.931700954 | 2.081660868 | 0.037373457 |
| STUB1 | ENST00000585559.1 | ENSG00000267117.1 | 0.869592357 | 1.956699438 | 0.050382816 |
| STUB1 | ENST00000586694.1 | ENSG00000267141.1 | 0.91303683 | 2.037718571 | 0.041578086 |
| STUB1 | ENST00000588380.1 | ENSG00000266990.1 | 0.849803568 | 1.895164873 | 0.058070562 |
| STUB1 | ENST00000593632.1 | ENSG00000180279.5 | 0.867263207 | 1.94906124 | 0.051288112 |
| STUB1 | ENST00000596135.1 | ENSG00000269843.1 | 0.832631794 | 1.881856322 | 0.059855526 |
| STUB1 | ENST00000597169.1 | ENSG00000269720.1 | 0.864209356 | 1.92341998 | 0.054427327 |
| STUB1 | ENST00000599259.1 | ENSG00000269352.1 | 0.943009161 | 2.106654934 | 0.035147501 |
| STUB1 | ENST00000599467.1 | ENSG00000244513.2 | 0.924710036 | 2.047408131 | 0.040618029 |

| | | | | | |
|---|---|---|---|---|---|
| STUB1 | ENST00000600889.1 | ENSG00000232675.3 | 0.811055377 | 1.838630126 | 0.065969607 |
| STUB1 | ENST00000601735.1 | ENSG00000244513.2 | 0.931688376 | 2.109225015 | 0.034925164 |
| STUB1 | ENST00000602594.1 | ENSG00000269930.1 | -0.91399003 | -2.03350769 | 0.042001257 |
| STUB1 | ENST00000602809.1 | ENSG00000270105.1 | -0.995157767 | -2.208284555 | 0.027224443 |
| STUB1 | ENST00000606377.1 | ENSG00000272286.1 | -0.925296044 | -2.086673073 | 0.036917697 |
| STUB1 | ENST00000606470.1 | ENSG00000271913.1 | 0.967827489 | 2.15415205 | 0.03122825 |
| STUB1 | ENST00000606841.1 | ENSG00000272411.1 | 0.826621671 | 1.84883186 | 0.064482095 |
| STUB1 | ENST00000606963.1 | ENSG00000272010.1 | -0.804788801 | -1.807277365 | 0.070719043 |
| STUB1 | ENST00000607876.1 | ENSG00000272848.1 | 0.861831866 | 1.935548474 | 0.052923026 |
| STUB1 | ENST00000608856.1 | ENSG00000272600.1 | -0.823179869 | -1.811593158 | 0.070049083 |
| STUB1 | ENST00000609610.1 | ENSG00000232675.3 | 0.824928348 | 1.872528112 | 0.061133576 |
| STUB1 | ENST00000609725.1 | ENSG00000231898.4 | 0.838423362 | 1.868554281 | 0.061684851 |
| STUB1 | ENST00000610145.1 | ENSG00000273175.1 | 0.846088359 | 1.896500062 | 0.057893952 |
| STUB1 | NR_003604.2 | ZFAS1 | 0.831059 | 1.858884183 | 0.063043557 |
| STUB1 | NR_003605.1 | ZFAS1 | 0.89697067 | 2.00466126 | 0.044999273 |
| STUB1 | NR_003606.2 | ZFAS1 | 0.817745498 | 1.828240043 | 0.067513534 |
| STUB1 | NR_026802.1 | FAM74A4 | 0.86059009 | 1.938734225 | 0.052533709 |
| STUB1 | NR_026951.1 | LINC00324 | 0.809710557 | 1.785845109 | 0.074124341 |
| STUB1 | NR_027271.1 | CIRBP-AS1 | 0.806934174 | 1.822260757 | 0.06841543 |
| STUB1 | NR_028324.1 | LINC01002 | 0.813505933 | 1.813439866 | 0.069764006 |
| STUB1 | NR_036480.1 | VPS9D1-AS1 | 0.90050641 | 2.01755265 | 0.043637872 |
| STUB1 | NR_036658.1 | ZFAS1 | 0.826893206 | 1.825803221 | 0.067879908 |
| STUB1 | NR_044996.1 | HCG23 | 0.808068054 | 1.788260851 | 0.073733933 |
| STUB1 | NR_046742.2 | ZNF630-AS1 | -0.865496653 | -1.95098717 | 0.051058572 |
| STUB1 | NR_046839.1 | AGBL4-IT1 | -0.860035226 | -1.94128659 | 0.052223525 |
| STUB1 | NR_072981.1 | LINC00957 | 0.811552002 | 1.831606464 | 0.067010073 |
| STUB1 | NR_103790.1 | LINC00581 | -0.854868679 | -1.913753476 | 0.055651663 |
| STUB1 | NR_105010.1 | LINC01333 | 0.867513612 | 1.932778516 | 0.053263488 |
| STUB1 | NR_108036.1 | CFAP58-AS1 | 0.836456034 | 1.891469198 | 0.058561739 |
| STUB1 | NR_109886.1 | RALY-AS1 | 0.878765489 | 1.97434391 | 0.048342651 |
| STUB1 | NR_110998.1 | FAM74A4 | 0.86059009 | 1.924220652 | 0.054326932 |
| STUB1 | NR_121188.1 | PGM5P3-AS1 | -0.889267963 | -1.979339737 | 0.047777768 |
| STUB1 | NR_121189.1 | PGM5P3-AS1 | -0.91756196 | -2.041412158 | 0.041209877 |
| STUB1 | NR_125957.1 | LOC101928626 | -0.910937152 | -2.045188169 | 0.04083631 |
| STUB1 | NR_126166.1 | FAM74A7 | 0.813190619 | 1.807768105 | 0.0706426 |
| STUB1 | NR_126522.1 | EXOC3-AS1 | 0.85851504 | 1.906344899 | 0.056605473 |
| STUB1 | NR_134252.1 | LOC105379030 | 0.811693565 | 1.806002156 | 0.070918003 |
| STUB1 | NR_134579.1 | LOC105372179 | 0.822288181 | 1.835359755 | 0.066452403 |
| STUB1 | NR_135024.1 | LOC105369747 | 0.806606305 | 1.801205119 | 0.071670556 |
| STUB1 | NR_135274.1 | LOC105370619 | 0.805980276 | 1.827033037 | 0.067694802 |
| STUB1 | NR_138038.1 | LINC00677 | 0.850996267 | 1.901496231 | 0.057237046 |
| STUB1 | NR_138084.1 | HCG24 | 0.89900222 | 2.02635346 | 0.042728577 |
| STUB1 | NR_144459.1 | ARSD-AS1 | 0.819106865 | 1.836136464 | 0.066337477 |
| SULT1A1 | ENST00000412348.1 | ENSG00000228959.1 | -0.844383825 | -1.868591401 | 0.061679683 |
| SULT1A1 | ENST00000418387.1 | ENSG00000235056.1 | -0.815663261 | -1.8138415 | 0.069702131 |
| SULT1A1 | ENST00000420828.1 | ENSG00000227718.1 | -0.880920324 | -1.970049904 | 0.048832651 |
| SULT1A1 | ENST00000422017.1 | ENSG00000232227.1 | -0.800619446 | -1.803506875 | 0.071308647 |
| SULT1A1 | ENST00000426302.1 | ENSG00000230454.1 | 0.852589309 | 1.912185021 | 0.055852467 |
| SULT1A1 | ENST00000433550.1 | ENSG00000232227.1 | -0.805613115 | -1.802492843 | 0.0714679 |
| SULT1A1 | ENST00000435315.2 | ENSG00000226751.2 | -0.833863687 | -1.884929045 | 0.059439418 |
| SULT1A1 | ENST00000436515.1 | ENSG00000224521.1 | -0.823324354 | -1.85640508 | 0.063395841 |

| | | | | | |
|---|---|---|---|---|---|
| SULT1A1 | ENST00000438409.1 | ENSG00000234174.1 | 0.821515036 | 1.824636499 | 0.068055902 |
| SULT1A1 | ENST00000438623.1 | ENSG00000224521.1 | -0.850097141 | -1.879600228 | 0.060162582 |
| SULT1A1 | ENST00000442435.2 | ENSG00000237781.2 | 0.808913106 | 1.822562277 | 0.068369713 |
| SULT1A1 | ENST00000449154.1 | ENSG00000226969.1 | 0.838609286 | 1.90442601 | 0.056854724 |
| SULT1A1 | ENST00000450304.1 | ENSG00000237886.1 | 0.866302128 | 1.952154044 | 0.050919918 |
| SULT1A1 | ENST00000450531.1 | ENSG00000229536.1 | 0.817133298 | 1.82437283 | 0.068095727 |
| SULT1A1 | ENST00000451034.1 | ENSG00000229805.1 | -0.942112778 | -2.11071287 | 0.034796999 |
| SULT1A1 | ENST00000504301.1 | ENSG00000250696.1 | -0.858647389 | -1.91022473 | 0.056104284 |
| SULT1A1 | ENST00000512882.2 | ENSG00000251575.2 | -0.860799949 | -1.941613806 | 0.05218387 |
| SULT1A1 | ENST00000522390.1 | ENSG00000254262.1 | -0.851461534 | -1.882574858 | 0.059758006 |
| SULT1A1 | ENST00000522600.1 | ENSG00000246582.2 | 0.868372377 | 1.908254267 | 0.056358361 |
| SULT1A1 | ENST00000527086.1 | ENSG00000255182.1 | 0.900993565 | 2.022829193 | 0.043090761 |
| SULT1A1 | ENST00000539963.1 | ENSG00000256116.1 | 0.930363249 | 2.094543825 | 0.036211563 |
| SULT1A1 | ENST00000543275.1 | ENSG00000256944.1 | 0.811320042 | 1.838659549 | 0.065965277 |
| SULT1A1 | ENST00000543494.1 | ENSG00000256514.1 | 0.944265579 | 2.151157809 | 0.031463746 |
| SULT1A1 | ENST00000548210.1 | ENSG00000257784.1 | 0.864880352 | 1.933897303 | 0.053125756 |
| SULT1A1 | ENST00000549683.1 | ENSG00000257953.1 | 0.844546757 | 1.849274209 | 0.064418228 |
| SULT1A1 | ENST00000551135.1 | ENSG00000258294.1 | -0.849306451 | -1.907591769 | 0.056444 |
| SULT1A1 | ENST00000558575.1 | ENSG00000259687.1 | 0.856190792 | 1.918136813 | 0.055093666 |
| SULT1A1 | ENST00000561699.1 | ENSG00000259813.1 | 0.877849515 | 1.961117969 | 0.049865263 |
| SULT1A1 | ENST00000563610.1 | ENSG00000260051.1 | 0.804644357 | 1.792013897 | 0.07313074 |
| SULT1A1 | ENST00000565667.1 | ENSG00000261253.1 | 0.965870321 | 2.137930851 | 0.032522356 |
| SULT1A1 | ENST00000565798.2 | ENSG00000259786.2 | -0.958265219 | -2.159327297 | 0.030824784 |
| SULT1A1 | ENST00000567261.1 | ENSG00000261320.1 | -0.806589418 | -1.800705186 | 0.07174936 |
| SULT1A1 | ENST00000568659.1 | ENSG00000260004.1 | -0.965940831 | -2.17572863 | 0.029575558 |
| SULT1A1 | ENST00000570974.1 | ENSG00000263300.1 | 0.904737009 | 2.020689485 | 0.043311919 |
| SULT1A1 | ENST00000573260.1 | ENSG00000262482.1 | -0.81018628 | -1.836180378 | 0.066330984 |
| SULT1A1 | ENST00000576086.1 | ENSG00000262823.1 | 0.861766976 | 1.904421244 | 0.056855345 |
| SULT1A1 | ENST00000577176.1 | ENSG00000262823.1 | 0.826839821 | 1.836394784 | 0.06629929 |
| SULT1A1 | ENST00000583916.1 | ENSG00000264196.1 | 0.885291003 | 1.995899576 | 0.045944855 |
| SULT1A1 | ENST00000588402.1 | ENSG00000267006.1 | -0.817656267 | -1.819424122 | 0.068846749 |
| SULT1A1 | ENST00000589673.1 | ENSG00000267755.1 | 0.8102287 | 1.820931863 | 0.068617215 |
| SULT1A1 | ENST00000590328.1 | ENSG00000256995.2 | -0.956312493 | -2.130538853 | 0.033127152 |
| SULT1A1 | ENST00000592518.1 | ENSG00000267786.1 | 0.809068035 | 1.792751539 | 0.073012661 |
| SULT1A1 | ENST00000598092.1 | ENSG00000228065.6 | -0.819411729 | -1.82618943 | 0.067821733 |
| SULT1A1 | ENST00000600007.1 | ENSG00000268655.1 | 0.981024171 | 2.19962169 | 0.027833747 |
| SULT1A1 | ENST00000600716.1 | ENSG00000269487.1 | 0.863779112 | 1.917914252 | 0.055121885 |
| SULT1A1 | ENST00000602881.1 | ENSG00000269965.1 | -0.825076186 | -1.862705317 | 0.062503737 |
| SULT1A1 | ENST00000606010.1 | ENSG00000272249.1 | -0.809157312 | -1.809877046 | 0.070314857 |
| SULT1A1 | ENST00000606855.1 | ENSG00000245937.3 | 0.819580553 | 1.838441915 | 0.065997314 |
| SULT1A1 | ENST00000607119.1 | ENSG00000272541.1 | -0.954380351 | -2.142482513 | 0.032154674 |
| SULT1A1 | ENST00000607665.1 | ENSG00000272254.1 | -0.831050404 | -1.850651949 | 0.064219641 |
| SULT1A1 | ENST00000608465.1 | ENSG00000272758.1 | -0.91342592 | -2.032420447 | 0.04211111 |
| SULT1A1 | ENST00000609113.1 | ENSG00000272827.1 | 0.926402886 | 2.076546503 | 0.037843435 |
| SULT1A1 | ENST00000609976.1 | ENSG00000272582.1 | 0.909045132 | 2.033075177 | 0.042044928 |
| SULT1A1 | ENST00000610270.1 | ENSG00000272576.1 | -0.908609648 | -2.057941781 | 0.039595722 |
| SULT1A1 | NR_024491.1 | LOC100128573 | -0.867784774 | -1.930897156 | 0.053495772 |
| SULT1A1 | NR_046571.1 | POTEH-AS1 | 0.95041741 | 2.139291751 | 0.032412047 |
| SULT1A1 | NR_073552.1 | LOC101059948 | 0.981024171 | 2.185023704 | 0.028887109 |
| SULT1A1 | NR_134566.1 | LOC105372695 | -0.820155054 | -1.844446357 | 0.065118117 |
| SULT1A1 | NR_135040.1 | LOC101927038 | 0.824553793 | 1.827221962 | 0.067666403 |

| | | | | | |
|---|---|---|---|---|---|
| SULT1A1 | NR_135041.1 | LOC101927038 | 0.81855565 | 1.842459867 | 0.065407912 |
| SULT1A1 | NR_136569.1 | LINC01660 | -0.810170474 | -1.826884165 | 0.067717188 |
| SULT1A3 | ENST00000412812.1 | ENSG00000225342.1 | 0.840945297 | 1.878516656 | 0.060310521 |
| SULT1A3 | ENST00000418080.1 | ENSG00000224091.1 | -0.885128938 | -1.973799644 | 0.048404529 |
| SULT1A3 | ENST00000418387.1 | ENSG00000235056.1 | 0.806276841 | 1.812184604 | 0.069957677 |
| SULT1A3 | ENST00000424342.1 | ENSG00000234988.1 | -0.806058444 | -1.791740152 | 0.0731746 |
| SULT1A3 | ENST00000425058.1 | ENSG00000226771.1 | 0.815553968 | 1.807087922 | 0.070748572 |
| SULT1A3 | ENST00000426929.1 | ENSG00000230184.1 | -0.86324714 | -1.947119647 | 0.051520392 |
| SULT1A3 | ENST00000428391.1 | ENSG00000224691.1 | -0.819139539 | -1.821052617 | 0.068598859 |
| SULT1A3 | ENST00000429916.1 | ENSG00000227708.1 | 0.948902163 | 2.087571249 | 0.036836528 |
| SULT1A3 | ENST00000430247.1 | ENSG00000232855.2 | 0.804867971 | 1.799395262 | 0.071956179 |
| SULT1A3 | ENST00000432265.1 | ENSG00000231170.1 | -0.95185837 | -2.11473366 | 0.034452654 |
| SULT1A3 | ENST00000433249.1 | ENSG00000236556.1 | 0.844116776 | 1.877869484 | 0.060399022 |
| SULT1A3 | ENST00000435828.1 | ENSG00000235612.1 | 0.877235154 | 1.952874522 | 0.050834465 |
| SULT1A3 | ENST00000437864.1 | ENSG00000231521.1 | 0.888798449 | 1.991255963 | 0.046452754 |
| SULT1A3 | ENST00000441295.1 | ENSG00000233960.1 | -0.812777775 | -1.821202715 | 0.068576048 |
| SULT1A3 | ENST00000441666.1 | ENSG00000230379.1 | 0.846390859 | 1.902045396 | 0.05716522 |
| SULT1A3 | ENST00000441991.1 | ENSG00000231210.2 | -0.866598244 | -1.952470921 | 0.05088232 |
| SULT1A3 | ENST00000442876.1 | ENSG00000233894.1 | 0.896543338 | 2.000064663 | 0.045493282 |
| SULT1A3 | ENST00000443066.2 | ENSG00000237633.2 | 0.962400442 | 2.166172957 | 0.030297973 |
| SULT1A3 | ENST00000445178.1 | ENSG00000234653.1 | 0.839664201 | 1.873376154 | 0.061016461 |
| SULT1A3 | ENST00000446816.1 | ENSG00000204685.5 | -0.842426242 | -1.87170904 | 0.061246868 |
| SULT1A3 | ENST00000448748.1 | ENSG00000231238.1 | 0.885904444 | 1.974521432 | 0.048322483 |
| SULT1A3 | ENST00000449903.1 | ENSG00000223872.1 | -0.885933289 | -1.960518205 | 0.049935252 |
| SULT1A3 | ENST00000450779.1 | ENSG00000228858.1 | 0.917228832 | 2.08560831 | 0.037014118 |
| SULT1A3 | ENST00000452511.1 | ENSG00000231876.3 | 0.934673839 | 2.091171445 | 0.036512698 |
| SULT1A3 | ENST00000454182.1 | ENSG00000230379.1 | 0.816410193 | 1.843259123 | 0.065291186 |
| SULT1A3 | ENST00000454387.1 | ENSG00000223726.1 | -0.801537756 | -1.794336691 | 0.072759445 |
| SULT1A3 | ENST00000456499.1 | ENSG00000237640.1 | -0.922505356 | -2.047895974 | 0.040570193 |
| SULT1A3 | ENST00000456999.1 | ENSG00000230690.1 | 0.882296662 | 1.958842873 | 0.050131191 |
| SULT1A3 | ENST00000457043.1 | ENSG00000231365.1 | 0.870407547 | 1.958875967 | 0.050127314 |
| SULT1A3 | ENST00000458082.1 | ENSG00000231210.2 | -0.81591106 | -1.829059458 | 0.067390702 |
| SULT1A3 | ENST00000479039.1 | ENSG00000241224.2 | -0.815971597 | -1.837090141 | 0.066196589 |
| SULT1A3 | ENST00000485338.1 | ENSG00000239641.1 | 0.921179253 | 2.099499641 | 0.035772879 |
| SULT1A3 | ENST00000490375.1 | ENSG00000240032.1 | 0.901414845 | 1.978047424 | 0.047923357 |
| SULT1A3 | ENST00000502467.1 | ENSG00000250530.1 | 0.833450657 | 1.829893417 | 0.067265878 |
| SULT1A3 | ENST00000503470.1 | ENSG00000248559.1 | 0.844165636 | 1.873699741 | 0.060971822 |
| SULT1A3 | ENST00000503505.1 | ENSG00000248629.1 | 0.847783919 | 1.910919557 | 0.056014919 |
| SULT1A3 | ENST00000503987.1 | ENSG00000250075.1 | 0.943701093 | 2.121355511 | 0.033891896 |
| SULT1A3 | ENST00000504765.1 | ENSG00000249638.1 | 0.904687232 | 2.006267588 | 0.044827706 |
| SULT1A3 | ENST00000505978.1 | ENSG00000249982.1 | -0.929167788 | -2.048698312 | 0.040491624 |
| SULT1A3 | ENST00000506420.1 | ENSG00000250034.1 | -0.833521733 | -1.876105766 | 0.060640758 |
| SULT1A3 | ENST00000508687.1 | ENSG00000250538.1 | 0.90159477 | 1.99965908 | 0.04553709 |
| SULT1A3 | ENST00000510001.2 | ENSG00000249196.2 | -0.845948895 | -1.888434856 | 0.058967596 |
| SULT1A3 | ENST00000512882.2 | ENSG00000251575.2 | 0.822530972 | 1.819614684 | 0.068817704 |
| SULT1A3 | ENST00000514966.1 | ENSG00000251310.1 | -0.852226025 | -1.899050641 | 0.057557818 |
| SULT1A3 | ENST00000519062.1 | ENSG00000253658.1 | 0.825606949 | 1.82988914 | 0.067266518 |
| SULT1A3 | ENST00000522123.1 | ENSG00000253836.1 | 0.869738426 | 1.947568573 | 0.051466607 |
| SULT1A3 | ENST00000525855.1 | ENSG00000254746.1 | -0.803558 | -1.792667557 | 0.073026097 |
| SULT1A3 | ENST00000528607.1 | ENSG00000254604.1 | 0.903060913 | 2.035970461 | 0.041753322 |
| SULT1A3 | ENST00000532123.1 | ENSG00000255555.1 | 0.920386846 | 2.052867781 | 0.040085403 |

| | | | | | |
|---|---|---|---|---|---|
| SULT1A3 | ENST00000533101.1 | ENSG00000255311.1 | 0.82832006 | 1.839821252 | 0.065794484 |
| SULT1A3 | ENST00000537032.1 | ENSG00000255933.1 | -0.812043345 | -1.843661019 | 0.065232557 |
| SULT1A3 | ENST00000537492.1 | ENSG00000256637.2 | 0.986208908 | 2.218582447 | 0.026515145 |
| SULT1A3 | ENST00000545158.1 | ENSG00000256011.1 | 0.933007823 | 2.103065422 | 0.035460051 |
| SULT1A3 | ENST00000545572.1 | ENSG00000255680.1 | -0.873179432 | -1.961558264 | 0.049813935 |
| SULT1A3 | ENST00000546135.1 | ENSG00000256670.1 | -0.861949089 | -1.947767361 | 0.051442806 |
| SULT1A3 | ENST00000550138.1 | ENSG00000257467.1 | 0.801439675 | 1.799976763 | 0.071864307 |
| SULT1A3 | ENST00000553075.1 | ENSG00000257258.1 | 0.919051904 | 2.062102668 | 0.039197962 |
| SULT1A3 | ENST00000553954.1 | ENSG00000259052.1 | -0.862157473 | -1.958603838 | 0.0501592 |
| SULT1A3 | ENST00000555689.1 | ENSG00000259049.1 | -0.822402377 | -1.832412524 | 0.066889984 |
| SULT1A3 | ENST00000559041.1 | ENSG00000259713.1 | 0.88599577 | 1.978800025 | 0.047838526 |
| SULT1A3 | ENST00000560963.1 | ENSG00000259370.1 | -0.892926742 | -1.978811924 | 0.047837185 |
| SULT1A3 | ENST00000560969.1 | ENSG00000259176.1 | 0.839664201 | 1.866422847 | 0.061982229 |
| SULT1A3 | ENST00000561254.1 | ENSG00000259554.1 | 0.830229779 | 1.830729647 | 0.067140906 |
| SULT1A3 | ENST00000561544.1 | ENSG00000261532.1 | -0.980516541 | -2.22692349 | 0.025952388 |
| SULT1A3 | ENST00000566521.1 | ENSG00000261629.1 | 0.909145009 | 2.039948759 | 0.04135543 |
| SULT1A3 | ENST00000569328.1 | ENSG00000261638.1 | 0.867511647 | 1.920443782 | 0.054801868 |
| SULT1A3 | ENST00000570413.1 | ENSG00000263167.1 | -0.919614981 | -2.040650513 | 0.041285578 |
| SULT1A3 | ENST00000570700.1 | ENSG00000263011.1 | -0.875755106 | -1.99004897 | 0.046585541 |
| SULT1A3 | ENST00000572608.1 | ENSG00000263305.1 | 0.803912457 | 1.806914774 | 0.070775569 |
| SULT1A3 | ENST00000580729.1 | ENSG00000266176.1 | -0.909292847 | -2.049926595 | 0.040371594 |
| SULT1A3 | ENST00000584139.1 | ENSG00000263388.1 | 0.937180625 | 2.060180523 | 0.039381286 |
| SULT1A3 | ENST00000585810.1 | ENSG00000236172.2 | -0.85777329 | -1.932966202 | 0.053240361 |
| SULT1A3 | ENST00000585877.1 | ENSG00000267249.1 | -0.961615414 | -2.126790768 | 0.033437472 |
| SULT1A3 | ENST00000587702.1 | ENSG00000267378.1 | 0.800464561 | 1.772804055 | 0.076261181 |
| SULT1A3 | ENST00000590046.1 | ENSG00000266950.1 | -0.882350017 | -1.982334798 | 0.047441782 |
| SULT1A3 | ENST00000591414.1 | ENSG00000267011.1 | -0.818067482 | -1.856129795 | 0.06343506 |
| SULT1A3 | ENST00000592816.1 | ENSG00000236172.2 | -0.822798705 | -1.817010799 | 0.06921546 |
| SULT1A3 | ENST00000593568.1 | ENSG00000228065.6 | -0.822118164 | -1.832253051 | 0.066913728 |
| SULT1A3 | ENST00000595007.1 | ENSG00000231876.3 | 0.919866527 | 2.069809203 | 0.038470215 |
| SULT1A3 | ENST00000596473.1 | ENSG00000268650.3 | 0.879079403 | 1.957236026 | 0.050319725 |
| SULT1A3 | ENST00000597680.1 | ENSG00000269574.1 | 0.880927103 | 1.956244529 | 0.050436355 |
| SULT1A3 | ENST00000597755.1 | ENSG00000236194.2 | 0.828396604 | 1.86492333 | 0.062192152 |
| SULT1A3 | ENST00000597865.1 | ENSG00000268108.1 | 0.91455224 | 2.025929051 | 0.042772056 |
| SULT1A3 | ENST00000598887.1 | ENSG00000268475.1 | -0.803025512 | -1.801732368 | 0.071587523 |
| SULT1A3 | ENST00000602405.1 | ENSG00000269928.1 | -0.919180967 | -2.024733572 | 0.042894729 |
| SULT1A3 | ENST00000602454.1 | ENSG00000270139.1 | 0.919890523 | 2.043046316 | 0.041047853 |
| SULT1A3 | ENST00000603949.1 | ENSG00000270332.1 | 0.850316566 | 1.954596984 | 0.050630658 |
| SULT1A3 | ENST00000604312.1 | ENSG00000270947.1 | 0.87540555 | 1.953356948 | 0.050777314 |
| SULT1A3 | ENST00000606010.1 | ENSG00000272249.1 | 0.876754006 | 1.956651604 | 0.050388443 |
| SULT1A3 | ENST00000606942.1 | ENSG00000271835.1 | 0.895927395 | 2.032214115 | 0.042131985 |
| SULT1A3 | ENST00000607135.1 | ENSG00000272112.1 | 0.838781361 | 1.877268142 | 0.060481352 |
| SULT1A3 | ENST00000607549.1 | ENSG00000272293.1 | 0.861824992 | 1.905498129 | 0.05671535 |
| SULT1A3 | ENST00000607600.1 | ENSG00000272114.1 | -0.877373451 | -1.972556005 | 0.04854617 |
| SULT1A3 | ENST00000608133.1 | ENSG00000273193.1 | 0.808035621 | 1.814482967 | 0.069603403 |
| SULT1A3 | ENST00000608259.1 | ENSG00000272627.1 | 0.819432647 | 1.841004986 | 0.065620828 |
| SULT1A3 | ENST00000609182.1 | ENSG00000273248.1 | -0.86186431 | -1.931557237 | 0.053414178 |
| SULT1A3 | ENST00000609813.1 | ENSG00000272719.1 | -0.893028042 | -1.997107348 | 0.045813523 |
| SULT1A3 | ENST00000609955.1 | ENSG00000273275.1 | 0.919551734 | 2.024149321 | 0.04295479 |
| SULT1A3 | ENST00000610034.1 | ENSG00000272912.1 | 0.878705346 | 1.971535432 | 0.048662664 |
| SULT1A3 | NR_024470.1 | SLCO4A1-AS1 | -0.833000337 | -1.871392627 | 0.06129068 |

| | | | | | |
|---|---|---|---|---|---|
| SULT1A3 | NR_026962.1 | TTC28-AS1 | -0.834669586 | -1.904376779 | 0.056861131 |
| SULT1A3 | NR_027067.1 | LINC00114 | -0.904419861 | -2.075543447 | 0.037936197 |
| SULT1A3 | NR_047498.1 | LINC00853 | -0.808839374 | -1.803144114 | 0.071365585 |
| SULT1A3 | NR_102738.1 | LINC00911 | -0.915988103 | -2.019074949 | 0.04347943 |
| SULT1A3 | NR_110284.1 | LOC101927907 | 0.839664201 | 1.861006151 | 0.062743308 |
| SULT1A3 | NR_110702.1 | SEMA3B-AS1 | -0.867166587 | -1.955075673 | 0.050574139 |
| SULT1A3 | NR_110846.1 | LOC101928674 | -0.853501402 | -1.916823347 | 0.055260378 |
| SULT1A3 | NR_126370.1 | GACAT1 | -0.839857357 | -1.900428736 | 0.057376879 |
| SULT1A3 | NR_126409.1 | LINC00376 | 0.933309047 | 2.093041273 | 0.03634547 |
| SULT1A3 | NR_133907.1 | HLA-DQB1-AS1 | 0.939695661 | 2.095776261 | 0.036102043 |
| SULT1A3 | NR_134576.1 | LOC105372672 | -0.886889587 | -1.977266666 | 0.048011495 |
| SULT1A3 | NR_134910.1 | LOC102725254 | 0.911880781 | 2.016193321 | 0.043779765 |
| SULT1A3 | NR_135251.1 | LOC101928143 | -0.940687029 | -2.108956711 | 0.034948318 |
| SULT1A3 | NR_136569.1 | LINC01660 | 0.881529637 | 1.968331929 | 0.049029859 |
| TAS2R1 | ENST00000398777.3 | ENSG00000240152.2 | -0.855895308 | -1.940863939 | 0.052274783 |
| TAS2R1 | ENST00000417426.1 | ENSG00000233145.1 | -0.802915119 | -1.776179087 | 0.075703404 |
| TAS2R1 | ENST00000417782.1 | ENSG00000228587.1 | -0.828111533 | -1.837426826 | 0.06614691 |
| TAS2R1 | ENST00000418741.1 | ENSG00000227332.1 | -0.833560278 | -1.836745781 | 0.066247433 |
| TAS2R1 | ENST00000419103.1 | ENSG00000227014.1 | -0.843203859 | -1.885108108 | 0.059415244 |
| TAS2R1 | ENST00000421252.2 | ENSG00000250258.1 | 0.811143904 | 1.817434878 | 0.069150551 |
| TAS2R1 | ENST00000423667.1 | ENSG00000225970.1 | -0.881370336 | -1.928111479 | 0.053841262 |
| TAS2R1 | ENST00000425124.1 | ENSG00000232336.1 | -0.820509945 | -1.836947381 | 0.066217664 |
| TAS2R1 | ENST00000425624.1 | ENSG00000223779.4 | -0.963187185 | -2.145509593 | 0.031912125 |
| TAS2R1 | ENST00000426699.1 | ENSG00000229308.1 | -0.91649097 | -2.071268169 | 0.038333742 |
| TAS2R1 | ENST00000428769.1 | ENSG00000232738.1 | -0.845250666 | -1.863818999 | 0.062347127 |
| TAS2R1 | ENST00000432314.1 | ENSG00000231532.1 | -0.958931958 | -2.163749929 | 0.030483547 |
| TAS2R1 | ENST00000433344.1 | ENSG00000234083.1 | 0.915093065 | 2.058338604 | 0.039557641 |
| TAS2R1 | ENST00000434790.1 | ENSG00000240040.1 | 0.938688147 | 2.125684084 | 0.033529573 |
| TAS2R1 | ENST00000435992.2 | ENSG00000232675.3 | -0.898221491 | -2.014643208 | 0.043942047 |
| TAS2R1 | ENST00000436982.2 | ENSG00000235335.2 | 0.864215232 | 1.898063293 | 0.057687745 |
| TAS2R1 | ENST00000438222.1 | ENSG00000238034.1 | -0.847543086 | -1.887188801 | 0.059134936 |
| TAS2R1 | ENST00000439186.1 | ENSG00000237076.1 | -0.874883596 | -1.945856047 | 0.051672034 |
| TAS2R1 | ENST00000440947.1 | ENSG00000225472.1 | -0.808812889 | -1.834461008 | 0.066585591 |
| TAS2R1 | ENST00000441160.1 | ENSG00000228437.1 | 0.841964183 | 1.880574869 | 0.060029773 |
| TAS2R1 | ENST00000447514.1 | ENSG00000236753.1 | -0.860833165 | -1.914855289 | 0.055510961 |
| TAS2R1 | ENST00000447538.2 | ENSG00000224189.2 | -0.87857326 | -1.935367225 | 0.052945248 |
| TAS2R1 | ENST00000450226.1 | ENSG00000231512.1 | -0.878833516 | -1.960171111 | 0.049975794 |
| TAS2R1 | ENST00000450848.1 | ENSG00000225539.1 | -0.866317449 | -1.936735572 | 0.052777675 |
| TAS2R1 | ENST00000454526.1 | ENSG00000234136.1 | -0.810703924 | -1.823669618 | 0.068202035 |
| TAS2R1 | ENST00000455238.1 | ENSG00000231413.1 | -0.829021949 | -1.865971639 | 0.062045334 |
| TAS2R1 | ENST00000455699.1 | ENSG00000240996.1 | -0.970641064 | -2.131375056 | 0.033058256 |
| TAS2R1 | ENST00000455788.1 | ENSG00000236263.1 | -0.846313961 | -1.873269448 | 0.061031187 |
| TAS2R1 | ENST00000457169.1 | ENSG00000232408.1 | -0.858824895 | -1.926069999 | 0.054095634 |
| TAS2R1 | ENST00000458661.2 | ENSG00000236467.3 | -0.952360157 | -2.157990645 | 0.030928559 |
| TAS2R1 | ENST00000468444.2 | ENSG00000258525.1 | 0.815469574 | 1.820189905 | 0.06873009 |
| TAS2R1 | ENST00000469931.2 | ENSG00000272030.1 | -0.893259473 | -1.982388546 | 0.047435771 |
| TAS2R1 | ENST00000481334.1 | ENSG00000242440.1 | -0.818804635 | -1.860582974 | 0.062803091 |
| TAS2R1 | ENST00000484413.1 | ENSG00000271853.1 | -0.895814443 | -1.99153368 | 0.046422246 |
| TAS2R1 | ENST00000494509.1 | ENSG00000240095.1 | -0.888365553 | -1.974379749 | 0.048338579 |
| TAS2R1 | ENST00000501405.2 | ENSG00000247402.2 | 0.801163506 | 1.798735389 | 0.072060548 |
| TAS2R1 | ENST00000504344.1 | ENSG00000251438.1 | -0.880223695 | -1.930826546 | 0.053504506 |

| | | | | | |
|---|---|---|---|---|---|
| TAS2R1 | ENST00000504891.1 | ENSG00000249388.1 | -0.969400965 | -2.20518729 | 0.027440955 |
| TAS2R1 | ENST00000505498.1 | ENSG00000250908.1 | -0.824354357 | -1.856336998 | 0.063405539 |
| TAS2R1 | ENST00000507373.1 | ENSG00000250072.1 | -0.933580778 | -2.129249221 | 0.033233647 |
| TAS2R1 | ENST00000508986.1 | ENSG00000249491.1 | -0.837139205 | -1.897203962 | 0.057801024 |
| TAS2R1 | ENST00000513179.1 | ENSG00000251580.1 | -0.866105797 | -1.974085044 | 0.048372074 |
| TAS2R1 | ENST00000515205.1 | ENSG00000251580.1 | -0.8957055 | -1.979065261 | 0.047808659 |
| TAS2R1 | ENST00000517716.1 | ENSG00000253515.1 | 0.837362274 | 1.868904969 | 0.061636037 |
| TAS2R1 | ENST00000518473.1 | ENSG00000253985.1 | -0.993914438 | -2.201608504 | 0.027692975 |
| TAS2R1 | ENST00000521307.1 | ENSG00000253177.1 | -0.883132111 | -1.996834848 | 0.045843127 |
| TAS2R1 | ENST00000521953.1 | ENSG00000253214.1 | -0.876073948 | -1.972303027 | 0.048575024 |
| TAS2R1 | ENST00000522704.1 | ENSG00000254135.1 | -0.880329268 | -1.95222365 | 0.050911658 |
| TAS2R1 | ENST00000524818.1 | ENSG00000254473.1 | -0.822798281 | -1.856651732 | 0.063360719 |
| TAS2R1 | ENST00000525133.1 | ENSG00000255375.1 | -0.842399082 | -1.893698395 | 0.058265054 |
| TAS2R1 | ENST00000526186.1 | ENSG00000254510.1 | -0.838659858 | -1.887894803 | 0.059040074 |
| TAS2R1 | ENST00000526935.1 | ENSG00000255372.1 | -0.871543165 | -1.97140276 | 0.048677826 |
| TAS2R1 | ENST00000529837.1 | ENSG00000254687.1 | -0.869977144 | -1.956366734 | 0.050421968 |
| TAS2R1 | ENST00000531661.1 | ENSG00000254473.1 | -0.840594171 | -1.890769519 | 0.058655118 |
| TAS2R1 | ENST00000532680.1 | ENSG00000255458.1 | -0.924856238 | -2.08488758 | 0.037079506 |
| TAS2R1 | ENST00000545593.1 | ENSG00000256972.1 | -0.846651024 | -1.937550976 | 0.052678028 |
| TAS2R1 | ENST00000547207.1 | ENSG00000224189.2 | -0.853532965 | -1.894731193 | 0.058128023 |
| TAS2R1 | ENST00000547834.1 | ENSG00000258325.1 | -0.882404536 | -2.013181997 | 0.044095487 |
| TAS2R1 | ENST00000549140.1 | ENSG00000258332.1 | -0.928432367 | -2.07486696 | 0.037998867 |
| TAS2R1 | ENST00000549487.1 | ENSG00000257126.1 | -0.840862356 | -1.917215286 | 0.055210587 |
| TAS2R1 | ENST00000552156.1 | ENSG00000224189.2 | -0.82330755 | -1.852715476 | 0.063923151 |
| TAS2R1 | ENST00000554679.1 | ENSG00000258837.1 | -0.83490716 | -1.883457283 | 0.059638424 |
| TAS2R1 | ENST00000555966.1 | ENSG00000258843.1 | -0.874569543 | -1.931411081 | 0.053432236 |
| TAS2R1 | ENST00000556786.1 | ENSG00000258525.1 | 0.809648491 | 1.825421539 | 0.067937441 |
| TAS2R1 | ENST00000557965.1 | ENSG00000259681.1 | -0.865722511 | -1.959788652 | 0.050020498 |
| TAS2R1 | ENST00000558896.1 | ENSG00000259176.1 | -0.886319196 | -1.98019792 | 0.047681294 |
| TAS2R1 | ENST00000559003.1 | ENSG00000259520.1 | -0.957324509 | -2.155803559 | 0.031099007 |
| TAS2R1 | ENST00000560586.1 | ENSG00000259534.1 | -0.876049189 | -1.991280155 | 0.046450096 |
| TAS2R1 | ENST00000563841.1 | ENSG00000261029.1 | -0.958931702 | -2.139643635 | 0.032383577 |
| TAS2R1 | ENST00000565965.1 | ENSG00000261172.1 | -0.932703548 | -2.073033942 | 0.038169121 |
| TAS2R1 | ENST00000567089.1 | ENSG00000261822.1 | -0.874560629 | -1.939884977 | 0.05239367 |
| TAS2R1 | ENST00000567395.1 | ENSG00000261090.1 | -0.911813746 | -2.031436377 | 0.042210747 |
| TAS2R1 | ENST00000569981.1 | ENSG00000238045.5 | -0.818472833 | -1.826622572 | 0.067756537 |
| TAS2R1 | ENST00000570512.1 | ENSG00000262768.1 | -0.855285639 | -1.898046646 | 0.057689938 |
| TAS2R1 | ENST00000574365.1 | ENSG00000262837.1 | -0.938526405 | -2.094581525 | 0.036208209 |
| TAS2R1 | ENST00000577698.1 | ENSG00000265100.1 | -0.811622291 | -1.823707316 | 0.068196332 |
| TAS2R1 | ENST00000578265.1 | ENSG00000214719.7 | -0.85333136 | -1.913133542 | 0.055730959 |
| TAS2R1 | ENST00000580085.1 | ENSG00000266490.1 | -0.819623712 | -1.826005397 | 0.067849449 |
| TAS2R1 | ENST00000581362.1 | ENSG00000235300.3 | -0.897989845 | -2.0275581 | 0.042605369 |
| TAS2R1 | ENST00000584758.1 | ENSG00000265356.1 | -0.921996978 | -2.068424107 | 0.03860016 |
| TAS2R1 | ENST00000585559.1 | ENSG00000267117.1 | -0.826667553 | -1.8327853 | 0.066834506 |
| TAS2R1 | ENST00000588799.1 | ENSG00000267275.1 | -0.967484416 | -2.171599765 | 0.029885865 |
| TAS2R1 | ENST00000588945.1 | ENSG00000267275.1 | -0.986961879 | -2.170509602 | 0.029968262 |
| TAS2R1 | ENST00000589777.1 | ENSG00000261040.2 | -0.931616265 | -2.115879438 | 0.034355063 |
| TAS2R1 | ENST00000593861.1 | ENSG00000231898.4 | -0.907082485 | -2.012557578 | 0.044161194 |
| TAS2R1 | ENST00000597530.1 | ENSG00000228401.3 | -0.829979038 | -1.842617831 | 0.065384829 |
| TAS2R1 | ENST00000597550.1 | ENSG00000269051.1 | -0.905609538 | -2.019417586 | 0.043443835 |
| TAS2R1 | ENST00000599143.1 | ENSG00000269349.1 | -0.937415943 | -2.070114233 | 0.038441648 |

| | | | | | |
|---|---|---|---|---|---|
| TAS2R1 | ENST00000600489.1 | ENSG00000231898.4 | -0.930367695 | -2.061078229 | 0.039295577 |
| TAS2R1 | ENST00000601692.1 | ENSG00000267874.1 | 0.880730115 | 1.972174427 | 0.048589698 |
| TAS2R1 | ENST00000602592.1 | ENSG00000270049.1 | -0.828820189 | -1.817599084 | 0.069125432 |
| TAS2R1 | ENST00000602809.1 | ENSG00000270105.1 | 0.84269827 | 1.876003714 | 0.06065477 |
| TAS2R1 | ENST00000602954.1 | ENSG00000269906.1 | -0.845163657 | -1.887040788 | 0.05915484 |
| TAS2R1 | ENST00000603474.1 | ENSG00000258929.2 | -0.894737309 | -2.000300445 | 0.045467831 |
| TAS2R1 | ENST00000604464.1 | ENSG00000270462.1 | -0.840911 | -1.859124921 | 0.063009434 |
| TAS2R1 | ENST00000606457.1 | ENSG00000271830.1 | -0.920291804 | -2.051632748 | 0.040205367 |
| TAS2R1 | ENST00000606470.1 | ENSG00000271913.1 | -0.844743267 | -1.886973389 | 0.059163905 |
| TAS2R1 | ENST00000607284.1 | ENSG00000272389.1 | -0.897991397 | -2.019367439 | 0.043449043 |
| TAS2R1 | ENST00000607769.1 | ENSG00000272438.1 | 0.808205819 | 1.806203894 | 0.070886497 |
| TAS2R1 | ENST00000607876.1 | ENSG00000272848.1 | -0.905506397 | -2.063612279 | 0.039054492 |
| TAS2R1 | ENST00000608085.1 | ENSG00000231898.4 | -0.927649537 | -2.061463738 | 0.039258819 |
| TAS2R1 | ENST00000608476.1 | ENSG00000232675.3 | -0.868788618 | -1.950731346 | 0.051089012 |
| TAS2R1 | ENST00000609610.1 | ENSG00000232675.3 | -0.848946533 | -1.879880768 | 0.06012433 |
| TAS2R1 | ENST00000609701.1 | ENSG00000273284.1 | -0.873157964 | -1.920621301 | 0.054779468 |
| TAS2R1 | ENST00000609725.1 | ENSG00000231898.4 | -0.946673703 | -2.121900939 | 0.033846058 |
| TAS2R1 | ENST00000609972.1 | ENSG00000230651.3 | -0.919288123 | -2.04819528 | 0.040540869 |
| TAS2R1 | ENST00000610161.1 | ENSG00000273059.1 | -0.965901322 | -2.170825325 | 0.029944379 |
| TAS2R1 | NR_024410.1 | LINC00710 | -0.868436559 | -1.928706041 | 0.053767366 |
| TAS2R1 | NR_028324.1 | LINC01002 | -0.830016873 | -1.87106613 | 0.061335916 |
| TAS2R1 | NR_033371.1 | CDRT7 | -0.912294499 | -2.061763899 | 0.03923022 |
| TAS2R1 | NR_044996.1 | HCG23 | -0.905206554 | -2.060458289 | 0.039354749 |
| TAS2R1 | NR_110480.1 | LOC101927079 | -0.865904374 | -1.942796764 | 0.052040719 |
| TAS2R1 | NR_120335.1 | LOC101928414 | -0.947280822 | -2.104184782 | 0.035362331 |
| TAS2R1 | NR_120655.1 | KCNMA1-AS1 | -0.945250597 | -2.089271003 | 0.036683335 |
| TAS2R1 | NR_126166.1 | FAM74A7 | -0.973046657 | -2.140325216 | 0.032328494 |
| TAS2R1 | NR_134265.1 | LINC02103 | -0.837011092 | -1.895780033 | 0.057989137 |
| TAS2R1 | NR_134325.1 | LOC102723672 | -0.839877041 | -1.8560397 | 0.0634479 |
| TAS2R1 | NR_134597.1 | LOC105378068 | -0.919251571 | -2.055781527 | 0.039803579 |
| TAS2R1 | NR_135108.1 | LOC105369509 | -0.832481571 | -1.865508853 | 0.062110112 |
| TAS2R1 | NR_135258.1 | LOC105370489 | -0.908970737 | -2.083016074 | 0.037249758 |
| TAS2R1 | NR_138084.1 | HCG24 | -0.872155907 | -1.979395768 | 0.047771465 |
| TAS2R14 | ENST00000398777.3 | ENSG00000240152.2 | 0.860543407 | 1.914717022 | 0.055528602 |
| TAS2R14 | ENST00000413989.1 | ENSG00000242628.1 | 0.835479274 | 1.882369661 | 0.059785842 |
| TAS2R14 | ENST00000414377.1 | ENSG00000230470.1 | 0.900939043 | 2.025857113 | 0.042779429 |
| TAS2R14 | ENST00000416220.1 | ENSG00000236753.1 | 0.825495616 | 1.852038294 | 0.064020324 |
| TAS2R14 | ENST00000416641.1 | ENSG00000226956.1 | -0.803604769 | -1.797204086 | 0.072303226 |
| TAS2R14 | ENST00000417426.1 | ENSG00000233145.1 | 0.833476411 | 1.854493712 | 0.063668559 |
| TAS2R14 | ENST00000417782.1 | ENSG00000228587.1 | 0.86451364 | 1.935512311 | 0.052927459 |
| TAS2R14 | ENST00000418741.1 | ENSG00000227332.1 | 0.912047379 | 2.04187305 | 0.041164126 |
| TAS2R14 | ENST00000420830.1 | ENSG00000231512.1 | 0.809723791 | 1.806246343 | 0.070879869 |
| TAS2R14 | ENST00000421252.2 | ENSG00000250258.1 | -0.901326795 | -2.037658084 | 0.041584139 |
| TAS2R14 | ENST00000421498.1 | ENSG00000237978.1 | 0.8332407 | 1.873729892 | 0.060967664 |
| TAS2R14 | ENST00000425058.1 | ENSG00000226771.1 | 0.811425969 | 1.793723513 | 0.07285731 |
| TAS2R14 | ENST00000426213.1 | ENSG00000223660.1 | -0.910588953 | -2.041548737 | 0.041196315 |
| TAS2R14 | ENST00000427132.1 | ENSG00000232121.1 | -0.849056332 | -1.905046652 | 0.056774007 |
| TAS2R14 | ENST00000427524.1 | ENSG00000236065.2 | -0.907755303 | -2.032599089 | 0.042093044 |
| TAS2R14 | ENST00000429878.1 | ENSG00000224184.1 | -0.810656044 | -1.83047007 | 0.067179678 |
| TAS2R14 | ENST00000432431.1 | ENSG00000234940.1 | 0.820926625 | 1.82491502 | 0.068013854 |
| TAS2R14 | ENST00000434493.1 | ENSG00000224605.1 | 0.940052376 | 2.094825108 | 0.036186542 |

| | | | | | |
|---|---|---|---|---|---|
| TAS2R14 | ENST00000435271.1 | ENSG00000231132.1 | -0.936644691 | -2.084340126 | 0.03712924 |
| TAS2R14 | ENST00000435832.1 | ENSG00000229201.1 | 0.953662408 | 2.152487957 | 0.031358943 |
| TAS2R14 | ENST00000437680.1 | ENSG00000237133.1 | -0.904769015 | -2.020106604 | 0.043372331 |
| TAS2R14 | ENST00000439455.1 | ENSG00000233482.1 | -0.831625365 | -1.861427882 | 0.062683776 |
| TAS2R14 | ENST00000439529.1 | ENSG00000236526.1 | 0.864836626 | 1.962581262 | 0.04969485 |
| TAS2R14 | ENST00000440862.1 | ENSG00000223804.1 | -0.905807107 | -2.00338821 | 0.045135636 |
| TAS2R14 | ENST00000440947.1 | ENSG00000225472.1 | 0.831602679 | 1.859925912 | 0.062896009 |
| TAS2R14 | ENST00000441809.2 | ENSG00000237445.2 | -0.819019984 | -1.850165946 | 0.064289635 |
| TAS2R14 | ENST00000442579.1 | ENSG00000228719.1 | -0.903822221 | -2.008271233 | 0.044614477 |
| TAS2R14 | ENST00000444245.1 | ENSG00000236753.1 | 0.811399328 | 1.786857061 | 0.073960594 |
| TAS2R14 | ENST00000445617.2 | ENSG00000225751.2 | 0.881826132 | 1.964514421 | 0.049470466 |
| TAS2R14 | ENST00000447183.2 | ENSG00000271593.1 | 0.862868272 | 1.922635474 | 0.054525846 |
| TAS2R14 | ENST00000448001.1 | ENSG00000229639.1 | 0.892164797 | 1.995068068 | 0.046035456 |
| TAS2R14 | ENST00000448431.1 | ENSG00000232548.1 | 0.9252699 | 2.059316222 | 0.039463955 |
| TAS2R14 | ENST00000448674.1 | ENSG00000235119.1 | -0.878813233 | -2.00237036 | 0.045244914 |
| TAS2R14 | ENST00000450206.1 | ENSG00000234311.1 | -0.992198878 | -2.224922968 | 0.02608641 |
| TAS2R14 | ENST00000450365.1 | ENSG00000224404.1 | 0.926321606 | 2.08488318 | 0.037079906 |
| TAS2R14 | ENST00000450848.1 | ENSG00000225539.1 | 0.837224237 | 1.885264788 | 0.059394098 |
| TAS2R14 | ENST00000451267.1 | ENSG00000230410.1 | -0.84715413 | -1.912660125 | 0.055791577 |
| TAS2R14 | ENST00000451575.2 | ENSG00000224251.2 | 0.832945059 | 1.850376272 | 0.064259336 |
| TAS2R14 | ENST00000452553.1 | ENSG00000233973.1 | 0.900563985 | 2.017809414 | 0.043611114 |
| TAS2R14 | ENST00000453878.1 | ENSG00000224850.1 | 0.980367026 | 2.211074349 | 0.027030689 |
| TAS2R14 | ENST00000455416.1 | ENSG00000229337.1 | -0.930020299 | -2.065056053 | 0.038917697 |
| TAS2R14 | ENST00000457169.1 | ENSG00000232408.1 | 0.895204924 | 1.995877516 | 0.045947257 |
| TAS2R14 | ENST00000457632.1 | ENSG00000234248.1 | 0.896297595 | 2.01103278 | 0.044321994 |
| TAS2R14 | ENST00000482142.1 | ENSG00000243276.1 | 0.82771146 | 1.831543038 | 0.06701953 |
| TAS2R14 | ENST00000485347.1 | ENSG00000239991.1 | -0.801876046 | -1.775677113 | 0.075786152 |
| TAS2R14 | ENST00000488040.1 | ENSG00000243176.1 | 0.801169174 | 1.783987613 | 0.074425678 |
| TAS2R14 | ENST00000503938.1 | ENSG00000246095.2 | 0.811098865 | 1.814049265 | 0.069670142 |
| TAS2R14 | ENST00000504017.1 | ENSG00000248388.1 | 0.810254556 | 1.809702849 | 0.07034188 |
| TAS2R14 | ENST00000504344.1 | ENSG00000251438.1 | 0.889775167 | 1.962577115 | 0.049695332 |
| TAS2R14 | ENST00000505575.1 | ENSG00000248939.1 | -0.86701283 | -1.933308311 | 0.053198229 |
| TAS2R14 | ENST00000506058.1 | ENSG00000248261.1 | -0.897554258 | -2.02338233 | 0.043033744 |
| TAS2R14 | ENST00000506059.1 | ENSG00000248311.1 | 0.923546811 | 2.082591189 | 0.037288503 |
| TAS2R14 | ENST00000506379.1 | ENSG00000240152.2 | 0.825886638 | 1.861413751 | 0.06268577 |
| TAS2R14 | ENST00000506852.1 | ENSG00000250945.1 | 0.858010043 | 1.911416931 | 0.055951023 |
| TAS2R14 | ENST00000508845.1 | ENSG00000271724.1 | 0.912499166 | 2.042083882 | 0.041143211 |
| TAS2R14 | ENST00000509098.1 | ENSG00000250863.1 | 0.869509355 | 1.952265935 | 0.05090664 |
| TAS2R14 | ENST00000509983.1 | ENSG00000248173.1 | -0.810190797 | -1.798481537 | 0.072100732 |
| TAS2R14 | ENST00000511603.1 | ENSG00000249892.1 | 0.853811063 | 1.902088291 | 0.057159613 |
| TAS2R14 | ENST00000511631.1 | ENSG00000250402.1 | -0.842079139 | -1.863289052 | 0.06242161 |
| TAS2R14 | ENST00000511917.1 | ENSG00000250062.1 | 0.848169188 | 1.909194524 | 0.056237002 |
| TAS2R14 | ENST00000512036.1 | ENSG00000250993.1 | 0.813814683 | 1.82595618 | 0.067856863 |
| TAS2R14 | ENST00000513023.1 | ENSG00000248809.1 | 0.895076816 | 1.989829918 | 0.046609675 |
| TAS2R14 | ENST00000513179.1 | ENSG00000251580.1 | 0.813860789 | 1.82331471 | 0.06825574 |
| TAS2R14 | ENST00000514459.1 | ENSG00000248211.1 | -0.814263912 | -1.806968828 | 0.07076714 |
| TAS2R14 | ENST00000514802.1 | ENSG00000250190.1 | 0.967621248 | 2.16381555 | 0.030478508 |
| TAS2R14 | ENST00000517716.1 | ENSG00000253515.1 | -0.819505695 | -1.834250134 | 0.066616873 |
| TAS2R14 | ENST00000518416.1 | ENSG00000253901.1 | 0.934933995 | 2.10124029 | 0.035619878 |
| TAS2R14 | ENST00000518620.1 | ENSG00000253892.1 | -0.861531628 | -1.929993893 | 0.053607594 |
| TAS2R14 | ENST00000519005.1 | ENSG00000253507.1 | 0.900770817 | 1.986912288 | 0.046932118 |

| | | | | | |
|---|---|---|---|---|---|
| TAS2R14 | ENST00000519375.1 | ENSG00000253980.1 | 0.847814481 | 1.888667519 | 0.058936394 |
| TAS2R14 | ENST00000519660.1 | ENSG00000253416.1 | 0.917898909 | 2.061637742 | 0.039242238 |
| TAS2R14 | ENST00000519695.1 | ENSG00000253507.1 | 0.942465551 | 2.094941482 | 0.036176195 |
| TAS2R14 | ENST00000521378.1 | ENSG00000254222.1 | -0.903936535 | -2.027097006 | 0.042652493 |
| TAS2R14 | ENST00000521490.1 | ENSG00000253407.1 | -0.914560584 | -2.035670134 | 0.04178349 |
| TAS2R14 | ENST00000522408.1 | ENSG00000253484.1 | 0.883125407 | 1.976444258 | 0.048104483 |
| TAS2R14 | ENST00000523806.1 | ENSG00000253616.1 | -0.810842929 | -1.823872646 | 0.068171328 |
| TAS2R14 | ENST00000524133.1 | ENSG00000253174.2 | -0.898093295 | -2.006288427 | 0.044825484 |
| TAS2R14 | ENST00000524275.1 | ENSG00000253507.1 | 0.952850751 | 2.131001771 | 0.033088996 |
| TAS2R14 | ENST00000527579.1 | ENSG00000254574.1 | -0.858465675 | -1.889899446 | 0.05877141 |
| TAS2R14 | ENST00000527727.1 | ENSG00000255227.1 | 0.889080109 | 2.006882878 | 0.044762135 |
| TAS2R14 | ENST00000529837.1 | ENSG00000254687.1 | 0.830966201 | 1.854728285 | 0.063635037 |
| TAS2R14 | ENST00000529875.1 | ENSG00000254404.1 | -0.970686232 | -2.184409175 | 0.028932195 |
| TAS2R14 | ENST00000533938.1 | ENSG00000255142.1 | -0.872813746 | -1.970649637 | 0.048763965 |
| TAS2R14 | ENST00000535315.1 | ENSG00000250748.2 | 0.80151604 | 1.779474544 | 0.075161995 |
| TAS2R14 | ENST00000535324.1 | ENSG00000255968.1 | 0.859849169 | 1.935115667 | 0.052976103 |
| TAS2R14 | ENST00000536529.1 | ENSG00000256422.1 | 0.973194952 | 2.180498076 | 0.029220561 |
| TAS2R14 | ENST00000538294.1 | ENSG00000250748.2 | -0.8268753 | -1.838653012 | 0.065966239 |
| TAS2R14 | ENST00000538641.1 | ENSG00000256422.1 | 0.845965086 | 1.882885821 | 0.059715843 |
| TAS2R14 | ENST00000539313.1 | ENSG00000256588.1 | 0.802367684 | 1.760904884 | 0.078254503 |
| TAS2R14 | ENST00000548748.1 | ENSG00000258252.1 | -0.844402073 | -1.887526899 | 0.059089492 |
| TAS2R14 | ENST00000549329.1 | ENSG00000224189.2 | 0.939048488 | 2.076589916 | 0.037839424 |
| TAS2R14 | ENST00000549616.1 | ENSG00000258168.1 | 0.869308351 | 1.940385219 | 0.052332892 |
| TAS2R14 | ENST00000549756.1 | ENSG00000257769.1 | -0.917324131 | -2.063461792 | 0.039068774 |
| TAS2R14 | ENST00000550805.1 | ENSG00000244306.5 | -0.893026928 | -1.993848952 | 0.046168564 |
| TAS2R14 | ENST00000552156.1 | ENSG00000224189.2 | 0.898984877 | 2.011466198 | 0.044276237 |
| TAS2R14 | ENST00000553477.1 | ENSG00000259123.1 | 0.902644062 | 2.029823919 | 0.042374441 |
| TAS2R14 | ENST00000555636.1 | ENSG00000259072.1 | 0.909892472 | 2.024408884 | 0.042928098 |
| TAS2R14 | ENST00000555913.1 | ENSG00000259077.1 | 0.840572385 | 1.8860133 | 0.059293162 |
| TAS2R14 | ENST00000556458.1 | ENSG00000258504.2 | -0.964539063 | -2.152559773 | 0.031353293 |
| TAS2R14 | ENST00000557903.1 | ENSG00000259182.1 | 0.858791929 | 1.934833056 | 0.053010785 |
| TAS2R14 | ENST00000557965.1 | ENSG00000259681.1 | 0.830844744 | 1.858928626 | 0.063037256 |
| TAS2R14 | ENST00000558141.1 | ENSG00000259594.1 | 0.82754664 | 1.863758114 | 0.06235568 |
| TAS2R14 | ENST00000558221.1 | ENSG00000259704.1 | -0.899626187 | -2.037422698 | 0.041607701 |
| TAS2R14 | ENST00000560268.1 | ENSG00000259287.1 | -0.83614842 | -1.882030278 | 0.059831905 |
| TAS2R14 | ENST00000560586.1 | ENSG00000259534.1 | 0.808393239 | 1.813003171 | 0.069831332 |
| TAS2R14 | ENST00000563601.1 | ENSG00000260589.1 | -0.824676454 | -1.832863925 | 0.06682281 |
| TAS2R14 | ENST00000565271.1 | ENSG00000261335.1 | -0.892131936 | -1.967992951 | 0.049068849 |
| TAS2R14 | ENST00000565623.1 | ENSG00000261118.1 | -0.804711106 | -1.780714938 | 0.074959032 |
| TAS2R14 | ENST00000569849.1 | ENSG00000260640.1 | 0.803301155 | 1.796702657 | 0.072382837 |
| TAS2R14 | ENST00000572417.1 | ENSG00000263171.1 | 0.933420304 | 2.086820223 | 0.036904388 |
| TAS2R14 | ENST00000578035.1 | ENSG00000266743.1 | -0.856997732 | -1.90506007 | 0.056772263 |
| TAS2R14 | ENST00000581996.1 | ENSG00000265778.1 | 0.88324357 | 1.981122067 | 0.047577586 |
| TAS2R14 | ENST00000588177.1 | ENSG00000234899.5 | 0.983197481 | 2.220782828 | 0.026365674 |
| TAS2R14 | ENST00000591621.1 | ENSG00000232116.2 | 0.817718488 | 1.81773829 | 0.069104142 |
| TAS2R14 | ENST00000592431.1 | ENSG00000267475.1 | -0.951702686 | -2.154817723 | 0.031176101 |
| TAS2R14 | ENST00000592523.1 | ENSG00000226994.3 | 0.831875175 | 1.855271144 | 0.063557517 |
| TAS2R14 | ENST00000594589.1 | ENSG00000269321.1 | -0.90664901 | -2.05659957 | 0.03972476 |
| TAS2R14 | ENST00000594762.1 | ENSG00000231898.4 | 0.986047428 | 2.184595523 | 0.028918516 |
| TAS2R14 | ENST00000595409.1 | ENSG00000232729.3 | 0.910896649 | 2.02858912 | 0.042500158 |
| TAS2R14 | ENST00000595737.1 | ENSG00000228065.6 | 0.912156116 | 2.047923192 | 0.040567526 |

| | | | | | |
|---|---|---|---|---|---|
| TAS2R14 | ENST00000595892.1 | ENSG00000269640.1 | 0.819981046 | 1.83585977 | 0.066378399 |
| TAS2R14 | ENST00000595972.1 | ENSG00000230333.2 | 0.92179365 | 2.067204452 | 0.038714893 |
| TAS2R14 | ENST00000598356.1 | ENSG00000269640.1 | 0.849848524 | 1.913385044 | 0.055698778 |
| TAS2R14 | ENST00000598950.1 | ENSG00000269736.1 | 0.856799513 | 1.92591811 | 0.0541146 |
| TAS2R14 | ENST00000601420.1 | ENSG00000269560.1 | -0.896316966 | -1.983067911 | 0.047359844 |
| TAS2R14 | ENST00000602507.1 | ENSG00000270069.1 | 0.859697958 | 1.916424317 | 0.055311108 |
| TAS2R14 | ENST00000602592.1 | ENSG00000270049.1 | 0.906547743 | 2.041555009 | 0.041195692 |
| TAS2R14 | ENST00000602790.1 | ENSG00000270000.1 | -0.993178807 | -2.241670137 | 0.024982699 |
| TAS2R14 | ENST00000602835.1 | ENSG00000270096.1 | 0.949267755 | 2.128915678 | 0.033261237 |
| TAS2R14 | ENST00000602954.1 | ENSG00000269906.1 | 0.804220082 | 1.824802602 | 0.068030823 |
| TAS2R14 | ENST00000605021.1 | ENSG00000271401.1 | 0.864650284 | 1.930080052 | 0.05359692 |
| TAS2R14 | ENST00000606048.1 | ENSG00000272343.1 | 0.811643152 | 1.828292598 | 0.06750565 |
| TAS2R14 | ENST00000606457.1 | ENSG00000271830.1 | 0.831297233 | 1.865270467 | 0.062143503 |
| TAS2R14 | ENST00000606898.1 | ENSG00000272094.1 | -0.924350591 | -2.064840187 | 0.038938124 |
| TAS2R14 | ENST00000607594.1 | ENSG00000271766.1 | 0.94890661 | 2.131945004 | 0.033011368 |
| TAS2R14 | ENST00000607769.1 | ENSG00000272438.1 | -0.943568912 | -2.091439575 | 0.036488678 |
| TAS2R14 | ENST00000608289.1 | ENSG00000272958.1 | -0.886042621 | -1.998253869 | 0.045689144 |
| TAS2R14 | ENST00000608422.1 | ENSG00000272866.1 | 0.80024315 | 1.783126755 | 0.074565672 |
| TAS2R14 | ENST00000609270.1 | ENSG00000273073.1 | 0.804137031 | 1.788412965 | 0.073709406 |
| TAS2R14 | ENST00000609701.1 | ENSG00000273284.1 | 0.821584746 | 1.827061265 | 0.067690558 |
| TAS2R14 | ENST00000609775.1 | ENSG00000273232.1 | 0.814349538 | 1.80811183 | 0.070589097 |
| TAS2R14 | ENST00000609890.1 | ENSG00000231898.4 | 0.878724339 | 1.961672843 | 0.049800586 |
| TAS2R14 | ENST00000609952.1 | ENSG00000233766.3 | 0.879709358 | 1.970269197 | 0.048807527 |
| TAS2R14 | NR_022011.1 | PWARSN | 0.843071394 | 1.89749049 | 0.057763233 |
| TAS2R14 | NR_024410.1 | LINC00710 | 0.823939317 | 1.859841655 | 0.062907932 |
| TAS2R14 | NR_026932.1 | PDCD4-AS1 | -0.937046293 | -2.113990772 | 0.034516055 |
| TAS2R14 | NR_040049.1 | SDCBP2-AS1 | -0.842333087 | -1.863174027 | 0.062437786 |
| TAS2R14 | NR_046845.1 | DNM3-IT1 | 0.863019527 | 1.9193464 | 0.05494051 |
| TAS2R14 | NR_102703.1 | MAGEA8-AS1 | 0.850355035 | 1.884991159 | 0.059431031 |
| TAS2R14 | NR_104620.1 | LINC01672 | 0.824171941 | 1.842158375 | 0.065451987 |
| TAS2R14 | NR_104998.1 | LOC102467225 | 0.80937805 | 1.783587496 | 0.074490719 |
| TAS2R14 | NR_108046.1 | LINC00844 | 0.867772111 | 1.962057135 | 0.049755833 |
| TAS2R14 | NR_110370.1 | STAM-AS1 | -0.879486921 | -1.936123051 | 0.052852632 |
| TAS2R14 | NR_110480.1 | LOC101927079 | 0.804219492 | 1.784526903 | 0.074338087 |
| TAS2R14 | NR_110803.1 | LOC101927018 | -0.869941701 | -1.9301297 | 0.053590769 |
| TAS2R14 | NR_110824.1 | LINC01986 | 0.861984915 | 1.889305398 | 0.058850919 |
| TAS2R14 | NR_110879.1 | LOC101929064 | 0.835741381 | 1.849835426 | 0.064337273 |
| TAS2R14 | NR_120466.1 | LINC01489 | 0.855534592 | 1.901873382 | 0.05718771 |
| TAS2R14 | NR_126041.1 | LOC101930071 | -0.835645546 | -1.880062968 | 0.060099497 |
| TAS2R14 | NR_130916.1 | LOC105274304 | -0.901466806 | -2.006827623 | 0.044768021 |
| TAS2R14 | NR_131243.1 | SMCR2 | -0.803479805 | -1.78346711 | 0.074510297 |
| TAS2R14 | NR_134597.1 | LOC105378068 | 0.809889394 | 1.805520607 | 0.070993254 |
| TAS2R14 | NR_134632.1 | LOC105373051 | -0.897622157 | -2.015734636 | 0.043827732 |
| TAS2R14 | NR_134665.1 | LOC105374366 | 0.805995113 | 1.791649558 | 0.073189119 |
| TAS2R14 | NR_135549.1 | LOC101929411 | 0.841622859 | 1.891257463 | 0.058589984 |
| TAS2R14 | NR_135840.1 | LOC105376114 | 0.815551241 | 1.837268723 | 0.066170235 |
| TAS2R14 | NR_136320.1 | LOC105373656 | -0.869617139 | -1.971238402 | 0.048696613 |
| TAS2R40 | ENST00000390540.2 | ENSG00000254140.1 | 0.852187906 | 1.869047705 | 0.061616178 |
| TAS2R40 | ENST00000412647.2 | ENSG00000232964.2 | -0.925428538 | -2.067183761 | 0.038716842 |
| TAS2R40 | ENST00000413311.1 | ENSG00000226276.1 | -0.905716078 | -2.032268035 | 0.042126529 |
| TAS2R40 | ENST00000416220.1 | ENSG00000236753.1 | -0.825076256 | -1.85792101 | 0.063180232 |

| | | | | | |
|---|---|---|---|---|---|
| TAS2R40 | ENST00000422763.1 | ENSG00000231131.2 | -0.810428574 | -1.816333188 | 0.069319277 |
| TAS2R40 | ENST00000426213.1 | ENSG00000223660.1 | 0.850289263 | 1.906661846 | 0.056564391 |
| TAS2R40 | ENST00000426475.1 | ENSG00000239467.1 | 0.860530546 | 1.932652234 | 0.053279053 |
| TAS2R40 | ENST00000431727.2 | ENSG00000234938.2 | 0.832050294 | 1.859764742 | 0.062918818 |
| TAS2R40 | ENST00000432431.1 | ENSG00000234940.1 | -0.944844424 | -2.086863845 | 0.036900444 |
| TAS2R40 | ENST00000432699.1 | ENSG00000233334.2 | 0.843076883 | 1.907757449 | 0.056422573 |
| TAS2R40 | ENST00000435832.1 | ENSG00000229201.1 | -0.857672827 | -1.920126895 | 0.054841873 |
| TAS2R40 | ENST00000437680.1 | ENSG00000237133.1 | 0.877460986 | 1.954081408 | 0.05069159 |
| TAS2R40 | ENST00000439455.1 | ENSG00000233482.1 | 0.803354511 | 1.804511756 | 0.071151118 |
| TAS2R40 | ENST00000441866.1 | ENSG00000236743.1 | -0.862060124 | -1.93503273 | 0.052986279 |
| TAS2R40 | ENST00000448570.1 | ENSG00000224549.1 | 0.874174808 | 1.964459893 | 0.049476783 |
| TAS2R40 | ENST00000448674.1 | ENSG00000235119.1 | 0.808620774 | 1.809793483 | 0.070327819 |
| TAS2R40 | ENST00000450365.1 | ENSG00000224404.1 | -0.822563492 | -1.847805267 | 0.064630519 |
| TAS2R40 | ENST00000451575.2 | ENSG00000224251.2 | -0.894510019 | -1.984435735 | 0.047207286 |
| TAS2R40 | ENST00000452553.1 | ENSG00000233973.1 | -0.827664738 | -1.843463536 | 0.065261361 |
| TAS2R40 | ENST00000454262.2 | ENSG00000229923.2 | -0.838410751 | -1.844915967 | 0.065049764 |
| TAS2R40 | ENST00000455416.1 | ENSG00000229337.1 | 0.916735535 | 2.049317067 | 0.040431121 |
| TAS2R40 | ENST00000504017.1 | ENSG00000248388.1 | -0.89193311 | -2.015222604 | 0.04388133 |
| TAS2R40 | ENST00000506058.1 | ENSG00000248261.1 | 0.924645261 | 2.050294857 | 0.040335666 |
| TAS2R40 | ENST00000508081.1 | ENSG00000248254.1 | 0.915892728 | 2.063420931 | 0.039072653 |
| TAS2R40 | ENST00000508936.1 | ENSG00000250582.1 | -0.897257727 | -2.011078364 | 0.04431718 |
| TAS2R40 | ENST00000511631.1 | ENSG00000250402.1 | 0.825619872 | 1.851414895 | 0.064109888 |
| TAS2R40 | ENST00000513023.1 | ENSG00000248809.1 | -0.9670468 | -2.192462489 | 0.028346129 |
| TAS2R40 | ENST00000514802.1 | ENSG00000250190.1 | -0.915347662 | -2.054442893 | 0.039932845 |
| TAS2R40 | ENST00000518416.1 | ENSG00000253901.1 | -0.839595018 | -1.886686286 | 0.059202533 |
| TAS2R40 | ENST00000523806.1 | ENSG00000253616.1 | 0.832247302 | 1.860223482 | 0.062853914 |
| TAS2R40 | ENST00000527100.1 | ENSG00000255015.1 | -0.805449826 | -1.797422007 | 0.07226865 |
| TAS2R40 | ENST00000529160.1 | ENSG00000246790.2 | 0.931477534 | 2.072088858 | 0.038257155 |
| TAS2R40 | ENST00000535315.1 | ENSG00000250748.2 | -0.917647214 | -2.033978367 | 0.041953777 |
| TAS2R40 | ENST00000536529.1 | ENSG00000256422.1 | -0.830213506 | -1.8414659 | 0.065553313 |
| TAS2R40 | ENST00000545593.1 | ENSG00000256972.1 | -0.848840394 | -1.880045774 | 0.06010184 |
| TAS2R40 | ENST00000557368.1 | ENSG00000258444.1 | 0.846207651 | 1.890216472 | 0.058729015 |
| TAS2R40 | ENST00000560586.1 | ENSG00000259534.1 | -0.821179869 | -1.848676186 | 0.064504584 |
| TAS2R40 | ENST00000561653.1 | ENSG00000260095.1 | 0.94291728 | 2.120503372 | 0.033963617 |
| TAS2R40 | ENST00000572417.1 | ENSG00000263171.1 | -0.814418691 | -1.811002012 | 0.07014054 |
| TAS2R40 | ENST00000578035.1 | ENSG00000266743.1 | 0.834240793 | 1.88375124 | 0.059598632 |
| TAS2R40 | ENST00000579154.1 | ENSG00000265908.1 | -0.803486471 | -1.80489139 | 0.071091679 |
| TAS2R40 | ENST00000580622.1 | ENSG00000264634.1 | 0.817137241 | 1.835490561 | 0.066433036 |
| TAS2R40 | ENST00000586952.1 | ENSG00000226994.3 | -0.849637135 | -1.867531275 | 0.061827434 |
| TAS2R40 | ENST00000588182.2 | ENSG00000267453.2 | 0.824528868 | 1.859222802 | 0.062995564 |
| TAS2R40 | ENST00000588384.1 | ENSG00000236172.2 | 0.814580669 | 1.823074668 | 0.068292083 |
| TAS2R40 | ENST00000589380.1 | ENSG00000267488.1 | 0.914435638 | 2.020643181 | 0.043316716 |
| TAS2R40 | ENST00000592498.1 | ENSG00000267488.1 | 0.948248983 | 2.110511573 | 0.034814315 |
| TAS2R40 | ENST00000592525.1 | ENSG00000267214.1 | 0.80360249 | 1.784526615 | 0.074338134 |
| TAS2R40 | ENST00000593139.1 | ENSG00000267042.1 | 0.824696838 | 1.836805998 | 0.06623854 |
| TAS2R40 | ENST00000594850.1 | ENSG00000268093.1 | 0.8729415 | 1.967661804 | 0.049106963 |
| TAS2R40 | ENST00000595409.1 | ENSG00000232729.3 | -0.849972404 | -1.902307316 | 0.05713099 |
| TAS2R40 | ENST00000600242.1 | ENSG00000269583.1 | 0.80386044 | 1.79088547 | 0.073311676 |
| TAS2R40 | ENST00000602435.1 | ENSG00000269952.1 | -0.950409371 | -2.100552318 | 0.035680283 |
| TAS2R40 | ENST00000602507.1 | ENSG00000270069.1 | -0.916022827 | -2.050744104 | 0.040291874 |
| TAS2R40 | ENST00000605780.1 | ENSG00000270755.1 | 0.866279038 | 1.938643837 | 0.052544722 |

| | | | | | |
|---|---|---|---|---|---|
| TAS2R40 | ENST00000606048.1 | ENSG00000272343.1 | -0.911267608 | -2.044476663 | 0.040906481 |
| TAS2R40 | ENST00000607769.1 | ENSG00000272438.1 | 0.817386622 | 1.817300375 | 0.069171133 |
| TAS2R40 | ENST00000609270.1 | ENSG00000273073.1 | -0.847439758 | -1.879242685 | 0.060211364 |
| TAS2R40 | ENST00000609775.1 | ENSG00000273232.1 | -0.807853351 | -1.80002385 | 0.071856872 |
| TAS2R40 | ENST00000609934.1 | ENSG00000273271.1 | -0.970245434 | -2.154169174 | 0.031226907 |
| TAS2R40 | ENST00000609952.1 | ENSG00000233766.3 | -0.936611487 | -2.081780814 | 0.037362495 |
| TAS2R40 | NR_026813.1 | LINC00597 | -0.81971013 | -1.809207323 | 0.0704188 |
| TAS2R40 | NR_040049.1 | SDCBP2-AS1 | 0.813433462 | 1.795474483 | 0.072578135 |
| TAS2R40 | NR_040061.1 | SRP14-AS1 | 0.8263554 | 1.839258181 | 0.065877221 |
| TAS2R40 | NR_046224.1 | LINC00659 | 0.9135674 | 2.038055256 | 0.041544407 |
| TAS2R40 | NR_103776.1 | CHRM3-AS2 | -0.880313682 | -1.965302861 | 0.049379195 |
| TAS2R40 | NR_108046.1 | LINC00844 | -0.858794887 | -1.930354915 | 0.053562877 |
| TAS2R40 | NR_108077.1 | SMAD1-AS2 | -0.894721145 | -2.001018385 | 0.045390409 |
| TAS2R40 | NR_108106.1 | LINC01135 | 0.826975985 | 1.837216213 | 0.066177983 |
| TAS2R40 | NR_109831.1 | RASSF1-AS1 | 0.824813635 | 1.841760768 | 0.065510151 |
| TAS2R40 | NR_110803.1 | LOC101927018 | 0.869567842 | 1.954937502 | 0.050590447 |
| TAS2R40 | NR_110824.1 | LINC01986 | -0.871781848 | -1.971039028 | 0.048719412 |
| TAS2R40 | NR_120502.1 | JARID2-AS1 | 0.807932657 | 1.796518588 | 0.072412079 |
| TAS2R40 | NR_121624.1 | LOC103352541 | -0.822868927 | -1.837707169 | 0.066105567 |
| TAS2R40 | NR_135108.1 | LOC105369509 | -0.846174548 | -1.887026149 | 0.059156809 |
| TAS2R40 | NR_136215.1 | VCAN-AS1 | -0.86148502 | -1.935715264 | 0.052902584 |
| TAS2R40 | NR_136320.1 | LOC105373656 | 0.918347368 | 2.046529759 | 0.040704278 |
| TAS2R43 | ENST00000411804.1 | ENSG00000227415.1 | 0.8672037 | 1.973122469 | 0.048481611 |
| TAS2R43 | ENST00000414377.1 | ENSG00000230470.1 | 0.819950879 | 1.832374964 | 0.066895575 |
| TAS2R43 | ENST00000420830.1 | ENSG00000231512.1 | 0.832602442 | 1.850780926 | 0.064201076 |
| TAS2R43 | ENST00000421737.1 | ENSG00000232316.1 | 0.843728873 | 1.865678736 | 0.062086326 |
| TAS2R43 | ENST00000427524.1 | ENSG00000236065.2 | -0.840581402 | -1.869817916 | 0.061509108 |
| TAS2R43 | ENST00000428765.1 | ENSG00000230107.1 | -0.847635673 | -1.930926159 | 0.053492185 |
| TAS2R43 | ENST00000430534.1 | ENSG00000229297.1 | -0.903077968 | -2.00164346 | 0.045323091 |
| TAS2R43 | ENST00000430728.1 | ENSG00000232316.1 | 0.89409548 | 1.993778452 | 0.046176271 |
| TAS2R43 | ENST00000433174.1 | ENSG00000162947.4 | 0.83139258 | 1.842374489 | 0.065420391 |
| TAS2R43 | ENST00000435315.2 | ENSG00000226751.2 | 0.831154082 | 1.830399214 | 0.067190265 |
| TAS2R43 | ENST00000435832.1 | ENSG00000229201.1 | 0.828177152 | 1.855778745 | 0.063485101 |
| TAS2R43 | ENST00000440038.2 | ENSG00000237094.7 | -0.902092254 | -2.013354578 | 0.04407734 |
| TAS2R43 | ENST00000448431.1 | ENSG00000232548.1 | 0.815250343 | 1.836459653 | 0.066289704 |
| TAS2R43 | ENST00000451556.2 | ENSG00000228386.2 | 0.835882089 | 1.888282032 | 0.058988098 |
| TAS2R43 | ENST00000451575.2 | ENSG00000224251.2 | 0.818455341 | 1.848782519 | 0.064489223 |
| TAS2R43 | ENST00000468165.1 | ENSG00000239480.1 | -0.855714869 | -1.888731347 | 0.058927836 |
| TAS2R43 | ENST00000504301.1 | ENSG00000250696.1 | 0.85488477 | 1.89040978 | 0.058703176 |
| TAS2R43 | ENST00000508191.1 | ENSG00000250910.3 | 0.821874133 | 1.832836461 | 0.066826895 |
| TAS2R43 | ENST00000515789.1 | ENSG00000248571.1 | -0.872798766 | -1.950759647 | 0.051085644 |
| TAS2R43 | ENST00000521294.1 | ENSG00000253664.1 | -0.930212707 | -2.067920128 | 0.038647534 |
| TAS2R43 | ENST00000522300.1 | ENSG00000249484.4 | 0.815374683 | 1.825869363 | 0.067869942 |
| TAS2R43 | ENST00000523806.1 | ENSG00000253616.1 | -0.890113789 | -1.965011092 | 0.049412954 |
| TAS2R43 | ENST00000523935.1 | ENSG00000253567.1 | 0.836616038 | 1.863539437 | 0.062386409 |
| TAS2R43 | ENST00000524942.1 | ENSG00000255553.1 | -0.818013423 | -1.822274554 | 0.068413337 |
| TAS2R43 | ENST00000527086.1 | ENSG00000255182.1 | -0.848960376 | -1.918780701 | 0.055012093 |
| TAS2R43 | ENST00000527274.2 | ENSG00000255517.2 | -0.844740122 | -1.899877291 | 0.057449225 |
| TAS2R43 | ENST00000527727.1 | ENSG00000255227.1 | 0.843311652 | 1.884380344 | 0.059513547 |
| TAS2R43 | ENST00000535315.1 | ENSG00000250748.2 | 0.879957153 | 1.982136214 | 0.047463997 |
| TAS2R43 | ENST00000538641.1 | ENSG00000256422.1 | 0.893170172 | 2.012471021 | 0.044170308 |

| | | | | | |
|---|---|---|---|---|---|
| TAS2R43 | ENST00000541391.1 | ENSG00000256268.1 | 0.868994188 | 1.937847565 | 0.052641823 |
| TAS2R43 | ENST00000547207.1 | ENSG00000224189.2 | 0.804125746 | 1.7727894 | 0.076263611 |
| TAS2R43 | ENST00000548210.1 | ENSG00000257784.1 | -0.840363853 | -1.876967135 | 0.060522598 |
| TAS2R43 | ENST00000551135.1 | ENSG00000258294.1 | 0.897369472 | 2.0185582 | 0.043533159 |
| TAS2R43 | ENST00000552261.1 | ENSG00000257959.1 | 0.827529625 | 1.847947786 | 0.064609897 |
| TAS2R43 | ENST00000552541.1 | ENSG00000258294.1 | 0.885053449 | 1.986941094 | 0.046928925 |
| TAS2R43 | ENST00000554138.1 | ENSG00000258882.1 | 0.836400831 | 1.876844281 | 0.060539439 |
| TAS2R43 | ENST00000558575.1 | ENSG00000259687.1 | -0.855441334 | -1.886772931 | 0.059190874 |
| TAS2R43 | ENST00000563855.1 | ENSG00000260658.1 | 0.889062358 | 1.980896753 | 0.047602853 |
| TAS2R43 | ENST00000565055.1 | ENSG00000259912.1 | -0.864352464 | -1.949386118 | 0.051249331 |
| TAS2R43 | ENST00000565722.1 | ENSG00000245768.2 | 0.84108967 | 1.885967292 | 0.059299362 |
| TAS2R43 | ENST00000565735.1 | ENSG00000261213.1 | 0.820957238 | 1.834335828 | 0.066604159 |
| TAS2R43 | ENST00000568243.1 | ENSG00000261521.1 | 0.908914693 | 2.007018021 | 0.044747744 |
| TAS2R43 | ENST00000569025.1 | ENSG00000246379.2 | 0.891187876 | 1.988897569 | 0.04671251 |
| TAS2R43 | ENST00000569313.1 | ENSG00000261604.1 | 0.899958518 | 1.997029553 | 0.045821973 |
| TAS2R43 | ENST00000569849.1 | ENSG00000260640.1 | 0.813006236 | 1.825466041 | 0.067930731 |
| TAS2R43 | ENST00000579775.1 | ENSG00000264108.1 | -0.808706244 | -1.800066251 | 0.071850178 |
| TAS2R43 | ENST00000586952.1 | ENSG00000226994.3 | 0.804047311 | 1.804505013 | 0.071152174 |
| TAS2R43 | ENST00000589673.1 | ENSG00000267755.1 | -0.873285013 | -1.926073542 | 0.054095192 |
| TAS2R43 | ENST00000591469.1 | ENSG00000267374.1 | 0.842896984 | 1.853987627 | 0.06374093 |
| TAS2R43 | ENST00000592413.1 | ENSG00000266933.1 | -0.945247167 | -2.106596465 | 0.035152573 |
| TAS2R43 | ENST00000592518.1 | ENSG00000267786.1 | -0.834166713 | -1.858365241 | 0.063117165 |
| TAS2R43 | ENST00000593175.1 | ENSG00000229036.3 | 0.930115368 | 2.068638411 | 0.038580031 |
| TAS2R43 | ENST00000594589.1 | ENSG00000269321.1 | -0.843318 | -1.889749865 | 0.058791422 |
| TAS2R43 | ENST00000602736.1 | ENSG00000269976.1 | 0.82957494 | 1.857975614 | 0.063172477 |
| TAS2R43 | ENST00000606855.1 | ENSG00000245937.3 | -0.939856727 | -2.103484478 | 0.035423441 |
| TAS2R43 | ENST00000606869.1 | ENSG00000272349.1 | 0.816348047 | 1.820421291 | 0.068694873 |
| TAS2R43 | ENST00000607052.1 | ENSG00000271870.1 | 0.84302669 | 1.875484382 | 0.060726116 |
| TAS2R43 | ENST00000608943.1 | ENSG00000273368.1 | 0.989923671 | 2.228798924 | 0.025827287 |
| TAS2R43 | ENST00000609934.1 | ENSG00000273271.1 | 0.809707104 | 1.827899437 | 0.067564646 |
| TAS2R43 | ENST00000609952.1 | ENSG00000233766.3 | 0.862216815 | 1.927741135 | 0.053887333 |
| TAS2R43 | ENST00000610270.1 | ENSG00000272576.1 | 0.905377888 | 2.032249314 | 0.042128423 |
| TAS2R43 | NR_033914.1 | LINC00254 | 0.842914312 | 1.88158843 | 0.059891918 |
| TAS2R43 | NR_046845.1 | DNM3-IT1 | 0.826219268 | 1.855152612 | 0.063574436 |
| TAS2R43 | NR_046871.1 | LINC00333 | 0.82040329 | 1.814939783 | 0.069533164 |
| TAS2R43 | NR_103776.1 | CHRM3-AS2 | 0.941198776 | 2.126063138 | 0.033498003 |
| TAS2R43 | NR_110123.1 | GRM7-AS3 | 0.849831624 | 1.901351301 | 0.057256014 |
| TAS2R43 | NR_110556.1 | LOC102724890 | 0.804570487 | 1.790140344 | 0.073431353 |
| TAS2R43 | NR_110824.1 | LINC01986 | 0.881487157 | 1.963595827 | 0.049576982 |
| TAS2R43 | NR_120502.1 | JARID2-AS1 | -0.867296693 | -1.934789239 | 0.053016164 |
| TAS2R43 | NR_133930.1 | LOC105375556 | 0.983296961 | 2.191715651 | 0.028400045 |
| TAS2R43 | NR_135041.1 | LOC101927038 | -0.910848589 | -2.032819486 | 0.042070764 |
| TCTEX1D4 | ENST00000411489.1 | ENSG00000227112.1 | -0.899465911 | -2.010766346 | 0.044350142 |
| TCTEX1D4 | ENST00000418080.1 | ENSG00000224091.1 | 0.869062797 | 1.93643888 | 0.052813971 |
| TCTEX1D4 | ENST00000418416.1 | ENSG00000226218.1 | -0.852550861 | -1.901517526 | 0.057234259 |
| TCTEX1D4 | ENST00000422017.1 | ENSG00000232227.1 | -0.844346699 | -1.868895193 | 0.061637397 |
| TCTEX1D4 | ENST00000435733.1 | ENSG00000226377.1 | 0.877808989 | 1.974244159 | 0.048353987 |
| TCTEX1D4 | ENST00000442876.1 | ENSG00000233894.1 | -0.820014271 | -1.81686294 | 0.069238102 |
| TCTEX1D4 | ENST00000448748.1 | ENSG00000231238.1 | -0.898479397 | -1.993316437 | 0.046226808 |
| TCTEX1D4 | ENST00000450779.1 | ENSG00000228858.1 | -0.830832471 | -1.866415265 | 0.061983289 |
| TCTEX1D4 | ENST00000452176.1 | ENSG00000223659.1 | -0.825423364 | -1.832816286 | 0.066829896 |

| | | | | | |
|---|---|---|---|---|---|
| TCTEX1D4 | ENST00000452412.1 | ENSG00000233860.1 | 0.805836176 | 1.795533214 | 0.072568786 |
| TCTEX1D4 | ENST00000455788.1 | ENSG00000236263.1 | 0.956120651 | 2.141491136 | 0.032234453 |
| TCTEX1D4 | ENST00000457371.1 | ENSG00000237401.2 | 0.834339297 | 1.85560686 | 0.063509615 |
| TCTEX1D4 | ENST00000485338.1 | ENSG00000239641.1 | -0.870157153 | -1.933711457 | 0.053148614 |
| TCTEX1D4 | ENST00000489690.1 | ENSG00000243944.1 | -0.865651068 | -1.933485854 | 0.053176374 |
| TCTEX1D4 | ENST00000494509.1 | ENSG00000240095.1 | 0.814513704 | 1.835596667 | 0.066417331 |
| TCTEX1D4 | ENST00000500496.2 | ENSG00000245479.2 | 0.944003118 | 2.100694227 | 0.035667816 |
| TCTEX1D4 | ENST00000502467.1 | ENSG00000250530.1 | -0.919829259 | -2.037898252 | 0.041560109 |
| TCTEX1D4 | ENST00000513836.1 | ENSG00000251266.1 | -0.821974815 | -1.862169981 | 0.062579134 |
| TCTEX1D4 | ENST00000521307.1 | ENSG00000253177.1 | 0.875923158 | 1.947382724 | 0.051488868 |
| TCTEX1D4 | ENST00000527789.1 | ENSG00000255173.1 | 0.825719437 | 1.824528505 | 0.068072211 |
| TCTEX1D4 | ENST00000534178.1 | ENSG00000255120.1 | 0.843797076 | 1.911290353 | 0.055967278 |
| TCTEX1D4 | ENST00000547834.1 | ENSG00000258325.1 | 0.86024102 | 1.928411305 | 0.053803987 |
| TCTEX1D4 | ENST00000548722.2 | ENSG00000257194.2 | -0.850782287 | -1.894632771 | 0.05814107 |
| TCTEX1D4 | ENST00000554431.1 | ENSG00000258616.1 | -0.837997458 | -1.872123921 | 0.061189461 |
| TCTEX1D4 | ENST00000557602.1 | ENSG00000258616.1 | -0.827747246 | -1.853665527 | 0.063787027 |
| TCTEX1D4 | ENST00000558475.1 | ENSG00000259604.1 | 0.844898308 | 1.870473858 | 0.061418044 |
| TCTEX1D4 | ENST00000562970.1 | ENSG00000260145.1 | 0.85133518 | 1.92068586 | 0.054771323 |
| TCTEX1D4 | ENST00000566521.1 | ENSG00000261629.1 | -0.854074809 | -1.946307264 | 0.051617842 |
| TCTEX1D4 | ENST00000567127.1 | ENSG00000260264.1 | -0.839478929 | -1.867866728 | 0.06178065 |
| TCTEX1D4 | ENST00000567395.1 | ENSG00000261090.1 | 0.812296825 | 1.792607594 | 0.073035691 |
| TCTEX1D4 | ENST00000569981.1 | ENSG00000238045.5 | 0.856042181 | 1.912168802 | 0.055854546 |
| TCTEX1D4 | ENST00000570413.1 | ENSG00000263167.1 | 0.85846676 | 1.923121945 | 0.054464737 |
| TCTEX1D4 | ENST00000570493.2 | ENSG00000261898.2 | 0.866866066 | 1.940083252 | 0.052369573 |
| TCTEX1D4 | ENST00000570512.1 | ENSG00000262768.1 | 0.807200766 | 1.79139515 | 0.073229907 |
| TCTEX1D4 | ENST00000577807.1 | ENSG00000263427.1 | 0.875932761 | 1.932591765 | 0.053286508 |
| TCTEX1D4 | ENST00000580311.1 | ENSG00000266803.1 | -0.818344458 | -1.842789967 | 0.065359682 |
| TCTEX1D4 | ENST00000584139.1 | ENSG00000263388.1 | -0.872849808 | -1.954596804 | 0.050630679 |
| TCTEX1D4 | ENST00000584758.1 | ENSG00000265356.1 | 0.812561133 | 1.849933056 | 0.064323199 |
| TCTEX1D4 | ENST00000585559.1 | ENSG00000267117.1 | 0.828850898 | 1.86786814 | 0.061780453 |
| TCTEX1D4 | ENST00000596643.1 | ENSG00000269439.1 | 0.841347234 | 1.890067712 | 0.058748905 |
| TCTEX1D4 | ENST00000599259.1 | ENSG00000269352.1 | 0.822832033 | 1.841764407 | 0.065509618 |
| TCTEX1D4 | ENST00000599467.1 | ENSG00000244513.2 | 0.880892923 | 1.972556187 | 0.048546149 |
| TCTEX1D4 | ENST00000600716.1 | ENSG00000269487.1 | 0.813073497 | 1.826636861 | 0.067754387 |
| TCTEX1D4 | ENST00000602809.1 | ENSG00000270105.1 | -0.807561463 | -1.802313788 | 0.07149605 |
| TCTEX1D4 | ENST00000606470.1 | ENSG00000271913.1 | 0.858288305 | 1.925788779 | 0.054130753 |
| TCTEX1D4 | ENST00000608258.1 | ENSG00000229042.2 | -0.842149664 | -1.879603044 | 0.060162198 |
| TCTEX1D4 | ENST00000609813.1 | ENSG00000272719.1 | 0.842089162 | 1.917017597 | 0.055235696 |
| TCTEX1D4 | NR_028324.1 | LINC01002 | 0.831617874 | 1.884841388 | 0.059451255 |
| TCTEX1D4 | NR_036480.1 | VPS9D1-AS1 | 0.820971186 | 1.856264705 | 0.063415837 |
| TCTEX1D4 | NR_047498.1 | LINC00853 | 0.872580928 | 1.96708982 | 0.049172857 |
| TCTEX1D4 | NR_110846.1 | LOC101928674 | 0.813648055 | 1.822097601 | 0.068440178 |
| TCTEX1D4 | NR_120371.1 | LINC01585 | 0.955164458 | 2.159046767 | 0.030846539 |
| TCTEX1D4 | NR_125957.1 | LOC101928626 | -0.825423364 | -1.828441411 | 0.067483331 |
| TCTEX1D4 | NR_133941.1 | LOC105377247 | -0.821974815 | -1.826198639 | 0.067820346 |
| TCTEX1D4 | NR_134576.1 | LOC105372672 | 0.930554954 | 2.058493052 | 0.039542828 |
| TCTEX1D4 | NR_138038.1 | LINC00677 | 0.869456759 | 1.945983942 | 0.051656669 |
| TCTEX1D4 | NR_144459.1 | ARSD-AS1 | 0.87026043 | 1.938753446 | 0.052531367 |
| TFPT | ENST00000318291.4 | ENSG00000177406.4 | 0.907919357 | 2.051693154 | 0.040199492 |
| TFPT | ENST00000411694.1 | ENSG00000225331.1 | 0.890673083 | 1.968422736 | 0.049019418 |
| TFPT | ENST00000412085.1 | ENSG00000233825.1 | 0.902428678 | 2.027076335 | 0.042654607 |

| | | | | | |
|---|---|---|---|---|---|
| TFPT | ENST00000412759.1 | ENSG00000236933.1 | 0.937164976 | 2.094363604 | 0.036227602 |
| TFPT | ENST00000412772.1 | ENSG00000231507.1 | 0.821908453 | 1.826169376 | 0.067824753 |
| TFPT | ENST00000414740.2 | ENSG00000229646.2 | 0.901117077 | 2.011745437 | 0.044246778 |
| TFPT | ENST00000419662.1 | ENSG00000228265.1 | 0.862672882 | 1.93299417 | 0.053236916 |
| TFPT | ENST00000420315.1 | ENSG00000228072.1 | 0.821090548 | 1.829865004 | 0.067270128 |
| TFPT | ENST00000420981.2 | ENSG00000230438.5 | 0.934292443 | 2.12167502 | 0.033865038 |
| TFPT | ENST00000421020.1 | ENSG00000231407.1 | 0.917745752 | 2.044935417 | 0.040861226 |
| TFPT | ENST00000424181.1 | ENSG00000224977.1 | 0.851772681 | 1.881432304 | 0.059913136 |
| TFPT | ENST00000426519.1 | ENSG00000234142.1 | 0.924369056 | 2.064601029 | 0.038960766 |
| TFPT | ENST00000429608.1 | ENSG00000237480.1 | 0.826849778 | 1.843569795 | 0.065245861 |
| TFPT | ENST00000430920.1 | ENSG00000234203.1 | 0.882268172 | 1.956220483 | 0.050439187 |
| TFPT | ENST00000433036.1 | ENSG00000228989.1 | 0.833866504 | 1.871893756 | 0.061221304 |
| TFPT | ENST00000434627.1 | ENSG00000230074.1 | 0.907388847 | 2.019201009 | 0.043466332 |
| TFPT | ENST00000435434.1 | ENSG00000231233.1 | 0.814005934 | 1.820383292 | 0.068700655 |
| TFPT | ENST00000435733.1 | ENSG00000226377.1 | 0.864528385 | 1.90506934 | 0.056771058 |
| TFPT | ENST00000435892.1 | ENSG00000233635.2 | 0.834711138 | 1.854912553 | 0.063608715 |
| TFPT | ENST00000438190.1 | ENSG00000227214.2 | 0.893445992 | 2.007878089 | 0.044656248 |
| TFPT | ENST00000439184.1 | ENSG00000233985.1 | -0.80248431 | -1.765781059 | 0.077432587 |
| TFPT | ENST00000442069.1 | ENSG00000225655.1 | -0.870541764 | -1.940854604 | 0.052275916 |
| TFPT | ENST00000442829.1 | ENSG00000225284.1 | 0.83649797 | 1.871749349 | 0.061241289 |
| TFPT | ENST00000442850.1 | ENSG00000232600.2 | -0.802520763 | -1.79771354 | 0.072222415 |
| TFPT | ENST00000447206.1 | ENSG00000230839.1 | 0.823259596 | 1.841622052 | 0.065530453 |
| TFPT | ENST00000452176.1 | ENSG00000223659.1 | -0.877057708 | -1.935421453 | 0.052938599 |
| TFPT | ENST00000453051.1 | ENSG00000229407.1 | 0.951342765 | 2.150167234 | 0.031541989 |
| TFPT | ENST00000454530.1 | ENSG00000226649.1 | -0.883783198 | -1.967445682 | 0.049131852 |
| TFPT | ENST00000457115.1 | ENSG00000227245.1 | 0.914315101 | 2.063627712 | 0.039053028 |
| TFPT | ENST00000457253.1 | ENSG00000225173.1 | 0.943958362 | 2.086492393 | 0.036934044 |
| TFPT | ENST00000458154.1 | ENSG00000235578.1 | 0.900388939 | 2.005137461 | 0.044948354 |
| TFPT | ENST00000458194.1 | ENSG00000226193.1 | 0.963796382 | 2.164416318 | 0.030432413 |
| TFPT | ENST00000458364.1 | ENSG00000225655.1 | -0.862143961 | -1.956683416 | 0.050384701 |
| TFPT | ENST00000459985.1 | ENSG00000273066.1 | 0.909494536 | 2.045047102 | 0.040850215 |
| TFPT | ENST00000463255.1 | ENSG00000243305.1 | -0.854458756 | -1.905462722 | 0.056719949 |
| TFPT | ENST00000466431.2 | ENSG00000254485.1 | 0.952669681 | 2.144319359 | 0.032007307 |
| TFPT | ENST00000489077.1 | ENSG00000244198.1 | 0.841845798 | 1.873359915 | 0.061018701 |
| TFPT | ENST00000490013.1 | ENSG00000184115.12 | 0.856154009 | 1.923056387 | 0.054472969 |
| TFPT | ENST00000493123.1 | ENSG00000242428.1 | 0.809819476 | 1.82708884 | 0.067686413 |
| TFPT | ENST00000498358.1 | ENSG00000184115.12 | 0.824217602 | 1.881812434 | 0.059861487 |
| TFPT | ENST00000498693.1 | ENSG00000244198.1 | 0.911680395 | 2.059373277 | 0.039458494 |
| TFPT | ENST00000505196.1 | ENSG00000248131.1 | 0.82941856 | 1.856704248 | 0.063353243 |
| TFPT | ENST00000505556.1 | ENSG00000249409.1 | 0.862716078 | 1.927335379 | 0.053937847 |
| TFPT | ENST00000506100.1 | ENSG00000249409.1 | 0.848014308 | 1.894528348 | 0.058154915 |
| TFPT | ENST00000506394.1 | ENSG00000251665.1 | 0.832484475 | 1.863765952 | 0.062354579 |
| TFPT | ENST00000506791.1 | ENSG00000251131.1 | 0.846312433 | 1.888877311 | 0.05890827 |
| TFPT | ENST00000508083.1 | ENSG00000249343.1 | 0.856006167 | 1.936495407 | 0.052807055 |
| TFPT | ENST00000508188.1 | ENSG00000250999.1 | 0.889509357 | 1.998069513 | 0.045709124 |
| TFPT | ENST00000509036.1 | ENSG00000251131.1 | 0.938705798 | 2.098922701 | 0.035823715 |
| TFPT | ENST00000517300.1 | ENSG00000254144.2 | 0.937715815 | 2.083565046 | 0.037199749 |
| TFPT | ENST00000517846.1 | ENSG00000254485.1 | 0.893625493 | 2.021660313 | 0.043211456 |
| TFPT | ENST00000519451.1 | ENSG00000253363.1 | 0.838104316 | 1.840604865 | 0.065679484 |
| TFPT | ENST00000520603.1 | ENSG00000254001.1 | -0.801586601 | -1.80251036 | 0.071465146 |
| TFPT | ENST00000521207.1 | ENSG00000253716.1 | 0.831464174 | 1.873272043 | 0.061030828 |

| | | | | | |
|---|---|---|---|---|---|
| TFPT | ENST00000522600.1 | ENSG00000246582.2 | 0.902817996 | 2.020398203 | 0.0433421 |
| TFPT | ENST00000526154.1 | ENSG00000254511.1 | 0.941787163 | 2.111860566 | 0.034698411 |
| TFPT | ENST00000526611.1 | ENSG00000246982.2 | 0.807378721 | 1.820336916 | 0.068707713 |
| TFPT | ENST00000526694.1 | ENSG00000231999.2 | 0.813469406 | 1.816059112 | 0.069361305 |
| TFPT | ENST00000527757.1 | ENSG00000255109.1 | -0.803229218 | -1.804940789 | 0.071083948 |
| TFPT | ENST00000528000.1 | ENSG00000254804.1 | 0.856625544 | 1.91259142 | 0.055800379 |
| TFPT | ENST00000529247.1 | ENSG00000254741.1 | 0.847838745 | 1.885853543 | 0.059314693 |
| TFPT | ENST00000532688.1 | ENSG00000255441.1 | 0.917971792 | 2.076374869 | 0.037859294 |
| TFPT | ENST00000537269.1 | ENSG00000257084.1 | 0.852989152 | 1.905603242 | 0.056701701 |
| TFPT | ENST00000543072.1 | ENSG00000256092.2 | -0.907238942 | -2.027391775 | 0.042622362 |
| TFPT | ENST00000543275.1 | ENSG00000256944.1 | 0.949423571 | 2.139078677 | 0.032429297 |
| TFPT | ENST00000543494.1 | ENSG00000256514.1 | 0.800930037 | 1.764738765 | 0.07760768 |
| TFPT | ENST00000545177.3 | ENSG00000230438.5 | 0.975598693 | 2.138695739 | 0.032460318 |
| TFPT | ENST00000548722.2 | ENSG00000257194.2 | -0.901169144 | -2.013413692 | 0.044071126 |
| TFPT | ENST00000549806.1 | ENSG00000257252.1 | 0.91241464 | 2.050422138 | 0.040323255 |
| TFPT | ENST00000549878.1 | ENSG00000257284.1 | 0.845039986 | 1.907859537 | 0.056409373 |
| TFPT | ENST00000552525.1 | ENSG00000257286.1 | 0.895550977 | 1.987993507 | 0.046812408 |
| TFPT | ENST00000558515.1 | ENSG00000259182.1 | 0.876600164 | 1.965084777 | 0.049404426 |
| TFPT | ENST00000558575.1 | ENSG00000259687.1 | 0.83893747 | 1.870796512 | 0.061373291 |
| TFPT | ENST00000560522.1 | ENSG00000259661.1 | 0.813209504 | 1.813215341 | 0.069798615 |
| TFPT | ENST00000563044.1 | ENSG00000260978.1 | 0.875091602 | 1.95463904 | 0.05062569 |
| TFPT | ENST00000563611.1 | ENSG00000261583.1 | 0.830088397 | 1.858592066 | 0.063084983 |
| TFPT | ENST00000563806.1 | ENSG00000238045.5 | 0.945212035 | 2.11547989 | 0.034389067 |
| TFPT | ENST00000565823.1 | ENSG00000260686.1 | -0.925096836 | -2.057175567 | 0.039669341 |
| TFPT | ENST00000565829.1 | ENSG00000260148.1 | 0.89948362 | 2.029306693 | 0.042427062 |
| TFPT | ENST00000569981.1 | ENSG00000238045.5 | 0.835664159 | 1.856947984 | 0.063318555 |
| TFPT | ENST00000570493.2 | ENSG00000261898.2 | 0.81630072 | 1.813477681 | 0.069758178 |
| TFPT | ENST00000570843.1 | ENSG00000261889.1 | 0.902209288 | 2.006502048 | 0.04480271 |
| TFPT | ENST00000570929.1 | ENSG00000262223.2 | 0.887165132 | 1.995860821 | 0.045949074 |
| TFPT | ENST00000570974.1 | ENSG00000263300.1 | 0.800116247 | 1.768233593 | 0.07702186 |
| TFPT | ENST00000574460.1 | ENSG00000263051.1 | 0.823563992 | 1.827785196 | 0.067581796 |
| TFPT | ENST00000577064.1 | ENSG00000262823.1 | 0.800929443 | 1.755099002 | 0.079242375 |
| TFPT | ENST00000578757.1 | ENSG00000175061.13 | 0.815144604 | 1.817805327 | 0.069093892 |
| TFPT | ENST00000578800.1 | ENSG00000264235.1 | 0.941239091 | 2.120928835 | 0.033927792 |
| TFPT | ENST00000578936.1 | ENSG00000265547.1 | 0.945184622 | 2.109898977 | 0.034867058 |
| TFPT | ENST00000586694.1 | ENSG00000267141.1 | 0.827764004 | 1.88453515 | 0.059492625 |
| TFPT | ENST00000588182.2 | ENSG00000267453.2 | 0.806838495 | 1.781348287 | 0.074855571 |
| TFPT | ENST00000588290.1 | ENSG00000267751.1 | 0.85458938 | 1.919584529 | 0.0549104 |
| TFPT | ENST00000588380.1 | ENSG00000266990.1 | 0.857388865 | 1.919552175 | 0.05491449 |
| TFPT | ENST00000588402.1 | ENSG00000267006.1 | -0.80424844 | -1.817137599 | 0.069196047 |
| TFPT | ENST00000589817.1 | ENSG00000231616.4 | 0.883973881 | 1.961198296 | 0.049855896 |
| TFPT | ENST00000590813.1 | ENSG00000231616.4 | 0.82883414 | 1.834170203 | 0.066628733 |
| TFPT | ENST00000591174.1 | ENSG00000267289.1 | 0.951040304 | 2.118875453 | 0.034100994 |
| TFPT | ENST00000593632.1 | ENSG00000180279.5 | 0.877745742 | 1.951052669 | 0.051050781 |
| TFPT | ENST00000593642.1 | ENSG00000267858.1 | 0.928467985 | 2.091183409 | 0.036511626 |
| TFPT | ENST00000594492.1 | ENSG00000250910.3 | 0.833968276 | 1.865959266 | 0.062047065 |
| TFPT | ENST00000594590.2 | ENSG00000268199.2 | 0.868347613 | 1.930018903 | 0.053604496 |
| TFPT | ENST00000595955.1 | ENSG00000268401.1 | 0.9014384 | 2.003672508 | 0.045105153 |
| TFPT | ENST00000596887.1 | ENSG00000237031.3 | -0.878094838 | -1.955461018 | 0.05052868 |
| TFPT | ENST00000597169.1 | ENSG00000269720.1 | 0.851454856 | 1.899081575 | 0.057553751 |
| TFPT | ENST00000597256.1 | ENSG00000267986.1 | 0.846086543 | 1.871662141 | 0.06125336 |

| | | | | | |
|---|---|---|---|---|---|
| TFPT | ENST00000599259.1 | ENSG00000269352.1 | 0.893397759 | 1.989239102 | 0.046674818 |
| TFPT | ENST00000600234.1 | ENSG00000268078.1 | 0.808803115 | 1.786340979 | 0.074044066 |
| TFPT | ENST00000600534.1 | ENSG00000267858.1 | 0.89235455 | 1.979142874 | 0.047799922 |
| TFPT | ENST00000600726.1 | ENSG00000267858.1 | 0.891277713 | 2.02382729 | 0.042987925 |
| TFPT | ENST00000600889.1 | ENSG00000232675.3 | 0.902959052 | 2.033791094 | 0.041972663 |
| TFPT | ENST00000601033.1 | ENSG00000268401.1 | 0.934206489 | 2.076009084 | 0.037893111 |
| TFPT | ENST00000601735.1 | ENSG00000244513.2 | 0.804424347 | 1.777834613 | 0.075431022 |
| TFPT | ENST00000602532.1 | ENSG00000270091.1 | 0.904778334 | 2.023154425 | 0.043057229 |
| TFPT | ENST00000604142.1 | ENSG00000271308.1 | 0.876436659 | 1.901600789 | 0.057223365 |
| TFPT | ENST00000606277.1 | ENSG00000272145.1 | 0.842249136 | 1.879704593 | 0.060148349 |
| TFPT | ENST00000606470.1 | ENSG00000271913.1 | 0.808502383 | 1.804475955 | 0.071156725 |
| TFPT | ENST00000607476.1 | ENSG00000272540.1 | 0.887910753 | 1.976183625 | 0.048133984 |
| TFPT | ENST00000607943.1 | ENSG00000273188.1 | 0.87715623 | 1.962606305 | 0.049691938 |
| TFPT | ENST00000608367.1 | ENSG00000273361.1 | 0.871627225 | 1.94566971 | 0.051694427 |
| TFPT | ENST00000608677.1 | ENSG00000273350.1 | 0.867729484 | 1.945824149 | 0.051675867 |
| TFPT | ENST00000608940.1 | ENSG00000272763.1 | 0.927427579 | 2.101956484 | 0.035557088 |
| TFPT | ENST00000609113.1 | ENSG00000272827.1 | 0.849913914 | 1.905053332 | 0.056773139 |
| TFPT | ENST00000610145.1 | ENSG00000273175.1 | 0.826180072 | 1.836199858 | 0.066328104 |
| TFPT | NR_003604.2 | ZFAS1 | 0.941886978 | 2.089923289 | 0.036624691 |
| TFPT | NR_003605.1 | ZFAS1 | 0.899194637 | 2.016505281 | 0.043747167 |
| TFPT | NR_003606.2 | ZFAS1 | 0.926685453 | 2.088329163 | 0.036768153 |
| TFPT | NR_026802.1 | FAM74A4 | 0.964852685 | 2.191110876 | 0.02844377 |
| TFPT | NR_026951.1 | LINC00324 | 0.917547635 | 2.058566283 | 0.039535805 |
| TFPT | NR_027052.1 | THAP7-AS1 | 0.918149026 | 2.04929749 | 0.040433034 |
| TFPT | NR_027271.1 | CIRBP-AS1 | 0.940231078 | 2.120952933 | 0.033925764 |
| TFPT | NR_027334.2 | MZF1-AS1 | 0.885741404 | 1.99249013 | 0.046317307 |
| TFPT | NR_036480.1 | VPS9D1-AS1 | 0.945969701 | 2.093400232 | 0.036313442 |
| TFPT | NR_036658.1 | ZFAS1 | 0.927738068 | 2.084935207 | 0.037075182 |
| TFPT | NR_038421.1 | LINC01220 | 0.84606291 | 1.875191726 | 0.060766352 |
| TFPT | NR_038923.1 | SSSCA1-AS1 | 0.861326575 | 1.898985107 | 0.057566434 |
| TFPT | NR_040096.1 | LOC643339 | 0.874022336 | 1.960799692 | 0.049902394 |
| TFPT | NR_046839.1 | AGBL4-IT1 | -0.802218116 | -1.803358526 | 0.071331927 |
| TFPT | NR_103790.1 | LINC00581 | -0.871391286 | -1.96568476 | 0.049335036 |
| TFPT | NR_104158.1 | NRG1-IT1 | 0.850739891 | 1.891094687 | 0.058611706 |
| TFPT | NR_105010.1 | LINC01333 | 0.898674853 | 2.021035527 | 0.043276088 |
| TFPT | NR_108036.1 | CFAP58-AS1 | 0.828018888 | 1.832276395 | 0.066910252 |
| TFPT | NR_109886.1 | RALY-AS1 | 0.869060722 | 1.93177915 | 0.053386771 |
| TFPT | NR_110998.1 | FAM74A4 | 0.964852685 | 2.145212325 | 0.031935875 |
| TFPT | NR_111951.1 | LINC00869 | 0.907173838 | 2.041009266 | 0.041249906 |
| TFPT | NR_111952.1 | LINC00869 | 0.921939544 | 2.077914138 | 0.037717268 |
| TFPT | NR_111953.1 | LINC00869 | 0.900364463 | 2.012480238 | 0.044169338 |
| TFPT | NR_117097.1 | LINC01353 | 0.821908453 | 1.850181695 | 0.064287366 |
| TFPT | NR_117098.1 | LINC01353 | 0.858548286 | 1.916930254 | 0.055246793 |
| TFPT | NR_121188.1 | PGM5P3-AS1 | -0.889729546 | -1.971460156 | 0.048671266 |
| TFPT | NR_121189.1 | PGM5P3-AS1 | -0.89582603 | -1.993724014 | 0.046182224 |
| TFPT | NR_121661.1 | ZBTB20-AS5 | -0.810402804 | -1.807888946 | 0.070623786 |
| TFPT | NR_125957.1 | LOC101928626 | -0.877057708 | -1.975896474 | 0.048166504 |
| TFPT | NR_126522.1 | EXOC3-AS1 | 0.964631543 | 2.166199229 | 0.030295966 |
| TFPT | NR_130143.1 | LOC104968399 | 0.942546634 | 2.096102364 | 0.036073111 |
| TFPT | NR_134579.1 | LOC105372179 | 0.909878011 | 2.042842721 | 0.04106801 |
| TFPT | NR_135024.1 | LOC105369747 | 0.954071027 | 2.151017127 | 0.031474848 |

| | | | | | |
|---|---|---|---|---|---|
| TFPT | NR_135032.1 | LOC105369635 | 0.852989152 | 1.928041273 | 0.053849993 |
| TFPT | NR_135644.1 | LOC105371506 | -0.81516821 | -1.825991352 | 0.067851565 |
| TFPT | NR_138038.1 | LINC00677 | 0.868528006 | 1.946518115 | 0.051592534 |
| TFPT | NR_144459.1 | ARSD-AS1 | 0.803336633 | 1.799902265 | 0.071876072 |
| TMEM27 | ENST00000414377.1 | ENSG00000230470.1 | 0.832218412 | 1.866923567 | 0.061912262 |
| TMEM27 | ENST00000416002.1 | ENSG00000230233.1 | -0.805406537 | -1.825338185 | 0.067950011 |
| TMEM27 | ENST00000418621.1 | ENSG00000224731.1 | 0.842028968 | 1.862179581 | 0.062577782 |
| TMEM27 | ENST00000420828.1 | ENSG00000227718.1 | 0.830163591 | 1.851045559 | 0.064162999 |
| TMEM27 | ENST00000423193.1 | ENSG00000224239.1 | 0.864674376 | 1.929186714 | 0.053707687 |
| TMEM27 | ENST00000426302.1 | ENSG00000230454.1 | -0.877316294 | -1.96409908 | 0.049518603 |
| TMEM27 | ENST00000427524.1 | ENSG00000236065.2 | -0.931827856 | -2.077345002 | 0.037769728 |
| TMEM27 | ENST00000434292.1 | ENSG00000229796.1 | 0.805115137 | 1.793821421 | 0.072841677 |
| TMEM27 | ENST00000435271.1 | ENSG00000231132.1 | -0.846762007 | -1.888916214 | 0.058903057 |
| TMEM27 | ENST00000435315.2 | ENSG00000226751.2 | 0.916627683 | 2.004946342 | 0.044968784 |
| TMEM27 | ENST00000435832.1 | ENSG00000229201.1 | 0.855727223 | 1.897251618 | 0.057794737 |
| TMEM27 | ENST00000438409.1 | ENSG00000234174.1 | -0.828236076 | -1.871405109 | 0.061288951 |
| TMEM27 | ENST00000440038.2 | ENSG00000237094.7 | -0.904129402 | -2.031331276 | 0.042221401 |
| TMEM27 | ENST00000441809.2 | ENSG00000237445.2 | -0.82165297 | -1.83385198 | 0.066675969 |
| TMEM27 | ENST00000448431.1 | ENSG00000232548.1 | 0.911541196 | 2.032126047 | 0.042140897 |
| TMEM27 | ENST00000448674.1 | ENSG00000235119.1 | -0.808455966 | -1.810865831 | 0.070161623 |
| TMEM27 | ENST00000450206.1 | ENSG00000234311.1 | -0.802905058 | -1.798497837 | 0.072098151 |
| TMEM27 | ENST00000450365.1 | ENSG00000224404.1 | 0.819640116 | 1.82594206 | 0.06785899 |
| TMEM27 | ENST00000451034.1 | ENSG00000229805.1 | 0.87150453 | 1.950776353 | 0.051083656 |
| TMEM27 | ENST00000451090.1 | ENSG00000235215.2 | 0.915018243 | 2.048647917 | 0.040496556 |
| TMEM27 | ENST00000451267.1 | ENSG00000230410.1 | -0.849167902 | -1.8979422 | 0.057703696 |
| TMEM27 | ENST00000451556.2 | ENSG00000228386.2 | 0.809424722 | 1.816999107 | 0.06921725 |
| TMEM27 | ENST00000455373.1 | ENSG00000226097.1 | 0.834302426 | 1.846778407 | 0.064779263 |
| TMEM27 | ENST00000457043.1 | ENSG00000231365.1 | 0.834687286 | 1.867890172 | 0.061777381 |
| TMEM27 | ENST00000457632.1 | ENSG00000234248.1 | 0.841308767 | 1.880081566 | 0.060096962 |
| TMEM27 | ENST00000504301.1 | ENSG00000250696.1 | 0.860209123 | 1.913712328 | 0.055656923 |
| TMEM27 | ENST00000505575.1 | ENSG00000248939.1 | -0.835169686 | -1.851782342 | 0.064057084 |
| TMEM27 | ENST00000506379.1 | ENSG00000240152.2 | 0.853025092 | 1.916665559 | 0.055280433 |
| TMEM27 | ENST00000508823.1 | ENSG00000250716.1 | 0.815225796 | 1.839930794 | 0.065778398 |
| TMEM27 | ENST00000509098.1 | ENSG00000250863.1 | 0.838462642 | 1.893702438 | 0.058264518 |
| TMEM27 | ENST00000512882.2 | ENSG00000251575.2 | 0.858708964 | 1.917851979 | 0.055129783 |
| TMEM27 | ENST00000519005.1 | ENSG00000253507.1 | 0.92099662 | 2.060732245 | 0.039328592 |
| TMEM27 | ENST00000519695.1 | ENSG00000253507.1 | 0.892349183 | 1.998753979 | 0.045634979 |
| TMEM27 | ENST00000520192.1 | ENSG00000253807.1 | 0.898452099 | 2.017101958 | 0.043684874 |
| TMEM27 | ENST00000521294.1 | ENSG00000253664.1 | -0.929001605 | -2.052318166 | 0.040138752 |
| TMEM27 | ENST00000521725.1 | ENSG00000253396.1 | 0.956588596 | 2.164711841 | 0.03040976 |
| TMEM27 | ENST00000522300.1 | ENSG00000249484.4 | 0.871793698 | 1.962768295 | 0.049673103 |
| TMEM27 | ENST00000522390.1 | ENSG00000254262.1 | 0.889695929 | 1.991130373 | 0.046466556 |
| TMEM27 | ENST00000523806.1 | ENSG00000253616.1 | -0.876809289 | -1.933989647 | 0.053114401 |
| TMEM27 | ENST00000523935.1 | ENSG00000253567.1 | 0.804547165 | 1.791095836 | 0.073277917 |
| TMEM27 | ENST00000524275.1 | ENSG00000253507.1 | 0.877735898 | 1.94622833 | 0.051627318 |
| TMEM27 | ENST00000527086.1 | ENSG00000255182.1 | -0.886974669 | -1.97043903 | 0.048788076 |
| TMEM27 | ENST00000527274.2 | ENSG00000255517.2 | -0.893761573 | -1.996214952 | 0.045910531 |
| TMEM27 | ENST00000527727.1 | ENSG00000255227.1 | 0.961156158 | 2.153094198 | 0.031311276 |
| TMEM27 | ENST00000529875.1 | ENSG00000254404.1 | -0.831640318 | -1.876249039 | 0.060621091 |
| TMEM27 | ENST00000536529.1 | ENSG00000256422.1 | 0.850453052 | 1.900489039 | 0.057368972 |
| TMEM27 | ENST00000538641.1 | ENSG00000256422.1 | 0.979926366 | 2.197265919 | 0.02800146 |

| | | | | | |
|---|---|---|---|---|---|
| TMEM27 | ENST00000539313.1 | ENSG00000256588.1 | 0.818028633 | 1.800709089 | 0.071748744 |
| TMEM27 | ENST00000541391.1 | ENSG00000256268.1 | 0.848498642 | 1.920697228 | 0.054769889 |
| TMEM27 | ENST00000548210.1 | ENSG00000257784.1 | -0.941312618 | -2.103325368 | 0.035437337 |
| TMEM27 | ENST00000549329.1 | ENSG00000224189.2 | 0.80313268 | 1.802394446 | 0.071483368 |
| TMEM27 | ENST00000549616.1 | ENSG00000258168.1 | 0.832800092 | 1.883517428 | 0.05963028 |
| TMEM27 | ENST00000550279.1 | ENSG00000258338.1 | 0.870670334 | 1.946416936 | 0.051604677 |
| TMEM27 | ENST00000550805.1 | ENSG00000244306.5 | -0.811965059 | -1.822824306 | 0.068330005 |
| TMEM27 | ENST00000551135.1 | ENSG00000258294.1 | 0.904215148 | 2.023646809 | 0.043006505 |
| TMEM27 | ENST00000552541.1 | ENSG00000258294.1 | 0.858791255 | 1.916707704 | 0.055275076 |
| TMEM27 | ENST00000553954.1 | ENSG00000259052.1 | -0.818189518 | -1.834997602 | 0.066506045 |
| TMEM27 | ENST00000555460.1 | ENSG00000259042.1 | -0.806600173 | -1.826179123 | 0.067823285 |
| TMEM27 | ENST00000555636.1 | ENSG00000259072.1 | 0.938472814 | 2.119402305 | 0.034056482 |
| TMEM27 | ENST00000558221.1 | ENSG00000259704.1 | -0.856413029 | -1.939741898 | 0.052411065 |
| TMEM27 | ENST00000558237.1 | ENSG00000259684.1 | 0.826626356 | 1.849626742 | 0.064367366 |
| TMEM27 | ENST00000560268.1 | ENSG00000259287.1 | -0.826687841 | -1.843258555 | 0.065291269 |
| TMEM27 | ENST00000561254.1 | ENSG00000259554.1 | 0.849220819 | 1.916024197 | 0.055362016 |
| TMEM27 | ENST00000563610.1 | ENSG00000260051.1 | -0.80951049 | -1.815100054 | 0.069508535 |
| TMEM27 | ENST00000563855.1 | ENSG00000260658.1 | 0.956830651 | 2.12377445 | 0.033689008 |
| TMEM27 | ENST00000565667.1 | ENSG00000261253.1 | -0.821480657 | -1.835641731 | 0.066410661 |
| TMEM27 | ENST00000565722.1 | ENSG00000245768.2 | 0.918520531 | 2.049100793 | 0.04045226 |
| TMEM27 | ENST00000565735.1 | ENSG00000261213.1 | 0.861563117 | 1.929692823 | 0.05364491 |
| TMEM27 | ENST00000565798.2 | ENSG00000259786.2 | 0.820244228 | 1.81763984 | 0.069119198 |
| TMEM27 | ENST00000568659.1 | ENSG00000260004.1 | 0.897084546 | 2.008543862 | 0.04458553 |
| TMEM27 | ENST00000569313.1 | ENSG00000261604.1 | 0.924470371 | 2.095143144 | 0.03615827 |
| TMEM27 | ENST00000569849.1 | ENSG00000260640.1 | 0.822090549 | 1.860297016 | 0.062843515 |
| TMEM27 | ENST00000572193.1 | ENSG00000261872.1 | 0.84048463 | 1.889929454 | 0.058767396 |
| TMEM27 | ENST00000573260.1 | ENSG00000262482.1 | 0.955874367 | 2.120794997 | 0.033939058 |
| TMEM27 | ENST00000577853.1 | ENSG00000264207.1 | -0.819329001 | -1.822914525 | 0.068316338 |
| TMEM27 | ENST00000581996.1 | ENSG00000265778.1 | 0.854683596 | 1.899207427 | 0.057537209 |
| TMEM27 | ENST00000588402.1 | ENSG00000267006.1 | 0.825317494 | 1.850036399 | 0.064308304 |
| TMEM27 | ENST00000588842.1 | ENSG00000235779.3 | 0.841577749 | 1.878696403 | 0.060285959 |
| TMEM27 | ENST00000591469.1 | ENSG00000267374.1 | 0.92663643 | 2.074952184 | 0.037990967 |
| TMEM27 | ENST00000591621.1 | ENSG00000232116.2 | 0.86561904 | 1.954441788 | 0.050648993 |
| TMEM27 | ENST00000592518.1 | ENSG00000267786.1 | -0.833701181 | -1.857394358 | 0.063255069 |
| TMEM27 | ENST00000593175.1 | ENSG00000229036.3 | 0.93770505 | 2.085304239 | 0.037041693 |
| TMEM27 | ENST00000594589.1 | ENSG00000269321.1 | -0.947522908 | -2.078161203 | 0.037694514 |
| TMEM27 | ENST00000595737.1 | ENSG00000228065.6 | 0.869225506 | 1.936907877 | 0.052756605 |
| TMEM27 | ENST00000595972.1 | ENSG00000230333.2 | 0.812938669 | 1.829099247 | 0.067384742 |
| TMEM27 | ENST00000596567.1 | ENSG00000226647.2 | 0.818242677 | 1.824170145 | 0.068126354 |
| TMEM27 | ENST00000597755.1 | ENSG00000236194.2 | 0.867019197 | 1.928653624 | 0.053773877 |
| TMEM27 | ENST00000598065.1 | ENSG00000231731.3 | -0.900875631 | -2.03162967 | 0.042191161 |
| TMEM27 | ENST00000600007.1 | ENSG00000268655.1 | -0.80091687 | -1.816496987 | 0.06929417 |
| TMEM27 | ENST00000601420.1 | ENSG00000269560.1 | -0.85005336 | -1.894798236 | 0.058119137 |
| TMEM27 | ENST00000602835.1 | ENSG00000270096.1 | 0.869402735 | 1.95738333 | 0.050302417 |
| TMEM27 | ENST00000602881.1 | ENSG00000269965.1 | 0.937187403 | 2.115977889 | 0.034346688 |
| TMEM27 | ENST00000606010.1 | ENSG00000272249.1 | 0.80055332 | 1.769561847 | 0.076800159 |
| TMEM27 | ENST00000606855.1 | ENSG00000245937.3 | -0.900315327 | -2.01034019 | 0.044395196 |
| TMEM27 | ENST00000606885.1 | ENSG00000231698.2 | 0.845054008 | 1.87283424 | 0.061091278 |
| TMEM27 | ENST00000607119.1 | ENSG00000272541.1 | 0.867265824 | 1.944133142 | 0.051879398 |
| TMEM27 | ENST00000607665.1 | ENSG00000272254.1 | 0.807965999 | 1.80062221 | 0.071762446 |
| TMEM27 | ENST00000608465.1 | ENSG00000272758.1 | 0.899034161 | 2.01595576 | 0.043804602 |

| | | | | | |
|---|---|---|---|---|---|
| TMEM27 | ENST00000608943.1 | ENSG00000273368.1 | 0.842789989 | 1.869486317 | 0.061555186 |
| TMEM27 | ENST00000610270.1 | ENSG00000272576.1 | 0.892770647 | 1.996457333 | 0.045884166 |
| TMEM27 | NR_027402.1 | FAM223B | -0.870870544 | -1.923247559 | 0.054448967 |
| TMEM27 | NR_027425.1 | FAM66D | -0.917197339 | -2.057837387 | 0.039605746 |
| TMEM27 | NR_033914.1 | LINC00254 | 0.805521859 | 1.806457485 | 0.070846909 |
| TMEM27 | NR_046571.1 | POTEH-AS1 | -0.825365745 | -1.830913401 | 0.06711347 |
| TMEM27 | NR_046748.1 | ARHGAP31-AS1 | 0.812747069 | 1.830859167 | 0.067121566 |
| TMEM27 | NR_046845.1 | DNM3-IT1 | 0.94679116 | 2.132552399 | 0.032961462 |
| TMEM27 | NR_073552.1 | LOC101059948 | -0.80091687 | -1.821198115 | 0.068576747 |
| TMEM27 | NR_102703.1 | MAGEA8-AS1 | 0.911368354 | 2.019875162 | 0.043396338 |
| TMEM27 | NR_103776.1 | CHRM3-AS2 | 0.85512091 | 1.888938152 | 0.058900117 |
| TMEM27 | NR_109877.1 | LINC01470 | 0.861961243 | 1.911296995 | 0.055966425 |
| TMEM27 | NR_110556.1 | LOC102724890 | 0.832575746 | 1.87206286 | 0.061197907 |
| TMEM27 | NR_110731.1 | LINC01232 | -0.903932485 | -2.017170292 | 0.043677745 |
| TMEM27 | NR_110824.1 | LINC01986 | 0.83477461 | 1.850350236 | 0.064263086 |
| TMEM27 | NR_120466.1 | LINC01489 | 0.822444666 | 1.823643817 | 0.068205938 |
| TMEM27 | NR_125849.1 | LOC101928140 | 0.850338 | 1.892663924 | 0.058402577 |
| TMEM27 | NR_133930.1 | LOC105375556 | 0.887439174 | 1.979419542 | 0.04776879 |
| TMEM27 | NR_134566.1 | LOC105372695 | 0.911948084 | 2.048320153 | 0.04052864 |
| TMEM27 | NR_134632.1 | LOC105373051 | -0.880610052 | -1.92875487 | 0.053761301 |
| TMEM27 | NR_135040.1 | LOC101927038 | -0.94445264 | -2.117727335 | 0.034198166 |
| TMEM27 | NR_135041.1 | LOC101927038 | -0.89916859 | -2.02308816 | 0.043064059 |
| TMEM27 | NR_135549.1 | LOC101929411 | 0.816403739 | 1.8328917 | 0.066818678 |
| TMEM27 | NR_136218.1 | MEF2C-AS1 | 0.844208012 | 1.89719079 | 0.057802762 |
| TMSB15A | ENST00000295549.4 | ENSG00000163364.5 | -0.868519553 | -1.94767463 | 0.051453908 |
| TMSB15A | ENST00000381106.4 | ENSG00000205663.5 | -0.801804626 | -1.7775378 | 0.075479797 |
| TMSB15A | ENST00000414098.2 | ENSG00000234428.2 | -0.815743702 | -1.831287007 | 0.067057716 |
| TMSB15A | ENST00000414896.1 | ENSG00000223374.1 | -0.806329055 | -1.807618349 | 0.07066592 |
| TMSB15A | ENST00000417654.1 | ENSG00000224893.1 | 0.808225121 | 1.801455041 | 0.071631187 |
| TMSB15A | ENST00000418621.1 | ENSG00000224731.1 | -0.82857497 | -1.847381914 | 0.064691809 |
| TMSB15A | ENST00000419296.1 | ENSG00000204588.5 | 0.808573664 | 1.789461858 | 0.073540465 |
| TMSB15A | ENST00000420044.1 | ENSG00000225956.1 | -0.870494403 | -1.942708453 | 0.052051394 |
| TMSB15A | ENST00000422807.1 | ENSG00000227683.1 | -0.870283658 | -1.944067239 | 0.051887343 |
| TMSB15A | ENST00000423796.1 | ENSG00000235146.2 | -0.96475304 | -2.144371787 | 0.032003109 |
| TMSB15A | ENST00000424274.1 | ENSG00000232120.1 | -0.834732949 | -1.864296511 | 0.062280077 |
| TMSB15A | ENST00000424342.1 | ENSG00000234988.1 | 0.940634162 | 2.088990234 | 0.036708602 |
| TMSB15A | ENST00000424678.1 | ENSG00000229600.1 | -0.928605228 | -2.077735873 | 0.037733693 |
| TMSB15A | ENST00000426444.1 | ENSG00000239395.1 | -0.898415182 | -2.013171017 | 0.044096641 |
| TMSB15A | ENST00000428160.1 | ENSG00000236897.1 | -0.806480728 | -1.804205942 | 0.071199028 |
| TMSB15A | ENST00000428853.2 | ENSG00000229206.2 | -0.906081744 | -2.02383348 | 0.042987288 |
| TMSB15A | ENST00000430247.1 | ENSG00000232855.2 | -0.952273104 | -2.136582054 | 0.032632001 |
| TMSB15A | ENST00000430751.1 | ENSG00000232222.1 | 0.879969976 | 1.995221122 | 0.046018768 |
| TMSB15A | ENST00000434292.1 | ENSG00000229796.1 | -0.916515139 | -2.053770449 | 0.039997914 |
| TMSB15A | ENST00000435828.1 | ENSG00000235612.1 | -0.874300575 | -1.955370871 | 0.050539312 |
| TMSB15A | ENST00000438173.2 | ENSG00000227733.4 | -0.871888891 | -1.940466054 | 0.052323076 |
| TMSB15A | ENST00000441029.2 | ENSG00000229188.2 | 0.866205242 | 1.925312851 | 0.054190231 |
| TMSB15A | ENST00000442017.1 | ENSG00000229660.1 | 0.938889374 | 2.107636462 | 0.035062447 |
| TMSB15A | ENST00000442852.1 | ENSG00000237923.1 | 0.952550305 | 2.138550907 | 0.032472057 |
| TMSB15A | ENST00000443123.1 | ENSG00000229457.1 | -0.804183963 | -1.801651423 | 0.071600265 |
| TMSB15A | ENST00000445233.1 | ENSG00000233928.1 | -0.963199786 | -2.168009847 | 0.030157937 |
| TMSB15A | ENST00000447111.1 | ENSG00000231903.1 | -0.996552565 | -2.207630719 | 0.027270026 |

| | | | | | |
|---|---|---|---|---|---|
| TMSB15A | ENST00000449749.1 | ENSG00000230834.1 | -0.803481501 | -1.784847339 | 0.074286082 |
| TMSB15A | ENST00000450227.1 | ENSG00000229941.1 | -0.835063948 | -1.866937953 | 0.061910253 |
| TMSB15A | ENST00000450696.1 | ENSG00000235146.2 | -0.852862417 | -1.928867783 | 0.053747278 |
| TMSB15A | ENST00000451697.1 | ENSG00000233823.1 | 0.83550416 | 1.880593555 | 0.060027229 |
| TMSB15A | ENST00000453584.1 | ENSG00000233613.1 | 0.814691205 | 1.813267339 | 0.069790598 |
| TMSB15A | ENST00000456499.1 | ENSG00000237640.1 | 0.828190871 | 1.867384709 | 0.061847884 |
| TMSB15A | ENST00000457998.2 | ENSG00000233006.2 | 0.831399833 | 1.854025899 | 0.063735455 |
| TMSB15A | ENST00000479039.1 | ENSG00000241224.2 | 0.853525898 | 1.918269362 | 0.055076865 |
| TMSB15A | ENST00000480904.2 | ENSG00000206573.4 | 0.826688999 | 1.839542337 | 0.065835457 |
| TMSB15A | ENST00000483283.1 | ENSG00000240571.1 | -0.932730963 | -2.093421523 | 0.036311543 |
| TMSB15A | ENST00000498199.1 | ENSG00000206573.4 | 0.808262514 | 1.804874012 | 0.071094399 |
| TMSB15A | ENST00000500498.2 | ENSG00000245311.2 | 0.870563702 | 1.946550058 | 0.051588701 |
| TMSB15A | ENST00000503987.1 | ENSG00000250075.1 | -0.835775385 | -1.8637333 | 0.062359167 |
| TMSB15A | ENST00000506420.1 | ENSG00000250034.1 | 0.864541799 | 1.930421188 | 0.053554672 |
| TMSB15A | ENST00000506723.2 | ENSG00000249484.4 | -0.803380864 | -1.789357862 | 0.073557201 |
| TMSB15A | ENST00000507997.1 | ENSG00000250551.1 | 0.859869362 | 1.916330049 | 0.055323098 |
| TMSB15A | ENST00000508191.1 | ENSG00000250910.3 | -0.840978804 | -1.871325362 | 0.061299997 |
| TMSB15A | ENST00000508687.1 | ENSG00000250538.1 | -0.896128211 | -2.019465157 | 0.043438895 |
| TMSB15A | ENST00000508823.1 | ENSG00000250716.1 | -0.893092839 | -1.987824522 | 0.046831101 |
| TMSB15A | ENST00000510198.1 | ENSG00000248733.1 | -0.828606633 | -1.831303309 | 0.067055284 |
| TMSB15A | ENST00000512563.1 | ENSG00000249547.1 | -0.927239043 | -2.092521341 | 0.036391905 |
| TMSB15A | ENST00000518339.1 | ENSG00000253470.1 | -0.880347695 | -1.971506299 | 0.048665993 |
| TMSB15A | ENST00000518837.1 | ENSG00000253947.1 | -0.80714885 | -1.800266585 | 0.071818554 |
| TMSB15A | ENST00000520192.1 | ENSG00000253807.1 | -0.90214417 | -2.050692803 | 0.040296872 |
| TMSB15A | ENST00000522190.1 | ENSG00000254165.1 | -0.811935304 | -1.810206244 | 0.07026381 |
| TMSB15A | ENST00000522281.1 | ENSG00000253376.1 | -0.856486223 | -1.927377023 | 0.053932661 |
| TMSB15A | ENST00000522300.1 | ENSG00000249484.4 | -0.835332676 | -1.83482195 | 0.066532075 |
| TMSB15A | ENST00000522426.1 | ENSG00000253538.1 | -0.938797799 | -2.111550137 | 0.034725053 |
| TMSB15A | ENST00000531977.1 | ENSG00000224023.6 | -0.906204946 | -2.054259164 | 0.039950615 |
| TMSB15A | ENST00000535914.1 | ENSG00000256894.1 | -0.858690496 | -1.931314415 | 0.053444182 |
| TMSB15A | ENST00000536412.1 | ENSG00000256072.1 | 0.869212624 | 1.931514205 | 0.053419494 |
| TMSB15A | ENST00000546135.1 | ENSG00000256670.1 | 0.912728457 | 2.014140838 | 0.043994749 |
| TMSB15A | ENST00000548199.1 | ENSG00000257614.1 | -0.891790838 | -1.985435345 | 0.047096057 |
| TMSB15A | ENST00000551067.1 | ENSG00000257891.1 | 0.957809835 | 2.125601638 | 0.033536443 |
| TMSB15A | ENST00000551174.1 | ENSG00000257762.1 | 0.866891218 | 1.937924609 | 0.052632421 |
| TMSB15A | ENST00000552558.1 | ENSG00000257947.1 | -0.808643265 | -1.8026192 | 0.07144804 |
| TMSB15A | ENST00000553537.1 | ENSG00000258481.1 | 0.867695612 | 1.936568302 | 0.052798136 |
| TMSB15A | ENST00000555460.1 | ENSG00000259042.1 | 0.878653587 | 1.976147046 | 0.048138125 |
| TMSB15A | ENST00000558618.1 | ENSG00000259209.1 | 0.839088588 | 1.878932286 | 0.06025374 |
| TMSB15A | ENST00000562582.1 | ENSG00000259779.1 | -0.813160714 | -1.829091524 | 0.067385898 |
| TMSB15A | ENST00000562834.1 | ENSG00000261116.1 | -0.885931201 | -1.989035299 | 0.046697307 |
| TMSB15A | ENST00000563342.1 | ENSG00000259914.1 | -0.882191641 | -1.983293115 | 0.047334698 |
| TMSB15A | ENST00000565310.1 | ENSG00000261118.1 | 0.805259266 | 1.814239596 | 0.069640847 |
| TMSB15A | ENST00000565944.1 | ENSG00000260331.1 | -0.836766362 | -1.868757641 | 0.061656541 |
| TMSB15A | ENST00000566449.1 | ENSG00000259791.1 | -0.812248451 | -1.821538611 | 0.068525023 |
| TMSB15A | ENST00000568410.1 | ENSG00000260277.1 | -0.821280284 | -1.835894932 | 0.066373197 |
| TMSB15A | ENST00000568836.1 | ENSG00000259967.1 | -0.918812058 | -2.044928153 | 0.040861942 |
| TMSB15A | ENST00000569778.1 | ENSG00000260823.1 | 0.891764414 | 1.988779556 | 0.04672554 |
| TMSB15A | ENST00000569998.1 | ENSG00000260975.1 | 0.803455535 | 1.78275683 | 0.074625896 |
| TMSB15A | ENST00000570919.1 | ENSG00000263321.1 | -0.931878364 | -2.102543393 | 0.035505703 |
| TMSB15A | ENST00000576021.1 | ENSG00000262413.1 | 0.873010555 | 1.920561419 | 0.054787023 |

| | | | | | |
|---|---|---|---|---|---|
| TMSB15A | ENST00000577360.1 | ENSG00000264273.1 | -0.934860593 | -2.102989947 | 0.035466648 |
| TMSB15A | ENST00000578334.1 | ENSG00000265148.1 | -0.840934809 | -1.862730173 | 0.062500238 |
| TMSB15A | ENST00000585684.1 | ENSG00000267057.1 | -0.94409558 | -2.121074964 | 0.033915494 |
| TMSB15A | ENST00000586297.1 | ENSG00000267633.1 | -0.844544373 | -1.890701921 | 0.058664146 |
| TMSB15A | ENST00000586399.1 | ENSG00000228430.4 | -0.847004608 | -1.90516599 | 0.056758498 |
| TMSB15A | ENST00000588842.1 | ENSG00000235779.3 | -0.879842861 | -1.968004707 | 0.049067496 |
| TMSB15A | ENST00000589983.1 | ENSG00000267057.1 | -0.930288274 | -2.092249811 | 0.036416175 |
| TMSB15A | ENST00000592045.1 | ENSG00000267057.1 | -0.964716307 | -2.144907233 | 0.031960265 |
| TMSB15A | ENST00000597680.1 | ENSG00000269574.1 | -0.875718391 | -1.953653566 | 0.050742201 |
| TMSB15A | ENST00000597755.1 | ENSG00000236194.2 | -0.821762241 | -1.827089868 | 0.067686258 |
| TMSB15A | ENST00000598065.1 | ENSG00000231731.3 | 0.888279411 | 1.999370356 | 0.045568297 |
| TMSB15A | ENST00000599050.1 | ENSG00000268366.1 | -0.861235146 | -1.903268358 | 0.057005537 |
| TMSB15A | ENST00000600956.1 | ENSG00000232732.5 | 0.831362004 | 1.86851085 | 0.061690899 |
| TMSB15A | ENST00000602051.1 | ENSG00000227877.2 | 0.860495368 | 1.928868574 | 0.05374718 |
| TMSB15A | ENST00000602620.1 | ENSG00000215386.6 | -0.874162475 | -1.936212478 | 0.052841683 |
| TMSB15A | ENST00000603949.1 | ENSG00000270332.1 | -0.890986016 | -2.004088869 | 0.045060542 |
| TMSB15A | ENST00000604312.1 | ENSG00000270947.1 | -0.901992968 | -2.011556191 | 0.044266741 |
| TMSB15A | ENST00000606482.1 | ENSG00000272416.1 | -0.854322061 | -1.873752358 | 0.060964566 |
| TMSB15A | ENST00000606869.1 | ENSG00000272349.1 | -0.817304046 | -1.820383707 | 0.068700592 |
| TMSB15A | ENST00000607051.1 | ENSG00000271771.1 | -0.869363484 | -1.941252105 | 0.052227706 |
| TMSB15A | ENST00000608088.1 | ENSG00000272632.1 | -0.818764128 | -1.835602407 | 0.066416481 |
| TMSB15A | ENST00000608133.1 | ENSG00000273193.1 | -0.886905985 | -1.98832154 | 0.04677614 |
| TMSB15A | ENST00000609349.1 | ENSG00000272861.1 | -0.882248371 | -1.971506785 | 0.048665938 |
| TMSB15A | ENST00000609807.1 | ENSG00000272700.1 | -0.889329976 | -2.000395289 | 0.045457597 |
| TMSB15A | NR_027067.1 | LINC00114 | 0.821086185 | 1.817793606 | 0.069095684 |
| TMSB15A | NR_027402.1 | FAM223B | 0.888414061 | 2.003543411 | 0.045118993 |
| TMSB15A | NR_027425.1 | FAM66D | 0.825449202 | 1.835399625 | 0.066446499 |
| TMSB15A | NR_027440.1 | LOC100272217 | 0.950810328 | 2.131863646 | 0.033018058 |
| TMSB15A | NR_040001.2 | LINC01116 | -0.834701913 | -1.887518449 | 0.059090627 |
| TMSB15A | NR_046748.1 | ARHGAP31-AS1 | -0.896881661 | -2.021261224 | 0.043252731 |
| TMSB15A | NR_046766.1 | ATP2B2-IT2 | -0.91873671 | -2.091777258 | 0.036458446 |
| TMSB15A | NR_047040.1 | LINC00424 | -0.803941242 | -1.771584993 | 0.076463473 |
| TMSB15A | NR_047698.1 | VWC2L-IT1 | -0.892515211 | -1.992494797 | 0.046316795 |
| TMSB15A | NR_104618.1 | LINC01017 | -0.825447321 | -1.851494522 | 0.064098442 |
| TMSB15A | NR_109877.1 | LINC01470 | -0.838191247 | -1.867681027 | 0.061806545 |
| TMSB15A | NR_109975.1 | ARNTL2-AS1 | 0.826971967 | 1.851812974 | 0.064052684 |
| TMSB15A | NR_110007.1 | ADNP-AS1 | -0.865278816 | -1.930707174 | 0.053519275 |
| TMSB15A | NR_110117.1 | LOC101927769 | -0.84027656 | -1.861871245 | 0.062621241 |
| TMSB15A | NR_125769.1 | LINC01269 | 0.912077594 | 2.063424667 | 0.039072298 |
| TMSB15A | NR_125839.1 | LOC101927020 | -0.814782773 | -1.827073442 | 0.067688728 |
| TMSB15A | NR_126334.1 | LOC101927932 | -0.846376216 | -1.88979578 | 0.058785278 |
| TMSB15A | NR_134573.1 | GS1-124K5.4 | -0.949733207 | -2.111736252 | 0.034709078 |
| TMSB15A | NR_134610.1 | LOC105375014 | 0.944553338 | 2.095092018 | 0.036162813 |
| TMSB15A | NR_135076.1 | LOC102723838 | -0.920831305 | -2.033539902 | 0.041998006 |
| TMSB15A | NR_136178.1 | LOC101928166 | -0.894514884 | -1.997617462 | 0.045758149 |
| TMSB15A | NR_136218.1 | MEF2C-AS1 | -0.859336298 | -1.909378623 | 0.056213266 |
| TNFAIP8L2 | ENST00000390540.2 | ENSG00000254140.1 | 0.843197303 | 1.893329507 | 0.058314063 |
| TNFAIP8L2 | ENST00000411694.1 | ENSG00000225331.1 | 0.82016441 | 1.847631605 | 0.064655654 |
| TNFAIP8L2 | ENST00000415205.1 | ENSG00000182057.4 | 0.878826737 | 1.985807612 | 0.04705469 |
| TNFAIP8L2 | ENST00000416595.1 | ENSG00000223623.1 | 0.861890602 | 1.930204282 | 0.053581531 |
| TNFAIP8L2 | ENST00000419662.1 | ENSG00000228265.1 | 0.864059674 | 1.955870442 | 0.050480418 |

| | | | | | |
|---|---|---|---|---|---|
| TNFAIP8L2 | ENST00000422763.1 | ENSG00000231131.2 | -0.935695316 | -2.095219686 | 0.036151468 |
| TNFAIP8L2 | ENST00000424257.1 | ENSG00000231626.1 | -0.853364412 | -1.921266451 | 0.054698124 |
| TNFAIP8L2 | ENST00000425881.1 | ENSG00000239636.1 | 0.948674286 | 2.086276844 | 0.036953553 |
| TNFAIP8L2 | ENST00000426475.1 | ENSG00000239467.1 | 0.961795343 | 2.173594488 | 0.029735603 |
| TNFAIP8L2 | ENST00000431730.1 | ENSG00000237401.2 | 0.848193075 | 1.900327197 | 0.057390194 |
| TNFAIP8L2 | ENST00000432699.1 | ENSG00000233334.2 | 0.819131427 | 1.827484987 | 0.067626881 |
| TNFAIP8L2 | ENST00000433035.1 | ENSG00000230483.1 | 0.821377143 | 1.834123679 | 0.066635637 |
| TNFAIP8L2 | ENST00000433051.1 | ENSG00000233193.1 | 0.932127753 | 2.097642031 | 0.035936779 |
| TNFAIP8L2 | ENST00000434250.1 | ENSG00000234055.1 | 0.876497789 | 1.957354506 | 0.050305803 |
| TNFAIP8L2 | ENST00000439184.1 | ENSG00000233985.1 | -0.851242337 | -1.89140871 | 0.058569807 |
| TNFAIP8L2 | ENST00000439443.1 | ENSG00000236911.2 | 0.821029979 | 1.829689648 | 0.06729636 |
| TNFAIP8L2 | ENST00000440595.1 | ENSG00000228265.1 | 0.959873103 | 2.155930582 | 0.031089086 |
| TNFAIP8L2 | ENST00000441592.2 | ENSG00000224078.8 | 0.993476994 | 2.227440668 | 0.025917837 |
| TNFAIP8L2 | ENST00000442649.1 | ENSG00000234089.1 | -0.918986171 | -2.062161538 | 0.039192359 |
| TNFAIP8L2 | ENST00000446562.1 | ENSG00000233896.1 | 0.923295414 | 2.061944192 | 0.03921305 |
| TNFAIP8L2 | ENST00000448570.1 | ENSG00000224549.1 | 0.890895549 | 1.983889588 | 0.04726815 |
| TNFAIP8L2 | ENST00000450072.1 | ENSG00000228486.5 | 0.802610033 | 1.787806159 | 0.073807287 |
| TNFAIP8L2 | ENST00000451507.1 | ENSG00000229539.1 | 0.946099914 | 2.102168531 | 0.035538515 |
| TNFAIP8L2 | ENST00000456091.1 | ENSG00000226985.1 | 0.891072881 | 1.990422046 | 0.046544463 |
| TNFAIP8L2 | ENST00000463255.1 | ENSG00000243305.1 | -0.922824212 | -2.068327936 | 0.038609197 |
| TNFAIP8L2 | ENST00000488310.1 | ENSG00000240449.1 | 0.823267828 | 1.852194282 | 0.06399793 |
| TNFAIP8L2 | ENST00000489077.1 | ENSG00000244198.1 | 0.825508835 | 1.835587949 | 0.066418621 |
| TNFAIP8L2 | ENST00000489090.1 | ENSG00000240045.1 | -0.909312632 | -2.045759724 | 0.040780016 |
| TNFAIP8L2 | ENST00000503723.1 | ENSG00000250472.1 | -0.833469827 | -1.839356076 | 0.06586283 |
| TNFAIP8L2 | ENST00000504916.1 | ENSG00000248112.1 | -0.943431278 | -2.137791911 | 0.032533636 |
| TNFAIP8L2 | ENST00000509453.1 | ENSG00000249145.1 | 0.853499944 | 1.909660674 | 0.056176917 |
| TNFAIP8L2 | ENST00000510570.1 | ENSG00000250438.1 | -0.847115731 | -1.891382278 | 0.058573333 |
| TNFAIP8L2 | ENST00000510922.1 | ENSG00000250777.1 | -0.864602771 | -1.932455759 | 0.053303278 |
| TNFAIP8L2 | ENST00000517846.1 | ENSG00000254485.1 | 0.873269261 | 1.936761617 | 0.05277449 |
| TNFAIP8L2 | ENST00000520603.1 | ENSG00000254001.1 | -0.812339858 | -1.811963952 | 0.069991767 |
| TNFAIP8L2 | ENST00000521653.1 | ENSG00000253301.1 | 0.880359868 | 1.966600421 | 0.049229295 |
| TNFAIP8L2 | ENST00000529247.1 | ENSG00000254741.1 | 0.857509197 | 1.934174812 | 0.053091638 |
| TNFAIP8L2 | ENST00000530435.1 | ENSG00000254630.1 | 0.934646776 | 2.07254612 | 0.03821454 |
| TNFAIP8L2 | ENST00000534065.1 | ENSG00000254458.1 | 0.812974378 | 1.826380082 | 0.06779303 |
| TNFAIP8L2 | ENST00000543403.1 | ENSG00000256684.1 | -0.844975593 | -1.897101965 | 0.057814482 |
| TNFAIP8L2 | ENST00000545254.1 | ENSG00000256633.1 | 0.859272453 | 1.92627821 | 0.054069645 |
| TNFAIP8L2 | ENST00000549806.1 | ENSG00000257252.1 | 0.851901767 | 1.902147688 | 0.05715185 |
| TNFAIP8L2 | ENST00000557368.1 | ENSG00000258444.1 | 0.817230216 | 1.839086175 | 0.065902512 |
| TNFAIP8L2 | ENST00000562191.1 | ENSG00000261292.1 | -0.875150059 | -1.930547576 | 0.053539026 |
| TNFAIP8L2 | ENST00000564809.1 | ENSG00000261471.1 | 0.810571592 | 1.820662544 | 0.068658169 |
| TNFAIP8L2 | ENST00000565955.1 | ENSG00000261055.1 | 0.911316438 | 2.061832984 | 0.03922364 |
| TNFAIP8L2 | ENST00000571660.1 | ENSG00000262848.1 | 0.918205132 | 2.057573267 | 0.039631115 |
| TNFAIP8L2 | ENST00000572471.1 | ENSG00000262721.1 | 0.851728492 | 1.905805167 | 0.056675488 |
| TNFAIP8L2 | ENST00000573315.1 | ENSG00000270168.1 | 0.877744312 | 1.9476308 | 0.051459156 |
| TNFAIP8L2 | ENST00000579154.1 | ENSG00000265908.1 | -0.859635923 | -1.9190869 | 0.054973337 |
| TNFAIP8L2 | ENST00000580622.1 | ENSG00000264634.1 | 0.952418231 | 2.12109136 | 0.033914115 |
| TNFAIP8L2 | ENST00000582044.1 | ENSG00000263715.2 | 0.942509507 | 2.107430996 | 0.035080237 |
| TNFAIP8L2 | ENST00000583067.1 | ENSG00000266126.1 | -0.902587565 | -2.01773099 | 0.043619285 |
| TNFAIP8L2 | ENST00000585761.1 | ENSG00000267198.1 | 0.812102999 | 1.832838861 | 0.066826538 |
| TNFAIP8L2 | ENST00000586348.1 | ENSG00000267198.1 | 0.858971565 | 1.908818872 | 0.056285461 |
| TNFAIP8L2 | ENST00000586694.1 | ENSG00000267141.1 | 0.831941797 | 1.885913624 | 0.059306595 |

| | | | | | |
|---|---|---|---|---|---|
| TNFAIP8L2 | ENST00000588182.2 | ENSG00000267453.2 | 0.813698257 | 1.813368747 | 0.069774967 |
| TNFAIP8L2 | ENST00000588380.1 | ENSG00000266990.1 | 0.872335932 | 1.950928541 | 0.051065547 |
| TNFAIP8L2 | ENST00000589380.1 | ENSG00000267488.1 | 0.905510097 | 2.041361617 | 0.041214897 |
| TNFAIP8L2 | ENST00000589395.1 | ENSG00000267143.1 | 0.892786508 | 2.00410155 | 0.045059183 |
| TNFAIP8L2 | ENST00000592498.1 | ENSG00000267488.1 | 0.892849222 | 2.007297039 | 0.044718044 |
| TNFAIP8L2 | ENST00000592525.1 | ENSG00000267214.1 | 0.938791238 | 2.095935429 | 0.036087919 |
| TNFAIP8L2 | ENST00000593139.1 | ENSG00000267042.1 | 0.939978951 | 2.089029961 | 0.036705026 |
| TNFAIP8L2 | ENST00000593218.1 | ENSG00000267421.2 | 0.847948023 | 1.89649581 | 0.057894514 |
| TNFAIP8L2 | ENST00000593967.1 | ENSG00000232732.5 | 0.838943897 | 1.870701518 | 0.061386464 |
| TNFAIP8L2 | ENST00000594850.1 | ENSG00000268093.1 | 0.888814415 | 2.005811864 | 0.044876324 |
| TNFAIP8L2 | ENST00000596971.1 | ENSG00000269463.1 | 0.930919956 | 2.112752683 | 0.034621942 |
| TNFAIP8L2 | ENST00000597256.1 | ENSG00000267986.1 | 0.91124734 | 2.017886234 | 0.043603111 |
| TNFAIP8L2 | ENST00000600242.1 | ENSG00000269583.1 | 0.86617666 | 1.92474468 | 0.054261308 |
| TNFAIP8L2 | ENST00000603948.1 | ENSG00000222041.6 | 0.892390577 | 1.953361371 | 0.05077679 |
| TNFAIP8L2 | ENST00000604183.1 | ENSG00000271185.1 | 0.838426782 | 1.865028168 | 0.062177456 |
| TNFAIP8L2 | ENST00000606441.1 | ENSG00000272277.1 | 0.974224957 | 2.165475911 | 0.030351258 |
| TNFAIP8L2 | ENST00000606743.1 | ENSG00000272221.1 | 0.803446154 | 1.7860106 | 0.074097542 |
| TNFAIP8L2 | ENST00000606909.1 | ENSG00000271821.1 | 0.885263856 | 2.000644108 | 0.045430757 |
| TNFAIP8L2 | ENST00000606963.1 | ENSG00000272010.1 | -0.838891032 | -1.878541116 | 0.060307178 |
| TNFAIP8L2 | ENST00000608367.1 | ENSG00000273361.1 | 0.855404989 | 1.900604878 | 0.057353786 |
| TNFAIP8L2 | ENST00000608677.1 | ENSG00000273350.1 | 0.842517984 | 1.899433593 | 0.05750749 |
| TNFAIP8L2 | NR_026774.1 | LINC00239 | 0.863528128 | 1.959427185 | 0.05006278 |
| TNFAIP8L2 | NR_026813.1 | LINC00597 | -0.95698806 | -2.130949937 | 0.033093267 |
| TNFAIP8L2 | NR_026951.1 | LINC00324 | 0.876925089 | 1.962720559 | 0.049678653 |
| TNFAIP8L2 | NR_034131.1 | LINC00272 | 0.843155199 | 1.896529932 | 0.057890006 |
| TNFAIP8L2 | NR_037169.1 | LOC100507547 | 0.827329981 | 1.851688838 | 0.064070518 |
| TNFAIP8L2 | NR_037170.1 | LOC100507547 | 0.819549071 | 1.828730005 | 0.067440065 |
| TNFAIP8L2 | NR_038923.1 | SSSCA1-AS1 | 0.832182032 | 1.832593348 | 0.066863068 |
| TNFAIP8L2 | NR_040047.1 | SDCBP2-AS1 | 0.828032273 | 1.858092573 | 0.063155869 |
| TNFAIP8L2 | NR_040049.1 | SDCBP2-AS1 | 0.837085495 | 1.860124764 | 0.062867876 |
| TNFAIP8L2 | NR_046224.1 | LINC00659 | 0.803372334 | 1.796728896 | 0.072378669 |
| TNFAIP8L2 | NR_047116.1 | HIF1A-AS1 | -0.925395274 | -2.029267226 | 0.04243108 |
| TNFAIP8L2 | NR_073155.1 | C1orf145 | -0.809541396 | -1.827714196 | 0.067592456 |
| TNFAIP8L2 | NR_108106.1 | LINC01135 | 0.814003487 | 1.868681048 | 0.061667202 |
| TNFAIP8L2 | NR_109831.1 | RASSF1-AS1 | 0.848017949 | 1.899738655 | 0.057467425 |
| TNFAIP8L2 | NR_109885.1 | RALY-AS1 | 0.946333883 | 2.109404266 | 0.034909702 |
| TNFAIP8L2 | NR_109886.1 | RALY-AS1 | 0.837815935 | 1.870424295 | 0.061424921 |
| TNFAIP8L2 | NR_110559.1 | LOC101927023 | -0.913606156 | -2.065397935 | 0.038885364 |
| TNFAIP8L2 | NR_110941.1 | MIR762HG | 0.880561181 | 1.975108579 | 0.048255827 |
| TNFAIP8L2 | NR_121624.1 | LOC103352541 | -0.843863408 | -1.873086354 | 0.061056461 |
| TNFAIP8L2 | NR_126522.1 | EXOC3-AS1 | 0.812166102 | 1.830792489 | 0.067131522 |
| TNFAIP8L2 | NR_134252.1 | LOC105379030 | 0.850124769 | 1.91041711 | 0.05607953 |
| TNFAIP8L2 | NR_134520.1 | LOC727993 | 0.929318956 | 2.087899189 | 0.036806929 |
| TNFAIP8L2 | NR_135024.1 | LOC105369747 | 0.832962944 | 1.857609868 | 0.063224436 |
| TNFAIP8L2 | NR_135097.1 | LOC105369443 | -0.844975593 | -1.918502042 | 0.055047383 |
| TNFAIP8L2 | NR_135626.1 | LOC100505585 | 0.822837027 | 1.830789645 | 0.067131946 |
| TNFAIP8L2 | NR_136215.1 | VCAN-AS1 | -0.849541283 | -1.903452296 | 0.056981553 |
| TNFAIP8L2 | NR_136320.1 | LOC105373656 | 0.832531896 | 1.847908119 | 0.064615636 |
| TPGS1 | ENST00000412085.1 | ENSG00000233825.1 | 0.89602813 | 1.976757412 | 0.048069058 |
| TPGS1 | ENST00000412759.1 | ENSG00000236933.1 | 0.893485894 | 1.982055926 | 0.047472982 |
| TPGS1 | ENST00000412772.1 | ENSG00000231507.1 | 0.940866143 | 2.086206462 | 0.036959925 |

| | | | | | |
|---|---|---|---|---|---|
| TPGS1 | ENST00000414740.2 | ENSG00000229646.2 | 0.885067419 | 1.995351391 | 0.046004569 |
| TPGS1 | ENST00000429608.1 | ENSG00000237480.1 | 0.882069464 | 1.956612775 | 0.050393012 |
| TPGS1 | ENST00000430920.1 | ENSG00000234203.1 | 0.885930225 | 1.963153671 | 0.049628321 |
| TPGS1 | ENST00000433876.2 | ENSG00000228423.2 | 0.851982137 | 1.906759373 | 0.056551755 |
| TPGS1 | ENST00000433905.2 | ENSG00000229299.2 | 0.803247886 | 1.767221216 | 0.077191188 |
| TPGS1 | ENST00000434627.1 | ENSG00000230074.1 | 0.845597808 | 1.92340281 | 0.054429482 |
| TPGS1 | ENST00000442850.1 | ENSG00000232600.2 | −0.832053278 | −1.864373207 | 0.062269313 |
| TPGS1 | ENST00000451034.1 | ENSG00000229805.1 | −0.835701141 | −1.881342486 | 0.059925345 |
| TPGS1 | ENST00000453051.1 | ENSG00000229407.1 | 0.923122944 | 2.040013394 | 0.041348992 |
| TPGS1 | ENST00000454530.1 | ENSG00000226649.1 | −0.855469499 | −1.923736976 | 0.054387561 |
| TPGS1 | ENST00000458194.1 | ENSG00000226193.1 | 0.877127409 | 1.943128112 | 0.052000681 |
| TPGS1 | ENST00000459985.1 | ENSG00000273066.1 | 0.87192733 | 1.927124361 | 0.053964133 |
| TPGS1 | ENST00000466431.2 | ENSG00000254485.1 | 0.870726834 | 2.020698044 | 0.043311032 |
| TPGS1 | ENST00000468165.1 | ENSG00000239480.1 | 0.90639621 | 2.037579598 | 0.041591994 |
| TPGS1 | ENST00000490013.1 | ENSG00000184115.12 | 0.932611737 | 2.101617445 | 0.0355868 |
| TPGS1 | ENST00000498358.1 | ENSG00000184115.12 | 0.931329176 | 2.062615533 | 0.039149171 |
| TPGS1 | ENST00000498693.1 | ENSG00000244198.1 | 0.824993869 | 1.853240561 | 0.063847886 |
| TPGS1 | ENST00000504301.1 | ENSG00000250696.1 | −0.855741445 | −1.899386516 | 0.057513675 |
| TPGS1 | ENST00000506394.1 | ENSG00000251665.1 | 0.846175208 | 1.889778906 | 0.058787536 |
| TPGS1 | ENST00000521207.1 | ENSG00000253716.1 | 0.881823498 | 1.957239977 | 0.050319261 |
| TPGS1 | ENST00000522600.1 | ENSG00000246582.2 | 0.910056435 | 2.032577581 | 0.042095218 |
| TPGS1 | ENST00000524942.1 | ENSG00000255553.1 | 0.903276686 | 1.968413254 | 0.049020508 |
| TPGS1 | ENST00000527086.1 | ENSG00000255182.1 | 0.921557862 | 2.041909458 | 0.041160513 |
| TPGS1 | ENST00000527757.1 | ENSG00000255109.1 | −0.846492418 | −1.908823727 | 0.056284835 |
| TPGS1 | ENST00000528000.1 | ENSG00000254804.1 | 0.913422969 | 1.995068423 | 0.046035418 |
| TPGS1 | ENST00000543072.1 | ENSG00000256092.2 | −0.916955988 | −2.031836799 | 0.04217018 |
| TPGS1 | ENST00000543275.1 | ENSG00000256944.1 | 0.968482906 | 2.146175035 | 0.031859017 |
| TPGS1 | ENST00000543494.1 | ENSG00000256514.1 | 0.921553508 | 2.05686377 | 0.039699332 |
| TPGS1 | ENST00000548731.1 | ENSG00000257809.1 | 0.817535201 | 1.833100393 | 0.066787643 |
| TPGS1 | ENST00000551135.1 | ENSG00000258294.1 | −0.839654367 | −1.883029834 | 0.059696325 |
| TPGS1 | ENST00000552525.1 | ENSG00000257286.1 | 0.860412835 | 1.921204366 | 0.054705948 |
| TPGS1 | ENST00000554049.1 | ENSG00000258763.1 | 0.844508588 | 1.910034598 | 0.056128759 |
| TPGS1 | ENST00000558575.1 | ENSG00000259687.1 | 0.975281175 | 2.170630982 | 0.029959078 |
| TPGS1 | ENST00000563018.1 | ENSG00000260193.1 | 0.809809495 | 1.830013578 | 0.067247909 |
| TPGS1 | ENST00000563611.1 | ENSG00000261583.1 | 0.854792222 | 1.934185222 | 0.053090359 |
| TPGS1 | ENST00000563806.1 | ENSG00000238045.5 | 0.883384303 | 1.96559805 | 0.04934506 |
| TPGS1 | ENST00000565359.1 | ENSG00000260601.1 | 0.872282894 | 1.990983166 | 0.046482738 |
| TPGS1 | ENST00000565798.2 | ENSG00000259786.2 | −0.828587308 | −1.859543221 | 0.062950179 |
| TPGS1 | ENST00000565823.1 | ENSG00000260686.1 | −0.849406121 | −1.886662159 | 0.05920578 |
| TPGS1 | ENST00000565829.1 | ENSG00000260148.1 | 0.877250968 | 1.969428055 | 0.048903957 |
| TPGS1 | ENST00000568659.1 | ENSG00000260004.1 | −0.852651058 | −1.914595397 | 0.055544123 |
| TPGS1 | ENST00000569025.1 | ENSG00000246379.2 | −0.800686056 | −1.803911407 | 0.071245196 |
| TPGS1 | ENST00000570843.1 | ENSG00000261889.1 | 0.860590177 | 1.925727715 | 0.054138381 |
| TPGS1 | ENST00000570929.1 | ENSG00000262223.2 | 0.830142553 | 1.840092361 | 0.065754679 |
| TPGS1 | ENST00000574460.1 | ENSG00000263051.1 | 0.872213733 | 1.964464243 | 0.049476279 |
| TPGS1 | ENST00000576086.1 | ENSG00000262823.1 | 0.877517191 | 1.963104005 | 0.04963409 |
| TPGS1 | ENST00000577064.1 | ENSG00000262823.1 | 0.832815275 | 1.847491056 | 0.064676003 |
| TPGS1 | ENST00000578800.1 | ENSG00000264235.1 | 0.880992294 | 1.98963918 | 0.046630697 |
| TPGS1 | ENST00000578936.1 | ENSG00000265547.1 | 0.907719303 | 2.033022482 | 0.042050252 |
| TPGS1 | ENST00000583138.1 | ENSG00000263393.1 | 0.847965219 | 1.899684195 | 0.057474576 |
| TPGS1 | ENST00000583916.1 | ENSG00000264196.1 | 0.88726541 | 1.971777753 | 0.048634983 |

| | | | | | |
|---|---|---|---|---|---|
| TPGS1 | ENST00000586051.1 | ENSG00000267576.1 | 0.814186071 | 1.816528815 | 0.069289292 |
| TPGS1 | ENST00000588290.1 | ENSG00000267751.1 | 0.802771304 | 1.83232629 | 0.066902822 |
| TPGS1 | ENST00000588402.1 | ENSG00000267006.1 | -0.833255517 | -1.858519294 | 0.063095306 |
| TPGS1 | ENST00000588908.1 | ENSG00000267751.1 | 0.887655379 | 1.984354276 | 0.04721636 |
| TPGS1 | ENST00000589673.1 | ENSG00000267755.1 | 0.975323392 | 2.164738252 | 0.030407736 |
| TPGS1 | ENST00000590328.1 | ENSG00000256995.2 | -0.847511882 | -1.884793106 | 0.059457776 |
| TPGS1 | ENST00000591174.1 | ENSG00000267289.1 | 0.884917196 | 1.961729265 | 0.049794013 |
| TPGS1 | ENST00000592413.1 | ENSG00000266933.1 | 0.921723592 | 2.023389502 | 0.043033006 |
| TPGS1 | ENST00000592518.1 | ENSG00000267786.1 | 0.888006286 | 1.976940572 | 0.048048348 |
| TPGS1 | ENST00000593642.1 | ENSG00000267858.1 | 0.87738034 | 1.938564709 | 0.052554364 |
| TPGS1 | ENST00000594590.2 | ENSG00000268199.2 | 0.899655117 | 2.047885622 | 0.040571208 |
| TPGS1 | ENST00000595955.1 | ENSG00000268401.1 | 0.929155002 | 2.073169907 | 0.03815647 |
| TPGS1 | ENST00000596887.1 | ENSG00000237031.3 | -0.88945888 | -2.003873461 | 0.045083617 |
| TPGS1 | ENST00000598092.1 | ENSG00000228065.6 | -0.811645366 | -1.822419808 | 0.068391311 |
| TPGS1 | ENST00000599352.1 | ENSG00000240401.4 | -0.819639481 | -1.856924048 | 0.063321961 |
| TPGS1 | ENST00000600071.1 | ENSG00000269199.1 | 0.860102158 | 1.925685676 | 0.054143634 |
| TPGS1 | ENST00000600534.1 | ENSG00000267858.1 | 0.852447168 | 1.906499164 | 0.056585474 |
| TPGS1 | ENST00000600726.1 | ENSG00000267858.1 | 0.861096099 | 1.940189665 | 0.052356644 |
| TPGS1 | ENST00000601033.1 | ENSG00000268401.1 | 0.864230063 | 1.913338187 | 0.055704772 |
| TPGS1 | ENST00000602532.1 | ENSG00000270091.1 | 0.904071387 | 2.010562295 | 0.04437171 |
| TPGS1 | ENST00000606068.1 | ENSG00000272342.1 | 0.856691389 | 1.941729856 | 0.052169812 |
| TPGS1 | ENST00000607052.1 | ENSG00000271870.1 | -0.850738066 | -1.898632649 | 0.057612793 |
| TPGS1 | ENST00000607119.1 | ENSG00000272541.1 | -0.871942235 | -1.937315053 | 0.052706843 |
| TPGS1 | ENST00000607943.1 | ENSG00000273188.1 | 0.808620762 | 1.800696735 | 0.071750692 |
| TPGS1 | ENST00000608940.1 | ENSG00000272763.1 | 0.911533793 | 2.013196563 | 0.044093955 |
| TPGS1 | ENST00000609113.1 | ENSG00000272827.1 | 0.949329298 | 2.134557542 | 0.032797169 |
| TPGS1 | ENST00000609146.1 | ENSG00000272851.1 | -0.890838875 | -2.009142217 | 0.044522054 |
| TPGS1 | ENST00000609976.1 | ENSG00000272582.1 | 0.846127133 | 1.888215378 | 0.058997042 |
| TPGS1 | ENST00000610270.1 | ENSG00000272576.1 | -0.930037181 | -2.09233221 | 0.036408808 |
| TPGS1 | NR_003604.2 | ZFAS1 | 0.84988606 | 1.892564137 | 0.058415857 |
| TPGS1 | NR_003606.2 | ZFAS1 | 0.836653465 | 1.880065917 | 0.060099095 |
| TPGS1 | NR_027052.1 | THAP7-AS1 | 0.919592393 | 2.02542362 | 0.042823884 |
| TPGS1 | NR_027271.1 | CIRBP-AS1 | 0.85039451 | 1.882672202 | 0.059744805 |
| TPGS1 | NR_027334.2 | MZF1-AS1 | 0.837830166 | 1.861138787 | 0.06272458 |
| TPGS1 | NR_036658.1 | ZFAS1 | 0.830388442 | 1.837751052 | 0.066099097 |
| TPGS1 | NR_038421.1 | LINC01220 | 0.886202032 | 2.008571373 | 0.04458261 |
| TPGS1 | NR_038923.1 | SSSCA1-AS1 | 0.850553384 | 1.914133572 | 0.055603091 |
| TPGS1 | NR_040096.1 | LOC643339 | 0.914552052 | 1.994464395 | 0.046101327 |
| TPGS1 | NR_046454.1 | LINC00907 | -0.826200169 | -1.800300723 | 0.071813167 |
| TPGS1 | NR_046571.1 | POTEH-AS1 | 0.894955029 | 2.010809889 | 0.044345541 |
| TPGS1 | NR_103841.1 | LINC00539 | 0.823657461 | 1.846495655 | 0.06482027 |
| TPGS1 | NR_104158.1 | NRG1-IT1 | 0.87859618 | 1.955972031 | 0.050468449 |
| TPGS1 | NR_108106.1 | LINC01135 | 0.812616395 | 1.812730844 | 0.069873345 |
| TPGS1 | NR_111951.1 | LINC00869 | 0.927623459 | 2.091215884 | 0.036508716 |
| TPGS1 | NR_111952.1 | LINC00869 | 0.947240707 | 2.126235248 | 0.033483677 |
| TPGS1 | NR_111953.1 | LINC00869 | 0.926816174 | 2.057676216 | 0.039621225 |
| TPGS1 | NR_117097.1 | LINC01353 | 0.940866143 | 2.15763367 | 0.030956325 |
| TPGS1 | NR_117098.1 | LINC01353 | 0.942848789 | 2.117046617 | 0.034255892 |
| TPGS1 | NR_130143.1 | LOC104968399 | 0.88944957 | 1.963303818 | 0.049610882 |
| TPGS1 | NR_135041.1 | LOC101927038 | 0.885477986 | 1.985939288 | 0.047040066 |
| TPGS1 | NR_135644.1 | LOC105371506 | -0.876411195 | -1.936574285 | 0.052797404 |

| | | | | | |
|---|---|---|---|---|---|
| TYROBP | ENST00000318291.4 | ENSG00000177406.4 | 0.915134394 | 2.02279779 | 0.043094 |
| TYROBP | ENST00000399186.2 | ENSG00000214888.2 | 0.848840197 | 1.893226164 | 0.058327799 |
| TYROBP | ENST00000412085.1 | ENSG00000233825.1 | 0.92664402 | 2.085885466 | 0.036988999 |
| TYROBP | ENST00000412759.1 | ENSG00000236933.1 | 0.973801484 | 2.156804654 | 0.031020888 |
| TYROBP | ENST00000412772.1 | ENSG00000231507.1 | 0.847986941 | 1.903874705 | 0.056926504 |
| TYROBP | ENST00000413650.1 | ENSG00000230880.2 | 0.805293537 | 1.804201167 | 0.071199776 |
| TYROBP | ENST00000413991.1 | ENSG00000237614.1 | 0.816477274 | 1.81655753 | 0.069284891 |
| TYROBP | ENST00000414740.2 | ENSG00000229646.2 | 0.954696207 | 2.130681832 | 0.033115363 |
| TYROBP | ENST00000419734.1 | ENSG00000234646.1 | -0.807880558 | -1.813509873 | 0.069753217 |
| TYROBP | ENST00000420365.1 | ENSG00000225214.1 | 0.805014108 | 1.790813259 | 0.073323267 |
| TYROBP | ENST00000420465.1 | ENSG00000167355.3 | 0.802168412 | 1.811007831 | 0.07013964 |
| TYROBP | ENST00000420981.2 | ENSG00000230438.5 | 0.829907095 | 1.851812799 | 0.064052709 |
| TYROBP | ENST00000421020.1 | ENSG00000231407.1 | 0.884676283 | 1.982826095 | 0.047386858 |
| TYROBP | ENST00000423869.1 | ENSG00000227848.1 | 0.827600353 | 1.869170528 | 0.061599093 |
| TYROBP | ENST00000424181.1 | ENSG00000224977.1 | 0.81713108 | 1.832102251 | 0.066936188 |
| TYROBP | ENST00000426519.1 | ENSG00000234142.1 | 0.971955112 | 2.147921866 | 0.031719963 |
| TYROBP | ENST00000429608.1 | ENSG00000237480.1 | 0.844158779 | 1.882780644 | 0.059730101 |
| TYROBP | ENST00000433876.2 | ENSG00000228423.2 | 0.914295865 | 2.053754782 | 0.039999431 |
| TYROBP | ENST00000434627.1 | ENSG00000230074.1 | 0.974012199 | 2.167086277 | 0.030228276 |
| TYROBP | ENST00000435434.1 | ENSG00000231233.1 | 0.906770105 | 2.034622697 | 0.041888852 |
| TYROBP | ENST00000435892.1 | ENSG00000233635.2 | 0.959787561 | 2.120171353 | 0.033991597 |
| TYROBP | ENST00000438107.1 | ENSG00000234449.2 | 0.816187112 | 1.823039901 | 0.068297348 |
| TYROBP | ENST00000438190.1 | ENSG00000227214.2 | 0.910648344 | 2.039674682 | 0.041382738 |
| TYROBP | ENST00000442069.1 | ENSG00000225655.1 | -0.907408878 | -2.045138318 | 0.040841224 |
| TYROBP | ENST00000442850.1 | ENSG00000232600.2 | -0.915633846 | -2.059797681 | 0.039417886 |
| TYROBP | ENST00000443380.1 | ENSG00000224371.1 | 0.884477115 | 1.976594839 | 0.048087446 |
| TYROBP | ENST00000444665.1 | ENSG00000228852.2 | 0.827757713 | 1.840192228 | 0.065740021 |
| TYROBP | ENST00000447206.1 | ENSG00000230839.1 | 0.959184497 | 2.149386641 | 0.031603764 |
| TYROBP | ENST00000450109.1 | ENSG00000225376.1 | 0.866395853 | 1.941957225 | 0.052142279 |
| TYROBP | ENST00000453051.1 | ENSG00000229407.1 | 0.953170775 | 2.117865512 | 0.034186459 |
| TYROBP | ENST00000454530.1 | ENSG00000226649.1 | -0.967950978 | -2.151576857 | 0.031430697 |
| TYROBP | ENST00000456715.1 | ENSG00000224893.1 | 0.820503691 | 1.832546333 | 0.066870065 |
| TYROBP | ENST00000457115.1 | ENSG00000227245.1 | 0.927882248 | 2.073190783 | 0.038154528 |
| TYROBP | ENST00000457253.1 | ENSG00000225173.1 | 0.885537415 | 1.971044019 | 0.048718841 |
| TYROBP | ENST00000457848.1 | ENSG00000226412.1 | 0.81141596 | 1.816247857 | 0.06933236 |
| TYROBP | ENST00000458154.1 | ENSG00000235578.1 | 0.880453727 | 1.971360347 | 0.048682673 |
| TYROBP | ENST00000458194.1 | ENSG00000226193.1 | 0.994849932 | 2.237144729 | 0.025276882 |
| TYROBP | ENST00000458364.1 | ENSG00000225655.1 | -0.929862494 | -2.065882188 | 0.038839605 |
| TYROBP | ENST00000459985.1 | ENSG00000273066.1 | 0.973458889 | 2.153666715 | 0.031266318 |
| TYROBP | ENST00000466431.2 | ENSG00000254485.1 | 0.853146595 | 1.907230631 | 0.056490729 |
| TYROBP | ENST00000489077.1 | ENSG00000244198.1 | 0.807291734 | 1.79875359 | 0.072057668 |
| TYROBP | ENST00000489557.2 | ENSG00000257045.1 | 0.821684408 | 1.827171682 | 0.06767396 |
| TYROBP | ENST00000490013.1 | ENSG00000184115.12 | 0.928126029 | 2.095867636 | 0.036093934 |
| TYROBP | ENST00000493123.1 | ENSG00000242428.1 | 0.866490677 | 1.929509701 | 0.053667617 |
| TYROBP | ENST00000498358.1 | ENSG00000184115.12 | 0.852933629 | 1.88389343 | 0.059579392 |
| TYROBP | ENST00000498693.1 | ENSG00000244198.1 | 0.942201712 | 2.134613 | 0.032792635 |
| TYROBP | ENST00000505196.1 | ENSG00000248131.1 | 0.936410066 | 2.071409334 | 0.038320559 |
| TYROBP | ENST00000505556.1 | ENSG00000249409.1 | 0.950978071 | 2.115736672 | 0.03436721 |
| TYROBP | ENST00000506100.1 | ENSG00000249409.1 | 0.94844082 | 2.101602167 | 0.03558814 |
| TYROBP | ENST00000506791.1 | ENSG00000251131.1 | 0.820349802 | 1.836809814 | 0.066237977 |
| TYROBP | ENST00000508083.1 | ENSG00000249343.1 | 0.943452209 | 2.122063897 | 0.033832373 |

| | | | | | |
|---|---|---|---|---|---|
| TYROBP | ENST00000508188.1 | ENSG00000250999.1 | 0.922903615 | 2.047562455 | 0.040602891 |
| TYROBP | ENST00000509036.1 | ENSG00000251131.1 | 0.909262876 | 2.019173031 | 0.043469238 |
| TYROBP | ENST00000514411.1 | ENSG00000250882.1 | 0.82587065 | 1.864242901 | 0.062287601 |
| TYROBP | ENST00000514877.1 | ENSG00000248685.1 | 0.898116721 | 2.068466637 | 0.038596165 |
| TYROBP | ENST00000517300.1 | ENSG00000254144.2 | 0.944888313 | 2.113215575 | 0.034582321 |
| TYROBP | ENST00000519451.1 | ENSG00000253363.1 | 0.89668686 | 2.027727132 | 0.042588105 |
| TYROBP | ENST00000519852.1 | ENSG00000253716.1 | 0.863300437 | 1.918554813 | 0.055040699 |
| TYROBP | ENST00000521207.1 | ENSG00000253716.1 | 0.923302104 | 2.090650314 | 0.036559422 |
| TYROBP | ENST00000522547.1 | ENSG00000253430.1 | -0.874450166 | -1.961356366 | 0.049837466 |
| TYROBP | ENST00000522600.1 | ENSG00000246582.2 | 0.925770981 | 2.072495663 | 0.03821924 |
| TYROBP | ENST00000524335.1 | ENSG00000253716.1 | 0.894092115 | 2.008031679 | 0.044639925 |
| TYROBP | ENST00000526154.1 | ENSG00000254511.1 | 0.843189427 | 1.896032909 | 0.057955693 |
| TYROBP | ENST00000526611.1 | ENSG00000246982.2 | 0.909046607 | 2.039344024 | 0.041415705 |
| TYROBP | ENST00000528000.1 | ENSG00000254804.1 | 0.941040154 | 2.100773802 | 0.035660827 |
| TYROBP | ENST00000528887.1 | ENSG00000254501.1 | 0.8295818 | 1.842051317 | 0.065467644 |
| TYROBP | ENST00000532688.1 | ENSG00000255441.1 | 0.855207125 | 1.916430622 | 0.055310306 |
| TYROBP | ENST00000536141.1 | ENSG00000256969.1 | 0.891152972 | 1.984355008 | 0.047216278 |
| TYROBP | ENST00000537269.1 | ENSG00000257084.1 | 0.941154129 | 2.112906839 | 0.034608742 |
| TYROBP | ENST00000543072.1 | ENSG00000256092.2 | -0.986186076 | -2.200652617 | 0.027760626 |
| TYROBP | ENST00000543275.1 | ENSG00000256944.1 | 0.940551229 | 2.09525492 | 0.036148338 |
| TYROBP | ENST00000543494.1 | ENSG00000256514.1 | 0.804773419 | 1.799336634 | 0.071965447 |
| TYROBP | ENST00000545177.3 | ENSG00000230438.5 | 0.904869639 | 2.027048459 | 0.042657457 |
| TYROBP | ENST00000548722.2 | ENSG00000257194.2 | -0.81190077 | -1.809094002 | 0.070436401 |
| TYROBP | ENST00000549878.1 | ENSG00000257284.1 | 0.906720414 | 2.012951752 | 0.044119705 |
| TYROBP | ENST00000554049.1 | ENSG00000258763.1 | 0.831444644 | 1.871699882 | 0.061248136 |
| TYROBP | ENST00000558515.1 | ENSG00000259182.1 | 0.861796224 | 1.91364679 | 0.055665302 |
| TYROBP | ENST00000558575.1 | ENSG00000259687.1 | 0.851271777 | 1.911486149 | 0.055942135 |
| TYROBP | ENST00000559569.1 | ENSG00000259760.1 | -0.819993064 | -1.806173465 | 0.070891248 |
| TYROBP | ENST00000560522.1 | ENSG00000259661.1 | 0.812178026 | 1.822586459 | 0.068366048 |
| TYROBP | ENST00000561529.1 | ENSG00000260886.1 | 0.821666503 | 1.857537064 | 0.063234783 |
| TYROBP | ENST00000563044.1 | ENSG00000260978.1 | 0.823385643 | 1.845136849 | 0.065017634 |
| TYROBP | ENST00000563611.1 | ENSG00000261583.1 | 0.800449837 | 1.798705833 | 0.072065226 |
| TYROBP | ENST00000563806.1 | ENSG00000238045.5 | 0.844574088 | 1.934868297 | 0.05300646 |
| TYROBP | ENST00000565359.1 | ENSG00000260601.1 | 0.821563492 | 1.848093262 | 0.064588852 |
| TYROBP | ENST00000565823.1 | ENSG00000260686.1 | -0.940705419 | -2.091605167 | 0.03647385 |
| TYROBP | ENST00000565829.1 | ENSG00000260148.1 | 0.985702233 | 2.205294543 | 0.027433433 |
| TYROBP | ENST00000570843.1 | ENSG00000261889.1 | 0.986737958 | 2.224898611 | 0.026088045 |
| TYROBP | ENST00000570929.1 | ENSG00000262223.2 | 0.966697973 | 2.15372877 | 0.031261449 |
| TYROBP | ENST00000571815.1 | ENSG00000262810.1 | 0.853662368 | 1.909263407 | 0.05622812 |
| TYROBP | ENST00000574460.1 | ENSG00000263051.1 | 0.924140998 | 2.058997687 | 0.03949446 |
| TYROBP | ENST00000575139.1 | ENSG00000263072.1 | 0.854394442 | 1.927387058 | 0.053931411 |
| TYROBP | ENST00000576086.1 | ENSG00000262823.1 | 0.836060058 | 1.876286249 | 0.060615984 |
| TYROBP | ENST00000577064.1 | ENSG00000262823.1 | 0.923957109 | 2.04749205 | 0.040609797 |
| TYROBP | ENST00000577176.1 | ENSG00000262823.1 | 0.821652372 | 1.819469683 | 0.068839804 |
| TYROBP | ENST00000578757.1 | ENSG00000175061.13 | 0.824548848 | 1.845331875 | 0.064989276 |
| TYROBP | ENST00000578800.1 | ENSG00000264235.1 | 0.898102234 | 2.025630838 | 0.042802629 |
| TYROBP | ENST00000578936.1 | ENSG00000265547.1 | 0.955807491 | 2.136704284 | 0.032622051 |
| TYROBP | ENST00000581905.1 | ENSG00000264235.1 | 0.901803183 | 2.016679415 | 0.04372898 |
| TYROBP | ENST00000583138.1 | ENSG00000263393.1 | 0.825656645 | 1.859003319 | 0.063026668 |
| TYROBP | ENST00000585072.1 | ENSG00000263745.1 | 0.816795104 | 1.822573764 | 0.068367972 |
| TYROBP | ENST00000586051.1 | ENSG00000267576.1 | 0.905524359 | 2.007704029 | 0.044674752 |

| | | | | | |
|---|---|---|---|---|---|
| TYROBP | ENST00000589673.1 | ENSG00000267755.1 | 0.841627484 | 1.894955082 | 0.058098353 |
| TYROBP | ENST00000589817.1 | ENSG00000231616.4 | 0.847099831 | 1.890166426 | 0.058735705 |
| TYROBP | ENST00000591174.1 | ENSG00000267289.1 | 0.93191906 | 2.072906421 | 0.038180989 |
| TYROBP | ENST00000593588.1 | ENSG00000269635.1 | 0.805408194 | 1.826039241 | 0.067844351 |
| TYROBP | ENST00000593632.1 | ENSG00000180279.5 | 0.931675457 | 2.069693065 | 0.038481097 |
| TYROBP | ENST00000593642.1 | ENSG00000267858.1 | 0.843480309 | 1.872584489 | 0.061125785 |
| TYROBP | ENST00000594590.2 | ENSG00000268199.2 | 0.958696691 | 2.144000876 | 0.032032817 |
| TYROBP | ENST00000595955.1 | ENSG00000268401.1 | 0.923791068 | 2.040575328 | 0.041293057 |
| TYROBP | ENST00000597169.1 | ENSG00000269720.1 | 0.868699746 | 1.936843892 | 0.052764429 |
| TYROBP | ENST00000597309.1 | ENSG00000232098.2 | -0.893626702 | -2.001690486 | 0.04531803 |
| TYROBP | ENST00000599259.1 | ENSG00000269352.1 | 0.882603904 | 1.980394567 | 0.04765921 |
| TYROBP | ENST00000599352.1 | ENSG00000240401.4 | -0.823260506 | -1.81894742 | 0.068919452 |
| TYROBP | ENST00000600071.1 | ENSG00000269199.1 | 0.847608784 | 1.923991916 | 0.054355597 |
| TYROBP | ENST00000600234.1 | ENSG00000268078.1 | 0.910189815 | 2.048933157 | 0.040468652 |
| TYROBP | ENST00000600534.1 | ENSG00000267858.1 | 0.978186739 | 2.199722678 | 0.027826577 |
| TYROBP | ENST00000600726.1 | ENSG00000267858.1 | 0.877426632 | 1.948366297 | 0.05137115 |
| TYROBP | ENST00000600889.1 | ENSG00000232675.3 | 0.929624425 | 2.076364059 | 0.037860293 |
| TYROBP | ENST00000601033.1 | ENSG00000268401.1 | 0.910184666 | 2.036996527 | 0.04165039 |
| TYROBP | ENST00000601735.1 | ENSG00000244513.2 | 0.806099069 | 1.804027915 | 0.071226931 |
| TYROBP | ENST00000602532.1 | ENSG00000270091.1 | 0.983737755 | 2.214871004 | 0.02676892 |
| TYROBP | ENST00000604142.1 | ENSG00000271308.1 | 0.85234808 | 1.912207037 | 0.055849644 |
| TYROBP | ENST00000606068.1 | ENSG00000272342.1 | 0.90616156 | 2.054968975 | 0.039882001 |
| TYROBP | ENST00000606277.1 | ENSG00000272145.1 | 0.850534046 | 1.892255688 | 0.058456922 |
| TYROBP | ENST00000606841.1 | ENSG00000272411.1 | 0.80811368 | 1.815611134 | 0.069430045 |
| TYROBP | ENST00000607014.1 | ENSG00000272345.1 | -0.801845025 | -1.78548515 | 0.074182658 |
| TYROBP | ENST00000607224.1 | ENSG00000272521.1 | 0.835063252 | 1.86309095 | 0.062449471 |
| TYROBP | ENST00000607476.1 | ENSG00000272540.1 | 0.864740524 | 1.92065392 | 0.054775353 |
| TYROBP | ENST00000607943.1 | ENSG00000273188.1 | 0.835645125 | 1.883630954 | 0.059614912 |
| TYROBP | ENST00000608677.1 | ENSG00000273350.1 | 0.826791441 | 1.863217289 | 0.062431701 |
| TYROBP | ENST00000608940.1 | ENSG00000272763.1 | 0.903292791 | 2.013526119 | 0.04405931 |
| TYROBP | ENST00000609113.1 | ENSG00000272827.1 | 0.82210384 | 1.845030015 | 0.065033173 |
| TYROBP | ENST00000609281.1 | ENSG00000273320.1 | 0.88654449 | 1.989895361 | 0.046602464 |
| TYROBP | ENST00000610145.1 | ENSG00000273175.1 | 0.907657193 | 2.025259699 | 0.042840704 |
| TYROBP | NR_003604.2 | ZFAS1 | 0.986006604 | 2.229105424 | 0.025806891 |
| TYROBP | NR_003605.1 | ZFAS1 | 0.911798418 | 2.026438099 | 0.04271991 |
| TYROBP | NR_003606.2 | ZFAS1 | 0.983949538 | 2.194720892 | 0.028183625 |
| TYROBP | NR_026802.1 | FAM74A4 | 0.912102318 | 2.044304121 | 0.040923512 |
| TYROBP | NR_027052.1 | THAP7-AS1 | 0.848836474 | 1.903360654 | 0.056993501 |
| TYROBP | NR_027271.1 | CIRBP-AS1 | 0.972050815 | 2.17454708 | 0.029664074 |
| TYROBP | NR_027334.2 | MZF1-AS1 | 0.974369435 | 2.181762393 | 0.029127073 |
| TYROBP | NR_034037.1 | LINC00582 | -0.882497986 | -1.987354765 | 0.046883097 |
| TYROBP | NR_036480.1 | VPS9D1-AS1 | 0.883201217 | 1.975844919 | 0.048172344 |
| TYROBP | NR_036658.1 | ZFAS1 | 0.981429842 | 2.182768542 | 0.029052859 |
| TYROBP | NR_038421.1 | LINC01220 | 0.810496551 | 1.823168137 | 0.068277929 |
| TYROBP | NR_040096.1 | LOC643339 | 0.878143108 | 2.009333439 | 0.044501784 |
| TYROBP | NR_046454.1 | LINC00907 | -0.874084922 | -1.948586581 | 0.051344816 |
| TYROBP | NR_103790.1 | LINC00581 | -0.913301275 | -2.0416672 | 0.041184554 |
| TYROBP | NR_104158.1 | NRG1-IT1 | 0.936227924 | 2.084309607 | 0.037132014 |
| TYROBP | NR_105010.1 | LINC01333 | 0.924386855 | 2.071366973 | 0.038324515 |
| TYROBP | NR_108036.1 | CFAP58-AS1 | 0.90776635 | 2.001367041 | 0.04535285 |
| TYROBP | NR_109886.1 | RALY-AS1 | 0.807019423 | 1.800825995 | 0.07173031 |

| | | | | | |
|---|---|---|---|---|---|
| TYROBP | NR_110568.1 | LOC101927661 | 0.833126825 | 1.865854312 | 0.062061751 |
| TYROBP | NR_110998.1 | FAM74A4 | 0.912102318 | 2.052810938 | 0.040090917 |
| TYROBP | NR_111951.1 | LINC00869 | 0.819983144 | 1.844294409 | 0.065140246 |
| TYROBP | NR_111952.1 | LINC00869 | 0.858444602 | 1.917989982 | 0.055112281 |
| TYROBP | NR_111953.1 | LINC00869 | 0.811314891 | 1.808590326 | 0.070514673 |
| TYROBP | NR_117097.1 | LINC01353 | 0.847986941 | 1.908366521 | 0.056343861 |
| TYROBP | NR_117098.1 | LINC01353 | 0.912723776 | 2.015011411 | 0.043903453 |
| TYROBP | NR_121188.1 | PGM5P3-AS1 | -0.892673567 | -1.995036929 | 0.046038852 |
| TYROBP | NR_121189.1 | PGM5P3-AS1 | -0.931265821 | -2.104713051 | 0.035316294 |
| TYROBP | NR_126522.1 | EXOC3-AS1 | 0.86987375 | 1.933293049 | 0.053200108 |
| TYROBP | NR_130143.1 | LOC104968399 | 0.929532055 | 2.092492363 | 0.036394494 |
| TYROBP | NR_130144.1 | LOC104968399 | 0.901803183 | 2.010157128 | 0.044414561 |
| TYROBP | NR_135024.1 | LOC105369747 | 0.825512509 | 1.852741348 | 0.06391944 |
| TYROBP | NR_135032.1 | LOC105369635 | 0.941154129 | 2.127583907 | 0.033371598 |
| TYROBP | NR_135644.1 | LOC105371506 | -0.936487915 | -2.101197608 | 0.035623623 |
| U2AF2 | ENST00000411694.1 | ENSG00000225331.1 | 0.962630555 | 2.166069977 | 0.03030584 |
| U2AF2 | ENST00000415205.1 | ENSG00000182057.4 | 0.881066976 | 1.972703764 | 0.048529323 |
| U2AF2 | ENST00000417260.1 | ENSG00000231734.4 | -0.846426109 | -1.898828932 | 0.057586972 |
| U2AF2 | ENST00000419662.1 | ENSG00000228265.1 | 0.94344738 | 2.12890536 | 0.033262091 |
| U2AF2 | ENST00000420981.2 | ENSG00000230438.5 | 0.81545345 | 1.811891324 | 0.07000299 |
| U2AF2 | ENST00000421020.1 | ENSG00000231407.1 | 0.823702104 | 1.85682645 | 0.063335849 |
| U2AF2 | ENST00000422763.1 | ENSG00000231131.2 | -0.800915401 | -1.78038176 | 0.075013505 |
| U2AF2 | ENST00000425124.1 | ENSG00000232336.1 | 0.815205581 | 1.809775496 | 0.070330609 |
| U2AF2 | ENST00000425881.1 | ENSG00000239636.1 | 0.842866853 | 1.900619961 | 0.057351809 |
| U2AF2 | ENST00000426030.2 | ENSG00000228686.2 | -0.886596633 | -1.979769077 | 0.047729483 |
| U2AF2 | ENST00000426475.1 | ENSG00000239467.1 | 0.851172208 | 1.898220391 | 0.057667056 |
| U2AF2 | ENST00000430920.1 | ENSG00000234203.1 | 0.857741888 | 1.896856867 | 0.057846831 |
| U2AF2 | ENST00000431730.1 | ENSG00000237401.2 | 0.916871504 | 2.033089939 | 0.042043437 |
| U2AF2 | ENST00000433035.1 | ENSG00000230483.1 | 0.843715118 | 1.877216618 | 0.060488411 |
| U2AF2 | ENST00000433051.1 | ENSG00000233193.1 | 0.900754466 | 2.015308385 | 0.043872347 |
| U2AF2 | ENST00000438190.1 | ENSG00000227214.2 | 0.828413853 | 1.853631358 | 0.063791918 |
| U2AF2 | ENST00000439184.1 | ENSG00000233985.1 | -0.819389354 | -1.830923142 | 0.067112016 |
| U2AF2 | ENST00000440595.1 | ENSG00000228265.1 | 0.920525811 | 2.073659994 | 0.038110899 |
| U2AF2 | ENST00000441592.2 | ENSG00000224078.8 | 0.955308628 | 2.129881042 | 0.033181436 |
| U2AF2 | ENST00000442649.1 | ENSG00000234089.1 | -0.854304312 | -1.924040465 | 0.054349512 |
| U2AF2 | ENST00000442829.1 | ENSG00000225284.1 | 0.849778629 | 1.890489676 | 0.0586925 |
| U2AF2 | ENST00000446562.1 | ENSG00000233896.1 | 0.911493154 | 2.034148155 | 0.04193666 |
| U2AF2 | ENST00000451507.1 | ENSG00000229539.1 | 0.957758728 | 2.123668488 | 0.033697874 |
| U2AF2 | ENST00000452176.1 | ENSG00000223659.1 | -0.950270828 | -2.137002855 | 0.032597759 |
| U2AF2 | ENST00000457253.1 | ENSG00000225173.1 | 0.855906606 | 1.926941952 | 0.053986865 |
| U2AF2 | ENST00000457371.1 | ENSG00000237401.2 | 0.905559944 | 1.986881885 | 0.046935487 |
| U2AF2 | ENST00000458154.1 | ENSG00000235578.1 | 0.861454271 | 1.912014069 | 0.05587439 |
| U2AF2 | ENST00000463255.1 | ENSG00000243305.1 | -0.992842734 | -2.23594675 | 0.025355259 |
| U2AF2 | ENST00000466431.2 | ENSG00000254485.1 | 0.800899459 | 1.769230492 | 0.076855418 |
| U2AF2 | ENST00000488310.1 | ENSG00000240449.1 | 0.823352275 | 1.832859628 | 0.066823449 |
| U2AF2 | ENST00000489077.1 | ENSG00000244198.1 | 0.898617366 | 2.001408065 | 0.045348432 |
| U2AF2 | ENST00000489090.1 | ENSG00000240045.1 | -0.948592078 | -2.138267806 | 0.032495014 |
| U2AF2 | ENST00000489690.1 | ENSG00000243944.1 | -0.823589357 | -1.844429158 | 0.065120621 |
| U2AF2 | ENST00000498693.1 | ENSG00000244198.1 | 0.802327645 | 1.783261378 | 0.074543765 |
| U2AF2 | ENST00000503571.1 | ENSG00000249592.1 | 0.823305313 | 1.840634609 | 0.065675122 |
| U2AF2 | ENST00000503723.1 | ENSG00000250472.1 | -0.86835258 | -1.907126477 | 0.056504211 |

| | | | | | |
|---|---|---|---|---|---|
| U2AF2 | ENST00000504916.1 | ENSG00000248112.1 | -0.854901674 | -1.899470252 | 0.057502674 |
| U2AF2 | ENST00000506791.1 | ENSG00000251131.1 | 0.889236292 | 1.990344318 | 0.046553019 |
| U2AF2 | ENST00000509036.1 | ENSG00000251131.1 | 0.900937669 | 1.998225838 | 0.045692182 |
| U2AF2 | ENST00000509192.1 | ENSG00000250765.1 | 0.842570234 | 1.904655946 | 0.056824809 |
| U2AF2 | ENST00000509453.1 | ENSG00000249145.1 | 0.857948999 | 1.928534292 | 0.053788703 |
| U2AF2 | ENST00000517846.1 | ENSG00000254485.1 | 0.912893869 | 2.053760817 | 0.039998847 |
| U2AF2 | ENST00000520603.1 | ENSG00000254001.1 | -0.899897005 | -2.035790201 | 0.041771427 |
| U2AF2 | ENST00000521653.1 | ENSG00000253301.1 | 0.873740021 | 1.939502844 | 0.052440139 |
| U2AF2 | ENST00000529247.1 | ENSG00000254741.1 | 0.936405301 | 2.081817772 | 0.037359118 |
| U2AF2 | ENST00000530435.1 | ENSG00000254630.1 | 0.81592457 | 1.828736419 | 0.067439104 |
| U2AF2 | ENST00000534178.1 | ENSG00000255120.1 | 0.837497766 | 1.870912609 | 0.061357195 |
| U2AF2 | ENST00000543403.1 | ENSG00000256684.1 | -0.888636465 | -1.985461194 | 0.047093184 |
| U2AF2 | ENST00000545177.3 | ENSG00000230438.5 | 0.891394162 | 1.974611855 | 0.048312213 |
| U2AF2 | ENST00000545254.1 | ENSG00000256633.1 | 0.87704879 | 1.991964728 | 0.046374928 |
| U2AF2 | ENST00000548722.2 | ENSG00000257194.2 | -0.878521476 | -1.956070087 | 0.050456898 |
| U2AF2 | ENST00000549806.1 | ENSG00000257252.1 | 0.939434173 | 2.092884799 | 0.03635944 |
| U2AF2 | ENST00000550263.1 | ENSG00000257605.1 | 0.816090733 | 1.819423806 | 0.068846797 |
| U2AF2 | ENST00000554431.1 | ENSG00000258616.1 | -0.889872776 | -1.990409155 | 0.046545882 |
| U2AF2 | ENST00000557602.1 | ENSG00000258616.1 | -0.877089758 | -1.975735579 | 0.048184733 |
| U2AF2 | ENST00000558568.1 | ENSG00000272639.1 | 0.807828092 | 1.798841917 | 0.072043691 |
| U2AF2 | ENST00000559673.1 | ENSG00000259604.1 | 0.850308143 | 1.927434468 | 0.053925508 |
| U2AF2 | ENST00000562191.1 | ENSG00000261292.1 | -0.916059435 | -2.065304777 | 0.038894172 |
| U2AF2 | ENST00000562995.1 | ENSG00000261253.1 | 0.871500997 | 1.936140002 | 0.052850556 |
| U2AF2 | ENST00000563611.1 | ENSG00000261583.1 | 0.815033975 | 1.810738262 | 0.070181377 |
| U2AF2 | ENST00000564809.1 | ENSG00000261471.1 | 0.839334852 | 1.859926522 | 0.062895922 |
| U2AF2 | ENST00000569981.1 | ENSG00000238045.5 | 0.851849747 | 1.867281223 | 0.061862326 |
| U2AF2 | ENST00000570493.2 | ENSG00000261898.2 | 0.861917915 | 1.952220402 | 0.050912043 |
| U2AF2 | ENST00000571660.1 | ENSG00000262848.1 | 0.842027176 | 1.875363101 | 0.060742788 |
| U2AF2 | ENST00000578800.1 | ENSG00000264235.1 | 0.849304805 | 1.906050653 | 0.056643634 |
| U2AF2 | ENST00000580311.1 | ENSG00000266803.1 | -0.885248265 | -1.948332455 | 0.051375196 |
| U2AF2 | ENST00000580622.1 | ENSG00000264634.1 | 0.866279072 | 1.9497551 | 0.051205315 |
| U2AF2 | ENST00000582044.1 | ENSG00000263715.2 | 0.939944808 | 2.085002898 | 0.037069037 |
| U2AF2 | ENST00000582558.1 | ENSG00000264569.1 | 0.803932628 | 1.801245228 | 0.071664237 |
| U2AF2 | ENST00000586694.1 | ENSG00000267141.1 | 0.936464751 | 2.093247202 | 0.036327093 |
| U2AF2 | ENST00000587696.1 | ENSG00000225313.2 | 0.801928935 | 1.797142201 | 0.072313048 |
| U2AF2 | ENST00000588380.1 | ENSG00000266990.1 | 0.9905289 | 2.219421558 | 0.026458058 |
| U2AF2 | ENST00000588402.1 | ENSG00000267006.1 | -0.803131946 | -1.777471437 | 0.075490706 |
| U2AF2 | ENST00000589395.1 | ENSG00000267143.1 | 0.856659274 | 1.896109326 | 0.05794559 |
| U2AF2 | ENST00000591174.1 | ENSG00000267289.1 | 0.814053532 | 1.813130805 | 0.069811649 |
| U2AF2 | ENST00000592525.1 | ENSG00000267214.1 | 0.887583913 | 2.001366589 | 0.045352899 |
| U2AF2 | ENST00000593139.1 | ENSG00000267042.1 | 0.903573721 | 2.008229789 | 0.044618879 |
| U2AF2 | ENST00000596887.1 | ENSG00000237031.3 | -0.85251321 | -1.911760711 | 0.055906893 |
| U2AF2 | ENST00000597169.1 | ENSG00000269720.1 | 0.826856994 | 1.84734268 | 0.064697491 |
| U2AF2 | ENST00000597256.1 | ENSG00000267986.1 | 0.88716217 | 1.975772477 | 0.048180552 |
| U2AF2 | ENST00000600242.1 | ENSG00000269583.1 | 0.873430393 | 1.963073449 | 0.04963764 |
| U2AF2 | ENST00000600726.1 | ENSG00000267858.1 | 0.802069419 | 1.796291806 | 0.07244812 |
| U2AF2 | ENST00000602594.1 | ENSG00000269930.1 | -0.912939436 | -2.055422129 | 0.03983825 |
| U2AF2 | ENST00000603948.1 | ENSG00000222041.6 | 0.80009084 | 1.813290127 | 0.069787085 |
| U2AF2 | ENST00000604142.1 | ENSG00000271308.1 | 0.891430419 | 1.976455172 | 0.048103248 |
| U2AF2 | ENST00000604183.1 | ENSG00000271185.1 | 0.889182724 | 1.979182677 | 0.047795443 |
| U2AF2 | ENST00000606277.1 | ENSG00000272145.1 | 0.832635125 | 1.850210249 | 0.064283252 |

| | | | | | |
|---|---|---|---|---|---|
| U2AF2 | ENST00000606377.1 | ENSG00000272286.1 | -0.863147068 | -1.924646409 | 0.054273609 |
| U2AF2 | ENST00000606441.1 | ENSG00000272277.1 | 0.907605157 | 2.040834665 | 0.041267264 |
| U2AF2 | ENST00000606470.1 | ENSG00000271913.1 | 0.842087576 | 1.879596742 | 0.060163058 |
| U2AF2 | ENST00000606743.1 | ENSG00000272221.1 | 0.859544041 | 1.908197159 | 0.056365739 |
| U2AF2 | ENST00000606909.1 | ENSG00000271821.1 | 0.890041024 | 1.997700687 | 0.04574912 |
| U2AF2 | ENST00000606963.1 | ENSG00000272010.1 | -0.854110599 | -1.898316276 | 0.057654431 |
| U2AF2 | ENST00000607476.1 | ENSG00000272540.1 | 0.884910448 | 1.97121083 | 0.048699766 |
| U2AF2 | ENST00000607943.1 | ENSG00000273188.1 | 0.872433665 | 1.94691365 | 0.051545088 |
| U2AF2 | ENST00000608159.1 | ENSG00000273093.1 | -0.861461881 | -1.889767253 | 0.058789095 |
| U2AF2 | ENST00000608367.1 | ENSG00000273361.1 | 0.9729224 | 2.171247543 | 0.029912465 |
| U2AF2 | ENST00000608677.1 | ENSG00000273350.1 | 0.830314563 | 1.838115689 | 0.06604536 |
| U2AF2 | NR_026802.1 | FAM74A4 | 0.919638921 | 2.051896358 | 0.040179736 |
| U2AF2 | NR_026813.1 | LINC00597 | -0.844578872 | -1.877066057 | 0.060509041 |
| U2AF2 | NR_026951.1 | LINC00324 | 0.979372351 | 2.190986488 | 0.02845277 |
| U2AF2 | NR_027052.1 | THAP7-AS1 | 0.84584492 | 1.895974882 | 0.057963366 |
| U2AF2 | NR_036480.1 | VPS9D1-AS1 | 0.855141892 | 1.920819727 | 0.054754439 |
| U2AF2 | NR_037169.1 | LOC100507547 | 0.902775381 | 2.002549757 | 0.045225637 |
| U2AF2 | NR_037170.1 | LOC100507547 | 0.886513107 | 1.995325909 | 0.046007346 |
| U2AF2 | NR_038923.1 | SSSCA1-AS1 | 0.880218486 | 1.973641781 | 0.048422489 |
| U2AF2 | NR_047116.1 | HIF1A-AS1 | -0.818372718 | -1.834389029 | 0.066596267 |
| U2AF2 | NR_072981.1 | LINC00957 | 0.882097681 | 1.9620248 | 0.049759597 |
| U2AF2 | NR_072982.1 | LINC00957 | 0.8718773 | 1.932852554 | 0.053254364 |
| U2AF2 | NR_073155.1 | C1orf145 | -0.836967372 | -1.855610278 | 0.063509128 |
| U2AF2 | NR_105010.1 | LINC01333 | 0.814624817 | 1.799656791 | 0.071914848 |
| U2AF2 | NR_108106.1 | LINC01135 | 0.818060982 | 1.823434786 | 0.068237566 |
| U2AF2 | NR_109831.1 | RASSF1-AS1 | 0.846163771 | 1.886090904 | 0.059282706 |
| U2AF2 | NR_109885.1 | RALY-AS1 | 0.924383456 | 2.049118295 | 0.040450549 |
| U2AF2 | NR_109886.1 | RALY-AS1 | 0.934993587 | 2.094136917 | 0.036247785 |
| U2AF2 | NR_110630.1 | LOC101927478 | 0.824917483 | 1.846438844 | 0.064828512 |
| U2AF2 | NR_110941.1 | MIR762HG | 0.822775373 | 1.837021097 | 0.066206781 |
| U2AF2 | NR_110998.1 | FAM74A4 | 0.919638921 | 2.059726716 | 0.039424674 |
| U2AF2 | NR_111951.1 | LINC00869 | 0.826853002 | 1.853382403 | 0.063827568 |
| U2AF2 | NR_111952.1 | LINC00869 | 0.813127875 | 1.807445038 | 0.070692917 |
| U2AF2 | NR_111953.1 | LINC00869 | 0.819883086 | 1.823468744 | 0.068232427 |
| U2AF2 | NR_125957.1 | LOC101928626 | -0.950270828 | -2.104564625 | 0.035329224 |
| U2AF2 | NR_126522.1 | EXOC3-AS1 | 0.955954797 | 2.108688034 | 0.034971518 |
| U2AF2 | NR_130143.1 | LOC104968399 | 0.817645713 | 1.850368902 | 0.064260398 |
| U2AF2 | NR_134252.1 | LOC105379030 | 0.863117121 | 1.909090132 | 0.056250465 |
| U2AF2 | NR_134520.1 | LOC727993 | 0.924035411 | 2.049189593 | 0.040443579 |
| U2AF2 | NR_134579.1 | LOC105372179 | 0.908994261 | 2.045202756 | 0.040834873 |
| U2AF2 | NR_135024.1 | LOC105369747 | 0.964237241 | 2.178457209 | 0.029372015 |
| U2AF2 | NR_135097.1 | LOC105369443 | -0.888636465 | -1.996312437 | 0.045899925 |
| U2AF2 | NR_135626.1 | LOC100505585 | 0.905541239 | 2.043262818 | 0.041026428 |
| U2AF2 | NR_144459.1 | ARSD-AS1 | 0.817435261 | 1.83035441 | 0.06719696 |
| UBE2M | ENST00000415448.1 | ENSG00000228329.1 | 0.933541127 | 2.101449838 | 0.035601497 |
| UBE2M | ENST00000420828.1 | ENSG00000227718.1 | -0.808476171 | -1.812845954 | 0.069855584 |
| UBE2M | ENST00000432743.1 | ENSG00000189229.10 | -0.886789594 | -1.996090229 | 0.045924102 |
| UBE2M | ENST00000433550.1 | ENSG00000232227.1 | -0.80192825 | -1.807935284 | 0.070616573 |
| UBE2M | ENST00000446107.1 | ENSG00000227029.1 | -0.892230215 | -1.985221603 | 0.047119822 |
| UBE2M | ENST00000462300.1 | ENSG00000241912.1 | 0.89652655 | 1.985421649 | 0.047097579 |
| UBE2M | ENST00000508925.2 | ENSG00000249196.2 | 0.831778178 | 1.86758186 | 0.061820377 |

| | | | | | |
|---|---|---|---|---|---|
| UBE2M | ENST00000518044.1 | ENSG00000254275.2 | 0.811349339 | 1.815574714 | 0.069435635 |
| UBE2M | ENST00000525097.1 | ENSG00000254530.1 | -0.913155913 | -2.016578015 | 0.043739569 |
| UBE2M | ENST00000537149.1 | ENSG00000256862.1 | 0.89587384 | 1.999274364 | 0.045578676 |
| UBE2M | ENST00000545163.1 | ENSG00000256862.1 | 0.970964149 | 2.146546381 | 0.031829413 |
| UBE2M | ENST00000545357.1 | ENSG00000256862.1 | 0.905782955 | 2.029734986 | 0.042383485 |
| UBE2M | ENST00000547547.1 | ENSG00000257241.1 | 0.816314609 | 1.832576659 | 0.066865552 |
| UBE2M | ENST00000557817.1 | ENSG00000259176.1 | -0.851773304 | -1.878553407 | 0.060305498 |
| UBE2M | ENST00000559026.1 | ENSG00000259732.1 | -0.868764467 | -1.956012015 | 0.050463739 |
| UBE2M | ENST00000565441.1 | ENSG00000261013.1 | -0.843297737 | -1.903307094 | 0.057000486 |
| UBE2M | ENST00000566390.1 | ENSG00000260213.1 | -0.905260456 | -2.027425399 | 0.042618927 |
| UBE2M | ENST00000567261.1 | ENSG00000261320.1 | -0.811281498 | -1.8190875 | 0.068898082 |
| UBE2M | ENST00000568414.1 | ENSG00000260986.1 | -0.975130412 | -2.162872995 | 0.030550949 |
| UBE2M | ENST00000572193.1 | ENSG00000261872.1 | -0.835532846 | -1.868656213 | 0.06167066 |
| UBE2M | ENST00000573953.1 | ENSG00000261971.2 | -0.803947206 | -1.812433885 | 0.069919181 |
| UBE2M | ENST00000576632.1 | ENSG00000262172.1 | 0.955897854 | 2.132082774 | 0.033000042 |
| UBE2M | ENST00000587693.1 | ENSG00000267373.1 | 0.853593223 | 1.925448633 | 0.054173256 |
| UBE2M | ENST00000589754.1 | ENSG00000206129.3 | -0.87047303 | -1.961928964 | 0.049770755 |
| UBE2M | ENST00000591225.1 | ENSG00000228290.2 | -0.941556479 | -2.110011163 | 0.034857394 |
| UBE2M | ENST00000592090.1 | ENSG00000235779.3 | 0.815357153 | 1.818448391 | 0.068995628 |
| UBE2M | ENST00000592681.1 | ENSG00000228290.2 | -0.896984007 | -2.026953078 | 0.042667212 |
| UBE2M | ENST00000600644.1 | ENSG00000267313.2 | 0.886443869 | 1.977056979 | 0.04803519 |
| UBE2M | ENST00000606899.1 | ENSG00000272426.1 | 0.901328317 | 2.020543514 | 0.043327041 |
| UBE2M | ENST00000609428.1 | ENSG00000273096.1 | -0.879677399 | -1.960656663 | 0.049919088 |
| UBE2M | NR_024491.1 | LOC100128573 | -0.816244528 | -1.815434086 | 0.069457227 |
| UBE2M | NR_047115.1 | PPP2R2B-IT1 | -0.839797935 | -1.864814659 | 0.062207388 |
| UFSP1 | ENST00000390540.2 | ENSG00000254140.1 | 0.824321202 | 1.842601244 | 0.065387252 |
| UFSP1 | ENST00000414992.1 | ENSG00000233613.1 | 0.857269384 | 1.931515623 | 0.053419319 |
| UFSP1 | ENST00000421006.1 | ENSG00000234548.1 | -0.861249305 | -1.893838662 | 0.058246428 |
| UFSP1 | ENST00000422763.1 | ENSG00000231131.2 | -0.875822577 | -1.908667887 | 0.056304948 |
| UFSP1 | ENST00000423925.1 | ENSG00000223536.1 | 0.814048317 | 1.805639904 | 0.070974605 |
| UFSP1 | ENST00000425881.1 | ENSG00000239636.1 | 0.896151092 | 2.014000177 | 0.044009515 |
| UFSP1 | ENST00000426213.1 | ENSG00000223660.1 | 0.859541646 | 1.88862475 | 0.058942128 |
| UFSP1 | ENST00000427132.1 | ENSG00000232121.1 | 0.973436434 | 2.167692542 | 0.030182087 |
| UFSP1 | ENST00000428440.1 | ENSG00000232827.2 | 0.840975145 | 1.87621575 | 0.06062566 |
| UFSP1 | ENST00000429878.1 | ENSG00000224184.1 | 0.877399892 | 1.938986033 | 0.052503039 |
| UFSP1 | ENST00000432431.1 | ENSG00000234940.1 | -0.821864972 | -1.834268927 | 0.066614084 |
| UFSP1 | ENST00000432957.1 | ENSG00000231534.1 | 0.856538822 | 1.909203635 | 0.056235828 |
| UFSP1 | ENST00000434250.1 | ENSG00000234055.1 | 0.93306636 | 2.102290686 | 0.03552782 |
| UFSP1 | ENST00000434493.1 | ENSG00000224605.1 | -0.810167349 | -1.798901935 | 0.072034194 |
| UFSP1 | ENST00000435357.1 | ENSG00000225444.1 | -0.842300286 | -1.908054278 | 0.056384201 |
| UFSP1 | ENST00000437334.1 | ENSG00000226134.1 | 0.942032363 | 2.102823675 | 0.035481186 |
| UFSP1 | ENST00000437680.1 | ENSG00000237133.1 | 0.835472464 | 1.872137266 | 0.061187616 |
| UFSP1 | ENST00000439443.1 | ENSG00000236911.2 | 0.894255188 | 1.991903704 | 0.046381624 |
| UFSP1 | ENST00000439455.1 | ENSG00000233482.1 | 0.8659686 | 1.956671377 | 0.050386117 |
| UFSP1 | ENST00000440518.1 | ENSG00000226571.1 | 0.898483767 | 1.979947364 | 0.047709444 |
| UFSP1 | ENST00000440862.1 | ENSG00000223804.1 | 0.897286308 | 1.989725699 | 0.04662116 |
| UFSP1 | ENST00000445617.2 | ENSG00000225751.2 | -0.925602219 | -2.046454656 | 0.040711659 |
| UFSP1 | ENST00000445631.1 | ENSG00000231052.1 | 0.957155208 | 2.121858236 | 0.033849645 |
| UFSP1 | ENST00000446562.1 | ENSG00000233896.1 | 0.842387785 | 1.88260774 | 0.059753547 |
| UFSP1 | ENST00000448001.1 | ENSG00000229639.1 | -0.870572679 | -1.921376544 | 0.054684253 |
| UFSP1 | ENST00000448650.1 | ENSG00000223536.1 | 0.839086249 | 1.861594683 | 0.062660243 |

| | | | | | |
|---|---|---|---|---|---|
| UFSP1 | ENST00000448674.1 | ENSG00000235119.1 | 0.821727085 | 1.827928155 | 0.067560335 |
| UFSP1 | ENST00000449586.1 | ENSG00000235257.4 | 0.820150781 | 1.825877151 | 0.067868769 |
| UFSP1 | ENST00000450206.1 | ENSG00000234311.1 | 0.831427981 | 1.851291431 | 0.064127638 |
| UFSP1 | ENST00000450500.1 | ENSG00000225790.1 | 0.957729775 | 2.133779624 | 0.032860825 |
| UFSP1 | ENST00000452553.1 | ENSG00000233973.1 | -0.95342183 | -2.142450771 | 0.032157226 |
| UFSP1 | ENST00000453878.1 | ENSG00000224850.1 | -0.86923311 | -1.962434059 | 0.049711971 |
| UFSP1 | ENST00000454709.1 | ENSG00000237280.1 | 0.873799955 | 1.929948082 | 0.053613271 |
| UFSP1 | ENST00000455416.1 | ENSG00000229337.1 | 0.944968009 | 2.085185027 | 0.037052509 |
| UFSP1 | ENST00000456091.1 | ENSG00000226985.1 | 0.816976103 | 1.816796941 | 0.069248211 |
| UFSP1 | ENST00000457632.1 | ENSG00000234248.1 | -0.829256835 | -1.844200674 | 0.0651539 |
| UFSP1 | ENST00000479233.1 | ENSG00000243150.1 | -0.915305662 | -2.021977689 | 0.043178657 |
| UFSP1 | ENST00000482142.1 | ENSG00000243276.1 | -0.842989713 | -1.872976795 | 0.061071589 |
| UFSP1 | ENST00000485347.1 | ENSG00000239991.1 | 0.935075391 | 2.091415539 | 0.03649083 |
| UFSP1 | ENST00000488310.1 | ENSG00000240449.1 | 0.875014543 | 1.939982192 | 0.052381854 |
| UFSP1 | ENST00000507808.1 | ENSG00000250333.1 | 0.871453539 | 1.966721164 | 0.049215366 |
| UFSP1 | ENST00000508081.1 | ENSG00000248254.1 | 0.810753071 | 1.821612612 | 0.068513786 |
| UFSP1 | ENST00000508845.1 | ENSG00000271724.1 | -0.810824509 | -1.835142668 | 0.066484553 |
| UFSP1 | ENST00000509629.1 | ENSG00000250164.1 | 0.813935063 | 1.787633045 | 0.07383523 |
| UFSP1 | ENST00000509983.1 | ENSG00000248173.1 | 0.862239745 | 1.952220583 | 0.050912021 |
| UFSP1 | ENST00000511234.1 | ENSG00000250865.1 | 0.81824912 | 1.851948233 | 0.064033257 |
| UFSP1 | ENST00000513023.1 | ENSG00000248809.1 | -0.841935602 | -1.880958691 | 0.059977538 |
| UFSP1 | ENST00000514459.1 | ENSG00000248211.1 | 0.927642016 | 2.078359873 | 0.037676226 |
| UFSP1 | ENST00000514802.1 | ENSG00000250190.1 | -0.81663015 | -1.842334826 | 0.065426189 |
| UFSP1 | ENST00000518416.1 | ENSG00000253901.1 | -0.824029996 | -1.84870294 | 0.064500719 |
| UFSP1 | ENST00000518620.1 | ENSG00000253892.1 | 0.846144163 | 1.89208351 | 0.058479855 |
| UFSP1 | ENST00000519375.1 | ENSG00000253980.1 | -0.839413593 | -1.884796239 | 0.059457353 |
| UFSP1 | ENST00000519660.1 | ENSG00000253416.1 | -0.820712875 | -1.836321175 | 0.06631017 |
| UFSP1 | ENST00000521359.1 | ENSG00000253140.1 | -0.849006763 | -1.909031534 | 0.056258024 |
| UFSP1 | ENST00000521378.1 | ENSG00000254222.1 | 0.913485305 | 2.025821337 | 0.042783097 |
| UFSP1 | ENST00000522408.1 | ENSG00000253484.1 | -0.833891625 | -1.859607049 | 0.062941141 |
| UFSP1 | ENST00000524133.1 | ENSG00000253174.2 | 0.906339294 | 2.020341706 | 0.043347956 |
| UFSP1 | ENST00000524309.1 | ENSG00000240915.2 | 0.840678905 | 1.858388781 | 0.063113824 |
| UFSP1 | ENST00000525855.1 | ENSG00000254746.1 | 0.829617363 | 1.861911644 | 0.062615546 |
| UFSP1 | ENST00000528869.1 | ENSG00000255443.1 | -0.814128341 | -1.790049358 | 0.073445977 |
| UFSP1 | ENST00000531071.1 | ENSG00000255248.2 | 0.81552955 | 1.797607931 | 0.072239161 |
| UFSP1 | ENST00000533938.1 | ENSG00000255142.1 | 0.912574781 | 2.089356065 | 0.036675683 |
| UFSP1 | ENST00000538294.1 | ENSG00000250748.2 | 0.861695341 | 1.901576203 | 0.057226581 |
| UFSP1 | ENST00000543403.1 | ENSG00000256684.1 | -0.801731136 | -1.797942196 | 0.072186168 |
| UFSP1 | ENST00000548748.1 | ENSG00000258252.1 | 0.889363097 | 1.996169586 | 0.045915467 |
| UFSP1 | ENST00000549616.1 | ENSG00000258168.1 | -0.826174747 | -1.847407609 | 0.064688087 |
| UFSP1 | ENST00000549756.1 | ENSG00000257769.1 | 0.881303137 | 1.984892643 | 0.047156417 |
| UFSP1 | ENST00000552378.1 | ENSG00000257294.1 | 0.945063311 | 2.100440044 | 0.03569015 |
| UFSP1 | ENST00000552634.1 | ENSG00000257496.1 | 0.85823399 | 1.916167763 | 0.055343745 |
| UFSP1 | ENST00000553464.1 | ENSG00000258418.1 | -0.87012922 | -1.941142963 | 0.052240939 |
| UFSP1 | ENST00000553668.1 | ENSG00000258733.1 | -0.848723062 | -1.86865672 | 0.061670589 |
| UFSP1 | ENST00000555913.1 | ENSG00000259077.1 | -0.912509021 | -2.057677173 | 0.039621133 |
| UFSP1 | ENST00000556458.1 | ENSG00000258504.2 | 0.830636193 | 1.87043186 | 0.061423871 |
| UFSP1 | ENST00000557368.1 | ENSG00000258444.1 | 0.898520665 | 1.998844901 | 0.045625138 |
| UFSP1 | ENST00000557855.1 | ENSG00000259176.1 | -0.820898804 | -1.864191557 | 0.062294809 |
| UFSP1 | ENST00000558221.1 | ENSG00000259704.1 | 0.827282941 | 1.874007499 | 0.060929391 |
| UFSP1 | ENST00000563601.1 | ENSG00000260589.1 | 0.826960171 | 1.85898659 | 0.063029039 |

| | | | | | |
|---|---|---|---|---|---|
| UFSP1 | ENST00000565623.1 | ENSG00000261118.1 | 0.924138687 | 2.058416195 | 0.039550198 |
| UFSP1 | ENST00000566639.1 | ENSG00000261061.1 | 0.863841231 | 1.908492051 | 0.05632765 |
| UFSP1 | ENST00000569147.1 | ENSG00000261592.1 | 0.834065515 | 1.855850093 | 0.063474928 |
| UFSP1 | ENST00000569215.1 | ENSG00000260756.1 | -0.894197692 | -2.024397106 | 0.042929309 |
| UFSP1 | ENST00000571660.1 | ENSG00000262848.1 | 0.837521589 | 1.868577372 | 0.061681636 |
| UFSP1 | ENST00000572471.1 | ENSG00000262721.1 | 0.853730962 | 1.882876352 | 0.059717127 |
| UFSP1 | ENST00000573315.1 | ENSG00000270168.1 | 0.892512302 | 2.024741083 | 0.042893958 |
| UFSP1 | ENST00000576271.1 | ENSG00000263342.1 | 0.870420976 | 1.949897004 | 0.051188396 |
| UFSP1 | ENST00000580622.1 | ENSG00000264634.1 | 0.80959249 | 1.787220454 | 0.073901865 |
| UFSP1 | ENST00000585761.1 | ENSG00000267198.1 | 0.887982932 | 1.979171526 | 0.047796698 |
| UFSP1 | ENST00000586338.1 | ENSG00000219410.4 | 0.819205973 | 1.870853276 | 0.061365421 |
| UFSP1 | ENST00000586348.1 | ENSG00000267198.1 | 0.911623945 | 2.017800545 | 0.043612038 |
| UFSP1 | ENST00000587850.1 | ENSG00000267683.1 | 0.846365655 | 1.878360814 | 0.060331822 |
| UFSP1 | ENST00000588177.1 | ENSG00000234899.5 | -0.843989931 | -1.885122245 | 0.059413335 |
| UFSP1 | ENST00000589380.1 | ENSG00000267488.1 | 0.881748139 | 1.989024582 | 0.04669849 |
| UFSP1 | ENST00000592431.1 | ENSG00000267475.1 | 0.807575461 | 1.797929381 | 0.072188199 |
| UFSP1 | ENST00000592498.1 | ENSG00000267488.1 | 0.834785782 | 1.846112615 | 0.064875856 |
| UFSP1 | ENST00000593967.1 | ENSG00000232732.5 | 0.920773467 | 2.030299036 | 0.042326152 |
| UFSP1 | ENST00000596567.1 | ENSG00000226647.2 | -0.803653312 | -1.810335218 | 0.070243819 |
| UFSP1 | ENST00000596971.1 | ENSG00000269463.1 | 0.852377141 | 1.921872164 | 0.054621845 |
| UFSP1 | ENST00000597420.1 | ENSG00000269564.1 | 0.934526987 | 2.097361965 | 0.035961545 |
| UFSP1 | ENST00000602507.1 | ENSG00000270069.1 | -0.834147489 | -1.859963697 | 0.062890662 |
| UFSP1 | ENST00000602790.1 | ENSG00000270000.1 | 0.882040145 | 1.990190292 | 0.046569977 |
| UFSP1 | ENST00000603948.1 | ENSG00000222041.6 | 0.939985564 | 2.08861425 | 0.036742461 |
| UFSP1 | ENST00000605692.1 | ENSG00000270810.1 | 0.851466672 | 1.898085963 | 0.057684759 |
| UFSP1 | ENST00000606441.1 | ENSG00000272277.1 | 0.803808186 | 1.817839609 | 0.069088651 |
| UFSP1 | ENST00000606757.1 | ENSG00000237188.3 | 0.895892943 | 1.979678102 | 0.047739711 |
| UFSP1 | ENST00000606898.1 | ENSG00000272094.1 | 0.866666918 | 1.959807362 | 0.05001831 |
| UFSP1 | ENST00000606921.1 | ENSG00000272402.1 | 0.884391168 | 1.957774762 | 0.050256448 |
| UFSP1 | ENST00000608289.1 | ENSG00000272958.1 | 0.875866926 | 1.99272404 | 0.046291673 |
| UFSP1 | ENST00000608422.1 | ENSG00000272866.1 | -0.815904992 | -1.810748679 | 0.070179764 |
| UFSP1 | ENST00000609612.1 | ENSG00000273424.1 | 0.829693915 | 1.854050769 | 0.063731897 |
| UFSP1 | ENST00000609789.1 | ENSG00000272707.1 | 0.913082248 | 2.06731182 | 0.038704781 |
| UFSP1 | NR_026932.1 | PDCD4-AS1 | 0.914944595 | 2.052438257 | 0.04012709 |
| UFSP1 | NR_034131.1 | LINC00272 | 0.93230626 | 2.074536585 | 0.038029505 |
| UFSP1 | NR_040047.1 | SDCBP2-AS1 | 0.941625458 | 2.116984059 | 0.034261201 |
| UFSP1 | NR_040049.1 | SDCBP2-AS1 | 0.926440408 | 2.064595579 | 0.038961282 |
| UFSP1 | NR_040061.1 | SRP14-AS1 | 0.904330328 | 1.964546323 | 0.04946677 |
| UFSP1 | NR_073155.1 | C1orf145 | -0.83706294 | -1.875172169 | 0.060769042 |
| UFSP1 | NR_102746.1 | ROPN1L-AS1 | 0.903558634 | 2.008661141 | 0.044573082 |
| UFSP1 | NR_108046.1 | LINC00844 | -0.887682163 | -1.972123512 | 0.048595509 |
| UFSP1 | NR_109985.1 | LOC101927830 | 0.832014825 | 1.889321872 | 0.058848712 |
| UFSP1 | NR_110370.1 | STAM-AS1 | 0.863020474 | 1.956066902 | 0.050457274 |
| UFSP1 | NR_110504.1 | LOC101929572 | 0.83950139 | 1.883020969 | 0.059697526 |
| UFSP1 | NR_110559.1 | LOC101927023 | -0.88176866 | -1.948882445 | 0.051309465 |
| UFSP1 | NR_121624.1 | LOC103352541 | -0.807693431 | -1.78044729 | 0.075002788 |
| UFSP1 | NR_126041.1 | LOC101930071 | 0.926570402 | 2.057804432 | 0.03960891 |
| UFSP1 | NR_126412.1 | SCEL-AS1 | 0.81356353 | 1.840667312 | 0.065670327 |
| UFSP1 | NR_131204.1 | XACT | 0.81163318 | 1.800109347 | 0.071843374 |
| UFSP1 | NR_131243.1 | SMCR2 | 0.82852106 | 1.851527781 | 0.064093662 |
| UFSP1 | NR_133942.1 | LOC105377247 | -0.80556272 | -1.775733294 | 0.075776887 |

| | | | | | |
|---|---|---|---|---|---|
| UFSP1 | NR_134520.1 | LOC727993 | 0.823410998 | 1.856560963 | 0.063373642 |
| UFSP1 | NR_135097.1 | LOC105369443 | -0.801731136 | -1.79405609 | 0.072804217 |
| UFSP1 | NR_135239.1 | LINC01867 | -0.856767017 | -1.918111565 | 0.055096866 |
| UFSP1 | NR_135816.1 | LOC100996664 | 0.938574658 | 2.118807889 | 0.034106706 |
| UFSP1 | NR_136320.1 | LOC105373656 | 0.943800854 | 2.119754418 | 0.034026761 |
| UGT2B17 | ENST00000413311.1 | ENSG00000226276.1 | -0.872623854 | -1.951856124 | 0.050955289 |
| UGT2B17 | ENST00000420356.1 | ENSG00000227757.1 | 0.8501956 | 1.89653412 | 0.057889453 |
| UGT2B17 | ENST00000423380.1 | ENSG00000230537.1 | 0.91406189 | 2.046383796 | 0.040718625 |
| UGT2B17 | ENST00000424257.1 | ENSG00000231626.1 | -0.816183719 | -1.848381127 | 0.064547227 |
| UGT2B17 | ENST00000428391.1 | ENSG00000224691.1 | -0.938053615 | -2.095871534 | 0.036093588 |
| UGT2B17 | ENST00000432431.1 | ENSG00000234940.1 | -0.808747221 | -1.810972789 | 0.070145064 |
| UGT2B17 | ENST00000432699.1 | ENSG00000233334.2 | 0.843923973 | 1.892167673 | 0.058468644 |
| UGT2B17 | ENST00000454515.1 | ENSG00000236753.1 | -0.831712378 | -1.845258225 | 0.064999984 |
| UGT2B17 | ENST00000454622.1 | ENSG00000227757.1 | 0.8501956 | 1.886519315 | 0.059225008 |
| UGT2B17 | ENST00000486285.1 | ENSG00000241818.1 | 0.924534799 | 2.074467425 | 0.038035921 |
| UGT2B17 | ENST00000510274.1 | ENSG00000245864.2 | 0.838830386 | 1.871873704 | 0.061224078 |
| UGT2B17 | ENST00000524808.1 | ENSG00000254812.1 | -0.898636666 | -2.015873348 | 0.043813221 |
| UGT2B17 | ENST00000544663.1 | ENSG00000256281.1 | -0.852538009 | -1.891180743 | 0.058600221 |
| UGT2B17 | ENST00000567261.1 | ENSG00000261320.1 | 0.809440176 | 1.810696891 | 0.070187784 |
| UGT2B17 | ENST00000570022.1 | ENSG00000261399.1 | 0.882031756 | 1.991225292 | 0.046456125 |
| UGT2B17 | ENST00000578443.1 | ENSG00000265204.1 | -0.941120316 | -2.134638476 | 0.032790552 |
| UGT2B17 | ENST00000583122.1 | ENSG00000264695.1 | 0.821614841 | 1.846359519 | 0.064840021 |
| UGT2B17 | ENST00000593568.1 | ENSG00000228065.6 | -0.827262954 | -1.841286808 | 0.06557954 |
| UGT2B17 | ENST00000597357.1 | ENSG00000268309.1 | -0.963276196 | -2.163740783 | 0.030484249 |
| UGT2B17 | ENST00000602435.1 | ENSG00000269952.1 | -0.805865042 | -1.806601975 | 0.070824361 |
| UGT2B17 | ENST00000602741.1 | ENSG00000270061.1 | -0.881687958 | -1.978938481 | 0.047822933 |
| UGT2B17 | ENST00000610034.1 | ENSG00000272912.1 | 0.806104616 | 1.80674127 | 0.07080263 |
| UGT2B17 | NR_026713.1 | FAM182A | 0.900518209 | 1.993405949 | 0.046217013 |
| UGT2B17 | NR_046224.1 | LINC00659 | 0.865688869 | 1.935249726 | 0.052959658 |
| UGT2B17 | NR_110702.1 | SEMA3B-AS1 | -0.930002782 | -2.091193382 | 0.036510732 |
| UGT2B17 | NR_110846.1 | LOC101928674 | -0.820181366 | -1.850777469 | 0.064201574 |
| UGT2B17 | NR_121577.1 | NALT1 | -0.807309341 | -1.80479812 | 0.071106279 |
| UGT2B17 | NR_125774.1 | LINC01170 | 0.850101026 | 1.902775942 | 0.057069788 |
| UGT2B17 | NR_135251.1 | LOC101928143 | -0.822012128 | -1.845514885 | 0.064962675 |
| UGT2B28 | ENST00000415448.1 | ENSG00000228329.1 | -0.924581701 | -2.06809274 | 0.038631303 |
| UGT2B28 | ENST00000420828.1 | ENSG00000227718.1 | 0.878102632 | 1.976669929 | 0.048078952 |
| UGT2B28 | ENST00000422914.1 | ENSG00000236120.2 | -0.817620122 | -1.824953645 | 0.068008025 |
| UGT2B28 | ENST00000423193.1 | ENSG00000224239.1 | 0.850311422 | 1.904934144 | 0.056788632 |
| UGT2B28 | ENST00000432743.1 | ENSG00000189229.10 | 0.970703695 | 2.152707711 | 0.031341657 |
| UGT2B28 | ENST00000438409.1 | ENSG00000234174.1 | -0.908584126 | -2.032035277 | 0.042150085 |
| UGT2B28 | ENST00000440194.1 | ENSG00000234753.1 | -0.88290306 | -1.955195883 | 0.050559954 |
| UGT2B28 | ENST00000441809.2 | ENSG00000237445.2 | -0.886670151 | -1.998258774 | 0.045688612 |
| UGT2B28 | ENST00000442579.1 | ENSG00000228719.1 | -0.800926296 | -1.778455496 | 0.075329075 |
| UGT2B28 | ENST00000445278.1 | ENSG00000223410.1 | 0.838477293 | 1.879040181 | 0.060239007 |
| UGT2B28 | ENST00000450304.1 | ENSG00000237886.1 | -0.832059907 | -1.846613531 | 0.064803172 |
| UGT2B28 | ENST00000454965.1 | ENSG00000235435.1 | 0.898646657 | 1.999837187 | 0.045517848 |
| UGT2B28 | ENST00000457113.1 | ENSG00000227407.1 | -0.913939436 | -2.014718396 | 0.043934164 |
| UGT2B28 | ENST00000462300.1 | ENSG00000241912.1 | -0.946989179 | -2.079148752 | 0.03760368 |
| UGT2B28 | ENST00000481334.1 | ENSG00000242440.1 | 0.863772482 | 1.917389119 | 0.055188515 |
| UGT2B28 | ENST00000496247.1 | ENSG00000241882.1 | 0.85823825 | 1.939848912 | 0.052398054 |
| UGT2B28 | ENST00000504301.1 | ENSG00000250696.1 | 0.841427962 | 1.863364957 | 0.062410937 |

| | | | | | |
|---|---|---|---|---|---|
| UGT2B28 | ENST00000508825.1 | ENSG00000250775.1 | 0.909227093 | 2.037485454 | 0.041601418 |
| UGT2B28 | ENST00000509098.1 | ENSG00000250863.1 | 0.82947268 | 1.868899676 | 0.061636773 |
| UGT2B28 | ENST00000511631.1 | ENSG00000250402.1 | -0.855054846 | -1.935306576 | 0.052952686 |
| UGT2B28 | ENST00000511917.1 | ENSG00000250062.1 | 0.889026609 | 1.982425168 | 0.047431675 |
| UGT2B28 | ENST00000512036.1 | ENSG00000250993.1 | 0.817841456 | 1.836077422 | 0.066346207 |
| UGT2B28 | ENST00000515136.1 | ENSG00000251274.1 | 0.902328053 | 2.003287589 | 0.045146429 |
| UGT2B28 | ENST00000515205.1 | ENSG00000251580.1 | 0.800746585 | 1.773169241 | 0.076200667 |
| UGT2B28 | ENST00000525097.1 | ENSG00000254530.1 | 0.848539866 | 1.890886169 | 0.058639541 |
| UGT2B28 | ENST00000529266.1 | ENSG00000254468.1 | 0.933690088 | 2.114247692 | 0.034494117 |
| UGT2B28 | ENST00000539313.1 | ENSG00000256588.1 | 0.912851146 | 2.064931315 | 0.0389295 |
| UGT2B28 | ENST00000549487.1 | ENSG00000257126.1 | 0.829291309 | 1.862703472 | 0.062503997 |
| UGT2B28 | ENST00000559026.1 | ENSG00000259732.1 | 0.972318749 | 2.147437722 | 0.03175845 |
| UGT2B28 | ENST00000560268.1 | ENSG00000259287.1 | -0.930024877 | -2.0539604 | 0.039979525 |
| UGT2B28 | ENST00000568414.1 | ENSG00000260986.1 | 0.80546465 | 1.791262719 | 0.073251146 |
| UGT2B28 | ENST00000571775.1 | ENSG00000262456.1 | -0.828573212 | -1.855425921 | 0.063535429 |
| UGT2B28 | ENST00000572193.1 | ENSG00000261872.1 | 0.879312192 | 1.968003173 | 0.049067673 |
| UGT2B28 | ENST00000580085.1 | ENSG00000266490.1 | 0.890425447 | 1.968106503 | 0.049055785 |
| UGT2B28 | ENST00000580184.1 | ENSG00000264914.1 | -0.966202451 | -2.161315221 | 0.030670998 |
| UGT2B28 | ENST00000583916.1 | ENSG00000264196.1 | -0.836501599 | -1.855834688 | 0.063477125 |
| UGT2B28 | ENST00000588384.1 | ENSG00000236172.2 | -0.835769261 | -1.880069716 | 0.060098577 |
| UGT2B28 | ENST00000591225.1 | ENSG00000228290.2 | 0.886407214 | 1.997490288 | 0.045771948 |
| UGT2B28 | ENST00000592681.1 | ENSG00000228290.2 | 0.869157901 | 1.953845568 | 0.050719483 |
| UGT2B28 | ENST00000595409.1 | ENSG00000232729.3 | 0.808955206 | 1.807374427 | 0.070703919 |
| UGT2B28 | ENST00000595737.1 | ENSG00000228065.6 | 0.855213648 | 1.917272504 | 0.055203321 |
| UGT2B28 | ENST00000595972.1 | ENSG00000230333.2 | 0.822980601 | 1.831596454 | 0.067011565 |
| UGT2B28 | ENST00000601420.1 | ENSG00000269560.1 | -0.834025155 | -1.858476118 | 0.063101432 |
| UGT2B28 | ENST00000606899.1 | ENSG00000272426.1 | -0.803519648 | -1.791507518 | 0.073211889 |
| UGT2B28 | ENST00000607119.1 | ENSG00000272541.1 | 0.815407436 | 1.830114551 | 0.067232812 |
| UGT2B28 | ENST00000608264.1 | ENSG00000273473.1 | -0.827486772 | -1.864881356 | 0.062198036 |
| UGT2B28 | ENST00000608509.1 | ENSG00000273245.1 | 0.88413876 | 1.980452018 | 0.04765276 |
| UGT2B28 | ENST00000609428.1 | ENSG00000273096.1 | 0.908552278 | 1.994248638 | 0.046124889 |
| UGT2B28 | NR_047115.1 | PPP2R2B-IT1 | 0.910766269 | 2.019495123 | 0.043435784 |
| UGT2B28 | NR_102703.1 | MAGEA8-AS1 | 0.831881353 | 1.85120694 | 0.064139788 |
| UGT2B28 | NR_103830.1 | LINC00587 | 0.832115439 | 1.851169881 | 0.064145117 |
| UGT2B28 | NR_103841.1 | LINC00539 | -0.862026407 | -1.915393191 | 0.055442379 |
| UGT2B28 | NR_110160.1 | LOC100996249 | -0.893309031 | -1.98124252 | 0.047564083 |
| UGT2B28 | NR_110879.1 | LOC101929064 | 0.862571196 | 1.916733372 | 0.055271813 |
| UGT2B28 | NR_131963.1 | LVCAT5 | 0.88828918 | 1.985402232 | 0.047099738 |
| UGT2B28 | NR_134265.1 | LINC02103 | 0.802082917 | 1.792934417 | 0.072983411 |
| UGT2B28 | NR_134566.1 | LOC105372695 | 0.893394388 | 1.98650444 | 0.04697734 |
| USP17L10 | ENST00000412085.1 | ENSG00000233825.1 | 0.960952324 | 2.178381737 | 0.029377628 |
| USP17L10 | ENST00000412759.1 | ENSG00000236933.1 | 0.868084323 | 1.945391909 | 0.051727828 |
| USP17L10 | ENST00000412772.1 | ENSG00000231507.1 | 0.991746756 | 2.20802343 | 0.02724264 |
| USP17L10 | ENST00000414740.2 | ENSG00000229646.2 | 0.9324784 | 2.097467 | 0.035952255 |
| USP17L10 | ENST00000421866.1 | ENSG00000233875.1 | -0.830932832 | -1.903792976 | 0.056937152 |
| USP17L10 | ENST00000429608.1 | ENSG00000237480.1 | 0.922763468 | 2.067126034 | 0.03872228 |
| USP17L10 | ENST00000430920.1 | ENSG00000234203.1 | 0.840859812 | 1.872890176 | 0.061083552 |
| USP17L10 | ENST00000433036.1 | ENSG00000228989.1 | 0.914397279 | 2.039055612 | 0.041444478 |
| USP17L10 | ENST00000433876.2 | ENSG00000228423.2 | 0.878485724 | 1.935494872 | 0.052929597 |
| USP17L10 | ENST00000434627.1 | ENSG00000230074.1 | 0.863256167 | 1.89881266 | 0.057589112 |
| USP17L10 | ENST00000442850.1 | ENSG00000232600.2 | -0.88787506 | -2.039979043 | 0.041352413 |

| | | | | | |
|---|---|---|---|---|---|
| USP17L10 | ENST00000443380.1 | ENSG00000224371.1 | 0.827129088 | 1.833057572 | 0.06679401 |
| USP17L10 | ENST00000447343.2 | ENSG00000229299.2 | 0.814494497 | 1.834214272 | 0.066622194 |
| USP17L10 | ENST00000453051.1 | ENSG00000229407.1 | 0.943943168 | 2.119477503 | 0.034050133 |
| USP17L10 | ENST00000454530.1 | ENSG00000226649.1 | -0.875049811 | -1.983857666 | 0.047271709 |
| USP17L10 | ENST00000455010.1 | ENSG00000233079.1 | 0.852444588 | 1.909770398 | 0.056162782 |
| USP17L10 | ENST00000458194.1 | ENSG00000226193.1 | 0.854584065 | 1.900342521 | 0.057388185 |
| USP17L10 | ENST00000458443.1 | ENSG00000238232.1 | 0.842001204 | 1.912762489 | 0.055778465 |
| USP17L10 | ENST00000466431.2 | ENSG00000254485.1 | 0.895795222 | 2.004324462 | 0.045035315 |
| USP17L10 | ENST00000468165.1 | ENSG00000239480.1 | 0.851873424 | 1.925961674 | 0.05410916 |
| USP17L10 | ENST00000490013.1 | ENSG00000184115.12 | 0.967508096 | 2.135899589 | 0.032687599 |
| USP17L10 | ENST00000498358.1 | ENSG00000184115.12 | 0.987301918 | 2.173611014 | 0.029734361 |
| USP17L10 | ENST00000503034.1 | ENSG00000248936.1 | 0.87664137 | 1.964813861 | 0.049435785 |
| USP17L10 | ENST00000504301.1 | ENSG00000250696.1 | -0.834527664 | -1.858595317 | 0.063084521 |
| USP17L10 | ENST00000506394.1 | ENSG00000251665.1 | 0.887419512 | 1.99362327 | 0.04619324 |
| USP17L10 | ENST00000508188.1 | ENSG00000250999.1 | 0.838110667 | 1.871817935 | 0.061231796 |
| USP17L10 | ENST00000517846.1 | ENSG00000254485.1 | 0.847970254 | 1.894378625 | 0.058174772 |
| USP17L10 | ENST00000521207.1 | ENSG00000253716.1 | 0.853400617 | 1.911950239 | 0.055882577 |
| USP17L10 | ENST00000524942.1 | ENSG00000255553.1 | 0.981248985 | 2.199210214 | 0.027862978 |
| USP17L10 | ENST00000527086.1 | ENSG00000255182.1 | 0.840117203 | 1.879946203 | 0.06011541 |
| USP17L10 | ENST00000527757.1 | ENSG00000255109.1 | -0.843973592 | -1.891475299 | 0.058560925 |
| USP17L10 | ENST00000528000.1 | ENSG00000254804.1 | 0.945941237 | 2.152179028 | 0.031383257 |
| USP17L10 | ENST00000532249.1 | ENSG00000234899.5 | 0.801004053 | 1.810432132 | 0.0702288 |
| USP17L10 | ENST00000543072.1 | ENSG00000256092.2 | -0.870103165 | -1.97122924 | 0.048697661 |
| USP17L10 | ENST00000543275.1 | ENSG00000256944.1 | 0.899623674 | 1.989970804 | 0.046594152 |
| USP17L10 | ENST00000545177.3 | ENSG00000230438.5 | 0.808909843 | 1.803277419 | 0.071344657 |
| USP17L10 | ENST00000549806.1 | ENSG00000257252.1 | 0.803600742 | 1.80600727 | 0.070917204 |
| USP17L10 | ENST00000552525.1 | ENSG00000257286.1 | 0.884294863 | 1.966635061 | 0.049225298 |
| USP17L10 | ENST00000554049.1 | ENSG00000258763.1 | 0.863929678 | 1.965634084 | 0.049340894 |
| USP17L10 | ENST00000558515.1 | ENSG00000259182.1 | 0.880892982 | 1.958754437 | 0.050141552 |
| USP17L10 | ENST00000558575.1 | ENSG00000259687.1 | 0.89929476 | 2.018459712 | 0.043543406 |
| USP17L10 | ENST00000561529.1 | ENSG00000260886.1 | 0.81597869 | 1.83153733 | 0.067020381 |
| USP17L10 | ENST00000563806.1 | ENSG00000238045.5 | 0.905782527 | 2.028410546 | 0.042518365 |
| USP17L10 | ENST00000565359.1 | ENSG00000260601.1 | 0.870691678 | 1.950742104 | 0.051087732 |
| USP17L10 | ENST00000569025.1 | ENSG00000246379.2 | -0.887707341 | -1.992519143 | 0.046314127 |
| USP17L10 | ENST00000569459.1 | ENSG00000261346.1 | 0.868768916 | 1.912400649 | 0.055824825 |
| USP17L10 | ENST00000574460.1 | ENSG00000263051.1 | 0.920592809 | 2.045257722 | 0.040829456 |
| USP17L10 | ENST00000578800.1 | ENSG00000264235.1 | 0.922856774 | 2.058305677 | 0.0395608 |
| USP17L10 | ENST00000578936.1 | ENSG00000265547.1 | 0.945566519 | 2.111913738 | 0.034693849 |
| USP17L10 | ENST00000583138.1 | ENSG00000263393.1 | 0.954469806 | 2.196692464 | 0.028042417 |
| USP17L10 | ENST00000583916.1 | ENSG00000264196.1 | 0.841935049 | 1.883615787 | 0.059616965 |
| USP17L10 | ENST00000586051.1 | ENSG00000267576.1 | 0.800786858 | 1.797797597 | 0.072209088 |
| USP17L10 | ENST00000586503.1 | ENSG00000267205.1 | -0.818763279 | -1.844020696 | 0.065180124 |
| USP17L10 | ENST00000588182.2 | ENSG00000267453.2 | 0.92019678 | 2.04048735 | 0.04130181 |
| USP17L10 | ENST00000588290.1 | ENSG00000267751.1 | 0.929741042 | 2.078319718 | 0.037679922 |
| USP17L10 | ENST00000588908.1 | ENSG00000267751.1 | 0.948588306 | 2.166097114 | 0.030303767 |
| USP17L10 | ENST00000589457.1 | ENSG00000267751.1 | 0.821470498 | 1.851217018 | 0.064138338 |
| USP17L10 | ENST00000589673.1 | ENSG00000267755.1 | 0.953563259 | 2.144261542 | 0.032011936 |
| USP17L10 | ENST00000590292.1 | ENSG00000267751.1 | 0.892650194 | 1.989582308 | 0.046636967 |
| USP17L10 | ENST00000591836.1 | ENSG00000267776.1 | 0.817908675 | 1.811783166 | 0.070019707 |
| USP17L10 | ENST00000592413.1 | ENSG00000266933.1 | 0.948276438 | 2.119214969 | 0.034072304 |
| USP17L10 | ENST00000592518.1 | ENSG00000267786.1 | 0.879730489 | 1.970712131 | 0.048756812 |

| | | | | | |
|---|---|---|---|---|---|
| USP17L10 | ENST00000593642.1 | ENSG00000267858.1 | 0.846349719 | 1.877455497 | 0.060455691 |
| USP17L10 | ENST00000594590.2 | ENSG00000268199.2 | 0.826641567 | 1.839528849 | 0.065837439 |
| USP17L10 | ENST00000595955.1 | ENSG00000268401.1 | 0.981829872 | 2.185071892 | 0.028883576 |
| USP17L10 | ENST00000596887.1 | ENSG00000237031.3 | -0.849780822 | -1.918800657 | 0.055009567 |
| USP17L10 | ENST00000597256.1 | ENSG00000267986.1 | 0.846317963 | 1.894749399 | 0.05812561 |
| USP17L10 | ENST00000599352.1 | ENSG00000240401.4 | -0.907446838 | -2.04774716 | 0.04058478 |
| USP17L10 | ENST00000600071.1 | ENSG00000269199.1 | 0.919815044 | 2.090328163 | 0.036588331 |
| USP17L10 | ENST00000601033.1 | ENSG00000268401.1 | 0.937487622 | 2.070967981 | 0.038361788 |
| USP17L10 | ENST00000602532.1 | ENSG00000270091.1 | 0.902400058 | 1.99506467 | 0.046035827 |
| USP17L10 | ENST00000606068.1 | ENSG00000272342.1 | 0.874299136 | 1.920649558 | 0.054775903 |
| USP17L10 | ENST00000607052.1 | ENSG00000271870.1 | -0.938775489 | -2.145807947 | 0.031888304 |
| USP17L10 | ENST00000608264.1 | ENSG00000273473.1 | 0.82638942 | 1.84112068 | 0.065603876 |
| USP17L10 | ENST00000608677.1 | ENSG00000273350.1 | 0.88494783 | 1.990642095 | 0.046520249 |
| USP17L10 | ENST00000608940.1 | ENSG00000272763.1 | 0.956379172 | 2.117760353 | 0.034195369 |
| USP17L10 | ENST00000609113.1 | ENSG00000272827.1 | 0.816482272 | 1.846636681 | 0.064799814 |
| USP17L10 | ENST00000609146.1 | ENSG00000272851.1 | -0.952231434 | -2.163191805 | 0.03052643 |
| USP17L10 | ENST00000610270.1 | ENSG00000272576.1 | -0.82094891 | -1.856373608 | 0.063400324 |
| USP17L10 | NR_027052.1 | THAP7-AS1 | 0.911962854 | 2.019080065 | 0.043478898 |
| USP17L10 | NR_027271.1 | CIRBP-AS1 | 0.802074006 | 1.808922214 | 0.070463089 |
| USP17L10 | NR_034037.1 | LINC00582 | -0.821285645 | -1.832572921 | 0.066866108 |
| USP17L10 | NR_038923.1 | SSSCA1-AS1 | 0.826931095 | 1.847769106 | 0.064635752 |
| USP17L10 | NR_040096.1 | LOC643339 | 0.991084293 | 2.2529857 | 0.024260051 |
| USP17L10 | NR_046454.1 | LINC00907 | -0.858185817 | -1.925459012 | 0.054171959 |
| USP17L10 | NR_046571.1 | POTEH-AS1 | 0.805033946 | 1.82698547 | 0.067701954 |
| USP17L10 | NR_103841.1 | LINC00539 | 0.832696862 | 1.847747795 | 0.064638836 |
| USP17L10 | NR_103851.1 | TAT-AS1 | 0.811849535 | 1.79494198 | 0.072662945 |
| USP17L10 | NR_104158.1 | NRG1-IT1 | 0.815682137 | 1.811882822 | 0.070004304 |
| USP17L10 | NR_108106.1 | LINC01135 | 0.84514928 | 1.887745828 | 0.059060081 |
| USP17L10 | NR_110318.1 | MACROD2-AS1 | 0.809803539 | 1.848009486 | 0.064600971 |
| USP17L10 | NR_110635.1 | LINC00687 | 0.846304504 | 1.90193423 | 0.057179753 |
| USP17L10 | NR_111951.1 | LINC00869 | 0.894821191 | 2.0329116 | 0.042061455 |
| USP17L10 | NR_111952.1 | LINC00869 | 0.91840693 | 2.073905058 | 0.038088129 |
| USP17L10 | NR_111953.1 | LINC00869 | 0.898658796 | 2.003642792 | 0.045108338 |
| USP17L10 | NR_117097.1 | LINC01353 | 0.991746756 | 2.167387234 | 0.03020534 |
| USP17L10 | NR_117098.1 | LINC01353 | 0.96228344 | 2.146586117 | 0.031826246 |
| USP17L10 | NR_120502.1 | JARID2-AS1 | 0.811754237 | 1.806457516 | 0.070846904 |
| USP17L10 | NR_130143.1 | LOC104968399 | 0.92382621 | 2.055232985 | 0.039856507 |
| USP17L10 | NR_133930.1 | LOC105375556 | -0.808864634 | -1.806783829 | 0.070795991 |
| USP17L10 | NR_135041.1 | LOC101927038 | 0.846126086 | 1.888127559 | 0.059008828 |
| USP17L10 | NR_135644.1 | LOC105371506 | -0.847082989 | -1.883926075 | 0.059574976 |
| USP17L10 | NR_138041.1 | LINC00384 | 0.836419106 | 1.865358586 | 0.062131158 |
| USP17L13 | ENST00000412085.1 | ENSG00000233825.1 | 0.941525065 | 2.061737997 | 0.039232687 |
| USP17L13 | ENST00000412759.1 | ENSG00000236933.1 | 0.8534841 | 1.955461314 | 0.050528645 |
| USP17L13 | ENST00000412772.1 | ENSG00000231507.1 | 0.988057211 | 2.183889326 | 0.028970382 |
| USP17L13 | ENST00000414740.2 | ENSG00000229646.2 | 0.936834044 | 2.079204006 | 0.037598604 |
| USP17L13 | ENST00000421617.1 | ENSG00000237342.1 | 0.840083259 | 1.883791801 | 0.059593143 |
| USP17L13 | ENST00000421866.1 | ENSG00000233875.1 | -0.866789056 | -1.929534065 | 0.053664595 |
| USP17L13 | ENST00000429608.1 | ENSG00000237480.1 | 0.923907691 | 2.071940176 | 0.038271021 |
| USP17L13 | ENST00000433036.1 | ENSG00000228989.1 | 0.854094748 | 1.944930989 | 0.051783285 |
| USP17L13 | ENST00000433876.2 | ENSG00000228423.2 | 0.931543505 | 2.052412989 | 0.040129543 |
| USP17L13 | ENST00000434627.1 | ENSG00000230074.1 | 0.877489051 | 2.018632025 | 0.04352548 |

| | | | | | |
|---|---|---|---|---|---|
| USP17L13 | ENST00000435892.1 | ENSG00000233635.2 | 0.827648294 | 1.824097188 | 0.068137381 |
| USP17L13 | ENST00000438488.1 | ENSG00000223812.1 | 0.824112017 | 1.845029582 | 0.065033236 |
| USP17L13 | ENST00000442850.1 | ENSG00000232600.2 | -0.931817866 | -2.106856973 | 0.035129979 |
| USP17L13 | ENST00000443380.1 | ENSG00000224371.1 | 0.870896798 | 1.976001025 | 0.048154661 |
| USP17L13 | ENST00000447206.1 | ENSG00000230839.1 | 0.820817813 | 1.825278547 | 0.067959006 |
| USP17L13 | ENST00000448942.1 | ENSG00000237499.2 | 0.844756695 | 1.917394083 | 0.055187885 |
| USP17L13 | ENST00000453051.1 | ENSG00000229407.1 | 0.910825289 | 2.04866833 | 0.040494558 |
| USP17L13 | ENST00000454530.1 | ENSG00000226649.1 | -0.900711124 | -1.996941805 | 0.045831505 |
| USP17L13 | ENST00000455010.1 | ENSG00000233079.1 | 0.892517564 | 1.985704174 | 0.047066181 |
| USP17L13 | ENST00000458194.1 | ENSG00000226193.1 | 0.842907402 | 1.885155485 | 0.059408849 |
| USP17L13 | ENST00000458443.1 | ENSG00000238232.1 | 0.904014797 | 1.988562082 | 0.04674956 |
| USP17L13 | ENST00000466431.2 | ENSG00000254485.1 | 0.814503973 | 1.81410748 | 0.06966118 |
| USP17L13 | ENST00000468165.1 | ENSG00000239480.1 | 0.818768252 | 1.816892694 | 0.069233546 |
| USP17L13 | ENST00000490013.1 | ENSG00000184115.12 | 0.983337808 | 2.185755684 | 0.028833485 |
| USP17L13 | ENST00000498358.1 | ENSG00000184115.12 | 0.985799179 | 2.185572913 | 0.028846866 |
| USP17L13 | ENST00000503034.1 | ENSG00000248936.1 | 0.946885912 | 2.116451209 | 0.034306451 |
| USP17L13 | ENST00000504301.1 | ENSG00000250696.1 | -0.801865424 | -1.793752173 | 0.072852734 |
| USP17L13 | ENST00000505196.1 | ENSG00000248131.1 | 0.812612695 | 1.827469812 | 0.067629161 |
| USP17L13 | ENST00000508188.1 | ENSG00000250999.1 | 0.837443461 | 1.861281105 | 0.06270449 |
| USP17L13 | ENST00000508199.1 | ENSG00000247810.2 | 0.80122905 | 1.807897252 | 0.070622494 |
| USP17L13 | ENST00000514411.1 | ENSG00000250882.1 | 0.87366399 | 1.935537093 | 0.052924421 |
| USP17L13 | ENST00000514877.1 | ENSG00000248685.1 | 0.838268866 | 1.861153866 | 0.062722451 |
| USP17L13 | ENST00000519451.1 | ENSG00000253363.1 | 0.806942314 | 1.78063788 | 0.074971627 |
| USP17L13 | ENST00000520838.1 | ENSG00000253404.1 | 0.817122407 | 1.815876356 | 0.069389341 |
| USP17L13 | ENST00000521207.1 | ENSG00000253716.1 | 0.898390963 | 2.038686763 | 0.0414813 |
| USP17L13 | ENST00000524942.1 | ENSG00000255553.1 | 0.942111687 | 2.097776156 | 0.035924924 |
| USP17L13 | ENST00000528000.1 | ENSG00000254804.1 | 0.971102653 | 2.167394405 | 0.030204794 |
| USP17L13 | ENST00000531136.1 | ENSG00000255558.1 | -0.833052288 | -1.884556093 | 0.059489795 |
| USP17L13 | ENST00000532947.1 | ENSG00000255322.1 | 0.845832326 | 1.903593442 | 0.056963154 |
| USP17L13 | ENST00000543072.1 | ENSG00000256092.2 | -0.887377655 | -1.946559027 | 0.051587625 |
| USP17L13 | ENST00000543275.1 | ENSG00000256944.1 | 0.866977938 | 1.941283685 | 0.052223877 |
| USP17L13 | ENST00000548731.1 | ENSG00000257809.1 | 0.841155153 | 1.890235945 | 0.058726411 |
| USP17L13 | ENST00000554049.1 | ENSG00000258763.1 | 0.937663641 | 2.082370652 | 0.037308627 |
| USP17L13 | ENST00000554798.1 | ENSG00000258483.1 | 0.879810661 | 1.978839237 | 0.047834109 |
| USP17L13 | ENST00000557412.1 | ENSG00000257621.3 | -0.828793584 | -1.832018487 | 0.066948666 |
| USP17L13 | ENST00000558515.1 | ENSG00000259182.1 | 0.854249953 | 1.920335468 | 0.054815539 |
| USP17L13 | ENST00000558575.1 | ENSG00000259687.1 | 0.881848117 | 1.968447191 | 0.049016607 |
| USP17L13 | ENST00000558875.1 | ENSG00000259737.2 | 0.856638413 | 1.882974514 | 0.059703822 |
| USP17L13 | ENST00000561529.1 | ENSG00000260886.1 | 0.857187403 | 1.895323235 | 0.058049592 |
| USP17L13 | ENST00000563806.1 | ENSG00000238045.5 | 0.824382614 | 1.876515454 | 0.060584535 |
| USP17L13 | ENST00000565359.1 | ENSG00000260601.1 | 0.938090561 | 2.099854073 | 0.03574168 |
| USP17L13 | ENST00000565829.1 | ENSG00000260148.1 | 0.826690929 | 1.847924474 | 0.064613269 |
| USP17L13 | ENST00000569025.1 | ENSG00000246379.2 | -0.909445622 | -2.07246585 | 0.038222018 |
| USP17L13 | ENST00000569459.1 | ENSG00000261346.1 | 0.891086514 | 1.96684495 | 0.049201089 |
| USP17L13 | ENST00000569742.1 | ENSG00000260787.1 | 0.802283137 | 1.766819394 | 0.07725848 |
| USP17L13 | ENST00000570843.1 | ENSG00000261889.1 | 0.815718944 | 1.813568628 | 0.069744164 |
| USP17L13 | ENST00000574460.1 | ENSG00000263051.1 | 0.948026997 | 2.120094392 | 0.033998086 |
| USP17L13 | ENST00000578800.1 | ENSG00000264235.1 | 0.858845218 | 1.92232495 | 0.054564883 |
| USP17L13 | ENST00000578936.1 | ENSG00000265547.1 | 0.923907691 | 2.049266209 | 0.040436091 |
| USP17L13 | ENST00000581905.1 | ENSG00000264235.1 | 0.827805835 | 1.844907432 | 0.065051005 |
| USP17L13 | ENST00000583138.1 | ENSG00000263393.1 | 0.971545057 | 2.1516704 | 0.031423323 |

| | | | | | |
|---|---|---|---|---|---|
| USP17L13 | ENST00000583916.1 | ENSG00000264196.1 | 0.802063618 | 1.796209584 | 0.072461191 |
| USP17L13 | ENST00000586051.1 | ENSG00000267576.1 | 0.859827593 | 1.952574026 | 0.050870091 |
| USP17L13 | ENST00000586503.1 | ENSG00000267205.1 | -0.818517452 | -1.820023942 | 0.068755359 |
| USP17L13 | ENST00000588182.2 | ENSG00000267453.2 | 0.8402953 | 1.887586046 | 0.059081545 |
| USP17L13 | ENST00000588290.1 | ENSG00000267751.1 | 0.863321965 | 1.922990545 | 0.054481237 |
| USP17L13 | ENST00000588908.1 | ENSG00000267751.1 | 0.942410899 | 2.111610787 | 0.034719847 |
| USP17L13 | ENST00000589457.1 | ENSG00000267751.1 | 0.846416189 | 1.888800127 | 0.058918616 |
| USP17L13 | ENST00000589673.1 | ENSG00000267755.1 | 0.970067945 | 2.121348006 | 0.033892527 |
| USP17L13 | ENST00000590292.1 | ENSG00000267751.1 | 0.881977232 | 1.992789587 | 0.046284492 |
| USP17L13 | ENST00000591836.1 | ENSG00000267776.1 | 0.89273736 | 2.011974544 | 0.04422262 |
| USP17L13 | ENST00000592413.1 | ENSG00000266933.1 | 0.937610663 | 2.119662952 | 0.034034479 |
| USP17L13 | ENST00000594590.2 | ENSG00000268199.2 | 0.848975505 | 1.910398987 | 0.056081861 |
| USP17L13 | ENST00000595508.1 | ENSG00000269749.1 | 0.804627912 | 1.820049668 | 0.068751441 |
| USP17L13 | ENST00000595955.1 | ENSG00000268401.1 | 0.966684177 | 2.135247908 | 0.032740765 |
| USP17L13 | ENST00000599352.1 | ENSG00000240401.4 | -0.914990761 | -2.064287562 | 0.03899046 |
| USP17L13 | ENST00000600071.1 | ENSG00000269199.1 | 0.970064241 | 2.156076181 | 0.031077717 |
| USP17L13 | ENST00000601033.1 | ENSG00000268401.1 | 0.888143513 | 1.967130538 | 0.049168164 |
| USP17L13 | ENST00000601752.1 | ENSG00000268051.1 | 0.80070126 | 1.76538124 | 0.077499714 |
| USP17L13 | ENST00000602532.1 | ENSG00000270091.1 | 0.922375274 | 2.140971779 | 0.032276314 |
| USP17L13 | ENST00000606068.1 | ENSG00000272342.1 | 0.9275239 | 2.098470766 | 0.03586358 |
| USP17L13 | ENST00000606778.1 | ENSG00000271930.1 | 0.805537265 | 1.815320537 | 0.069474665 |
| USP17L13 | ENST00000607052.1 | ENSG00000271870.1 | -0.949365635 | -2.094489327 | 0.036216413 |
| USP17L13 | ENST00000608677.1 | ENSG00000273350.1 | 0.816462009 | 1.821649405 | 0.068508199 |
| USP17L13 | ENST00000608940.1 | ENSG00000272763.1 | 0.920704838 | 2.080950167 | 0.037438468 |
| USP17L13 | ENST00000609146.1 | ENSG00000272851.1 | -0.976348785 | -2.260342826 | 0.023799982 |
| USP17L13 | ENST00000609281.1 | ENSG00000273320.1 | 0.824788693 | 1.82341136 | 0.068241111 |
| USP17L13 | ENST00000610270.1 | ENSG00000272576.1 | -0.802922735 | -1.781700903 | 0.07479802 |
| USP17L13 | NR_027052.1 | THAP7-AS1 | 0.836931385 | 1.866788904 | 0.061931073 |
| USP17L13 | NR_034037.1 | LINC00582 | -0.888479119 | -2.005987027 | 0.044857632 |
| USP17L13 | NR_040096.1 | LOC643339 | 0.965571499 | 2.165339443 | 0.0303617 |
| USP17L13 | NR_046454.1 | LINC00907 | -0.92852454 | -2.070149151 | 0.038438379 |
| USP17L13 | NR_103851.1 | TAT-AS1 | 0.83389278 | 1.872602777 | 0.061123258 |
| USP17L13 | NR_104158.1 | NRG1-IT1 | 0.857638207 | 1.921924092 | 0.05461531 |
| USP17L13 | NR_110318.1 | MACROD2-AS1 | 0.883689258 | 1.997401415 | 0.045781594 |
| USP17L13 | NR_110568.1 | LOC101927661 | 0.816759477 | 1.844523378 | 0.065106902 |
| USP17L13 | NR_110635.1 | LINC00687 | 0.897799417 | 1.986120916 | 0.047019899 |
| USP17L13 | NR_111951.1 | LINC00869 | 0.814528109 | 1.823300263 | 0.068257927 |
| USP17L13 | NR_111952.1 | LINC00869 | 0.851160595 | 1.885744217 | 0.059329431 |
| USP17L13 | NR_111953.1 | LINC00869 | 0.817569321 | 1.808121502 | 0.070587592 |
| USP17L13 | NR_117097.1 | LINC01353 | 0.988057211 | 2.219288629 | 0.026467095 |
| USP17L13 | NR_117098.1 | LINC01353 | 0.977354721 | 2.104140932 | 0.035366155 |
| USP17L13 | NR_120423.1 | LOC101929140 | -0.864125962 | -1.921354855 | 0.054686986 |
| USP17L13 | NR_130143.1 | LOC104968399 | 0.877727817 | 1.983860969 | 0.047271341 |
| USP17L13 | NR_130144.1 | LOC104968399 | 0.827805835 | 1.849416135 | 0.064397747 |
| USP17L13 | NR_134245.1 | LOC105379194 | 0.81675763 | 1.790035701 | 0.073448172 |
| USP17L13 | NR_135644.1 | LOC105371506 | -0.905505958 | -2.059252564 | 0.03947005 |
| USP17L13 | NR_138041.1 | LINC00384 | 0.899026154 | 1.978288877 | 0.047896127 |
| USP17L2 | ENST00000362684.1 | ENSG00000228549.2 | 0.906277304 | 2.013744383 | 0.044036378 |
| USP17L2 | ENST00000412896.1 | ENSG00000197585.5 | 0.831205221 | 1.859182857 | 0.063001224 |
| USP17L2 | ENST00000413564.1 | ENSG00000224500.1 | 0.858125626 | 1.946135234 | 0.051638497 |
| USP17L2 | ENST00000413887.1 | ENSG00000236948.1 | 0.835792134 | 1.863321794 | 0.062417006 |

| | | | | | |
|---|---|---|---|---|---|
| USP17L2 | ENST00000413989.1 | ENSG00000242628.1 | -0.831460268 | -1.866816853 | 0.061927168 |
| USP17L2 | ENST00000417315.1 | ENSG00000242486.1 | 0.89900035 | 2.027964222 | 0.0425639 |
| USP17L2 | ENST00000419734.1 | ENSG00000234646.1 | 0.816455814 | 1.823721193 | 0.068194233 |
| USP17L2 | ENST00000421498.1 | ENSG00000237978.1 | -0.821682747 | -1.836221496 | 0.066324905 |
| USP17L2 | ENST00000421597.1 | ENSG00000227851.1 | -0.871104855 | -1.932261451 | 0.053327244 |
| USP17L2 | ENST00000422118.1 | ENSG00000231189.1 | 0.809537688 | 1.809396697 | 0.070389396 |
| USP17L2 | ENST00000422807.1 | ENSG00000227683.1 | -0.884644844 | -1.971075917 | 0.048715193 |
| USP17L2 | ENST00000424342.1 | ENSG00000234988.1 | 0.882717511 | 1.967212635 | 0.049158702 |
| USP17L2 | ENST00000425058.1 | ENSG00000226771.1 | -0.836146327 | -1.872417371 | 0.061148884 |
| USP17L2 | ENST00000426125.1 | ENSG00000223653.1 | -0.913497369 | -2.058864051 | 0.039507264 |
| USP17L2 | ENST00000426444.1 | ENSG00000239395.1 | -0.850721836 | -1.900417432 | 0.057378361 |
| USP17L2 | ENST00000426929.1 | ENSG00000230184.1 | 0.875866561 | 1.966263556 | 0.049268174 |
| USP17L2 | ENST00000429916.1 | ENSG00000227708.1 | -0.898104028 | -2.013644735 | 0.044046846 |
| USP17L2 | ENST00000430025.1 | ENSG00000233508.1 | 0.819694877 | 1.838595942 | 0.065974639 |
| USP17L2 | ENST00000430247.1 | ENSG00000232855.2 | -0.844558872 | -1.905022081 | 0.056777201 |
| USP17L2 | ENST00000430545.1 | ENSG00000237153.1 | -0.811226412 | -1.804053996 | 0.071222843 |
| USP17L2 | ENST00000430842.1 | ENSG00000230433.1 | 0.885771042 | 2.023785146 | 0.042992263 |
| USP17L2 | ENST00000433249.1 | ENSG00000236556.1 | -0.814530217 | -1.810554104 | 0.070209902 |
| USP17L2 | ENST00000435357.1 | ENSG00000225444.1 | -0.805857066 | -1.784188367 | 0.074393062 |
| USP17L2 | ENST00000435828.1 | ENSG00000235612.1 | -0.882448763 | -2.006536976 | 0.044798988 |
| USP17L2 | ENST00000436582.1 | ENSG00000236525.1 | 0.802461444 | 1.800382708 | 0.071800229 |
| USP17L2 | ENST00000439529.1 | ENSG00000236526.1 | -0.80247169 | -1.773952597 | 0.076070992 |
| USP17L2 | ENST00000441295.1 | ENSG00000233960.1 | 0.886182445 | 1.986303901 | 0.04699959 |
| USP17L2 | ENST00000441666.1 | ENSG00000230379.1 | -0.8692547 | -1.958825789 | 0.050133192 |
| USP17L2 | ENST00000441991.1 | ENSG00000231210.2 | 0.921400019 | 2.055848016 | 0.039797168 |
| USP17L2 | ENST00000442852.1 | ENSG00000237923.1 | 0.898451711 | 2.027203713 | 0.042641584 |
| USP17L2 | ENST00000443066.2 | ENSG00000237633.2 | -0.94468825 | -2.114414602 | 0.034479872 |
| USP17L2 | ENST00000443162.1 | ENSG00000234183.1 | -0.974276731 | -2.174342563 | 0.029679418 |
| USP17L2 | ENST00000444731.1 | ENSG00000227131.1 | 0.817244041 | 1.820997417 | 0.06860725 |
| USP17L2 | ENST00000445178.1 | ENSG00000234653.1 | -0.907757278 | -2.033544161 | 0.041997577 |
| USP17L2 | ENST00000445233.1 | ENSG00000233928.1 | -0.818434617 | -1.85004484 | 0.064307087 |
| USP17L2 | ENST00000447111.1 | ENSG00000231903.1 | -0.839560156 | -1.872069004 | 0.061197058 |
| USP17L2 | ENST00000448086.1 | ENSG00000237571.1 | 0.932186133 | 2.062272109 | 0.039181837 |
| USP17L2 | ENST00000448365.1 | ENSG00000231114.1 | -0.803079722 | -1.786351347 | 0.074042388 |
| USP17L2 | ENST00000449903.1 | ENSG00000223872.1 | 0.93145823 | 2.075956494 | 0.037897975 |
| USP17L2 | ENST00000451697.1 | ENSG00000233823.1 | 0.875271366 | 1.928916557 | 0.053741222 |
| USP17L2 | ENST00000453584.1 | ENSG00000233613.1 | 0.860257502 | 1.925196891 | 0.054204731 |
| USP17L2 | ENST00000454387.1 | ENSG00000223726.1 | 0.806380225 | 1.818910685 | 0.068925057 |
| USP17L2 | ENST00000456499.1 | ENSG00000237640.1 | 0.808893342 | 1.801476392 | 0.071627825 |
| USP17L2 | ENST00000456999.1 | ENSG00000230690.1 | -0.932513044 | -2.114068116 | 0.03450945 |
| USP17L2 | ENST00000477643.1 | ENSG00000241224.2 | 0.821086444 | 1.832788967 | 0.066833961 |
| USP17L2 | ENST00000479039.1 | ENSG00000241224.2 | 0.995434516 | 2.219711014 | 0.026438391 |
| USP17L2 | ENST00000500498.2 | ENSG00000245311.2 | 0.887238365 | 1.97147122 | 0.048670002 |
| USP17L2 | ENST00000503323.1 | ENSG00000249881.1 | 0.855243068 | 1.914940303 | 0.055500117 |
| USP17L2 | ENST00000503470.1 | ENSG00000248559.1 | -0.995995126 | -2.221865644 | 0.026292387 |
| USP17L2 | ENST00000503987.1 | ENSG00000250075.1 | -0.916484406 | -2.047998206 | 0.040560175 |
| USP17L2 | ENST00000504765.1 | ENSG00000249638.1 | -0.823428849 | -1.858555309 | 0.063090197 |
| USP17L2 | ENST00000505404.1 | ENSG00000249941.1 | 0.915496717 | 2.064407868 | 0.038979061 |
| USP17L2 | ENST00000505978.1 | ENSG00000249982.1 | 0.877014121 | 1.958557307 | 0.050164654 |
| USP17L2 | ENST00000506420.1 | ENSG00000250034.1 | 0.926924642 | 2.054421754 | 0.039934889 |
| USP17L2 | ENST00000506852.1 | ENSG00000250945.1 | -0.877318287 | -1.984890654 | 0.047156638 |

| | | | | | |
|---|---|---|---|---|---|
| USP17L2 | ENST00000508687.1 | ENSG00000250538.1 | -0.863103602 | -1.930803192 | 0.053507396 |
| USP17L2 | ENST00000510001.2 | ENSG00000249196.2 | 0.972648365 | 2.192452863 | 0.028346823 |
| USP17L2 | ENST00000510602.1 | ENSG00000249122.1 | 0.822108289 | 1.802384337 | 0.071484958 |
| USP17L2 | ENST00000518894.1 | ENSG00000204758.3 | -0.808630126 | -1.80958499 | 0.070360169 |
| USP17L2 | ENST00000519368.1 | ENSG00000253215.1 | -0.81203552 | -1.801120248 | 0.071683929 |
| USP17L2 | ENST00000519375.1 | ENSG00000253980.1 | -0.83101771 | -1.868853008 | 0.061643268 |
| USP17L2 | ENST00000519844.1 | ENSG00000253824.1 | 0.86876366 | 1.951250286 | 0.05102728 |
| USP17L2 | ENST00000520849.1 | ENSG00000253553.1 | -0.908908243 | -2.040093466 | 0.041341017 |
| USP17L2 | ENST00000521403.1 | ENSG00000253603.1 | 0.875546808 | 1.951832089 | 0.050958143 |
| USP17L2 | ENST00000521490.1 | ENSG00000253407.1 | 0.809829117 | 1.814516682 | 0.069598217 |
| USP17L2 | ENST00000522123.1 | ENSG00000253836.1 | -0.916577826 | -2.044321621 | 0.040921785 |
| USP17L2 | ENST00000522190.1 | ENSG00000254165.1 | -0.907039334 | -2.035771045 | 0.041773351 |
| USP17L2 | ENST00000533101.1 | ENSG00000255311.1 | -0.88899895 | -1.992078508 | 0.046362445 |
| USP17L2 | ENST00000537492.1 | ENSG00000256637.2 | -0.817543989 | -1.803336862 | 0.071335327 |
| USP17L2 | ENST00000546135.1 | ENSG00000256670.1 | 0.939431162 | 2.088827435 | 0.03672326 |
| USP17L2 | ENST00000550805.1 | ENSG00000244306.5 | 0.81731885 | 1.840036954 | 0.065762812 |
| USP17L2 | ENST00000551067.1 | ENSG00000257891.1 | 0.907864673 | 2.021846149 | 0.043192249 |
| USP17L2 | ENST00000553075.1 | ENSG00000257258.1 | -0.809329274 | -1.778038478 | 0.075397536 |
| USP17L2 | ENST00000553348.1 | ENSG00000258829.1 | 0.968128501 | 2.176571603 | 0.029512546 |
| USP17L2 | ENST00000553537.1 | ENSG00000258481.1 | 0.924291341 | 2.04916616 | 0.04044587 |
| USP17L2 | ENST00000556145.1 | ENSG00000258829.1 | 0.990455047 | 2.196871198 | 0.028029646 |
| USP17L2 | ENST00000556978.1 | ENSG00000258693.1 | -0.915803839 | -2.066486129 | 0.038782601 |
| USP17L2 | ENST00000559041.1 | ENSG00000259713.1 | -0.950170086 | -2.138589295 | 0.032468945 |
| USP17L2 | ENST00000560969.1 | ENSG00000259176.1 | -0.893601219 | -1.990489766 | 0.04653701 |
| USP17L2 | ENST00000561039.1 | ENSG00000259536.1 | 0.823664804 | 1.834945752 | 0.066513728 |
| USP17L2 | ENST00000561254.1 | ENSG00000259554.1 | -0.839719578 | -1.864926538 | 0.062191702 |
| USP17L2 | ENST00000561847.1 | ENSG00000260293.1 | 0.818258909 | 1.829741319 | 0.067288629 |
| USP17L2 | ENST00000563449.2 | ENSG00000261613.2 | -0.857374438 | -1.900337203 | 0.057388882 |
| USP17L2 | ENST00000565271.1 | ENSG00000261335.1 | 0.841284196 | 1.871448731 | 0.06128291 |
| USP17L2 | ENST00000565944.1 | ENSG00000260331.1 | -0.879467988 | -1.942620917 | 0.052061978 |
| USP17L2 | ENST00000569328.1 | ENSG00000261638.1 | -0.885218933 | -1.986722625 | 0.046953143 |
| USP17L2 | ENST00000569742.1 | ENSG00000260787.1 | -0.800685323 | -1.794631033 | 0.072712505 |
| USP17L2 | ENST00000569778.1 | ENSG00000260823.1 | 0.911060077 | 2.053893693 | 0.039985982 |
| USP17L2 | ENST00000577360.1 | ENSG00000264273.1 | -0.83245261 | -1.842347946 | 0.065424271 |
| USP17L2 | ENST00000582895.1 | ENSG00000264729.1 | 0.951315836 | 2.150271216 | 0.031533768 |
| USP17L2 | ENST00000585877.1 | ENSG00000267249.1 | 0.810456034 | 1.809164417 | 0.070425464 |
| USP17L2 | ENST00000586297.1 | ENSG00000267633.1 | -0.801072552 | -1.784070318 | 0.07441224 |
| USP17L2 | ENST00000587049.1 | ENSG00000235535.3 | 0.841318947 | 1.86647928 | 0.06197434 |
| USP17L2 | ENST00000590046.1 | ENSG00000266950.1 | 0.899312582 | 2.015586756 | 0.043843206 |
| USP17L2 | ENST00000591137.1 | ENSG00000267405.1 | -0.814783684 | -1.835107628 | 0.066489744 |
| USP17L2 | ENST00000592523.1 | ENSG00000226994.3 | -0.87237793 | -1.928404264 | 0.053804862 |
| USP17L2 | ENST00000593588.1 | ENSG00000269635.1 | -0.804538105 | -1.795213131 | 0.072619749 |
| USP17L2 | ENST00000596473.1 | ENSG00000268650.3 | -0.847082332 | -1.893476806 | 0.05829449 |
| USP17L2 | ENST00000597680.1 | ENSG00000269574.1 | -0.805477716 | -1.787853332 | 0.073799674 |
| USP17L2 | ENST00000597755.1 | ENSG00000236194.2 | -0.809958753 | -1.809697486 | 0.070342713 |
| USP17L2 | ENST00000598065.1 | ENSG00000231731.3 | 0.832583613 | 1.848253552 | 0.064565672 |
| USP17L2 | ENST00000598131.1 | ENSG00000269043.1 | -0.837506945 | -1.883682507 | 0.059607934 |
| USP17L2 | ENST00000598356.1 | ENSG00000269640.1 | -0.80054131 | -1.795428833 | 0.072585402 |
| USP17L2 | ENST00000600959.1 | ENSG00000269303.1 | -0.82853651 | -1.85391785 | 0.063750914 |
| USP17L2 | ENST00000602051.1 | ENSG00000227877.2 | 0.845892941 | 1.901194993 | 0.057276477 |
| USP17L2 | ENST00000602405.1 | ENSG00000269928.1 | 0.808961882 | 1.809848414 | 0.070319298 |

| | | | | | |
|---|---|---|---|---|---|
| USP17L2 | ENST00000602443.1 | ENSG00000270076.1 | -0.830043957 | -1.885391539 | 0.059376996 |
| USP17L2 | ENST00000602900.1 | ENSG00000270179.1 | 0.871394121 | 1.952947326 | 0.050825837 |
| USP17L2 | ENST00000603612.1 | ENSG00000270996.1 | -0.865680341 | -1.914156667 | 0.055600141 |
| USP17L2 | ENST00000603949.1 | ENSG00000270332.1 | -0.876828737 | -1.945388958 | 0.051728183 |
| USP17L2 | ENST00000604312.1 | ENSG00000270947.1 | -0.858528588 | -1.907930683 | 0.056400176 |
| USP17L2 | ENST00000606778.1 | ENSG00000271930.1 | -0.803816815 | -1.800024671 | 0.071856743 |
| USP17L2 | ENST00000607051.1 | ENSG00000271771.1 | -0.841121061 | -1.855776924 | 0.063485361 |
| USP17L2 | ENST00000607136.1 | ENSG00000267546.2 | 0.910237864 | 2.04746964 | 0.040611995 |
| USP17L2 | ENST00000607321.1 | ENSG00000272371.1 | 0.863304154 | 1.93111528 | 0.053468798 |
| USP17L2 | ENST00000607580.1 | ENSG00000272545.1 | 0.831906391 | 1.85810748 | 0.063153753 |
| USP17L2 | ENST00000608133.1 | ENSG00000273193.1 | -0.879342294 | -1.979019582 | 0.047813802 |
| USP17L2 | ENST00000608173.1 | ENSG00000197099.4 | -0.831735098 | -1.869819769 | 0.06150885 |
| USP17L2 | ENST00000609182.1 | ENSG00000273248.1 | 0.820270741 | 1.869726864 | 0.061521757 |
| USP17L2 | ENST00000609218.1 | ENSG00000272945.1 | -0.871506262 | -1.945979669 | 0.051657182 |
| USP17L2 | ENST00000609238.1 | ENSG00000272703.1 | 0.923104117 | 2.07475693 | 0.038009068 |
| USP17L2 | ENST00000609953.1 | ENSG00000272825.1 | -0.81594919 | -1.829239049 | 0.067363805 |
| USP17L2 | ENST00000610034.1 | ENSG00000272912.1 | -0.813113427 | -1.832666188 | 0.066852229 |
| USP17L2 | NR_002765.2 | ASAP1-IT1 | -0.850438101 | -1.883412216 | 0.059644526 |
| USP17L2 | NR_024470.1 | SLCO4A1-AS1 | 0.812631666 | 1.808897522 | 0.070466925 |
| USP17L2 | NR_027067.1 | LINC00114 | 0.843732292 | 1.890048162 | 0.058751519 |
| USP17L2 | NR_027425.1 | FAM66D | 0.836360607 | 1.846485963 | 0.064821676 |
| USP17L2 | NR_027440.1 | LOC100272217 | 0.81560379 | 1.840962341 | 0.065627078 |
| USP17L2 | NR_046556.1 | RBMS3-AS1 | -0.937630093 | -2.087764543 | 0.036819079 |
| USP17L2 | NR_046748.1 | ARHGAP31-AS1 | -0.890972227 | -2.000732676 | 0.045421206 |
| USP17L2 | NR_046783.1 | KCND3-IT1 | 0.812651385 | 1.822963545 | 0.068308912 |
| USP17L2 | NR_047040.1 | LINC00424 | -0.836557082 | -1.895884003 | 0.057975385 |
| USP17L2 | NR_102737.1 | LINC00911 | 0.920759514 | 2.04862504 | 0.040498794 |
| USP17L2 | NR_102738.1 | LINC00911 | 0.958672188 | 2.164490868 | 0.030426697 |
| USP17L2 | NR_109975.1 | ARNTL2-AS1 | 0.832703203 | 1.872315153 | 0.061163016 |
| USP17L2 | NR_110007.1 | ADNP-AS1 | -0.93358276 | -2.097141345 | 0.035981065 |
| USP17L2 | NR_110008.1 | ADNP-AS1 | -0.809747353 | -1.820821131 | 0.068634051 |
| USP17L2 | NR_110009.1 | ADNP-AS1 | -0.809747353 | -1.811047867 | 0.070133442 |
| USP17L2 | NR_110284.1 | LOC101927907 | -0.907757278 | -2.038008235 | 0.041549109 |
| USP17L2 | NR_110504.1 | LOC101929572 | 0.82511928 | 1.844791694 | 0.065067846 |
| USP17L2 | NR_120330.1 | LOC101928227 | 0.801976059 | 1.78793064 | 0.073787199 |
| USP17L2 | NR_120466.1 | LINC01489 | -0.871248238 | -1.968286399 | 0.049035094 |
| USP17L2 | NR_125769.1 | LINC01269 | 0.892774412 | 2.011765876 | 0.044244623 |
| USP17L2 | NR_126354.1 | LINC01331 | 0.923565974 | 2.082287569 | 0.037316211 |
| USP17L2 | NR_126370.1 | GACAT1 | 0.883070364 | 1.98948313 | 0.046647902 |
| USP17L2 | NR_126409.1 | LINC00376 | -0.870053979 | -1.941931682 | 0.052145371 |
| USP17L2 | NR_130916.1 | LOC105274304 | 0.833557926 | 1.888494869 | 0.058959546 |
| USP17L2 | NR_133907.1 | HLA-DQB1-AS1 | -0.872242212 | -1.934755691 | 0.053020283 |
| USP17L2 | NR_134245.1 | LOC105379194 | -0.802702317 | -1.80988395 | 0.070313786 |
| USP17L2 | NR_134610.1 | LOC105375014 | 0.881001841 | 2.001393817 | 0.045349967 |
| USP17L2 | NR_134910.1 | LOC102725254 | -0.889019505 | -1.976409898 | 0.048108371 |
| USP17L2 | NR_135076.1 | LOC102723838 | -0.881948965 | -1.966842282 | 0.049201396 |
| USP17L2 | NR_135239.1 | LINC01867 | -0.800260415 | -1.791227083 | 0.073256862 |
| USP17L22 | ENST00000398777.3 | ENSG00000240152.2 | -0.968296801 | -2.154069521 | 0.03123472 |
| USP17L22 | ENST00000413650.1 | ENSG00000230880.2 | -0.81503569 | -1.851186171 | 0.064142775 |
| USP17L22 | ENST00000413989.1 | ENSG00000242628.1 | -0.92927906 | -2.096317905 | 0.036053999 |
| USP17L22 | ENST00000416401.1 | ENSG00000237756.1 | 0.807702143 | 1.794218382 | 0.072778319 |

| | | | | | |
|---|---|---|---|---|---|
| USP17L22 | ENST00000417315.1 | ENSG00000242486.1 | 0.838924425 | 1.879353081 | 0.060196298 |
| USP17L22 | ENST00000417426.1 | ENSG00000233145.1 | -0.965407831 | -2.171564455 | 0.02988853 |
| USP17L22 | ENST00000418741.1 | ENSG00000227332.1 | -0.893667721 | -2.004055782 | 0.045064085 |
| USP17L22 | ENST00000421252.2 | ENSG00000250258.1 | 0.965493443 | 2.166427327 | 0.030278548 |
| USP17L22 | ENST00000421498.1 | ENSG00000237978.1 | -0.949966758 | -2.140878835 | 0.03228381 |
| USP17L22 | ENST00000421597.1 | ENSG00000227851.1 | -0.803918449 | -1.785416856 | 0.074193727 |
| USP17L22 | ENST00000422697.1 | ENSG00000236414.1 | 0.899794469 | 2.01390716 | 0.044019282 |
| USP17L22 | ENST00000423667.1 | ENSG00000225970.1 | -0.854633346 | -1.895052078 | 0.058085503 |
| USP17L22 | ENST00000423925.1 | ENSG00000223536.1 | 0.827016238 | 1.840746219 | 0.065658757 |
| USP17L22 | ENST00000424241.1 | ENSG00000237311.1 | 0.873318951 | 1.964013556 | 0.04952852 |
| USP17L22 | ENST00000426504.1 | ENSG00000234190.1 | 0.880274303 | 1.978940505 | 0.047822705 |
| USP17L22 | ENST00000427064.1 | ENSG00000238031.1 | -0.946392046 | -2.123043691 | 0.033750191 |
| USP17L22 | ENST00000429796.1 | ENSG00000231858.1 | 0.828605879 | 1.862403739 | 0.062546202 |
| USP17L22 | ENST00000429878.1 | ENSG00000224184.1 | 0.801219402 | 1.769398032 | 0.076827474 |
| USP17L22 | ENST00000433614.1 | ENSG00000228534.1 | 0.904449139 | 2.009874314 | 0.044444493 |
| USP17L22 | ENST00000434493.1 | ENSG00000224605.1 | -0.935941772 | -2.097029456 | 0.035990968 |
| USP17L22 | ENST00000435271.1 | ENSG00000231132.1 | 0.865610788 | 1.944884641 | 0.051788864 |
| USP17L22 | ENST00000436582.1 | ENSG00000236525.1 | 0.844675209 | 1.905629858 | 0.056698245 |
| USP17L22 | ENST00000436982.2 | ENSG00000235335.2 | 0.859922105 | 1.910298069 | 0.056094846 |
| USP17L22 | ENST00000438107.1 | ENSG00000234449.2 | -0.891760212 | -2.020950293 | 0.043284911 |
| USP17L22 | ENST00000439186.1 | ENSG00000237076.1 | -0.888092576 | -1.99770779 | 0.045748349 |
| USP17L22 | ENST00000439529.1 | ENSG00000236526.1 | -0.910088163 | -2.031644632 | 0.042189645 |
| USP17L22 | ENST00000440862.1 | ENSG00000223804.1 | 0.861022267 | 1.925493851 | 0.054167604 |
| USP17L22 | ENST00000440947.1 | ENSG00000225472.1 | -0.861279664 | -1.910267892 | 0.05609873 |
| USP17L22 | ENST00000442579.1 | ENSG00000228719.1 | 0.907929913 | 2.025772204 | 0.042788134 |
| USP17L22 | ENST00000444665.1 | ENSG00000228852.2 | -0.855112498 | -1.880111851 | 0.060092836 |
| USP17L22 | ENST00000447183.2 | ENSG00000271593.1 | -0.897715611 | -1.982926867 | 0.047375599 |
| USP17L22 | ENST00000447514.1 | ENSG00000236753.1 | -0.824578046 | -1.838371339 | 0.066007705 |
| USP17L22 | ENST00000448086.1 | ENSG00000237571.1 | 0.802581948 | 1.792847446 | 0.072997321 |
| USP17L22 | ENST00000450206.1 | ENSG00000234311.1 | 0.84563939 | 1.880074889 | 0.060097872 |
| USP17L22 | ENST00000450848.1 | ENSG00000225539.1 | -0.912331665 | -2.064630608 | 0.038957965 |
| USP17L22 | ENST00000451656.1 | ENSG00000228417.1 | -0.808438211 | -1.804171891 | 0.071204364 |
| USP17L22 | ENST00000453878.1 | ENSG00000224850.1 | -0.905599836 | -2.031670782 | 0.042186996 |
| USP17L22 | ENST00000455699.1 | ENSG00000240996.1 | -0.812822487 | -1.84616073 | 0.064868871 |
| USP17L22 | ENST00000457169.1 | ENSG00000232408.1 | -0.962483662 | -2.183146564 | 0.029025018 |
| USP17L22 | ENST00000457975.2 | ENSG00000236744.2 | 0.854438931 | 1.898023233 | 0.057693022 |
| USP17L22 | ENST00000458661.2 | ENSG00000236467.3 | -0.856832773 | -1.918660414 | 0.055027324 |
| USP17L22 | ENST00000469931.2 | ENSG00000272030.1 | -0.865251282 | -1.908572602 | 0.056317249 |
| USP17L22 | ENST00000481334.1 | ENSG00000242440.1 | -0.891152945 | -2.005711882 | 0.044886997 |
| USP17L22 | ENST00000482142.1 | ENSG00000243276.1 | -0.852206256 | -1.935657402 | 0.052909675 |
| USP17L22 | ENST00000488040.1 | ENSG00000243176.1 | -0.892049782 | -1.982443937 | 0.047429576 |
| USP17L22 | ENST00000502421.1 | ENSG00000250284.1 | 0.925894914 | 2.060691296 | 0.039332501 |
| USP17L22 | ENST00000503938.1 | ENSG00000246095.2 | -0.942709846 | -2.121682556 | 0.033864404 |
| USP17L22 | ENST00000504344.1 | ENSG00000251438.1 | -0.845025231 | -1.885919445 | 0.059305811 |
| USP17L22 | ENST00000504795.1 | ENSG00000250723.1 | -0.941995947 | -2.09264227 | 0.0363811 |
| USP17L22 | ENST00000506059.1 | ENSG00000248311.1 | -0.840398135 | -1.861441973 | 0.062681788 |
| USP17L22 | ENST00000506305.1 | ENSG00000249994.1 | 0.943522827 | 2.133487836 | 0.032884728 |
| USP17L22 | ENST00000506852.1 | ENSG00000250945.1 | -0.853219959 | -1.923230963 | 0.05445105 |
| USP17L22 | ENST00000508845.1 | ENSG00000271724.1 | -0.95669171 | -2.13664426 | 0.032626937 |
| USP17L22 | ENST00000509983.1 | ENSG00000248173.1 | 0.807781135 | 1.822950821 | 0.06831084 |
| USP17L22 | ENST00000511603.1 | ENSG00000249892.1 | -0.901117661 | -2.002848644 | 0.045193537 |

| | | | | | |
|---|---|---|---|---|---|
| USP17L22 | ENST00000511917.1 | ENSG00000250062.1 | -0.807857216 | -1.842065578 | 0.065465558 |
| USP17L22 | ENST00000513179.1 | ENSG00000251580.1 | -0.866947355 | -1.919956229 | 0.054863429 |
| USP17L22 | ENST00000517716.1 | ENSG00000253515.1 | 0.951728318 | 2.121348724 | 0.033892467 |
| USP17L22 | ENST00000518260.1 | ENSG00000253628.1 | 0.83691292 | 1.862774259 | 0.062494033 |
| USP17L22 | ENST00000518416.1 | ENSG00000253901.1 | -0.834780194 | -1.864213878 | 0.062291675 |
| USP17L22 | ENST00000518620.1 | ENSG00000253892.1 | 0.856023794 | 1.931447494 | 0.053427737 |
| USP17L22 | ENST00000519660.1 | ENSG00000253416.1 | -0.930502225 | -2.092594878 | 0.036385334 |
| USP17L22 | ENST00000519695.1 | ENSG00000253507.1 | -0.832419776 | -1.862397674 | 0.062547057 |
| USP17L22 | ENST00000519844.1 | ENSG00000253824.1 | 0.854684101 | 1.922965772 | 0.054484349 |
| USP17L22 | ENST00000521490.1 | ENSG00000253407.1 | 0.925028468 | 2.049754926 | 0.040388352 |
| USP17L22 | ENST00000524275.1 | ENSG00000253507.1 | -0.864499254 | -1.9384567 | 0.052567529 |
| USP17L22 | ENST00000524818.1 | ENSG00000254473.1 | -0.934736084 | -2.08815634 | 0.036783734 |
| USP17L22 | ENST00000525133.1 | ENSG00000255375.1 | -0.967388269 | -2.162697105 | 0.030564484 |
| USP17L22 | ENST00000526186.1 | ENSG00000254510.1 | -0.894734713 | -1.984461413 | 0.047204426 |
| USP17L22 | ENST00000526935.1 | ENSG00000255372.1 | -0.877052504 | -1.95318458 | 0.050797728 |
| USP17L22 | ENST00000529875.1 | ENSG00000254404.1 | 0.833338284 | 1.852710382 | 0.063923881 |
| USP17L22 | ENST00000533938.1 | ENSG00000255142.1 | 0.811727508 | 1.810854671 | 0.070163351 |
| USP17L22 | ENST00000535324.1 | ENSG00000255968.1 | -0.993158538 | -2.237083436 | 0.025280887 |
| USP17L22 | ENST00000536529.1 | ENSG00000256422.1 | -0.806367898 | -1.797277889 | 0.072291515 |
| USP17L22 | ENST00000538294.1 | ENSG00000250748.2 | 0.869294965 | 1.942340515 | 0.052095891 |
| USP17L22 | ENST00000544089.1 | ENSG00000256273.1 | -0.892198214 | -2.004086315 | 0.045060815 |
| USP17L22 | ENST00000547175.1 | ENSG00000257395.1 | 0.849257815 | 1.903147099 | 0.057021354 |
| USP17L22 | ENST00000549140.1 | ENSG00000258332.1 | -0.832209542 | -1.82656486 | 0.067765221 |
| USP17L22 | ENST00000549329.1 | ENSG00000224189.2 | -0.872047425 | -1.948063048 | 0.05140742 |
| USP17L22 | ENST00000549756.1 | ENSG00000257769.1 | 0.86275996 | 1.940939226 | 0.052265649 |
| USP17L22 | ENST00000552156.1 | ENSG00000224189.2 | -0.852178435 | -1.909563379 | 0.056189454 |
| USP17L22 | ENST00000552469.1 | ENSG00000258325.1 | -0.858801204 | -1.906024074 | 0.056647082 |
| USP17L22 | ENST00000556458.1 | ENSG00000258504.2 | 0.837585122 | 1.872113627 | 0.061190885 |
| USP17L22 | ENST00000556786.1 | ENSG00000258525.1 | 0.84829337 | 1.902352057 | 0.057125144 |
| USP17L22 | ENST00000556978.1 | ENSG00000258693.1 | -0.836389598 | -1.851425771 | 0.064108324 |
| USP17L22 | ENST00000557855.1 | ENSG00000259176.1 | -0.831360972 | -1.864396073 | 0.062266104 |
| USP17L22 | ENST00000557903.1 | ENSG00000259182.1 | -0.805438443 | -1.792492531 | 0.073054105 |
| USP17L22 | ENST00000558141.1 | ENSG00000259594.1 | -0.959477998 | -2.155174943 | 0.031148147 |
| USP17L22 | ENST00000558312.1 | ENSG00000259176.1 | -0.95133642 | -2.097368465 | 0.03596097 |
| USP17L22 | ENST00000558896.1 | ENSG00000259176.1 | -0.875359437 | -1.992892079 | 0.046273266 |
| USP17L22 | ENST00000559003.1 | ENSG00000259520.1 | -0.894735504 | -2.018872993 | 0.043500422 |
| USP17L22 | ENST00000560134.1 | ENSG00000259176.1 | -0.835990606 | -1.863426418 | 0.062402296 |
| USP17L22 | ENST00000560193.1 | ENSG00000259176.1 | -0.845030803 | -1.894304903 | 0.058184551 |
| USP17L22 | ENST00000563408.1 | ENSG00000260733.1 | -0.931424619 | -2.093539864 | 0.036300989 |
| USP17L22 | ENST00000563601.1 | ENSG00000260589.1 | 0.831263176 | 1.867098261 | 0.061887867 |
| USP17L22 | ENST00000563841.1 | ENSG00000261029.1 | -0.855943376 | -1.955959102 | 0.050469972 |
| USP17L22 | ENST00000565271.1 | ENSG00000261335.1 | 0.900891969 | 1.995756935 | 0.045960386 |
| USP17L22 | ENST00000565965.1 | ENSG00000261172.1 | -0.894591218 | -1.98357957 | 0.047302729 |
| USP17L22 | ENST00000575139.1 | ENSG00000263072.1 | -0.827195999 | -1.846466879 | 0.064824444 |
| USP17L22 | ENST00000577678.1 | ENSG00000265415.1 | -0.855999069 | -1.912065237 | 0.055867827 |
| USP17L22 | ENST00000578265.1 | ENSG00000214719.7 | -0.854483834 | -1.925285418 | 0.054193661 |
| USP17L22 | ENST00000581940.1 | ENSG00000265484.1 | -0.814131627 | -1.823410358 | 0.068241263 |
| USP17L22 | ENST00000581996.1 | ENSG00000265778.1 | -0.812986462 | -1.799531934 | 0.071934577 |
| USP17L22 | ENST00000586010.1 | ENSG00000267606.1 | -0.859206878 | -1.926307727 | 0.054065961 |
| USP17L22 | ENST00000588177.1 | ENSG00000234899.5 | -0.886534737 | -2.002019583 | 0.045282625 |
| USP17L22 | ENST00000588799.1 | ENSG00000267275.1 | -0.837529879 | -1.841537647 | 0.065542809 |

| | | | | | |
|---|---|---|---|---|---|
| USP17L22 | ENST00000591137.1 | ENSG00000267405.1 | -0.878751611 | -1.963785891 | 0.049554927 |
| USP17L22 | ENST00000592100.1 | ENSG00000226686.3 | 0.801040251 | 1.820575091 | 0.068671472 |
| USP17L22 | ENST00000592431.1 | ENSG00000267475.1 | 0.848817132 | 1.888104763 | 0.059011888 |
| USP17L22 | ENST00000592523.1 | ENSG00000226994.3 | -0.883457872 | -1.991014579 | 0.046479284 |
| USP17L22 | ENST00000594762.1 | ENSG00000231898.4 | -0.927703234 | -2.090135535 | 0.036605626 |
| USP17L22 | ENST00000595737.1 | ENSG00000228065.6 | -0.803884798 | -1.801046218 | 0.071695596 |
| USP17L22 | ENST00000595892.1 | ENSG00000269640.1 | -0.869584675 | -1.932861074 | 0.053253314 |
| USP17L22 | ENST00000597550.1 | ENSG00000269051.1 | -0.844584723 | -1.883621493 | 0.059616193 |
| USP17L22 | ENST00000598356.1 | ENSG00000269640.1 | -0.967417107 | -2.176876063 | 0.029489816 |
| USP17L22 | ENST00000598950.1 | ENSG00000269736.1 | -0.917231708 | -2.062742805 | 0.039137071 |
| USP17L22 | ENST00000600489.1 | ENSG00000231898.4 | -0.822033352 | -1.827307083 | 0.067653611 |
| USP17L22 | ENST00000600959.1 | ENSG00000269303.1 | -0.8852152 | -2.005161053 | 0.044945832 |
| USP17L22 | ENST00000601420.1 | ENSG00000269560.1 | 0.875608154 | 1.995689228 | 0.04596776 |
| USP17L22 | ENST00000602592.1 | ENSG00000270049.1 | -0.956298346 | -2.136503738 | 0.032638377 |
| USP17L22 | ENST00000602790.1 | ENSG00000270000.1 | 0.901639662 | 2.012918252 | 0.04412323 |
| USP17L22 | ENST00000602835.1 | ENSG00000270096.1 | -0.866849025 | -1.938206915 | 0.052597983 |
| USP17L22 | ENST00000602872.1 | ENSG00000270067.1 | -0.907057088 | -2.007370359 | 0.044710243 |
| USP17L22 | ENST00000602900.1 | ENSG00000270179.1 | 0.803613413 | 1.793965744 | 0.072818637 |
| USP17L22 | ENST00000602954.1 | ENSG00000269906.1 | -0.951270883 | -2.126159337 | 0.033489995 |
| USP17L22 | ENST00000603612.1 | ENSG00000270996.1 | -0.860851402 | -1.913605288 | 0.055670609 |
| USP17L22 | ENST00000605021.1 | ENSG00000271401.1 | -0.879331007 | -1.957404243 | 0.05029996 |
| USP17L22 | ENST00000606374.1 | ENSG00000272312.1 | 0.852545463 | 1.891394461 | 0.058571707 |
| USP17L22 | ENST00000606457.1 | ENSG00000271830.1 | -0.906094723 | -2.045200638 | 0.040835082 |
| USP17L22 | ENST00000607594.1 | ENSG00000271766.1 | -0.955340513 | -2.158782569 | 0.030867039 |
| USP17L22 | ENST00000607769.1 | ENSG00000272438.1 | 0.858897326 | 1.923821497 | 0.054376962 |
| USP17L22 | ENST00000608085.1 | ENSG00000231898.4 | -0.811539528 | -1.819248733 | 0.068873491 |
| USP17L22 | ENST00000608173.1 | ENSG00000197099.4 | -0.865640592 | -1.962673372 | 0.049684139 |
| USP17L22 | ENST00000608289.1 | ENSG00000272958.1 | 0.817992016 | 1.833534527 | 0.066723119 |
| USP17L22 | ENST00000608422.1 | ENSG00000272866.1 | -0.887366127 | -1.959880276 | 0.050009786 |
| USP17L22 | ENST00000608509.1 | ENSG00000273245.1 | -0.849584211 | -1.89408518 | 0.058213705 |
| USP17L22 | ENST00000609067.1 | ENSG00000272849.1 | -0.924573232 | -2.052150043 | 0.040155083 |
| USP17L22 | ENST00000609238.1 | ENSG00000272703.1 | 0.839610043 | 1.871835533 | 0.061229361 |
| USP17L22 | ENST00000609725.1 | ENSG00000231898.4 | -0.856936034 | -1.932810987 | 0.053259486 |
| USP17L22 | ENST00000609890.1 | ENSG00000231898.4 | -0.820477158 | -1.839814616 | 0.065795459 |
| USP17L22 | ENST00000609972.1 | ENSG00000230651.3 | -0.842461465 | -1.895129105 | 0.0580753 |
| USP17L22 | ENST00000610007.1 | ENSG00000272660.1 | -0.836906831 | -1.850988169 | 0.064171255 |
| USP17L22 | NR_046742.2 | ZNF630-AS1 | 0.807895653 | 1.809929725 | 0.070306686 |
| USP17L22 | NR_047115.1 | PPP2R2B-IT1 | -0.845256612 | -1.917793084 | 0.055137253 |
| USP17L22 | NR_102703.1 | MAGEA8-AS1 | -0.802391844 | -1.782506741 | 0.074666633 |
| USP17L22 | NR_110160.1 | LOC100996249 | 0.805836959 | 1.814788852 | 0.069556364 |
| USP17L22 | NR_110370.1 | STAM-AS1 | 0.922931232 | 2.051274277 | 0.040240244 |
| USP17L22 | NR_110480.1 | LOC101927079 | -0.958763342 | -2.149348943 | 0.031606749 |
| USP17L22 | NR_110481.1 | LOC101927079 | -0.95133642 | -2.142621501 | 0.032143503 |
| USP17L22 | NR_120335.1 | LOC101928414 | -0.862186201 | -1.929761156 | 0.053636439 |
| USP17L22 | NR_120466.1 | LINC01489 | -0.839604207 | -1.885641803 | 0.05934324 |
| USP17L22 | NR_120655.1 | KCNMA1-AS1 | -0.838283092 | -1.876255351 | 0.060620225 |
| USP17L22 | NR_130916.1 | LOC105274304 | 0.842932864 | 1.929408501 | 0.053680169 |
| USP17L22 | NR_131963.1 | LVCAT5 | -0.861116869 | -1.922853741 | 0.054498421 |
| USP17L22 | NR_134597.1 | LOC105378068 | -0.828217409 | -1.867426408 | 0.061842065 |
| USP17L22 | NR_134664.1 | LOC105374366 | -0.939040647 | -2.099631777 | 0.035761245 |
| USP17L22 | NR_134665.1 | LOC105374366 | -0.949214698 | -2.108033373 | 0.035028103 |

| | | | | | |
|---|---|---|---|---|---|
| USP17L4 | ENST00000415205.1 | ENSG00000182057.4 | 0.86785602 | 1.903321415 | 0.056998618 |
| USP17L4 | ENST00000426302.1 | ENSG00000230454.1 | 0.820012088 | 1.845567399 | 0.064955044 |
| USP17L4 | ENST00000426475.1 | ENSG00000239467.1 | 0.83323809 | 1.869016273 | 0.06162055 |
| USP17L4 | ENST00000427524.1 | ENSG00000236065.2 | 0.912850425 | 2.044964012 | 0.040858406 |
| USP17L4 | ENST00000428765.1 | ENSG00000230107.1 | 0.844366288 | 1.880186451 | 0.060082671 |
| USP17L4 | ENST00000429080.1 | ENSG00000233047.1 | -0.812156223 | -1.789580395 | 0.073521393 |
| USP17L4 | ENST00000430920.1 | ENSG00000234203.1 | 0.937334875 | 2.105594529 | 0.035239588 |
| USP17L4 | ENST00000433905.2 | ENSG00000229299.2 | 0.801255426 | 1.775051525 | 0.07588938 |
| USP17L4 | ENST00000435832.1 | ENSG00000229201.1 | -0.85758917 | -1.931733006 | 0.053392469 |
| USP17L4 | ENST00000438409.1 | ENSG00000234174.1 | 0.80465923 | 1.803634375 | 0.071288644 |
| USP17L4 | ENST00000438969.2 | ENSG00000228031.2 | -0.800139039 | -1.758906491 | 0.078593393 |
| USP17L4 | ENST00000441809.2 | ENSG00000237445.2 | 0.877409913 | 1.944600223 | 0.051823112 |
| USP17L4 | ENST00000442829.1 | ENSG00000225284.1 | 0.839505744 | 1.894593726 | 0.058146247 |
| USP17L4 | ENST00000446562.1 | ENSG00000233896.1 | 0.840878671 | 1.888570497 | 0.058949403 |
| USP17L4 | ENST00000448431.1 | ENSG00000232548.1 | -0.864555885 | -1.902397274 | 0.057119237 |
| USP17L4 | ENST00000448674.1 | ENSG00000235119.1 | 0.916240033 | 2.062159625 | 0.039192541 |
| USP17L4 | ENST00000450365.1 | ENSG00000224404.1 | -0.803849964 | -1.8154658 | 0.069452357 |
| USP17L4 | ENST00000451090.1 | ENSG00000235215.2 | -0.856411176 | -1.898572509 | 0.057620706 |
| USP17L4 | ENST00000452553.1 | ENSG00000233973.1 | -0.810815717 | -1.846602039 | 0.064804838 |
| USP17L4 | ENST00000457632.1 | ENSG00000234248.1 | -0.806996846 | -1.822180172 | 0.068427652 |
| USP17L4 | ENST00000468165.1 | ENSG00000239480.1 | 0.888651079 | 1.979200543 | 0.047793432 |
| USP17L4 | ENST00000503723.1 | ENSG00000250472.1 | -0.852587593 | -1.895397357 | 0.058039779 |
| USP17L4 | ENST00000504301.1 | ENSG00000250696.1 | -0.82583869 | -1.837864286 | 0.066082406 |
| USP17L4 | ENST00000506394.1 | ENSG00000251665.1 | 0.941116059 | 2.103923772 | 0.035385097 |
| USP17L4 | ENST00000511631.1 | ENSG00000250402.1 | 0.883435589 | 1.995586341 | 0.045978968 |
| USP17L4 | ENST00000512036.1 | ENSG00000250993.1 | -0.81673955 | -1.818384109 | 0.069005445 |
| USP17L4 | ENST00000517846.1 | ENSG00000254485.1 | 0.893459198 | 1.995803831 | 0.04595528 |
| USP17L4 | ENST00000519005.1 | ENSG00000253507.1 | -0.809327289 | -1.827526557 | 0.067620637 |
| USP17L4 | ENST00000521294.1 | ENSG00000253664.1 | 0.915005387 | 2.042507448 | 0.041101221 |
| USP17L4 | ENST00000521653.1 | ENSG00000253301.1 | 0.887738954 | 1.97405061 | 0.048375988 |
| USP17L4 | ENST00000523806.1 | ENSG00000253616.1 | 0.969115948 | 2.16177615 | 0.030635435 |
| USP17L4 | ENST00000524942.1 | ENSG00000255553.1 | 0.863331695 | 1.941095107 | 0.052246742 |
| USP17L4 | ENST00000527086.1 | ENSG00000255182.1 | 0.924127944 | 2.092243395 | 0.036416748 |
| USP17L4 | ENST00000527274.2 | ENSG00000255517.2 | 0.84075278 | 1.874306112 | 0.060888246 |
| USP17L4 | ENST00000527579.1 | ENSG00000254574.1 | 0.815532025 | 1.830728458 | 0.067141083 |
| USP17L4 | ENST00000527727.1 | ENSG00000255227.1 | -0.838254554 | -1.883523664 | 0.059629436 |
| USP17L4 | ENST00000527757.1 | ENSG00000255109.1 | -0.9447658 | -2.112877881 | 0.034611222 |
| USP17L4 | ENST00000529160.1 | ENSG00000246790.2 | 0.878808398 | 1.959762849 | 0.050023515 |
| USP17L4 | ENST00000529247.1 | ENSG00000254741.1 | 0.880335884 | 1.966062216 | 0.049291424 |
| USP17L4 | ENST00000535315.1 | ENSG00000250748.2 | -0.923251975 | -2.074829414 | 0.038002347 |
| USP17L4 | ENST00000536529.1 | ENSG00000256422.1 | -0.831057601 | -1.83621936 | 0.066325221 |
| USP17L4 | ENST00000538641.1 | ENSG00000256422.1 | -0.865220015 | -1.90907743 | 0.056252104 |
| USP17L4 | ENST00000543275.1 | ENSG00000256944.1 | 0.802839002 | 1.797323417 | 0.072284291 |
| USP17L4 | ENST00000543403.1 | ENSG00000256684.1 | -0.822612746 | -1.811089704 | 0.070126967 |
| USP17L4 | ENST00000548210.1 | ENSG00000257784.1 | 0.814066176 | 1.809485708 | 0.070375578 |
| USP17L4 | ENST00000549616.1 | ENSG00000258168.1 | -0.823193209 | -1.810166523 | 0.070269967 |
| USP17L4 | ENST00000549806.1 | ENSG00000257252.1 | 0.876251787 | 1.948636955 | 0.051338796 |
| USP17L4 | ENST00000550279.1 | ENSG00000258338.1 | -0.898283162 | -2.026509547 | 0.042712595 |
| USP17L4 | ENST00000551135.1 | ENSG00000258294.1 | -0.83451639 | -1.871755798 | 0.061240396 |
| USP17L4 | ENST00000552525.1 | ENSG00000257286.1 | 0.846472421 | 1.888757973 | 0.058924267 |
| USP17L4 | ENST00000552541.1 | ENSG00000258294.1 | -0.843661488 | -1.890750395 | 0.058657672 |

| | | | | | |
|---|---|---|---|---|---|
| USP17L4 | ENST00000558221.1 | ENSG00000259704.1 | 0.882366947 | 1.97690889 | 0.04805193 |
| USP17L4 | ENST00000558575.1 | ENSG00000259687.1 | 0.861110945 | 1.934431878 | 0.05306005 |
| USP17L4 | ENST00000561653.1 | ENSG00000260095.1 | 0.832316366 | 1.841675921 | 0.065522568 |
| USP17L4 | ENST00000562191.1 | ENSG00000261292.1 | -0.807169313 | -1.816852216 | 0.069239745 |
| USP17L4 | ENST00000563610.1 | ENSG00000260051.1 | 0.804259758 | 1.778880484 | 0.075259358 |
| USP17L4 | ENST00000563611.1 | ENSG00000261583.1 | 0.860324702 | 1.915429507 | 0.055437751 |
| USP17L4 | ENST00000563806.1 | ENSG00000238045.5 | 0.802430065 | 1.79209116 | 0.073118365 |
| USP17L4 | ENST00000564809.1 | ENSG00000261471.1 | 0.930803875 | 2.075857488 | 0.037907134 |
| USP17L4 | ENST00000565735.1 | ENSG00000261213.1 | -0.854548352 | -1.916150071 | 0.055345997 |
| USP17L4 | ENST00000568243.1 | ENSG00000261521.1 | -0.811759882 | -1.820544641 | 0.068676104 |
| USP17L4 | ENST00000569313.1 | ENSG00000261604.1 | -0.973505459 | -2.200851041 | 0.027746571 |
| USP17L4 | ENST00000571775.1 | ENSG00000262456.1 | 0.802289701 | 1.788391196 | 0.073712916 |
| USP17L4 | ENST00000577853.1 | ENSG00000264207.1 | 0.852756404 | 1.912267989 | 0.055841829 |
| USP17L4 | ENST00000578800.1 | ENSG00000264235.1 | 0.829889761 | 1.872561561 | 0.061128954 |
| USP17L4 | ENST00000579775.1 | ENSG00000264108.1 | 0.913282179 | 2.047012978 | 0.04065681 |
| USP17L4 | ENST00000580622.1 | ENSG00000264634.1 | 0.871708919 | 1.924542785 | 0.054286583 |
| USP17L4 | ENST00000582044.1 | ENSG00000263715.2 | 0.844121374 | 1.884376733 | 0.059514035 |
| USP17L4 | ENST00000588182.2 | ENSG00000267453.2 | 0.887627012 | 2.006637069 | 0.044788321 |
| USP17L4 | ENST00000588402.1 | ENSG00000267006.1 | -0.918761521 | -2.043805137 | 0.040972802 |
| USP17L4 | ENST00000589380.1 | ENSG00000267488.1 | 0.88929609 | 1.978936972 | 0.047823103 |
| USP17L4 | ENST00000591621.1 | ENSG00000232116.2 | -0.821908904 | -1.826660716 | 0.067750798 |
| USP17L4 | ENST00000592413.1 | ENSG00000266933.1 | 0.889355075 | 1.966472116 | 0.0492441 |
| USP17L4 | ENST00000592498.1 | ENSG00000267488.1 | 0.912999566 | 2.011994348 | 0.044220533 |
| USP17L4 | ENST00000592518.1 | ENSG00000267786.1 | 0.953838243 | 2.119861542 | 0.034017724 |
| USP17L4 | ENST00000592525.1 | ENSG00000267214.1 | 0.902648976 | 2.025044444 | 0.042862801 |
| USP17L4 | ENST00000593139.1 | ENSG00000267042.1 | 0.910671631 | 2.033879088 | 0.041963788 |
| USP17L4 | ENST00000593175.1 | ENSG00000229036.3 | -0.927049803 | -2.06603426 | 0.038825245 |
| USP17L4 | ENST00000594589.1 | ENSG00000269321.1 | 0.820289721 | 1.814493763 | 0.069601742 |
| USP17L4 | ENST00000594850.1 | ENSG00000268093.1 | 0.898488415 | 1.988367934 | 0.046771013 |
| USP17L4 | ENST00000595972.1 | ENSG00000230333.2 | -0.826503771 | -1.862309116 | 0.062559531 |
| USP17L4 | ENST00000596567.1 | ENSG00000226647.2 | -0.842217621 | -1.886658284 | 0.059206302 |
| USP17L4 | ENST00000596887.1 | ENSG00000237031.3 | -0.943251251 | -2.123970718 | 0.033672592 |
| USP17L4 | ENST00000597256.1 | ENSG00000267986.1 | 0.878466446 | 1.951682465 | 0.050975916 |
| USP17L4 | ENST00000600242.1 | ENSG00000269583.1 | 0.965204168 | 2.144318315 | 0.03200739 |
| USP17L4 | ENST00000602507.1 | ENSG00000270069.1 | -0.833658953 | -1.869405407 | 0.061566433 |
| USP17L4 | ENST00000604183.1 | ENSG00000271185.1 | 0.878866778 | 1.953380868 | 0.050774482 |
| USP17L4 | ENST00000608677.1 | ENSG00000273350.1 | 0.800408992 | 1.77955012 | 0.075149616 |
| USP17L4 | ENST00000608943.1 | ENSG00000273368.1 | -0.864541799 | -1.915395904 | 0.055442033 |
| USP17L4 | ENST00000609113.1 | ENSG00000272827.1 | 0.824653811 | 1.846410298 | 0.064832653 |
| USP17L4 | ENST00000609934.1 | ENSG00000273271.1 | -0.866540742 | -1.946389297 | 0.051607994 |
| USP17L4 | ENST00000609952.1 | ENSG00000233766.3 | -0.880706551 | -1.9603945 | 0.049949698 |
| USP17L4 | ENST00000610270.1 | ENSG00000272576.1 | -0.867010866 | -1.933372462 | 0.053190331 |
| USP17L4 | NR_027052.1 | THAP7-AS1 | 0.918741015 | 2.064780645 | 0.03894376 |
| USP17L4 | NR_038421.1 | LINC01220 | 0.854321312 | 1.90605886 | 0.056642569 |
| USP17L4 | NR_038923.1 | SSSCA1-AS1 | 0.933252215 | 2.08775967 | 0.036819519 |
| USP17L4 | NR_040049.1 | SDCBP2-AS1 | 0.856002281 | 1.901442438 | 0.057244085 |
| USP17L4 | NR_046871.1 | LINC00333 | -0.878469215 | -1.94246375 | 0.052080984 |
| USP17L4 | NR_103776.1 | CHRM3-AS2 | -0.968457103 | -2.136251248 | 0.03265894 |
| USP17L4 | NR_103841.1 | LINC00539 | 0.866555132 | 1.932806409 | 0.053260051 |
| USP17L4 | NR_108106.1 | LINC01135 | 0.981108627 | 2.178532127 | 0.029366443 |
| USP17L4 | NR_109831.1 | RASSF1-AS1 | 0.972980014 | 2.188258737 | 0.028650761 |

| | | | | | |
|---|---|---|---|---|---|
| USP17L4 | NR_110556.1 | LOC102724890 | -0.821843492 | -1.851776399 | 0.064057938 |
| USP17L4 | NR_110803.1 | LOC101927018 | 0.910874518 | 2.053960987 | 0.039979468 |
| USP17L4 | NR_110824.1 | LINC01986 | -0.912243771 | -2.0335839 | 0.041993566 |
| USP17L4 | NR_111951.1 | LINC00869 | 0.930228767 | 2.078356665 | 0.037676521 |
| USP17L4 | NR_111952.1 | LINC00869 | 0.912734029 | 2.027900392 | 0.042570415 |
| USP17L4 | NR_111953.1 | LINC00869 | 0.936871091 | 2.120324812 | 0.033978663 |
| USP17L4 | NR_120502.1 | JARID2-AS1 | 0.851665189 | 1.889083786 | 0.058880603 |
| USP17L4 | NR_125849.1 | LOC101928140 | -0.960221944 | -2.156597089 | 0.031037072 |
| USP17L4 | NR_131204.1 | XACT | 0.821900989 | 1.812469133 | 0.069913739 |
| USP17L4 | NR_133930.1 | LOC105375556 | -0.943624332 | -2.095679334 | 0.036110646 |
| USP17L4 | NR_134520.1 | LOC727993 | 0.846119301 | 1.88693239 | 0.05916942 |
| USP17L4 | NR_135040.1 | LOC101927038 | 0.865592716 | 1.943865829 | 0.051911633 |
| USP17L4 | NR_135041.1 | LOC101927038 | 0.97362231 | 2.176794388 | 0.029495912 |
| USP17L4 | NR_135097.1 | LOC105369443 | -0.822612746 | -1.841720811 | 0.065515998 |
| USP17L4 | NR_136215.1 | VCAN-AS1 | -0.921649718 | -2.033733842 | 0.041978438 |
| USP17L4 | NR_136320.1 | LOC105373656 | 0.830169753 | 1.849467634 | 0.064390317 |
| USP17L7 | ENST00000362684.1 | ENSG00000228549.2 | 0.895735546 | 2.004084904 | 0.045060966 |
| USP17L7 | ENST00000416002.1 | ENSG00000230233.1 | 0.841322387 | 1.854146756 | 0.063718167 |
| USP17L7 | ENST00000417315.1 | ENSG00000242486.1 | 0.855232286 | 1.916354754 | 0.055319956 |
| USP17L7 | ENST00000418621.1 | ENSG00000224731.1 | -0.803709312 | -1.782930998 | 0.074597536 |
| USP17L7 | ENST00000421597.1 | ENSG00000227851.1 | -0.855845619 | -1.937854477 | 0.052640979 |
| USP17L7 | ENST00000422807.1 | ENSG00000227683.1 | -0.937624475 | -2.121879432 | 0.033847864 |
| USP17L7 | ENST00000423193.1 | ENSG00000224239.1 | -0.845156221 | -1.898744947 | 0.057598019 |
| USP17L7 | ENST00000424342.1 | ENSG00000234988.1 | 0.956656431 | 2.145146096 | 0.031941168 |
| USP17L7 | ENST00000425058.1 | ENSG00000226771.1 | -0.878464392 | -1.956693374 | 0.050383529 |
| USP17L7 | ENST00000426125.1 | ENSG00000223653.1 | -0.843060188 | -1.862081024 | 0.06259167 |
| USP17L7 | ENST00000430247.1 | ENSG00000232855.2 | -0.901852075 | -2.028150388 | 0.042544902 |
| USP17L7 | ENST00000432265.1 | ENSG00000231170.1 | 0.843435918 | 1.88714772 | 0.05914046 |
| USP17L7 | ENST00000434292.1 | ENSG00000229796.1 | -0.838958356 | -1.871735204 | 0.061243247 |
| USP17L7 | ENST00000435271.1 | ENSG00000231132.1 | 0.825107773 | 1.823468398 | 0.068232479 |
| USP17L7 | ENST00000435828.1 | ENSG00000235612.1 | -0.839104327 | -1.887897008 | 0.059039778 |
| USP17L7 | ENST00000439529.1 | ENSG00000236526.1 | -0.869925514 | -1.941791586 | 0.052162336 |
| USP17L7 | ENST00000442017.1 | ENSG00000229660.1 | 0.844574912 | 1.871286108 | 0.061305435 |
| USP17L7 | ENST00000442852.1 | ENSG00000237923.1 | 0.897467316 | 2.032299201 | 0.042123375 |
| USP17L7 | ENST00000443066.2 | ENSG00000237633.2 | -0.902706221 | -2.027165868 | 0.042645453 |
| USP17L7 | ENST00000443162.1 | ENSG00000234183.1 | -0.872151648 | -1.931081213 | 0.05347301 |
| USP17L7 | ENST00000445178.1 | ENSG00000234653.1 | -0.813156461 | -1.835553687 | 0.066423692 |
| USP17L7 | ENST00000445233.1 | ENSG00000233928.1 | -0.864413946 | -1.932702981 | 0.053272798 |
| USP17L7 | ENST00000447111.1 | ENSG00000231903.1 | -0.830238729 | -1.872592464 | 0.061124683 |
| USP17L7 | ENST00000448086.1 | ENSG00000237571.1 | 0.900331315 | 1.99762803 | 0.045757002 |
| USP17L7 | ENST00000449903.1 | ENSG00000223872.1 | 0.864421172 | 1.959560692 | 0.05004716 |
| USP17L7 | ENST00000451267.1 | ENSG00000230410.1 | 0.828431551 | 1.86478326 | 0.062211791 |
| USP17L7 | ENST00000451697.1 | ENSG00000233823.1 | 0.809455779 | 1.801126817 | 0.071682894 |
| USP17L7 | ENST00000451828.1 | ENSG00000228549.2 | 0.834050942 | 1.877688147 | 0.060423839 |
| USP17L7 | ENST00000452511.1 | ENSG00000231876.3 | -0.809605233 | -1.807232913 | 0.070725971 |
| USP17L7 | ENST00000453584.1 | ENSG00000233613.1 | 0.806011924 | 1.784775154 | 0.074297795 |
| USP17L7 | ENST00000455373.1 | ENSG00000226097.1 | -0.81141437 | -1.813394884 | 0.069770938 |
| USP17L7 | ENST00000456499.1 | ENSG00000237640.1 | 0.814917543 | 1.867060785 | 0.0618931 |
| USP17L7 | ENST00000457043.1 | ENSG00000231365.1 | -0.902214729 | -2.011105209 | 0.044314345 |
| USP17L7 | ENST00000457856.1 | ENSG00000228549.2 | 0.801676056 | 1.79643806 | 0.072424875 |
| USP17L7 | ENST00000479039.1 | ENSG00000241224.2 | 0.916896463 | 2.044080822 | 0.040945563 |

| | | | | | |
|---|---|---|---|---|---|
| USP17L7 | ENST00000483283.1 | ENSG00000240571.1 | -0.830076453 | -1.851462783 | 0.064103004 |
| USP17L7 | ENST00000503323.1 | ENSG00000249881.1 | 0.90497634 | 2.003033899 | 0.04517365 |
| USP17L7 | ENST00000503470.1 | ENSG00000248559.1 | -0.885803687 | -1.966363339 | 0.049256655 |
| USP17L7 | ENST00000503987.1 | ENSG00000250075.1 | -0.931593112 | -2.100294912 | 0.035702907 |
| USP17L7 | ENST00000504765.1 | ENSG00000249638.1 | -0.923353467 | -2.078794158 | 0.037636274 |
| USP17L7 | ENST00000505575.1 | ENSG00000248939.1 | 0.87852658 | 1.960429652 | 0.049945593 |
| USP17L7 | ENST00000505978.1 | ENSG00000249982.1 | 0.922298366 | 2.071581293 | 0.038304506 |
| USP17L7 | ENST00000506379.1 | ENSG00000240152.2 | -0.876968703 | -1.938855743 | 0.052518906 |
| USP17L7 | ENST00000506852.1 | ENSG00000250945.1 | -0.914059101 | -2.01792586 | 0.043598983 |
| USP17L7 | ENST00000508687.1 | ENSG00000250538.1 | -0.851569376 | -1.919149547 | 0.05496541 |
| USP17L7 | ENST00000508823.1 | ENSG00000250716.1 | -0.838746551 | -1.874892344 | 0.060807536 |
| USP17L7 | ENST00000508925.2 | ENSG00000249196.2 | 0.875275069 | 1.970422025 | 0.048790023 |
| USP17L7 | ENST00000510001.2 | ENSG00000249196.2 | 0.886822032 | 1.96534099 | 0.049374784 |
| USP17L7 | ENST00000512882.2 | ENSG00000251575.2 | -0.909545018 | -2.047553342 | 0.040603785 |
| USP17L7 | ENST00000519005.1 | ENSG00000253507.1 | -0.833503605 | -1.868635936 | 0.061673483 |
| USP17L7 | ENST00000519375.1 | ENSG00000253980.1 | -0.807405283 | -1.790566471 | 0.073362892 |
| USP17L7 | ENST00000520192.1 | ENSG00000253807.1 | -0.903951548 | -2.021383999 | 0.04324003 |
| USP17L7 | ENST00000521490.1 | ENSG00000253407.1 | 0.832053429 | 1.883571051 | 0.059623021 |
| USP17L7 | ENST00000521725.1 | ENSG00000253396.1 | -0.955036702 | -2.095107739 | 0.036161416 |
| USP17L7 | ENST00000522190.1 | ENSG00000254165.1 | -0.871090231 | -1.934918278 | 0.053000325 |
| USP17L7 | ENST00000522390.1 | ENSG00000254262.1 | -0.866753156 | -1.953704915 | 0.050736125 |
| USP17L7 | ENST00000527727.1 | ENSG00000255227.1 | -0.814717826 | -1.781303341 | 0.074862909 |
| USP17L7 | ENST00000531009.1 | ENSG00000255208.1 | -0.815682085 | -1.826681691 | 0.067747643 |
| USP17L7 | ENST00000546135.1 | ENSG00000256670.1 | 0.95912091 | 2.160636596 | 0.030723422 |
| USP17L7 | ENST00000550805.1 | ENSG00000244306.5 | 0.875546739 | 1.943177183 | 0.051994754 |
| USP17L7 | ENST00000551067.1 | ENSG00000257891.1 | 0.942900614 | 2.103012791 | 0.035464651 |
| USP17L7 | ENST00000553075.1 | ENSG00000257258.1 | -0.827911811 | -1.863060623 | 0.062453737 |
| USP17L7 | ENST00000553348.1 | ENSG00000258829.1 | 0.889324736 | 1.9951939 | 0.046021736 |
| USP17L7 | ENST00000553537.1 | ENSG00000258481.1 | 0.973263765 | 2.160813556 | 0.030709744 |
| USP17L7 | ENST00000553954.1 | ENSG00000259052.1 | 0.888954236 | 2.009342563 | 0.044500817 |
| USP17L7 | ENST00000555460.1 | ENSG00000259042.1 | 0.842701211 | 1.867062053 | 0.061892923 |
| USP17L7 | ENST00000555636.1 | ENSG00000259072.1 | -0.812615615 | -1.839773736 | 0.065801463 |
| USP17L7 | ENST00000556145.1 | ENSG00000258829.1 | 0.911248664 | 2.02414416 | 0.042955321 |
| USP17L7 | ENST00000559041.1 | ENSG00000259713.1 | -0.947857828 | -2.11190563 | 0.034694544 |
| USP17L7 | ENST00000560969.1 | ENSG00000259176.1 | -0.942336544 | -2.098511095 | 0.035860021 |
| USP17L7 | ENST00000561039.1 | ENSG00000259536.1 | 0.93075201 | 2.097938353 | 0.035910591 |
| USP17L7 | ENST00000561254.1 | ENSG00000259554.1 | -0.96767162 | -2.14900926 | 0.031633666 |
| USP17L7 | ENST00000563855.1 | ENSG00000260658.1 | -0.82191563 | -1.85582284 | 0.063478814 |
| USP17L7 | ENST00000565271.1 | ENSG00000261335.1 | 0.836848842 | 1.850231798 | 0.064280148 |
| USP17L7 | ENST00000565441.1 | ENSG00000261013.1 | -0.860501915 | -1.933570813 | 0.053165919 |
| USP17L7 | ENST00000569328.1 | ENSG00000261638.1 | -0.821286912 | -1.804971396 | 0.071079158 |
| USP17L7 | ENST00000569778.1 | ENSG00000260823.1 | 0.943320592 | 2.118222329 | 0.034156243 |
| USP17L7 | ENST00000570700.1 | ENSG00000263011.1 | 0.801004432 | 1.79199193 | 0.073134259 |
| USP17L7 | ENST00000572193.1 | ENSG00000261872.1 | -0.82021524 | -1.844961206 | 0.065043182 |
| USP17L7 | ENST00000573260.1 | ENSG00000262482.1 | -0.948504852 | -2.109693575 | 0.034884758 |
| USP17L7 | ENST00000580729.1 | ENSG00000266176.1 | 0.863554488 | 1.935270093 | 0.05295716 |
| USP17L7 | ENST00000581996.1 | ENSG00000265778.1 | -0.818206033 | -1.831904151 | 0.066965702 |
| USP17L7 | ENST00000582895.1 | ENSG00000264729.1 | 0.808353076 | 1.84012422 | 0.065750002 |
| USP17L7 | ENST00000588842.1 | ENSG00000235779.3 | -0.84716667 | -1.864162365 | 0.062298907 |
| USP17L7 | ENST00000591469.1 | ENSG00000267374.1 | -0.837651649 | -1.858747412 | 0.06306295 |
| USP17L7 | ENST00000592523.1 | ENSG00000226994.3 | -0.879648968 | -1.967179478 | 0.049162523 |

| | | | | | |
|---|---|---|---|---|---|
| USP17L7 | ENST00000595007.1 | ENSG00000231876.3 | -0.821852388 | -1.846610961 | 0.064803544 |
| USP17L7 | ENST00000597680.1 | ENSG00000269574.1 | -0.86583719 | -1.923668576 | 0.054396139 |
| USP17L7 | ENST00000597755.1 | ENSG00000236194.2 | -0.931343912 | -2.070610336 | 0.038395225 |
| USP17L7 | ENST00000598065.1 | ENSG00000231731.3 | 0.957456853 | 2.156653529 | 0.03103267 |
| USP17L7 | ENST00000598887.1 | ENSG00000268475.1 | 0.815419164 | 1.809804068 | 0.070326177 |
| USP17L7 | ENST00000598950.1 | ENSG00000269736.1 | -0.859542408 | -1.915060864 | 0.055484742 |
| USP17L7 | ENST00000602051.1 | ENSG00000227877.2 | 0.809995527 | 1.787593306 | 0.073841646 |
| USP17L7 | ENST00000602405.1 | ENSG00000269928.1 | 0.801007965 | 1.79851095 | 0.072096075 |
| USP17L7 | ENST00000602881.1 | ENSG00000269965.1 | -0.944430402 | -2.128066455 | 0.033331574 |
| USP17L7 | ENST00000602900.1 | ENSG00000270179.1 | 0.817085721 | 1.831878982 | 0.066969453 |
| USP17L7 | ENST00000604312.1 | ENSG00000270947.1 | -0.911805847 | -2.03590228 | 0.041760169 |
| USP17L7 | ENST00000605021.1 | ENSG00000271401.1 | -0.80856808 | -1.785257159 | 0.074219614 |
| USP17L7 | ENST00000606010.1 | ENSG00000272249.1 | -0.879006555 | -1.971551525 | 0.048660826 |
| USP17L7 | ENST00000606885.1 | ENSG00000231698.2 | -0.87190752 | -1.939170602 | 0.052480568 |
| USP17L7 | ENST00000607135.1 | ENSG00000272112.1 | -0.877765428 | -1.944651488 | 0.051816938 |
| USP17L7 | ENST00000607665.1 | ENSG00000272254.1 | -0.837801951 | -1.891770201 | 0.058521605 |
| USP17L7 | ENST00000608133.1 | ENSG00000273193.1 | -0.905469064 | -2.022093323 | 0.043166712 |
| USP17L7 | ENST00000608465.1 | ENSG00000272758.1 | -0.891550947 | -1.979210928 | 0.047792263 |
| USP17L7 | ENST00000608934.1 | ENSG00000273063.1 | -0.840906774 | -1.887088095 | 0.059148478 |
| USP17L7 | ENST00000609238.1 | ENSG00000272703.1 | 0.812889028 | 1.816333424 | 0.069319241 |
| USP17L7 | NR_027067.1 | LINC00114 | 0.922342661 | 2.067652039 | 0.038672755 |
| USP17L7 | NR_027402.1 | FAM223B | 0.834429905 | 1.894682473 | 0.058134481 |
| USP17L7 | NR_027425.1 | FAM66D | 0.978429036 | 2.166417588 | 0.030279291 |
| USP17L7 | NR_027440.1 | LOC100272217 | 0.891337943 | 1.99359368 | 0.046196477 |
| USP17L7 | NR_046748.1 | ARHGAP31-AS1 | -0.962838427 | -2.145445728 | 0.031917226 |
| USP17L7 | NR_102703.1 | MAGEA8-AS1 | -0.827900574 | -1.859495913 | 0.062956878 |
| USP17L7 | NR_102738.1 | LINC00911 | 0.961268719 | 2.167329438 | 0.030209743 |
| USP17L7 | NR_110007.1 | ADNP-AS1 | -0.803447267 | -1.792517793 | 0.073050062 |
| USP17L7 | NR_110117.1 | LOC101927769 | -0.805126905 | -1.827507921 | 0.067623436 |
| USP17L7 | NR_110284.1 | LOC101927907 | -0.813156461 | -1.812424453 | 0.069920637 |
| USP17L7 | NR_110731.1 | LINC01232 | 0.95037808 | 2.12896875 | 0.033256846 |
| USP17L7 | NR_110808.1 | LOC101927557 | 0.823556203 | 1.819580451 | 0.068822921 |
| USP17L7 | NR_120466.1 | LINC01489 | -0.932189003 | -2.073892666 | 0.03808928 |
| USP17L7 | NR_125769.1 | LINC01269 | 0.867373564 | 1.936022819 | 0.052864906 |
| USP17L7 | NR_126409.1 | LINC00376 | -0.928093773 | -2.077500691 | 0.037755372 |
| USP17L7 | NR_130916.1 | LOC105274304 | 0.860771899 | 1.937447766 | 0.052690633 |
| USP17L7 | NR_134610.1 | LOC105375014 | 0.879714118 | 1.952346582 | 0.050897071 |
| USP17L7 | NR_134632.1 | LOC105373051 | 0.825178002 | 1.844922129 | 0.065048867 |
| USP17L7 | NR_135040.1 | LOC101927038 | 0.804864537 | 1.805009327 | 0.071073223 |
| USP17L7 | NR_136218.1 | MEF2C-AS1 | -0.8248463 | -1.838495938 | 0.06598936 |
| USP17L8 | ENST00000412896.1 | ENSG00000197585.5 | 0.849120181 | 1.893340791 | 0.058312564 |
| USP17L8 | ENST00000413564.1 | ENSG00000224500.1 | 0.863313839 | 1.923509908 | 0.054416043 |
| USP17L8 | ENST00000413650.1 | ENSG00000230880.2 | -0.815583179 | -1.814188788 | 0.069648666 |
| USP17L8 | ENST00000413989.1 | ENSG00000242628.1 | -0.943404814 | -2.101545072 | 0.035593146 |
| USP17L8 | ENST00000414992.1 | ENSG00000233613.1 | 0.864172097 | 1.92831392 | 0.053816091 |
| USP17L8 | ENST00000417315.1 | ENSG00000242486.1 | 0.812269577 | 1.815280396 | 0.06948083 |
| USP17L8 | ENST00000417426.1 | ENSG00000233145.1 | -0.821543723 | -1.846652261 | 0.064797555 |
| USP17L8 | ENST00000420572.2 | ENSG00000233358.2 | -0.844846214 | -1.919604036 | 0.054907934 |
| USP17L8 | ENST00000421498.1 | ENSG00000237978.1 | -0.894669157 | -2.004245254 | 0.045043795 |
| USP17L8 | ENST00000421597.1 | ENSG00000227851.1 | -0.808623291 | -1.809200677 | 0.070419833 |
| USP17L8 | ENST00000423925.1 | ENSG00000223536.1 | 0.972550039 | 2.176206151 | 0.029539849 |

| | | | | | |
|---|---|---|---|---|---|
| USP17L8 | ENST00000424241.1 | ENSG00000237311.1 | 0.893677155 | 2.009022081 | 0.044534792 |
| USP17L8 | ENST00000425058.1 | ENSG00000226771.1 | -0.840502528 | -1.874386205 | 0.060877214 |
| USP17L8 | ENST00000425364.1 | ENSG00000231046.1 | 0.841934799 | 1.886015008 | 0.059292932 |
| USP17L8 | ENST00000426125.1 | ENSG00000223653.1 | -0.918779541 | -2.051373013 | 0.040230635 |
| USP17L8 | ENST00000426504.1 | ENSG00000234190.1 | 0.918227463 | 2.052644904 | 0.040107029 |
| USP17L8 | ENST00000426929.1 | ENSG00000230184.1 | 0.886555775 | 1.981054226 | 0.047585193 |
| USP17L8 | ENST00000427064.1 | ENSG00000238031.1 | -0.8394529 | -1.869963746 | 0.061488852 |
| USP17L8 | ENST00000427132.1 | ENSG00000232121.1 | 0.888863659 | 1.953080783 | 0.050810024 |
| USP17L8 | ENST00000428440.1 | ENSG00000232827.2 | 0.84721798 | 1.8842012 | 0.059537765 |
| USP17L8 | ENST00000429796.1 | ENSG00000231858.1 | 0.875785279 | 1.966241831 | 0.049270682 |
| USP17L8 | ENST00000429878.1 | ENSG00000224184.1 | 0.92053825 | 2.058338287 | 0.039557671 |
| USP17L8 | ENST00000430545.1 | ENSG00000237153.1 | -0.896698628 | -2.010316786 | 0.044397671 |
| USP17L8 | ENST00000430842.1 | ENSG00000230433.1 | 0.865675691 | 1.939963993 | 0.052384066 |
| USP17L8 | ENST00000431290.1 | ENSG00000183822.2 | -0.814844106 | -1.838765519 | 0.065949682 |
| USP17L8 | ENST00000432244.1 | ENSG00000234265.1 | -0.834133137 | -1.847260196 | 0.064709439 |
| USP17L8 | ENST00000432559.2 | ENSG00000228229.2 | -0.872496635 | -1.950391308 | 0.051129497 |
| USP17L8 | ENST00000433249.1 | ENSG00000236556.1 | -0.830304361 | -1.855488471 | 0.063526504 |
| USP17L8 | ENST00000434493.1 | ENSG00000224605.1 | -0.936500164 | -2.102934435 | 0.035471501 |
| USP17L8 | ENST00000435357.1 | ENSG00000225444.1 | -0.952373087 | -2.124803504 | 0.033603012 |
| USP17L8 | ENST00000435984.1 | ENSG00000204792.2 | -0.8900182 | -1.978688701 | 0.047851066 |
| USP17L8 | ENST00000436582.1 | ENSG00000236525.1 | 0.907517262 | 2.049157292 | 0.040446737 |
| USP17L8 | ENST00000437334.1 | ENSG00000226134.1 | 0.866390609 | 1.909902534 | 0.056145764 |
| USP17L8 | ENST00000439529.1 | ENSG00000236526.1 | -0.802333482 | -1.786293307 | 0.07405178 |
| USP17L8 | ENST00000440518.1 | ENSG00000226571.1 | 0.800082957 | 1.791761458 | 0.073171185 |
| USP17L8 | ENST00000440862.1 | ENSG00000223804.1 | 0.853228404 | 1.900916089 | 0.057313004 |
| USP17L8 | ENST00000441532.1 | ENSG00000234206.1 | 0.855447222 | 1.922885995 | 0.054494369 |
| USP17L8 | ENST00000443162.1 | ENSG00000234183.1 | -0.841429583 | -1.903343913 | 0.056995684 |
| USP17L8 | ENST00000444665.1 | ENSG00000228852.2 | -0.837322584 | -1.855596173 | 0.06351114 |
| USP17L8 | ENST00000444731.1 | ENSG00000227131.1 | 0.963631222 | 2.145685811 | 0.031898054 |
| USP17L8 | ENST00000445178.1 | ENSG00000234653.1 | -0.907222105 | -2.040019209 | 0.041348413 |
| USP17L8 | ENST00000445617.2 | ENSG00000225751.2 | -0.919525371 | -2.035961708 | 0.041754201 |
| USP17L8 | ENST00000445631.1 | ENSG00000231052.1 | 0.954125323 | 2.140224327 | 0.032336642 |
| USP17L8 | ENST00000447183.2 | ENSG00000271593.1 | -0.841562885 | -1.872426492 | 0.061147623 |
| USP17L8 | ENST00000448001.1 | ENSG00000229639.1 | -0.817334721 | -1.81321135 | 0.06979923 |
| USP17L8 | ENST00000448650.1 | ENSG00000223536.1 | 0.96175007 | 2.149207473 | 0.031617957 |
| USP17L8 | ENST00000449586.1 | ENSG00000235257.4 | 0.948913869 | 2.091510071 | 0.036482365 |
| USP17L8 | ENST00000449903.1 | ENSG00000223872.1 | 0.840480517 | 1.858391926 | 0.063113378 |
| USP17L8 | ENST00000450206.1 | ENSG00000234311.1 | 0.806370708 | 1.820965311 | 0.06861213 |
| USP17L8 | ENST00000452002.1 | ENSG00000236501.1 | 0.895807042 | 2.022681611 | 0.043105984 |
| USP17L8 | ENST00000453584.1 | ENSG00000233613.1 | 0.881169475 | 1.967393875 | 0.04913782 |
| USP17L8 | ENST00000453878.1 | ENSG00000224850.1 | -0.911013244 | -2.045309358 | 0.040824369 |
| USP17L8 | ENST00000454182.1 | ENSG00000230379.1 | -0.814243495 | -1.795290724 | 0.072607392 |
| USP17L8 | ENST00000454387.1 | ENSG00000223726.1 | 0.800520821 | 1.795663931 | 0.072547982 |
| USP17L8 | ENST00000454709.1 | ENSG00000237280.1 | 0.919944217 | 2.064744691 | 0.038947164 |
| USP17L8 | ENST00000457602.1 | ENSG00000237576.1 | 0.838627869 | 1.872598884 | 0.061123796 |
| USP17L8 | ENST00000457848.1 | ENSG00000226412.1 | -0.851566064 | -1.898038872 | 0.057690962 |
| USP17L8 | ENST00000460993.1 | ENSG00000241231.1 | -0.83627831 | -1.869731175 | 0.061521158 |
| USP17L8 | ENST00000476099.1 | ENSG00000244158.1 | 0.883441866 | 1.989652019 | 0.046629282 |
| USP17L8 | ENST00000477643.1 | ENSG00000241224.2 | 0.835358975 | 1.860588754 | 0.062802274 |
| USP17L8 | ENST00000479233.1 | ENSG00000243150.1 | -0.893730924 | -2.027470546 | 0.042614314 |
| USP17L8 | ENST00000482142.1 | ENSG00000243276.1 | -0.987861691 | -2.19588705 | 0.028100029 |

| | | | | | |
|---|---|---|---|---|---|
| USP17L8 | ENST00000485347.1 | ENSG00000239991.1 | 0.917240795 | 2.059460328 | 0.039450162 |
| USP17L8 | ENST00000488040.1 | ENSG00000243176.1 | -0.896732758 | -1.970252575 | 0.048809431 |
| USP17L8 | ENST00000502421.1 | ENSG00000250284.1 | 0.883162489 | 1.969772741 | 0.048864422 |
| USP17L8 | ENST00000503938.1 | ENSG00000246095.2 | -0.813040573 | -1.797009354 | 0.072334135 |
| USP17L8 | ENST00000505404.1 | ENSG00000249941.1 | 0.899406766 | 1.991945547 | 0.046377033 |
| USP17L8 | ENST00000506305.1 | ENSG00000249994.1 | 0.836352656 | 1.858672925 | 0.063073513 |
| USP17L8 | ENST00000506852.1 | ENSG00000250945.1 | -0.856080464 | -1.948157834 | 0.05139608 |
| USP17L8 | ENST00000507558.1 | ENSG00000248445.1 | -0.921285308 | -2.049349431 | 0.040427958 |
| USP17L8 | ENST00000507808.1 | ENSG00000250333.1 | 0.876285091 | 1.961426951 | 0.049829239 |
| USP17L8 | ENST00000508845.1 | ENSG00000271724.1 | -0.932264424 | -2.07395814 | 0.038083199 |
| USP17L8 | ENST00000509983.1 | ENSG00000248173.1 | 0.947084015 | 2.146369185 | 0.031843536 |
| USP17L8 | ENST00000510602.1 | ENSG00000249122.1 | 0.902745001 | 2.028037175 | 0.042556454 |
| USP17L8 | ENST00000511234.1 | ENSG00000250865.1 | 0.838528076 | 1.890754361 | 0.058657142 |
| USP17L8 | ENST00000511603.1 | ENSG00000249892.1 | -0.92684402 | -2.057089412 | 0.039677626 |
| USP17L8 | ENST00000514459.1 | ENSG00000248211.1 | 0.931965913 | 2.116749152 | 0.034281143 |
| USP17L8 | ENST00000515077.1 | ENSG00000251206.1 | -0.830724922 | -1.851026103 | 0.064165798 |
| USP17L8 | ENST00000518260.1 | ENSG00000253628.1 | 0.938628259 | 2.092130759 | 0.03642682 |
| USP17L8 | ENST00000518620.1 | ENSG00000253892.1 | 0.971744042 | 2.168227661 | 0.030141369 |
| USP17L8 | ENST00000518894.1 | ENSG00000204758.3 | -0.881824324 | -1.966259919 | 0.049268594 |
| USP17L8 | ENST00000519038.2 | ENSG00000254054.2 | 0.908894239 | 2.017866064 | 0.043605212 |
| USP17L8 | ENST00000519375.1 | ENSG00000253980.1 | -0.932451539 | -2.107400084 | 0.035082914 |
| USP17L8 | ENST00000519660.1 | ENSG00000253416.1 | -0.922787153 | -2.083463125 | 0.037209029 |
| USP17L8 | ENST00000519844.1 | ENSG00000253824.1 | 0.956440925 | 2.142900547 | 0.032121085 |
| USP17L8 | ENST00000521378.1 | ENSG00000254222.1 | 0.822461888 | 1.833256023 | 0.066764506 |
| USP17L8 | ENST00000521490.1 | ENSG00000253407.1 | 0.905968664 | 2.033311685 | 0.042021043 |
| USP17L8 | ENST00000522190.1 | ENSG00000254165.1 | -0.833302204 | -1.86961405 | 0.061537433 |
| USP17L8 | ENST00000522408.1 | ENSG00000253484.1 | -0.815384129 | -1.814056101 | 0.069669089 |
| USP17L8 | ENST00000524133.1 | ENSG00000253174.2 | 0.911684612 | 2.027198781 | 0.042642088 |
| USP17L8 | ENST00000524309.1 | ENSG00000240915.2 | 0.829199002 | 1.860700871 | 0.062786431 |
| USP17L8 | ENST00000525855.1 | ENSG00000254746.1 | 0.851378757 | 1.909608315 | 0.056183664 |
| USP17L8 | ENST00000528818.1 | ENSG00000232995.3 | -0.807445005 | -1.814410153 | 0.069614604 |
| USP17L8 | ENST00000528869.1 | ENSG00000255443.1 | -0.950401397 | -2.139767559 | 0.032373556 |
| USP17L8 | ENST00000531071.1 | ENSG00000255248.2 | 0.94769004 | 2.090103204 | 0.03660853 |
| USP17L8 | ENST00000533938.1 | ENSG00000255142.1 | 0.97954709 | 2.203602503 | 0.027552311 |
| USP17L8 | ENST00000535324.1 | ENSG00000255968.1 | -0.86442827 | -1.919589422 | 0.054909781 |
| USP17L8 | ENST00000538294.1 | ENSG00000250748.2 | 0.997092391 | 2.224669843 | 0.02610341 |
| USP17L8 | ENST00000544089.1 | ENSG00000256273.1 | -0.801765887 | -1.793433185 | 0.072903685 |
| USP17L8 | ENST00000547175.1 | ENSG00000257395.1 | 0.82739373 | 1.848473606 | 0.064533859 |
| USP17L8 | ENST00000548748.1 | ENSG00000258252.1 | 0.951496996 | 2.124803287 | 0.03360303 |
| USP17L8 | ENST00000549756.1 | ENSG00000257769.1 | 0.88209409 | 1.988990693 | 0.04670223 |
| USP17L8 | ENST00000550805.1 | ENSG00000244306.5 | 0.84910775 | 1.882457439 | 0.059773933 |
| USP17L8 | ENST00000553464.1 | ENSG00000258418.1 | -0.802557385 | -1.780565786 | 0.074983413 |
| USP17L8 | ENST00000553668.1 | ENSG00000258733.1 | -0.972115147 | -2.184281023 | 0.028941604 |
| USP17L8 | ENST00000554430.1 | ENSG00000258646.1 | 0.833133489 | 1.869468122 | 0.061557715 |
| USP17L8 | ENST00000555689.1 | ENSG00000259049.1 | 0.813071892 | 1.82714412 | 0.067678103 |
| USP17L8 | ENST00000556458.1 | ENSG00000258504.2 | 0.857110324 | 1.901819411 | 0.057194768 |
| USP17L8 | ENST00000556978.1 | ENSG00000258693.1 | -0.8761778 | -1.963672426 | 0.049568092 |
| USP17L8 | ENST00000557855.1 | ENSG00000259176.1 | -0.91680338 | -2.027523817 | 0.042608871 |
| USP17L8 | ENST00000558141.1 | ENSG00000259594.1 | -0.880299694 | -1.977936958 | 0.047935819 |
| USP17L8 | ENST00000560193.1 | ENSG00000259176.1 | -0.883260111 | -1.981504569 | 0.047534717 |
| USP17L8 | ENST00000561847.1 | ENSG00000260293.1 | 0.863732915 | 1.926223654 | 0.054076453 |

| | | | | | |
|---|---|---|---|---|---|
| USP17L8 | ENST00000563601.1 | ENSG00000260589.1 | 0.956626117 | 2.109357752 | 0.034913713 |
| USP17L8 | ENST00000565271.1 | ENSG00000261335.1 | 0.912544477 | 1.995855464 | 0.045949658 |
| USP17L8 | ENST00000565623.1 | ENSG00000261118.1 | 0.913090798 | 2.012596699 | 0.044157075 |
| USP17L8 | ENST00000569215.1 | ENSG00000260756.1 | -0.909930667 | -2.071613181 | 0.03830153 |
| USP17L8 | ENST00000571815.1 | ENSG00000262810.1 | -0.825822747 | -1.836551854 | 0.06627608 |
| USP17L8 | ENST00000572608.1 | ENSG00000263305.1 | -0.824428049 | -1.826092314 | 0.067836358 |
| USP17L8 | ENST00000582895.1 | ENSG00000264729.1 | 0.867741092 | 1.932617641 | 0.053283318 |
| USP17L8 | ENST00000586010.1 | ENSG00000267606.1 | -0.823154473 | -1.834768476 | 0.066540001 |
| USP17L8 | ENST00000586338.1 | ENSG00000219410.4 | 0.81468152 | 1.829477474 | 0.067328111 |
| USP17L8 | ENST00000587049.1 | ENSG00000235535.3 | 0.947359429 | 2.119712483 | 0.0340303 |
| USP17L8 | ENST00000588177.1 | ENSG00000234899.5 | -0.878972591 | -1.980595814 | 0.047636619 |
| USP17L8 | ENST00000589281.1 | ENSG00000267707.1 | 0.886936352 | 1.979285521 | 0.047783869 |
| USP17L8 | ENST00000591103.1 | ENSG00000272895.1 | -0.801726149 | -1.790294362 | 0.073406602 |
| USP17L8 | ENST00000591137.1 | ENSG00000267405.1 | -0.877255912 | -1.977611346 | 0.047972568 |
| USP17L8 | ENST00000592431.1 | ENSG00000267475.1 | 0.864163143 | 1.923002679 | 0.054479713 |
| USP17L8 | ENST00000592523.1 | ENSG00000226994.3 | -0.873859978 | -1.963028129 | 0.049642906 |
| USP17L8 | ENST00000597420.1 | ENSG00000269564.1 | 0.873065877 | 1.957041344 | 0.050342608 |
| USP17L8 | ENST00000598131.1 | ENSG00000269043.1 | -0.819598713 | -1.818000855 | 0.069064002 |
| USP17L8 | ENST00000598356.1 | ENSG00000269640.1 | -0.900182032 | -2.010274187 | 0.044402177 |
| USP17L8 | ENST00000598887.1 | ENSG00000268475.1 | 0.818691989 | 1.849783119 | 0.064344815 |
| USP17L8 | ENST00000599387.1 | ENSG00000227733.4 | -0.815556458 | -1.844496449 | 0.065110823 |
| USP17L8 | ENST00000600848.1 | ENSG00000228065.6 | -0.866531511 | -1.949890275 | 0.051189199 |
| USP17L8 | ENST00000600959.1 | ENSG00000269303.1 | -0.830905263 | -1.849624909 | 0.06436763 |
| USP17L8 | ENST00000602443.1 | ENSG00000270076.1 | -0.851127407 | -1.888219476 | 0.058996492 |
| USP17L8 | ENST00000602790.1 | ENSG00000270000.1 | 0.883260111 | 1.989777397 | 0.046615462 |
| USP17L8 | ENST00000603612.1 | ENSG00000270996.1 | -0.924484894 | -2.075164371 | 0.037971304 |
| USP17L8 | ENST00000605021.1 | ENSG00000271401.1 | -0.877237908 | -1.96606481 | 0.049291125 |
| USP17L8 | ENST00000606757.1 | ENSG00000237188.3 | 0.803457598 | 1.780562275 | 0.074983987 |
| USP17L8 | ENST00000606898.1 | ENSG00000272094.1 | 0.844716165 | 1.876639463 | 0.060567525 |
| USP17L8 | ENST00000606921.1 | ENSG00000272402.1 | 0.924506911 | 2.074351632 | 0.038046666 |
| USP17L8 | ENST00000606938.1 | ENSG00000272198.1 | 0.83567165 | 1.87710882 | 0.060503181 |
| USP17L8 | ENST00000607594.1 | ENSG00000271766.1 | -0.853907453 | -1.906670929 | 0.056563214 |
| USP17L8 | ENST00000607740.1 | ENSG00000271916.1 | -0.874893504 | -1.944718818 | 0.051808829 |
| USP17L8 | ENST00000608173.1 | ENSG00000197099.4 | -0.833969121 | -1.869813414 | 0.061509733 |
| USP17L8 | ENST00000608289.1 | ENSG00000272958.1 | 0.955390202 | 2.117863225 | 0.034186653 |
| USP17L8 | ENST00000608422.1 | ENSG00000272866.1 | -0.929051593 | -2.09940868 | 0.03578089 |
| USP17L8 | ENST00000609067.1 | ENSG00000272849.1 | -0.82372459 | -1.810462244 | 0.070224134 |
| USP17L8 | ENST00000609238.1 | ENSG00000272703.1 | 0.887845592 | 2.008164605 | 0.044625803 |
| USP17L8 | ENST00000609789.1 | ENSG00000272707.1 | 0.829997653 | 1.844547981 | 0.06510332 |
| USP17L8 | ENST00000609890.1 | ENSG00000231898.4 | -0.801208642 | -1.785682733 | 0.074150643 |
| USP17L8 | NR_002765.2 | ASAP1-IT1 | -0.805961136 | -1.795615678 | 0.072555661 |
| USP17L8 | NR_022011.1 | PWARSN | -0.853206177 | -1.909796089 | 0.056159473 |
| USP17L8 | NR_026932.1 | PDCD4-AS1 | 0.858757186 | 1.937139692 | 0.05272827 |
| USP17L8 | NR_034111.1 | TRAF3IP2-AS1 | -0.826964982 | -1.863559287 | 0.06238362 |
| USP17L8 | NR_046556.1 | RBMS3-AS1 | -0.846458092 | -1.901578871 | 0.057226232 |
| USP17L8 | NR_102746.1 | ROPN1L-AS1 | 0.819984625 | 1.840637846 | 0.065674648 |
| USP17L8 | NR_109870.1 | LINC01723 | -0.843272433 | -1.878959821 | 0.06024998 |
| USP17L8 | NR_110284.1 | LOC101927907 | -0.907222105 | -2.057813393 | 0.03960805 |
| USP17L8 | NR_110370.1 | STAM-AS1 | 0.963449374 | 2.15590752 | 0.031090887 |
| USP17L8 | NR_110504.1 | LOC101929572 | 0.943983427 | 2.108915319 | 0.034951892 |
| USP17L8 | NR_120466.1 | LINC01489 | -0.808042866 | -1.795152954 | 0.072629334 |

| | | | | | |
|---|---|---|---|---|---|
| USP17L8 | NR_125407.1 | LOC102724604 | -0.872084945 | -1.942842533 | 0.052035187 |
| USP17L8 | NR_125925.1 | LOC101929448 | -0.926696409 | -2.075291276 | 0.037959548 |
| USP17L8 | NR_126041.1 | LOC101930071 | 0.952223521 | 2.12811395 | 0.033327637 |
| USP17L8 | NR_126412.1 | SCEL-AS1 | 0.804154016 | 1.784956691 | 0.074268342 |
| USP17L8 | NR_130916.1 | LOC105274304 | 0.895761629 | 1.984440545 | 0.04720675 |
| USP17L8 | NR_131243.1 | SMCR2 | 0.865042461 | 1.95215548 | 0.050919748 |
| USP17L8 | NR_133658.1 | HTR3E-AS1 | 0.824318049 | 1.836123732 | 0.066339359 |
| USP17L8 | NR_133942.1 | LOC105377247 | -0.851694609 | -1.925265656 | 0.054196132 |
| USP17L8 | NR_135239.1 | LINC01867 | -0.966530797 | -2.185889315 | 0.028823704 |
| USP17L8 | NR_135816.1 | LOC100996664 | 0.877470595 | 1.952235119 | 0.050910296 |
| VEGFB | ENST00000318291.4 | ENSG00000177406.4 | 0.919530365 | 2.058268606 | 0.039564356 |
| VEGFB | ENST00000381106.4 | ENSG00000205663.5 | -0.801486031 | -1.800418124 | 0.071794641 |
| VEGFB | ENST00000412759.1 | ENSG00000236933.1 | 0.882397255 | 1.996538019 | 0.045875392 |
| VEGFB | ENST00000412809.1 | ENSG00000229938.1 | -0.813938588 | -1.827923947 | 0.067560966 |
| VEGFB | ENST00000421207.1 | ENSG00000231768.1 | 0.907985638 | 2.044114583 | 0.040942229 |
| VEGFB | ENST00000423667.1 | ENSG00000225970.1 | 0.820699833 | 1.814520477 | 0.069597633 |
| VEGFB | ENST00000424735.1 | ENSG00000237457.2 | -0.831983816 | -1.862401217 | 0.062546558 |
| VEGFB | ENST00000425624.1 | ENSG00000223779.4 | 0.849032855 | 1.892294547 | 0.058451747 |
| VEGFB | ENST00000426237.2 | ENSG00000235527.2 | 0.946672431 | 2.113272774 | 0.034577428 |
| VEGFB | ENST00000426519.1 | ENSG00000234142.1 | 0.876964083 | 1.96995818 | 0.048843164 |
| VEGFB | ENST00000426699.1 | ENSG00000229308.1 | 0.882625506 | 1.981288271 | 0.047558955 |
| VEGFB | ENST00000433344.1 | ENSG00000234083.1 | -0.848493587 | -1.905359883 | 0.056733306 |
| VEGFB | ENST00000433614.1 | ENSG00000228534.1 | -0.825951963 | -1.840235857 | 0.065733618 |
| VEGFB | ENST00000435434.1 | ENSG00000231233.1 | 0.958186658 | 2.167495424 | 0.030197098 |
| VEGFB | ENST00000435892.1 | ENSG00000233635.2 | 0.891071348 | 2.001922449 | 0.045293073 |
| VEGFB | ENST00000435992.2 | ENSG00000232675.3 | 0.824704708 | 1.853862325 | 0.063758859 |
| VEGFB | ENST00000436982.2 | ENSG00000235335.2 | -0.813752336 | -1.835619467 | 0.066413956 |
| VEGFB | ENST00000438107.1 | ENSG00000234449.2 | 0.881630701 | 1.972565301 | 0.04854511 |
| VEGFB | ENST00000438190.1 | ENSG00000227214.2 | 0.934618202 | 2.07963549 | 0.037558979 |
| VEGFB | ENST00000439186.1 | ENSG00000237076.1 | 0.831622016 | 1.850440539 | 0.064250081 |
| VEGFB | ENST00000440714.1 | ENSG00000237609.1 | 0.814320174 | 1.811989145 | 0.069987874 |
| VEGFB | ENST00000441875.1 | ENSG00000239203.1 | 0.893127517 | 1.97747044 | 0.047988479 |
| VEGFB | ENST00000442069.1 | ENSG00000225655.1 | -0.804756358 | -1.816602351 | 0.069278023 |
| VEGFB | ENST00000447206.1 | ENSG00000230839.1 | 0.881452527 | 1.967252931 | 0.049154059 |
| VEGFB | ENST00000456715.1 | ENSG00000224893.1 | 0.868683019 | 1.927877687 | 0.053870342 |
| VEGFB | ENST00000458154.1 | ENSG00000235578.1 | 0.884906065 | 1.979481173 | 0.047761857 |
| VEGFB | ENST00000458194.1 | ENSG00000226193.1 | 0.847388226 | 1.901211049 | 0.057274374 |
| VEGFB | ENST00000458364.1 | ENSG00000225655.1 | -0.881635953 | -1.962291305 | 0.049728579 |
| VEGFB | ENST00000459985.1 | ENSG00000273066.1 | 0.915215212 | 2.043488583 | 0.041004096 |
| VEGFB | ENST00000484413.1 | ENSG00000271853.1 | 0.841457207 | 1.884517961 | 0.059494948 |
| VEGFB | ENST00000489077.1 | ENSG00000244198.1 | 0.84758494 | 1.907608388 | 0.05644185 |
| VEGFB | ENST00000489557.2 | ENSG00000257045.1 | 0.92958449 | 2.089218124 | 0.036688093 |
| VEGFB | ENST00000498693.1 | ENSG00000244198.1 | 0.938854516 | 2.109006413 | 0.034944028 |
| VEGFB | ENST00000505498.1 | ENSG00000250908.1 | 0.853578951 | 1.914367406 | 0.055573227 |
| VEGFB | ENST00000505556.1 | ENSG00000249409.1 | 0.909183002 | 2.029617589 | 0.042395426 |
| VEGFB | ENST00000506100.1 | ENSG00000249409.1 | 0.913783395 | 2.028845367 | 0.042474043 |
| VEGFB | ENST00000506791.1 | ENSG00000251131.1 | 0.874223656 | 1.939050402 | 0.052495201 |
| VEGFB | ENST00000508083.1 | ENSG00000249343.1 | 0.964295367 | 2.168697938 | 0.030105624 |
| VEGFB | ENST00000509036.1 | ENSG00000251131.1 | 0.843377426 | 1.904779422 | 0.05680875 |
| VEGFB | ENST00000509192.1 | ENSG00000250765.1 | 0.906169356 | 2.014770098 | 0.043928744 |
| VEGFB | ENST00000510941.1 | ENSG00000251339.1 | -0.804923654 | -1.787837736 | 0.073802191 |

| | | | | | |
|---|---|---|---|---|---|
| VEGFB | ENST00000515128.1 | ENSG00000248215.1 | -0.831813676 | -1.849220052 | 0.064426044 |
| VEGFB | ENST00000520603.1 | ENSG00000254001.1 | -0.842130979 | -1.870646692 | 0.061394068 |
| VEGFB | ENST00000522547.1 | ENSG00000253430.1 | -0.901562356 | -2.000635976 | 0.045431634 |
| VEGFB | ENST00000522600.1 | ENSG00000246582.2 | 0.830150067 | 1.847937146 | 0.064611436 |
| VEGFB | ENST00000524818.1 | ENSG00000254473.1 | 0.828739524 | 1.848452818 | 0.064536864 |
| VEGFB | ENST00000526186.1 | ENSG00000254510.1 | 0.825519475 | 1.832724832 | 0.066843502 |
| VEGFB | ENST00000526611.1 | ENSG00000246982.2 | 0.864750254 | 1.945978581 | 0.051657312 |
| VEGFB | ENST00000528887.1 | ENSG00000254501.1 | 0.920041545 | 2.04066955 | 0.041283684 |
| VEGFB | ENST00000543072.1 | ENSG00000256092.2 | -0.893941318 | -1.993556812 | 0.046200509 |
| VEGFB | ENST00000543275.1 | ENSG00000256944.1 | 0.806207644 | 1.801336262 | 0.071649896 |
| VEGFB | ENST00000547834.1 | ENSG00000258325.1 | 0.805780799 | 1.784976957 | 0.074265055 |
| VEGFB | ENST00000549878.1 | ENSG00000257284.1 | 0.813165613 | 1.819432452 | 0.06884548 |
| VEGFB | ENST00000552469.1 | ENSG00000258325.1 | 0.894419429 | 2.01369782 | 0.044041269 |
| VEGFB | ENST00000556397.1 | ENSG00000258654.1 | 0.814638492 | 1.806499197 | 0.0708404 |
| VEGFB | ENST00000556786.1 | ENSG00000258525.1 | -0.859013979 | -1.963009683 | 0.049645049 |
| VEGFB | ENST00000563018.1 | ENSG00000260193.1 | 0.856800894 | 1.902360983 | 0.057123978 |
| VEGFB | ENST00000564038.1 | ENSG00000261760.2 | 0.848760283 | 1.894894183 | 0.058106422 |
| VEGFB | ENST00000565823.1 | ENSG00000260686.1 | -0.86307357 | -1.92726425 | 0.053946707 |
| VEGFB | ENST00000565829.1 | ENSG00000260148.1 | 0.884541491 | 1.981663783 | 0.047516883 |
| VEGFB | ENST00000566170.1 | ENSG00000261071.1 | 0.820869749 | 1.818903005 | 0.068926229 |
| VEGFB | ENST00000569981.1 | ENSG00000238045.5 | 0.819609709 | 1.837062866 | 0.066200615 |
| VEGFB | ENST00000570493.2 | ENSG00000261898.2 | 0.818828559 | 1.840468616 | 0.065699468 |
| VEGFB | ENST00000570512.1 | ENSG00000262768.1 | 0.819395592 | 1.830787102 | 0.067132326 |
| VEGFB | ENST00000570843.1 | ENSG00000261889.1 | 0.933590527 | 2.076538499 | 0.037844174 |
| VEGFB | ENST00000570929.1 | ENSG00000262223.2 | 0.9557428 | 2.150247892 | 0.031535612 |
| VEGFB | ENST00000575139.1 | ENSG00000263072.1 | 0.831576722 | 1.833864732 | 0.066674076 |
| VEGFB | ENST00000576086.1 | ENSG00000262823.1 | 0.864594919 | 1.906750723 | 0.056552875 |
| VEGFB | ENST00000577064.1 | ENSG00000262823.1 | 0.851456622 | 1.91205695 | 0.05586889 |
| VEGFB | ENST00000577698.1 | ENSG00000265100.1 | 0.813943994 | 1.830939421 | 0.067109586 |
| VEGFB | ENST00000582558.1 | ENSG00000264569.1 | 0.825770542 | 1.857638088 | 0.063220426 |
| VEGFB | ENST00000584705.1 | ENSG00000264569.1 | 0.828751112 | 1.819674304 | 0.068808619 |
| VEGFB | ENST00000585559.1 | ENSG00000267117.1 | 0.883099718 | 1.957314871 | 0.05031046 |
| VEGFB | ENST00000586051.1 | ENSG00000267576.1 | 0.881534099 | 1.970025382 | 0.048835462 |
| VEGFB | ENST00000591174.1 | ENSG00000267289.1 | 0.875148974 | 1.958340019 | 0.050190128 |
| VEGFB | ENST00000592400.1 | ENSG00000267735.1 | 0.959955824 | 2.122267351 | 0.033815294 |
| VEGFB | ENST00000594590.2 | ENSG00000268199.2 | 0.932143358 | 2.097806051 | 0.035922282 |
| VEGFB | ENST00000595478.1 | ENSG00000237031.3 | -0.861992209 | -1.941691526 | 0.052174455 |
| VEGFB | ENST00000597169.1 | ENSG00000269720.1 | 0.887580838 | 1.964904311 | 0.049425314 |
| VEGFB | ENST00000597309.1 | ENSG00000232098.2 | -0.852134178 | -1.921569628 | 0.054659933 |
| VEGFB | ENST00000599259.1 | ENSG00000269352.1 | 0.867329964 | 1.947271501 | 0.051502194 |
| VEGFB | ENST00000600534.1 | ENSG00000267858.1 | 0.896110799 | 2.001168793 | 0.045374203 |
| VEGFB | ENST00000600726.1 | ENSG00000267858.1 | 0.889069897 | 2.008505793 | 0.044589571 |
| VEGFB | ENST00000601692.1 | ENSG00000267874.1 | -0.888653858 | -1.990390232 | 0.046547965 |
| VEGFB | ENST00000601735.1 | ENSG00000244513.2 | 0.815635417 | 1.808052022 | 0.070598404 |
| VEGFB | ENST00000602532.1 | ENSG00000270091.1 | 0.81377474 | 1.829023473 | 0.067396092 |
| VEGFB | ENST00000602872.1 | ENSG00000270067.1 | 0.823095176 | 1.847729237 | 0.064641522 |
| VEGFB | ENST00000604142.1 | ENSG00000271308.1 | 0.881197273 | 1.959938795 | 0.050002944 |
| VEGFB | ENST00000606277.1 | ENSG00000272145.1 | 0.815236148 | 1.806358815 | 0.07086231 |
| VEGFB | ENST00000606374.1 | ENSG00000272312.1 | -0.807133746 | -1.798367961 | 0.072118716 |
| VEGFB | ENST00000607224.1 | ENSG00000272521.1 | 0.840314942 | 1.859666943 | 0.062932662 |
| VEGFB | ENST00000607284.1 | ENSG00000272389.1 | 0.801001421 | 1.755181863 | 0.079228205 |

| | | | | | |
|---|---|---|---|---|---|
| VEGFB | ENST00000607476.1 | ENSG00000272540.1 | 0.85999398 | 1.960904151 | 0.049890205 |
| VEGFB | ENST00000607943.1 | ENSG00000273188.1 | 0.858507976 | 1.907462944 | 0.056460665 |
| VEGFB | ENST00000609281.1 | ENSG00000273320.1 | 0.830592101 | 1.864508772 | 0.062250291 |
| VEGFB | ENST00000609972.1 | ENSG00000230651.3 | 0.883498037 | 1.979343781 | 0.047777314 |
| VEGFB | ENST00000610145.1 | ENSG00000273175.1 | 0.905486997 | 2.021960608 | 0.043180422 |
| VEGFB | NR_003604.2 | ZFAS1 | 0.921588452 | 2.049202351 | 0.040442332 |
| VEGFB | NR_003605.1 | ZFAS1 | 0.93732067 | 2.092254006 | 0.0364158 |
| VEGFB | NR_003606.2 | ZFAS1 | 0.926307284 | 2.076113634 | 0.037883442 |
| VEGFB | NR_027271.1 | CIRBP-AS1 | 0.915972653 | 2.056201208 | 0.039763126 |
| VEGFB | NR_027334.2 | MZF1-AS1 | 0.914996066 | 2.01795793 | 0.043595643 |
| VEGFB | NR_036480.1 | VPS9D1-AS1 | 0.815000469 | 1.822855888 | 0.068325221 |
| VEGFB | NR_036658.1 | ZFAS1 | 0.928280717 | 2.03280291 | 0.042072439 |
| VEGFB | NR_044996.1 | HCG23 | 0.909319624 | 2.029152107 | 0.0424428 |
| VEGFB | NR_045114.1 | PVRL3-AS1 | -0.875108787 | -1.963475976 | 0.049590893 |
| VEGFB | NR_072981.1 | LINC00957 | 0.850950994 | 1.883232141 | 0.059668915 |
| VEGFB | NR_072982.1 | LINC00957 | 0.850849464 | 1.896008683 | 0.057958896 |
| VEGFB | NR_105010.1 | LINC01333 | 0.927137028 | 2.062803774 | 0.039131275 |
| VEGFB | NR_108036.1 | CFAP58-AS1 | 0.95616831 | 2.133258376 | 0.032903537 |
| VEGFB | NR_110919.1 | LOC101928530 | 0.812043157 | 1.821929653 | 0.06846566 |
| VEGFB | NR_120335.1 | LOC101928414 | 0.813424793 | 1.814682158 | 0.069572768 |
| VEGFB | NR_121188.1 | PGM5P3-AS1 | -0.811101485 | -1.837936358 | 0.066071784 |
| VEGFB | NR_121189.1 | PGM5P3-AS1 | -0.888869705 | -1.970223889 | 0.048812717 |
| VEGFB | NR_126166.1 | FAM74A7 | 0.804150853 | 1.810899625 | 0.070156391 |
| VEGFB | NR_135584.1 | LOC101927596 | 0.829483575 | 1.848044051 | 0.06459597 |

Gene1: Differential gene, Differential lncRNA, Gene2: Differential gene for lncRNA
